# Proteomics of *Fusobacterium nucleatum* within a model developing oral microbial community

**DOI:** 10.1002/mbo3.204

**Published:** 2014-08-25

**Authors:** Erik L Hendrickson, Tiansong Wang, David A C Beck, Brittany C Dickinson, Christopher J Wright, Richard J Lamont, Murray Hackett

**Affiliations:** 1 Department of Chemical Engineering and Center for Microbial Proteomics, University of WashingtonBox 355014, Seattle, Washington, 98195; 2 Department of Microbiology and Center for Microbial Proteomics, University of WashingtonBox 357242, Seattle, Washington, 98195; 3 Department of Chemical Engineering and the eScience Institute, University of WashingtonBox 355014, Seattle, Washington, 98195; 4 Department of Oral Biology, University of FloridaGainesville, Florida, 32610; 5 Center for Oral Health and Systemic Disease, University of LouisvilleLouisville, Kentucky, 40292

**Keywords:** Biofilm, *Fusobacterium nucleatum*, microbial proteomics, microbiome, oral microbial ecology

## Abstract

*Fusobacterium nucleatum* is a common oral organism that can provide adhesive and metabolic support to developing periodontal bacterial communities. It is within the context of these communities that disease occurs. We have previously reported whole cell proteomics analyses of *Porphyromonas gingivalis* and *Streptococcus gordonii* in early-stage communities with each other and with *F. nucleatum*, modeled using 18 h pellets. Here, we report the adaptation of *F. nucleatum* to the same experimental conditions as measured by differential protein expression. About 1210 *F. nucleatum* proteins were detected in single species *F. nucleatum* control samples, 1192 in communities with *P. gingivalis*, 1224 with *S. gordonii*, and 1135 with all three species. Quantitative comparisons among the proteomes revealed important changes in all mixed samples with distinct responses to *P. gingivalis* or *S. gordonii* alone and in combination. The results were inspected manually and an ontology analysis conducted using DAVID (Database for annotation, visualization, and integrated discovery). Extensive changes were detected in energy metabolism. All multispecies comparisons showed reductions in amino acid fermentation and a shift toward butanoate as a metabolic byproduct, although the two organism model community with *S. gordonii* showed increases in alanine, threonine, methionine, and cysteine pathways, and in the three species samples there were increases in lysine and methionine. The communities with *P. gingivalis* or all three organisms showed reduced glycolysis proteins, but *F. nucleatum* paired with *S. gordonii* displayed increased glycolysis/gluconeogenesis proteins. The *S. gordonii* containing two organism model also showed increases in the ethanolamine pathway while the three species sample showed decreases relative to the *F. nucleatum* single organism control. All of the nascent model communities displayed reduced translation, lipopolysaccharide, and cell wall biosynthesis, DNA replication and DNA repair.

## Introduction

Oral infections are among the most common human bacterial infections, and the gram-negative anaerobe *Fusobacterium nucleatum* (Baumgartner et al. 1992) is one of the most frequently detected organisms in periodontal disease pockets (Bolstad et al. 1996). However, *F. nucleatum* is also one of the most common species found in the subgingival crevice even in the absence of disease (Bolstad et al. 1996), and the categorization of *F. nucleatum* as a traditional pathogen or commensal is debatable. However, in current models of oral pathogenicity, health or disease states are not viewed in the context of a single pathogenic organism but rather of dysbiotic microbial communities (Hajishengallis and Lamont 2012). A number of in vivo studies have shown the potential importance of these community interactions, including elevated pathogenicity of polymicrobial communities in rodent abscess models (Ochiai et al. 1993; Yoneda et al. 2001; Kesavalu et al. 2007) and alveolar bone loss models (Daep et al. 2011; Orth et al. 2011; Settem et al. 2012). It has recently been demonstrated that nutrient transfer between species can increase pathogenicity (Ramsey et al. 2011). *Aggregatibacter actinomycetemcomitans,* an important organism in Localized Aggressive Periodontitis, had increased virulence when grown in the presence of *Streptococcus gordonii*. The increase was dependent on the ability of *A. actinomycetemcomitans* to utilize l-lactate, a byproduct of *S. gordonii* energy metabolism, as an energy source. A mutant strain unable to utilize l-lactate showed significantly decreased virulence in the coculture, turning synergy into antagonism (Ramsey et al. 2011). However, not all interactions are synergistic. For example, *S. cristatus* produces extracellular arginine deiminase which down regulates production of fimbrial adhesins by *Porphyromonas gingivalis* and thus abrogates colonization (Lin et al. 2008; Wang et al. 2009).

With ∼200 microbial species in any individual's oral cavity, dental plaque represents a complex and dynamic biofilm (Rosan and Lamont 2000; Aas et al. 2005). In order to examine oral microbial interactions in a more experimentally tractable system (Merritt et al. 2005), we have developed a model of nascent community interactions (Kuboniwa et al. 2009) using three representative species of oral bacteria, *F. nucleatum*, *S. gordonii,* and *P. gingivalis*. *Streptococcus gordonii* is a representative of the early colonizing organisms that bind to host derived molecules coating the oral surfaces (Nyvad and Kilian 1987). Secondary colonizing species, such as *F. nucleatum* and *P. gingivalis*, then adhere to the early colonizers. *Porphyromonas gingivalis* is an invasive intracellular oral pathogen (Lamont et al. 1995). *Fusobacterium nucleatum* and *P. gingivalis* are syngergistically pathogenic in mouse abscess models (Baumgartner et al. 1992). *Fusobacterium nucleatum* is also known for its ability to adhere to diverse microbes (Kolenbrander et al. 1989) and may reduce oxygen levels, thus aiding less aerotolerant organisms like *P. gingivalis* (Bradshaw and Marsh 1998). Moreover, a potential role is emerging for *F. nucleatum* in serious systemic conditions such as preterm delivery of low birth weight infants and colorectal cancer (Otomo-Corgel et al. 2012; Rubinstein et al. 2013).

We have previously examined the response of *P. gingivalis* (Kuboniwa et al. 2009) and *S. gordonii* (Hendrickson et al. 2012) in multispecies model communities with *F. nucleatum*. Both species yielded evidence for synergy. For example, *P. gingivalis* showed increased translation and decreased stress proteins in the presence of both *F. nucleatum* and *S. gordonii*. *Streptococcus gordonii* showed increased translation in the presence of *P. gingivalis* and increased energy metabolism in all of the mixed communities. Here, we report the changes in *F. nucleatum* protein levels in the same nascent model communities with *S. gordonii*, *P. gingivalis*, and all three species combined. Key biochemical pathways are presented in the figures so the reader may see at a glance the degree to which each pathway is populated and the direction of change. In general, pathways that are more highly populated with detected proteins engender a higher degree of confidence with respect to interpretation.

## Materials and Methods

### Bacteria and culture conditions

*Fusobacterium nucleatum* subsp. *nucleatum* ATCC 25586 and *P. gingivalis* ATCC 33277 were grown anaerobically (85% N_2_, 10% H_2_, 5% CO_2_) at 37°C in trypticase soy broth supplemented with 1 mg/mL yeast extract, 1 *μ*g/mL menadione, and 5 *μ*g/mL hemin. *Streptococcus gordonii* DL1 was grown anaerobically at 37°C in Todd-Hewitt broth (THB).

### Chemicals

HPLC grade acetonitrile was from Burdick & Jackson (Muskegon, MI); high purity acetic acid (99.99%) and ammonium acetate (99.99%), from Aldrich (Milwaukee, WI). High purity water was generated with a NANOpure UV system (Barnstead, Dubuque, IA).

### Proteomics of model bacterial communities

Sample preparation, capillary separations, and mass spectrometry data acquisition were handled as described in Kuboniwa et al. (2009). In brief, bacteria were cultured to mid-log phase, harvested by centrifugation and resuspended in prereduced phosphate buffered saline (PBS) (rPBS) at a pH of 7.2 for 18 h before harvesting for proteomics processing. 1 × 10^9^ cells of *F. nucleatum* were mixed with an equal number of *P. gingivalis*, *S. gordonii*, or both as combinations of the species. *Fusobacterium nucleatum* cells alone were also used as a control. Two independent biological replicates from separate experiments, comprised of at least two technical replicates, were analyzed. After being centrifuged at 3000*g* for 5 min the pelleted mixtures of bacteria were held in 1 mL rPBS in an anaerobic chamber at 37°C for 18 h (Merritt et al. 2005). Bacterial cells were lysed in resuspension buffer (15 mmol/L Tris HCl pH 9.5, 0.02% Rapigest™ Waters, Milford, MA) in a boiling water bath followed by sonication and bead beating. Proteins were then digested with trypsin then fractionated into five prefractions (Bosch et al. 2008). The 2D capillary HPLC/MS/MS analyses were conducted on a Thermo LTQ mass spectrometer (Thermo Fisher Corp., San Jose, CA). Peptides were eluted with a seven-step salt gradient (0, 10, 25, 50, 100, 250, and 500 mmol/L ammonium acetate) followed by an acetonitrile gradient elution (Solvent A: 99.5% water, 0.5% acetic acid. Solvent B: 99.5% acetonitrile, 0.5% acetic acid). The MS^1^ scan range for all samples was 400–2000 *m/z*. Each MS^1^ scan was followed by 10 MS^2^ scans in a data-dependent manner for the 10 most intense ions in the MS^1^ scan. Default parameters under Xcalibur 1.4 data acquisition software (Thermo Fisher) were used, with the exception of an isolation width of 3.0 *m/z* units and normalized collision energy of 40%.

### Data processing and protein identification

The proteomics data processing was performed using open source tools, as follows: The Thermo RAW files were cataloged by our pipeline and uploaded to a high availability data archive. They were then converted to compressed mzXML files with 64-bit data representation using ProteoWizard’ msconvert tool version 2.1.2131 (Kessner et al. 2008). The protein database was constructed from the predicted protein sequences from each of the three genomes, (*P. gingivalis* ACTT 33277 (Naito et al. 2008, *P. gingivalis* W83 Genome n.d. [http://cmr.jcvi.org/tigr-scripts/CMR/GenomePage.cgi?org=gpg]) [GenBank: AP009380], *S. gordonii* Challis NCTC7868 (*S. gordonii* ChallisNCTC7868 Genome n.d. [http://cmr.jcvi.org/tigrscripts/CMR/GenomePage.cgi?org=gsg]) [GenBank: CP00725.1], *F. nucleatum* ATCC 25586 (*F. nucleatum* ATCC 25586 Genome n.d.) [GenBank: AE009951.1]) and reversed sequences as decoys (tagged by the prefix DECOY), and four additional enzymes: trypsin precursor (gi 136429), DNase I precursor (gi 6647483), RNase A precursor (gi 133198), and lysyl endopeptidase (gi 7463016). The compressed mzXML files were sent, along with an associated *sequest.params* file and the reference protein database, to our shared, centrally managed university research supercomputer, Hyak (http://escience.washington.edu/content/hyak-0). Once there, peptide-spectra matching was performed with SEQUEST version 2011.01.1 (Eng et al. 1994). The SEQUEST*.out* files were converted to pepXML format with Transproteomic Pipeline (TPP, version 4.4.1) *Out2XML* tool (Deutsch et al. 2010). The results were archived as with the RAW files.

Next, the pepXML from technical replicates were pooled and analyzed with PeptideProphet (Keller et al. 2002) using TPP's *xinteract*. During this step, default arguments were used except for the following: peptide results were filtered if the PeptideProphet probability was below 0.85 (-p 0.85), only peptides longer than 6 amino acids long were considered (-l 7), and the nonparametric probability model was used with decoy sequence tags with the prefix ‘DECOY’ (-OP –dDECOY). The resulting pepXML was then processed by ProteinProphet (Nesvizhskii et al. 2003) to produce a protXML file. The default ProteinProphet parameters were used. The protXML was converted to a tabular text file using TPP's *protxml2html.pl*.

Protein relative abundances were estimated on the basis of spectral count values (Xia et al. 2007a,b) that have been demonstrated in the literature to correlate with protein abundance (Gao et al. 2003; Liu et al. 2004). The data from individual replicates and experimental conditions were compared to compute differential abundance. MySQL (http://www.mysql.com) was used to load the tabular ProteinProphet results into tables, which were then joined on the locus tag (i.e., ORF ID) key (MySQL n.d.). The resulting master table was exported to *R* for statistical comparisons and multiple hypothesis testing correction (Team RDC 2010). To calculate protein abundance ratios, a normalization scheme was applied such that the total spectral counts for all *F. nucleatum* proteins in each condition were set equal for each comparison. The samples were compared using *t*-tests of spectral count data as described previously (Bosch et al. 2008). The resulting *P-*values were treated with the *R* package *q*value (Storey 2002). If a protein was not detected by 3 or more unique peptides in a replicate, it was treated as not-observed (i.e., value of NA in *R*) and exempt from abundance ratio calculations and statistical tests. All processed data were subsequently uploaded to a FileMaker™ (Santa Clara, CA, USA) database maintained in-house. The data tables found in the Supplementary Information are static PDF files derived from this continually evolving database. Copies of the original FileMaker™ database are available from the corresponding author. The mass spectrometry data sets supporting the results of this article will be available upon publication in the University of Washington Libraries digital archive in mzXML format, http://dx.doi.org/10.6069/H5H41PB9.

### Electron microscopy

Samples were prepared for transmission electron microscopy by harvesting via centrifugation and resuspending in fixative (2% paraformaldehyde, 2.5% glutaraldehyde, 0.1% phosphate buffer). The electron microscopy was conducted at the University of Washington Electron Microscopy Lab. The samples were washed with 0.1 PB buffer twice for 10 min each, then put in 1% OsO_4_ in PB for 60 min., then twice in buffer wash for 10 min each, and dehydrated with 50% ethanol (ETOH), 70% ETOH, 90% ETOH, 100% ETOH, and 100% ETOH for 10 min each. After 2 changes of propylene oxide for 10 min each, the samples were then placed in a propylene oxide: resin mix (1:1) for 1.5 h. The mixture was replaced with 100% resin for 2 h. Samples were then embedded in 100% polybed resin and incubated at 60°C overnight. The resulting block was cut in 1 *μ*m sections using an ultramicrotone (Reichert-Jung Ultracut E, Buffalo, NY, USA) and stained with Richardson's stain (methylene blue, sodium borate, Azure 11). Stained blocks were cut to 0.2 *μ*m thin sections and put on EM grid stain with uranyl acetate and lead citrate. The sections were examined and images taken with a TeccnaiSpritBT TEM with an AMT DDK digital camera (2Kx2K).

### Ontology analysis

An overall list of detected proteins, as well as lists of proteins that showed increased or decreased levels in the community comparisons, were prepared using Entrez gene identifiers. Ontology analyses were then conducted using the DAVID (Huang et al. 2007) functional annotation clustering feature with the default databases. Both increased and decreased protein level lists were analyzed using the overall list of detected proteins as the background. Potentially interesting clusters identified by DAVID were then examined manually.

## Results and Discussion

Previous examinations of *S. gordonii* and *P. gingivalis* responses under the same conditions indicated synergistic effects in the mixed communities (Kuboniwa et al. 2009; Hendrickson et al. 2012). The *F. nucleatum* response was more complex and difficult to interpret. *Fusobacterium nucleatum* showed distinct responses in pellets with *S. gordonii* and with *P. gingivalis*. In addition, the model community with all three organisms produced a different response than either organism alone. In general, *F. nucleatum* was less able to compete in the three species community compared to either of the two species environments, based on the nutrient uptake and utilization considerations for carbohydrates and amino acids discussed below in the sections entitled “Glycolysis/Gluconeogenesis” and “Amino Acid Fermentation.” This may be the most significant observation of the entire study. The mixed results implying both synergistic and competitive interactions between *F. nucleatum* and the other common oral microbes provide insights into what might be expected in a more complex oral microbial community in vivo.

### Protein detection in community samples

A simple early-stage microbial community was produced by centrifugation into a pellet, a model originally developed by Merritt et al. (2005). The advantages of this system are that it is tightly controlled and reproducible, and large numbers of bacteria can be employed thus allowing downstream proteomic analyses. Exogenous nutrients were not added to the system, thus mimicking the periods of nutrient limitation that are common in the oral cavity. However, bacteria remain metabolically active and mobilize nutrient reserves during periods of low nutrient availability. Moreover, metabolic cross-feeding can occur, as we have demonstrated for other organisms in this model system (Kuboniwa et al. 2009; Hendrickson et al. 2012). The whole cell proteome of *F. nucleatum* was measured either alone (Fn) or in assembled communities with *S. gordonii* (FnSg), *P. gingivalis* (FnPg), or both *P. gingivalis* and *S. gordonii* (FnPgSg). *Fusobacterium nucleatum* proteins were considered identified in a sample if three or more unique peptides were detected for a protein in either of two biological replicates. Of 2067 predicted ORFs, (Kapatral et al. 2002) 1210 were detected in the Fn samples, 1224 in FnSg, 1192 in FnPg, and 1135 in FnPgSg.

Protein levels, as measured by spectral counting, were compared as relative abundance ratios among all conditions. A *q*-value cutoff of 0.005 or lower was assigned to determine significant change between conditions. The numbers of increased, decreased, and unchanged proteins for all six possible relative abundance comparisons are shown in Table1. The FnPg samples were not as reproducible as the other conditions, resulting in reduced statistical power for the FnPg comparisons, as shown in the summary tables, see the list of Supplementary Information for details. The other two and three organism comparisons show extensive (46 to 54%) changes in the proteome. Compared to Fn alone the community samples showed more proteins with reduced than increased levels. The relative abundance differences observed between the multiorganism samples, for example, FnPgSg/FnSg, were generally more balanced. The complete data sets, including all relative abundance ratios for each of the six comparisons, are presented at different levels of detail in the tables presented in the Supplementary Information. Included in the tables is information regarding protein coverage, numbers of peptides recovered (spectral counts, both raw, and normalized) and significance testing. Smaller subsets of the quantitative relative abundance data organized by protein class or putative function that parallel the discussion are also presented as Supplementary Tables. See the Supplementary Information at the end of the main text for a complete description of each Supplementary Table and other additional material.

**Table 1:** Significant differences between conditions.

Comparisons	Increased	Decreased	Unchanged
FnSg versus Fn	192	401	522
FnPg versus Fn	37	186	841
FnPgSg versus Fn	109	430	522
FnPgSg versus FnPg	76	35	921
FnSg versus FnPg	18	20	1074
FnPgSg versus FnSg	236	240	550

### Translation, ribosomal proteins, and tRNA synthetases

Previous studies of the other organisms, *P. gingivalis* and *S. gordonii*, indicated increased translation in mixed communities compared to each species alone (Kuboniwa et al. 2009; Hendrickson et al. 2012). In contrast, *F. nucleatum* appeared to have reduced translation in the mixed-species samples as seen in Table2. For FnSg compared to Fn 50 of the 78 detected proteins showed decreased levels with only 6 increased. FnPg versus Fn showed 19 of 82 decreased and only 1 increased. The case is weaker for FnPgSg. While 33 proteins showed decreased levels for FnPgSg versus Fn alone there were also 19 proteins with increased levels. While implying possibly reduced translation for Fn with Pg and Sg compared to Fn alone, the results would indicate that FnPgSg had higher translated protein levels than FnSg or FnPg. This is confirmed by the comparisons of FnPgSg to FnSg and FnPg, which showed 43 increased, 8 decrease and 15 increased, no decreased proteins, respectively.

**Table 2:** Translation, ribosomal proteins, and tRNA synthetases.

	FnPg versus Fn	FnSg versus Fn	FnPgSg versus Fn	FnPgSg versus FnPg	FnPg versus FnSg	FnPgSg versus FnSg
Translation^1^						
Total	9	9	9	9	9	9
Increased	0	1	2	2	1	4
Decreased	3	6	4	0	0	0
Unchanged	6	2	3	7	8	5
Ribosomal proteins^2^						
Total	46	43	49	47	43	43
Increase	1	2	14	11	0	28
Decrease	12	33	19	0	1	2
Unchanged	33	8	16	36	42	13
tRNA synthetases^3^						
Total	27	26	27	27	26	26
Increased	0	3	3	2	0	11
Decreased	4	11	10	0	2	6
Unchanged	23	12	14	25	24	9

1 Covers FN0327, FN0720, FN1287, FN1332, FN1546, FN1555, FN1556, FN1621, FN2020.

2 Covers FN0284, FN0325, FN0326, FN0329, FN0330, FN0430, FN0482, FN1117, FN1119, FN1282, FN1284, FN1285, FN1286, FN1364, FN1392, FN1437, FN1557, FN1558, FN1620, FN1623, FN1625, FN1626, FN1627, FN1628, FN1629, FN1630, FN1631, FN1632, FN1634, FN1635, FN1637, FN1638, FN1639, FN1640, FN1641, FN1642, FN1643, FN1644, FN1645, FN1646, FN1656, FN1657, FN1781, FN1828, FN1879, FN1979, FN2037, FN2038, FN2039, FN2040.

3 Covers FN0040, FN0054, FN0067, FN0069, FN0070, FN0110, FN0298, FN0299, FN0405, FN0466, FN0506, FN0611, FN0697, FN0753, FN0754, FN0755, FN1268, FN1340, FN1489, FN1517, FN1579, FN1597, FN1658, FN1977, FN2011, FN2122, FN2123.

Ribosomal proteins are noted to correlate with growth rates (Nomura et al. 1984). However, the cells were not given exogenous nutrients so growth in any of the samples is relatively unlikely. Thus, reduced growth in the mixed samples compared to Fn alone is not a favored explanation. It is more likely that the mixed cultures experienced reduced protein synthesis, a result in keeping with the predominance of reduced over increased protein levels seen in Table1. This is in sharp contrast to the results seen with *P. gingivalis* and *S. gordonii*. Pg showed increased protein synthesis machinery in the presence of Sg and Fn and Sg showed increased levels in the presence of Pg, though not Fn, possibly indicating cross-feeding from Pg but not Fn. There are two likely explanations for changes in overall metabolism. First, they reflect a response to changes in available nutrients. However, as no exogenous nutrients were supplied, this explanation is unlikely. Second, these species, which share the same ecological niche within the oral cavity, have developed programed responses to the presence of other organisms with the potential for nutritional cross-feeding. This latter concept is supported by the finding that *S. gordonii* showed protein responses indicative of high sugar levels in the mixed communities in the absence of exogenous sugar (Hendrickson et al. 2012).

### Amino acid fermentation

Energy metabolism in *F. nucleatum* is a complex interplay of amino acids, sugars, and intracellular polyglucose (Bolstad et al. 1996). Amino acid fermentation is the preferred energy source. Though the exact amino acids that can be utilized by each strain varies, glutamate, histidine, and aspartate fermentation are common (Bakken et al. 1989; Bolstad et al. 1996). ATCC 25586 appears to have proteins to utilize glutamate, glutamine, aspartate, asparagine, histidine, lysine, serine, cysteine, methionine, threonine, alanine, and glycine (Kapatral et al. 2002). Glutamate and histidine are likely employed before other amino acids. While strain ATCC 25586 has not been examined, in all of the *F. nucleatum* strains tested glutamate and histidine were utilized first (Bakken et al. 1989).

*Streptococcus gordonii* is a saccharolytic organism and is unlikely to directly compete for amino acids in terms of energy metabolism, so the widespread changes in amino acid metabolism were unexpected. There was no real change in the pathway from histidine to glutamate in the presence of Sg, though there was a trend toward higher levels in one protein (FN0792) that did not reach the level of statistical significance (see Fig. S2). There may have been a shift from 5,10 methenyl THF toward formimide as a byproduct of histidine fermentation. However, further breakdown along the glutamate fermentation pathway appeared to be reduced, as did glutamine, asparagine, aspartate, serine, and lysine fermentation. There was an increase in the pathway from pyruvate to butanoate. This may come from a shift away from acetate as an end product. Pyruvate could also be provided by other sources. Some possible sources are alanine, glycine, threonine, or methionine. The pathway for fermenting these amino acids to pyruvate appeared to be increased in the presence of Sg (Fig. S8). The pathway to propionate appeared to be down, though the proteins are shared with acetate production from pyruvate, and a reduction in either pathway might have been the primary driving force for the lower protein levels. If, like other strains, ATCC 25586 preferentially utilizes histidine and glutamate the implication is low concentrations of these amino acids. With the pathway to acetate down, but the pathway to butanoate increased, the pathway for the preferred amino acids and several others down, but some amino acid pathways increased, the overall picture is one of multiple adjustments in response to energy availability.

In contrast to Sg, *P. gingivalis* is an asaccharolytic organism that derives energy from amino acid fermentation, so could provide a direct competition for amino acids (Bakken et al. 1989). Conversely, the extensive endopeptidase activities of Pg could provide peptides for the growth of Fn (Rogers et al. 1992) and biofilm formation by Fn is stimulated by the presence of Pg (Saito et al. 2008). Energy pathway regulation in Fn tends to be very dependent on exact conditions (Robrish et al. 1987; Rogers et al. 1998) and Pg may help or hinder Fn amino acid fermentation, depending on the relative numbers of different bacteria and growth conditions of the biofilm.

In the FnPg community histidine to glutamate again appeared unchanged, but glutamate fermentation appeared to be reduced (Fig. S1). Reductions were also seen in glutamine, serine, and lysine fermentation. No real change was seen in the alanine, glycine, threonine, cysteine, or methionine pathways to pyruvate (Fig. S7). The same shift away from acetate toward butanoate as an end point for pyruvate metabolism was observed. The results were consistent with an overall reduction in amino acid fermentation in the presence of Pg.

The presence of both Sg and Pg produced different results then either organism alone. While Pg did not change the histidine to glutamate pathway and Sg might have slightly increased it, in the FnPgSg community histidine fermentation was reduced (Fig. S3). There also appeared to be an increase rather than a decrease in the pathway to 5,10 methenyl THF. Another striking difference was a possible increase, rather than decrease, in lysine fermentation. More in keeping with the two species communities were decreases in glutamate, serine, asparagine, and aspartate fermentation as well as an increase in the pathway to butanoate as a byproduct. The pathway from acetyl-CoA to acetate appeared unchanged, but pyruvate to lactate was reduced. This may represent a shift from lactate to butanoate production rather than the acetate to butanoate shift seen with FnPg and FnSg. In contrast to FnSg, the three species community had reduced glycine fermentation and possibly reduced cysteine fermentation (Fig. S9). Like FnSg, methionine fermentation appeared to be increased. The mixture of increased and decreased fermentation pathways implied changes in amino acid consumption with an emphasis on lysine and methionine fermentation. The overall effect on energy availability was unclear, though glycolysis also appeared to be reduced (see Glycolysis/gluconeogenesis below).

The differences seen in FnPgSg versus Fn and FnSg versus Fn were generally supported by the FnPgSg versus FnSg comparisons (Figs. S6, S12). The comparisons between FnPg and FnSg or FnPgSg showed few statistical differences (Figs. S4, S5, S10, S11).

Most amino acid fermentation pathways were reduced in the mixed community samples compared to Fn alone. This included the glutamate pathway even though glutamate and histidine are likely the favored amino acids for fermentation (Bakken et al. 1989) and histidine fermentation feeds into the glutamate pathway. Glutamate is also a preferred metabolite for Pg (Takahashi et al. 2000) so competition for this amino acid might be an explanation in communities with Pg, but FnSg also showed decreases in the glutamate pathway. Some pathways showed increased levels in FnPgSg, lysine, and methionine, or FnSg, alanine, glycine, cysteine, methionine, and threonine, so there appeared to be decided shifts in amino acid availability or usage but the overall effect on energy availability is unclear.

The byproducts of energy metabolism can alter pH and have cytotoxic effects on host cells (Singer and Buckner 1981; Bradshaw and Marsh 1998; Lamont and Jenkinson 1998). Extensive shifts in end products were seen with the Sg community samples (Hendrickson et al. 2012). Thus, it was unsurprising to see what appeared to be shifts from acetate toward butanoate in FnPg and FnSg and from lactate toward butanoate in FnPgSg.

### Glycolysis/gluconeogenesis

*Fusobacterium* species have often been referred to as weakly saccharolytic (Bolstad et al. 1996). Glucose and galactose are not transported into the cells via the classical phosphoenolpyruvate-dependent phosphotransferase system (PTS), but rather by an, as yet unidentified, amino acid-dependent transport system (Robrish et al. 1987). On the other hand, fructose appeared to employ a PTS (Robrish and Thompson 1990) and the ATCC 25586 genome encodes for a fructose-specific PTS component homolog (FN1441). Because of the amino acid dependence, glucose and galactose were not utilized strongly in the absence of amino acids. In the presence of amino acids, *F. nucleatum* ATCC 10953 utilized glucose and galactose, but to form intracellular polyglucose rather than to produce energy (Robrish et al. 1987). The resulting granules could be observed by electron microscopy (Robrish and Thompson 1990). Only when amino acid fermentation ceased did the cells employ the polyglucose for energy (Robrish and Thompson 1988). Fructose was not dependent on amino acid availability and ATCC 10953 could grow on fructose as the sole energy source (Robrish and Thompson 1990).

**Figure 1:**
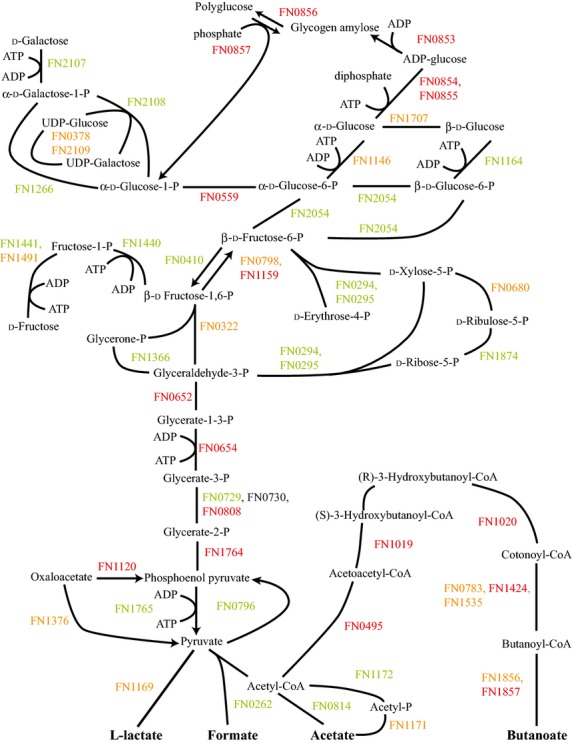
FnSg versus Fn energy metabolism and end products. Proteins catalyzing each step are shown by their *Fusobacterium nucleatum* FN number. Red numbers indicate increased levels in the first condition compared to the second condition, green decreased levels, yellow no statistical change, and black undetected in at least one of the conditions.

The presence of Sg in the FnSg community resulted in significant alterations in glycolysis and associated pathways, as seen in Figure1. This mixed culture showed decreases in proteins for fructose and galactose utilization compared to Fn alone. Galactose transport has been shown to be amino acid dependent, but Robrish et al. found that in ATCC 10953 glutamate caused a reduction in fructose-1-phosphate kinase activity (FN1440 for ATCC 25586) (Robrish et al. 1987; Robrish and Thompson 1990). These studies imply that fructose and galactose should be counter regulated. Regulation may be different in ATCC 25586 or there may be a second level of regulation based on substrate availability. Proteins for the pentose phosphate pathway were also mostly reduced.

Proteins in the glycolysis pathway and those for synthesizing and utilizing polyglucose were mostly increased compared to Fn alone. There are two likely explanations for these changes. The bacteria might have been mobilizing stored polyglucose for energy. The studies here were done in buffer without exogenous nutrients and the communities left for 18 h before sampling. If Fn had built significant quantities of polyglucose during growth it would be expected to mobilize those resources once deprived of other energy sources. If this were the case, however, it would be expected that Fn alone would also catabolize the polyglucose resulting in no difference between FnSg and Fn. It's possible that FnSg had a stronger mobilization response resulting in the higher protein levels.

The second likely explanation is an increase in gluconeogenesis and polyglucose synthesis rather than energy production. Increased polyglucose synthesis occurs when Fn has sources of both sugar and amino acids. Given the lack of exogenous nutrients in the experimental conditions and in a community with Sg, which could act as competition for sugars, it is unclear where these nutrients would originate. Arguing against gluconeogenesis were reductions in glutamate and lysine fermentation pathways, with histidine fermentation feeding into the reduced glutamate pathway (Fig. S2). In ATCC 10953 glucose accumulation was supported primarily by glutamate and histidine with lysine supporting significant accumulation only after glutamate was depleted (Robrish et al. 1987). Also, the reduction in ribosomal and translational proteins would argue against energy sufficiency. However, FnSg did show increases in the pathways for alanine, threonine, glycine, cysteine, and methionine fermentation (Fig. S8). Sugar transport might be determined more by energy availability from amino acids rather than specific fermentation pathways (Robrish et al. 1987; Bakken et al. 1989). Arguing for increased gluconeogenesis, FnSg showed decreased levels of 6-phosphokinase (FN0410) catalyzing the pathway in the direction of glycolysis but increased levels for one of the two fructose-1,6-bisphosphatase (FN1159) catalyzing gluconeogensis.

**Figure 2:**
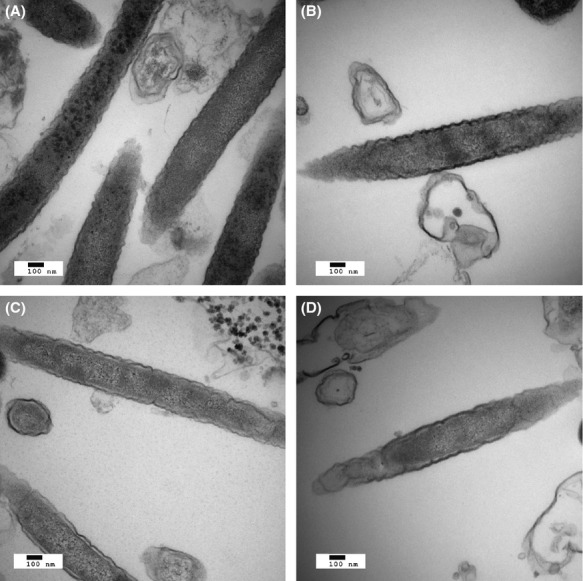
Electron micrographs. Thin-section transmission electron micrographs after 18 h in a model biofilm of (A) *Fusobacterium nucleatum* alone, (B) *F. nucleatum* with *Porphyromonas gingivalis*, (C) *F. nucleatum* with *Streptococcus gordonii*, or (D) *F. nucleatum* with both *P. gingivalis* and *S. gordonii*. Magnification: ×49,000.

In order to examine these possibilities further, Fn samples were subject to electron microscopy, looking for polyglucose granules in Fn alone and the mixed samples (Fig.2). While granules were clearly visible in Fn alone (Fig.2A) the mixed communities showed fewer if any granules. Increased gluconeogenesis would be expected to produce more granules in FnSg (Fig.2C) than in Fn alone, arguing for an increase in glycolysis instead. Conclusive evidence would require a time course measurement of the glycolysis/gluconeogenesis metabolites.

**Figure 3:**
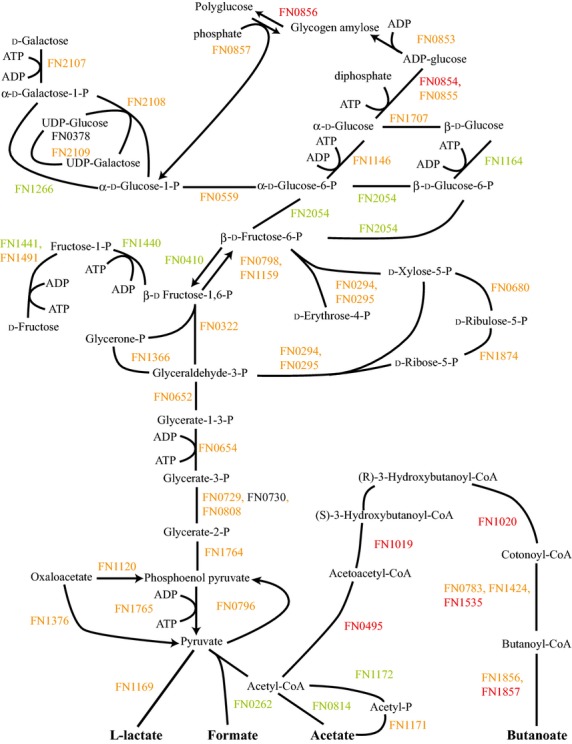
FnPg versus Fn energy metabolism and end products. The diagram shows a schematic of the glycolysis and pentose phosphate pathways for Fn including the end products of the metabolism, formate, acetate, l-lactate, and butanoate, for the *Fusobacterium nucleatum* with *Porphyromonas gingivalis* sample compared to *F. nucleatum*. Labels and color coding as described for Figure1.

FnPg compared to Fn alone showed a small number of differences (Fig.3). Again, proteins for fructose utilization were down. However, galactose utilization remained mostly unchanged. Some increase in proteins for polyglucose synthesis was also seen as well as a decrease in 6-phosphokinase (FN0410). This might indicate a slight increase in polyglucose synthesis in the community with Pg, though FnPg showed unchanged or decreased levels for all amino acid fermentation proteins (Figs. S1, S7) and the ribosomal and translation proteins implied lower energy availability (see above). Nor did FnPg show an increase in polyglucose granules (Fig.2B).

**Figure 4:**
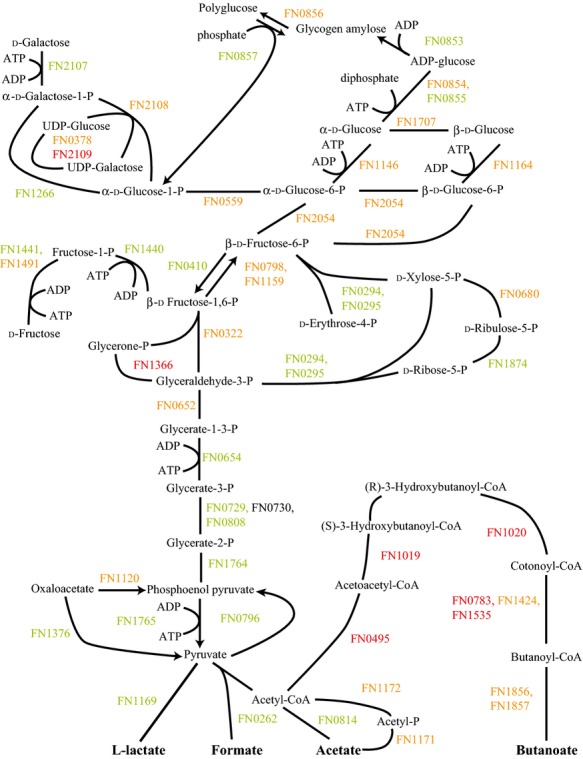
FnPgSg versus Fn energy metabolism and end products. Labels and color coding as described for Figure1, for the *Fusobacterium nucleatum* with *Porphyromonas gingivalis* and *Streptococcus gordonii* comparison to *F. nucleatum*.

In sharp contrast to communities of Fn with only Sg or only Pg, in the three species community Fn showed significant decreases in almost all carbohydrate metabolism proteins (Fig.4). Reductions were seen in fructose and galactose utilization, glycolysis, the pentose phosphate pathway, and polyglucose synthesis. This implied a significant reduction in carbohydrate catabolism. This might be the result of suppression by amino acids. In ATCC 10953, glycolysis of stored polyglucose was suppressed by glutamate, histidine, and lysine (Robrish and Thompson 1988). However, this should also lead to increased polyglucose synthesis (Schulz and Schumann 1996) while the results showed decreased levels in several of the proteins for polyglucose accumulation and a decrease was seen in polyglucose granules (Fig.2D). As discussed under amino acid fermentation, FnPgSg also showed decreased levels compared to Fn alone in almost all amino acid pathways (Figs. S3, S9). The two exceptions were methionine and lysine. Fn may be significantly nutrient restricted in the three species community and poorly competitive for sugars from Sg and for amino acids from Pg.

Relatively few proteins made the cutoff for significant change between FnPg and the other multispecies communities, see Figures6. Figure7 shows the comparison between FnPgSg and FnSg. While both FnPgSg and FnSg had reduced fructose, galactose, and pentose phosphate pathway proteins compared to Fn alone (Figs.4) this showed that the reduction in FnPgSg was not as extensive as the reduction in FnSg.

**Figure 5:**
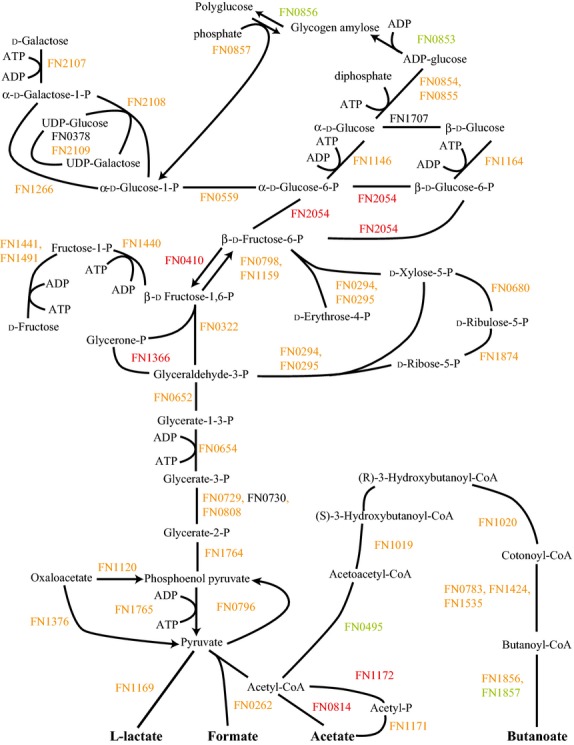
FnPgSg versus FnPg energy metabolism and end products. Labels and color coding as described for Figure1, for the *Fusobacterium nucleatum* with *Porphyromonas gingivalis* and *Streptococcus gordonii* comparison to *F. nucleatum* with *P. gingivalis*.

**Figure 6:**
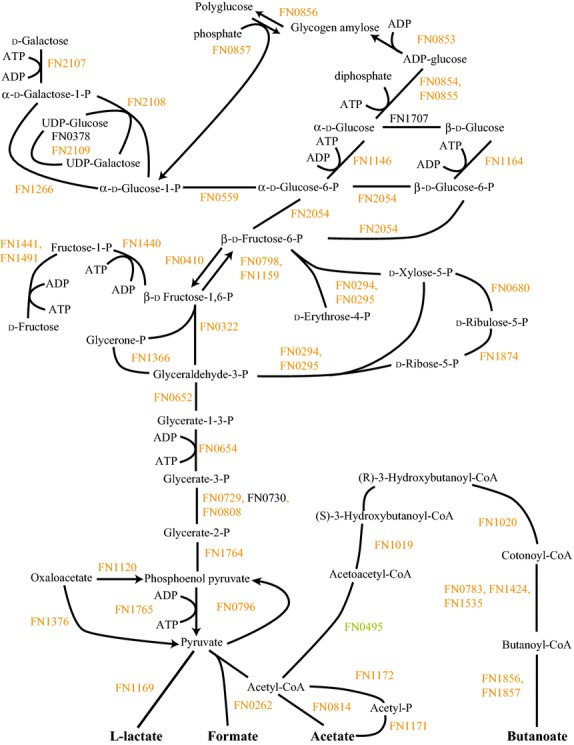
FnSg versus FnPg energy metabolism and end products. Labels and color coding as described for Figure1, for the *Fusobacterium nucleatum* with *Streptococcus gordonii* comparison to *F. nucleatum* with *Porphyromonas gingivalis*.

**Figure 7:**
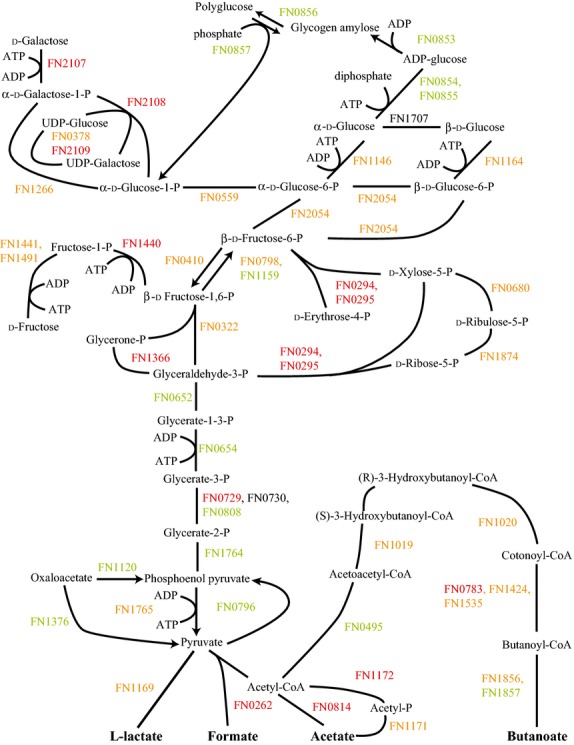
FnPgSg versus FnSgEnergy metabolism and end products. Labels and color coding as described for Figure1, for the *Fusobacterium nucleatum* with *Porphyromonas gingivalis* and *Streptococcus gordonii* comparison to *F. nucleatum* with *S. gordonii*.

### Ethanolamine metabolism

Another possible energy source that is often associated with pathogenesis is ethanolamine (Garsin 2010). Ethanolamine can be derived from phosphatidylethanolamine, a lipid found in biological membranes. While Fn does not appear to be able to break down phosphatidylethanolamine directly, it does code for three glycerolphosphoryl diester phosphodiesterase proteins (FN1891, FN1908, and FN1954) that can provide ethanolamine from glycerophosphoethanolamine, a breakdown product of phosphatidylethanolamine. While ethanolamine can diffuse through the membrane under low pH conditions, diffusion has been amplified by ethanolamine permease (FN0089) (Garsin 2010).

Ethanolamine ammonia-lyase (FN0079, FN0080) catalyzes the conversion of ethanolamine into ammonia and acetaldehyde. The acetaldehyde can then be converted into acetyl-CoA by acetylaldehyde dehydrogenase (FN0084) which can in turn be converted to acetate and ATP. In many organisms, the reaction appears to take place in a microcompartment formed by associated proteins (FN0081-0083, FN0087). The catalytic enzymes are reactivated by another protein (FN0078) and require an adenosylcobalamin cofactor formed from cobalamin by ethanolamine utilization cobalamin adenosyltransferase (FN0085). The proteins are commonly expressed as an operon controlled by a two-component regulator (FN0076-0077) in response to ethanolamine.

Table S9 shows that most of the proteins from the ethanolamine utilization operon remained undetected. However, the catalytic enzymes were detected (FN0079-0084) and the detected proteins showed similar expression patterns. Most showed an increase in FnSg versus Fn alone, and possibly in FnPg though it did not make the statistical cutoff, and a decrease in FnPgSg compared to Fn. Once again, Fn in the three organism community displayed changes not seen in either of the two organism pellets. The two detected glycerophosphoryl diester phosphodiesterases (FN1891, FN1908) also showed a similar pattern. The cobalamin adenosyltransferase (FN0085) was undetected and cobalamin biosynthesis proteins were relatively unchanged, though cobalamin would also be utilized in other pathways.

Fn in the presence of Sg, and possibly Pg, appeared to utilize ethanolamine. This was presumably derived from the breakdown of bacterial cell membranes. Interestingly, the presence of both Pg and Sg appeared to shut down the pathway. This could be due to reduced membrane breakdown in the three species community or Pg and Sg together might out compete Fn for this resource, though neither of those organisms encodes for an ethanolamine utilization operon.

### ATP synthases

The ATP synthases can convert H^+^ gradients into ATP and may serve as an indicator of the cell's energy metabolism. Run in the other direction, the ATP synthases can use energy to produce an acidic environment. The ATCC 25586 genome predicts two ATP synthase complexes (FN0357-0364, FN1734-1741) though one (FN1734-1741) is a V-type sodium ATP synthase that is primarily involved in sodium export (Hosaka et al. 2006). Table3 shows the results for the ATP synthases. For the ATP synthase, only the three species community showed a significant directional change with 3 of the 7 detected proteins having reduced levels indicating that energy from the proton gradient is likely reduced. The V-type sodium ATP synthase also appeared to be decreased in the three species community but increased in FnSg compared to Fn alone. This might indicate increased sodium export in the FnSg community and decreased export in FnPgSg.

**Table 3:** ATP synthases.

	FnPg versus Fn	FnSg versus Fn	FnPgSg versus Fn	FnPgSg versus FnPg	FnPg versus FnSg	FnPgSg versus FnSg
ATP synthase^1^						
Total	7	6	7	7	6	6
Increased	1	1	0	0	0	0
Decreased	1	1	3	0	0	2
Unchanged	5	4	4	7	6	4
V-type sodium ATP synthase^2^						
Total	6	6	5	5	7	5
Increase	1	4	0	1	2	0
Decrease	0	0	3	1	0	3
Unchanged	5	2	2	3	5	2

1 Covers FN0357, FN0358, FN0359, FN0360, FN0361, FN0362, FN0364.

2 Covers FN1734, FN1735, FN1737, FN1738, FN1739, FN1740, FN1741.

### Adhesion and outer membrane proteins

One property of *F. nucleatum* is promiscuity in coadhesion with many different oral microbes (Kolenbrander et al. 1989). In order to obtain the large sample sizes needed for these experiments, the pellets were produced by centrifugation, but there is no indication that once in cell–cell contact the organisms do not use the known adhesion factors for interaction. In *S. gordonii*, we found significant reduction in adhesion proteins in the mixed nascent communities compared to Sg alone (Hendrickson et al. 2012). We hypothesized that after 18 h in close association with other organisms Sg no longer needed these molecules for aggregation (Hendrickson et al. 2012).

The FadA protein (FN0264) has been shown to be important for binding to host cells and for invasion (Han et al. 2005; Ikegami et al. 2009). As seen in Table S10 and in the overall results given in Table S1, FadA is significantly lower in FnSg and FnPgSg than in Fn alone, though the magnitude of the FnPgSg change is relatively small. ATCC 25586 also contains a FadA paralog FN1529 (Han et al. 2005). This protein showed a significant reduction in FnSg compared to Fn and to FnPgSg. Fn has also been shown to bind to fibronectin on gingival cells (Babu et al. 1995). A fibronectin binding protein homolog FN0682 showed no change in the community samples however.

The Fn outer membrane protein FomA (FN1859) is a nonspecific porin which acts not only as a virulence factor and major immunogen but also plays a role in binding to *Streptococcus sanguinis* and *P. gingivalis* (Liu et al. 2010). As seen in Table S10, FN1859 showed a significant reduction in FnPgSg versus Fn, though possibly an increase in FnSg. If FomA is important for binding to both streptococcal species and Pg, then the presence of both partners in a mixed pellet may explain the reduced levels in FnPgSg. The arginine inhibitable adherence protein RadD (FN1526) is important for binding a number of streptococcal species, including *S. gordonii*, and *Actinomyces naeslundii* (Kaplan et al. 2009). RadD levels were lower in FnPgSg and FnSg than with Fn alone, though the levels in FnSg were much lower than those in FnPgSg.

In addition to the known adhesins, Fn 25586 expresses a number of outer membrane proteins with no known function. Some of these proteins may also function in the well-known broad binding shown by Fn species. Table4 summarizes the detected changes in these outer membrane proteins. Several of these are annotated as porins (FN0335, FN1003, FN1124) and presumably function in membrane transport. However, the known adhesin FomA is also a porin (Liu et al. 2010) and these proteins might also act in binding. Approximately half of the detected proteins are down in the mixed cultures compared to Fn alone. This is different from the more specific decreases discussed for the known adhesins and might argue that these proteins do not function in cell binding.

**Table 4:** Other outer membrane proteins.

	FnPg versus Fn	FnSg versus Fn	FnPgSg versus Fn	FnPgSg versus FnPg	PgSg versus FnPg	FnPgSg versus FnSg
Total	14	15	15	14	14	15
Increased	0	2	0	2	0	2
Decreased	7	8	8	0	0	2
Unchanged	7	5	7	12	14	11

Covers FN0254, FN0335, FN0387, FN0394, FN1003, FN1124, FN1265, FN1449, FN1554, FN1893, FN1905, FN1911, FN2047, FN2058. FN0253, FN2048, andFN2059 are duplicates and indistinguishable at the protein level, they are counted as one protein.

### Lipopolysaccharides

Other outer membrane components that often play a role in pathogenicity are the lipopolysaccharides. As seen in Table5, the lipopolysaccharide biosynthetic proteins are generally decreased in the mixed-species communities compared to Fn alone. This may be a response to limit the toxic effects or triggered immune response of these molecules, though it might also be part of a more general reduction in membrane and cell wall components.

**Table 5:** Lipopolysaccharide synthesis.

	FnPg versus Fn	FnSg versus Fn	FnPgSg versus Fn	FnPgSg versus FnPg	PgSg versus FnPg	FnPgSg versus FnSg
Total	12	12	12	12	12	12
Increased	0	0	1	0	0	3
Decreased	3	7	7	1	1	4
Unchanged	9	5	4	11	11	5

Covers FN0213, FN0502, FN0544, FN0593, FN0595, FN0597, FN0807, FN1016, FN1130, FN1224, FN1240, FN1606, FN1703, FN1909.

### Cell wall, fatty acid, and phospholipid biosynthesis

*Fusobacterium nucleatum* species have an unusual peptidoglycan forming their cell walls. Rather than *meso*-diaminopimelic acid *F. nucleatum* employs *meso*-lanthionine (Bolstad et al. 1996). Table6 summarizes the changes seen in cell wall biosynthetic proteins. All mixed-species communities showed reduced protein levels, with FnPgSg showing 7 of 15 proteins with lower levels, though one showed increased levels. Fatty acid biosynthetic proteins are summarized in Table7. They appear somewhat reduced in Fn with either Pg or Sg but not in the presence of both (FnPgSg). Phospholipid biosynthesis is summarized in Table8. Phospholipid synthesis appeared to be down in FnSg and FnPgSg compared to Fn alone. Two proteins, CDP-diacylglycerol serine-O-phosphatidyltransferase (FN0991) and phosphotidylserine decarboxylase (FN0347), that synthesize the ethanolamine component of the cellular membrane showed different regulation. FN0347 had increased levels in FnPgSg versus Fn alone while FN0991 showed reduced levels in FnPgSg and FnSg compared to Fn. In general, cell wall peptidoglycan synthesis appeared to be reduced in the mixed communities, fatty acid synthesis was reduced in the presence of either Sg or Pg but not in the presence of both, while phospholipid biosynthesis was reduced in FnSg and FnPgSg. While the exact pathways reduced varied with bacterial partner, there was a reduction in components for cell wall and membranes in the mixed communities.

**Table 6:** Cell wall biosynthesis.

	FnPg versus Fn	FnSg versus Fn	FnPgSg versus Fn	FnPgSg versus FnPg	PgSg versus FnPg	FnPgSg versus FnSg
Total	16	17	15	15	16	15
Increased	0	0	1	1	0	3
Decreased	5	6	7	0	0	0
Unchanged	11	11	7	14	16	12

Covers FN0060, FN0406, FN0525, FN0580, FN1155, FN1161, FN1211, FN1225, FN1326, FN1454, FN1455, FN1456, FN1457, FN1458, FN1461, FN1520, FN1991.

**Table 7:** Fatty acid biosynthesis.

	FnPg versus Fn	FnSg versus Fn	FnPgSg versus Fn	FnPgSg versus FnPg	PgSg versus FnPg	FnPgSg versus FnSg
Total	11	11	10	10	11	10
Increased	0	0	1	3	0	5
Decreased	3	5	2	0	0	0
Unchanged	8	6	7	7	11	5

Covers: FN0147, FN0148, FN0149, FN0150, FN0151, FN0174, FN0408, FN0409, FN0494, FN0594, FN1850.

**Table 8:** Phospholipid biosynthesis.

	FnPg versus Fn	FnSg versus Fn	FnPgSg versus Fn	FnPgSg versus FnPg	PgSg versus FnPg	FnPgSg versus FnSg
Total	13	13	13	13	13	13
Increased	0	1	1	0	0	2
Decreased	1	6	6	2	0	3
Unchanged	12	6	6	11	13	8

Covers: FN0147, FN0347, FN0593, FN0594, FN0595, FN0597, FN0906, FN0991, FN1130, FN1781, FN1909.

### Cell division

As mentioned above, the samples were maintained without exogenous nutrients for 18 h before collecting cells for the proteomics measurements. Under these conditions, it is expected that cell division in all of the samples would be extremely low, resulting in no difference between Fn alone and Fn in the mixed communities. However, Fn can store carbon and energy in polyglucose and may employ this resource over the course of the incubation. Table9 shows a summary of the proteins for DNA replication and cell division. There appears to be a general decrease in proteins for DNA replication in the mixed communities compared to Fn alone. In keeping with this result, there also appeared to be a decrease in proteins for cell division in the presence of other species. While FnSg did show three proteins with increased levels compared to Fn alone two of these, FN0175 and FN0176, are cell division inhibitors, though the other is the important cell division protein FtsZ (FN1451). The FnPgSg versus FnSg results indicated that levels were even lower in FnSg than the reduced levels seen in FnPgSg. While it seems unlikely that cell division is occurring at a rate where it could be significantly reduced in the mixed communities, the results imply reduced cell division, or a programed response to reduce the machinery for cell division. This would be consistent with the reduced levels seen for peptidoglycan biosynthesis, fatty acid biosynthesis, and the general reduction in outer membrane protein levels.

**Table 9:** DNA replication and cell division.

	FnPg versus Fn	FnSg versus Fn	FnPgSg versus Fn	FnPgSg versus FnPg	PgSg versus FnPg	FnPgSg versus FnSg
DNA replication^1^						
Total	14	14	15	15	15	15
Increased	0	1	1	0	0	3
Decreased	3	8	9	0	0	4
Unchanged	11	5	5	15	15	8
Cell division^2^						
Total	21	21	20	20	20	19
Increase	0	3	1	1	2	7
Decrease	4	8	7	0	0	3
Unchanged	17	10	12	19	18	9

1 Covers FN0102, FN0118, FN0281, FN0536, FN0617, FN0633, FN0705, FN1069, FN1304, FN1319, FN1383, FN1581, FN1614, FN1717, FN1827, FN1830.

2 Covers FN0022, FN0175, FN0176, FN0177, FN0265, FN0562, FN1010, FN1155, FN1211, FN1225, FN1326, FN1451, FN1452, FN1455, FN1456, FN1457, FN1458, FN1461, FN1520, FN1978, FN2013, FN2017.

### Stress

Studies with the other two organisms, Pg and Sg, found evidence of physiological support between the species (Kuboniwa et al. 2009; Hendrickson et al. 2012). DNA repair proteins were reduced in both Pg and Sg in mixed communities. Sg showed increased antioxidant activity in communities with Fn or Pg which may provide protection for the more oxygen sensitive species (Hendrickson et al. 2012). Fn has been shown to provide such protection to Pg in chemostat cultures (Diaz et al. 2002). Pg in chemostats sparged with oxygen failed to grow, but a mixed Pg plus Fn culture exposed to the same oxygen levels showed Pg growth.

As with the other organisms, Fn appeared to have reduced levels of DNA repair proteins in the mixed communities compared to Fn alone (Table10). While 13 of the 26 detected proteins were reduced in FnSg versus Fn the reduction did not seem as extensive as in the FnPgSg samples. Three proteins showed increased levels in FnSg versus Fn and FnPgSg showed more reduced than increased levels compared to FnSg.

**Table 10:** Stress proteins.

	FnPg versus Fn	FnSg versus Fn	FnPgSg versus Fn	FnPgSg versus FnPg	PgSg versus FnPg	FnPgSg versus FnSg
DNA repair^1^						
Total	24	26	23	23	24	23
Increased	0	3	0	0	1	5
Decreased	7	13	14	0	0	7
Unchanged	17	10	9	23	23	11
Antioxidant activity^2^						
Total	6	7	7	6	6	7
Increase	0	1	4	1	0	3
Decrease	2	4	1	0	0	0
Unchanged	4	2	2	5	6	4
Other stress proteins^3^						
Total	9	10	9	9	9	9
Increase	1	4	1	0	1	1
Decrease	1	2	2	2	0	3
Unchanged	7	4	6	7	8	5

1 Covers: FN0019, FN0047, FN0065, FN0117, FN0157, FN0158, FN0224, FN0268, FN0297, FN0374, FN0462, FN0524, FN0547, FN0592, FN0622, FN0693, FN0705, FN0962, FN1103, FN1149, FN1217, FN1226, FN1304, FN1581, FN1660, FN1717, FN1864, FN2018.

2 Covers: FN1088, FN1123, FN1163, FN1983, FN1984, FN2007, FN2067.

3 Covers: FN0113, FN0114, FN0116, FN0118, FN0221, FN0321, FN0722, FN1079, FN1660, FN1941.

Antioxidant activity showed an interesting pattern. Protein levels appeared to be reduced in FnSg versus Fn alone, possibly due to the increased levels seen in Sg in the FnSg culture (Hendrickson et al. 2012). However, antioxidant levels might also have been down in FnPg versus Fn, in sharp contrast to what would be expected from the chemostat oxygen experiments. FnPgSg on the other hand did appear to have increased antioxidant proteins compared to Fn alone or FnSg. Given the increased levels of antioxidant proteins in Sg in the FnPgSg community (Hendrickson et al. 2012), it is unclear why Fn would have higher levels in FnPgSg but not in FnPg where Fn would be the only non-Pg source for antioxidant activity.

### Proteolysis

In addition to normal housekeeping functions, Fn employs proteases for nutrition and virulence (Bolstad et al. 1996). As discussed above, the preferred energy source for Fn is amino acid fermentation (Bolstad et al. 1996). However, Fn is known for a relatively low amount of endopeptidase activity that would be needed to break down peptides and proteins from the environment (Grenier 1994; Diaz et al. 2002). It has been suggested that the large amounts of extracellular endopeptidase produced by Pg increases amino acid availability for Fn and provides synergism between the organisms (Rogers et al. 1992) and that this explains why, while large amounts of trypsin, chemotrypsin, or peptidase K are bacteriocidal, small amounts actually increase Fn growth (Grenier 1994). Proteases can also act as virulence factors (Bolstad et al. 1996) destroying host tissue. For example, Fn 25586 encodes for two homologs of Protease IV (FN0873, FN1271) that has been shown to be a factor in *Pseudomonas aeruginosa* virulence and degrades a variety of molecules associated with the innate and humoral immune responses to infection (Engel et al. 1998).

Table11 summarizes the results for the protease proteins. All of the mixed samples showed a sizable number of proteins with decreased levels compared to Fn alone. FnSg does stand out in that several proteases also showed increased levels, though those proteins (FN0590, FN0873, FN1186, FN1280, FN1281, FN1906) are spread across a number of different functions. Of the three annotated endopeptidases (FN0266, FN0477, FN1280), one (FN0477) showed decreased levels in all mixed samples, though not to the same extent in FnSg, while another (FN1280) showed significant increases in both FnPg and FnSg compared to Fn alone but not in FnPgSg. Reduced levels in the presence of Pg could be explained by Pg supplying exopeptidase activity but that is not the pattern seen in the cultures and changes in endopeptidase levels are presumably being driven by some other factor.

**Table 11:** Proteolysis.

	FnPg versus Fn	FnSg versus Fn	FnPgSg versus Fn	FnPgSg versus FnPg	PgSg versus FnPg	FnPgSg versus FnSg
Total	38	37	35	34	36	32
Increased	1	6	1	3	2	8
Decreased	12	15	17	2	2	10
Unchanged	25	16	17	29	32	14

Covers FN0060, FN0061, FN0266, FN0278, FN0370, FN0477, FN0549, FN0583, FN0590, FN0733, FN0752, FN0775, FN0873, FN0887, FN0920, FN0928, FN1029, FN1063, FN1128, FN1145, FN1186, FN1205, FN1271, FN1277, FN1280, FN1281, FN1297, FN1322, FN1426, FN1449, FN1728, FN1804, FN1826, FN1906, FN1931, FN1950, FN1978, FN2014, FN2016, FN2100.

### Protein secretion

As seen in Table S11, the detected protein secretion proteins were virtually all decreased in mixed communities compared to Fn alone. The one exception was general secretion pathway protein G (FN2093) in FnPg versus Fn which showed reduced levels but did not make the statistical cutoff. For three proteins (FN0699, FN0700, FN0826), FnPgSg showed reduced levels compared to Fn alone but statistically higher compared to FnSg. Given the general reduction in translational machinery and outer membrane proteins discussed above, the reduction in proteins for protein secretion may simply reflect reduced need for protein export.

### Transporters

Table12 shows a general reduction in transport proteins. The Fn plus Sg community did show eight proteins with increased levels when compared to Fn alone. Three of these (FN0375, FN0376, FN0308) are iron transporters and as seen in the table iron transport may be increased in the presence of Sg. Three of the increased proteins are dipeptide transporters (FN0998, FN1359, FN1363). However, three of the ten detected dipeptide transporters showed decreased levels in FnSg. It is unknown what substrate specificity may be displayed by the different dipeptide transporters. As mentioned under amino acid fermentation Fn in the presence of Sg seems to shift away from glutamate, glutamine, aspartate, asparagine, serine, and lysine fermentation but had increased protein levels for alanine, glycine, threonine, and methionine fermentation. The mixed results for dipeptide transporters might represent a shift in substrates to favor the amino acids being utilized in the presence of Sg. While amino acid fermentation is preferred over glycolysis for energy production, *F. nucleatum* favors dipeptides rather than amino acids as an amino acid source (Bolstad et al. 1996). The amino acid transporters showed few consistent changes in the mixed communities, though more showed reduced than increased levels in Fn with Pg and Sg.

**Table 12:** Transporters.

	FnPg versus Fn	FnSg versus Fn	FnPgSg versus Fn	FnPgSg versus FnPg	PgSg versus FnPg	FnPgSg versus FnSg
Transporters[Table-fn tf12-1]						
Total	54	54	59	57	56	55
Increased	0	8	4	2	3	10
Decreased	11	17	21	3	1	15
Unchanged	43	29	34	52	52	30
ABC transporters[Table-fn tf12-2]						
Total	17	18	18	17	17	18
Increase	0	0	0	2	0	4
Decrease	5	8	8	0	1	4
Unchanged	12	10	10	15	16	10
Amino acid transporters[Table-fn tf12-3]						
Total	6	6	6	6	6	6
Increase	0	1	1	0	0	2
Decrease	0	1	2	0	0	0
Unchanged	6	4	3	6	6	4
Dipeptide transporters[Table-fn tf12-4]						
Total	10	10	11	9	10	10
Increase	1	3	1	1	0	2
Decrease	2	3	5	0	0	5
Unchanged	7	4	5	8	10	3
Iron transporters[Table-fn tf12-5]						
Total	4	5	6	4	4	5
Increase	0	3	1	0	1	1
Decrease	1	1	2	1	0	2
Unchanged	3	1	3	3	3	2

1 Covers FN0023, FN0130, FN0236, FN0258, FN0276, FN0308, FN0309, FN0310, FN0332, FN0341, FN0352, FN0375, FN0376, FN0377, FN0397, FN0398, FN0399, FN0400, FN0450, FN0598, FN0658, FN0660, FN0685, FN0695, FN0793, FN0800, FN0801, FN0827, FN0828, FN1022, FN1080, FN1086, FN1135, FN1136, FN1166, FN1167, FN1187, FN1190, FN1198, FN1256, FN1301, FN1348, FN1349, FN1352, FN1353, FN1354, FN1362, FN1363, FN1398, FN1420, FN1432, FN1480, FN1525, FN1701, FN1734, FN1735, FN1737, FN1738, FN1739, FN1740, FN1741, FN1797, FN1798, FN1801, FN1811, FN1833, FN1834, FN1858, FN1860, FN1898, FN1989, FN2008, FN2009, FN2102, FN2105, FN2106.

2 Covers FN0130, FN0236, FN0450, FN0598, FN0658, FN0660, FN0695, FN0827, FN0828, FN1080, FN1301, FN1348, FN1349, FN1352, FN1353, FN1354, FN1701,FN2102.

3 Covers FN0660, FN0793, FN0800, FN0801, FN1187, FN1398, FN1432, FN1801.

4 Covers FN0192, FN0396, FN0397, FN0399, FN0400, FN0998, FN1111, FN1359, FN1362, FN1363, FN1523, FN1525.

5 Covers FN0308, FN0309, FN0310, FN0375, FN0376, FN0377.

In contrast to FnSg, FnPg and FnPgSg displayed predominantly unchanged and reduced transporter protein levels in all categories when compared to Fn alone. This indicates a general reduction in transport in the mixed communities, except for increased iron transport and a possible shift in dipeptide transport in FnSg.

### Transcriptional regulators

Table S12 shows the transcriptional regulatory proteins that were significantly different in at least one comparison. Most of these proteins only have a general prediction as transcriptional regulators with no indication of their target genes. A number of these are sigma factors (FN1091, FN1317, FN1318) that tend to have very broad effects on transcription and one is an antisigma factor antagonist (FN1914) that is predicted to block the action of an antisigma factor that would in turn block the activity of a sigma factor.

Some of the proteins, however, have specific enough homology to imply a possible function. *Fusobacterium nucleatum* ATCC 25586 encodes for two homologs of the acetoacetate metabolism regulatory protein AtoC (FN1321, FN1831). This protein is part of a two-component regulatory system that controls the genes for short-chain fatty acid catabolism (Matta et al. 2007). However, ATCC 25586 does not possess the target genes or a homolog of the sensor kinase AtoS. AtoC can also function as an antizyme to directly inhibit the activity of ornithine decarboxylase (Lioliou and Kyriakidis 2004). ATCC 25586 does encode for an ornithine decarboxylase protein (FN0501) which showed increased levels in the FnSg community and decreased levels in FnPgSg compared to Fn alone. Interestingly, both FN1321 and FN1831 showed increased levels in the FnSg community and FN1831 showed decreased levels in FnPgSg. It seems likely that the AtoC proteins are present for their antizyme activity, rather than transcriptional regulation.

A homolog of the heat-inducible transcription repressor HrcA (FN0113) showed a significant increase in FnSg and a significant decrease in the FnPgSg community. HrcA controls the expression of chaperones such as GroEL, GroES, and DnaK (FN0675, FN0676, FN0116) in response to temperature (Schulz and Schumann 1996). Of the proteins expected to be subject to HrcA regulation, only GroES showed significant changes being reduced in both FnSg and FnPgSg. The mismatch between putative targets and the regulator could be explained by the signal state of the regulator. Altered levels of the regulatory protein do not necessarily directly result in altered expression, in the absence of the proper regulatory stimulus.

FN0528 has homology to cold-shock proteins which are induced under cold conditions and in turn control the expression of other genes, such as those affecting chromosome structures like gyrase A (Jones and Inouye 2006). In addition to cold, the cold-shock response can be induced by the addition of certain translation inhibitors and the state of the ribosome might act as the physiological sensor for cold-shock induction (Jones and Inouye 2006). The cells should not be seeing any significant temperature differences between the mixed cultures and Fn alone. However, the cold-shock protein showed reduced levels in all of the mixed communities versus Fn. This might be a translational rather than temperature induced change, but the ribosomal protein levels are reduced in FnPg and FnSg and possibly in FnPgSg. This would imply the opposite change than that seen in the cold-shock protein.

### pH response

In our study of *S. gordonii*, we found a number of responses consistent with a low pH environment (Hendrickson et al. 2012). Chew et al. (2012) have examined the response of Fn ATCC 10953 biofilms to elevated pH. They found a number of proteins were changed between biofilms at pH 8.2 compared to those at pH 7.4. These differences included increased levels of the metabolic proteins gluaconyl-CoA decarboxylase A subunit (FN0204 in ATCC 25586) and phosphate acetyltransferase (FN1172), the important adhesion FomA (FN1859), RecA (FN0547), and the stress protein GroEL (FN0675). While we saw no changes in GroEL levels we saw reductions in one or more of the pellets for the others (Table S1). They also found several proteins with decreased levels including the metabolic proteins glutamate formiminotransferase (FN0741), butanoate acetoacetate CoA transferase alpha subunit (FN1857), and butyryl-CoA dehydrogenase (three homologs FN00783, FN 1424, FN1535), the stress protein DnaK (FN0116), and several proteins involved in translation. Again we saw no change in the stress protein, DnaK, but the metabolic proteins showed increased levels in one or more of our communities compared to Fn alone. However, we did see a general reduction in translational machinery (see the section Translation, ribosomal proteins, and tRNA synthetases above and Table2) though translation would be expected to respond to many factors beyond pH. Overall, these results were consistent with the idea that our mixed-species samples were at a lower 18 h pH than Fn alone, similar to our findings with *S. gordonii* (Hendrickson et al. 2012). As expected, bulk scale measurements of pH at *t* = 0 and *t* = 18 h for all pellets used in the study were inconclusive (data not shown), yielding both a starting and ending pH of 7.2 in each pellet. Any excess acid production would have to be of sufficient magnitude to overwhelm the buffering capacity of the residual PBS used to suspend the cells (see Materials and methods), an unlikely occurrence under these experimental conditions.

## Supporting Information

**Data S1.** Diagrams of the purine and pyrimidine pathways for each comparison. Labeling conventions are the same as those used in the main text pathway figures. This material is not discussed explicitly in the text, but we include it here for the sake of completeness.

**Figure S1.** FnPg versus Fn amino acid fermentation. The diagram shows a schematic of l-histidine, l-glutamine, l-glutamate, l-asparagines, l-aspartate, l-serine, and l-lysine fermentation for the *Fusobacterium nucleatum* with *Porphyromonas gingivalis* sample compared to *F. nucleatum*. Proteins catalyzing each step are shown by their *F. nucleatum* FN number. Red numbers indicate increased levels in the first condition compared to the second condition, green decreased levels, yellow no statistical change, black undetected in at least one of the conditions, and purple for Acetyl-CoA which appears as a substrate or product in many reactions. **Figure S2.** FnSg versus Fn amino acid fermentation. Labels and color coding as described for Figure S1, for the *Fusobacterium nucleatum* with *Streptococcus gordonii* comparison to *F. nucleatum*. **Figure S3.** FnPgSg versus Fn amino acid fermentation. Labels and color coding as described for Figure S1, for the *Fusobacterium nucleatum* with *Porphyromonas gingivalis* and *Streptococcus gordonii* comparison to *F. nucleatum*. **Figure S4**. FnPgSg versus FnPg amino acid fermentation. Labels and color coding as described for Figure S1, for the *Fusobacterium nucleatum* with *Porphyromonas gingivalis* and *Streptococcus gordonii* comparison to *F. nucleatum* with *P. gingivalis*. **Figure S5**. FnSg versus FnPg amino acid fermentation. Labels and color coding as described for Figure S1, for the *Fusobacterium nucleatum* with *Streptococcus gordonii* comparison to *F. nucleatum* with *Porphyromonas gingivalis*. **Figure S6.** FnPgSg versus FnSg amino acid fermentation. Labels and color coding as described for Figure S1, for the *Fusobacterium nucleatum* with *Porphyromonas gingivalis* and *Streptococcus gordonii* comparison to *F. nucleatum* with *S. gordonii*. **Figure S7**. FnPg versus Fn other amino acid fermentation. The diagram shows a schematic of l-alanine, l-cysteine, glycine, l-methionine, and l-threonine fermentation for the *Fusobacterium nucleatum* with *Porphyromonas gingivalis* sample compared to *F. nucleatum*. Proteins catalyzing each step are shown by their *F. nucleatum* FN number. Red numbers indicate increased levels in the first condition compared to the second condition, green decreased levels, yellow no statistical change, and black undetected in at least one of the conditions. **Figure S8.** FnSg versus Fn other amino acid fermentation. Labels and color coding as described for Figure S7, for the *Fusobacterium nucleatum* with *S. gordonii* comparison to *F. nucleatum*. **Figure S9.** FnPgSg versus Fn other amino acid fermentation. Labels and color coding as described for Figure S7, for the *Fusobacterium nucleatum* with *Porphyromonas gingivalis* and *Streptococcus gordonii* comparison to *F. nucleatum*. **Figure S10.** FnPgSg versus FnPg other amino acid fermentation. Labels and color coding as described for Figure S7, for the *Fusobacterium nucleatum* with *Porphyromonas gingivalis* and *Streptococcus gordonii* comparison to *F. nucleatum* with *P. gingivalis*. **Figure S11.** FnSg versus FnPg other amino acid fermentation. Labels and color coding as described for Figure S7, for the *Fusobacterium nucleatum* with *Streptococcus gordonii* comparison to *F. nucleatum* with *Porphyromonas gingivalis*. **Figure S12.** FnPgSg versus FnSg other amino acid fermentation. Labels and color coding as described for Figure S7, for the *Fusobacterium nucleatum* with *Porphyromonas gingivalis* and *Streptococcus gordonii* comparison to *F. nucleatum* with *S. gordonii*.

Table S1 The overall results for the entire study, relative abundance ratios presented with a minimum of analytical detail.

Table S2 Details for the qualitative coverage observed for each protein identified in this study.

Table S3 A more detailed presentation of the quantitative data for this comparison, including *P*-values, *q*-values, raw and normalized spectral counts, abundance ratios, and protein descriptors.

Table S4 See description for Table S3.

Table S5 See description for Table S3.

Table S6 See description for Table S3.

Table S7 See description for Table S3.

Table S8 See description for Table S3.

Table S9 FileMaker results for ethanolamine metabolism proteins. Proteins are listed by FN number. For each of the six comparisons, the log_2_ ratio of the normalized spectral counts, log_2_ sum of normalized spectral counts, *P*-value, and *q*-value are shown. Statistically significant increases are shown in red with the corresponding *q*-value in light red while decreases are shown in green with the corresponding *q*-value in light green. Color-coded bar graphs are presented for all six ratio comparisons.

Table S10 FileMaker results for adhesion and outer membrane proteins. Results and color coding as listed in Table S9 above.

Table S11 FileMaker results for protein secretion proteins. Results and color coding as listed in Table S9 above.

Table S12 FileMaker results for significantly altered transcriptional regulators. Results and color coding as listed in Table S9 above.
