# Supplementary figures and images for: Proteomics of Fusobacterium nucleatum within a model developing oral microbial community
Source: Microbiologyopen. 2014 Aug 25;3(5):729–51. doi: 10.1002/mbo3.204 (PMC4234264; doi:10.1002/mbo3.204)

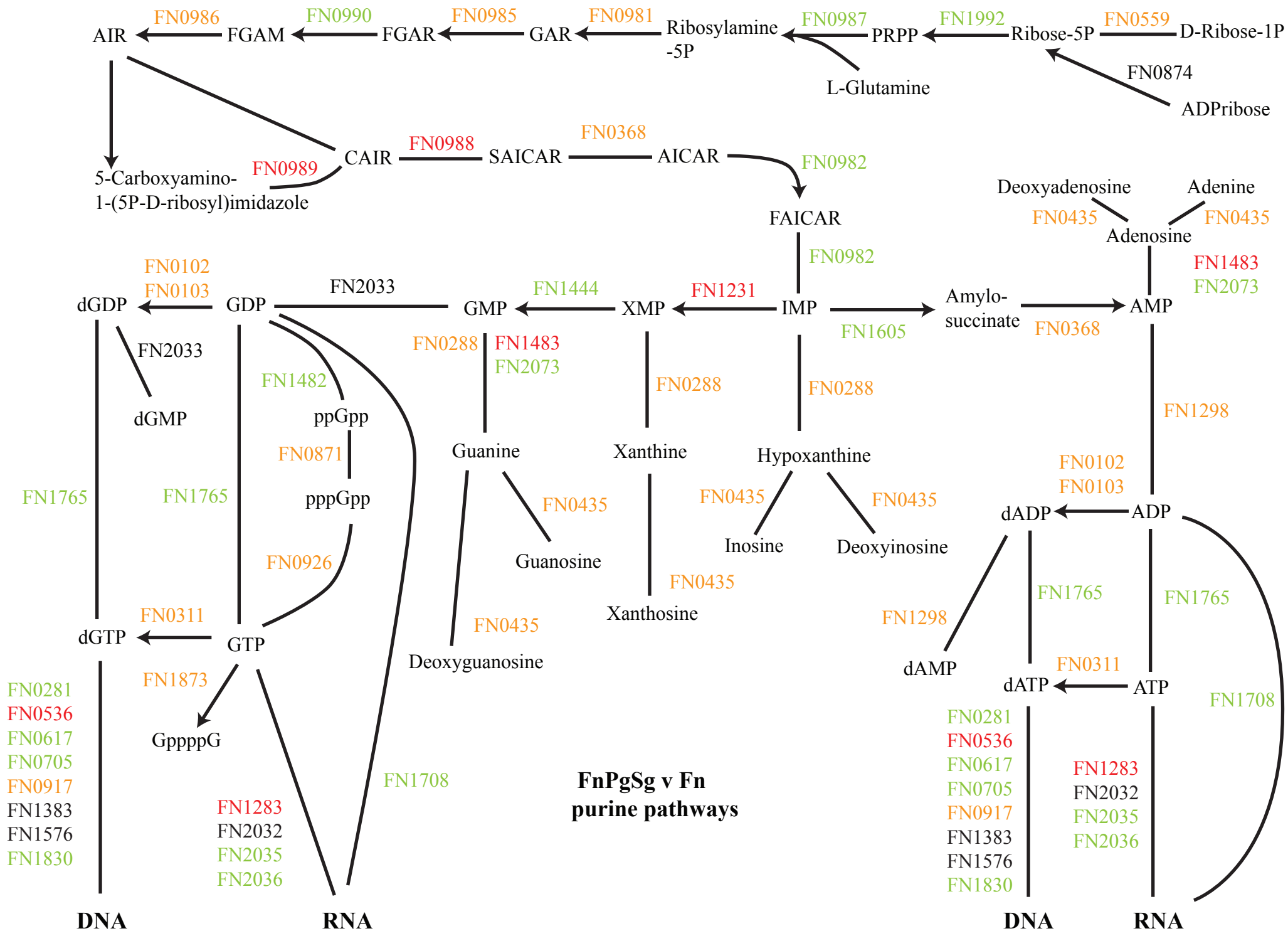

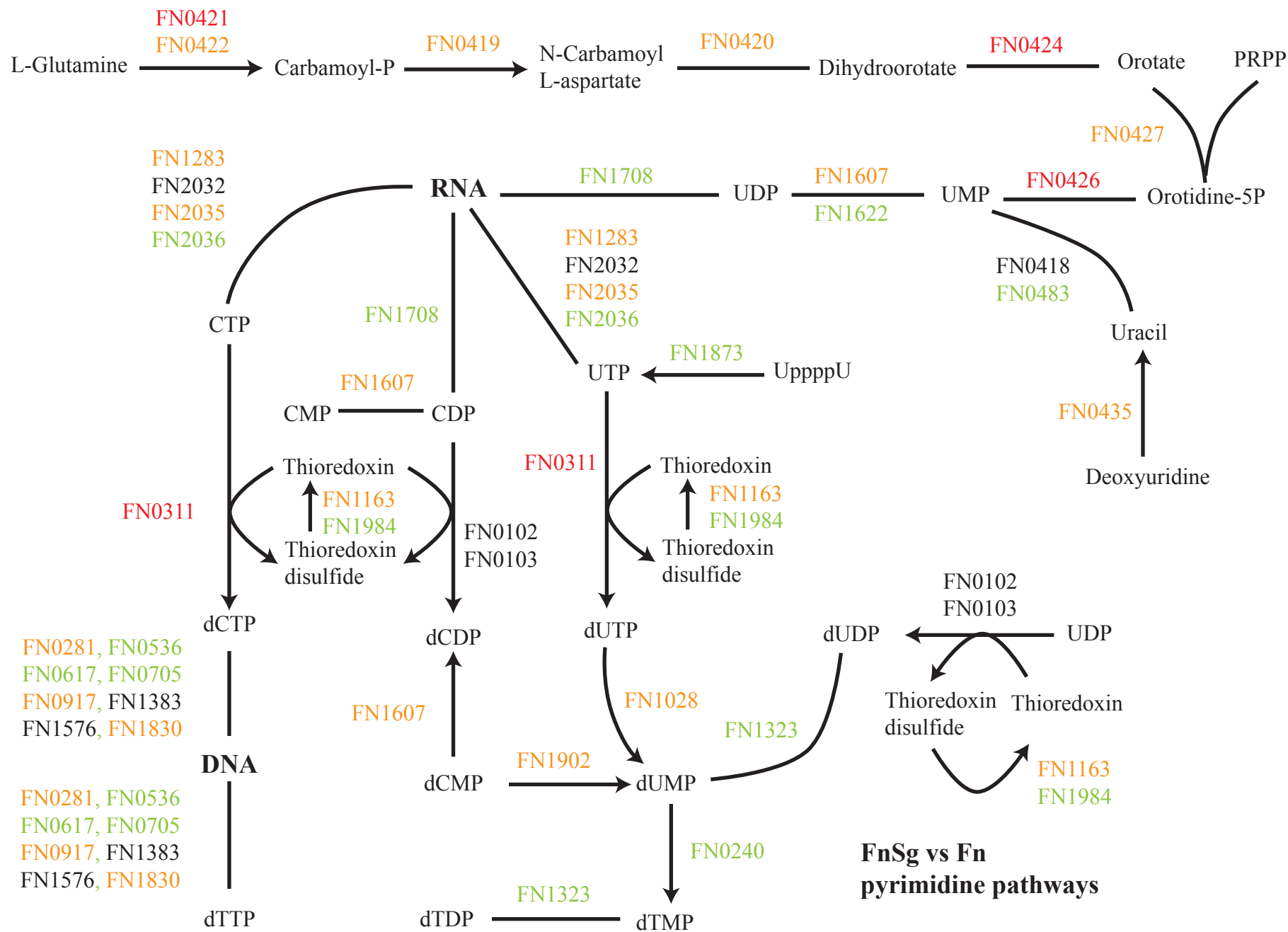

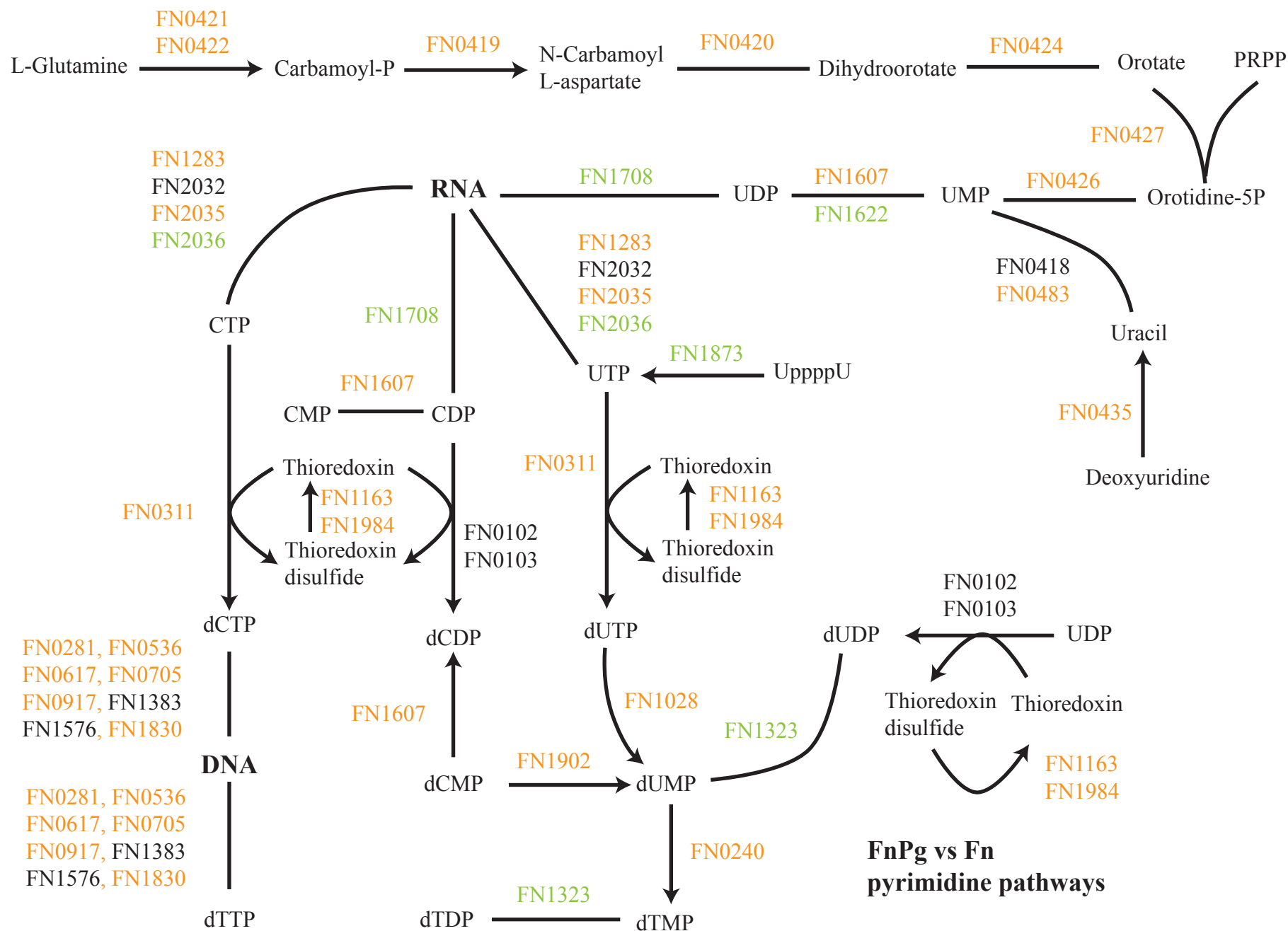

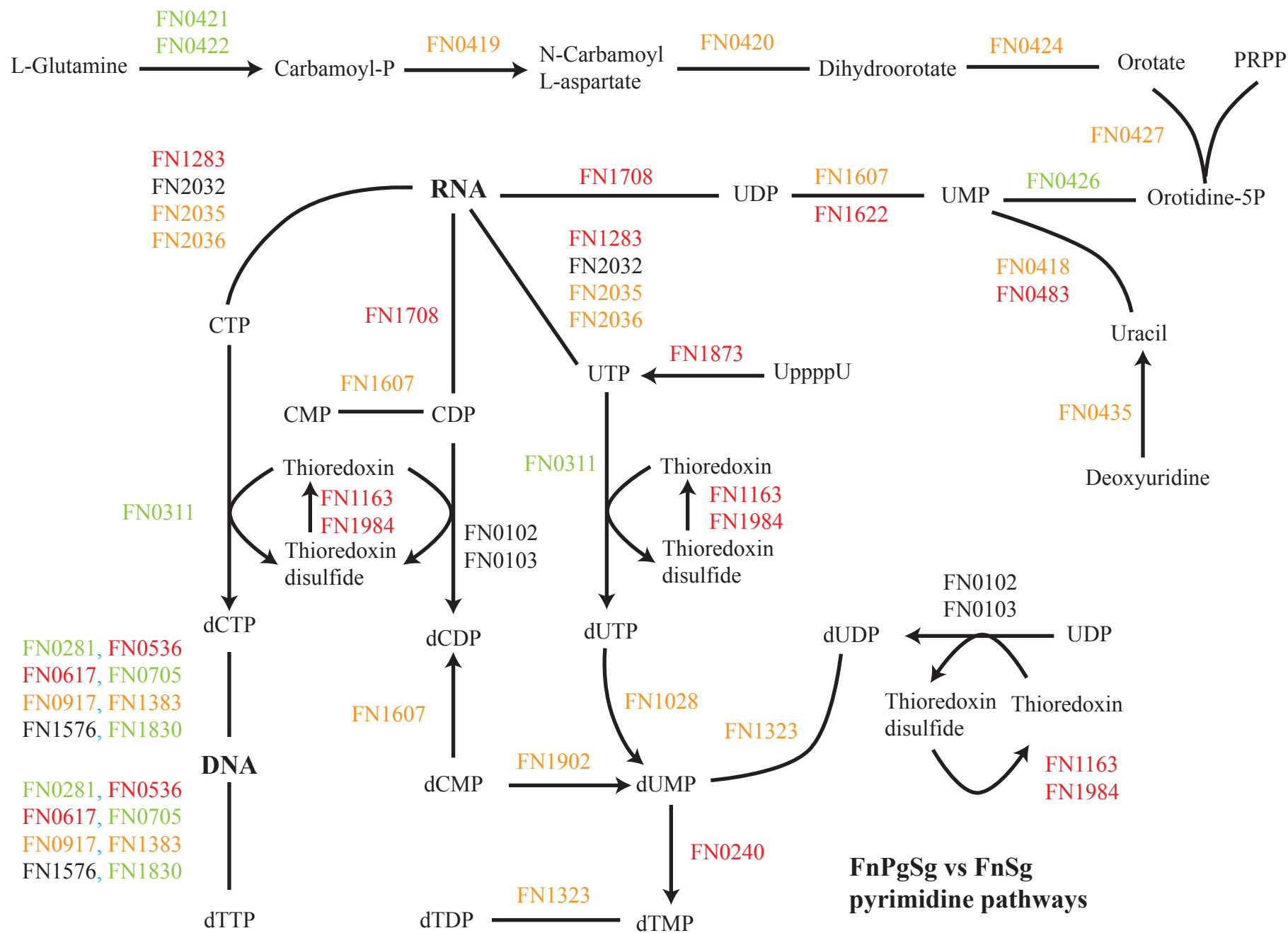

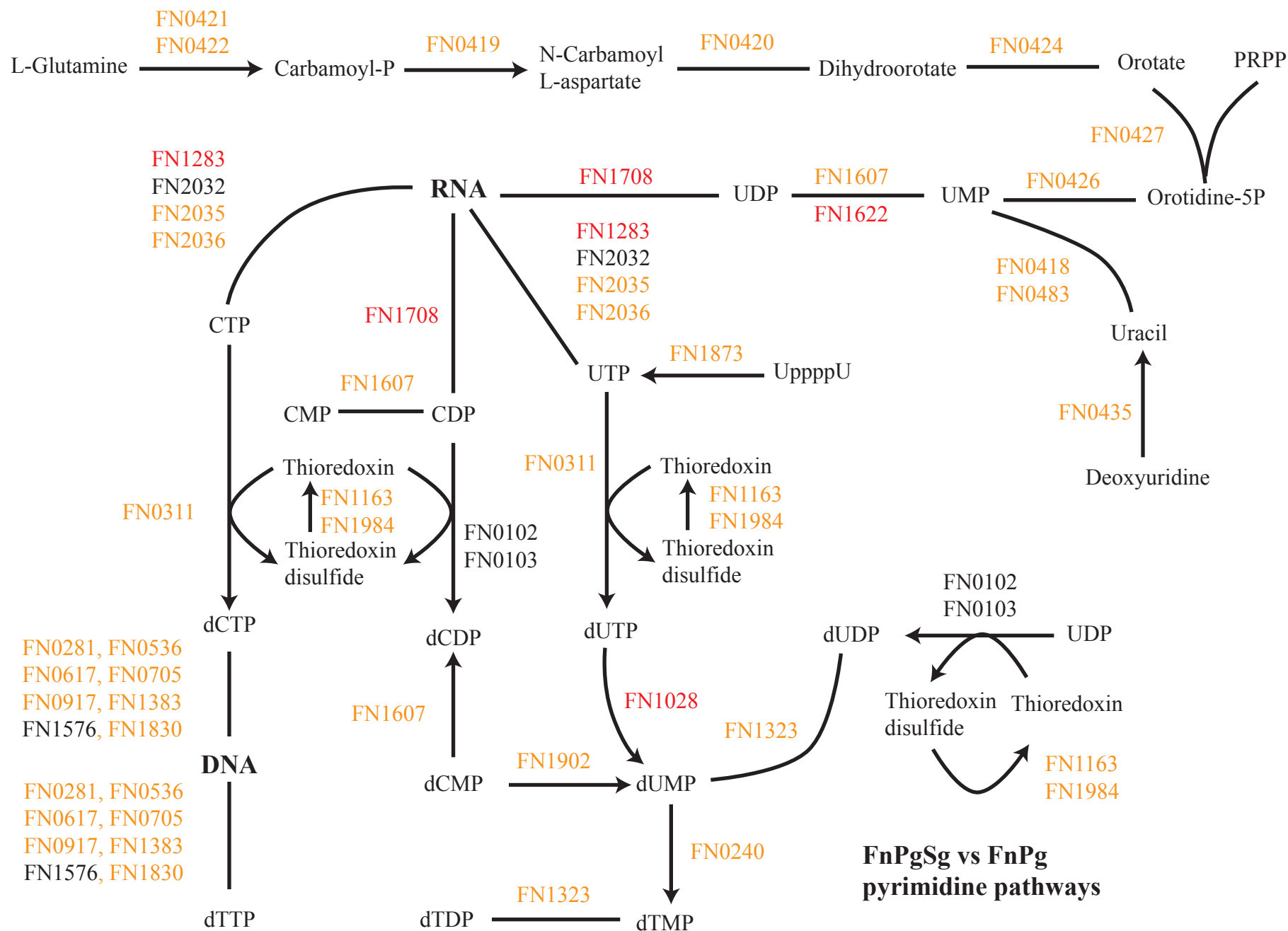

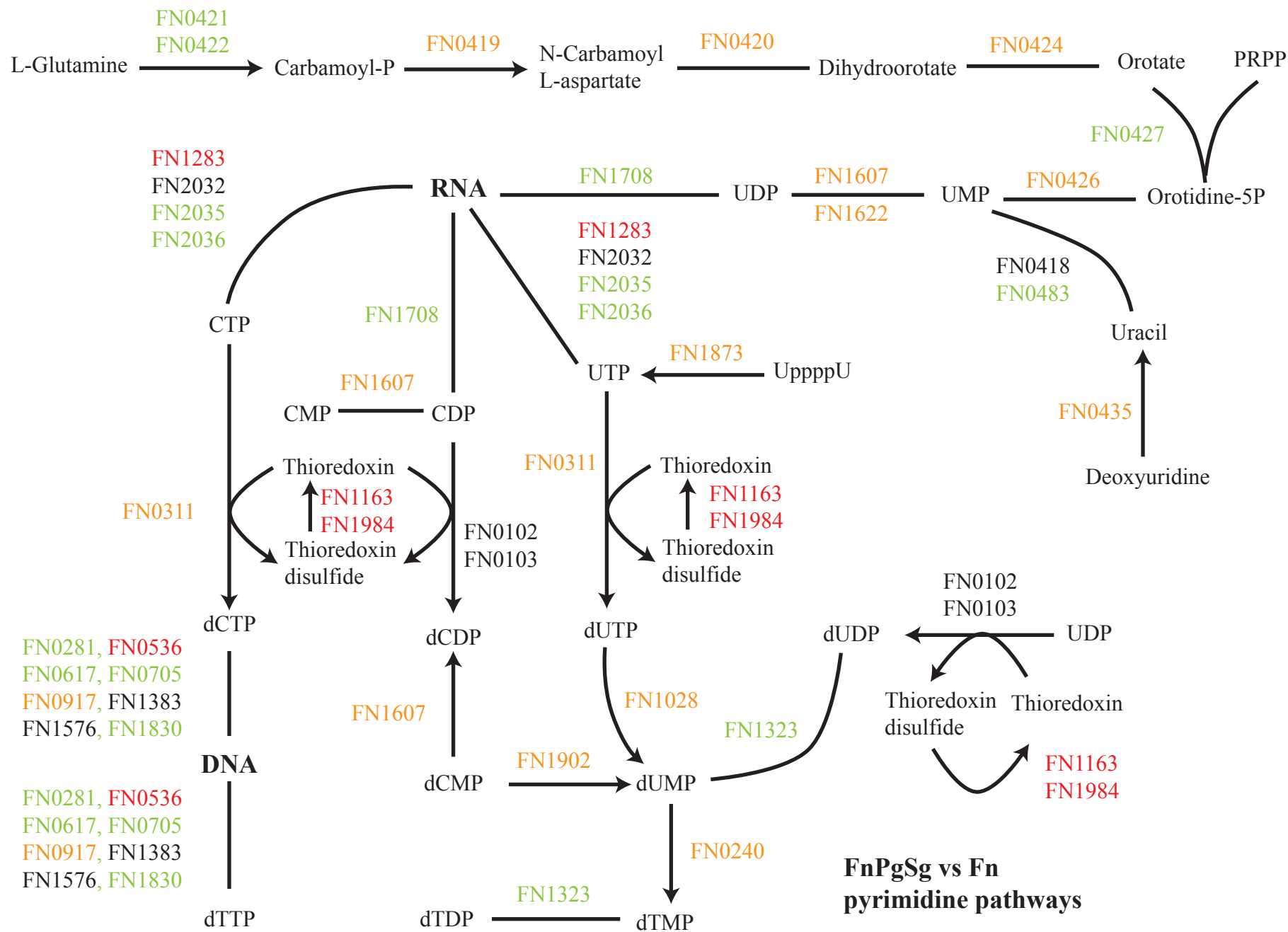

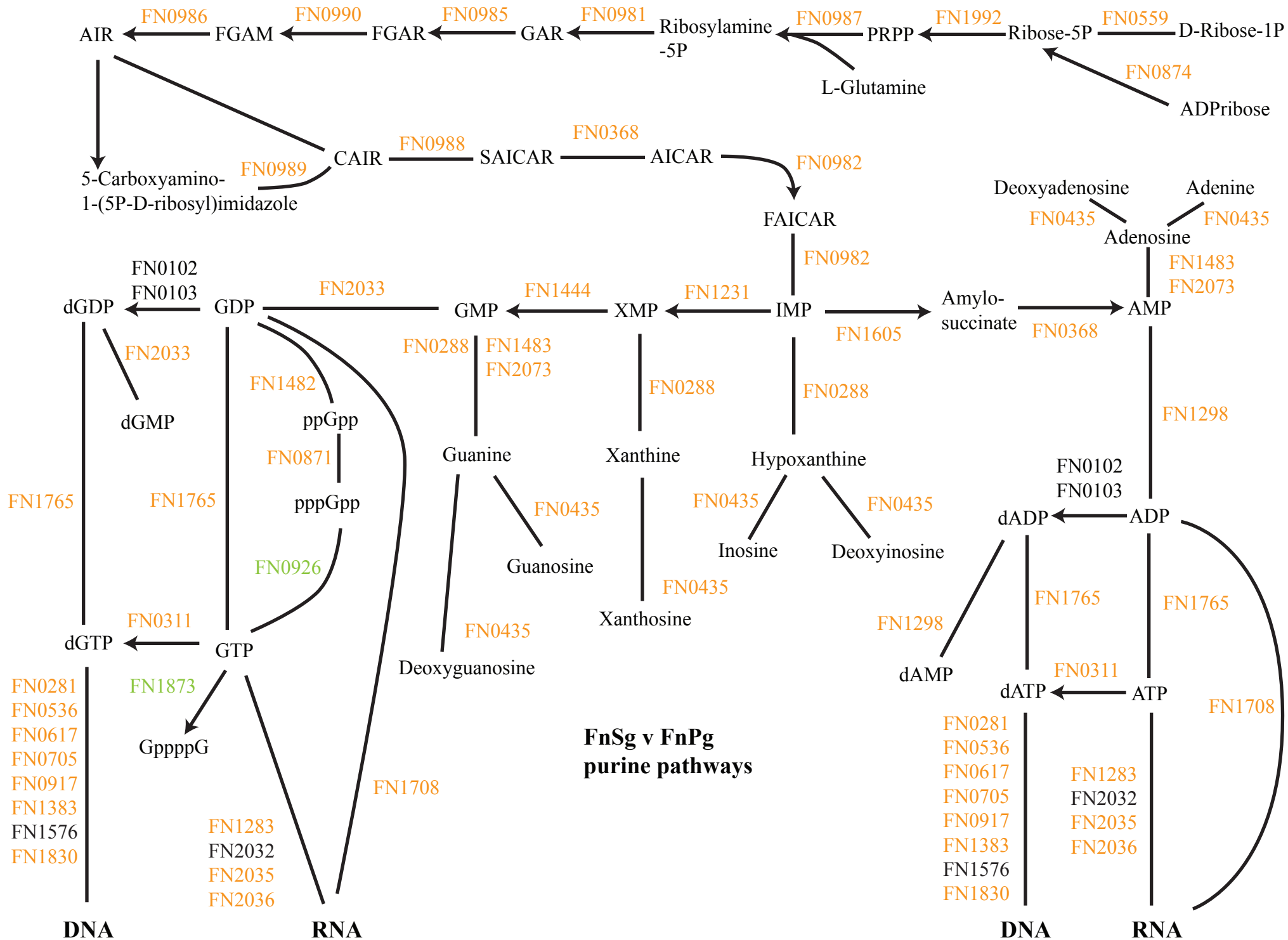

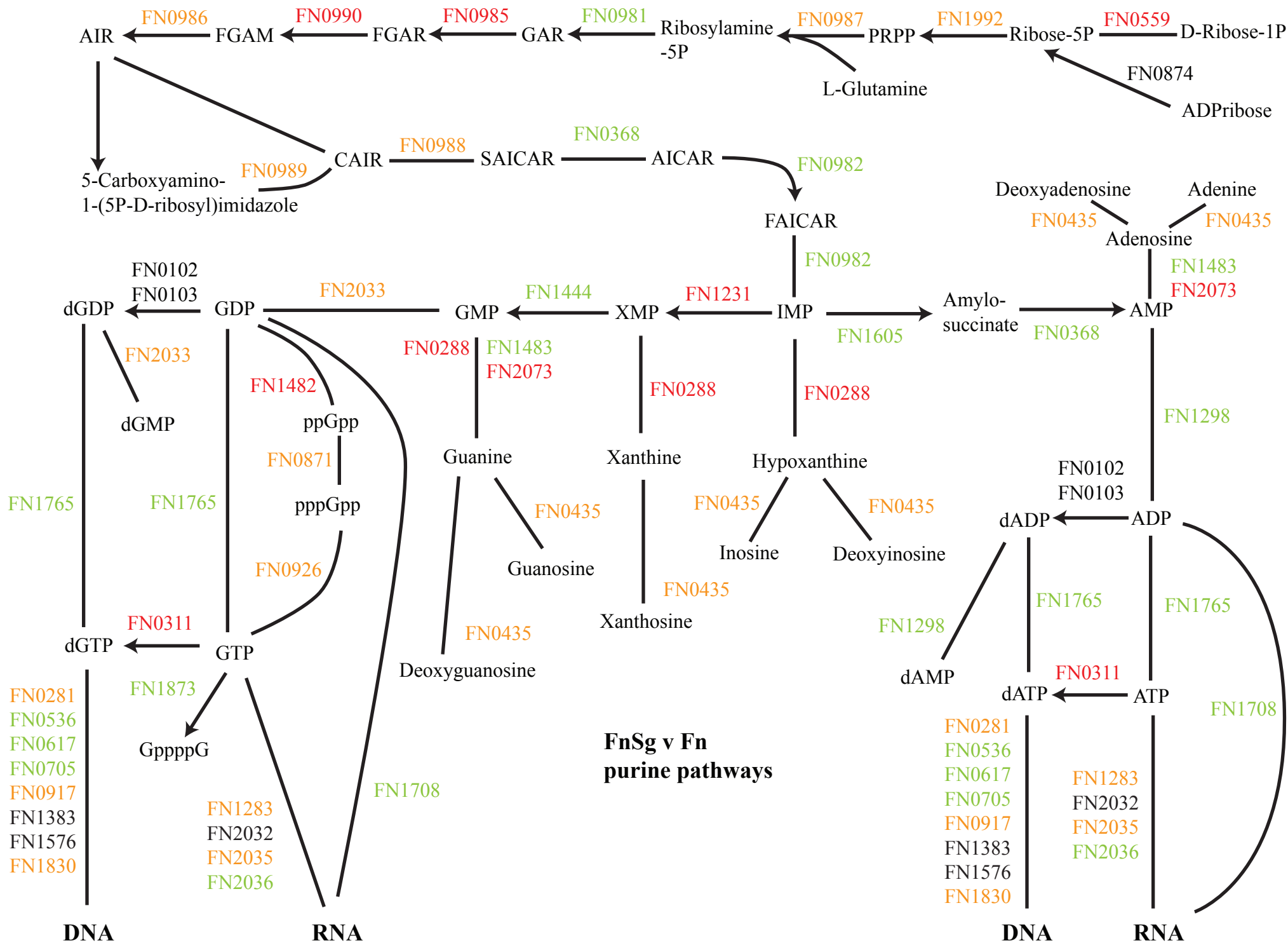

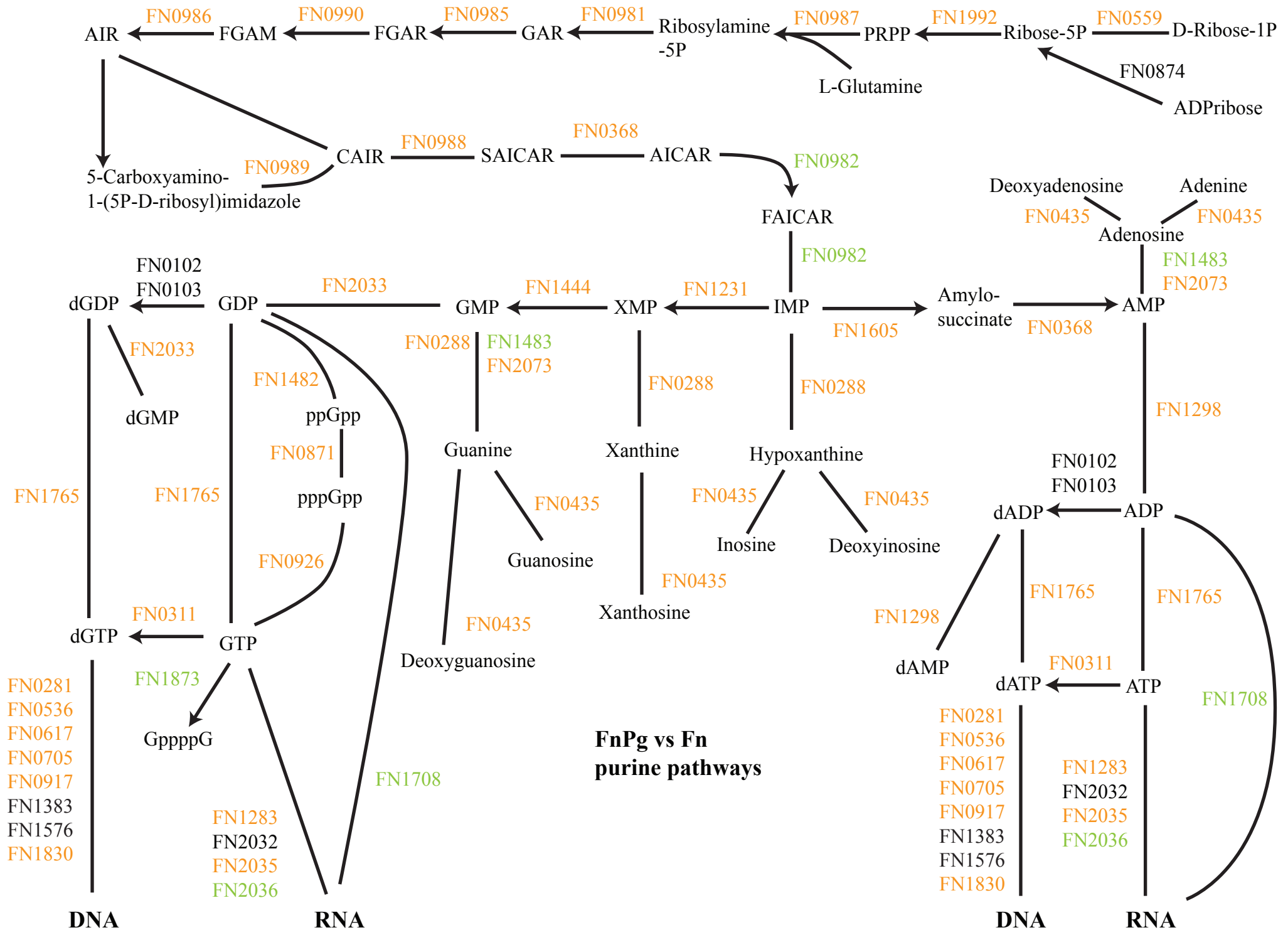

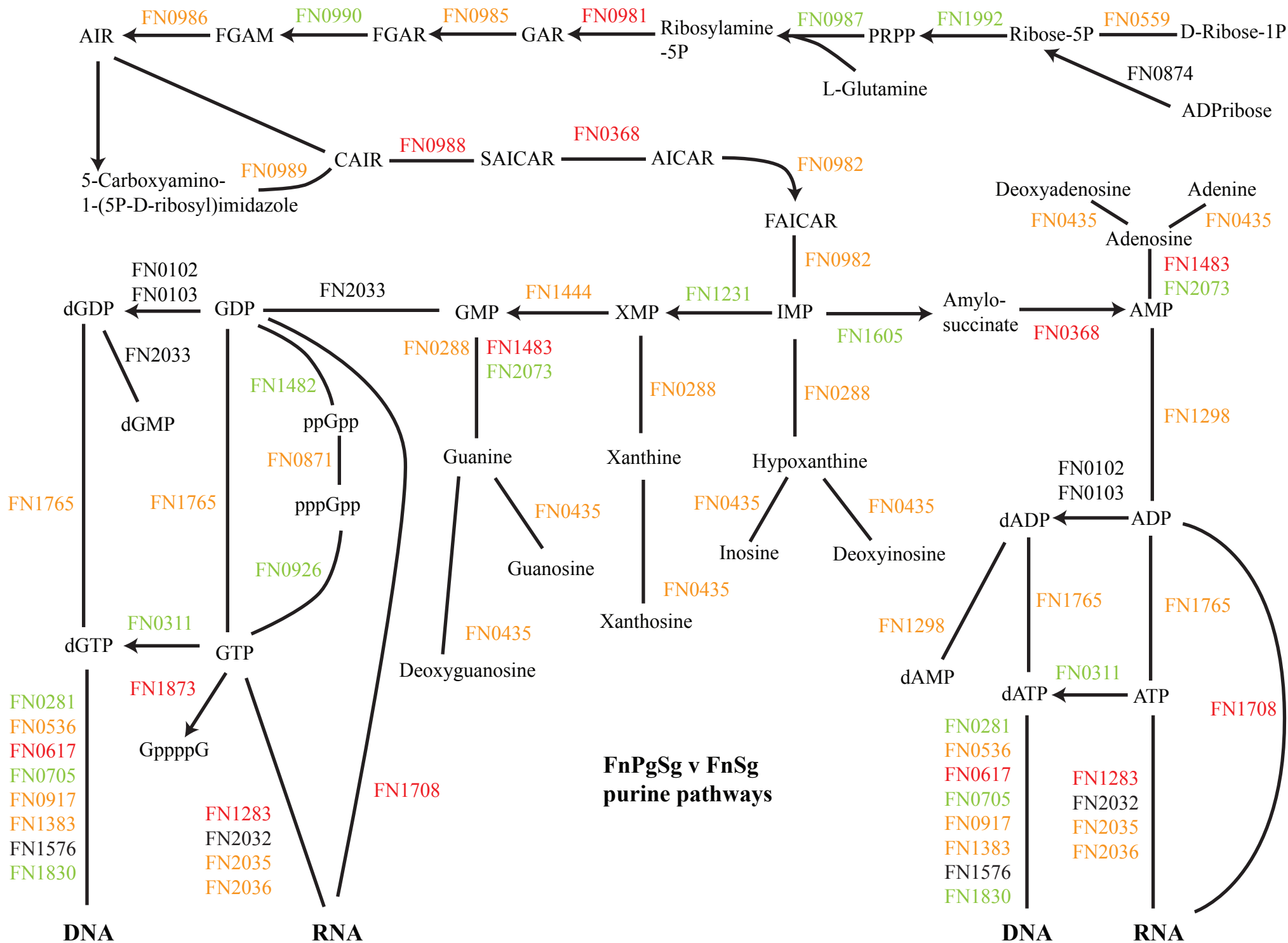

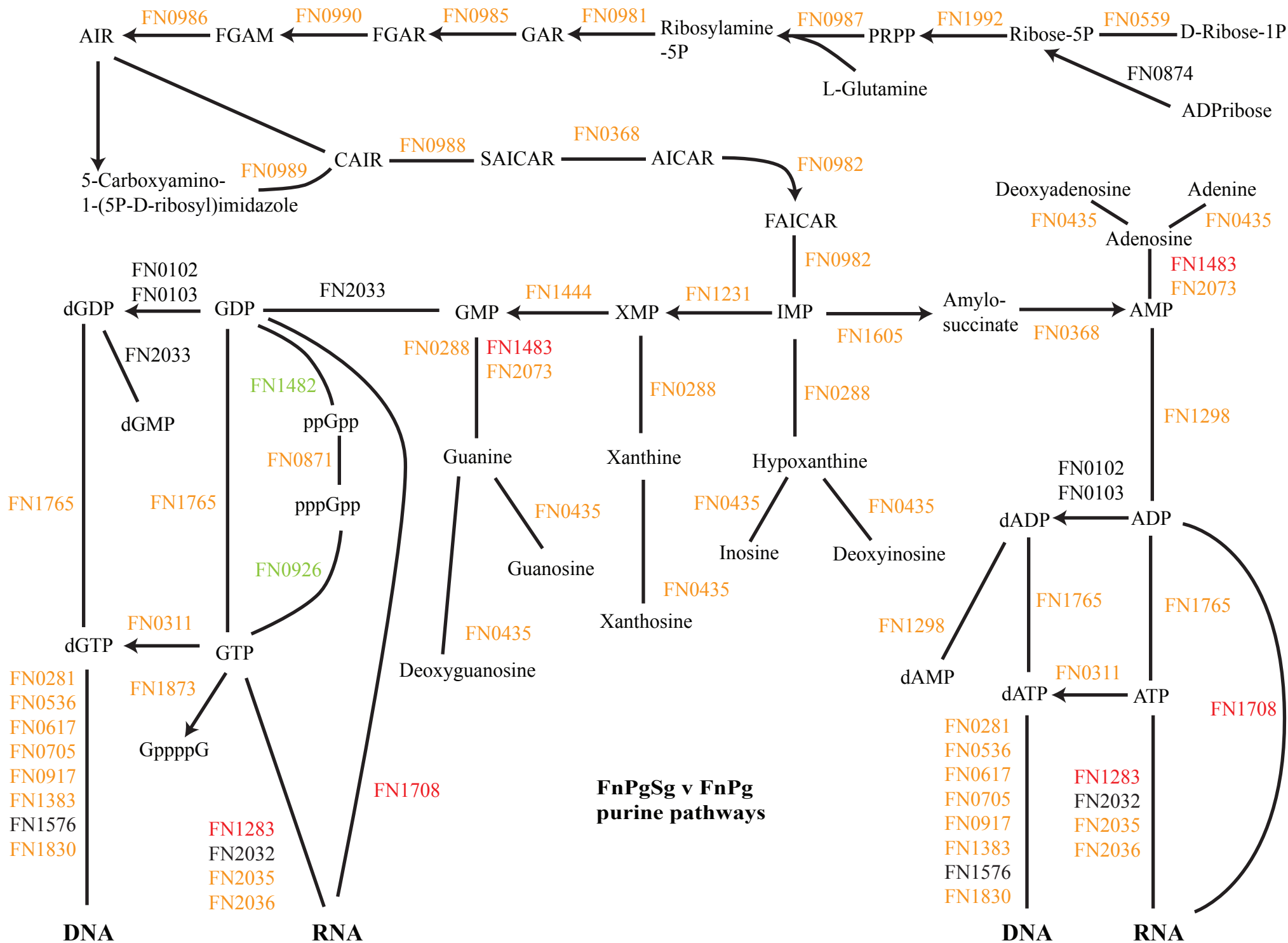

Supplement: Supplementary file 1 — Data S1. Diagrams of the purine and pyrimidine pathways for each comparison. Labeling conventions are the same as those used in the main text pathway figures. This material is not discussed explicitly in the text, but we include it here for the sake of completeness. [file mbo30003-0729-sd1.pdf]
