# Supplementary material for: Proteomics of Fusobacterium nucleatum within a model developing oral microbial community
Source: Microbiologyopen. 2014 Aug 25;3(5):729–51. doi: 10.1002/mbo3.204 (PMC4234264; doi:10.1002/mbo3.204)
Supplement: Supplementary file 2 — Figure S1. FnPg versus Fn amino acid fermentation. The diagram shows a schematic of l-histidine, l-glutamine, l-glutamate, l-asparagines, l-aspartate, l-serine, and l-lysine fermentation for the Fusobacterium nucleatum with Porphyromonas gingivalis sample compared to F. nucleatum. Proteins catalyzing each step are shown by their F. nucleatum FN number. Red numbers indicate increased levels in the first condition compared to the second condition, green decreased levels, yellow no statistical change, black undetected in at least one of the conditions, and purple for Acetyl-CoA which appears as a substrate or product in many reactions. Figure S2. FnSg versus Fn amino acid fermentation. Labels and color coding as described for Figure S1, for the Fusobacterium nucleatum with Streptococcus gordonii comparison to F. nucleatum. Figure S3. FnPgSg versus Fn amino acid fermentation. Labels and color coding as described for Figure S1, for the Fusobacterium nucleatum with Porphyromonas gingivalis and Streptococcus gordonii comparison to F. nucleatum. Figure S4. FnPgSg versus FnPg amino acid fermentation. Labels and color coding as described for Figure S1, for the Fusobacterium nucleatum with Porphyromonas gingivalis and Streptococcus gordonii comparison to F. nucleatum with P. gingivalis. Figure S5. FnSg versus FnPg amino acid fermentation. Labels and color coding as described for Figure S1, for the Fusobacterium nucleatum with Streptococcus gordonii comparison to F. nucleatum with Porphyromonas gingivalis. Figure S6. FnPgSg versus FnSg amino acid fermentation. Labels and color coding as described for Figure S1, for the Fusobacterium nucleatum with Porphyromonas gingivalis and Streptococcus gordonii comparison to F. nucleatum with S. gordonii. Figure S7. FnPg versus Fn other amino acid fermentation. The diagram shows a schematic of l-alanine, l-cysteine, glycine, l-methionine, and l-threonine fermentation for the Fusobacterium nucleatum with Porphyromonas gingivalis sample compa [file mbo30003-0729-sd2.pdf]

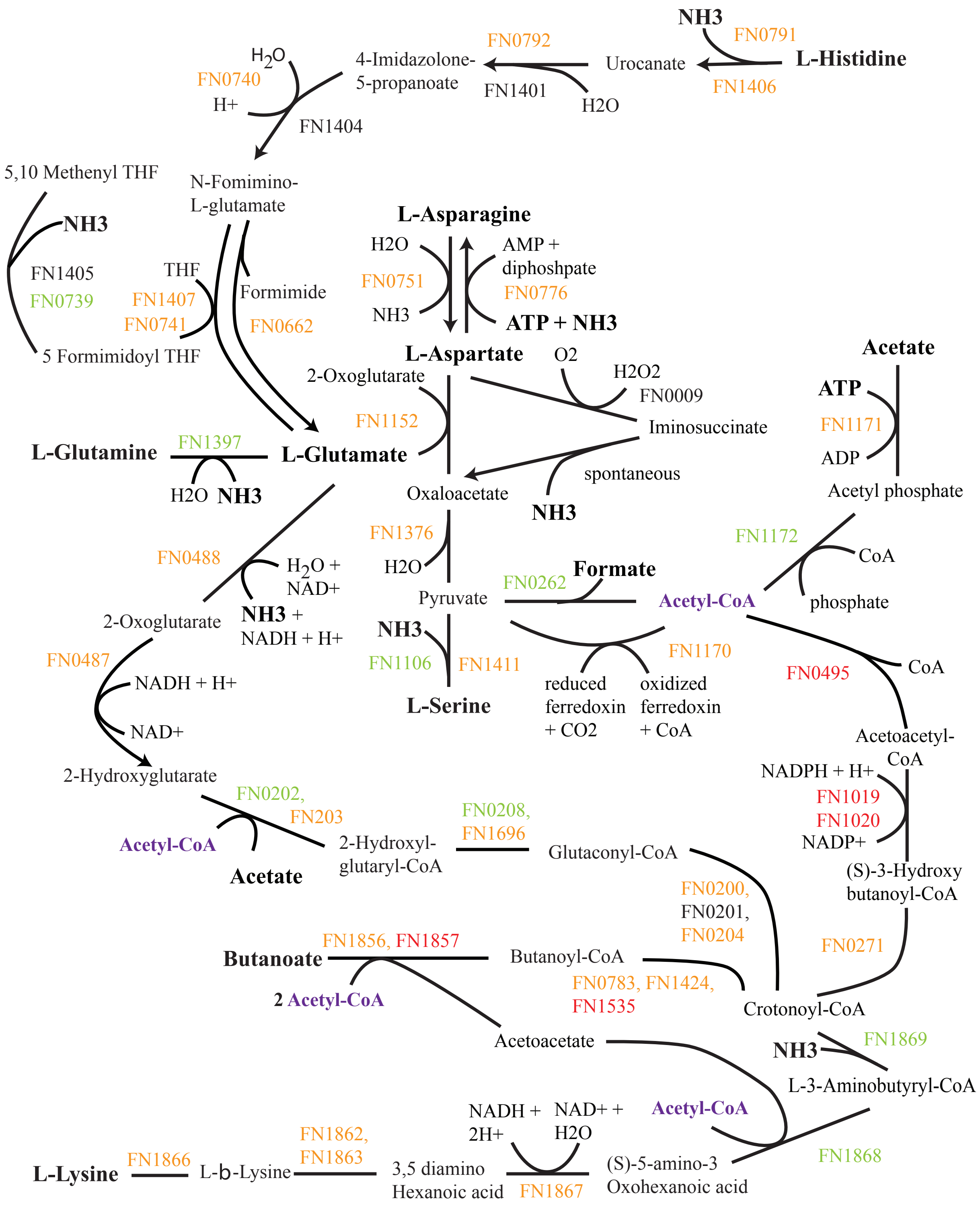

Figure S1. FnPg vs Fn Amino Acid Fermentation. The diagram shows a schematic of L-histidine, L-glutamine, L-glutamate, L-asparagines, L-aspartate, L-serine, and L-lysine

fermentation for the *F. nucleatum* with *P. gingivalis* sample compared to *F. nucleatum*. Proteins catalyzing each step are shown by their *F. nucleatum* FN number. Red numbers indicate increased levels in the first condition compared to the second condition, green decreased levels, yellow no statistical change, black undetected in at least one of the conditions, and purple for Acetyl-CoA which appears as a substrate or product in many reactions.

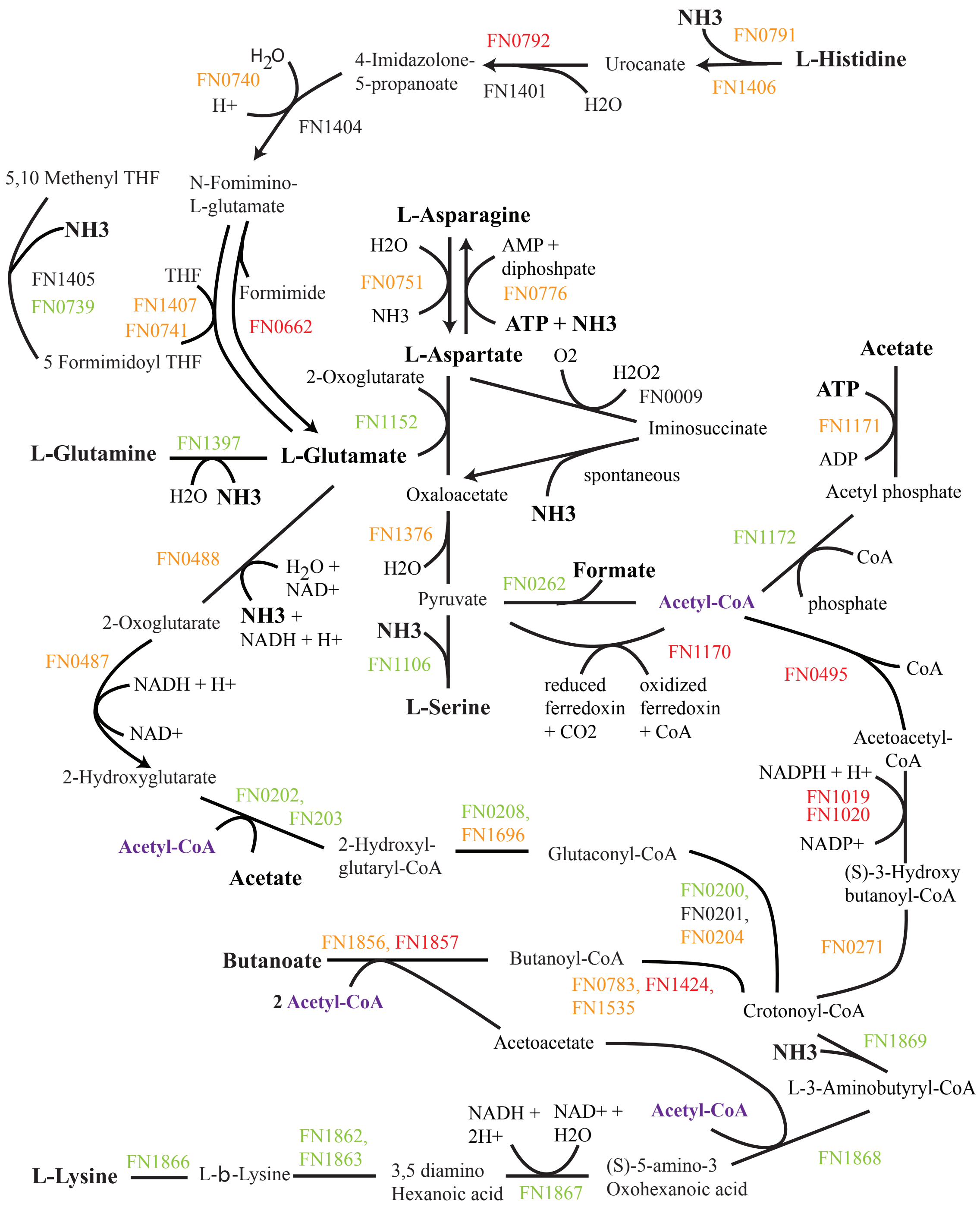

Figure S2. FnSg vs Fn Amino Acid Fermentation. Labels and color coding as described for Figure S1, for the *F. nucleatum* with *S. gordonii* comparison to *F. nucleatum*.

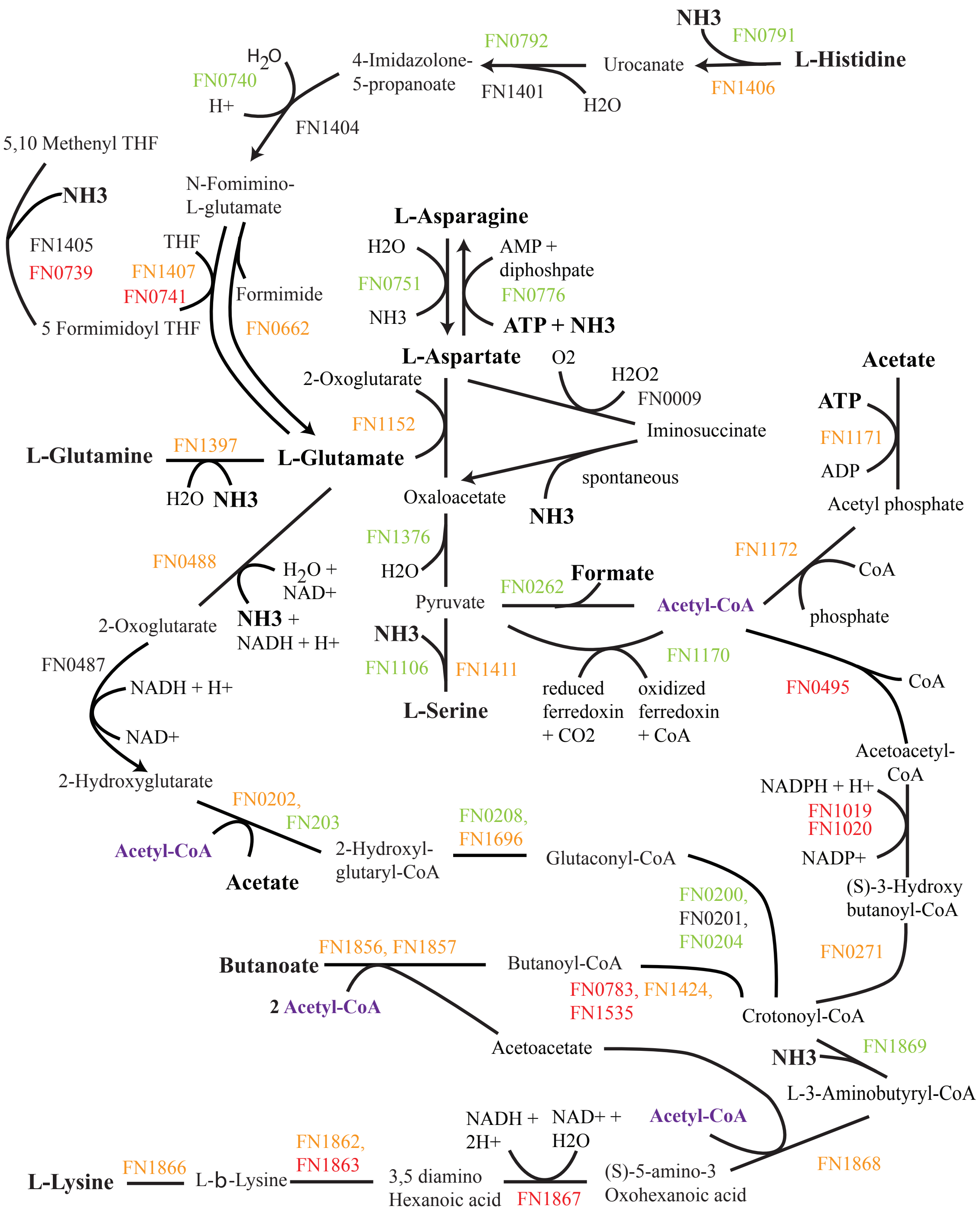

Figure S3. FnPgSg vs Fn Amino Acid Fermentation. Labels and color coding as described for Figure S1, for the *F. nucleatum* with *P. gingivalis* and *S. gordonii* comparison to *F. nucleatum*.

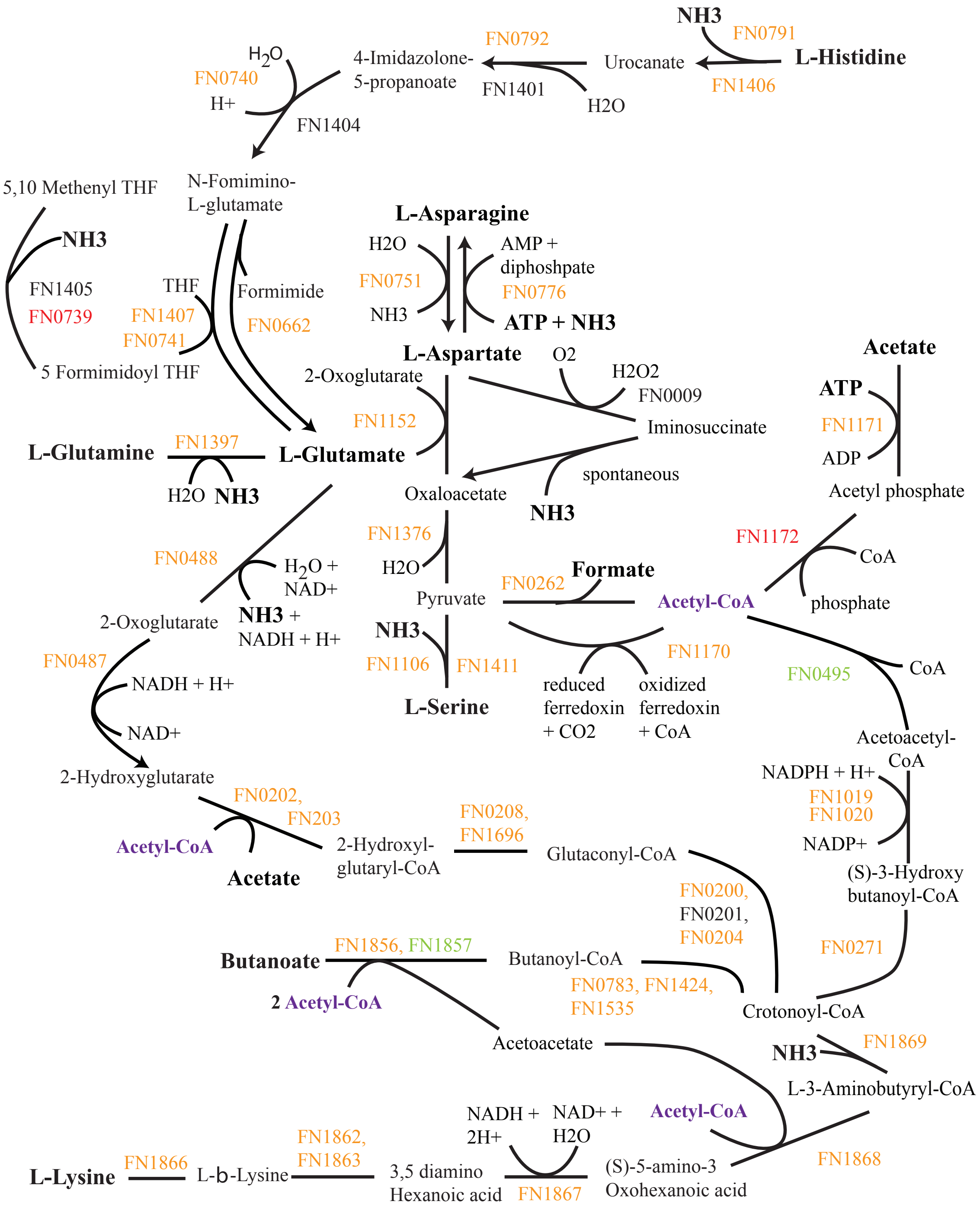

Figure S4. **FnPgSg vs FnPg Amino Acid Fermentation.** Labels and color coding as described for Figure S1, for the *F. nucleatum* with *P. gingivalis* and *S. gordonii* comparison to *F. nucleatum* with *P. gingivalis*.

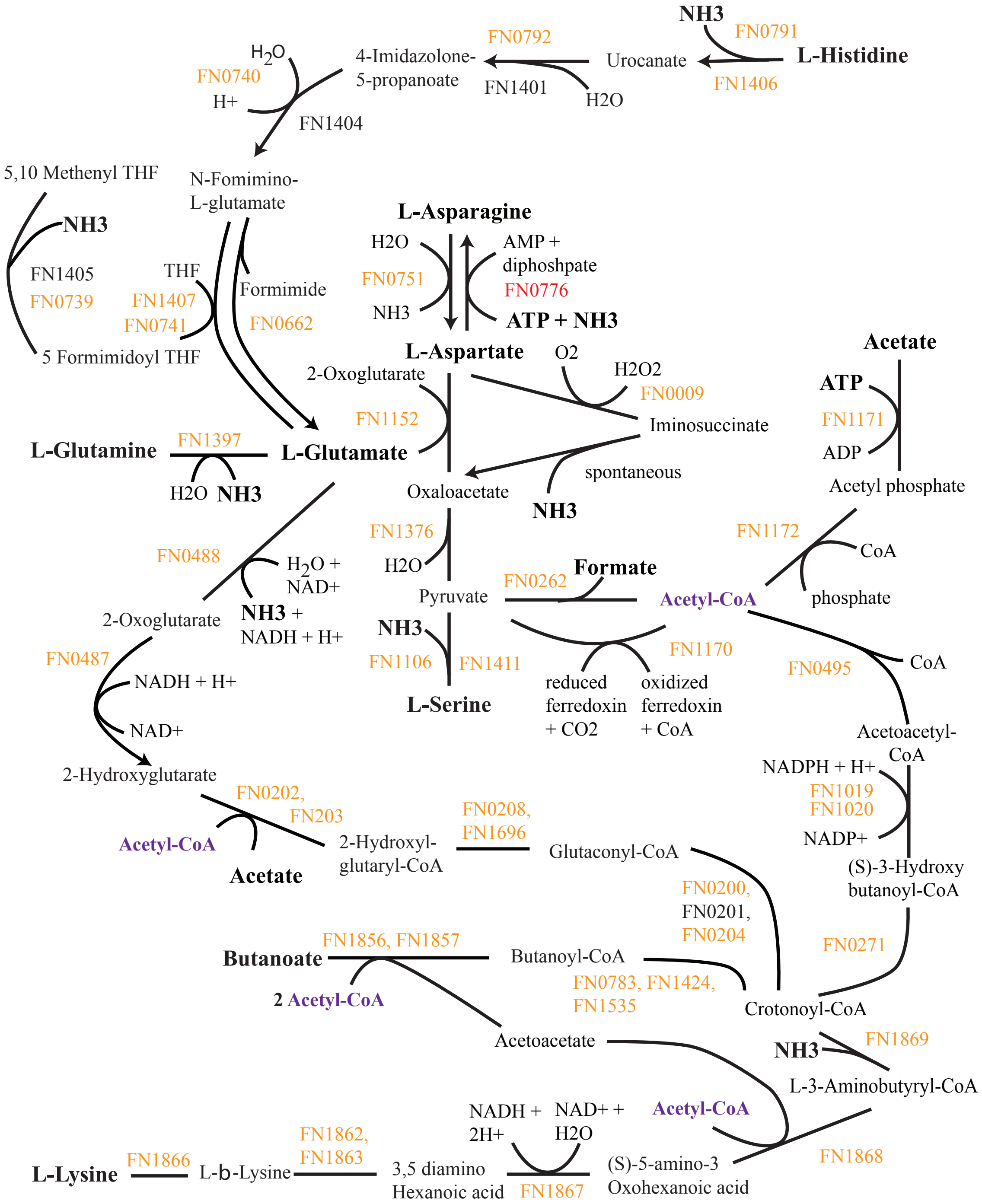

Figure S5. **FnSg vs FnPg Amino Acid Fermentation.** Labels and color coding as described for Figure S1, for the *F. nucleatum* with *S. gordonii* comparison to *F. nucleatum* with *P. gingivalis*.

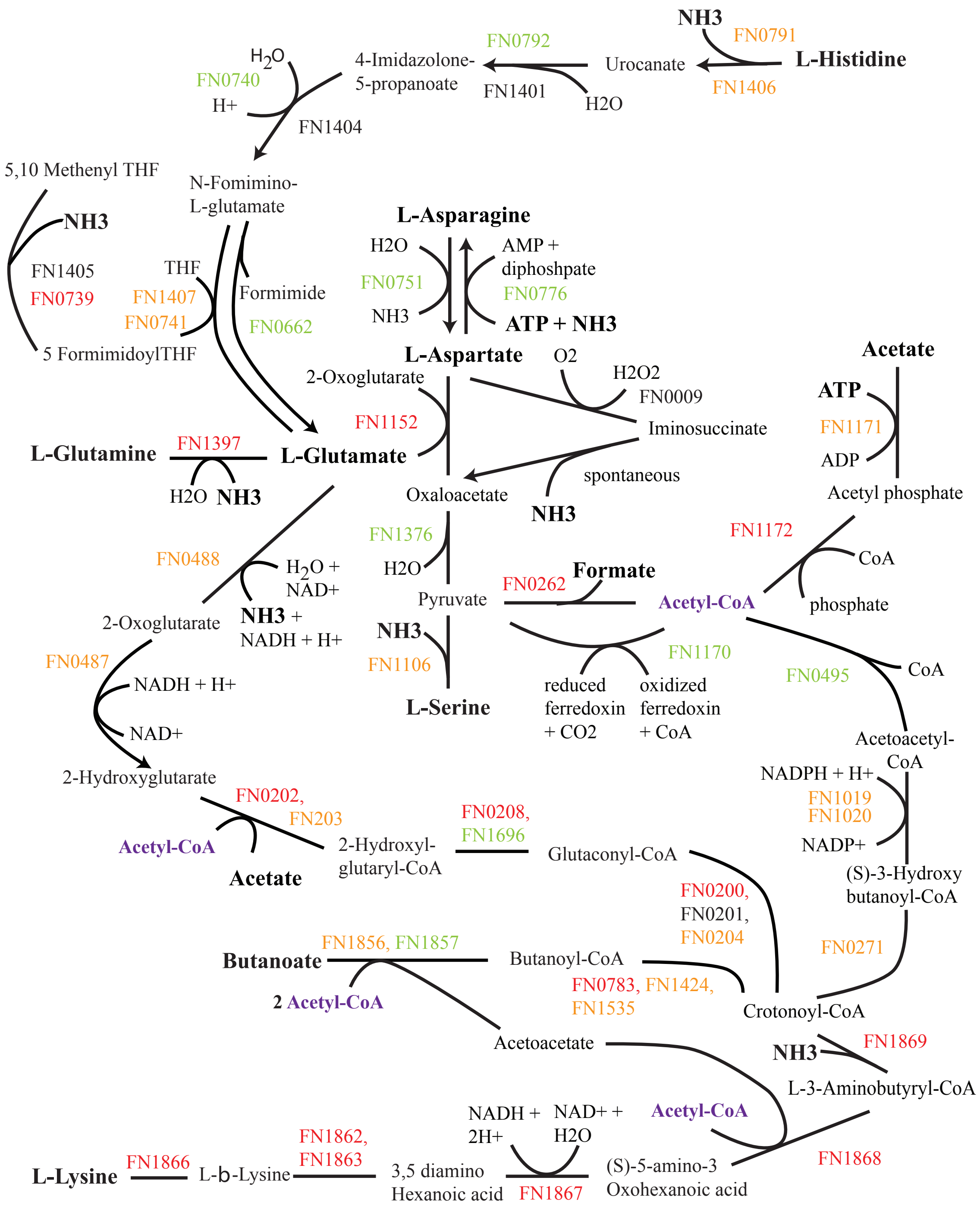

Figure S6. FnPgSg vs FnSg Amino Acid Fermentation. Labels and color coding as described for Figure S1, for the *F. nucleatum* with *P. gingivalis* and *S. gordonii* comparison to *F. nucleatum* with *S. gordonii*.

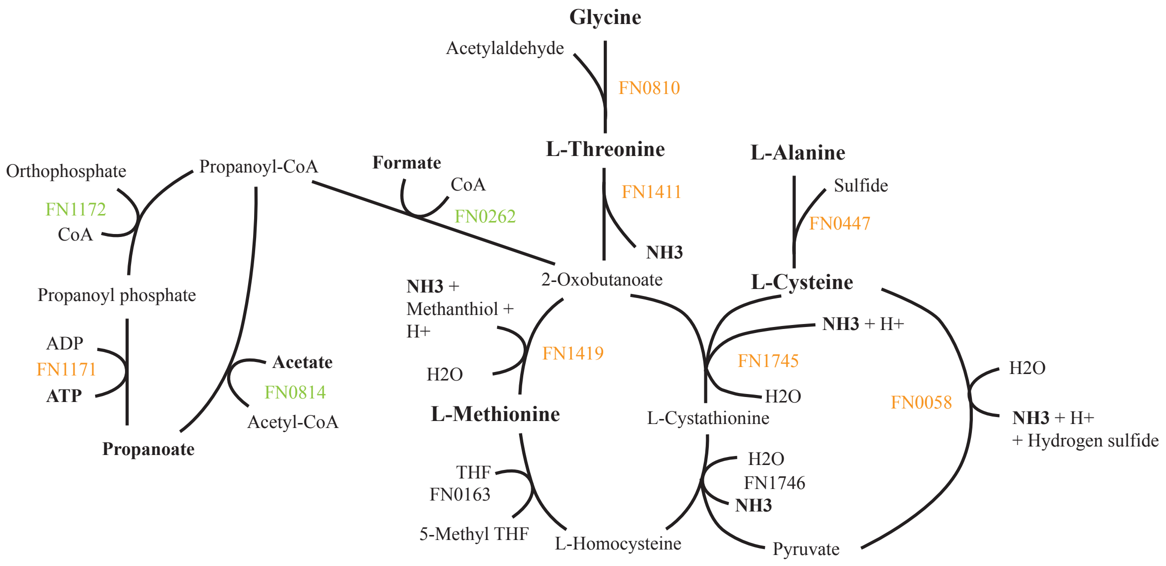

Figure S7. **FnPg vs Fn Other Amino Acid Fermentation.** The diagram shows a schematic of L-alanine, L-cysteine, glycine, L-methionine, and L-threonine fermentation for the *F. nucleatum* with *P. gingivalis* sample compared to *F. nucleatum*. Proteins catalyzing each step are shown by their *F. nucleatum* FN number. Red numbers indicate increased levels in the first condition compared to the second condition, green decreased levels, yellow no statistical change, and black undetected in at least one of the conditions.

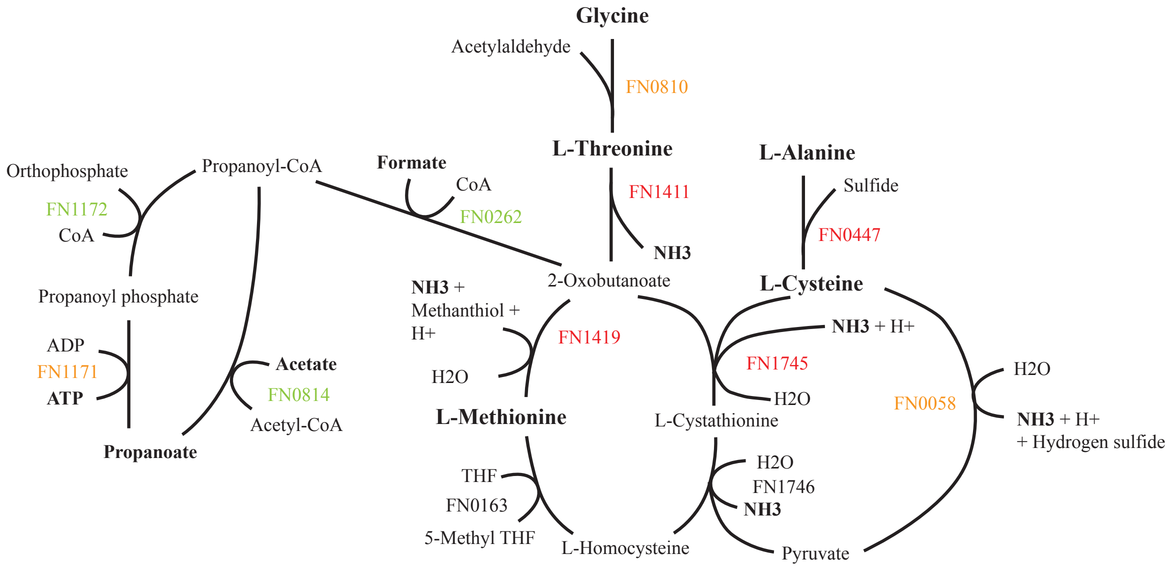

Figure S8. FnSg vs Fn Other Amino Acid Fermentation. Labels and color coding as described for Figure S7, for the *F. nucleatum* with *S. gordonii* comparison to *F. nucleatum*.

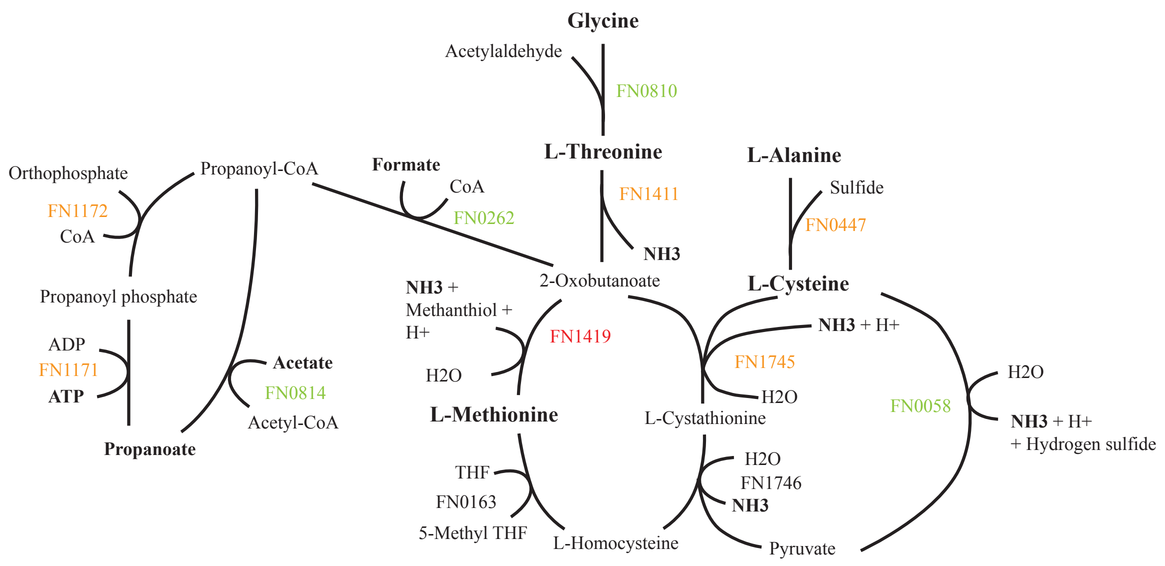

Figure S9. FnPgSg vs Fn Other Amino Acid Fermentation. Labels and color coding as described for Figure S7, for the *F. nucleatum* with *P. gingivalis* and *S. gordonii* comparison to *F. nucleatum*.

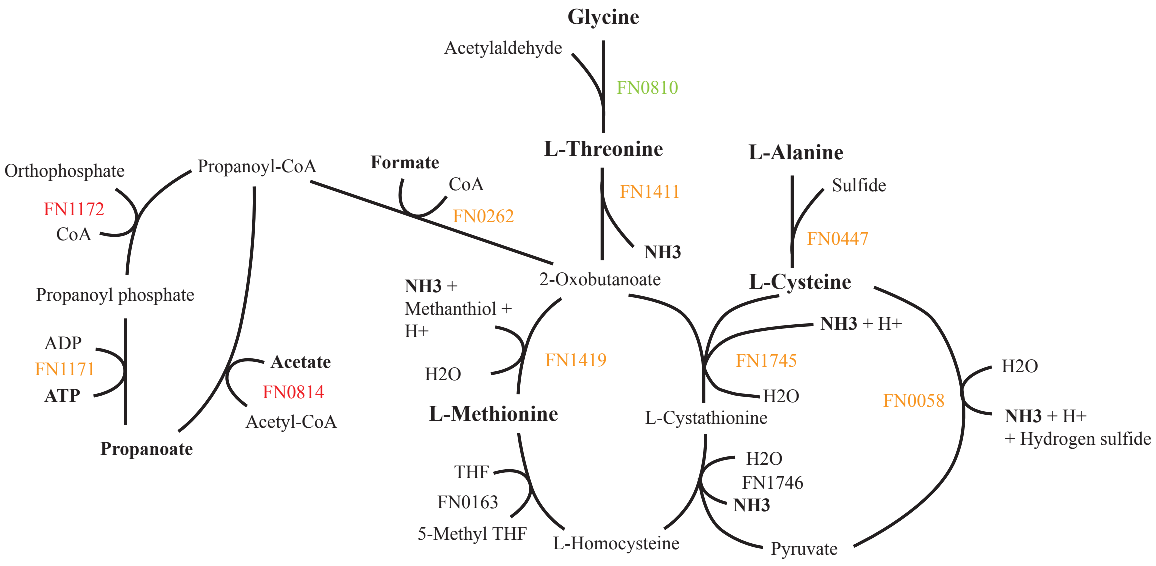

Figure S10. FnPgSg vs FnPg Other Amino Acid Fermentation. Labels and color coding as described for Figure S7, for the *F. nucleatum* with *P. gingivalis* and *S. gordonii* comparison to *F. nucleatum* with *P. gingivalis*.

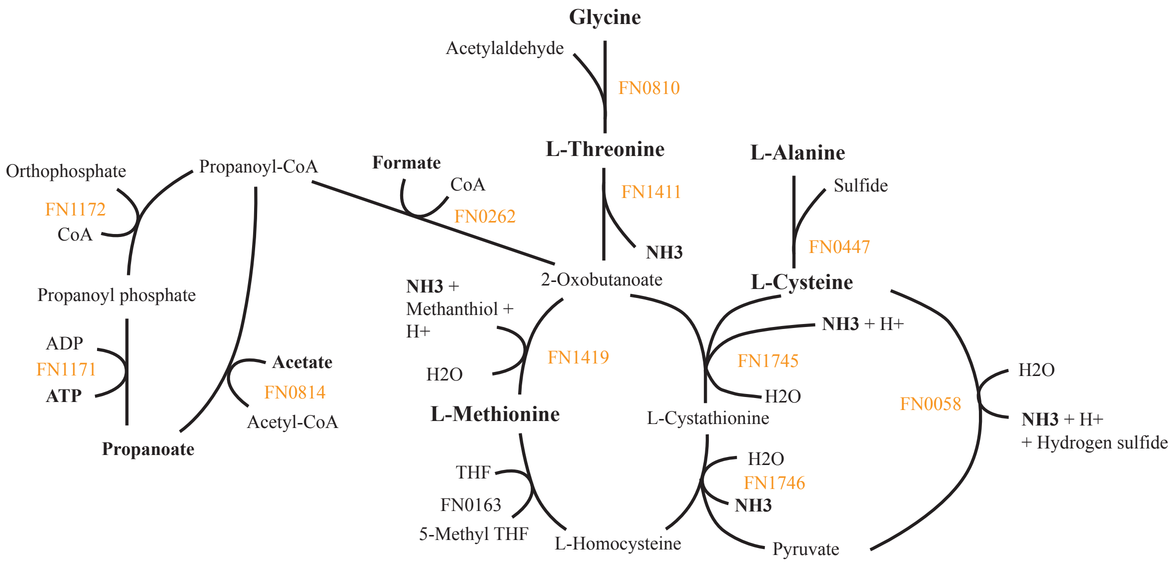

Figure S11. FnSg vs FnPg Other Amino Acid Fermentation. Labels and color coding as described for Figure S7, for the *F. nucleatum* with *S. gordonii* comparison to *F. nucleatum* with *P. gingivalis*.

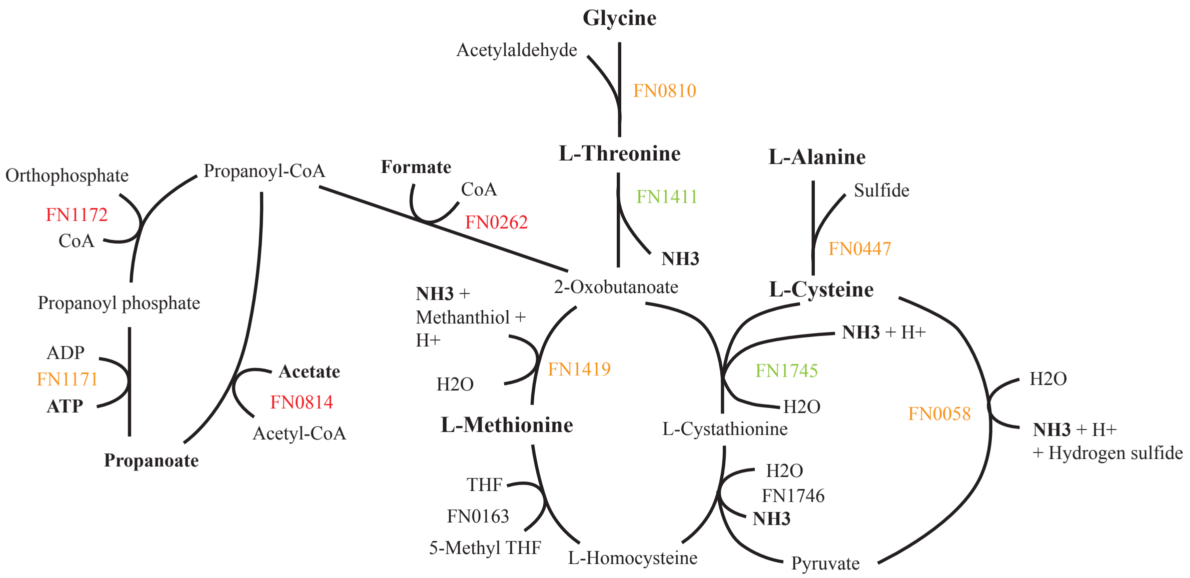

Figure S12. FnPgSg vs FnSg Other Amino Acid Fermentation. Labels and color coding as described for Figure S7, for the *F. nucleatum* with *P. gingivalis* and *S. gordonii* comparison to *F. nucleatum* with *S. gordonii*.
