# Supplementary material for: Proteomics of Fusobacterium nucleatum within a model developing oral microbial community
Source: Microbiologyopen. 2014 Aug 25;3(5):729–51. doi: 10.1002/mbo3.204 (PMC4234264; doi:10.1002/mbo3.204)

		FnPg vs Fn				*FnSg vs Fn*				*FnPgSg vs Fn*				*FnPgSg vs FnPg*				*FnSg vs FnPg*				*FnPgSg vs FnSg*			
ORF	Protein	Ratio	Sum	*q*-Val	*p*-Val	Ratio	Sum	*q*-Val	*p*-Val	Ratio	Sum	*q*-Val	*p*-Val	Ratio	Sum	*q*-Val	*p*-Val	Ratio	Sum	*q*-Val	*p*-Val	Ratio	Sum	*q*-Val	*p*-Val
**FN0001**	AAL94214.1| Chromosomal replication initiator protein dnaA	-0.861	8.211	5.373e-2	9.891e-2	-0.721	8.536	3.344e-3	1.286e-2	-1.995	6.873	5.464e-4	1.117e-3	-1.135	6.216	1e-1	2.195e-1	0.140	7.675	3.783e-1	7.944e-1	-1.275	6.541	7.573e-4	1.334e-3
**FN0004**	AAL94217.1| Inner membrane protein	-0.611	11.841			-1.041	11.595	1.344e-4	2.314e-4	0.743	12.991	1.429e-2	6.168e-2	1.354	12.583			-0.430	10.984			1.784	12.338	4.439e-3	1.286e-2
**FN0005**	AAL94218.1| Jag protein	-2.353	9.381	1.156e-3	5.965e-4	-1.546	10.372	4.85e-4	1.179e-3	0.049	11.579	1.256e-1	7.273e-1	2.402	9.430	1.077e-4	9.734e-6	0.807	8.020	9.688e-2	8.044e-2	1.595	10.422	8.613e-5	7.124e-5
**FN0006**	AAL94219.1| Thiophene and furan oxidation protein THDF	-0.118	8.902			0.271	9.476	6.231e-3	2.671e-2	-0.914	7.902	1.152e-3	2.895e-3	-0.797	7.988			0.389	9.358			-1.186	8.562	7.973e-6	1.958e-6
**FN0007**	AAL94220.1| Glucose inhibited division protein A	-1.490	8.166	7.004e-4	2.603e-4	-0.993	8.847	3.101e-3	1.17e-2	-1.690	7.762	1.687e-4	2.265e-4	-0.200	6.476	1.591e-1	4.339e-1	0.497	7.357	1.598e-1	2.137e-1	-0.696	7.157	2.992e-2	1.242e-1
**FN0008**	AAL94221.1| Quinolinate synthetase A																	-0.559	7.026						
**FN0009**	AAL94222.1| L-aspartate oxidase																	-0.306	9.594						
**FN0017**	AAL94230.1| Hypothetical protein					-0.525	5.744	3.621e-3	1.424e-2																
**FN0018**	AAL94231.1| Hypothetical protein	-1.322	10.369			-1.340	10.536	7.478e-5	1.095e-4	-0.591	10.897	1.44e-4	1.826e-4	0.731	9.778			-0.018	9.214			0.749	9.945	1.226e-4	1.125e-4
**FN0019**	AAL94232.1| Transcription-repair coupling factor					0.493	4.631			0.483	4.232											-0.010	5.114		
**FN0021**	AAL94234.1| 4-diphosphocytidyl-2-C-methyl-D-erythritol kinase																								
**FN0022**	AAL94235.1| Hypothetical protein	0.356	12.744	2.373e-1	6.936e-1	-2.619	9.954	5.088e-7	5.976e-8	-0.906	11.279	2.08e-3	6.345e-3	-1.261	11.838	1.31e-1	3.315e-1	-2.975	10.310	1.469e-1	1.814e-1	1.713	9.048	4.072e-3	1.141e-2
**FN0023**	AAL94236.1| Short-chain fatty acids transporter													0.322	7.241			0.355	7.459			-0.033	7.781		
**FN0024**	AAL94237.1| Hypothetical exported 24-amino acid repeat protein	-2.410	10.338	2.211e-4	4.451e-5	-2.296	10.636			-1.508	11.036	3.217e-4	5.553e-4	0.902	8.830	3.244e-2	4.269e-2	0.113	8.227			0.788	9.128		
**FN0025**	AAL94238.1| Hypothetical exported 24-amino acid repeat protein	-2.614	8.229			-2.514	8.514			-2.155	8.484			0.459	6.074			0.100	5.900			0.359	6.359		
**FN0026**	AAL94239.1| Hypothetical exported 24-amino acid repeat protein									0.872	9.200	1.2e-2	5.066e-2												
**FN0029**	AAL94242.1| Flavodoxin									-0.920	5.564														

Log_2 Ratios

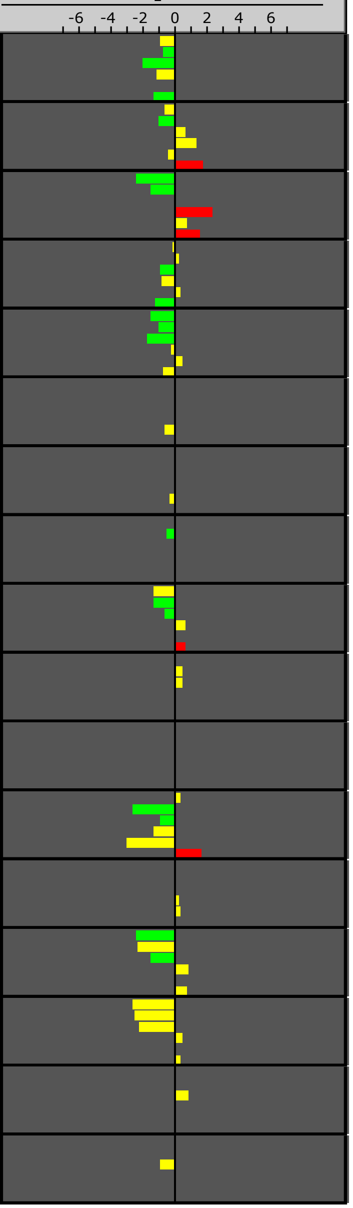

○ Show detected proteins only
○ Show all proteins
☐ Filter by category:
GO: amino acid transport

Proteins found: 1424

Enter (or paste) list of ORFs

Find ORFs

Test	Cutoff
q-Value	.005
p-Value	

Dot Plots
Dot Plots

	Signif	Direction	Applies To
red	yes	+	ratios, bars
yellow	no	n/a	bars
green	yes	–	ratios, bars
pink	yes	+	*p*- , *q*-Values
light green	yes	–	*p*- , *q*-Values

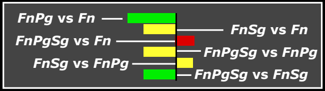

		FnPg vs *Fn*				*FnSg* vs *Fn*				*FnPgSg* vs *Fn*				*FnPgSg* vs *FnPg*				*FnSg* vs *FnPg*				*FnPgSg* vs *FnSg*			
ORF	Description	Ratio	Sum	*q*-Val	*p*-Val	Ratio	Sum	*q*-Val	*p*-Val	Ratio	Sum	*q*-Val	*p*-Val	Ratio	Sum	*q*-Val	*p*-Val	Ratio	Sum	*q*-Val	*p*-Val	Ratio	Sum	*q*-Val	*p*-Val
**FN0030**	AAL94243.1| 5-nitroimidazole antibiotic resistance protein	0.941	12.742	2.833e-2	4.197e-2	0.770	12.755	3.016e-4	6.467e-4	-0.029	11.568	1.437e-1	8.494e-1	-0.969	12.713	2.885e-2	3.595e-2	-0.171	13.696	2.765e-1	4.991e-1	-0.799	12.726	5.533e-4	8.615e-4
**FN0031**	AAL94244.1| unknown	1.926	7.752	6.48e-3	6.475e-3	1.640	7.650	3.089e-6	9.391e-7	0.497	6.119	3.703e-2	1.839e-1	-1.429	8.250	8.203e-3	5.576e-3	-0.287	9.576	1.584e-1	2.104e-1	-1.142	8.147	1.66e-3	3.674e-3
**FN0033**	AAL94246.1| unknown	-0.537	12.649	5.583e-2	1.044e-1	-0.389	12.980	1.059e-7	6.028e-9	-1.022	11.959	2.845e-8	7.664e-10	-0.485	11.626	1.056e-1	2.401e-1	0.147	12.444	3.222e-1	6.206e-1	-0.633	11.958	1.07e-6	1.34e-7
**FN0034**	AAL94247.1| unknown	0.033	9.683	2.871e-1	8.979e-1	-0.699	9.136	2.259e-5	1.945e-5	0.619	10.066	1.553e-3	4.415e-3	0.587	10.302	3.262e-2	4.304e-2	-0.732	9.168	1.017e-1	8.763e-2	1.319	9.755	6.41e-4	1.067e-3
**FN0038**	AAL94251.1| unknown																								
**FN0039**	AAL94252.1| DNA primase (bacterial type) and small primase-like proteins	0.032	6.887			0.521	7.561	7.269e-4	2.007e-3									0.489	7.593						
**FN0040**	AAL94253.1| Asparaginyl-tRNA synthetase	0.040	18.005	2.345e-1	6.828e-1	0.442	18.592	1.777e-5	1.378e-5	-0.097	17.664	1.467e-2	6.352e-2	-0.137	17.908	1.011e-1	2.234e-1	0.402	18.632	2.871e-2	8.462e-3	-0.539	18.495	1.488e-5	5.633e-6
**FN0041**	AAL94254.1| unknown	-0.530	6.145							-0.402	6.068	5.601e-2	2.941e-1	0.127	5.742										
**FN0043**	AAL94256.1| Hypothetical exported 24-amino acid repeat protein	-0.908	6.908							-1.806	5.806			-0.898	5.102										
**FN0045**	AAL94258.1| Shikimate 5-dehydrogenase					-0.622	6.237																		
**FN0046**	AAL94259.1| 3-dehydroquinate dehydratase	0.453	5.099							1.136	5.579			0.683	6.236										
**FN0047**	AAL94260.1| Exodeoxyribonuclease III	-0.754	12.407	6.949e-3	7.143e-3	-0.690	12.656	1.944e-4	3.696e-4	-0.392	12.565	8.829e-4	2.036e-3	0.362	12.015	5.7e-2	9.732e-2	0.064	11.902	3.588e-1	7.305e-1	0.298	12.263	7.296e-5	5.701e-5
**FN0048**	AAL94261.1| 4-nitrophenylphosphatase	-1.806	9.090	4.751e-4	1.424e-4	-0.904	10.176	7.755e-4	2.196e-3	-1.375	9.318	4.649e-4	9.091e-4	0.432	7.715	1.458e-2	1.347e-2	0.902	8.370	7.266e-3	1.077e-3	-0.470	8.802	1.982e-5	9.147e-6
**FN0049**	AAL94262.1| Hypothetical protein	2.461	6.414			3.827	7.964											1.366	10.425	1.414e-2	2.841e-3				
**FN0050**	AAL94263.1| Fumarate reductase flavoprotein subunit	-2.087	19.021	5.604e-5	5.869e-6	-2.906	18.386	8.682e-5	1.311e-4	-0.308	20.596	2.719e-2	1.28e-1	1.779	18.713	7.945e-3	5.254e-3	-0.820	16.299	8.04e-2	5.463e-2	2.598	18.078	2.221e-3	5.272e-3
**FN0052**	AAL94265.1| Arsenate reductase	-1.633	9.711							-0.988	10.152	5.96e-5	5.203e-5	0.645	8.723										
**FN0054**	AAL94267.1| Tyrosyl-tRNA synthetase	-0.656	14.433	4.46e-2	7.663e-2	-0.304	14.970	6.079e-6	2.647e-6	0.180	15.065	7.739e-4	1.728e-3	0.836	14.613	2.865e-2	3.557e-2	0.352	14.314	1.918e-1	2.926e-1	0.484	15.149	1.567e-4	1.577e-4

Log$_2$ Ratios

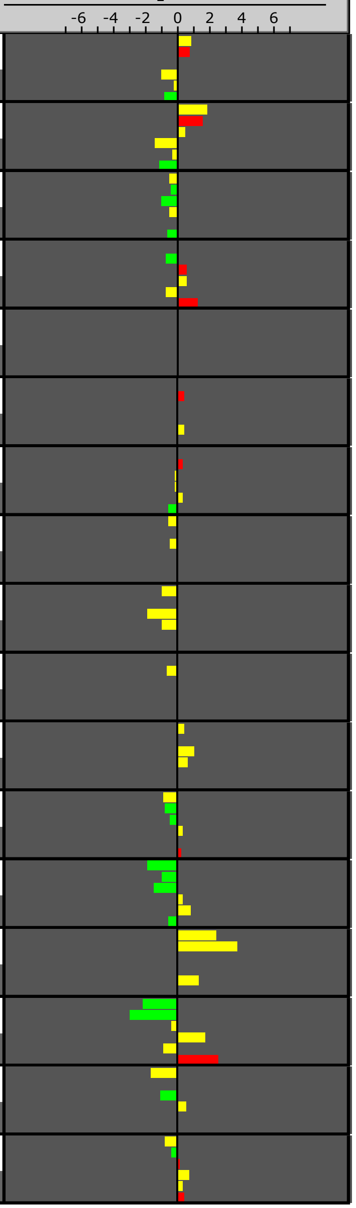

Show detected proteins only
Show all proteins
Filter by category:
GO: amino acid transport

Proteins found: 1424

Enter (or paste) list of ORFs

Find ORFs

Test	Cutoff
q-Value	.005
p-Value	

Dot Plots

Dot Plots

	Signif	Direction	Applies To
(red)	yes	+	ratios, bars
(yellow)	no	n/a	bars
(green)	yes	–	ratios, bars
(pink)	yes	+	*p*- , *q*-Values
(light green)	yes	–	

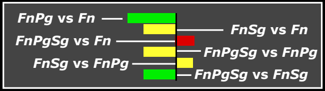

Fusobacterium nucleatum

ORF	Protein	*FnPg vs Fn* Ratio	Sum	*q*-Val	*p*-Val	*FnSg vs Fn* Ratio	Sum	*q*-Val	*p*-Val	*FnPgSg vs Fn* Ratio	Sum	*q*-Val	*p*-Val
FN0058	AAL94271.1| Cysteine desulfhydrase	-0.140	15.152	1.373e-1	3.386e-1	0.314	15.791	8.433e-2	4.894e-1	-0.885	14.203	1.496e-5	5.701e-6
FN0059	AAL94272.1| NifU protein	-1.187	10.292	1.566e-3	9.088e-4	-0.273	11.390	6.53e-2	3.713e-1	-0.329	10.946	5.812e-3	2.184e-2
FN0060	AAL94273.1| D-alanyl-D-alanine carboxypeptidase	-1.856	8.478	1.087e-3	5.368e-4	-2.452	8.067			-0.809	9.321	1.62e-5	6.499e-6
FN0061	AAL94274.1| Thermostable carboxypeptidase 1	0.092	12.784	2.844e-1	8.859e-1	0.005	12.882	1.523e-1	9.547e-1	0.017	12.506	1.065e-1	6.032e-1
FN0062	AAL94275.1| Hypothetical cytosolic protein	-0.934	6.104							0.342	7.177	3.453e-2	1.697e-1
FN0063	AAL94276.1| unknown												
FN0065	AAL94278.1| Transcription accessory protein (S1 RNA binding domain)	-1.267	13.695	4.057e-4	1.097e-4	-0.894	14.252	2.279e-4	4.492e-4	-0.753	14.005	4.603e-4	8.975e-4
FN0066	AAL94279.1| Two component system histidine kinase												
FN0067	AAL94280.1| Isoleucyl-tRNA synthetase	-0.988	17.171	2.064e-3	1.353e-3	-1.276	17.068	1.701e-5	1.293e-5	-0.530	17.426	2.072e-5	9.503e-6
FN0069	AAL94282.1| Glycyl-tRNA synthetase alpha chain	0.373	15.301	6.215e-2	1.221e-1	-0.019	15.094	1.435e-1	8.903e-1	-0.085	14.640	7.328e-2	3.959e-1
FN0070	AAL94283.1| Glycyl-tRNA synthetase beta chain	-0.301	17.538	4.828e-2	8.539e-2	0.067	18.090	2.792e-2	1.478e-1	-0.461	17.173	1.237e-4	1.47e-4
FN0071	AAL94284.1| GTP cyclohydrolase I	-0.392	6.392			-0.296	6.671						
FN0072	AAL94285.1| 2-amino-4-hydroxy-6-hydroxymethyldihydropteridine pyrophosphokinase	0.159	12.943	2.316e-1	6.721e-1	0.620	13.588	2.807e-4	5.872e-4	-0.484	12.097	7.448e-5	7.088e-5
FN0073	AAL94286.1| Dihydropteroate synthase	-0.702	10.009	6.226e-2	1.224e-1	-0.528	10.367	2.285e-2	1.186e-1	-1.025	9.482	1.825e-5	7.944e-6
FN0074	AAL94287.1| Ethanolamine utilization protein eutS	-0.272	8.272			-0.234	8.494	9.103e-2	5.322e-1	-0.710	7.629		
FN0076	AAL94289.1| Ethanolamine two-component response regulator												
FN0077	AAL94290.1| Ethanolamine two-component sensor kinase	-1.392	4.561			0.632	6.769						

ORF	Protein	*FnPgSg vs FnPg* Ratio	Sum	*q*-Val	*p*-Val	*FnSg vs FnPg* Ratio	Sum	*q*-Val	*p*-Val	*FnPgSg vs FnSg* Ratio	Sum	*q*-Val	*p*-Val
FN0058	AAL94271.1| Cysteine desulfhydrase	-0.746	14.267	1.756e-2	1.775e-2	0.454	15.651	2.212e-1	3.627e-1	-1.200	14.906	2.644e-2	1.08e-1
FN0059	AAL94272.1| NifU protein	0.859	9.963	9.856e-4	2.352e-4	0.914	10.203	1.057e-1	9.419e-2	-0.055	11.061	1.66e-1	8.61e-1
FN0060	AAL94273.1| D-alanyl-D-alanine carboxypeptidase	1.047	7.669	1.152e-2	9.384e-3	-0.596	6.211			1.643	7.258		
FN0061	AAL94274.1| Thermostable carboxypeptidase 1	-0.075	12.801	2.744e-1	9.066e-1	-0.086	12.974	4.062e-1	8.929e-1	0.012	12.899	1.725e-1	9.02e-1
FN0062	AAL94275.1| Hypothetical cytosolic protein	1.277	6.447										
FN0063	AAL94276.1| unknown												
FN0065	AAL94278.1| Transcription accessory protein (S1 RNA binding domain)	0.513	12.942	1.35e-3	3.901e-4	0.372	12.985	1.016e-1	8.749e-2	0.141	13.499	7.609e-2	3.562e-1
FN0066	AAL94279.1| Two component system histidine kinase												
FN0067	AAL94280.1| Isoleucyl-tRNA synthetase	0.458	16.642	2.677e-2	3.218e-2	-0.288	16.080	1.432e-1	1.712e-1	0.746	16.538	2.253e-5	1.099e-5
FN0069	AAL94282.1| Glycyl-tRNA synthetase alpha chain	-0.457	15.217	5.162e-2	8.355e-2	-0.392	15.467	1.169e-1	1.137e-1	-0.066	15.009	1.227e-1	6.048e-1
FN0070	AAL94283.1| Glycyl-tRNA synthetase beta chain	-0.161	17.077	1.322e-1	3.363e-1	0.368	17.790	7.383e-2	4.585e-2	-0.528	17.629	5.561e-4	8.676e-4
FN0071	AAL94284.1| GTP cyclohydrolase I					0.095	6.280						
FN0072	AAL94285.1| 2-amino-4-hydroxy-6-hydroxymethyldihydropteridine pyrophosphokinase	-0.643	12.460	9.599e-2	2.054e-1	0.461	13.747	1.498e-1	1.899e-1	-1.103	13.105	8.499e-5	6.994e-5
FN0073	AAL94286.1| Dihydropteroate synthase	-0.323	8.984	1.884e-1	5.471e-1	0.174	9.665	3.632e-1	7.446e-1	-0.496	9.342	5.559e-2	2.512e-1
FN0074	AAL94287.1| Ethanolamine utilization protein eutS	-0.439	7.561			0.038	8.222			-0.476	7.784		
FN0076	AAL94289.1| Ethanolamine two-component response regulator												
FN0077	AAL94290.1| Ethanolamine two-component sensor kinase					2.023	5.378						

Log$_2$ Ratios: -6 -4 -2 0 2 4 6

Show detected proteins only
Show all proteins
Filter by category:
GO: amino acid transport

Proteins found: 1424

Enter (or paste) list of ORFs

Find ORFs

Test	Cutoff
q-Value	.005
p-Value	

Dot Plots
Dot Plots

	Signif	Direction	Applies To
red	yes	+	ratios, bars
yellow	no	n/a	bars
green	yes	–	ratios, bars
pink	yes	+	*p*- , *q*-Values
light green	yes	–	*p*- , *q*-Values

FnPg vs Fn
FnPgSg vs Fn
FnSg vs FnPg
FnSg vs Fn
FnPgSg vs FnPg
FnPgSg vs FnSg

Spectral Counts
Fn Summary Table

Fusobacterium nucleatum

Hackett Laboratory UW

Fn Summary Table | FnPg vs Fn | FnSg vs Fn | FnPgSg vs Fn | FnPgSg vs FnPg | FnSg vs FnPg | FnPgSg vs FnSg | Fn Coverage

ORF	Protein	*FnPg vs Fn* Ratio	Sum	*q*-Val	*p*-Val	*FnSg vs Fn* Ratio	Sum	*q*-Val	*p*-Val	*FnPgSg vs Fn* Ratio	Sum	*q*-Val	*p*-Val	*FnPgSg vs FnPg* Ratio	Sum	*q*-Val	*p*-Val	*FnSg vs FnPg* Ratio	Sum	*q*-Val	*p*-Val	*FnPgSg vs FnSg* Ratio	Sum	*q*-Val	*p*-Val
FN0078	AAL94291.1| Ethanolamine utilization protein eutA	1.882	7.835			1.437	7.574			-0.209	5.540			-2.092	7.626	8.903e-4	2.004e-4	-0.445	9.457	4.438e-2	1.819e-2	-1.646	7.365	7.87e-5	6.302e-5
FN0079	AAL94292.1| Ethanolamine ammonia-lyase heavy chain	0.842	14.037	1.25e-1	3.005e-1	1.007	14.387	4.635e-5	5.506e-5	-1.622	11.369	8.866e-5	9.085e-5	-2.464	12.415	5.957e-2	1.037e-1	0.165	15.228	3.665e-1	7.552e-1	-2.629	12.764	5.808e-5	4.252e-5
FN0080	AAL94293.1| Ethanolamine ammonia-lyase light chain	0.902	14.623	4.69e-4	1.392e-4	0.180	14.085	3.374e-2	1.823e-1	-0.932	12.585	9.368e-4	2.197e-3	-1.834	13.691	3.228e-6	6.998e-8	-0.723	14.987	3.075e-3	2.684e-4	-1.112	13.153	5.42e-4	8.365e-4
FN0081	AAL94294.1| Ethanolamine utilization protein eutL	1.042	14.363	7.517e-2	1.565e-1	1.350	14.856	2.767e-3	1.014e-2	-1.614	11.503	3.069e-5	1.767e-5	-2.656	12.749	3.831e-2	5.419e-2	0.308	15.898	2.664e-1	4.741e-1	-2.964	13.242	1.679e-3	3.728e-3
FN0082	AAL94295.1| Ethanolamine utilization protein eutM	1.056	8.758			1.343	9.229			0.534	8.032			-0.522	9.292			0.287	10.285			-0.809	9.763	2.699e-2	1.106e-1
FN0083	AAL94296.1| Ethanolamine utilization protein eutM precursor	1.151	15.460	6.376e-2	1.263e-1	0.714	15.208	2.881e-3	1.066e-2	-0.929	13.177	1.214e-3	3.115e-3	-2.079	14.531	4.203e-2	6.167e-2	-0.436	16.359	2.349e-1	3.977e-1	-1.643	14.280	1.247e-3	2.574e-3
FN0084	AAL94297.1| Acetaldehyde dehydrogenase (acetylating)	0.119	10.872	4.019e-2	6.639e-2	-0.211	10.727	3.136e-2	1.681e-1	-0.929	9.620	4.369e-4	8.395e-4	-1.048	9.943	4.175e-3	1.977e-3	-0.329	10.846	8.358e-2	5.918e-2	-0.719	9.798	4.306e-3	1.232e-2
FN0086	AAL94299.1| Hypothetical protein																								
FN0087	AAL94300.1| Ethanolamine utilization protein eutN																	-1.925	6.569						
FN0088	AAL94301.1| Hypothetical protein					0.686	5.654																		
FN0089	AAL94302.1| Ethanolamine permease																								
FN0090	AAL94303.1| Ethanolamine utilization protein eutQ																	-0.576	6.001	1.706e-1	2.397e-1				
FN0091	AAL94304.1| Phosphoserine phosphatase																								
FN0092	AAL94305.1| NADPH-dependent butanol dehydrogenase	0.492	10.680	1.89e-1	5.192e-1	0.830	11.203	1.614e-5	1.197e-5	-1.780	8.204	3.482e-5	2.242e-5	-2.272	8.900	7.374e-2	1.389e-1	0.338	11.695	2.975e-1	5.529e-1	-2.611	9.423	1.505e-6	2e-7
FN0093	AAL94306.1| Thioredoxin	-0.842	16.090	4.414e-3	3.817e-3	-1.914	15.202	4.362e-4	1.028e-3	-0.043	16.685	1.195e-1	6.867e-1	0.798	16.047	1.861e-4	2.318e-5	-1.072	14.361	1.411e-3	6.286e-5	1.871	15.159	1.909e-5	8.535e-6
FN0100	AAL94309.1| Flavodoxins/hemoproteins	-1.317	9.040	1.103e-2	1.274e-2	-1.531	9.011	2.386e-3	8.457e-3	-0.431	9.723	2.228e-2	1.019e-1	0.886	8.609	3.179e-3	1.339e-3	-0.214	7.693	1.124e-1	1.056e-1	1.100	8.579	1.371e-4	1.308e-4
FN0102	AAL94311.1| Ribonucleoside-diphosphate reductase alpha chain									-0.826	6.687	7.66e-3	3.016e-2												

Log_2 Ratios

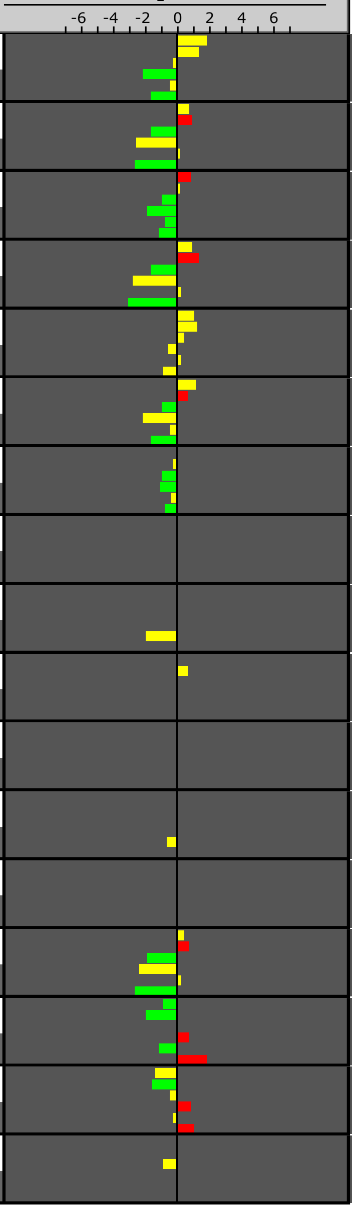

Show detected proteins only
Show all proteins
Filter by category:
GO: amino acid transport

Proteins found: 1424

Enter (or paste) list of ORFs

Find ORFs

Test	Cutoff
q-Value	.005
p-Value	

Dot Plots
Dot Plots

	Signif	Direction	Applies To
red	yes	+	ratios, bars
yellow	no	n/a	bars
green	yes	–	ratios, bars
pink	yes	+	*p*- , *q*-Values
light green	yes	–	*p*- , *q*-Values

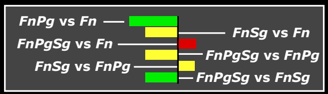

Spectral Counts
Fn Summary Table

Fusobacterium nucleatum

Hackett Laboratory UW

Fn Summary Table | FnPg vs Fn | FnSg vs Fn | FnPgSg vs Fn | FnPgSg vs FnPg | FnSg vs FnPg | FnPgSg vs FnSg | Fn Coverage

ORF	*FnPg vs Fn*				*FnSg vs Fn*				*FnPgSg vs Fn*				*FnPgSg vs FnPg*				*FnSg vs FnPg*				*FnPgSg vs FnSg*				Protein
	Ratio	Sum	*q*-Val	*p*-Val	Ratio	Sum	*q*-Val	*p*-Val	Ratio	Sum	*q*-Val	*p*-Val	Ratio	Sum	*q*-Val	*p*-Val	Ratio	Sum	*q*-Val	*p*-Val	Ratio	Sum	*q*-Val	*p*-Val	
**FN0103**									-1.146	5.146															AAL94312.1| Ribonucleoside-diphosphate reductase beta chain
**FN0106**	0.481	13.751	4.514e-3	3.942e-3	0.078	13.533	4.921e-2	2.742e-1	0.122	13.189	3.395e-2	1.665e-1	-0.359	13.873	1.242e-2	1.048e-2	-0.403	14.014	2.89e-2	8.559e-3	0.044	13.655	1.123e-1	5.472e-1	AAL94315.1| Hypothetical protein
**FN0108**	1.611	7.947	4.839e-3	4.367e-3	1.487	8.008	9.4e-4	2.763e-3	0.104	6.236			-1.507	8.051			-0.124	9.619	2.944e-1	5.448e-1	-1.384	8.112			AAL94317.1| Microcin C7 self-immunity protein mccF
**FN0110**	0.226	17.666	1.41e-1	3.503e-1	-0.130	17.494	4.738e-3	1.946e-2	-0.076	17.160	6.415e-2	3.414e-1	-0.302	17.590	1.079e-1	2.49e-1	-0.357	17.720	1.514e-1	1.94e-1	0.054	17.418	1.069e-1	5.177e-1	AAL94319.1| Seryl-tRNA synthetase
**FN0111**																									AAL94320.1| unknown
**FN0113**	0.647	11.693	1.201e-2	1.411e-2	1.648	12.879	2.225e-5	1.905e-5	-1.470	9.373	3.872e-5	2.807e-5	-2.117	10.223	2.542e-3	9.639e-4	1.001	13.526	1.855e-3	1.012e-4	-3.118	11.409	3.382e-6	5.898e-7	AAL94322.1| Heat-inducible transcription repressor hrcA
**FN0114**	-0.198	13.784	6.152e-3	6.025e-3	-0.075	14.091	1.259e-1	7.656e-1	0.309	14.087	1.204e-2	5.086e-2	0.507	14.093	1.711e-2	1.713e-2	0.123	13.893	3.326e-1	6.506e-1	0.384	14.400	3.79e-2	1.627e-1	AAL94323.1| GrpE protein
**FN0116**	0.278	20.274	1.647e-1	4.297e-1	0.796	20.976	2.799e-2	1.482e-1	0.016	19.808	1.367e-1	8.016e-1	-0.263	20.290	1.638e-1	4.51e-1	0.517	21.255	1.923e-1	2.94e-1	-0.780	20.992	3.583e-2	1.525e-1	AAL94325.1| Chaperone protein dnaK
**FN0117**	1.094	6.521			1.687	7.298											0.593	8.392							AAL94326.1| O6-methylguanine-DNA methyltransferase
**FN0118**	-0.984	12.145	2.653e-3	1.89e-3	-0.444	12.870	2.452e-3	8.742e-3	-0.308	12.617	5.011e-6	1.143e-6	0.675	11.837	1.078e-2	8.521e-3	0.540	11.886	3.846e-2	1.406e-2	0.135	12.562	4.615e-2	2.036e-1	AAL94327.1| Chaperone protein dnaJ
**FN0119**	1.972	9.137			2.217	9.566	7.543e-3	3.351e-2									0.245	11.539							AAL94328.1| Flavodoxin
**FN0123**	-0.480	5.124			-0.005	5.782	1.538e-1	9.655e-1									0.474	5.303							AAL94332.1| ATPase
**FN0127**																	0.209	5.302							AAL94333.1| Fe-S oxidoreductase
**FN0128**	-0.982	9.077	3.37e-3	2.639e-3	-0.987	9.255	6.848e-3	2.989e-2	-1.639	8.215	3.046e-4	5.156e-4	-0.658	7.437	8.064e-3	5.4e-3	-0.006	8.274	4.311e-1	9.893e-1	-0.652	7.616	6.216e-2	2.842e-1	AAL94334.1| Spermidine/putrescine-binding protein
**FN0129**																									AAL94335.1| Urease accessory protein ureG
**FN0130**	-1.187	7.187			-0.093	8.466			-0.159	8.012			1.028	7.028			1.094	7.279			-0.066	8.307	1.747e-1	9.157e-1	AAL94336.1| ABC transporter ATP-binding protein
**FN0132**	-0.378	5.575			-0.484	5.654			0.425	6.174			0.803	5.999	3.244e-2	4.268e-2	-0.105	5.275			0.908	6.078			AAL93916.1| Hemolysin

Log_2 Ratios

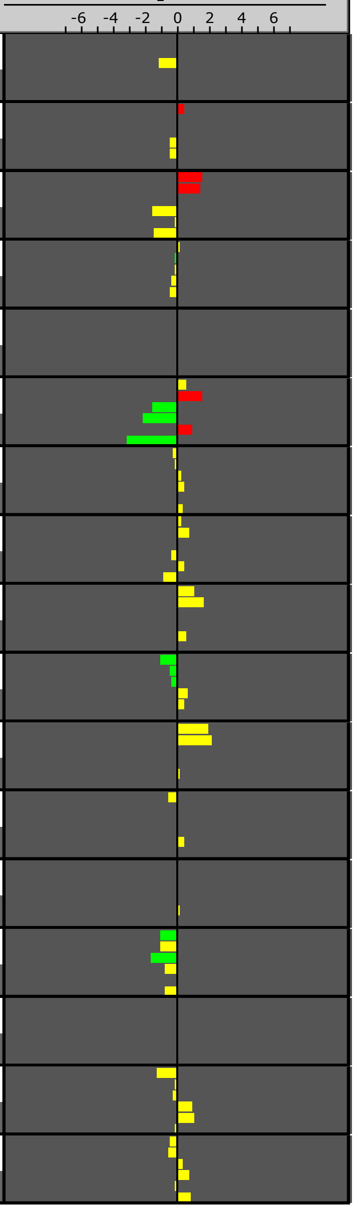

Show detected proteins only
Show all proteins
Filter by category:
GO: amino acid transport

Proteins found: 1424

Enter (or paste) list of ORFs

Find ORFs

Test	Cutoff
q-Value	.005
p-Value	

Dot Plots
Dot Plots

	Signif	Direction	Applies To
(red)	yes	+	ratios, bars
(yellow)	no	n/a	bars
(green)	yes	–	ratios, bars
(pink)	yes	+	*p*- , *q*-Values
(light green)	yes	–	*p*- , *q*-Values

FnPg vs Fn
FnSg vs Fn
FnPgSg vs Fn
FnPgSg vs FnPg
FnSg vs FnPg
FnPgSg vs FnSg

Fusobacterium nucleatum

	FnPg vs Fn				*FnSg vs Fn*				*FnPgSg vs Fn*				*FnPgSg vs FnPg*				*FnSg vs FnPg*				*FnPgSg vs FnSg*				
ORF	Ratio	Sum	*q*-Val	*p*-Val	Ratio	Sum	*q*-Val	*p*-Val	Ratio	Sum	*q*-Val	*p*-Val	Ratio	Sum	*q*-Val	*p*-Val	Ratio	Sum	*q*-Val	*p*-Val	Ratio	Sum	*q*-Val	*p*-Val	Protein
**FN0147**	-0.966	10.162	1.213e-2	1.427e-2	-0.653	10.659	7.029e-4	1.917e-3	-0.837	10.087	3.929e-6	8.249e-7	0.129	9.325	2.133e-1	6.501e-1	0.313	9.694	1.912e-1	2.911e-1	-0.184	9.822	2.855e-2	1.178e-1	AAL94353.1| PLSX protein
**FN0148**	-0.177	16.026	5.895e-2	1.129e-1	-0.203	16.184	7.082e-2	4.05e-1	-0.054	15.945	3.576e-2	1.767e-1	0.123	15.972	1.034e-1	2.317e-1	-0.026	16.008	4.133e-1	9.196e-1	0.149	16.131	1.1e-1	5.344e-1	AAL94354.1| 3-oxoacyl-[acyl-carrier-protein] synthase III
**FN0149**	-0.210	15.914	1.751e-1	4.672e-1	-1.231	15.077	7.309e-4	2.022e-3	-0.142	15.778	8.342e-2	4.585e-1	0.068	15.772	2.541e-1	8.167e-1	-1.021	14.867	7.968e-2	5.358e-2	1.089	14.935	1.855e-3	4.21e-3	AAL94355.1| Malonyl-CoA-[acyl-carrier-protein] transacylase
**FN0150**	-0.746	11.656	8.561e-4	3.661e-4	-1.071	11.516	6.755e-5	9.361e-5	-0.199	12.000	1.166e-2	4.904e-2	0.548	11.458	3.038e-3	1.249e-3	-0.325	10.769	6.703e-2	3.818e-2	0.873	11.317	2.254e-4	2.589e-4	AAL94356.1| Acyl carrier protein
**FN0151**	-0.793	19.262	5.296e-2	9.695e-2	-0.661	19.578	7.627e-3	3.394e-2	-0.016	19.835	1.398e-1	8.225e-1	0.776	19.246	5.85e-2	1.01e-1	0.132	18.786	3.797e-1	7.991e-1	0.645	19.562	9.456e-3	3.276e-2	AAL94357.1| 3-oxoacyl-[acyl-carrier-protein] synthase
**FN0152**	0.663	5.337			0.571	5.429			0.444	4.915			-0.219	5.781			-0.092	6.092			-0.126	5.874			AAL94358.1| Ribonuclease III
**FN0153**																									AAL94359.1| Oxygen-independent coproporphyrinogen III oxidase
**FN0154**									-0.307	5.666															AAL94360.1| Ribonuclease G
**FN0155**					-1.221	5.865			-0.070	6.627											1.151	5.795			AAL94361.1| Hypothetical protein
**FN0156**	-0.497	7.347	1.051e-1	2.418e-1	-0.196	7.832	1.188e-1	7.161e-1	0.307	7.947	1.625e-4	2.15e-4	0.804	7.654	4.903e-2	7.721e-2	0.301	7.335	3.425e-1	6.801e-1	0.503	8.139	7.204e-2	3.354e-1	AAL94362.1| Phosphopantetheine adenylyltransferase
**FN0157**					-0.237	6.014	1.592e-3	5.208e-3																	AAL94363.1| DNA repair protein RadA
**FN0158**	-0.530	9.206	3.161e-2	4.837e-2	0.623	10.543	2.694e-3	9.808e-3	-0.730	8.802	5.307e-3	1.963e-2	-0.200	8.476	1.591e-1	4.339e-1	1.153	10.013	6.506e-3	8.764e-4	-1.353	9.813	4.684e-4	6.842e-4	AAL94364.1| DNA-binding protein
**FN0161**																									AAL94367.1| RNA-directed DNA polymerase
**FN0164**	-0.769	15.858	1.169e-3	6.076e-4	-0.239	16.573	5.724e-2	3.226e-1	-1.297	15.126	3.319e-4	5.799e-4	-0.528	14.561	2.508e-2	2.927e-2	0.530	15.804	1.25e-1	1.294e-1	-1.058	15.276	1.113e-2	3.991e-2	AAL94370.1| Anhydro-N-acetylmuramyl-tripeptide amidase
**FN0170**	0.112	13.730	2.35e-1	6.85e-1	0.706	14.509	8.89e-3	4.064e-2	0.137	13.551	1.406e-3	3.848e-3	0.025	13.867	2.779e-1	9.227e-1	0.594	14.620	9.347e-2	7.553e-2	-0.569	14.645	1.633e-2	6.229e-2	AAL94376.1| GTP-binding protein
**FN0173**	-0.778	10.805	8.21e-2	1.76e-1	0.018	11.786	1.478e-1	9.213e-1	0.284	11.663	6.328e-3	2.416e-2	1.062	11.089	4.654e-2	7.143e-2	0.796	11.007	1.436e-1	1.723e-1	0.266	12.069	4.161e-2	1.811e-1	AAL94379.1| Hypothetical protein
**FN0174**	-0.124	12.708	7.411e-2	1.536e-1	-0.094	12.923	4.424e-2	2.442e-1	0.849	13.478	3.396e-3	1.143e-2	0.973	13.558	1.273e-2	1.087e-2	0.030	12.799	1.465e-1	1.801e-1	0.943	13.772	4.162e-3	1.176e-2	AAL94380.1| Enoyl-[acyl-carrier-protein] reductase

Log$_2$ Ratios

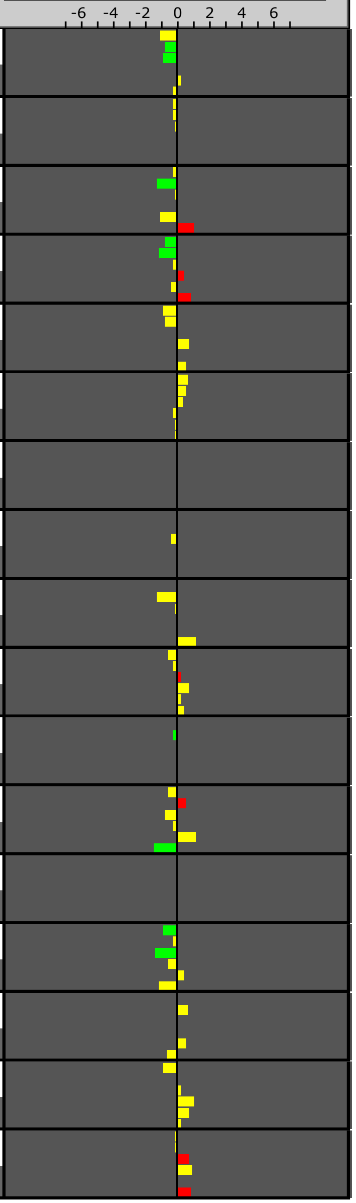

Show detected proteins only
Show all proteins
Filter by category:
GO: amino acid transport

Proteins found: 1424

Enter (or paste) list of ORFs

Find ORFs

Test	Cutoff
q-Value	.005
p-Value	

Dot Plots
Dot Plots

	Signif	Direction	Applies To
(red)	yes	+	ratios, bars
(yellow)	no	n/a	bars
(green)	yes	–	ratios, bars
(pink)	yes	+	*p*- , *q*-Values
(light green)	yes	–	*p*- , *q*-Values

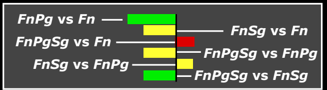

	FnPg vs Fn				*FnSg vs Fn*				*FnPgSg vs Fn*				*FnPgSg vs FnPg*				*FnSg vs FnPg*				*FnPgSg vs FnSg*				
ORF	Ratio	Sum	*q*-Val	*p*-Val	Ratio	Sum	*q*-Val	*p*-Val	Ratio	Sum	*q*-Val	*p*-Val	Ratio	Sum	*q*-Val	*p*-Val	Ratio	Sum	*q*-Val	*p*-Val	Ratio	Sum	*q*-Val	*p*-Val	Description
**FN0175**	-0.146	8.119	1.763e-1	4.719e-1	0.907	9.357	1.103e-3	3.347e-3	-0.214	7.848			-0.068	7.905			1.054	9.211	6.296e-3	8.271e-4	-1.121	9.143			AAL94381.1| Cell division inhibitor MinC
**FN0176**	0.146	15.160	7.733e-2	1.624e-1	0.785	15.983	1.232e-4	2.054e-4	0.209	15.019	2.682e-2	1.26e-1	0.063	15.369	2.052e-1	6.188e-1	0.638	16.129	3.194e-3	2.913e-4	-0.576	16.192	7.056e-4	1.212e-3	AAL94382.1| Cell division inhibitor MinD
**FN0177**	-1.762	5.841							-0.050	7.349	1.285e-1	7.461e-1	1.712	5.790											AAL94383.1| Cell division inhibitor MinE
**FN0178**	-1.229	11.977	5.813e-4	1.931e-4	-1.525	11.866	9.301e-7	1.343e-7	-0.978	12.024	3.175e-5	1.88e-5	0.251	10.999	7.306e-2	1.371e-1	-0.296	10.637	1.257e-1	1.31e-1	0.546	10.888	1.39e-3	2.937e-3	AAL94384.1| UNC-44 ankyrins
**FN0179**	1.260	11.281	9.709e-2	2.186e-1	-0.200	10.006	8.015e-2	4.63e-1	-0.461	9.357	1.498e-2	6.506e-2	-1.721	10.821	8.15e-2	1.602e-1	-1.460	11.266	1.493e-1	1.882e-1	-0.260	9.545	9.701e-2	4.647e-1	AAL94385.1| Ankyrin repeat proteins
**FN0180**																					0.708	5.680			AAL94386.1| Tetratricopeptide repeat family protein
**FN0181**	0.337	9.382	1.942e-1	5.371e-1	0.505	9.734	1.944e-2	9.927e-2	-0.669	8.172	3.035e-2	1.456e-1	-1.006	8.713	8.424e-2	1.682e-1	0.168	10.072	3.454e-1	6.89e-1	-1.174	9.065	1.618e-3	3.554e-3	AAL94387.1| Hypothetical protein
**FN0182**	1.741	13.880	9.767e-2	2.203e-1	3.099	15.422	3.534e-7	3.74e-8	-0.020	11.915	1.551e-1	9.294e-1	-1.761	13.860	9.97e-2	2.183e-1	1.358	17.164	6.972e-2	4.109e-2	-3.119	15.402	1.447e-5	5.349e-6	AAL94388.1| Sarcosine oxidase alpha subunit
**FN0183**	0.669	14.512	1.728e-1	4.589e-1	1.523	15.550	3.969e-5	4.325e-5	0.222	13.861	4.094e-2	2.068e-1	-0.448	14.734	1.98e-1	5.877e-1	0.853	16.220	1.397e-1	1.623e-1	-1.301	15.772	4.587e-5	3.095e-5	AAL94389.1| Glycerol-3-phosphate dehydrogenase
**FN0185**	-0.190	8.139	2.186e-1	6.238e-1	0.084	8.597	1.319e-1	8.073e-1	-0.703	7.422	3.725e-2	1.852e-1	-0.513	7.436	6.967e-3	4.177e-3	0.274	8.408	8.045e-3	1.272e-3	-0.787	7.895	3.582e-4	4.845e-4	AAL94391.1| Hypothetical protein
**FN0188**																									AAL94394.1| Peptide methionine sulfoxide reductase
**FN0189**													-1.400	7.244			-0.627	8.202			-0.773	6.802	4.787e-3	1.42e-2	AAL94395.1| Two-component response regulator yesN
**FN0190**	-0.711	5.687			-0.535	6.047											0.175	5.336	3.371e-1	6.64e-1					AAL94396.1| Two-component sensor kinase yesM
**FN0191**	0.135	9.220	2.069e-1	5.817e-1	0.605	9.874	1.279e-2	6.207e-2	-0.956	7.924	5.339e-3	1.977e-2	-1.092	8.263	2.389e-2	2.73e-2	0.470	10.009	1.254e-1	1.304e-1	-1.562	8.917	2.196e-3	5.195e-3	AAL94397.1| helix-turn-helix DNA-binding protein
**FN0192**	-2.863	10.863			-2.532	11.379	5.468e-5	6.886e-5	-1.560	11.963	2.817e-4	4.648e-4	1.303	9.303			0.331	8.516			0.972	9.819	7.334e-4	1.277e-3	AAL94398.1| Dipeptide-binding protein
**FN0197**					-0.162	4.806																			AAL94403.1| Methyltransferase
**FN0198**									0.119	5.211	1.087e-1	6.173e-1													AAL94404.1| Transcriptional regulatory protein

Log$_2$ Ratios: -6 -4 -2 0 2 4 6

Show detected proteins only
Show all proteins
Filter by category:
GO: amino acid transport

Proteins found: 1424

Enter (or paste) list of ORFs

Find ORFs

Test	Cutoff
q-Value	.005
p-Value	

Dot Plots
Dot Plots

	Signif	Direction	Applies To
red	yes	+	ratios, bars
yellow	no	n/a	bars
green	yes	–	ratios, bars
pink	yes	+	*p*- , *q*-Values
light green	yes	–	*p*- , *q*-Values

FnPg vs Fn — FnSg vs Fn — FnPgSg vs Fn — FnPgSg vs FnPg — FnSg vs FnPg — FnPgSg vs FnSg

ORF	FnPg vs Fn Ratio	Sum	*q*-Val	*p*-Val	FnSg vs Fn Ratio	Sum	*q*-Val	*p*-Val	FnPgSg vs Fn Ratio	Sum	*q*-Val	*p*-Val	FnPgSg vs FnPg Ratio	Sum	*q*-Val	*p*-Val	FnSg vs FnPg Ratio	Sum	*q*-Val	*p*-Val	FnPgSg vs FnSg Ratio	Sum	*q*-Val	*p*-Val
**FN0199**	0.589	11.112	1.742e-3	1.054e-3	0.776	11.484	1.755e-4	3.272e-4	0.034	10.353	1.476e-1	8.765e-1	-0.556	11.146	2.543e-2	2.985e-2	0.187	12.073	9.129e-2	7.165e-2	-0.742	11.518	3.161e-3	8.198e-3
	AAL94405.1| Hypothetical protein																							
**FN0200**	-1.336	20.587	1.877e-2	2.497e-2	-2.133	19.975	1.742e-7	1.324e-8	-1.580	20.140	6.631e-7	7.146e-8	-0.244	19.007	2.26e-1	6.999e-1	-0.797	18.638	2.076e-1	3.302e-1	0.553	18.395	8.884e-6	2.354e-6
	AAL94406.1| Biotin carboxyl carrier protein of glutaconyl-COA decarboxylase																							
**FN0202**	-0.590	19.813	2.697e-3	1.932e-3	-0.967	19.622	6.741e-5	9.332e-5	0.046	20.246	1.346e-1	7.871e-1	0.637	19.860	3.092e-2	3.975e-2	-0.376	19.031	1.858e-2	4.165e-3	1.013	19.668	4.71e-3	1.39e-2
	AAL94408.1| Glutaconate CoA-transferase subunit A																							
**FN0203**	0.084	21.144	2.282e-1	6.59e-1	-0.582	20.663	7.506e-4	2.098e-3	-0.570	20.287	9.913e-4	2.364e-3	-0.654	20.575	3.03e-2	3.86e-2	-0.666	20.747	6.635e-2	3.747e-2	0.012	20.093	1.755e-1	9.207e-1
	AAL94409.1| Glutaconate CoA-transferase subunit B																							
**FN0204**	-0.493	21.327	1.772e-2	2.309e-2	-0.312	21.692	7.16e-3	3.155e-2	-0.211	21.405	4.17e-3	1.466e-2	0.282	21.116	5.991e-2	1.045e-1	0.181	21.199	1.987e-1	3.098e-1	0.101	21.482	7.827e-2	3.674e-1
	AAL94410.1| Glutaconyl-CoA decarboxylase A subunit																							
**FN0206**	-0.444	12.567	1.01e-1	2.3e-1	-1.164	12.032	2.516e-4	5.09e-4	-0.994	11.813	1.258e-6	1.822e-7	-0.550	11.572	1.166e-1	2.787e-1	-0.719	11.587	1.542e-1	2.005e-1	0.170	11.038	5.194e-2	2.327e-1
	AAL94412.1| Activator of (R)-2-hydroxyglutaryl-CoA dehydratase																							
**FN0207**	-0.327	19.040	1.127e-1	2.635e-1	-0.565	18.986	1.392e-5	9.757e-6	-0.338	18.825	8.945e-5	9.205e-5	-0.011	18.701	2.878e-1	9.689e-1	-0.238	18.659	2.657e-1	4.725e-1	0.227	18.648	1.91e-4	2.051e-4
	AAL94413.1| (R)-2-hydroxyglutaryl-CoA dehydratase alpha-subunit																							
**FN0208**	-0.455	17.834	2.923e-3	2.161e-3	-0.732	17.742	3.443e-4	7.682e-4	-0.233	17.852	4.31e-4	8.251e-4	0.222	17.601	1.793e-2	1.826e-2	-0.277	17.287	5.334e-2	2.581e-2	0.499	17.509	8.199e-4	1.491e-3
	AAL94414.1| (R)-2-hydroxyglutaryl-CoA dehydratase beta-subunit																							
**FN0209**	-0.386	17.986	7.596e-4	2.975e-4	-0.604	17.952	5.39e-3	2.255e-2	-0.455	17.713	3.089e-5	1.788e-5	-0.069	17.531	8.653e-2	1.75e-1	-0.218	17.566	1.837e-1	2.717e-1	0.149	17.497	9.177e-2	4.371e-1
	AAL94415.1| Hypothetical cytosolic protein																							
**FN0212**	-0.009	9.149	3.045e-1	9.76e-1	-0.493	8.850	1.5e-3	4.861e-3	-0.593	8.361	5.343e-3	1.979e-2	-0.584	8.556	8.801e-2	1.795e-1	-0.484	8.841	1.657e-1	2.279e-1	-0.101	8.257	1.233e-1	6.084e-1
	AAL94418.1| Hypothetical protein																							
**FN0214**																								
	AAL94420.1| Crossover junction endodeoxyribonuclease ruvC																							
**FN0218**	0.516	14.609	6.593e-2	1.321e-1	-0.964	13.314	7.957e-5	1.179e-4	-1.149	12.740	1.827e-4	2.538e-4	-1.665	13.460	1.814e-2	1.856e-2	-1.480	13.829	4.689e-2	2.01e-2	-0.185	12.164	4.958e-2	2.207e-1
	AAL94424.1| Anthranilate synthase component II																							
**FN0219**	0.304	4.866			1.435	6.180			0.986	5.344			0.682	5.852			1.130	6.485			-0.448	7.166		
	AAL94425.1| Autolysin response regulator																							
**FN0221**	0.220	11.462	3.049e-2	4.613e-2	-0.581	10.846	2.334e-5	2.036e-5	0.683	11.721	9.094e-3	3.691e-2	0.463	12.145	4.803e-2	7.484e-2	-0.801	11.066	1.214e-2	2.271e-3	1.264	11.528	4.095e-3	1.15e-2
	AAL94427.1| Carbon starvation protein A																							
**FN0224**	-0.910	8.992	1.518e-2	1.886e-2	-1.410	8.676	1.294e-3	4.071e-3	-0.636	9.062	7.015e-3	2.723e-2	0.274	8.356	1.616e-1	4.431e-1	-0.500	7.766	1.859e-1	2.773e-1	0.774	8.040	1.765e-2	6.82e-2
	AAL94430.1| Excinuclease ABC subunit B																							
**FN0225**																	-0.507	3.677						
	AAL94431.1| Gluconate permease																							
**FN0226**	-0.258	12.152	4.659e-3	4.129e-3	-1.034	11.561	2.8e-4	5.852e-4	-0.441	11.765	3.279e-6	6.51e-7	-0.183	11.711	1.556e-2	1.502e-2	-0.776	11.303	3.264e-3	3.059e-4	0.592	11.120	1.971e-3	4.537e-3
	AAL94432.1| Pyridoxal phosphate biosynthetic protein pdxA																							

Log$_2$ Ratios

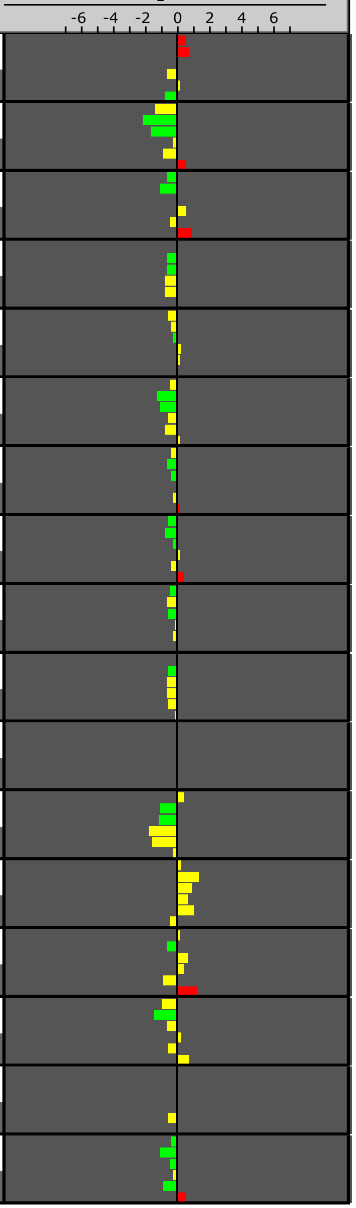

Show detected proteins only
Show all proteins
Filter by category:
GO: amino acid transport

Proteins found: 1424

Enter (or paste) list of ORFs

Find ORFs

Test	Cutoff
q-Value	.005
p-Value	

Dot Plots
Dot Plots

	Signif	Direction	Applies To
(red)	yes	+	ratios, bars
(yellow)	no	n/a	bars
(green)	yes	–	ratios, bars
(pink)	yes	+	*p*- , *q*-Values
(light green)	yes	–	*p*- , *q*-Values

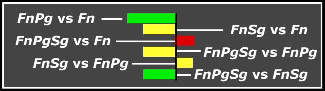

	FnPg vs Fn				*FnSg vs Fn*				*FnPgSg vs Fn*				*FnPgSg vs FnPg*				*FnSg vs FnPg*				*FnPgSg vs FnSg*				
ORF	Ratio	Sum	*q*-Val	*p*-Val	Ratio	Sum	*q*-Val	*p*-Val	Ratio	Sum	*q*-Val	*p*-Val	Ratio	Sum	*q*-Val	*p*-Val	Ratio	Sum	*q*-Val	*p*-Val	Ratio	Sum	*q*-Val	*p*-Val	Description
FN0227																	0.230	4.414							AAL94433.1| Hypothetical protein
FN0233	-0.178	12.478	1.701e-1	4.491e-1	-0.734	12.106	2.775e-4	5.783e-4	-0.444	12.009	2.958e-3	9.715e-3	-0.266	12.035	1.317e-1	3.341e-1	-0.557	11.929	1.174e-1	1.145e-1	0.291	11.663	6.601e-3	2.126e-2	AAL94439.1| Hypothetical protein
FN0234	-0.582	7.501			-0.310	7.958	1.264e-2	6.125e-2									0.272	7.375							AAL94440.1| unknown
FN0235																									AAL94441.1| ABC transporter ATP-binding protein
FN0236	0.731	16.378	1.67e-1	4.38e-1	-1.222	14.608	2.996e-4	6.409e-4	-0.360	15.082	2.733e-3	8.803e-3	-1.091	16.017	1.278e-1	3.191e-1	-1.954	15.340	1.515e-1	1.943e-1	0.862	14.248	6.066e-4	9.851e-4	AAL94442.1| ABC transporter substrate-binding protein
FN0237																									AAL94443.1| ABC transporter permease protein
FN0238	-0.136	10.982	2.095e-1	5.91e-1	-1.033	10.270	1.535e-4	2.777e-4	-0.547	10.368	8.482e-3	3.398e-2	-0.410	10.436	1.077e-1	2.483e-1	-0.897	10.134	8.445e-2	6.049e-2	0.486	9.723	2.967e-2	1.23e-1	AAL94444.1| Hypothetical protein
FN0240	-0.078	13.135	1.549e-1	3.959e-1	-0.797	12.601	8.865e-4	2.574e-3	-0.155	12.854	3.066e-2	1.474e-1	-0.077	12.981	1.268e-1	3.154e-1	-0.719	12.523	2.403e-2	6.248e-3	0.642	12.446	3.401e-3	8.998e-3	AAL94446.1| Thymidylate synthase
FN0241	0.165	9.300	2.215e-1	6.345e-1	0.394	9.714	2.811e-2	1.489e-1	-1.298	7.633	8.746e-3	3.524e-2	-1.464	8.002	2.475e-2	2.871e-2	0.229	9.879	2.275e-1	3.785e-1	-1.693	8.416	6.776e-4	1.148e-3	AAL94447.1| Dihydrofolate reductase
FN0242	-0.011	12.011			0.936	13.142	2.68e-3	9.744e-3	-1.748	10.070	2.019e-5	9.157e-6	-1.737	10.263			0.947	13.131			-2.684	11.394	8.481e-4	1.565e-3	AAL94448.1| Trk system potassium uptake protein trkA
FN0243	0.210	9.190			0.487	9.651	5.927e-2	3.348e-1	-2.031	6.746			-2.241	7.160			0.276	9.862			-2.517	7.621			AAL94449.1| Poly(A) polymerase
FN0244	-1.191	7.835			-1.640	7.571											-0.449	6.379							AAL94450.1| COP associated protein
FN0245	-0.922	6.922			-0.330	7.699	2.374e-3	8.403e-3	-1.235	6.405			-0.313	5.687			0.592	6.777			-0.905	6.464			AAL94451.1| Copper-exporting ATPase
FN0247	-1.109	14.471	1.669e-2	2.132e-2	-0.290	15.475	6.662e-8	3.15e-9	-0.127	15.249	9.125e-2	5.071e-1	0.982	14.343	2.788e-2	3.415e-2	0.819	14.365	8.486e-2	6.112e-2	0.163	15.347	9.41e-2	4.493e-1	AAL94453.1| Hypothetical cytosolic protein
FN0248													-2.120	8.664			0.180	11.149			-2.300	9.029			AAL94454.1| Hypothetical Exported Protein
FN0249	-1.626	14.269	5.543e-3	5.222e-3	-1.817	14.263	4.337e-4	1.021e-3	-0.813	14.879	2.894e-5	1.594e-5	0.814	13.456	5.184e-2	8.412e-2	-0.190	12.637	3.647e-1	7.496e-1	1.004	13.451	5.799e-3	1.815e-2	AAL94455.1| unknown
FN0250	-0.589	13.778	8.361e-2	1.803e-1	-0.996	13.556	3.358e-3	1.293e-2	0.063	14.226	5.06e-2	2.621e-1	0.652	13.840	7.563e-2	1.439e-1	-0.406	12.967	2.74e-1	4.926e-1	1.058	13.619	4.575e-3	1.338e-2	AAL94456.1| unknown

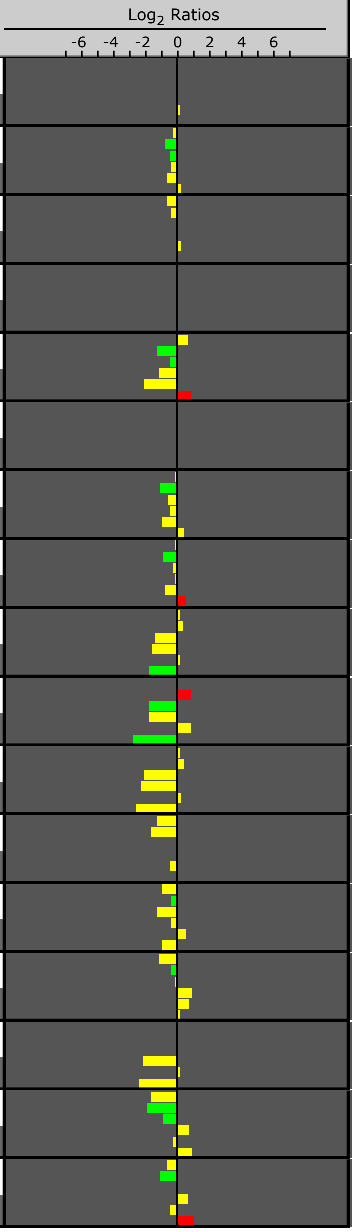

Show detected proteins only
Show all proteins
Filter by category:
GO: amino acid transport

Proteins found: 1424

Enter (or paste) list of ORFs

Find ORFs

Test	Cutoff
q-Value	.005
p-Value	

Dot Plots
Dot Plots

	Signif	Direction	Applies To
(red)	yes	+	ratios, bars
(yellow)	no	n/a	bars
(green)	yes	–	ratios, bars
(pink)	yes	+	*p*- , *q*-Values
(light green)	yes	–	

FnPg vs Fn — FnSg vs Fn
FnPgSg vs Fn — FnPgSg vs FnPg
FnSg vs FnPg — FnPgSg vs FnSg

	FnPg vs *Fn*				*FnSg* vs *Fn*				*FnPgSg* vs *Fn*				*FnPgSg* vs *FnPg*				*FnSg* vs *FnPg*				*FnPgSg* vs *FnSg*				
ORF	Ratio	Sum	*q*-Val	*p*-Val	Ratio	Sum	*q*-Val	*p*-Val	Ratio	Sum	*q*-Val	*p*-Val	Ratio	Sum	*q*-Val	*p*-Val	Ratio	Sum	*q*-Val	*p*-Val	Ratio	Sum	*q*-Val	*p*-Val	Description
**FN0251**	-0.361	15.648	1.844e-1	5.025e-1	-2.496	13.698	1.488e-4	2.659e-4	-0.104	15.702	1.066e-1	6.041e-1	0.257	15.544	2.138e-1	6.518e-1	-2.135	13.336	1.251e-1	1.297e-1	2.392	13.594	2.816e-3	7.096e-3	AAL94457.1| Hypothetical membrane-spanning Protein
**FN0252**	1.100	17.889	6.173e-2	1.209e-1	0.575	17.548	2.44e-4	4.894e-4	-0.338	16.247	1.241e-3	3.212e-3	-1.438	17.551	5.197e-2	8.444e-2	-0.525	18.648	1.974e-1	3.071e-1	-0.913	17.211	3.001e-5	1.622e-5	AAL94458.1| unknown
**FN0253**	-0.332	15.532			0.460	16.509	4.768e-2	2.649e-1	0.155	15.816	2.243e-2	1.027e-1	0.488	15.687			0.792	16.177			-0.305	16.664	8.723e-2	4.134e-1	AAL94459.1| Outer membrane protein
**FN0254**	-0.864	19.214	3.416e-3	2.688e-3	-1.035	19.228	3.087e-6	9.382e-7	-1.120	18.754	4.687e-4	9.187e-4	-0.256	18.094	1.078e-1	2.488e-1	-0.171	18.364	2.091e-1	3.337e-1	-0.085	18.108	1.214e-1	5.977e-1	AAL94460.1| Fusobacterium outer membrane protein family
**FN0258**	-1.074	6.733	3.329e-2	5.181e-2	-1.188	6.803			-1.444	6.159			-0.370	5.289			-0.115	5.730			-0.256	5.359			AAL94464.1| Zinc-transporting ATPase
**FN0259**																									AAL94465.1| Zinc-transporting ATPase
**FN0260**																									AAL94466.1| Transcriptional repressor smtB
**FN0261**	-1.252	9.331			-1.840	8.927	1.912e-5	1.542e-5	-1.556	8.822	3.891e-5	2.839e-5	-0.304	7.774			-0.588	7.675			0.284	7.370	4.485e-2	1.971e-1	AAL94467.1| Pyruvate formate-lyase activating enzyme
**FN0262**	-1.824	23.015	4.137e-3	3.483e-3	-2.335	22.689	1.237e-5	8.314e-6	-0.198	24.438	9.858e-4	2.347e-3	1.627	22.818	9.508e-3	6.973e-3	-0.511	20.865	2.411e-1	4.134e-1	2.137	22.492	8.649e-11	5.458e-13	AAL94468.1| Formate acetyltransferase
**FN0263**	-0.370	16.995	1.843e-1	5.019e-1	-1.850	15.700	1.178e-5	7.68e-6	-1.266	15.895	2.808e-5	1.515e-5	-0.896	15.729	1.265e-1	3.142e-1	-1.480	15.329	1.508e-1	1.925e-1	0.584	14.433	4.604e-3	1.349e-2	AAL94469.1| Peptidyl-prolyl cis-trans isomerase
**FN0264**	-0.911	16.196	1.619e-2	2.05e-2	-0.960	16.331	3.951e-3	1.586e-2	-0.264	16.639	2.791e-3	9.033e-3	0.647	15.932	4.078e-2	5.91e-2	-0.049	15.420	4.096e-1	9.058e-1	0.696	16.067	1.196e-2	4.339e-2	AAL94470.1| Hypothetical protein
**FN0265**	-1.308	8.227			-1.763	7.956	4.807e-4	1.165e-3	-2.080	7.250			-0.773	6.146			-0.455	6.649			-0.318	5.876			AAL94471.1| Cell division protein ftsX
**FN0266**	-1.145	7.145							-2.100	5.985			-0.956	5.044											AAL94472.1| membrane protein related to metalloendopeptidase
**FN0267**	-0.759	5.929			-0.436	6.436											0.323	5.677							AAL94473.1| ATP-NAD kinase
**FN0268**	-0.137	7.964			0.250	8.535			-2.006	5.891			-1.869	5.958			0.386	8.398			-2.256	6.529			AAL94474.1| DNA repair protein recN
**FN0270**					-1.169	5.872	7.405e-4	2.059e-3	-1.136	5.516	2.84e-4	4.698e-4									0.033	4.735	1.521e-1	7.763e-1	AAL94476.1| GTP-binding protein era
**FN0271**	0.783	6.832			0.093	6.327	1.263e-1	7.684e-1	-0.923	4.923			-1.705	5.909			-0.690	7.110			-1.016	5.404			AAL94477.1| Enoyl-CoA hydratase

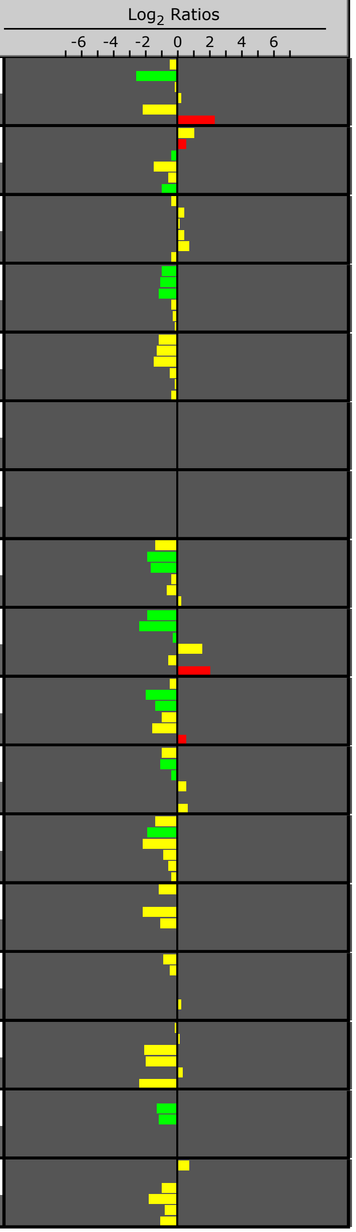

Show detected proteins only
Show all proteins
Filter by category:
GO: amino acid transport

Proteins found: 1424

Enter (or paste) list of ORFs

Find ORFs

Test	Cutoff
q-Value	.005
p-Value	

Dot Plots
Dot Plots

	Signif	Direction	Applies To
red	yes	+	ratios, bars
yellow	no	n/a	bars
green	yes	–	ratios, bars
pink	yes	+	*p*- , *q*-Values
light green	yes	–	*p*- , *q*-Values

FnPg vs Fn
FnPgSg vs Fn
FnSg vs FnPg
FnSg vs Fn
FnPgSg vs FnPg
FnPgSg vs FnSg

	FnPg vs Fn				*FnSg vs Fn*				*FnPgSg vs Fn*				*FnPgSg vs FnPg*				*FnSg vs FnPg*				*FnPgSg vs FnSg*				
ORF	Ratio	Sum	*q*-Val	*p*-Val	Ratio	Sum	*q*-Val	*p*-Val	Ratio	Sum	*q*-Val	*p*-Val	Ratio	Sum	*q*-Val	*p*-Val	Ratio	Sum	*q*-Val	*p*-Val	Ratio	Sum	*q*-Val	*p*-Val	Description
**FN0272**	-0.713	4.713			0.588	6.199											1.301	5.486							AAL94478.1| Acetoacetate:butyrate/acetate coenzyme A transferase
**FN0273**																	-1.074	7.111							AAL94479.1| Butyrate-acetoacetate CoA-transferase subunit B
**FN0276**	1.319	9.021			1.928	9.815			-1.749	5.749			-3.068	7.272			0.609	11.134			-3.677	8.066			AAL94482.1| Sodium-dependent phosphate transporter
**FN0277**	-0.304	7.393	7.804e-2	1.644e-1	-0.494	7.388	2.194e-2	1.134e-1	-0.579	6.914	1.193e-2	5.032e-2	-0.275	6.814	1.447e-2	1.332e-2	-0.190	7.083	2.604e-1	4.596e-1	-0.085	6.808	1.476e-1	7.491e-1	AAL94483.1| Hypothetical protein
**FN0278**	-0.096	16.203	9.868e-2	2.233e-1	-0.560	15.924	4.407e-5	5.09e-5	0.277	16.373	2.187e-2	9.985e-2	0.373	16.480	3.447e-2	4.684e-2	-0.464	15.828	2.93e-2	8.761e-3	0.837	16.201	3.591e-3	9.653e-3	AAL94484.1| Xaa-His dipeptidase
**FN0279**	0.946	13.128	1.364e-1	3.36e-1	1.561	13.927	1.692e-4	3.134e-4	-0.558	11.420	6.44e-6	1.66e-6	-1.504	12.570	1.006e-1	2.215e-1	0.615	14.873	1.932e-1	2.964e-1	-2.119	13.369	1.953e-4	2.115e-4	AAL94485.1| Lipoprotein
**FN0280**	-0.987	13.362	4.062e-2	6.736e-2	-0.787	13.745	4.364e-5	5.015e-5	-0.804	13.341	7.714e-6	2.203e-6	0.183	12.558	2.323e-1	7.252e-1	0.199	12.759	3.492e-1	7.006e-1	-0.017	12.941	1.276e-1	6.33e-1	AAL94486.1| Hypothetical protein
**FN0281**	-0.491	11.913	9.66e-2	2.172e-1	0.841	13.430	6.426e-3	2.77e-2	-1.928	10.273	2.801e-5	1.509e-5	-1.437	9.985	5.448e-2	9.099e-2	1.332	12.939	2.707e-2	7.662e-3	-2.769	11.502	1.689e-3	3.756e-3	AAL94487.1| DNA polymerase III alpha subunit
**FN0282**	-0.017	10.968	2.942e-1	9.293e-1	0.138	11.307	6.655e-2	3.789e-1	-0.165	10.616	8.002e-2	4.372e-1	-0.148	10.803	1.813e-1	5.182e-1	0.154	11.290	2.31e-1	3.874e-1	-0.302	11.142	3.537e-2	1.503e-1	AAL94488.1| Hypothetical protein
**FN0283**	-1.392	8.035			-1.636	7.976			-1.932	7.291			-0.541	6.103			-0.244	6.584			-0.296	6.044			AAL94489.1| tRNA (Guanine-N1) -methyltransferase
**FN0284**	1.792	9.609			1.667	9.668	1.303e-3	4.103e-3	0.337	7.950	7.893e-2	4.305e-1	-1.455	9.946			-0.125	11.460			-1.330	10.005	2.036e-3	4.722e-3	AAL94490.1| 16S rRNA processing protein rimM
**FN0285**	2.474	7.035							1.943	6.300			-0.531	8.978											AAL94491.1| RNA binding protein
**FN0287**	0.047	7.354			-0.460	7.032	2.728e-2	1.441e-1	-0.675	6.428	2.962e-4	4.968e-4	-0.722	6.679			-0.507	7.079			-0.215	6.357	1.081e-1	5.241e-1	AAL94493.1| Dimethyladenosine transferase
**FN0288**	0.806	10.873	8.985e-2	1.977e-1	0.674	10.925	4.516e-5	5.287e-5	0.338	10.201	3.316e-2	1.618e-1	-0.468	11.210	1.418e-1	3.721e-1	-0.132	11.731	3.674e-1	7.582e-1	-0.336	11.262	2.47e-2	1.002e-1	AAL94494.1| Hypoxanthine-guanine phosphoribosyltransferase
**FN0290**																									AAL94496.1| Hemolysin
**FN0291**	1.345	5.298			0.253	4.391			0.270	4.019			-1.075	5.568			-1.092	5.736			0.017	4.661			AAL94497.1| Hemolysin
**FN0294**	-0.806	15.296	8.268e-3	9.034e-3	-1.901	14.385	2.457e-5	2.192e-5	-0.403	15.495	3.238e-4	5.605e-4	0.403	14.893	5.492e-2	9.217e-2	-1.095	13.579	4.651e-2	1.98e-2	1.498	13.983	4.768e-4	7.007e-4	AAL94500.1| Transketolase subunit A

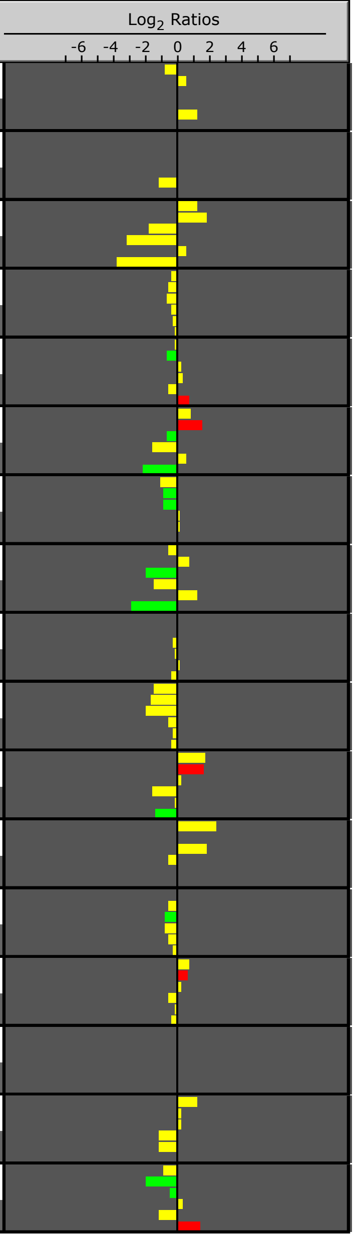

Show detected proteins only
Show all proteins
Filter by category:
GO: amino acid transport

Proteins found: 1424

Enter (or paste) list of ORFs

Find ORFs

Test	Cutoff
q-Value	.005
p-Value	

Dot Plots
Dot Plots

	Signif	Direction	Applies To
(red)	yes	+	ratios, bars
(yellow)	no	n/a	bars
(green)	yes	–	ratios, bars
(pink)	yes	+	*p*- , *q*-Values
(light green)	yes	–	*p*- , *q*-Values

FnPg vs Fn — *FnSg vs Fn*
FnPgSg vs Fn — *FnPgSg vs FnPg*
FnSg vs FnPg — *FnPgSg vs FnSg*

ORF	Protein	*FnPg* vs *Fn*				*FnSg* vs *Fn*				*FnPgSg* vs *Fn*				*FnPgSg* vs *FnPg*				*FnSg* vs *FnPg*				*FnPgSg* vs *FnSg*			
		Ratio	Sum	*q*-Val	*p*-Val	Ratio	Sum	*q*-Val	*p*-Val	Ratio	Sum	*q*-Val	*p*-Val	Ratio	Sum	*q*-Val	*p*-Val	Ratio	Sum	*q*-Val	*p*-Val	Ratio	Sum	*q*-Val	*p*-Val
**FN0295**	AAL94501.1| Transketolase	0.025	14.942	2.939e-1	9.281e-1	-0.904	14.198	1.199e-4	1.978e-4	-0.486	14.228	1.671e-3	4.845e-3	-0.510	14.457	8.978e-2	1.851e-1	-0.929	14.223	9.08e-2	7.081e-2	0.418	13.712	3.996e-3	1.113e-2
**FN0296**	AAL94502.1| Hypothetical cytosolic protein	0.286	11.332	1.34e-1	3.283e-1	0.166	11.396	6.794e-4	1.83e-3	0.648	11.491	2.681e-3	8.596e-3	0.363	11.980	8.364e-2	1.664e-1	-0.120	11.682	3.291e-1	6.404e-1	0.483	12.045	5.828e-3	1.827e-2
**FN0297**	AAL94503.1| ATPase associated with chromosome architecture/replication	0.274	7.325	2.215e-1	6.344e-1	1.165	8.400	1.973e-3	6.701e-3	-0.481	6.366			-0.755	6.844			0.890	8.674	8.369e-2	5.936e-2	-1.646	7.919		
**FN0298**	AAL94504.1| Histidyl-tRNA synthetase	0.398	15.651	1.757e-1	4.697e-1	0.466	15.903	6.287e-4	1.652e-3	-0.036	15.012	9.846e-2	5.526e-1	-0.434	15.615	1.604e-1	4.385e-1	0.068	16.301	4.025e-1	8.796e-1	-0.502	15.867	7.901e-8	2.493e-9
**FN0299**	AAL94505.1| Aspartyl-tRNA synthetase	-0.529	15.954	6.556e-2	1.311e-1	-0.265	16.401	6.204e-4	1.623e-3	-0.090	16.188	1.752e-2	7.775e-2	0.439	15.864	9.365e-2	1.976e-1	0.263	15.873	2.449e-1	4.227e-1	0.175	16.311	3.052e-3	7.844e-3
**FN0305**	AAL94511.1| Iron(III) dicitrate-binding protein									-0.100	3.649														
**FN0307**	AAL94513.1| Iron(III) dicitrate transport ATP-binding protein fecE																								
**FN0308**	AAL94514.1| Iron(III)-binding protein	-0.517	16.825	1.128e-3	5.716e-4	0.247	17.772	2.211e-3	7.692e-3	-0.343	16.795	1.812e-2	8.08e-2	0.174	16.482	1.426e-1	3.748e-1	0.763	17.256	1.419e-3	6.376e-5	-0.590	17.429	3.99e-3	1.11e-2
**FN0309**	AAL94515.1| Iron(III)-transport system permease protein sfuB									-0.359	6.220														
**FN0310**	AAL94516.1| Iron(III)-transport ATP-binding protein sfuC	-0.888	13.719	1.399e-2	1.701e-2	-1.748	13.044	7.401e-6	3.645e-6	-0.601	13.803	3.806e-3	1.311e-2	0.287	13.118	1.467e-1	3.896e-1	-0.860	12.155	1.081e-1	9.826e-2	1.147	12.443	4.279e-3	1.222e-2
**FN0311**	AAL94517.1| Anaerobic ribonucleoside-triphosphate reductase	-0.932	13.015	1.996e-2	2.706e-2	0.278	14.409	2.202e-3	7.657e-3	-0.226	13.517	1.285e-2	5.469e-2	0.706	12.789	4.3e-2	6.368e-2	1.210	13.477	2.582e-2	7.09e-3	-0.504	14.183	8.717e-4	1.621e-3
**FN0313**	AAL94519.1| 16S rRNA m(5)C 967 methyltransferase	-0.943	8.558			-0.127	9.558	1.263e-1	7.682e-1	-2.327	6.970			-1.384	6.231			0.816	8.615			-2.199	7.231		
**FN0314**	AAL94520.1| Caffeoyl-CoA O-methyltransferase																	0.280	6.465						
**FN0315**	AAL94521.1| Transcriptional regulator, AraC family																	-0.369	6.459						
**FN0316**	AAL94522.1| Hypothetical protein	-0.584	9.667			-1.888	8.548	1.29e-3	4.053e-3	-0.223	9.825	4.806e-2	2.474e-1	0.361	9.445			-1.304	7.964			1.665	8.325	5.114e-4	7.712e-4
**FN0317**	AAL94523.1| Tryptophan synthase beta chain	0.441	8.024	1.622e-1	4.21e-1	1.397	9.164	1.666e-6	3.453e-7	-0.857	6.522	1.105e-2	4.61e-2	-1.298	7.167	6.799e-2	1.241e-1	0.956	9.605	7.221e-2	4.392e-2	-2.254	8.308	2.366e-4	2.776e-4
**FN0319**	AAL94525.1| Citrate (pro-3S)-lyase ligase	-0.418	6.033			-0.061	6.574	1.346e-1	8.262e-1	-0.766	5.481			-0.348	5.267			0.357	6.156			-0.705	5.808		

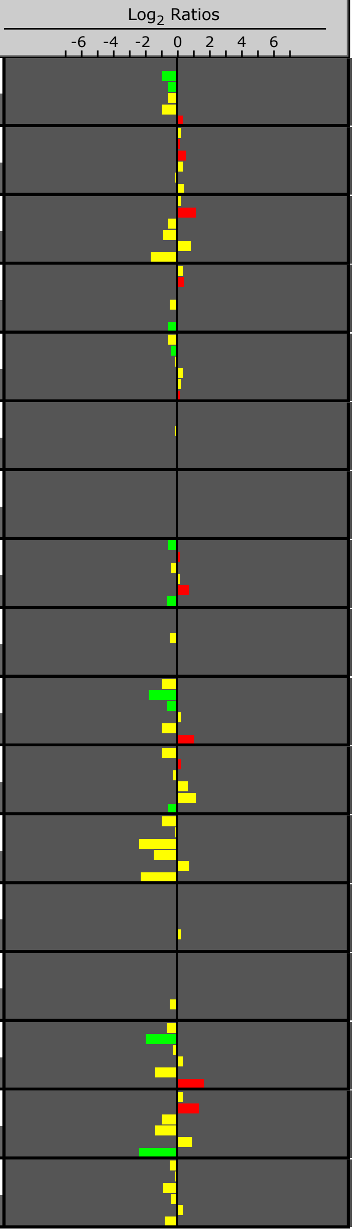

○ Show detected proteins only
○ Show all proteins
☐ Filter by category:
GO: amino acid transport

Proteins found: 1424

Enter (or paste) list of ORFs

Find ORFs

Test	Cutoff
q-Value	.005
p-Value	

Dot Plots
Dot Plots

	Signif	Direction	Applies To
red	yes	+	ratios, bars
yellow	no	n/a	bars
green	yes	–	ratios, bars
pink	yes	+	*p*- , *q*-Values
light green	yes	–	*p*- , *q*-Values

FnPg vs *Fn* — *FnSg* vs *Fn*
FnPgSg vs *Fn* — *FnPgSg* vs *FnPg*
FnSg vs *FnPg* — *FnPgSg* vs *FnSg*

	FnPg vs *Fn*				*FnSg* vs *Fn*				*FnPgSg* vs *Fn*				*FnPgSg* vs *FnPg*				*FnSg* vs *FnPg*				*FnPgSg* vs *FnSg*			
ORF	Ratio	Sum	*q*-Val	*p*-Val	Ratio	Sum	*q*-Val	*p*-Val	Ratio	Sum	*q*-Val	*p*-Val	Ratio	Sum	*q*-Val	*p*-Val	Ratio	Sum	*q*-Val	*p*-Val	Ratio	Sum	*q*-Val	*p*-Val
**FN0320**	-0.089	9.430	1.812e-1	4.901e-1	-1.308	8.395	3.111e-4	6.747e-4	0.484	9.799	1.113e-5	3.673e-6	0.573	9.914	9.886e-3	7.411e-3	-1.219	8.306	9.754e-3	1.653e-3	1.792	8.879	1.383e-4	1.325e-4
	AAL94526.1| Hypothetical cytosolic protein																							
**FN0321**	0.268	13.895	1.544e-1	3.944e-1	0.889	14.701	4.346e-3	1.766e-2	0.075	13.499	8.253e-2	4.529e-1	-0.193	13.971	1.807e-1	5.161e-1	0.621	14.969	8.387e-2	5.963e-2	-0.814	14.776	6.964e-3	2.27e-2
	AAL94527.1| Heat shock protein htpG																							
**FN0322**	0.176	21.395	1.898e-1	5.221e-1	0.336	21.739	1.279e-2	6.211e-2	0.111	21.126	3.666e-2	1.818e-1	-0.065	21.505	2.509e-1	8.028e-1	0.160	21.915	2.94e-1	5.436e-1	-0.225	21.849	3.356e-2	1.415e-1
	AAL94528.1| Fructose-bisphosphate aldolase																							
**FN0325**	-4.103	8.182			-4.235	8.235			1.139	13.220	2.875e-3	9.372e-3	5.242	9.320			-0.132	4.132			5.373	9.373		
	AAL94529.1| LSU ribosomal protein L20P																							
**FN0326**									1.302	7.274														
	AAL94530.1| LSU ribosomal protein L35P																							
**FN0327**	-2.002	11.049			-1.747	11.489	4.453e-4	1.056e-3	-1.855	10.992	1.798e-4	2.48e-4	0.147	9.194			0.255	9.487			-0.108	9.634	1.507e-1	7.677e-1
	AAL94531.1| Bacterial Protein Translation Initiation Factor 3 (IF-3)																							
**FN0329**	-0.359	18.255	9.469e-2	2.116e-1	-0.950	17.849	1.405e-4	2.458e-4	-0.651	17.760	3.448e-4	6.117e-4	-0.292	17.604	1.452e-1	3.843e-1	-0.590	17.490	1.37e-1	1.557e-1	0.299	17.198	7.56e-3	2.512e-2
	AAL94533.1| LSU ribosomal protein L13P																							
**FN0330**	-1.152	12.311	2.453e-3	1.701e-3	-1.857	11.791	2.129e-4	4.128e-4	0.826	14.085	1.409e-4	1.774e-4	1.977	13.137	1.227e-4	1.328e-5	-0.705	10.639	1.334e-1	1.474e-1	2.682	12.617	1.481e-5	5.583e-6
	AAL94534.1| SSU ribosomal protein S9P																							
**FN0331**	-1.077	11.208	3.941e-2	6.465e-2	-0.199	12.270	2.748e-2	1.452e-1	-0.216	11.866	1.345e-6	2.051e-7	0.862	10.992	6.879e-2	1.261e-1	0.878	11.193	1.185e-1	1.166e-1	-0.016	12.055	1.707e-1	8.905e-1
	AAL94535.1| Hypothetical protein																							
**FN0332**													0.102	5.717			0.740	6.539			-0.638	6.641		
	AAL94536.1| Magnesium and cobalt transport protein corA																							
**FN0333**																	-0.137	6.047						
	AAL94537.1| Glycerol uptake operon antiterminator regulatory protein																							
**FN0334**	-0.173	14.224	1.787e-1	4.807e-1	0.615	15.196	1.787e-3	5.959e-3	-0.088	14.104	2.244e-3	6.939e-3	0.084	14.135	2.331e-1	7.288e-1	0.788	15.023	3.433e-2	1.159e-2	-0.704	15.108	2.038e-3	4.73e-3
	AAL94538.1| Aspartate/aromatic aminotransferase																							
**FN0335**	-0.593	20.284	5.665e-2	1.066e-1	0.528	21.589	2.478e-3	8.853e-3	-0.800	19.873	1.018e-4	1.121e-4	-0.207	19.484	1.993e-1	5.932e-1	1.121	20.996	2.564e-2	7.002e-3	-1.328	20.789	8.524e-4	1.576e-3
	AAL94539.1| Outer membrane porin F																							
**FN0336**	-1.726	11.635			-1.423	12.122	2.481e-4	5e-4	-0.757	12.400	1.222e-3	3.141e-3	0.969	10.878			0.303	10.396			0.666	11.366	9.5e-3	3.294e-2
	AAL94540.1| Hypothetical protein																							
**FN0341**	-0.776	11.420			-2.957	9.423	2.092e-4	4.04e-4	0.452	12.443	1.446e-3	4.002e-3	1.228	11.871			-2.181	8.648			3.408	9.875	2.655e-6	4.131e-7
	AAL94545.1| transport protein																							
**FN0342**	-0.221	10.720	2.038e-1	5.707e-1	-0.833	10.292	1.718e-2	8.673e-2	0.166	10.903	8.716e-2	4.817e-1	0.387	10.885	1.069e-1	2.45e-1	-0.612	10.071	1.795e-1	2.61e-1	0.999	10.458	9.333e-3	3.225e-2
	AAL94546.1| Peptidyl-prolyl cis-trans isomerase																							
**FN0343**																								
	AAL94547.1| Hypothetical protein																							

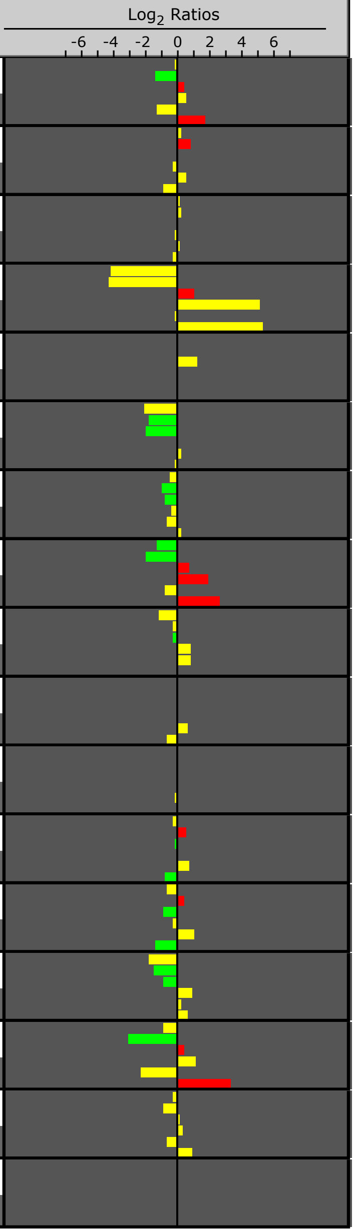

Show detected proteins only
Show all proteins
Filter by category:
GO: amino acid transport

Proteins found: 1424

Enter (or paste) list of ORFs

Find ORFs

Test	Cutoff
q-Value	.005
p-Value	

Dot Plots
Dot Plots

	Signif	Direction	Applies To
red	yes	+	ratios, bars
yellow	no	n/a	bars
green	yes	–	ratios, bars
light red	yes	+	*p*- , *q*-Values
light green	yes	–	*p*- , *q*-Values

FnPg vs Fn — FnSg vs Fn
FnPgSg vs Fn — FnPgSg vs FnPg
FnSg vs FnPg — FnPgSg vs FnSg

	FnPg vs Fn				*FnSg vs Fn*				*FnPgSg vs Fn*				*FnPgSg vs FnPg*				*FnSg vs FnPg*				*FnPgSg vs FnSg*			
ORF	Ratio	Sum	*q*-Val	*p*-Val	Ratio	Sum	*q*-Val	*p*-Val	Ratio	Sum	*q*-Val	*p*-Val	Ratio	Sum	*q*-Val	*p*-Val	Ratio	Sum	*q*-Val	*p*-Val	Ratio	Sum	*q*-Val	*p*-Val
**FN0344**					-2.069	5.239																		
	AAL94548.1| Methyltransferase																							
**FN0347**	-1.193	12.183	1.878e-2	2.499e-2	-0.674	12.887	4.826e-4	1.171e-3	-1.941	11.232	2.077e-4	3.076e-4	-0.747	10.242	1.255e-1	3.104e-1	0.519	11.693	1.886e-1	2.841e-1	-1.266	10.946	1.861e-4	1.979e-4
	AAL94551.1| Phosphatidylserine decarboxylase																							
**FN0348**	-0.459	16.128	5.352e-2	9.837e-2	-0.391	16.381	1.208e-2	5.806e-2	-0.598	15.785	1.01e-3	2.422e-3	-0.139	15.529	2.036e-1	6.118e-1	0.069	15.922	3.841e-1	8.143e-1	-0.208	15.783	6.699e-2	3.091e-1
	AAL94552.1| Nicotinate phosphoribosyltransferase																							
**FN0349**	1.092	7.766	3.465e-2	5.461e-2	1.812	8.670	3.665e-3	1.446e-2	0.087	6.557			-1.005	7.853			0.720	9.762	9.009e-2	6.962e-2	-1.725	8.757		
	AAL94553.1| D-Tyr-tRNATyr deacylase																							
**FN0351**	-0.512	14.137	1.493e-1	3.774e-1	-2.482	12.351	3.184e-4	6.951e-4	-0.271	14.174	1.764e-2	7.835e-2	0.241	13.866	2.225e-1	6.861e-1	-1.971	11.839	1.408e-1	1.652e-1	2.212	12.080	7.791e-6	1.885e-6
	AAL94555.1| unknown																							
**FN0352**	0.044	11.266	2.911e-1	9.157e-1	-0.286	11.121	3.59e-2	1.95e-1	-0.787	10.231	4.061e-3	1.419e-2	-0.831	10.479	8.637e-2	1.745e-1	-0.330	11.164	2.681e-1	4.782e-1	-0.501	10.333	1.264e-2	4.63e-2
	AAL94556.1| NA+/H+ antiporter NHAC																							
**FN0355**	-0.213	15.127	1.534e-1	3.911e-1	-0.041	15.483	1.287e-1	7.85e-1	-0.606	14.529	2.716e-4	4.432e-4	-0.394	14.520	1.029e-1	2.298e-1	0.172	15.270	2.916e-1	5.374e-1	-0.566	14.877	1.074e-2	3.819e-2
	AAL94558.1| S-adenosylmethionine synthetase																							
**FN0356**	-0.716	10.040	4.226e-2	7.109e-2	-2.283	8.658			-1.801	8.752	2.524e-7	1.913e-8	-1.084	8.239	6.211e-2	1.098e-1	-1.566	7.942			0.482	6.858		
	AAL94559.1| Lactoylglutathione lyase																							
**FN0357**	-0.326	7.596	1.831e-1	4.972e-1	-0.449	7.657	4.661e-2	2.584e-1	-0.138	7.580	1.187e-1	6.818e-1	0.188	7.458	2.124e-1	6.466e-1	-0.124	7.331	3.745e-1	7.816e-1	0.311	7.519	4.074e-2	1.768e-1
	AAL94560.1| ATP synthase epsilon chain, sodium ion specific																							
**FN0358**	-0.929	17.355	1.835e-7	2.97e-9	-0.347	18.121	1.156e-2	5.518e-2	-0.992	17.088	2.477e-7	1.823e-8	-0.063	16.363	2.578e-2	3.046e-2	0.581	17.192	6.231e-2	3.35e-2	-0.644	17.129	8.106e-3	2.734e-2
	AAL94561.1| ATP synthase beta chain, sodium ion specific																							
**FN0359**	-0.634	11.962	2.378e-2	3.367e-2	-0.269	12.511	2.135e-3	7.37e-3	-0.584	11.809	1.868e-4	2.623e-4	0.050	11.378	2.594e-1	8.397e-1	0.365	11.877	1.394e-1	1.615e-1	-0.314	11.927	4.047e-3	1.132e-2
	AAL94562.1| ATP synthase gamma chain, sodium ion specific																							
**FN0360**	0.202	13.804	2.111e-3	1.401e-3	0.487	14.273	2.811e-3	1.034e-2	0.041	13.439	8.798e-2	4.868e-1	-0.161	13.845	2.234e-2	2.484e-2	0.285	14.476	7.053e-2	4.199e-2	-0.446	14.314	3.751e-3	1.022e-2
	AAL94563.1| ATP synthase alpha chain, sodium ion specific																							
**FN0361**	-0.808	10.031	2.108e-2	2.895e-2	-0.759	10.265	9.707e-3	4.517e-2	-0.550	10.085	2.28e-4	3.525e-4	0.258	9.481	1.512e-1	4.057e-1	0.049	9.456	4.111e-1	9.113e-1	0.209	9.714	1.088e-1	5.281e-1
	AAL94564.1| ATP synthase delta chain, sodium ion specific																							
**FN0362**	-0.554	10.462			-0.065	11.135	1.364e-1	8.393e-1	-0.536	10.276	3.621e-2	1.792e-1	0.017	9.926			0.489	10.582			-0.471	10.599	2.038e-2	8.06e-2
	AAL94565.1| ATP synthase B chain, sodium ion specific																							
**FN0364**	1.724	5.677							1.071	4.820			-0.653	6.748										
	AAL94567.1| ATP synthase A chain, sodium ion specific																							
**FN0366**	-0.510	15.695	5.096e-3	4.671e-3	-0.416	15.974	6.335e-3	2.724e-2	0.100	16.101	4.609e-2	2.361e-1	0.610	15.795	2.972e-3	1.209e-3	0.094	15.463	2.964e-1	5.5e-1	0.516	16.073	2.89e-3	7.328e-3
	AAL94569.1| Phosphoglucosamine mutase																							
**FN0368**	0.324	13.237	1.183e-1	2.803e-1	-0.530	12.567	2.842e-3	1.048e-2	0.078	12.788	7.982e-2	4.36e-1	-0.245	13.315	1.467e-1	3.897e-1	-0.854	12.891	8.103e-2	5.551e-2	0.609	12.646	2.347e-3	5.658e-3
	AAL94571.1| Adenylosuccinate lyase																							

Log_2 Ratios

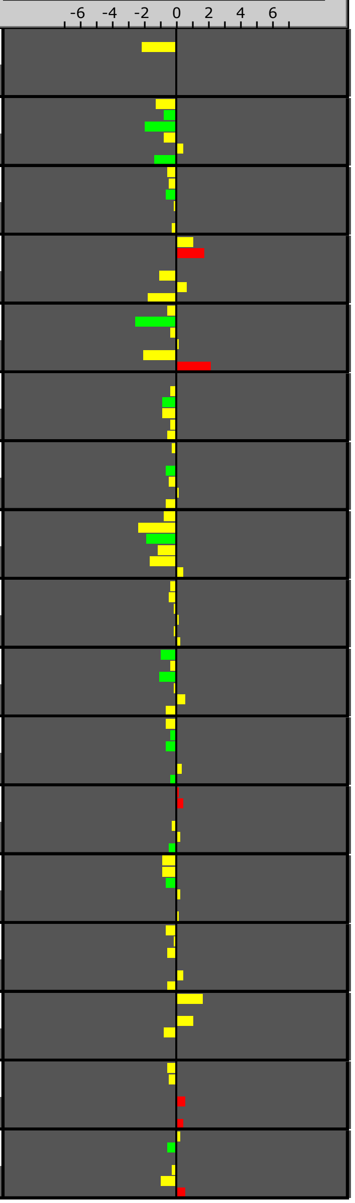

Show detected proteins only
Show all proteins
Filter by category:
GO: amino acid transport

Proteins found: 1424

Enter (or paste) list of ORFs

Find ORFs

Test	Cutoff
q-Value	.005
p-Value	

Dot Plots
Dot Plots

	Signif	Direction	Applies To
red	yes	+	ratios, bars
yellow	no	n/a	bars
green	yes	−	ratios, bars
pink	yes	+	*p*- , *q*-Values
light green	yes	−	*p*- , *q*-Values

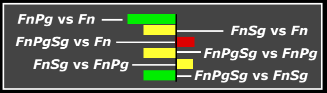

Fusobacterium nucleatum

Hackett Laboratory UW

ORF	Protein	FnPg vs Fn				FnSg vs Fn				FnPgSg vs Fn				FnPgSg vs FnPg				FnSg vs FnPg				FnPgSg vs FnSg			
		Ratio	Sum	*q*-Val	*p*-Val	Ratio	Sum	*q*-Val	*p*-Val	Ratio	Sum	*q*-Val	*p*-Val	Ratio	Sum	*q*-Val	*p*-Val	Ratio	Sum	*q*-Val	*p*-Val	Ratio	Sum	*q*-Val	*p*-Val
FN0370	AAL94573.1| Signal peptidase I	-0.675	10.059	6.105e-2	1.189e-1	-0.772	10.147	5.368e-3	2.244e-2	-1.004	9.526	2.752e-3	8.88e-3	-0.329	9.055	1.841e-1	5.298e-1	-0.097	9.472	3.919e-1	8.415e-1	-0.233	9.143	1.014e-1	4.882e-1
FN0371	AAL94574.1| Hypothetical protein	-1.635	11.082	9.831e-3	1.113e-2	-2.901	10.001			0.167	12.681	6.273e-2	3.331e-1	1.802	11.250	5.343e-3	2.773e-3	-1.266	8.366			3.068	10.169		
FN0374	AAL94577.1| Single-stranded-DNA-specific exonuclease recJ	1.120	5.425	2.593e-2	3.754e-2	2.077	6.567											0.957	7.687						
FN0375	AAL94578.1| Iron(III)-binding protein	0.509	17.587	7.141e-3	7.429e-3	1.101	18.363	1.719e-3	5.694e-3	0.529	17.403	1.105e-3	2.734e-3	0.020	18.116	2.581e-1	8.339e-1	0.592	18.873	3.983e-2	1.494e-2	-0.572	18.893	5.766e-3	1.801e-2
FN0376	AAL94579.1| Iron(III)-transport ATP-binding protein sfuC	0.466	12.667	1.783e-2	2.329e-2	1.150	13.536	3.241e-4	7.114e-4	-0.708	11.289	6.328e-5	5.649e-5	-1.174	11.959	4.776e-3	2.368e-3	0.684	14.002	6.587e-3	8.96e-4	-1.858	12.828	2.183e-4	2.474e-4
FN0377	AAL94580.1| Iron(III)-transport system permease protein sfuB					-1.332	5.976			1.124	8.044											2.456	7.100		
FN0378	AAL94581.1| UDP-glucose 4-epimerase					-0.339	6.134	7.115e-2	4.071e-1	0.286	6.371	8.949e-2	4.962e-1									0.625	6.421	6.098e-2	2.782e-1
FN0379	AAL94582.1| Hypothetical protein	-0.651	6.644	4.499e-2	7.757e-2	-1.155	6.325			-0.107	6.985	1.231e-1	7.105e-1	0.544	6.537	9.755e-2	2.107e-1	-0.504	5.674			1.048	6.218		
FN0380	AAL94583.1| unknown	-2.167	7.337							-1.707	7.592			0.459	5.629										
FN0381	AAL94584.1| unknown									0.286	8.570	4.251e-3	1.502e-2												
FN0382	AAL94585.1| Hypothetical protein																								
FN0383	AAL94586.1| Lipopolysaccharide N-acetylglucosaminyltransferase																								
FN0384	AAL94587.1| Hypothetical protein	-3.389	7.467			-3.317	7.723	2.241e-4	4.398e-4	-1.709	8.942	4.412e-4	8.5e-4	1.679	5.758			0.071	4.334			1.608	6.013	2.007e-5	9.367e-6
FN0385	AAL94588.1| Hypothetical protein																								
FN0386	AAL94589.1| Hypothetical protein																								
FN0387	AAL94590.1| Fusobacterium outer membrane protein family	-2.777	11.772	7.82e-5	9.377e-6	-2.598	12.135	2.352e-4	4.672e-4	-1.823	12.522	3.938e-5	2.918e-5	0.954	9.949	9.124e-4	2.082e-4	0.178	9.358	3.829e-1	8.102e-1	0.776	10.312	4.616e-2	2.036e-1
FN0390	AAL94593.1| Hypothetical protein	1.070	11.829	5.147e-2	9.318e-2	0.760	11.704	2.097e-3	7.21e-3	1.213	11.769	2.133e-3	6.538e-3	0.143	13.042	2.207e-1	6.789e-1	-0.310	12.774	2.522e-1	4.404e-1	0.453	12.917	1.121e-2	4.025e-2

Log_2 Ratios: -6 -4 -2 0 2 4 6

Show detected proteins only
Show all proteins
Filter by category:
GO: amino acid transport

Proteins found: 1424

Enter (or paste) list of ORFs

Find ORFs

Test	Cutoff
q-Value	.005
p-Value	

Dot Plots
Dot Plots

	Signif	Direction	Applies To
red	yes	+	ratios, bars
yellow	no	n/a	bars
green	yes	–	ratios, bars
light red	yes	+	*p*- , *q*-Values
light green	yes	–	*p*- , *q*-Values

FnPg vs Fn
FnSg vs Fn
FnPgSg vs Fn
FnPgSg vs FnPg
FnSg vs FnPg
FnPgSg vs FnSg

ORF	Description	*FnPg vs Fn*				*FnSg vs Fn*				*FnPgSg vs Fn*				*FnPgSg vs FnPg*				*FnSg vs FnPg*				*FnPgSg vs FnSg*			
		Ratio	Sum	*q*-Val	*p*-Val	Ratio	Sum	*q*-Val	*p*-Val	Ratio	Sum	*q*-Val	*p*-Val	Ratio	Sum	*q*-Val	*p*-Val	Ratio	Sum	*q*-Val	*p*-Val	Ratio	Sum	*q*-Val	*p*-Val
FN0391	AAL94594.1| Hydrolase (HAD superfamily)	0.598	9.079	1.758e-1	4.7e-1	0.573	9.238	6.047e-5	7.95e-5	-0.312	7.965	2.99e-3	9.845e-3	-0.910	8.767	1.328e-1	3.384e-1	-0.025	9.836	4.26e-1	9.689e-1	-0.885	8.926	3.102e-4	4.034e-4
FN0392	AAL94595.1| Oxygen-independent coproporphyrinogen III oxidase	-1.819	6.462			-1.839	6.626	3.636e-5	3.78e-5	-1.162	6.915	6.901e-6	1.857e-6	0.656	5.300			-0.021	4.808			0.677	5.464	5.732e-3	1.788e-2
FN0393	AAL94596.1| Polysaccharide deacetylase	0.932	7.413	1.252e-2	1.484e-2	1.616	8.281	3.461e-3	1.344e-2	-0.799	5.478	6.439e-5	5.787e-5	-1.731	6.615	7.869e-3	5.162e-3	0.683	9.213	7.93e-2	5.305e-2	-2.414	7.482	3.101e-3	8.002e-3
FN0394	AAL94597.1| Outer membrane protein	0.531	7.684	1.818e-1	4.925e-1	-0.481	6.856			-0.889	6.059			-1.421	6.795			-1.012	7.388			-0.409	5.967		
FN0396	AAL94599.1| Dipeptide-binding protein	0.402	24.004	5.688e-4	1.869e-4	0.640	24.426	9.475e-3	4.386e-2	0.388	23.786	1.779e-4	2.444e-4	-0.014	24.392	2.315e-1	7.221e-1	0.238	24.828	1.754e-1	2.514e-1	-0.252	24.814	5.178e-2	2.319e-1
FN0397	AAL94600.1| Dipeptide transport system permease protein dppB	-0.052	10.905			-0.015	11.126	1.467e-1	9.138e-1	0.292	11.044	9.168e-3	3.727e-2	0.344	11.196			0.037	11.074			0.307	11.418	7.009e-3	2.287e-2
FN0398	AAL94601.1| Dipeptide transport system permease protein dppC	-1.058	11.387	8.884e-4	3.919e-4	-0.502	12.128	1.843e-3	6.179e-3	-0.506	11.735	2.89e-3	9.437e-3	0.552	10.881	6.125e-3	3.399e-3	0.556	11.070	3.375e-2	1.123e-2	-0.005	11.622	1.823e-1	9.645e-1
FN0399	AAL94602.1| Dipeptide transport ATP-binding protein dppD	-0.543	15.278	1.413e-2	1.723e-2	-0.236	15.770	3.738e-2	2.036e-1	-0.367	15.250	9.994e-3	4.12e-2	0.176	14.911	1.494e-1	3.993e-1	0.307	15.227	1.549e-1	2.022e-1	-0.132	15.402	1.068e-1	5.174e-1
FN0400	AAL94603.1| Dipeptide transport ATP-binding protein dppF	-0.684	17.806	3.158e-3	2.414e-3	-0.487	18.187	6.672e-4	1.787e-3	-1.174	17.112	1.348e-4	1.662e-4	-0.490	16.632	1.36e-2	1.205e-2	0.197	17.503	1.169e-1	1.136e-1	-0.687	17.013	2.663e-4	3.311e-4
FN0405	AAL94608.1| Tryptophanyl-tRNA synthetase	-0.284	12.911	1.443e-1	3.61e-1	-0.293	13.086	7.528e-3	3.343e-2	0.742	13.733	4.952e-3	1.804e-2	1.026	13.653	1.135e-2	9.178e-3	-0.009	12.802	4.281e-1	9.774e-1	1.035	13.828	3.145e-3	8.145e-3
FN0406	AAL94609.1| Alanine racemase, biosynthetic	-1.164	11.391	1.616e-7	2.24e-9	-1.516	11.223	5.528e-4	1.399e-3	-0.395	11.956	2.87e-3	9.355e-3	0.769	10.997	6.987e-3	4.197e-3	-0.352	10.060	1.482e-1	1.851e-1	1.121	10.829	8.129e-4	1.473e-3
FN0407	AAL94610.1| Hypothetical protein	-2.102	10.887			-1.735	11.438	4.116e-5	4.583e-5	0.167	12.951	3.356e-2	1.642e-1	2.268	11.053			0.367	9.336			1.902	11.604	3.189e-4	4.188e-4
FN0408	AAL94611.1| Acetyl-coenzyme A carboxylase carboxyl transferase subunit beta	-0.747	12.388	5.938e-2	1.142e-1	-0.129	13.191	4.838e-2	2.692e-1	-0.372	12.560	6.445e-3	2.47e-2	0.375	12.016	1.543e-1	4.166e-1	0.618	12.444	1.478e-1	1.84e-1	-0.243	12.819	1.574e-2	5.965e-2
FN0409	AAL94612.1| Acetyl-coenzyme A carboxylase carboxyl transferase subunit alpha	-1.080	17.074	1.773e-3	1.08e-3	-0.987	17.352	2.609e-6	6.883e-7	0.047	17.998	1.012e-1	5.699e-1	1.127	17.122	9.283e-4	2.139e-4	0.093	16.272	2.99e-1	5.569e-1	1.034	17.399	9.59e-4	1.833e-3
FN0410	AAL94613.1| 6-phosphofructokinase	-0.698	13.649	2.14e-4	4.225e-5	-0.464	14.068	6.416e-4	1.696e-3	-0.330	13.814	1.17e-5	3.94e-6	0.368	13.319	1.163e-3	3.104e-4	0.234	13.369	4.817e-2	2.113e-2	0.134	13.738	2.035e-2	8.044e-2
FN0411	AAL94614.1| putative alpha helix protein					0.597	7.565																		
FN0412	AAL94615.1| Recombination protein recR																								

Log$_2$ Ratios

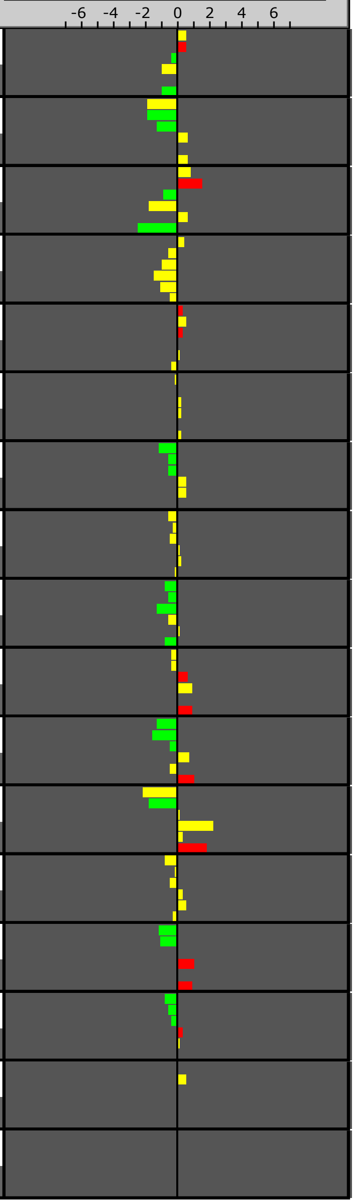

Show detected proteins only
Show all proteins
Filter by category:
GO: amino acid transport

Proteins found: 1424

Enter (or paste) list of ORFs

Find ORFs

Test	Cutoff
q-Value	.005
p-Value	

Dot Plots
Dot Plots

	Signif	Direction	Applies To
red	yes	+	ratios, bars
yellow	no	n/a	bars
green	yes	–	ratios, bars
pink	yes	+	*p*-, *q*-Values
light green	yes	–	*p*-, *q*-Values

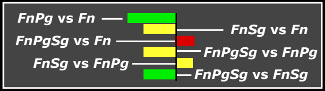

ORF	Description	FnPg vs Fn Ratio	FnPg vs Fn Sum	FnPg vs Fn q-Val	FnPg vs Fn p-Val	FnSg vs Fn Ratio	FnSg vs Fn Sum	FnSg vs Fn q-Val	FnSg vs Fn p-Val	FnPgSg vs Fn Ratio	FnPgSg vs Fn Sum	FnPgSg vs Fn q-Val	FnPgSg vs Fn p-Val	FnPgSg vs FnPg Ratio	FnPgSg vs FnPg Sum	FnPgSg vs FnPg q-Val	FnPgSg vs FnPg p-Val	FnSg vs FnPg Ratio	FnSg vs FnPg Sum	FnSg vs FnPg q-Val	FnSg vs FnPg p-Val	FnPgSg vs FnSg Ratio	FnPgSg vs FnSg Sum	FnPgSg vs FnSg q-Val	FnPgSg vs FnSg p-Val
FN0413	AAL94616.1| unknown	0.938	4.669							0.236	3.764			-0.701	4.905										
FN0414	AAL94617.1| ATP-dependent helicase HEPA																					0.779	4.779		
FN0416	AAL94619.1| Type III restriction-modification system methylation subunit	-1.077	8.326							-1.240	7.959	1.825e-3	5.409e-3	-0.163	7.086										
FN0417	AAL94620.1| Type III restriction-modification system restriction subunit	-1.788	9.867			-2.598	9.242			-2.222	9.230	1.308e-4	1.591e-4	-0.434	7.645			-0.810	7.453			0.376	7.020		
FN0418	AAL94621.1| Uracil phosphoribosyltransferase													-3.964	8.168			0.413	12.730			-4.377	8.766		
FN0419	AAL94622.1| Aspartate carbamoyltransferase	0.272	7.477			-0.540	6.849			-0.696	6.305			-0.968	6.780	1.024e-2	7.839e-3	-0.812	7.121	3.494e-2	1.199e-2	-0.156	6.153	1.149e-1	5.614e-1
FN0420	AAL94623.1| Dihydroorotase	1.054	8.770	1.263e-1	3.048e-1	1.325	9.226	7.44e-3	3.298e-2	-2.171	5.341			-3.225	6.599			0.271	10.280	3.414e-1	6.767e-1	-3.496	7.055		
FN0421	AAL94624.1| Carbamoyl-phosphate synthase small chain	0.774	9.870			0.333	9.614	2.941e-3	1.095e-2	-1.542	7.350	3.29e-5	2.008e-5	-2.316	8.328			-0.441	10.388			-1.876	8.072	3.952e-5	2.469e-5
FN0422	AAL94625.1| Carbamoyl-phosphate synthase large chain	-0.715	13.950	9.791e-2	2.211e-1	0.151	15.001	2.346e-2	1.22e-1	-1.107	13.354	2.957e-4	4.957e-4	-0.392	12.843	2.026e-1	6.076e-1	0.866	14.286	1.306e-1	1.411e-1	-1.258	13.894	5.972e-4	9.625e-4
FN0423	AAL94626.1| Dihydroorotate dehydrogenase electron transfer subunit	0.481	7.519			1.262	8.485	7.005e-4	1.908e-3	-0.906	5.929	1.149e-2	4.821e-2	-1.386	6.614			0.781	8.966			-2.168	7.580	3.677e-7	3.48e-8
FN0424	AAL94627.1| Dihydroorotate dehydrogenase	0.993	5.667	1.049e-1	2.411e-1	1.988	6.847	1.193e-3	3.68e-3	-0.650	3.820			-1.643	5.017			0.995	7.839	8.033e-2	5.452e-2	-2.638	6.196		
FN0426	AAL94629.1| Orotidine 5'-phosphate decarboxylase	1.087	9.071	1.221e-1	2.916e-1	1.669	9.837	1.307e-4	2.226e-4	0.490	8.271	2.317e-2	1.066e-1	-0.596	9.561	1.705e-1	4.762e-1	0.582	10.924	2.025e-1	3.186e-1	-1.179	10.328	4.122e-4	5.785e-4
FN0427	AAL94630.1| Orotate phosphoribosyltransferase	-0.092	11.470	1.324e-1	3.233e-1	-0.086	11.660	7.203e-2	4.125e-1	-0.336	11.021	1.975e-3	5.97e-3	-0.244	11.133	4.001e-2	5.756e-2	0.006	11.569	4.234e-1	9.585e-1	-0.251	11.324	1.969e-2	7.742e-2
FN0430	AAL94633.1| LSU ribosomal protein L19P	-1.332	14.971	4.591e-3	4.041e-3	-0.701	15.787	1.786e-4	3.339e-4	-0.602	15.497	3.365e-4	5.911e-4	0.730	14.369	3.995e-2	5.745e-2	0.631	14.454	9.84e-2	8.267e-2	0.099	15.184	7.447e-2	3.479e-1
FN0435	AAL94634.1| Purine nucleoside phosphorylase	0.230	11.661	2.416e-1	7.102e-1	0.052	11.668	1.009e-1	5.961e-1	-0.657	10.570	3.348e-2	1.637e-1	-0.888	11.004	1.248e-1	3.081e-1	-0.178	11.898	3.704e-1	7.682e-1	-0.710	11.011	3.245e-2	1.362e-1
FN0436	AAL94635.1| regulator of kinase autophosphorylation inhibitor	1.228	11.009	3.199e-2	4.913e-2	1.333	11.299	1.074e-2	5.071e-2	-0.470	9.107	3.133e-2	1.512e-1	-1.698	10.539	2.224e-2	2.468e-2	0.105	12.527	3.77e-1	7.9e-1	-1.804	10.829	8.356e-3	2.832e-2
FN0437	AAL94636.1| kinase autophosphorylation inhibitor KipI	0.279	7.134	2.359e-1	6.883e-1	0.806	7.847	7.057e-4	1.927e-3	-0.598	6.054	4.03e-2	2.03e-1	-0.877	6.536	1.355e-1	3.493e-1	0.528	8.125	2.155e-1	3.488e-1	-1.404	7.249	2.962e-3	7.557e-3

Log$_2$ Ratios

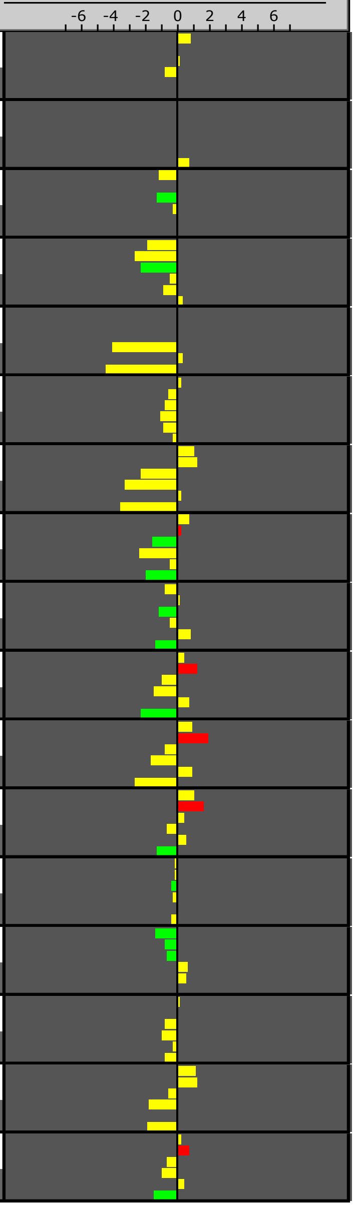

Show detected proteins only
Show all proteins
Filter by category:
GO: amino acid transport

Proteins found: 1424

Enter (or paste) list of ORFs
Find ORFs

Test	Cutoff
q-Value	.005
p-Value	

Dot Plots
Dot Plots

	Signif	Direction	Applies To
red	yes	+	ratios, bars
yellow	no	n/a	bars
green	yes	–	ratios, bars
pink	yes	+	p- , q-Values
light green	yes	–	p- , q-Values

FnPg vs Fn
FnSg vs Fn
FnPgSg vs Fn
FnPgSg vs FnPg
FnSg vs FnPg
FnPgSg vs FnSg

ORF	Protein	*FnPg vs Fn*				*FnSg vs Fn*				*FnPgSg vs Fn*				*FnPgSg vs FnPg*				*FnSg vs FnPg*				*FnPgSg vs FnSg*			
		Ratio	Sum	*q*-Val	*p*-Val	Ratio	Sum	*q*-Val	*p*-Val	Ratio	Sum	*q*-Val	*p*-Val	Ratio	Sum	*q*-Val	*p*-Val	Ratio	Sum	*q*-Val	*p*-Val	Ratio	Sum	*q*-Val	*p*-Val
**FN0438**	AAL94637.1| transporter protein																								
**FN0439**	AAL94638.1| Lactam utilization protein LAMB	0.336	12.037	2.36e-1	6.889e-1	-0.040	11.845	1.436e-1	8.912e-1	-0.011	11.486	1.562e-1	9.369e-1	-0.348	12.026	2.208e-1	6.794e-1	-0.376	12.182	3.377e-1	6.657e-1	0.029	11.834	1.751e-1	9.185e-1
**FN0445**	AAL94641.1| Hypothetical protein					-0.008	5.178			0.077	4.875	7.806e-2	4.251e-1									0.085	5.255		
**FN0446**	AAL94642.1| Hypothetical protein	-1.008	7.281	6.665e-5	7.553e-6	0.027	8.501	1.438e-1	8.924e-1	-0.567	7.518	1.677e-4	2.247e-4	0.441	6.714	7.506e-3	4.744e-3	1.035	7.493	5.234e-2	2.483e-2	-0.594	7.934	1.861e-2	7.25e-2
**FN0447**	AAL94643.1| NIFS protein	0.262	4.908			1.685	6.516	9.326e-4	2.737e-3	-0.221	4.221			-0.483	4.687			1.424	6.778			-1.907	6.295		
**FN0448**	AAL94644.1| DNA-repair protein																								
**FN0450**	AAL94646.1| ABC transporter ATP-binding protein	-1.177	9.532	4.1e-2	6.82e-2	-0.482	10.411	4.827e-2	2.685e-1	-0.980	9.525	1.959e-2	8.824e-2	0.197	8.552	9.774e-2	2.114e-1	0.695	9.234	9.329e-2	7.519e-2	-0.497	9.431	3.205e-2	1.343e-1
**FN0451**	AAL94647.1| Hypothetical protein	-0.516	6.131			-0.319	6.512	3.795e-2	2.069e-1	-0.864	5.579			-0.348	5.267			0.197	5.996			-0.545	5.649		
**FN0452**	AAL94648.1| Glucosamine--fructose-6-phosphate aminotransferase (isomerizing)	-0.429	18.476	1.721e-1	4.565e-1	-1.837	17.252	5.216e-6	2.031e-6	0.015	18.716	9.886e-2	5.552e-1	0.444	18.490	1.609e-1	4.405e-1	-1.408	16.823	1.636e-1	2.228e-1	1.852	17.267	8.434e-5	6.921e-5
**FN0453**	AAL94649.1| Xaa-Pro aminopeptidase	0.787	16.502	9.092e-3	1.012e-2	0.296	16.195	2.768e-3	1.014e-2	-0.220	15.291	4.987e-3	1.819e-2	-1.007	16.282	7.377e-3	4.603e-3	-0.491	16.982	5.095e-2	2.353e-2	-0.516	15.975	4.527e-4	6.539e-4
**FN0454**	AAL94650.1| Aldehyde dehydrogenase B	0.855	13.882	7.932e-2	1.68e-1	1.417	14.629	1.243e-3	3.873e-3	-0.507	12.316	4.712e-5	3.749e-5	-1.363	13.375	5.399e-2	8.967e-2	0.562	15.484	1.351e-1	1.513e-1	-1.924	14.121	1.148e-3	2.325e-3
**FN0455**	AAL94651.1| Rubrerythrin	1.074	16.135	4.143e-3	3.49e-3	1.718	16.964	4.709e-4	1.134e-3	1.180	16.037	2.472e-3	7.79e-3	0.106	17.315	1.844e-1	5.307e-1	0.644	18.038	1.388e-2	2.762e-3	-0.538	18.144	3.535e-3	9.458e-3
**FN0456**	AAL94652.1| Hypothetical cytosolic protein	-2.394	10.091	2.686e-4	6.088e-5	-2.542	10.127	1.164e-5	7.519e-6	-1.123	11.157	2.866e-5	1.568e-5	1.270	8.967	1.495e-3	4.502e-4	-0.148	7.733	3.022e-1	5.655e-1	1.418	9.004	1.978e-5	9.115e-6
**FN0458**	AAL94654.1| Hypothetical Exported Protein	1.533	5.806	2.136e-2	2.941e-2	0.808	5.266	1.363e-2	6.682e-2	0.645	4.714			-0.888	6.451			-0.725	6.798	1.092e-1	1.002e-1	-0.163	5.910		
**FN0459**	AAL94655.1| Hypothetical exported 24-amino acid repeat protein																								
**FN0460**	AAL94656.1| Delta-aminolevulinic acid dehydratase	-0.196	11.810			-2.267	9.924	2.218e-5	1.897e-5	-1.497	10.305	6.626e-5	6.025e-5	-1.301	10.313			-2.071	9.728			0.770	8.427	5.348e-3	1.637e-2
**FN0461**	AAL94657.1| Probable sigma(54) modulation protein	0.389	16.697	1.504e-1	3.809e-1	-0.447	16.045	3.707e-5	3.893e-5	-0.409	15.695	3.45e-4	6.121e-4	-0.798	16.288	8.224e-2	1.623e-1	-0.836	16.434	1.356e-1	1.524e-1	0.038	15.636	1.155e-1	5.651e-1

Log$_2$ Ratios

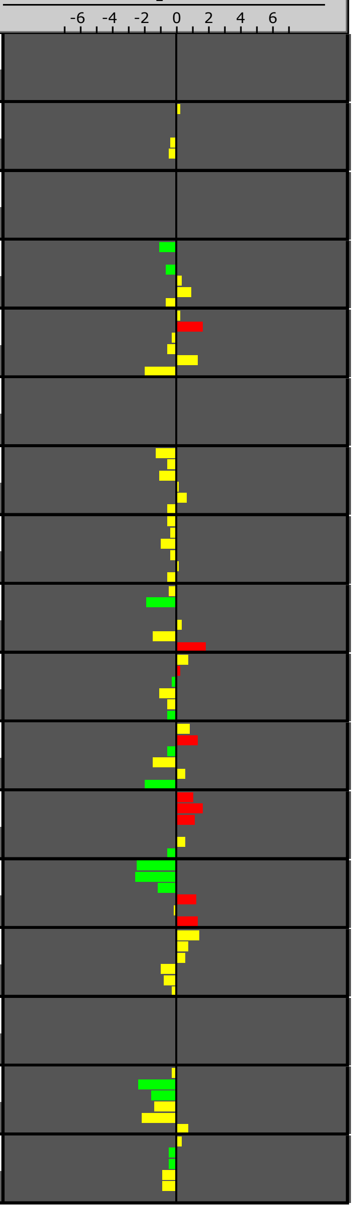

Show detected proteins only
Show all proteins
Filter by category:
GO: amino acid transport

Proteins found: 1424

Enter (or paste) list of ORFs

Find ORFs

Test	Cutoff
q-Value	.005
p-Value	

Dot Plots
Dot Plots

	Signif	Direction	Applies To
red	yes	+	ratios, bars
yellow	no	n/a	bars
green	yes	–	ratios, bars
pink	yes	+	*p*- , *q*-Values
light green	yes	–	*p*- , *q*-Values

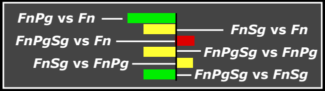

		FnPg vs *Fn*				*FnSg* vs *Fn*				*FnPgSg* vs *Fn*				*FnPgSg* vs *FnPg*				*FnSg* vs *FnPg*				*FnPgSg* vs *FnSg*			
ORF	Protein	Ratio	Sum	*q*-Val	*p*-Val	Ratio	Sum	*q*-Val	*p*-Val	Ratio	Sum	*q*-Val	*p*-Val	Ratio	Sum	*q*-Val	*p*-Val	Ratio	Sum	*q*-Val	*p*-Val	Ratio	Sum	*q*-Val	*p*-Val
**FN0462**	AAL94658.1| DNA mismatch repair protein mutL	0.045	11.170	2.852e-1	8.896e-1	1.070	12.379	5.154e-3	2.142e-2	-1.400	9.521	1.37e-6	2.122e-7	-1.445	9.770	3.763e-2	5.285e-2	1.025	12.425	4.38e-2	1.773e-2	-2.470	10.980	2.488e-3	6.106e-3
**FN0465**	AAL94661.1| Hypothetical protein	0.270	16.294	2.351e-1	6.853e-1	-0.612	15.596	3.061e-3	1.151e-2	-1.256	14.564	4.782e-4	9.431e-4	-1.526	15.038	8.973e-2	1.849e-1	-0.882	15.866	2.019e-1	3.172e-1	-0.644	14.340	1.392e-2	5.177e-2
**FN0466**	AAL94662.1| Lysyl-tRNA synthetase	0.383	17.561	5.203e-2	9.458e-2	0.347	17.710	9.443e-3	4.369e-2	-0.060	16.914	1.128e-1	6.434e-1	-0.443	17.501	4.501e-2	6.802e-2	-0.036	18.093	3.916e-1	8.402e-1	-0.408	17.650	9.611e-3	3.34e-2
**FN0469**	AAL94665.1| Copper homeostasis protein cutC																								
**FN0470**	AAL94666.1| Putative efflux pump component MtrF	-0.034	18.282	2.706e-1	8.272e-1	-0.411	18.091	3.507e-3	1.366e-2	-0.264	17.849	9.081e-3	3.685e-2	-0.229	18.019	9.585e-2	2.049e-1	-0.376	18.056	9.883e-2	8.33e-2	0.147	17.827	4.969e-2	2.213e-1
**FN0472**	AAL94668.1| Flavodoxin	1.659	24.440	5.982e-3	5.795e-3	1.030	23.996	1.242e-7	7.722e-9	1.747	24.324	1.612e-8	2.726e-10	0.089	26.187	1.916e-1	5.605e-1	-0.628	25.655	6.442e-2	3.553e-2	0.717	25.743	1.814e-7	9.116e-9
**FN0474**	AAL94670.1| Acriflavin resistance protein B					-0.688	5.449																		
**FN0475**	AAL94671.1| MIAB protein	-1.548	10.467			-1.090	11.110	3.04e-4	6.535e-4	-2.051	9.760	1.464e-4	1.867e-4	-0.503	8.416			0.458	9.562			-0.961	9.059	1.569e-3	3.417e-3
**FN0476**	AAL94672.1| Transcription termination factor rho	-0.244	11.690	1.564e-1	4.011e-1	0.309	12.427	8.648e-3	3.932e-2	0.349	12.079	2.206e-3	6.801e-3	0.593	12.038	4.505e-2	6.809e-2	0.553	12.183	9.89e-2	8.341e-2	0.040	12.776	1.414e-1	7.123e-1
**FN0477**	AAL94673.1| Cell wall endopeptidase family M23/M37	-2.372	10.157	4.722e-6	2.484e-7	-1.264	11.449	3.036e-6	9.079e-7	-2.734	9.591	5.255e-6	1.223e-6	-0.362	7.423	1.064e-2	8.346e-3	1.108	9.077	4.597e-3	5.034e-4	-1.470	8.715	3.416e-4	4.569e-4
**FN0478**	AAL94674.1| GcpE protein	1.361	8.814			1.215	8.853	5.409e-8	1.765e-9	-0.326	6.924	2.915e-2	1.388e-1	-1.686	8.489			-0.145	10.214			-1.541	8.528	4.136e-4	5.812e-4
**FN0479**	AAL94675.1| RNA polymerase sigma-E factor																								
**FN0480**	AAL94676.1| unknown																								
**FN0481**	AAL94677.1| unknown	-0.697	7.616			-1.008	7.489	1.335e-2	6.525e-2									-0.311	6.792						
**FN0482**	AAL94678.1| LSU ribosomal protein L31P	-2.778	7.948							0.455	10.977	2.631e-2	1.232e-1	3.233	8.403										
**FN0483**	AAL94679.1| Uracil phosphoribosyltransferase	-1.016	13.224	8.106e-3	8.797e-3	-1.286	13.140	8.803e-6	4.795e-6	-0.298	13.739	1.011e-3	2.428e-3	0.718	12.927	2.902e-2	3.626e-2	-0.269	12.123	2.504e-1	4.361e-1	0.988	12.842	4.783e-5	3.29e-5
**FN0484**	AAL94680.1| Lipase	-0.959	5.603			-0.185	6.561			-1.179	5.179			-0.220	4.424			0.773	5.602			-0.994	5.382		

Log_2 Ratios (-6, -4, -2, 0, 2, 4, 6)

- Show detected proteins only
- Show all proteins
- Filter by category: GO: amino acid transport

Proteins found: 1424

Enter (or paste) list of ORFs

Find ORFs

Test	Cutoff
q-Value	.005
p-Value	

Dot Plots

Dot Plots

	Signif	Direction	Applies To
red	yes	+	ratios, bars
yellow	no	n/a	bars
green	yes	–	ratios, bars
pink	yes	+	*p*- , *q*-Values
light green	yes	–	*p*- , *q*-Values

FnPg vs Fn — FnSg vs Fn
FnPgSg vs Fn — FnPgSg vs FnPg
FnSg vs FnPg — FnPgSg vs FnSg

ORF	Description	*FnPg vs Fn*				*FnSg vs Fn*				*FnPgSg vs Fn*				*FnPgSg vs FnPg*				*FnSg vs FnPg*				*FnPgSg vs FnSg*			
		Ratio	Sum	*q*-Val	*p*-Val	Ratio	Sum	*q*-Val	*p*-Val	Ratio	Sum	*q*-Val	*p*-Val	Ratio	Sum	*q*-Val	*p*-Val	Ratio	Sum	*q*-Val	*p*-Val	Ratio	Sum	*q*-Val	*p*-Val
FN0487	AAL94683.1| 2-hydroxyglutarate dehydrogenase	-0.121	20.283	2.279e-1	6.579e-1	-0.012	20.576	1.472e-1	9.17e-1	-0.002	20.199	1.601e-1	9.651e-1	0.119	20.282	2.164e-1	6.62e-1	0.108	20.456	3.514e-1	7.074e-1	0.010	20.575	1.767e-1	9.282e-1
FN0488	AAL94684.1| NAD-specific glutamate dehydrogenase	0.305	23.556	1.465e-1	3.683e-1	0.468	23.904	5.984e-3	2.547e-2	0.232	23.279	6.43e-3	2.463e-2	-0.073	23.789	2.513e-1	8.049e-1	0.163	24.209	3.054e-1	5.741e-1	-0.236	24.136	2.745e-2	1.127e-1
FN0489	AAL94685.1| Prolipoprotein diacylglyceryl transferase	-1.186	8.105			-1.567	7.907			0.489	9.576	9.597e-3	3.936e-2	1.675	8.594			-0.382	6.722			2.057	8.397		
FN0490	AAL94686.1| Integral membrane protein																								
FN0491	AAL94687.1| Alanine racemase	1.570	13.188	5.768e-2	1.094e-1	1.725	13.527	1.111e-3	3.377e-3	-0.523	10.891	3.977e-2	1.999e-1	-2.093	12.665	5.09e-2	8.176e-2	0.155	15.097	3.622e-1	7.414e-1	-2.248	13.004	2.198e-3	5.202e-3
FN0493	AAL94689.1| Hypothetical protein	-0.264	8.762	1.677e-1	4.405e-1	-0.771	8.440	8.499e-3	3.853e-2	-2.267	6.556	9.329e-4	2.185e-3	-2.003	6.496	3.013e-2	3.827e-2	-0.507	8.176	1.956e-1	3.031e-1	-1.495	6.173	1.472e-2	5.518e-2
FN0494	AAL94690.1| Short chain dehydrogenase	-1.417	17.059	1.5e-4	2.529e-5	-2.331	16.329	9.05e-6	5.001e-6	-0.350	17.923	1.919e-3	5.765e-3	1.067	16.710	9.095e-4	2.072e-4	-0.915	14.912	3.853e-2	1.411e-2	1.982	15.979	3.216e-5	1.793e-5
FN0495	AAL94691.1| Acetyl-CoA acetyltransferase	1.116	25.792	5.159e-5	5.034e-6	0.783	25.644	2.586e-6	6.78e-7	0.423	24.896	3.67e-5	2.497e-5	-0.693	26.215	5.156e-5	2.831e-6	-0.333	26.760	6.737e-4	1.257e-5	-0.360	26.067	8.608e-6	2.227e-6
FN0496	AAL94692.1| unknown																								
FN0497	AAL94693.1| Plasmid addiction system poison protein																								
FN0501	AAL94697.1| Ornithine decarboxylase	0.635	16.730	9.468e-2	2.115e-1	1.084	17.363	2.303e-4	4.551e-4	-0.572	15.318	1.268e-3	3.314e-3	-1.208	16.157	5.254e-2	8.588e-2	0.449	17.998	1.558e-1	2.044e-1	-1.656	16.791	3.928e-4	5.439e-4
FN0502	AAL94698.1| Phosphoheptose isomerase	1.032	7.887			1.003	8.044			1.159	7.811	1.78e-5	7.62e-6	0.127	9.046			-0.028	9.075			0.155	9.203		
FN0503	AAL94699.1| Transcriptional regulatory protein, LYSR family	-0.592	11.037	4.431e-2	7.591e-2	-0.224	11.590	8.655e-3	3.936e-2	-0.137	11.289	2.524e-2	1.175e-1	0.456	10.900	7.943e-2	1.543e-1	0.369	10.998	1.683e-1	2.34e-1	0.087	11.453	4.988e-4	7.451e-4
FN0504	AAL94700.1| Arginine permease																								
FN0505	AAL94701.1| Anthranilate synthase component II	0.947	11.277	7.569e-2	1.579e-1	-0.293	10.222	3.952e-2	2.162e-1	-0.498	9.628	3.89e-4	7.23e-4	-1.445	10.779	5.441e-2	9.08e-2	-1.240	11.169	1.138e-1	1.081e-1	-0.205	9.723	8.974e-2	4.265e-1
FN0506	AAL94702.1| Arginyl-tRNA synthetase	-0.638	14.994	2.51e-2	3.606e-2	-1.284	14.532	3.941e-5	4.277e-5	0.209	15.637	4.281e-2	2.173e-1	0.847	15.202	1.271e-2	1.085e-2	-0.647	13.894	1.081e-1	9.817e-2	1.493	14.740	1.575e-3	3.434e-3
FN0511	AAL94707.1| D-lactate dehydrogenase	0.404	11.177	2.162e-1	6.149e-1	0.593	11.550	2.722e-3	9.935e-3	-0.866	9.704	2.724e-3	8.769e-3	-1.270	10.311	1.143e-1	2.706e-1	0.189	11.954	3.714e-1	7.714e-1	-1.458	10.684	1.359e-3	2.857e-3

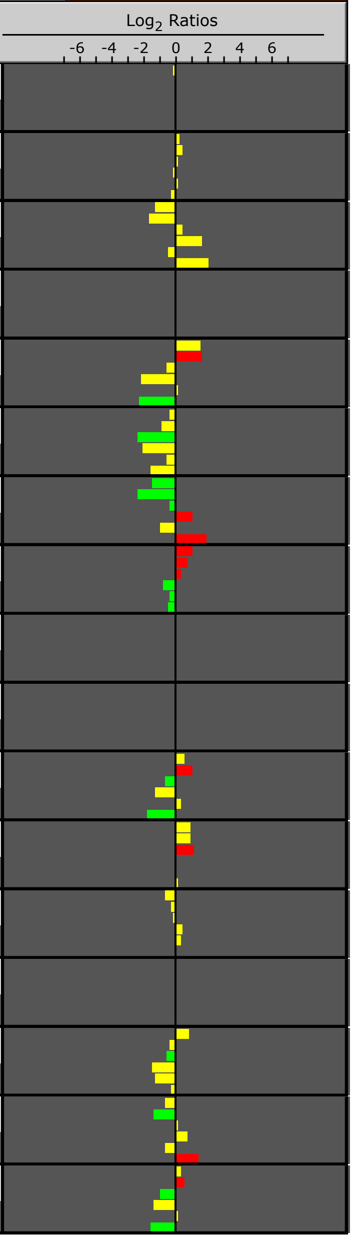

Show detected proteins only
Show all proteins
Filter by category:
GO: amino acid transport

Proteins found: 1424

Enter (or paste) list of ORFs

Find ORFs

Test	Cutoff
q-Value	.005
p-Value	

Dot Plots
Dot Plots

	Signif	Direction	Applies To
(red)	yes	+	ratios, bars
(yellow)	no	n/a	bars
(green)	yes	–	ratios, bars
(pink)	yes	+	*p*- , *q*-Values
(light green)	yes	–	

FnPg vs Fn
FnPgSg vs Fn
FnSg vs FnPg
FnSg vs Fn
FnPgSg vs FnPg
FnPgSg vs FnSg

ORF	Description	*FnPg vs Fn* Ratio	Sum	*q*-Val	*p*-Val	*FnSg vs Fn* Ratio	Sum	*q*-Val	*p*-Val	*FnPgSg vs Fn* Ratio	Sum	*q*-Val	*p*-Val	*FnPgSg vs FnPg* Ratio	Sum	*q*-Val	*p*-Val	*FnSg vs FnPg* Ratio	Sum	*q*-Val	*p*-Val	*FnPgSg vs FnSg* Ratio	Sum	*q*-Val	*p*-Val
FN0512	AAL94708.1| Flavoprotein	0.714	15.779	4.243e-4	1.178e-4	0.226	15.475	1.275e-3	3.995e-3	0.175	15.036	4.996e-3	1.823e-2	-0.540	15.954	2.949e-7	1.311e-9	-0.489	16.189	2.394e-3	1.65e-4	-0.051	15.650	4.334e-2	1.896e-1
FN0513	AAL94709.1| Flavodoxin	-1.712	8.768	1.029e-3	4.909e-4	-1.158	9.508	5.895e-4	1.519e-3	0.391	10.668	3.591e-5	2.388e-5	2.104	9.160	5.135e-4	9.2e-5	0.555	7.795	1.212e-1	1.219e-1	1.549	9.899	2.193e-4	2.491e-4
FN0515	AAL94711.1| Acriflavin resistance protein D	-0.928	4.098			0.321	5.532	6.431e-2	3.652e-1									1.249	4.604						
FN0516	AAL94712.1| Acriflavin resistance protein E																								
FN0517	AAL94713.1| Outer membrane protein tolC																	0.979	5.808						
FN0519	AAL94715.1| Hypothetical exported 24-amino acid repeat protein	0.080	6.962	2.602e-1	7.842e-1	-0.386	6.680	6.403e-2	3.635e-1	-1.396	5.281			-1.477	5.566			-0.466	6.760	1.258e-1	1.313e-1	-1.010	5.284		
FN0522	AAL94718.1| Exonuclease SBCC	0.833	5.810			1.464	6.626	2.139e-3	7.388e-3	-0.444	4.329			-1.278	5.366			0.631	7.459			-1.908	6.182		
FN0523	AAL94719.1| Exonuclease SBCD	-2.435	6.514			-1.612	7.522	1.603e-3	5.249e-3									0.823	5.086						
FN0524	AAL94720.1| DNA helicase II													1.188	7.161	1.317e-3	3.751e-4	0.131	6.289	3.37e-1	6.635e-1	1.057	7.477	3.061e-3	7.872e-3
FN0525	AAL94721.1| Penicillin-binding protein	-1.036	14.898	3.154e-2	4.824e-2	-0.149	15.969	6.84e-2	3.902e-1	-0.997	14.733	2.544e-5	1.291e-5	0.038	13.901	2.818e-1	9.405e-1	0.887	14.933	9.659e-2	8.002e-2	-0.848	14.972	6.896e-3	2.242e-2
FN0526	AAL94722.1| Florfenicol resistance protein	0.310	11.406			0.275	11.555	8.998e-3	4.123e-2	-0.169	10.723	3.901e-2	1.955e-1	-0.479	11.237			-0.035	11.865			-0.443	11.386	4.394e-3	1.267e-2
FN0527	AAL94723.1| Alanyl-tRNA synthetase	0.950	9.787	6.384e-3	6.342e-3	1.118	10.139	1.279e-3	4.01e-3	-0.976	7.656	7.51e-4	1.665e-3	-1.927	8.810	5.171e-3	2.646e-3	0.168	11.089	2.08e-1	3.312e-1	-2.095	9.163	1.056e-3	2.084e-3
FN0528	AAL94724.1| Cold shock protein	-2.764	18.819	1.531e-3	8.813e-4	-5.943	15.825	2.971e-6	8.705e-7	-0.907	20.473	7.938e-5	7.754e-5	1.857	17.912	9.068e-3	6.487e-3	-3.178	13.061	1.321e-1	1.446e-1	5.036	14.918	2e-4	2.185e-4
FN0535	AAL94731.1| Hypothetical protein	0.648	10.623	3.787e-3	3.085e-3	-0.144	10.016	6.605e-2	3.758e-1	1.124	10.896	7.88e-5	7.674e-5	0.476	11.748	1.946e-3	6.75e-4	-0.792	10.664	1.084e-2	1.924e-3	1.268	11.141	2.193e-4	2.491e-4
FN0536	AAL94732.1| DNA polymerase III, beta chain	-0.352	15.236	7.724e-2	1.622e-1	-0.290	15.482	2.938e-3	1.093e-2	0.227	15.611	3.896e-5	2.846e-5	0.579	15.463	3.396e-2	4.578e-2	0.062	15.130	3.784e-1	7.947e-1	0.517	15.709	9.179e-4	1.732e-3
FN0540	AAL94736.1| Glutamate-1-semialdehyde 2,1-aminomutase	-0.944	11.020	4.112e-2	6.849e-2	-0.525	11.624	7.07e-4	1.932e-3	-0.795	10.965	1.277e-4	1.538e-4	0.149	10.225	2.428e-1	7.688e-1	0.419	10.679	2.342e-1	3.957e-1	-0.270	10.828	1.773e-2	6.856e-2
FN0541	AAL94737.1| polysaccharide deacetylase					-0.612	7.079	5.117e-2	2.862e-1																

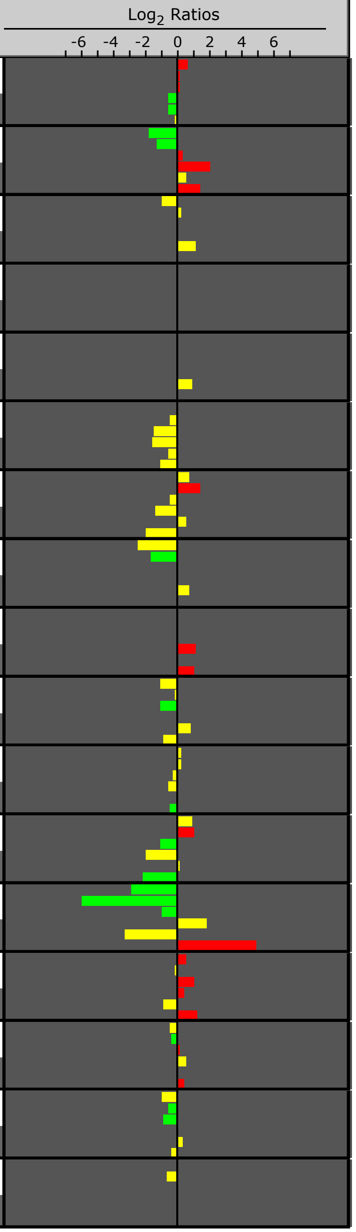

Show detected proteins only
Show all proteins
Filter by category:
GO: amino acid transport

Proteins found: 1424

Enter (or paste) list of ORFs
Find ORFs

Test	Cutoff
q-Value	.005
p-Value	

Dot Plots
Dot Plots

Signif	Direction	Applies To
yes	+	ratios, bars
no	n/a	bars
yes	–	ratios, bars
yes	+	*p*- , *q*-Values
yes	–	

FnPg vs Fn
FnPgSg vs Fn
FnSg vs FnPg
FnSg vs Fn
FnPgSg vs FnPg
FnPgSg vs FnSg

ORF	Description	*FnPg vs Fn* Ratio	Sum	*q*-Val	*p*-Val	*FnSg vs Fn* Ratio	Sum	*q*-Val	*p*-Val	*FnPgSg vs Fn* Ratio	Sum	*q*-Val	*p*-Val
FN0542	AAL94738.1| Beta 1,4 glucosyltransferase	0.849	8.771	1.668e-1	4.372e-1	1.042	9.148	2.806e-3	1.032e-2	0.228	7.946		
FN0543	AAL94739.1| Lipopolysaccharide heptosyltransferase-1	-1.094	10.321	2.493e-2	3.574e-2	-1.605	9.994	5.674e-6	2.34e-6	-0.931	10.280	3.401e-5	2.141e-5
FN0544	AAL94740.1| ADP-heptose:LPS heptosyltransferase II												
FN0546	AAL94742.1| Lipopolysaccharide core biosynthesis protein rfaQ	-0.281	5.451										
FN0547	AAL94743.1| RecA protein	-1.334	16.732	7.476e-3	7.902e-3	-0.434	17.816	9.961e-5	1.555e-4	-0.841	17.021	1.858e-3	5.534e-3
FN0549	AAL94745.1| O-sialoglycoprotein endopeptidase					1.632	5.769						
FN0550	AAL94746.1| hypothetical Protein												
FN0552	AAL94748.1| Serine racemase	0.538	8.751	4.343e-2	7.384e-2	0.596	8.994	2.955e-2	1.574e-1	-1.197	6.812		
FN0553	AAL94749.1| D-serine dehydratase	0.711	13.762	9.265e-2	2.057e-1	1.235	14.471	3.442e-3	1.334e-2	-0.155	12.692	3.125e-2	1.508e-1
FN0554	AAL94750.1| D-serine permease	1.694	6.477			1.980	6.947			-0.705	3.875		
FN0555	AAL94751.1| Transcriptional regulator, MerR family												
FN0556	AAL94752.1| unknown	-0.194	12.234	2.387e-1	6.989e-1	-0.711	11.901	3.251e-4	7.142e-4	-2.016	10.209	3.889e-5	2.836e-5
FN0557	AAL94753.1| unknown	1.582	12.845	4.794e-2	8.458e-2	2.218	13.666	4.033e-3	1.624e-2	-0.995	10.065	2.426e-3	7.614e-3
FN0558	AAL94754.1| TraT complement resistance protein precursor	2.171	14.188			0.685	12.886	1.435e-2	7.097e-2	-1.116	10.697	1.296e-4	1.57e-4
FN0559	AAL94755.1| Phosphoglucomutase	0.991	14.564	4.928e-2	8.779e-2	0.719	14.477	4.336e-6	1.524e-6	0.204	13.574	4.672e-2	2.397e-1
FN0560	AAL94756.1| Oxygen-independent coproporphyrinogen III oxidase												
FN0561	AAL94757.1| Proline synthetase associated protein	-0.125	11.120	1.507e-1	3.821e-1	-0.899	10.531	3.948e-5	4.288e-5	-0.851	10.190	1.578e-5	6.215e-6

ORF	*FnPgSg vs FnPg* Ratio	Sum	*q*-Val	*p*-Val	*FnSg vs FnPg* Ratio	Sum	*q*-Val	*p*-Val	*FnPgSg vs FnSg* Ratio	Sum	*q*-Val	*p*-Val
FN0542	-0.621	9.000			0.192	9.997	3.784e-1	7.949e-1	-0.813	9.376		
FN0543	0.163	9.390	2.35e-1	7.363e-1	-0.511	8.900	2.444e-1	4.214e-1	0.674	9.063	1.376e-3	2.901e-3
FN0544												
FN0546												
FN0547	0.493	15.891	9.93e-2	2.168e-1	0.900	16.482	6.395e-2	3.507e-2	-0.407	16.976	1.557e-2	5.891e-2
FN0549												
FN0550	-0.696	9.240			-1.381	8.739	1.179e-1	1.155e-1	0.685	8.043		
FN0552	-1.736	7.554			0.058	9.532	3.897e-1	8.338e-1	-1.794	7.797		
FN0553	-0.866	13.607	8.074e-2	1.58e-1	0.524	15.182	1.491e-1	1.876e-1	-1.390	14.316	3.49e-3	9.303e-3
FN0554	-2.399	5.773			0.286	8.642	2.214e-1	3.633e-1	-2.684	6.243		
FN0555												
FN0556	-1.821	10.218	6.762e-2	1.232e-1	-0.517	11.707	2.519e-1	4.396e-1	-1.304	9.886	1.066e-3	2.11e-3
FN0557	-2.576	11.851	3.589e-2	4.95e-2	0.637	15.248	1.458e-1	1.784e-1	-3.213	12.672	3.976e-3	1.105e-2
FN0558	-3.288	13.072			-1.486	15.058			-1.801	11.770	4.484e-3	1.303e-2
FN0559	-0.786	14.769	6.733e-2	1.224e-1	-0.272	15.468	2.57e-1	4.516e-1	-0.515	14.682	5.467e-3	1.683e-2
FN0560												
FN0561	-0.726	10.269	1.729e-2	1.737e-2	-0.774	10.406	4.26e-2	1.684e-2	0.048	9.680	8.862e-2	4.206e-1

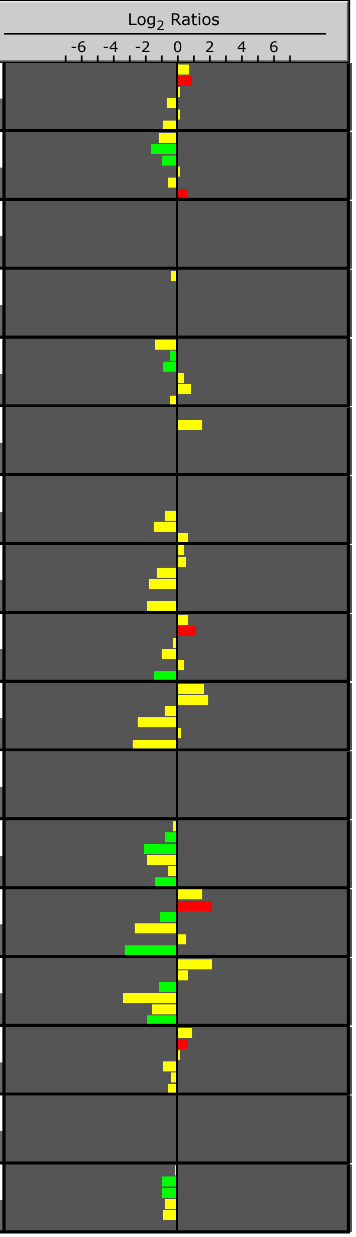

○ Show detected proteins only
○ Show all proteins
☐ Filter by category:
GO: amino acid transport

Proteins found: 1424

Enter (or paste) list of ORFs

Find ORFs

Test	Cutoff
q-Value	.005
p-Value	

Dot Plots
Dot Plots

	Signif	Direction	Applies To
(red)	yes	+	ratios, bars
(yellow)	no	n/a	bars
(green)	yes	–	ratios, bars
(pink)	yes	+	*p*- , *q*-Values
(light green)	yes	–	

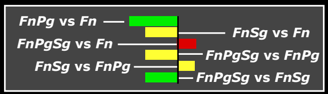

ORF	*FnPg* vs *Fn*				*FnSg* vs *Fn*				*FnPgSg* vs *Fn*				*FnPgSg* vs *FnPg*				*FnSg* vs *FnPg*				*FnPgSg* vs *FnSg*				Description
	Ratio	Sum	*q*-Val	*p*-Val	Ratio	Sum	*q*-Val	*p*-Val	Ratio	Sum	*q*-Val	*p*-Val	Ratio	Sum	*q*-Val	*p*-Val	Ratio	Sum	*q*-Val	*p*-Val	Ratio	Sum	*q*-Val	*p*-Val	
**FN0562**	-0.332	13.764	6.792e-2	1.372e-1	-0.432	13.848	6.443e-3	2.779e-2	0.267	14.159	8.121e-3	3.229e-2	0.599	14.030	1.887e-2	1.958e-2	-0.100	13.516	3.412e-1	6.762e-1	0.699	14.114	1.092e-3	2.181e-3	AAL94758.1| Hypothetical cytosolic protein
**FN0563**	-1.631	7.755	9.49e-3	1.066e-2	-1.280	8.291	6.724e-8	3.251e-9	-1.470	7.713	5.619e-4	1.157e-3	0.162	6.285	2.466e-1	7.849e-1	0.351	6.660	2.811e-1	5.104e-1	-0.190	6.821	8.957e-2	4.256e-1	AAL94759.1| putative tRNA (5-methylaminomethyl-2-thiouridylate) -methyltransferase
**FN0574**									0.236	3.764															AAL94770.1| Hypothetical cytosolic protein
**FN0576**	-0.794	11.518	4.822e-3	4.344e-3	0.165	12.661	8.411e-2	4.88e-1	-1.044	11.064	9.375e-4	2.199e-3	-0.250	10.474	1.173e-1	2.812e-1	0.959	11.867	6.031e-2	3.168e-2	-1.209	11.617	6.242e-3	1.987e-2	AAL94772.1| hypothetical protein
**FN0577**	1.068	5.851			1.419	6.387											0.351	7.455							AAL94773.1| Hypothetical protein
**FN0579**	1.502	18.883	2.34e-2	3.3e-2	0.552	18.117	1.224e-2	5.897e-2	-0.091	17.086	9.155e-3	3.72e-2	-1.593	18.792	2.565e-2	3.024e-2	-0.950	19.619	8.566e-2	6.238e-2	-0.643	18.026	1.183e-2	4.285e-2	AAL94775.1| Hypothetical cytosolic protein
**FN0580**	-2.833	6.833			-1.525	8.325	2.408e-4	4.815e-4									1.308	5.492							AAL94776.1| Penicillin-binding protein
**FN0581**	-1.229	7.229			-0.356	8.287			-1.448	6.807			-0.219	5.781			0.873	7.058			-1.092	6.839			AAL94777.1| Lipoprotein releasing system transmembrane protein lolE
**FN0582**	-1.440	9.780			-0.387	11.018	1.503e-2	7.478e-2	-1.230	9.787	1.291e-3	3.398e-3	0.210	8.550			1.053	9.578			-0.843	9.788	7.256e-3	2.388e-2	AAL94778.1| Lipoprotein releasing system ATP-binding protein lolD
**FN0583**	1.304	7.480			0.411	6.772											-0.893	8.076							AAL94779.1| Hypothetical Exported Protein
**FN0585**	0.893	7.198	1.115e-1	2.601e-1	0.034	6.524	1.367e-1	8.413e-1	-1.050	5.050			-1.944	6.148			-0.859	7.417	1.832e-1	2.703e-1	-1.085	5.473			AAL94781.1| Two-component response regulator czcR
**FN0586**	0.879	6.522			0.459	6.286	5.033e-2	2.811e-1	-0.720	4.720			-1.599	5.802			-0.420	7.165			-1.178	5.567			AAL94782.1| Two-component sensor kinase czcS
**FN0590**	0.905	8.602	1.142e-1	2.679e-1	1.373	9.254	1.808e-3	6.042e-3	-1.114	6.380	3.959e-3	1.375e-2	-2.019	7.489	6.446e-2	1.154e-1	0.468	10.160	2.207e-1	3.614e-1	-2.486	8.141	1.893e-3	4.316e-3	AAL94786.1| N-acyl-L-amino acid amidohydrolase
**FN0592**	-0.593	12.849	8.665e-4	3.742e-4	-0.258	13.368	5.754e-2	3.244e-1	-0.765	12.472	9.214e-4	2.15e-3	-0.173	12.083	1.066e-1	2.44e-1	0.335	12.775	1.947e-1	3.006e-1	-0.508	12.602	3.992e-2	1.727e-1	AAL94788.1| ATP-dependent DNA helicase pcrA
**FN0593**	-0.011	11.085	2.828e-1	8.792e-1	-0.312	10.969	3.579e-3	1.403e-2	0.036	10.928	1.128e-1	6.434e-1	0.047	11.121	1.803e-1	5.145e-1	-0.301	10.957	3.108e-2	9.699e-3	0.348	11.005	3.27e-3	8.556e-3	AAL94789.1| UDP-3-O-[3-hydroxymyristoyl] N-acetylglucosamine deacetylase
**FN0594**	4.420	10.596			3.705	10.066			0.014	5.986			-4.406	10.610			-0.715	14.485			-3.691	10.080			AAL94790.1| (3R)-hydroxymyristoyl-[acyl carrier protein] dehydratase
**FN0595**	0.421	9.089			-0.840	8.012	5.036e-5	6.152e-5	0.824	9.288	5.432e-2	2.84e-1	0.403	9.913			-1.261	8.433			1.664	8.836	3.179e-2	1.33e-1	AAL94791.1| Acyl-[acyl-carrier-protein]-UDP-N-acetylglucosamine O-acyltransferase

Log$_2$ Ratios

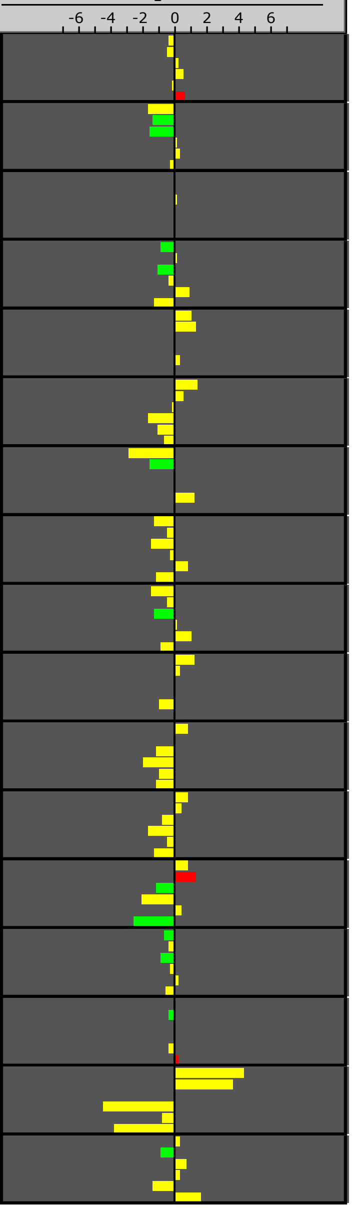

Show detected proteins only
Show all proteins
Filter by category:
GO: amino acid transport

Proteins found:
1424

Enter (or paste) list of ORFs

Find ORFs

Test	Cutoff
q-Value	.005
p-Value	

Dot Plots
Dot Plots

	Signif	Direction	Applies To
(red)	yes	+	ratios, bars
(yellow)	no	n/a	bars
(green)	yes	–	ratios, bars
(pink)	yes	+	*p*- , *q*-Values
(light green)	yes	–	*p*- , *q*-Values

FnPg vs Fn
FnPgSg vs Fn
FnSg vs FnPg
FnSg vs Fn
FnPgSg vs FnPg
FnPgSg vs FnSg

ORF	*FnPg* vs *Fn*				*FnSg* vs *Fn*				*FnPgSg* vs *Fn*				*FnPgSg* vs *FnPg*				*FnSg* vs *FnPg*				*FnPgSg* vs *FnSg*				Description
	Ratio	Sum	*q*-Val	*p*-Val	Ratio	Sum	*q*-Val	*p*-Val	Ratio	Sum	*q*-Val	*p*-Val	Ratio	Sum	*q*-Val	*p*-Val	Ratio	Sum	*q*-Val	*p*-Val	Ratio	Sum	*q*-Val	*p*-Val	
**FN0596**	-0.887	9.143	1.623e-2	2.057e-2	-0.667	9.548	3.358e-3	1.293e-2	-1.328	8.498			-0.441	7.815			0.221	8.661	2.788e-1	5.048e-1	-0.661	8.220			AAL94792.1| Hypothetical protein
**FN0597**	-0.057	10.396	2.393e-1	7.015e-1	-0.322	10.315	2.133e-2	1.099e-1	-1.240	9.009	1.167e-3	2.945e-3	-1.184	9.156	2.985e-3	1.217e-3	-0.266	10.258	1.98e-2	4.58e-3	-0.918	9.075	1.111e-3	2.232e-3	AAL94793.1| Lipid-A-disaccharide synthase
**FN0598**	-1.214	8.786	2.897e-3	2.133e-3	-0.762	9.422	2.596e-3	9.369e-3	-0.350	9.446	1.566e-2	6.843e-2	0.864	8.436	1.403e-2	1.267e-2	0.452	8.208	1.233e-1	1.26e-1	0.412	9.072	2.045e-3	4.748e-3	AAL94794.1| Phospholipid-lipopolysaccharide ABC transporter
**FN0600**	-0.134	12.447	2.673e-1	8.134e-1	-0.004	12.761	1.483e-1	9.256e-1	0.162	12.539	5.949e-2	3.142e-1	0.296	12.609	2.003e-1	5.977e-1	0.130	12.627	3.857e-1	8.196e-1	0.166	12.923	6.675e-2	3.078e-1	AAL94796.1| Hypothetical protein
**FN0601**																									AAL94797.1| Hypothetical exported 24-amino acid repeat protein
**FN0602**	-0.450	14.400	1.856e-2	2.459e-2	-1.050	13.985	1.203e-5	7.965e-6	-0.483	14.163	1.868e-4	2.623e-4	-0.033	13.917	2.575e-1	8.315e-1	-0.600	13.535	6.163e-2	3.287e-2	0.567	13.502	1.503e-4	1.489e-4	AAL94798.1| Hypothetical protein
**FN0603**	0.608	5.392			1.234	6.202											0.626	6.810							AAL94799.1| Transcriptional regulatory protein, LYSR family
**FN0605**																									AAL94801.1| Aspartate aminotransferase
**FN0608**	1.321	12.367	3.34e-2	5.204e-2	1.631	12.861	3.087e-8	6.398e-10	0.080	10.922	2.224e-2	1.017e-1	-1.241	12.447	3.975e-2	5.704e-2	0.310	14.182	1.945e-1	2.999e-1	-1.551	12.941	4.05e-8	1.022e-9	AAL94804.1| Exoribonuclease II
**FN0609**																	0.782	6.137							AAL94805.1| Small protein B
**FN0610**	-0.386	15.997	1.266e-1	3.055e-1	-0.706	15.862	7.534e-6	3.747e-6	-0.543	15.636	2.075e-4	3.071e-4	-0.157	15.454	2.281e-1	7.085e-1	-0.320	15.476	2.7e-1	4.828e-1	0.163	15.319	2.146e-2	8.568e-2	AAL94806.1| unknown
**FN0611**	0.008	18.655	2.588e-1	7.784e-1	0.328	19.160	3.215e-3	1.223e-2	-0.494	17.949	1.756e-3	5.151e-3	-0.502	18.161	8.272e-3	5.665e-3	0.320	19.168	3.881e-2	1.429e-2	-0.822	18.665	2.054e-4	2.268e-4	AAL94807.1| Threonyl-tRNA synthetase
**FN0612**	-1.943	12.544	1.296e-3	7.08e-4	-1.557	13.116	7.648e-4	2.154e-3	-0.449	13.835	3.803e-2	1.897e-1	1.495	12.095	3.393e-2	4.572e-2	0.387	11.172	2.415e-1	4.143e-1	1.108	12.667	2.04e-2	8.069e-2	AAL94808.1| Hypothetical protein
**FN0614**																									AAL94810.1| Export ABC transporter
**FN0615**																									AAL94811.1| Export ABC transporter
**FN0616**	-3.077	8.692							-0.494	11.072	1.783e-2	7.93e-2	2.583	8.198											AAL94812.1| Hypothetical protein
**FN0617**	0.513	16.384	1.465e-1	3.681e-1	-1.376	14.679	5.643e-6	2.319e-6	-0.472	15.195	9.75e-5	1.048e-4	-0.985	15.912	8.778e-2	1.788e-1	-1.889	15.192	9.783e-2	8.183e-2	0.904	14.207	8.709e-6	2.273e-6	AAL94813.1| DNA polymerase III, beta chain

Log_2 Ratios

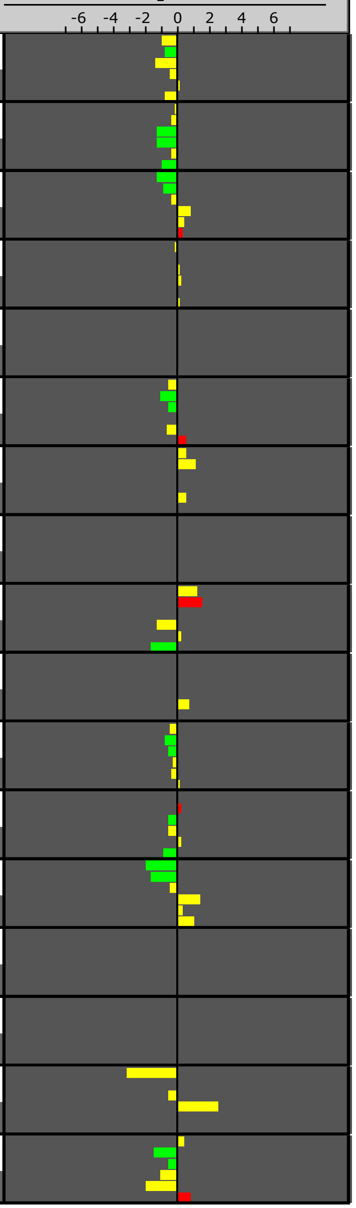

Show detected proteins only
Show all proteins
Filter by category:
GO: amino acid transport

Proteins found: 1424

Enter (or paste) list of ORFs

Find ORFs

Test	Cutoff
q-Value	.005
p-Value	

Dot Plots
Dot Plots

	Signif	Direction	Applies To
red	yes	+	ratios, bars
yellow	no	n/a	bars
green	yes	–	ratios, bars
pink	yes	+	*p*- , *q*-Values
light green	yes	–	

FnPg vs Fn
FnSg vs Fn
FnPgSg vs Fn
FnPgSg vs FnPg
FnSg vs FnPg
FnPgSg vs FnSg

ORF	Description	FnPg vs Fn Ratio	FnPg vs Fn Sum	FnPg vs Fn *q*-Val	FnPg vs Fn *p*-Val	FnSg vs Fn Ratio	FnSg vs Fn Sum	FnSg vs Fn *q*-Val	FnSg vs Fn *p*-Val	FnPgSg vs Fn Ratio	FnPgSg vs Fn Sum	FnPgSg vs Fn *q*-Val	FnPgSg vs Fn *p*-Val	FnPgSg vs FnPg Ratio	FnPgSg vs FnPg Sum	FnPgSg vs FnPg *q*-Val	FnPgSg vs FnPg *p*-Val	FnSg vs FnPg Ratio	FnSg vs FnPg Sum	FnSg vs FnPg *q*-Val	FnSg vs FnPg *p*-Val	FnPgSg vs FnSg Ratio	FnPgSg vs FnSg Sum	FnPgSg vs FnSg *q*-Val	FnPgSg vs FnSg *p*-Val
FN0618	AAL94814.1| Spermidine/putrescine-binding protein	-0.465	14.090	1.212e-1	2.89e-1	-0.791	13.948	2.997e-3	1.121e-2	-1.074	13.277	4.123e-5	3.105e-5	-0.609	13.016	1.303e-1	3.288e-1	-0.326	13.483	2.987e-1	5.562e-1	-0.283	12.874	5.864e-2	2.664e-1
FN0619	AAL94815.1| Small-conductance mechanosensitive channel	-1.035	8.489	3.386e-2	5.301e-2	-1.120	8.589	4.304e-4	1.011e-3	-0.843	8.477			0.192	7.646			-0.085	7.554	4.027e-1	8.801e-1	0.277	7.746		
FN0621	AAL94817.1| 4-hydroxybutyrate coenzyme A transferase	-0.074	11.610	2.679e-1	8.158e-1	-0.505	11.363	2.115e-3	7.286e-3	0.032	11.512	1.478e-1	8.778e-1	0.106	11.642	2.437e-1	7.723e-1	-0.431	11.289	1.955e-1	3.027e-1	0.537	11.395	2.307e-2	9.287e-2
FN0622	AAL94818.1| 8-oxoguanine DNA glycosylase	2.520	7.838	4.561e-2	7.908e-2	3.782	9.284	1.265e-3	3.955e-3	-0.586	4.529	1.697e-4	2.285e-4	-3.105	7.252	4.485e-2	6.767e-2	1.262	11.804	4.144e-2	1.602e-2	-4.367	8.699	1.66e-3	3.674e-3
FN0625	AAL94821.1| Aspartate aminotransferase	-0.443	9.165			-0.989	8.803			0.628	10.033	2.237e-2	1.024e-1	1.071	9.793			-0.547	8.360			1.618	9.431		
FN0626	AAL94822.1| Hypothetical cytosolic protein																								
FN0627	AAL94823.1| Glucosamine--fructose-6-phosphate aminotransferase (isomerizing)	-1.099	14.249	1.614e-2	2.042e-2	-0.867	14.665	2.127e-4	4.124e-4	0.549	15.693	1.368e-4	1.7e-4	1.648	14.798	6.612e-3	3.834e-3	0.231	13.566	3.026e-1	5.665e-1	1.417	15.214	4.805e-7	4.851e-8
FN0628	AAL94824.1| Glucosamine--fructose-6-phosphate aminotransferase (isomerizing)					-0.018	5.529	1.536e-1	9.644e-1	1.666	6.825	6.355e-4	1.355e-3									1.684	7.195	1.338e-4	1.266e-4
FN0629	AAL94825.1| PTS system, IID component	-0.251	8.251			0.344	9.031	4.925e-2	2.745e-1	1.101	9.400	3.787e-4	6.97e-4	1.353	9.353			0.595	8.780			0.757	10.133	5.895e-3	1.853e-2
FN0630	AAL94826.1| PTS system, IIC component									0.839	8.337														
FN0631	AAL94827.1| PTS system, IIB component	-0.334	8.509			-0.404	8.623	5.102e-3	2.117e-2	-0.712	7.927	6.122e-3	2.322e-2	-0.378	7.797			-0.070	8.289			-0.308	7.911	5.295e-2	2.379e-1
FN0633	AAL94829.1| Replication protein	0.570	7.244			0.057	6.916	7.709e-2	4.439e-1	-0.507	5.963	5.275e-2	2.747e-1	-1.077	6.737			-0.513	7.485			-0.564	6.408	5.091e-2	2.275e-1
FN0634	AAL94830.1| GTP-binding protein TypA/BipA	-0.481	14.010	1.474e-1	3.711e-1	-2.139	12.536	1.339e-4	2.301e-4	-0.464	13.823	1.253e-3	3.257e-3	0.016	13.546	2.897e-1	9.78e-1	-1.658	12.056	1.414e-1	1.665e-1	1.675	12.072	3.101e-5	1.7e-5
FN0636	AAL94832.1| Hypothetical protein																								
FN0637	AAL94833.1| Hypothetical exported 24-amino acid repeat protein									-0.561	5.319	7.934e-3	3.142e-2												
FN0643	AAL94839.1| hypothetical DNA-binding protein	0.764	9.037	6.087e-2	1.184e-1	0.129	8.587	1.19e-1	7.175e-1	-0.252	7.818	3.311e-2	1.615e-1	-1.016	8.785	4.744e-2	7.347e-2	-0.635	9.351	1.48e-1	1.846e-1	-0.381	8.335	7.704e-2	3.611e-1
FN0644	AAL94840.1| Uroporphyrin-III C-methyltransferase	-0.006	11.352	2.955e-1	9.351e-1	-0.635	10.907	3.776e-3	1.502e-2	-0.400	10.754	3.796e-2	1.893e-1	-0.395	10.952	9.26e-2	1.941e-1	-0.629	10.902	4.995e-2	2.264e-2	0.235	10.507	1.079e-1	5.229e-1

Log_2 Ratios

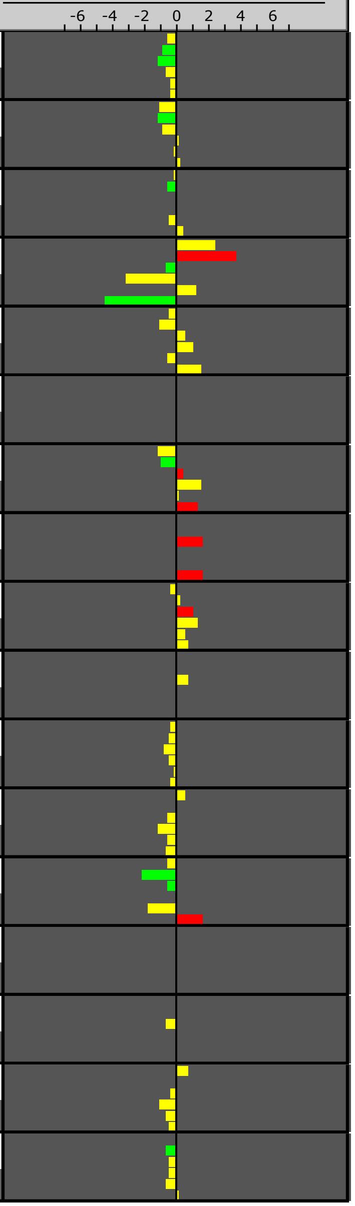

Show detected proteins only
Show all proteins
Filter by category:
GO: amino acid transport

Proteins found:
1424

Enter (or paste) list of ORFs

Find ORFs

Test	Cutoff
q-Value	.005
p-Value	

Dot Plots
Dot Plots

	Signif	Direction	Applies To
(red)	yes	+	ratios, bars
(yellow)	no	n/a	bars
(green)	yes	–	ratios, bars
(pink)	yes	+	*p*- , *q*-Values
(light green)	yes	–	*p*- , *q*-Values

FnPg vs Fn
FnSg vs Fn
FnPgSg vs Fn
FnPgSg vs FnPg
FnSg vs FnPg
FnPgSg vs FnSg

ORF	Description	FnPg vs Fn Ratio	Sum	*q*-Val	*p*-Val	FnSg vs Fn Ratio	Sum	*q*-Val	*p*-Val	FnPgSg vs Fn Ratio	Sum	*q*-Val	*p*-Val	FnPgSg vs FnPg Ratio	Sum	*q*-Val	*p*-Val	FnSg vs FnPg Ratio	Sum	*q*-Val	*p*-Val	FnPgSg vs FnSg Ratio	Sum	*q*-Val	*p*-Val
FN0645	AAL94841.1| Porphobilinogen deaminase	-0.441	6.247	1.22e-1	2.915e-1	-0.851	6.021			-0.577	5.907	9.526e-3	3.902e-2	-0.136	5.670	2.437e-1	7.726e-1	-0.410	5.580			0.275	5.444		
FN0646	AAL94842.1| Glutamyl-tRNA reductase																					2.069	5.239		
FN0647	AAL94843.1| transcriptional regulator																								
FN0649	AAL94845.1| Exoenzymes regulatory protein aepA precursor																								
FN0651	AAL94847.1| Ribosomal large subunit pseudouridine synthase D																								
FN0652	AAL94848.1| Glyceraldehyde 3-phosphate dehydrogenase	1.042	23.635	5.399e-2	9.959e-2	1.685	24.463	5.153e-4	1.277e-3	-0.162	22.228	2.716e-2	1.279e-1	-1.204	23.474	5.014e-2	7.989e-2	0.643	25.505	9.371e-2	7.596e-2	-1.847	24.301	4.383e-4	6.266e-4
FN0653	AAL94849.1| unknown	0.280	11.739	1.59e-1	4.101e-1	0.939	12.582	4.025e-3	1.62e-2	-1.204	10.051	6.921e-4	1.509e-3	-1.484	10.535	2.845e-2	3.52e-2	0.659	12.862	8.06e-2	5.492e-2	-2.143	11.378	1.208e-3	2.477e-3
FN0654	AAL94850.1| Phosphoglycerate kinase	-0.077	18.744	2.383e-1	6.975e-1	0.924	19.929	7.066e-4	1.93e-3	-0.605	18.012	6.725e-4	1.457e-3	-0.528	18.139	5.338e-2	8.806e-2	1.001	19.852	7.062e-3	1.02e-3	-1.529	19.324	2.485e-4	2.983e-4
FN0655	AAL94851.1| unknown	-2.886	9.805			-2.362	10.514	8.012e-4	2.282e-3	-0.599	11.889	1.606e-2	7.04e-2	2.287	9.206			0.524	7.628			1.763	9.915	4.916e-3	1.47e-2
FN0656	AAL94852.1| Hypothetical protein									-1.318	11.420	1.331e-3	3.554e-3												
FN0657	AAL94853.1| Acetyltransferase	1.009	7.910			2.260	9.346											1.251	10.355						
FN0658	AAL94854.1| ABC transporter substrate-binding protein	-1.362	13.326	3.023e-4	7.136e-5	-1.617	13.256	5.919e-5	7.705e-5	0.052	14.536	4.079e-2	2.059e-1	1.414	13.378	1.064e-6	1.419e-8	-0.254	11.894	2.477e-3	1.78e-4	1.668	13.308	4.392e-6	8.367e-7
FN0660	AAL94856.1| ABC transporter ATP-binding protein	-0.313	7.005	6.954e-2	1.414e-1	-0.733	6.770	1.002e-2	4.685e-2	-0.653	6.461	3.459e-3	1.168e-2	-0.340	6.351	1.009e-1	2.225e-1	-0.419	6.456	1.751e-1	2.506e-1	0.080	6.117	1.598e-1	8.224e-1
FN0662	AAL94858.1| Formiminoglutamase	0.384	11.041	2.322e-1	6.744e-1	0.581	11.423	6.587e-4	1.756e-3	0.263	10.716	1.425e-2	6.149e-2	-0.121	11.303	2.691e-1	8.829e-1	0.198	11.806	3.768e-1	7.893e-1	-0.318	11.685	2.059e-3	4.791e-3
FN0664	AAL94860.1| 2-nitropropane dioxygenase	-1.329	13.462	7.1e-3	7.367e-3	-0.313	14.662	9.048e-2	5.286e-1	-0.587	14.000	2.851e-3	9.277e-3	0.742	12.874	5.797e-2	9.97e-2	1.016	13.333	1.705e-1	2.394e-1	-0.274	14.075	1.307e-1	6.503e-1
FN0666	AAL94862.1| Hypothetical protein					-1.071	6.290	1.61e-4	2.956e-4																
FN0668	AAL94864.1| High-affinity zinc uptake system protein znuA precursor	-0.885	9.350	3.602e-2	5.742e-2	-0.869	9.550	1.004e-6	1.511e-7	-1.596	8.436	1.109e-3	2.747e-3	-0.710	7.754	1.171e-1	2.803e-1	0.016	8.665	4.265e-1	9.708e-1	-0.727	7.955	1.69e-2	6.487e-2

Log$_2$ Ratios

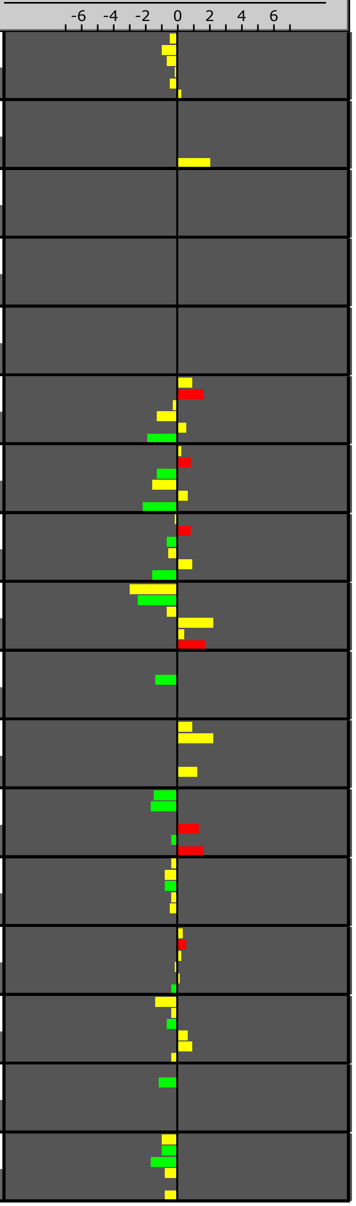

Show detected proteins only
Show all proteins
Filter by category:
GO: amino acid transport

Proteins found: 1424

Enter (or paste) list of ORFs

Find ORFs

Test	Cutoff
q-Value	.005
p-Value	

Dot Plots
Dot Plots

	Signif	Direction	Applies To
red	yes	+	ratios, bars
yellow	no	n/a	bars
green	yes	–	ratios, bars
pink	yes	+	*p*- , *q*-Values
light green	yes	–	

FnPg vs Fn
FnPgSg vs Fn
FnSg vs FnPg
FnSg vs Fn
FnPgSg vs FnPg
FnPgSg vs FnSg

ORF	Protein	FnPg vs Fn Ratio	Sum	*q*-Val	*p*-Val	FnSg vs Fn Ratio	Sum	*q*-Val	*p*-Val	FnPgSg vs Fn Ratio	Sum	*q*-Val	*p*-Val	FnPgSg vs FnPg Ratio	Sum	*q*-Val	*p*-Val	FnSg vs FnPg Ratio	Sum	*q*-Val	*p*-Val	FnPgSg vs FnSg Ratio	Sum	*q*-Val	*p*-Val
FN0672	AAL94868.1| ATPase													0.629	6.629			0.958	7.142			-0.328	7.772		
FN0675	AAL94871.1| 60 kDa chaperonin GROEL	0.293	20.681	2.319e-1	6.732e-1	1.552	22.124	6.365e-3	2.739e-2	-0.047	20.137	9.545e-2	5.335e-1	-0.340	20.634	2.088e-1	6.325e-1	1.259	22.417	7.412e-2	4.62e-2	-1.599	22.077	7.717e-3	2.578e-2
FN0676	AAL94872.1| 10 kDa chaperonin GROES	-2.238	13.222			-2.415	13.229	3.02e-4	6.479e-4	-1.245	14.012	5.316e-4	1.079e-3	0.993	11.977			-0.177	10.991			1.171	11.985	2.937e-4	3.755e-4
FN0677	AAL94873.1| Hypothetical protein	-0.452	12.428	7.458e-2	1.549e-1	-0.420	12.645	9.004e-4	2.623e-3	-1.849	10.827	1.647e-5	6.684e-6	-1.397	10.579	3.454e-2	4.698e-2	0.032	12.193	4.123e-1	9.158e-1	-1.430	10.796	9.163e-5	7.743e-5
FN0678	AAL94874.1| Ser/Thr protein kinase	-2.001	10.975	3.84e-3	3.143e-3	-0.741	12.419	6.85e-5	9.561e-5	-0.784	11.988	9.635e-5	1.029e-4	1.217	10.191	3.294e-2	4.369e-2	1.260	10.418	6.35e-2	3.463e-2	-0.043	11.635	1.3e-1	6.463e-1
FN0679	AAL94875.1| GTPase	-0.047	8.116	2.976e-1	9.447e-1	-1.004	7.344			-2.037	5.922			-1.990	6.079			-0.957	7.297			-1.033	5.307		
FN0680	AAL94876.1| Ribulose-phosphate 3-epimerase													-0.422	5.578			0.608	6.793			-1.031	6.370		
FN0681	AAL94877.1| Transcriptional regulator, MarR family	0.072	16.439	2.266e-1	6.533e-1	-0.045	16.507	1.015e-1	6.006e-1	-0.629	15.534	5.92e-4	1.237e-3	-0.701	15.810	2.402e-2	2.75e-2	-0.117	16.579	2.764e-1	4.986e-1	-0.585	15.877	2.382e-3	5.769e-3
FN0682	AAL94878.1| Fibronectin-binding protein-like protein A	-0.287	8.933	1.441e-1	3.602e-1	0.352	9.756	1.795e-2	9.098e-2	-0.398	8.618	4.682e-2	2.403e-1	-0.111	8.535	2.443e-1	7.748e-1	0.639	9.469	6.915e-2	4.046e-2	-0.750	9.358	8.597e-3	2.928e-2
FN0684	AAL94880.1| Prismane protein													-2.867	6.240			-0.796	8.495	1.86e-1	2.775e-1	-2.070	5.628		
FN0685	AAL94881.1| Sodium/pantothenate symporter	1.715	11.795			1.229	11.493	8.405e-3	3.802e-2	-1.816	8.059	3.693e-5	2.531e-5	-3.531	9.978			-0.486	13.208			-3.046	9.677	4.206e-3	1.193e-2
FN0688	AAL94884.1| Hypothetical protein	-0.814	10.428			0.481	11.908	1.35e-4	2.328e-4	-2.434	8.604	1.698e-8	3.432e-10	-1.621	7.994			1.295	11.094			-2.916	9.474	1.716e-5	7.086e-6
FN0689	AAL94885.1| Hypothetical protein	-1.400	15.034	7.674e-3	8.181e-3	-0.705	15.913	7.982e-3	3.579e-2	-1.277	14.954	3.587e-5	2.382e-5	0.123	13.758	2.44e-1	7.739e-1	0.695	14.514	1.458e-1	1.783e-1	-0.571	14.637	3.858e-2	1.661e-1
FN0692	AAL94888.1| Nitrogen regulation protein NIFR3					-1.624	7.070	3.245e-3	1.238e-2	-2.211	6.096											-0.587	4.860		
FN0693	AAL94889.1| DNA mismatch repair protein mutS	-0.715	9.438							-1.515	8.434			-0.800	7.923										
FN0694	AAL94890.1| S-layer protein	-1.205	11.775	2.013e-3	1.303e-3	-0.540	12.624	2.427e-3	8.634e-3	-0.881	11.895	4.819e-4	9.525e-4	0.324	10.894	2.604e-2	3.092e-2	0.664	11.419	5.857e-4	8.19e-6	-0.341	11.743	4.346e-3	1.248e-2
FN0695	AAL94891.1| ABC transporter ATP-binding protein	-0.255	9.735	1.841e-1	5.011e-1	0.494	10.669	8.939e-3	4.09e-2	-0.332	9.455	4.54e-2	2.321e-1	-0.077	9.403	2.599e-1	8.421e-1	0.749	10.414	7.742e-2	5.045e-2	-0.826	10.337	9.512e-4	1.814e-3

Log_2 Ratios

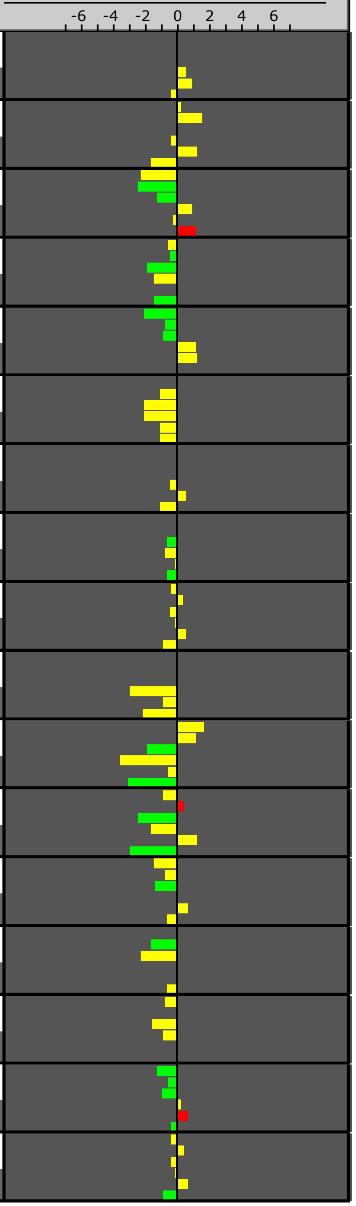

Show detected proteins only
Show all proteins
Filter by category:
GO: amino acid transport

Proteins found: 1424

Enter (or paste) list of ORFs

Find ORFs

Test	Cutoff
q-Value	.005
p-Value	

Dot Plots
Dot Plots

	Signif	Direction	Applies To
red	yes	+	ratios, bars
yellow	no	n/a	bars
green	yes	–	ratios, bars
pink	yes	+	*p*- , *q*-Values
light green	yes	–	*p*- , *q*-Values

FnPg vs Fn
FnPgSg vs Fn
FnSg vs FnPg
FnSg vs Fn
FnPgSg vs FnPg
FnPgSg vs FnSg

ORF	Protein	FnPg vs Fn Ratio	FnPg vs Fn Sum	FnPg vs Fn q-Val	FnPg vs Fn p-Val	FnSg vs Fn Ratio	FnSg vs Fn Sum	FnSg vs Fn q-Val	FnSg vs Fn p-Val	FnPgSg vs Fn Ratio	FnPgSg vs Fn Sum	FnPgSg vs Fn q-Val	FnPgSg vs Fn p-Val	FnPgSg vs FnPg Ratio	FnPgSg vs FnPg Sum	FnPgSg vs FnPg q-Val	FnPgSg vs FnPg p-Val	FnSg vs FnPg Ratio	FnSg vs FnPg Sum	FnSg vs FnPg q-Val	FnSg vs FnPg p-Val	FnPgSg vs FnSg Ratio	FnPgSg vs FnSg Sum	FnPgSg vs FnSg q-Val	FnPgSg vs FnSg p-Val
FN0697	AAL94893.1| Alanyl-tRNA synthetase	-0.888	15.023	2.426e-3	1.676e-3	-0.654	15.441	2.525e-4	5.114e-4	-0.279	15.429	1.298e-3	3.428e-3	0.610	14.744	8.259e-3	5.648e-3	0.234	14.553	1.472e-1	1.822e-1	0.376	15.163	2.452e-3	5.992e-3
FN0699	AAL94895.1| Protein translocase subunit secD	-2.041	12.095	2.411e-4	5.118e-5	-2.538	11.782	4.124e-5	4.597e-5	-0.586	13.346	2.026e-3	6.156e-3	1.455	11.509	6.894e-3	4.104e-3	-0.498	9.741	1.948e-1	3.009e-1	1.952	11.196	1.933e-4	2.085e-4
FN0700	AAL94896.1| Protein translocase subunit secF	-1.970	10.170	8.457e-4	3.581e-4	-3.208	9.117	4.85e-4	1.179e-3	-1.124	10.813	1.864e-3	5.557e-3	0.847	9.046	2.532e-2	2.968e-2	-1.237	7.147	8.815e-2	6.643e-2	2.084	7.994	3.144e-6	5.274e-7
FN0701	AAL94897.1| Methyltransferase	-0.323	15.171	7.1e-4	2.664e-4	-0.888	14.791	1.253e-5	8.483e-6	-0.241	15.049	4.958e-4	9.89e-4	0.082	14.930	7.371e-2	1.388e-1	-0.565	14.468	2.414e-3	1.68e-4	0.647	14.550	8.241e-5	6.706e-5
FN0705	AAL94901.1| DNA polymerase I	-0.948	13.356	6.438e-3	6.416e-3	-0.554	13.935	1.123e-4	1.815e-4	-0.928	13.173	3.658e-5	2.481e-5	0.020	12.428	2.809e-1	9.364e-1	0.395	12.987	1.301e-1	1.401e-1	-0.375	13.007	1.004e-3	1.947e-3
FN0706	AAL94902.1| Hypothetical cytosolic protein					-1.443	5.443			-0.664	5.834											0.779	4.779		
FN0707	AAL94903.1| Riboflavin kinase	-0.919	8.340	2.628e-2	3.818e-2	-1.677	7.767	9.49e-4	2.796e-3	-1.492	7.564	1.176e-3	2.978e-3	-0.572	6.849	1.271e-1	3.164e-1	-0.758	6.848	1.654e-1	2.271e-1	0.185	6.275	1.028e-1	4.958e-1
FN0710	AAL94906.1| Hypothetical protein	-0.325	11.975	3.353e-2	5.231e-2	-0.551	11.934	2.344e-3	8.271e-3	-0.639	11.457	1.889e-3	5.652e-3	-0.314	11.337	5.238e-2	8.549e-2	-0.226	11.609	1.391e-1	1.607e-1	-0.088	11.295	1.796e-2	6.956e-2
FN0711	AAL94907.1| Phosphopantothenate--cysteine ligase	0.574	7.601			1.073	8.284	1.013e-2	4.741e-2	-0.279	6.543	6.685e-2	3.574e-1	-0.854	7.321			0.499	8.858			-1.353	8.005	8.191e-3	2.767e-2
FN0714	AAL94910.1| NADH oxidase																	0.579	6.868	8.837e-2	6.68e-2				
FN0715	AAL94911.1| Hypothetical protein	0.137	14.316	2.68e-1	8.164e-1	0.029	14.392	9.823e-2	5.789e-1	0.854	14.829	4.094e-3	1.433e-2	0.717	15.170	8.524e-2	1.711e-1	-0.108	14.529	3.952e-1	8.53e-1	0.825	15.246	4.6e-3	1.348e-2
FN0716	AAL94912.1| hypothetical protein	-0.970	11.302	2.889e-2	4.303e-2	-0.235	12.221	2.347e-2	1.221e-1	-1.301	10.767	1.141e-3	2.855e-3	-0.331	10.001	1.889e-1	5.491e-1	0.735	11.251	1.181e-1	1.158e-1	-1.066	10.920	1.651e-5	6.648e-6
FN0717	AAL94913.1| Ribosomal small subunit pseudouridine synthase A					-0.747	5.391			-0.553	5.197											0.194	4.838		
FN0719	AAL94915.1| Hypothetical cytosolic protein																								
FN0720	AAL94916.1| Protein Translation Elongation Factor P (EF-P)	-1.271	13.663	1.901e-4	3.531e-5	-1.166	13.952	2.368e-3	8.377e-3	-1.880	12.850	7.657e-5	7.375e-5	-0.610	11.783	2.682e-2	3.226e-2	0.104	12.681	3.747e-1	7.823e-1	-0.714	12.072	4.008e-2	1.736e-1
FN0721	AAL94917.1| Hypothetical protein	-2.523	10.323	1.122e-3	5.666e-4	-1.317	11.714	9.684e-7	1.428e-7	-1.131	11.511	2.317e-4	3.61e-4	1.392	9.191	9.474e-3	6.935e-3	1.207	9.191	5.226e-2	2.476e-2	0.185	10.583	4.316e-2	1.888e-1
FN0722	AAL94918.1| WD-repeat family protein	-0.674	7.275	4.278e-2	7.23e-2	-0.475	7.658	1.291e-2	6.278e-2	-1.872	5.872			-1.198	5.402			0.199	6.984	3.202e-1	6.151e-1	-1.397	5.786		

Log2 Ratios

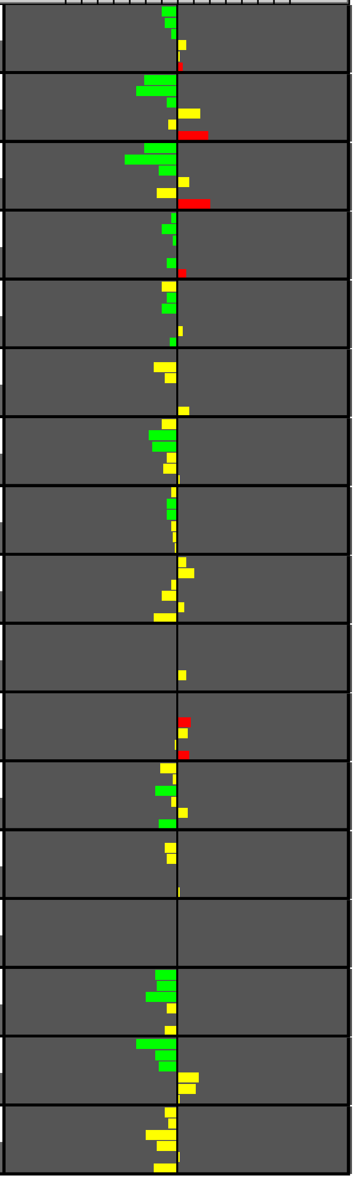

Show detected proteins only
Show all proteins
Filter by category:
GO: amino acid transport

Proteins found: 1424

Enter (or paste) list of ORFs

Find ORFs

Test	Cutoff
q-Value	.005
p-Value	

Dot Plots
Dot Plots

	Signif	Direction	Applies To
red	yes	+	ratios, bars
yellow	no	n/a	bars
green	yes	–	ratios, bars
pink	yes	+	p- , q-Values
light green	yes	–	p- , q-Values

FnPg vs Fn — FnSg vs Fn
FnPgSg vs Fn — FnPgSg vs FnPg
FnSg vs FnPg — FnPgSg vs FnSg

	FnPg vs *Fn*				*FnSg* vs *Fn*				*FnPgSg* vs *Fn*				*FnPgSg* vs *FnPg*				*FnSg* vs *FnPg*				*FnPgSg* vs *FnSg*			
ORF	Ratio	Sum	*q*-Val	*p*-Val	Ratio	Sum	*q*-Val	*p*-Val	Ratio	Sum	*q*-Val	*p*-Val	Ratio	Sum	*q*-Val	*p*-Val	Ratio	Sum	*q*-Val	*p*-Val	Ratio	Sum	*q*-Val	*p*-Val
**FN0723**																	0.230	4.414						
	AAL94919.1| Hypothetical protein																							
**FN0724**	0.527	10.961	1.99e-3	1.28e-3	0.725	11.343	9.616e-3	4.466e-2	0.344	10.574	1.666e-2	7.341e-2	-0.183	11.305	9.02e-2	1.864e-1	0.198	11.870	2.208e-1	3.618e-1	-0.381	11.687	3.506e-2	1.488e-1
	AAL94920.1| Flavodoxin																							
**FN0725**	0.639	7.469	1.276e-1	3.087e-1	1.581	8.595	3.629e-3	1.428e-2	0.016	6.642	1.604e-1	9.67e-1	-0.623	7.485	1.33e-1	3.392e-1	0.942	9.234	8.216e-2	5.711e-2	-1.565	8.611	3.401e-3	8.996e-3
	AAL94921.1| Molybdopterin biosynthesis MoeB protein																							
**FN0728**	0.390	10.411	3.142e-3	2.396e-3	0.434	10.640	5.332e-4	1.337e-3	0.298	10.116	9.09e-4	2.113e-3	-0.092	10.709	2.338e-2	2.648e-2	0.044	11.030	2.072e-2	4.912e-3	-0.136	10.938	2.482e-3	6.088e-3
	AAL94924.1| Hypothetical protein																							
**FN0729**	-0.595	15.069	5.641e-2	1.06e-1	-1.268	14.581	6.792e-5	9.439e-5	-0.521	14.939	6.743e-4	1.462e-3	0.074	14.548	2.571e-1	8.297e-1	-0.672	13.985	1.529e-1	1.974e-1	0.747	14.060	2.999e-3	7.674e-3
	AAL94925.1| Phosphoglycerate mutase																							
**FN0731**	0.969	9.971	6.08e-2	1.182e-1	-0.446	8.740	2.745e-2	1.451e-1	-0.059	8.739	7.61e-2	4.131e-1	-1.028	9.912	6.179e-2	1.091e-1	-1.416	9.709	8.692e-2	6.439e-2	0.387	8.681	4.482e-2	1.97e-1
	AAL94927.1| Hypothetical protein																							
**FN0733**	-0.641	13.087	1.256e-2	1.49e-2	-1.280	12.632	1.505e-5	1.085e-5	0.483	14.007	3.863e-5	2.793e-5	1.123	13.570	3.011e-3	1.233e-3	-0.640	11.992	7.913e-2	5.28e-2	1.763	13.115	3.459e-6	6.111e-7
	AAL94929.1| Peptidase T																							
**FN0734**					-1.617	5.582	5.426e-5	6.813e-5																
	AAL94930.1| Fe-S oxidoreductase																							
**FN0735**	0.018	13.715	3.04e-1	9.736e-1	-1.818	12.063	7.361e-6	3.616e-6	-0.054	13.439	1.183e-1	6.795e-1	-0.072	13.661	2.727e-1	8.987e-1	-1.836	12.081	1.241e-1	1.275e-1	1.765	12.009	7.314e-4	1.272e-3
	AAL94931.1| Cell surface protein																							
**FN0736**													0.349	5.716	1.385e-1	3.605e-1	1.465	7.017	7.327e-3	1.095e-3	-1.116	7.366	1.799e-3	4.056e-3
	AAL94932.1| Methyltransferase																							
**FN0737**													-0.908	8.707										
	AAL94933.1| Hypothetical protein																							
**FN0738**	-0.806	11.458	7.814e-4	3.119e-4	-0.943	11.505	2.79e-3	1.024e-2	-1.700	10.360	5.852e-5	5.076e-5	-0.894	9.757	9.526e-3	6.993e-3	-0.137	10.699	3.267e-1	6.335e-1	-0.757	9.805	2.537e-2	1.032e-1
	AAL94934.1| Hypothetical exported 24-amino acid repeat protein																							
**FN0739**	-0.513	15.451	1.985e-3	1.275e-3	-0.733	15.416	5.84e-4	1.501e-3	0.591	16.351	9.206e-5	9.607e-5	1.104	16.041	5.335e-4	9.82e-5	-0.219	14.903	1.404e-1	1.64e-1	1.323	16.007	3.31e-5	1.872e-5
	AAL94935.1| Formiminotetrahydrofolate cyclodeaminase																							
**FN0740**	-0.223	17.608	2.153e-1	6.116e-1	0.434	18.450	1.528e-2	7.616e-2	-0.616	17.012	1.28e-4	1.542e-4	-0.393	16.992	1.717e-1	4.807e-1	0.658	18.227	1.348e-1	1.506e-1	-1.050	17.835	4.295e-3	1.228e-2
	AAL94936.1| Imidazolonepropionase																							
**FN0741**	-0.254	18.286	1.884e-1	5.171e-1	-0.038	18.686	1.211e-1	7.32e-1	0.288	18.624	2.784e-3	9.005e-3	0.542	18.574	8.821e-2	1.802e-1	0.216	18.432	3.115e-1	5.908e-1	0.326	18.975	7.731e-3	2.584e-2
	AAL94937.1| Glutamate formiminotransferase																							
**FN0742**	-0.400	12.066			-0.576	12.074	7.048e-3	3.095e-2	-0.460	11.802	2.817e-2	1.334e-1	-0.060	11.605			-0.176	11.674			0.116	11.614	1.398e-1	7.029e-1
	AAL94938.1| unknown																							
**FN0743**					-1.291	5.907	1.824e-4	3.424e-4	0.211	7.022	5.456e-3	2.028e-2									1.503	6.119	4.719e-5	3.226e-5
	AAL94939.1| ATP-dependent helicase, DinG family																							

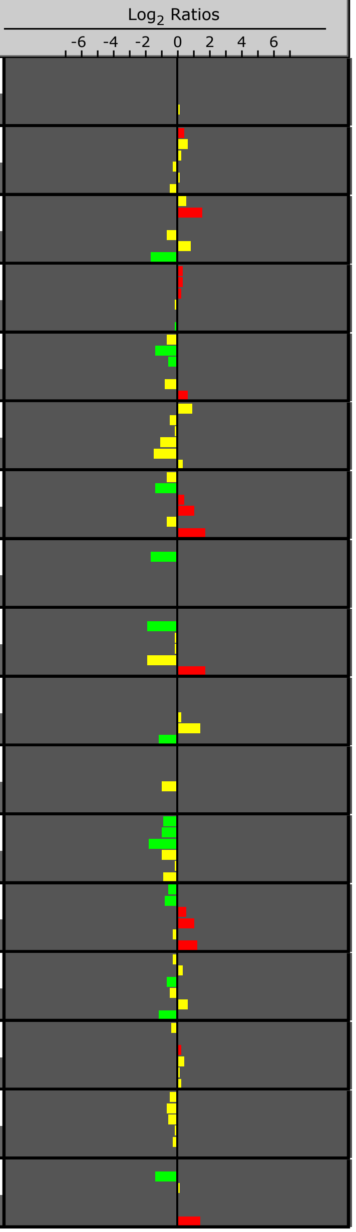

Show detected proteins only
Show all proteins
Filter by category:
GO: amino acid transport

Proteins found: 1424

Enter (or paste) list of ORFs
Find ORFs

Test	Cutoff
q-Value	.005
p-Value	

Dot Plots
Dot Plots

	Signif	Direction	Applies To
(red)	yes	+	ratios, bars
(yellow)	no	n/a	bars
(green)	yes	–	ratios, bars
(pink)	yes	+	*p*- , *q*-Values
(light green)	yes	–	

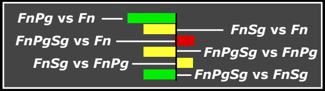

ORF	FnPg vs Fn				FnSg vs Fn				FnPgSg vs Fn				FnPgSg vs FnPg				FnSg vs FnPg				FnPgSg vs FnSg			
	Ratio	Sum	*q*-Val	*p*-Val	Ratio	Sum	*q*-Val	*p*-Val	Ratio	Sum	*q*-Val	*p*-Val	Ratio	Sum	*q*-Val	*p*-Val	Ratio	Sum	*q*-Val	*p*-Val	Ratio	Sum	*q*-Val	*p*-Val
**FN0745** AAL94941.1| metal dependent phosphohydrolase	-0.804	14.359	2.971e-2	4.461e-2	-0.104	15.243	1.099e-1	6.559e-1	-0.765	14.194	3.351e-3	1.126e-2	0.039	13.594	2.765e-1	9.162e-1	0.700	14.439	1.084e-1	9.875e-2	-0.661	14.479	1.661e-2	6.357e-2
**FN0746** AAL94942.1| Hypothetical Metal-Binding Protein	0.470	7.371			0.239	7.325											-0.231	7.795	3.577e-1	7.271e-1				
**FN0749** AAL94945.1| Hypothetical protein									-0.864	5.579														
**FN0750** AAL94946.1| Hypothetical protein	-0.814	9.463	6.976e-3	7.184e-3	0.682	11.143	3.125e-5	3.036e-5	-1.869	8.204	2.312e-4	3.598e-4	-1.055	7.594	2.892e-2	3.608e-2	1.496	10.329	3.169e-3	2.864e-4	-2.551	9.274	5.349e-5	3.806e-5
**FN0751** AAL94947.1| L-asparaginase I	-0.553	8.549	4.505e-2	7.772e-2	0.312	9.598	2.544e-2	1.334e-1	-0.623	8.276	2.707e-3	8.698e-3	-0.070	7.927	2.538e-1	8.153e-1	0.864	9.045	4.183e-2	1.629e-2	-0.934	8.976	4.039e-3	1.129e-2
**FN0752** AAL94948.1| Proline iminopeptidase	-0.408	8.171	1.659e-1	4.341e-1	0.104	8.868	3.433e-2	1.859e-1	-1.588	6.788	1.331e-4	1.632e-4	-1.180	6.584	1.005e-1	2.212e-1	0.512	8.460	2.058e-1	3.26e-1	-1.692	7.280	3.767e-5	2.284e-5
**FN0753** AAL94949.1| Glutamyl-tRNA(Gln) amidotransferase subunit B	0.623	12.598	6.089e-3	5.94e-3	-0.116	12.044	8.057e-2	4.656e-1	1.007	12.778	5.188e-4	1.047e-3	0.384	13.604	4.261e-4	6.832e-5	-0.738	12.667	7.267e-4	1.671e-5	1.122	13.051	3.642e-7	3.4e-8
**FN0754** AAL94950.1| Glutamyl-tRNA(Gln) amidotransferase subunit A	-0.166	13.764	1.542e-1	3.937e-1	-0.420	13.694	9.565e-3	4.437e-2	-0.034	13.692	1.354e-1	7.925e-1	0.132	13.730	1.904e-1	5.553e-1	-0.254	13.528	2.054e-1	3.252e-1	0.386	13.660	2.115e-2	8.418e-2
**FN0755** AAL94951.1| Glutamyl-tRNA(Gln) amidotransferase subunit C	-0.990	10.984	4.575e-3	4.019e-3	-1.054	11.105	7.568e-4	2.122e-3	-0.741	11.029	1.173e-3	2.969e-3	0.249	10.243	6.245e-2	1.106e-1	-0.064	10.115	2.386e-1	4.074e-1	0.313	10.364	1.69e-2	6.487e-2
**FN0758** AAL94954.1| Rod shape-determining protein mreB	-0.636	15.014	6.197e-4	2.131e-4	-0.123	15.712	8.126e-2	4.7e-1	-0.951	14.496	1.389e-4	1.738e-4	-0.315	14.064	2.591e-2	3.069e-2	0.513	15.076	8.518e-2	6.162e-2	-0.828	14.761	7.804e-3	2.615e-2
**FN0761** AAL94957.1| Bvg accessory factor	1.146	9.350			0.070	8.459	1.305e-1	7.978e-1	-0.342	7.659	4.446e-2	2.267e-1	-1.488	9.008			-1.076	9.604			-0.412	8.116	3.712e-2	1.588e-1
**FN0765** AAL94961.1| tRNA (5-methylaminomethyl-2-thiouridylate) -methyltransferase	-0.706	6.321			-2.020	5.190			-1.411	5.411			-0.705	4.909			-1.315	4.485			0.609	3.779		
**FN0766** AAL94962.1| Large-conductance mechanosensitive channel									0.488	8.871														
**FN0768** AAL94964.1| Hemin receptor	-1.501	5.501							-1.399	5.399			0.102	4.102										
**FN0771** AAL94967.1| Oxygen-independent coproporphyrinogen III oxidase					-1.844	6.738																		
**FN0772** AAL94968.1| Flavodoxin																								
**FN0774** AAL94970.1| Hypothetical cytosolic protein	-1.485	13.578	9.811e-4	4.556e-4	-3.087	12.161	1.752e-5	1.35e-5	-0.436	14.423	9.202e-3	3.744e-2	1.049	13.142	9.962e-3	7.501e-3	-1.602	10.676	4.351e-2	1.752e-2	2.651	11.725	1.671e-3	3.706e-3

Log$_2$ Ratios

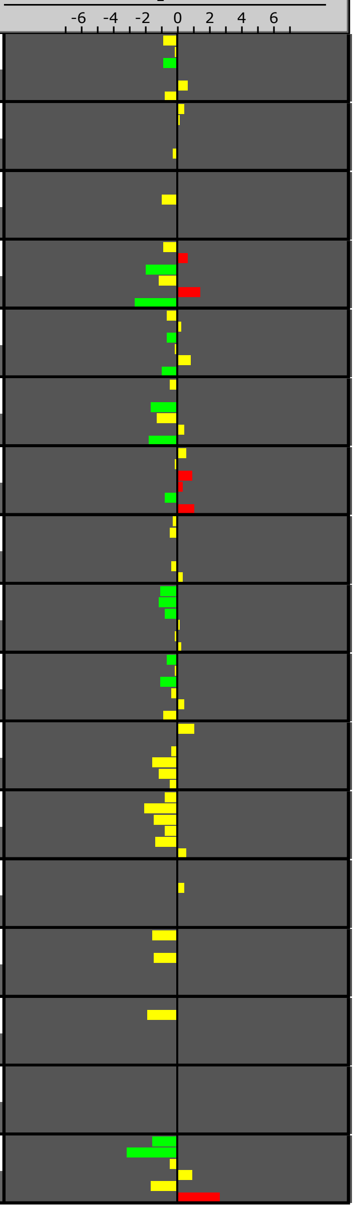

Show detected proteins only
Show all proteins
Filter by category:
GO: amino acid transport

Proteins found: 1424

Enter (or paste) list of ORFs

Find ORFs

Test	Cutoff
q-Value	.005
p-Value	

Dot Plots
Dot Plots

	Signif	Direction	Applies To
(red)	yes	+	ratios, bars
(yellow)	no	n/a	bars
(green)	yes	–	ratios, bars
(pink)	yes	+	*p*- , *q*-Values
(light green)	yes	–	

FnPg vs Fn
FnPgSg vs Fn
FnSg vs FnPg
FnSg vs Fn
FnPgSg vs FnPg
FnPgSg vs FnSg

ORF	Description	FnPg vs Fn Ratio	FnPg vs Fn Sum	FnPg vs Fn *q*-Val	FnPg vs Fn *p*-Val	FnSg vs Fn Ratio	FnSg vs Fn Sum	FnSg vs Fn *q*-Val	FnSg vs Fn *p*-Val	FnPgSg vs Fn Ratio	FnPgSg vs Fn Sum	FnPgSg vs Fn *q*-Val	FnPgSg vs Fn *p*-Val	FnPgSg vs FnPg Ratio	FnPgSg vs FnPg Sum	FnPgSg vs FnPg *q*-Val	FnPgSg vs FnPg *p*-Val	FnSg vs FnPg Ratio	FnSg vs FnPg Sum	FnSg vs FnPg *q*-Val	FnSg vs FnPg *p*-Val	FnPgSg vs FnSg Ratio	FnPgSg vs FnSg Sum	FnPgSg vs FnSg *q*-Val	FnPgSg vs FnSg *p*-Val
**FN0775**	AAL94971.1| Aspartyl aminopeptidase	-0.365	16.737	1.277e-1	3.09e-1	-0.491	16.796	1.102e-4	1.771e-4	-0.699	16.200	1.849e-6	3.037e-7	-0.334	16.039	1.62e-1	4.443e-1	-0.126	16.431	3.637e-1	7.462e-1	-0.208	16.097	4.753e-3	1.406e-2
**FN0776**	AAL94972.1| Aspartate-ammonia ligase	-0.544	12.818	9.877e-3	1.119e-2	0.001	13.548	1.573e-1	9.918e-1	-0.808	12.351	1.605e-3	4.605e-3	-0.264	12.011	1.524e-2	1.451e-2	0.545	13.004	2.901e-3	2.384e-4	-0.809	12.740	1.938e-5	8.774e-6
**FN0777**	AAL94973.1| GTP-binding protein lepA	-0.718	11.661	1.643e-2	2.09e-2	0.035	12.600	1.364e-1	8.395e-1	0.357	12.533	1.327e-2	5.67e-2	1.076	12.019	1.108e-4	1.037e-5	0.754	11.881	1.469e-2	2.998e-3	0.322	12.957	6.432e-3	2.06e-2
**FN0778**	AAL94974.1| Methyltransferase	-1.013	9.000	3.391e-2	5.311e-2	-0.442	9.755	5.398e-4	1.358e-3	-1.087	8.721			-0.074	7.912			0.571	8.742	1.696e-1	2.373e-1	-0.645	8.667		
**FN0779**	AAL94975.1| Putative GTPases (G3E family)																	0.819	5.648						
**FN0783**	AAL94979.1| acyl-CoA dehydrogenase	0.114	23.085	1.388e-1	3.433e-1	-0.196	22.959	1.153e-2	5.503e-2	0.931	23.698	4.028e-3	1.405e-2	0.817	24.016	1.433e-2	1.31e-2	-0.310	23.073	8.181e-2	5.661e-2	1.127	23.890	3.135e-3	8.112e-3
**FN0784**	AAL94980.1| Electron transfer flavoprotein beta-subunit	-0.300	22.413	8.082e-2	1.723e-1	-0.325	22.572	7.081e-3	3.113e-2	-0.082	22.427	9.113e-2	5.064e-1	0.218	22.331	1.348e-1	3.467e-1	-0.025	22.273	4.104e-1	9.089e-1	0.243	22.490	3.765e-2	1.615e-1
**FN0785**	AAL94981.1| Electron transfer flavoprotein alpha-subunit	0.019	21.833	2.747e-1	8.446e-1	-0.067	21.932	1.008e-1	5.956e-1	-0.348	21.263	1.182e-3	2.999e-3	-0.366	21.485	1.978e-2	2.089e-2	-0.085	21.951	2.939e-1	5.434e-1	-0.281	21.584	2.38e-2	9.609e-2
**FN0788**	AAL94984.1| unknown	0.597	9.459	5.714e-3	5.441e-3	0.484	9.531	9.059e-3	4.156e-2	0.016	8.675	1.563e-1	9.377e-1	-0.581	9.475	1.342e-2	1.181e-2	-0.113	10.128	2.482e-1	4.307e-1	-0.468	9.547	1.429e-2	5.336e-2
**FN0790**	AAL94986.1| Xylose repressor	-1.542	6.739	1.755e-3	1.065e-3	-0.605	7.860	1.985e-5	1.632e-5	-1.717	6.361			-0.174	5.022			0.937	6.318	3.515e-2	1.213e-2	-1.111	6.143		
**FN0791**	AAL94987.1| Histidine ammonia-lyase	-0.142	18.534	1.267e-1	3.059e-1	-0.247	18.614	2.21e-2	1.143e-1	-0.404	18.068	1.095e-3	2.699e-3	-0.262	18.129	7.291e-2	1.367e-1	-0.105	18.471	3.015e-1	5.636e-1	-0.157	18.209	6.807e-2	3.146e-1
**FN0792**	AAL94988.1| Urocanate hydratase	0.041	20.440	2.873e-1	8.985e-1	0.238	20.822	5.849e-4	1.504e-3	-0.341	19.854	2.672e-4	4.339e-4	-0.382	20.099	1.319e-1	3.348e-1	0.197	20.863	2.865e-1	5.241e-1	-0.579	20.481	3.913e-5	2.429e-5
**FN0793**	AAL94989.1| Sodium/glutamate symport carrier protein	-0.749	7.919							0.585	9.049			1.334	8.504										
**FN0794**	AAL94990.1| Hypothetical protein	0.570	7.244			1.117	7.976	1.338e-4	2.299e-4	-0.428	6.043			-0.998	6.816			0.547	8.545			-1.545	7.548		
**FN0796**	AAL94992.1| Pyruvate,phosphate dikinase	-0.165	15.072	1.399e-1	3.468e-1	-0.217	15.204	1.154e-3	3.536e-3	-0.552	14.481	1.323e-4	1.618e-4	-0.387	14.519	6.494e-2	1.166e-1	-0.052	15.039	3.704e-1	7.68e-1	-0.335	14.652	5.547e-4	8.644e-4
**FN0798**	AAL94994.1| Fructose-1,6-bisphosphatase	-0.232	7.274	2.163e-1	6.154e-1	1.292	8.982	2.66e-2	1.401e-1	0.280	7.582	1.017e-1	5.729e-1	0.512	7.554	1.03e-1	2.302e-1	1.523	8.750	1.185e-1	1.167e-1	-1.012	9.262	4.355e-2	1.907e-1
**FN0799**	AAL94995.1| Isoamylase	0.417	8.365	1.651e-1	4.315e-1	0.442	8.575	8.966e-3	4.105e-2	-0.172	7.573			-0.589	8.193			0.025	8.992	4.223e-1	9.542e-1	-0.614	8.403		

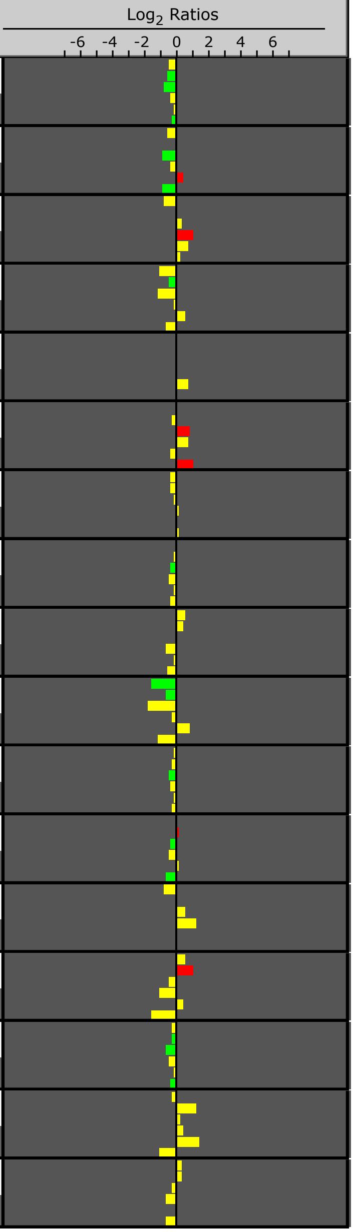

Show detected proteins only
Show all proteins
Filter by category:
GO: amino acid transport

Proteins found: 1424

Enter (or paste) list of ORFs

Find ORFs

Test	Cutoff
***q*-Value**	.005
p-Value	

Dot Plots
Dot Plots

	Signif	Direction	Applies To
(red)	yes	+	ratios, bars
(yellow)	no	n/a	bars
(green)	yes	–	ratios, bars
(pink)	yes	+	*p*- , *q*-Values
(light green)	yes	–	*p*- , *q*-Values

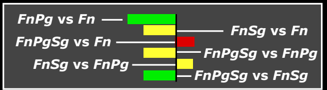

ORF	Protein	*FnPg* vs *Fn* Ratio	Sum	*q*-Val	*p*-Val	*FnSg* vs *Fn* Ratio	Sum	*q*-Val	*p*-Val	*FnPgSg* vs *Fn* Ratio	Sum	*q*-Val	*p*-Val	*FnPgSg* vs *FnPg* Ratio	Sum	*q*-Val	*p*-Val	*FnSg* vs *FnPg* Ratio	Sum	*q*-Val	*p*-Val	*FnPgSg* vs *FnSg* Ratio	Sum	*q*-Val	*p*-Val
FN0800	AAL94996.1| Amino acid-binding protein	-0.203	10.414	2.558e-1	7.663e-1	0.133	10.934	4.685e-2	2.599e-1	1.398	11.810	1.159e-3	2.917e-3	1.601	11.811	1.504e-2	1.419e-2	0.336	10.731	3.194e-1	6.127e-1	1.264	12.332	1.287e-3	2.673e-3
FN0801	AAL94997.1| Amino acid transport ATP-binding protein	0.663	7.677			0.906	8.105	6.488e-6	2.993e-6									0.243	8.768						
FN0802	AAL94998.1| Amino acid transport system permease protein																								
FN0803	AAL94999.1| Cytochrome C-TYPE biogenesis protein ccdA	-1.108	12.619	9.426e-3	1.057e-2	-1.679	12.233	1.802e-3	6.016e-3	-1.139	12.385	3.573e-3	1.215e-2	-0.030	11.480	2.651e-1	8.648e-1	-0.571	11.124	6.377e-2	3.49e-2	0.540	11.094	4.987e-3	1.498e-2
FN0805	AAL95001.1| Hypothetical protein	1.098	6.525			0.966	6.577			-0.640	4.583			-1.738	5.884	1.516e-3	4.597e-4	-0.132	7.675	1.304e-1	1.407e-1	-1.606	5.937	1.328e-4	1.252e-4
FN0806	AAL95002.1| SpoIID homolog	-0.933	12.992	3.472e-3	2.747e-3	-0.810	13.300	1.075e-3	3.245e-3	-0.680	13.041	1.185e-3	3.01e-3	0.253	12.312	5.498e-2	9.234e-2	0.123	12.367	1.288e-1	1.374e-1	0.130	12.620	6.766e-2	3.125e-1
FN0807	AAL95003.1| 3-deoxy-manno-octulosonate cytidylyltransferase	-0.123	10.335	2.081e-1	5.857e-1	-1.467	9.175	3.587e-3	1.407e-2	-0.330	9.925	4.398e-2	2.24e-1	-0.207	10.006	2.849e-2	3.526e-2	-1.344	9.052	1.066e-3	3.371e-5	1.138	8.846	1.433e-4	1.392e-4
FN0808	AAL95004.1| Phosphoglycerate mutase	0.194	12.115	7.773e-2	1.635e-1	0.309	12.415	3.683e-3	1.456e-2	-0.392	11.325	4.92e-3	1.79e-2	-0.586	11.723	1.062e-2	8.316e-3	0.115	12.609	2.173e-1	3.531e-1	-0.701	12.023	5.939e-4	9.545e-4
FN0809	AAL95005.1| 23S rRNA methyltransferase	-1.688	7.688			-0.780	8.780			-1.127	8.045			0.561	6.561			0.908	7.092			-0.346	7.654		
FN0810	AAL95006.1| Low-specificity threonine aldolase	-0.040	11.620	2.449e-1	7.231e-1	0.027	11.872	1.541e-1	9.681e-1	-1.644	9.813	1.044e-4	1.167e-4	-1.603	9.977	3.696e-3	1.683e-3	0.068	11.832	4.141e-1	9.228e-1	-1.671	10.229	4.747e-2	2.101e-1
FN0811	AAL95007.1| Hypothetical protein																								
FN0813	AAL95009.1| Transcriptional regulator, TetR family	1.746	8.898			2.271	9.608	2.515e-6	6.469e-7	-0.317	6.632	9.265e-2	5.159e-1	-2.063	8.581			0.526	11.354			-2.588	9.291	1.507e-4	1.494e-4
FN0814	AAL95010.1| Propionate CoA-transferase	-3.939	13.406	4.364e-6	2.23e-7	-4.936	12.593	2.075e-5	1.732e-5	-0.729	16.413	3.378e-5	2.113e-5	3.210	12.677	1.028e-4	8.812e-6	-0.998	8.654	1.97e-1	3.062e-1	4.207	11.864	3.637e-5	2.161e-5
FN0815	AAL95011.1| Propionate permease									0.171	11.483	1.807e-2	8.055e-2												
FN0816	AAL95012.1| dehydrogenase with MaoC-like domain	-2.404	13.956							-1.830	14.326	1.691e-5	6.994e-6	0.574	12.126										
FN0818	AAL95014.1| DNA-binding protein HU	-0.807	19.104	2.353e-2	3.323e-2	-2.046	18.049	3.655e-5	3.81e-5	-0.030	19.677	1.22e-1	7.032e-1	0.777	19.073	3.084e-2	3.959e-2	-1.239	17.242	9.136e-2	7.177e-2	2.016	18.019	2.237e-5	1.089e-5
FN0819	AAL95015.1| Tetratricopeptide repeat family protein	-0.455	11.840	4.324e-2	7.337e-2	-0.439	12.040	2.865e-3	1.059e-2	-0.003	12.089	1.633e-1	9.878e-1	0.453	11.837	6.012e-2	1.051e-1	0.016	11.585	4.201e-1	9.459e-1	0.437	12.038	1.701e-2	6.535e-2

Log$_2$ Ratios

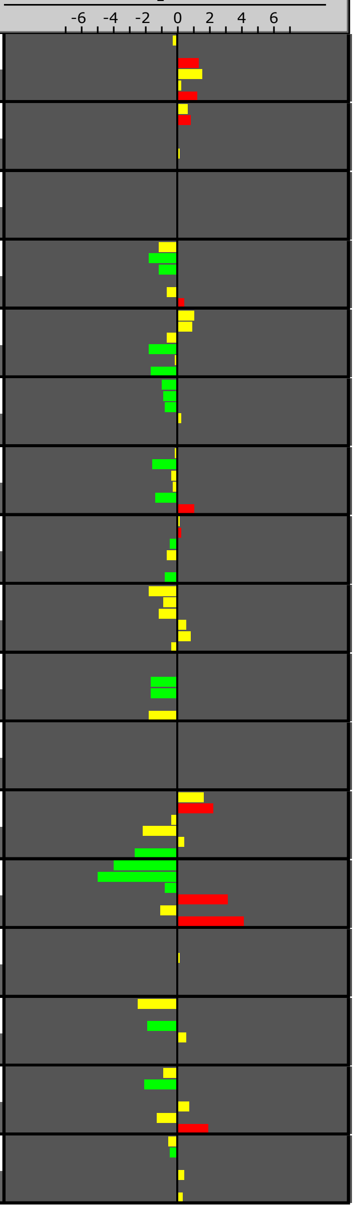

Show detected proteins only
Show all proteins
Filter by category:
GO: amino acid transport

Proteins found: 1424

Enter (or paste) list of ORFs

Find ORFs

Test	Cutoff
q-Value	.005
p-Value	

Dot Plots
Dot Plots

	Signif	Direction	Applies To
(red)	yes	+	ratios, bars
(yellow)	no	n/a	bars
(green)	yes	–	ratios, bars
(pink)	yes	+	*p*- , *q*-Values
(light green)	yes	–	*p*- , *q*-Values

FnPg vs Fn
FnPgSg vs Fn
FnSg vs FnPg
FnSg vs Fn
FnPgSg vs FnPg
FnPgSg vs FnSg

ORF	Protein	*FnPg vs Fn* Ratio	Sum	*q*-Val	*p*-Val	*FnSg vs Fn* Ratio	Sum	*q*-Val	*p*-Val	*FnPgSg vs Fn* Ratio	Sum	*q*-Val	*p*-Val	*FnPgSg vs FnPg* Ratio	Sum	*q*-Val	*p*-Val	*FnSg vs FnPg* Ratio	Sum	*q*-Val	*p*-Val	*FnPgSg vs FnSg* Ratio	Sum	*q*-Val	*p*-Val
FN0820	AAL95016.1| Mercuric reductase	-0.033	14.729	2.787e-1	8.615e-1	-0.261	14.685	3.42e-2	1.851e-1	-0.244	14.313	7.001e-3	2.717e-2	-0.211	14.484	1.36e-1	3.515e-1	-0.228	14.652	2.305e-1	3.862e-1	0.017	14.440	1.768e-1	9.29e-1
FN0821	AAL95017.1| Hypothetical protein	-0.301	13.129	1.883e-1	5.166e-1	-1.906	11.708	3.531e-5	3.619e-5	-1.142	12.084	2.269e-4	3.499e-4	-0.841	11.986	1.092e-1	2.534e-1	-1.605	11.407	1.204e-1	1.202e-1	0.764	10.566	4.059e-3	1.136e-2
FN0823	AAL95019.1| GTP-binding protein hflX	1.149	9.558	2.902e-2	4.329e-2	2.457	11.051	2.501e-6	6.407e-7	0.170	8.375	8.063e-2	4.41e-1	-0.979	9.728	4.119e-2	5.994e-2	1.308	12.200	8.822e-3	1.439e-3	-2.287	11.221	8.633e-6	2.239e-6
FN0824	AAL95020.1| hypothetical cytosolic protein																								
FN0825	AAL95021.1| Hypothetical cytosolic protein	-1.068	12.189	1.567e-2	1.965e-2	-1.849	11.592	1.905e-6	4.211e-7	-1.529	11.525	7.45e-5	7.091e-5	-0.461	10.661	1.347e-1	3.462e-1	-0.782	10.524	1.472e-1	1.822e-1	0.321	10.063	2.875e-2	1.187e-1
FN0826	AAL95022.1| periplasmic component of efflux system	-1.809	9.442	4.137e-4	1.131e-4	-1.468	9.967	3.602e-4	8.111e-4	-0.526	10.521	1.786e-3	5.264e-3	1.282	8.916	4.308e-3	2.06e-3	0.340	8.158	2.634e-1	4.668e-1	0.942	9.440	4.821e-3	1.433e-2
FN0827	AAL95023.1| ABC transporter ATP-binding protein	-2.166	9.449	9.592e-3	1.08e-2	-0.594	11.207	1.769e-2	8.952e-2	-1.579	9.833	4.789e-3	1.733e-2	0.587	7.870	6.144e-3	3.415e-3	1.573	9.040	6.343e-3	8.378e-4	-0.986	9.627	1.874e-3	4.262e-3
FN0828	AAL95024.1| ABC transporter permease protein					-1.864	6.988	2.085e-4	4.025e-4	-1.647	6.817											0.217	5.341		
FN0830	AAL95026.1| Hypothetical protein	1.059	11.207	1.03e-3	4.918e-4	1.224	11.557	1.181e-4	1.94e-4	-1.340	8.606	4.172e-3	1.467e-2	-2.398	9.868	1.125e-3	2.942e-4	0.165	12.616	1.271e-1	1.338e-1	-2.563	10.218	3.223e-4	4.247e-4
FN0831	AAL95027.1| Hemin receptor																								
FN0832	AAL95028.1| Hypothetical protein	0.110	12.638	2.816e-1	8.741e-1	-1.488	11.225	9.803e-5	1.524e-4	0.226	12.551	1.779e-2	7.913e-2	0.117	12.865	2.631e-1	8.56e-1	-1.597	11.335	1.593e-1	2.126e-1	1.714	11.452	1.427e-4	1.383e-4
FN0833	AAL95029.1| Hypothetical protein	-3.561	8.205			-1.099	10.852	1.368e-3	4.349e-3	-2.045	9.517	7.314e-4	1.613e-3	1.516	6.160			2.462	7.291			-0.946	8.807	1.439e-2	5.376e-2
FN0834	AAL95030.1| Hypothetical Exported Protein	-0.508	10.209	1.577e-1	4.055e-1	-1.783	9.118	2.469e-5	2.207e-5	-0.963	9.550	1.062e-5	3.442e-6	-0.455	9.246	1.962e-1	5.8e-1	-1.275	8.610	1.84e-1	2.724e-1	0.820	8.155	1.883e-3	4.288e-3
FN0835	AAL95031.1| Hypothetical protein																								
FN0836	AAL95032.1| Hypothetical protein	1.061	8.887	5.62e-2	1.054e-1	-0.069	7.941	9.826e-2	5.79e-1	0.384	8.006	7.241e-2	3.906e-1	-0.677	9.270	1.011e-1	2.233e-1	-1.130	9.002	1.078e-1	9.769e-2	0.453	8.325	7.05e-2	3.273e-1
FN0837	AAL95033.1| Integrase/recombinase																								
FN0846	AAL95042.1| Hypothetical Exported Protein	0.094	13.359	1.674e-1	4.394e-1	-0.255	13.195	1.815e-2	9.21e-2	-1.032	12.029	1.323e-3	3.524e-3	-1.126	12.326	1.663e-3	5.269e-4	-0.348	13.288	3.225e-2	1.035e-2	-0.778	12.163	1.024e-3	2e-3

Log$_2$ Ratios: -6 -4 -2 0 2 4 6

Show detected proteins only
Show all proteins
Filter by category:
GO: amino acid transport

Proteins found: 1424

Enter (or paste) list of ORFs

Find ORFs

Test	Cutoff
q-Value	.005
p-Value	

Dot Plots
Dot Plots

	Signif	Direction	Applies To
red	yes	+	ratios, bars
yellow	no	n/a	bars
green	yes	–	ratios, bars
pink	yes	+	*p*- , *q*-Values
light green	yes	–	*p*- , *q*-Values

FnPg vs Fn — FnSg vs Fn
FnPgSg vs Fn — FnPgSg vs FnPg
FnSg vs FnPg — FnPgSg vs FnSg

ORF	Description	*FnPg vs Fn* Ratio	Sum	*q*-Val	*p*-Val	*FnSg vs Fn* Ratio	Sum	*q*-Val	*p*-Val	*FnPgSg vs Fn* Ratio	Sum	*q*-Val	*p*-Val	*FnPgSg vs FnPg* Ratio	Sum	*q*-Val	*p*-Val	*FnSg vs FnPg* Ratio	Sum	*q*-Val	*p*-Val	*FnPgSg vs FnSg* Ratio	Sum	*q*-Val	*p*-Val
FN0847	AAL95043.1| TPR-repeat-containing proteins	-1.659	7.659			-1.184	8.319	7.413e-5	1.081e-4	-1.892	7.222	1.49e-5	5.662e-6	-0.233	5.767			0.475	6.660			-0.708	6.427	4.613e-3	1.353e-2
FN0848	AAL95044.1| Hypothetical protein																								
FN0849	AAL95045.1| 8-amino-7-oxononanoate synthase	0.764	11.033	3.726e-3	3.018e-3	0.565	11.018	1.443e-3	4.635e-3	-0.787	9.278	3.527e-3	1.196e-2	-1.551	10.246	3.315e-6	7.371e-8	-0.199	11.782	7.91e-2	5.276e-2	-1.352	10.231	4.431e-4	6.356e-4
FN0850	AAL95046.1| Hypothetical cytosolic protein	0.248	9.184	1.255e-1	3.022e-1	1.012	10.133	2.691e-5	2.476e-5	-1.330	7.402	1.025e-4	1.134e-4	-1.578	7.854	1.143e-2	9.277e-3	0.764	10.380	3.355e-2	1.111e-2	-2.342	8.803	3.762e-5	2.279e-5
FN0853	AAL95049.1| Glycogen synthase	0.961	12.611	6.882e-3	7.045e-3	0.915	12.750	1.01e-3	3.013e-3	-0.782	10.665	6.092e-6	1.522e-6	-1.742	11.830	4.891e-3	2.447e-3	-0.045	13.711	3.647e-1	7.496e-1	-1.697	11.969	6.011e-4	9.718e-4
FN0854	AAL95050.1| Glucose-1-phosphate adenylyltransferase	1.630	13.097	4.36e-3	3.751e-3	1.493	13.145	4.839e-5	5.831e-5	0.226	11.489	3.804e-2	1.898e-1	-1.404	13.323	6.587e-3	3.811e-3	-0.137	14.775	2.379e-1	4.056e-1	-1.267	13.371	5.758e-5	4.202e-5
FN0855	AAL95051.1| Glucose-1-phosphate adenylyltransferase	0.488	14.558	6.521e-2	1.302e-1	1.104	15.358	5.2e-5	6.425e-5	-0.839	13.027	4.245e-7	3.817e-8	-1.327	13.719	2.082e-2	2.247e-2	0.616	15.846	5.671e-2	2.86e-2	-1.942	14.519	3.09e-5	1.691e-5
FN0856	AAL95052.1| 1,4-alpha-glucan branching enzyme	1.224	13.568	1.528e-4	2.598e-5	0.971	13.500	1.32e-4	2.256e-4	-0.627	11.514	7.566e-3	2.973e-2	-1.851	12.942	1.324e-3	3.785e-4	-0.253	14.724	4.334e-2	1.739e-2	-1.598	12.873	7.824e-5	6.253e-5
FN0857	AAL95053.1| Glycogen phosphorylase	0.317	15.822	9.16e-2	2.026e-1	0.743	16.433	5.978e-5	7.818e-5	-0.346	14.955	3.947e-3	1.37e-2	-0.663	15.476	3.839e-2	5.435e-2	0.426	16.750	8.631e-2	6.341e-2	-1.089	16.087	3.235e-6	5.507e-7
FN0858	AAL95054.1| 4-alpha-glucanotransferase	1.039	10.442	1.199e-3	6.356e-4	1.165	10.752	1.401e-2	6.904e-2	-2.182	7.017	4.324e-4	8.285e-4	-3.221	8.261	6.376e-4	1.248e-4	0.125	11.791	3.54e-1	7.155e-1	-3.346	8.570	6.341e-3	2.025e-2
FN0865	AAL95061.1| unknown	2.292	12.857	9.565e-2	2.144e-1	2.373	13.122	4.153e-6	1.431e-6	-0.763	9.598	1.47e-2	6.369e-2	-3.055	12.095	8.846e-2	1.809e-1	0.081	15.415	4.125e-1	9.168e-1	-3.136	12.360	3.176e-6	5.356e-7
FN0867	AAL95063.1| Long-chain-fatty-acid--CoA ligase	-2.181	12.264	1.015e-4	1.38e-5	-1.510	13.119	1.533e-6	3.026e-7	-0.731	13.510	4.801e-5	3.853e-5	1.450	11.533	1.722e-3	5.555e-4	0.670	10.938	2.326e-2	5.917e-3	0.779	12.388	2.152e-4	2.424e-4
FN0868	AAL95064.1| ATPases of the PP superfamily					-0.117	6.117																		
FN0869	AAL95065.1| Hydrolase (HAD superfamily)	-0.713	4.713			0.239	5.851			-1.027	4.197			-0.313	3.687			0.953	5.137			-1.266	4.824		
FN0870	AAL95066.1| Rhodanese-related sulfurtransferases									0.817	6.040														
FN0871	AAL95067.1| 3-dehydroquinate synthase	0.193	7.807			-0.520	7.278	2.177e-2	1.125e-1	-0.535	6.875			-0.728	7.272			-0.713	7.471			-0.015	6.743		
FN0873	AAL95069.1| Protease IV	1.534	10.390	7.542e-2	1.571e-1	1.275	10.316	2.23e-3	7.777e-3	-0.043	8.609	1.209e-1	6.963e-1	-1.577	10.347	7.887e-2	1.527e-1	-0.259	11.850	3.409e-1	6.754e-1	-1.318	10.273	3.073e-3	7.913e-3

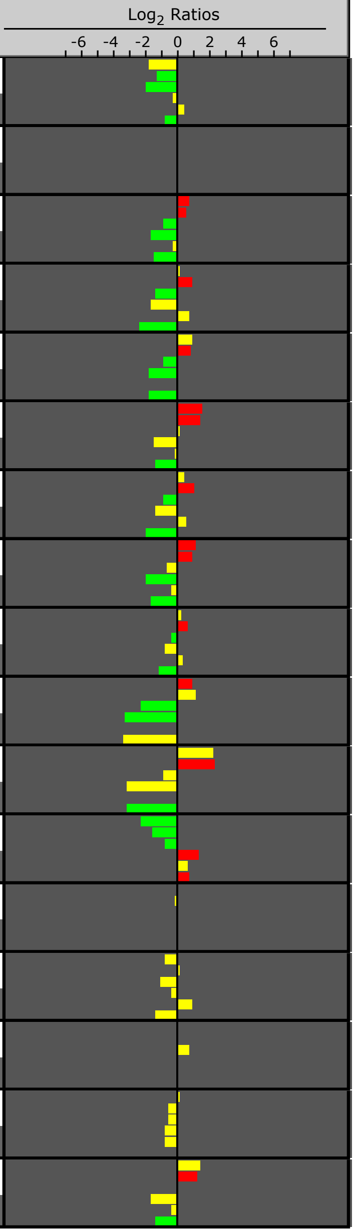

Show detected proteins only
Show all proteins
Filter by category:
GO: amino acid transport

Proteins found: 1424

Enter (or paste) list of ORFs

Find ORFs

Test	Cutoff
q-Value	.005
p-Value	

Dot Plots
Dot Plots

	Signif	Direction	Applies To
red	yes	+	ratios, bars
yellow	no	n/a	bars
green	yes	–	ratios, bars
pink	yes	+	*p*- , *q*-Values
light green	yes	–	*p*- , *q*-Values

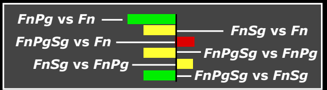

ORF	Protein	*FnPg vs Fn* Ratio	Sum	*q*-Val	*p*-Val	*FnSg vs Fn* Ratio	Sum	*q*-Val	*p*-Val	*FnPgSg vs Fn* Ratio	Sum	*q*-Val	*p*-Val	*FnPgSg vs FnPg* Ratio	Sum	*q*-Val	*p*-Val	*FnSg vs FnPg* Ratio	Sum	*q*-Val	*p*-Val	*FnPgSg vs FnSg* Ratio	Sum	*q*-Val	*p*-Val
**FN0874**	AAL95070.1| Phosphohydrolase (MUTT/NUDIX family protein)																	-1.230	5.874						
**FN0875**	AAL95071.1| 23S rRNA methyltransferase																					-0.449	5.481		
**FN0878**	AAL95074.1| Transcriptional regulator, GntR family	-0.755	8.025	4.545e-2	7.868e-2	0.183	9.147	5.429e-2	3.049e-1	0.358	8.934	1.732e-2	7.67e-2	1.112	8.383	1.551e-2	1.494e-2	0.937	8.392	6.663e-2	3.776e-2	0.175	9.505	2.076e-2	8.236e-2
**FN0884**	AAL95079.1| Hemin transport system permease protein hmuU																								
**FN0885**	AAL95081.1| Hemin-binding periplasmic protein hmuT precursor																								
**FN0886**	AAL95082.1| Hemin receptor									-2.221	5.391														
**FN0887**	AAL95083.1| Oligoendopeptidase F	-1.252	11.062	2.731e-3	1.965e-3	-1.043	11.455	8.556e-6	4.576e-6	-0.665	11.445	1.368e-3	3.7e-3	0.587	10.397	3.454e-2	4.699e-2	0.209	10.204	2.359e-1	4.001e-1	0.378	10.791	1.65e-2	6.304e-2
**FN0888**	AAL95084.1| Uracil permease	-1.032	9.253	3.171e-3	2.428e-3					-0.277	9.804	4.042e-2	2.037e-1	0.754	8.976	3.335e-2	4.452e-2								
**FN0889**	AAL95085.1| hypothetical protein	1.569	8.230	9.02e-2	1.987e-1	-0.253	6.592											-1.822	8.162						
**FN0892**	AAL95088.1| Phosphoserine phosphatase																								
**FN0893**	AAL95089.1| Hypothetical protein	-1.479	7.093			-0.571	8.186			-0.745	7.623	5.864e-3	2.207e-2	0.733	6.348			0.908	6.707			-0.174	7.440		
**FN0896**	AAL95092.1| Hypothetical protein	-0.599	8.237	6.117e-2	1.193e-1	-1.044	7.977	1.496e-4	2.678e-4	-0.412	8.220	1.307e-2	5.572e-2	0.187	7.824	2.098e-1	6.365e-1	-0.444	7.378	2.198e-1	3.593e-1	0.631	7.565	1.248e-2	4.56e-2
**FN0898**	AAL95094.1| Hypothetical protein																	0.373	5.202						
**FN0900**	AAL95096.1| Metal dependent hydrolase	-0.025	5.001	2.996e-1	9.537e-1	1.716	6.927											1.742	6.902						
**FN0901**	AAL95097.1| DNA polymerase, bacteriophage-type	0.456	5.883			1.053	6.665											0.597	7.121						
**FN0902**	AAL95098.1| 5-formyltetrahydrofolate cyclo-ligase																								
**FN0903**	AAL95099.1| Polysialic acid capsule expression protein kpsF	0.985	8.149	1.034e-2	1.181e-2	0.982	8.331	4.677e-4	1.124e-3	0.458	7.418	3.434e-3	1.158e-2	-0.527	8.607	2.865e-2	3.556e-2	-0.003	9.315	4.299e-1	9.845e-1	-0.524	8.788	7.871e-4	1.408e-3

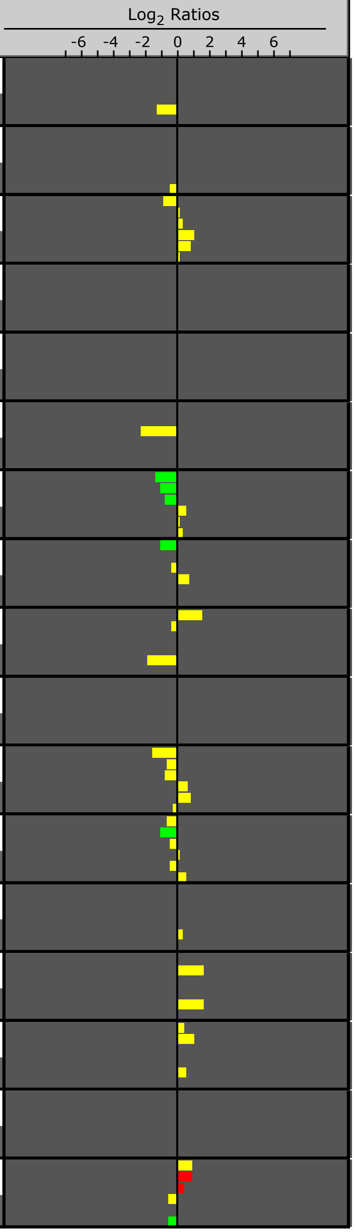

Show detected proteins only
Show all proteins
Filter by category:
GO: amino acid transport

Proteins found: 1424

Enter (or paste) list of ORFs

Find ORFs

Test	Cutoff
q-Value	.005
p-Value	

Dot Plots
Dot Plots

	Signif	Direction	Applies To
red	yes	+	ratios, bars
yellow	no	n/a	bars
green	yes	–	ratios, bars
pink	yes	+	*p*- , *q*-Values
light green	yes	–	*p*- , *q*-Values

ORF	Description	*FnPg vs Fn*				*FnSg vs Fn*				*FnPgSg vs Fn*				*FnPgSg vs FnPg*				*FnSg vs FnPg*				*FnPgSg vs FnSg*			
		Ratio	Sum	*q*-Val	*p*-Val	Ratio	Sum	*q*-Val	*p*-Val	Ratio	Sum	*q*-Val	*p*-Val	Ratio	Sum	*q*-Val	*p*-Val	Ratio	Sum	*q*-Val	*p*-Val	Ratio	Sum	*q*-Val	*p*-Val
**FN0904**	AAL95100.1| NAD(FAD)-utilizing dehydrogenases																								
**FN0905**	AAL95101.1| Hypothetical protein									-0.658	7.805	1.363e-4	1.691e-4												
**FN0906**	AAL95102.1| Glycerol-3-phosphate dehydrogenase [NAD(P)+]	-0.153	9.234	8.966e-2	1.971e-1	0.421	9.992	3.309e-3	1.269e-2	-1.134	8.049	5.811e-4	1.208e-3	-0.981	8.100	3.369e-3	1.468e-3	0.573	9.839	1.33e-2	2.589e-3	-1.555	8.858	7.235e-5	5.64e-5
**FN0908**	AAL95104.1| Tpl protein	-0.956	8.770			-1.015	8.896	5.304e-4	1.328e-3	-0.797	8.726	1.811e-3	5.357e-3	0.159	7.973			-0.058	7.940			0.218	8.099	9.648e-2	4.619e-1
**FN0909**	AAL95105.1| DNA repair protein radC																								
**FN0910**	AAL95106.1| Nicotinate-nucleotide--dimethylbenzimidazole phosphoribosyltransferase	0.050	5.950			-0.352	5.732	9.654e-2	5.678e-1									-0.402	5.782						
**FN0911**	AAL95107.1| Alpha-ribazole-5'-phosphate phosphatase	-0.258	6.598			-0.764	6.276	1.296e-3	4.076e-3	-0.519	6.133			-0.261	6.079			-0.506	6.018			0.246	5.757		
**FN0912**	AAL95108.1| Cobalamin [5'-phosphate] synthase																	-1.114	7.566						
**FN0913**	AAL95109.1| Cobinamide kinase	0.177	6.466			-0.237	6.237			-1.458	4.628			-1.635	5.009			-0.414	6.414			-1.221	4.779		
**FN0915**	AAL95111.1| PTS system, N-acetylglucosamine-specific IIA component	0.563	10.087	1.446e-1	3.619e-1	1.056	10.765	3.335e-3	1.282e-2	0.092	9.413	1.574e-3	4.491e-3	-0.471	10.179	1.562e-1	4.232e-1	0.493	11.328	1.881e-1	2.829e-1	-0.964	10.857	5.083e-3	1.535e-2
**FN0916**	AAL95112.1| Hypothetical Exported Protein	-0.426	14.600	6.671e-2	1.341e-1	-0.058	15.153	8.072e-2	4.666e-1	0.625	15.448	5.657e-3	2.116e-2	1.052	15.226	7.365e-3	4.589e-3	0.368	14.727	1.485e-1	1.858e-1	0.683	15.778	5.564e-3	1.721e-2
**FN0917**	AAL95113.1| Hypothetical protein	-1.434	5.434			0.180	7.233	1.004e-1	5.928e-1	-1.390	5.275			0.044	4.044			1.614	5.799			-1.570	5.843		
**FN0920**	AAL95116.1| Protease HTPX	0.123	6.521							-0.352	5.842			-0.475	6.169										
**FN0921**	AAL95117.1| Hypothetical protein	2.442	8.542			1.838	8.123	1.24e-4	2.07e-4	0.422	6.319	4.465e-2	2.278e-1	-2.019	8.964			-0.604	10.565			-1.416	8.545	2.97e-4	3.811e-4
**FN0922**	AAL95118.1| Homoserine kinase																								
**FN0923**	AAL95119.1| Cardiolipin synthetase																								
**FN0924**	AAL95120.1| Hypothetical protein																								

Log$_2$ Ratios: -6 -4 -2 0 2 4 6

Show detected proteins only
Show all proteins
Filter by category:
GO: amino acid transport

Proteins found: 1424

Enter (or paste) list of ORFs

Find ORFs

Test	Cutoff
q-Value	.005
p-Value	

Dot Plots
Dot Plots

	Signif	Direction	Applies To
red	yes	+	ratios, bars
yellow	no	n/a	bars
green	yes	–	ratios, bars
light red	yes	+	*p*- , *q*-Values
light green	yes	–	*p*- , *q*-Values

FnPg vs Fn
FnPgSg vs Fn
FnSg vs FnPg
FnSg vs Fn
FnPgSg vs FnPg
FnPgSg vs FnSg

ORF	Description	*FnPg vs Fn*				*FnSg vs Fn*				*FnPgSg vs Fn*				*FnPgSg vs FnPg*				*FnSg vs FnPg*				*FnPgSg vs FnSg*			
		Ratio	Sum	*q*-Val	*p*-Val	Ratio	Sum	*q*-Val	*p*-Val	Ratio	Sum	*q*-Val	*p*-Val	Ratio	Sum	*q*-Val	*p*-Val	Ratio	Sum	*q*-Val	*p*-Val	Ratio	Sum	*q*-Val	*p*-Val
FN0925	AAL95121.1| Hypothetical protein	-1.009	6.179			-0.349	7.023	7.453e-2	4.28e-1	-0.431	6.553	2.228e-2	1.019e-1	0.578	5.748			0.660	6.014			-0.082	6.592	1.654e-1	8.574e-1
FN0926	AAL95122.1| GTP pyrophosphokinase	2.552	8.283			2.328	8.244			0.381	5.908			-2.171	8.664	1.13e-4	1.085e-5	-0.223	10.796	1e-3	2.979e-5	-1.947	8.625	5.59e-5	4.037e-5
FN0928	AAL95124.1| O-sialoglycoprotein endopeptidase	0.234	6.410			-0.189	6.172			-0.420	5.552			-0.654	5.990			-0.423	6.406			-0.231	5.752	1.363e-1	6.826e-1
FN0929	AAL95125.1| ATP/GTP hydrolase													-1.182	7.271			0.453	9.091	2.564e-2	7.001e-3	-1.635	7.908		
FN0930	AAL95126.1| Glycerol-3-phosphate cytidylyltransferase	-1.586	6.895	6.277e-3	6.195e-3	-2.011	6.654											-0.425	5.068						
FN0932	AAL95128.1| Hypothetical protein	-0.282	8.096			-0.123	8.440	1.146e-1	6.879e-1	-1.612	6.563	8.808e-5	8.998e-5	-1.329	6.485			0.159	8.158			-1.489	6.828	1.288e-2	4.732e-2
FN0933	AAL95129.1| 3-phosphoshikimate 1-carboxyvinyltransferase																								
FN0934	AAL95130.1| Chorismate synthase	1.256	7.529			1.772	8.230	2.848e-3	1.051e-2									0.517	9.486						
FN0938	AAL95134.1| Hypothetical protein																	-0.128	11.165						
FN0940	AAL95136.1| Hypothetical protein													-1.827	8.888			-1.050	9.850			-0.777	8.022	3.662e-3	9.905e-3
FN0941	AAL95137.1| Gamma-glutamyltranspeptidase	1.593	10.529	3.764e-3	3.06e-3	0.983	10.104	7.167e-3	3.159e-2	0.493	9.225	3.183e-3	1.059e-2	-1.101	11.022	6.863e-3	4.075e-3	-0.610	11.697	5.384e-2	2.631e-2	-0.490	10.596	2.751e-2	1.13e-1
FN0943	AAL95139.1| Sensory Transduction Protein Kinase	1.943	8.232			1.376	7.849	8.893e-3	4.065e-2	-0.898	5.187	1.816e-4	2.518e-4	-2.841	7.334			-0.567	9.792			-2.274	6.951	6.423e-3	2.057e-2
FN0944	AAL95140.1| Na+ driven multidrug efflux pump																								
FN0947	AAL95143.1| Hypothetical protein	-0.292	12.982	1.46e-1	3.666e-1	-1.150	12.309	8.938e-4	2.6e-3	0.067	13.138	1.119e-1	6.376e-1	0.359	13.050	1.111e-1	2.599e-1	-0.858	12.017	1.148e-1	1.099e-1	1.217	12.376	2.641e-4	3.269e-4
FN0949	AAL95145.1| DNA helicase	-1.101	15.535	5.008e-2	8.975e-2	-2.512	14.308	4.18e-5	4.698e-5	-0.722	15.710	1.517e-4	1.958e-4	0.379	14.813	1.925e-1	5.644e-1	-1.411	13.207	1.839e-1	2.72e-1	1.790	13.586	6.204e-6	1.333e-6
FN0951	AAL95147.1| Precorrin-3B C17-methyltransferase	-0.699	7.680	6.285e-2	1.24e-1	-1.474	7.089											-0.775	6.390						
FN0952	AAL95148.1| Cobalamin biosynthesis protein G																								

Log_2 Ratios

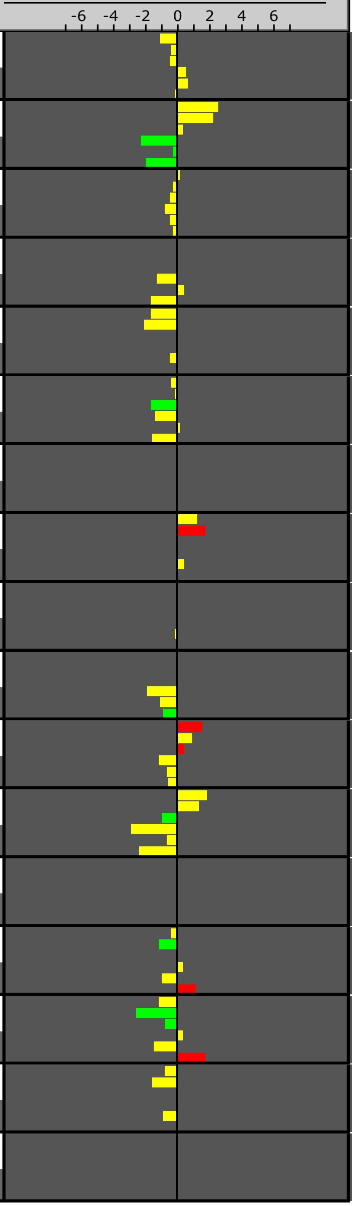

Show detected proteins only
Show all proteins
Filter by category:
GO: amino acid transport

Proteins found: 1424

Enter (or paste) list of ORFs

Find ORFs

Test	Cutoff
q-Value	.005
p-Value	

Dot Plots
Dot Plots

	Signif	Direction	Applies To
(red)	yes	+	ratios, bars
(yellow)	no	n/a	bars
(green)	yes	–	ratios, bars
(pink)	yes	+	*p*- , *q*-Values
(light green)	yes	–	*p*- , *q*-Values

FnPg vs Fn
FnPgSg vs Fn
FnSg vs FnPg
FnSg vs Fn
FnPgSg vs FnPg
FnPgSg vs FnSg

ORF	Protein	*FnPg vs Fn* Ratio	Sum	*q*-Val	*p*-Val	*FnSg vs Fn* Ratio	Sum	*q*-Val	*p*-Val	*FnPgSg vs Fn* Ratio	Sum	*q*-Val	*p*-Val	*FnPgSg vs FnPg* Ratio	Sum	*q*-Val	*p*-Val	*FnSg vs FnPg* Ratio	Sum	*q*-Val	*p*-Val	*FnPgSg vs FnSg* Ratio	Sum	*q*-Val	*p*-Val
**FN0957**	AAL95153.1| Precorrin-4 C11-methyltransferase	0.610	8.568	5.36e-2	9.857e-2	-0.807	7.335	1.344e-2	6.575e-2	0.546	8.300	2.589e-2	1.21e-1	-0.064	9.114	2.587e-1	8.367e-1	-1.418	7.945	3.972e-2	1.487e-2	1.354	7.881	5.589e-3	1.731e-2
**FN0958**	AAL95154.1| unknown	0.088	9.464			-1.973	7.588											-2.061	7.676						
**FN0959**	AAL95155.1| Precorrin-2 C20-methyltransferase	-1.417	8.838	6.722e-3	6.814e-3	-2.898	7.542			-1.205	8.846	2.769e-3	8.945e-3	0.212	7.633	2.124e-1	6.467e-1	-1.481	6.125			1.692	6.336		
**FN0961**	AAL95157.1| Hypothetical protein	1.043	6.383	5.123e-2	9.257e-2	1.938	7.463											0.896	8.505						
**FN0962**	AAL95158.1| Hypothetical cytosolic protein	-0.529	10.719	3.717e-3	3.008e-3	-1.888	9.545	1.759e-5	1.358e-5	-1.249	9.795	3.758e-5	2.628e-5	-0.721	9.470	7.793e-3	5.072e-3	-1.359	9.016	5.476e-3	6.561e-4	0.638	8.295	9.328e-3	3.223e-2
**FN0964**	AAL95160.1| Precorrin-8W decarboxylase													0.098	5.465			0.321	5.873	2.515e-2	6.763e-3	-0.223	5.970		
**FN0965**	AAL95161.1| D-3-phosphoglycerate dehydrogenase	0.981	10.948	4.022e-3	3.349e-3	0.869	11.020	6.598e-3	2.859e-2	0.503	10.266	2.95e-2	1.408e-1	-0.478	11.451	4.843e-2	7.579e-2	-0.112	12.001	3.106e-1	5.882e-1	-0.366	11.523	5.008e-2	2.233e-1
**FN0966**	AAL95162.1| Precorrin-6Y C5,15-methyltransferase (decarboxylating)																	0.645	3.999						
**FN0967**	AAL95163.1| CbiD protein													-1.575	4.949			-0.769	5.939			-0.806	4.364		
**FN0970**	AAL95166.1| Precorrin-8X methylmutase	-0.918	8.166			-1.208	8.061	2.347e-3	8.283e-3	-0.337	8.543	4.225e-3	1.49e-2	0.581	7.829			-0.290	7.143			0.871	7.724	8.098e-3	2.731e-2
**FN0971**	AAL95167.1| hypothetical cytosolic protein																								
**FN0972**	AAL95168.1| Cobyrinic acid a,c-diamide synthase																	0.608	8.205	2.417e-1	4.149e-1				
**FN0974**	AAL95170.1| Lactoylglutathione lyase																								
**FN0976**	AAL95172.1| Hypothetical protein	0.212	10.842	8.715e-3	9.619e-3	0.356	11.171	3.725e-2	2.029e-1	0.098	10.525	2.476e-2	1.149e-1	-0.113	10.941	1.466e-2	1.36e-2	0.144	11.383	2.891e-1	5.31e-1	-0.258	11.269	6.759e-2	3.121e-1
**FN0977**	AAL95173.1| Cobyric acid synthase	0.466	7.027			1.904	8.650			0.906	7.263			0.440	7.934	1.22e-1	2.977e-1	1.438	9.116	2.056e-2	4.855e-3	-0.998	9.556	4.871e-3	1.453e-2
**FN0981**	AAL95177.1| Phosphoribosylamine--glycine ligase	-0.541	13.269	9.925e-2	2.25e-1	-1.046	12.949	1.416e-3	4.534e-3	0.225	13.832	1.476e-2	6.401e-2	0.767	13.495	5.68e-2	9.683e-2	-0.504	12.408	2.381e-1	4.061e-1	1.271	13.175	2.399e-4	2.832e-4
**FN0982**	AAL95178.1| Phosphoribosylaminoimidazolecarboxamide formyltransferase	-0.982	15.812	3.046e-3	2.291e-3	-0.717	16.261	7.407e-5	1.079e-4	-0.629	15.961	6.087e-4	1.282e-3	0.353	15.183	5.256e-2	8.594e-2	0.265	15.280	1.408e-1	1.651e-1	0.088	15.633	8.976e-2	4.266e-1

Log_2 Ratios

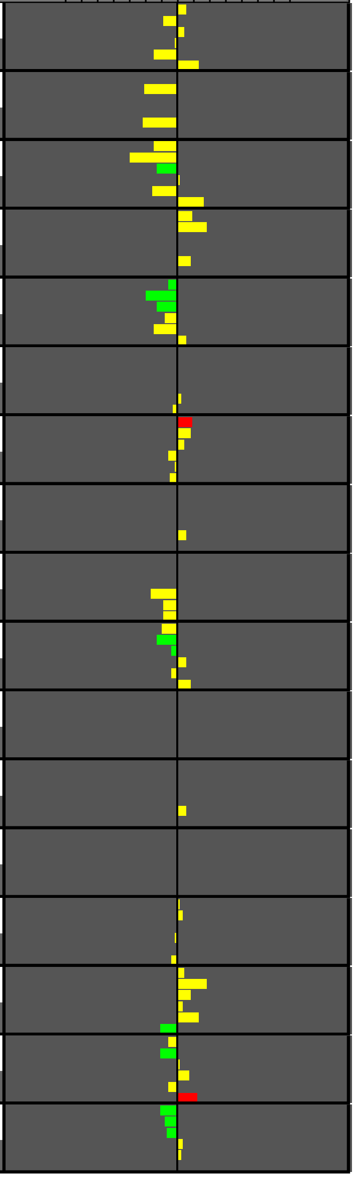

Show detected proteins only
Show all proteins
Filter by category:
GO: amino acid transport

Proteins found: 1424

Enter (or paste) list of ORFs

Find ORFs

Test	Cutoff
q-Value	.005
p-Value	

Dot Plots
Dot Plots

	Signif	Direction	Applies To
red	yes	+	ratios, bars
yellow	no	n/a	bars
green	yes	–	ratios, bars
pink	yes	+	*p*- , *q*-Values
light green	yes	–	*p*- , *q*-Values

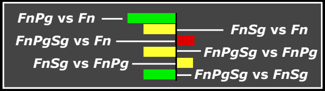

ORF	Description	FnPg vs Fn Ratio	Sum	q-Val	p-Val	FnSg vs Fn Ratio	Sum	q-Val	p-Val	FnPgSg vs Fn Ratio	Sum	q-Val	p-Val	FnPgSg vs FnPg Ratio	Sum	q-Val	p-Val	FnSg vs FnPg Ratio	Sum	q-Val	p-Val	FnPgSg vs FnSg Ratio	Sum	q-Val	p-Val
FN0983	AAL95179.1| Hypothetical protein	-0.487	14.330	1.85e-2	2.448e-2	-0.131	14.870	3.37e-2	1.821e-1	-0.751	13.862	3.496e-5	2.26e-5	-0.264	13.579	9.395e-2	1.985e-1	0.355	14.383	8.978e-2	6.911e-2	-0.620	14.119	1.557e-3	3.386e-3
FN0984	AAL95180.1| Tetracenomycin polyketide synthesis O-methyltransferase tcmP	0.262	9.711	6.168e-2	1.208e-1	1.135	10.768	2.169e-6	5.104e-7	-1.455	7.790	2.059e-4	3.036e-4	-1.717	8.256	5.642e-3	3.003e-3	0.873	11.030	5.226e-3	6.098e-4	-2.590	9.313	3.545e-5	2.077e-5
FN0985	AAL95181.1| Phosphoribosylglycinamide formyltransferase	-0.225	6.225			0.877	7.512	3.077e-3	1.159e-2	0.822	7.069	5.329e-3	1.973e-2	1.047	7.047			1.102	7.287			-0.055	8.334	1.523e-1	7.774e-1
FN0986	AAL95182.1| Phosphoribosylformylglycinamidine cyclo-ligase	0.244	14.606	2.176e-1	6.201e-1	0.475	15.021	3.343e-2	1.805e-1	0.300	14.458	9.588e-3	3.932e-2	0.056	14.906	2.727e-1	8.988e-1	0.231	15.265	3.259e-1	6.311e-1	-0.175	15.321	1.11e-1	5.399e-1
FN0987	AAL95183.1| Amidophosphoribosyltransferase	-0.757	13.564	8.981e-2	1.976e-1	0.022	14.528	1.348e-1	8.278e-1	-0.848	13.270	1.14e-3	2.853e-3	-0.090	12.716	2.716e-1	8.939e-1	0.779	13.771	1.482e-1	1.851e-1	-0.870	13.680	1.512e-4	1.5e-4
FN0988	AAL95184.1| Phosphoribosylamidoimidazole-succinocarboxamide synthase	-0.446	16.009	5.721e-2	1.081e-1	-0.386	16.254	1.078e-2	5.092e-2	0.276	16.528	9.733e-4	2.308e-3	0.723	16.285	2.183e-2	2.406e-2	0.060	15.807	3.888e-1	8.305e-1	0.662	16.530	2.478e-3	6.075e-3
FN0989	AAL95185.1| Phosphoribosylaminoimidazole carboxylase catalytic subunit	0.155	14.899	8.33e-2	1.794e-1	0.205	15.133	7.669e-2	4.414e-1	0.366	14.907	2.992e-3	9.854e-3	0.212	15.265	5.29e-2	8.682e-2	0.050	15.288	3.92e-1	8.416e-1	0.162	15.499	1.027e-1	4.95e-1
FN0990	AAL95186.1| Phosphoribosylformylglycinamidine synthase	-0.490	20.379	9.099e-2	2.009e-1	0.344	21.397	1.098e-5	6.798e-6	-0.210	20.455	1.551e-3	4.408e-3	0.279	20.169	1.644e-1	4.532e-1	0.834	20.908	7.738e-2	5.039e-2	-0.554	21.187	4.339e-5	2.86e-5
FN0991	AAL95187.1| CDP-diacylglycerol--serine O-phosphatidyltransferase	0.655	8.967	1.629e-2	2.067e-2	0.565	9.062	8.382e-3	3.79e-2	0.894	9.003	2.917e-3	9.545e-3	0.239	9.861	2.17e-2	2.385e-2	-0.090	9.717	2.554e-1	4.478e-1	0.329	9.956	8.242e-3	2.788e-2
FN0992	AAL95188.1| ADP-heptose:LPS heptosyltransferase II	-1.170	9.310	1.187e-2	1.391e-2	-0.278	10.387	6.06e-3	2.585e-2	-0.556	9.721	1.545e-3	4.381e-3	0.614	8.754	6.609e-2	1.194e-1	0.892	9.216	6.184e-2	3.307e-2	-0.278	9.830	1.901e-2	7.43e-2
FN0994	AAL95190.1| Hypothetical protein	-0.521	13.603	1.042e-1	2.392e-1	-0.481	13.827	1.253e-2	6.059e-2	0.167	14.088	6.077e-4	1.279e-3	0.689	13.770	7.093e-2	1.315e-1	0.040	13.306	4.174e-1	9.355e-1	0.649	13.995	7.483e-3	2.481e-2
FN0997	AAL95193.1| Hypothetical protein	0.196	10.477	2.442e-1	7.205e-1	0.845	11.311	3.85e-3	1.54e-2	-0.204	9.873	5.1e-2	2.645e-1	-0.400	10.273	1.803e-1	5.145e-1	0.649	11.507	1.465e-1	1.803e-1	-1.049	11.106	2.015e-3	4.662e-3
FN0998	AAL95194.1| Dipeptide-binding protein	-0.464	14.207	8.798e-2	1.924e-1	0.938	15.794	2.681e-5	2.464e-5	-0.900	13.567	7.919e-4	1.777e-3	-0.437	13.307	1.323e-1	3.367e-1	1.402	15.330	2.05e-2	4.831e-3	-1.839	14.893	1.011e-5	2.998e-6
FN0999	AAL95195.1| Deblocking aminopeptidase	1.169	10.601	1.08e-1	2.5e-1	0.532	10.149	6.355e-3	2.734e-2	-1.794	7.434	1.731e-3	5.06e-3	-2.963	8.807	6.27e-2	1.112e-1	-0.636	11.318	2.471e-1	4.279e-1	-2.326	8.355	1.002e-5	2.944e-6
FN1000	AAL95196.1| Biotin synthase	-1.723	10.632							-0.673	11.477	3.788e-4	6.974e-4	1.050	9.958										
FN1001	AAL95197.1| Dethiobiotin synthetase	0.363	10.024	1.78e-1	4.78e-1	0.526	10.371	7.727e-4	2.185e-3	-0.383	9.074	1.276e-2	5.425e-2	-0.746	9.641	1.053e-1	2.39e-1	0.163	10.734	3.475e-1	6.953e-1	-0.909	9.988	4.845e-4	7.161e-4
FN1002	AAL95198.1| Adenosylmethionine-8-amino-7-oxononanoate aminotransferase	0.038	12.127	2.916e-1	9.176e-1	-1.433	10.841	7.26e-5	1.046e-4	-0.790	11.096	8.906e-4	2.059e-3	-0.828	11.337	7.835e-2	1.513e-1	-1.471	10.879	8.808e-2	6.632e-2	0.643	10.051	1.64e-3	3.618e-3

Log$_2$ Ratios

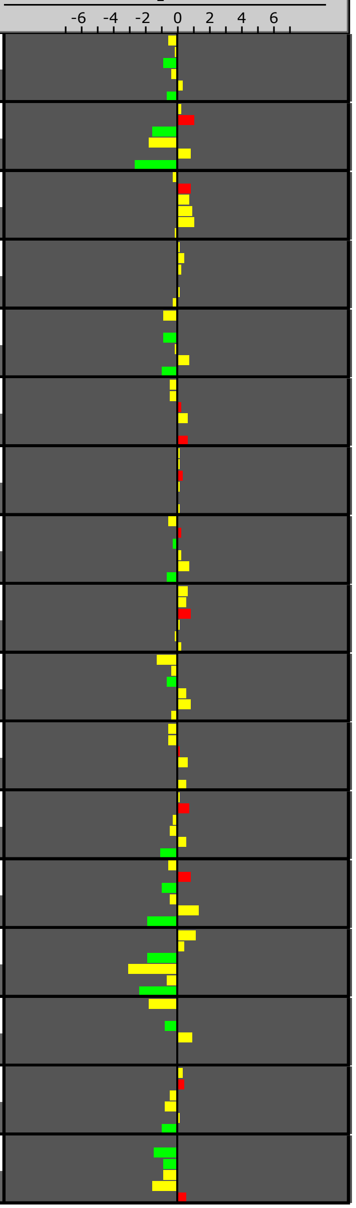

Show detected proteins only
Show all proteins
Filter by category:
GO: amino acid transport

Proteins found: 1424

Enter (or paste) list of ORFs

Find ORFs

Test	Cutoff
q-Value	.005
p-Value	

Dot Plots
Dot Plots

(color)	Signif	Direction	Applies To
red	yes	+	ratios, bars
yellow	no	n/a	bars
green	yes	–	ratios, bars
pink	yes	+	p- , q-Values
light green	yes	–	

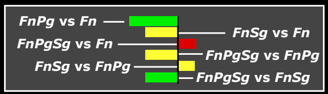

ORF	Description	FnPg vs Fn Ratio	FnPg vs Fn Sum	FnPg vs Fn *q*-Val	FnPg vs Fn *p*-Val	FnSg vs Fn Ratio	FnSg vs Fn Sum	FnSg vs Fn *q*-Val	FnSg vs Fn *p*-Val	FnPgSg vs Fn Ratio	FnPgSg vs Fn Sum	FnPgSg vs Fn *q*-Val	FnPgSg vs Fn *p*-Val	FnPgSg vs FnPg Ratio	FnPgSg vs FnPg Sum	FnPgSg vs FnPg *q*-Val	FnPgSg vs FnPg *p*-Val	FnSg vs FnPg Ratio	FnSg vs FnPg Sum	FnSg vs FnPg *q*-Val	FnSg vs FnPg *p*-Val	FnPgSg vs FnSg Ratio	FnPgSg vs FnSg Sum	FnPgSg vs FnSg *q*-Val	FnPgSg vs FnSg *p*-Val
FN1003	AAL95199.1| Outer membrane protein P1 precursor	2.452	12.779	1.031e-1	2.361e-1	1.665	12.176	3.113e-4	6.751e-4	0.334	10.456	1.132e-2	4.74e-2	-2.119	13.112	1.106e-1	2.583e-1	-0.787	14.629	2.781e-1	5.028e-1	-1.332	12.510	3.394e-4	4.533e-4
FN1004	AAL95200.1| Transcriptional regulator, TetR family	-0.043	8.170	2.818e-1	8.748e-1	0.192	8.589	1.141e-1	6.845e-1	-0.465	7.544	4.058e-2	2.047e-1	-0.423	7.705	1.482e-2	1.385e-2	0.234	8.546	3.101e-1	5.867e-1	-0.657	8.123	5.251e-2	2.356e-1
FN1005	AAL95201.1| Hypothetical protein	-1.383	11.558			-0.969	12.157	4.793e-4	1.161e-3	-1.648	11.090	2.823e-5	1.529e-5	-0.264	9.910			0.414	10.773			-0.678	10.509	1.288e-2	4.73e-2
FN1006	AAL95202.1| Acetyltransferase																	-0.900	8.070						
FN1008	AAL95204.1| Hypothetical protein																								
FN1009	AAL95205.1| Hypothetical protein																								
FN1010	AAL95206.1| Hypothetical cytosolic protein	0.010	14.731	3.047e-1	9.77e-1	-0.758	14.147	2.453e-4	4.93e-4	0.075	14.592	3.787e-2	1.888e-1	0.065	14.806	2.626e-1	8.539e-1	-0.769	14.158	1.418e-1	1.676e-1	0.833	14.222	6.179e-4	1.013e-3
FN1011	AAL95207.1| MGPA protein	-0.119	7.081	2.358e-1	6.877e-1	-0.124	7.260	1.113e-1	6.653e-1	-1.140	5.855			-1.022	5.940			-0.006	7.141	4.221e-1	9.536e-1	-1.016	6.119		
FN1012	AAL95208.1| HPR(Ser) kinase	0.540	9.286	1.597e-2	2.014e-2	0.830	9.761	3.931e-3	1.577e-2	-0.881	7.661	8.138e-4	1.838e-3	-1.421	8.405	4.169e-3	1.973e-3	0.290	10.301	1.471e-1	1.82e-1	-1.711	8.880	1.899e-3	4.333e-3
FN1014	AAL95210.1| Folylpolyglutamate synthase	-0.074	8.992			0.418	9.669	4.723e-2	2.622e-1	-1.798	7.064	2.864e-3	9.327e-3	-1.725	7.194			0.492	9.595			-2.216	7.871	6.417e-3	2.054e-2
FN1015	AAL95211.1| 5'-methylthioadenosine nucleosidase	0.151	9.565			-1.189	8.409	6.532e-3	2.825e-2	-0.513	8.696	2.865e-2	1.361e-1	-0.665	9.051			-1.341	8.560			0.676	7.895	7.405e-3	2.448e-2
FN1016	AAL95212.1| Lipid A biosynthesis lauroyl acyltransferase	-0.913	8.474	4.779e-2	8.424e-2	0.269	9.840	1.155e-2	5.513e-2	-1.958	7.224	1.7e-7	8.462e-9	-1.045	6.515	1.04e-1	2.341e-1	1.182	8.927	5.703e-2	2.886e-2	-2.227	7.881	4.995e-4	7.466e-4
FN1017	AAL95213.1| Hypothetical Exported Protein	-0.810	13.336	2.757e-2	4.055e-2	-0.888	13.443	3.443e-4	7.683e-4	-0.389	13.554	1.469e-3	4.093e-3	0.421	12.947	1.035e-1	2.322e-1	-0.078	12.632	3.919e-1	8.413e-1	0.499	13.054	7.066e-3	2.31e-2
FN1019	AAL95215.1| 3-hydroxybutyryl-CoA dehydrogenase	1.988	22.716	7.01e-4	2.607e-4	1.342	22.254	4.353e-5	4.996e-5	1.629	22.153	4.008e-4	7.532e-4	-0.359	24.345	8.252e-3	5.639e-3	-0.646	24.242	7.914e-3	1.238e-3	0.287	23.883	8.102e-3	2.732e-2
FN1020	AAL95216.1| 3-hydroxybutyryl-CoA dehydratase	1.393	17.148	8.937e-5	1.135e-5	1.016	16.956	7.01e-3	3.075e-2	0.845	16.396	4.69e-3	1.689e-2	-0.548	17.993	1.455e-2	1.343e-2	-0.377	18.349	1.128e-1	1.064e-1	-0.171	17.801	1.036e-1	5.001e-1
FN1022	AAL95218.1| Calcium-transporting ATPase									-1.792	9.007	6.154e-3	2.337e-2												
FN1023	AAL95219.1| 5-Nitroimidazole antibiotic resistance protein													-1.178	6.997			0.351	8.711			-1.530	7.533		

Log $_2$ Ratios

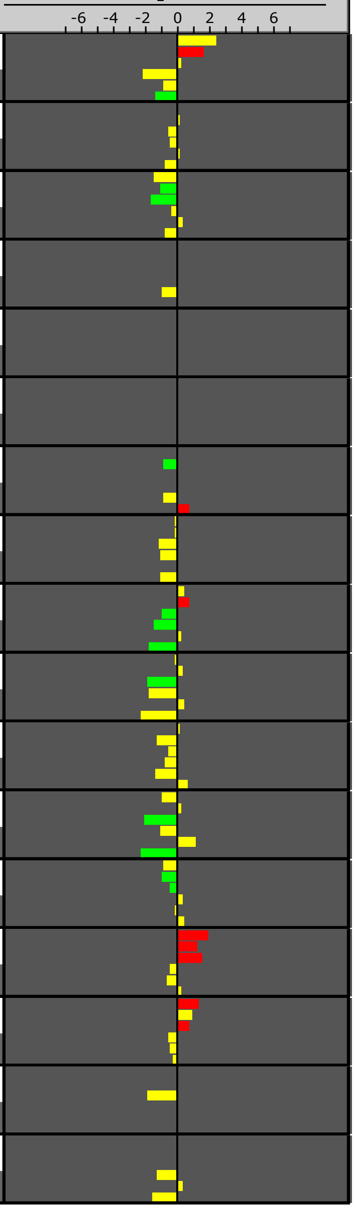

Show detected proteins only
Show all proteins
Filter by category:
GO: amino acid transport

Proteins found: 1424

Enter (or paste) list of ORFs

Find ORFs

Test	Cutoff
q-Value	.005
p-Value	

Dot Plots
Dot Plots

	Signif	Direction	Applies To
red	yes	+	ratios, bars
yellow	no	n/a	bars
green	yes	–	ratios, bars
pink	yes	+	*p*- , *q*-Values
light green	yes	–	*p*- , *q*-Values

FnPg vs Fn — FnSg vs Fn
FnPgSg vs Fn — FnPgSg vs FnPg
FnSg vs FnPg — FnPgSg vs FnSg

	FnPg vs Fn				*FnSg vs Fn*				*FnPgSg vs Fn*				*FnPgSg vs FnPg*				*FnSg vs FnPg*				*FnPgSg vs FnSg*			
ORF	Ratio	Sum	*q*-Val	*p*-Val	Ratio	Sum	*q*-Val	*p*-Val	Ratio	Sum	*q*-Val	*p*-Val	Ratio	Sum	*q*-Val	*p*-Val	Ratio	Sum	*q*-Val	*p*-Val	Ratio	Sum	*q*-Val	*p*-Val
**FN1024**	-0.259	20.496	5.749e-2	1.089e-1	-0.340	20.600	1.236e-2	5.962e-2	-0.164	20.387	8.373e-2	4.604e-1	0.096	20.332	2.291e-1	7.125e-1	-0.080	20.340	3.402e-1	6.731e-1	0.176	20.436	1.063e-1	5.144e-1
AAL95220.1| DNA-binding protein HU																								
**FN1025**	0.257	8.083			-0.683	7.327			0.114	7.736	6.586e-2	3.515e-1	-0.143	8.197			-0.940	7.584			0.797	7.441		
AAL95221.1| Guanine-hypoxanthine permease																								
**FN1026**					-1.144	6.314																		
AAL95222.1| tRNA pseudouridine synthase A																								
**FN1028**	-0.690	10.293	4.263e-2	7.194e-2	-0.896	10.271	8.173e-3	3.679e-2	-0.275	10.505	5.765e-2	3.036e-1	0.415	10.018	9.453e-4	2.2e-4	-0.206	9.581	2.31e-1	3.874e-1	0.622	9.997	9.951e-3	3.482e-2
AAL95224.1| Deoxyuridine 5'-triphosphate nucleotidohydrolase																								
**FN1029**	-0.677	13.217	6.642e-4	2.383e-4	-1.376	12.703	1.812e-5	1.419e-5	-0.888	12.803	6.058e-4	1.274e-3	-0.211	12.329	9.931e-2	2.169e-1	-0.699	12.026	5.539e-4	7.104e-6	0.488	11.815	1.641e-2	6.267e-2
AAL95225.1| Zinc protease																								
**FN1030**	2.719	6.451			2.408	6.323			1.236	4.764			-1.483	7.687			-0.312	9.043			-1.171	7.560		
AAL95226.1| Hypothetical membrane-spanning protein																								
**FN1031**	0.330	6.840			0.112	6.807			-1.211	5.096			-1.541	5.629			-0.218	7.137			-1.323	5.596		
AAL95227.1| Hypothetical membrane-spanning protein																								
**FN1033**	-0.493	12.379	2.38e-2	3.371e-2	-0.763	12.293	2.02e-3	6.894e-3	-1.756	10.912	9.872e-4	2.351e-3	-1.263	10.623	3.927e-5	1.921e-6	-0.270	11.800	1.251e-1	1.298e-1	-0.993	10.538	4.381e-3	1.262e-2
AAL95229.1| Methyltransferase																								
**FN1034**																					-1.092	5.480		
AAL95230.1| Transcriptional regulator, TetR family																								
**FN1035**																								
AAL95231.1| Iron-sulfur flavoprotein																								
**FN1036**																								
AAL95232.1| Hypothetical protein																								
**FN1037**													-2.220	5.594			0.102	8.100			-2.322	5.880		
AAL95233.1| Hypothetical cytosolic protein																								
**FN1041**													-0.327	6.701	1.623e-1	4.455e-1	0.452	7.665	1.624e-1	2.198e-1	-0.779	7.337	4.189e-3	1.186e-2
AAL95237.1| Acetyltransferase																								
**FN1042**	-2.915	10.932	2.608e-4	5.805e-5	-2.845	11.186	1.938e-5	1.574e-5	-2.360	11.283	1.743e-4	2.372e-4	0.555	8.572	1.808e-2	1.847e-2	0.070	8.271	3.84e-1	8.14e-1	0.485	8.826	2.127e-2	8.474e-2
AAL95238.1| S1 RNA binding domain																								
**FN1045**																								
AAL95241.1| Hypothetical protein																								
**FN1048**																					-0.186	3.744		
AAL95244.1| Hypothetical membrane-spanning protein																								
**FN1050**	0.963	7.139																						
AAL95246.1| Lactoylglutathione lyase																								

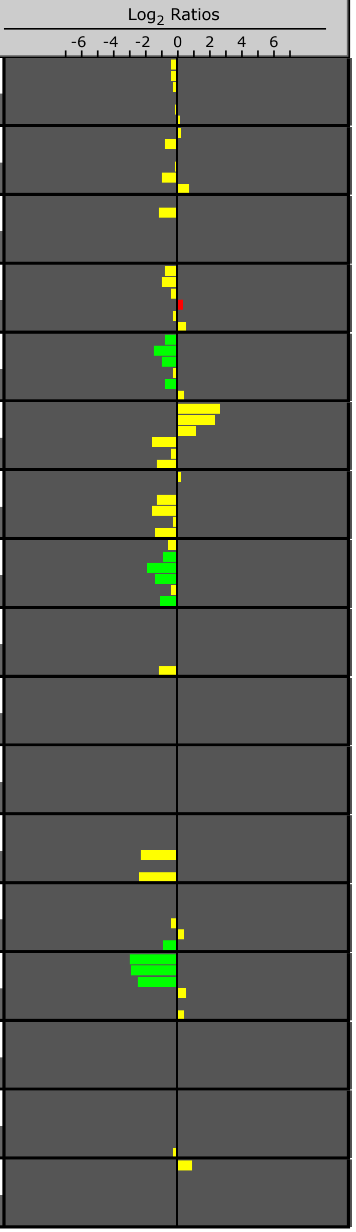

Show detected proteins only
Show all proteins
Filter by category:
GO: amino acid transport

Proteins found: 1424

Enter (or paste) list of ORFs

Find ORFs

Test	Cutoff
q-Value	.005
p-Value	

Dot Plots
Dot Plots

	Signif	Direction	Applies To
red	yes	+	ratios, bars
yellow	no	n/a	bars
green	yes	–	ratios, bars
pink	yes	+	*p*- , *q*-Values
light green	yes	–	*p*- , *q*-Values

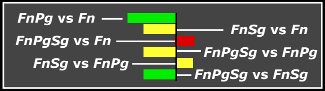

ORF	*FnPg vs Fn* Ratio	Sum	*q*-Val	*p*-Val	*FnSg vs Fn* Ratio	Sum	*q*-Val	*p*-Val	*FnPgSg vs Fn* Ratio	Sum	*q*-Val	*p*-Val	*FnPgSg vs FnPg* Ratio	Sum	*q*-Val	*p*-Val	*FnSg vs FnPg* Ratio	Sum	*q*-Val	*p*-Val	*FnPgSg vs FnSg* Ratio	Sum	*q*-Val	*p*-Val
**FN1053** AAL95249.1| Hypothetical protein																								
**FN1055** AAL95251.1| Cysteine synthase	0.210	11.779	1.959e-1	5.433e-1	0.291	12.045	8.966e-3	4.105e-2	-0.421	10.944	7.886e-3	3.12e-2	-0.631	11.357	8.252e-2	1.631e-1	0.082	12.254	3.757e-1	7.855e-1	-0.712	11.624	6.778e-4	1.149e-3
**FN1057** AAL95253.1| Diamine acetyltransferase					0.533	6.990	7.972e-2	4.603e-1																
**FN1060** AAL95256.1| hypothetical cytosolic protein	0.259	9.702	2.678e-2	3.909e-2	0.612	10.239	1.057e-3	3.183e-3	-0.041	9.198	1.263e-1	7.317e-1	-0.300	9.661	1.346e-2	1.186e-2	0.353	10.498	1.031e-2	1.79e-3	-0.653	10.198	8.286e-4	1.513e-3
**FN1062** AAL95258.1| Hydrolase	-0.585	12.657	2.795e-2	4.127e-2	-1.435	11.992	5.281e-4	1.32e-3	0.379	13.417	1.426e-3	3.923e-3	0.964	13.036	6.708e-3	3.924e-3	-0.849	11.407	7.556e-2	4.801e-2	1.814	12.371	3.726e-5	2.245e-5
**FN1063** AAL95259.1| N-acyl-L-amino acid amidohydrolase	1.195	8.197	1.172e-1	2.768e-1	1.285	8.472	8.544e-3	3.876e-2	-1.399	5.399			-2.594	6.798			0.090	9.667	4.069e-1	8.958e-1	-2.684	7.073		
**FN1066** AAL95262.1| Exodeoxyribonuclease VII large subunit	-0.017	6.272	3.01e-1	9.601e-1	0.902	7.375	1.146e-5	7.309e-6	0.018	6.103	1.388e-1	8.16e-1	0.035	6.290	2.769e-1	9.179e-1	0.919	7.358	5.128e-2	2.383e-2	-0.884	7.393	2.037e-5	9.641e-6
**FN1067** AAL95263.1| Tetratricopeptide repeat family protein	-1.650	11.075	8.144e-4	3.35e-4	-0.182	12.727	3.395e-2	1.836e-1	-1.263	11.258	4.003e-4	7.519e-4	0.386	9.812	1.109e-1	2.592e-1	1.468	11.077	6.324e-3	8.334e-4	-1.081	11.464	3.276e-4	4.341e-4
**FN1068** AAL95264.1| Smf protein																	0.658	7.502	1.579e-1	2.093e-1				
**FN1069** AAL95265.1| DNA topoisomerase I	-0.652	12.442	4.102e-4	1.116e-4	-1.288	11.991	5.692e-4	1.452e-3	-0.952	11.939	2.083e-4	3.089e-4	-0.299	11.491	1.064e-2	8.34e-3	-0.636	11.338	7.169e-2	4.331e-2	0.337	11.039	5.03e-2	2.244e-1
**FN1070** AAL95266.1| Glucose inhibited division protein A	-1.251	7.591			-1.303	7.724	9.517e-4	2.805e-3	-0.598	8.041	3.333e-3	1.118e-2	0.653	6.993			-0.052	6.472			0.705	7.125	9.987e-3	3.497e-2
**FN1071** AAL95267.1| Integrase/recombinase					-0.484	4.484			0.295	4.875											0.779	4.779		
**FN1072** AAL95268.1| GTP-binding protein	-0.740	11.295	5.045e-3	4.61e-3	-1.506	10.713	3.42e-4	7.616e-4	-1.230	10.601	5.007e-5	4.1e-5	-0.490	10.065	5.198e-2	8.445e-2	-0.766	9.973	7.671e-2	4.95e-2	0.276	9.483	8.59e-2	4.065e-1
**FN1073** AAL95269.1| Hypothetical protein	-1.142	6.312			-0.586	7.052	3.602e-3	1.414e-2	-1.682	5.567			-0.541	4.629			0.556	5.911			-1.097	5.370		
**FN1074** AAL95270.1| Signal recognition particle receptor FtsY	-0.644	9.742	1.966e-2	2.653e-2	-0.271	10.300	1.087e-2	5.138e-2	-0.890	9.292	1.448e-3	4.01e-3	-0.246	8.852	1.45e-1	3.834e-1	0.373	9.655	1.244e-1	1.284e-1	-0.619	9.409	3.889e-3	1.073e-2
**FN1075** AAL95271.1| Hypothetical protein																								
**FN1077** AAL95273.1| Hypothetical protein	2.041	8.217			0.234	6.594			2.394	8.366			0.353	10.611			-1.808	8.636			2.160	8.988	2.331e-4	2.717e-4

Log$_2$ Ratios

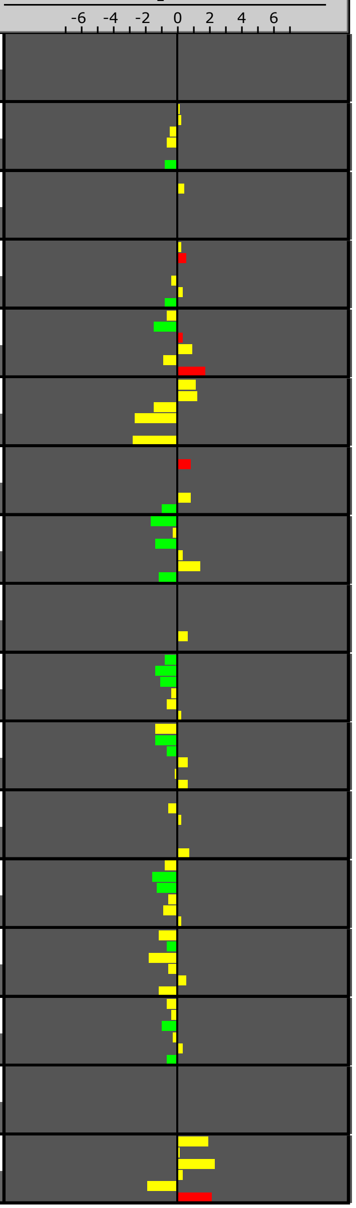

Show detected proteins only
Show all proteins
Filter by category:
GO: amino acid transport

Proteins found: 1424

Enter (or paste) list of ORFs

Find ORFs

Test	Cutoff
q-Value	.005
p-Value	

Dot Plots
Dot Plots

	Signif	Direction	Applies To
red	yes	+	ratios, bars
yellow	no	n/a	bars
green	yes	–	ratios, bars
pink	yes	+	*p*- , *q*-Values
light green	yes	–	*p*- , *q*-Values

Fusobacterium nucleatum

ORF	*FnPg vs Fn*				*FnSg vs Fn*				*FnPgSg vs Fn*				*FnPgSg vs FnPg*				*FnSg vs FnPg*				*FnPgSg vs FnSg*			
	Ratio	Sum	*q*-Val	*p*-Val	Ratio	Sum	*q*-Val	*p*-Val	Ratio	Sum	*q*-Val	*p*-Val	Ratio	Sum	*q*-Val	*p*-Val	Ratio	Sum	*q*-Val	*p*-Val	Ratio	Sum	*q*-Val	*p*-Val
**FN1078**	0.316	15.414	5.335e-2	9.794e-2	-0.089	15.193	5.043e-2	2.817e-1	-0.160	14.734	5.6e-2	2.94e-1	-0.476	15.254	3.081e-2	3.954e-2	-0.405	15.509	8.587e-2	6.27e-2	-0.071	15.034	1.212e-1	5.969e-1
	AAL95274.1| Hypothetical exported 24-amino acid repeat protein																							
**FN1079**	0.523	18.003	1.197e-1	2.843e-1	0.970	18.635	7.349e-4	2.037e-3	0.561	17.838	1.771e-3	5.207e-3	0.039	18.565	2.765e-1	9.161e-1	0.447	19.158	1.624e-1	2.199e-1	-0.408	19.196	3.785e-3	1.035e-2
	AAL95275.1| Neutrophil-activating protein A																							
**FN1080**	-0.972	4.632	3.24e-3	2.504e-3	0.710	6.498	5.753e-3	2.432e-2	-0.224	5.175	3.174e-2	1.536e-1	0.748	4.408	3.152e-3	1.322e-3	1.682	5.526	2.047e-2	4.82e-3	-0.934	6.274	5.287e-3	1.613e-2
	AAL95276.1| Export ABC transporter																							
**FN1081**	-0.113	10.309							-0.891	9.327	1.83e-3	5.426e-3	-0.778	9.418										
	AAL95277.1| unknown																							
**FN1082**																								
	AAL95278.1| unknown																							
**FN1084**	-1.661	11.727	1.27e-3	6.878e-4	-2.644	10.928	5.242e-4	1.307e-3	-0.039	13.144	1.443e-1	8.537e-1	1.622	11.687	7.835e-3	5.122e-3	-0.983	9.267	8.285e-2	5.811e-2	2.604	10.889	2.093e-3	4.89e-3
	AAL95280.1| unknown																							
**FN1085**	1.380	12.544	3.034e-3	2.278e-3	1.003	12.351	6.321e-4	1.663e-3	0.163	11.123	7.055e-2	3.794e-1	-1.217	12.707	1.667e-3	5.287e-4	-0.378	13.731	5.469e-2	2.697e-2	-0.840	12.514	7.008e-4	1.201e-3
	AAL95281.1| 4-methyl-5(B-hydroxyethyl)-thiazole monophosphate biosynthesis enzyme																							
**FN1086**	-0.345	5.960			1.114	7.603	7.851e-3	3.51e-2	0.388	6.489	5.178e-2	2.69e-1	0.733	6.348			1.459	7.258			-0.726	7.992	2.121e-2	8.449e-2
	AAL95282.1| Transporter																							
**FN1088**	-0.324	9.206	8.026e-2	1.707e-1	0.353	10.067	7.738e-4	2.19e-3	0.379	9.704	3.74e-3	1.283e-2	0.703	9.585	1.4e-2	1.262e-2	0.677	9.744	4.406e-2	1.794e-2	0.025	10.446	1.533e-1	7.834e-1
	AAL95284.1| NADH oxidase																							
**FN1089**	2.082	12.161	1.92e-2	2.572e-2	2.829	13.093	6.809e-4	1.836e-3	-0.071	9.804	1.148e-1	6.566e-1	-2.153	12.090	2.185e-2	2.41e-2	0.747	15.175	5.694e-2	2.879e-2	-2.901	13.022	8.523e-4	1.576e-3
	AAL95285.1| ATP-binding protein (contains P-loop)																							
**FN1091**	-1.787	7.371	8.297e-4	3.461e-4	-0.843	8.500	2.384e-3	8.445e-3	-0.748	8.207	3.136e-4	5.364e-4	1.040	6.623	9.028e-3	6.443e-3	0.944	6.713	7.9e-2	5.261e-2	0.095	7.752	1.414e-1	7.125e-1
	AAL95287.1| Sigma factor sigB regulation protein rsbU																							
**FN1092**	0.197	10.888	2.661e-1	8.086e-1	0.908	11.784	1.32e-3	4.169e-3	-0.497	9.991	4.951e-2	2.558e-1	-0.693	10.392	1.753e-1	4.947e-1	0.711	11.981	1.841e-1	2.727e-1	-1.405	11.288	3.51e-3	9.371e-3
	AAL95288.1| Hypothetical protein																							
**FN1093**	-1.444	11.607	7.89e-4	3.171e-4	-1.931	11.305	1.177e-4	1.931e-4	-0.599	12.248	8.443e-6	2.533e-6	0.845	11.009	6.844e-3	4.056e-3	-0.487	9.861	1.025e-1	8.896e-2	1.332	10.706	8.764e-4	1.633e-3
	AAL95289.1| Hypothetical protein																							
**FN1094**																	-0.782	9.491						
	AAL95290.1| Dolichol-phosphate mannosyltransferase																							
**FN1095**																								
	AAL95291.1| unknown																							
**FN1096**	-0.169	10.967	2.421e-1	7.121e-1	-0.910	10.411	7.755e-4	2.196e-3	-0.245	10.688	7.222e-2	3.895e-1	-0.076	10.723	2.707e-1	8.896e-1	-0.741	10.242	1.873e-1	2.807e-1	0.666	10.166	3.181e-2	1.332e-1
	AAL95292.1| Hypothetical protein																							
**FN1097**	-1.064	11.373			-3.311	9.311			-2.213	10.020	2.738e-3	8.822e-3	-1.149	9.161			-2.247	8.247			1.098	7.098		
	AAL95293.1| Hypothetical protein																							

Log_2 Ratios

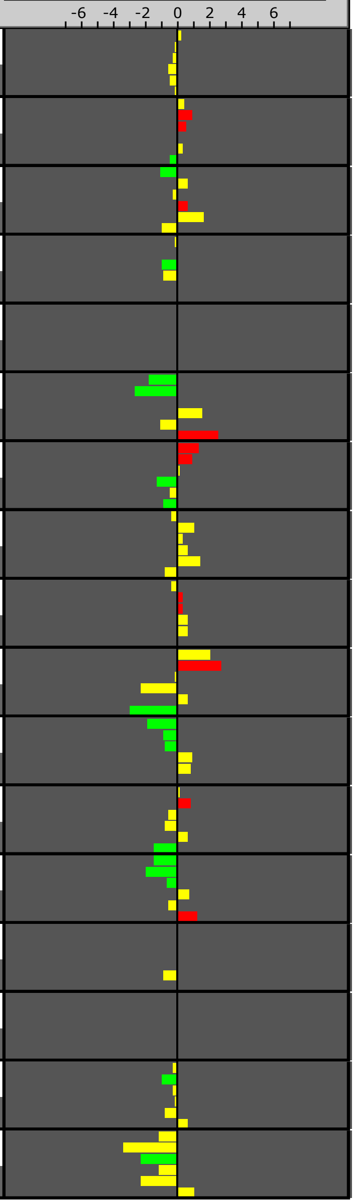

Show detected proteins only
Show all proteins
Filter by category:
GO: amino acid transport

Proteins found:
1424

Enter (or paste) list of ORFs

Find ORFs

Test	Cutoff
q-Value	.005
p-Value	

Dot Plots
Dot Plots

	Signif	Direction	Applies To
(red)	yes	+	ratios, bars
(yellow)	no	n/a	bars
(green)	yes	–	ratios, bars
(pink)	yes	+	*p*- , *q*-Values
(light green)	yes	–	

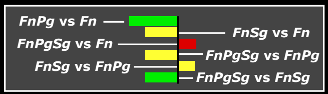

ORF	Description	*FnPg vs Fn*				*FnSg vs Fn*				*FnPgSg vs Fn*				*FnPgSg vs FnPg*				*FnSg vs FnPg*				*FnPgSg vs FnSg*			
		Ratio	Sum	*q*-Val	*p*-Val	Ratio	Sum	*q*-Val	*p*-Val	Ratio	Sum	*q*-Val	*p*-Val	Ratio	Sum	*q*-Val	*p*-Val	Ratio	Sum	*q*-Val	*p*-Val	Ratio	Sum	*q*-Val	*p*-Val
**FN1101**	AAL95297.1| ATPase					0.305	5.695			-0.916	4.086											-1.221	4.779		
**FN1102**	AAL95298.1| tRNA 2'phosphotransferase					-2.018	8.662																		
**FN1103**	AAL95299.1| Excinuclease ABC subunit A	-1.735	11.758	2.231e-4	4.514e-5	-0.839	12.838	1.675e-4	3.098e-4	-2.179	11.110	1.714e-5	7.156e-6	-0.444	9.579	6.127e-2	1.078e-1	0.896	11.103	2.65e-2	7.402e-3	-1.340	10.659	5.227e-4	7.95e-4
**FN1104**	AAL95300.1| Holliday junction DNA helicase ruvA																								
**FN1105**	AAL95301.1| Hypothetical protein	-0.290	13.052	1.489e-1	3.76e-1	-1.252	12.275	5.963e-4	1.541e-3	-0.344	12.794	7.461e-3	2.925e-2	-0.054	12.708	2.666e-1	8.714e-1	-0.962	11.985	1.112e-1	1.035e-1	0.907	11.931	7.415e-7	8.423e-8
**FN1106**	AAL95302.1| L-serine dehydratase	-0.657	12.631	1.085e-3	5.357e-4	-0.775	12.697	3.518e-4	7.885e-4	-1.220	11.864	3.345e-4	5.862e-4	-0.563	11.411	1.249e-2	1.057e-2	-0.118	12.041	2.593e-1	4.571e-1	-0.445	11.477	1.421e-2	5.298e-2
**FN1111**	AAL95307.1| Dipeptide-binding protein					0.485	7.453			-0.122	6.457											-0.607	7.330	2.488e-3	6.108e-3
**FN1117**	AAL95313.1| LSU ribosomal protein L21P	-0.613	10.930	8.015e-2	1.704e-1	-1.871	9.855	8.249e-4	2.362e-3	0.673	12.012	2.563e-3	8.139e-3	1.286	11.603	1.516e-2	1.439e-2	-1.259	9.243	1.266e-1	1.329e-1	2.544	10.529	1.757e-5	7.374e-6
**FN1119**	AAL95315.1| LSU ribosomal protein L27P	-0.859	13.803	3.573e-2	5.682e-2	-2.497	12.350	1.089e-5	6.706e-6	0.519	14.977	6.182e-4	1.308e-3	1.378	14.322	9.072e-3	6.491e-3	-1.638	11.491	1.031e-1	8.995e-2	3.015	12.869	2.552e-5	1.297e-5
**FN1120**	AAL95316.1| Phosphoenolpyruvate carboxykinase (ATP)	0.799	15.297	6.978e-3	7.186e-3	0.750	15.433	8.342e-4	2.394e-3	-0.154	14.141	5.19e-2	2.697e-1	-0.953	15.144	1.162e-2	9.5e-3	-0.049	16.232	3.515e-1	7.078e-1	-0.904	15.279	1.126e-5	3.526e-6
**FN1121**	AAL95317.1| hypothetical cytosolic protein	-0.372	12.103	4.579e-2	7.952e-2	-0.163	12.497	2.032e-2	1.042e-1	0.155	12.426	3.124e-2	1.507e-1	0.527	12.258	2.112e-2	2.294e-2	0.209	12.124	1.857e-1	2.765e-1	0.318	12.651	5.013e-3	1.509e-2
**FN1122**	AAL95318.1| Long-chain-fatty-acid--CoA ligase	-2.018	13.763	2.365e-3	1.621e-3	-1.834	14.132	2.495e-4	5.036e-4	-0.692	14.885	3.039e-3	1.003e-2	1.326	13.071	2.949e-4	4.086e-5	0.184	12.114	2.358e-1	3.999e-1	1.142	13.439	4.401e-4	6.299e-4
**FN1123**	AAL95319.1| Thioredoxin-like protein	-2.046	7.413	5.593e-5	5.847e-6	-1.550	8.094	1.478e-3	4.775e-3	0.332	9.587	7.741e-2	4.211e-1	2.377	7.745	2.844e-2	3.518e-2	0.496	6.048	2.256e-1	3.737e-1	1.882	8.425	1.103e-2	3.946e-2
**FN1124**	AAL95320.1| Outer membrane porin F	-1.027	16.457	1.293e-3	7.057e-4	-0.264	17.404	3.451e-5	3.499e-5	-0.247	17.032	5.061e-3	1.852e-2	0.779	16.209	1.569e-3	4.833e-4	0.763	16.378	1.047e-2	1.829e-3	0.016	17.157	1.577e-1	8.097e-1
**FN1125**	AAL95321.1| LemA protein	0.845	11.386	1.311e-1	3.194e-1	1.202	11.928	1.85e-3	6.205e-3	0.472	10.810	1.275e-3	3.34e-3	-0.372	11.858	1.974e-1	5.849e-1	0.357	12.772	2.829e-1	5.15e-1	-0.730	12.400	5.505e-3	1.698e-2
**FN1127**	AAL95323.1| Hypothetical membrane-spanning protein	1.087	11.860	2.91e-2	4.344e-2	0.590	11.548	8.464e-4	2.436e-3	-0.015	10.554	1.511e-1	9.013e-1	-1.102	11.845	3.37e-2	4.523e-2	-0.497	12.635	1.423e-1	1.688e-1	-0.605	11.533	1.751e-4	1.824e-4
**FN1128**	AAL95324.1| Acylamino-acid-releasing enzyme	0.334	16.075	1.092e-1	2.535e-1	-0.399	15.526	2.826e-4	5.924e-4	-0.418	15.119	4.669e-4	9.141e-4	-0.752	15.657	4.541e-2	6.888e-2	-0.734	15.860	9.162e-2	7.223e-2	-0.018	15.108	1.414e-1	7.122e-1

Log$_2$ Ratios

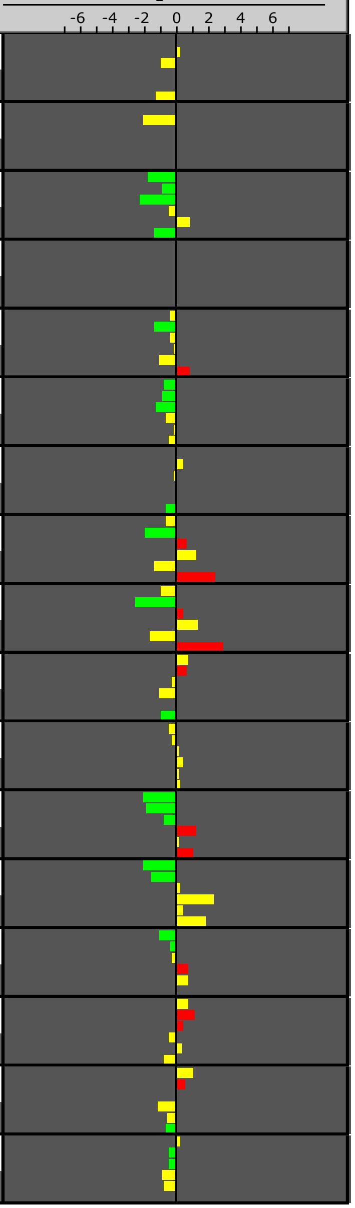

Show detected proteins only
Show all proteins
Filter by category:
GO: amino acid transport

Proteins found: 1424

Enter (or paste) list of ORFs

Find ORFs

Test	Cutoff
***q*-Value**	.005
p-Value	

Dot Plots
Dot Plots

	Signif	Direction	Applies To
(red)	yes	+	ratios, bars
(yellow)	no	n/a	bars
(green)	yes	–	ratios, bars
(pink)	yes	+	*p*- , *q*-Values
(light green)	yes	–	*p*- , *q*-Values

FnPg vs Fn
FnSg vs Fn
FnPgSg vs Fn
FnPgSg vs FnPg
FnSg vs FnPg
FnPgSg vs FnSg

	FnPg vs Fn				*FnSg vs Fn*				*FnPgSg vs Fn*				*FnPgSg vs FnPg*				*FnSg vs FnPg*				*FnPgSg vs FnSg*				
ORF	Ratio	Sum	*q*-Val	*p*-Val	Ratio	Sum	*q*-Val	*p*-Val	Ratio	Sum	*q*-Val	*p*-Val	Ratio	Sum	*q*-Val	*p*-Val	Ratio	Sum	*q*-Val	*p*-Val	Ratio	Sum	*q*-Val	*p*-Val	Protein
**FN1129**	-1.843	7.013			-1.502	7.539	2.65e-4	5.444e-4	-1.290	7.362	1.808e-4	2.501e-4	0.553	5.723			0.341	5.696			0.212	6.249	1.142e-1	5.575e-1	AAL95325.1| Chromosome partition protein smc
**FN1130**	-1.596	6.239			-0.550	7.469			-0.816	6.816			0.780	5.424			1.045	5.874			-0.265	6.654			AAL95326.1| Tetraacyldisaccharide 4'-kinase
**FN1131**	0.502	6.899			-0.484	6.098			-1.512	4.682			-2.014	5.387			-0.985	6.600			-1.028	4.586			AAL95327.1| Hypothetical protein
**FN1133**	0.535	10.186	1.525e-1	3.881e-1	0.040	9.875	1.23e-1	7.452e-1	0.110	9.557	7.076e-2	3.807e-1	-0.425	10.296	1.69e-1	4.706e-1	-0.495	10.411	2.427e-1	4.173e-1	0.070	9.985	1.316e-1	6.553e-1	AAL95329.1| N-acetylglucosamine-6-phosphate deacetylase
**FN1134**	-0.750	10.293	1.882e-2	2.506e-2	-2.614	8.614			-0.962	9.877	3.515e-4	6.284e-4	-0.212	9.331	1.827e-1	5.238e-1	-1.864	7.864			1.652	7.652			AAL95330.1| Hypothetical cytosolic protein
**FN1135**	0.232	15.087	2.28e-1	6.583e-1	-0.530	14.510	1.273e-3	3.987e-3	-0.346	14.305	2.104e-4	3.139e-4	-0.578	14.741	1.373e-1	3.561e-1	-0.761	14.742	1.829e-1	2.695e-1	0.184	14.164	2.162e-2	8.642e-2	AAL95331.1| Phosphonates-binding protein
**FN1136**	1.345	7.825			1.407	8.072	9.993e-4	2.976e-3	0.355	6.632	3.615e-5	2.421e-5	-0.989	8.181			0.062	9.417			-1.052	8.428	2.163e-3	5.096e-3	AAL95332.1| Phosphonates transport ATP-binding protein phnC
**FN1137**																									AAL95333.1| Phosphonates transport system permease protein phnE
**FN1138**	-0.268	18.871	1.05e-1	2.414e-1	-0.681	18.642	6.319e-3	2.716e-2	0.417	19.352	2.834e-2	1.343e-1	0.684	19.288	3.659e-2	5.085e-2	-0.414	18.374	1.623e-1	2.196e-1	1.098	19.059	4.851e-3	1.445e-2	AAL95334.1| Hypothetical cytosolic protein
**FN1139**	0.086	12.492	2.847e-1	8.874e-1	1.010	13.601	1.441e-3	4.628e-3	-2.980	9.223	1e-6	1.261e-7	-3.066	9.513	5.823e-2	1.003e-1	0.924	13.686	1.044e-1	9.191e-2	-3.989	10.621	4.891e-4	7.255e-4	AAL95335.1| Activator of (R)-2-hydroxyglutaryl-CoA dehydratase
**FN1140**	1.396	7.892			1.120	7.800	2.05e-2	1.052e-1									-0.276	9.196							AAL95336.1| hypothetical protein
**FN1142**	-1.475	6.119			-1.166	6.612	3.314e-4	7.316e-4	-2.110	5.280			-0.635	4.009			0.309	5.137			-0.944	4.502			AAL95338.1| Oxygen-independent coproporphyrinogen III oxidase
**FN1143**	0.301	13.417	2.046e-1	5.737e-1	1.365	14.666	4.372e-3	1.777e-2	0.012	12.924	1.573e-1	9.45e-1	-0.289	13.429	1.993e-1	5.93e-1	1.064	14.967	6.498e-2	3.608e-2	-1.354	14.678	4.952e-3	1.485e-2	AAL95339.1| Glucosamine-6-phosphate isomerase
**FN1144**	-2.024	13.841	1.351e-3	7.472e-4	-1.023	15.026	1.15e-5	7.354e-6	-0.252	15.409	1.186e-3	3.015e-3	1.772	13.589	4.621e-3	2.264e-3	1.001	13.002	6.259e-2	3.377e-2	0.771	14.774	6.158e-5	4.586e-5	AAL95340.1| Hypothetical Exported Protein
**FN1145**	-0.160	8.872	2.423e-1	7.13e-1	-0.261	8.956	8.845e-2	5.156e-1	-0.030	8.799	1.475e-1	8.757e-1	0.131	8.843	2.379e-1	7.483e-1	-0.101	8.796	3.956e-1	8.544e-1	0.231	8.926	1.092e-1	5.303e-1	AAL95341.1| Oligoendopeptidase F
**FN1146**	-1.752	7.831							-0.320	9.059	1.974e-2	8.9e-2	1.432	7.511											AAL95342.1| Hypothetical exported 24-amino acid repeat protein
**FN1147**									1.021	9.901	9.502e-3	3.89e-2													AAL95343.1| Hypothetical protein

Log$_2$ Ratios

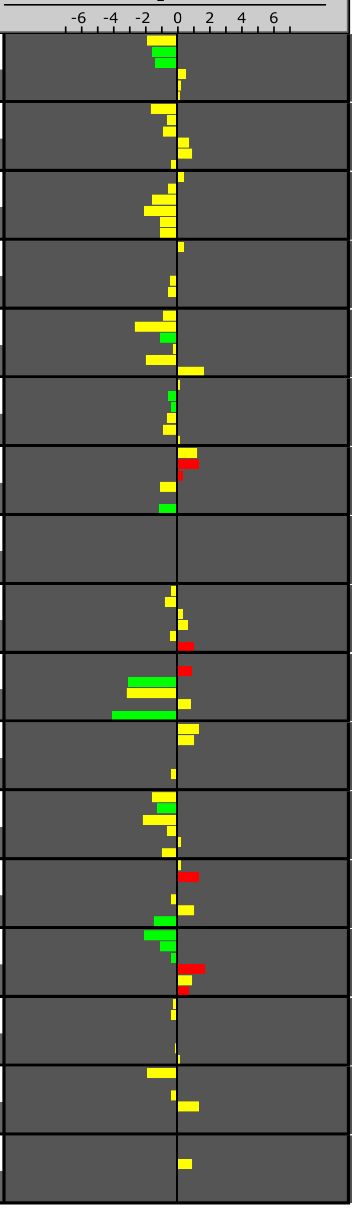

Show detected proteins only
Show all proteins
Filter by category:
GO: amino acid transport

Proteins found:
1424

Enter (or paste) list of ORFs
Find ORFs

Test	Cutoff
q-Value	.005
p-Value	

Dot Plots
Dot Plots

	Signif	Direction	Applies To
red	yes	+	ratios, bars
yellow	no	n/a	bars
green	yes	–	ratios, bars
pink	yes	+	*p*- , *q*-Values
light green	yes	–	*p*- , *q*-Values

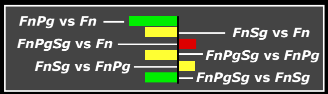

ORF	Description	FnPg vs Fn Ratio	FnPg vs Fn Sum	FnPg vs Fn *q*-Val	FnPg vs Fn *p*-Val	FnSg vs Fn Ratio	FnSg vs Fn Sum	FnSg vs Fn *q*-Val	FnSg vs Fn *p*-Val	FnPgSg vs Fn Ratio	FnPgSg vs Fn Sum	FnPgSg vs Fn *q*-Val	FnPgSg vs Fn *p*-Val	FnPgSg vs FnPg Ratio	FnPgSg vs FnPg Sum	FnPgSg vs FnPg *q*-Val	FnPgSg vs FnPg *p*-Val	FnSg vs FnPg Ratio	FnSg vs FnPg Sum	FnSg vs FnPg *q*-Val	FnSg vs FnPg *p*-Val	FnPgSg vs FnSg Ratio	FnPgSg vs FnSg Sum	FnPgSg vs FnSg *q*-Val	FnPgSg vs FnSg *p*-Val
FN1148	AAL95344.1| Serine/threonine sodium symporter	0.121	11.123	2.843e-1	8.857e-1	0.232	11.418	5.646e-3	2.379e-2	-0.087	10.711	5.158e-2	2.678e-1	-0.208	11.035	2.527e-1	8.105e-1	0.111	11.539	4.044e-1	8.864e-1	-0.319	11.331	2.232e-3	5.304e-3
FN1149	AAL95345.1| ATP-dependent nuclease subunit A	-0.897	7.170	1.237e-4	1.925e-5	-0.203	8.048	2.973e-3	1.11e-2	-1.099	6.764	5.055e-3	1.849e-2	-0.202	6.072	2.01e-1	6.004e-1	0.693	7.151	2.336e-3	1.566e-4	-0.895	6.949	1.095e-2	3.909e-2
FN1150	AAL95346.1| unknown	-1.199	5.199			-0.484	6.098			-0.739	5.455			0.459	4.459			0.715	4.900			-0.256	5.359		
FN1152	AAL95348.1| Aspartate aminotransferase	-0.607	13.861	5.176e-2	9.389e-2	-0.662	13.990	9.151e-5	1.399e-4	-0.040	14.224	9.982e-2	5.613e-1	0.567	13.821	6.229e-2	1.102e-1	-0.055	13.383	4.011e-1	8.743e-1	0.622	13.950	1.393e-3	2.945e-3
FN1153	AAL95349.1| Hypothetical protein									0.032	10.393	1.326e-1	7.737e-1												
FN1154	AAL95350.1| Ribonuclease BN	1.123	7.521			0.060	6.643											-1.063	7.766						
FN1155	AAL95351.1| Cell division protein ftsI					-1.457	5.766	5.269e-3	2.197e-2																
FN1156	AAL95352.1| Primosomal protein N'																								
FN1157	AAL95353.1| Polypeptide deformylase									0.980	7.065	1.114e-2	4.655e-2												
FN1159	AAL95355.1| Fructose-1,6-bisphosphatase	0.507	13.609	4.817e-2	8.513e-2	0.801	14.088	3.377e-4	7.494e-4	-0.035	12.863	1.354e-1	7.929e-1	-0.542	13.574	4.806e-2	7.492e-2	0.294	14.595	1.371e-1	1.56e-1	-0.836	14.052	1.714e-4	1.773e-4
FN1160	AAL95356.1| SWF/SNF family helicase	-1.457	4.627			-0.124	6.144	7.389e-2	4.24e-1	0.227	6.107	1.842e-2	8.233e-2	1.684	4.854			1.333	4.687			0.352	6.372	1.012e-2	3.554e-2
FN1161	AAL95357.1| Glutamate racemase	-2.264	5.434			-0.986	6.896	3.008e-3	1.126e-2	-1.389	6.104			0.874	4.044			1.278	4.632			-0.403	5.507		
FN1162	AAL95358.1| Hydroxyacylglutathione hydrolase	1.679	7.106			0.121	5.732											-1.558	7.411						
FN1163	AAL95359.1| Thioredoxin reductase	0.016	11.964	3.015e-1	9.621e-1	-0.499	11.635	1.334e-2	6.521e-2	0.429	12.174	1.094e-3	2.695e-3	0.413	12.393	9.506e-2	2.023e-1	-0.514	11.650	1.768e-1	2.547e-1	0.927	12.063	3.219e-3	8.388e-3
FN1164	AAL95360.1| Glucokinase	-1.042	7.626	4.611e-5	4.151e-6	-0.764	8.088	5.827e-4	1.496e-3	-1.552	6.911			-0.511	6.073			0.278	7.046	1.106e-1	1.026e-1	-0.788	6.536		
FN1165	AAL95361.1| D-galactose-binding protein	-0.474	23.494	3.39e-4	8.379e-5	0.138	24.291	6.285e-2	3.564e-1	0.707	24.471	1.037e-4	1.155e-4	1.181	24.201	5.659e-5	3.271e-6	0.612	23.817	5.094e-2	2.352e-2	0.568	24.997	2.725e-3	6.819e-3
FN1166	AAL95362.1| Galactoside transport ATP-binding protein mglA	-0.484	15.297	6.935e-3	7.123e-3	-0.107	15.858	4.275e-2	2.353e-1	-0.912	14.665	6.242e-4	1.324e-3	-0.428	14.385	5.173e-4	9.314e-5	0.377	15.375	1.558e-3	7.615e-5	-0.805	14.946	1.643e-5	6.592e-6

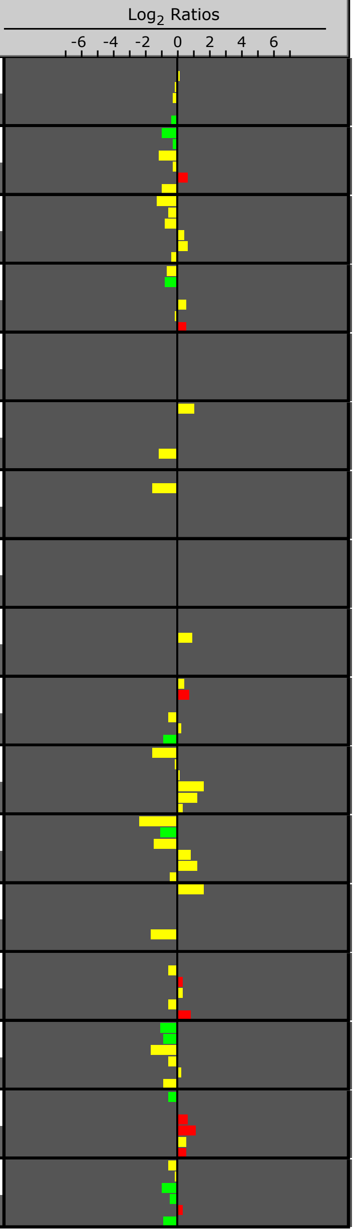

Show detected proteins only
Show all proteins
Filter by category:
GO: amino acid transport

Proteins found: 1424

Enter (or paste) list of ORFs
Find ORFs

Test	Cutoff
q-Value	.005
p-Value	

Dot Plots
Dot Plots

	Signif	Direction	Applies To
red	yes	+	ratios, bars
yellow	no	n/a	bars
green	yes	–	ratios, bars
pink	yes	+	*p*- , *q*-Values
light green	yes	–	*p*- , *q*-Values

FnPg vs Fn — FnSg vs Fn
FnPgSg vs Fn — FnPgSg vs FnPg
FnSg vs FnPg — FnPgSg vs FnSg

ORF	Protein	*FnPg vs Fn* Ratio	Sum	*q*-Val	*p*-Val	*FnSg vs Fn* Ratio	Sum	*q*-Val	*p*-Val	*FnPgSg vs Fn* Ratio	Sum	*q*-Val	*p*-Val	*FnPgSg vs FnPg* Ratio	Sum	*q*-Val	*p*-Val	*FnSg vs FnPg* Ratio	Sum	*q*-Val	*p*-Val	*FnPgSg vs FnSg* Ratio	Sum	*q*-Val	*p*-Val
**FN1167**	AAL95363.1| Galactoside transport system permease protein mglC					-1.960	9.059	1.49e-2	7.405e-2	-0.858	9.773	4.028e-2	2.029e-1									1.102	8.201	4.179e-3	1.182e-2
**FN1169**	AAL95365.1| L-lactate dehydrogenase	-0.356	16.815	4.448e-2	7.633e-2	-0.078	17.277	1.066e-1	6.339e-1	-0.641	16.326	5.206e-5	4.348e-5	-0.285	16.174	9.312e-2	1.958e-1	0.278	16.921	1.73e-1	2.456e-1	-0.563	16.637	1.318e-2	4.862e-2
**FN1170**	AAL95366.1| Pyruvate-flavodoxin oxidoreductase	0.506	25.229	7.054e-2	1.441e-1	0.364	25.271	8.773e-6	4.768e-6	-0.301	24.218	6.627e-4	1.43e-3	-0.807	24.928	4.348e-2	6.47e-2	-0.142	25.777	3.064e-1	5.768e-1	-0.665	24.970	2.019e-5	9.477e-6
**FN1171**	AAL95367.1| Acetate kinase	0.209	19.881	1.436e-1	3.589e-1	-0.013	19.843	1.435e-1	8.903e-1	-0.180	19.288	7.07e-3	2.748e-2	-0.388	19.701	7.863e-2	1.521e-1	-0.222	20.052	2.133e-1	3.435e-1	-0.167	19.664	3.173e-2	1.328e-1
**FN1172**	AAL95368.1| Phosphate acetyltransferase	-0.630	18.578	1.192e-4	1.8e-5	-0.600	18.793	4.171e-5	4.682e-5	-0.131	18.873	1.678e-2	7.399e-2	0.499	18.447	2.845e-3	1.133e-3	0.030	18.163	3.338e-1	6.54e-1	0.469	18.661	7.401e-4	1.293e-3
**FN1176**	AAL95372.1| Hypothetical cytosolic protein																								
**FN1179**	AAL95375.1| ATP-dependent RNA helicase	1.870	6.653			0.101	5.069											-1.769	6.939						
**FN1180**	AAL95376.1| Hypothetical protein	0.361	7.191			-0.700	6.314											-1.061	6.676						
**FN1181**	AAL95377.1| unknown	1.353	15.098	1.258e-1	3.03e-1	-0.991	12.938	1.55e-4	2.816e-4	-0.603	12.939	1.358e-3	3.661e-3	-1.956	14.495	1.022e-1	2.275e-1	-2.344	14.291	1.54e-1	2.001e-1	0.388	12.335	1.265e-2	4.632e-2
**FN1182**	AAL95378.1| Hypothetical protein	0.348	7.204			-0.935	6.105											-1.284	6.453						
**FN1183**	AAL95379.1| Hypothetical cytosolic protein	1.050	6.875	1.941e-2	2.608e-2	0.873	6.883	1.428e-2	7.06e-2	-0.372	5.250	3.84e-2	1.919e-1	-1.422	6.503	1.305e-2	1.13e-2	-0.176	7.933	3.097e-1	5.857e-1	-1.245	6.511	9.701e-3	3.377e-2
**FN1184**	AAL95380.1| Hypothetical protein																								
**FN1185**	AAL95381.1| SIR2 family protein	0.578	10.599	1.313e-1	3.2e-1	-0.111	10.095	5.219e-2	2.924e-1	-0.022	9.796	1.28e-1	7.429e-1	-0.600	10.578	1.248e-1	3.079e-1	-0.689	10.673	1.81e-1	2.649e-1	0.090	10.073	7.936e-2	3.729e-1
**FN1186**	AAL95382.1| Amidohydrolase	4.221	12.526	4.992e-2	8.934e-2	4.461	12.950	1.95e-5	1.59e-5	0.571	8.672	1.738e-2	7.7e-2	-3.651	13.097	5.602e-2	9.491e-2	0.240	17.171	3.387e-1	6.688e-1	-3.890	13.521	1.311e-5	4.501e-6
**FN1187**	AAL95383.1| Amino acid-binding protein													-0.476	10.886	1.992e-1	5.927e-1	0.650	12.196	2.121e-1	3.408e-1	-1.126	11.721	1.251e-2	4.572e-2
**FN1188**	AAL95384.1| Hypothetical protein	-0.936	11.845	5.604e-2	1.05e-1	0.226	13.192	1.158e-2	5.527e-2	-0.774	11.803	1.582e-3	4.52e-3	0.162	11.071	2.475e-1	7.886e-1	1.162	12.256	7.563e-2	4.809e-2	-1.000	12.418	2.38e-4	2.799e-4
**FN1189**	AAL95385.1| Hypothetical protein	-0.482	12.482	1.891e-3	1.186e-3	-0.599	12.549	5.336e-3	2.229e-2	-0.844	11.917	9.974e-5	1.086e-4	-0.362	11.639	4.426e-4	7.239e-5	-0.118	12.067	2.978e-1	5.538e-1	-0.244	11.706	6.726e-2	3.104e-1

Log_2 Ratios: -6 -4 -2 0 2 4 6

Show detected proteins only
Show all proteins
Filter by category:
GO: amino acid transport

Proteins found: 1424

Enter (or paste) list of ORFs

Find ORFs

Test	Cutoff
q-Value	.005
p-Value	

Dot Plots
Dot Plots

	Signif	Direction	Applies To
(red)	yes	+	ratios, bars
(yellow)	no	n/a	bars
(green)	yes	–	ratios, bars
(pink)	yes	+	*p*- , *q*-Values
(light green)	yes	–	*p*- , *q*-Values

FnPg vs Fn — FnSg vs Fn
FnPgSg vs Fn — FnPgSg vs FnPg
FnSg vs FnPg — FnPgSg vs FnSg

ORF	Description	*FnPg vs Fn* Ratio	Sum	*q*-Val	*p*-Val	*FnSg vs Fn* Ratio	Sum	*q*-Val	*p*-Val	*FnPgSg vs Fn* Ratio	Sum	*q*-Val	*p*-Val	*FnPgSg vs FnPg* Ratio	Sum	*q*-Val	*p*-Val	*FnSg vs FnPg* Ratio	Sum	*q*-Val	*p*-Val	*FnPgSg vs FnSg* Ratio	Sum	*q*-Val	*p*-Val
FN1190	AAL95386.1| Probable cadmium-transporting ATPase	-0.937	13.526	2.184e-5	1.409e-6	0.102	14.748	2.297e-3	8.066e-3	-0.773	13.486	2.781e-5	1.491e-5	0.164	12.753	4.181e-2	6.12e-2	1.038	13.812	8.211e-5	4.721e-7	-0.874	13.976	1.307e-5	4.48e-6
FN1191	AAL95387.1| unknown	0.044	10.940			1.230	12.311	5.734e-3	2.423e-2	-1.310	9.382	1.205e-4	1.418e-4	-1.354	9.630			1.187	12.355			-2.540	11.001	3.237e-3	8.446e-3
FN1192	AAL95388.1| unknown	0.086	16.985	2.075e-1	5.839e-1	0.877	17.961	2.91e-3	1.08e-2	0.899	17.594	2.076e-4	3.074e-4	0.813	17.884	2.569e-3	9.785e-4	0.791	18.046	2.599e-2	7.173e-3	0.022	18.859	1.68e-1	8.733e-1
FN1198	AAL95394.1| Transporter	0.382	9.619	6.09e-2	1.185e-1	1.280	10.701	1.881e-5	1.503e-5	-0.032	9.001	8.343e-2	4.585e-1	-0.414	9.587	5.934e-2	1.031e-1	0.898	11.084	1.113e-2	2.001e-3	-1.312	10.670	5.405e-5	3.859e-5
FN1200	AAL95396.1| Hypothetical protein													-5.133	12.533			-0.742	17.108			-4.391	11.975	5.214e-4	7.921e-4
FN1201	AAL95397.1| unknown																								
FN1202	AAL95398.1| NH(3)-dependent NAD(+) synthetase	1.492	8.053			1.295	8.041			0.633	6.991			-0.858	8.687	6.926e-3	4.136e-3	-0.197	9.533	2.819e-1	5.125e-1	-0.662	8.674	3.534e-2	1.502e-1
FN1203	AAL95399.1| GTP-binding protein	-0.717	6.710			-0.412	7.199											0.305	6.482	2.465e-1	4.265e-1				
FN1204	AAL95400.1| Methyltransferase	0.388	7.090	7.563e-2	1.577e-1	0.512	7.398	1.83e-2	9.29e-2									0.124	7.786	2.531e-1	4.425e-1				
FN1205	AAL95401.1| Protease	-0.683	12.230	6.338e-2	1.254e-1	-0.761	12.337	9.387e-3	4.337e-2	-0.383	12.326	4.428e-3	1.578e-2	0.300	11.847	1.744e-1	4.912e-1	-0.078	11.654	4.044e-1	8.864e-1	0.378	11.953	5.801e-2	2.633e-1
FN1206	AAL95402.1| Hemolysin																								
FN1208	AAL95404.1| 1-deoxyxylulose-5-phosphate synthase	-0.395	6.435	3.627e-2	5.794e-2	0.580	7.594			-0.991	5.635			-0.596	5.444			0.975	7.199			-1.571	6.604		
FN1209	AAL95405.1| Hypothetical RNA binding protein	-2.359	10.173			-1.872	10.844	2.67e-3	9.698e-3	-0.479	11.848	3.301e-2	1.609e-1	1.880	9.694			0.487	8.485			1.393	10.365	1.062e-2	3.767e-2
FN1210	AAL95406.1| Metal dependent hydrolase	-1.499	11.414	1.487e-2	1.838e-2	-1.032	12.066	5.966e-3	2.538e-2	-1.246	11.463	5.322e-3	1.97e-2	0.253	10.168	1.046e-1	2.361e-1	0.467	10.567	1.481e-1	1.847e-1	-0.214	10.820	1.025e-1	4.939e-1
FN1211	AAL95407.1| Cell division protein ftsI	-1.699	7.067	5.425e-4	1.742e-4	-0.699	8.252	3.479e-3	1.353e-2	-0.425	8.138	4.291e-2	2.179e-1	1.275	6.642	4.827e-2	7.542e-2	1.001	6.553	6.418e-2	3.53e-2	0.274	7.827	1.073e-1	5.2e-1
FN1213	AAL95409.1| Hypothetical protein	-1.264	11.300	7.2e-3	7.517e-3	-0.421	12.327	2.328e-3	8.199e-3	-0.785	11.575	2.858e-4	4.737e-4	0.479	10.515	1.034e-1	2.319e-1	0.842	11.063	8.008e-2	5.416e-2	-0.364	11.542	5.795e-3	1.813e-2
FN1214	AAL95410.1| Fe-S oxidoreductase																								

Log$_2$ Ratios

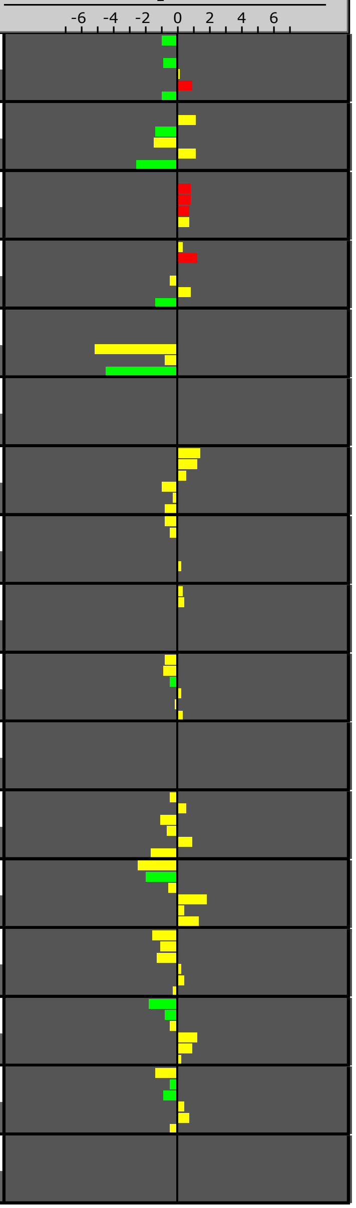

- Show detected proteins only
- Show all proteins
- Filter by category:
- GO: amino acid transport

Proteins found: 1424

Enter (or paste) list of ORFs

Find ORFs

Test	Cutoff
q-Value	.005
p-Value	

Dot Plots

Dot Plots

	Signif	Direction	Applies To
(red)	yes	+	ratios, bars
(yellow)	no	n/a	bars
(green)	yes	–	ratios, bars
(pink)	yes	+	*p*- , *q*-Values
(light green)	yes	–	*p*- , *q*-Values

FnPg vs Fn
FnPgSg vs Fn
FnSg vs FnPg
FnSg vs Fn
FnPgSg vs FnPg
FnPgSg vs FnSg

ORF	Protein	*FnPg vs Fn*				*FnSg vs Fn*				*FnPgSg vs Fn*				*FnPgSg vs FnPg*				*FnSg vs FnPg*				*FnPgSg vs FnSg*			
		Ratio	Sum	*q*-Val	*p*-Val	Ratio	Sum	*q*-Val	*p*-Val	Ratio	Sum	*q*-Val	*p*-Val	Ratio	Sum	*q*-Val	*p*-Val	Ratio	Sum	*q*-Val	*p*-Val	Ratio	Sum	*q*-Val	*p*-Val
**FN1216**	AAL95412.1| RRF2 family protein	-1.368	8.377	1.593e-2	2.007e-2	-0.881	9.049	1.107e-2	5.249e-2	-2.043	7.499	2.413e-3	7.568e-3	-0.675	6.335	8.412e-2	1.678e-1	0.487	7.681	7.205e-2	4.373e-2	-1.162	7.006	8.666e-3	2.956e-2
**FN1217**	AAL95413.1| Holliday junction DNA helicase ruvB	-1.747	7.361			-0.122	9.171	9.118e-2	5.332e-1	-2.308	6.597	1.333e-3	3.563e-3	-0.561	5.054			1.625	7.424			-2.186	6.863	7.813e-4	1.393e-3
**FN1218**	AAL95414.1| unknown																	0.374	7.959						
**FN1219**	AAL95415.1| Hypothetical protein	0.350	7.953	5.396e-2	9.952e-2	0.075	7.863	9.847e-2	5.804e-1	0.422	7.821	3.929e-2	1.971e-1	0.072	8.375	2.487e-1	7.936e-1	-0.275	8.213	1.35e-1	1.511e-1	0.347	8.285	5.689e-2	2.576e-1
**FN1220**	AAL95416.1| Cysteine synthase	-0.155	15.303	5.815e-2	1.107e-1	0.134	15.776	9.04e-2	5.281e-1	0.018	15.272	1.344e-1	7.861e-1	0.173	15.321	5.507e-2	9.256e-2	0.289	15.621	1.709e-1	2.404e-1	-0.116	15.794	1.186e-1	5.823e-1
**FN1221**	AAL95417.1| Hypothetical protein	0.131	8.428	2.718e-1	8.321e-1	0.628	9.110	1.698e-2	8.559e-2	0.092	8.185	8.491e-2	4.678e-1	-0.040	8.520	2.832e-1	9.47e-1	0.497	9.241	2.284e-1	3.808e-1	-0.537	9.201	2.795e-2	1.15e-1
**FN1222**	AAL95418.1| Hypothetical protein	-1.403	8.987	3.455e-4	8.614e-5	-2.965	7.609			-1.412	8.774	5.603e-4	1.153e-3	-0.009	7.575	2.889e-1	9.741e-1	-1.562	6.206			1.553	6.197		
**FN1223**	AAL95419.1| Oxygen-insensitive NAD(P)H nitroreductase	0.428	12.381	5.721e-2	1.081e-1	-1.197	10.940	1.036e-5	6.173e-6	0.552	12.301	1.365e-4	1.694e-4	0.124	12.932	1.753e-1	4.947e-1	-1.625	11.368	2.843e-2	8.322e-3	1.749	11.492	7.912e-6	1.933e-6
**FN1224**	AAL95420.1| 2-dehydro-3-deoxyphosphooctonate aldolase	-0.623	13.846	5.546e-2	1.034e-1	-1.022	13.632	4.285e-4	1.006e-3	-0.646	13.620	8.024e-4	1.807e-3	-0.022	13.201	2.847e-1	9.542e-1	-0.398	13.009	2.364e-1	4.016e-1	0.376	12.987	2.757e-2	1.133e-1
**FN1225**	AAL95421.1| UDP-N-acetylmuramoyl-L-alanyl-D-glutamate--meso-lanthionine ligase	-0.240	13.494	1.81e-1	4.893e-1	-0.427	13.491	1.198e-2	5.752e-2	0.346	13.875	9.908e-3	4.08e-2	0.586	13.839	5.862e-2	1.013e-1	-0.187	13.251	3.249e-1	6.283e-1	0.773	13.837	6.241e-4	1.028e-3
**FN1226**	AAL95422.1| Uracil-DNA glycosylase	-1.125	13.443	2.289e-2	3.208e-2	0.365	15.117	2.48e-2	1.297e-1	-0.774	13.590	2.599e-7	2.069e-8	0.351	12.669	1.612e-1	4.415e-1	1.490	13.992	2.328e-2	5.925e-3	-1.139	14.343	4.941e-3	1.48e-2
**FN1229**	AAL95425.1| Hypothetical protein																								
**FN1230**	AAL95426.1| Hypothetical cytosolic protein									0.003	11.544	1.62e-1	9.785e-1												
**FN1231**	AAL95427.1| Inosine-5'-monophosphate dehydrogenase	0.451	18.536	1.967e-1	5.46e-1	0.921	19.191	1.093e-4	1.752e-4	0.337	18.219	1.457e-3	4.047e-3	-0.113	18.873	2.642e-1	8.607e-1	0.471	19.642	2.362e-1	4.012e-1	-0.584	19.529	3.977e-5	2.495e-5
**FN1233**	AAL95429.1| Putative NAD(P)H oxidoreductase					0.340	4.830																		
**FN1234**	AAL95430.1| Hypothetical protein					0.160	8.625			-1.190	6.887	2.012e-5	9.116e-6									-1.350	7.435		
**FN1235**	AAL95431.1| Ankyrin repeat proteins	1.403	7.579			1.768	8.128			0.553	6.525			-0.850	8.132	1.028e-1	2.297e-1	0.365	9.531	2.504e-1	4.362e-1	-1.215	8.682	5.925e-3	1.866e-2

Log$_2$ Ratios

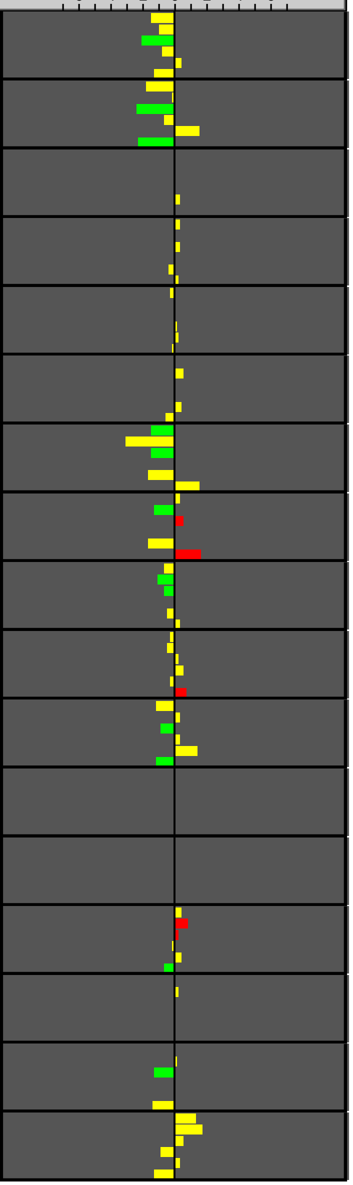

Show detected proteins only
Show all proteins
Filter by category:
GO: amino acid transport

Proteins found: 1424

Enter (or paste) list of ORFs

Find ORFs

Test	Cutoff
q-Value	.005
p-Value	

Dot Plots
Dot Plots

	Signif	Direction	Applies To
red	yes	+	ratios, bars
yellow	no	n/a	bars
green	yes	–	ratios, bars
pink	yes	+	*p*- , *q*-Values
light green	yes	–	*p*- , *q*-Values

FnPg vs Fn
FnPgSg vs Fn
FnSg vs FnPg
FnSg vs Fn
FnPgSg vs FnPg
FnPgSg vs FnSg

ORF	Protein	FnPg vs Fn Ratio	FnPg vs Fn Sum	FnPg vs Fn q-Val	FnPg vs Fn p-Val	FnSg vs Fn Ratio	FnSg vs Fn Sum	FnSg vs Fn q-Val	FnSg vs Fn p-Val	FnPgSg vs Fn Ratio	FnPgSg vs Fn Sum	FnPgSg vs Fn q-Val	FnPgSg vs Fn p-Val	FnPgSg vs FnPg Ratio	FnPgSg vs FnPg Sum	FnPgSg vs FnPg q-Val	FnPgSg vs FnPg p-Val	FnSg vs FnPg Ratio	FnSg vs FnPg Sum	FnSg vs FnPg q-Val	FnSg vs FnPg p-Val	FnPgSg vs FnSg Ratio	FnPgSg vs FnSg Sum	FnPgSg vs FnSg q-Val	FnPgSg vs FnSg p-Val
FN1237	AAL95433.1| Choline kinase	1.575	10.956	1.626e-3	9.569e-4	2.287	11.853	2.375e-4	4.73e-4	0.144	9.322	1.478e-2	6.409e-2	-1.431	11.101	2.257e-3	8.196e-4	0.712	13.428	6.986e-3	9.988e-4	-2.143	11.997	3.259e-4	4.31e-4
FN1238	AAL95434.1| Hypothetical protein																	-0.414	4.414						
FN1239	AAL95435.1| Hypothetical protein																								
FN1240	AAL95436.1| Lipopolysaccharide core biosynthesis protein rfaY	-1.829	8.757	4.347e-4	1.226e-4	-2.598	8.173	6.78e-5	9.412e-5	-2.083	8.300	2.962e-5	1.66e-5	-0.254	6.675	1.82e-1	5.212e-1	-0.769	6.344	1.319e-1	1.439e-1	0.515	6.090	1.651e-2	6.312e-2
FN1241	AAL95437.1| polysaccharide biosynthesis protein	-0.139	5.668	2.521e-1	7.514e-1	0.491	6.482	1.888e-2	9.616e-2									0.629	6.343	1.099e-1	1.014e-1				
FN1242	AAL95438.1| Polysaccharide deacetylase	0.657	7.683			1.104	8.315	6.253e-5	8.35e-5	-0.936	5.887	2.737e-3	8.818e-3	-1.592	6.748			0.448	8.972			-2.040	7.380	2.034e-5	9.613e-6
FN1243	AAL95439.1| Glycosyl transferase	0.021	7.594			-0.234	7.523	3.163e-2	1.697e-1	-0.940	6.429	2.902e-3	9.485e-3	-0.961	6.654			-0.255	7.544			-0.706	6.584	4.124e-5	2.651e-5
FN1244	AAL95440.1| Polysaccharide deacetylase	0.587	8.322	1.743e-1	4.643e-1	0.771	8.692	3.719e-3	1.473e-2	-1.254	6.278	2.128e-3	6.519e-3	-1.841	7.068	8.382e-2	1.669e-1	0.185	9.278	3.677e-1	7.594e-1	-2.025	7.437	1.644e-3	3.627e-3
FN1245	AAL95441.1| Glycosyl transferase	-2.175	6.550	1.589e-2	2.001e-2	-1.567	7.344	7.032e-3	3.087e-2									0.609	5.168	4.202e-3	4.429e-4				
FN1246	AAL95442.1| Lipooligosaccharide cholinephosphotransferase	-0.372	8.186			-0.141	8.601	7.953e-2	4.591e-1	-2.234	6.119			-1.862	5.951			0.231	8.229			-2.093	6.366		
FN1247	AAL95443.1| LOS biosynthesis enzyme LBGB	-0.619	8.705	3.85e-2	6.267e-2	0.349	9.857	5.002e-2	2.792e-1	-1.668	7.452	5.921e-4	1.237e-3	-1.049	7.036	4.859e-2	7.617e-2	0.968	9.238	7.704e-2	4.994e-2	-2.017	8.189	4.351e-3	1.25e-2
FN1248	AAL95444.1| Hypothetical cytosolic protein																								
FN1250	AAL95446.1| Guanine-hypoxanthine permease	-1.376	9.925	1.084e-3	5.351e-4	-1.336	10.150	4.866e-4	1.184e-3	-1.028	10.069	5.004e-4	1.001e-3	0.348	8.897	1.072e-1	2.464e-1	0.040	8.774	4.033e-1	8.823e-1	0.308	9.122	3.567e-2	1.518e-1
FN1251	AAL95447.1| High-affinity iron permease	-0.530	6.145			0.025	6.884	1.53e-1	9.601e-1	-0.127	6.344	8.453e-2	4.654e-1	0.403	6.018			0.555	6.354			-0.152	6.757	1.525e-1	7.785e-1
FN1252	AAL95448.1| 34 kDa membrane antigen precursor	-1.973	17.489	1.155e-3	5.951e-4	-1.187	18.460	1.159e-4	1.892e-4	-0.402	18.857	4.009e-3	1.397e-2	1.571	17.087	3.862e-3	1.786e-3	0.786	16.487	5.381e-2	2.628e-2	0.785	18.058	1.531e-3	3.314e-3
FN1253	AAL95449.1| unknown	-0.833	16.061	6.623e-2	1.328e-1	-0.732	16.347	1.505e-4	2.701e-4	-0.279	16.411	8.377e-3	3.349e-2	0.554	15.782	1.26e-1	3.123e-1	0.102	15.514	3.977e-1	8.62e-1	0.452	16.068	4.431e-3	1.282e-2
FN1254	AAL95450.1| Oxygen-insensitive NAD(P)H nitroreductase	-0.817	12.908	2.362e-2	3.338e-2	-1.036	12.873	8.79e-5	1.331e-4	0.021	13.541	1.426e-1	8.416e-1	0.837	12.928	2.476e-2	2.872e-2	-0.219	12.056	3.057e-1	5.75e-1	1.056	12.894	2.61e-4	3.21e-4

Log$_2$ Ratios

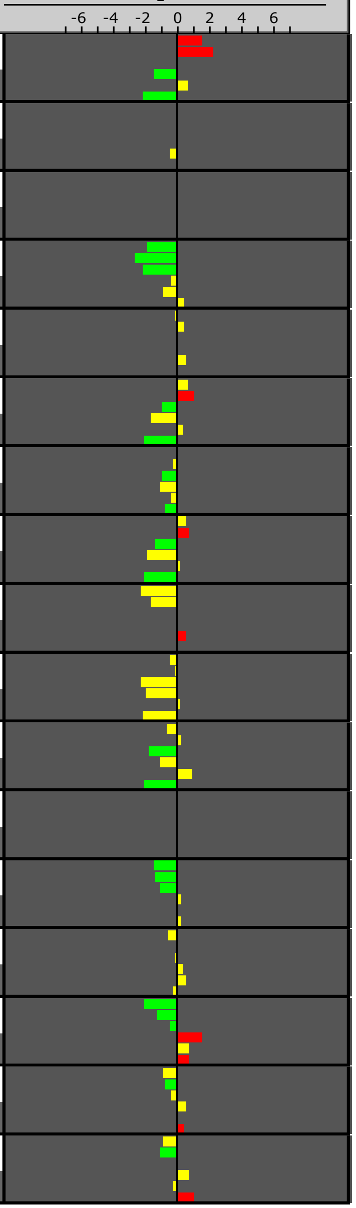

Show detected proteins only
Show all proteins
Filter by category:
GO: amino acid transport

Proteins found: 1424

Enter (or paste) list of ORFs

Find ORFs

Test	Cutoff
q-Value	.005
p-Value	

Dot Plots
Dot Plots

	Signif	Direction	Applies To
red	yes	+	ratios, bars
yellow	no	n/a	bars
green	yes	–	ratios, bars
pink	yes	+	p- , q-Values
light green	yes	–	p- , q-Values

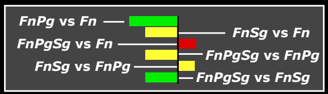

Fusobacterium nucleatum

Hackett Laboratory UW

	FnPg vs *Fn*				*FnSg* vs *Fn*				*FnPgSg* vs *Fn*				*FnPgSg* vs *FnPg*				*FnSg* vs *FnPg*				*FnPgSg* vs *FnSg*				
ORF	Ratio	Sum	*q*-Val	*p*-Val	Ratio	Sum	*q*-Val	*p*-Val	Ratio	Sum	*q*-Val	*p*-Val	Ratio	Sum	*q*-Val	*p*-Val	Ratio	Sum	*q*-Val	*p*-Val	Ratio	Sum	*q*-Val	*p*-Val	Description
**FN1255**																									AAL95451.1| NagD protein
**FN1256**	-0.603	4.603							-0.558	4.443			0.044	4.044											AAL95452.1| C4-dicarboxylate transporter large subunit
**FN1258**	-0.787	19.783	2.008e-3	1.297e-3	-0.557	20.198	1.495e-4	2.677e-4	-0.263	20.103	6.578e-5	5.964e-5	0.524	19.520	1.03e-2	7.915e-3	0.230	19.411	1.1e-1	1.015e-1	0.294	19.935	2.606e-3	6.457e-3	AAL95454.1| C4-dicarboxylate-binding protein
**FN1259**																									AAL95455.1| hypothetical protein
**FN1260**									-0.594	3.764															AAL95456.1| Sensory Transduction Protein Kinase
**FN1261**	1.385	6.168																							AAL95457.1| Two-component response regulator
**FN1262**	1.719	6.924			1.407	6.796											-0.313	8.516							AAL95458.1| Integral membrane protein
**FN1263**	-0.934	8.230	4.534e-3	3.968e-3	-0.159	9.189	1.724e-2	8.706e-2	-0.555	8.405	3.136e-6	6.101e-7	0.379	7.675	5.1e-2	8.2e-2	0.775	8.255	1.612e-2	3.402e-3	-0.396	8.634	4.722e-3	1.395e-2	AAL95459.1| Cobalt chelatase
**FN1264**					-0.747	8.802	6.873e-3	3.003e-2	-1.171	7.990	3.768e-3	1.295e-2									-0.424	7.631	2.497e-4	3.004e-4	AAL95460.1| Hypothetical protein
**FN1265**	0.033	13.133	2.501e-1	7.434e-1	-0.208	13.076	3.209e-2	1.724e-1	-0.502	12.394	7.692e-3	3.031e-2	-0.535	12.631	2.142e-2	2.341e-2	-0.241	13.109	1.249e-1	1.294e-1	-0.294	12.574	4.543e-2	2e-1	AAL95461.1| Outer membrane protein
**FN1266**	-0.752	14.116	4.763e-3	4.265e-3	-0.726	14.326	1.444e-3	4.639e-3	-0.561	14.103	1.174e-3	2.972e-3	0.191	13.555	1.125e-1	2.645e-1	0.026	13.574	4.062e-1	8.932e-1	0.165	13.765	6.992e-2	3.243e-1	AAL95462.1| UTP--glucose-1-phosphate uridylyltransferase
**FN1267**	0.132	10.711	1.759e-1	4.702e-1	-0.323	10.440	3.336e-2	1.8e-1	-0.318	10.057	2.622e-7	2.119e-8	-0.450	10.393	5.226e-2	8.518e-2	-0.455	10.572	1.202e-1	1.199e-1	0.005	10.122	1.85e-1	9.817e-1	AAL95463.1| Hypothetical protein
**FN1268**	-0.507	15.890	1.852e-2	2.451e-2	-1.071	15.511	1.415e-3	4.529e-3	-0.610	15.583	4.232e-3	1.493e-2	-0.103	15.280	3.814e-2	5.386e-2	-0.563	15.004	6.929e-4	1.394e-5	0.461	14.901	1.65e-4	1.688e-4	AAL95464.1| Methionyl-tRNA synthetase
**FN1269**	-2.042	6.042			-0.946	7.322			-1.997	5.882			0.044	4.044			1.095	5.280			-1.051	5.324			AAL95465.1| Hypothetical lipoprotein
**FN1270**	-2.654	11.438			-3.588	10.689			0.528	14.416	4.406e-4	8.485e-4	3.182	11.966			-0.934	8.035			4.116	11.216			AAL95466.1| Hypothetical cytosolic protein
**FN1271**	-0.989	10.819	3.867e-2	6.304e-2	-0.271	11.721	3.333e-2	1.799e-1	-0.310	11.294	4.592e-3	1.648e-2	0.679	10.509	8.683e-2	1.759e-1	0.718	10.732	1.407e-1	1.649e-1	-0.039	11.411	1.637e-1	8.465e-1	AAL95467.1| Protease IV
**FN1273**	0.174	10.980	2.748e-1	8.449e-1	0.531	11.522	2.203e-2	1.139e-1	-1.363	9.239	1.301e-2	5.546e-2	-1.537	9.617	1.198e-1	2.898e-1	0.357	11.696	3.223e-1	6.208e-1	-1.894	10.159	3.127e-7	2.423e-8	AAL95469.1| Outer membrane protein tolC

Log$_2$ Ratios

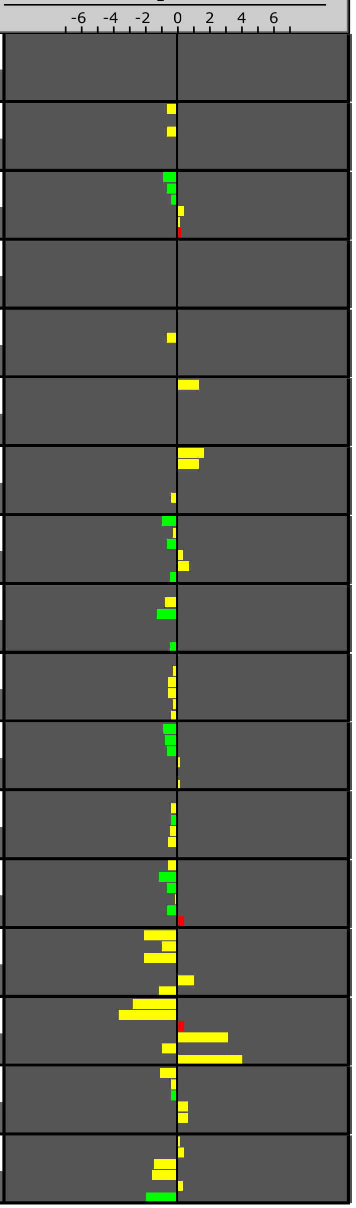

Show detected proteins only
Show all proteins
Filter by category:
GO: amino acid transport

Proteins found: 1424

Enter (or paste) list of ORFs

Find ORFs

Test	Cutoff
q-Value	.005
p-Value	

Dot Plots
Dot Plots

	Signif	Direction	Applies To
(red)	yes	+	ratios, bars
(yellow)	no	n/a	bars
(green)	yes	–	ratios, bars
(pink)	yes	+	*p*- , *q*-Values
(light green)	yes	–	*p*- , *q*-Values

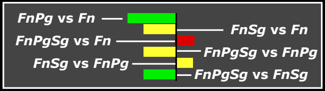

ORF	Description	FnPg vs Fn				FnSg vs Fn				FnPgSg vs Fn				FnPgSg vs FnPg				FnSg vs FnPg				FnPgSg vs FnSg			
		Ratio	Sum	*q*-Val	*p*-Val	Ratio	Sum	*q*-Val	*p*-Val	Ratio	Sum	*q*-Val	*p*-Val	Ratio	Sum	*q*-Val	*p*-Val	Ratio	Sum	*q*-Val	*p*-Val	Ratio	Sum	*q*-Val	*p*-Val
FN1274	AAL95470.1| Acriflavin resistance protein E	-2.178	9.111	8.321e-4	3.478e-4	-1.250	10.223	5.095e-4	1.258e-3	-1.745	9.340	4.319e-4	8.273e-4	0.433	7.366	1.519e-1	4.081e-1	0.927	8.045	1.006e-1	8.603e-2	-0.494	8.478	8.989e-4	1.686e-3
FN1275	AAL95471.1| Acriflavin resistance protein B	-1.966	9.836	2.602e-4	5.783e-5	-1.572	10.413	2.811e-4	5.884e-4	-1.300	10.297	7.017e-5	6.519e-5	0.666	8.536	4.553e-2	6.916e-2	0.393	8.448	1.715e-1	2.418e-1	0.273	9.113	3.324e-2	1.4e-1
FN1276	AAL95472.1| Hypothetical protein													-1.157	7.168	3.697e-3	1.684e-3	0.028	8.537	3.846e-1	8.16e-1	-1.184	7.380	7.721e-4	1.37e-3
FN1277	AAL95473.1| Aminoacyl-histidine dipeptidase	0.370	15.287	1.416e-1	3.524e-1	0.267	15.369	6.773e-3	2.95e-2	0.072	14.785	6.748e-2	3.611e-1	-0.298	15.359	1.58e-1	4.298e-1	-0.103	15.739	3.686e-1	7.621e-1	-0.195	15.440	1.632e-2	6.224e-2
FN1278	AAL95474.1| Acetyltransferase													2.044	5.214			1.782	5.137			0.262	7.181		
FN1279	AAL95475.1| Zinc metallohydrolase, glyoxalase II family	-0.542	12.225	3.008e-3	2.25e-3	-0.890	12.061	1.02e-4	1.603e-4	-0.736	11.827	8.152e-4	1.842e-3	-0.194	11.489	8.004e-2	1.56e-1	-0.348	11.520	6.965e-2	4.101e-2	0.154	11.326	2.489e-2	1.01e-1
FN1280	AAL95476.1| Serine protease, V8 family	2.279	9.432	9.521e-5	1.248e-5	3.038	10.375	3.156e-4	6.873e-4	0.286	7.234	1.051e-2	4.36e-2	-1.994	9.717	5.39e-4	9.995e-5	0.758	12.654	2.161e-2	5.248e-3	-2.752	10.660	5.523e-4	8.591e-4
FN1281	AAL95477.1| Cysteine protease	-0.305	8.518	8.875e-2	1.946e-1	0.629	9.636	4.332e-3	1.759e-2									0.934	9.331	1.248e-3	4.666e-5				
FN1282	AAL95478.1| LSU ribosomal protein L17P	0.471	12.375	8.354e-2	1.801e-1	0.250	12.338	1.182e-2	5.664e-2	0.704	12.404	1.554e-5	6.064e-6	0.233	13.079	1.369e-1	3.545e-1	-0.221	12.809	2.555e-1	4.482e-1	0.455	13.042	1.408e-3	2.984e-3
FN1283	AAL95479.1| DNA-directed RNA polymerase alpha chain	-0.083	17.505	1.086e-1	2.515e-1	0.423	18.195	1.589e-2	7.951e-2	1.182	18.566	6.679e-6	1.76e-6	1.265	18.687	8.716e-5	6.423e-6	0.506	18.113	7.992e-2	5.392e-2	0.759	19.378	2.37e-3	5.732e-3
FN1284	AAL95480.1| SSU ribosomal protein S4P	-0.803	18.736	3.716e-2	5.98e-2	-1.208	18.516	3.448e-7	3.598e-8	-0.356	18.980	3.92e-5	2.886e-5	0.448	18.380	1.071e-1	2.46e-1	-0.404	17.713	2.38e-1	4.058e-1	0.852	18.160	1.686e-5	6.882e-6
FN1285	AAL95481.1| SSU ribosomal protein S11P	1.408	14.708	1.326e-1	3.242e-1	-2.246	11.238	2.49e-5	2.234e-5	0.344	13.440	1.377e-3	3.737e-3	-1.064	15.052	1.489e-1	3.973e-1	-3.654	12.646	1.466e-1	1.806e-1	2.590	11.582	1.366e-4	1.301e-4
FN1286	AAL95482.1| SSU ribosomal protein S13P	-2.940	14.401	1.158e-4	1.712e-5	-2.156	15.369	1.562e-6	3.129e-7	-1.897	15.240	3.128e-6	6.078e-7	1.043	12.504	1.179e-2	9.704e-3	0.784	12.429	2.419e-2	6.321e-3	0.259	13.472	4.274e-2	1.867e-1
FN1287	AAL95483.1| Bacterial Protein Translation Initiation Factor 1 (IF-1)	-1.021	9.866	2.957e-2	4.434e-2	-2.227	8.844	5.401e-5	6.771e-5	-2.396	8.287	3.07e-5	1.768e-5	-1.375	7.470	7.396e-2	1.394e-1	-1.206	7.823	1.397e-1	1.624e-1	-0.169	6.448	1.021e-1	4.92e-1
FN1290	AAL95486.1| Hypothetical protein	0.645	10.431	1.852e-1	5.055e-1	-0.935	9.035	3.475e-3	1.35e-2	-1.483	8.099	2.174e-4	3.29e-4	-2.128	8.949	9.586e-2	2.05e-1	-1.581	9.681	1.784e-1	2.584e-1	-0.547	7.552	4.987e-2	2.222e-1
FN1293	AAL95489.1| Hypothetical protein					-0.864	5.625			-0.693	5.408											0.171	4.932		
FN1296	AAL95492.1| unknown									-1.566	7.856	5.053e-3	1.849e-2												

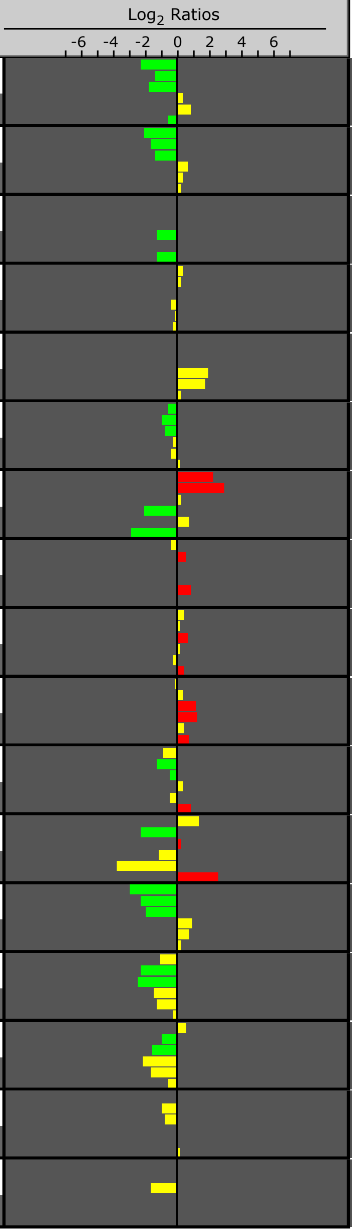

Show detected proteins only
Show all proteins
Filter by category:
GO: amino acid transport

Proteins found: 1424

Enter (or paste) list of ORFs

Find ORFs

Test	Cutoff
q-Value	.005
p-Value	

Dot Plots
Dot Plots

	Signif	Direction	Applies To
red	yes	+	ratios, bars
yellow	no	n/a	bars
green	yes	–	ratios, bars
pink	yes	+	*p*- , *q*-Values
light green	yes	–	*p*- , *q*-Values

FnPg vs Fn
FnSg vs Fn
FnPgSg vs Fn
FnPgSg vs FnPg
FnSg vs FnPg
FnPgSg vs FnSg

ORF	Protein	*FnPg vs Fn*				*FnSg vs Fn*				*FnPgSg vs Fn*				*FnPgSg vs FnPg*				*FnSg vs FnPg*				*FnPgSg vs FnSg*			
		Ratio	Sum	*q*-Val	*p*-Val	Ratio	Sum	*q*-Val	*p*-Val	Ratio	Sum	*q*-Val	*p*-Val	Ratio	Sum	*q*-Val	*p*-Val	Ratio	Sum	*q*-Val	*p*-Val	Ratio	Sum	*q*-Val	*p*-Val
FN1297	AAL95493.1| Methionine aminopeptidase	-0.841	9.444	4.337e-3	3.722e-3	-0.261	10.208	5.042e-3	2.088e-2	0.451	10.532	6.444e-3	2.469e-2	1.292	9.895	1.69e-3	5.399e-4	0.580	9.368	2.583e-2	7.093e-3	0.712	10.659	2.065e-3	4.807e-3
FN1298	AAL95494.1| Adenylate kinase	-0.719	13.058	7.377e-3	7.764e-3	-1.411	12.551	2.195e-3	7.624e-3	-0.430	13.144	1.609e-2	7.057e-2	0.289	12.628	1.183e-1	2.843e-1	-0.692	11.831	1.109e-1	1.031e-1	0.982	12.121	1.02e-2	3.586e-2
FN1299	AAL95495.1| dTDP-glucose 4,6-dehydratase																	-0.068	5.287						
FN1301	AAL95497.1| ABC transporter ATP-binding protein	-0.775	12.818	4.601e-2	8.006e-2	-1.250	12.528	5.191e-6	2.015e-6	-0.404	12.985	8.358e-4	1.9e-3	0.371	12.414	1.407e-1	3.68e-1	-0.475	11.753	2.3e-1	3.849e-1	0.846	12.124	3.104e-4	4.039e-4
FN1302	AAL95498.1| Hypothetical protein	0.143	17.191	2.688e-1	8.195e-1	-1.858	15.374	1.188e-3	3.661e-3	0.595	17.439	1.779e-3	5.238e-3	0.452	17.786	1.5e-1	4.013e-1	-2.002	15.517	1.294e-1	1.387e-1	2.453	15.969	4.184e-4	5.898e-4
FN1303	AAL95499.1| hypothetical cytosolic protein	-0.634	12.859	6.586e-4	2.35e-4	-0.799	12.879	3.786e-5	4.019e-5	-0.764	12.525	5.782e-5	4.994e-5	-0.130	12.095	1.106e-1	2.58e-1	-0.165	12.244	1.421e-1	1.683e-1	0.035	12.114	1.474e-1	7.479e-1
FN1304	AAL95500.1| Single-strand DNA binding protein	-0.297	14.350	3.374e-2	5.275e-2	-0.525	14.306	1.29e-4	2.185e-4	-0.135	14.308	1.864e-2	8.348e-2	0.162	14.215	1.013e-1	2.242e-1	-0.228	14.009	1.296e-1	1.391e-1	0.391	14.171	1.726e-3	3.858e-3
FN1305	AAL95501.1| Hypothetical cytosolic protein	0.390	11.020	1.948e-3	1.24e-3	-0.035	10.780	1.491e-1	9.309e-1	-0.462	9.965	2.969e-2	1.419e-1	-0.851	10.558	1.935e-2	2.027e-2	-0.424	11.170	1.855e-1	2.762e-1	-0.427	10.319	8.954e-2	4.254e-1
FN1306	AAL95502.1| Methyltransferase	0.930	14.022			1.030	14.306	2.074e-3	7.116e-3	-1.696	11.192	1.299e-5	4.587e-6	-2.626	12.326			0.100	15.236			-2.726	12.610	7.839e-4	1.4e-3
FN1309	AAL95505.1| Hypothetical protein	-1.262	15.446	2.089e-3	1.378e-3	-1.337	15.556	2.779e-4	5.794e-4	0.143	16.647	2.402e-2	1.11e-1	1.405	15.589	4.227e-4	6.75e-5	-0.074	14.294	3.691e-1	7.639e-1	1.479	15.699	4.567e-5	3.076e-5
FN1311	AAL95507.1| Biopolymer transport exbD protein																								
FN1312	AAL95508.1| Biopolymer transport exbB protein																								
FN1313	AAL95509.1| Oligopeptide-binding protein oppA	-0.725	12.654	8.857e-4	3.897e-4	-0.678	12.885	1.501e-4	2.691e-4	-0.522	12.653	1.252e-2	5.31e-2	0.203	12.132	1.645e-1	4.536e-1	0.047	12.160	3.583e-1	7.289e-1	0.157	12.363	1.121e-1	5.463e-1
FN1315	AAL95511.1| Hypothetical protein					-0.215	6.145																		
FN1317	AAL95513.1| RNA polymerase sigma factor	-0.425	7.763	6.64e-2	1.333e-1	-0.766	7.607	6.868e-4	1.857e-3									-0.341	7.182	1.95e-1	3.013e-1				
FN1318	AAL95514.1| RNA polymerase sigma factor rpoD	-0.535	10.215	1.379e-2	1.671e-2	-0.955	9.979	9.638e-4	2.849e-3	-0.272	10.274	9.167e-3	3.726e-2	0.262	9.943	8.204e-2	1.617e-1	-0.420	9.445	1.354e-1	1.52e-1	0.683	9.707	7.783e-3	2.606e-2
FN1319	AAL95515.1| DNA primase	-0.561	7.146	7.728e-3	8.256e-3	0.640	8.531	4.834e-2	2.689e-1	-0.848	6.655	8.414e-4	1.916e-3	-0.286	6.298	7.803e-2	1.504e-1	1.201	7.970	1.214e-1	1.221e-1	-1.487	7.683	2.276e-2	9.15e-2

Log$_2$ Ratios

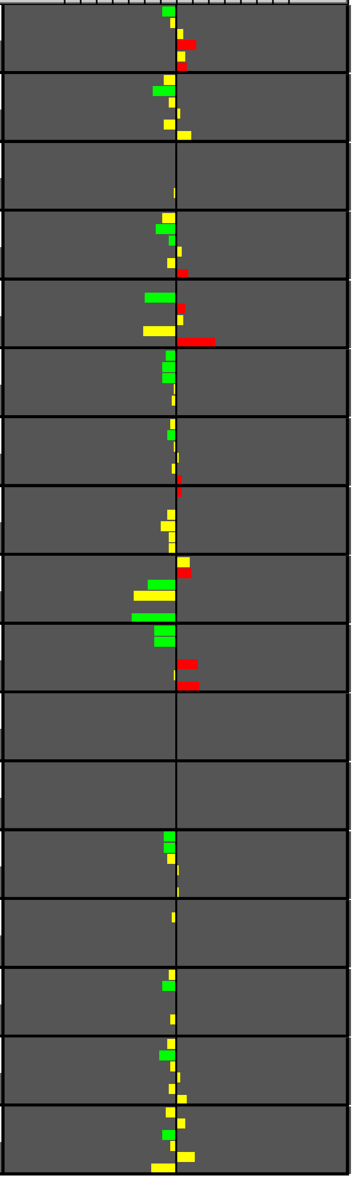

Show detected proteins only
Show all proteins
Filter by category:
GO: amino acid transport

Proteins found: 1424

Enter (or paste) list of ORFs

Find ORFs

Test	Cutoff
q-Value	.005
p-Value	

Dot Plots
Dot Plots

	Signif	Direction	Applies To
(red)	yes	+	ratios, bars
(yellow)	no	n/a	bars
(green)	yes	–	ratios, bars
(pink)	yes	+	*p*- , *q*-Values
(light green)	yes	–	*p*- , *q*-Values

FnPg vs Fn
FnPgSg vs Fn
FnSg vs FnPg
FnSg vs Fn
FnPgSg vs FnPg
FnPgSg vs FnSg

ORF	FnPg vs Fn Ratio	Sum	*q*-Val	*p*-Val	FnSg vs Fn Ratio	Sum	*q*-Val	*p*-Val	FnPgSg vs Fn Ratio	Sum	*q*-Val	*p*-Val	FnPgSg vs FnPg Ratio	Sum	*q*-Val	*p*-Val	FnSg vs FnPg Ratio	Sum	*q*-Val	*p*-Val	FnPgSg vs FnSg Ratio	Sum	*q*-Val	*p*-Val
**FN1320**	-1.481	16.169	1.26e-4	1.988e-5	-1.364	16.471	5.345e-5	6.673e-5	-0.838	16.608	3.695e-4	6.744e-4	0.643	15.331	1.754e-2	1.772e-2	0.117	14.990	2.657e-1	4.725e-1	0.526	15.632	7.282e-3	2.398e-2
	AAL95516.1| Peptidyl-prolyl cis-trans isomerase																							
**FN1321**	0.002	19.560	3.006e-1	9.58e-1	0.313	20.055	7.656e-4	2.157e-3	0.034	19.388	1.389e-1	8.162e-1	0.032	19.594	2.554e-1	8.225e-1	0.311	20.057	2.495e-2	6.668e-3	-0.279	20.089	2.344e-2	9.448e-2
	AAL95517.1| Acetoacetate metabolism regulatory protein atoC																							
**FN1322**	0.997	9.177			0.166	8.530	7.725e-2	4.449e-1	-1.666	6.310			-2.664	7.511			-0.832	9.528			-1.832	6.864		
	AAL95518.1| Membrane metalloprotease																							
**FN1323**	-0.817	7.250	2.046e-4	3.943e-5	-1.237	7.014	7.975e-6	4.09e-6	-0.909	6.954	2.423e-3	7.603e-3	-0.092	6.341	2.242e-1	6.929e-1	-0.420	6.197	2.669e-3	2.034e-4	0.328	6.105	5.967e-2	2.716e-1
	AAL95519.1| Thymidylate kinase																							
**FN1324**	0.097	10.185	2.672e-1	8.128e-1	0.430	10.702	4.974e-3	2.057e-2	-0.681	9.203	7.383e-3	2.889e-2	-0.778	9.503	8.943e-2	1.84e-1	0.333	10.799	2.213e-1	3.63e-1	-1.111	10.021	8.493e-4	1.568e-3
	AAL95520.1| 1-deoxy-D-xylulose 5-phosphate reductoisomerase																							
**FN1326**	-0.927	6.927			-1.212	6.826			-0.660	6.990			0.267	6.267			-0.285	5.900			0.552	6.166		
	AAL95522.1| Undecaprenyl pyrophosphate synthetase																							
**FN1327**					1.946	6.083			1.125	4.875											-0.820	7.208		
	AAL95523.1| Dimethylallyltransferase																							
**FN1328**									-1.790	5.733	3.431e-4	6.073e-4												
	AAL95524.1| Exodeoxyribonuclease VII small subunit																							
**FN1330**	-0.281	3.451			0.042	3.958											0.323	3.677						
	AAL95526.1| S-adenosylmethionine:tRNA ribosyltransferase-isomerase																							
**FN1331**									0.309	6.206	9.769e-2	5.477e-1												
	AAL95527.1| Methyltransferase																							
**FN1332**	-0.954	11.223	1.642e-6	5.352e-8	-1.174	11.189	2.072e-4	3.995e-4	-0.278	11.697	8.455e-3	3.385e-2	0.677	10.946	9.577e-3	7.052e-3	-0.219	10.234	1.362e-1	1.538e-1	0.896	10.911	6.166e-4	1.009e-3
	AAL95528.1| Bacterial Peptide Chain Release Factor 1 (RF-1)																							
**FN1333**																								
	AAL95529.1| Hypothetical protein																							
**FN1334**	0.666	8.947	1.665e-1	4.361e-1	-1.623	6.842	6.51e-6	3.013e-6	-0.454	7.624			-1.119	8.493			-2.289	7.508	1.342e-1	1.492e-1	1.170	6.389		
	AAL95530.1| N-acetylmuramoyl-L-alanine amidase																							
**FN1335**	-0.510	12.806	3.382e-2	5.293e-2	-1.236	12.265	2.61e-3	9.432e-3	1.254	14.366	4.279e-3	1.514e-2	1.764	14.060	1.02e-2	7.791e-3	-0.725	11.755	1.196e-1	1.187e-1	2.490	13.519	1.696e-3	3.778e-3
	AAL95531.1| Protein translocase subunit YajC																							
**FN1336**	-0.345	9.253			-1.084	8.699											-0.739	8.354						
	AAL95532.1| Hypothetical protein																							
**FN1337**	-2.173	7.980	6.754e-3	6.861e-3	-2.361	7.976			-2.390	7.560			-0.216	5.590			-0.188	5.803			-0.028	5.586		
	AAL95533.1| unknown																							
**FN1340**	-0.495	16.632	5.847e-2	1.116e-1	-0.680	16.630	3.063e-4	6.604e-4	0.070	16.993	9.03e-2	5.012e-1	0.565	16.702	4.847e-2	7.589e-2	-0.186	16.136	3.042e-1	5.707e-1	0.751	16.700	6.195e-4	1.016e-3
	AAL95536.1| Glutamyl-tRNA synthetase																							

Log$_2$ Ratios

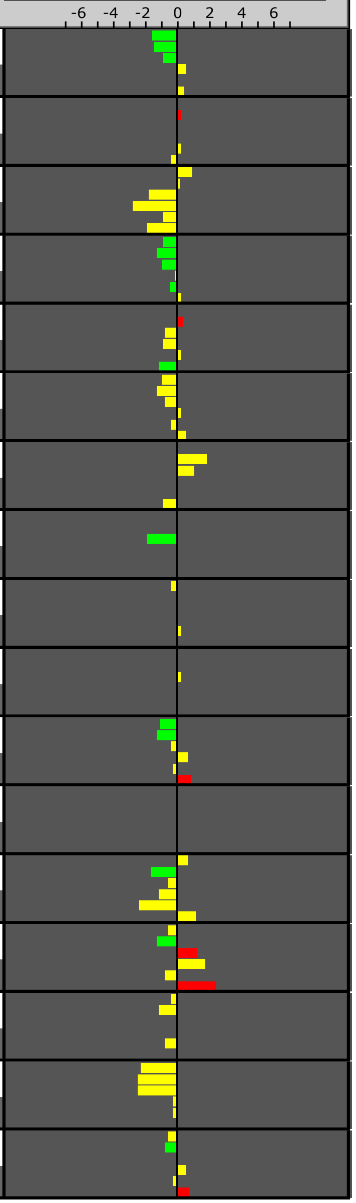

Show detected proteins only
Show all proteins
Filter by category:
GO: amino acid transport

Proteins found: 1424

Enter (or paste) list of ORFs

Find ORFs

Test	Cutoff
q-Value	.005
p-Value	

Dot Plots
Dot Plots

	Signif	Direction	Applies To
red	yes	+	ratios, bars
yellow	no	n/a	bars
green	yes	–	ratios, bars
pink	yes	+	*p*- , *q*-Values
light green	yes	–	

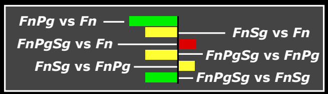

ORF	Protein	*FnPg vs Fn*				*FnSg vs Fn*				*FnPgSg vs Fn*				*FnPgSg vs FnPg*				*FnSg vs FnPg*				*FnPgSg vs FnSg*			
		Ratio	Sum	*q*-Val	*p*-Val	Ratio	Sum	*q*-Val	*p*-Val	Ratio	Sum	*q*-Val	*p*-Val	Ratio	Sum	*q*-Val	*p*-Val	Ratio	Sum	*q*-Val	*p*-Val	Ratio	Sum	*q*-Val	*p*-Val
FN1341	AAL95537.1| Bacterial Peptide Chain Release Factor 2 (RF-2)	-0.268	7.438			-0.459	7.432	2.206e-3	7.673e-3	0.410	7.913	2.967e-2	1.418e-1	0.678	7.848			-0.191	7.163			0.869	7.842	9.887e-3	3.455e-2
FN1343	AAL95539.1| seC-independent protein TATD	1.511	9.473			0.735	8.881											-0.776	10.392						
FN1345	AAL95541.1| 2-hydroxy-6-oxo-6-phenylhexa-2,4-dienoate hydrolase																								
FN1346	AAL95542.1| Hypothetical cytosolic protein																	1.590	6.945						
FN1347	AAL95543.1| Hypothetical cytosolic protein	-1.806	7.591	1.867e-3	1.164e-3	-2.035	7.547	5.083e-4	1.254e-3	-1.168	8.025	8.866e-4	2.047e-3	0.638	6.423	1.853e-3	6.23e-4	-0.229	5.740	6.766e-2	3.884e-2	0.867	6.378	5.785e-4	9.184e-4
FN1348	AAL95544.1| ABC transporter ATP-binding protein	-1.177	8.158	1.437e-2	1.76e-2	0.261	9.781	3.153e-2	1.691e-1	-0.313	8.818	1.086e-1	6.168e-1	0.864	7.845	1.433e-1	3.773e-1	1.438	8.604	2.334e-2	5.951e-3	-0.574	9.468	7.142e-2	3.322e-1
FN1349	AAL95545.1| ABC transporter permease protein	-2.532	8.146			-2.175	8.687	1.369e-4	2.374e-4	-2.237	8.237			0.295	5.909			0.357	6.156			-0.062	6.451		
FN1351	AAL95547.1| 15 kDa lipoprotein precursor	-0.416	11.067	8.498e-2	1.841e-1	-0.432	11.235	3.188e-2	1.711e-1	-1.101	10.178	6.715e-3	2.589e-2	-0.686	9.966	2.08e-2	2.244e-2	-0.016	10.820	4.085e-1	9.017e-1	-0.670	10.134	7.207e-3	2.367e-2
FN1352	AAL95548.1| ABC transporter ATP-binding protein	-0.521	11.686	3.2e-3	2.46e-3	-0.474	11.918	3.852e-3	1.541e-2	-0.191	11.812	1.493e-2	6.484e-2	0.330	11.495	1.903e-2	1.981e-2	0.047	11.397	3.625e-1	7.424e-1	0.283	11.727	1.938e-2	7.597e-2
FN1353	AAL95549.1| ABC transporter permease protein	-0.149	7.764			-0.205	7.892	1.059e-1	6.293e-1	-0.576	7.133	5.372e-2	2.804e-1	-0.427	7.188			-0.056	7.743			-0.371	7.316	5.968e-2	2.716e-1
FN1354	AAL95550.1| ABC transporter permease protein	-0.116	10.024			-0.699	9.626	3.802e-3	1.516e-2	-0.511	9.426	7.899e-3	3.126e-2	-0.395	9.513			-0.583	9.510			0.188	9.115	7.149e-2	3.325e-1
FN1355	AAL95551.1| Integral membrane protein									-1.125	4.295														
FN1358	AAL95554.1| Hypothetical protein													-0.327	10.843			-0.455	10.899			0.128	10.572	1.125e-1	5.481e-1
FN1359	AAL95555.1| Dipeptide-binding protein	1.675	16.313	6.126e-2	1.195e-1	2.297	17.119	1.388e-3	4.424e-3	-0.764	13.669	1.43e-3	3.94e-3	-2.440	15.548	5.137e-2	8.292e-2	0.621	18.794	1.536e-1	1.993e-1	-3.061	16.354	1.608e-3	3.526e-3
FN1362	AAL95558.1| Dipeptide transport ATP-binding protein dppD																	0.703	12.295	7.088e-2	4.238e-2				
FN1363	AAL95559.1| Dipeptide transport ATP-binding protein dppF	1.759	8.809	3.939e-2	6.462e-2	2.705	9.941	2.934e-4	6.228e-4	-1.279	5.568	1.554e-2	6.78e-2	-3.037	7.531	3.106e-2	4.001e-2	0.947	11.699	5.358e-2	2.605e-2	-3.984	8.662	7.381e-4	1.288e-3
FN1364	AAL95560.1| LSU ribosomal protein L32P	-1.345	12.067							-0.834	12.373	1.019e-3	2.451e-3	0.510	11.233										

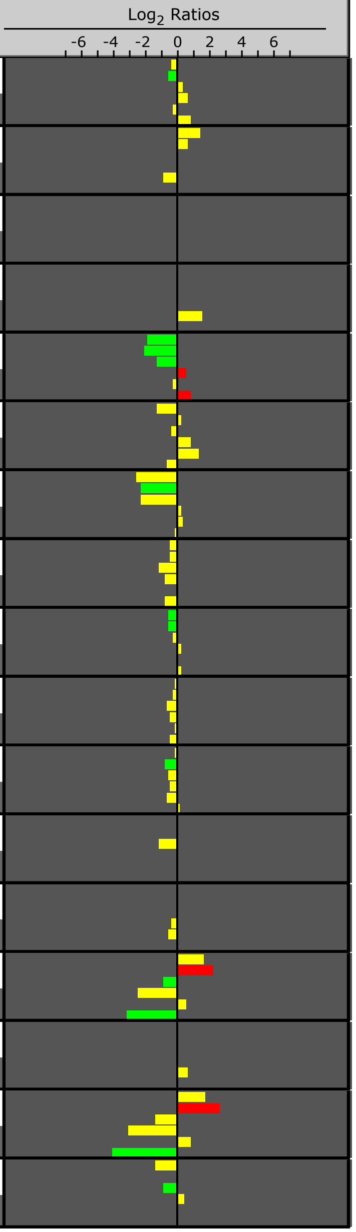

Show detected proteins only
Show all proteins
Filter by category:
GO: amino acid transport

Proteins found: 1424

Enter (or paste) list of ORFs

Find ORFs

Test	Cutoff
q-Value	.005
p-Value	

Dot Plots
Dot Plots

	Signif	Direction	Applies To
red	yes	+	ratios, bars
yellow	no	n/a	bars
green	yes	–	ratios, bars
pink	yes	+	*p*- , *q*-Values
light green	yes	–	*p*- , *q*-Values

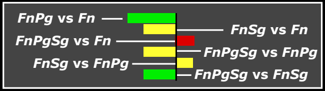

ORF	Protein	*FnPg vs Fn* Ratio	Sum	*q*-Val	*p*-Val	*FnSg vs Fn* Ratio	Sum	*q*-Val	*p*-Val	*FnPgSg vs Fn* Ratio	Sum	*q*-Val	*p*-Val	*FnPgSg vs FnPg* Ratio	Sum	*q*-Val	*p*-Val	*FnSg vs FnPg* Ratio	Sum	*q*-Val	*p*-Val	*FnPgSg vs FnSg* Ratio	Sum	*q*-Val	*p*-Val
FN1365	AAL95561.1| GTP-binding protein	-0.595	14.347	8.129e-2	1.736e-1	0.155	15.281	6.373e-2	3.617e-1	0.213	14.951	5.136e-3	1.885e-2	0.808	14.560	4.973e-2	7.89e-2	0.750	14.686	1.07e-1	9.628e-2	0.059	15.494	1.367e-1	6.849e-1
FN1366	AAL95562.1| Triosephosphate isomerase	-0.402	15.015	2.211e-2	3.071e-2	-0.778	14.823	8.148e-4	2.328e-3	0.931	16.145	5.722e-5	4.925e-5	1.333	15.947	1.247e-4	1.386e-5	-0.376	14.422	1.007e-1	8.614e-2	1.709	15.755	1.438e-5	5.293e-6
FN1368	AAL95564.1| COMF operon protein 3																								
FN1371	AAL95567.1| Ribonuclease HII																	0.230	4.414						
FN1373	AAL95569.1| regulatory protein																								
FN1374	AAL95570.1| Transcriptional regulator	-0.919	6.534			-0.819	6.819			-0.455	6.795			0.464	6.079			0.100	5.900			0.364	6.364		
FN1375	AAL95571.1| Citrate-sodium symport	1.676	11.582			0.467	10.557	2.935e-2	1.562e-1	-0.308	9.394	4.839e-3	1.754e-2	-1.984	11.274			-1.210	12.234			-0.775	10.249	1.654e-2	6.324e-2
FN1376	AAL95572.1| Oxaloacetate decarboxylase alpha chain	-0.022	18.502	2.977e-1	9.453e-1	0.702	19.411	1.056e-2	4.97e-2	-1.002	17.319	3.112e-4	5.309e-4	-0.980	17.500	5.701e-2	9.735e-2	0.724	19.389	8.708e-2	6.465e-2	-1.704	18.409	3.514e-3	9.385e-3
FN1377	AAL95573.1| CITG protein																								
FN1378	AAL95574.1| Citrate lyase acyl carrier protein	-0.796	9.834	7.855e-2	1.658e-1	-0.568	10.247			-0.500	9.927	1.515e-3	4.27e-3	0.296	9.334	2.048e-1	6.171e-1	0.229	9.451			0.068	9.747		
FN1379	AAL95575.1| Citrate lyase beta chain	0.395	15.728	2.098e-1	5.92e-1	0.954	16.471	1.937e-5	1.573e-5	-0.144	14.985	3.171e-2	1.534e-1	-0.539	15.584	1.748e-1	4.929e-1	0.559	16.866	2.039e-1	3.218e-1	-1.098	16.327	2.599e-5	1.329e-5
FN1380	AAL95576.1| Citrate lyase beta chain	0.460	17.821	2.113e-1	5.973e-1	0.865	18.410	1.552e-2	7.749e-2	-0.006	17.151	1.519e-1	9.066e-1	-0.467	17.815	1.994e-1	5.936e-1	0.404	18.871	3.009e-1	5.619e-1	-0.871	18.404	1.935e-2	7.586e-2
FN1381	AAL95577.1| unknown									-0.293	4.178														
FN1382	AAL95578.1| ATPase																	-0.985	6.600						
FN1383	AAL95579.1| DNA polymerase III alpha subunit													-1.428	4.802			0.715	7.130	9.362e-2	7.579e-2	-2.143	5.701		
FN1385	AAL95581.1| Hypothetical protein	-1.567	4.737			0.228	6.718	1.125e-1	6.736e-1									1.796	5.150						
FN1386	AAL95582.1| SWF/SNF family helicase	0.648	6.271			1.846	7.654	2.145e-3	7.413e-3	1.058	6.478	6.956e-3	2.696e-2	0.411	7.330			1.199	8.302			-0.788	8.713	1.139e-2	4.104e-2

Log$_2$ Ratios

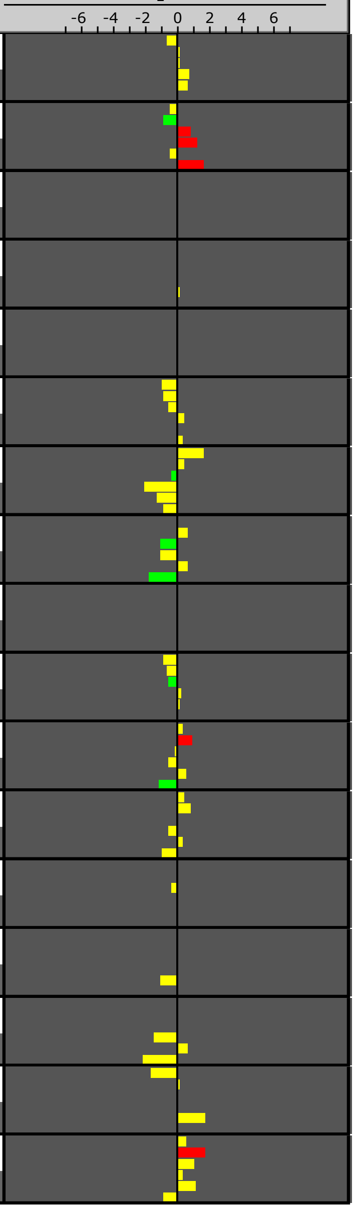

Show detected proteins only
Show all proteins
Filter by category:
GO: amino acid transport

Proteins found: 1424

Enter (or paste) list of ORFs

Find ORFs

Test	Cutoff
q-Value	.005
p-Value	

Dot Plots
Dot Plots

	Signif	Direction	Applies To
red	yes	+	ratios, bars
yellow	no	n/a	bars
green	yes	–	ratios, bars
pink	yes	+	*p*- , *q*-Values
light green	yes	–	*p*- , *q*-Values

ORF	Protein	*FnPg vs Fn*				*FnSg vs Fn*				*FnPgSg vs Fn*				*FnPgSg vs FnPg*				*FnSg vs FnPg*				*FnPgSg vs FnSg*				Log_2 Ratios
		Ratio	Sum	*q*-Val	*p*-Val	Ratio	Sum	*q*-Val	*p*-Val	Ratio	Sum	*q*-Val	*p*-Val	Ratio	Sum	*q*-Val	*p*-Val	Ratio	Sum	*q*-Val	*p*-Val	Ratio	Sum	*q*-Val	*p*-Val	-6 -4 -2 0 2 4 6
**FN1391**	AAL95584.1| Acetyltransferase	-1.043	12.722	2.913e-3	2.15e-3	-1.705	12.244	3.054e-4	6.578e-4	-1.264	12.297	8.207e-4	1.858e-3	-0.221	11.457	1.331e-1	3.396e-1	-0.662	11.201	7.426e-2	4.638e-2	0.441	10.980	2.531e-2	1.029e-1	
**FN1392**	AAL95585.1| SSU ribosomal protein S16P	-0.672	14.866	9.864e-2	2.232e-1	-1.845	13.878	1.15e-8	1.589e-10	-0.204	15.130	3.254e-3	1.087e-2	0.468	14.662	1.487e-1	3.967e-1	-1.173	13.206	1.734e-1	2.466e-1	1.640	13.674	9.612e-5	8.249e-5	
**FN1393**	AAL95586.1| Signal recognition particle, subunit FFH/SRP54	-2.009	9.995	1.006e-3	4.741e-4	-0.634	11.554	1.046e-3	3.141e-3	-1.034	10.766	3.196e-4	5.504e-4	0.974	8.961	3.775e-2	5.309e-2	1.375	9.546	3.124e-2	9.79e-3	-0.400	10.520	1.032e-2	3.637e-2	
**FN1397**	AAL95590.1| Glutaminase	-1.469	13.773	4.132e-3	3.477e-3	-1.981	13.445	3.795e-6	1.258e-6	-0.464	14.574	8.85e-3	3.573e-2	1.005	13.309	1.533e-2	1.465e-2	-0.513	11.976	1.751e-1	2.505e-1	1.517	12.981	2.961e-3	7.552e-3	
**FN1398**	AAL95591.1| Amino acid carrier protein alsT	-1.087	12.699	3.05e-2	4.617e-2	-1.505	12.465	3.555e-3	1.391e-2	0.635	14.217	5.686e-3	2.129e-2	1.722	13.334	9.724e-3	7.22e-3	-0.419	11.378	2.868e-1	5.25e-1	2.140	13.100	2.006e-7	1.127e-8	
**FN1399**	AAL95592.1| Hypothetical cytosolic protein																									
**FN1400**	AAL95593.1| serine/threonine kinase													1.437	5.097											
**FN1406**	AAL95599.1| Histidine ammonia-lyase	0.209	11.969	2.406e-1	7.065e-1	0.273	12.219	1.037e-2	4.869e-2	-0.021	11.536	1.483e-1	8.813e-1	-0.229	11.949	2.214e-1	6.816e-1	0.065	12.427	4.069e-1	8.956e-1	-0.294	12.198	1.098e-2	3.923e-2	
**FN1407**	AAL95600.1| Glutamate formiminotransferase	1.487	8.513			1.445	8.655	9.711e-3	4.519e-2	0.617	7.439	6.913e-2	3.709e-1	-0.870	9.130			-0.042	10.142			-0.828	9.272	3.635e-2	1.551e-1	
**FN1411**	AAL95604.1| Threonine dehydratase	1.414	15.743	2.48e-2	3.551e-2	2.355	16.868	1.15e-8	1.52e-10	0.339	14.464	4.53e-2	2.315e-1	-1.075	16.082	3.508e-2	4.799e-2	0.940	18.282	3.584e-2	1.251e-2	-2.016	17.207	1.444e-4	1.406e-4	
**FN1412**	AAL95605.1| 5-methylthioribose kinase																	-1.714	8.023							
**FN1413**	AAL95606.1| Translation initiation factor EIF-2B subunit 1													-2.089	5.463			-1.868	5.868			-0.221	3.779			
**FN1415**	AAL95608.1| NADH-dependent butanol dehydrogenase A																	0.144	7.257							
**FN1416**	AAL95609.1| Transcriptional regulator, GntR family													-2.338	6.832			-2.677	6.677			0.339	4.339			
**FN1417**	AAL95610.1| L-fuculose phosphate aldolase	1.041	5.824			0.839	5.807			0.032	4.612			-1.009	5.856			-0.201	6.848	2.65e-1	4.708e-1	-0.807	5.839			
**FN1418**	AAL95611.1| Transcriptional regulator, GntR family	-0.352	4.431			-0.696	4.271											-0.344	3.919							
**FN1419**	AAL95612.1| Methionine gamma-lyase	3.256	21.695	2.928e-2	4.378e-2	2.422	21.045	1.67e-3	5.504e-3	2.033	20.268	1.133e-6	1.531e-7	-1.223	23.727	6.688e-2	1.213e-1	-0.834	24.301	1.532e-1	1.982e-1	-0.389	23.078	3.057e-2	1.273e-1	

Show detected proteins only
Show all proteins
Filter by category:
GO: amino acid transport

Proteins found:
1424

Enter (or paste) list of ORFs

Find ORFs

Test	Cutoff
q-Value	.005
p-Value	

Dot Plots
Dot Plots

	Signif	Direction	Applies To
red	yes	+	ratios, bars
yellow	no	n/a	bars
green	yes	–	ratios, bars
pink	yes	+	*p*- , *q*-Values
light green	yes	–	*p*- , *q*-Values

FnPg vs Fn
FnPgSg vs Fn
FnSg vs FnPg
FnSg vs Fn
FnPgSg vs FnPg
FnPgSg vs FnSg

ORF	Description	*FnPg vs Fn* Ratio	Sum	*q*-Val	*p*-Val	*FnSg vs Fn* Ratio	Sum	*q*-Val	*p*-Val	*FnPgSg vs Fn* Ratio	Sum	*q*-Val	*p*-Val	*FnPgSg vs FnPg* Ratio	Sum	*q*-Val	*p*-Val	*FnSg vs FnPg* Ratio	Sum	*q*-Val	*p*-Val	*FnPgSg vs FnSg* Ratio	Sum	*q*-Val	*p*-Val
FN1420	AAL95613.1| NA+/H+ antiporter NHAC													-1.344	9.163										
FN1421	AAL95614.1| Pyruvate-flavodoxin oxidoreductase	1.019	21.168	5.457e-2	1.011e-1	0.598	20.931	6.024e-5	7.904e-5	0.210	20.155	2.186e-3	6.729e-3	-0.809	21.378	7.487e-2	1.419e-1	-0.421	21.950	2.063e-1	3.271e-1	-0.388	21.141	1.186e-5	3.823e-6
FN1423	AAL95616.1| Flavoprotein	-0.853	14.131	4.087e-2	6.792e-2	0.373	15.542	1.053e-4	1.669e-4	0.330	15.111	3.541e-3	1.202e-2	1.183	14.461	1.71e-2	1.712e-2	1.226	14.689	4.488e-2	1.858e-2	-0.043	15.872	1.138e-1	5.554e-1
FN1424	AAL95617.1| ACYL-COA dehydrogenase, short-chain specific	-1.272	15.849	1.827e-2	2.406e-2	0.371	17.677	4.366e-3	1.774e-2	0.207	17.125	5.448e-2	2.85e-1	1.480	16.056	1.038e-2	8.016e-3	1.643	16.405	1.887e-2	4.262e-3	-0.164	17.884	7.677e-2	3.597e-1
FN1426	AAL95619.1| Serine protease	-2.115	16.397	2.673e-3	1.909e-3	-1.045	17.652	1.195e-6	1.975e-7	-1.863	16.445	4.383e-5	3.382e-5	0.252	14.534	2.048e-1	6.173e-1	1.070	15.537	7.141e-2	4.299e-2	-0.819	15.789	9.156e-4	1.726e-3
FN1427	AAL95620.1| Phenazine biosynthesis protein phzF													-0.744	5.900										
FN1429	AAL95622.1| Branched-chain amino acid transport ATP-binding protein livG																								
FN1432	AAL95625.1| Leucine-, isoleucine-, valine-, threonine-, and alanine-binding protein					-3.913	7.913			-1.094	10.343	1.523e-3	4.298e-3									2.819	6.819		
FN1433	AAL95626.1| Short chain dehydrogenase	0.148	16.031	1.611e-2	2.037e-2	-0.433	15.634	7.703e-4	2.176e-3	0.426	16.104	3.541e-3	1.202e-2	0.278	16.457	3.338e-2	4.457e-2	-0.581	15.783	8.306e-4	2.149e-5	0.859	16.060	1.59e-3	3.476e-3
FN1434	AAL95627.1| Tetratricopeptide repeat family protein	-0.562	13.916	3.737e-2	6.026e-2	-0.115	14.548	2.146e-2	1.107e-1	-0.705	13.570	1.409e-4	1.774e-4	-0.143	13.211	2.049e-1	6.177e-1	0.447	13.985	1.195e-1	1.186e-1	-0.590	13.843	5.628e-4	8.827e-4
FN1437	AAL95630.1| LSU ribosomal protein L28P	-1.261	14.910	4.208e-3	3.566e-3	-2.113	14.242	4.812e-6	1.785e-6	-0.930	15.037	9.446e-5	9.988e-5	0.330	13.980	1.178e-1	2.828e-1	-0.852	12.982	1.088e-1	9.949e-2	1.182	13.312	1.097e-3	2.193e-3
FN1439	AAL95632.1| Transcriptional regulator, DeoR family	-2.376	11.423			-3.837	10.146	9.237e-4	2.705e-3	-1.963	11.632	1.338e-3	3.582e-3	0.413	9.460			-1.461	7.770			1.874	8.183	2.529e-3	6.227e-3
FN1440	AAL95633.1| 1-phosphofructokinase	-1.976	12.297	1.173e-3	6.117e-4	-2.917	11.540	7.387e-5	1.075e-4	-1.580	12.489	4.442e-4	8.573e-4	0.397	10.717	1.84e-2	1.892e-2	-0.941	9.564	4.847e-2	2.137e-2	1.337	9.961	1.364e-3	2.869e-3
FN1441	AAL95634.1| PTS system, fructose-specific IIABC component	-2.055	13.602	1.297e-3	7.084e-4	-0.907	14.935	6.787e-5	9.427e-5	-0.637	14.817	4.58e-5	3.599e-5	1.419	12.966	9.978e-3	7.52e-3	1.148	12.880	4.152e-2	1.608e-2	0.270	14.298	1.316e-2	4.852e-2
FN1444	AAL95637.1| GMP synthase (glutamine-hydrolyzing)	0.031	17.717	2.477e-1	7.34e-1	-0.325	17.546	2.296e-3	8.058e-3	-0.271	17.212	1.343e-3	3.603e-3	-0.302	17.446	2.514e-2	2.937e-2	-0.356	17.577	4.469e-2	1.842e-2	0.054	17.275	1.14e-1	5.564e-1
FN1445	AAL95638.1| DNA helicase	-1.799	10.317	3.063e-3	2.309e-3	-2.200	10.101	1.068e-3	3.221e-3	-1.001	10.911	3.582e-3	1.218e-2	0.798	9.316	9.897e-3	7.423e-3	-0.401	8.302	1.329e-1	1.462e-1	1.199	9.100	9.208e-6	2.511e-6
FN1448	AAL95641.1| Hypothetical cytosolic protein																								

Log$_2$ Ratios

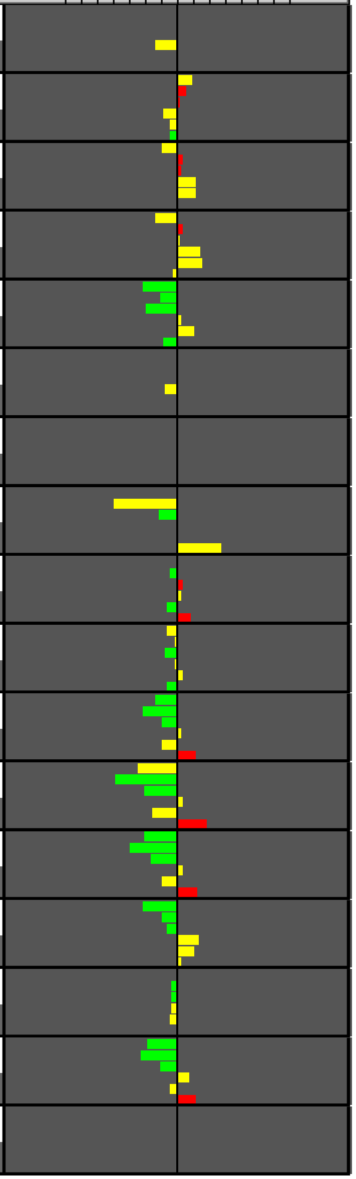

Show detected proteins only
Show all proteins
Filter by category:
GO: amino acid transport

Proteins found: 1424

Enter (or paste) list of ORFs

Find ORFs

Test	Cutoff
q-Value	.005
p-Value	

Dot Plots
Dot Plots

	Signif	Direction	Applies To
(red)	yes	+	ratios, bars
(yellow)	no	n/a	bars
(green)	yes	–	ratios, bars
(pink)	yes	+	*p*- , *q*-Values
(light green)	yes	–	*p*- , *q*-Values

FnPg vs Fn
FnPgSg vs Fn
FnSg vs FnPg
FnSg vs Fn
FnPgSg vs FnPg
FnPgSg vs FnSg

ORF	FnPg vs Fn Ratio	FnPg vs Fn Sum	FnPg vs Fn q-Val	FnPg vs Fn p-Val	FnSg vs Fn Ratio	FnSg vs Fn Sum	FnSg vs Fn q-Val	FnSg vs Fn p-Val	FnPgSg vs Fn Ratio	FnPgSg vs Fn Sum	FnPgSg vs Fn q-Val	FnPgSg vs Fn p-Val	FnPgSg vs FnPg Ratio	FnPgSg vs FnPg Sum	FnPgSg vs FnPg q-Val	FnPgSg vs FnPg p-Val	FnSg vs FnPg Ratio	FnSg vs FnPg Sum	FnSg vs FnPg q-Val	FnSg vs FnPg p-Val	FnPgSg vs FnSg Ratio	FnPgSg vs FnSg Sum	FnPgSg vs FnSg q-Val	FnPgSg vs FnSg p-Val
FN1449	-3.578	15.303	3.516e-7	7.114e-9	-4.909	14.158	5.988e-6	2.575e-6	-1.634	17.044	1.317e-4	1.608e-4	1.945	13.670	8.015e-3	5.339e-3	-1.330	10.579	6.598e-2	3.709e-2	3.275	12.524	2.129e-3	4.994e-3
	AAL95642.1| Fusobacterium outer membrane protein family																							
FN1450	-0.451	8.791			0.525	9.952			-1.110	7.929	2.511e-7	1.888e-8	-0.659	7.681			0.976	9.500			-1.635	8.842		
	AAL95643.1| Integral membrane protein																							
FN1451	-0.039	14.819	7.148e-2	1.465e-1	0.586	15.628	1.133e-4	1.837e-4	-0.363	14.291	5.929e-3	2.236e-2	-0.324	14.456	2.679e-2	3.221e-2	0.625	15.589	2.416e-3	1.684e-4	-0.949	15.265	1.036e-4	9.063e-5
	AAL95644.1| Cell division protein ftsZ																							
FN1452	0.036	13.611	2.74e-1	8.416e-1	0.362	14.120	6.061e-3	2.585e-2	-0.615	12.755	3.825e-3	1.319e-2	-0.652	12.996	1.991e-2	2.109e-2	0.325	14.157	8.838e-2	6.682e-2	-0.977	13.505	2.168e-4	2.45e-4
	AAL95645.1| Cell division protein ftsA																							
FN1454	-0.702	9.486			-0.589	9.784	4.074e-2	2.233e-1	-0.302	9.682	5.884e-3	2.216e-2	0.400	9.184			0.113	9.082			0.287	9.481	1.131e-1	5.516e-1
	AAL95647.1| D-alanine--D-alanine ligase																							
FN1455	0.157	8.630	1.9e-1	5.228e-1	-0.013	8.644	1.432e-1	8.879e-1	-0.511	7.759	2.654e-2	1.245e-1	-0.668	8.119	5.658e-2	9.629e-2	-0.170	8.801	2.695e-1	4.816e-1	-0.498	8.134	3.193e-2	1.337e-1
	AAL95648.1| UDP-N-acetylenolpyruvoylglucosamine reductase																							
FN1456	-0.507	11.985	4.608e-3	4.063e-3	-0.316	12.360	2.725e-3	9.948e-3	-0.648	11.640	7.203e-4	1.583e-3	-0.141	11.337	1.179e-1	2.831e-1	0.191	11.853	6.143e-2	3.269e-2	-0.332	11.712	1.046e-2	3.699e-2
	AAL95649.1| UDP-N-acetylmuramate--alanine ligase																							
FN1457	-0.631	11.388	8.193e-2	1.755e-1	-0.428	11.775	2.24e-2	1.161e-1	-1.513	10.302	3.401e-6	6.872e-7	-0.882	9.875	1.051e-1	2.38e-1	0.203	11.144	3.468e-1	6.931e-1	-1.085	10.262	1.183e-2	4.286e-2
	AAL95650.1| UDP-N-acetylglucosamine-N-acetylmuramyl-Pentapeptide pyrophosphoryl-undecaprenol N-acetylglucosamine transferase																							
FN1458	0.050	12.577	2.882e-1	9.029e-1	-0.350	12.361	2.451e-2	1.28e-1	-0.832	11.491	4.331e-5	3.325e-5	-0.882	11.745	8.629e-2	1.743e-1	-0.401	12.411	2.487e-1	4.318e-1	-0.481	11.529	2.93e-2	1.213e-1
	AAL95651.1| UDP-N-acetylmuramoylalanine--D-glutamate ligase																							
FN1459																								
	AAL95652.1| Phospho-N-acetylmuramoyl-pentapeptide-transferase																							
FN1461	-0.759	10.290	5.368e-8	4.345e-10	-1.210	10.023	5.029e-3	2.083e-2	-1.278	9.567	2.113e-4	3.16e-4	-0.519	9.012	1.223e-2	1.024e-2	-0.451	9.264	1.945e-1	2.999e-1	-0.068	8.745	1.704e-1	8.883e-1
	AAL95654.1| Histidinol-phosphatase																							
FN1462																								
	AAL95655.1| Transcriptional regulator, GntR family																							
FN1463	0.182	16.609	1.215e-1	2.9e-1	0.404	17.015	5.54e-5	7.014e-5	0.642	16.865	5.185e-5	4.321e-5	0.460	17.251	2.016e-2	2.146e-2	0.222	17.197	1.422e-1	1.686e-1	0.238	17.657	9.733e-4	1.869e-3
	AAL95656.1| pyridoxine biosynthesis protein																							
FN1464	-0.124	15.788	2.528e-1	7.543e-1	0.524	16.620	6.288e-5	8.419e-5	-0.738	14.970	2.936e-4	4.909e-4	-0.614	15.050	1.163e-1	2.776e-1	0.648	16.496	1.192e-1	1.179e-1	-1.262	15.882	8.35e-6	2.115e-6
	AAL95657.1| 1-deoxyxylulose-5-phosphate synthase																							
FN1470	-2.269	10.228	3.663e-4	9.386e-5	-4.032	8.649	2.359e-4	4.691e-4	-0.032	12.261	1.353e-1	7.919e-1	2.237	10.196	2.787e-4	3.797e-5	-1.764	6.380	5.236e-2	2.485e-2	4.001	8.617	2.536e-4	3.074e-4
	AAL95663.1| Hypothetical protein																							
FN1471					-1.987	5.952	7.154e-3	3.152e-2	-1.454	6.098											0.533	4.499		
	AAL95664.1| LACI-family transcription regulator																							
FN1472	-3.274	12.762	4.674e-4	1.384e-4	-3.429	12.792	4.04e-4	9.34e-4	-1.228	14.605	1.337e-3	3.577e-3	2.046	11.534	5.896e-3	3.207e-3	-0.154	9.518	3.807e-1	8.026e-1	2.200	11.564	1.217e-7	4.607e-9
	AAL95665.1| N-acetylneuraminate-binding protein																							

Log$_2$ Ratios

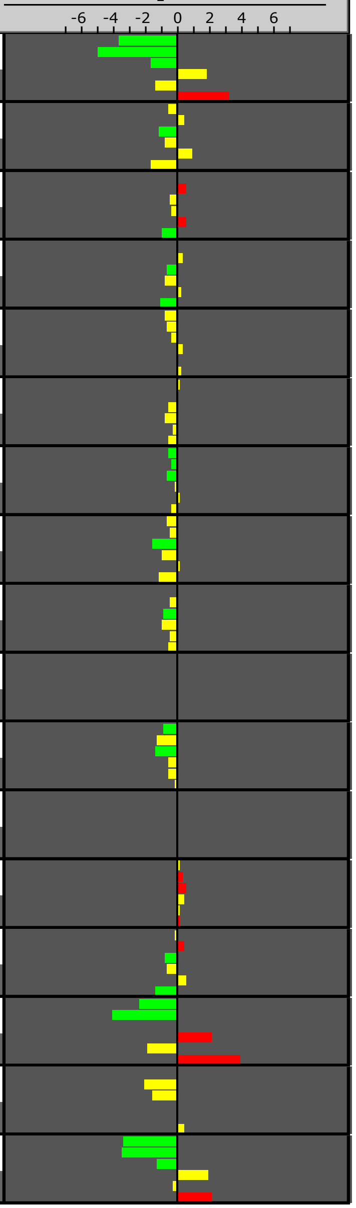

Show detected proteins only
Show all proteins
Filter by category:
GO: amino acid transport

Proteins found: 1424

Enter (or paste) list of ORFs

Find ORFs

Test	Cutoff
q-Value	.005
p-Value	

Dot Plots
Dot Plots

	Signif	Direction	Applies To
red	yes	+	ratios, bars
yellow	no	n/a	bars
green	yes	–	ratios, bars
pink	yes	+	p- , q-Values
light green	yes	–	p- , q-Values

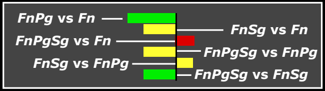

ORF	Protein	*FnPg vs Fn* Ratio	Sum	*q*-Val	*p*-Val	*FnSg vs Fn* Ratio	Sum	*q*-Val	*p*-Val	*FnPgSg vs Fn* Ratio	Sum	*q*-Val	*p*-Val	*FnPgSg vs FnPg* Ratio	Sum	*q*-Val	*p*-Val	*FnSg vs FnPg* Ratio	Sum	*q*-Val	*p*-Val	*FnPgSg vs FnSg* Ratio	Sum	*q*-Val	*p*-Val
FN1474	AAL95667.1| N-acetylmannosamine kinase																								
FN1475	AAL95668.1| N-acetylneuraminate lyase	-1.481	9.773	5.317e-3	4.94e-3	-2.397	9.041			0.996	12.046	9.879e-4	2.354e-3	2.477	10.769	6.392e-4	1.253e-4	-0.916	7.560			3.394	10.037		
FN1476	AAL95669.1| N-acetylmannosamine-6-phosphate 2-epimerase									-0.949	7.937	1.144e-7	4.624e-9												
FN1478	AAL95671.1| Hypothetical protein													-0.894	8.153			-0.235	8.996			-0.659	8.102		
FN1479	AAL95672.1| Hypothetical protein	-0.899	9.593	1.151e-2	1.341e-2	-0.758	9.918	2.551e-4	5.183e-4	-0.809	9.479	2.012e-3	6.102e-3	0.090	8.784	2.494e-1	7.965e-1	0.140	9.019	3.305e-1	6.446e-1	-0.050	9.109	1.613e-1	8.319e-1
FN1480	AAL95673.1| MG2+ transporter MGTE	-0.025	8.554	2.755e-1	8.479e-1	0.738	9.502	6.281e-4	1.649e-3	-1.505	6.871	1.481e-3	4.139e-3	-1.480	7.050	3.565e-3	1.595e-3	0.763	9.477	5.914e-3	7.434e-4	-2.243	7.997	3.816e-5	2.332e-5
FN1481	AAL95674.1| Queuine tRNA-ribosyltransferase	0.921	6.728	7.301e-3	7.657e-3	0.319	6.311	1.055e-1	6.27e-1	-0.802	4.802			-1.722	5.926			-0.602	7.232	1.709e-1	2.403e-1	-1.121	5.509		
FN1482	AAL95675.1| Guanosine-3',5'-bis(Diphosphate) 3'-pyrophosphohydrolase	-0.212	11.556	1.174e-1	2.775e-1	0.667	12.619	3.33e-3	1.279e-2	-1.728	9.836	1.616e-3	4.647e-3	-1.516	9.828	1.034e-4	8.913e-6	0.879	12.407	3.005e-2	9.148e-3	-2.395	10.891	8.523e-4	1.576e-3
FN1483	AAL95676.1| Adenine phosphoribosyltransferase	-0.713	10.454	9.174e-4	4.111e-4	-1.057	10.294	1.05e-4	1.663e-4	0.263	11.227	1.932e-3	5.818e-3	0.976	10.717	9.76e-5	7.935e-6	-0.344	9.581	4.053e-2	1.54e-2	1.321	10.557	9.241e-6	2.527e-6
FN1484	AAL95677.1| Tetratricopeptide repeat family protein					-0.972	6.825	5.444e-3	2.281e-2	-0.856	6.553	8.42e-3	3.369e-2									0.116	5.969	1.375e-1	6.898e-1
FN1485	AAL95678.1| Transporter																								
FN1486	AAL95679.1| magnesium and cobalt efflux protein CorC					-0.572	5.216			-0.555	4.844	4.252e-3	1.502e-2									0.017	4.661		
FN1487	AAL95681.1| Chorismate mutase	-1.415	14.595	4.163e-3	3.513e-3	-0.972	15.222	1.898e-6	4.186e-7	-0.519	15.287	6.114e-3	2.319e-2	0.896	14.076	2.117e-2	2.301e-2	0.443	13.807	1.471e-1	1.82e-1	0.453	14.703	2.011e-2	7.932e-2
FN1488	AAL95682.1| Methylenetetrahydrofolate dehydrogenase (NADP+)													-0.722	8.073	9.775e-2	2.114e-1	-0.402	8.577			-0.320	7.855		
FN1489	AAL95683.1| Methionyl-tRNA formyltransferase	-0.585	9.086	2.168e-2	2.997e-2	-0.369	9.486	1.483e-2	7.364e-2	-1.776	7.690	5.003e-4	1.001e-3	-1.192	7.310	1.698e-2	1.695e-2	0.216	8.901	1.864e-1	2.785e-1	-1.407	7.710	2.615e-3	6.485e-3
FN1490	AAL95684.1| putative regulatory protein	0.744	7.459	5.827e-2	1.111e-1	1.571	8.471	6.866e-3	2.999e-2	-0.449	6.063			-1.192	7.011			0.828	9.215	1.076e-1	9.738e-2	-2.020	8.023		
FN1491	AAL95685.1| PTS system, IIA component	-1.070	7.713			-0.293	8.675											0.777	7.605						

Log$_2$ Ratios

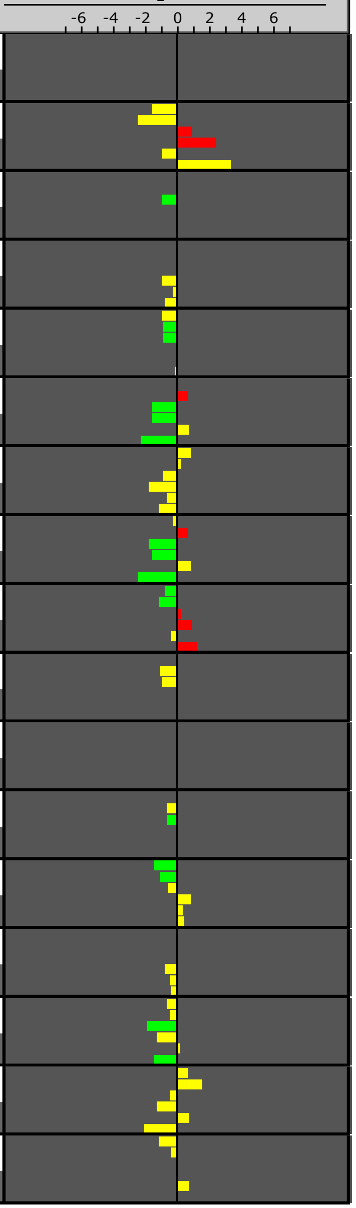

Show detected proteins only
Show all proteins
Filter by category:
GO: amino acid transport

Proteins found: 1424

Enter (or paste) list of ORFs

Find ORFs

Test	Cutoff
q-Value	.005
p-Value	

Dot Plots
Dot Plots

	Signif	Direction	Applies To
red	yes	+	ratios, bars
yellow	no	n/a	bars
green	yes	–	ratios, bars
pink	yes	+	*p*- , *q*-Values
light green	yes	–	*p*- , *q*-Values

FnPg vs Fn
FnPgSg vs Fn
FnSg vs FnPg
FnSg vs Fn
FnPgSg vs FnPg
FnPgSg vs FnSg

ORF	Protein	*FnPg vs Fn* Ratio	Sum	*q*-Val	*p*-Val	*FnSg vs Fn* Ratio	Sum	*q*-Val	*p*-Val	*FnPgSg vs Fn* Ratio	Sum	*q*-Val	*p*-Val	*FnPgSg vs FnPg* Ratio	Sum	*q*-Val	*p*-Val	*FnSg vs FnPg* Ratio	Sum	*q*-Val	*p*-Val	*FnPgSg vs FnSg* Ratio	Sum	*q*-Val	*p*-Val
FN1492	AAL95686.1| DNA repair protein recO																								
FN1493	AAL95687.1| Hypothetical protein	-1.101	7.965	1.442e-2	1.768e-2	-2.101	7.150	2.027e-3	6.924e-3									-1.000	6.049	8.03e-3	1.268e-3				
FN1494	AAL95680.1| Rod shape-determining protein mreC	-1.569	8.212			-1.536	8.430	4.557e-4	1.088e-3	-1.044	8.533	8.809e-7	1.048e-7	0.525	7.169			0.033	6.861			0.492	7.386	2.162e-2	8.641e-2
FN1496	AAL95680.1| Rod shape-determining protein mreC	-1.569	8.212			-1.536	8.430	4.557e-4	1.088e-3	-1.044	8.533	8.809e-7	1.048e-7	0.525	7.169			0.033	6.861			0.492	7.386	2.162e-2	8.641e-2
FN1499	AAL93625.1| Cell surface protein	-0.356	11.888	1.919e-1	5.294e-1	-1.628	10.800	8.446e-4	2.429e-3	0.793	12.833	1.634e-3	4.711e-3	1.149	12.680	3.316e-2	4.413e-2	-1.272	10.444	1.659e-1	2.285e-1	2.421	11.593	8.024e-4	1.446e-3
FN1501	AAL93627.1| Nickel transport ATP-binding protein nikD																								
FN1504	AAL93630.1| Nickel-binding protein	-1.567	13.803	5.632e-5	5.926e-6	-0.873	14.681	3.772e-3	1.5e-2	-0.874	14.292	9.725e-5	1.044e-4	0.693	12.929	7.972e-3	5.287e-3	0.694	13.114	1.224e-1	1.242e-1	-0.001	13.807	1.875e-1	9.983e-1
FN1505	AAL93631.1| 6,7-dimethyl-8-ribityllumazine synthase	-0.020	16.220	2.836e-1	8.827e-1	-0.463	15.961	1.136e-3	3.469e-3	-0.776	15.260	2.495e-4	3.975e-4	-0.756	15.444	1.474e-2	1.372e-2	-0.443	15.941	6.778e-2	3.897e-2	-0.313	15.185	1.25e-2	4.571e-2
FN1506	AAL93632.1| Diaminohydroxyphosphoribosylaminopyrimidine deaminase	0.684	7.889			-0.525	6.865			0.717	7.719			0.033	8.606	2.755e-1	9.117e-1	-1.209	7.549			1.242	7.582		
FN1507	AAL93633.1| Riboflavin synthase alpha chain																								
FN1508	AAL93634.1| GTP cyclohydrolase II	0.227	13.127	1.712e-1	4.533e-1	-0.566	12.519	1.493e-2	7.418e-2	0.624	13.320	1.54e-4	1.998e-4	0.397	13.751	7.947e-2	1.544e-1	-0.792	12.746	1.003e-1	8.55e-2	1.190	13.143	2.274e-3	5.431e-3
FN1512	AAL93638.1| hypothetical exported 24-amino acid repeat protein									-1.700	5.249	5.512e-4	1.129e-3												
FN1517	AAL93643.1| Leucyl-tRNA synthetase	-0.545	15.824	4.167e-2	6.973e-2	-0.956	15.597	5.742e-4	1.469e-3	-0.240	15.926	1.464e-2	6.337e-2	0.306	15.584	1.128e-1	2.654e-1	-0.411	15.052	1.736e-1	2.47e-1	0.717	15.358	4.067e-5	2.59e-5
FN1518	AAL93644.1| RNA polymerase sigma-H factor					-0.740	5.910																		
FN1519	AAL93645.1| 23S rRNA methyltransferase	-0.956	8.357			-0.504	8.993	1.798e-3	6.002e-3	-0.052	9.057	8.335e-2	4.58e-1	0.904	8.305			0.452	8.038			0.452	8.942	2.137e-3	5.019e-3
FN1520	AAL93646.1| UDP-N-acetylglucosamine 1-carboxyvinyltransferase	-1.151	10.981	2.418e-2	3.44e-2	-1.051	11.266	4.193e-5	4.721e-5	0.821	12.750	1.369e-3	3.704e-3	1.973	11.802	1.938e-3	6.702e-4	0.100	10.114	3.942e-1	8.496e-1	1.873	12.087	3.96e-4	5.496e-4
FN1521	AAL93647.1| Dipeptide transport system permease protein dppB																								

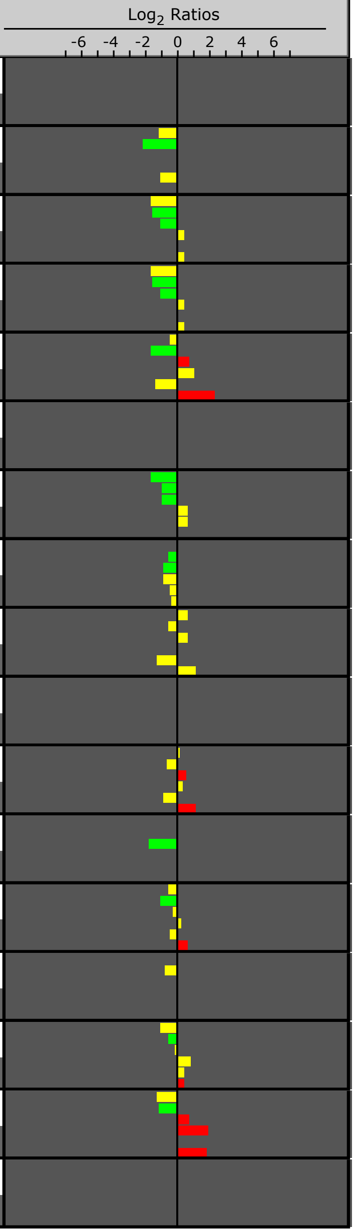

Show detected proteins only
Show all proteins
Filter by category:
GO: amino acid transport

Proteins found: 1424

Enter (or paste) list of ORFs

Find ORFs

Test	Cutoff
q-Value	.005
p-Value	

Dot Plots
Dot Plots

	Signif	Direction	Applies To
(red)	yes	+	ratios, bars
(yellow)	no	n/a	bars
(green)	yes	–	ratios, bars
(pink)	yes	+	*p*- , *q*-Values
(light green)	yes	–	*p*- , *q*-Values

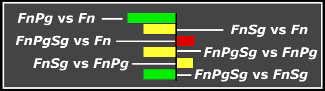

ORF	Description	FnPg vs Fn Ratio	Sum	*q*-Val	*p*-Val	FnSg vs Fn Ratio	Sum	*q*-Val	*p*-Val	FnPgSg vs Fn Ratio	Sum	*q*-Val	*p*-Val	FnPgSg vs FnPg Ratio	Sum	*q*-Val	*p*-Val	FnSg vs FnPg Ratio	Sum	*q*-Val	*p*-Val	FnPgSg vs FnSg Ratio	Sum	*q*-Val	*p*-Val
FN1523	AAL93649.1| Dipeptide-binding protein	-2.022	15.040	1.623e-5	9.748e-7	-2.225	15.021	2.363e-7	2.072e-8	-0.519	16.339	1.033e-4	1.148e-4	1.503	14.521	7.781e-4	1.643e-4	-0.203	13.000	1.072e-1	9.664e-2	1.706	14.503	1.385e-5	4.947e-6
FN1524	AAL93650.1| Dipeptide transport ATP-binding protein dppD																								
FN1525	AAL93651.1| Dipeptide transport ATP-binding protein dppF									0.442	5.443														
FN1526	AAL93652.1| Fusobacterium outer membrane protein family	-0.352	22.555	1.721e-1	4.564e-1	-2.550	20.542	5.399e-6	2.151e-6	-0.638	22.065	3.842e-4	7.109e-4	-0.286	21.917	2.052e-1	6.188e-1	-2.198	20.190	1.055e-1	9.383e-2	1.911	19.903	4.134e-4	5.808e-4
FN1527	AAL93653.1| Hypothetical protein	-2.812	12.685	4.914e-4	1.512e-4	-3.030	12.651	5.453e-5	6.86e-5	-0.874	14.420	1.588e-4	2.084e-4	1.938	11.812	2.33e-3	8.551e-4	-0.218	9.840	1.995e-1	3.118e-1	2.156	11.778	2.965e-4	3.802e-4
FN1528	AAL93654.1| Hypothetical protein	-1.987	13.504	7.129e-4	2.683e-4	-1.980	13.695	7.134e-5	1.018e-4	-0.950	14.337	1.05e-3	2.551e-3	1.037	12.554	1.318e-2	1.148e-2	0.007	11.708	4.299e-1	9.843e-1	1.030	12.745	3.81e-3	1.044e-2
FN1529	AAL93655.1| Hypothetical protein	-0.496	12.897	1.244e-1	2.989e-1	-2.743	10.835	1.903e-5	1.53e-5	-0.438	12.752	2.029e-2	9.178e-2	0.058	12.459	2.761e-1	9.146e-1	-2.247	10.339	1.041e-1	9.151e-2	2.305	10.397	2.849e-3	7.201e-3
FN1531	AAL93657.1| murein hydrolase export regulator	0.267	9.845	1.848e-1	5.038e-1	0.931	10.693	2.863e-5	2.691e-5	0.821	10.195	1.847e-2	8.262e-2	0.554	10.667	9.789e-2	2.119e-1	0.663	10.961	9.296e-2	7.46e-2	-0.110	11.514	1.356e-1	6.788e-1
FN1533	AAL93659.1| Electron transfer flavoprotein alpha-subunit	0.427	19.096	1.955e-1	5.418e-1	-0.564	18.290	6.836e-4	1.846e-3	0.285	18.751	1.004e-4	1.097e-4	-0.141	19.382	2.545e-1	8.186e-1	-0.991	18.717	1.852e-1	2.753e-1	0.849	18.575	1.527e-4	1.522e-4
FN1534	AAL93660.1| Electron transfer flavoprotein beta-subunit	0.680	20.164	1.196e-1	2.84e-1	-0.096	19.572	3.63e-2	1.973e-1	0.052	19.332	8.7e-2	4.807e-1	-0.628	20.216	1.251e-1	3.091e-1	-0.777	20.252	1.727e-1	2.449e-1	0.149	19.624	1.102e-2	3.942e-2
FN1535	AAL93661.1| Acyl-CoA dehydrogenase, short-chain specific	1.049	21.288	4.761e-3	4.263e-3	0.483	20.907	6.664e-3	2.893e-2	0.258	20.294	7.127e-4	1.563e-3	-0.790	21.547	1.079e-2	8.541e-3	-0.565	21.956	3.373e-2	1.122e-2	-0.225	21.166	3.56e-2	1.514e-1
FN1536	AAL93662.1| (S)-2-hydroxy-acid oxidase chain D	1.713	19.223	2.984e-3	2.224e-3	1.421	19.115	1.569e-3	5.121e-3	0.403	17.709	1.128e-4	1.295e-4	-1.310	19.626	6.391e-3	3.632e-3	-0.292	20.828	1.193e-1	1.182e-1	-1.018	19.518	3.619e-3	9.751e-3
FN1537	AAL93663.1| Arsenical pump-driving ATPase	-0.815	5.724			-1.040	5.684											-0.225	4.868						
FN1538	AAL93664.1| Arsenical pump-driving ATPase	-0.071	7.783	2.556e-1	7.654e-1	0.712	8.750	4.796e-3	1.973e-2	-0.655	6.995			-0.584	7.128			0.783	8.680	4.243e-2	1.672e-2	-1.367	8.095		
FN1539	AAL93665.1| Iron-sulfur cluster-binding protein	0.182	16.003	1.694e-1	4.465e-1	-0.327	15.678	1.615e-4	2.967e-4	-0.327	15.290	2.81e-4	4.633e-4	-0.510	15.676	6.401e-2	1.143e-1	-0.510	15.861	1.176e-1	1.149e-1	0.000	15.351	1.875e-1	9.985e-1
FN1540	AAL93666.1| Iron-sulfur cluster-binding protein	1.067	16.840	2.35e-2	3.318e-2	0.786	16.744	1.611e-4	2.958e-4	-0.732	14.837	7.582e-5	7.271e-5	-1.799	16.108	1.516e-2	1.439e-2	-0.281	17.811	1.944e-1	2.997e-1	-1.518	16.012	7.272e-5	5.677e-5
FN1544	AAL93670.1| Probable electron transfer flavoprotein-quinone oxidoreductase ydiS	1.827	17.153	4.206e-2	7.062e-2	1.512	17.022	4.949e-4	1.211e-3	-0.117	15.005	4.955e-2	2.56e-1	-1.944	17.036	4.389e-2	6.559e-2	-0.315	18.849	2.807e-1	5.095e-1	-1.629	16.905	3.492e-4	4.696e-4

Log$_2$ Ratios: -6 -4 -2 0 2 4 6

Show detected proteins only
Show all proteins
Filter by category:
GO: amino acid transport

Proteins found: 1424

Enter (or paste) list of ORFs

Find ORFs

Test	Cutoff
q-Value	.005
p-Value	

Dot Plots
Dot Plots

	Signif	Direction	Applies To
red	yes	+	ratios, bars
yellow	no	n/a	bars
green	yes	–	ratios, bars
pink	yes	+	*p*-, *q*-Values
light green	yes	–	*p*-, *q*-Values

FnPg vs Fn
FnSg vs Fn
FnPgSg vs Fn
FnPgSg vs FnPg
FnSg vs FnPg
FnPgSg vs FnSg

ORF	Description	FnPg vs Fn				FnSg vs Fn				FnPgSg vs Fn				FnPgSg vs FnPg				FnSg vs FnPg				FnPgSg vs FnSg			
		Ratio	Sum	q-Val	p-Val	Ratio	Sum	q-Val	p-Val	Ratio	Sum	q-Val	p-Val	Ratio	Sum	q-Val	p-Val	Ratio	Sum	q-Val	p-Val	Ratio	Sum	q-Val	p-Val
FN1545	AAL93671.1| Ferredoxin like protein	2.272	11.903	7.697e-2	1.614e-1	0.334	10.149	2.383e-2	1.242e-1	-0.225	9.202	7.222e-2	3.895e-1	-2.497	11.678	7.813e-2	1.507e-1	-1.939	12.421	1.475e-1	1.832e-1	-0.558	9.924	5.84e-3	1.832e-2
FN1546	AAL93672.1| Protein Translation Elongation Factor G (EF-G)	1.071	20.811	7.679e-2	1.609e-1	0.545	20.470	5.559e-4	1.409e-3	0.606	20.143	2.58e-4	4.147e-4	-0.465	21.417	1.501e-1	4.019e-1	-0.526	21.541	2.201e-1	3.6e-1	0.061	21.076	7.716e-2	3.617e-1
FN1547	AAL93673.1| PTS permease for N-acetylglucosamine and glucose	-0.720	14.595	5.234e-3	4.838e-3	-0.863	14.636	2.552e-5	2.31e-5	-0.316	14.795	3.208e-5	1.915e-5	0.404	14.279	2.769e-2	3.382e-2	-0.143	13.916	2.21e-1	3.623e-1	0.547	14.320	3.02e-4	3.895e-4
FN1548	AAL93674.1| Hypothetical protein	-0.490	10.041	8.847e-4	3.888e-4	-0.370	10.345	7.522e-2	4.322e-1	0.477	10.804	3.204e-2	1.553e-1	0.967	10.518	3.4e-2	4.586e-2	0.120	9.855	3.86e-1	8.207e-1	0.847	10.823	2.627e-2	1.073e-1
FN1549	AAL93675.1| Stomatin like protein	-0.602	19.011	6.144e-3	6.014e-3	-0.254	19.544	4.367e-2	2.407e-1	0.343	19.753	2.978e-3	9.795e-3	0.945	19.354	1.889e-3	6.427e-4	0.348	18.942	1.557e-1	2.042e-1	0.597	19.887	7.166e-3	2.351e-2
FN1552	AAL93678.1| abortive phage resistance protein	-1.922	5.922			-0.844	7.184											1.078	5.262						
FN1553	AAL93679.1| abortive phage resistance protein	-1.121	7.900	2.257e-3	1.526e-3	-0.142	9.062	1.157e-1	6.955e-1	-0.038	8.779	1.476e-1	8.762e-1	1.083	7.862	2.959e-2	3.729e-2	0.978	7.942	1.243e-1	1.279e-1	0.105	9.025	1.563e-1	8.012e-1
FN1554	AAL93680.1| Fusobacterium outer membrane protein family	-0.887	16.740	1.18e-3	6.182e-4	-1.338	16.473	1.007e-4	1.577e-4	-0.953	16.471	3.379e-4	5.945e-4	-0.065	15.787	2.053e-1	6.193e-1	-0.451	15.586	8.303e-2	5.837e-2	0.386	15.521	2.281e-2	9.171e-2
FN1555	AAL93681.1| Protein Translation Elongation Factor Tu	-0.096	23.668	1.81e-2	2.377e-2	0.072	24.020	1.043e-2	4.901e-2	0.494	24.054	7.898e-6	2.287e-6	0.590	24.162	3.299e-5	1.409e-6	0.168	23.924	2.456e-3	1.746e-4	0.422	24.514	3.167e-5	1.753e-5
FN1556	AAL93682.1| Protein Translation Elongation Factor G (EF-G)	-0.929	20.112	1.178e-3	6.165e-4	-1.342	19.883	4.77e-4	1.154e-3	0.097	20.934	2.3e-2	1.057e-1	1.026	20.209	1.398e-4	1.616e-5	-0.413	18.954	9.26e-2	7.395e-2	1.439	19.980	9.46e-5	8.076e-5
FN1557	AAL93683.1| SSU ribosomal protein S7P	0.863	13.989	7.109e-7	1.726e-8	0.505	13.816	6.96e-5	9.795e-5	2.045	14.967	9.541e-5	1.014e-4	1.182	16.033	9.898e-4	2.369e-4	-0.358	14.678	1.171e-3	4.066e-5	1.540	15.860	1.154e-4	1.039e-4
FN1558	AAL93684.1| SSU ribosomal protein S12P													0.138	13.531										
FN1560	AAL93686.1| unknown	-0.560	13.953	1.565e-1	4.016e-1	0.726	15.424	1.205e-5	7.989e-6	-0.656	13.653	1.924e-4	2.739e-4	-0.096	13.297	2.736e-1	9.028e-1	1.286	14.864	8.326e-2	5.871e-2	-1.383	14.767	9.93e-6	2.895e-6
FN1562	AAL93688.1| Phospho-2-dehydro-3-deoxyheptonate aldolase	-1.156	6.793	8.204e-3	8.941e-3	-0.144	7.989	2.775e-2	1.468e-1	-1.550	6.194			-0.395	5.243			1.011	6.833	4.761e-2	2.068e-2	-1.406	6.438		
FN1576	AAL93691.1| ATPases and helicase subunits involved in DNA replication																								
FN1577	AAL93692.1| Rod shape-determining protein mreB	0.437	15.288	1.17e-1	2.763e-1	1.133	16.169	4.636e-5	5.509e-5	-0.079	14.568	3.613e-2	1.788e-1	-0.517	15.208	1.011e-1	2.234e-1	0.696	16.606	7.488e-2	4.715e-2	-1.213	16.089	2.63e-4	3.247e-4
FN1579	AAL93694.1| Cysteinyl-tRNA synthetase	-0.033	12.799	2.884e-1	9.037e-1	0.259	13.276	1.429e-2	7.063e-2	-0.459	12.170	4.335e-3	1.539e-2	-0.425	12.341	1.029e-1	2.3e-1	0.292	13.243	1.894e-1	2.863e-1	-0.718	12.817	1.064e-3	2.105e-3

Log$_2$ Ratios: -6 -4 -2 0 2 4 6

Show detected proteins only
Show all proteins
Filter by category:
GO: amino acid transport

Proteins found: 1424

Enter (or paste) list of ORFs

Find ORFs

Test	Cutoff
q-Value	.005
p-Value	

Dot Plots
Dot Plots

	Signif	Direction	Applies To
red	yes	+	ratios, bars
yellow	no	n/a	bars
green	yes	–	ratios, bars
pink	yes	+	p- , q-Values
light green	yes	–	p- , q-Values

FnPg vs Fn — FnSg vs Fn
FnPgSg vs Fn — FnPgSg vs FnPg
FnSg vs FnPg — FnPgSg vs FnSg

ORF	Description	FnPg vs Fn Ratio	FnPg vs Fn Sum	FnPg vs Fn *q*-Val	FnPg vs Fn *p*-Val	FnSg vs Fn Ratio	FnSg vs Fn Sum	FnSg vs Fn *q*-Val	FnSg vs Fn *p*-Val	FnPgSg vs Fn Ratio	FnPgSg vs Fn Sum	FnPgSg vs Fn *q*-Val	FnPgSg vs Fn *p*-Val	FnPgSg vs FnPg Ratio	FnPgSg vs FnPg Sum	FnPgSg vs FnPg *q*-Val	FnPgSg vs FnPg *p*-Val	FnSg vs FnPg Ratio	FnSg vs FnPg Sum	FnSg vs FnPg *q*-Val	FnSg vs FnPg *p*-Val	FnPgSg vs FnSg Ratio	FnPgSg vs FnSg Sum	FnPgSg vs FnSg *q*-Val	FnPgSg vs FnSg *p*-Val
FN1580	AAL93695.1| 2-C-methyl-D-erythritol 4-phosphate cytidylyltransferase					-1.373	4.543																		
FN1581	AAL93696.1| DNA mismatch repair protein mutS	-0.576	10.825	1.57e-2	1.97e-2	0.409	11.994	2.001e-2	1.025e-1	-0.610	10.586	4.59e-3	1.647e-2	-0.035	10.214	2.59e-1	8.38e-1	0.985	11.419	3.48e-2	1.189e-2	-1.020	11.384	5.226e-3	1.589e-2
FN1582	AAL93697.1| Hypothetical protein	0.608	8.310			0.568	8.455											-0.040	9.063						
FN1586	AAL93701.1| O-succinylbenzoate-CoA synthase																	-0.890	7.635						
FN1589	AAL93704.1| LexA repressor	-1.771	9.665	2.161e-3	1.443e-3	-0.798	10.823	3.784e-3	1.506e-2	-1.276	9.956	2.001e-3	6.064e-3	0.495	8.389	1.205e-1	2.925e-1	0.973	9.052	8.254e-2	5.765e-2	-0.478	9.546	2.575e-7	1.687e-8
FN1590	AAL93705.1| Hypothetical lipoprotein																	-0.431	4.397						
FN1591	AAL93706.1| RNFB-related protein	-0.869	15.690	1.049e-3	5.071e-4	-0.828	15.915	1.573e-3	5.135e-3	-0.438	15.917	1.34e-4	1.648e-4	0.431	15.252	1.435e-2	1.313e-2	0.041	15.046	3.943e-1	8.498e-1	0.390	15.478	1.912e-2	7.478e-2
FN1592	AAL93707.1| Na(+)-translocating NADH-quinone reductase subunit D													0.344	7.745			-1.208	6.378			1.552	6.722		
FN1594	AAL93709.1| Nitrogen fixation protein RNFG	-0.519	11.095	2.673e-2	3.9e-2	-0.772	11.027	3.114e-4	6.754e-4	0.815	12.225	3.051e-5	1.748e-5	1.334	11.910	2.603e-3	9.967e-4	-0.252	10.508	2.101e-1	3.36e-1	1.586	11.842	4.133e-5	2.661e-5
FN1595	AAL93710.1| Na(+)-translocating NADH-quinone reductase subunit B	0.081	14.238	2.867e-1	8.961e-1	-0.390	13.951	3.337e-3	1.283e-2	0.443	14.395	9.011e-4	2.09e-3	0.361	14.681	1.798e-1	5.125e-1	-0.471	14.033	2.854e-1	5.215e-1	0.832	14.394	1.055e-4	9.263e-5
FN1596	AAL93711.1| Nitrogen fixation iron-sulphur protein RNFC	-0.888	19.764	2.767e-2	4.074e-2	-1.721	19.116	5.396e-5	6.761e-5	-0.538	19.910	1.288e-3	3.388e-3	0.350	19.226	1.36e-1	3.515e-1	-0.833	18.228	1.435e-1	1.721e-1	1.183	18.578	2.504e-4	3.016e-4
FN1597	AAL93712.1| Peptidyl-tRNA hydrolase	0.789	8.764							-1.886	5.886			-2.674	6.878										
FN1600	AAL93715.1| tRNA pseudouridine synthase A	0.594	6.325			-0.202	5.714											-0.796	6.307						
FN1601	AAL93716.1| Hypothetical cytosolic protein					0.726	5.694																		
FN1602	AAL93717.1| Hypothetical cytosolic protein																								
FN1603	AAL93718.1| 2',3'-cyclic nucleotide 3'-phosphodiesterase	-2.122	7.031			-2.466	6.871	4.153e-4	9.665e-4	-1.777	7.171	2.058e-4	3.034e-4	0.345	5.253			-0.344	4.749			0.688	5.094	4.226e-2	1.843e-1
FN1605	AAL93720.1| Adenylosuccinate synthetase	-0.300	16.736	7.979e-2	1.693e-1	-0.305	16.915	9.648e-4	2.853e-3	-0.533	16.299	3.651e-4	6.636e-4	-0.233	16.203	1.305e-1	3.296e-1	-0.006	16.615	4.282e-1	9.776e-1	-0.227	16.382	1.356e-5	4.77e-6

Log$_2$ Ratios: -6 -4 -2 0 2 4 6

Show detected proteins only
Show all proteins
Filter by category:
GO: amino acid transport

Proteins found: 1424

Enter (or paste) list of ORFs

Find ORFs

Test	Cutoff
q-Value	.005
p-Value	

Dot Plots
Dot Plots

	Signif	Direction	Applies To
red	yes	+	ratios, bars
yellow	no	n/a	bars
green	yes	–	ratios, bars
pink	yes	+	*p*- , *q*-Values
light green	yes	–	*p*- , *q*-Values

FnPg vs Fn
FnPgSg vs Fn
FnSg vs FnPg
FnSg vs Fn
FnPgSg vs FnPg
FnPgSg vs FnSg

		FnPg vs Fn				*FnSg vs Fn*				*FnPgSg vs Fn*				*FnPgSg vs FnPg*				*FnSg vs FnPg*				*FnPgSg vs FnSg*			
ORF		Ratio	Sum	*q*-Val	*p*-Val	Ratio	Sum	*q*-Val	*p*-Val	Ratio	Sum	*q*-Val	*p*-Val	Ratio	Sum	*q*-Val	*p*-Val	Ratio	Sum	*q*-Val	*p*-Val	Ratio	Sum	*q*-Val	*p*-Val
FN1606	AAL93721.1| 3-deoxy-D-manno-octulosonic-acid transferase	-1.640	10.113	4.178e-4	1.15e-4	-0.891	11.047	8.397e-5	1.259e-4	-1.934	9.616	5.688e-6	1.373e-6	-0.294	8.179	1.352e-1	3.481e-1	0.749	9.406	4.704e-2	2.022e-2	-1.043	9.113	2.985e-4	3.836e-4
FN1607	AAL93722.1| Cytidylate kinase	-0.404	8.668	4.08e-2	6.775e-2	-0.342	8.914	5.076e-2	2.837e-1	-1.077	7.792			-0.673	7.592			0.062	8.510	3.944e-1	8.503e-1	-0.734	7.838		
FN1608	AAL93723.1| Ribosomal protein L11 methyltransferase																	0.087	6.271						
FN1609	AAL93724.1| Hypothetical protein	-2.360	9.530			-3.883	8.192	1.548e-4	2.81e-4	-3.843	7.843			-1.483	5.687			-1.523	5.832			0.040	4.349		
FN1610	AAL93725.1| 33 kDa chaperonin	0.674	9.850	5.91e-4	1.98e-4	0.512	9.873	4.198e-4	9.797e-4	-0.837	8.135	3.9e-4	7.255e-4	-1.511	9.013	3.332e-5	1.433e-6	-0.161	10.547	3.236e-2	1.041e-2	-1.349	9.036	1.228e-5	4.038e-6
FN1611	AAL93726.1| Competence protein																								
FN1613	AAL93728.1| Hypothetical protein	-0.193	8.730	2.094e-2	2.87e-2	-0.305	8.802	5.135e-2	2.873e-1	-0.131	8.589	3.769e-2	1.877e-1	0.062	8.599	1.88e-1	5.455e-1	-0.112	8.610	3.461e-1	6.909e-1	0.174	8.672	1.115e-1	5.429e-1
FN1614	AAL93729.1| MG(2+) chelatase family protein	-0.398	10.108	6.677e-2	1.343e-1	-1.318	9.373	3.635e-3	1.431e-2	-0.923	9.380	6.385e-3	2.443e-2	-0.524	9.186	2.064e-2	2.22e-2	-0.920	8.975	2.841e-2	8.311e-3	0.395	8.451	5.26e-3	1.603e-2
FN1616	AAL93731.1| N utilization substance protein B	-0.145	8.145			-0.652	7.822			1.703	9.788	1.199e-2	5.059e-2	1.848	9.848			-0.507	7.677			2.355	9.525		
FN1618	AAL93733.1| Hypothetical protein	0.013	10.298	3.024e-1	9.667e-1	0.513	10.982	2.119e-3	7.304e-3	0.513	10.595	1.399e-3	3.82e-3	0.500	10.812	6.944e-2	1.277e-1	0.500	10.996	1.242e-1	1.277e-1	0.001	11.496	1.87e-1	9.948e-1
FN1619	AAL93734.1| Hypothetical cytosolic protein	1.079	14.402	5.791e-3	5.542e-3	1.088	14.596	8.853e-3	4.044e-2	1.804	14.923	1.135e-3	2.836e-3	0.725	16.205	1.195e-2	9.897e-3	0.010	15.674	4.265e-1	9.71e-1	0.716	16.400	6.592e-3	2.122e-2
FN1620	AAL93735.1| SSU ribosomal protein S2P	-0.686	18.440	3.672e-2	5.888e-2	-1.141	18.170	5.5e-5	6.944e-5	-0.186	18.736	7.55e-3	2.966e-2	0.500	18.254	7.539e-2	1.433e-1	-0.455	17.483	1.904e-1	2.889e-1	0.955	17.983	4.72e-5	3.227e-5
FN1621	AAL93736.1| Protein Translation Elongation Factor Ts	-0.815	20.738	1.155e-2	1.346e-2	-1.772	19.966	2.151e-6	5.041e-7	-0.264	21.085	1.658e-2	7.3e-2	0.551	20.474	3.868e-2	5.494e-2	-0.957	19.151	7.665e-2	4.943e-2	1.508	19.702	1.141e-3	2.309e-3
FN1622	AAL93737.1| Uridylate kinase	-1.405	14.538	1.889e-3	1.184e-3	-1.300	14.828	2.136e-3	7.376e-3	-0.173	15.566	1.593e-2	6.974e-2	1.232	14.365	1.129e-3	2.962e-4	0.105	13.423	3.79e-1	7.969e-1	1.127	14.655	3.045e-3	7.82e-3
FN1623	AAL93738.1| Ribosome Recycling Factor (RRF)	-1.639	12.064	1.382e-3	7.695e-4	-2.822	11.066	3.864e-4	8.838e-4	-0.657	12.842	3.933e-3	1.364e-2	0.982	11.407	3.319e-3	1.433e-3	-1.182	9.427	2.799e-2	8.104e-3	2.164	10.409	3.734e-6	6.834e-7
FN1624	AAL93739.1| Protein translocase subunit secY	-2.007	10.926			-2.311	10.807			-0.341	12.388	6.462e-4	1.384e-3	1.666	10.584			-0.304	8.800			1.969	10.465		
FN1625	AAL93740.1| LSU ribosomal protein L15P	-1.012	13.664	1.985e-2	2.686e-2	-1.252	13.608	3.134e-5	3.048e-5	-0.387	14.085	1.329e-3	3.546e-3	0.625	13.277	6.993e-2	1.29e-1	-0.240	12.596	3.144e-1	5.987e-1	0.865	13.221	6.324e-5	4.749e-5

Log_2 Ratios: -6 -4 -2 0 2 4 6

Show detected proteins only
Show all proteins
Filter by category:
GO: amino acid transport

Proteins found: 1424

Enter (or paste) list of ORFs

Find ORFs

Test	Cutoff
q-Value	.005
p-Value	

Dot Plots
Dot Plots

	Signif	Direction	Applies To
red	yes	+	ratios, bars
yellow	no	n/a	bars
green	yes	–	ratios, bars
pink	yes	+	*p*- , *q*-Values
light green	yes	–	*p*- , *q*-Values

FnPg vs Fn — FnSg vs Fn
FnPgSg vs Fn — FnPgSg vs FnPg
FnSg vs FnPg — FnPgSg vs FnSg

ORF	Protein	FnPg vs Fn Ratio	FnPg vs Fn Sum	FnPg vs Fn q-Val	FnPg vs Fn p-Val	FnSg vs Fn Ratio	FnSg vs Fn Sum	FnSg vs Fn q-Val	FnSg vs Fn p-Val	FnPgSg vs Fn Ratio	FnPgSg vs Fn Sum	FnPgSg vs Fn q-Val	FnPgSg vs Fn p-Val	FnPgSg vs FnPg Ratio	FnPgSg vs FnPg Sum	FnPgSg vs FnPg q-Val	FnPgSg vs FnPg p-Val	FnSg vs FnPg Ratio	FnSg vs FnPg Sum	FnSg vs FnPg q-Val	FnSg vs FnPg p-Val	FnPgSg vs FnSg Ratio	FnPgSg vs FnSg Sum	FnPgSg vs FnSg q-Val	FnPgSg vs FnSg p-Val
FN1626	AAL93741.1| LSU ribosomal protein L30P	-3.376	9.716			-4.316	8.960			-0.900	11.987	7.562e-5	7.244e-5	2.475	8.815			-0.940	5.584			3.416	8.060		
FN1627	AAL93742.1| SSU ribosomal protein S5P	-0.912	16.987	2.226e-4	4.499e-5	-1.029	17.054	6.439e-6	2.95e-6	0.508	18.203	1.979e-2	8.923e-2	1.420	17.495	1.469e-2	1.365e-2	-0.117	16.142	1.286e-1	1.369e-1	1.537	17.562	4.071e-3	1.141e-2
FN1628	AAL93743.1| LSU ribosomal protein L18P	0.419	15.521			-3.678	11.608			0.274	15.172	3.376e-2	1.653e-1	-0.145	15.795			-4.097	12.028			3.952	11.883		
FN1629	AAL93744.1| LSU ribosomal protein L6P	-0.876	17.655	3.913e-2	6.405e-2	-1.720	16.996	1.419e-6	2.647e-7	-1.235	17.092	1.41e-5	5.192e-6	-0.359	16.420	1.793e-1	5.103e-1	-0.844	16.119	1.659e-1	2.283e-1	0.485	15.761	4.258e-3	1.213e-2
FN1630	AAL93745.1| SSU ribosomal protein S8P	-0.713	13.210	1.009e-3	4.763e-4	-0.930	13.178	2.757e-5	2.558e-5	0.382	14.101	6.248e-2	3.316e-1	1.095	13.592	4.901e-2	7.716e-2	-0.217	12.465	6.395e-2	3.507e-2	1.312	13.560	1.543e-2	5.831e-2
FN1631	AAL93746.1| SSU ribosomal protein S14P	-1.281	5.924			-1.730	5.660			1.383	8.384			2.663	7.307			-0.449	4.379			3.112	7.043		
FN1632	AAL93747.1| LSU ribosomal protein L5P	-1.015	16.452	7.678e-4	3.029e-4	-0.907	16.744	6.301e-5	8.445e-5	-0.245	17.018	1.921e-3	5.772e-3	0.770	16.207	3.245e-4	4.639e-5	0.107	15.729	2.263e-1	3.754e-1	0.663	16.499	9.545e-4	1.822e-3
FN1634	AAL93749.1| LSU ribosomal protein L24P	0.794	11.224			-1.227	9.388			0.349	10.575	2.107e-2	9.573e-2	-0.445	11.573			-2.020	10.182			1.575	9.737		
FN1635	AAL93750.1| LSU ribosomal protein L14P	-1.370	9.635	6.473e-5	7.232e-6	-2.045	9.144	5.747e-6	2.393e-6	0.958	11.759	4.306e-3	1.526e-2	2.328	10.593	6.093e-3	3.372e-3	-0.675	7.774	3.498e-2	1.201e-2	3.003	10.102	1.381e-3	2.914e-3
FN1636	AAL93751.1| SSU ribosomal protein S17P	-1.670	10.912	5.613e-3	5.312e-3	-0.115	12.651	8.779e-2	5.114e-1	-1.114	11.264	2.879e-3	9.391e-3	0.556	9.799	1.266e-1	3.146e-1	1.554	10.981	3.977e-2	1.49e-2	-0.998	11.537	5.464e-4	8.462e-4
FN1637	AAL93752.1| LSU ribosomal protein L29P									-0.289	10.697	1.072e-1	6.077e-1												
FN1638	AAL93753.1| LSU ribosomal protein L16P	-2.223	14.563	2.355e-4	4.924e-5	-3.449	13.521	2.494e-5	2.24e-5	-0.088	16.494	2.375e-2	1.096e-1	2.135	14.475	1.758e-3	5.738e-4	-1.226	11.299	9.84e-2	8.267e-2	3.360	13.433	1.988e-8	3.763e-10
FN1639	AAL93754.1| SSU ribosomal protein S3P	-0.602	19.260	3.975e-2	6.54e-2	-1.073	18.973	3.058e-6	9.209e-7	0.286	19.945	1.71e-2	7.562e-2	0.889	19.546	1.293e-2	1.113e-2	-0.471	18.371	1.656e-1	2.276e-1	1.360	19.260	1.117e-3	2.248e-3
FN1640	AAL93755.1| LSU ribosomal protein L22P	-0.669	13.873	4.407e-2	7.534e-2	-2.077	12.650	1.394e-6	2.562e-7	1.630	15.969	1.477e-3	4.125e-3	2.300	15.503	1.902e-3	6.501e-4	-1.408	11.980	8.823e-2	6.657e-2	3.707	14.280	8.917e-4	1.669e-3
FN1641	AAL93756.1| SSU ribosomal protein S19P	-0.944	14.982	3.583e-2	5.703e-2	-3.818	12.292	1.272e-4	2.145e-4	-0.028	15.694	1.364e-1	7.991e-1	0.916	14.954	4.351e-2	6.477e-2	-2.874	11.348	9.194e-2	7.279e-2	3.790	12.264	8.772e-5	7.307e-5
FN1642	AAL93757.1| LSU ribosomal protein L2P	-1.187	17.039	2.304e-2	3.236e-2	-1.562	16.848	1.176e-5	7.651e-6	-1.107	16.915	2.324e-4	3.626e-4	0.080	15.932	2.689e-1	8.816e-1	-0.375	15.662	3.009e-1	5.621e-1	0.455	15.741	5.236e-3	1.593e-2
FN1643	AAL93758.1| LSU ribosomal protein L23P	0.230	13.378	2.394e-1	7.017e-1	-1.515	11.817	4.153e-5	4.649e-5	-0.010	12.933	1.377e-1	8.084e-1	-0.241	13.367	2.237e-1	6.907e-1	-1.746	12.047	1.294e-1	1.385e-1	1.505	11.806	9.626e-6	2.727e-6

Log$_2$ Ratios: -6 -4 -2 0 2 4 6

Show detected proteins only
Show all proteins
Filter by category:
GO: amino acid transport

Proteins found: 1424

Enter (or paste) list of ORFs

Find ORFs

Test	Cutoff
q-Value	.005
p-Value	

Dot Plots
Dot Plots

	Signif	Direction	Applies To
red	yes	+	ratios, bars
yellow	no	n/a	bars
green	yes	–	ratios, bars
pink	yes	+	p- , q-Values
light green	yes	–	p- , q-Values

FnPg vs Fn — FnSg vs Fn
FnPgSg vs Fn — FnPgSg vs FnPg
FnSg vs FnPg — FnPgSg vs FnSg

ORF	Protein	*FnPg vs Fn* Ratio	Sum	*q*-Val	*p*-Val	*FnSg vs Fn* Ratio	Sum	*q*-Val	*p*-Val	*FnPgSg vs Fn* Ratio	Sum	*q*-Val	*p*-Val
FN1644	AAL93759.1| LSU ribosomal protein L1E	-0.619	19.366	1.84e-2	2.429e-2	-0.267	19.903	2.718e-4	5.627e-4	0.360	20.141	3.73e-5	2.586e-5
FN1645	AAL93760.1| LSU ribosomal protein L3P	-1.468	17.056	7.871e-3	8.459e-3	-2.777	15.931	4.367e-5	5.02e-5	0.481	18.801	1.256e-4	1.503e-4
FN1646	AAL93761.1| SSU ribosomal protein S10P	-0.665	15.556	5.081e-2	9.155e-2	-1.473	14.933	1.283e-6	2.219e-7	-0.451	15.567	4.517e-3	1.616e-2
FN1647	AAL93762.1| Hypothetical protein	-0.316	17.063	2.085e-1	5.874e-1	-1.901	15.663	6.584e-4	1.755e-3	0.273	17.449	3.025e-2	1.451e-1
FN1652	AAL93767.1| Oligopeptide-binding protein oppA	-1.992	13.422	1.418e-4	2.337e-5	-1.609	13.990	1.009e-6	1.521e-7	-1.164	14.047	4.821e-5	3.877e-5
FN1654	AAL93769.1| Hypothetical protein	0.914	10.762	9.644e-2	2.167e-1	1.958	11.991	2.458e-7	2.208e-8	0.302	9.947	1.47e-2	6.368e-2
FN1655	AAL93770.1| Hypothetical cytosolic protein	-0.122	12.930	2.315e-1	6.717e-1	0.897	14.134	1.694e-2	8.536e-2	-1.198	11.651	1.272e-6	1.857e-7
FN1656	AAL93771.1| SSU ribosomal protein S18P												
FN1657	AAL93772.1| SSU ribosomal protein S6P	-0.914	14.576	6.547e-2	1.308e-1	-2.064	13.610	1.355e-4	2.341e-4	-1.602	13.685	6.451e-5	5.802e-5
FN1658	AAL93773.1| Prolyl-tRNA synthetase	-1.402	14.043	1.551e-3	8.967e-4	-0.854	14.776	4.036e-4	9.329e-4	0.034	15.275	1.224e-1	7.062e-1
FN1660	AAL93775.1| ATP-dependent DNA helicase recG					-0.162	4.806						
FN1661	AAL93776.1| Hypothetical cytosolic protein	-0.541	10.636	5.53e-2	1.03e-1	-1.264	10.098	4.791e-3	1.971e-2	-0.035	10.939	1.472e-1	8.735e-1
FN1662	AAL93777.1| Hypothetical protein	-0.791	9.927	1.872e-2	2.488e-2	-0.007	10.895	1.503e-1	9.397e-1	-0.570	9.943	6.345e-3	2.424e-2
FN1663	AAL93778.1| Hypothetical protein	-0.412	6.752			-0.316	7.033	5.836e-2	3.294e-1	0.687	7.648	8.592e-6	2.604e-6
FN1666	AAL93781.1| Hypothetical protein												
FN1667	AAL93782.1| dTDP-glucose 4,6-dehydratase												
FN1668	AAL93783.1| Cholinephosphate cytidylyltransferase												

ORF	*FnPgSg vs FnPg* Ratio	Sum	*q*-Val	*p*-Val	*FnSg vs FnPg* Ratio	Sum	*q*-Val	*p*-Val	*FnPgSg vs FnSg* Ratio	Sum	*q*-Val	*p*-Val
FN1644	0.978	19.726	7.507e-3	4.746e-3	0.352	19.284	1.219e-1	1.231e-1	0.626	20.263	2.405e-5	1.199e-5
FN1645	1.949	17.537	3.325e-3	1.438e-3	-1.309	14.463	1.333e-1	1.471e-1	3.258	16.412	5.771e-5	4.215e-5
FN1646	0.215	15.106	1.93e-1	5.663e-1	-0.808	14.268	1.432e-1	1.714e-1	1.022	14.483	2.781e-3	6.99e-3
FN1647	0.589	17.336	1.227e-1	3.002e-1	-1.585	15.347	1.509e-1	1.925e-1	2.174	15.936	1.044e-3	2.05e-3
FN1652	0.828	12.259	2.003e-5	6.731e-7	0.383	11.998	3.084e-2	9.571e-3	0.445	12.827	1.013e-3	1.971e-3
FN1654	-0.612	11.064	1.319e-1	3.35e-1	1.045	12.905	6.716e-2	3.832e-2	-1.656	12.293	2.333e-5	1.151e-5
FN1655	-1.076	11.732	4.52e-2	6.843e-2	1.019	14.012	8.593e-2	6.28e-2	-2.095	12.936	7.488e-3	2.483e-2
FN1656												
FN1657	-0.687	12.974	1.618e-1	4.438e-1	-1.150	12.696	1.914e-1	2.915e-1	0.463	12.009	4.037e-3	1.128e-2
FN1658	1.436	14.077	1.326e-3	3.795e-4	0.548	13.374	1.277e-2	2.44e-3	0.888	14.810	1.659e-4	1.699e-4
FN1660												
FN1661	0.507	10.602	6.373e-3	3.615e-3	-0.723	9.557	1.101e-2	1.97e-3	1.229	10.064	4.444e-4	6.38e-4
FN1662	0.220	9.356	1.743e-1	4.909e-1	0.783	10.104	4.415e-2	1.8e-2	-0.563	10.324	6.141e-3	1.949e-2
FN1663	1.099	7.439			0.096	6.620			1.003	7.720	5.653e-3	1.756e-2
FN1666												
FN1667					-0.547	3.716						
FN1668					-0.829	3.999						

Log$_2$ Ratios

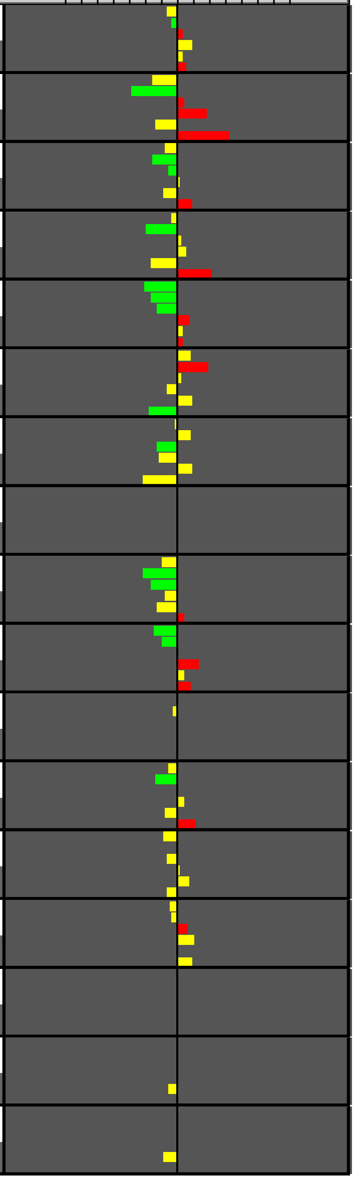

Show detected proteins only
Show all proteins
Filter by category:
GO: amino acid transport

Proteins found: 1424

Enter (or paste) list of ORFs

Find ORFs

Test	Cutoff
q-Value	.005
p-Value	

Dot Plots
Dot Plots

	Signif	Direction	Applies To
red	yes	+	ratios, bars
yellow	no	n/a	bars
green	yes	–	ratios, bars
pink	yes	+	*p*- , *q*-Values
light green	yes	–	*p*- , *q*-Values

ORF	Description	FnPg vs Fn Ratio	FnPg vs Fn Sum	FnPg vs Fn *q*-Val	FnPg vs Fn *p*-Val	FnSg vs Fn Ratio	FnSg vs Fn Sum	FnSg vs Fn *q*-Val	FnSg vs Fn *p*-Val	FnPgSg vs Fn Ratio	FnPgSg vs Fn Sum	FnPgSg vs Fn *q*-Val	FnPgSg vs Fn *p*-Val	FnPgSg vs FnPg Ratio	FnPgSg vs FnPg Sum	FnPgSg vs FnPg *q*-Val	FnPgSg vs FnPg *p*-Val	FnSg vs FnPg Ratio	FnSg vs FnPg Sum	FnSg vs FnPg *q*-Val	FnSg vs FnPg *p*-Val	FnPgSg vs FnSg Ratio	FnPgSg vs FnSg Sum	FnPgSg vs FnSg *q*-Val	FnPgSg vs FnSg *p*-Val
FN1670	AAL93785.1| Choline kinase	-1.175	11.013	1.198e-3	6.347e-4	-1.179	11.194	6.02e-5	7.896e-5	-1.431	10.553	5.101e-5	4.216e-5	-0.256	9.582	8.4e-2	1.675e-1	-0.004	10.019	4.282e-1	9.776e-1	-0.252	9.763	5.485e-2	2.474e-1
FN1671	AAL93786.1| hypothetical exported protein																								
FN1676	AAL93791.1| Transposase																								
FN1679	AAL93794.1| LPS biosynthesis protein WbpG	0.384	17.222	8.534e-2	1.851e-1	0.683	17.706	1.196e-4	1.972e-4	0.061	16.695	9.283e-2	5.17e-1	-0.323	17.283	1.052e-1	2.385e-1	0.299	18.089	1.499e-1	1.9e-1	-0.622	17.766	2.498e-4	3.005e-4
FN1683	AAL93798.1| Acetyltransferase	-1.996	11.806	1.071e-4	1.503e-5	-1.444	12.543	3.667e-5	3.829e-5	-1.101	12.498	1.248e-3	3.237e-3	0.896	10.706	4.808e-2	7.497e-2	0.552	10.547	8.934e-2	6.837e-2	0.343	11.442	7.21e-2	3.357e-1
FN1684	AAL93799.1| N-acetylneuraminate synthase	0.315	16.924	1.126e-1	2.633e-1	-0.478	16.316	1.065e-2	5.019e-2	0.149	16.554	8.54e-3	3.426e-2	-0.166	17.072	1.777e-1	5.042e-1	-0.793	16.631	7.831e-2	5.166e-2	0.626	16.464	5.469e-3	1.683e-2
FN1685	AAL93800.1| dTDP-4-dehydrorhamnose reductase	-0.127	12.373	2.538e-1	7.584e-1	-0.582	12.102	5.844e-3	2.477e-2	-0.397	11.898	1.175e-2	4.947e-2	-0.271	11.975	1.921e-1	5.627e-1	-0.455	11.975	2.275e-1	3.784e-1	0.185	11.704	1.554e-2	5.879e-2
FN1686	AAL93801.1| Spore coat polysaccharide biosynthesis protein spsF	0.226	15.857	2.062e-1	5.79e-1	0.013	15.829	1.43e-1	8.869e-1	0.065	15.493	1.181e-1	6.782e-1	-0.161	15.922	2.246e-1	6.942e-1	-0.213	16.055	3.157e-1	6.022e-1	0.052	15.894	1.496e-1	7.61e-1
FN1687	AAL93802.1| Gluconate 5-dehydrogenase	-0.251	15.505	7.081e-2	1.448e-1	-0.434	15.507	1.237e-2	5.971e-2	-0.132	15.420	7.712e-2	4.193e-1	0.119	15.373	1.23e-1	3.015e-1	-0.183	15.256	1.884e-1	2.837e-1	0.302	15.375	3.05e-2	1.269e-1
FN1688	AAL93803.1| Oxidoreductase	1.263	13.004	9.311e-2	2.07e-1	-0.403	11.523	5.263e-3	2.194e-2	0.776	12.314	4.389e-5	3.388e-5	-0.486	13.781	1.738e-1	4.888e-1	-1.666	12.786	1.371e-1	1.559e-1	1.180	12.300	2.947e-4	3.771e-4
FN1689	AAL93804.1| UDP-N-acetylglucosamine 4,6-dehydratase	-0.982	17.502	4.804e-5	4.446e-6	-1.273	17.395	6.858e-6	3.251e-6	-0.178	18.102	1.501e-3	4.217e-3	0.804	17.324	1.119e-3	2.917e-4	-0.291	16.414	5.993e-2	3.134e-2	1.095	17.218	1.834e-4	1.941e-4
FN1690	AAL93805.1| Hypothetical protein	-0.698	11.765	3.028e-2	4.573e-2	-0.929	11.719	4.889e-4	1.191e-3	-1.672	10.587	3.332e-5	2.057e-5	-0.975	10.093	4.943e-2	7.816e-2	-0.231	11.021	2.826e-1	5.143e-1	-0.743	10.046	5.163e-3	1.566e-2
FN1692	AAL93807.1| Glycosyl transferase	-1.382	11.280	6.028e-4	2.042e-4	-0.777	12.069	4.528e-4	1.079e-3	-0.883	11.575	7.625e-3	3e-2	0.499	10.397	1.218e-1	2.969e-1	0.605	10.687	1.438e-3	6.615e-5	-0.106	11.186	1.502e-1	7.645e-1
FN1693	AAL93808.1| Hypothetical protein	-0.295	8.291	1.261e-1	3.04e-1	-0.842	7.929	1.27e-3	3.976e-3	-0.112	8.271	1.084e-1	6.151e-1	0.183	8.180	1.997e-1	5.95e-1	-0.547	7.634	1.491e-1	1.877e-1	0.730	7.817	1.56e-2	5.904e-2
FN1694	AAL93809.1| UDP-N-acetyl-D-quinovosamine 4-epimerase	-1.263	12.735	6.092e-3	5.943e-3	-1.700	12.483	1.062e-5	6.429e-6	-0.393	13.401	3.491e-5	2.253e-5	0.870	12.342	2.391e-2	2.733e-2	-0.437	11.220	1.945e-1	3.001e-1	1.307	12.090	2.917e-5	1.558e-5
FN1695	AAL93810.1| Probable quinovosaminephosphotransferae	-1.327	8.752	3.036e-3	2.28e-3	-0.820	9.444	1.831e-3	6.132e-3	0.292	10.167	2.494e-2	1.159e-1	1.619	9.044	2.539e-3	9.623e-4	0.507	8.117	1.551e-1	2.027e-1	1.111	9.736	1.35e-3	2.833e-3
FN1696	AAL93811.1| UDP-N-acetylglucosamine 4,6-dehydratase	-0.164	14.848	1.569e-1	4.028e-1	0.243	15.439	1.068e-2	5.038e-2	-0.144	14.664	1.379e-2	5.92e-2	0.020	14.703	2.774e-1	9.202e-1	0.407	15.275	9.183e-2	7.259e-2	-0.387	15.295	3.749e-3	1.021e-2

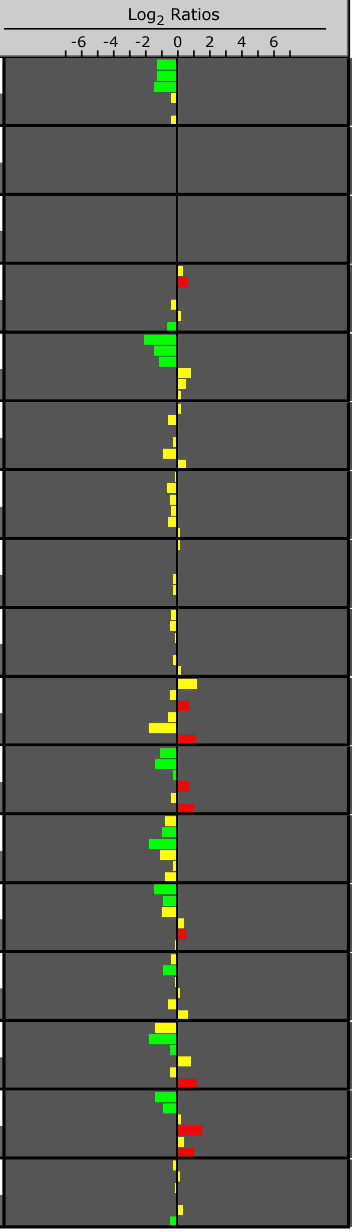

Show detected proteins only
Show all proteins
Filter by category:
GO: amino acid transport

Proteins found:
1424

Enter (or paste) list of ORFs

Find ORFs

Test	Cutoff
q-Value	.005
p-Value	

Dot Plots
Dot Plots

	Signif	Direction	Applies To
(red)	yes	+	ratios, bars
(yellow)	no	n/a	bars
(green)	yes	–	ratios, bars
(pink)	yes	+	*p*- , *q*-Values
(light green)	yes	–	

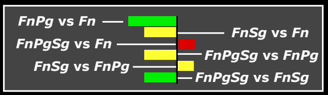

ORF	Protein	FnPg vs Fn				FnSg vs Fn				FnPgSg vs Fn				FnPgSg vs FnPg				FnSg vs FnPg				FnPgSg vs FnSg			
		Ratio	Sum	*q*-Val	*p*-Val	Ratio	Sum	*q*-Val	*p*-Val	Ratio	Sum	*q*-Val	*p*-Val	Ratio	Sum	*q*-Val	*p*-Val	Ratio	Sum	*q*-Val	*p*-Val	Ratio	Sum	*q*-Val	*p*-Val
FN1697	AAL93812.1| Hypothetical protein	-1.012	14.080	1.832e-2	2.416e-2	-0.736	14.540	1.497e-3	4.847e-3	-1.167	13.721	6.157e-4	1.301e-3	-0.155	12.913	2.349e-1	7.36e-1	0.276	13.528	2.76e-1	4.976e-1	-0.430	13.373	4.397e-3	1.269e-2
FN1698	AAL93813.1| dTDP-4-dehydrorhamnose reductase	0.660	13.812	6.211e-2	1.22e-1	1.236	14.573	1.44e-4	2.542e-4	-0.074	12.875	1.006e-1	5.662e-1	-0.733	13.739	5.955e-2	1.036e-1	0.576	15.233	8.787e-2	6.597e-2	-1.310	14.499	5.128e-5	3.6e-5
FN1700	AAL93815.1| Hypothetical protein					-1.688	5.619																		
FN1701	AAL93816.1| ABC transporter ATP-binding protein	-0.897	11.336	3.078e-3	2.325e-3	-0.217	12.200	1.37e-2	6.722e-2	-1.255	10.774	1.84e-5	8.057e-6	-0.358	10.081	5.329e-2	8.782e-2	0.680	11.303	1.601e-2	3.37e-3	-1.038	10.946	1.166e-3	2.371e-3
FN1703	AAL93818.1| ADP-L-glycero-D-manno-heptose-6-epimerase	-0.139	14.496	2.479e-1	7.349e-1	-0.011	14.808	1.465e-1	9.123e-1	-0.918	13.513	3.033e-5	1.73e-5	-0.779	13.578	1.007e-1	2.219e-1	0.128	14.669	3.682e-1	7.608e-1	-0.907	13.890	1.637e-3	3.608e-3
FN1704	AAL93819.1| Serine protease	-1.213	7.629	9.445e-3	1.06e-2	-0.812	8.215	4.075e-5	4.509e-5	-1.512	7.127			-0.299	6.117			0.401	7.002	1.814e-1	2.657e-1	-0.700	6.703		
FN1707	AAL93822.1| Aldose 1-epimerase																								
FN1708	AAL93823.1| Polyribonucleotide nucleotidyltransferase	-0.861	18.285	2.669e-5	1.839e-6	-0.649	18.681	3.79e-7	4.189e-8	-0.504	18.437	5.877e-6	1.441e-6	0.356	17.781	5.334e-4	9.817e-5	0.212	17.821	1.129e-2	2.045e-3	0.145	18.177	1.074e-3	2.131e-3
FN1711	AAL93826.1| Methyltransferase									-2.157	5.706	2.347e-6	4.111e-7												
FN1713	AAL93828.1| tRNA (Uracil-5-) -methyltransferase	0.483	6.436			1.188	7.325											0.705	7.808						
FN1715	AAL93830.1| ATPase																	1.695	5.050						
FN1716	AAL93831.1| Hypothetical protein																								
FN1717	AAL93832.1| NAD-dependent DNA ligase	-2.038	10.431	1.683e-4	2.964e-5	-2.513	10.140	1.731e-5	1.326e-5	-2.442	9.822	2.502e-4	3.989e-4	-0.404	7.988	1.247e-1	3.078e-1	-0.475	8.102	1.956e-1	3.031e-1	0.071	7.697	1.593e-1	8.197e-1
FN1718	AAL93833.1| Protein translocase subunit secA	-1.083	17.982	2.449e-4	5.252e-5	-1.084	18.165	3.61e-4	8.134e-4	-0.953	17.908	2.743e-4	4.488e-4	0.130	17.029	1.095e-1	2.543e-1	-0.001	17.082	4.314e-1	9.907e-1	0.131	17.212	1.109e-2	3.973e-2
FN1719	AAL93834.1| Hypothetical protein	-0.539	16.963	1.975e-3	1.266e-3	-0.008	17.678	1.521e-1	9.536e-1	-0.715	16.583	3.33e-5	2.054e-5	-0.176	16.248	7.711e-2	1.479e-1	0.530	17.139	6.164e-2	3.289e-2	-0.707	16.963	6.007e-3	1.898e-2
FN1722	AAL93837.1| Glucose inhibited division protein B					-0.891	4.856	9.382e-3	4.335e-2																
FN1723	AAL93838.1| Glucose inhibited division protein A	-1.008	12.769	4.075e-3	3.41e-3	-0.775	13.186	1.12e-3	3.41e-3	-0.389	13.184	8.235e-3	3.282e-2	0.619	12.380	8.064e-3	5.4e-3	0.233	12.178	1.456e-1	1.778e-1	0.386	12.797	1.054e-2	3.733e-2

Log$_2$ Ratios

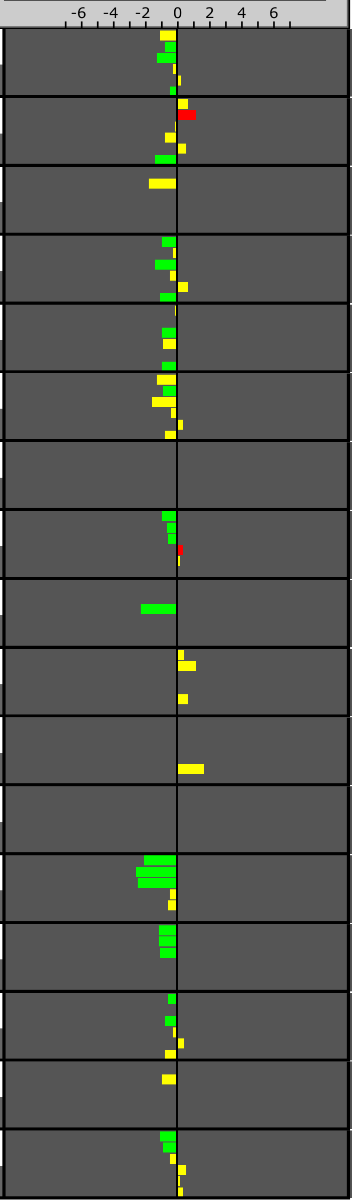

Show detected proteins only
Show all proteins
Filter by category:
GO: amino acid transport

Proteins found: 1424

Enter (or paste) list of ORFs

Find ORFs

Test	Cutoff
q-Value	.005
p-Value	

Dot Plots
Dot Plots

	Signif	Direction	Applies To
red	yes	+	ratios, bars
yellow	no	n/a	bars
green	yes	–	ratios, bars
pink	yes	+	*p*- , *q*-Values
light green	yes	–	*p*- , *q*-Values

FnPg vs Fn — FnSg vs Fn
FnPgSg vs Fn — FnPgSg vs FnPg
FnSg vs FnPg — FnPgSg vs FnSg

ORF	Description	*FnPg vs Fn*				*FnSg vs Fn*				*FnPgSg vs Fn*				*FnPgSg vs FnPg*				*FnSg vs FnPg*				*FnPgSg vs FnSg*			
		Ratio	Sum	*q*-Val	*p*-Val	Ratio	Sum	*q*-Val	*p*-Val	Ratio	Sum	*q*-Val	*p*-Val	Ratio	Sum	*q*-Val	*p*-Val	Ratio	Sum	*q*-Val	*p*-Val	Ratio	Sum	*q*-Val	*p*-Val
**FN1724**	AAL93839.1| Potassium uptake protein KtrA	0.576	8.057			1.009	8.673			-0.577	6.699			-1.154	7.479	1.061e-1	2.421e-1	0.432	9.250	2.942e-1	5.443e-1	-1.586	8.096	2.555e-2	1.04e-1
**FN1727**	AAL93842.1| Chloride channel protein																					-0.318	5.086		
**FN1728**	AAL93843.1| Pyrrolidone-carboxylate peptidase	-0.386	8.629	1.038e-3	4.983e-4	-1.320	7.878	3.995e-6	1.353e-6	-1.893	6.917	2.063e-6	3.481e-7	-1.508	6.735	8.793e-5	6.524e-6	-0.935	7.493	6.035e-4	8.891e-6	-0.573	5.985	1.109e-3	2.227e-3
**FN1730**	AAL93845.1| Para-aminobenzoate synthase component I	-0.644	8.109	2.431e-2	3.463e-2	0.233	9.170	2.828e-3	1.042e-2	-1.332	7.217			-0.688	6.777			0.876	8.526	3.813e-2	1.386e-2	-1.565	7.838		
**FN1731**	AAL93846.1| Anthranilate synthase component II													0.965	7.239			-0.441	6.017	3.786e-2	1.37e-2	1.407	6.982		
**FN1732**	AAL93847.1| Hypothetical protein	0.941	10.311	6.894e-2	1.399e-1	1.181	10.736	1.07e-3	3.228e-3	-0.305	8.862	6.336e-2	3.367e-1	-1.246	10.006	5.457e-2	9.121e-2	0.240	11.677	2.866e-1	5.244e-1	-1.486	10.431	6.47e-4	1.081e-3
**FN1733**	AAL93848.1| V-type sodium ATP synthase subunit D																	0.983	7.168						
**FN1734**	AAL93849.1| V-type sodium ATP synthase subunit B	0.411	13.885	1.689e-3	1.008e-3	0.735	14.394	8.88e-5	1.348e-4	0.282	13.552	2.984e-2	1.427e-1	-0.129	14.167	1.446e-1	3.821e-1	0.324	14.804	4.872e-3	5.482e-4	-0.453	14.675	6.476e-3	2.077e-2
**FN1735**	AAL93850.1| V-type sodium ATP synthase subunit A	0.738	12.791	8.608e-2	1.872e-1	1.703	13.940	3.393e-5	3.414e-5	-0.552	11.297	1.147e-3	2.875e-3	-1.290	12.238	5.271e-2	8.632e-2	0.965	14.678	5.033e-2	2.297e-2	-2.255	13.388	7.896e-5	6.329e-5
**FN1736**	AAL93851.1| V-type sodium ATP synthase subunit A	1.129	8.712	3.761e-2	6.075e-2	1.958	9.725	3.525e-5	3.611e-5	-0.692	6.687	3.585e-4	6.464e-4	-1.821	8.019	2.54e-2	2.981e-2	0.829	10.854	4.839e-2	2.131e-2	-2.650	9.033	1.827e-5	7.893e-6
**FN1737**	AAL93852.1| V-type sodium ATP synthase subunit G	-0.822	5.498	5.269e-2	9.624e-2	0.730	7.235	8.316e-3	3.755e-2									1.553	6.413	3.046e-3	2.633e-4				
**FN1738**	AAL93853.1| V-type sodium ATP synthase subunit C	1.946	11.018	3.79e-2	6.138e-2	1.818	11.075	9.609e-5	1.487e-4	-0.713	8.155	3.759e-3	1.291e-2	-2.659	10.305	3.368e-2	4.519e-2	-0.128	13.021	3.688e-1	7.629e-1	-2.531	10.361	1.929e-4	2.079e-4
**FN1739**	AAL93854.1| V-type sodium ATP synthase subunit E	0.306	5.733			-0.103	5.508			1.461	6.684			1.155	7.194	8.725e-7	7.771e-9	-0.410	5.814			1.565	6.969		
**FN1740**	AAL93855.1| V-type sodium ATP synthase subunit K																	-0.791	14.457						
**FN1741**	AAL93856.1| V-type sodium ATP synthase subunit I	0.670	9.257	8.139e-3	8.846e-3	0.833	9.603	2.926e-5	2.772e-5	-1.416	6.966	4.968e-5	4.052e-5	-2.087	7.840	2.797e-3	1.105e-3	0.162	10.274	1.494e-1	1.884e-1	-2.249	8.187	3.242e-7	2.611e-8
**FN1742**	AAL93857.1| V-type sodium ATP synthase subunit G																								
**FN1743**	AAL93858.1| Multidrug-efflux transporter 2 regulator																								

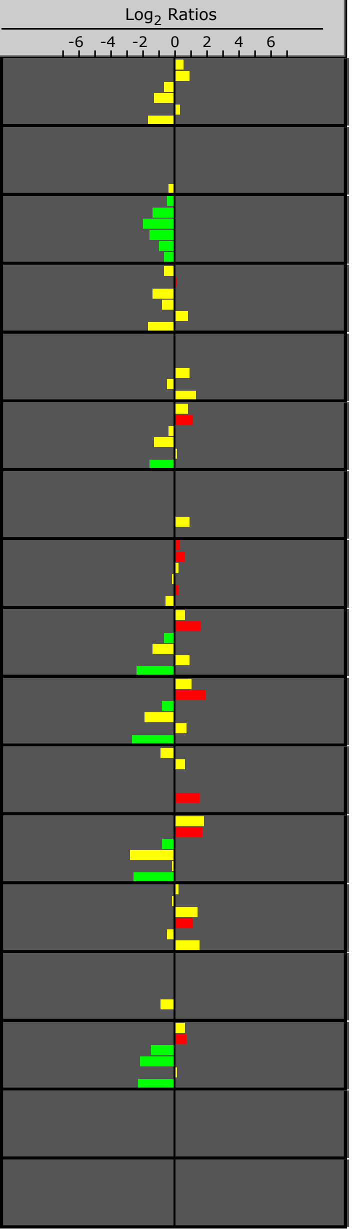

Show detected proteins only
Show all proteins
Filter by category:
GO: amino acid transport

Proteins found:
1424

Enter (or paste) list of ORFs

Find ORFs

Test	Cutoff
q-Value	.005
p-Value	

Dot Plots
Dot Plots

	Signif	Direction	Applies To
(red)	yes	+	ratios, bars
(yellow)	no	n/a	bars
(green)	yes	–	ratios, bars
(pink)	yes	+	*p*- , *q*-Values
(light green)	yes	–	*p*- , *q*-Values

FnPg vs Fn
FnSg vs Fn
FnPgSg vs Fn
FnPgSg vs FnPg
FnSg vs FnPg
FnPgSg vs FnSg

ORF	Protein	FnPg vs Fn				FnSg vs Fn				FnPgSg vs Fn				FnPgSg vs FnPg				FnSg vs FnPg				FnPgSg vs FnSg			
		Ratio	Sum	*q*-Val	*p*-Val	Ratio	Sum	*q*-Val	*p*-Val	Ratio	Sum	*q*-Val	*p*-Val	Ratio	Sum	*q*-Val	*p*-Val	Ratio	Sum	*q*-Val	*p*-Val	Ratio	Sum	*q*-Val	*p*-Val
FN1745	AAL93860.1| Cystathionine gamma-synthase	2.072	7.098			2.001	7.212	1.028e-5	6.096e-6	0.611	5.434	2.23e-2	1.021e-1	-1.461	7.709			-0.070	9.284			-1.390	7.823	8.096e-4	1.464e-3
FN1746	AAL93861.1| Cystathionine beta-lyase																	0.768	6.567						
FN1752	AAL93867.1| Regulatory protein TENI																								
FN1754	AAL93869.1| Thiazole biosynthesis protein thiG																								
FN1762	AAL93875.1| Protein yaaA																								
FN1763	AAL93876.1| Hypothetical cytosolic protein	1.959	10.009	1.247e-2	1.477e-2	2.008	10.242	9.797e-6	5.649e-6	-0.243	7.602			-2.203	9.765			0.049	12.201	3.905e-1	8.363e-1	-2.251	9.999		
FN1764	AAL93877.1| Enolase	0.187	23.848	2.596e-1	7.817e-1	0.963	24.808	9.803e-6	5.654e-6	-0.224	23.234	2.337e-4	3.655e-4	-0.410	23.624	1.963e-1	5.803e-1	0.776	24.995	1.441e-1	1.735e-1	-1.186	24.584	2.024e-7	1.149e-8
FN1765	AAL93878.1| Pyruvate kinase	-1.291	19.164	6.487e-3	6.485e-3	-0.922	19.718	1.549e-4	2.812e-4	-0.880	19.371	5.355e-5	4.511e-5	0.411	18.283	1.14e-1	2.698e-1	0.370	18.426	2.014e-1	3.161e-1	0.041	18.838	1.158e-1	5.666e-1
FN1780	AAL93879.1| Hypothetical protein	-1.339	8.610	5.36e-3	4.992e-3	-1.274	8.859	6.736e-5	9.32e-5	-0.124	9.621	7.404e-2	4.005e-1	1.215	8.485	1.004e-2	7.598e-3	0.065	7.520	4.014e-1	8.754e-1	1.150	8.735	1.46e-3	3.122e-3
FN1781	AAL93880.1| LytB protein	-0.691	18.542	1.231e-2	1.454e-2	-0.665	18.753	7.157e-5	1.022e-4	-0.570	18.459	1.946e-4	2.787e-4	0.121	17.972	1.867e-1	5.402e-1	0.027	18.062	4.073e-1	8.973e-1	0.095	18.183	5.114e-2	2.286e-1
FN1783	AAL93882.1| Ethanolamine utilization protein eutJ																	-0.552	6.552						
FN1784	AAL93883.1| unknown	-1.541	8.185			-0.466	9.445	1.392e-2	6.853e-2	-2.082	7.441			-0.541	6.103			1.075	7.903			-1.616	7.363		
FN1785	AAL93884.1| Hypothetical protein	-1.171	8.572			-1.124	8.804			0.622	10.161	4.805e-2	2.473e-1	1.793	9.194			0.047	7.632			1.746	9.425		
FN1786	AAL93885.1| ADP-heptose synthase	-1.607	10.856	5.276e-3	4.89e-3	-0.867	11.781	8.295e-6	4.353e-6	-0.317	11.942	6.397e-4	1.366e-3	1.290	10.539	1.576e-2	1.535e-2	0.740	10.174	1.092e-1	1.002e-1	0.549	11.463	6.926e-4	1.182e-3
FN1787	AAL93886.1| Tetratricopeptide repeat family protein	-2.139	10.221	8.616e-4	3.704e-4	-2.366	10.179			-1.042	11.114	1.322e-3	3.521e-3	1.097	9.179	1.653e-2	1.635e-2	-0.226	8.040			1.323	9.137		
FN1788	AAL93887.1| 2C-methyl-D-erythritol 2,4-cyclodiphosphate synthase	0.089	9.167	2.6e-1	7.834e-1	-0.505	8.757	3.007e-3	1.126e-2	0.133	9.007	7.27e-2	3.924e-1	0.044	9.299	2.706e-1	8.895e-1	-0.594	8.846	1.461e-1	1.792e-1	0.638	8.890	6.02e-3	1.903e-2
FN1790	AAL93889.1| Cob(I)alamin adenosyltransferase	3.150	7.103			3.530	7.668			1.328	5.077			-1.822	8.430	1.002e-1	2.2e-1	0.381	10.817	3.163e-1	6.041e-1	-2.203	8.995	1.027e-2	3.619e-2

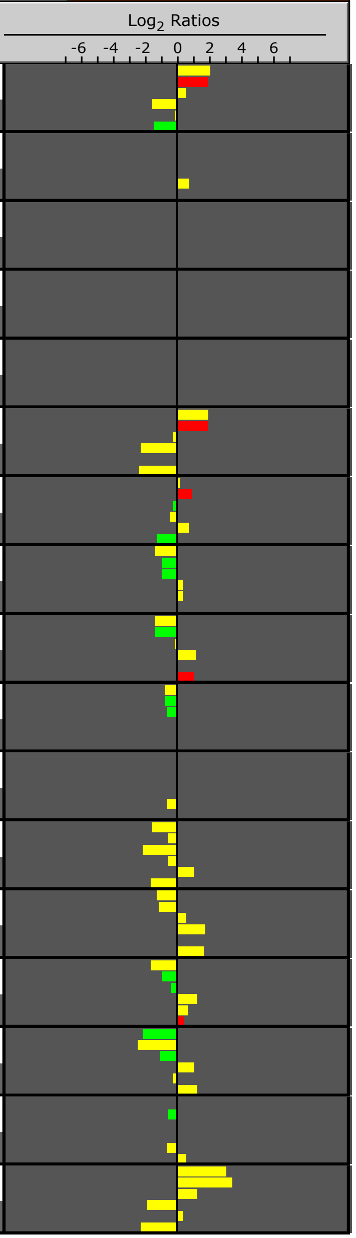

○ Show detected proteins only
○ Show all proteins
☐ Filter by category:
GO: amino acid transport

Proteins found:
1424

Enter (or paste) list of ORFs

Find ORFs

Test	Cutoff
q-Value	.005
p-Value	

Dot Plots
Dot Plots

	Signif	Direction	Applies To
red	yes	+	ratios, bars
yellow	no	n/a	bars
green	yes	–	ratios, bars
pink	yes	+	*p*- , *q*-Values
light green	yes	–	*p*- , *q*-Values

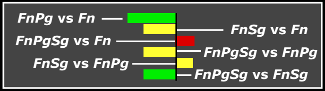

ORF	Protein	*FnPg vs Fn* Ratio	Sum	*q*-Val	*p*-Val	*FnSg vs Fn* Ratio	Sum	*q*-Val	*p*-Val	*FnPgSg vs Fn* Ratio	Sum	*q*-Val	*p*-Val	*FnPgSg vs FnPg* Ratio	Sum	*q*-Val	*p*-Val	*FnSg vs FnPg* Ratio	Sum	*q*-Val	*p*-Val	*FnPgSg vs FnSg* Ratio	Sum	*q*-Val	*p*-Val	Log$_2$ Ratios (-6 -4 -2 0 2 4 6) 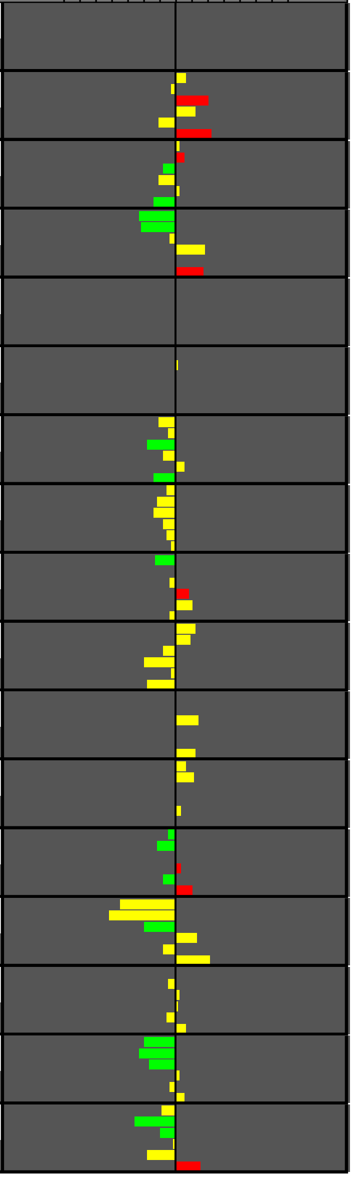
FN1791	AAL93890.1| Mutator MutT protein																									
FN1792	AAL93891.1| Hypothetical protein	0.744	22.973	7.001e-2	1.427e-1	-0.244	22.170	3.243e-2	1.745e-1	2.062	24.088	8.106e-9	5.46e-11	1.318	25.036	7.919e-3	5.222e-3	-0.988	22.914	1.031e-1	8.992e-2	2.306	24.232	4.189e-5	2.723e-5	
FN1793	AAL93892.1| Phosphoenolpyruvate-protein phosphotransferase	0.340	14.830	2.264e-1	6.524e-1	0.619	15.293	1.327e-4	2.272e-4	-0.653	13.634	8.342e-5	8.319e-5	-0.993	14.177	1.286e-1	3.223e-1	0.279	15.633	3.318e-1	6.484e-1	-1.272	14.641	7.563e-5	5.977e-5	
FN1794	AAL93893.1| Phosphocarrier protein HPr	-2.237	12.907	4.652e-4	1.373e-4	-2.139	13.189	5.94e-5	7.745e-5	-0.325	14.614	2.967e-2	1.418e-1	1.911	12.582	1.253e-2	1.062e-2	0.097	10.952	3.806e-1	8.022e-1	1.814	12.863	2.338e-3	5.629e-3	
FN1795	AAL93894.1| Hypothetical protein																									
FN1796	AAL93895.1| unknown					0.194	5.806																			
FN1797	AAL93896.1| Spermidine/putrescine transport ATP-binding protein potA	-0.965	12.188	1.807e-2	2.372e-2	-0.397	12.940	5.661e-3	2.387e-2	-1.666	11.284	1.595e-5	6.328e-6	-0.700	10.522	9.57e-2	2.044e-1	0.568	11.975	1.291e-1	1.38e-1	-1.268	11.275	1.183e-3	2.414e-3	
FN1798	AAL93897.1| Spermidine/putrescine transport system permease protein potB	-0.529	7.173			-1.055	6.832			-1.273	6.225			-0.744	5.900			-0.526	6.303			-0.219	5.558	9.413e-3	3.258e-2	
FN1800	AAL93899.1| Peptidyl-prolyl cis-trans isomerase	-1.164	14.275	1.624e-3	9.55e-4	-0.041	15.581	1.268e-1	7.719e-1	-0.311	14.924	9.349e-3	3.815e-2	0.853	13.964	4.957e-3	2.494e-3	1.122	14.418	2.047e-2	4.821e-3	-0.269	15.271	4.052e-2	1.758e-1	
FN1801	AAL93900.1| Sodium/glutamate symport carrier protein	1.297	8.419			1.049	8.356			-0.652	6.267			-1.949	7.767			-0.248	9.652			-1.701	7.704			
FN1803	AAL93902.1| Transcriptional regulator, TetR family					0.128	6.488			1.473	7.445											1.345	7.962			
FN1804	AAL93903.1| Aminoacyl-histidine dipeptidase	0.733	8.186			1.168	8.806	7.785e-3	3.476e-2									0.435	9.539							
FN1807	AAL93906.1| Hypothetical protein	-0.405	17.120	4.755e-4	1.426e-4	-1.080	16.630	3.196e-5	3.133e-5	0.029	17.351	5.788e-2	3.049e-1	0.435	17.149	1.233e-3	3.388e-4	-0.674	16.225	3.372e-3	3.296e-4	1.109	16.659	1.354e-4	1.286e-4	
FN1808	AAL93907.1| Hypothetical protein	-3.354	10.524			-4.066	9.997			-1.913	11.762	3.078e-4	5.231e-4	1.442	8.612			-0.712	6.642			2.154	8.084			
FN1809	AAL93908.1| Iron/zinc/copper-binding protein	0.046	8.944	2.993e-1	9.525e-1	-0.448	8.634	4.384e-2	2.418e-1	0.257	8.951	5.993e-2	3.168e-1	0.210	9.201	2.412e-1	7.621e-1	-0.495	8.680	3.109e-1	5.889e-1	0.705	8.891	8.761e-3	2.993e-2	
FN1811	AAL93910.1| Manganese transport system ATP-binding protein mntA	-1.950	10.824	6.988e-4	2.593e-4	-2.238	10.721	4.003e-5	4.383e-5	-1.648	10.922	3.729e-5	2.584e-5	0.301	9.176	1.695e-1	4.725e-1	-0.288	8.771	2.86e-1	5.229e-1	0.589	9.072	2.171e-2	8.683e-2	
FN1812	AAL93911.1| Manganese-binding protein	-0.764	14.623	7.852e-2	1.657e-1	-2.463	13.109	6.056e-6	2.629e-6	-0.884	14.299	1.088e-4	1.234e-4	-0.120	13.739	2.616e-1	8.492e-1	-1.699	12.344	1.422e-1	1.687e-1	1.579	12.225	6.106e-4	9.948e-4	

Show detected proteins only
Show all proteins
Filter by category:
GO: amino acid transport

Proteins found: 1424

Enter (or paste) list of ORFs

Find ORFs

Test	Cutoff
q-Value	.005
p-Value	

Dot Plots
Dot Plots

	Signif	Direction	Applies To
red	yes	+	ratios, bars
yellow	no	n/a	bars
green	yes	–	ratios, bars
pink	yes	+	*p*- , *q*-Values
light green	yes	–	*p*- , *q*-Values

FnPg vs Fn
FnPgSg vs Fn
FnSg vs FnPg
FnSg vs Fn
FnPgSg vs FnPg
FnPgSg vs FnSg

ORF	Protein	*FnPg vs Fn* Ratio	Sum	*q*-Val	*p*-Val	*FnSg vs Fn* Ratio	Sum	*q*-Val	*p*-Val	*FnPgSg vs Fn* Ratio	Sum	*q*-Val	*p*-Val	*FnPgSg vs FnPg* Ratio	Sum	*q*-Val	*p*-Val	*FnSg vs FnPg* Ratio	Sum	*q*-Val	*p*-Val	*FnPgSg vs FnSg* Ratio	Sum	*q*-Val	*p*-Val	Log_2 Ratios
FN1813	AAL93912.1| Manganese-binding protein	-2.125	8.768			-2.467	8.610	4.626e-5	5.489e-5	-1.315	9.374	1.684e-4	2.261e-4	0.809	7.453			-0.343	6.485			1.152	7.295	7.008e-6	1.596e-6	
FN1814	AAL93913.1| Hypothetical protein					-1.854	8.615			-1.431	8.650	2.654e-3	8.491e-3									0.423	7.184			
FN1816	AAL93915.1| unknown	0.093	6.397	2.275e-1	6.566e-1	1.563	8.052			-0.306	5.795	3.338e-2	1.631e-1	-0.398	6.092	4.697e-2	7.241e-2	1.470	8.145			-1.868	7.746			
FN1817	AAL93916.1| Hemolysin	-0.378	5.575			-0.484	5.654			0.425	6.174			0.803	5.999	3.244e-2	4.268e-2	-0.105	5.275			0.908	6.078			
FN1819	AAL93918.1| Export ABC transporter																									
FN1820	AAL93919.1| Export ABC transporter																									
FN1822	AAL93921.1| Flavodoxin																									
FN1825	AAL93924.1| Hypothetical protein																									
FN1826	AAL93925.1| Protease	-1.242	11.001	8.325e-4	3.482e-4	-1.115	11.313	5.451e-4	1.375e-3	-0.410	11.630	5.195e-3	1.912e-2	0.832	10.592	3.215e-3	1.363e-3	0.127	10.071	1.876e-1	2.815e-1	0.705	10.903	1.813e-4	1.911e-4	
FN1827	AAL93926.1| Replicative DNA helicase	-0.457	11.493	5.739e-2	1.087e-1	0.562	12.697	3.596e-3	1.412e-2	-0.895	10.851	6.268e-3	2.389e-2	-0.438	10.598	7.193e-2	1.341e-1	1.020	12.239	6.817e-3	9.539e-4	-1.457	11.802	6.894e-4	1.175e-3	
FN1828	AAL93927.1| LSU ribosomal protein L9P	-0.343	13.069	1.914e-1	5.278e-1	-1.611	11.985	1.875e-4	3.539e-4	-1.285	11.923	1.042e-4	1.164e-4	-0.942	11.784	1.199e-1	2.902e-1	-1.269	11.642	1.606e-1	2.157e-1	0.327	10.700	1.534e-2	5.792e-2	
FN1830	AAL93929.1| DNA polymerase III subunits gamma and tau	0.319	10.276	1.633e-1	4.251e-1	0.831	10.973	7.067e-3	3.105e-2	-0.965	8.788	1.069e-4	1.205e-4	-1.284	9.311	4.641e-2	7.113e-2	0.512	11.292	1.423e-1	1.689e-1	-1.796	10.007	3.083e-3	7.944e-3	
FN1831	AAL93930.1| Nitrogen assimilation regulatory protein	0.505	12.687	8.971e-3	9.957e-3	0.765	13.132	3.158e-6	9.818e-7	-0.796	11.183	3.185e-3	1.06e-2	-1.301	11.892	9.341e-4	2.159e-4	0.260	13.637	5.632e-2	2.828e-2	-1.561	12.336	2.961e-4	3.795e-4	
FN1832	AAL93931.1| TonB protein																					-0.771	5.159			
FN1833	AAL93932.1| Biopolymer transport exbD protein													0.448	8.623											
FN1834	AAL93933.1| Biopolymer transport exbB protein	-1.228	10.793	3.924e-4	1.042e-4	-1.470	10.736	1.335e-5	9.22e-6	-0.833	10.984	2.285e-4	3.536e-4	0.395	9.960	3.835e-2	5.428e-2	-0.241	9.508	1.259e-1	1.314e-1	0.636	9.903	4.62e-3	1.356e-2	
FN1836	AAL93935.1| Tetratricopeptide repeat family protein	-1.370	12.374	6.756e-4	2.451e-4	-1.081	12.848	2.971e-5	2.831e-5	-0.525	13.016	6.48e-5	5.838e-5	0.846	11.849	6.353e-3	3.598e-3	0.289	11.477	1.511e-1	1.931e-1	0.556	12.323	9.94e-4	1.922e-3	

Log2 Ratios axis: -6 -4 -2 0 2 4 6

Show detected proteins only
Show all proteins
Filter by category:
GO: amino acid transport

Proteins found: 1424

Enter (or paste) list of ORFs

Find ORFs

Test	Cutoff
q-Value	.005
p-Value	

Dot Plots
Dot Plots

	Signif	Direction	Applies To
(red)	yes	+	ratios, bars
(yellow)	no	n/a	bars
(green)	yes	–	ratios, bars
(pink)	yes	+	*p*- , *q*-Values
(light green)	yes	–	*p*- , *q*-Values

FnPg vs Fn
FnPgSg vs Fn
FnSg vs FnPg
FnSg vs Fn
FnPgSg vs FnPg
FnPgSg vs FnSg

ORF	Protein	*FnPg vs Fn* Ratio	Sum	*q*-Val	*p*-Val	*FnSg vs Fn* Ratio	Sum	*q*-Val	*p*-Val	*FnPgSg vs Fn* Ratio	Sum	*q*-Val	*p*-Val	*FnPgSg vs FnPg* Ratio	Sum	*q*-Val	*p*-Val	*FnSg vs FnPg* Ratio	Sum	*q*-Val	*p*-Val	*FnPgSg vs FnSg* Ratio	Sum	*q*-Val	*p*-Val
FN1838	AAL93937.1| Glycerol uptake facilitator protein													-1.439	8.561										
FN1839	AAL93938.1| Glycerol kinase	1.968	16.740	3.183e-2	4.881e-2	2.490	17.447	1.467e-3	4.729e-3	1.061	15.629	1.862e-3	5.55e-3	-0.907	17.801	7.273e-2	1.362e-1	0.522	19.415	1.45e-1	1.761e-1	-1.429	18.508	3.099e-3	7.996e-3
FN1840	AAL93939.1| Dihydroxyacetone kinase	1.484	11.328	4.759e-3	4.259e-3	1.229	11.257	8.826e-6	4.817e-6	1.679	11.320	5.464e-7	5.367e-8	0.195	13.008	8.779e-2	1.789e-1	-0.255	12.741	1.327e-1	1.457e-1	0.451	12.937	7.111e-7	7.961e-8
FN1841	AAL93940.1| Dihydroxyacetone kinase	-0.413	8.863	1.55e-1	3.963e-1	-1.358	8.103	9.292e-3	4.284e-2	-0.747	8.326	2.398e-2	1.108e-1	-0.334	8.117	1.795e-1	5.11e-1	-0.945	7.690	1.431e-1	1.711e-1	0.611	7.356	1.443e-2	5.393e-2
FN1842	AAL93941.1| Dihydroxyacetone kinase phosphotransfer protein	-0.169	9.365	2.024e-1	5.658e-1	0.002	9.721	1.576e-1	9.943e-1	0.943	10.274	6.731e-4	1.459e-3	1.112	10.308	5.622e-3	2.987e-3	0.171	9.552	3.228e-1	6.224e-1	0.941	10.664	1.201e-3	2.46e-3
FN1843	AAL93942.1| Surface antigen																								
FN1844	AAL93943.1| Ketoacyl reductase hetN	-1.392	6.035			-0.425	7.186											0.966	5.794						
FN1847	AAL93946.1| DTDP-4-dehydrorhamnose 3,5-epimerase	0.729	7.165	1.321e-1	3.225e-1	1.144	7.764	3.63e-3	1.429e-2									0.414	8.493	2.27e-1	3.773e-1				
FN1848	AAL93947.1| Metal dependent hydrolase													0.138	4.781										
FN1849	AAL93948.1| Coenzyme F390 synthetase	-0.266	9.436			0.102	9.988	1.094e-1	6.529e-1	-2.749	6.749			-2.483	6.687			0.368	9.722			-2.851	7.239		
FN1850	AAL93949.1| 3-oxoacyl-[acyl-carrier-protein] synthase III	2.654	8.516			2.599	8.646	5.51e-3	2.313e-2									-0.055	11.300						
FN1851	AAL93950.1| Ribonuclease PH	0.490	12.612	1.857e-1	5.072e-1	1.168	13.475	6.998e-5	9.877e-5	-0.557	11.361	7.598e-5	7.293e-5	-1.047	12.055	1.144e-1	2.709e-1	0.678	13.965	1.604e-1	2.152e-1	-1.725	12.918	1.134e-4	1.016e-4
FN1852	AAL93951.1| unknown	-0.404	10.445	8.45e-2	1.828e-1	-0.796	10.238	1.207e-2	5.8e-2	0.067	10.712	6.879e-2	3.689e-1	0.471	10.512	6.999e-2	1.291e-1	-0.392	9.834	2.384e-1	4.069e-1	0.863	10.305	1.141e-2	4.112e-2
FN1853	AAL93952.1| Methylaspartate mutase	0.477	8.050	1.379e-1	3.407e-1	-0.311	7.446	1.997e-2	1.022e-1	-1.173	6.196	1.402e-3	3.832e-3	-1.649	6.877	4.563e-2	6.937e-2	-0.788	7.923	1.487e-1	1.865e-1	-0.862	6.274	6.623e-4	1.114e-3
FN1854	AAL93953.1| Methylaspartate mutase	-0.562	11.028	1.92e-2	2.572e-2	-0.685	11.090	4.229e-3	1.713e-2	-0.915	10.472	6.436e-7	6.825e-8	-0.352	10.114	8.161e-2	1.605e-1	-0.123	10.528	3.29e-1	6.402e-1	-0.229	10.175	7.205e-2	3.355e-1
FN1855	AAL93954.1| Methylaspartate mutase																	0.517	4.702						
FN1856	AAL93955.1| Butyrate-acetoacetate CoA-transferase subunit B	-0.155	23.372	4.956e-2	8.848e-2	0.079	23.790	1.052e-1	6.249e-1	-0.236	23.088	9.181e-3	3.733e-2	-0.081	23.136	1.603e-1	4.384e-1	0.234	23.635	1.666e-1	2.3e-1	-0.314	23.555	3.291e-2	1.384e-1

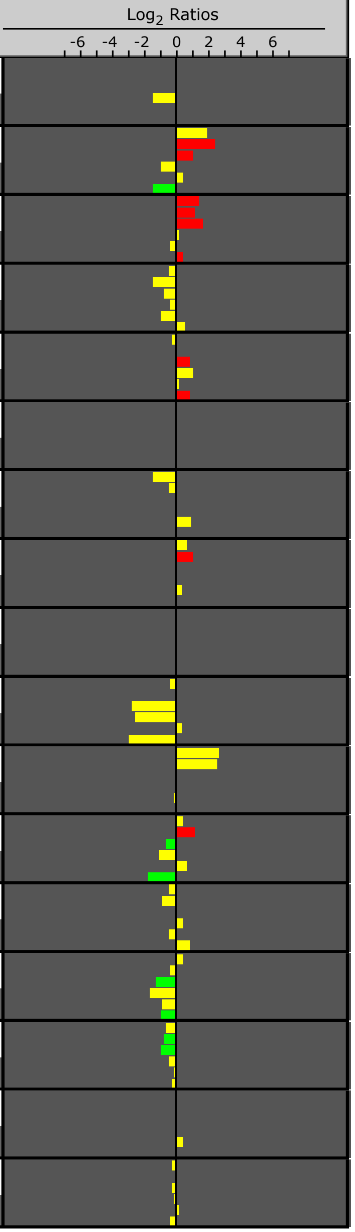

Show detected proteins only
Show all proteins
Filter by category:
GO: amino acid transport

Proteins found: 1424

Enter (or paste) list of ORFs
Find ORFs

Test	Cutoff
q-Value	.005
p-Value	

Dot Plots
Dot Plots

	Signif	Direction	Applies To
	yes	+	ratios, bars
	no	n/a	bars
	yes	–	ratios, bars
	yes	+	*p*- , *q*-Values
	yes	–	*p*- , *q*-Values

FnPg vs Fn
FnSg vs Fn
FnPgSg vs Fn
FnPgSg vs FnPg
FnSg vs FnPg
FnPgSg vs FnSg

	FnPg vs Fn				*FnSg vs Fn*				*FnPgSg vs Fn*				*FnPgSg vs FnPg*				*FnSg vs FnPg*				*FnPgSg vs FnSg*			
ORF	Ratio	Sum	*q*-Val	*p*-Val	Ratio	Sum	*q*-Val	*p*-Val	Ratio	Sum	*q*-Val	*p*-Val	Ratio	Sum	*q*-Val	*p*-Val	Ratio	Sum	*q*-Val	*p*-Val	Ratio	Sum	*q*-Val	*p*-Val
**FN1857**	0.801	19.791	1.135e-4	1.655e-5	0.978	20.153	6.443e-6	2.954e-6	0.190	18.976	2.879e-2	1.369e-1	-0.611	19.981	3.737e-3	1.711e-3	0.178	20.954	2.462e-2	6.517e-3	-0.789	20.343	1.503e-4	1.488e-4
	AAL93956.1| Acetoacetate:butyrate/acetate coenzyme A transferase																							
**FN1858**	-0.870	11.593							0.610	12.869	5.854e-3	2.203e-2	1.480	12.203										
	AAL93957.1| Short-chain fatty acids transporter																							
**FN1859**	-0.003	24.501	3.09e-1	9.971e-1	1.188	25.876	6.908e-3	3.021e-2	-1.875	22.425	5.322e-4	1.08e-3	-1.872	22.626	1.071e-1	2.46e-1	1.191	25.873	1.008e-1	8.631e-2	-3.063	24.001	3.561e-3	9.549e-3
	AAL93958.1| Major outer membrane protein																							
**FN1860**	1.392	10.825			-0.166	9.450	8.739e-2	5.089e-1	0.578	9.807	2.499e-2	1.162e-1	-0.814	11.403			-1.559	10.843			0.744	10.028	1.785e-2	6.91e-2
	AAL93959.1| NA+/H+ antiporter NHAC																							
**FN1862**	-0.052	15.804	2.805e-1	8.691e-1	-1.177	14.864	2.363e-5	2.073e-5	0.075	15.728	4.34e-2	2.207e-1	0.128	15.880	2.215e-1	6.821e-1	-1.125	14.812	9.372e-2	7.598e-2	1.252	14.940	9.526e-6	2.674e-6
	AAL93961.1| L-beta-lysine 5,6-aminomutase beta subunit																							
**FN1863**	1.287	17.930	1.008e-1	2.297e-1	-0.558	16.269	7.702e-4	2.175e-3	0.824	17.264	5.428e-4	1.107e-3	-0.463	18.754	1.856e-1	5.357e-1	-1.845	17.556	1.401e-1	1.632e-1	1.383	17.094	5.912e-4	9.482e-4
	AAL93962.1| L-beta-lysine 5,6-aminomutase alpha subunit																							
**FN1864**	-0.800	8.341	8.261e-2	1.774e-1	-2.269	7.056	2.158e-3	7.469e-3	-0.129	8.808	9.99e-2	5.618e-1	0.671	8.212	1.062e-1	2.425e-1	-1.469	6.256	1.605e-1	2.155e-1	2.141	6.927	9.178e-5	7.761e-5
	AAL93963.1| DNA mismatch repair protein mutS																							
**FN1865**									-0.594	3.764														
	AAL93964.1| Hypothetical protein																							
**FN1866**	-0.479	20.901	5.418e-2	1.001e-1	-0.891	20.673	3.507e-5	3.583e-5	-0.041	21.135	7.773e-2	4.231e-1	0.438	20.860	6.768e-2	1.233e-1	-0.412	20.194	1.69e-1	2.359e-1	0.850	20.632	3.986e-4	5.542e-4
	AAL93965.1| Lysine 2,3-aminomutase																							
**FN1867**	0.241	19.771	2.013e-1	5.62e-1	-1.299	18.415	7.08e-6	3.409e-6	0.438	19.764	1.161e-3	2.926e-3	0.197	20.209	1.97e-1	5.833e-1	-1.540	18.656	8.989e-2	6.929e-2	1.737	18.853	2.003e-4	2.19e-4
	AAL93966.1| Zn-dependent alcohol dehydrogenases and related dehydrogenases																							
**FN1868**	-0.951	14.428	3.495e-3	2.773e-3	-1.524	14.040	1.152e-4	1.877e-4	-0.191	14.985	7.034e-3	2.731e-2	0.761	14.237	1.486e-2	1.39e-2	-0.572	13.089	1.043e-1	9.186e-2	1.333	13.850	3.369e-7	2.837e-8
	AAL93967.1| Hypothetical cytosolic protein																							
**FN1869**	-0.432	15.372	7.765e-2	1.633e-1	-1.440	14.549	9.958e-5	1.555e-4	-0.375	15.226	4.489e-3	1.604e-2	0.057	14.998	2.608e-1	8.458e-1	-1.008	14.117	9.304e-2	7.475e-2	1.065	14.174	1.298e-4	1.215e-4
	AAL93968.1| Hypothetical protein																							
**FN1870**									-0.932	4.817														
	AAL93969.1| unknown																							
**FN1871**																								
	AAL93970.1| unknown																							
**FN1872**					-2.280	6.924																		
	AAL93971.1| unknown																							
**FN1873**	-1.080	11.153	5.665e-4	1.858e-4	-2.301	10.116	1.864e-5	1.482e-5	-0.124	11.905	5.623e-2	2.954e-1	0.956	11.028	6.534e-3	3.762e-3	-1.221	9.036	2.383e-3	1.633e-4	2.177	9.992	6.49e-4	1.085e-3
	AAL93972.1| Bis(5'-nucleosyl)-tetraphosphatase																							
**FN1874**	-1.394	10.062	5.462e-3	5.12e-3	-1.455	10.186	3.119e-5	3.028e-5	-1.083	10.169	9.898e-5	1.073e-4	0.311	8.979	1.413e-1	3.702e-1	-0.061	8.791	3.998e-1	8.696e-1	0.372	9.103	7.796e-3	2.612e-2
	AAL93973.1| Ribose 5-phosphate isomerase																							

Log$_2$ Ratios

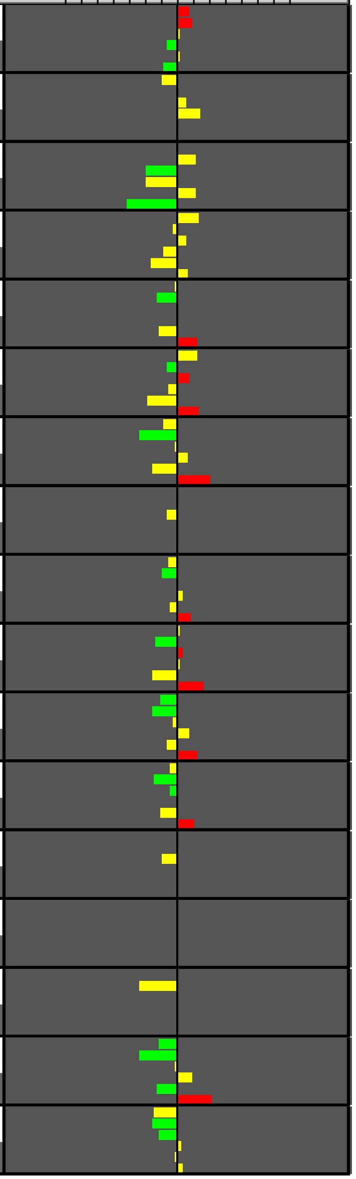

Show detected proteins only
Show all proteins
Filter by category:
GO: amino acid transport

Proteins found: 1424

Enter (or paste) list of ORFs

Find ORFs

Test	Cutoff
q-Value	.005
p-Value	

Dot Plots
Dot Plots

	Signif	Direction	Applies To
red	yes	+	ratios, bars
yellow	no	n/a	bars
green	yes	–	ratios, bars
pink	yes	+	*p*- , *q*-Values
light green	yes	–	*p*- , *q*-Values

FnPg vs Fn
FnPgSg vs Fn
FnSg vs FnPg
FnSg vs Fn
FnPgSg vs FnPg
FnPgSg vs FnSg

ORF	*FnPg vs Fn* Ratio	Sum	*q*-Val	*p*-Val	*FnSg vs Fn* Ratio	Sum	*q*-Val	*p*-Val	*FnPgSg vs Fn* Ratio	Sum	*q*-Val	*p*-Val	*FnPgSg vs FnPg* Ratio	Sum	*q*-Val	*p*-Val	*FnSg vs FnPg* Ratio	Sum	*q*-Val	*p*-Val	*FnPgSg vs FnSg* Ratio	Sum	*q*-Val	*p*-Val
**FN1875**	0.043	14.147	2.717e-1	8.319e-1	0.587	14.875	4.876e-3	2.011e-2	-0.055	13.845	1.35e-1	7.898e-1	-0.098	14.092	1.804e-1	5.147e-1	0.544	14.919	2.095e-2	4.997e-3	-0.642	14.821	1.012e-3	1.968e-3
	AAL93974.1| Peptidyl-prolyl cis-trans isomerase																							
**FN1877**													0.324	5.494										
	AAL93976.1| Guanine-hypoxanthine permease																							
**FN1878**									-1.230	5.874														
	AAL93977.1| unknown																							
**FN1879**									-0.567	10.101														
	AAL93978.1| SSU ribosomal protein S20P																							
**FN1880**	1.807	12.789	2.521e-6	1.122e-7	1.246	12.413	1.623e-3	5.324e-3	0.261	11.039	6.04e-2	3.195e-1	-1.546	13.050	1.109e-3	2.871e-4	-0.560	14.219	3.269e-2	1.06e-2	-0.985	12.674	2.301e-3	5.514e-3
	AAL93979.1| Oxygen-insensitive NAD(P)H nitroreductase																							
**FN1881**													-0.635	6.009			0.309	7.137			-0.944	6.502		
	AAL93980.1| Esterase																							
**FN1884**	-0.444	11.353							0.857	12.449	9.932e-3	4.091e-2	1.301	12.209										
	AAL93983.1| unknown																							
**FN1890**	2.010	7.817							1.438	7.041	7.304e-4	1.61e-3	-0.572	9.255										
	AAL93989.1| Hypothetical protein																							
**FN1891**	0.939	8.361	1.275e-1	3.085e-1	1.317	8.923	3.924e-4	9.01e-4	0.151	7.369	1.013e-1	5.703e-1	-0.788	8.512	1.377e-1	3.577e-1	0.377	9.862	2.765e-1	4.99e-1	-1.166	9.074	2.615e-4	3.219e-4
	AAL93990.1| Glycerophosphoryl diester phosphodiesterase																							
**FN1893**	-0.583	18.694	1.202e-4	1.828e-5	-0.896	18.566	4.388e-6	1.551e-6	-1.052	18.021	3.598e-4	6.499e-4	-0.469	17.642	1.688e-2	1.682e-2	-0.312	17.983	1.168e-2	2.149e-3	-0.156	17.514	5.534e-2	2.499e-1
	AAL93991.1| Fusobacterium outer membrane protein family																							
**FN1895**																								
	AAL93994.1| Hypothetical protein																							
**FN1898**	-1.157	10.576	1.509e-2	1.872e-2	-0.046	11.871	1.335e-1	8.185e-1	-0.600	10.928	5.152e-3	1.893e-2	0.557	9.976	1.009e-1	2.226e-1	1.111	10.714	5.218e-2	2.468e-2	-0.554	11.271	1.264e-2	4.628e-2
	AAL93997.1| Sugar transport ATP-binding protein																							
**FN1899**	0.060	15.420	2.563e-1	7.682e-1	0.424	15.968	6.625e-4	1.77e-3	-0.635	14.521	4.938e-4	9.835e-4	-0.695	14.785	3.412e-2	4.611e-2	0.364	16.028	1.089e-1	9.967e-2	-1.058	15.333	1.374e-4	1.312e-4
	AAL93998.1| Hypothetical lipoprotein																							
**FN1901**																								
	AAL94000.1| Transcription regulator, CRP family																							
**FN1902**	-0.028	10.100	2.452e-1	7.242e-1	-0.543	9.769	8.229e-3	3.709e-2	-0.214	9.710	3.108e-2	1.498e-1	-0.186	9.886	1.019e-1	2.263e-1	-0.516	9.741	6.493e-2	3.603e-2	0.329	9.555	4.047e-2	1.755e-1
	AAL94001.1| Deoxycytidylate deaminase																							
**FN1903**	-2.264	9.805	3.089e-3	2.337e-3					1.093	12.958	4.744e-5	3.787e-5	3.357	10.898	1.618e-4	1.943e-5								
	AAL94002.1| Coenzyme A disulfide reductase/ disulfide bond regulator domain																							
**FN1905**					-0.069	4.069			-0.290	3.460											-0.221	3.779		
	AAL94004.1| outer membrane protein																							

Log$_2$ Ratios

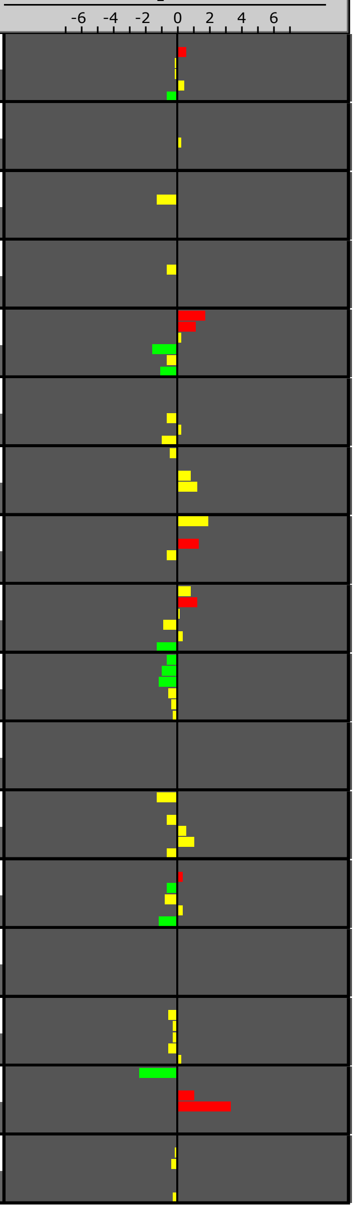

Show detected proteins only
Show all proteins
Filter by category:
GO: amino acid transport

Proteins found:
1424

Enter (or paste) list of ORFs
Find ORFs

Test	Cutoff
q-Value	.005
p-Value	

Dot Plots
Dot Plots

	Signif	Direction	Applies To
red	yes	+	ratios, bars
yellow	no	n/a	bars
green	yes	–	ratios, bars
pink	yes	+	*p*- , *q*-Values
light green	yes	–	*p*- , *q*-Values

FnPg vs Fn — FnSg vs Fn
FnPgSg vs Fn — FnPgSg vs FnPg
FnSg vs FnPg — FnPgSg vs FnSg

Spectral Counts
Fn Summary Table

Fusobacterium nucleatum

Hackett Laboratory UW

Fn Summary Table | FnPg vs Fn | FnSg vs Fn | FnPgSg vs Fn | FnPgSg vs FnPg | FnSg vs FnPg | FnPgSg vs FnSg | Fn Coverage

ORF	*FnPg vs Fn* Ratio	Sum	*q*-Val	*p*-Val	*FnSg vs Fn* Ratio	Sum	*q*-Val	*p*-Val	*FnPgSg vs Fn* Ratio	Sum	*q*-Val	*p*-Val	*FnPgSg vs FnPg* Ratio	Sum	*q*-Val	*p*-Val	*FnSg vs FnPg* Ratio	Sum	*q*-Val	*p*-Val	*FnPgSg vs FnSg* Ratio	Sum	*q*-Val	*p*-Val
**FN1906**	0.592	17.159	8.944e-2	1.965e-1	0.904	17.655	3.077e-3	1.158e-2	-0.001	16.361	1.635e-1	9.896e-1	-0.593	17.157	9.311e-2	1.958e-1	0.312	18.247	2.136e-1	3.442e-1	-0.905	17.654	2.89e-3	7.328e-3
	AAL94005.1| Cytosol aminopeptidase																							
**FN1908**	0.384	16.395	2.985e-2	4.49e-2	0.545	16.741	6.616e-4	1.767e-3	-0.372	15.435	6.722e-3	2.592e-2	-0.756	16.023	1.28e-2	1.097e-2	0.161	17.125	1.713e-1	2.414e-1	-0.917	16.369	6.053e-4	9.818e-4
	AAL94007.1| Glycerophosphoryl diester phosphodiesterase																							
**FN1909**	-0.951	13.699	1.873e-3	1.17e-3	-1.182	13.653	2.194e-5	1.868e-5	-0.228	14.218	5.994e-4	1.256e-3	0.723	13.471	8.19e-3	5.559e-3	-0.231	12.702	1.614e-1	2.176e-1	0.954	13.425	3.667e-4	4.989e-4
	AAL94008.1| UDP-3-O-[3-hydroxymyristoyl] glucosamine N-acyltransferase																							
**FN1910**	-1.075	15.909	2.784e-3	2.019e-3	-1.868	15.301	2.212e-4	4.327e-4	0.532	17.313	1.498e-3	4.204e-3	1.607	16.441	1.193e-4	1.237e-5	-0.793	14.225	6.115e-2	3.244e-2	2.400	15.833	1.251e-5	4.166e-6
	AAL94009.1| periplasmic protein																							
**FN1911**	0.539	21.645	2.035e-2	2.771e-2	-0.044	21.247	1.147e-1	6.882e-1	0.105	21.007	3.143e-2	1.518e-1	-0.434	21.749	3.584e-2	4.941e-2	-0.583	21.785	4.635e-2	1.968e-2	0.148	21.351	4.061e-2	1.762e-1
	AAL94010.1| Outer membrane protein																							
**FN1912**	-1.505	8.335	6.81e-4	2.483e-4	-1.482	8.543	3.111e-4	6.746e-4	-2.460	7.175			-0.955	5.874			0.023	7.038	4.131e-1	9.189e-1	-0.979	6.082		
	AAL94011.1| Hypothetical protein																							
**FN1913**	-0.697	12.644	4.043e-2	6.692e-2	-0.721	12.805	9.018e-4	2.628e-3	-0.991	12.146	2.015e-4	2.936e-4	-0.294	11.654	1.722e-1	4.826e-1	-0.024	12.108	4.204e-1	9.471e-1	-0.270	11.814	8.888e-3	3.045e-2
	AAL94012.1| hydrolase (HD superfamily)																							
**FN1914**	0.655	13.979	1.845e-1	5.025e-1	-0.288	13.220	8.128e-3	3.655e-2	-0.623	12.497	1.233e-3	3.184e-3	-1.278	13.356	1.248e-1	3.081e-1	-0.943	13.875	2.323e-1	3.908e-1	-0.335	12.597	1.324e-2	4.887e-2
	AAL94013.1| Anti-sigma F factor antagonist																							
**FN1917**	0.608	4.561			1.633	5.771											1.025	6.379						
	AAL94016.1| tRNA delta(2)-isopentenylpyrophosphate transferase																							
**FN1918**	-0.558	11.698	7.937e-2	1.682e-1	0.327	12.767	1.635e-5	1.22e-5	0.288	12.341	2.08e-2	9.434e-2	0.846	11.987	3.456e-2	4.702e-2	0.884	12.210	7.514e-2	4.748e-2	-0.038	13.056	1.471e-1	7.463e-1
	AAL94017.1| SPO0B-associated GTP-binding protein																							
**FN1919**	-2.878	8.684	3.404e-5	2.617e-6	-2.663	9.083	2.691e-6	7.264e-7	-2.787	8.571	1.538e-5	5.96e-6	0.091	5.897	2.678e-1	8.767e-1	0.215	6.205	3.322e-1	6.495e-1	-0.124	6.296	1.559e-1	7.988e-1
	AAL94018.1| Methyltransferase																							
**FN1920**																	-1.354	5.354						
	AAL94019.1| tRNA (5-methylaminomethyl-2-thiouridylate) -methyltransferase																							
**FN1922**	0.598	7.441	2.103e-6	7.977e-8	-0.162	6.865	2.667e-4	5.49e-4	-0.377	6.262			-0.975	7.064			-0.761	7.463	8.127e-6	2.336e-8	-0.215	6.488		
	AAL94021.1| Hypothetical protein																							
**FN1923**					-0.788	3.958																		
	AAL94022.1| Adenine-specific methyltransferase																							
**FN1925**																								
	AAL94024.1| Arsenical pump membrane protein																							
**FN1926**	0.597	12.116	1.235e-1	2.962e-1	0.441	12.145	1.104e-5	6.865e-6	0.306	11.621	2.064e-2	9.352e-2	-0.291	12.422	1.894e-1	5.513e-1	-0.156	12.741	3.582e-1	7.286e-1	-0.135	12.450	6.65e-2	3.065e-1
	AAL94025.1| Nitrogen regulatory IIA protein																							
**FN1927**	0.658	14.381	9.963e-2	2.262e-1	1.612	15.520	5.807e-5	7.498e-5	-0.585	12.934	1.919e-3	5.766e-3	-1.243	13.796	5.654e-2	9.618e-2	0.955	16.177	5.265e-2	2.513e-2	-2.197	14.935	2.101e-4	2.342e-4
	AAL94026.1| DEGV protein																							

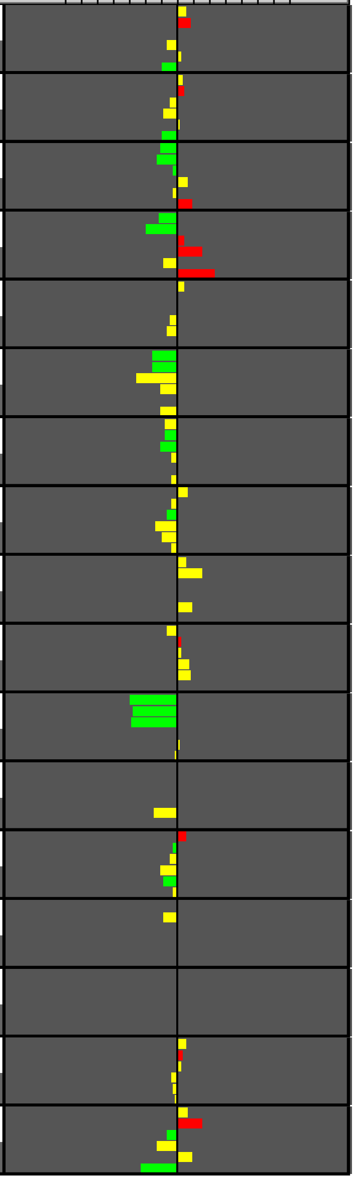

Show detected proteins only
Show all proteins
Filter by category:
GO: amino acid transport

Proteins found: 1424

Enter (or paste) list of ORFs

Find ORFs

Test	Cutoff
q-Value	.005
p-Value	

Dot Plots
Dot Plots

	Signif	Direction	Applies To
red	yes	+	ratios, bars
yellow	no	n/a	bars
green	yes	–	ratios, bars
pink	yes	+	*p*- , *q*-Values
light green	yes	–	*p*- , *q*-Values

FnPg vs Fn — *FnSg vs Fn*
FnPgSg vs Fn — *FnPgSg vs FnPg*
FnSg vs FnPg — *FnPgSg vs FnSg*

ORF	Protein	*FnPg vs Fn* Ratio	Sum	*q*-Val	*p*-Val	*FnSg vs Fn* Ratio	Sum	*q*-Val	*p*-Val	*FnPgSg vs Fn* Ratio	Sum	*q*-Val	*p*-Val
FN1928	AAL94027.1| Transcriptional regulator, MerR family	1.094	8.521			0.431	8.043			0.186	7.409		
FN1929	AAL94028.1| Competence-damage protein cinA	-0.664	11.498	1.087e-1	2.519e-1	0.184	12.529	1.035e-1	6.135e-1	-1.330	10.628	1.076e-2	4.475e-2
FN1931	AAL94030.1| Protease	0.294	5.321			1.095	6.306						
FN1933	AAL94032.1| Hypothetical protein	-1.191	6.775	1.782e-3	1.088e-3	-0.669	7.482	2.596e-2	1.364e-1	-0.152	7.611	5.258e-2	2.737e-1
FN1935	AAL94034.1| Adenine-specific methyltransferase	-0.856	6.026			-0.378	6.688	5.619e-2	3.163e-1	-1.754	4.924		
FN1939	AAL94038.1| Hypothetical protein	0.385	8.111			-0.375	7.536	5.459e-2	3.067e-1	-0.314	7.208	2.067e-2	9.369e-2
FN1941	AAL94040.1| ClpB protein	1.481	15.151	1.812e-4	3.292e-5	1.994	15.848	1.202e-3	3.715e-3	-0.193	13.273	7.688e-3	3.029e-2
FN1942	AAL94041.1| putative DNA-binding protein	1.174	5.735			-0.373	4.373						
FN1943	AAL94042.1| Tryptophanase	2.132	22.304	3.643e-3	2.929e-3	1.724	22.081	1.794e-5	1.398e-5	-0.290	19.679	1.37e-3	3.709e-3
FN1948	AAL94044.1| Hypothetical protein												
FN1949	AAL94045.1| Xaa-Pro dipeptidase	2.365	9.195	2.717e-2	3.982e-2	2.545	9.559	1.518e-7	1.049e-8	-1.370	5.255		
FN1950	AAL94046.1| Serine protease									-3.486	8.201		
FN1951	AAL94047.1| ATPase associated with chromosome architecture/replication					-0.914	4.914						
FN1956	AAL94052.1| Hypothetical protein												
FN1964	AAL94054.1| O-linked GLCNAC transferase	-0.609	9.042	5.524e-5	5.711e-6	-1.125	8.710	1.536e-4	2.78e-4	-0.805	8.642	6.811e-3	2.631e-2
FN1965	AAL94055.1| Tetratricopeptide repeat family protein	0.486	10.923	3.23e-2	4.977e-2	0.148	10.770	9.843e-2	5.801e-1	0.256	10.490	2.682e-2	1.26e-1
FN1966	AAL94056.1| Hypothetical protein	-0.851	10.944	7.543e-3	7.998e-3	0.307	12.286	1.663e-2	8.362e-2	-0.059	11.533	1.202e-1	6.915e-1

ORF	*FnPgSg vs FnPg* Ratio	Sum	*q*-Val	*p*-Val	*FnSg vs FnPg* Ratio	Sum	*q*-Val	*p*-Val	*FnPgSg vs FnSg* Ratio	Sum	*q*-Val	*p*-Val
FN1928	-0.908	8.707			-0.663	9.137			-0.245	8.229	1.09e-1	5.294e-1
FN1929	-0.666	10.168	1.428e-1	3.756e-1	0.847	11.866	1.253e-1	1.302e-1	-1.513	11.200	7.144e-3	2.342e-2
FN1931					0.801	6.600						
FN1933	1.040	6.623	9.028e-3	6.443e-3	0.522	6.291	2.27e-1	3.773e-1	0.517	7.330	5.416e-2	2.44e-1
FN1935	-0.898	4.272			0.477	5.832			-1.375	4.934		
FN1939	-0.699	7.797			-0.760	7.920			0.061	7.221	1.685e-1	8.766e-1
FN1941	-1.674	14.958	4.273e-6	1.14e-7	0.513	17.330	7.283e-2	4.464e-2	-2.187	15.656	1.467e-3	3.14e-3
FN1942					-1.547	5.547						
FN1943	-2.422	22.014	5.062e-3	2.568e-3	-0.408	24.213	8.632e-2	6.342e-2	-2.014	21.791	6.105e-5	4.534e-5
FN1948					0.350	6.149						
FN1949	-3.735	7.824			0.180	11.924	3.175e-1	6.074e-1	-3.915	8.189		
FN1950												
FN1951												
FN1956					-0.298	7.700						
FN1964	-0.196	8.237	1.749e-1	4.93e-1	-0.516	8.101	3.728e-2	1.334e-2	0.320	7.906	7.999e-2	3.761e-1
FN1965	-0.230	11.179	1.057e-1	2.403e-1	-0.338	11.256	1.657e-1	2.279e-1	0.108	11.026	1.362e-1	6.822e-1
FN1966	0.792	10.885	9.561e-3	7.033e-3	1.158	11.435	6.287e-3	8.25e-4	-0.366	12.227	8.625e-3	2.939e-2

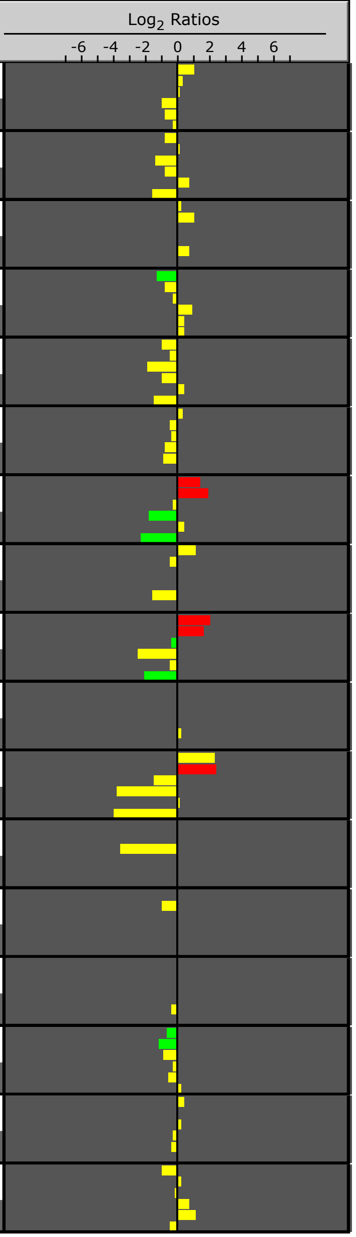

○ Show detected proteins only
○ Show all proteins
☐ Filter by category:
GO: amino acid transport

Proteins found: 1424

Enter (or paste) list of ORFs

Find ORFs

Test	Cutoff
q-Value	.005
p-Value	

Dot Plots
Dot Plots

	Signif	Direction	Applies To
red	yes	+	ratios, bars
yellow	no	n/a	bars
green	yes	–	ratios, bars
pink	yes	+	*p*- , *q*-Values
light green	yes	–	*p*- , *q*-Values

FnPg vs Fn, *FnSg vs Fn*, *FnPgSg vs Fn*, *FnPgSg vs FnPg*, *FnSg vs FnPg*, *FnPgSg vs FnSg*

ORF	Description	*FnPg vs Fn* Ratio	Sum	*q*-Val	*p*-Val	*FnSg vs Fn* Ratio	Sum	*q*-Val	*p*-Val	*FnPgSg vs Fn* Ratio	Sum	*q*-Val	*p*-Val	*FnPgSg vs FnPg* Ratio	Sum	*q*-Val	*p*-Val	*FnSg vs FnPg* Ratio	Sum	*q*-Val	*p*-Val	*FnPgSg vs FnSg* Ratio	Sum	*q*-Val	*p*-Val
**FN1970**	AAL94060.1| Hemin-binding periplasmic protein hmuT precursor									1.396	6.219														
**FN1971**	AAL94061.1| Hemin receptor	0.778	9.641			-1.568	7.478	9.772e-4	2.897e-3	0.109	8.767	9.539e-2	5.332e-1	-0.670	9.749			-2.347	8.257			1.677	7.587	5.685e-4	8.956e-4
**FN1972**	AAL94062.1| unknown	2.277	9.584			1.441	8.932	1.657e-2	8.333e-2	0.231	7.335	4.225e-2	2.142e-1	-2.046	9.816			-0.837	11.209			-1.209	9.164	2.551e-2	1.038e-1
**FN1973**	AAL94063.1| Translation initiation inhibitor	2.182	12.026	1.656e-3	9.815e-4	0.927	10.956	5.059e-3	2.097e-2	2.612	12.252	3.902e-5	2.856e-5	0.430	14.638	8.101e-3	5.447e-3	-1.255	13.137	3.793e-3	3.849e-4	1.685	13.567	1.902e-5	8.481e-6
**FN1974**	AAL94064.1| DNA/RNA helicase (DEAD/DEAH BOX family)					-0.437	6.052																		
**FN1975**	AAL94065.1| ATP-dependent RNA helicase	-1.400	13.702	4.849e-4	1.476e-4	-0.734	14.551	6.127e-4	1.597e-3	-0.653	14.244	1.97e-4	2.837e-4	0.746	13.048	2.137e-3	7.62e-4	0.665	13.152	5.242e-2	2.491e-2	0.081	13.898	1.217e-1	5.996e-1
**FN1976**	AAL94066.1| 4-amino-4-deoxychorismate lyase	-0.800	10.426	7.915e-2	1.675e-1	0.411	11.821	1.555e-2	7.767e-2	-1.202	9.819	9.477e-4	2.23e-3	-0.403	9.223	2.016e-1	6.031e-1	1.211	11.021	6.886e-2	4.014e-2	-1.613	10.618	9.677e-4	1.855e-3
**FN1977**	AAL94067.1| Cell cycle protein MesJ	0.237	6.286			0.169	6.403	1.065e-1	6.335e-1	-0.923	4.923			-1.160	5.364			-0.068	6.640			-1.092	5.480		
**FN1978**	AAL94068.1| Cell division protein ftsH	-1.935	13.741	2.614e-3	1.852e-3	-1.358	14.502	1.201e-5	7.948e-6	-0.599	14.873	1.937e-4	2.766e-4	1.336	13.142	1.571e-2	1.528e-2	0.577	12.567	1.734e-1	2.467e-1	0.759	13.903	4.993e-4	7.463e-4
**FN1979**	AAL94069.1| SSU ribosomal protein S15P	-2.888	10.456	2.983e-4	7.006e-5					1.316	14.456	1.183e-3	3.004e-3	4.204	11.772	2.512e-3	9.481e-4								
**FN1980**	AAL94070.1| Transporter																					-0.244	4.517		
**FN1983**	AAL94073.1| Alkyl hydroperoxide reductase C22 protein	-1.070	16.582	6.53e-4	2.317e-4	-1.088	16.748	5.663e-6	2.333e-6	0.554	18.002	2.665e-3	8.536e-3	1.624	17.136	1.102e-3	2.838e-4	-0.019	15.679	4.017e-1	8.765e-1	1.642	17.302	4.642e-4	6.762e-4
**FN1984**	AAL94074.1| Thioredoxin reductase	0.205	16.782	1.926e-1	5.319e-1	-0.591	16.170	4.172e-4	9.723e-4	0.514	16.887	2.091e-3	6.385e-3	0.309	17.296	1.223e-1	2.988e-1	-0.796	16.375	1.125e-1	1.059e-1	1.105	16.684	5.058e-4	7.595e-4
**FN1985**	AAL94075.1| Inner membrane protein	-0.583	12.690	1.336e-1	3.273e-1	-0.248	13.209	2.038e-2	1.046e-1	-0.717	12.352	1.767e-3	5.194e-3	-0.134	11.973	2.613e-1	8.482e-1	0.335	12.626	3.078e-1	5.806e-1	-0.469	12.492	1.799e-3	4.055e-3
**FN1986**	AAL94076.1| Hypothetical protein	-0.000	16.421	3.094e-1	9.99e-1	-0.445	16.161	1.66e-2	8.349e-2	-0.356	15.861	2.635e-3	8.419e-3	-0.356	16.065	6.746e-2	1.227e-1	-0.445	16.160	1.209e-1	1.211e-1	0.089	15.805	1.387e-1	6.968e-1
**FN1987**	AAL94077.1| Transcriptional regulator, GntR family	2.122	7.705			-0.299	5.469			-0.545	4.834	1.357e-3	3.658e-3	-2.667	7.160			-2.421	7.591			-0.246	4.924		
**FN1988**	AAL94078.1| Tyrosine phenol-lyase	3.596	15.052	3.588e-2	5.713e-2	4.705	16.346	1.624e-3	5.33e-3	0.987	12.239	3.608e-4	6.525e-4	-2.609	16.039	4.636e-2	7.101e-2	1.109	19.941	6.103e-2	3.232e-2	-3.718	17.333	2.444e-3	5.965e-3

Log$_2$ Ratios

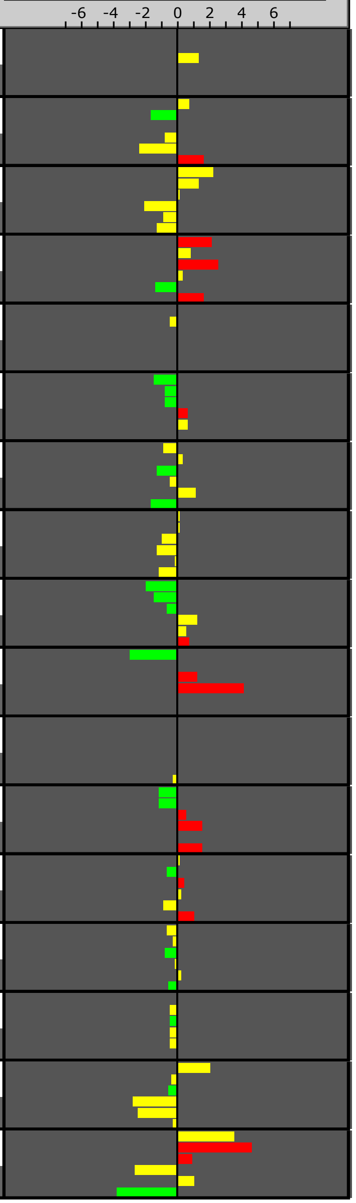

Show detected proteins only
Show all proteins
Filter by category:
GO: amino acid transport

Proteins found:
1424

Enter (or paste) list of ORFs

Find ORFs

Test	Cutoff
q-Value	.005
p-Value	

Dot Plots
Dot Plots

	Signif	Direction	Applies To
red	yes	+	ratios, bars
yellow	no	n/a	bars
green	yes	–	ratios, bars
pink	yes	+	*p*- , *q*-Values
light green	yes	–	*p*- , *q*-Values

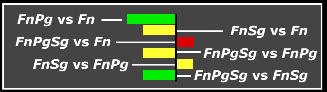

ORF	Protein	*FnPg vs Fn* Ratio	Sum	*q*-Val	*p*-Val	*FnSg vs Fn* Ratio	Sum	*q*-Val	*p*-Val	*FnPgSg vs Fn* Ratio	Sum	*q*-Val	*p*-Val	*FnPgSg vs FnPg* Ratio	Sum	*q*-Val	*p*-Val	*FnSg vs FnPg* Ratio	Sum	*q*-Val	*p*-Val	*FnPgSg vs FnSg* Ratio	Sum	*q*-Val	*p*-Val
FN1989	AAL94079.1| Sodium-dependent tyrosine transporter																	-0.530	7.654						
FN1991	AAL94081.1| Glucosamine-1-phosphate acetyltransferase	0.141	17.361	4.567e-2	7.922e-2	-0.176	17.229	1.402e-2	6.907e-2	-0.018	16.999	9.589e-2	5.363e-1	-0.159	17.343	3.886e-2	5.53e-2	-0.317	17.369	3.052e-2	9.394e-3	0.158	17.211	2.173e-2	8.692e-2
FN1992	AAL94082.1| Ribose-phosphate pyrophosphokinase	0.581	16.276	1.639e-2	2.083e-2	0.654	16.533	5.373e-3	2.247e-2	-0.264	15.227	2.353e-4	3.693e-4	-0.845	16.012	1.1e-2	8.775e-3	0.073	17.114	3.394e-1	6.708e-1	-0.918	16.269	3.882e-3	1.07e-2
FN1993	AAL94083.1| SUA5 protein	-0.018	5.187			-0.248	5.142			0.179	5.180			0.196	5.366			-0.230	5.124			0.427	5.321		
FN1994	AAL94084.1| Hypothetical protein	-0.089	11.457	2.786e-1	8.611e-1	-1.234	10.496	1.32e-2	6.439e-2	-0.324	11.019	5.567e-3	2.076e-2	-0.235	11.134	2.212e-1	6.809e-1	-1.145	10.407	1.637e-1	2.231e-1	0.911	10.173	3.862e-2	1.663e-1
FN1995	AAL94085.1| Hypothetical protein	-1.152	5.152							1.009	7.110	3.173e-2	1.535e-1	2.161	6.161										
FN2001	AAL94091.1| Hypothetical protein	0.901	7.743			0.893	7.921	1.367e-3	4.347e-3									-0.007	8.821						
FN2002	AAL94092.1| Permease																								
FN2007	AAL94097.1| Glutathione peroxidase	-0.841	8.192	5.876e-2	1.124e-1	-1.896	7.321	1.205e-3	3.725e-3	-1.867	6.961	1.598e-3	4.581e-3	-1.027	6.324	1.069e-1	2.45e-1	-1.055	6.480	1.705e-1	2.394e-1	0.028	5.454	1.798e-1	9.482e-1
FN2008	AAL94098.1| Glycine betaine transport ATP-binding protein	2.271	6.224			0.312	4.449											-1.960	6.720						
FN2009	AAL94099.1| Glycine betaine transport system permease protein																	-0.109	6.075						
FN2011	AAL94101.1| Valyl-tRNA synthetase	-1.337	14.011	5.679e-3	5.396e-3	-0.766	14.767	1.108e-2	5.252e-2	-0.958	14.186	2.145e-4	3.228e-4	0.379	13.053	1.128e-1	2.655e-1	0.571	13.430	1.852e-1	2.754e-1	-0.192	13.809	1.261e-1	6.243e-1
FN2013	AAL94103.1| GTP-binding protein	-0.281	4.924			-0.050	5.340											0.231	5.059						
FN2014	AAL94104.1| ATP-dependent protease La	-1.417	15.225	3.257e-5	2.448e-6	-1.139	15.688	8.765e-6	4.761e-6	-0.887	15.552	2.858e-5	1.561e-5	0.531	14.339	5.067e-3	2.571e-3	0.278	14.270	7.903e-2	5.265e-2	0.253	14.801	5.745e-3	1.793e-2
FN2015	AAL94105.1| ATP-dependent clp protease ATP-binding subunit clpX	-0.748	13.545	2.051e-3	1.34e-3	-0.392	14.086	2.191e-3	7.61e-3	-0.687	13.402	3.541e-4	6.352e-4	0.061	12.857	1.945e-1	5.725e-1	0.357	13.338	5.169e-2	2.421e-2	-0.296	13.399	1.369e-2	5.077e-2
FN2016	AAL94106.1| ATP-dependent Clp protease proteolytic subunit	-0.730	10.446			-1.657	9.704	6.144e-5	8.136e-5	-0.190	10.782	3.211e-2	1.557e-1	0.540	10.256			-0.927	8.974			1.466	9.513	2.541e-4	3.084e-4
FN2017	AAL94107.1| Trigger factor, ppiase	-0.572	18.961	3.324e-2	5.171e-2	-1.253	18.465	1.917e-5	1.548e-5	-0.023	19.307	1.39e-1	8.169e-1	0.549	18.938	3.72e-2	5.202e-2	-0.681	17.893	1.044e-1	9.202e-2	1.230	18.442	8.06e-4	1.455e-3

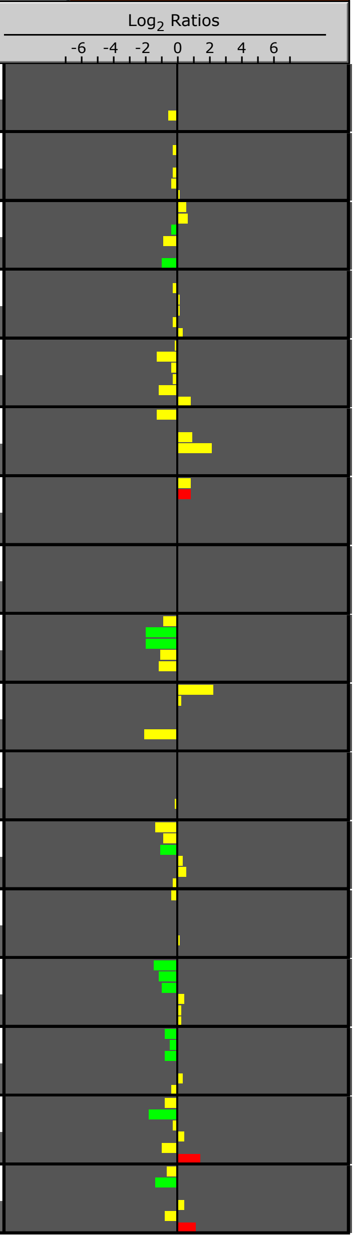

Show detected proteins only
Show all proteins
Filter by category:
GO: amino acid transport

Proteins found: 1424

Enter (or paste) list of ORFs
Find ORFs

Test	Cutoff
q-Value	.005
p-Value	

Dot Plots
Dot Plots

	Signif	Direction	Applies To
red	yes	+	ratios, bars
yellow	no	n/a	bars
green	yes	–	ratios, bars
pink	yes	+	*p*- , *q*-Values
light green	yes	–	*p*- , *q*-Values

FnPg vs Fn — FnSg vs Fn
FnPgSg vs Fn — FnPgSg vs FnPg
FnSg vs FnPg — FnPgSg vs FnSg

ORF	Description	*FnPg vs Fn* Ratio	Sum	*q*-Val	*p*-Val	*FnSg vs Fn* Ratio	Sum	*q*-Val	*p*-Val	*FnPgSg vs Fn* Ratio	Sum	*q*-Val	*p*-Val	*FnPgSg vs FnPg* Ratio	Sum	*q*-Val	*p*-Val	*FnSg vs FnPg* Ratio	Sum	*q*-Val	*p*-Val	*FnPgSg vs FnSg* Ratio	Sum	*q*-Val	*p*-Val
FN2018	AAL94108.1| Single-stranded-DNA-specific exonuclease recJ	-1.837	9.299	1.959e-3	1.25e-3	-1.817	9.504	3.3e-5	3.28e-5	-0.729	10.204	3.476e-4	6.186e-4	1.108	8.570	2.515e-2	2.937e-2	0.021	7.667	4.257e-1	9.677e-1	1.088	8.775	2.493e-4	2.997e-4
FN2019	AAL94109.1| Ribosome-binding factor A	-0.242	10.866			-3.061	8.231			-0.674	10.230	1.126e-2	4.709e-2	-0.432	10.192			-2.820	7.990			2.387	7.557		
FN2020	AAL94110.1| Bacterial Protein Translation Initiation Factor 2 (IF-2)	-0.269	16.155	7.182e-3	7.491e-3	0.002	16.611	1.571e-1	9.905e-1	-0.211	16.009	6.937e-5	6.416e-5	0.058	15.945	1.263e-1	3.136e-1	0.271	16.342	1.647e-1	2.254e-1	-0.213	16.400	6.688e-2	3.084e-1
FN2022	AAL94112.1| N utilization substance protein A	-1.021	12.451	1.198e-8	4.849e-11	-0.330	13.326	4.754e-4	1.149e-3	-0.503	12.765	7.657e-6	2.177e-6	0.518	11.948	3.487e-5	1.551e-6	0.690	12.305	3.297e-3	3.129e-4	-0.172	12.823	3.686e-3	9.988e-3
FN2023	AAL94113.1| Hypothetical cytosolic protein	0.590	7.446	1.739e-1	4.628e-1	0.634	7.675	2.435e-3	8.665e-3	0.064	6.716	1.182e-1	6.787e-1	-0.526	7.510	1.771e-1	5.018e-1	0.044	8.265	4.195e-1	9.433e-1	-0.570	7.739	4.309e-3	1.233e-2
FN2030	AAL94115.1| Inorganic pyrophosphatase	-2.984	13.505	3.26e-5	2.451e-6	-2.696	13.977	1.006e-4	1.575e-4	-0.842	15.443	1.972e-4	2.842e-4	2.142	12.663	2.631e-4	3.529e-5	0.288	10.993	2.211e-1	3.625e-1	1.854	13.135	1.219e-4	1.117e-4
FN2031	AAL94116.1| Thiamine biosynthesis lipoprotein apbE	-1.652	6.561			-2.199	6.199			-0.397	7.612	5.555e-2	2.913e-1	1.255	6.164			-0.547	4.547			1.801	5.801		
FN2033	AAL94118.1| Guanylate kinase	0.225	8.498			-0.528	7.929											-0.753	8.154						
FN2034	AAL94119.1| Protein yicC	-0.476	8.706	9.662e-2	2.172e-1	-0.761	8.605	3.446e-3	1.336e-2	0.512	9.490	4.057e-3	1.417e-2	0.987	9.218	2.408e-2	2.759e-2	-0.285	8.130	2.835e-1	5.167e-1	1.273	9.117	4.011e-5	2.53e-5
FN2035	AAL94120.1| DNA-directed RNA polymerase beta' chain	-0.356	18.018	2.15e-2	2.966e-2	-0.199	18.360	1.235e-2	5.959e-2	-0.320	17.851	1.616e-4	2.134e-4	0.036	17.698	2.41e-1	7.613e-1	0.157	18.004	1.824e-1	2.683e-1	-0.121	18.040	4.997e-2	2.227e-1
FN2036	AAL94121.1| DNA-directed RNA polymerase beta chain	-0.901	17.232	3.329e-3	2.596e-3	-0.392	17.925	8.896e-4	2.585e-3	-0.543	17.386	4.911e-4	9.764e-4	0.358	16.689	6.347e-2	1.131e-1	0.508	17.024	6.91e-2	4.04e-2	-0.151	17.382	1.088e-2	3.88e-2
FN2037	AAL94122.1| LSU ribosomal protein L12P (L7/L12)	0.290	19.709	2.004e-1	5.588e-1	-0.522	19.081	2.186e-2	1.13e-1	2.706	21.921	2.355e-5	1.146e-5	2.416	22.416	1.069e-3	2.693e-4	-0.812	19.372	1.645e-1	2.249e-1	3.228	21.788	1.093e-6	1.38e-7
FN2038	AAL94123.1| LSU ribosomal protein L10P	-0.837	16.073	4.147e-2	6.929e-2	-1.449	15.646	1.934e-3	6.543e-3	0.235	16.942	3.202e-2	1.552e-1	1.072	16.309	2.7e-2	3.258e-2	-0.612	14.809	2.041e-1	3.221e-1	1.684	15.881	2.981e-9	3.763e-11
FN2039	AAL94124.1| LSU ribosomal protein L1P	-0.613	16.846	1.035e-3	4.962e-4	-0.652	16.992	1.718e-3	5.69e-3	-0.291	16.964	2.548e-4	4.082e-4	0.322	16.555	3.595e-3	1.615e-3	-0.039	16.379	3.701e-1	7.671e-1	0.361	16.701	1.01e-2	3.546e-2
FN2040	AAL94125.1| LSU ribosomal protein L11P	-1.158	12.697	7.93e-3	8.544e-3	-2.637	11.403	5.239e-4	1.306e-3	0.868	14.519	2.292e-4	3.552e-4	2.026	13.565	5.44e-4	1.016e-4	-1.479	10.245	9.707e-2	8.072e-2	3.504	12.270	1.581e-4	1.596e-4
FN2041	AAL94126.1| Transcription antitermination protein nusG	0.560	12.042	1.587e-1	4.09e-1	-1.140	10.526	2.006e-4	3.838e-4	-0.606	10.671	3.983e-4	7.467e-4	-1.166	11.436	9.352e-2	1.971e-1	-1.700	11.086	1.258e-1	1.312e-1	0.534	9.920	3.5e-3	9.337e-3
FN2045	AAL94129.1| Ferric uptake regulation protein																	-0.556	7.969						

Log$_2$ Ratios

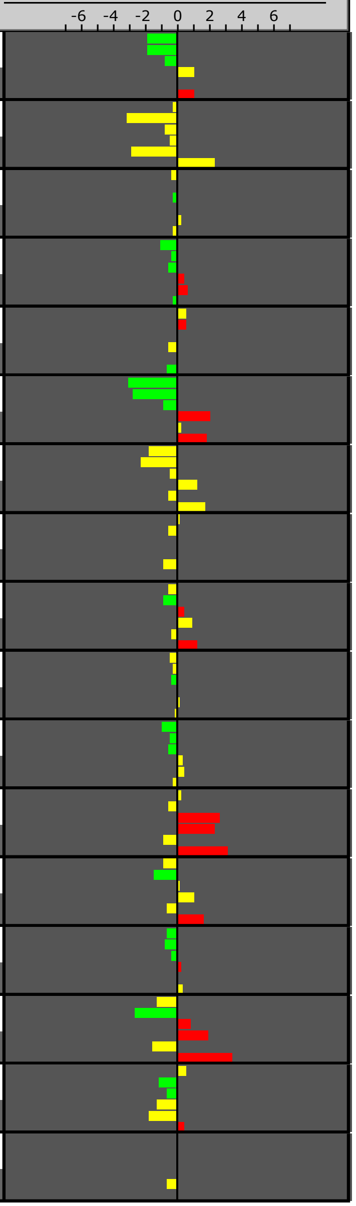

Show detected proteins only
Show all proteins
Filter by category:
GO: amino acid transport

Proteins found: 1424

Enter (or paste) list of ORFs

Find ORFs

Test	Cutoff
q-Value	.005
p-Value	

Dot Plots
Dot Plots

	Signif	Direction	Applies To
red	yes	+	ratios, bars
yellow	no	n/a	bars
green	yes	–	ratios, bars
pink	yes	+	*p*- , *q*-Values
light green	yes	–	*p*- , *q*-Values

FnPg vs Fn
FnSg vs Fn
FnPgSg vs Fn
FnPgSg vs FnPg
FnSg vs FnPg
FnPgSg vs FnSg

ORF	FnPg vs Fn Ratio	Sum	*q*-Val	*p*-Val	FnSg vs Fn Ratio	Sum	*q*-Val	*p*-Val	FnPgSg vs Fn Ratio	Sum	*q*-Val	*p*-Val	FnPgSg vs FnPg Ratio	Sum	*q*-Val	*p*-Val	FnSg vs FnPg Ratio	Sum	*q*-Val	*p*-Val	FnPgSg vs FnSg Ratio	Sum	*q*-Val	*p*-Val	Description
FN2046																	-0.555	8.799							AAL94130.1| Acetyltransferase
FN2047	-3.129	16.328	1.148e-6	3.253e-8	-2.828	16.814	1.022e-6	1.554e-7	-2.902	16.351	6.366e-8	2.144e-9	0.226	13.426	1.36e-1	3.511e-1	0.301	13.685	1.497e-1	1.896e-1	-0.075	13.911	1.179e-1	5.781e-1	AAL94131.1| Fusobacterium outer membrane protein family
FN2048	-0.332	15.532			0.460	16.509	4.768e-2	2.649e-1	0.155	15.816	2.243e-2	1.027e-1	0.488	15.687			0.792	16.177			-0.305	16.664	8.723e-2	4.134e-1	AAL94132.1| Outer membrane protein
FN2049	1.100	17.889	6.173e-2	1.209e-1	0.575	17.548	2.44e-4	4.894e-4	-0.338	16.247	1.241e-3	3.212e-3	-1.438	17.551	5.197e-2	8.444e-2	-0.525	18.648	1.974e-1	3.071e-1	-0.913	17.211	3.001e-5	1.622e-5	AAL94133.1| unknown
FN2050	-0.361	15.648	1.844e-1	5.025e-1	-2.496	13.698	1.488e-4	2.659e-4	-0.104	15.702	1.066e-1	6.041e-1	0.257	15.544	2.138e-1	6.518e-1	-2.135	13.336	1.251e-1	1.297e-1	2.392	13.594	2.816e-3	7.096e-3	AAL94134.1| Hypothetical membrane-spanning protein
FN2051	-0.589	13.778	8.361e-2	1.803e-1	-0.996	13.556	3.358e-3	1.293e-2	0.063	14.226	5.06e-2	2.621e-1	0.652	13.840	7.563e-2	1.439e-1	-0.406	12.967	2.74e-1	4.926e-1	1.058	13.619	4.575e-3	1.338e-2	AAL94135.1| unknown
FN2052	-1.626	14.269	5.543e-3	5.222e-3	-1.817	14.263	4.337e-4	1.021e-3	-0.813	14.879	2.894e-5	1.594e-5	0.814	13.456	5.184e-2	8.412e-2	-0.190	12.637	3.647e-1	7.496e-1	1.004	13.451	5.799e-3	1.815e-2	AAL94136.1| unknown
FN2053	-0.050	12.119	2.835e-1	8.823e-1	-0.412	11.942	8.627e-3	3.921e-2	0.822	12.787	1.662e-3	4.812e-3	0.872	12.941	2.047e-2	2.193e-2	-0.362	11.892	2.323e-1	3.908e-1	1.233	12.764	6.316e-4	1.045e-3	AAL94137.1| Serine/threonine sodium symporter
FN2054	-0.665	15.074	8.638e-4	3.721e-4	-1.117	14.807	4.601e-5	5.443e-5	-0.004	15.531	1.536e-1	9.185e-1	0.661	15.070	3.764e-4	5.696e-5	-0.452	14.142	1.419e-2	2.855e-3	1.112	14.803	1.403e-5	5.063e-6	AAL94138.1| Glucose-6-phosphate isomerase
FN2058	0.479	18.198	1.417e-1	3.525e-1	-1.249	16.655	1.385e-6	2.534e-7	-0.596	16.919	8.122e-5	8.007e-5	-1.075	17.602	7.17e-2	1.335e-1	-1.728	17.134	9.199e-2	7.287e-2	0.653	16.059	5.724e-4	9.045e-4	AAL94142.1| Fusobacterium outer membrane protein family
FN2059	-0.332	15.532			0.460	16.509	4.768e-2	2.649e-1	0.155	15.816	2.243e-2	1.027e-1	0.488	15.687			0.792	16.177			-0.305	16.664	8.723e-2	4.134e-1	AAL94143.1| Outer membrane protein
FN2060	1.100	17.889	6.173e-2	1.209e-1	0.575	17.548	2.44e-4	4.894e-4	-0.338	16.247	1.241e-3	3.212e-3	-1.438	17.551	5.197e-2	8.444e-2	-0.525	18.648	1.974e-1	3.071e-1	-0.913	17.211	3.001e-5	1.622e-5	AAL94144.1| unknown
FN2061	-0.361	15.648	1.844e-1	5.025e-1	-2.496	13.698	1.488e-4	2.659e-4	-0.104	15.702	1.066e-1	6.041e-1	0.257	15.544	2.138e-1	6.518e-1	-2.135	13.336	1.251e-1	1.297e-1	2.392	13.594	2.816e-3	7.096e-3	AAL94145.1| Hypothetical membrane-spanning protein
FN2062	-0.589	13.778	8.361e-2	1.803e-1	-0.996	13.556	3.358e-3	1.293e-2	0.063	14.226	5.06e-2	2.621e-1	0.652	13.840	7.563e-2	1.439e-1	-0.406	12.967	2.74e-1	4.926e-1	1.058	13.619	4.575e-3	1.338e-2	AAL94146.1| unknown
FN2063	-1.626	14.269	5.543e-3	5.222e-3	-1.817	14.263	4.337e-4	1.021e-3	-0.813	14.879	2.894e-5	1.594e-5	0.814	13.456	5.184e-2	8.412e-2	-0.190	12.637	3.647e-1	7.496e-1	1.004	13.451	5.799e-3	1.815e-2	AAL94147.1| unknown
FN2067					-0.420	5.590			0.925	6.547	1.012e-2	4.178e-2									1.346	6.516			AAL94151.1| Thiol:disulfide interchange protein tlpA
FN2068	1.581	7.165	9.753e-3	1.102e-2	1.442	7.210	2.632e-2	1.385e-1	-0.690	4.690			-2.271	6.475			-0.139	8.791	3.744e-1	7.812e-1	-2.132	6.520			AAL94152.1| dGTP triphosphohydrolase

Log$_2$ Ratios: -6 -4 -2 0 2 4 6

Show detected proteins only
Show all proteins
Filter by category:
GO: amino acid transport

Proteins found: 1424

Enter (or paste) list of ORFs

Find ORFs

Test	Cutoff
q-Value	.005
p-Value	

Dot Plots
Dot Plots

	Signif	Direction	Applies To
red	yes	+	ratios, bars
yellow	no	n/a	bars
green	yes	–	ratios, bars
pink	yes	+	*p*- , *q*-Values
light green	yes	–	*p*- , *q*-Values

FnPg vs Fn — FnSg vs Fn
FnPgSg vs Fn — FnPgSg vs FnPg
FnSg vs FnPg — FnPgSg vs FnSg

ORF	*FnPg vs Fn* Ratio	Sum	*q*-Val	*p*-Val	*FnSg vs Fn* Ratio	Sum	*q*-Val	*p*-Val	*FnPgSg vs Fn* Ratio	Sum	*q*-Val	*p*-Val
**FN2069**												
	AAL94153.1| Amino acid carrier protein alsT											
**FN2070**	-0.830	7.749			-0.680	8.083	4.726e-6	1.736e-6	-2.188	6.188		
	AAL94154.1| Cobyric acid synthase											
**FN2073**	0.466	9.147	1.814e-1	4.908e-1	1.727	10.592	2.867e-3	1.06e-2	-0.859	7.618	1.318e-3	3.504e-3
	AAL94157.1| Adenine phosphoribosyltransferase											
**FN2074**												
	AAL94158.1| BslIM											
**FN2075**	-1.552	10.261	2.351e-6	9.778e-8	-1.773	10.224	6.886e-4	1.864e-3	-0.932	10.677	8.5e-4	1.941e-3
	AAL94159.1| Hypothetical protein											
**FN2076**												
	AAL94160.1| MunI regulatory protein											
**FN2078**	-0.696	3.866			0.627	5.373						
	AAL94162.1| Transcriptional regulator, DeoR family											
**FN2081**												
	AAL94165.1| ABC transporter substrate-binding protein											
**FN2082**	0.066	20.838	2.795e-1	8.648e-1	0.195	21.152	1.292e-2	6.282e-2	0.006	20.575	1.619e-1	9.778e-1
	AAL94166.1| Formate--tetrahydrofolate ligase											
**FN2093**	-0.816	10.423	5.559e-2	1.038e-1	-1.909	9.515	7.33e-5	1.061e-4	-0.781	10.254	4.272e-3	1.511e-2
	AAL94177.1| General secretion pathway protein G											
**FN2098**												
	AAL94182.1| MRP-family nucleotide-binding protein											
**FN2100**	-1.098	11.054	2.063e-3	1.352e-3	-1.352	10.984	5.926e-8	2.217e-9	-0.577	11.371	6.915e-6	1.863e-6
	AAL94184.1| Hypothetical protein											
**FN2102**	-0.535	8.617	5.344e-2	9.817e-2	0.005	9.342	1.537e-1	9.65e-1	-1.320	7.629	1.419e-3	3.898e-3
	AAL94186.1| ABC transporter ATP-binding protein											
**FN2103**	-0.584	19.422	1.5e-2	1.858e-2	-0.489	19.701	5.074e-4	1.251e-3	-0.354	19.448	2.946e-4	4.93e-4
	AAL94187.1| tricarboxylate-binding protein											
**FN2105**	-0.841	6.455							0.010	7.103	1.571e-1	9.434e-1
	AAL94189.1| tricarboxylate transport membrane protein RctA											
**FN2106**	0.480	15.105	1.619e-1	4.202e-1	-0.528	14.281	5.001e-4	1.227e-3	-0.334	14.087	9.885e-4	2.356e-3
	AAL94190.1| Transporter											
**FN2107**	-1.519	11.505	5.936e-3	5.733e-3	-2.959	10.249	4.61e-4	1.104e-3	-0.627	12.192	4.277e-3	1.513e-2
	AAL94191.1| Galactokinase											

ORF	*FnPgSg vs FnPg* Ratio	Sum	*q*-Val	*p*-Val	*FnSg vs FnPg* Ratio	Sum	*q*-Val	*p*-Val	*FnPgSg vs FnSg* Ratio	Sum	*q*-Val	*p*-Val
**FN2069**												
**FN2070**	-1.358	5.561			0.150	7.253			-1.507	5.895		
**FN2073**	-1.325	8.288	8.984e-2	1.853e-1	1.261	11.058	5.149e-2	2.403e-2	-2.586	9.733	2.449e-3	5.982e-3
**FN2074**	0.874	4.044										
**FN2075**	0.620	9.329	3.111e-2	4.011e-2	-0.222	8.673	3.026e-1	5.664e-1	0.841	9.292	1.359e-2	5.034e-2
**FN2076**												
**FN2078**					1.323	4.677						
**FN2081**												
**FN2082**	-0.060	20.845	2.705e-1	8.89e-1	0.129	21.217	3.573e-1	7.257e-1	-0.188	21.158	8.588e-2	4.064e-1
**FN2093**	0.035	9.642	2.836e-1	9.489e-1	-1.092	8.699	1.561e-1	2.051e-1	1.127	8.734	7.759e-3	2.596e-2
**FN2098**	-1.026	4.400			0.662	6.272	9.451e-2	7.707e-2	-1.688	5.246		
**FN2100**	0.521	10.477	2.095e-2	2.267e-2	-0.254	9.886	1.564e-1	2.059e-1	0.775	10.407	3.135e-5	1.727e-5
**FN2102**	-0.784	7.298	7.281e-2	1.364e-1	0.540	8.807	1.079e-1	9.792e-2	-1.324	8.022	3.408e-3	9.02e-3
**FN2103**	0.230	19.069	1.024e-1	2.28e-1	0.094	19.117	3.212e-1	6.177e-1	0.136	19.347	3.788e-2	1.626e-1
**FN2105**	0.851	6.466										
**FN2106**	-0.814	14.771	1.085e-1	2.51e-1	-1.009	14.761	1.525e-1	1.965e-1	0.194	13.947	7.47e-3	2.475e-2
**FN2107**	0.891	10.878	5.318e-2	8.754e-2	-1.441	8.730	1.394e-1	1.617e-1	2.332	9.622	2.264e-6	3.328e-7

Log_2 Ratios

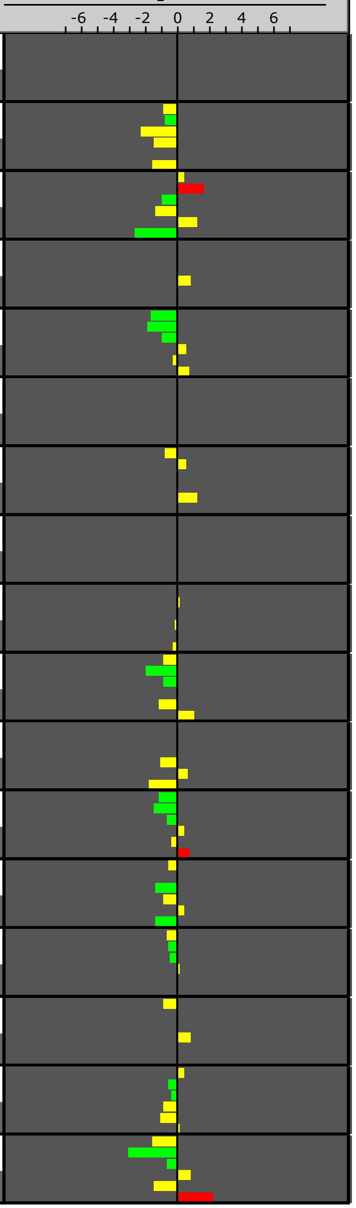

Show detected proteins only
Show all proteins
Filter by category:
GO: amino acid transport

Proteins found: 1424

Enter (or paste) list of ORFs

Find ORFs

Test	Cutoff
q-Value	.005
p-Value	

Dot Plots
Dot Plots

	Signif	Direction	Applies To
red	yes	+	ratios, bars
yellow	no	n/a	bars
green	yes	–	ratios, bars
pink	yes	+	*p*- , *q*-Values
light green	yes	–	*p*- , *q*-Values

FnPg vs Fn
FnSg vs Fn
FnPgSg vs Fn
FnPgSg vs FnPg
FnSg vs FnPg
FnPgSg vs FnSg

Spectral Counts
Fn Summary Table

Fusobacterium nucleatum

Hackett Laboratory UW

Fn Summary Table | FnPg vs Fn | FnSg vs Fn | FnPgSg vs Fn | FnPgSg vs FnPg | FnSg vs FnPg | FnPgSg vs FnSg | Fn Coverage

ORF	Description	*FnPg vs Fn* Ratio	Sum	*q*-Val	*p*-Val	*FnSg vs Fn* Ratio	Sum	*q*-Val	*p*-Val	*FnPgSg vs Fn* Ratio	Sum	*q*-Val	*p*-Val	*FnPgSg vs FnPg* Ratio	Sum	*q*-Val	*p*-Val	*FnSg vs FnPg* Ratio	Sum	*q*-Val	*p*-Val	*FnPgSg vs FnSg* Ratio	Sum	*q*-Val	*p*-Val
**FN2108**	AAL94192.1| Galactose-1-phosphate uridylyltransferase	-0.937	10.048	5.403e-3	5.046e-3	-1.343	9.826	9.941e-4	2.957e-3	-0.390	10.391	1.343e-2	5.75e-2	0.547	9.658	7.714e-3	4.98e-3	-0.407	8.889	4.741e-2	2.051e-2	0.954	9.436	3.829e-4	5.266e-4
**FN2109**	AAL94193.1| UDP-glucose 4-epimerase	0.357	12.048	5.849e-2	1.117e-1	-0.173	11.703	9.735e-3	4.533e-2	0.186	11.674	2.964e-3	9.74e-3	-0.171	12.235	1.342e-1	3.441e-1	-0.530	12.060	7.629e-2	4.895e-2	0.359	11.889	8.152e-4	1.479e-3
**FN2116**	AAL94200.1| Hypothetical exported 24-amino acid repeat protein	-1.796	11.084			-2.952	10.112	5.602e-6	2.29e-6	-3.826	8.850	9.781e-5	1.053e-4	-2.030	7.258			-1.156	8.316			-0.874	6.286	3.594e-2	1.531e-1
**FN2117**	AAL94201.1| Hypothetical exported 24-amino acid repeat protein	-1.864	7.034			-1.371	7.711											0.493	5.847						
**FN2118**	AAL94202.1| Hypothetical exported 24-amino acid repeat protein	-2.982	8.596			-3.296	8.466			-1.537	9.837	1.799e-4	2.482e-4	1.444	7.059			-0.315	5.485			1.759	6.929		
**FN2119**	AAL94203.1| Hypothetical exported 24-amino acid repeat protein	0.059	10.437			-1.111	9.452	7.31e-4	2.023e-3	-0.938	9.237	1.852e-4	2.59e-4	-0.996	9.500			-1.170	9.511			0.173	8.514	1.09e-1	5.294e-1
**FN2120**	AAL94204.1| Hypothetical exported 24-amino acid repeat protein																								
**FN2121**	AAL94205.1| Hypothetical exported 24-amino acid repeat protein	-1.562	13.957	1.544e-2	1.929e-2	-4.149	11.554			-1.244	14.071	1.016e-5	3.242e-6	0.317	12.713	2.038e-1	6.13e-1	-2.588	9.992			2.905	10.309		
**FN2122**	AAL94206.1| Phenylalanyl-tRNA synthetase beta chain	0.322	17.106	6.198e-2	1.216e-1	-0.033	16.936	3.383e-2	1.829e-1	-0.165	16.415	1.034e-2	4.28e-2	-0.487	16.941	3.608e-2	4.988e-2	-0.355	17.257	1.096e-1	1.008e-1	-0.132	16.770	1.905e-2	7.448e-2
**FN2123**	AAL94207.1| Phenylalanyl-tRNA synthetase alpha chain	-0.043	13.279	2.912e-1	9.161e-1	0.116	13.623	1.097e-1	6.545e-1	-0.650	12.469	1.363e-3	3.681e-3	-0.606	12.629	1.18e-1	2.833e-1	0.160	13.580	3.568e-1	7.243e-1	-0.766	12.973	1.906e-2	7.453e-2
**FN2125**	AAL94209.1| DNA gyrase subunit A	-1.221	13.987	1.167e-2	1.362e-2	-1.475	13.917	6.497e-5	8.833e-5	-1.679	13.325	1.06e-5	3.434e-6	-0.458	12.308	1.447e-1	3.824e-1	-0.254	12.696	3.099e-1	5.864e-1	-0.204	12.238	7.585e-3	2.523e-2
**FN2126**	AAL94210.1| DNA gyrase subunit B	-1.937	11.781	3.32e-4	8.134e-5	-2.209	11.693	9.972e-5	1.558e-4	-0.407	13.107	3.654e-4	6.646e-4	1.530	11.374	1.21e-4	1.281e-5	-0.272	9.756	2.109e-1	3.38e-1	1.802	11.286	7.438e-5	5.847e-5
**FN2128**	AAL94212.1| RECF protein																								

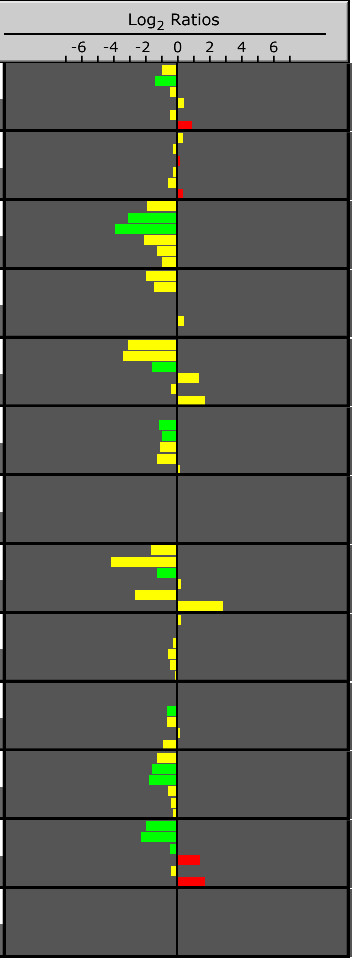

Show detected proteins only
Show all proteins
Filter by category:
GO: amino acid transport

Proteins found:
1424

Enter (or paste) list of ORFs

Find ORFs

Test	Cutoff
***q*-Value**	**.005**
p-Value	

Dot Plots
Dot Plots

	Signif	Direction	Applies To
red	yes	+	ratios, bars
yellow	no	n/a	bars
green	yes	–	ratios, bars
pink	yes	+	*p*- , *q*-Values
light green	yes	–	*p*- , *q*-Values

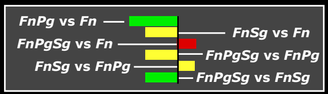

Supplement: Table S1 — The overall results for the entire study, relative abundance ratios presented with a minimum of analytical detail. [file mbo30003-0729-sd3.pdf]
