# Supplementary material for: Proteomics of Fusobacterium nucleatum within a model developing oral microbial community
Source: Microbiologyopen. 2014 Aug 25;3(5):729–51. doi: 10.1002/mbo3.204 (PMC4234264; doi:10.1002/mbo3.204)
Supplement: Table S2 — Details for the qualitative coverage observed for each protein identified in this study. [file mbo30003-0729-sd4.pdf]

Fn Coverage

Fusobacterium nucleatum

Hackett Laboratory | UW

Fn Summary Table | FnPg vs Fn | FnSg vs Fn | FnPgSg vs Fn | FnPgSg vs FnPg | FnSg vs FnPg | FnPgSg vs FnSg | Fn Coverage

ORF	*Fn* n	*Fn* coverage	*Fn* prob	*FnPg* n	*FnPg* coverage	*FnPg* prob	*FnSg* n	*FnSg* coverage	*FnSg* prob	*FnPgSg* n	*FnPgSg* coverage	*FnPgSg* prob	Description
FN0001	8	12.4%	1.000	7	7.7%	1.000	6	11.5%	1.000	6	12.2%	1.000	AAL94214.1| Chromosomal replication initiator protein dnaA
	9	14.0%	1.000	3	5.8%	1.000	9	12.9%	1.000	3	6.9%	0.998	
FN0004	5	34.1%	1.000	2	11.2%	1.000	4	22.0%	1.000	3	18.0%	1.000	AAL94217.1| Inner membrane protein
	3	18.0%	1.000	4	22.0%	1.000	3	15.1%	1.000	3	18.0%	1.000	
FN0005	6	30.7%	1.000	4	25.8%	1.000	3	19.6%	1.000	5	30.1%	1.000	AAL94218.1| Jag protein
	7	30.7%	1.000	4	25.2%	1.000	6	34.4%	1.000	8	34.4%	1.000	
FN0006	6	14.9%	1.000	2	5.1%	0.999	10	30.5%	1.000	6	20.9%	1.000	AAL94219.1| Thiophene and furan oxidation protein THDF
	7	21.8%	1.000	9	26.6%	1.000	11	32.7%	1.000	5	13.2%	1.000	
FN0007	10	18.9%	1.000	5	7.0%	1.000	4	10.5%	1.000	6	14.2%	1.000	AAL94220.1| Glucose inhibited division protein A
	11	26.8%	1.000	6	13.4%	1.000	9	17.4%	1.000	3	7.6%	0.999	
FN0008	0	0.0%		0	0.0%	0.000	3	16.1%	1.000	0	0.0%		AAL94221.1| Quinolinate synthetase A
	0	0.0%		3	17.4%	1.000	3	17.4%	1.000	0	0.0%		
FN0009	0	0.0%		0	0.0%	0.000	2	5.1%	0.997	2	11.5%	0.707	AAL94222.1| L-aspartate oxidase
	0	0.0%		16	34.7%	1.000	14	32.6%	1.000	0	0.0%	0.000	
FN0017	4	8.4%	1.000	0	0.0%	0.000	3	6.3%	0.999	0	0.0%	0.000	AAL94230.1| Hypothetical protein
	4	8.7%	1.000	1	3.1%	0.753	4	13.5%	1.000	1	5.1%	0.320	
FN0018	8	47.4%	1.000	2	9.6%	0.743	5	36.5%	1.000	8	55.1%	1.000	AAL94231.1| Hypothetical protein
	10	48.1%	1.000	5	34.0%	1.000	6	35.9%	1.000	7	42.9%	1.000	
FN0019	2	2.0%	0.455	2	3.5%	1.000	5	5.9%	0.997	0	0.0%	0.000	AAL94232.1| Transcription-repair coupling factor
	3	3.5%	0.869	2	2.0%	0.762	3	3.1%	0.993	4	6.2%	0.910	
FN0021	1	7.8%	0.981	0	0.0%	0.000	1	3.1%	0.796	1	5.4%	0.539	AAL94234.1| 4-diphosphocytidyl -2-C-methyl-D-erythritol kinase
	3	16.0%	1.000	2	8.5%	0.994	2	5.4%	0.996	1	3.1%	0.986	
FN0022	8	64.5%	1.000	8	48.4%	1.000	3	17.2%	1.000	6	46.2%	1.000	AAL94235.1| Hypothetical protein
	7	48.4%	1.000	3	33.3%	1.000	5	34.4%	1.000	6	46.2%	1.000	
FN0023	1	3.0%	0.977	1	4.0%	0.984	1	2.0%	0.977	1	2.0%	0.966	AAL94236.1| Short-chain fatty acids transporter
	2	7.4%	0.812	3	7.8%	1.000	3	8.0%	0.999	3	9.8%	1.000	
FN0024	14	45.6%	1.000	3	19.7%	1.000	2	14.5%	0.994	9	39.5%	1.000	AAL94237.1| Hypothetical exported 24-amino acid repeat protein
	13	43.0%	1.000	8	46.1%	1.000	5	31.6%	1.000	7	36.0%	1.000	
FN0025	6	33.1%	1.000	1	17.5%	0.271	0	0.0%	0.000	2	19.3%	0.997	AAL94238.1| Hypothetical exported 24-amino acid repeat protein
	6	35.5%	1.000	3	21.7%	1.000	3	20.5%	0.994	3	23.5%	0.999	
FN0026	3	20.0%	1.000	0	0.0%	0.000	0	0.0%	0.000	4	20.0%	1.000	AAL94239.1| Hypothetical exported 24-amino acid repeat protein
	4	25.2%	1.000	2	14.8%	1.000	1	6.5%	0.917	3	14.8%	1.000	

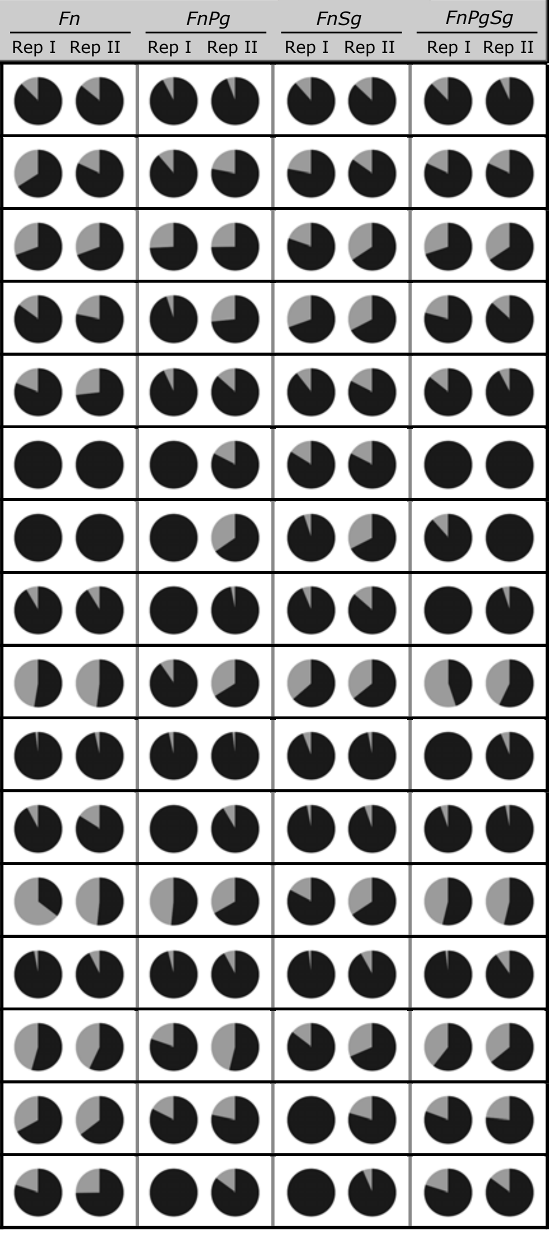

- Show detected proteins only
- Show all proteins
- Filter by category: GO: amino acid transport

Proteins found: 1424

Enter (or paste) list of ORFs

Find ORFs

Dot Plots | Dot Plots

Fn Coverage

Fusobacterium nucleatum

Hackett Laboratory | UW

Fn Summary Table | FnPg vs Fn | FnSg vs Fn | FnPgSg vs Fn | FnPgSg vs FnPg | FnSg vs FnPg | FnPgSg vs FnSg | Fn Coverage

ORF	*Fn* n	*Fn* coverage	*Fn* prob	*FnPg* n	*FnPg* coverage	*FnPg* prob	*FnSg* n	*FnSg* coverage	*FnSg* prob	*FnPgSg* n	*FnPgSg* coverage	*FnPgSg* prob	Description
FN0029	4	32.2%	1.000	1	9.8%	0.981	1	9.8%	0.985	3	21.0%	0.998	AAL94242.1| Flavodoxin
	5	49.7%	1.000	2	16.1%	0.999	2	16.1%	1.000	2	16.1%	0.999	
FN0030	13	63.9%	1.000	10	65.2%	1.000	12	67.7%	1.000	12	60.1%	1.000	AAL94243.1| 5-nitroimidazole antibiotic resistance protein
	13	63.9%	1.000	20	77.2%	1.000	16	67.7%	1.000	15	77.8%	1.000	
FN0031	6	26.2%	1.000	8	34.3%	1.000	7	29.9%	1.000	4	15.1%	1.000	AAL94244.1| unknown
	3	12.9%	1.000	9	30.6%	1.000	8	26.9%	1.000	6	28.0%	1.000	
FN0033	31	19.5%	1.000	26	17.1%	1.000	28	18.2%	1.000	26	17.8%	1.000	AAL94246.1| unknown
	29	19.2%	1.000	26	19.1%	1.000	34	24.3%	1.000	21	14.1%	1.000	
FN0034	8	56.0%	1.000	6	46.8%	1.000	8	48.6%	1.000	8	56.0%	1.000	AAL94247.1| unknown
	6	45.4%	1.000	8	42.1%	1.000	7	37.5%	1.000	9	56.5%	1.000	
FN0038	1	9.0%	0.975	5	32.0%	1.000	0	0.0%	0.000	1	9.0%	0.956	AAL94251.1| unknown
	0	0.0%	0.000	0	0.0%	0.000	1	9.0%	0.978	1	9.0%	0.975	
FN0039	4	25.1%	1.000	1	10.9%	0.984	4	25.7%	1.000	2	16.4%	1.000	AAL94252.1| DNA primase (bacterial type) and small primase-like proteins
	3	16.4%	1.000	5	28.4%	1.000	6	34.4%	1.000	2	16.4%	1.000	
FN0040	44	65.7%	1.000	33	60.1%	1.000	44	65.9%	1.000	45	63.8%	1.000	AAL94253.1| Asparaginyl-tRNA synthetase
	44	57.7%	1.000	42	67.2%	1.000	43	69.2%	1.000	43	64.4%	1.000	
FN0041	5	37.1%	1.000	2	25.8%	0.998	1	19.4%	0.974	3	30.6%	0.999	AAL94254.1| unknown
	5	37.1%	1.000	3	33.9%	1.000	0	0.0%	0.000	3	33.9%	1.000	
FN0043	7	34.2%	1.000	0	0.0%	0.000	1	10.1%	0.985	3	25.3%	1.000	AAL94256.1| Hypothetical exported 24-amino acid repeat protein
	4	24.1%	1.000	4	27.8%	1.000	2	8.2%	0.999	1	6.3%	0.438	
FN0045	5	34.1%	1.000	1	5.2%	0.622	2	11.6%	0.996	1	6.4%	0.982	AAL94258.1| Shikimate 5-dehydrogenase
	3	15.7%	1.000	2	11.2%	0.999	3	25.3%	1.000	2	13.7%	0.977	
FN0046	3	17.7%	1.000	4	42.9%	1.000	1	10.2%	0.985	2	21.1%	0.993	AAL94259.1| 3-dehydroquinate dehydratase
	3	30.6%	1.000	1	10.2%	0.972	2	16.3%	0.997	3	36.7%	0.995	
FN0047	15	36.4%	1.000	14	39.5%	1.000	17	60.5%	1.000	15	51.8%	1.000	AAL94260.1| Exodeoxyribonuclease III
	14	39.5%	1.000	18	55.7%	1.000	19	59.7%	1.000	13	46.2%	1.000	
FN0048	9	27.4%	1.000	4	10.3%	1.000	9	19.8%	1.000	5	19.4%	1.000	AAL94261.1| 4-nitrophenylphosphatase
	9	25.4%	1.000	9	32.1%	1.000	11	36.9%	1.000	7	19.4%	1.000	
FN0049	2	9.3%	0.985	4	35.2%	1.000	5	56.2%	1.000	0	0.0%	0.000	AAL94262.1| Hypothetical protein
	3	29.0%	1.000	7	51.9%	1.000	9	56.2%	1.000	2	9.3%	0.994	
FN0050	40	55.9%	1.000	27	45.0%	1.000	30	55.9%	1.000	37	59.9%	1.000	AAL94263.1| Fumarate reductase flavoprotein subunit
	39	62.5%	1.000	38	59.3%	1.000	29	53.2%	1.000	41	57.7%	1.000	

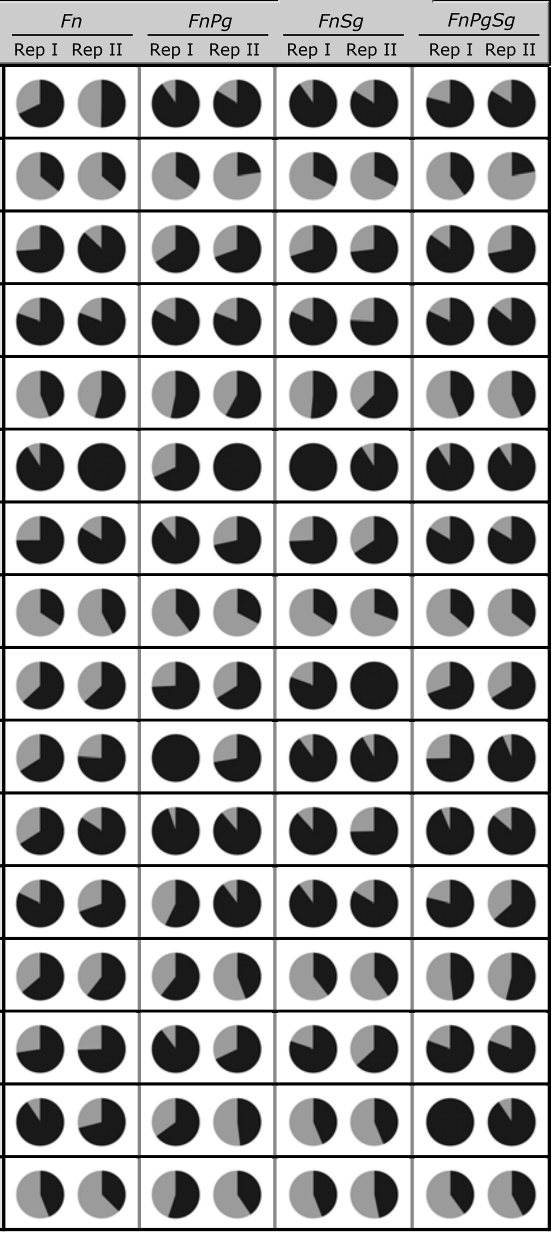

Show detected proteins only
Show all proteins
Filter by category:
GO: amino acid transport

Proteins found: 1424

Enter (or paste) list of ORFs

Find ORFs

Dot Plots | Dot Plots

ORF	*Fn* n	*Fn* coverage	*Fn* prob	*FnPg* n	*FnPg* coverage	*FnPg* prob	*FnSg* n	*FnSg* coverage	*FnSg* prob	*FnPgSg* n	*FnPgSg* coverage	*FnPgSg* prob	Description
**FN0052**	10	60.8%	1.000	4	29.2%	1.000	0	0.0%	0.000	5	36.7%	1.000	AAL94265.1| Arsenate reductase
	11	60.8%	1.000	2	18.3%	1.000	1	8.3%	0.984	8	49.2%	1.000	
**FN0054**	30	45.1%	1.000	25	50.2%	1.000	26	51.5%	1.000	30	56.4%	1.000	AAL94267.1| Tyrosyl-tRNA synthetase
	33	49.0%	1.000	32	55.9%	1.000	28	51.5%	1.000	30	51.5%	1.000	
**FN0058**	23	45.6%	1.000	15	46.3%	1.000	22	52.1%	1.000	26	64.0%	1.000	AAL94271.1| Cysteine desulfhydrase
	19	39.5%	1.000	25	64.2%	1.000	33	75.1%	1.000	19	55.7%	1.000	
**FN0059**	8	64.1%	1.000	4	48.4%	1.000	8	56.2%	1.000	7	63.3%	1.000	AAL94272.1| NifU protein
	6	72.7%	1.000	6	56.2%	1.000	10	72.7%	1.000	6	56.2%	1.000	
**FN0060**	9	25.3%	1.000	3	8.7%	0.999	0	0.0%	0.000	8	23.9%	1.000	AAL94273.1| D-alanyl-D-alanine carboxypeptidase
	8	20.7%	1.000	6	22.3%	1.000	3	11.1%	1.000	6	19.0%	1.000	
**FN0061**	26	46.0%	1.000	13	22.8%	1.000	24	38.3%	1.000	24	42.7%	1.000	AAL94274.1| Thermostable carboxypeptidase 1
	27	46.0%	1.000	28	44.0%	1.000	23	33.5%	1.000	21	38.5%	1.000	
**FN0062**	4	25.0%	1.000	1	11.5%	0.345	0	0.0%		4	25.0%	1.000	AAL94275.1| Hypothetical cytosolic protein
	4	27.1%	1.000	4	27.1%	1.000	0	0.0%		4	25.0%	1.000	
**FN0063**	0	0.0%		0	0.0%	0.000	1	10.0%	0.984	0	0.0%		AAL94276.1| unknown
	0	0.0%		1	8.2%	0.939	3	13.6%	1.000	0	0.0%		
**FN0065**	35	43.0%	1.000	19	28.4%	1.000	23	30.2%	1.000	29	38.5%	1.000	AAL94278.1| Transcription accessory protein (S1 RNA binding domain)
	36	44.5%	1.000	30	39.4%	1.000	32	44.7%	1.000	29	36.5%	1.000	
**FN0066**	0	0.0%		0	0.0%	0.000	2	3.4%	0.643	1	2.7%	0.744	AAL94279.1| Two component system histidine kinase
	0	0.0%		2	3.1%	0.995	3	3.4%	1.000	2	2.6%	0.935	
**FN0067**	53	54.4%	1.000	46	50.9%	1.000	43	48.3%	1.000	51	53.8%	1.000	AAL94280.1| Isoleucyl-tRNA synthetase
	56	56.8%	1.000	52	53.7%	1.000	47	52.6%	1.000	53	45.8%	1.000	
**FN0069**	24	60.3%	1.000	18	50.3%	1.000	25	60.3%	1.000	27	59.0%	1.000	AAL94282.1| Glycyl-tRNA synthetase alpha chain
	23	57.2%	1.000	29	60.3%	1.000	27	60.3%	1.000	25	51.0%	1.000	
**FN0070**	55	50.7%	1.000	43	48.3%	1.000	54	53.5%	1.000	50	53.9%	1.000	AAL94283.1| Glycyl-tRNA synthetase beta chain
	55	50.1%	1.000	62	64.0%	1.000	54	62.4%	1.000	47	50.4%	1.000	
**FN0071**	2	8.6%	0.995	0	0.0%	0.000	3	14.4%	0.997	2	7.5%	0.621	AAL94284.1| GTP cyclohydrolase I
	3	14.4%	1.000	4	21.4%	1.000	2	10.7%	0.994	0	0.0%	0.000	
**FN0072**	10	47.4%	1.000	7	23.4%	1.000	16	52.2%	1.000	11	39.8%	1.000	AAL94285.1| 2-amino-4-hydroxy -6-hydroxymethyldihydropteridine pyrophosphokinase
	9	37.6%	1.000	16	52.2%	1.000	16	52.2%	1.000	10	38.7%	1.000	
**FN0073**	11	47.7%	1.000	5	29.6%	1.000	7	32.1%	1.000	7	30.0%	1.000	AAL94286.1| Dihydropteroate synthase
	10	45.8%	1.000	10	44.0%	1.000	9	41.5%	1.000	9	40.4%	1.000	

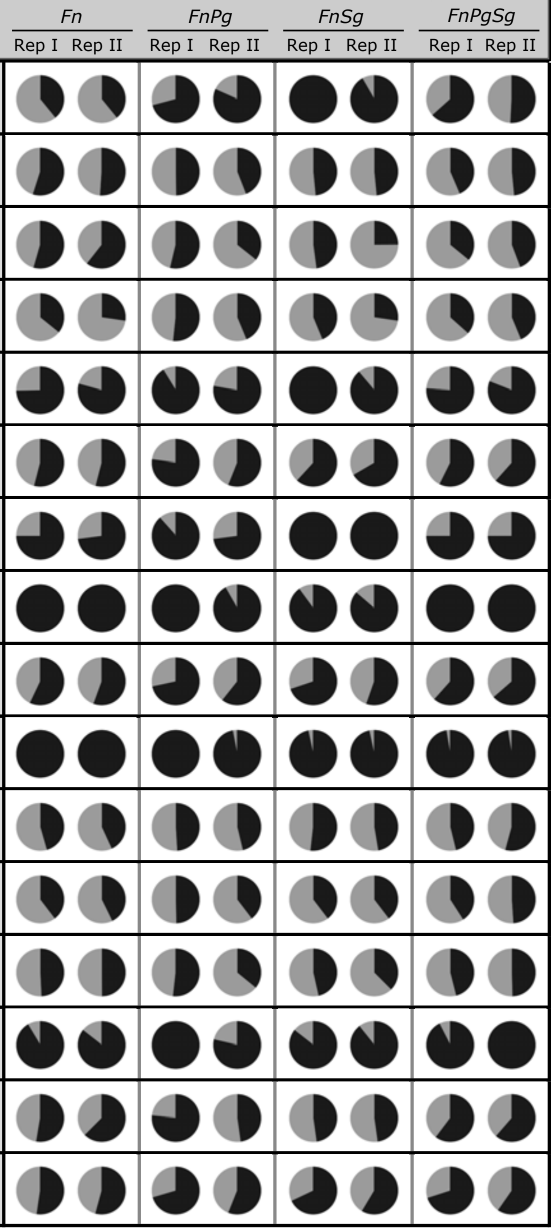

Show detected proteins only
Show all proteins
Filter by category:
GO: amino acid transport

Proteins found: 1424

Enter (or paste) list of ORFs

Find ORFs

Dot Plots Dot Plots

Fn Coverage

Fusobacterium nucleatum

Hackett Laboratory UW

ORF	*Fn* n	*Fn* coverage	*Fn* prob	*FnPg* n	*FnPg* coverage	*FnPg* prob	*FnSg* n	*FnSg* coverage	*FnSg* prob	*FnPgSg* n	*FnPgSg* coverage	*FnPgSg* prob	Description
FN0074	5	41.8%	1.000	2	34.4%	0.990	3	30.3%	0.998	5	40.2%	1.000	AAL94287.1| Ethanolamine utilization protein eutS
	5	41.8%	1.000	7	57.4%	1.000	4	30.3%	1.000	2	10.7%	0.992	
FN0076	1	5.7%	0.395	0	0.0%	0.000	0	0.0%	0.000	0	0.0%		AAL94289.1| Ethanolamine two-component response regulator
	0	0.0%	0.000	3	37.5%	1.000	1	5.7%	0.985	0	0.0%		
FN0077	1	3.9%	0.267	1	3.9%	0.265	1	3.9%	0.347	1	3.9%	0.395	AAL94290.1| Ethanolamine two-component sensor kinase
	4	10.2%	1.000	3	12.1%	0.850	6	17.7%	1.000	0	0.0%	0.000	
FN0078	2	7.8%	0.981	11	25.8%	1.000	11	35.3%	1.000	4	11.6%	0.997	AAL94291.1| Ethanolamine utilization protein eutA
	3	11.6%	1.000	11	31.1%	1.000	9	26.7%	1.000	3	8.6%	0.895	
FN0079	13	33.6%	1.000	11	34.5%	1.000	26	60.7%	1.000	15	44.4%	1.000	AAL94292.1| Ethanolamine ammonia-lyase heavy chain
	12	31.6%	1.000	29	55.4%	1.000	22	53.6%	1.000	8	28.8%	1.000	
FN0080	20	45.1%	1.000	16	29.8%	1.000	20	43.4%	1.000	14	38.3%	1.000	AAL94293.1| Ethanolamine ammonia-lyase light chain
	16	36.6%	1.000	23	46.4%	1.000	18	40.3%	1.000	16	44.1%	1.000	
FN0081	10	72.4%	1.000	5	41.9%	1.000	10	71.9%	1.000	7	52.5%	1.000	AAL94294.1| Ethanolamine utilization protein eutL
	10	72.4%	1.000	13	88.9%	1.000	14	85.7%	1.000	10	72.4%	1.000	
FN0082	2	23.0%	0.999	2	23.0%	1.000	6	37.8%	1.000	6	50.0%	1.000	AAL94295.1| Ethanolamine utilization protein eutM
	4	32.4%	1.000	5	37.2%	1.000	6	40.5%	1.000	6	46.6%	1.000	
FN0083	4	53.2%	1.000	4	39.4%	1.000	5	66.0%	1.000	7	75.5%	1.000	AAL94296.1| Ethanolamine utilization protein eutM precursor
	3	53.2%	1.000	5	62.8%	1.000	4	53.2%	1.000	6	66.0%	1.000	
FN0084	13	39.2%	1.000	10	31.7%	1.000	10	31.2%	1.000	12	30.6%	1.000	AAL94297.1| Acetaldehyde dehydrogenase (acetylating)
	11	34.8%	1.000	15	34.0%	1.000	17	55.2%	1.000	8	18.5%	1.000	
FN0086	2	12.8%	0.696	4	28.2%	1.000	1	9.2%	0.845	0	0.0%	0.000	AAL94299.1| Hypothetical protein
	1	4.1%	0.428	2	9.2%	0.922	2	13.3%	0.915	1	7.2%	0.679	
FN0087	2	32.9%	0.999	3	51.2%	1.000	2	39.0%	0.994	2	39.0%	0.996	AAL94300.1| Ethanolamine utilization protein eutN
	2	28.0%	0.968	2	32.9%	0.990	3	57.3%	1.000	2	35.4%	0.688	
FN0088	2	21.6%	0.999	2	10.8%	0.992	2	17.1%	0.786	0	0.0%	0.000	AAL94301.1| Hypothetical protein
	3	21.6%	0.999	2	18.9%	0.989	3	25.2%	1.000	2	8.1%	0.889	
FN0089	1	7.2%	0.363	1	7.2%	0.917	1	7.2%	0.665	3	20.8%	0.916	AAL94302.1| Ethanolamine permease
	1	7.2%	0.329	1	7.2%	0.953	1	7.2%	0.985	2	13.3%	0.845	
FN0090	1	8.7%	0.481	4	31.5%	1.000	3	28.2%	1.000	1	10.7%	0.983	AAL94303.1| Ethanolamine utilization protein eutQ
	2	19.5%	0.992	3	25.5%	1.000	3	24.8%	1.000	2	17.4%	0.643	
FN0091	2	5.5%	0.990	3	10.1%	1.000	1	3.8%	0.826	2	5.7%	0.694	AAL94304.1| Phosphoserine phosphatase
	2	6.3%	0.927	2	9.3%	0.991	2	6.6%	0.996	2	6.8%	0.988	

Fn Rep I Rep II | *FnPg* Rep I Rep II | *FnSg* Rep I Rep II | *FnPgSg* Rep I Rep II

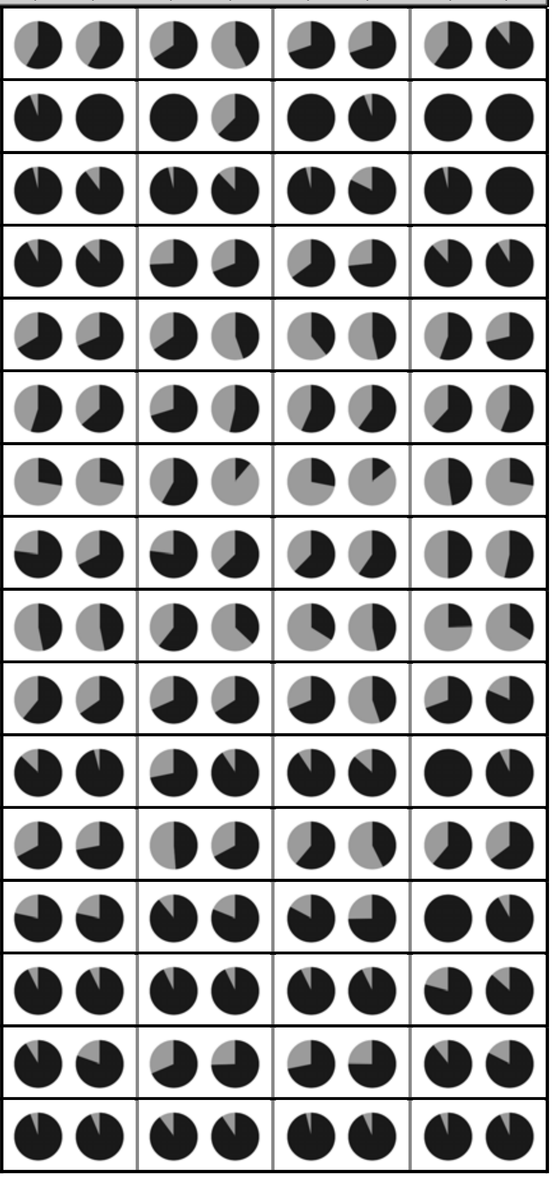

Show detected proteins only
Show all proteins
Filter by category:
GO: amino acid transport

Proteins found:
1424

Enter (or paste) list of ORFs

Find ORFs

Dot Plots Dot Plots

Fn Coverage

Fusobacterium nucleatum

Hackett Laboratory UW

Fn Summary Table | FnPg vs Fn | FnSg vs Fn | FnPgSg vs Fn | FnPgSg vs FnPg | FnSg vs FnPg | FnPgSg vs FnSg | Fn Coverage

ORF	*Fn* n	*Fn* coverage	*Fn* prob	*FnPg* n	*FnPg* coverage	*FnPg* prob	*FnSg* n	*FnSg* coverage	*FnSg* prob	*FnPgSg* n	*FnPgSg* coverage	*FnPgSg* prob	Description
**FN0092**	5	15.9%	1.000	6	22.8%	1.000	13	47.3%	1.000	3	15.6%	1.000	AAL94305.1| NADPH-dependent butanol dehydrogenase
	6	18.5%	1.000	17	53.5%	1.000	13	40.3%	1.000	7	27.2%	1.000	
**FN0093**	10	77.7%	1.000	11	69.9%	1.000	5	58.3%	1.000	10	67.0%	1.000	AAL94306.1| Thioredoxin
	9	73.8%	1.000	10	80.6%	1.000	6	58.3%	1.000	9	73.8%	1.000	
**FN0100**	9	41.9%	1.000	5	32.1%	1.000	5	24.2%	1.000	13	54.9%	1.000	AAL94309.1| Flavodoxins/hemoproteins
	11	45.1%	1.000	8	35.3%	1.000	7	39.5%	1.000	8	38.6%	1.000	
**FN0102**	7	11.9%	1.000	1	2.6%	0.932	1	2.6%	0.985	3	6.8%	0.994	AAL94311.1| Ribonucleoside-diphosphate reductase alpha chain
	7	11.8%	1.000	1	1.3%	0.551	2	5.7%	0.997	6	11.3%	1.000	
**FN0103**	4	15.5%	1.000	1	4.9%	0.685	0	0.0%	0.000	4	16.4%	1.000	AAL94312.1| Ribonucleoside-diphosphate reductase beta chain
	3	12.4%	0.996	0	0.0%	0.000	1	4.0%	0.306	1	2.6%	0.922	
**FN0106**	12	65.0%	1.000	9	55.7%	1.000	11	65.0%	1.000	9	65.0%	1.000	AAL94315.1| Hypothetical protein
	11	65.0%	1.000	11	65.0%	1.000	10	65.0%	1.000	10	65.0%	1.000	
**FN0108**	4	24.9%	1.000	5	30.2%	1.000	9	42.9%	1.000	5	24.9%	1.000	AAL94317.1| Microcin C7 self-immunity protein mccF
	5	19.5%	1.000	9	45.3%	1.000	10	37.0%	1.000	2	14.2%	0.975	
**FN0110**	38	60.4%	1.000	39	62.3%	1.000	40	74.3%	1.000	40	64.9%	1.000	AAL94319.1| Seryl-tRNA synthetase
	39	60.1%	1.000	43	71.2%	1.000	42	73.1%	1.000	48	72.4%	1.000	
**FN0111**	2	45.1%	0.999	1	19.8%	0.290	0	0.0%		0	0.0%	0.000	AAL94320.1| unknown
	2	45.1%	0.999	3	60.4%	0.997	0	0.0%		1	12.1%	0.514	
**FN0113**	10	34.5%	1.000	10	29.1%	1.000	24	56.1%	1.000	8	28.8%	1.000	AAL94322.1| Heat-inducible transcription repressor hrcA
	10	30.2%	1.000	19	44.7%	1.000	23	50.4%	1.000	10	41.0%	1.000	
**FN0114**	17	40.2%	1.000	15	51.8%	1.000	11	34.2%	1.000	12	34.7%	1.000	AAL94323.1| GrpE protein
	18	40.2%	1.000	13	39.2%	1.000	16	58.8%	1.000	13	34.7%	1.000	
**FN0116**	51	61.3%	1.000	36	55.7%	1.000	54	69.7%	1.000	54	65.7%	1.000	AAL94325.1| Chaperone protein dnaK
	55	64.3%	1.000	66	75.3%	1.000	64	72.0%	1.000	59	71.5%	1.000	
**FN0117**	2	14.1%	0.987	1	6.5%	0.726	7	32.9%	1.000	0	0.0%		AAL94326.1| O6-methylguanine-DNA methyltransferase
	4	12.9%	1.000	5	25.9%	1.000	12	47.6%	1.000	0	0.0%		
**FN0118**	18	39.5%	1.000	7	12.5%	1.000	10	18.4%	1.000	13	25.0%	1.000	AAL94327.1| Chaperone protein dnaJ
	20	41.1%	1.000	16	37.8%	1.000	11	32.4%	1.000	15	28.8%	1.000	
**FN0119**	4	26.2%	1.000	2	17.7%	0.997	5	41.5%	1.000	2	21.3%	0.999	AAL94328.1| Flavodoxin
	4	26.2%	1.000	9	64.6%	1.000	13	68.3%	1.000	2	15.2%	0.988	
**FN0123**	5	9.9%	1.000	1	4.8%	0.970	3	11.5%	1.000	2	4.8%	0.823	AAL94332.1| ATPase
	5	12.3%	1.000	5	11.9%	1.000	5	13.7%	1.000	1	1.8%	0.343	

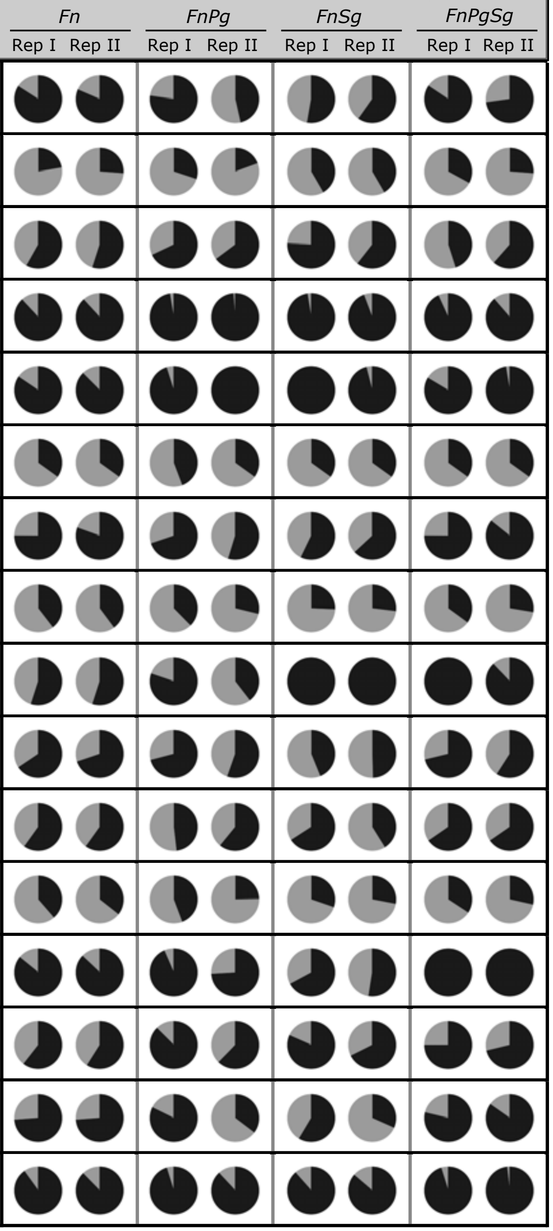

Show detected proteins only
Show all proteins
Filter by category:
GO: amino acid transport

Proteins found: 1424

Enter (or paste) list of ORFs

Find ORFs

Dot Plots Dot Plots

ORF	*Fn* n	*Fn* coverage	*Fn* prob	*FnPg* n	*FnPg* coverage	*FnPg* prob	*FnSg* n	*FnSg* coverage	*FnSg* prob	*FnPgSg* n	*FnPgSg* coverage	*FnPgSg* prob	Description
FN0127	1	3.2%	0.977	3	19.7%	0.999	4	23.6%	1.000	2	8.8%	0.994	AAL94333.1| Fe-S oxidoreductase
	1	3.2%	0.964	1	4.9%	0.983	4	21.8%	1.000	2	6.7%	0.991	
FN0128	8	31.8%	1.000	8	30.3%	1.000	3	16.9%	0.999	6	22.6%	1.000	AAL94334.1| Spermidine/putrescine-binding protein
	7	28.0%	1.000	7	29.6%	1.000	11	36.6%	1.000	5	19.1%	1.000	
FN0129	3	21.6%	0.990	1	10.8%	0.303	1	4.8%	0.465	1	3.9%	0.471	AAL94335.1| Urease accessory protein ureG
	2	11.3%	0.896	0	0.0%	0.000	2	12.6%	0.912	2	11.3%	0.998	
FN0130	5	26.5%	1.000	1	6.0%	0.981	4	22.8%	1.000	4	16.4%	1.000	AAL94336.1| ABC transporter ATP-binding protein
	2	12.3%	1.000	3	19.0%	1.000	11	39.6%	1.000	5	26.5%	1.000	
FN0132	0	0.0%	0.000	3	2.0%	0.000	0	0.0%	0.000	12	6.9%	0.000	AAL93916.1| Hemolysin
	5	2.5%	0.000	8	4.8%	0.258	6	2.8%	0.000	4	2.0%	0.000	
FN0147	12	37.7%	1.000	7	25.0%	1.000	11	43.4%	1.000	7	32.2%	1.000	AAL94353.1| PLSX protein
	12	39.8%	1.000	8	34.3%	1.000	9	34.3%	1.000	7	31.9%	1.000	
FN0148	17	57.9%	1.000	13	54.0%	1.000	18	65.5%	1.000	17	61.6%	1.000	AAL94354.1| 3-oxoacyl-[acyl-carrier-protein] synthase III
	17	54.9%	1.000	17	58.5%	1.000	15	59.5%	1.000	16	59.5%	1.000	
FN0149	23	65.9%	1.000	18	56.2%	1.000	18	64.9%	1.000	27	74.6%	1.000	AAL94355.1| Malonyl-CoA-[acyl-carrier-protein] transacylase
	19	65.2%	1.000	21	70.2%	1.000	15	60.9%	1.000	26	74.6%	1.000	
FN0150	6	21.3%	1.000	4	21.3%	1.000	3	21.3%	1.000	6	21.3%	1.000	AAL94356.1| Acyl carrier protein
	4	21.3%	1.000	3	18.7%	1.000	3	18.7%	1.000	6	21.3%	1.000	
FN0151	27	72.6%	1.000	21	65.1%	1.000	28	80.4%	1.000	29	79.4%	1.000	AAL94357.1| 3-oxoacyl-[acyl-carrier-protein] synthase
	27	72.6%	1.000	34	80.4%	1.000	26	78.0%	1.000	29	68.3%	1.000	
FN0152	3	17.5%	0.988	2	10.3%	0.851	2	10.3%	0.999	2	6.8%	0.998	AAL94358.1| Ribonuclease III
	3	10.3%	0.999	4	17.1%	1.000	3	13.7%	1.000	4	14.1%	1.000	
FN0153	0	0.0%	0.000	0	0.0%	0.000	1	2.3%	0.832	0	0.0%		AAL94359.1| Oxygen-independent coproporphyrinogen III oxidase
	1	2.6%	0.325	1	2.3%	0.852	4	23.3%	1.000	0	0.0%		
FN0154	3	9.6%	0.768	1	3.7%	0.452	2	5.2%	0.760	2	3.5%	0.669	AAL94360.1| Ribonuclease G
	2	7.2%	0.832	1	3.7%	0.983	2	5.7%	0.956	3	7.6%	0.738	
FN0155	6	16.5%	1.000	1	4.8%	0.941	0	0.0%	0.000	8	21.8%	1.000	AAL94361.1| Hypothetical protein
	1	2.4%	0.760	0	0.0%	0.000	5	13.6%	1.000	3	7.5%	0.998	
FN0156	5	36.2%	1.000	4	33.1%	1.000	3	20.2%	0.997	7	48.5%	1.000	AAL94362.1| Phosphopantetheine adenylyltransferase
	5	29.4%	1.000	7	41.1%	1.000	6	31.9%	1.000	5	33.1%	1.000	
FN0157	5	11.7%	1.000	2	5.3%	0.975	5	10.0%	1.000	2	8.4%	0.830	AAL94363.1| DNA repair protein RadA
	4	8.6%	1.000	1	1.8%	0.786	5	11.9%	1.000	1	1.8%	0.489	

Coverage columns: *Fn*, *FnPg*, *FnSg*, *FnPgSg* — Rep I, Rep II

Show detected proteins only
Show all proteins
Filter by category:
GO: amino acid transport

Proteins found:
1424

Enter (or paste) list of ORFs

Find ORFs

Dot Plots Dot Plots

ORF	*Fn* n	*Fn* coverage	*Fn* prob	*FnPg* n	*FnPg* coverage	*FnPg* prob	*FnSg* n	*FnSg* coverage	*FnSg* prob	*FnPgSg* n	*FnPgSg* coverage	*FnPgSg* prob	Description
**FN0158**	9	35.0%	1.000	6	20.3%	1.000	11	40.1%	1.000	8	29.8%	1.000	AAL94364.1| DNA-binding protein
	12	42.4%	1.000	7	26.9%	1.000	12	42.1%	1.000	7	26.1%	1.000	
**FN0161**	1	3.4%	0.969	0	0.0%	0.000	1	4.3%	0.305	1	2.3%	0.535	AAL94367.1| RNA-directed DNA polymerase
	0	0.0%	0.000	0	0.0%	0.000	3	4.6%	0.863	1	3.4%	0.485	
**FN0164**	22	67.4%	1.000	19	63.2%	1.000	24	66.3%	1.000	22	61.1%	1.000	AAL94370.1| Anhydro-N-acetylmuramyl-tripeptide amidase
	24	67.4%	1.000	23	66.0%	1.000	22	66.0%	1.000	21	62.8%	1.000	
**FN0170**	17	41.8%	1.000	14	38.2%	1.000	36	80.5%	1.000	27	61.6%	1.000	AAL94376.1| GTP-binding protein
	22	49.8%	1.000	32	65.0%	1.000	28	56.6%	1.000	22	52.5%	1.000	
**FN0173**	11	27.3%	1.000	5	15.8%	1.000	19	32.5%	1.000	13	28.9%	1.000	AAL94379.1| Hypothetical protein
	11	18.9%	1.000	16	36.4%	1.000	12	23.6%	1.000	9	18.9%	1.000	
**FN0174**	11	41.5%	1.000	8	35.2%	1.000	12	45.3%	1.000	15	44.0%	1.000	AAL94380.1| Enoyl-[acyl-carrier-protein] reductase
	14	48.7%	1.000	15	50.6%	1.000	14	50.9%	1.000	16	44.0%	1.000	
**FN0175**	5	25.9%	1.000	5	25.5%	1.000	6	42.1%	1.000	2	16.7%	1.000	AAL94381.1| Cell division inhibitor MinC
	4	25.9%	1.000	6	38.4%	1.000	9	51.4%	1.000	3	16.7%	1.000	
**FN0176**	19	75.4%	1.000	14	44.7%	1.000	20	61.4%	1.000	19	69.7%	1.000	AAL94382.1| Cell division inhibitor MinD
	20	68.2%	1.000	22	72.7%	1.000	21	77.7%	1.000	19	63.6%	1.000	
**FN0177**	5	49.5%	1.000	3	27.3%	1.000	1	11.1%	0.985	6	49.5%	1.000	AAL94383.1| Cell division inhibitor MinE
	4	42.4%	1.000	0	0.0%	0.000	1	17.2%	0.433	5	49.5%	1.000	
**FN0178**	12	48.8%	1.000	7	35.9%	1.000	8	36.8%	1.000	12	39.6%	1.000	AAL94384.1| UNC-44 ankyrins
	11	45.1%	1.000	9	35.3%	1.000	7	32.2%	1.000	11	39.9%	1.000	
**FN0179**	9	27.2%	1.000	12	37.0%	1.000	8	29.1%	1.000	10	30.2%	1.000	AAL94385.1| Ankyrin repeat proteins
	8	22.5%	1.000	10	30.2%	1.000	12	37.5%	1.000	6	19.9%	1.000	
**FN0180**	2	19.0%	0.998	2	27.8%	0.999	3	40.5%	0.994	3	32.9%	1.000	AAL94386.1| Tetratricopeptide repeat family protein
	2	19.0%	0.981	2	33.5%	0.988	4	45.6%	1.000	2	12.0%	0.999	
**FN0181**	5	51.8%	1.000	3	40.4%	1.000	6	45.6%	1.000	4	31.6%	1.000	AAL94387.1| Hypothetical protein
	5	51.8%	1.000	7	57.0%	1.000	7	57.0%	1.000	5	31.6%	1.000	
**FN0182**	11	26.4%	1.000	10	37.5%	1.000	30	57.0%	1.000	15	48.7%	1.000	AAL94388.1| Sarcosine oxidase alpha subunit
	14	43.0%	1.000	33	58.2%	1.000	30	58.9%	1.000	12	36.3%	1.000	
**FN0183**	28	50.4%	1.000	12	30.3%	1.000	40	67.3%	1.000	26	49.8%	1.000	AAL94389.1| Glycerol-3-phosphate dehydrogenase
	27	47.6%	1.000	42	66.3%	1.000	38	63.5%	1.000	26	55.0%	1.000	
**FN0185**	6	17.8%	1.000	3	9.2%	1.000	5	11.2%	1.000	7	20.8%	1.000	AAL94391.1| Hypothetical protein
	10	24.0%	1.000	9	24.0%	1.000	8	26.8%	1.000	6	16.2%	1.000	

Coverage pie charts per ORF: *Fn*, *FnPg*, *FnSg*, *FnPgSg* — Rep I, Rep II

◉ Show detected proteins only
○ Show all proteins
☐ Filter by category:
GO: amino acid transport

Proteins found:
1424

Enter (or paste) list of ORFs

Find ORFs

Dot Plots | Dot Plots

ORF	Fn n	Fn coverage	Fn prob	FnPg n	FnPg coverage	FnPg prob	FnSg n	FnSg coverage	FnSg prob	FnPgSg n	FnPgSg coverage	FnPgSg prob	Description
FN0188	3	13.4%	1.000	2	10.7%	0.486	2	7.4%	0.979	0	0.0%	0.000	AAL94394.1| Peptide methionine sulfoxide reductase
	2	6.4%	0.987	0	0.0%	0.000	0	0.0%	0.000	1	4.0%	0.953	
FN0189	2	8.4%	0.963	1	6.9%	0.984	8	38.7%	1.000	4	13.0%	1.000	AAL94395.1| Two-component response regulator yesN
	1	5.0%	0.293	7	34.9%	1.000	5	16.9%	1.000	3	7.7%	0.998	
FN0190	2	5.6%	0.998	3	7.6%	1.000	4	10.7%	1.000	1	2.9%	0.418	AAL94396.1| Two-component sensor kinase yesM
	6	13.4%	1.000	3	7.1%	1.000	3	11.2%	1.000	2	5.6%	0.991	
FN0191	4	11.1%	1.000	4	12.4%	1.000	8	19.1%	1.000	5	13.0%	1.000	AAL94397.1| helix-turn-helix DNA-binding protein
	4	10.9%	1.000	9	23.1%	1.000	11	32.7%	1.000	5	13.2%	1.000	
FN0192	20	35.9%	1.000	2	4.3%	0.996	7	20.4%	1.000	14	28.2%	1.000	AAL94398.1| Dipeptide-binding protein
	22	40.2%	1.000	7	13.6%	1.000	14	40.0%	1.000	13	27.2%	1.000	
FN0197	2	7.1%	0.998	1	2.6%	0.943	0	0.0%	0.000	2	7.1%	0.988	AAL94403.1| Methyltransferase
	3	7.1%	0.996	1	4.5%	0.928	3	7.1%	0.998	2	5.6%	0.985	
FN0198	3	7.0%	0.998	2	5.0%	0.850	1	2.9%	0.377	5	8.0%	1.000	AAL94404.1| Transcriptional regulatory protein
	3	5.3%	0.999	2	5.3%	0.606	2	5.2%	0.990	4	6.7%	1.000	
FN0199	5	47.0%	1.000	3	38.0%	1.000	3	38.0%	1.000	7	54.0%	1.000	AAL94405.1| Hypothetical protein
	4	27.0%	1.000	5	51.0%	1.000	6	51.0%	1.000	4	38.0%	1.000	
FN0200	14	58.2%	1.000	11	59.7%	1.000	7	50.7%	1.000	10	47.8%	1.000	AAL94406.1| Biotin carboxyl carrier protein of glutaconyl-COA decarboxylase
	11	58.2%	1.000	9	56.7%	1.000	10	56.7%	1.000	9	54.5%	1.000	
FN0202	36	73.5%	1.000	28	68.8%	1.000	29	81.3%	1.000	34	73.8%	1.000	AAL94408.1| Glutaconate CoA-transferase subunit A
	34	75.4%	1.000	30	72.0%	1.000	25	67.0%	1.000	33	65.1%	1.000	
FN0203	34	86.5%	1.000	31	77.5%	1.000	21	80.9%	1.000	30	84.6%	1.000	AAL94409.1| Glutaconate CoA-transferase subunit B
	33	76.4%	1.000	32	89.9%	1.000	25	82.4%	1.000	33	86.5%	1.000	
FN0204	80	80.3%	1.000	55	72.4%	1.000	57	78.4%	1.000	66	78.3%	1.000	AAL94410.1| Glutaconyl-CoA decarboxylase A subunit
	76	75.3%	1.000	66	83.6%	1.000	59	80.3%	1.000	66	78.3%	1.000	
FN0206	13	51.3%	1.000	8	41.9%	1.000	9	33.6%	1.000	10	41.5%	1.000	AAL94412.1| Activator of (R)-2-hydroxyglutaryl-CoA dehydratase
	14	54.7%	1.000	12	55.8%	1.000	11	46.8%	1.000	7	41.5%	1.000	
FN0207	44	77.8%	1.000	34	65.8%	1.000	43	78.3%	1.000	51	80.5%	1.000	AAL94413.1| (R)-2-hydroxyglutaryl-CoA dehydratase alpha-subunit
	42	80.8%	1.000	46	78.7%	1.000	39	74.9%	1.000	50	81.7%	1.000	
FN0208	26	53.1%	1.000	29	63.4%	1.000	27	62.0%	1.000	28	56.8%	1.000	AAL94414.1| (R)-2-hydroxyglutaryl-CoA dehydratase beta-subunit
	25	55.0%	1.000	30	57.6%	1.000	26	55.0%	1.000	31	58.1%	1.000	
FN0209	22	79.7%	1.000	21	68.0%	1.000	21	80.7%	1.000	22	80.2%	1.000	AAL94415.1| Hypothetical cytosolic protein
	23	79.7%	1.000	25	81.2%	1.000	23	80.7%	1.000	24	80.2%	1.000	

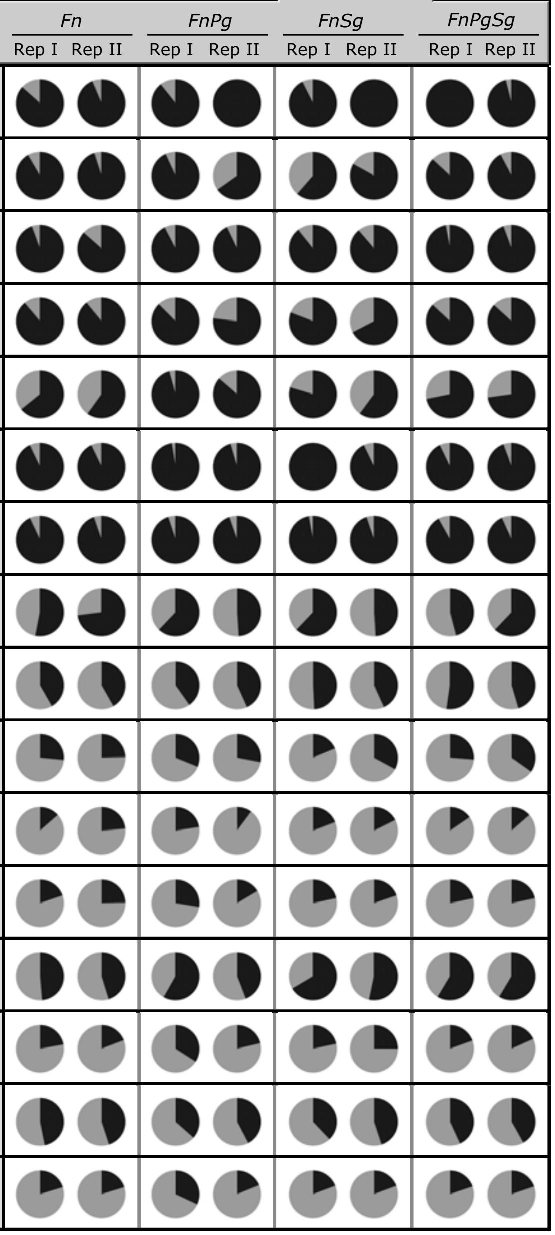

Show detected proteins only
Show all proteins
Filter by category:
GO: amino acid transport

Proteins found: 1424

Enter (or paste) list of ORFs

Find ORFs

Dot Plots Dot Plots

ORF	*Fn* n	*Fn* coverage	*Fn* prob	*FnPg* n	*FnPg* coverage	*FnPg* prob	*FnSg* n	*FnSg* coverage	*FnSg* prob	*FnPgSg* n	*FnPgSg* coverage	*FnPgSg* prob	Description
FN0212	10	42.2%	1.000	7	42.2%	1.000	5	22.2%	1.000	8	41.1%	1.000	AAL94418.1| Hypothetical protein
	6	30.6%	1.000	5	27.2%	1.000	6	27.2%	1.000	7	35.6%	1.000	
FN0214	1	15.8%	0.436	3	35.8%	0.960	0	0.0%	0.000	1	15.8%	0.974	AAL94420.1| Crossover junction endodeoxyribonuclease ruvC
	0	0.0%	0.000	0	0.0%	0.000	1	4.7%	0.287	0	0.0%	0.000	
FN0218	15	46.7%	1.000	13	49.5%	1.000	12	45.3%	1.000	13	41.9%	1.000	AAL94424.1| Anthranilate synthase component II
	14	46.7%	1.000	19	54.3%	1.000	15	58.8%	1.000	11	41.9%	1.000	
FN0219	3	13.3%	0.987	2	5.4%	0.736	1	5.4%	0.981	2	8.3%	0.999	AAL94425.1| Autolysin response regulator
	2	9.6%	0.985	3	15.8%	0.998	6	23.3%	1.000	3	12.5%	1.000	
FN0221	6	12.2%	1.000	9	25.9%	1.000	6	13.5%	1.000	8	17.3%	1.000	AAL94427.1| Carbon starvation protein A
	7	12.2%	1.000	9	16.9%	1.000	6	15.0%	1.000	7	15.8%	1.000	
FN0224	7	10.9%	1.000	9	17.3%	1.000	4	11.6%	1.000	7	11.3%	1.000	AAL94430.1| Excinuclease ABC subunit B
	6	12.5%	1.000	8	17.5%	1.000	12	19.3%	1.000	7	9.8%	1.000	
FN0225	2	8.8%	0.826	2	6.4%	0.928	0	0.0%	0.000	1	2.0%	0.951	AAL94431.1| Gluconate permease
	1	2.0%	0.877	3	10.8%	0.962	3	8.2%	0.987	1	2.0%	0.694	
FN0226	13	58.4%	1.000	12	41.0%	1.000	8	37.7%	1.000	13	44.0%	1.000	AAL94432.1| Pyridoxal phosphate biosynthetic protein pdxA
	12	47.0%	1.000	12	46.1%	1.000	11	49.4%	1.000	10	45.2%	1.000	
FN0227	0	0.0%	0.000	0	0.0%	0.000	2	6.4%	0.995	2	6.4%	0.713	AAL94433.1| Hypothetical protein
	1	1.6%	0.651	4	12.9%	0.987	4	11.1%	1.000	2	6.8%	0.672	
FN0233	14	38.7%	1.000	11	29.9%	1.000	11	25.8%	1.000	16	42.1%	1.000	AAL94439.1| Hypothetical protein
	16	26.7%	1.000	7	22.6%	1.000	10	25.8%	1.000	13	38.7%	1.000	
FN0234	5	36.7%	1.000	2	7.5%	0.991	5	36.7%	1.000	0	0.0%	0.000	AAL94440.1| unknown
	6	37.4%	1.000	4	29.3%	1.000	7	49.0%	1.000	1	7.5%	0.688	
FN0235	2	12.0%	0.987	6	23.6%	1.000	2	9.5%	0.679	0	0.0%	0.000	AAL94441.1| ABC transporter ATP-binding protein
	1	5.4%	0.890	0	0.0%	0.000	0	0.0%	0.000	1	2.9%	0.561	
FN0236	19	55.1%	1.000	29	65.3%	1.000	15	47.6%	1.000	25	65.6%	1.000	AAL94442.1| ABC transporter substrate-binding protein
	21	62.6%	1.000	18	68.9%	1.000	18	56.3%	1.000	20	56.3%	1.000	
FN0237	0	0.0%		3	23.7%	0.999	0	0.0%	0.000	2	11.6%	0.993	AAL94443.1| ABC transporter permease protein
	0	0.0%		0	0.0%	0.000	1	3.3%	0.958	1	6.2%	0.722	
FN0238	8	25.5%	1.000	10	24.8%	1.000	8	27.1%	1.000	6	15.8%	1.000	AAL94444.1| Hypothetical protein
	8	25.5%	1.000	10	29.7%	1.000	9	25.8%	1.000	6	18.1%	1.000	
FN0240	14	38.2%	1.000	11	34.9%	1.000	13	40.0%	1.000	14	44.0%	1.000	AAL94446.1| Thymidylate synthase
	13	30.9%	1.000	19	52.7%	1.000	12	33.8%	1.000	18	47.3%	1.000	

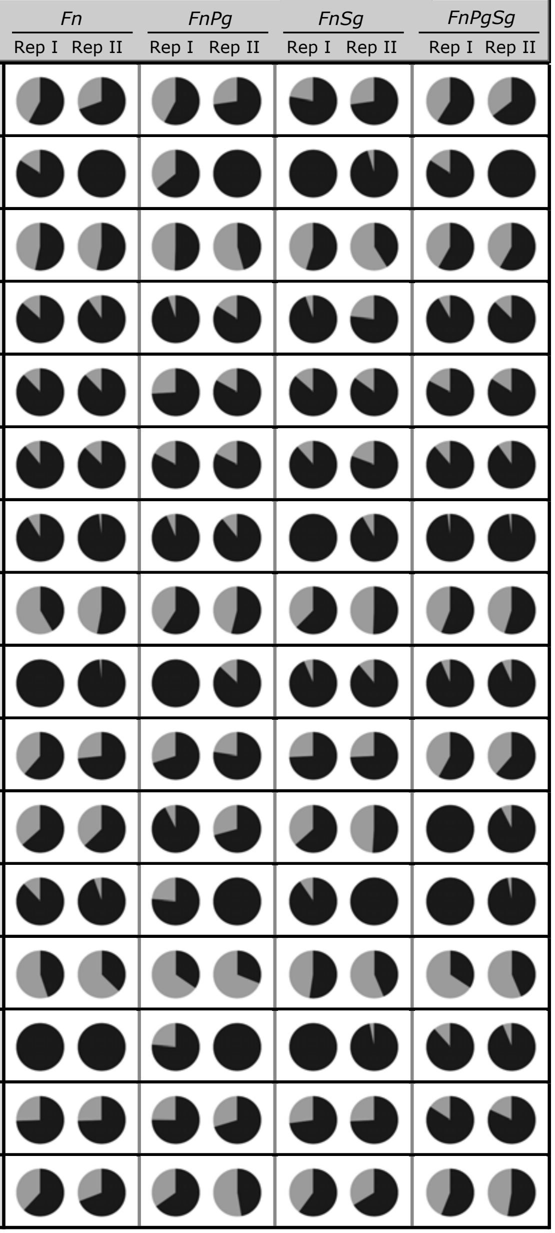

Show detected proteins only
Show all proteins
Filter by category:
GO: amino acid transport

Proteins found:
1424

Enter (or paste) list of ORFs
Find ORFs

Dot Plots Dot Plots

ORF	Fn n	Fn coverage	Fn prob	FnPg n	FnPg coverage	FnPg prob	FnSg n	FnSg coverage	FnSg prob	FnPgSg n	FnPgSg coverage	FnPgSg prob	Description
FN0241	5	36.6%	1.000	3	15.2%	1.000	9	53.0%	1.000	6	42.1%	1.000	AAL94447.1| Dihydrofolate reductase
	5	29.9%	1.000	11	65.9%	1.000	13	59.8%	1.000	4	25.0%	1.000	
FN0242	14	33.4%	1.000	2	5.3%	1.000	19	44.7%	1.000	7	16.8%	1.000	AAL94448.1| Trk system potassium uptake protein trkA
	17	38.3%	1.000	16	40.3%	1.000	22	46.9%	1.000	5	13.5%	1.000	
FN0243	4	13.7%	1.000	1	4.2%	0.984	8	22.4%	1.000	1	2.4%	0.471	AAL94449.1| Poly(A) polymerase
	3	10.9%	1.000	9	25.7%	1.000	17	41.2%	1.000	4	8.9%	1.000	
FN0244	4	53.6%	1.000	2	35.7%	0.999	4	53.6%	0.999	2	50.0%	0.999	AAL94450.1| COP associated protein
	5	53.6%	1.000	3	53.6%	0.999	2	32.1%	0.992	2	50.0%	0.995	
FN0245	7	8.8%	1.000	0	0.0%	0.000	5	7.5%	1.000	4	7.2%	1.000	AAL94451.1| Copper-exporting ATPase
	7	10.1%	1.000	6	8.5%	1.000	9	14.2%	1.000	0	0.0%	0.000	
FN0247	26	79.8%	1.000	11	54.8%	1.000	17	72.6%	1.000	19	63.9%	1.000	AAL94453.1| Hypothetical cytosolic protein
	25	72.2%	1.000	21	77.6%	1.000	20	76.4%	1.000	15	51.3%	1.000	
FN0248	1	4.5%	0.309	1	9.1%	0.564	9	36.7%	1.000	5	16.9%	1.000	AAL94454.1| Hypothetical Exported Protein
	0	0.0%	0.000	11	38.0%	1.000	13	37.3%	1.000	2	7.8%	1.000	
FN0249	14	39.5%		9	39.5%		8	39.5%		14	39.5%		AAL94455.1| unknown
	13	39.5%		9	39.5%		10	39.5%		14	39.5%		
FN0250	13	57.0%		12	54.7%		10	58.7%		11	56.4%		AAL94456.1| unknown
	12	57.0%		10	58.7%		9	54.2%		10	56.4%		
FN0251	11	47.6%		6	42.9%		9	58.7%		12	53.2%		AAL94457.1| Hypothetical membrane-spanning Protein
	10	46.8%		10	58.7%		7	53.2%		12	52.4%		
FN0252	9	54.9%		8	45.1%		7	48.8%		8	54.9%		AAL94458.1| unknown
	8	48.8%		7	54.9%		5	45.1%		7	45.1%		
FN0253	15	70.2%		0	0.0%	1.000	9	70.2%		16	70.2%		AAL94459.1| Outer membrane protein
	13	70.2%		12	70.2%	0.000	9	70.2%		13	52.3%		
FN0254	67	50.7%	1.000	38	32.1%	1.000	59	48.2%	1.000	51	43.6%	1.000	AAL94460.1| Fusobacterium outer membrane protein family
	74	51.5%	1.000	74	56.1%	1.000	61	54.5%	1.000	56	43.3%	1.000	
FN0258	8	14.7%	1.000	3	8.3%	1.000	1	2.8%	0.984	1	3.1%	0.982	AAL94464.1| Zinc-transporting ATPase
	5	9.3%	1.000	4	9.4%	1.000	5	14.3%	1.000	4	10.9%	1.000	
FN0259	2	26.0%	0.999	2	26.0%	0.998	0	0.0%		4	56.2%	1.000	AAL94465.1| Zinc-transporting ATPase
	2	26.0%	1.000	0	0.0%	0.000	0	0.0%		5	57.5%	1.000	
FN0260	2	22.4%	0.979	1	12.8%	0.955	0	0.0%	0.000	1	12.0%	0.982	AAL94466.1| Transcriptional repressor smtB
	3	22.4%	0.995	1	12.0%	0.364	1	9.6%	0.690	1	9.6%	0.655	

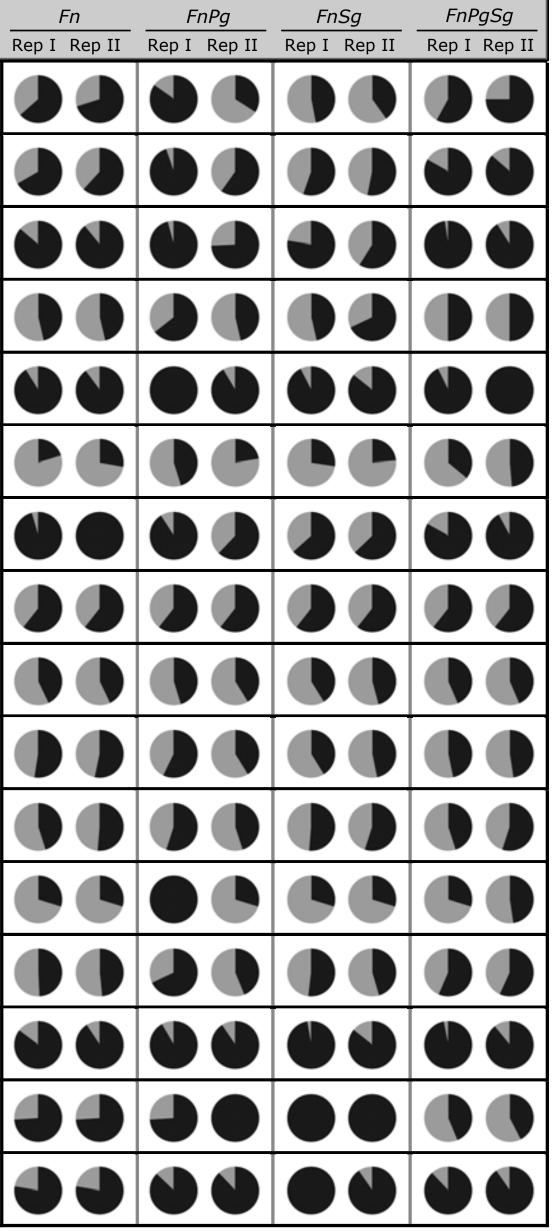

Show detected proteins only
Show all proteins
Filter by category:
GO: amino acid transport

Proteins found: 1424

Enter (or paste) list of ORFs

Find ORFs

Dot Plots Dot Plots

Fn Coverage

Fusobacterium nucleatum

Hackett Laboratory UW

ORF	*Fn* n	*Fn* coverage	*Fn* prob	*FnPg* n	*FnPg* coverage	*FnPg* prob	*FnSg* n	*FnSg* coverage	*FnSg* prob	*FnPgSg* n	*FnPgSg* coverage	*FnPgSg* prob	Description
FN0261	12	37.9%	1.000	4	16.0%	1.000	7	23.5%	1.000	6	25.1%	1.000	AAL94467.1| Pyruvate formate-lyase activating enzyme
	8	28.4%	1.000	2	4.9%	1.000	4	10.7%	1.000	6	31.7%	1.000	
FN0262	98	78.3%	1.000	86	75.4%	1.000	77	82.6%	1.000	103	81.7%	1.000	AAL94468.1| Formate acetyltransferase
	103	80.1%	1.000	74	77.5%	1.000	80	79.7%	1.000	104	81.8%	1.000	
FN0263	22	66.7%	1.000	19	60.6%	1.000	18	63.2%	1.000	25	66.2%	1.000	AAL94469.1| Peptidyl-prolyl cis-trans isomerase
	22	65.4%	1.000	20	62.8%	1.000	17	61.5%	1.000	19	64.1%	1.000	
FN0264	15	74.4%	1.000	9	68.2%	1.000	11	74.4%	1.000	13	72.1%	1.000	AAL94470.1| Hypothetical protein
	14	74.4%	1.000	11	77.5%	1.000	9	40.3%	1.000	12	72.1%	1.000	
FN0265	4	16.9%	1.000	0	0.0%	0.000	4	14.0%	1.000	4	21.4%	0.998	AAL94471.1| Cell division protein ftsX
	6	20.1%	1.000	5	16.9%	1.000	4	22.1%	1.000	2	8.1%	0.995	
FN0266	4	13.2%	1.000	1	2.7%	0.955	2	6.9%	1.000	0	0.0%	0.000	AAL94472.1| membrane protein related to metalloendopeptidase
	4	13.2%	1.000	6	16.6%	0.995	2	2.7%	0.998	3	14.1%	0.876	
FN0267	5	19.1%	1.000	1	9.0%	0.697	0	0.0%	0.000	2	10.1%	0.922	AAL94473.1| ATP-NAD kinase
	6	35.6%	1.000	5	24.0%	1.000	5	21.3%	1.000	1	10.9%	0.340	
FN0268	8	19.7%	1.000	5	17.9%	1.000	2	7.5%	0.985	2	5.9%	0.965	AAL94474.1| DNA repair protein recN
	10	21.0%	1.000	2	4.1%	0.798	11	24.7%	1.000	3	9.9%	0.883	
FN0270	4	12.2%	1.000	0	0.0%	0.000	3	9.8%	1.000	4	12.2%	0.989	AAL94476.1| GTP-binding protein era
	5	12.2%	1.000	2	7.4%	0.999	4	18.2%	1.000	3	12.2%	0.997	
FN0271	4	21.2%	1.000	1	10.2%	0.972	3	8.7%	0.998	4	23.5%	1.000	AAL94477.1| Enoyl-CoA hydratase
	4	21.2%	1.000	7	25.0%	1.000	6	30.3%	1.000	0	0.0%	0.000	
FN0272	2	3.8%	0.955	2	3.8%	1.000	6	24.3%	1.000	2	10.0%	0.937	AAL94478.1| Acetoacetate: butyrate/acetate coenzyme A transferase
	3	16.7%	0.999	3	18.0%	0.974	5	15.9%	1.000	0	0.0%	0.000	
FN0273	1	6.8%	0.467	0	0.0%	0.000	4	17.4%	1.000	1	4.6%	0.989	AAL94479.1| Butyrate-acetoacetate CoA-transferase subunit B
	0	0.0%	0.000	6	32.4%	1.000	6	28.3%	1.000	2	11.4%	0.912	
FN0276	2	2.7%	0.999	2	2.7%	0.999	10	18.5%	1.000	3	8.4%	1.000	AAL94482.1| Sodium-dependent phosphate transporter
	3	5.3%	1.000	9	18.3%	1.000	12	21.7%	1.000	2	5.1%	0.985	
FN0277	6	20.3%	1.000	3	19.0%	1.000	4	22.1%	1.000	4	14.1%	1.000	AAL94483.1| Hypothetical protein
	6	22.4%	1.000	6	24.1%	1.000	7	32.1%	1.000	4	13.8%	1.000	
FN0278	35	71.9%	1.000	30	61.7%	1.000	31	69.7%	1.000	34	70.8%	1.000	AAL94484.1| Xaa-His dipeptidase
	32	64.2%	1.000	39	79.4%	1.000	34	73.0%	1.000	36	74.1%	1.000	
FN0279	8	38.5%	1.000	8	41.5%	1.000	15	71.9%	1.000	7	35.4%	1.000	AAL94485.1| Lipoprotein
	11	55.8%	1.000	16	68.5%	1.000	14	68.5%	1.000	7	35.4%	1.000	

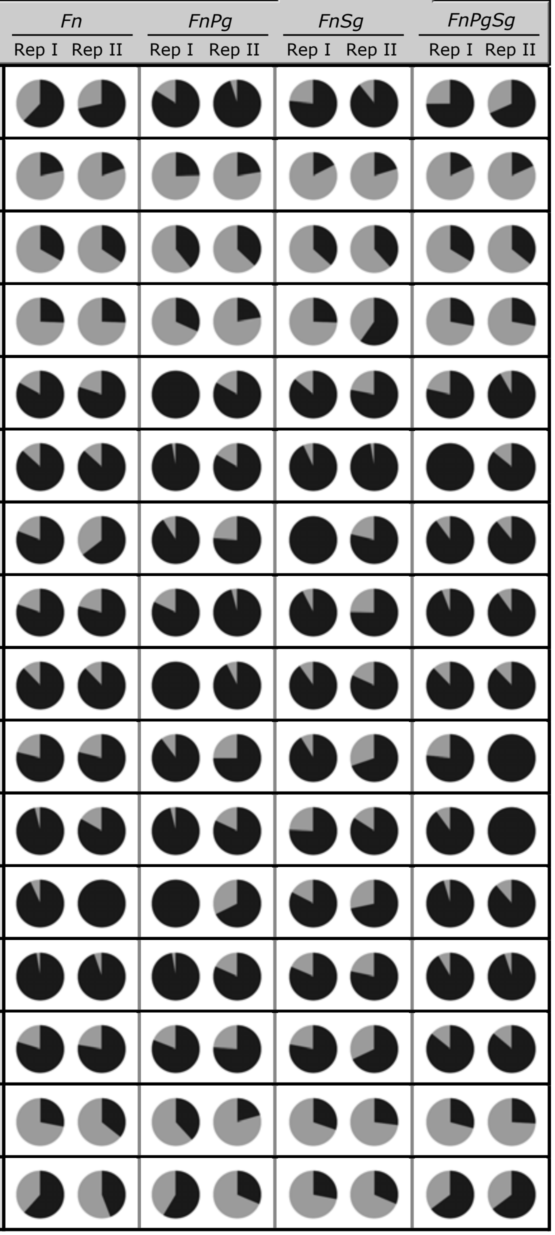

Show detected proteins only
Show all proteins
Filter by category:
GO: amino acid transport

Proteins found: 1424

Enter (or paste) list of ORFs

Find ORFs

Dot Plots Dot Plots

ORF	*Fn* n	*Fn* coverage	*Fn* prob	*FnPg* n	*FnPg* coverage	*FnPg* prob	*FnSg* n	*FnSg* coverage	*FnSg* prob	*FnPgSg* n	*FnPgSg* coverage	*FnPgSg* prob	Description
FN0280	19	40.3%	1.000	10	28.3%	1.000	17	38.6%	1.000	13	36.3%	1.000	AAL94486.1| Hypothetical protein
	18	38.4%	1.000	22	41.2%	1.000	18	36.3%	1.000	13	37.7%	1.000	
FN0281	23	18.9%	1.000	9	8.5%	1.000	33	27.6%	1.000	9	8.5%	1.000	AAL94487.1| DNA polymerase III alpha subunit
	19	15.5%	1.000	30	25.4%	1.000	48	36.5%	1.000	8	8.8%	1.000	
FN0282	11	47.6%	1.000	5	26.2%	1.000	9	38.5%	1.000	9	46.5%	1.000	AAL94488.1| Hypothetical protein
	10	47.6%	1.000	11	54.5%	1.000	12	59.4%	1.000	11	55.1%	1.000	
FN0283	1	8.0%	0.979	1	5.9%	0.829	1	10.5%	0.985	1	4.6%	0.989	AAL94489.1| tRNA (Guanine-N1) -methyltransferase
	3	15.5%	1.000	5	31.9%	1.000	4	31.1%	1.000	3	20.6%	0.999	
FN0284	5	28.3%	1.000	0	0.0%	0.000	10	61.8%	1.000	3	14.5%	1.000	AAL94490.1| 16S rRNA processing protein rimM
	4	28.3%	1.000	8	40.5%	1.000	7	43.9%	1.000	7	38.2%	1.000	
FN0285	3	32.9%	1.000	1	21.5%	0.984	1	19.0%	0.896	7	63.3%	1.000	AAL94491.1| RNA binding protein
	1	11.4%	0.391	5	58.2%	1.000	1	16.5%	0.974	5	46.8%	1.000	
FN0287	4	21.2%	1.000	1	3.0%	0.406	4	22.3%	1.000	5	26.1%	1.000	AAL94493.1| Dimethyladenosine transferase
	5	25.8%	1.000	7	29.2%	1.000	9	50.0%	1.000	4	15.2%	0.998	
FN0288	10	52.0%	1.000	7	36.6%	1.000	9	47.4%	1.000	9	46.3%	1.000	AAL94494.1| Hypoxanthine-guanine phosphoribosyltransferase
	8	40.0%	1.000	12	49.1%	1.000	8	41.7%	1.000	8	33.7%	1.000	
FN0290	0	0.0%	0.000	0	0.0%	0.000	0	0.0%	0.000	1	2.5%	0.465	AAL94496.1| Hemolysin
	1	2.5%	0.278	1	2.5%	0.618	1	2.5%	0.541	3	6.6%	0.904	
FN0291	1	0.8%	0.278	2	1.2%	0.897	0	0.0%	0.000	4	2.9%	0.965	AAL94497.1| Hemolysin
	3	2.9%	0.857	6	5.1%	1.000	4	4.3%	0.980	3	1.9%	0.768	
FN0294	19	73.7%	1.000	14	57.8%	1.000	13	57.0%	1.000	18	75.2%	1.000	AAL94500.1| Transketolase subunit A
	20	75.9%	1.000	20	77.4%	1.000	15	63.7%	1.000	14	68.5%	1.000	
FN0295	11	58.3%	1.000	15	64.7%	1.000	11	60.8%	1.000	12	68.3%	1.000	AAL94501.1| Transketolase
	13	58.6%	1.000	14	63.8%	1.000	16	67.3%	1.000	11	61.2%	1.000	
FN0296	9	32.5%	1.000	9	27.5%	1.000	8	27.1%	1.000	11	34.1%	1.000	AAL94502.1| Hypothetical cytosolic protein
	9	32.9%	1.000	11	36.5%	1.000	9	30.6%	1.000	10	29.8%	1.000	
FN0297	4	14.5%	1.000	3	9.8%	1.000	7	23.6%	1.000	1	4.2%	0.989	AAL94503.1| ATPase associated with chromosome architecture/replication
	6	22.1%	1.000	10	23.3%	1.000	11	28.5%	1.000	4	15.2%	0.999	
FN0298	30	56.9%	1.000	26	43.1%	1.000	38	63.4%	1.000	33	63.9%	1.000	AAL94504.1| Histidyl-tRNA synthetase
	30	60.8%	1.000	46	76.0%	1.000	34	61.3%	1.000	31	51.6%	1.000	
FN0299	39	57.6%	1.000	27	40.2%	1.000	33	54.1%	1.000	38	58.1%	1.000	AAL94505.1| Aspartyl-tRNA synthetase
	39	54.7%	1.000	41	62.7%	1.000	42	66.4%	1.000	40	64.2%	1.000	

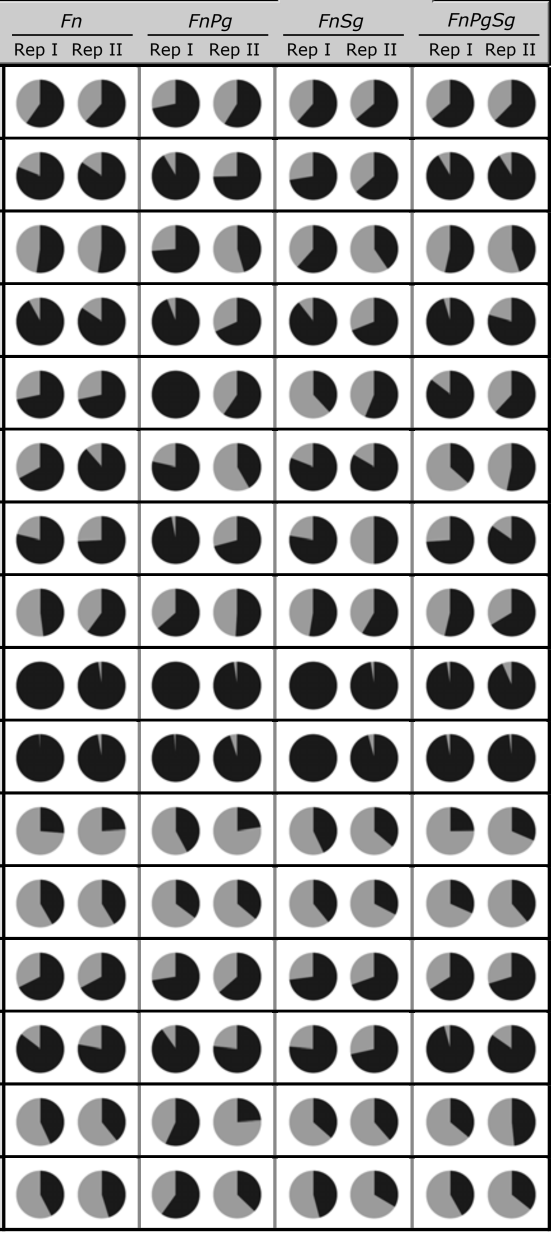

Show detected proteins only
Show all proteins
Filter by category:
GO: amino acid transport

Proteins found: 1424

Enter (or paste) list of ORFs

Find ORFs

Dot Plots Dot Plots

ORF	*Fn* n	*Fn* coverage	*Fn* prob	*FnPg* n	*FnPg* coverage	*FnPg* prob	*FnSg* n	*FnSg* coverage	*FnSg* prob	*FnPgSg* n	*FnPgSg* coverage	*FnPgSg* prob	Description
**FN0305**	1	3.9%	0.848	2	6.2%	0.992	1	3.9%	0.605	3	8.3%	0.996	AAL94511.1| Iron(III) dicitrate-binding protein
	3	10.1%	1.000	1	3.9%	0.983	1	3.9%	0.984	3	15.5%	0.997	
**FN0307**	0	0.0%		0	0.0%		0	0.0%		1	6.4%	0.950	AAL94513.1| Iron(III) dicitrate transport ATP-binding protein fecE
	0	0.0%		0	0.0%		0	0.0%		3	15.9%	0.877	
**FN0308**	32	61.9%	1.000	23	65.9%	1.000	29	69.6%	1.000	32	66.5%	1.000	AAL94514.1| Iron(III)-binding protein
	33	58.5%	1.000	28	69.6%	1.000	31	74.4%	1.000	33	71.3%	1.000	
**FN0309**	2	2.0%	0.999	2	8.0%	0.540	0	0.0%		3	8.2%	1.000	AAL94515.1| Iron(III)-transport system permease protein sfuB
	3	6.6%	1.000	1	4.6%	0.982	0	0.0%		4	10.0%	1.000	
**FN0310**	20	54.5%	1.000	15	46.6%	1.000	14	57.9%	1.000	20	57.9%	1.000	AAL94516.1| Iron(III)-transport ATP-binding protein sfuC
	19	54.5%	1.000	19	65.4%	1.000	14	51.1%	1.000	18	56.2%	1.000	
**FN0311**	28	37.5%	1.000	14	22.7%	1.000	29	39.8%	1.000	29	40.9%	1.000	AAL94517.1| Anaerobic ribonucleoside-triphosphate reductase
	27	37.0%	1.000	29	37.8%	1.000	28	38.7%	1.000	27	36.3%	1.000	
**FN0313**	4	10.3%	1.000	2	5.1%	0.742	8	18.6%	1.000	4	12.4%	1.000	AAL94519.1| 16S rRNA m(5)C 967 methyltransferase
	4	9.7%	1.000	6	14.7%	1.000	7	17.5%	1.000	2	4.1%	0.875	
**FN0314**	1	14.0%	0.804	0	0.0%	0.000	3	17.7%	1.000	2	12.6%	0.999	AAL94520.1| Caffeoyl-CoA O-methyltransferase
	1	7.0%	0.981	4	23.3%	1.000	3	19.1%	1.000	1	5.6%	0.981	
**FN0315**	2	9.3%	0.858	2	3.6%	1.000	3	9.6%	1.000	0	0.0%	0.000	AAL94521.1| Transcriptional regulator, AraC family
	1	3.2%	0.904	6	19.6%	1.000	6	20.6%	1.000	1	3.9%	0.967	
**FN0316**	6	42.4%	1.000	4	27.2%	1.000	3	13.9%	1.000	6	44.4%	1.000	AAL94522.1| Hypothetical protein
	7	41.7%	1.000	1	6.0%	0.983	3	23.2%	1.000	5	39.1%	1.000	
**FN0317**	7	22.8%	1.000	5	33.9%	1.000	12	47.1%	1.000	3	12.4%	1.000	AAL94523.1| Tryptophan synthase beta chain
	5	20.8%	1.000	12	51.6%	1.000	17	53.9%	1.000	5	21.5%	1.000	
**FN0319**	7	25.2%	1.000	2	13.0%	0.987	4	21.2%	1.000	2	7.0%	1.000	AAL94525.1| Citrate (pro-3S)-lyase ligase
	5	14.8%	1.000	3	12.8%	1.000	6	19.1%	1.000	3	17.7%	1.000	
**FN0320**	9	40.0%	1.000	5	32.8%	1.000	4	28.3%	1.000	10	41.7%	1.000	AAL94526.1| Hypothetical cytosolic protein
	8	35.0%	1.000	8	35.6%	1.000	3	17.8%	1.000	10	48.3%	1.000	
**FN0321**	29	47.3%	1.000	21	38.4%	1.000	35	52.2%	1.000	32	49.8%	1.000	AAL94527.1| Heat shock protein htpG
	23	40.0%	1.000	39	54.4%	1.000	41	58.6%	1.000	26	46.0%	1.000	
**FN0322**	39	72.5%	1.000	36	75.9%	1.000	33	82.7%	1.000	37	73.2%	1.000	AAL94528.1| Fructose-bisphosphate aldolase
	40	76.9%	1.000	33	78.0%	1.000	26	76.3%	1.000	37	76.3%	1.000	
**FN0325**	9	32.8%	1.000	3	20.7%	0.999	0	0.0%	0.000	9	32.8%	1.000	AAL94529.1| LSU ribosomal protein L20P
	9	32.8%	1.000	2	19.8%	0.858	3	31.0%	0.998	11	39.7%	1.000	

Fn: Rep I, Rep II — FnPg: Rep I, Rep II — FnSg: Rep I, Rep II — FnPgSg: Rep I, Rep II

◉ Show detected proteins only
○ Show all proteins
☐ Filter by category:
GO: amino acid transport

Proteins found: 1424

Enter (or paste) list of ORFs

Find ORFs

Dot Plots Dot Plots

ORF	*Fn* n	*Fn* coverage	*Fn* prob	*FnPg* n	*FnPg* coverage	*FnPg* prob	*FnSg* n	*FnSg* coverage	*FnSg* prob	*FnPgSg* n	*FnPgSg* coverage	*FnPgSg* prob	Description
FN0326	3	19.1%	0.999	2	19.1%	0.907	0	0.0%	0.000	5	32.4%	1.000	AAL94530.1| LSU ribosomal protein L35P
	2	19.1%	0.988	2	32.4%	0.995	1	14.7%	0.935	5	32.4%	1.000	
FN0327	6	17.6%	1.000	2	11.7%	0.968	3	13.8%	1.000	4	21.3%	1.000	AAL94531.1| Bacterial Protein Translation Initiation Factor 3 (IF -3)
	6	17.6%	1.000	4	17.0%	1.000	5	26.6%	1.000	4	16.5%	1.000	
FN0329	25	75.0%	1.000	22	65.3%	1.000	19	72.9%	1.000	17	65.3%	1.000	AAL94533.1| LSU ribosomal protein L13P
	24	72.2%	1.000	20	75.7%	1.000	19	72.9%	1.000	21	68.8%	1.000	
FN0330	11	51.9%	1.000	5	21.1%	1.000	5	36.1%	1.000	15	61.7%	1.000	AAL94534.1| SSU ribosomal protein S9P
	11	60.2%	1.000	9	51.1%	1.000	6	48.1%	1.000	15	58.6%	1.000	
FN0331	18	52.0%	1.000	6	25.2%	1.000	14	38.9%	1.000	16	54.4%	1.000	AAL94535.1| Hypothetical protein
	22	65.3%	1.000	15	48.3%	1.000	17	68.1%	1.000	13	50.5%	1.000	
FN0332	1	4.8%	0.434	1	2.6%	0.952	5	18.5%	1.000	3	13.7%	0.998	AAL94536.1| Magnesium and cobalt transport protein corA
	2	10.0%	1.000	5	16.8%	1.000	4	14.8%	1.000	0	0.0%	0.000	
FN0333	2	14.5%	0.983	2	9.1%	0.942	4	26.9%	1.000	1	8.6%	0.975	AAL94537.1| Glycerol uptake operon antiterminator regulatory protein
	2	14.5%	0.992	4	26.3%	1.000	3	27.4%	0.999	1	8.6%	0.986	
FN0334	27	61.4%	1.000	18	46.0%	1.000	33	68.4%	1.000	24	53.7%	1.000	AAL94538.1| Aspartate/aromatic aminotransferase
	28	61.2%	1.000	30	69.4%	1.000	31	66.3%	1.000	20	45.3%	1.000	
FN0335	23	81.4%	1.000	22	75.0%	1.000	25	81.4%	1.000	22	78.5%	1.000	AAL94539.1| Outer membrane porin F
	25	81.4%	1.000	22	81.4%	1.000	24	81.4%	1.000	19	77.3%	1.000	
FN0336	14	51.7%	1.000	1	10.4%	0.984	10	44.6%	1.000	12	47.1%	1.000	AAL94540.1| Hypothetical protein
	15	51.7%	1.000	11	45.0%	1.000	10	45.8%	1.000	13	50.8%	1.000	
FN0341	5	10.2%	1.000	2	7.7%	0.999	3	10.2%	0.999	6	12.2%	1.000	AAL94545.1| transport protein
	5	10.2%	1.000	7	18.3%	1.000	4	15.6%	1.000	5	10.9%	1.000	
FN0342	7	58.7%	1.000	5	53.9%	1.000	6	53.9%	1.000	11	83.8%	1.000	AAL94546.1| Peptidyl-prolyl cis-trans isomerase
	7	71.3%	1.000	5	53.9%	1.000	5	44.3%	1.000	11	69.5%	1.000	
FN0343	3	18.3%	0.998	0	0.0%		1	9.4%	0.385	0	0.0%	0.000	AAL94547.1| Hypothetical protein
	1	4.9%	0.942	0	0.0%		1	6.2%	0.498	1	6.2%	0.324	
FN0344	2	7.4%	0.993	2	4.7%	0.989	1	4.7%	0.974	1	2.9%	0.421	AAL94548.1| Methyltransferase
	5	15.3%	1.000	1	3.2%	0.930	3	9.0%	1.000	1	2.4%	0.821	
FN0347	18	43.7%	1.000	6	18.0%	1.000	18	43.7%	1.000	12	32.3%	1.000	AAL94551.1| Phosphatidylserine decarboxylase
	20	44.0%	1.000	23	50.7%	1.000	17	42.3%	1.000	9	29.7%	1.000	
FN0348	34	62.7%	1.000	16	36.9%	1.000	29	56.3%	1.000	29	62.1%	1.000	AAL94552.1| Nicotinate phosphoribosyltransferase
	35	61.7%	1.000	35	60.9%	1.000	27	55.1%	1.000	32	67.1%	1.000	

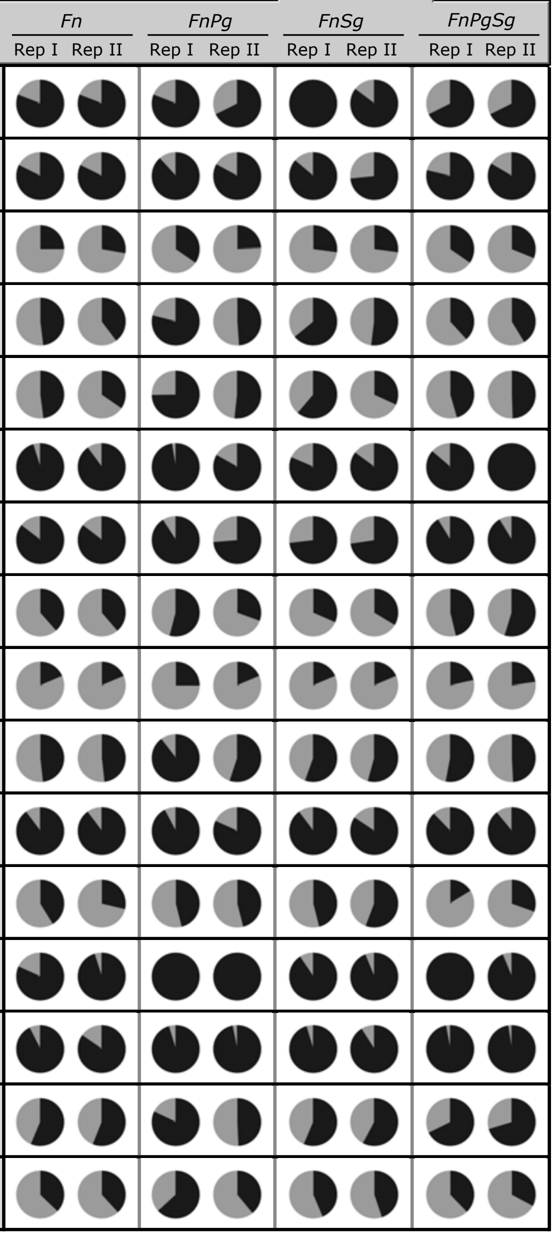

Show detected proteins only
Show all proteins
Filter by category:
GO: amino acid transport

Proteins found: 1424

Enter (or paste) list of ORFs

Find ORFs

Dot Plots Dot Plots

ORF	Fn n	Fn coverage	Fn prob	FnPg n	FnPg coverage	FnPg prob	FnSg n	FnSg coverage	FnSg prob	FnPgSg n	FnPgSg coverage	FnPgSg prob	Description
FN0349	3	30.5%	0.999	4	30.5%	1.000	5	41.6%	1.000	4	31.2%	1.000	AAL94553.1| D-Tyr-tRNATyr deacylase
	3	30.5%	0.998	7	53.2%	1.000	5	51.3%	1.000	2	10.4%	0.981	
FN0351	17	66.7%	1.000	12	53.5%	1.000	7	52.1%	1.000	16	64.6%	1.000	AAL94555.1| unknown
	19	66.7%	1.000	7	54.2%	1.000	7	57.6%	1.000	13	68.8%	1.000	
FN0352	4	5.9%	1.000	3	5.9%	1.000	6	11.1%	1.000	6	12.2%	1.000	AAL94556.1| NA+/H+ antiporter NHAC
	5	5.9%	1.000	7	13.5%	1.000	5	11.1%	1.000	4	12.2%	1.000	
FN0355	26	49.6%	1.000	14	34.2%	1.000	24	43.3%	1.000	26	52.2%	1.000	AAL94558.1| S-adenosylmethionine synthetase
	26	52.0%	1.000	27	47.5%	1.000	26	51.7%	1.000	21	46.0%	1.000	
FN0356	3	19.5%	1.000	4	23.6%	1.000	3	20.3%	1.000	5	38.2%	1.000	AAL94559.1| Lactoylglutathione lyase
	4	30.1%	1.000	4	30.1%	1.000	2	12.2%	1.000	4	27.6%	1.000	
FN0357	5	57.5%	1.000	3	36.6%	0.999	4	36.6%	1.000	4	35.8%	1.000	AAL94560.1| ATP synthase epsilon chain, sodium ion specific
	3	35.8%	1.000	7	46.3%	1.000	6	58.2%	1.000	4	35.8%	1.000	
FN0358	32	74.7%	1.000	29	72.5%	1.000	36	81.4%	1.000	28	70.6%	1.000	AAL94561.1| ATP synthase beta chain, sodium ion specific
	30	71.6%	1.000	33	82.5%	1.000	36	81.6%	1.000	29	70.6%	1.000	
FN0359	15	46.1%	1.000	9	38.3%	1.000	13	54.3%	1.000	10	47.2%	1.000	AAL94562.1| ATP synthase gamma chain, sodium ion specific
	15	51.4%	1.000	13	52.1%	1.000	14	53.9%	1.000	12	44.7%	1.000	
FN0360	22	32.0%	1.000	18	28.6%	1.000	23	31.0%	1.000	25	37.0%	1.000	AAL94563.1| ATP synthase alpha chain, sodium ion specific
	22	31.2%	1.000	26	36.4%	1.000	24	34.6%	1.000	26	39.8%	1.000	
FN0361	11	37.4%	1.000	5	14.9%	1.000	7	27.6%	1.000	9	33.3%	1.000	AAL94564.1| ATP synthase delta chain, sodium ion specific
	8	24.1%	1.000	5	25.9%	1.000	10	39.7%	1.000	6	19.0%	1.000	
FN0362	9	28.2%	1.000	1	7.4%	0.984	6	21.5%	1.000	7	25.8%	1.000	AAL94565.1| ATP synthase B chain, sodium ion specific
	7	33.7%	1.000	6	20.9%	1.000	8	28.2%	1.000	8	26.4%	1.000	
FN0364	1	11.0%	0.709	1	11.0%	0.721	1	11.0%	0.733	3	35.3%	0.992	AAL94567.1| ATP synthase A chain, sodium ion specific
	3	28.4%	0.876	4	40.4%	1.000	2	23.4%	0.998	3	28.4%	1.000	
FN0366	39	62.2%	1.000	20	39.2%	1.000	35	60.2%	1.000	35	62.2%	1.000	AAL94569.1| Phosphoglucosamine mutase
	42	64.4%	1.000	38	63.5%	1.000	28	55.8%	1.000	32	58.2%	1.000	
FN0368	16	30.2%	1.000	7	14.9%	1.000	16	32.7%	1.000	16	30.2%	1.000	AAL94571.1| Adenylosuccinate lyase
	15	30.0%	1.000	29	41.7%	1.000	21	34.0%	1.000	12	21.6%	1.000	
FN0370	13	49.0%	1.000	4	19.9%	1.000	10	39.5%	1.000	9	31.5%	1.000	AAL94573.1| Signal peptidase I
	16	42.3%	1.000	12	40.9%	1.000	8	27.6%	1.000	12	32.2%	1.000	
FN0371	14	36.1%	1.000	6	20.8%	1.000	3	22.4%	1.000	16	45.1%	1.000	AAL94574.1| Hypothetical protein
	14	36.1%	1.000	5	22.7%	1.000	2	14.1%	0.999	14	34.1%	1.000	

Show detected proteins only
Show all proteins
Filter by category:
GO: amino acid transport

Proteins found: 1424

Enter (or paste) list of ORFs

Find ORFs

Dot Plots Dot Plots

ORF	*Fn* n	*Fn* coverage	*Fn* prob	*FnPg* n	*FnPg* coverage	*FnPg* prob	*FnSg* n	*FnSg* coverage	*FnSg* prob	*FnPgSg* n	*FnPgSg* coverage	*FnPgSg* prob	Description
FN0374	3	5.0%	0.956	5	9.5%	1.000	2	4.0%	0.998	2	2.1%	0.999	AAL94577.1| Single-stranded-DNA-specific exonuclease recJ
	4	4.1%	1.000	4	5.9%	1.000	8	9.0%	1.000	2	2.3%	0.748	
FN0375	35	80.4%	1.000	33	73.3%	1.000	34	85.2%	1.000	35	83.2%	1.000	AAL94578.1| Iron(III)-binding protein
	37	79.3%	1.000	33	85.2%	1.000	30	82.4%	1.000	37	83.8%	1.000	
FN0376	12	35.8%	1.000	12	33.4%	1.000	23	53.9%	1.000	10	34.2%	1.000	AAL94579.1| Iron(III)-transport ATP-binding protein sfuC
	12	35.8%	1.000	22	50.4%	1.000	20	43.9%	1.000	13	41.5%	1.000	
FN0377	2	6.5%	0.999	2	9.5%	0.981	0	0.0%	0.000	6	15.6%	1.000	AAL94580.1| Iron(III)-transport system permease protein sfuB
	4	6.5%	1.000	1	4.4%	0.893	4	7.8%	1.000	3	8.4%	0.999	
FN0378	4	10.2%	1.000	1	7.1%	0.405	3	16.4%	0.995	3	12.0%	1.000	AAL94581.1| UDP-glucose 4-epimerase
	4	10.2%	1.000	2	7.1%	0.999	5	17.0%	1.000	4	14.2%	1.000	
FN0379	8	19.7%	1.000	5	13.9%	1.000	1	7.1%	0.805	7	25.1%	1.000	AAL94582.1| Hypothetical protein
	3	13.2%	1.000	4	13.9%	0.900	4	15.6%	1.000	5	21.0%	1.000	
FN0380	8	31.4%	1.000	2	7.9%	0.999	1	3.0%	0.959	2	7.3%	1.000	AAL94583.1| unknown
	3	14.5%	1.000	6	21.8%	1.000	2	7.3%	0.987	5	17.8%	1.000	
FN0381	7	27.3%	1.000	2	7.1%	0.973	1	5.7%	0.343	6	25.5%	1.000	AAL94584.1| unknown
	6	23.0%	1.000	2	8.9%	0.956	0	0.0%	0.000	7	28.7%	1.000	
FN0382	4	12.5%	1.000	0	0.0%	0.000	0	0.0%	0.000	1	4.4%	0.988	AAL94585.1| Hypothetical protein
	5	12.5%	1.000	0	0.0%	0.000	1	4.7%	0.860	0	0.0%	0.000	
FN0383	3	7.2%	1.000	1	2.3%	0.388	1	4.7%	0.391	1	2.3%	0.989	AAL94586.1| Lipopolysaccharide N-acetylglucosaminyltransferase
	4	9.7%	1.000	0	0.0%	0.000	2	4.2%	0.648	1	2.3%	0.982	
FN0384	11	12.8%	1.000	3	4.7%	1.000	3	7.9%	0.910	8	14.5%	1.000	AAL94587.1| Hypothetical protein
	12	16.3%	1.000	0	0.0%	0.000	3	6.5%	1.000	8	12.8%	1.000	
FN0385	1	3.9%	0.737	0	0.0%	0.000	0	0.0%		1	3.9%	0.605	AAL94588.1| Hypothetical protein
	0	0.0%	0.000	3	9.8%	0.999	0	0.0%		0	0.0%	0.000	
FN0386	3	12.6%	1.000	0	0.0%		0	0.0%		1	5.9%	0.939	AAL94589.1| Hypothetical protein
	4	12.6%	1.000	0	0.0%		0	0.0%		0	0.0%	0.000	
FN0387	27	22.6%	1.000	7	8.4%	1.000	5	6.1%	1.000	15	9.2%	1.000	AAL94590.1| Fusobacterium outer membrane protein family
	23	18.4%	1.000	11	9.6%	1.000	20	14.4%	1.000	19	15.6%	1.000	
FN0390	9	52.6%	1.000	8	31.6%	1.000	9	54.1%	1.000	12	54.1%	1.000	AAL94593.1| Hypothetical protein
	8	36.8%	1.000	12	60.9%	1.000	10	60.9%	1.000	11	60.9%	1.000	
FN0391	9	37.5%	1.000	4	22.5%	1.000	9	36.0%	1.000	8	31.8%	1.000	AAL94594.1| Hydrolase (HAD superfamily)
	8	37.5%	1.000	12	32.6%	1.000	10	39.7%	1.000	9	39.3%	1.000	

Fn Rep I Rep II | *FnPg* Rep I Rep II | *FnSg* Rep I Rep II | *FnPgSg* Rep I Rep II

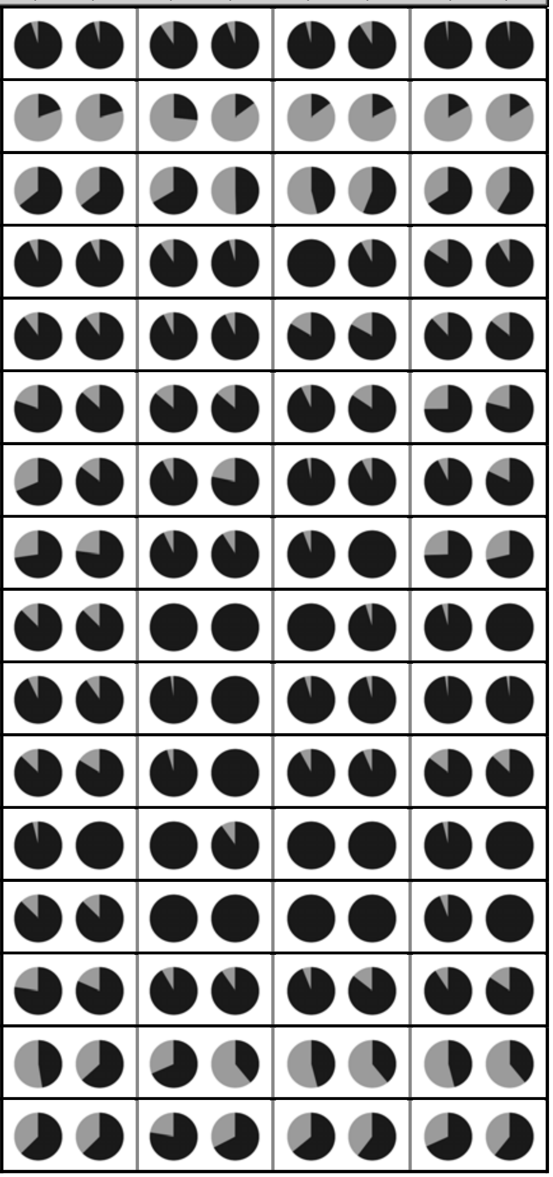

Show detected proteins only
Show all proteins
Filter by category:
GO: amino acid transport

Proteins found: 1424

Enter (or paste) list of ORFs

Find ORFs

Dot Plots Dot Plots

ORF	*Fn* n	*Fn* coverage	*Fn* prob	*FnPg* n	*FnPg* coverage	*FnPg* prob	*FnSg* n	*FnSg* coverage	*FnSg* prob	*FnPgSg* n	*FnPgSg* coverage	*FnPgSg* prob	Description
**FN0392**	9	23.2%	1.000	1	4.4%	0.669	5	23.9%	1.000	5	14.1%	1.000	AAL94595.1| Oxygen-independent coproporphyrinogen III oxidase
	8	21.5%	1.000	4	16.5%	1.000	3	7.1%	0.998	6	12.1%	1.000	
**FN0393**	4	8.5%	1.000	6	18.2%	1.000	14	25.3%	1.000	3	7.2%	0.997	AAL94596.1| Polysaccharide deacetylase
	5	12.9%	1.000	11	23.9%	1.000	14	31.4%	1.000	3	7.7%	0.999	
**FN0394**	7	30.5%	1.000	3	19.5%	0.999	4	22.4%	1.000	3	13.4%	1.000	AAL94597.1| Outer membrane protein
	5	20.7%	1.000	10	39.0%	1.000	2	9.3%	0.861	2	7.7%	0.559	
**FN0396**	58	74.2%	1.000	52	69.5%	1.000	68	83.6%	1.000	57	77.9%	1.000	AAL94599.1| Dipeptide-binding protein
	59	74.2%	1.000	61	79.1%	1.000	60	78.7%	1.000	59	77.7%	1.000	
**FN0397**	6	23.4%	1.000	1	4.2%	0.984	8	26.6%	1.000	6	12.0%	1.000	AAL94600.1| Dipeptide transport system permease protein dppB
	5	12.0%	1.000	5	12.7%	1.000	4	12.0%	1.000	7	14.9%	1.000	
**FN0398**	7	9.3%	1.000	6	8.7%	1.000	8	20.1%	1.000	8	23.9%	1.000	AAL94601.1| Dipeptide transport system permease protein dppC
	7	9.3%	1.000	7	14.2%	1.000	7	8.7%	1.000	7	10.0%	1.000	
**FN0399**	14	45.4%	1.000	17	54.9%	1.000	20	54.9%	1.000	21	64.8%	1.000	AAL94602.1| Dipeptide transport ATP-binding protein dppD
	15	45.4%	1.000	21	63.3%	1.000	21	63.3%	1.000	22	51.0%	1.000	
**FN0400**	34	61.2%	1.000	29	61.9%	1.000	35	73.4%	1.000	31	68.1%	1.000	AAL94603.1| Dipeptide transport ATP-binding protein dppF
	33	57.5%	1.000	38	72.5%	1.000	33	70.9%	1.000	26	60.6%	1.000	
**FN0405**	20	60.6%	1.000	12	42.5%	1.000	11	42.8%	1.000	22	66.8%	1.000	AAL94608.1| Tryptophanyl-tRNA synthetase
	16	45.5%	1.000	17	58.5%	1.000	11	42.5%	1.000	18	55.7%	1.000	
**FN0406**	10	30.2%	1.000	8	27.7%	1.000	7	30.2%	1.000	14	47.7%	1.000	AAL94609.1| Alanine racemase, biosynthetic
	14	36.7%	1.000	8	25.4%	1.000	11	36.7%	1.000	8	27.1%	1.000	
**FN0407**	12	42.5%	1.000	2	21.3%	0.994	7	44.3%	1.000	10	48.3%	1.000	AAL94610.1| Hypothetical protein
	11	42.5%	1.000	6	36.2%	1.000	6	39.7%	1.000	10	44.3%	1.000	
**FN0408**	15	50.7%	1.000	7	24.3%	1.000	13	50.3%	1.000	13	48.4%	1.000	AAL94611.1| Acetyl-coenzyme A carboxylase carboxyl transferase subunit beta
	14	45.4%	1.000	13	46.7%	1.000	12	44.4%	1.000	15	48.7%	1.000	
**FN0409**	32	66.5%	1.000	19	55.6%	1.000	27	77.6%	1.000	29	61.3%	1.000	AAL94612.1| Acetyl-coenzyme A carboxylase carboxyl transferase subunit alpha
	32	70.3%	1.000	33	79.9%	1.000	30	81.2%	1.000	32	70.0%	1.000	
**FN0410**	19	46.5%	1.000	14	44.4%	1.000	15	53.5%	1.000	16	45.9%	1.000	AAL94613.1| 6-phosphofructokinase
	22	54.7%	1.000	20	50.5%	1.000	14	51.7%	1.000	16	44.4%	1.000	
**FN0411**	2	18.4%	0.988	2	18.4%	0.995	3	48.0%	1.000	1	9.2%	0.988	AAL94614.1| putative alpha helix protein
	3	18.4%	0.999	2	18.4%	0.992	2	18.4%	0.999	1	9.2%	0.986	
**FN0412**	2	18.8%	0.977	2	21.8%	0.714	0	0.0%	0.000	1	4.6%	0.580	AAL94615.1| Recombination protein recR
	3	26.4%	0.999	0	0.0%	0.000	1	7.1%	0.343	2	12.2%	0.988	

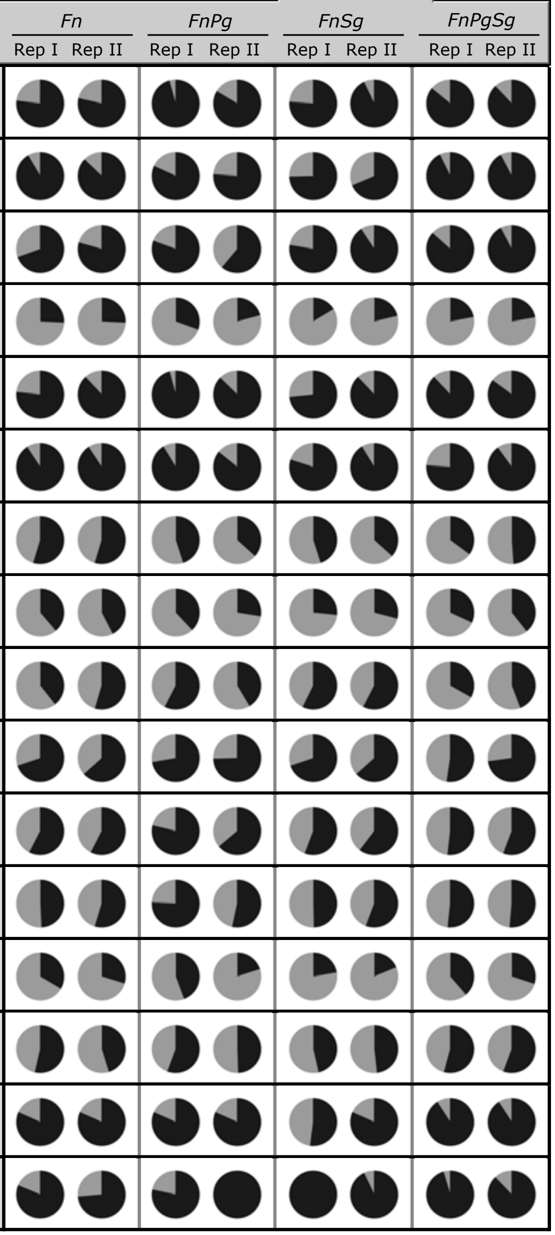

Show detected proteins only

Show all proteins

Filter by category:

GO: amino acid transport

Proteins found: 1424

Enter (or paste) list of ORFs

Find ORFs

Dot Plots Dot Plots

ORF	Fn n	Fn coverage	Fn prob	FnPg n	FnPg coverage	FnPg prob	FnSg n	FnSg coverage	FnSg prob	FnPgSg n	FnPgSg coverage	FnPgSg prob	Description
FN0413	3	17.3%	0.754	3	23.0%	0.999	0	0.0%	0.000	3	14.8%	0.985	AAL94616.1| unknown
	2	9.7%	0.644	3	14.8%	0.910	1	9.7%	0.896	1	5.1%	0.719	
FN0414	0	0.0%	0.000	1	2.1%	0.838	1	1.3%	0.385	3	4.0%	0.905	AAL94617.1| ATP-dependent helicase HEPA
	1	0.9%	0.315	1	2.5%	0.271	3	2.6%	0.872	1	1.8%	0.591	
FN0416	11	20.0%	1.000	7	15.4%	1.000	1	4.0%	0.378	6	14.1%	1.000	AAL94619.1| Type III restriction-modification system methylation subunit
	11	25.0%	1.000	1	2.3%	0.432	1	2.3%	0.539	5	10.3%	1.000	
FN0417	16	19.6%	1.000	5	7.8%	1.000	2	3.9%	0.930	9	12.6%	1.000	AAL94620.1| Type III restriction-modification system restriction subunit
	18	22.8%	1.000	0	0.0%	0.000	5	7.6%	1.000	5	5.9%	1.000	
FN0418	2	18.1%	0.984	2	15.3%	0.986	14	68.4%	1.000	3	16.9%	0.966	AAL94621.1| Uracil phosphoribosyltransferase
	2	16.4%	0.989	13	65.5%	1.000	13	65.5%	1.000	0	0.0%	0.000	
FN0419	4	17.4%	1.000	4	25.0%	1.000	3	14.1%	0.999	3	14.1%	0.989	AAL94622.1| Aspartate carbamoyltransferase
	2	10.9%	0.999	7	31.9%	1.000	6	36.8%	1.000	4	25.0%	1.000	
FN0420	6	15.3%	1.000	3	12.2%	1.000	12	39.1%	1.000	3	5.6%	1.000	AAL94623.1| Dihydroorotase
	5	12.2%	1.000	13	41.6%	1.000	12	38.4%	1.000	2	6.1%	0.957	
FN0421	10	43.9%	1.000	2	8.9%	0.983	11	38.8%	1.000	5	20.7%	1.000	AAL94624.1| Carbamoyl-phosphate synthase small chain
	11	28.8%	1.000	14	53.4%	1.000	8	31.8%	1.000	5	19.6%	1.000	
FN0422	26	30.5%	1.000	8	10.9%	1.000	37	42.1%	1.000	25	31.9%	1.000	AAL94625.1| Carbamoyl-phosphate synthase large chain
	31	41.1%	1.000	33	35.7%	1.000	40	40.7%	1.000	21	29.4%	1.000	
FN0423	4	18.5%	1.000	1	7.7%	0.966	10	38.2%	1.000	4	16.2%	1.000	AAL94626.1| Dihydroorotate dehydrogenase electron transfer subunit
	6	32.8%	1.000	10	34.7%	1.000	11	42.1%	1.000	4	12.0%	1.000	
FN0424	3	11.5%	0.997	3	20.4%	1.000	8	33.9%	1.000	3	17.1%	1.000	AAL94627.1| Dihydroorotate dehydrogenase
	3	11.5%	1.000	7	31.6%	1.000	10	34.9%	1.000	2	12.5%	0.989	
FN0426	5	27.0%	1.000	5	28.7%	1.000	9	50.2%	1.000	9	46.8%	1.000	AAL94629.1| Orotidine 5'-phosphate decarboxylase
	4	20.3%	1.000	16	63.7%	1.000	11	54.9%	1.000	5	24.5%	1.000	
FN0427	7	37.4%	1.000	5	20.9%	1.000	7	32.2%	1.000	6	33.2%	1.000	AAL94630.1| Orotate phosphoribosyltransferase
	9	37.4%	1.000	12	55.5%	1.000	8	33.6%	1.000	8	37.0%	1.000	
FN0430	12	57.8%	1.000	5	37.1%	1.000	8	55.2%	1.000	10	53.4%	1.000	AAL94633.1| LSU ribosomal protein L19P
	12	57.8%	1.000	9	55.2%	1.000	8	57.8%	1.000	9	47.4%	1.000	
FN0435	11	44.0%	1.000	7	46.5%	1.000	11	55.2%	1.000	10	48.5%	1.000	AAL94634.1| Purine nucleoside phosphorylase
	10	44.0%	1.000	19	57.7%	1.000	12	54.8%	1.000	5	28.6%	1.000	
FN0436	7	21.4%	1.000	8	19.0%	1.000	11	28.9%	1.000	5	14.9%	1.000	AAL94635.1| regulator of kinase autophosphorylation inhibitor
	6	21.4%	1.000	12	35.4%	1.000	15	46.7%	1.000	8	21.4%	1.000	

Show detected proteins only
Show all proteins
Filter by category:
GO: amino acid transport

Proteins found: 1424

Enter (or paste) list of ORFs

Find ORFs

Dot Plots Dot Plots

**Fn Coverage**	*Fusobacterium nucleatum*	**Hackett Laboratory** UW

ORF	*Fn* n	*Fn* coverage	*Fn* prob	*FnPg* n	*FnPg* coverage	*FnPg* prob	*FnSg* n	*FnSg* coverage	*FnSg* prob	*FnPgSg* n	*FnPgSg* coverage	*FnPgSg* prob	Description
**FN0437**	3	17.9%	1.000	3	21.8%	1.000	7	42.7%	1.000	3	17.9%	1.000	AAL94636.1| kinase autophosphorylation inhibitor KipI
	3	17.9%	1.000	6	32.4%	1.000	7	42.7%	1.000	4	22.9%	1.000	
**FN0438**	2	12.4%	0.992	0	0.0%	0.000	1	4.3%	0.985	1	4.3%	0.989	AAL94637.1| transporter protein
	3	10.9%	1.000	2	4.3%	1.000	1	4.3%	0.985	2	10.9%	0.995	
**FN0439**	11	49.0%	1.000	5	30.0%	1.000	10	48.2%	1.000	13	58.0%	1.000	AAL94638.1| Lactam utilization protein LAMB
	11	54.1%	1.000	20	70.4%	1.000	14	63.0%	1.000	12	58.0%	1.000	
**FN0445**	4	18.5%	1.000	1	2.7%	0.420	2	8.9%	0.881	5	29.5%	1.000	AAL94641.1| Hypothetical protein
	4	14.4%	1.000	2	9.6%	0.934	4	22.3%	1.000	3	12.3%	0.995	
**FN0446**	8	7.7%	1.000	5	5.2%	1.000	6	7.8%	1.000	8	10.2%	1.000	AAL94642.1| Hypothetical protein
	6	8.2%	1.000	5	6.7%	0.999	9	10.7%	1.000	4	6.9%	1.000	
**FN0447**	4	13.8%	1.000	2	3.9%	0.992	5	18.8%	1.000	3	10.2%	1.000	AAL94643.1| NIFS protein
	3	10.2%	0.995	4	13.1%	1.000	6	12.5%	1.000	2	7.3%	0.993	
**FN0448**	1	2.5%	0.400	0	0.0%	0.000	2	6.7%	0.932	2	5.1%	0.970	AAL94644.1| DNA-repair protein
	1	3.6%	0.384	0	0.0%	0.000	2	5.3%	0.779	3	11.0%	0.673	
**FN0450**	10	16.5%	1.000	8	11.9%	1.000	12	18.8%	1.000	12	24.1%	1.000	AAL94646.1| ABC transporter ATP-binding protein
	16	26.7%	1.000	12	20.6%	1.000	15	23.2%	1.000	8	14.3%	1.000	
**FN0451**	4	20.1%	1.000	2	10.4%	1.000	3	17.1%	1.000	1	6.7%	0.492	AAL94647.1| Hypothetical protein
	4	25.0%	1.000	3	20.1%	0.999	5	28.7%	1.000	3	15.9%	0.998	
**FN0452**	50	54.4%	1.000	43	52.1%	1.000	27	49.9%	1.000	51	62.6%	1.000	AAL94648.1| Glucosamine--fructose-6-phosphate aminotransferase (isomerizing)
	48	56.5%	1.000	28	50.2%	1.000	29	45.5%	1.000	48	59.0%	1.000	
**FN0453**	27	44.3%	1.000	30	48.3%	1.000	33	52.7%	1.000	36	51.5%	1.000	AAL94649.1| Xaa-Pro aminopeptidase
	25	42.0%	1.000	39	54.1%	1.000	38	57.0%	1.000	37	57.0%	1.000	
**FN0454**	21	51.9%	1.000	18	47.3%	1.000	28	60.9%	1.000	19	48.1%	1.000	AAL94650.1| Aldehyde dehydrogenase B
	19	49.3%	1.000	29	64.8%	1.000	30	71.1%	1.000	19	53.0%	1.000	
**FN0455**	10	60.9%	1.000	13	66.5%	1.000	11	62.6%	1.000	12	66.5%	1.000	AAL94651.1| Rubrerythrin
	9	59.2%	1.000	12	66.5%	1.000	9	54.7%	1.000	13	66.5%	1.000	
**FN0456**	14	44.2%	1.000	4	12.9%	1.000	4	15.0%	1.000	11	31.3%	1.000	AAL94652.1| Hypothetical cytosolic protein
	16	40.5%	1.000	8	23.2%	1.000	7	25.3%	1.000	11	28.9%	1.000	
**FN0458**	3	14.2%	0.994	4	16.0%	1.000	3	12.7%	0.964	1	3.3%	0.528	AAL94654.1| Hypothetical Exported Protein
	3	13.8%	1.000	5	22.5%	1.000	5	24.7%	1.000	3	13.1%	0.999	
**FN0459**	4	20.3%	1.000	0	0.0%	0.000	2	7.0%	0.950	1	2.8%	0.343	AAL94655.1| Hypothetical exported 24-amino acid repeat protein
	4	20.3%	1.000	2	8.0%	0.839	2	8.0%	0.994	1	10.5%	0.315	

Fn: Rep I, Rep II · FnPg: Rep I, Rep II · FnSg: Rep I, Rep II · FnPgSg: Rep I, Rep II

Show detected proteins only
Show all proteins
Filter by category:
GO: amino acid transport

Proteins found: 1424

Enter (or paste) list of ORFs

Find ORFs

Dot Plots | Dot Plots

ORF	*Fn* n	*Fn* coverage	*Fn* prob	*FnPg* n	*FnPg* coverage	*FnPg* prob	*FnSg* n	*FnSg* coverage	*FnSg* prob	*FnPgSg* n	*FnPgSg* coverage	*FnPgSg* prob	Description
**FN0460**	12	48.1%	1.000	2	14.6%	0.994	5	20.5%	1.000	9	28.9%	1.000	AAL94656.1| Delta-aminolevulinic acid dehydratase
	12	39.8%	1.000	15	50.6%	1.000	9	31.4%	1.000	9	37.3%	1.000	
**FN0461**	17	59.1%	1.000	19	63.0%	1.000	18	70.2%	1.000	18	65.7%	1.000	AAL94657.1| Probable sigma(54) modulation protein
	21	71.3%	1.000	18	72.9%	1.000	17	75.1%	1.000	19	70.2%	1.000	
**FN0462**	15	26.3%	1.000	10	14.8%	1.000	28	49.8%	1.000	9	20.7%	1.000	AAL94658.1| DNA mismatch repair protein mutL
	15	24.9%	1.000	23	41.7%	1.000	27	52.7%	1.000	7	14.2%	1.000	
**FN0465**	20	41.2%	1.000	15	35.6%	1.000	21	49.5%	1.000	20	44.4%	1.000	AAL94661.1| Hypothetical protein
	22	42.9%	1.000	18	45.9%	1.000	17	45.6%	1.000	20	42.4%	1.000	
**FN0466**	45	51.7%	1.000	45	48.1%	1.000	52	64.1%	1.000	47	55.4%	1.000	AAL94662.1| Lysyl-tRNA synthetase
	42	48.7%	1.000	52	60.6%	1.000	48	59.0%	1.000	49	56.4%	1.000	
**FN0469**	1	10.4%	0.981	0	0.0%	0.000	1	5.0%	0.306	1	4.5%	0.979	AAL94665.1| Copper homeostasis protein cutC
	1	10.4%	0.982	0	0.0%	0.000	3	15.3%	1.000	1	4.5%	0.943	
**FN0470**	17	16.4%	1.000	13	17.6%	1.000	15	16.6%	1.000	18	16.4%	1.000	AAL94666.1| Putative efflux pump component MtrF
	16	13.7%	1.000	14	16.6%	1.000	14	16.6%	1.000	18	16.4%	1.000	
**FN0472**	13	56.3%	1.000	18	61.7%	1.000	14	60.5%	1.000	18	61.7%	1.000	AAL94668.1| Flavodoxin
	16	59.9%	1.000	16	60.5%	1.000	11	56.9%	1.000	15	59.9%	1.000	
**FN0474**	1	1.5%	0.289	1	1.7%	0.654	4	7.8%	0.997	0	0.0%	0.000	AAL94670.1| Acriflavin resistance protein B
	4	5.4%	1.000	2	2.0%	0.521	2	4.5%	0.808	2	4.0%	0.662	
**FN0475**	12	24.8%	1.000	1	3.7%	0.645	10	27.6%	1.000	6	11.7%	1.000	AAL94671.1| MIAB protein
	12	25.5%	1.000	12	25.5%	1.000	10	21.8%	1.000	8	19.8%	1.000	
**FN0476**	16	42.1%	1.000	11	31.7%	1.000	17	40.4%	1.000	16	40.4%	1.000	AAL94672.1| Transcription termination factor rho
	14	39.5%	1.000	23	47.2%	1.000	19	43.3%	1.000	17	43.8%	1.000	
**FN0477**	11	39.3%	1.000	5	20.9%	1.000	7	35.8%	1.000	7	27.1%	1.000	AAL94673.1| Cell wall endopeptidase family M23/M37
	10	39.3%	1.000	5	23.7%	1.000	15	49.2%	1.000	6	27.4%	1.000	
**FN0478**	7	27.4%	1.000	2	10.7%	0.998	9	28.2%	1.000	5	20.1%	1.000	AAL94674.1| GcpE protein
	6	24.6%	1.000	13	40.1%	1.000	13	37.3%	1.000	5	18.4%	1.000	
**FN0479**	1	8.7%	0.942	1	14.8%	0.839	5	37.6%	1.000	1	12.1%	0.768	AAL94675.1| RNA polymerase sigma-E factor
	2	16.1%	0.999	2	16.1%	1.000	7	42.3%	1.000	1	8.7%	0.321	
**FN0480**	0	0.0%		1	18.8%	0.508	0	0.0%	0.000	0	0.0%		AAL94676.1| unknown
	0	0.0%		0	0.0%	0.000	4	59.4%	1.000	0	0.0%		
**FN0481**	3	20.5%	0.906	0	0.0%	0.000	3	21.2%	1.000	2	13.6%	0.993	AAL94677.1| unknown
	3	19.7%	0.999	5	34.1%	1.000	5	27.3%	1.000	1	6.8%	0.961	

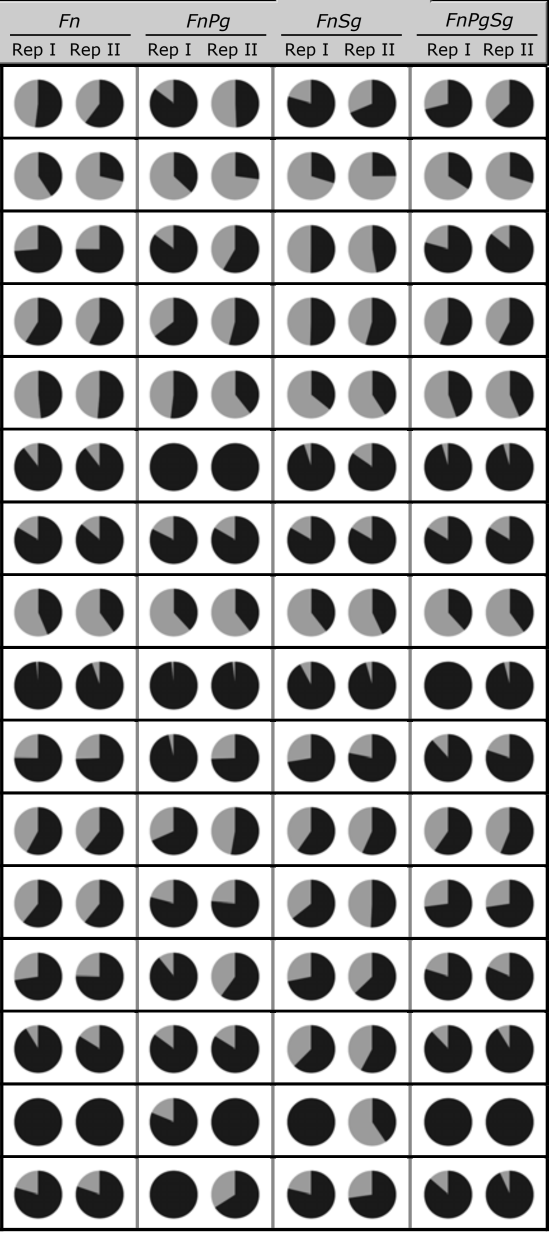

Show detected proteins only
Show all proteins
Filter by category:
GO: amino acid transport

Proteins found: 1424

Enter (or paste) list of ORFs

Find ORFs

Dot Plots Dot Plots

ORF	Fn n	Fn coverage	Fn prob	FnPg n	FnPg coverage	FnPg prob	FnSg n	FnSg coverage	FnSg prob	FnPgSg n	FnPgSg coverage	FnPgSg prob	Description
FN0482	3	48.1%	1.000	0	0.0%	0.000	2	39.5%	0.992	5	39.5%	1.000	AAL94678.1| LSU ribosomal protein L31P
	3	48.1%	1.000	3	51.9%	0.999	1	12.3%	0.985	3	12.3%	1.000	
FN0483	11	51.9%	1.000	9	50.0%	1.000	14	63.1%	1.000	13	57.5%	1.000	AAL94679.1| Uracil phosphoribosyltransferase
	11	51.9%	1.000	22	74.8%	1.000	9	45.8%	1.000	16	61.7%	1.000	
FN0484	4	22.9%	1.000	0	0.0%	0.000	3	12.1%	0.996	3	9.6%	0.999	AAL94680.1| Lipase
	2	7.5%	0.931	3	13.8%	0.998	1	3.8%	0.962	2	3.8%	0.999	
FN0487	52	71.3%	1.000	41	59.8%	1.000	39	70.7%	1.000	50	64.8%	1.000	AAL94683.1| 2-hydroxyglutarate dehydrogenase
	53	69.2%	1.000	40	68.0%	1.000	38	74.0%	1.000	51	63.0%	1.000	
FN0488	56	61.5%	1.000	45	66.7%	1.000	52	72.9%	1.000	49	66.7%	1.000	AAL94684.1| NAD-specific glutamate dehydrogenase
	52	59.5%	1.000	60	81.3%	1.000	56	76.5%	1.000	50	68.3%	1.000	
FN0489	4	17.4%	1.000	1	9.4%	0.984	2	17.0%	1.000	3	17.0%	1.000	AAL94685.1| Prolipoprotein diacylglyceryl transferase
	4	17.4%	1.000	3	17.0%	1.000	3	17.0%	1.000	3	17.0%	1.000	
FN0490	2	3.8%	0.999	0	0.0%	0.000	0	0.0%		0	0.0%	0.000	AAL94686.1| Integral membrane protein
	3	3.8%	1.000	2	13.5%	0.495	0	0.0%		1	3.5%	0.986	
FN0491	14	29.2%	1.000	13	33.4%	1.000	21	56.3%	1.000	13	28.4%	1.000	AAL94687.1| Alanine racemase
	9	19.5%	1.000	23	50.4%	1.000	22	59.9%	1.000	13	33.4%	1.000	
FN0493	6	13.3%	1.000	6	16.5%	1.000	6	16.5%	1.000	4	20.7%	1.000	AAL94689.1| Hypothetical protein
	7	23.6%	1.000	4	11.0%	1.000	5	13.6%	1.000	3	10.7%	0.985	
FN0494	19	56.0%	1.000	9	36.2%	1.000	13	55.6%	1.000	17	53.1%	1.000	AAL94690.1| Short chain dehydrogenase
	16	56.0%	1.000	14	57.6%	1.000	10	53.1%	1.000	17	56.0%	1.000	
FN0495	55	84.1%	1.000	59	85.6%	1.000	56	90.3%	1.000	58	88.8%	1.000	AAL94691.1| Acetyl-CoA acetyltransferase
	56	85.1%	1.000	62	93.5%	1.000	59	92.8%	1.000	58	88.8%	1.000	
FN0496	2	26.0%	0.999	2	37.0%	0.989	0	0.0%		0	0.0%		AAL94692.1| unknown
	4	42.5%	1.000	1	16.4%	0.982	0	0.0%		0	0.0%		
FN0497	1	12.2%	0.483	0	0.0%		0	0.0%	0.000	2	27.8%	0.940	AAL94693.1| Plasmid addiction system poison protein
	1	12.2%	0.860	0	0.0%		1	12.2%	0.665	3	27.8%	0.993	
FN0501	33	47.0%	1.000	31	44.6%	1.000	50	57.0%	1.000	29	42.0%	1.000	AAL94697.1| Ornithine decarboxylase
	35	46.0%	1.000	51	57.1%	1.000	49	61.7%	1.000	29	42.8%	1.000	
FN0502	3	21.6%	0.996	2	18.0%	0.997	2	18.0%	0.999	8	40.7%	1.000	AAL94698.1| Phosphoheptose isomerase
	3	21.6%	1.000	8	52.6%	1.000	4	37.1%	1.000	7	31.4%	1.000	
FN0503	12	41.4%	1.000	6	29.8%	1.000	11	46.4%	1.000	11	41.7%	1.000	AAL94699.1| Transcriptional regulatory protein, LYSR family
	12	38.7%	1.000	10	40.7%	1.000	13	49.0%	1.000	10	39.1%	1.000	

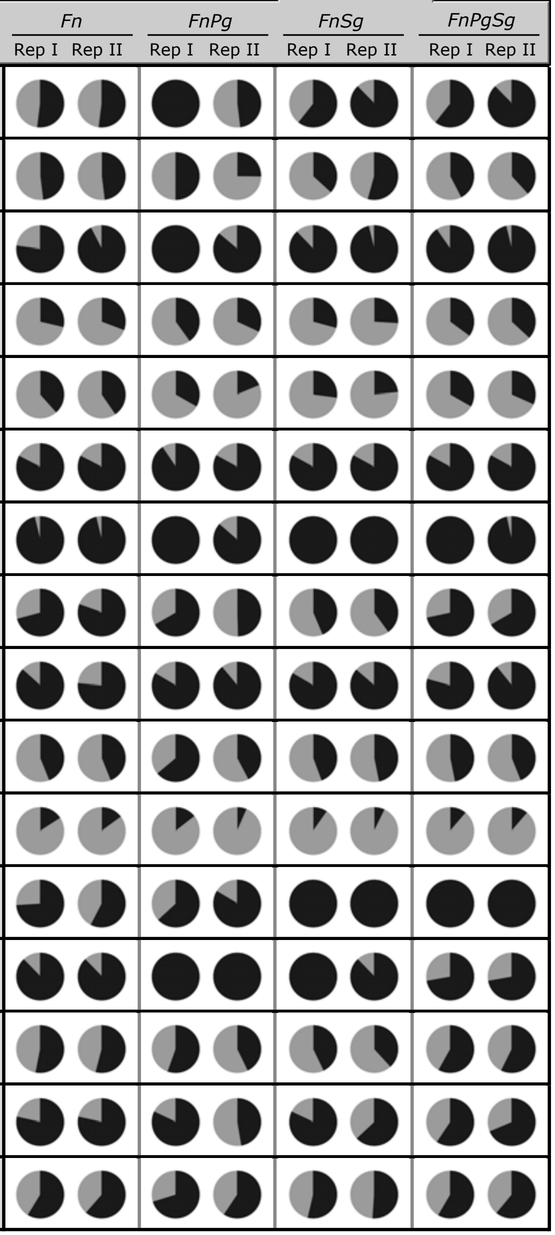

ORF	*Fn* n	*Fn* coverage	*Fn* prob	*FnPg* n	*FnPg* coverage	*FnPg* prob	*FnSg* n	*FnSg* coverage	*FnSg* prob	*FnPgSg* n	*FnPgSg* coverage	*FnPgSg* prob	Description	*Fn* Rep I	*Fn* Rep II	*FnPg* Rep I	*FnPg* Rep II	*FnSg* Rep I	*FnSg* Rep II	*FnPgSg* Rep I	*FnPgSg* Rep II
FN0504	2	8.6%	0.986	1	3.8%	0.436	0	0.0%		1	2.2%	0.989	AAL94700.1| Arginine permease								
	2	5.5%	0.937	1	7.1%	0.819	0	0.0%		3	5.5%	0.900									
FN0505	10	49.0%	1.000	7	30.5%	1.000	4	22.6%	1.000	11	58.0%	1.000	AAL94701.1| Anthranilate synthase component II								
	10	58.0%	1.000	14	64.6%	1.000	10	55.1%	1.000	8	39.9%	1.000									
FN0506	38	57.5%	1.000	19	32.3%	1.000	20	37.6%	1.000	37	62.6%	1.000	AAL94702.1| Arginyl-tRNA synthetase								
	32	54.5%	1.000	33	47.8%	1.000	27	50.4%	1.000	34	56.6%	1.000									
FN0511	13	48.4%	1.000	6	27.2%	1.000	16	52.8%	1.000	7	24.5%	1.000	AAL94707.1| D-lactate dehydrogenase								
	12	45.4%	1.000	19	53.1%	1.000	18	54.0%	1.000	8	28.1%	1.000									
FN0512	23	59.8%	1.000	26	49.9%	1.000	30	59.6%	1.000	24	57.3%	1.000	AAL94708.1| Flavoprotein								
	25	66.5%	1.000	31	63.8%	1.000	33	59.8%	1.000	25	57.6%	1.000									
FN0513	5	54.9%	1.000	4	43.7%	1.000	4	34.5%	1.000	6	54.9%	1.000	AAL94709.1| Flavodoxin								
	6	54.9%	1.000	4	54.9%	1.000	5	43.7%	1.000	6	54.9%	1.000									
FN0515	3	4.6%	0.991	0	0.0%	0.000	3	3.0%	0.987	2	1.9%	0.982	AAL94711.1| Acriflavin resistance protein D								
	3	6.8%	0.999	3	3.9%	0.963	7	9.0%	1.000	1	1.3%	0.446									
FN0516	2	13.7%	0.703	2	10.4%	0.983	0	0.0%		1	6.7%	0.892	AAL94712.1| Acriflavin resistance protein E								
	1	3.1%	0.408	4	14.6%	0.997	0	0.0%		1	2.8%	0.656									
FN0517	1	4.5%	0.977	1	6.9%	0.353	5	18.3%	1.000	1	1.8%	0.378	AAL94713.1| Outer membrane protein tolC								
	1	2.7%	0.921	5	16.7%	1.000	4	14.3%	1.000	1	2.2%	0.300									
FN0519	4	9.9%	1.000	4	12.8%	1.000	6	12.5%	1.000	1	4.4%	0.722	AAL94715.1| Hypothetical exported 24-amino acid repeat protein								
	6	19.0%	1.000	5	19.5%	1.000	4	14.0%	1.000	3	12.5%	1.000									
FN0522	4	6.1%	1.000	2	3.9%	0.961	7	11.0%	1.000	2	2.4%	0.834	AAL94718.1| Exonuclease SBCC								
	3	2.9%	0.991	7	7.4%	1.000	10	13.1%	1.000	3	2.6%	0.930									
FN0523	5	23.4%	1.000	3	11.7%	0.987	3	14.1%	1.000	2	12.0%	0.638	AAL94719.1| Exonuclease SBCD								
	5	15.8%	1.000	0	0.0%	0.000	4	14.1%	1.000	2	3.8%	1.000									
FN0524	1	1.5%	0.572	4	5.8%	0.998	4	6.2%	1.000	9	10.2%	1.000	AAL94720.1| DNA helicase II								
	2	2.3%	0.980	3	2.8%	0.999	10	11.4%	1.000	5	5.4%	1.000									
FN0525	29	41.2%	1.000	13	27.1%	1.000	35	47.9%	1.000	26	41.0%	1.000	AAL94721.1| Penicillin-binding protein								
	32	44.6%	1.000	34	42.7%	1.000	30	47.5%	1.000	26	39.9%	1.000									
FN0526	12	36.0%	1.000	1	7.0%	0.984	9	31.8%	1.000	9	33.8%	1.000	AAL94722.1| Florfenicol resistance protein								
	9	35.2%	1.000	12	43.3%	1.000	11	35.2%	1.000	6	28.2%	1.000									
FN0527	11	32.7%	1.000	9	31.4%	1.000	12	43.7%	1.000	9	25.7%	1.000	AAL94723.1| Alanyl-tRNA synthetase								
	9	25.5%	1.000	15	48.3%	1.000	17	52.5%	1.000	4	8.6%	1.000									

Show detected proteins only
Show all proteins
Filter by category:
GO: amino acid transport

Proteins found: 1424

Enter (or paste) list of ORFs

Find ORFs

Dot Plots Dot Plots

ORF	*Fn* n	*Fn* coverage	*Fn* prob	*FnPg* n	*FnPg* coverage	*FnPg* prob	*FnSg* n	*FnSg* coverage	*FnSg* prob	*FnPgSg* n	*FnPgSg* coverage	*FnPgSg* prob	Description
**FN0528**	13	78.9%	1.000	8	76.1%	1.000	4	70.4%	1.000	14	78.9%	1.000	AAL94724.1| Cold shock protein
	13	78.9%	1.000	5	70.4%	1.000	5	70.4%	1.000	14	78.9%	1.000	
**FN0535**	5	25.0%	1.000	5	19.3%	1.000	6	25.0%	1.000	6	25.0%	1.000	AAL94731.1| Hypothetical protein
	9	42.2%	1.000	7	30.2%	1.000	5	25.0%	1.000	8	37.0%	1.000	
**FN0536**	28	63.8%	1.000	24	53.0%	1.000	21	53.8%	1.000	25	61.2%	1.000	AAL94732.1| DNA polymerase III, beta chain
	28	60.4%	1.000	30	65.6%	1.000	24	56.2%	1.000	27	65.9%	1.000	
**FN0540**	15	39.6%	1.000	9	28.8%	1.000	12	34.3%	1.000	16	46.8%	1.000	AAL94736.1| Glutamate-1-semialdehyde 2,1-aminomutase
	15	41.2%	1.000	17	53.0%	1.000	19	52.8%	1.000	13	37.6%	1.000	
**FN0541**	5	12.3%	1.000	2	12.8%	0.988	6	16.5%	1.000	2	2.8%	0.997	AAL94737.1| polysaccharide deacetylase
	7	15.1%	1.000	1	2.6%	0.914	7	22.5%	1.000	2	6.8%	0.985	
**FN0542**	4	20.9%	1.000	3	19.0%	1.000	8	33.5%	1.000	5	26.6%	1.000	AAL94738.1| Beta 1,4 glucosyltransferase
	4	21.7%	1.000	10	47.5%	1.000	8	32.3%	1.000	2	8.4%	1.000	
**FN0543**	14	38.8%	1.000	5	21.4%	1.000	6	26.7%	1.000	10	32.2%	1.000	AAL94739.1| Lipopolysaccharide heptosyltransferase-1
	12	29.0%	1.000	12	36.8%	1.000	11	37.4%	1.000	10	31.0%	1.000	
**FN0544**	2	13.2%	0.999	1	6.4%	0.381	1	2.3%	0.428	1	6.4%	0.715	AAL94740.1| ADP-heptose:LPS heptosyltransferase II
	2	12.9%	0.997	3	11.4%	0.978	2	8.8%	0.989	1	6.4%	0.919	
**FN0546**	3	9.9%	0.997	1	4.2%	0.695	1	3.0%	0.288	1	3.0%	0.905	AAL94742.1| Lipopolysaccharide core biosynthesis protein rfaQ
	2	5.7%	0.982	5	16.1%	1.000	2	6.6%	0.822	1	3.0%	0.425	
**FN0547**	23	51.4%	1.000	15	45.7%	1.000	17	50.7%	1.000	21	65.9%	1.000	AAL94743.1| RecA protein
	23	54.9%	1.000	21	56.4%	1.000	17	44.6%	1.000	22	61.2%	1.000	
**FN0549**	2	7.6%	0.920	0	0.0%	0.000	2	7.3%	0.997	2	7.6%	0.955	AAL94745.1| O-sialoglycoprotein endopeptidase
	3	12.0%	0.999	2	8.5%	0.997	5	17.0%	1.000	1	3.2%	0.339	
**FN0550**	2	11.3%	0.998	7	69.5%	1.000	5	31.9%	1.000	5	39.0%	1.000	AAL94746.1| hypothetical Protein
	0	0.0%	0.000	7	52.5%	1.000	6	38.3%	1.000	2	25.5%	0.982	
**FN0552**	6	30.1%	1.000	5	27.9%	1.000	8	33.1%	1.000	4	14.4%	1.000	AAL94748.1| Serine racemase
	8	39.8%	1.000	11	37.1%	1.000	11	38.8%	1.000	2	5.1%	0.994	
**FN0553**	18	41.7%	1.000	15	32.2%	1.000	29	63.7%	1.000	20	40.1%	1.000	AAL94749.1| D-serine dehydratase
	21	46.5%	1.000	37	64.6%	1.000	35	62.6%	1.000	18	41.3%	1.000	
**FN0554**	2	3.3%	0.990	3	3.6%	1.000	5	12.7%	1.000	3	8.2%	0.998	AAL94750.1| D-serine permease
	3	8.7%	0.996	4	6.7%	1.000	4	6.7%	1.000	0	0.0%	0.000	
**FN0555**	1	6.0%	0.981	0	0.0%	0.000	3	20.1%	1.000	2	10.1%	0.988	AAL94751.1| Transcriptional regulator, MerR family
	2	10.6%	0.989	1	10.1%	0.317	2	8.5%	0.960	1	4.0%	0.979	

Fn (Rep I, Rep II) | *FnPg* (Rep I, Rep II) | *FnSg* (Rep I, Rep II) | *FnPgSg* (Rep I, Rep II)

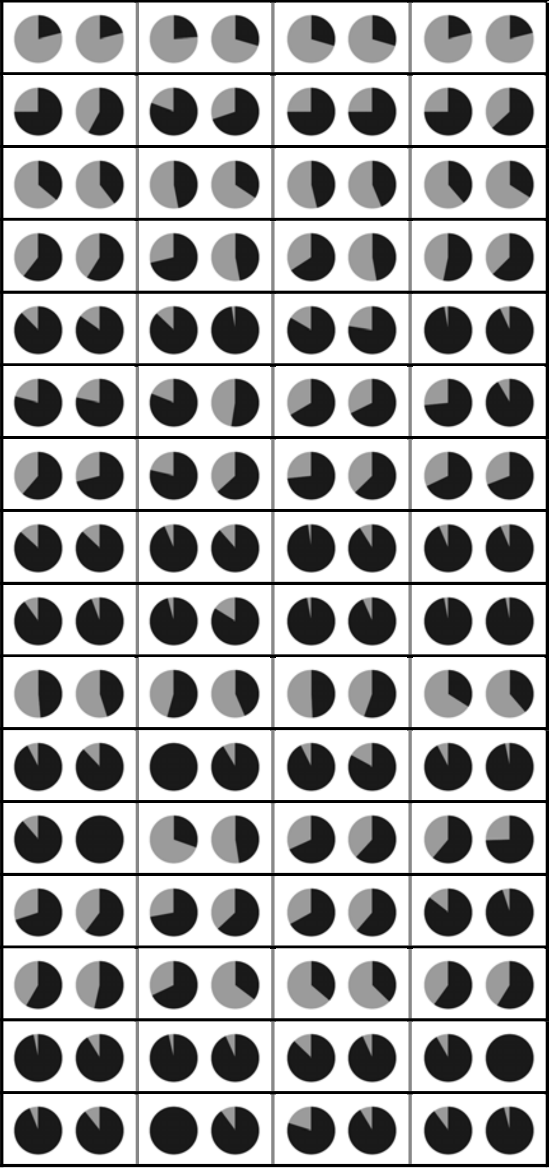

Show detected proteins only
Show all proteins
Filter by category:
GO: amino acid transport

Proteins found:
1424

Enter (or paste) list of ORFs

Find ORFs

Dot Plots Dot Plots

ORF	*Fn* n	*Fn* coverage	*Fn* prob	*FnPg* n	*FnPg* coverage	*FnPg* prob	*FnSg* n	*FnSg* coverage	*FnSg* prob	*FnPgSg* n	*FnPgSg* coverage	*FnPgSg* prob	Description
FN0556	13	60.2%	1.000	10	42.2%	1.000	10	55.8%	1.000	8	42.2%	1.000	AAL94752.1| unknown
	9	39.3%	1.000	8	38.8%	1.000	10	47.6%	1.000	8	35.0%	1.000	
FN0557	7	32.4%	1.000	8	32.8%	1.000	13	53.3%	1.000	9	40.2%	1.000	AAL94753.1| unknown
	6	31.6%	1.000	15	53.3%	1.000	10	46.3%	1.000	5	28.3%	1.000	
FN0558	8	39.8%	1.000	1	10.2%	0.926	14	63.4%	1.000	9	43.1%	1.000	AAL94754.1| TraT complement resistance protein precursor
	9	43.1%	1.000	13	50.5%	1.000	11	56.0%	1.000	9	42.6%	1.000	
FN0559	26	44.7%	1.000	18	41.4%	1.000	25	51.7%	1.000	18	29.1%	1.000	AAL94755.1| Phosphoglucomutase
	26	43.6%	1.000	34	62.4%	1.000	29	49.0%	1.000	28	50.9%	1.000	
FN0560	1	5.8%	0.901	0	0.0%		1	4.1%	0.955	2	11.0%	0.965	AAL94756.1| Oxygen-independent coproporphyrinogen III oxidase
	1	5.8%	0.979	0	0.0%		4	8.8%	1.000	1	2.7%	0.969	
FN0561	11	40.4%	1.000	9	28.7%	1.000	10	42.2%	1.000	10	46.6%	1.000	AAL94757.1| Proline synthetase associated protein
	11	34.5%	1.000	11	39.9%	1.000	13	47.5%	1.000	11	41.7%	1.000	
FN0562	13	75.7%	1.000	6	53.2%	1.000	12	78.4%	1.000	13	76.6%	1.000	AAL94758.1| Hypothetical cytosolic protein
	10	69.4%	1.000	13	81.1%	1.000	10	78.4%	1.000	14	67.6%	1.000	
FN0563	10	29.1%	1.000	4	22.8%	1.000	4	13.5%	1.000	6	18.9%	1.000	AAL94759.1| putative tRNA (5-methylaminomethyl-2-thiouridylate) -methyltransferase
	9	26.1%	1.000	3	16.2%	0.996	6	24.9%	1.000	4	14.1%	1.000	
FN0574	3	6.8%	1.000	2	5.0%	0.678	0	0.0%	0.000	3	5.2%	0.982	AAL94770.1| Hypothetical cytosolic protein
	0	0.0%	0.000	0	0.0%	0.000	1	2.3%	0.682	0	0.0%	0.000	
FN0576	19	48.5%	1.000	12	31.7%	1.000	22	54.2%	1.000	11	30.1%	1.000	AAL94772.1| hypothetical protein
	17	43.9%	1.000	14	46.3%	1.000	18	51.8%	1.000	8	24.1%	1.000	
FN0577	2	4.6%	0.969	2	4.6%	0.999	7	24.5%	1.000	1	5.4%	0.880	AAL94773.1| Hypothetical protein
	3	8.7%	1.000	5	23.2%	1.000	4	11.2%	1.000	1	5.4%	0.726	
FN0579	75	47.4%	1.000	68	39.3%	1.000	103	58.1%	1.000	85	52.8%	1.000	AAL94775.1| Hypothetical cytosolic protein
	76	47.1%	1.000	122	68.2%	1.000	89	51.3%	1.000	81	50.9%	1.000	
FN0580	7	10.9%	1.000	0	0.0%	0.000	4	8.3%	1.000	0	0.0%	0.000	AAL94776.1| Penicillin-binding protein
	7	14.8%	1.000	4	7.5%	0.990	5	10.1%	1.000	1	2.6%	0.470	
FN0581	4	8.0%	1.000	1	4.1%	0.984	5	15.2%	1.000	2	7.5%	1.000	AAL94777.1| Lipoprotein releasing system transmembrane protein lolE
	5	11.6%	1.000	3	9.8%	1.000	1	4.1%	0.956	3	9.5%	1.000	
FN0582	9	42.1%	1.000	2	11.2%	0.997	10	39.1%	1.000	10	40.3%	1.000	AAL94778.1| Lipoprotein releasing system ATP-binding protein lolD
	11	43.3%	1.000	7	34.8%	1.000	11	39.1%	1.000	6	34.3%	1.000	
FN0583	4	26.9%	1.000	1	16.6%	0.984	3	22.8%	1.000	0	0.0%		AAL94779.1| Hypothetical Exported Protein
	2	23.4%	1.000	5	27.6%	1.000	6	34.5%	1.000	0	0.0%		

Pie-chart columns: *Fn* Rep I, Rep II; *FnPg* Rep I, Rep II; *FnSg* Rep I, Rep II; *FnPgSg* Rep I, Rep II

Show detected proteins only
Show all proteins
Filter by category:
GO: amino acid transport

Proteins found: 1424

Enter (or paste) list of ORFs

Find ORFs

Dot Plots Dot Plots

ORF	*Fn* n	*Fn* coverage	*Fn* prob	*FnPg* n	*FnPg* coverage	*FnPg* prob	*FnSg* n	*FnSg* coverage	*FnSg* prob	*FnPgSg* n	*FnPgSg* coverage	*FnPgSg* prob	Description
FN0585	4	22.3%	1.000	7	21.0%	1.000	5	19.2%	1.000	3	32.1%	0.994	AAL94781.1| Two-component response regulator czcR
	5	22.3%	1.000	3	22.8%	1.000	6	22.8%	1.000	2	16.5%	0.676	
FN0586	3	9.0%	0.992	1	2.9%	0.979	4	14.8%	1.000	4	12.1%	0.991	AAL94782.1| Two-component sensor kinase czcS
	4	11.9%	1.000	4	9.0%	1.000	5	12.6%	1.000	1	2.2%	0.432	
FN0590	7	23.9%	1.000	3	13.0%	1.000	12	37.2%	1.000	5	17.6%	1.000	AAL94786.1| N-acyl-L-amino acid amidohydrolase
	5	25.4%	1.000	14	43.5%	1.000	16	46.6%	1.000	3	8.9%	0.998	
FN0592	31	45.6%	1.000	14	18.8%	1.000	33	46.8%	1.000	24	32.4%	1.000	AAL94788.1| ATP-dependent DNA helicase pcrA
	30	47.9%	1.000	28	39.3%	1.000	25	34.0%	1.000	21	26.1%	1.000	
FN0593	11	29.3%	1.000	8	25.4%	1.000	13	32.9%	1.000	13	36.0%	1.000	AAL94789.1| UDP-3-O-[3-hydroxymyristoyl] N-acetylglucosamine deacetylase
	14	42.0%	1.000	16	42.8%	1.000	12	33.6%	1.000	16	44.5%	1.000	
FN0594	3	25.5%	1.000	1	11.3%	0.957	11	42.6%	1.000	3	42.6%	1.000	AAL94790.1| (3R)-hydroxymyristoyl-[acyl carrier protein] dehydratase
	2	11.3%	0.999	13	43.3%	1.000	10	42.6%	1.000	1	11.3%	0.956	
FN0595	5	17.1%	1.000	2	5.4%	0.945	8	22.6%	1.000	7	28.8%	1.000	AAL94791.1| Acyl-[acyl-carrier-protein]-UDP-N-acetylglucosamine O-[illegible]
	8	20.6%	1.000	11	31.9%	1.000	7	23.0%	1.000	7	23.7%	1.000	
FN0596	7	29.2%	1.000	4	15.7%	1.000	8	28.5%	1.000	8	39.3%	1.000	AAL94792.1| Hypothetical protein
	9	30.0%	1.000	10	40.1%	1.000	9	36.0%	1.000	2	7.9%	0.998	
FN0597	5	13.5%	1.000	7	11.8%	1.000	11	21.9%	1.000	5	15.7%	1.000	AAL94793.1| Lipid-A-disaccharide synthase
	7	19.1%	1.000	14	35.4%	1.000	12	22.8%	1.000	5	19.7%	1.000	
FN0598	9	22.0%	1.000	3	8.1%	1.000	7	15.3%	1.000	9	16.0%	1.000	AAL94794.1| Phospholipid-lipopolysaccharide ABC transporter
	8	18.5%	1.000	9	20.6%	1.000	7	14.9%	1.000	11	22.1%	1.000	
FN0600	12	70.5%	1.000	6	36.7%	1.000	12	54.2%	1.000	12	66.9%	1.000	AAL94796.1| Hypothetical protein
	12	63.9%	1.000	20	80.1%	1.000	15	68.7%	1.000	13	66.9%	1.000	
FN0601	5	37.6%	1.000	0	0.0%	0.000	0	0.0%	0.000	2	18.4%	0.995	AAL94797.1| Hypothetical exported 24-amino acid repeat protein
	5	37.6%	1.000	0	0.0%	0.000	1	12.1%	0.975	1	6.4%	0.986	
FN0602	17	52.5%	1.000	16	48.7%	1.000	16	43.6%	1.000	12	40.3%	1.000	AAL94798.1| Hypothetical protein
	17	52.5%	1.000	18	47.9%	1.000	15	38.6%	1.000	14	51.7%	1.000	
FN0603	1	4.5%	0.967	1	4.1%	0.650	6	22.3%	1.000	1	3.5%	0.348	AAL94799.1| Transcriptional regulatory protein, LYSR family
	3	8.0%	0.999	7	20.7%	1.000	8	29.0%	1.000	0	0.0%	0.000	
FN0605	0	0.0%		1	6.7%	0.984	0	0.0%	0.000	1	3.0%	0.342	AAL94801.1| Aspartate aminotransferase
	0	0.0%		2	8.7%	0.490	1	6.7%	0.980	3	6.0%	0.979	
FN0608	17	26.6%	1.000	19	28.9%	1.000	26	41.7%	1.000	18	26.1%	1.000	AAL94804.1| Exoribonuclease II
	16	25.4%	1.000	33	44.7%	1.000	32	43.7%	1.000	19	30.3%	1.000	

Fn Rep I / Rep II | *FnPg* Rep I / Rep II | *FnSg* Rep I / Rep II | *FnPgSg* Rep I / Rep II

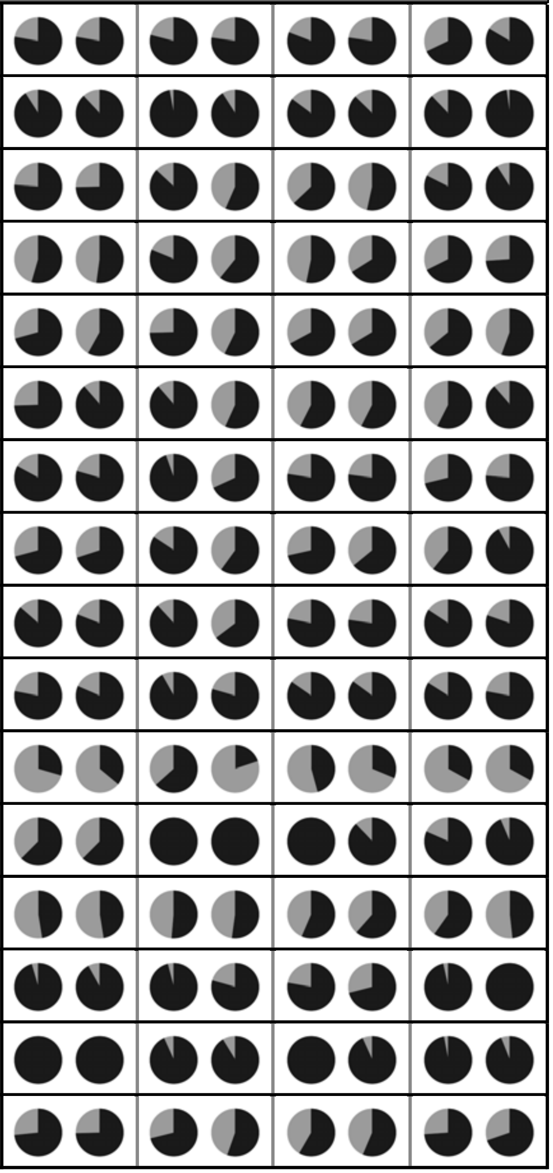

Show detected proteins only
Show all proteins
Filter by category:
GO: amino acid transport

Proteins found: 1424

Enter (or paste) list of ORFs

Find ORFs

Dot Plots Dot Plots

ORF	*Fn* n	*Fn* coverage	*Fn* prob	*FnPg* n	*FnPg* coverage	*FnPg* prob	*FnSg* n	*FnSg* coverage	*FnSg* prob	*FnPgSg* n	*FnPgSg* coverage	*FnPgSg* prob	Description
FN0609	2	15.5%	0.996	0	0.0%	0.000	2	15.5%	0.991	2	15.5%	0.994	AAL94805.1| Small protein B
	1	6.8%	0.957	3	15.5%	1.000	3	15.5%	0.999	0	0.0%	0.000	
FN0610	50	45.3%	1.000	21	19.0%	1.000	48	44.7%	1.000	42	40.6%	1.000	AAL94806.1| unknown
	54	49.7%	1.000	67	52.3%	1.000	49	44.6%	1.000	43	43.3%	1.000	
FN0611	36	60.8%	1.000	32	54.4%	1.000	47	68.1%	1.000	32	48.9%	1.000	AAL94807.1| Threonyl-tRNA synthetase
	39	60.8%	1.000	45	64.2%	1.000	42	66.1%	1.000	31	54.2%	1.000	
FN0612	11	59.6%	1.000	7	45.2%	1.000	9	44.6%	1.000	8	44.0%	1.000	AAL94808.1| Hypothetical protein
	10	45.2%	1.000	10	50.6%	1.000	9	45.2%	1.000	9	44.0%	1.000	
FN0614	0	0.0%	0.000	1	2.1%	0.371	0	0.0%	0.000	2	3.6%	0.901	AAL94810.1| Export ABC transporter
	1	5.5%	0.298	1	4.0%	0.469	3	5.7%	0.995	0	0.0%	0.000	
FN0615	0	0.0%		3	12.2%	0.935	1	3.7%	0.469	1	3.1%	0.606	AAL94811.1| Export ABC transporter
	0	0.0%		0	0.0%	0.000	0	0.0%	0.000	1	3.1%	0.298	
FN0616	12	25.7%	1.000	2	3.8%	0.988	1	4.0%	0.412	15	34.9%	1.000	AAL94812.1| Hypothetical protein
	12	25.1%	1.000	5	11.7%	1.000	0	0.0%	0.000	10	28.5%	1.000	
FN0617	25	57.4%	1.000	13	40.4%	1.000	20	53.3%	1.000	22	60.4%	1.000	AAL94813.1| DNA polymerase III, beta chain
	27	69.8%	1.000	28	70.3%	1.000	19	52.7%	1.000	23	62.6%	1.000	
FN0618	19	57.3%	1.000	12	40.6%	1.000	20	62.6%	1.000	18	48.8%	1.000	AAL94814.1| Spermidine/putrescine-binding protein
	19	55.3%	1.000	24	68.7%	1.000	16	50.9%	1.000	18	48.8%	1.000	
FN0619	4	15.3%	1.000	3	14.6%	0.998	3	10.7%	1.000	2	5.0%	1.000	AAL94815.1| Small-conductance mechanosensitive channel
	6	21.0%	1.000	5	21.0%	1.000	5	16.4%	1.000	3	9.6%	1.000	
FN0621	13	35.5%	1.000	7	19.8%	1.000	11	26.5%	1.000	14	30.6%	1.000	AAL94817.1| 4-hydroxybutyrate coenzyme A transferase
	13	34.6%	1.000	9	24.9%	1.000	13	26.7%	1.000	11	27.2%	1.000	
FN0622	5	21.2%	1.000	5	14.7%	1.000	10	41.9%	1.000	4	14.3%	1.000	AAL94818.1| 8-oxoguanine DNA glycosylase
	4	16.1%	1.000	16	49.8%	1.000	16	53.0%	1.000	3	15.2%	0.997	
FN0625	10	30.7%	1.000	9	30.7%	1.000	2	9.8%	0.909	14	34.2%	1.000	AAL94821.1| Aspartate aminotransferase
	10	37.2%	1.000	1	2.3%	0.376	8	24.9%	1.000	10	25.4%	1.000	
FN0626	2	7.6%	0.995	2	16.2%	0.794	1	7.6%	0.795	1	7.6%	0.954	AAL94822.1| Hypothetical cytosolic protein
	2	7.6%	0.996	1	7.6%	0.982	5	23.8%	1.000	2	7.6%	0.992	
FN0627	11	32.7%	1.000	6	17.0%	1.000	16	41.1%	1.000	18	41.4%	1.000	AAL94823.1| Glucosamine--fructose-6-phosphate aminotransferase (isomerizing)
	13	35.1%	1.000	18	44.9%	1.000	15	41.1%	1.000	18	37.5%	1.000	
FN0628	3	12.5%	0.989	0	0.0%	0.000	5	18.2%	1.000	5	19.6%	1.000	AAL94824.1| Glucosamine--fructose-6-phosphate aminotransferase (isomerizing)
	4	12.5%	1.000	1	4.3%	0.387	3	13.1%	0.999	6	19.3%	1.000	

Show detected proteins only
Show all proteins
Filter by category:
GO: amino acid transport

Proteins found: 1424

Enter (or paste) list of ORFs

Find ORFs

Dot Plots Dot Plots

ORF	*Fn* n	*Fn* coverage	*Fn* prob	*FnPg* n	*FnPg* coverage	*FnPg* prob	*FnSg* n	*FnSg* coverage	*FnSg* prob	*FnPgSg* n	*FnPgSg* coverage	*FnPgSg* prob	Description
**FN0629**	4	11.8%	1.000	0	0.0%	0.000	4	24.4%	1.000	7	24.4%	1.000	AAL94825.1| PTS system, IID component
	5	14.7%	1.000	3	16.1%	1.000	6	25.8%	1.000	8	25.4%	1.000	
**FN0630**	2	23.2%	0.986	0	0.0%	0.000	2	12.7%	0.994	4	15.8%	1.000	AAL94826.1| PTS system, IIC component
	3	17.8%	1.000	2	11.6%	1.000	0	0.0%	0.000	4	16.6%	1.000	
**FN0631**	7	50.3%	1.000	1	6.5%	0.852	5	46.4%	1.000	3	25.5%	1.000	AAL94827.1| PTS system, IIB component
	6	45.1%	1.000	5	35.9%	1.000	5	40.5%	1.000	4	23.5%	1.000	
**FN0633**	5	10.1%	1.000	1	3.4%	0.760	4	9.9%	1.000	3	8.7%	1.000	AAL94829.1| Replication protein
	4	9.4%	1.000	9	21.5%	1.000	8	22.9%	1.000	6	14.0%	1.000	
**FN0634**	19	30.6%	1.000	12	23.1%	1.000	12	30.1%	1.000	21	31.9%	1.000	AAL94830.1| GTP-binding protein TypA/BipA
	24	30.2%	1.000	15	27.9%	1.000	14	34.5%	1.000	17	27.1%	1.000	
**FN0636**	3	37.8%	1.000	2	26.1%	0.995	2	31.1%	0.991	0	0.0%		AAL94832.1| Hypothetical protein
	2	26.1%	0.998	2	26.1%	0.992	1	19.3%	0.985	0	0.0%		
**FN0637**	3	18.0%	1.000	1	9.3%	0.588	0	0.0%	0.000	4	24.4%	1.000	AAL94833.1| Hypothetical exported 24-amino acid repeat protein
	3	16.9%	0.997	2	10.5%	0.999	1	4.7%	0.575	3	20.3%	1.000	
**FN0643**	7	26.9%	1.000	6	26.9%	1.000	4	19.3%	1.000	5	41.4%	1.000	AAL94839.1| hypothetical DNA-binding protein
	5	26.2%	1.000	7	40.0%	1.000	8	42.1%	1.000	3	19.3%	1.000	
**FN0644**	13	41.0%	1.000	8	26.8%	1.000	11	32.8%	1.000	13	39.8%	1.000	AAL94840.1| Uroporphyrin-III C-methyltransferase
	15	44.3%	1.000	16	40.8%	1.000	13	32.8%	1.000	10	25.6%	1.000	
**FN0645**	5	21.1%	1.000	4	11.1%	1.000	2	8.1%	0.869	5	18.1%	1.000	AAL94841.1| Porphobilinogen deaminase
	7	25.5%	1.000	4	17.8%	0.997	6	20.5%	1.000	3	13.4%	1.000	
**FN0646**	1	3.3%	0.288	0	0.0%	0.000	0	0.0%	0.000	3	15.2%	0.998	AAL94842.1| Glutamyl-tRNA reductase
	1	3.3%	0.296	2	7.9%	0.998	3	10.9%	0.999	2	9.1%	0.995	
**FN0647**	2	3.5%	0.666	1	5.1%	0.664	0	0.0%	0.000	3	9.3%	0.984	AAL94843.1| transcriptional regulator
	0	0.0%	0.000	2	6.9%	0.714	2	6.6%	0.991	2	5.3%	0.916	
**FN0649**	1	1.5%	0.243	4	12.2%	1.000	1	1.5%	0.526	2	4.8%	0.879	AAL94845.1| Exoenzymes regulatory protein aepA precursor
	0	0.0%	0.000	2	3.7%	0.982	1	1.5%	0.373	2	3.5%	0.991	
**FN0651**	1	4.2%	0.981	1	7.3%	0.786	1	5.9%	0.977	1	3.8%	0.408	AAL94847.1| Ribosomal large subunit pseudouridine synthase D
	2	8.0%	0.988	1	4.2%	0.381	3	17.4%	1.000	1	4.2%	0.964	
**FN0652**	34	79.7%	1.000	27	73.7%	1.000	36	82.7%	1.000	31	78.2%	1.000	AAL94848.1| Glyceraldehyde 3-phosphate dehydrogenase
	33	79.7%	1.000	35	86.6%	1.000	34	81.8%	1.000	34	78.8%	1.000	
**FN0653**	17	64.6%	1.000	9	37.3%	1.000	13	52.4%	1.000	8	41.0%	1.000	AAL94849.1| unknown
	15	69.3%	1.000	17	58.5%	1.000	16	54.7%	1.000	8	37.3%	1.000	

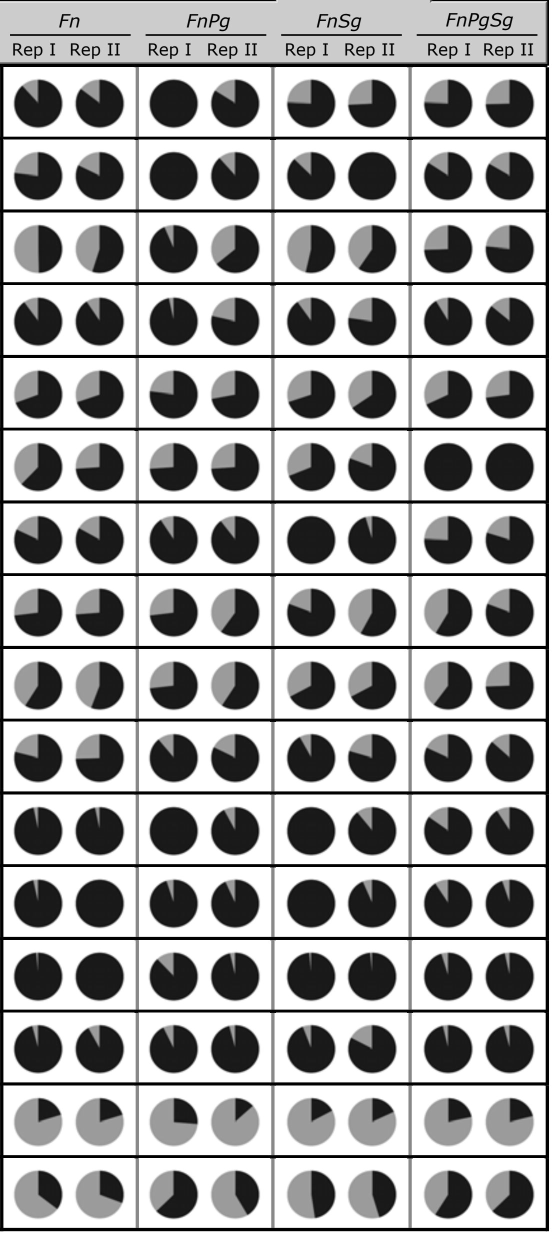

Show detected proteins only
Show all proteins
Filter by category:
GO: amino acid transport

Proteins found: 1424

Enter (or paste) list of ORFs

Find ORFs

Dot Plots Dot Plots

ORF	*Fn* n	*Fn* coverage	*Fn* prob	*FnPg* n	*FnPg* coverage	*FnPg* prob	*FnSg* n	*FnSg* coverage	*FnSg* prob	*FnPgSg* n	*FnPgSg* coverage	*FnPgSg* prob	Description
**FN0654**	32	66.1%	1.000	20	55.0%	1.000	39	81.2%	1.000	36	70.4%	1.000	AAL94850.1| Phosphoglycerate kinase
	33	66.3%	1.000	40	76.9%	1.000	35	76.9%	1.000	34	67.3%	1.000	
**FN0655**	8	33.0%	1.000	1	12.5%	0.983	4	33.0%	1.000	6	33.0%	1.000	AAL94851.1| unknown
	4	33.0%	1.000	4	33.9%	0.997	4	42.0%	1.000	7	33.0%	1.000	
**FN0656**	4	39.7%	1.000	0	0.0%	0.000	1	14.3%	0.625	4	39.7%	1.000	AAL94852.1| Hypothetical protein
	4	39.7%	1.000	2	42.1%	0.992	1	25.4%	0.463	4	39.7%	1.000	
**FN0657**	3	20.2%	1.000	2	18.5%	0.990	6	35.8%	1.000	0	0.0%	0.000	AAL94853.1| Acetyltransferase
	2	20.2%	0.994	6	39.9%	1.000	7	48.0%	1.000	1	11.0%	0.673	
**FN0658**	20	63.2%	1.000	10	42.5%	1.000	12	48.7%	1.000	23	65.5%	1.000	AAL94854.1| ABC transporter substrate-binding protein
	18	56.3%	1.000	16	59.4%	1.000	15	57.9%	1.000	20	59.0%	1.000	
**FN0660**	5	21.2%	1.000	3	10.4%	1.000	4	17.9%	1.000	4	20.0%	1.000	AAL94856.1| ABC transporter ATP-binding protein
	5	24.2%	1.000	4	20.0%	0.999	7	31.6%	1.000	4	18.5%	1.000	
**FN0662**	13	42.1%	1.000	3	11.6%	1.000	12	39.3%	1.000	13	41.8%	1.000	AAL94858.1| Formiminoglutamase
	11	37.4%	1.000	26	68.6%	1.000	17	55.7%	1.000	14	39.6%	1.000	
**FN0664**	22	66.0%	1.000	12	33.5%	1.000	15	43.5%	1.000	17	60.2%	1.000	AAL94860.1| 2-nitropropane dioxygenase
	23	73.0%	1.000	14	38.7%	1.000	30	81.9%	1.000	15	53.7%	1.000	
**FN0666**	5	39.0%	1.000	1	6.3%	0.374	3	21.5%	0.994	2	7.8%	0.999	AAL94862.1| Hypothetical protein
	6	31.2%	1.000	1	4.4%	0.745	4	28.8%	0.998	1	3.4%	0.985	
**FN0668**	8	34.6%	1.000	4	18.3%	1.000	7	37.5%	1.000	9	25.0%	1.000	AAL94864.1| High-affinity zinc uptake system protein znuA precursor
	12	45.8%	1.000	10	44.9%	1.000	9	36.2%	1.000	4	15.4%	1.000	
**FN0672**	1	5.9%	0.232	1	2.5%	0.564	7	20.1%	1.000	2	4.8%	0.998	AAL94868.1| ATPase
	0	0.0%	0.000	5	12.6%	1.000	7	18.5%	1.000	5	10.7%	1.000	
**FN0675**	49	63.8%	1.000	36	57.1%	1.000	57	76.4%	1.000	55	66.6%	1.000	AAL94871.1| 60 kDa chaperonin GROEL
	48	64.4%	1.000	62	86.5%	1.000	57	76.4%	1.000	53	69.0%	1.000	
**FN0676**	9	60.0%	1.000	2	17.8%	0.706	5	41.1%	1.000	8	51.1%	1.000	AAL94872.1| 10 kDa chaperonin GROES
	9	60.0%	1.000	8	68.9%	1.000	6	60.0%	1.000	8	50.0%	1.000	
**FN0677**	14	46.2%	1.000	9	27.2%	1.000	14	50.1%	1.000	7	19.3%	1.000	AAL94873.1| Hypothetical protein
	11	36.5%	1.000	16	46.5%	1.000	18	43.6%	1.000	7	22.1%	1.000	
**FN0678**	17	55.0%	1.000	4	21.5%	1.000	12	45.5%	1.000	14	54.5%	1.000	AAL94874.1| Ser/Thr protein kinase
	14	55.0%	1.000	9	50.0%	1.000	11	50.0%	1.000	11	54.5%	1.000	
**FN0679**	6	25.6%	1.000	3	8.1%	1.000	2	13.0%	0.984	1	6.0%	0.989	AAL94875.1| GTPase
	5	22.8%	1.000	4	21.8%	1.000	4	15.1%	1.000	3	15.1%	1.000	

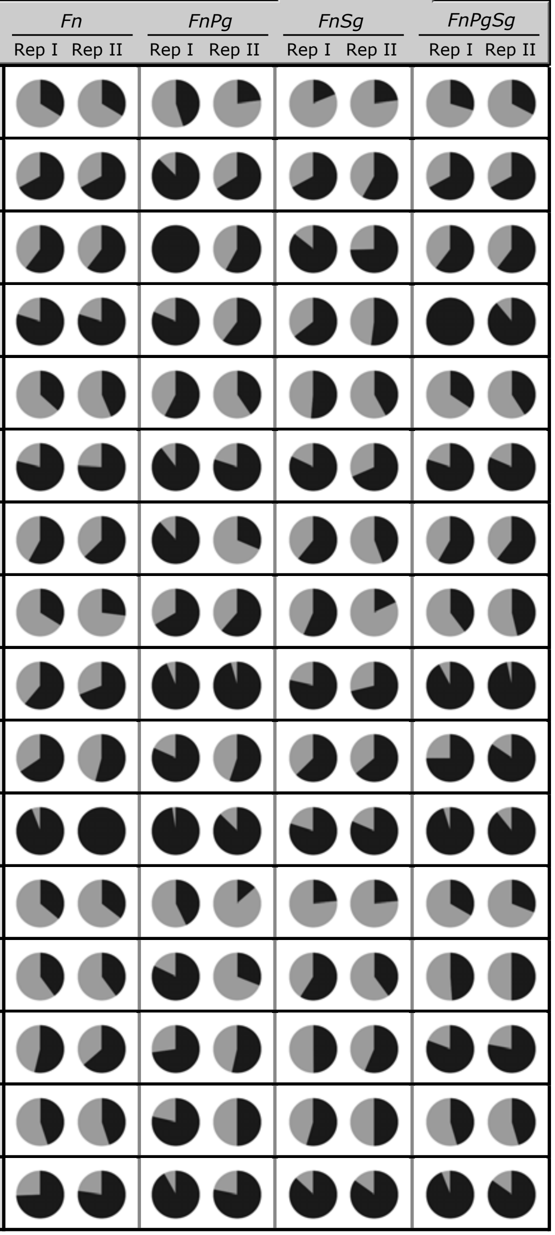

Show detected proteins only
Show all proteins
Filter by category:
GO: amino acid transport

Proteins found: 1424

Enter (or paste) list of ORFs

Find ORFs

Dot Plots Dot Plots

ORF	*Fn* n	*Fn* coverage	*Fn* prob	*FnPg* n	*FnPg* coverage	*FnPg* prob	*FnSg* n	*FnSg* coverage	*FnSg* prob	*FnPgSg* n	*FnPgSg* coverage	*FnPgSg* prob	Description	*Fn* Rep I	*Fn* Rep II	*FnPg* Rep I	*FnPg* Rep II	*FnSg* Rep I	*FnSg* Rep II	*FnPgSg* Rep I	*FnPgSg* Rep II
FN0680	2	12.6%	0.990	1	12.6%	0.907	2	19.5%	0.999	3	24.2%	1.000	AAL94876.1| Ribulose-phosphate 3-epimerase								
	2	18.1%	0.984	3	25.1%	1.000	3	25.1%	1.000	3	24.2%	1.000									
FN0681	19	60.4%	1.000	18	60.0%	1.000	23	74.7%	1.000	22	73.3%	1.000	AAL94877.1| Transcriptional regulator, MarR family								
	17	60.4%	1.000	23	71.1%	1.000	24	74.7%	1.000	17	63.6%	1.000									
FN0682	9	12.9%	1.000	8	13.9%	1.000	13	24.0%	1.000	10	21.4%	1.000	AAL94878.1| Fibronectin-binding protein-like protein A								
	8	8.9%	1.000	9	18.1%	1.000	15	27.7%	1.000	8	17.9%	1.000									
FN0684	0	0.0%		5	16.1%	1.000	8	21.2%	1.000	3	9.5%	0.909	AAL94880.1| Prismane protein								
	0	0.0%		16	38.5%	1.000	7	18.2%	1.000	1	2.7%	0.383									
FN0685	4	6.2%	1.000	2	2.5%	1.000	6	11.0%	1.000	4	11.8%	1.000	AAL94881.1| Sodium/pantothenate symporter								
	5	11.4%	1.000	8	10.1%	1.000	7	16.5%	1.000	3	10.5%	0.999									
FN0688	5	43.1%	1.000	0	0.0%	0.000	6	50.3%	1.000	3	24.8%	1.000	AAL94884.1| Hypothetical protein								
	5	43.1%	1.000	8	50.3%	1.000	8	50.3%	1.000	3	24.2%	1.000									
FN0689	20	75.2%	1.000	8	50.3%	1.000	17	79.7%	1.000	18	74.5%	1.000	AAL94885.1| Hypothetical protein								
	18	69.9%	1.000	15	79.7%	1.000	16	79.7%	1.000	15	74.5%	1.000									
FN0692	6	24.1%	1.000	2	10.1%	0.992	3	13.0%	0.998	2	9.4%	1.000	AAL94888.1| Nitrogen regulation protein NIFR3								
	9	37.5%	1.000	2	8.1%	1.000	6	23.5%	1.000	3	13.4%	0.999									
FN0693	11	16.4%	1.000	11	14.8%	1.000	1	0.8%	0.287	6	9.4%	1.000	AAL94889.1| DNA mismatch repair protein mutS								
	13	18.5%	1.000	1	0.8%	0.296	2	3.0%	0.555	2	2.8%	0.973									
FN0694	26	43.4%	1.000	10	20.7%	1.000	15	26.6%	1.000	21	36.1%	1.000	AAL94890.1| S-layer protein								
	23	37.2%	1.000	14	28.3%	1.000	24	36.2%	1.000	21	37.8%	1.000									
FN0695	9	40.7%	1.000	5	33.2%	1.000	11	54.8%	1.000	5	36.1%	1.000	AAL94891.1| ABC transporter ATP-binding protein								
	8	36.5%	1.000	8	35.7%	1.000	10	49.8%	1.000	5	27.4%	1.000									
FN0697	39	37.1%	1.000	29	39.6%	1.000	29	39.3%	1.000	30	31.7%	1.000	AAL94893.1| Alanyl-tRNA synthetase								
	36	37.3%	1.000	32	41.1%	1.000	36	42.1%	1.000	34	43.8%	1.000									
FN0699	11	30.9%	1.000	10	27.3%	1.000	10	38.0%	1.000	12	42.3%	1.000	AAL94895.1| Protein translocase subunit secD								
	13	36.3%	1.000	8	25.5%	1.000	8	30.7%	1.000	14	44.3%	1.000									
FN0700	6	16.7%	1.000	5	29.3%	1.000	3	12.6%	1.000	5	22.1%	1.000	AAL94896.1| Protein translocase subunit secF								
	7	19.9%	1.000	5	18.3%	1.000	5	24.6%	1.000	9	35.3%	1.000									
FN0701	22	48.9%	1.000	25	53.0%	1.000	20	43.5%	1.000	21	47.2%	1.000	AAL94897.1| Methyltransferase								
	22	46.8%	1.000	25	50.3%	1.000	23	49.9%	1.000	21	41.2%	1.000									
FN0705	35	38.6%	1.000	13	20.7%	1.000	31	32.1%	1.000	26	29.0%	1.000	AAL94901.1| DNA polymerase I								
	32	38.9%	1.000	36	41.1%	1.000	40	42.3%	1.000	21	25.5%	1.000									

Show detected proteins only
Show all proteins
Filter by category:
GO: amino acid transport

Proteins found: 1424

Enter (or paste) list of ORFs

Find ORFs

Dot Plots Dot Plots

Fn Coverage

Fusobacterium nucleatum

Hackett Laboratory UW

ORF	*Fn* n	*Fn* coverage	*Fn* prob	*FnPg* n	*FnPg* coverage	*FnPg* prob	*FnSg* n	*FnSg* coverage	*FnSg* prob	*FnPgSg* n	*FnPgSg* coverage	*FnPgSg* prob	Description
FN0706	3	12.4%	1.000	0	0.0%	0.000	1	5.7%	0.981	3	18.4%	1.000	AAL94902.1| Hypothetical cytosolic protein
	4	23.4%	1.000	1	6.4%	0.983	3	17.1%	0.999	1	5.7%	0.537	
FN0707	6	17.9%	1.000	7	26.3%	1.000	5	14.1%	1.000	5	15.4%	1.000	AAL94903.1| Riboflavin kinase
	9	27.6%	1.000	4	15.4%	1.000	6	19.1%	1.000	3	14.1%	1.000	
FN0710	13	38.2%	1.000	9	26.7%	1.000	12	35.6%	1.000	11	31.1%	1.000	AAL94906.1| Hypothetical protein
	13	38.7%	1.000	14	38.7%	1.000	12	27.6%	1.000	8	34.7%	1.000	
FN0711	3	6.9%	0.985	1	3.0%	0.983	5	11.4%	1.000	4	9.7%	0.998	AAL94907.1| Phosphopantothenate--cysteine ligase
	5	12.6%	1.000	7	24.0%	1.000	9	25.0%	1.000	6	11.9%	1.000	
FN0714	2	8.0%	0.886	4	19.4%	1.000	5	18.8%	1.000	2	9.6%	0.872	AAL94910.1| NADH oxidase
	2	8.0%	0.743	4	17.2%	1.000	6	30.3%	1.000	2	8.0%	0.986	
FN0715	11	40.3%	1.000	6	21.4%	1.000	7	25.5%	1.000	12	33.4%	1.000	AAL94911.1| Hypothetical protein
	9	33.4%	1.000	7	33.4%	1.000	6	29.0%	1.000	12	36.9%	1.000	
FN0716	16	42.7%	1.000	10	38.9%	1.000	12	46.2%	1.000	10	39.2%	1.000	AAL94912.1| hypothetical protein
	12	42.0%	1.000	7	28.7%	1.000	9	31.8%	1.000	10	31.2%	1.000	
FN0717	2	13.7%	0.984	1	5.3%	0.984	2	13.7%	0.943	4	25.2%	0.999	AAL94913.1| Ribosomal small subunit pseudouridine synthase A
	3	20.8%	0.996	1	8.4%	0.803	4	28.3%	0.998	1	7.1%	0.916	
FN0719	4	14.6%	1.000	1	4.9%	0.618	0	0.0%	0.000	1	2.9%	0.986	AAL94915.1| Hypothetical cytosolic protein
	4	12.0%	1.000	2	6.9%	0.961	1	2.0%	0.326	2	4.9%	0.995	
FN0720	11	46.0%	1.000	5	23.0%	1.000	6	36.4%	1.000	7	41.7%	1.000	AAL94916.1| Protein Translation Elongation Factor P (EF-P)
	7	41.7%	1.000	8	49.2%	1.000	8	42.8%	1.000	7	32.6%	1.000	
FN0721	15	49.4%	1.000	3	11.3%	1.000	6	25.5%	1.000	11	45.6%	1.000	AAL94917.1| Hypothetical protein
	12	38.1%	1.000	10	41.0%	1.000	5	24.3%	1.000	13	44.4%	1.000	
FN0722	3	5.9%	0.999	5	8.7%	1.000	6	10.2%	1.000	4	7.1%	1.000	AAL94918.1| WD-repeat family protein
	4	6.3%	1.000	3	5.6%	0.994	6	11.3%	1.000	0	0.0%	0.000	
FN0723	1	1.6%	0.977	1	3.9%	0.845	1	1.8%	0.949	1	3.1%	0.589	AAL94919.1| Hypothetical protein
	2	4.9%	0.994	4	8.1%	0.948	5	14.1%	1.000	0	0.0%	0.000	
FN0724	10	55.7%	1.000	10	57.5%	1.000	9	59.9%	1.000	9	53.3%	1.000	AAL94920.1| Flavodoxin
	8	46.7%	1.000	12	86.2%	1.000	14	86.2%	1.000	11	79.0%	1.000	
FN0725	5	23.9%	1.000	4	23.9%	0.996	8	47.0%	1.000	4	23.9%	1.000	AAL94921.1| Molybdopterin biosynthesis MoeB protein
	4	23.9%	1.000	8	33.3%	1.000	14	61.1%	1.000	5	20.1%	1.000	
FN0728	9	25.1%	1.000	9	44.1%	1.000	9	40.8%	1.000	7	23.7%	1.000	AAL94924.1| Hypothetical protein
	8	37.0%	1.000	13	60.7%	1.000	11	55.0%	1.000	7	23.7%	1.000	

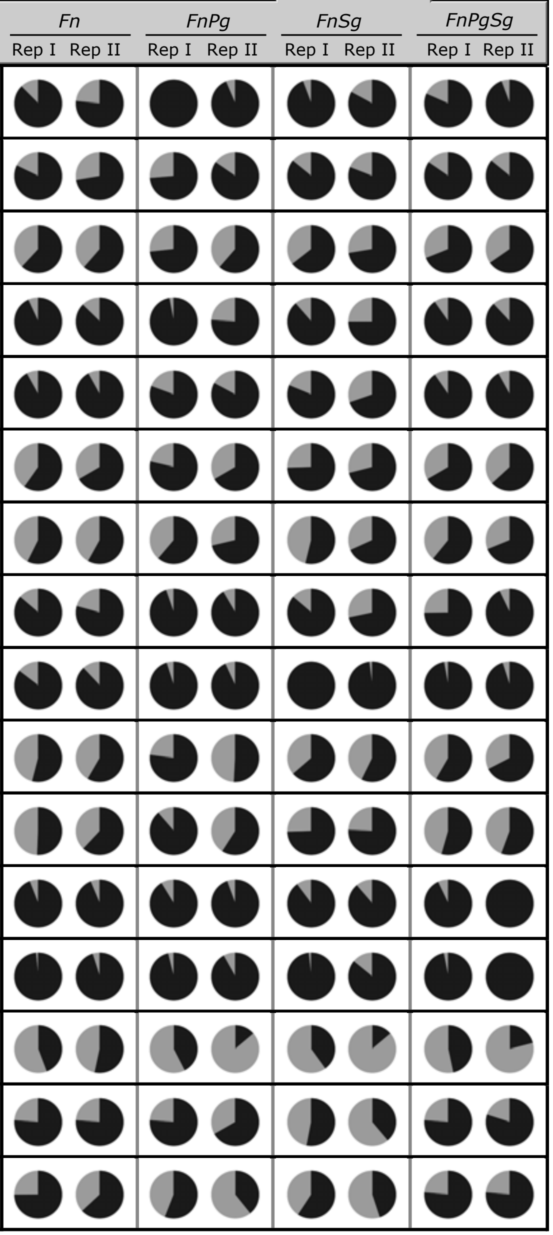

Show detected proteins only
Show all proteins
Filter by category:
GO: amino acid transport

Proteins found:
1424

Enter (or paste) list of ORFs

Find ORFs

Dot Plots Dot Plots

ORF	*Fn* n	*Fn* coverage	*Fn* prob	*FnPg* n	*FnPg* coverage	*FnPg* prob	*FnSg* n	*FnSg* coverage	*FnSg* prob	*FnPgSg* n	*FnPgSg* coverage	*FnPgSg* prob	Description
**FN0729**	18	36.8%	1.000	19	56.1%	1.000	14	40.8%	1.000	18	42.1%	1.000	AAL94925.1| Phosphoglycerate mutase
	18	44.7%	1.000	15	46.9%	1.000	14	56.1%	1.000	17	45.2%	1.000	
**FN0731**	9	29.4%	1.000	4	17.5%	1.000	5	22.6%	1.000	7	34.5%	1.000	AAL94927.1| Hypothetical protein
	7	23.2%	1.000	11	45.8%	1.000	5	22.6%	1.000	6	33.3%	1.000	
**FN0733**	21	54.9%	1.000	17	42.2%	1.000	15	44.4%	1.000	20	57.0%	1.000	AAL94929.1| Peptidase T
	25	54.9%	1.000	16	49.3%	1.000	16	49.0%	1.000	18	51.0%	1.000	
**FN0734**	5	10.4%	1.000	1	1.4%	0.303	3	8.3%	0.883	2	3.0%	0.708	AAL94930.1| Fe-S oxidoreductase
	5	8.6%	1.000	1	3.0%	0.260	3	5.6%	0.980	0	0.0%	0.000	
**FN0735**	18	41.2%	1.000	21	40.2%	1.000	8	14.9%	1.000	17	41.5%	1.000	AAL94931.1| Cell surface protein
	16	35.8%	1.000	17	30.6%	1.000	12	18.8%	1.000	16	43.3%	1.000	
**FN0736**	2	10.4%	0.965	3	10.4%	1.000	6	37.1%	1.000	3	19.1%	0.983	AAL94932.1| Methyltransferase
	2	10.8%	0.990	3	14.3%	1.000	8	39.0%	1.000	3	19.1%	1.000	
**FN0737**	1	10.1%	0.981	2	10.1%	0.989	2	17.4%	0.995	6	25.7%	1.000	AAL94933.1| Hypothetical protein
	1	10.1%	0.982	6	37.6%	1.000	2	17.4%	0.997	5	24.8%	1.000	
**FN0738**	12	36.5%	1.000	9	27.9%	1.000	10	21.9%	1.000	10	29.3%	1.000	AAL94934.1| Hypothetical exported 24-amino acid repeat protein
	13	33.9%	1.000	14	32.8%	1.000	11	28.8%	1.000	9	29.1%	1.000	
**FN0739**	18	61.3%	1.000	16	50.9%	1.000	16	57.1%	1.000	27	61.3%	1.000	AAL94935.1| Formiminotetrahydrofolate cyclodeaminase
	24	62.3%	1.000	18	58.0%	1.000	16	58.0%	1.000	25	61.3%	1.000	
**FN0740**	31	70.2%	1.000	21	54.5%	1.000	36	74.1%	1.000	29	65.9%	1.000	AAL94936.1| Imidazolonepropionase
	31	70.0%	1.000	41	78.7%	1.000	36	74.8%	1.000	33	74.1%	1.000	
**FN0741**	36	74.5%	1.000	29	65.7%	1.000	36	77.9%	1.000	44	76.9%	1.000	AAL94937.1| Glutamate formiminotransferase
	32	70.7%	1.000	43	80.4%	1.000	36	77.9%	1.000	47	76.9%	1.000	
**FN0742**	8	37.2%	1.000	1	10.9%	0.984	8	34.6%	1.000	6	37.8%	1.000	AAL94938.1| unknown
	9	42.9%	1.000	10	49.4%	1.000	9	50.0%	1.000	6	37.8%	1.000	
**FN0743**	5	8.4%	1.000	2	5.4%	0.595	3	6.1%	0.999	10	13.8%	1.000	AAL94939.1| ATP-dependent helicase, DinG family
	5	9.7%	1.000	0	0.0%	0.000	4	8.4%	0.997	6	8.8%	1.000	
**FN0745**	22	31.6%	1.000	10	18.6%	1.000	35	42.6%	1.000	24	40.4%	1.000	AAL94941.1| metal dependent phosphohydrolase
	24	30.8%	1.000	34	38.8%	1.000	34	43.2%	1.000	23	32.4%	1.000	
**FN0746**	4	29.0%	1.000	4	29.6%	1.000	3	24.7%	1.000	2	8.6%	1.000	AAL94942.1| Hypothetical Metal-Binding Protein
	2	20.4%	0.994	4	35.2%	1.000	6	48.1%	1.000	2	8.6%	1.000	
**FN0749**	4	12.3%	1.000	1	4.2%	0.894	0	0.0%		2	4.9%	0.984	AAL94945.1| Hypothetical protein
	3	10.9%	0.999	2	7.9%	0.913	0	0.0%		3	13.8%	1.000	

Show detected proteins only
Show all proteins
Filter by category:
GO: amino acid transport

Proteins found: 1424

Enter (or paste) list of ORFs

Find ORFs

Dot Plots Dot Plots

ORF	*Fn* n	*Fn* coverage	*Fn* prob	*FnPg* n	*FnPg* coverage	*FnPg* prob	*FnSg* n	*FnSg* coverage	*FnSg* prob	*FnPgSg* n	*FnPgSg* coverage	*FnPgSg* prob	Description	*Fn* Rep I	*Fn* Rep II	*FnPg* Rep I	*FnPg* Rep II	*FnSg* Rep I	*FnSg* Rep II	*FnPgSg* Rep I	*FnPgSg* Rep II
FN0750	8	39.1%	1.000	4	24.7%	1.000	9	38.3%	1.000	3	16.0%	1.000	AAL94946.1| Hypothetical protein								
	8	35.4%	1.000	9	36.6%	1.000	10	38.7%	1.000	4	22.2%	1.000									
FN0751	4	18.2%	1.000	5	21.4%	1.000	6	28.9%	1.000	6	21.1%	1.000	AAL94947.1| L-asparaginase I								
	5	21.1%	1.000	11	50.0%	1.000	8	33.3%	1.000	6	28.9%	1.000									
FN0752	5	10.7%	1.000	3	15.7%	1.000	9	28.5%	1.000	6	19.7%	1.000	AAL94948.1| Proline iminopeptidase								
	7	23.5%	1.000	9	26.0%	1.000	7	24.5%	1.000	3	12.9%	1.000									
FN0753	14	33.3%	1.000	9	21.0%	1.000	13	30.6%	1.000	24	49.5%	1.000	AAL94949.1| Glutamyl-tRNA(Gln) amidotransferase subunit B								
	18	40.3%	1.000	23	52.8%	1.000	17	39.5%	1.000	21	47.6%	1.000									
FN0754	23	48.5%	1.000	19	41.7%	1.000	22	53.2%	1.000	23	48.3%	1.000	AAL94950.1| Glutamyl-tRNA(Gln) amidotransferase subunit A								
	23	46.6%	1.000	25	51.1%	1.000	24	50.3%	1.000	21	48.7%	1.000									
FN0755	9	79.2%	1.000	5	56.2%	1.000	7	79.2%	1.000	6	68.8%	1.000	AAL94951.1| Glutamyl-tRNA(Gln) amidotransferase subunit C								
	10	79.2%	1.000	7	79.2%	1.000	6	68.8%	1.000	9	79.2%	1.000									
FN0758	22	40.8%	1.000	9	25.2%	1.000	23	48.4%	1.000	19	45.9%	1.000	AAL94954.1| Rod shape-determining protein mreB								
	21	36.0%	1.000	21	44.5%	1.000	27	54.1%	1.000	17	49.6%	1.000									
FN0761	8	32.0%	1.000	1	7.4%	0.766	10	52.7%	1.000	4	17.2%	1.000	AAL94957.1| Bvg accessory factor								
	8	42.6%	1.000	11	55.1%	1.000	6	34.0%	1.000	4	16.8%	1.000									
FN0765	5	20.7%	1.000	2	8.8%	0.970	1	6.9%	0.465	3	12.2%	0.979	AAL94961.1| tRNA (5-methylaminomethyl-2-thiouridylate) -methyltransferase								
	6	30.7%	1.000	4	15.5%	1.000	3	14.1%	0.980	1	3.6%	0.986									
FN0766	3	14.8%	0.999	1	6.3%	0.424	2	14.8%	0.988	2	16.2%	1.000	AAL94962.1| Large-conductance mechanosensitive channel								
	3	24.6%	1.000	2	14.8%	0.990	2	14.8%	1.000	4	24.6%	1.000									
FN0768	5	10.5%	1.000	0	0.0%	0.000	1	1.7%	0.304	3	4.1%	0.983	AAL94964.1| Hemin receptor								
	7	14.5%	1.000	4	12.3%	0.980	1	3.6%	0.504	1	1.1%	0.553									
FN0771	2	4.6%	0.972	1	1.9%	0.674	3	4.6%	0.998	0	0.0%	0.000	AAL94967.1| Oxygen-independent coproporphyrinogen III oxidase								
	4	9.2%	1.000	0	0.0%	0.000	3	4.6%	1.000	1	1.9%	0.816									
FN0772	5	23.7%	1.000	0	0.0%	0.000	0	0.0%		1	7.1%	0.525	AAL94968.1| Flavodoxin								
	3	14.8%	0.999	2	12.4%	0.922	0	0.0%		2	12.4%	0.937									
FN0774	19	60.9%	1.000	10	48.0%	1.000	8	43.5%	1.000	18	63.7%	1.000	AAL94970.1| Hypothetical cytosolic protein								
	16	60.5%	1.000	12	52.4%	1.000	5	23.8%	1.000	14	60.5%	1.000									
FN0775	32	61.8%	1.000	25	49.2%	1.000	26	58.3%	1.000	30	65.5%	1.000	AAL94971.1| Aspartyl aminopeptidase								
	33	62.2%	1.000	38	74.4%	1.000	29	66.4%	1.000	32	61.5%	1.000									
FN0776	17	52.6%	1.000	11	42.2%	1.000	17	58.4%	1.000	16	46.5%	1.000	AAL94972.1| Aspartate-ammonia ligase								
	16	52.0%	1.000	17	59.0%	1.000	19	61.2%	1.000	13	41.9%	1.000									

Show detected proteins only
Show all proteins
Filter by category:
GO: amino acid transport

Proteins found: 1424

Enter (or paste) list of ORFs

Find ORFs

Dot Plots Dot Plots

ORF	*Fn* n	*Fn* coverage	*Fn* prob	*FnPg* n	*FnPg* coverage	*FnPg* prob	*FnSg* n	*FnSg* coverage	*FnSg* prob	*FnPgSg* n	*FnPgSg* coverage	*FnPgSg* prob	Description
FN0777	15	32.5%	1.000	10	24.0%	1.000	20	41.6%	1.000	19	43.9%	1.000	AAL94973.1| GTP-binding protein lepA
	14	30.8%	1.000	16	33.1%	1.000	15	39.6%	1.000	18	39.1%	1.000	
FN0778	8	15.5%	1.000	5	16.7%	1.000	5	13.1%	1.000	2	3.4%	0.998	AAL94974.1| Methyltransferase
	9	21.1%	1.000	10	22.1%	1.000	7	18.2%	1.000	5	10.2%	1.000	
FN0779	1	3.7%	0.278	0	0.0%	0.000	4	20.7%	1.000	1	3.7%	0.989	AAL94975.1| Putative GTPases (G3E family)
	0	0.0%	0.000	3	11.6%	0.994	4	23.5%	1.000	1	3.7%	0.985	
FN0783	41	74.3%	1.000	34	63.3%	1.000	28	74.3%	1.000	40	70.3%	1.000	AAL94979.1| acyl-CoA dehydrogenase
	42	74.8%	1.000	37	78.5%	1.000	32	80.1%	1.000	40	71.7%	1.000	
FN0784	36	69.8%	1.000	23	60.3%	1.000	26	80.9%	1.000	36	72.5%	1.000	AAL94980.1| Electron transfer flavoprotein beta-subunit
	33	66.4%	1.000	29	84.4%	1.000	28	85.5%	1.000	33	64.1%	1.000	
FN0785	40	55.2%	1.000	29	61.9%	1.000	35	75.2%	1.000	40	66.2%	1.000	AAL94981.1| Electron transfer flavoprotein alpha-subunit
	39	54.0%	1.000	33	63.2%	1.000	35	75.7%	1.000	38	54.2%	1.000	
FN0788	8	46.8%	1.000	7	40.3%	1.000	4	34.5%	1.000	8	56.8%	1.000	AAL94984.1| unknown
	7	32.4%	1.000	9	59.0%	1.000	8	55.4%	1.000	6	46.8%	1.000	
FN0790	7	16.0%	1.000	3	7.5%	1.000	7	33.3%	1.000	4	8.5%	1.000	AAL94986.1| Xylose repressor
	5	16.5%	1.000	7	24.0%	1.000	10	29.5%	1.000	2	5.4%	0.956	
FN0791	41	63.6%	1.000	30	54.3%	1.000	33	64.3%	1.000	34	60.9%	1.000	AAL94987.1| Histidine ammonia-lyase
	41	60.3%	1.000	43	69.0%	1.000	35	64.3%	1.000	32	49.4%	1.000	
FN0792	62	63.7%	1.000	55	68.8%	1.000	64	71.5%	1.000	56	65.5%	1.000	AAL94988.1| Urocanate hydratase
	55	62.6%	1.000	67	72.5%	1.000	58	70.6%	1.000	64	73.3%	1.000	
FN0793	4	15.8%	1.000	2	6.3%	1.000	1	4.5%	0.781	2	6.3%	0.999	AAL94989.1| Sodium/glutamate symport carrier protein
	3	15.8%	1.000	3	15.8%	1.000	1	4.5%	0.985	4	15.8%	1.000	
FN0794	6	36.5%	1.000	2	20.4%	0.984	5	32.8%	1.000	3	20.4%	1.000	AAL94990.1| Hypothetical protein
	5	41.6%	1.000	7	38.7%	1.000	5	32.8%	1.000	2	14.6%	0.994	
FN0796	38	40.0%	1.000	28	30.2%	1.000	39	42.0%	1.000	39	48.3%	1.000	AAL94992.1| Pyruvate,phosphate dikinase
	33	41.1%	1.000	50	49.6%	1.000	47	50.1%	1.000	32	37.6%	1.000	
FN0798	3	5.4%	1.000	6	13.3%	1.000	12	22.9%	1.000	6	9.3%	1.000	AAL94994.1| Fructose-1,6-bisphosphatase
	7	9.1%	1.000	7	12.1%	1.000	10	24.0%	1.000	4	6.0%	1.000	
FN0799	5	13.6%	1.000	6	18.6%	1.000	6	14.7%	1.000	5	10.5%	1.000	AAL94995.1| Isoamylase
	3	7.6%	1.000	7	14.1%	1.000	10	24.2%	1.000	2	5.6%	0.996	
FN0800	7	52.6%	1.000	3	20.9%	0.999	9	44.8%	1.000	9	47.0%	1.000	AAL94996.1| Amino acid-binding protein
	9	46.5%	1.000	11	53.5%	1.000	10	44.8%	1.000	11	47.4%	1.000	

Show detected proteins only
Show all proteins
Filter by category:
GO: amino acid transport

Proteins found: 1424

Enter (or paste) list of ORFs

Find ORFs

Dot Plots Dot Plots

ORF	*Fn* n	*Fn* coverage	*Fn* prob	*FnPg* n	*FnPg* coverage	*FnPg* prob	*FnSg* n	*FnSg* coverage	*FnSg* prob	*FnPgSg* n	*FnPgSg* coverage	*FnPgSg* prob	Description
FN0801	4	18.2%	1.000	0	0.0%	0.000	7	36.0%	1.000	1	9.9%	0.988	AAL94997.1| Amino acid transport ATP-binding protein
	4	27.3%	1.000	7	45.5%	1.000	7	43.4%	1.000	2	15.3%	0.992	
FN0802	2	8.9%	0.503	0	0.0%	0.000	2	8.9%	1.000	0	0.0%	0.000	AAL94998.1| Amino acid transport system permease protein
	2	8.9%	0.984	4	19.1%	1.000	2	9.3%	0.997	1	4.2%	0.986	
FN0803	27	44.0%	1.000	10	22.0%	1.000	13	20.3%	1.000	19	26.8%	1.000	AAL94999.1| Cytochrome C-TYPE biogenesis protein ccdA
	22	39.3%	1.000	21	37.6%	1.000	19	34.5%	1.000	20	32.6%	1.000	
FN0805	2	9.6%	0.998	4	12.7%	1.000	6	23.9%	1.000	3	13.5%	1.000	AAL95001.1| Hypothetical protein
	3	13.1%	0.999	4	18.7%	1.000	6	27.5%	1.000	3	14.3%	0.992	
FN0806	18	61.3%	1.000	10	39.0%	1.000	17	57.7%	1.000	19	62.2%	1.000	AAL95002.1| SpoIID homolog
	18	52.0%	1.000	19	64.3%	1.000	16	57.1%	1.000	17	62.5%	1.000	
FN0807	10	38.8%	1.000	4	15.9%	1.000	6	29.4%	1.000	12	45.3%	1.000	AAL95003.1| 3-deoxy-manno-octulosonate cytidylyltransferase
	8	29.4%	1.000	12	43.3%	1.000	6	23.7%	1.000	8	38.8%	1.000	
FN0808	13	55.3%	1.000	8	35.4%	1.000	15	62.1%	1.000	13	55.3%	1.000	AAL95004.1| Phosphoglycerate mutase
	11	49.5%	1.000	15	68.0%	1.000	16	62.1%	1.000	11	52.9%	1.000	
FN0809	5	50.0%	1.000	2	22.7%	0.999	2	23.3%	1.000	3	32.0%	1.000	AAL95005.1| 23S rRNA methyltransferase
	4	45.3%	1.000	3	31.3%	0.998	5	40.7%	1.000	2	23.3%	0.996	
FN0810	10	27.4%	1.000	9	32.9%	1.000	4	17.9%	1.000	7	26.2%	1.000	AAL95006.1| Low-specificity threonine aldolase
	10	32.9%	1.000	17	45.6%	1.000	11	35.0%	1.000	8	31.5%	1.000	
FN0811	1	3.8%	0.559	1	5.7%	0.359	4	18.3%	1.000	0	0.0%	0.000	AAL95007.1| Hypothetical protein
	1	3.8%	0.435	2	7.3%	0.977	3	14.5%	0.999	1	2.2%	0.387	
FN0813	5	17.7%	1.000	0	0.0%	0.000	16	47.3%	1.000	7	19.6%	1.000	AAL95009.1| Transcriptional regulator, TetR family
	5	12.6%	1.000	13	33.9%	1.000	14	41.1%	1.000	3	9.4%	1.000	
FN0814	28	46.2%	1.000	10	24.4%	1.000	6	14.4%	1.000	20	42.0%	1.000	AAL95010.1| Propionate CoA-transferase
	26	46.2%	1.000	5	12.3%	1.000	7	14.8%	1.000	23	47.9%	1.000	
FN0815	6	15.9%	1.000	1	1.8%	0.984	0	0.0%	0.000	8	17.7%	1.000	AAL95011.1| Propionate permease
	6	15.2%	1.000	0	0.0%	0.000	2	8.9%	0.931	9	17.7%	1.000	
FN0816	11	71.6%	1.000	6	58.2%	1.000	0	0.0%	0.000	8	59.7%	1.000	AAL95012.1| dehydrogenase with MaoC-like domain
	11	64.9%	1.000	1	6.7%	0.885	1	8.2%	0.967	8	44.0%	1.000	
FN0818	8	41.8%	1.000	7	28.6%	1.000	3	28.6%	1.000	7	28.6%	1.000	AAL95014.1| DNA-binding protein HU
	8	41.8%	1.000	5	40.7%	1.000	4	28.6%	1.000	7	28.6%	1.000	
FN0819	16	24.7%	1.000	17	26.3%	1.000	16	28.1%	1.000	16	27.5%	1.000	AAL95015.1| Tetratricopeptide repeat family protein
	19	26.9%	1.000	13	17.7%	1.000	19	30.8%	1.000	15	26.2%	1.000	

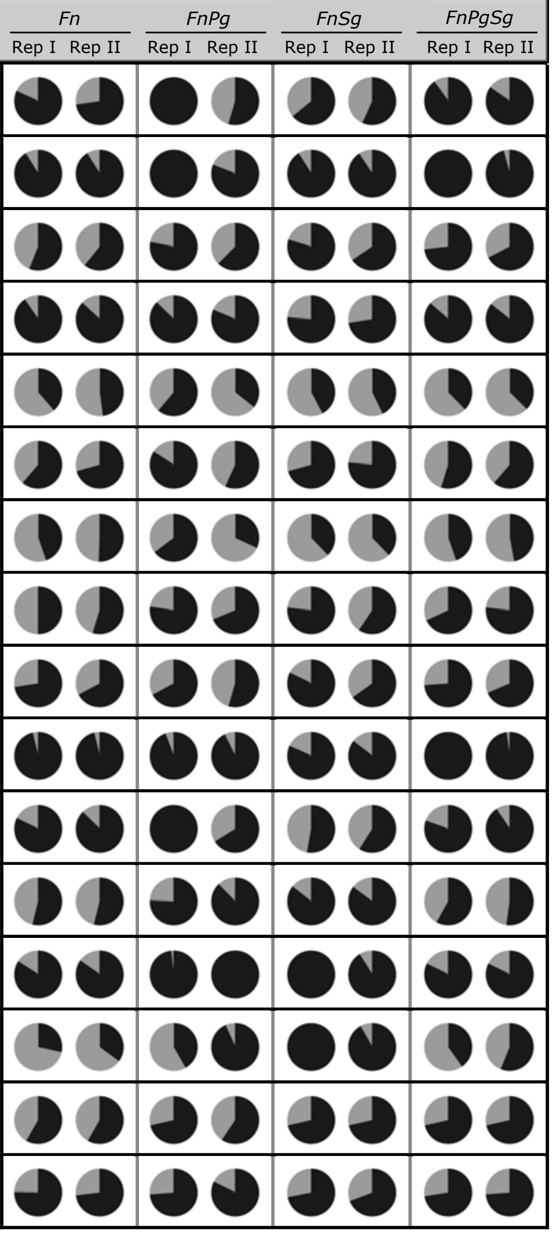

Show detected proteins only
Show all proteins
Filter by category:
GO: amino acid transport

Proteins found: 1424

Enter (or paste) list of ORFs

Find ORFs

Dot Plots Dot Plots

ORF	*Fn* n	*Fn* coverage	*Fn* prob	*FnPg* n	*FnPg* coverage	*FnPg* prob	*FnSg* n	*FnSg* coverage	*FnSg* prob	*FnPgSg* n	*FnPgSg* coverage	*FnPgSg* prob	Description
FN0820	21	44.2%	1.000	16	33.1%	1.000	18	37.9%	1.000	21	48.8%	1.000	AAL95016.1| Mercuric reductase
	23	48.4%	1.000	33	63.2%	1.000	24	49.2%	1.000	21	48.4%	1.000	
FN0821	11	36.8%	1.000	5	27.8%	1.000	8	23.6%	1.000	11	34.0%	1.000	AAL95017.1| Hypothetical protein
	11	27.4%	1.000	8	31.9%	1.000	6	24.7%	1.000	14	34.0%	1.000	
FN0823	6	17.2%	1.000	5	14.8%	1.000	25	44.2%	1.000	11	21.0%	1.000	AAL95019.1| GTP-binding protein hflX
	9	24.2%	1.000	15	29.3%	1.000	24	43.7%	1.000	8	21.0%	1.000	
FN0824	0	0.0%	0.000	0	0.0%	0.000	4	23.7%	1.000	1	4.6%	0.447	AAL95020.1| hypothetical cytosolic protein
	2	8.8%	0.957	1	3.9%	0.382	1	6.4%	0.351	1	3.9%	0.329	
FN0825	17	42.4%	1.000	6	23.9%	1.000	12	31.5%	1.000	11	26.8%	1.000	AAL95021.1| Hypothetical cytosolic protein
	17	44.9%	1.000	19	48.8%	1.000	12	36.6%	1.000	9	29.8%	1.000	
FN0826	12	49.4%	1.000	7	19.2%	1.000	7	30.8%	1.000	6	33.7%	1.000	AAL95022.1| periplasmic component of efflux system
	18	64.5%	1.000	6	25.4%	1.000	4	18.9%	1.000	9	39.6%	1.000	
FN0827	13	60.0%	1.000	3	23.6%	1.000	11	52.3%	1.000	6	41.4%	1.000	AAL95023.1| ABC transporter ATP-binding protein
	11	52.3%	1.000	6	37.3%	1.000	10	55.5%	1.000	7	40.9%	1.000	
FN0828	7	17.6%	1.000	2	7.6%	0.999	4	12.7%	1.000	3	7.4%	1.000	AAL95024.1| ABC transporter permease protein
	4	14.7%	1.000	2	6.1%	1.000	3	10.0%	1.000	2	5.1%	0.992	
FN0830	8	18.2%	1.000	14	27.6%	1.000	17	34.7%	1.000	8	18.2%	1.000	AAL95026.1| Hypothetical protein
	13	29.5%	1.000	18	40.6%	1.000	20	41.2%	1.000	7	19.5%	1.000	
FN0831	0	0.0%		5	6.9%	0.999	1	2.1%	0.947	1	2.0%	0.338	AAL95027.1| Hemin receptor
	0	0.0%		4	11.7%	0.995	2	4.4%	0.776	2	5.3%	0.713	
FN0832	15	79.2%	1.000	10	62.3%	1.000	4	40.9%	1.000	19	79.9%	1.000	AAL95028.1| Hypothetical protein
	14	70.1%	1.000	8	59.1%	1.000	10	70.1%	1.000	11	70.8%	1.000	
FN0833	17	39.5%	1.000	2	7.6%	1.000	14	33.2%	1.000	7	15.4%	1.000	AAL95029.1| Hypothetical protein
	17	37.1%	1.000	4	10.0%	1.000	11	28.6%	1.000	10	22.8%	1.000	
FN0834	12	23.9%	1.000	13	29.4%	1.000	5	14.1%	1.000	11	25.0%	1.000	AAL95030.1| Hypothetical Exported Protein
	15	28.2%	1.000	4	8.6%	1.000	9	14.5%	1.000	9	19.0%	1.000	
FN0835	1	9.1%	0.872	5	19.2%	1.000	1	6.1%	0.983	1	4.0%	0.971	AAL95031.1| Hypothetical protein
	2	15.2%	0.965	3	15.7%	1.000	2	11.6%	0.998	0	0.0%	0.000	
FN0836	7	40.8%	1.000	10	53.1%	1.000	5	46.9%	1.000	4	30.6%	1.000	AAL95032.1| Hypothetical protein
	6	40.8%	1.000	8	48.0%	1.000	5	31.6%	1.000	9	57.1%	1.000	
FN0837	1	2.7%	0.258	0	0.0%	0.000	2	6.1%	0.902	2	5.2%	0.987	AAL95033.1| Integrase/recombinase
	0	0.0%	0.000	2	5.8%	0.992	3	7.6%	0.973	0	0.0%	0.000	

Fn Rep I Rep II | *FnPg* Rep I Rep II | *FnSg* Rep I Rep II | *FnPgSg* Rep I Rep II

Show detected proteins only
Show all proteins
Filter by category:
GO: amino acid transport

Proteins found: 1424

Enter (or paste) list of ORFs

Find ORFs

Dot Plots Dot Plots

ORF	Fn n	Fn coverage	Fn prob	FnPg n	FnPg coverage	FnPg prob	FnSg n	FnSg coverage	FnSg prob	FnPgSg n	FnPgSg coverage	FnPgSg prob	Description
FN0846	20	56.2%	1.000	12	37.8%	1.000	15	47.7%	1.000	14	44.1%	1.000	AAL95042.1| Hypothetical Exported Protein
	19	55.3%	1.000	26	70.7%	1.000	15	47.7%	1.000	15	49.7%	1.000	
FN0847	11	26.5%	1.000	1	2.3%	0.983	5	10.2%	1.000	5	12.4%	1.000	AAL95043.1| TPR-repeat-containing proteins
	11	20.7%	1.000	6	15.5%	1.000	7	18.5%	1.000	5	7.3%	1.000	
FN0848	2	9.8%	0.924	3	25.5%	1.000	1	6.9%	0.985	1	3.4%	0.352	AAL95044.1| Hypothetical protein
	1	4.9%	0.589	0	0.0%	0.000	1	6.9%	0.777	0	0.0%	0.000	
FN0849	11	28.1%	1.000	7	24.1%	1.000	11	40.2%	1.000	7	20.2%	1.000	AAL95045.1| 8-amino-7-oxononanoate synthase
	8	21.8%	1.000	12	39.1%	1.000	14	38.6%	1.000	7	17.1%	1.000	
FN0850	5	21.4%	1.000	6	19.9%	1.000	10	46.4%	1.000	7	39.8%	1.000	AAL95046.1| Hypothetical cytosolic protein
	7	32.1%	1.000	9	45.4%	1.000	12	61.2%	1.000	4	19.9%	1.000	
FN0853	14	31.2%	1.000	17	30.4%	1.000	21	40.6%	1.000	15	31.7%	1.000	AAL95049.1| Glycogen synthase
	14	33.6%	1.000	23	43.8%	1.000	24	41.2%	1.000	15	33.4%	1.000	
FN0854	17	53.2%	1.000	17	48.6%	1.000	21	55.8%	1.000	18	55.3%	1.000	AAL95050.1| Glucose-1-phosphate adenylyltransferase
	18	53.0%	1.000	21	58.9%	1.000	20	53.0%	1.000	14	47.8%	1.000	
FN0855	21	58.3%	1.000	19	50.3%	1.000	29	68.0%	1.000	27	56.5%	1.000	AAL95051.1| Glucose-1-phosphate adenylyltransferase
	24	62.0%	1.000	36	83.9%	1.000	32	76.3%	1.000	24	53.9%	1.000	
FN0856	18	32.2%	1.000	22	28.3%	1.000	24	35.0%	1.000	17	34.4%	1.000	AAL95052.1| 1,4-alpha-glucan branching enzyme
	16	30.8%	1.000	31	41.9%	1.000	28	39.6%	1.000	13	25.4%	1.000	
FN0857	39	46.5%	1.000	30	49.7%	1.000	54	62.2%	1.000	39	56.0%	1.000	AAL95053.1| Glycogen phosphorylase
	38	48.7%	1.000	50	60.2%	1.000	52	59.3%	1.000	35	51.1%	1.000	
FN0858	9	21.5%	1.000	10	24.9%	1.000	16	38.3%	1.000	3	8.7%	1.000	AAL95054.1| 4-alpha-glucanotransferase
	11	27.1%	1.000	17	43.3%	1.000	24	48.8%	1.000	6	19.8%	1.000	
FN0865	13	56.0%	1.000	3	9.1%	1.000	18	60.2%	1.000	4	15.8%	1.000	AAL95061.1| unknown
	9	30.3%	1.000	22	71.8%	1.000	18	55.2%	1.000	7	26.6%	1.000	
FN0867	35	34.5%	1.000	8	10.0%	1.000	12	15.1%	1.000	24	26.0%	1.000	AAL95063.1| Long-chain-fatty-acid--CoA ligase
	30	27.6%	1.000	13	11.3%	1.000	20	22.2%	1.000	22	24.2%	1.000	
FN0868	4	10.8%	1.000	1	11.9%	0.364	0	0.0%	0.000	2	7.9%	0.843	AAL95064.1| ATPases of the PP superfamily
	3	7.2%	1.000	0	0.0%	0.000	5	17.0%	1.000	2	5.8%	0.859	
FN0869	2	14.8%	0.999	2	10.4%	0.993	4	17.0%	1.000	3	11.5%	0.998	AAL95065.1| Hydrolase (HAD superfamily)
	3	20.0%	1.000	4	21.5%	1.000	4	16.3%	1.000	2	7.0%	0.996	
FN0870	2	7.5%	0.815	2	11.2%	0.989	0	0.0%	0.000	6	20.8%	1.000	AAL95066.1| Rhodanese-related sulfurtransferases
	3	7.5%	0.976	1	5.8%	0.983	1	5.8%	0.883	6	21.7%	1.000	

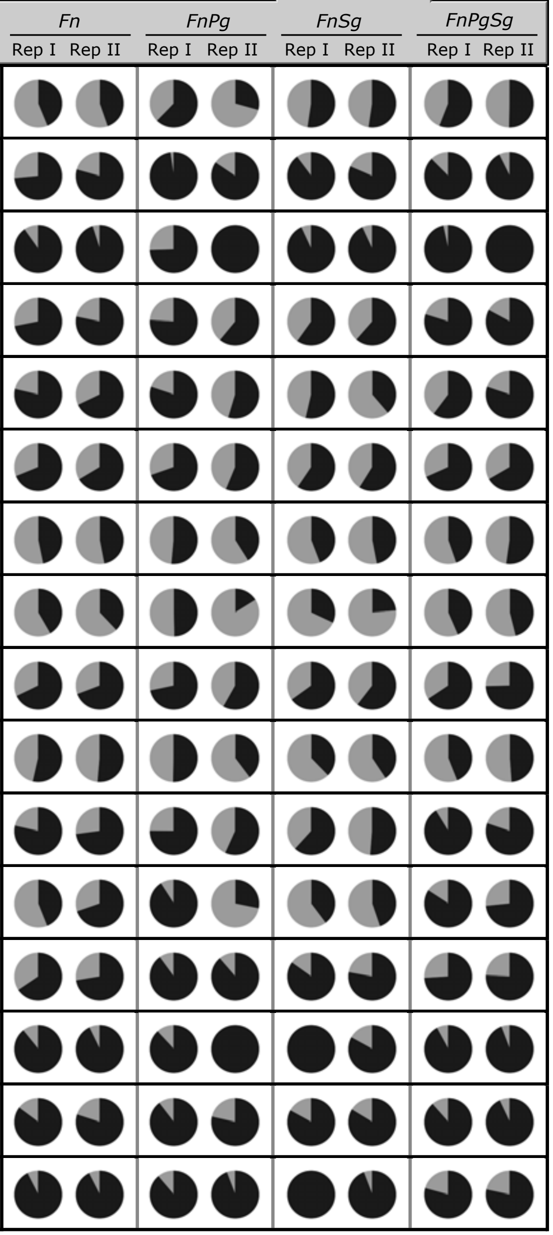

- Show detected proteins only
- Show all proteins
- Filter by category:

GO: amino acid transport

Proteins found:
1424

Enter (or paste) list of ORFs

Find ORFs

Dot Plots Dot Plots

ORF	*Fn* n	*Fn* coverage	*Fn* prob	*FnPg* n	*FnPg* coverage	*FnPg* prob	*FnSg* n	*FnSg* coverage	*FnSg* prob	*FnPgSg* n	*FnPgSg* coverage	*FnPgSg* prob	Description
FN0871	3	4.2%	0.999	2	4.4%	0.706	5	11.6%	1.000	6	11.3%	1.000	AAL95067.1| 3-dehydroquinate synthase
	3	6.5%	0.994	5	9.0%	1.000	6	14.0%	1.000	0	0.0%	0.000	
FN0873	11	26.1%	1.000	9	19.4%	1.000	16	34.2%	1.000	8	21.3%	1.000	AAL95069.1| Protease IV
	11	25.3%	1.000	24	52.0%	1.000	13	27.1%	1.000	7	15.6%	1.000	
FN0874	1	7.0%	0.744	0	0.0%	0.000	0	0.0%	0.000	0	0.0%		AAL95070.1| Phosphohydrolase (MUTT/NUDIX family protein)
	1	7.0%	0.936	6	24.0%	1.000	3	26.3%	0.999	0	0.0%		
FN0875	1	8.8%	0.582	1	7.7%	0.396	3	13.0%	0.985	4	18.8%	0.940	AAL95071.1| 23S rRNA methyltransferase
	0	0.0%	0.000	2	7.7%	0.600	2	7.7%	0.885	1	8.0%	0.402	
FN0878	6	28.5%	1.000	3	17.9%	1.000	8	30.6%	1.000	5	18.7%	1.000	AAL95074.1| Transcriptional regulator, GntR family
	6	26.0%	1.000	8	38.3%	1.000	7	25.5%	1.000	9	39.1%	1.000	
FN0884	1	7.5%	0.340	1	6.7%	0.449	3	18.6%	0.733	1	6.7%	0.543	AAL95079.1| Hemin transport system permease protein hmuU
	0	0.0%	0.000	2	13.9%	0.872	0	0.0%	0.000	1	6.7%	0.958	
FN0885	2	10.1%	0.998	0	0.0%	0.000	2	10.5%	0.934	1	4.5%	0.350	AAL95081.1| Hemin-binding periplasmic protein hmuT precursor
	3	18.9%	0.999	0	0.0%	0.000	0	0.0%	0.000	0	0.0%	0.000	
FN0886	8	13.3%	1.000	1	2.0%	0.473	2	3.8%	0.987	3	8.8%	1.000	AAL95082.1| Hemin receptor
	6	10.8%	1.000	1	1.4%	0.951	0	0.0%	0.000	2	3.3%	0.997	
FN0887	13	21.2%	1.000	6	13.8%	1.000	10	22.7%	1.000	14	25.3%	1.000	AAL95083.1| Oligoendopeptidase F
	14	21.5%	1.000	14	23.3%	1.000	11	24.7%	1.000	14	18.3%	1.000	
FN0888	4	20.9%	1.000	3	16.2%	1.000	2	13.8%	1.000	3	13.8%	1.000	AAL95084.1| Uracil permease
	3	15.7%	1.000	3	18.2%	1.000	2	13.8%	1.000	3	13.8%	1.000	
FN0889	4	56.7%	1.000	3	29.9%	1.000	2	17.3%	1.000	2	18.1%	0.994	AAL95085.1| hypothetical protein
	3	34.6%	0.997	5	44.1%	1.000	5	35.4%	1.000	2	18.1%	0.994	
FN0892	1	3.6%	0.757	0	0.0%	0.000	5	17.8%	1.000	2	4.0%	0.970	AAL95088.1| Phosphoserine phosphatase
	0	0.0%	0.000	2	4.0%	0.991	4	18.6%	1.000	1	4.0%	0.320	
FN0893	7	50.0%	1.000	1	8.6%	0.694	2	26.4%	0.997	6	37.1%	1.000	AAL95089.1| Hypothetical protein
	5	39.3%	1.000	3	39.3%	1.000	3	26.4%	1.000	3	17.9%	1.000	
FN0896	5	19.7%	1.000	3	19.7%	1.000	3	27.6%	1.000	5	39.4%	1.000	AAL95092.1| Hypothetical protein
	5	26.0%	1.000	3	18.1%	0.998	4	18.1%	1.000	6	33.1%	1.000	
FN0898	2	4.5%	0.997	1	2.1%	0.943	3	5.6%	0.992	1	2.2%	0.826	AAL95094.1| Hypothetical protein
	2	4.5%	0.772	4	7.2%	0.994	4	7.9%	1.000	2	4.5%	0.535	
FN0900	4	20.8%	0.983	3	15.4%	1.000	2	10.8%	1.000	2	8.5%	0.987	AAL95096.1| Metal dependent hydrolase
	3	20.4%	0.989	3	19.6%	0.994	7	18.8%	1.000	2	15.0%	0.974	

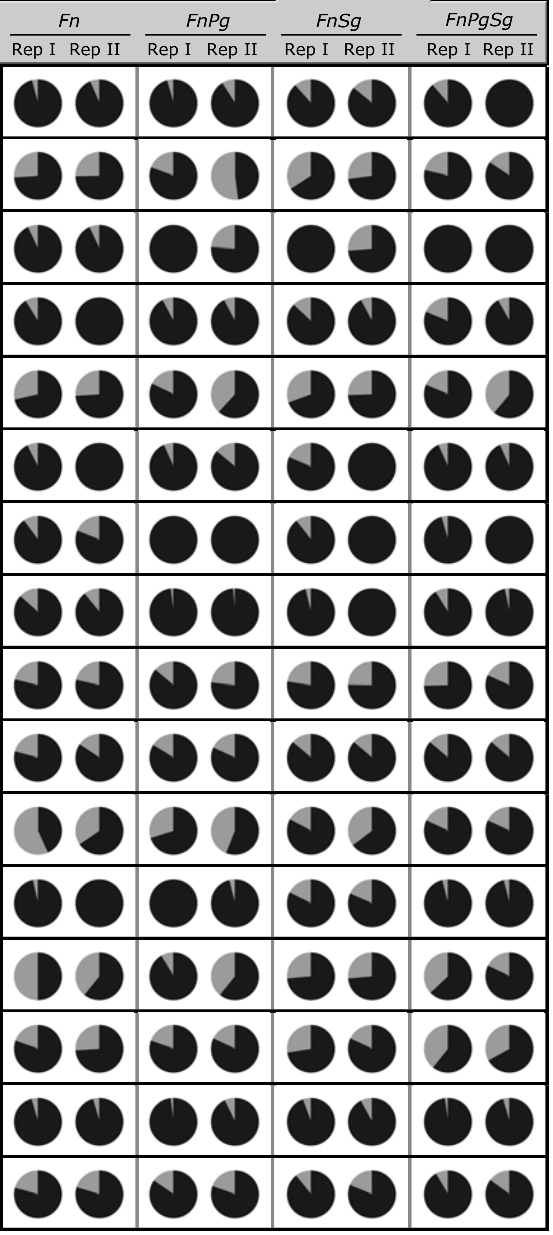

ORF	*Fn* n	*Fn* coverage	*Fn* prob	*FnPg* n	*FnPg* coverage	*FnPg* prob	*FnSg* n	*FnSg* coverage	*FnSg* prob	*FnPgSg* n	*FnPgSg* coverage	*FnPgSg* prob	Description
FN0901	1	9.7%	0.977	0	0.0%	0.000	6	40.0%	1.000	2	15.9%	0.994	AAL95097.1| DNA polymerase, bacteriophage-type
	3	20.0%	0.992	7	38.5%	1.000	8	42.1%	1.000	1	8.2%	0.365	
FN0902	2	7.7%	0.979	1	7.2%	0.274	1	6.1%	0.634	3	20.4%	0.996	AAL95098.1| 5-formyltetrahydrofolate cyclo-ligase
	1	7.7%	0.606	1	6.1%	0.282	1	6.1%	0.343	4	22.1%	0.979	
FN0903	5	16.7%	1.000	6	23.5%	1.000	6	21.7%	1.000	5	16.7%	1.000	AAL95099.1| Polysialic acid capsule expression protein kpsF
	5	18.3%	1.000	7	21.7%	1.000	9	38.1%	1.000	6	24.1%	1.000	
FN0904	2	5.9%	0.999	0	0.0%	0.000	1	2.5%	0.322	1	3.0%	0.952	AAL95100.1| NAD(FAD)-utilizing dehydrogenases
	2	4.6%	0.999	3	7.6%	1.000	0	0.0%	0.000	1	2.5%	0.691	
FN0905	4	41.6%	1.000	0	0.0%	0.000	1	9.9%	0.927	6	68.3%	1.000	AAL95101.1| Hypothetical protein
	3	33.7%	1.000	2	17.8%	0.990	1	9.9%	0.985	5	55.4%	1.000	
FN0906	11	34.6%	1.000	4	11.9%	1.000	15	47.5%	1.000	6	23.6%	1.000	AAL95102.1| Glycerol-3-phosphate dehydrogenase [NAD (P)+]
	10	31.0%	1.000	14	48.4%	1.000	13	35.8%	1.000	3	13.1%	1.000	
FN0908	8	26.3%	1.000	1	4.2%	0.936	8	25.6%	1.000	6	9.9%	1.000	AAL95104.1| Tpl protein
	9	22.4%	1.000	8	25.6%	1.000	9	25.3%	1.000	8	19.9%	1.000	
FN0909	0	0.0%	0.000	3	16.8%	1.000	0	0.0%	0.000	0	0.0%		AAL95105.1| DNA repair protein radC
	1	10.8%	0.253	2	11.2%	0.865	1	7.3%	0.985	0	0.0%		
FN0910	3	12.7%	0.987	1	3.1%	0.528	3	9.9%	0.998	1	4.0%	0.612	AAL95106.1| Nicotinate-nucleotide--dimethylbenzimidazole [illegible]
	3	9.0%	0.999	4	18.4%	1.000	6	29.4%	1.000	0	0.0%	0.000	
FN0911	3	16.2%	1.000	1	8.4%	0.970	3	16.2%	1.000	3	22.0%	1.000	AAL95107.1| Alpha-ribazole-5'-phosphate phosphatase
	5	27.7%	1.000	4	25.1%	1.000	3	25.1%	1.000	1	8.4%	0.986	
FN0912	2	10.4%	0.995	2	15.1%	0.843	3	8.6%	1.000	2	10.4%	0.997	AAL95108.1| Cobalamin [5'-phosphate] synthase
	2	10.4%	0.987	6	23.7%	1.000	4	14.4%	1.000	1	5.0%	0.986	
FN0913	3	18.2%	0.996	1	12.3%	0.938	1	4.8%	0.784	3	25.7%	1.000	AAL95109.1| Cobinamide kinase
	3	23.0%	1.000	4	27.3%	1.000	5	36.4%	1.000	1	7.0%	0.969	
FN0915	5	20.7%	1.000	5	20.7%	1.000	6	31.7%	1.000	8	40.2%	1.000	AAL95111.1| PTS system, N-acetylglucosamine-specific IIA component
	7	26.8%	1.000	9	40.9%	1.000	5	24.4%	1.000	7	34.1%	1.000	
FN0916	16	71.1%	1.000	9	53.7%	1.000	17	75.8%	1.000	16	71.1%	1.000	AAL95112.1| Hypothetical Exported Protein
	16	71.8%	1.000	16	75.8%	1.000	15	71.8%	1.000	17	71.1%	1.000	
FN0917	4	11.8%	1.000	2	8.7%	0.990	4	16.1%	1.000	1	3.1%	0.967	AAL95113.1| Hypothetical protein
	3	12.1%	0.999	3	9.0%	0.984	8	23.6%	1.000	3	9.6%	0.992	
FN0920	2	10.7%	0.999	0	0.0%	0.000	2	10.7%	0.998	5	22.0%	1.000	AAL95116.1| Protease HTPX
	4	16.2%	1.000	3	16.2%	1.000	0	0.0%	0.000	3	14.6%	1.000	

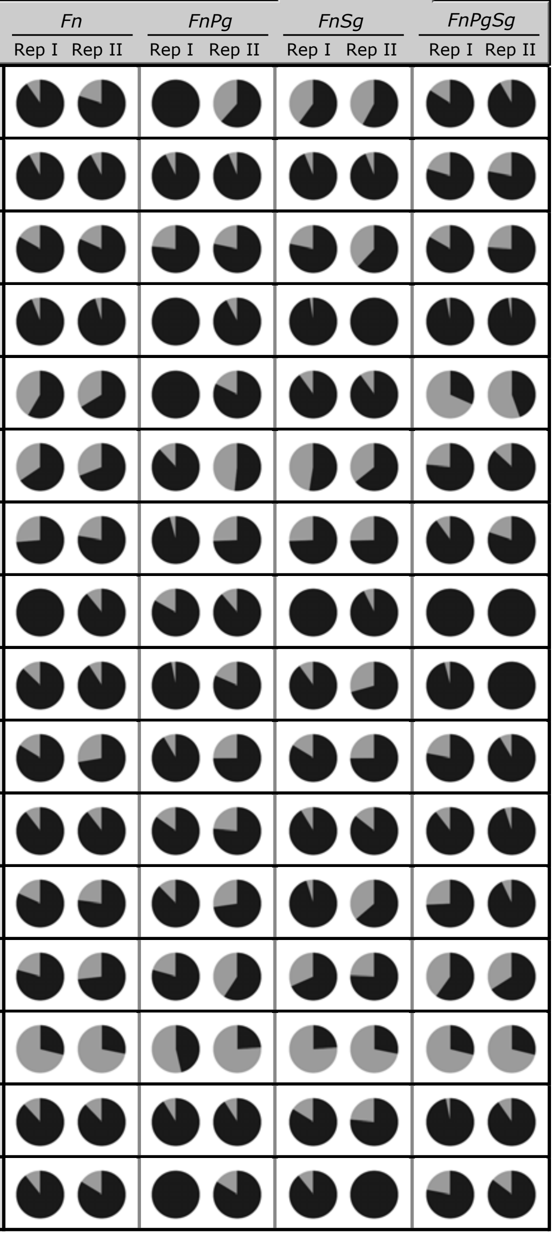

Fusobacterium nucleatum

ORF	*Fn* n	*Fn* coverage	*Fn* prob	*FnPg* n	*FnPg* coverage	*FnPg* prob	*FnSg* n	*FnSg* coverage	*FnSg* prob	*FnPgSg* n	*FnPgSg* coverage	*FnPgSg* prob	Description
**FN0921**	3	13.9%	0.999	2	3.9%	1.000	9	25.9%	1.000	4	11.3%	1.000	AAL95117.1| Hypothetical protein
	5	11.7%	1.000	10	31.7%	1.000	10	31.7%	1.000	5	15.2%	1.000	
**FN0922**	1	6.1%	0.317	0	0.0%		1	2.9%	0.741	3	14.7%	0.855	AAL95118.1| Homoserine kinase
	0	0.0%	0.000	0	0.0%		0	0.0%	0.000	0	0.0%	0.000	
**FN0923**	0	0.0%		0	0.0%	0.000	2	4.0%	0.994	0	0.0%	0.000	AAL95119.1| Cardiolipin synthetase
	0	0.0%		3	8.4%	0.974	2	3.5%	0.981	1	1.7%	0.969	
**FN0924**	1	5.3%	0.741	1	5.7%	0.290	1	5.3%	0.604	0	0.0%	0.000	AAL95120.1| Hypothetical protein
	1	5.3%	0.909	1	5.3%	0.980	1	5.3%	0.984	3	15.3%	0.936	
**FN0925**	4	17.1%	1.000	2	12.0%	0.996	3	17.1%	0.998	4	17.5%	1.000	AAL95121.1| Hypothetical protein
	3	14.0%	0.999	4	19.9%	0.999	6	26.4%	1.000	3	13.4%	0.995	
**FN0926**	3	13.5%	0.996	7	26.6%	1.000	11	42.9%	1.000	6	31.3%	1.000	AAL95122.1| GTP pyrophosphokinase
	2	11.2%	1.000	8	38.2%	1.000	12	45.9%	1.000	5	24.7%	1.000	
**FN0928**	3	24.8%	1.000	2	14.0%	0.989	3	22.9%	0.996	4	19.2%	1.000	AAL95124.1| O-sialoglycoprotein endopeptidase
	2	17.8%	0.999	7	46.3%	1.000	6	36.9%	1.000	3	20.6%	1.000	
**FN0929**	1	6.5%	0.955	4	23.5%	1.000	3	15.7%	1.000	2	15.7%	0.998	AAL95125.1| ATP/GTP hydrolase
	2	15.7%	0.998	3	15.7%	1.000	3	15.7%	1.000	3	15.7%	0.995	
**FN0930**	7	35.6%	1.000	3	36.9%	1.000	2	25.6%	0.925	1	6.9%	0.615	AAL95126.1| Glycerol-3-phosphate cytidylyltransferase
	4	30.0%	1.000	3	34.4%	0.993	3	34.4%	1.000	2	15.6%	0.905	
**FN0932**	6	19.8%	1.000	0	0.0%	0.000	3	7.8%	1.000	4	20.4%	0.999	AAL95128.1| Hypothetical protein
	7	20.4%	1.000	5	16.2%	1.000	6	22.2%	1.000	3	19.8%	0.996	
**FN0933**	0	0.0%	0.000	2	8.0%	0.989	0	0.0%	0.000	3	10.1%	0.998	AAL95129.1| 3-phosphoshikimate 1-carboxyvinyltransferase
	1	3.8%	0.979	1	4.2%	0.898	1	2.8%	0.958	1	5.9%	0.346	
**FN0934**	3	12.9%	1.000	1	4.2%	0.981	4	11.8%	1.000	1	3.4%	0.988	AAL95130.1| Chorismate synthase
	4	27.7%	1.000	7	17.9%	1.000	9	33.9%	1.000	0	0.0%	0.000	
**FN0938**	1	8.4%	0.942	0	0.0%	0.000	8	55.0%	1.000	1	4.6%	0.571	AAL95134.1| Hypothetical protein
	1	8.4%	0.982	10	60.7%	1.000	6	34.7%	1.000	1	8.4%	0.983	
**FN0940**	1	5.1%	0.295	1	13.0%	0.627	4	27.7%	1.000	5	34.5%	1.000	AAL95136.1| Hypothetical protein
	1	5.1%	0.352	5	32.8%	1.000	6	32.8%	1.000	4	16.9%	1.000	
**FN0941**	10	18.3%	1.000	12	19.7%	1.000	15	28.0%	1.000	12	23.0%	1.000	AAL95137.1| Gamma-glutamyltranspeptidase
	8	16.8%	1.000	21	37.7%	1.000	15	26.3%	1.000	14	25.4%	1.000	
**FN0943**	5	27.9%	1.000	0	0.0%	0.000	6	34.8%	1.000	5	25.0%	1.000	AAL95139.1| Sensory Transduction Protein Kinase
	4	21.6%	1.000	9	48.0%	1.000	8	38.2%	1.000	3	13.7%	1.000	

Fn Rep I / Rep II · *FnPg* Rep I / Rep II · *FnSg* Rep I / Rep II · *FnPgSg* Rep I / Rep II

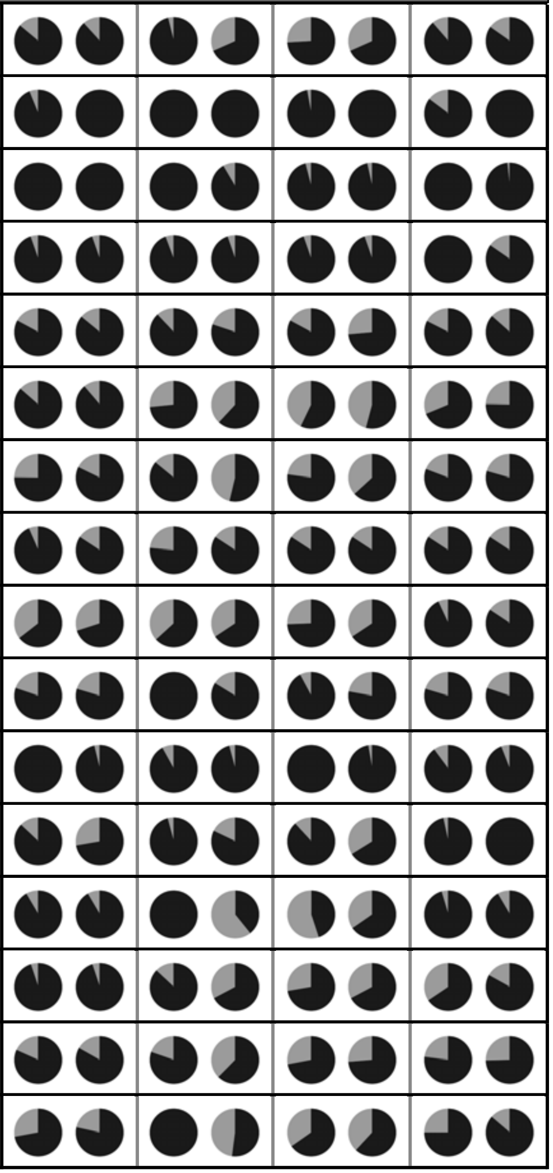

Show detected proteins only
Show all proteins
Filter by category:
GO: amino acid transport

Proteins found: 1424

Enter (or paste) list of ORFs

Find ORFs

Dot Plots Dot Plots

ORF	*Fn* n	coverage	prob	*FnPg* n	coverage	prob	*FnSg* n	coverage	prob	*FnPgSg* n	coverage	prob	Description
**FN0944**	2	7.9%	0.954	1	4.0%	0.290	1	6.8%	0.616	1	3.1%	0.854	AAL95140.1| Na+ driven multidrug efflux pump
	1	2.0%	0.975	3	16.9%	0.804	2	8.8%	0.773	1	3.1%	0.434	
**FN0947**	10	43.3%	1.000	9	30.4%	1.000	8	35.9%	1.000	10	40.7%	1.000	AAL95143.1| Hypothetical protein
	11	48.1%	1.000	9	40.7%	1.000	10	44.8%	1.000	10	40.7%	1.000	
**FN0949**	62	44.9%	1.000	35	28.7%	1.000	24	18.0%	1.000	53	34.9%	1.000	AAL95145.1| DNA helicase
	70	46.9%	1.000	19	15.1%	1.000	28	21.0%	1.000	47	32.8%	1.000	
**FN0951**	5	17.3%	1.000	3	18.9%	1.000	2	8.8%	0.998	2	11.6%	1.000	AAL95147.1| Precorrin-3B C17-methyltransferase
	6	17.3%	1.000	6	26.5%	1.000	3	15.7%	1.000	2	11.6%	1.000	
**FN0952**	0	0.0%	0.000	1	5.9%	0.631	0	0.0%	0.000	1	5.9%	0.919	AAL95148.1| Cobalamin biosynthesis protein G
	4	18.4%	1.000	1	4.2%	0.983	1	3.9%	0.363	1	5.9%	0.721	
**FN0957**	5	21.0%	1.000	6	30.4%	1.000	3	11.7%	1.000	6	28.4%	1.000	AAL95153.1| Precorrin-4 C11-methyltransferase
	4	21.4%	1.000	8	38.5%	1.000	7	29.2%	1.000	5	20.6%	1.000	
**FN0958**	7	28.8%	1.000	6	21.7%	1.000	2	15.9%	0.996	1	3.1%	0.351	AAL95154.1| unknown
	5	21.7%	1.000	1	9.3%	0.904	4	15.9%	0.999	2	9.7%	0.990	
**FN0959**	6	31.5%	1.000	6	25.4%	1.000	2	4.8%	0.989	6	27.8%	1.000	AAL95155.1| Precorrin-2 C20-methyltransferase
	6	26.2%	1.000	4	21.8%	1.000	3	13.3%	1.000	6	35.1%	1.000	
**FN0961**	3	24.7%	1.000	3	23.4%	1.000	2	11.0%	1.000	2	19.5%	0.996	AAL95157.1| Hypothetical protein
	3	24.7%	1.000	4	23.4%	1.000	3	16.2%	1.000	1	4.5%	0.800	
**FN0962**	11	49.8%	1.000	8	41.0%	1.000	5	24.7%	1.000	8	34.8%	1.000	AAL95158.1| Hypothetical cytosolic protein
	8	31.3%	1.000	9	45.4%	1.000	7	29.1%	1.000	7	31.3%	1.000	
**FN0964**	1	6.3%	0.977	3	12.7%	1.000	3	12.2%	1.000	1	6.3%	0.973	AAL95160.1| Precorrin-8W decarboxylase
	1	6.3%	0.871	4	20.1%	1.000	3	11.1%	1.000	3	11.1%	1.000	
**FN0965**	8	32.4%	1.000	10	40.2%	1.000	13	42.7%	1.000	12	32.1%	1.000	AAL95161.1| D-3-phosphoglycerate dehydrogenase
	9	37.7%	1.000	13	36.4%	1.000	13	42.1%	1.000	14	39.6%	1.000	
**FN0966**	1	4.4%	0.536	1	4.8%	0.806	2	7.4%	0.896	1	7.9%	0.967	AAL95162.1| Precorrin-6Y C5,15-methyltransferase (decarboxylating)
	0	0.0%	0.000	3	15.3%	1.000	3	15.3%	1.000	1	3.9%	0.971	
**FN0967**	2	5.3%	0.998	4	14.7%	1.000	1	4.5%	0.333	3	12.0%	0.999	AAL95163.1| CbiD protein
	2	5.3%	0.998	2	7.5%	0.627	4	14.7%	0.933	1	4.5%	0.304	
**FN0970**	4	22.8%	1.000	8	51.1%	1.000	3	12.8%	1.000	4	22.8%	1.000	AAL95166.1| Precorrin-8X methylmutase
	6	27.9%	1.000	2	12.3%	1.000	8	48.9%	1.000	4	22.8%	1.000	
**FN0971**	0	0.0%	0.000	1	4.2%	0.925	3	11.2%	0.931	0	0.0%		AAL95167.1| hypothetical cytosolic protein
	1	2.1%	0.397	0	0.0%	0.000	2	8.8%	0.738	0	0.0%		

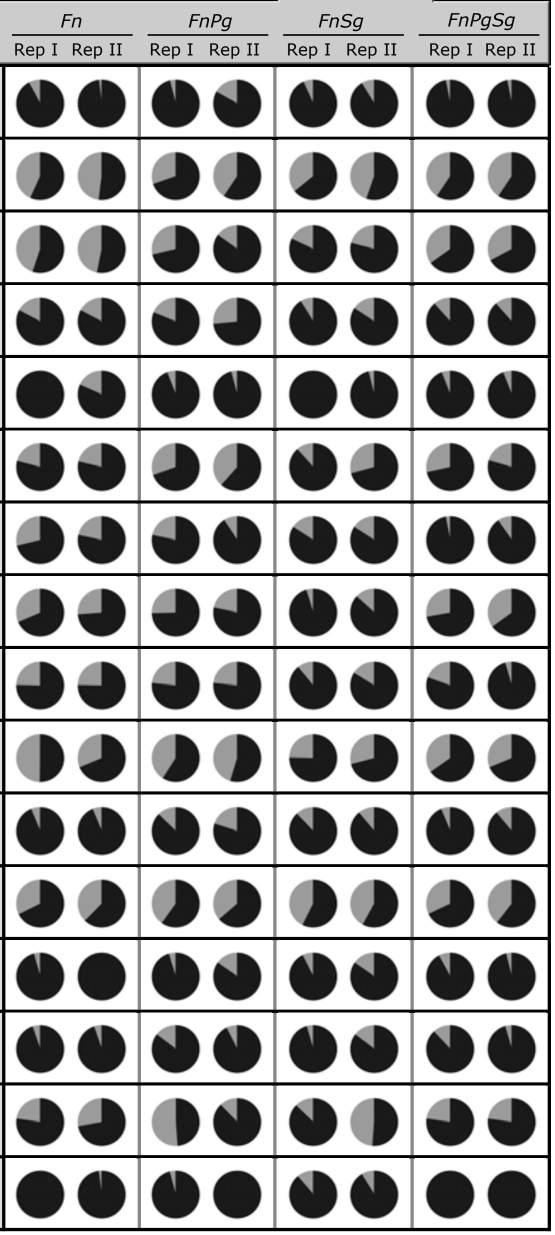

Show detected proteins only
Show all proteins
Filter by category:
GO: amino acid transport

Proteins found: 1424

Enter (or paste) list of ORFs
Find ORFs

Dot Plots Dot Plots

Fn Coverage

Fusobacterium nucleatum

Hackett Laboratory UW

ORF	*Fn* n	*Fn* coverage	*Fn* prob	*FnPg* n	*FnPg* coverage	*FnPg* prob	*FnSg* n	*FnSg* coverage	*FnSg* prob	*FnPgSg* n	*FnPgSg* coverage	*FnPgSg* prob	Description
FN0972	1	2.9%	0.372	3	11.7%	0.999	3	11.0%	1.000	1	1.8%	0.988	AAL95168.1| Cobyrinic acid a,c-diamide synthase
	1	4.1%	0.264	9	26.4%	1.000	10	30.9%	1.000	1	3.4%	0.624	
FN0974	2	19.0%	0.987	2	29.6%	0.998	1	11.3%	0.985	2	19.0%	0.956	AAL95170.1| Lactoylglutathione lyase
	2	19.0%	0.951	3	29.6%	1.000	1	11.3%	0.974	1	11.3%	0.974	
FN0976	12	44.9%	1.000	12	52.1%	1.000	10	41.3%	1.000	12	48.9%	1.000	AAL95172.1| Hypothetical protein
	13	47.5%	1.000	11	43.3%	1.000	12	53.8%	1.000	9	43.6%	1.000	
FN0977	3	14.1%	0.993	3	7.7%	0.996	9	29.4%	1.000	4	13.1%	1.000	AAL95173.1| Cobyric acid synthase
	2	8.3%	0.821	7	20.2%	1.000	9	22.0%	1.000	5	12.5%	1.000	
FN0981	21	42.0%	1.000	12	22.5%	1.000	16	39.2%	1.000	25	47.2%	1.000	AAL95177.1| Phosphoribosylamine--glycine ligase
	23	38.0%	1.000	24	50.2%	1.000	13	33.6%	1.000	22	38.7%	1.000	
FN0982	30	51.2%	1.000	19	37.3%	1.000	29	52.0%	1.000	32	53.6%	1.000	AAL95178.1| Phosphoribosylaminoimidazolecarboxamide formyltransferase
	34	52.8%	1.000	38	65.3%	1.000	35	56.7%	1.000	30	60.5%	1.000	
FN0983	13	46.1%	1.000	12	47.7%	1.000	12	46.1%	1.000	11	46.1%	1.000	AAL95179.1| Hypothetical protein
	13	46.1%	1.000	12	50.0%	1.000	13	46.1%	1.000	12	46.1%	1.000	
FN0984	7	30.1%	1.000	9	37.5%	1.000	12	38.7%	1.000	4	16.0%	1.000	AAL95180.1| Tetracenomycin polyketide synthesis O-methyltransferase tcmP
	6	30.1%	1.000	11	35.7%	1.000	11	35.7%	1.000	4	18.6%	1.000	
FN0985	4	14.4%	1.000	0	0.0%	0.000	7	42.2%	1.000	5	35.6%	1.000	AAL95181.1| Phosphoribosylglycinamide formyltransferase
	3	23.3%	1.000	3	12.2%	1.000	7	41.7%	1.000	4	23.3%	1.000	
FN0986	14	38.1%	1.000	9	37.8%	1.000	12	35.7%	1.000	18	40.4%	1.000	AAL95182.1| Phosphoribosylformylglycinamidine cyclo-ligase
	11	35.4%	1.000	18	47.8%	1.000	15	51.3%	1.000	19	42.8%	1.000	
FN0987	24	49.1%	1.000	9	26.3%	1.000	24	48.9%	1.000	18	47.5%	1.000	AAL95183.1| Amidophosphoribosyltransferase
	27	69.2%	1.000	26	56.5%	1.000	25	52.5%	1.000	20	53.1%	1.000	
FN0988	26	66.2%	1.000	22	62.9%	1.000	20	63.7%	1.000	23	65.8%	1.000	AAL95184.1| Phosphoribosylamidoimidazole-succinocarboxamide synthase
	25	57.4%	1.000	25	71.7%	1.000	25	67.9%	1.000	22	70.9%	1.000	
FN0989	16	73.9%	1.000	12	68.8%	1.000	12	73.9%	1.000	14	73.2%	1.000	AAL95185.1| Phosphoribosylaminoimidazole carboxylase catalytic subunit
	16	73.9%	1.000	15	73.9%	1.000	16	75.2%	1.000	11	73.2%	1.000	
FN0990	92	63.7%	1.000	52	46.0%	1.000	97	70.8%	1.000	98	66.3%	1.000	AAL95186.1| Phosphoribosylformylglycinamidine synthase
	83	59.0%	1.000	97	65.2%	1.000	98	66.1%	1.000	98	68.5%	1.000	
FN0991	4	18.4%	1.000	3	22.6%	1.000	4	22.6%	1.000	6	26.8%	1.000	AAL95187.1| CDP-diacylglycerol--serine O-phosphatidyltransferase
	5	18.4%	1.000	6	26.8%	1.000	4	26.8%	1.000	4	16.5%	1.000	
FN0992	14	43.9%	1.000	5	27.1%	1.000	11	45.8%	1.000	8	27.9%	1.000	AAL95188.1| ADP-heptose:LPS heptosyltransferase II
	9	28.8%	1.000	9	27.1%	1.000	14	43.9%	1.000	10	33.5%	1.000	

Fn Rep I Rep II | *FnPg* Rep I Rep II | *FnSg* Rep I Rep II | *FnPgSg* Rep I Rep II

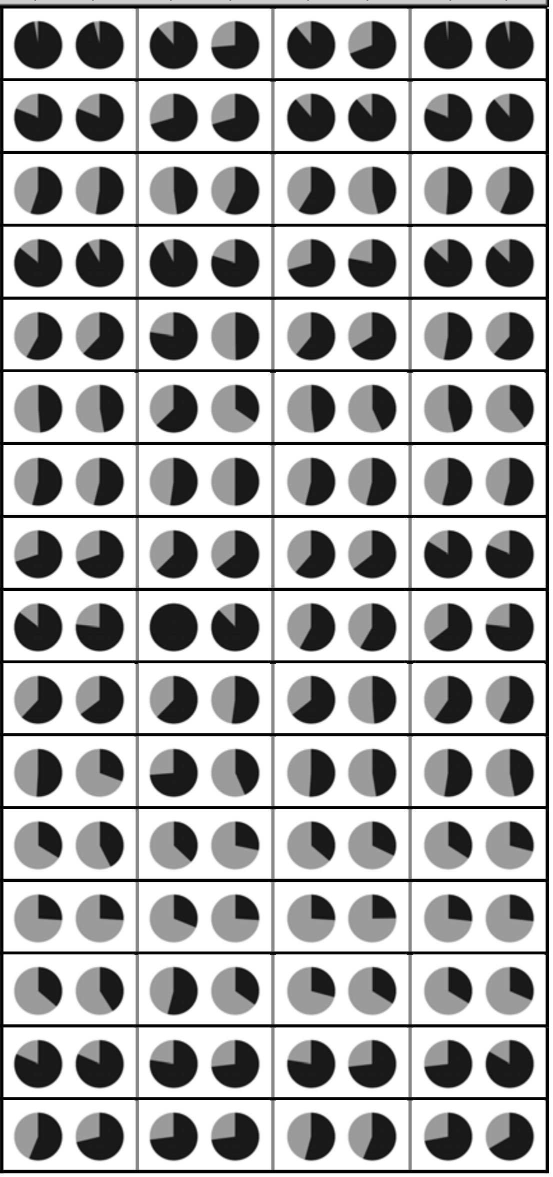

Show detected proteins only
Show all proteins
Filter by category:
GO: amino acid transport

Proteins found: 1424

Enter (or paste) list of ORFs

Find ORFs

Dot Plots Dot Plots

ORF	*Fn* n	*Fn* coverage	*Fn* prob	*FnPg* n	*FnPg* coverage	*FnPg* prob	*FnSg* n	*FnSg* coverage	*FnSg* prob	*FnPgSg* n	*FnPgSg* coverage	*FnPgSg* prob	Description
FN0994	13	43.2%	1.000	9	44.8%	1.000	13	43.2%	1.000	18	58.5%	1.000	AAL95190.1| Hypothetical protein
	14	49.4%	1.000	17	59.8%	1.000	17	46.9%	1.000	14	46.5%	1.000	
FN0997	6	28.2%	1.000	4	17.6%	1.000	16	58.8%	1.000	11	51.4%	1.000	AAL95193.1| Hypothetical protein
	6	20.4%	1.000	14	61.1%	1.000	14	53.7%	1.000	9	46.8%	1.000	
FN0998	29	49.6%	1.000	19	40.6%	1.000	33	58.2%	1.000	27	50.8%	1.000	AAL95194.1| Dipeptide-binding protein
	31	52.2%	1.000	37	62.4%	1.000	36	57.6%	1.000	25	47.2%	1.000	
FN0999	12	36.0%	1.000	3	7.2%	1.000	12	34.9%	1.000	4	14.4%	1.000	AAL95195.1| Deblocking aminopeptidase
	9	26.2%	1.000	21	51.6%	1.000	14	49.3%	1.000	4	20.5%	1.000	
FN1000	13	36.9%	1.000	4	18.1%	1.000	2	10.3%	0.915	9	40.8%	1.000	AAL95196.1| Biotin synthase
	13	45.6%	1.000	1	9.2%	0.983	2	13.9%	1.000	10	43.9%	1.000	
FN1001	9	36.5%	1.000	5	24.2%	1.000	13	57.1%	1.000	9	39.3%	1.000	AAL95197.1| Dethiobiotin synthetase
	8	30.1%	1.000	13	56.2%	1.000	16	65.8%	1.000	7	31.5%	1.000	
FN1002	18	50.9%	1.000	21	57.3%	1.000	10	29.4%	1.000	13	36.3%	1.000	AAL95198.1| Adenosylmethionine -8-amino-7-oxononanoate aminotransferase
	19	49.3%	1.000	15	43.6%	1.000	12	33.8%	1.000	15	39.8%	1.000	
FN1003	10	45.4%	1.000	4	15.8%	1.000	15	59.0%	1.000	10	42.5%	1.000	AAL95199.1| Outer membrane protein P1 precursor
	7	28.6%	1.000	25	81.7%	1.000	20	72.9%	1.000	10	42.1%	1.000	
FN1004	4	23.4%	1.000	5	27.1%	1.000	5	33.0%	1.000	5	28.7%	1.000	AAL95200.1| Transcriptional regulator, TetR family
	5	28.2%	1.000	3	18.6%	1.000	4	27.1%	1.000	4	23.4%	1.000	
FN1005	9	35.7%	1.000	1	7.6%	0.787	7	49.7%	1.000	6	35.0%	1.000	AAL95201.1| Hypothetical protein
	9	35.7%	1.000	9	43.9%	1.000	8	56.1%	1.000	12	53.5%	1.000	
FN1006	1	6.8%	0.237	1	11.4%	0.852	0	0.0%	0.000	0	0.0%		AAL95202.1| Acetyltransferase
	1	6.8%	0.451	7	47.0%	1.000	4	37.9%	1.000	0	0.0%		
FN1008	3	13.4%	0.997	1	5.6%	0.300	1	7.8%	0.985	0	0.0%	0.000	AAL95204.1| Hypothetical protein
	1	4.5%	0.259	2	8.4%	0.727	1	5.6%	0.615	1	4.5%	0.657	
FN1009	3	16.7%	1.000	0	0.0%		1	7.9%	0.903	1	8.8%	0.516	AAL95205.1| Hypothetical protein
	3	16.7%	0.990	0	0.0%		1	7.9%	0.978	0	0.0%	0.000	
FN1010	17	90.8%	1.000	10	72.4%	1.000	17	81.6%	1.000	16	89.8%	1.000	AAL95206.1| Hypothetical cytosolic protein
	18	90.8%	1.000	21	100.0%	1.000	16	90.8%	1.000	17	89.8%	1.000	
FN1011	4	16.2%	1.000	4	16.2%	1.000	5	27.1%	1.000	2	9.7%	0.999	AAL95207.1| MGPA protein
	3	16.2%	1.000	7	20.9%	1.000	6	23.7%	1.000	3	12.8%	1.000	
FN1012	8	10.2%	1.000	4	3.6%	1.000	11	16.4%	1.000	7	15.8%	1.000	AAL95208.1| HPR(Ser) kinase
	7	8.0%	1.000	12	17.9%	1.000	16	26.5%	1.000	4	6.8%	1.000	

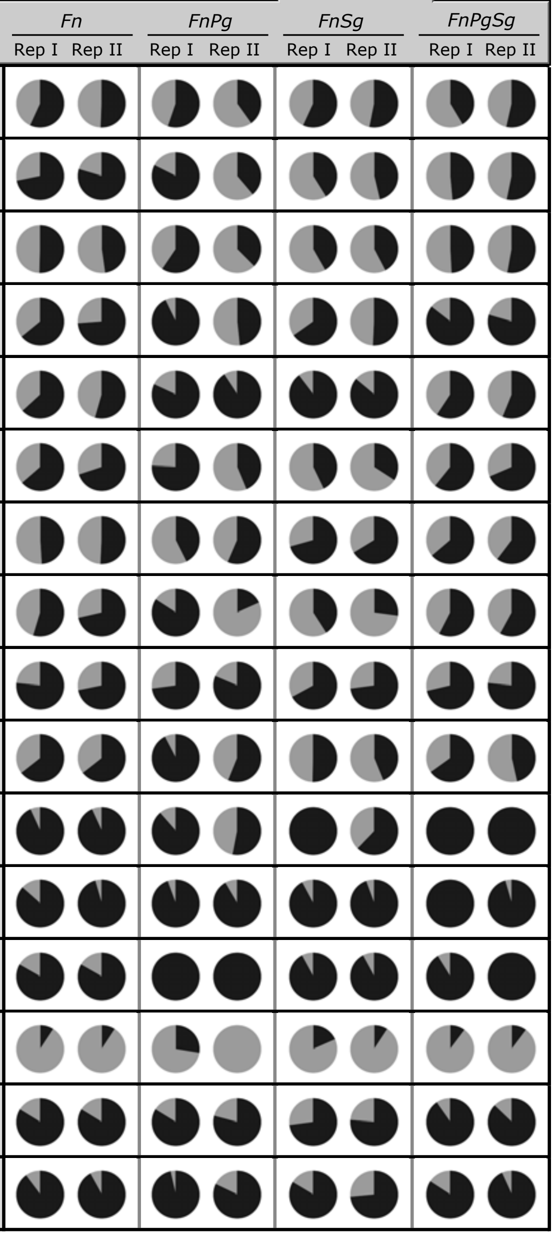

ORF	Fn n	Fn coverage	Fn prob	FnPg n	FnPg coverage	FnPg prob	FnSg n	FnSg coverage	FnSg prob	FnPgSg n	FnPgSg coverage	FnPgSg prob	Description
FN1014	9	19.0%	1.000	1	4.8%	0.363	9	28.2%	1.000	3	8.4%	1.000	AAL95210.1| Folylpolyglutamate synthase
	7	18.6%	1.000	9	24.8%	1.000	7	22.9%	1.000	5	10.8%	1.000	
FN1015	3	17.3%	1.000	1	6.3%	0.531	4	29.1%	1.000	5	30.0%	1.000	AAL95211.1| 5'-methylthioadenosine nucleosidase
	6	22.8%	1.000	8	43.5%	1.000	7	35.4%	1.000	4	23.2%	1.000	
FN1016	6	13.7%	1.000	3	9.7%	1.000	10	52.7%	1.000	3	8.8%	1.000	AAL95212.1| Lipid A biosynthesis lauroyl acyltransferase
	7	19.5%	1.000	9	35.4%	1.000	10	44.7%	1.000	3	8.8%	1.000	
FN1017	18	55.5%	1.000	11	45.6%	1.000	19	57.0%	1.000	17	55.5%	1.000	AAL95213.1| Hypothetical Exported Protein
	19	61.4%	1.000	19	66.9%	1.000	18	61.4%	1.000	17	58.8%	1.000	
FN1019	19	41.2%	1.000	22	68.1%	1.000	23	71.3%	1.000	21	58.8%	1.000	AAL95215.1| 3-hydroxybutyryl-CoA dehydrogenase
	19	41.9%	1.000	26	75.3%	1.000	23	71.3%	1.000	20	54.8%	1.000	
FN1020	14	52.7%	1.000	16	73.6%	1.000	19	77.5%	1.000	18	63.6%	1.000	AAL95216.1| 3-hydroxybutyryl-CoA dehydratase
	16	55.4%	1.000	18	64.7%	1.000	18	79.5%	1.000	19	75.2%	1.000	
FN1022	9	10.8%	1.000	2	5.6%	0.943	2	4.2%	0.947	7	8.4%	1.000	AAL95218.1| Calcium-transporting ATPase
	7	6.7%	1.000	1	0.8%	0.751	2	2.3%	0.899	6	8.5%	1.000	
FN1023	1	6.4%	0.935	2	17.3%	0.998	4	26.3%	1.000	3	30.1%	1.000	AAL95219.1| 5-Nitroimidazole antibiotic resistance protein
	2	14.7%	0.919	6	40.4%	1.000	5	34.6%	1.000	2	14.7%	0.999	
FN1024	15	54.9%	1.000	16	54.9%	1.000	15	66.7%	1.000	14	52.9%	1.000	AAL95220.1| DNA-binding protein HU
	15	54.9%	1.000	14	59.8%	1.000	14	66.7%	1.000	12	49.0%	1.000	
FN1025	3	7.1%	1.000	0	0.0%	0.000	1	2.1%	0.985	4	9.4%	1.000	AAL95221.1| Guanine-hypoxanthine permease
	4	9.0%	1.000	5	12.2%	1.000	3	7.8%	0.995	4	9.4%	1.000	
FN1026	4	14.9%	1.000	0	0.0%	0.000	2	9.3%	0.992	2	6.9%	0.961	AAL95222.1| tRNA pseudouridine synthase A
	4	14.9%	1.000	0	0.0%	0.000	5	15.2%	1.000	1	4.5%	0.982	
FN1028	11	66.4%	1.000	7	39.7%	1.000	6	49.3%	1.000	11	63.0%	1.000	AAL95224.1| Deoxyuridine 5'-triphosphate nucleotidohydrolase
	8	61.0%	1.000	8	42.5%	1.000	7	37.0%	1.000	11	62.3%	1.000	
FN1029	17	27.9%	1.000	13	41.4%	1.000	17	35.5%	1.000	20	33.3%	1.000	AAL95225.1| Zinc protease
	18	30.6%	1.000	21	41.7%	1.000	15	29.9%	1.000	15	31.6%	1.000	
FN1030	3	10.7%	0.999	0	0.0%	0.000	4	12.4%	1.000	4	17.4%	0.999	AAL95226.1| Hypothetical membrane-spanning protein
	1	3.6%	0.766	6	18.7%	1.000	4	10.7%	1.000	2	6.9%	0.992	
FN1031	5	13.1%	1.000	1	4.2%	0.890	2	6.7%	0.992	1	3.6%	0.881	AAL95227.1| Hypothetical membrane-spanning protein
	4	10.3%	1.000	5	12.8%	1.000	4	12.3%	1.000	3	10.3%	0.999	
FN1033	15	29.5%	1.000	11	17.8%	1.000	11	20.9%	1.000	11	21.4%	1.000	AAL95229.1| Methyltransferase
	15	23.4%	1.000	18	38.4%	1.000	15	38.4%	1.000	10	22.6%	1.000	

Fn: Rep I, Rep II · FnPg: Rep I, Rep II · FnSg: Rep I, Rep II · FnPgSg: Rep I, Rep II

ORF	*Fn* n	*Fn* coverage	*Fn* prob	*FnPg* n	*FnPg* coverage	*FnPg* prob	*FnSg* n	*FnSg* coverage	*FnSg* prob	*FnPgSg* n	*FnPgSg* coverage	*FnPgSg* prob	Description
FN1034	2	13.7%	0.473	2	6.3%	1.000	3	15.1%	0.991	3	17.6%	0.975	AAL95230.1| Transcriptional regulator, TetR family
	1	8.3%	0.982	2	14.6%	0.999	6	34.6%	1.000	2	6.3%	0.997	
FN1035	0	0.0%		0	0.0%	0.000	0	0.0%	0.000	0	0.0%		AAL95231.1| Iron-sulfur flavoprotein
	0	0.0%		2	15.7%	0.945	3	22.0%	0.999	0	0.0%		
FN1036	0	0.0%	0.000	1	10.4%	0.520	3	10.8%	0.871	0	0.0%	0.000	AAL95232.1| Hypothetical protein
	2	21.2%	0.642	2	10.4%	0.993	3	15.1%	1.000	1	10.4%	0.341	
FN1037	1	9.5%	0.971	0	0.0%	0.000	5	41.2%	1.000	3	22.3%	0.837	AAL95233.1| Hypothetical cytosolic protein
	0	0.0%	0.000	6	50.0%	1.000	6	51.4%	1.000	0	0.0%	0.000	
FN1041	1	3.3%	0.962	3	9.2%	0.999	4	13.0%	1.000	7	17.9%	1.000	AAL95237.1| Acetyltransferase
	2	7.4%	0.938	7	20.7%	1.000	10	24.6%	1.000	3	9.7%	0.999	
FN1042	13	40.9%	1.000	5	26.3%	1.000	5	20.6%	1.000	9	34.2%	1.000	AAL95238.1| S1 RNA binding domain
	12	33.1%	1.000	8	32.7%	1.000	8	27.0%	1.000	10	34.2%	1.000	
FN1045	5	20.1%	1.000	0	0.0%	0.000	1	5.9%	0.316	2	9.3%	0.804	AAL95241.1| Hypothetical protein
	3	16.0%	0.990	0	0.0%	0.000	2	11.5%	0.987	0	0.0%	0.000	
FN1048	1	4.4%	0.628	0	0.0%	0.000	3	8.7%	0.860	3	9.8%	0.841	AAL95244.1| Hypothetical membrane-spanning protein
	1	1.9%	0.907	0	0.0%	0.000	0	0.0%	0.000	0	0.0%	0.000	
FN1050	3	18.9%	0.998	4	37.0%	1.000	1	11.0%	0.336	0	0.0%	0.000	AAL95246.1| Lactoylglutathione lyase
	1	8.7%	0.610	3	28.3%	0.995	1	11.8%	0.733	1	10.2%	0.922	
FN1053	1	7.1%	0.859	3	17.0%	1.000	0	0.0%		0	0.0%		AAL95249.1| Hypothetical protein
	1	10.4%	0.972	0	0.0%	0.000	0	0.0%		0	0.0%		
FN1055	11	34.2%	1.000	7	17.3%	1.000	14	41.1%	1.000	13	38.7%	1.000	AAL95251.1| Cysteine synthase
	15	38.1%	1.000	15	44.9%	1.000	18	52.1%	1.000	12	31.5%	1.000	
FN1057	5	27.4%	1.000	2	8.9%	0.960	5	21.2%	1.000	1	7.5%	0.933	AAL95253.1| Diamine acetyltransferase
	3	19.9%	1.000	1	7.5%	0.306	3	8.9%	1.000	1	7.5%	0.451	
FN1060	3	16.1%	1.000	6	20.1%	1.000	12	56.7%	1.000	7	26.0%	1.000	AAL95256.1| hypothetical cytosolic protein
	3	15.7%	1.000	11	52.0%	1.000	13	52.0%	1.000	7	23.2%	1.000	
FN1062	15	38.5%	1.000	14	40.0%	1.000	9	34.2%	1.000	18	44.6%	1.000	AAL95258.1| Hydrolase
	14	35.8%	1.000	14	54.2%	1.000	13	43.1%	1.000	18	43.8%	1.000	
FN1063	6	16.5%	1.000	4	15.2%	1.000	9	32.2%	1.000	3	12.2%	1.000	AAL95259.1| N-acyl-L-amino acid amidohydrolase
	4	14.7%	1.000	17	55.3%	1.000	15	54.6%	1.000	2	5.6%	1.000	
FN1066	6	16.3%	1.000	3	11.1%	0.993	6	21.5%	1.000	5	13.9%	1.000	AAL95262.1| Exodeoxyribonuclease VII large subunit
	4	12.4%	1.000	6	19.6%	1.000	9	27.2%	1.000	5	12.1%	1.000	

Columns: *Fn* Rep I, Rep II; *FnPg* Rep I, Rep II; *FnSg* Rep I, Rep II; *FnPgSg* Rep I, Rep II

Show detected proteins only
Show all proteins
Filter by category:
GO: amino acid transport

Proteins found: 1424

Enter (or paste) list of ORFs

Find ORFs

Dot Plots Dot Plots

ORF	*Fn*			*FnPg*			*FnSg*			*FnPgSg*			Description
	n	coverage	prob	n	coverage	prob	n	coverage	prob	n	coverage	prob	
**FN1067**	10	52.3%	1.000	6	43.9%	1.000	8	42.2%	1.000	7	34.2%	1.000	AAL95263.1| Tetratricopeptide repeat family protein
	11	55.7%	1.000	8	46.4%	1.000	10	46.4%	1.000	9	49.4%	1.000	
**FN1068**	1	5.2%	0.447	3	8.7%	1.000	6	24.7%	1.000	1	7.6%	0.798	AAL95264.1| Smf protein
	2	9.7%	0.978	6	21.9%	1.000	6	23.6%	1.000	0	0.0%	0.000	
**FN1069**	30	38.1%	1.000	15	25.0%	1.000	12	20.6%	1.000	20	30.7%	1.000	AAL95265.1| DNA topoisomerase I
	34	36.0%	1.000	20	28.8%	1.000	16	24.9%	1.000	17	25.9%	1.000	
**FN1070**	6	20.0%	1.000	2	8.5%	0.999	3	12.7%	1.000	7	20.7%	1.000	AAL95266.1| Glucose inhibited division protein A
	7	18.9%	1.000	5	18.0%	1.000	8	27.0%	1.000	9	20.3%	1.000	
**FN1071**	1	3.1%	0.741	0	0.0%	0.000	1	4.8%	0.350	4	14.1%	0.998	AAL95267.1| Integrase/recombinase
	3	11.7%	0.997	2	5.9%	0.929	3	12.4%	0.998	0	0.0%	0.000	
**FN1072**	14	42.3%	1.000	12	38.0%	1.000	7	32.0%	1.000	12	41.8%	1.000	AAL95268.1| GTP-binding protein
	13	37.7%	1.000	11	36.9%	1.000	10	42.6%	1.000	14	43.7%	1.000	
**FN1073**	5	36.3%	1.000	2	7.7%	1.000	4	23.2%	1.000	2	14.3%	1.000	AAL95269.1| Hypothetical protein
	5	25.6%	1.000	3	25.0%	1.000	5	36.3%	1.000	3	12.5%	1.000	
**FN1074**	11	41.1%	1.000	8	23.1%	1.000	11	41.7%	1.000	8	27.6%	1.000	AAL95270.1| Signal recognition particle receptor FtsY
	10	37.8%	1.000	11	38.4%	1.000	11	36.0%	1.000	7	24.9%	1.000	
**FN1075**	1	4.2%	0.824	1	4.2%	0.814	2	12.1%	0.972	1	7.9%	0.936	AAL95271.1| Hypothetical protein
	2	9.8%	0.977	1	4.2%	0.883	3	17.7%	0.999	0	0.0%	0.000	
**FN1077**	3	43.4%	1.000	1	15.7%	0.961	5	51.8%	1.000	4	60.2%	1.000	AAL95273.1| Hypothetical protein
	2	26.5%	0.951	7	73.5%	1.000	3	41.0%	1.000	6	68.7%	1.000	
**FN1078**	17	55.8%	1.000	16	52.5%	1.000	19	63.0%	1.000	20	53.0%	1.000	AAL95274.1| Hypothetical exported 24-amino acid repeat protein
	15	53.0%	1.000	20	63.0%	1.000	18	55.2%	1.000	24	63.0%	1.000	
**FN1079**	26	83.3%	1.000	15	79.2%	1.000	22	93.1%	1.000	20	79.2%	1.000	AAL95275.1| Neutrophil-activating protein A
	25	84.7%	1.000	18	88.2%	1.000	19	88.2%	1.000	21	86.8%	1.000	
**FN1080**	5	11.2%	1.000	3	8.9%	0.942	5	12.1%	1.000	5	12.2%	1.000	AAL95276.1| Export ABC transporter
	6	11.5%	1.000	3	8.0%	0.993	7	16.1%	1.000	4	10.1%	0.999	
**FN1081**	5	43.5%	1.000	3	26.1%	1.000	1	17.4%	0.984	3	34.8%	1.000	AAL95277.1| unknown
	5	43.5%	1.000	2	26.1%	0.996	1	17.4%	0.932	4	43.5%	1.000	
**FN1082**	1	12.1%	0.876	0	0.0%	0.000	0	0.0%		3	51.6%	0.997	AAL95278.1| unknown
	0	0.0%	0.000	0	0.0%	0.000	0	0.0%		1	12.1%	0.766	
**FN1084**	4	49.4%	1.000	3	49.4%	1.000	3	49.4%	1.000	4	49.4%	1.000	AAL95280.1| unknown
	4	49.4%	1.000	3	49.4%	1.000	3	49.4%	1.000	4	61.2%	1.000	

Fn Rep I Rep II | *FnPg* Rep I Rep II | *FnSg* Rep I Rep II | *FnPgSg* Rep I Rep II

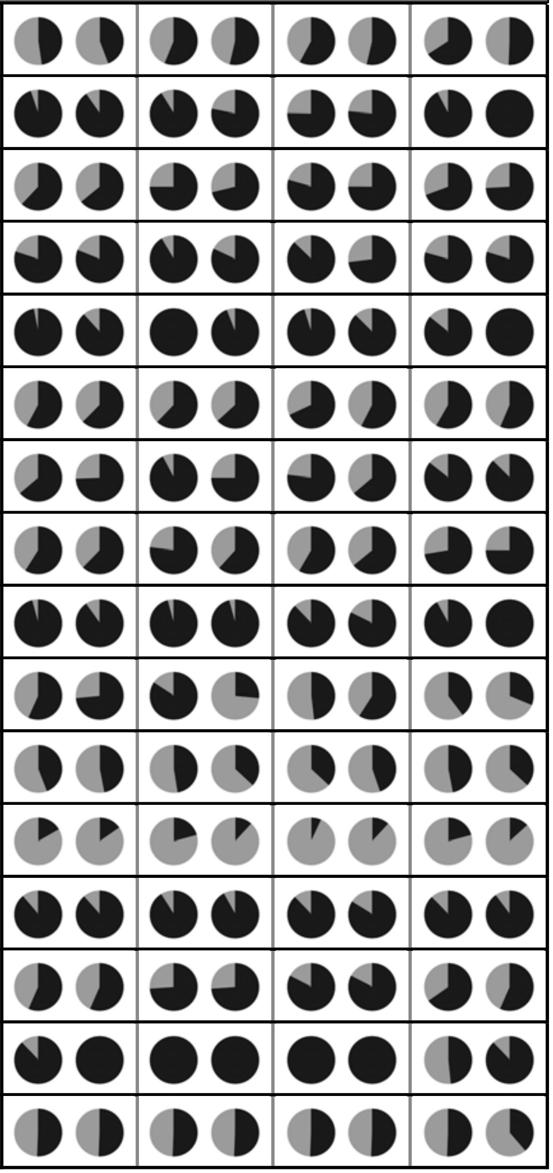

Show detected proteins only
Show all proteins
Filter by category:
GO: amino acid transport

Proteins found: 1424

Enter (or paste) list of ORFs

Find ORFs

Dot Plots Dot Plots

ORF	*Fn* n	*Fn* coverage	*Fn* prob	*FnPg* n	*FnPg* coverage	*FnPg* prob	*FnSg* n	*FnSg* coverage	*FnSg* prob	*FnPgSg* n	*FnPgSg* coverage	*FnPgSg* prob	Description
**FN1085**	8	46.2%	1.000	12	53.3%	1.000	16	65.9%	1.000	8	53.3%	1.000	AAL95281.1| 4-methyl-5(B-hydroxyethyl)-thiazole monophosphate biosynthesis
	7	39.0%	1.000	17	71.4%	1.000	13	67.0%	1.000	8	52.7%	1.000	
**FN1086**	4	7.7%	1.000	1	2.9%	0.902	10	19.5%	1.000	8	16.6%	1.000	AAL95282.1| Transporter
	6	11.6%	1.000	6	15.4%	1.000	10	19.5%	1.000	5	9.8%	1.000	
**FN1088**	10	21.6%	1.000	5	17.2%	1.000	11	27.6%	1.000	11	28.7%	1.000	AAL95284.1| NADH oxidase
	10	27.1%	1.000	9	27.6%	1.000	7	15.2%	1.000	10	25.3%	1.000	
**FN1089**	8	31.4%	1.000	14	38.6%	1.000	18	54.8%	1.000	8	31.0%	1.000	AAL95285.1| ATP-binding protein (contains P-loop)
	9	35.5%	1.000	22	71.7%	1.000	21	58.6%	1.000	4	21.0%	1.000	
**FN1091**	6	17.7%	1.000	4	8.9%	1.000	4	8.9%	1.000	3	12.1%	1.000	AAL95287.1| Sigma factor sigB regulation protein rsbU
	8	22.8%	1.000	4	9.6%	1.000	7	17.9%	1.000	4	10.7%	1.000	
**FN1092**	6	18.4%	1.000	4	17.4%	1.000	10	19.1%	1.000	8	19.7%	1.000	AAL95288.1| Hypothetical protein
	6	23.4%	1.000	13	26.3%	1.000	10	27.3%	1.000	8	24.7%	1.000	
**FN1093**	12	72.5%	1.000	7	43.0%	1.000	5	43.7%	1.000	12	72.5%	1.000	AAL95289.1| Hypothetical protein
	11	59.9%	1.000	9	53.5%	1.000	9	52.1%	1.000	10	59.9%	1.000	
**FN1094**	1	7.8%	0.979	1	7.8%	0.983	5	20.3%	1.000	2	13.9%	0.995	AAL95290.1| Dolichol-phosphate mannosyltransferase
	1	7.8%	0.982	7	23.9%	1.000	11	26.7%	1.000	2	10.3%	0.994	
**FN1095**	0	0.0%	0.000	3	18.3%	1.000	1	10.8%	0.662	2	20.8%	0.992	AAL95291.1| unknown
	1	8.3%	0.859	0	0.0%	0.000	1	10.8%	0.908	2	20.8%	0.801	
**FN1096**	13	30.2%	1.000	12	25.8%	1.000	6	15.9%	1.000	10	25.6%	1.000	AAL95292.1| Hypothetical protein
	11	28.3%	1.000	8	22.2%	1.000	13	29.2%	1.000	8	22.2%	1.000	
**FN1097**	12	17.1%	1.000	10	17.0%	1.000	0	0.0%	0.000	8	15.3%	1.000	AAL95293.1| Hypothetical protein
	10	16.1%	1.000	2	5.2%	0.999	5	7.4%	1.000	7	12.6%	1.000	
**FN1101**	3	7.7%	0.998	2	6.0%	0.952	1	1.7%	0.544	3	10.2%	1.000	AAL95297.1| ATPase
	2	6.0%	0.997	2	6.0%	0.992	5	17.0%	1.000	2	8.7%	0.808	
**FN1102**	4	17.9%	1.000	0	0.0%	0.000	2	10.1%	0.982	0	0.0%		AAL95298.1| tRNA 2'phosphotransferase
	3	17.9%	1.000	1	5.0%	0.873	3	18.4%	1.000	0	0.0%		
**FN1103**	27	39.4%	1.000	7	12.6%	1.000	18	27.0%	1.000	8	12.4%	1.000	AAL95299.1| Excinuclease ABC subunit A
	22	34.7%	1.000	17	25.7%	1.000	22	32.3%	1.000	11	17.2%	1.000	
**FN1104**	2	5.2%	0.999	0	0.0%	0.000	2	5.2%	0.997	4	24.7%	1.000	AAL95300.1| Holliday junction DNA helicase ruvA
	1	5.2%	0.975	1	5.2%	0.983	2	5.2%	0.988	1	3.6%	0.547	
**FN1105**	14	70.9%	1.000	10	55.2%	1.000	11	53.3%	1.000	17	75.8%	1.000	AAL95301.1| Hypothetical protein
	14	64.8%	1.000	14	70.9%	1.000	13	59.4%	1.000	13	75.2%	1.000	

Coverage pie charts: *Fn*, *FnPg*, *FnSg*, *FnPgSg* — Rep I, Rep II

Show detected proteins only
Show all proteins
Filter by category:
GO: amino acid transport

Proteins found: 1424

Enter (or paste) list of ORFs

Find ORFs

Dot Plots Dot Plots

ORF	Fn n	Fn coverage	Fn prob	FnPg n	FnPg coverage	FnPg prob	FnSg n	FnSg coverage	FnSg prob	FnPgSg n	FnPgSg coverage	FnPgSg prob	Description
FN1106	12	28.4%	1.000	9	23.0%	1.000	15	37.7%	1.000	10	26.7%	1.000	AAL95302.1| L-serine dehydratase
	10	23.0%	1.000	13	37.0%	1.000	12	32.6%	1.000	9	25.2%	1.000	
FN1111	2	4.8%	0.997	2	7.6%	0.609	6	17.3%	1.000	6	17.7%	1.000	AAL95307.1| Dipeptide-binding protein
	4	7.8%	1.000	2	4.8%	1.000	10	27.9%	1.000	7	19.9%	1.000	
FN1117	7	44.2%	1.000	4	44.2%	1.000	4	44.2%	1.000	6	40.4%	1.000	AAL95313.1| LSU ribosomal protein L21P
	5	51.0%	1.000	5	32.7%	1.000	4	26.9%	0.996	6	40.4%	1.000	
FN1119	6	35.1%	1.000	3	25.5%	1.000	4	25.5%	1.000	8	43.6%	1.000	AAL95315.1| LSU ribosomal protein L27P
	4	22.3%	1.000	5	35.1%	1.000	4	35.1%	1.000	8	43.6%	1.000	
FN1120	20	38.9%	1.000	28	55.6%	1.000	33	59.0%	1.000	25	52.2%	1.000	AAL95316.1| Phosphoenolpyruvate carboxykinase (ATP)
	22	40.8%	1.000	40	63.0%	1.000	33	61.5%	1.000	22	51.8%	1.000	
FN1121	26	41.9%	1.000	13	23.9%	1.000	17	31.6%	1.000	21	37.2%	1.000	AAL95317.1| hypothetical cytosolic protein
	21	38.7%	1.000	27	50.0%	1.000	27	43.7%	1.000	24	43.5%	1.000	
FN1122	29	31.3%	1.000	9	7.1%	1.000	18	20.8%	1.000	27	28.6%	1.000	AAL95318.1| Long-chain-fatty-acid--CoA ligase
	26	28.3%	1.000	16	18.2%	1.000	20	21.6%	1.000	24	29.1%	1.000	
FN1123	10	48.4%	1.000	3	22.9%	1.000	5	32.5%	1.000	7	47.8%	1.000	AAL95319.1| Thioredoxin-like protein
	7	48.4%	1.000	3	25.5%	1.000	3	24.2%	1.000	6	45.9%	1.000	
FN1124	24	67.0%	1.000	15	49.2%	1.000	24	67.3%	1.000	29	72.4%	1.000	AAL95320.1| Outer membrane porin F
	23	67.0%	1.000	23	75.2%	1.000	22	78.1%	1.000	26	72.4%	1.000	
FN1125	6	44.3%	1.000	7	37.2%	1.000	10	51.9%	1.000	9	44.3%	1.000	AAL95321.1| LemA protein
	7	44.3%	1.000	11	50.8%	1.000	9	50.8%	1.000	10	50.8%	1.000	
FN1127	11	23.9%	1.000	11	27.2%	1.000	16	36.1%	1.000	13	27.2%	1.000	AAL95323.1| Hypothetical membrane-spanning protein
	14	26.7%	1.000	14	29.0%	1.000	17	31.4%	1.000	10	20.6%	1.000	
FN1128	29	51.8%	1.000	34	55.3%	1.000	24	45.5%	1.000	26	44.5%	1.000	AAL95324.1| Acylamino-acid-releasing enzyme
	32	53.8%	1.000	37	58.3%	1.000	29	50.6%	1.000	24	42.0%	1.000	
FN1129	10	9.6%	1.000	2	2.4%	0.998	4	5.1%	1.000	9	10.8%	1.000	AAL95325.1| Chromosome partition protein smc
	13	14.2%	1.000	5	5.3%	1.000	8	9.4%	1.000	4	3.8%	0.969	
FN1130	7	24.9%	1.000	2	11.4%	0.997	2	9.2%	0.999	5	20.0%	1.000	AAL95326.1| Tetraacyldisaccharide 4'-kinase
	7	24.9%	1.000	3	11.1%	0.956	5	22.2%	1.000	2	7.7%	0.974	
FN1131	1	4.3%	0.977	1	6.2%	0.943	2	10.5%	1.000	3	10.5%	0.996	AAL95327.1| Hypothetical protein
	5	20.6%	1.000	5	20.6%	1.000	4	17.9%	1.000	2	7.0%	0.910	
FN1133	11	34.5%	1.000	8	32.6%	1.000	9	28.2%	1.000	14	38.9%	1.000	AAL95329.1| N-acetylglucosamine-6-phosphate deacetylase
	8	22.0%	1.000	19	53.4%	1.000	8	26.9%	1.000	13	37.6%	1.000	

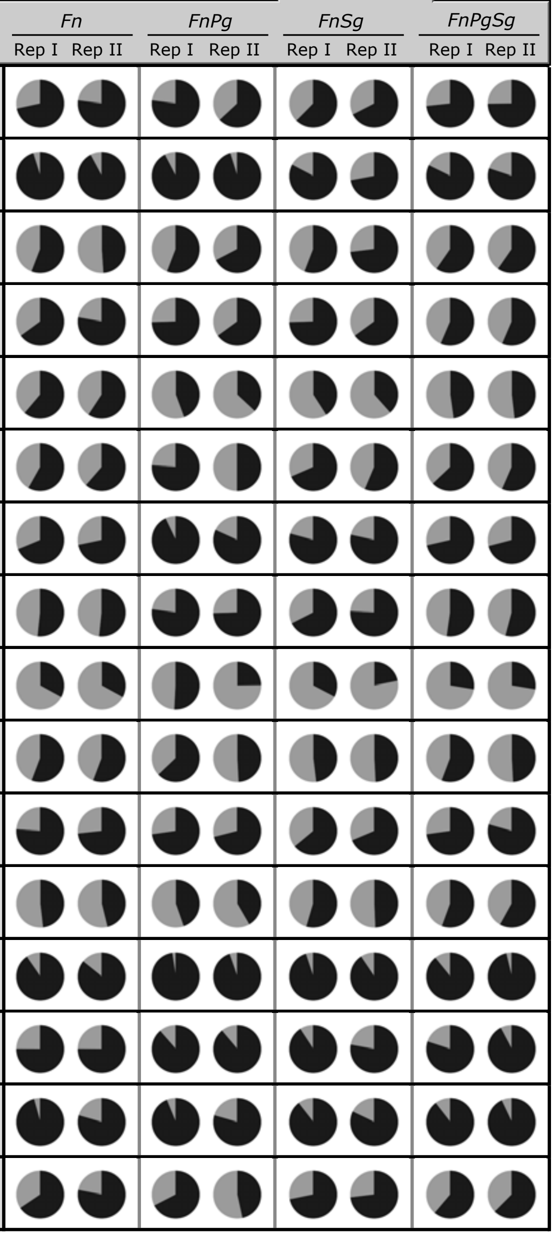

ORF	*Fn* n	*Fn* coverage	*Fn* prob	*FnPg* n	*FnPg* coverage	*FnPg* prob	*FnSg* n	*FnSg* coverage	*FnSg* prob	*FnPgSg* n	*FnPgSg* coverage	*FnPgSg* prob	Description
**FN1134**	10	47.7%	1.000	6	30.3%	1.000	2	10.3%	1.000	9	47.7%	1.000	AAL95330.1| Hypothetical cytosolic protein
	9	41.3%	1.000	6	34.8%	1.000	3	16.8%	1.000	6	41.3%	1.000	
**FN1135**	14	41.5%	1.000	8	25.9%	1.000	11	48.9%	1.000	14	41.1%	1.000	AAL95331.1| Phosphonates-binding protein
	14	38.3%	1.000	13	45.7%	1.000	13	36.2%	1.000	15	41.5%	1.000	
**FN1136**	3	17.8%	1.000	0	0.0%	0.000	6	27.1%	1.000	4	24.3%	1.000	AAL95332.1| Phosphonates transport ATP-binding protein phnC
	4	23.5%	1.000	8	31.6%	1.000	6	22.7%	1.000	5	29.1%	1.000	
**FN1137**	0	0.0%		0	0.0%	0.000	1	4.6%	0.400	1	3.6%	0.462	AAL95333.1| Phosphonates transport system permease protein phnE
	0	0.0%		1	4.6%	0.730	2	8.2%	0.876	3	4.4%	0.996	
**FN1138**	15	67.1%	1.000	10	63.6%	1.000	16	79.7%	1.000	16	67.1%	1.000	AAL95334.1| Hypothetical cytosolic protein
	14	67.1%	1.000	18	79.7%	1.000	14	72.0%	1.000	16	74.1%	1.000	
**FN1139**	18	22.5%	1.000	7	10.6%	1.000	30	38.9%	1.000	9	12.8%	1.000	AAL95335.1| Activator of (R)-2-hydroxyglutaryl-CoA dehydratase
	13	18.8%	1.000	32	39.5%	1.000	41	47.5%	1.000	5	6.9%	1.000	
**FN1140**	4	13.0%	1.000	2	5.2%	0.719	5	17.4%	1.000	1	2.7%	0.415	AAL95336.1| hypothetical protein
	5	17.0%	1.000	9	34.2%	1.000	12	38.8%	1.000	2	6.4%	0.966	
**FN1142**	6	18.1%	1.000	1	3.3%	0.979	4	17.1%	1.000	3	11.5%	0.988	AAL95338.1| Oxygen-independent coproporphyrinogen III oxidase
	6	15.5%	1.000	4	17.8%	0.930	5	14.8%	1.000	0	0.0%	0.000	
**FN1143**	16	50.7%	1.000	9	46.4%	1.000	19	71.9%	1.000	17	51.5%	1.000	AAL95339.1| Glucosamine-6-phosphate isomerase
	15	50.7%	1.000	24	83.2%	1.000	18	77.7%	1.000	13	49.6%	1.000	
**FN1144**	19	52.2%	1.000	6	26.5%	1.000	16	65.9%	1.000	22	55.4%	1.000	AAL95340.1| Hypothetical Exported Protein
	21	58.2%	1.000	17	65.9%	1.000	16	58.6%	1.000	23	58.2%	1.000	
**FN1145**	6	20.6%	1.000	5	14.3%	1.000	6	18.6%	1.000	9	14.0%	1.000	AAL95341.1| Oligoendopeptidase F
	7	20.2%	1.000	13	32.4%	1.000	19	32.7%	1.000	12	22.7%	1.000	
**FN1146**	5	39.8%	1.000	3	32.0%	1.000	0	0.0%	0.000	5	46.1%	1.000	AAL95342.1| Hypothetical exported 24-amino acid repeat protein
	5	39.8%	1.000	2	18.0%	0.994	1	7.8%	0.912	4	38.3%	1.000	
**FN1147**	9	34.1%	1.000	1	2.9%	0.942	0	0.0%		12	36.5%	1.000	AAL95343.1| Hypothetical protein
	11	37.7%	1.000	1	3.2%	0.969	0	0.0%		11	36.0%	1.000	
**FN1148**	6	12.8%	1.000	3	9.5%	1.000	5	11.0%	1.000	10	21.3%	1.000	AAL95344.1| Serine/threonine sodium symporter
	6	12.8%	1.000	8	17.4%	1.000	5	11.0%	1.000	4	11.0%	1.000	
**FN1149**	9	12.1%	1.000	5	8.0%	1.000	7	10.0%	1.000	4	6.2%	1.000	AAL95345.1| ATP-dependent nuclease subunit A
	7	8.2%	1.000	5	5.4%	1.000	11	14.1%	1.000	4	6.4%	1.000	
**FN1150**	0	0.0%	0.000	2	2.0%	0.980	1	1.3%	0.957	1	1.3%	0.979	AAL95346.1| unknown
	6	7.9%	1.000	4	5.3%	1.000	5	5.1%	1.000	4	5.9%	1.000	

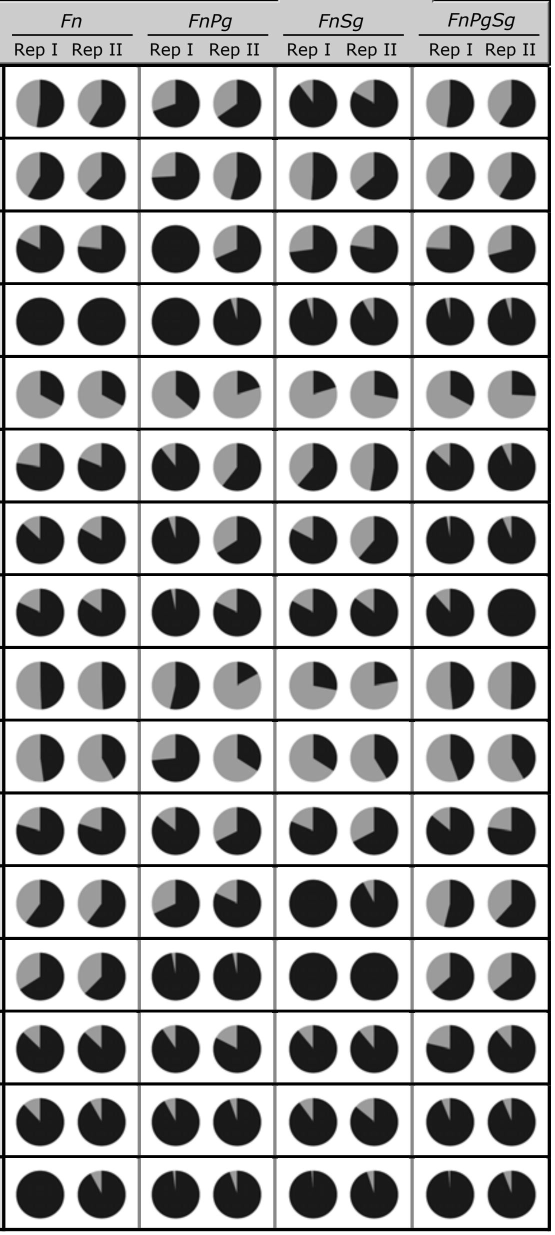

Show detected proteins only
Show all proteins
Filter by category:
GO: amino acid transport

Proteins found: 1424

Enter (or paste) list of ORFs

Find ORFs

Dot Plots Dot Plots

ORF	*Fn* n	*Fn* coverage	*Fn* prob	*FnPg* n	*FnPg* coverage	*FnPg* prob	*FnSg* n	*FnSg* coverage	*FnSg* prob	*FnPgSg* n	*FnPgSg* coverage	*FnPgSg* prob	Description
**FN1152**	24	50.8%	1.000	14	35.6%	1.000	22	48.2%	1.000	25	49.2%	1.000	AAL95348.1| Aspartate aminotransferase
	23	49.0%	1.000	25	49.5%	1.000	22	46.0%	1.000	26	51.5%	1.000	
**FN1153**	5	58.7%	1.000	0	0.0%	0.000	1	17.4%	0.985	4	57.8%	1.000	AAL95349.1| Hypothetical protein
	4	57.8%	1.000	1	17.4%	0.983	2	32.1%	1.000	4	57.8%	1.000	
**FN1154**	2	6.8%	0.997	1	2.3%	0.958	3	4.8%	0.999	1	4.5%	0.988	AAL95350.1| Ribonuclease BN
	3	11.9%	1.000	4	10.4%	1.000	3	12.9%	1.000	1	4.5%	0.901	
**FN1155**	3	8.7%	1.000	1	3.7%	0.274	3	5.2%	0.771	0	0.0%		AAL95351.1| Cell division protein ftsI
	3	9.0%	1.000	1	2.0%	0.598	3	7.2%	0.991	0	0.0%		
**FN1156**	2	2.2%	0.554	0	0.0%	0.000	3	4.3%	0.948	0	0.0%	0.000	AAL95352.1| Primosomal protein N'
	2	4.3%	0.497	2	4.4%	0.898	4	5.0%	0.991	1	1.4%	0.437	
**FN1157**	3	29.9%	1.000	1	16.7%	0.275	1	6.9%	0.444	7	31.0%	1.000	AAL95353.1| Polypeptide deformylase
	4	39.1%	1.000	2	11.5%	0.990	1	4.6%	0.283	5	21.8%	1.000	
**FN1159**	16	41.6%	1.000	13	44.2%	1.000	18	51.2%	1.000	17	39.9%	1.000	AAL95355.1| Fructose-1,6-bisphosphatase
	18	48.8%	1.000	20	53.8%	1.000	23	53.8%	1.000	16	37.6%	1.000	
**FN1160**	3	3.0%	0.993	1	0.8%	0.352	5	6.2%	1.000	4	4.9%	1.000	AAL95356.1| SWF/SNF family helicase
	3	3.4%	1.000	3	3.4%	0.914	7	10.2%	1.000	7	6.1%	1.000	
**FN1161**	5	24.2%	1.000	1	6.1%	0.978	4	21.6%	1.000	1	5.7%	0.895	AAL95357.1| Glutamate racemase
	3	17.4%	1.000	3	17.4%	0.993	6	30.7%	1.000	4	21.2%	1.000	
**FN1162**	1	6.3%	0.564	1	12.6%	0.984	3	30.4%	0.999	0	0.0%		AAL95358.1| Hydroxyacylglutathione hydrolase
	4	29.5%	1.000	4	22.2%	1.000	5	37.7%	1.000	0	0.0%		
**FN1163**	11	49.2%	1.000	8	29.5%	1.000	9	40.4%	1.000	9	42.6%	1.000	AAL95359.1| Thioredoxin reductase
	10	39.8%	1.000	13	52.4%	1.000	8	36.4%	1.000	9	39.5%	1.000	
**FN1164**	7	26.7%	1.000	4	7.6%	1.000	6	22.9%	1.000	2	10.2%	0.985	AAL95360.1| Glucokinase
	6	22.2%	1.000	7	22.5%	1.000	7	22.9%	1.000	3	12.1%	1.000	
**FN1165**	52	82.7%	1.000	47	76.2%	1.000	48	85.3%	1.000	52	86.8%	1.000	AAL95361.1| D-galactose-binding protein
	54	82.7%	1.000	45	91.5%	1.000	42	84.8%	1.000	52	84.2%	1.000	
**FN1166**	28	47.2%	1.000	26	44.8%	1.000	33	62.2%	1.000	26	52.6%	1.000	AAL95362.1| Galactoside transport ATP-binding protein mglA
	32	55.6%	1.000	35	54.8%	1.000	33	54.6%	1.000	24	48.8%	1.000	
**FN1167**	7	22.4%	1.000	1	4.1%	0.941	3	10.3%	0.954	6	25.4%	1.000	AAL95363.1| Galactoside transport system permease protein mglC
	5	15.9%	1.000	2	6.2%	0.942	3	15.3%	0.996	4	9.4%	1.000	
**FN1169**	20	50.6%	1.000	14	49.1%	1.000	19	73.6%	1.000	22	50.6%	1.000	AAL95365.1| L-lactate dehydrogenase
	20	48.4%	1.000	20	63.8%	1.000	18	61.6%	1.000	21	50.6%	1.000	

Fn Rep I Rep II | *FnPg* Rep I Rep II | *FnSg* Rep I Rep II | *FnPgSg* Rep I Rep II

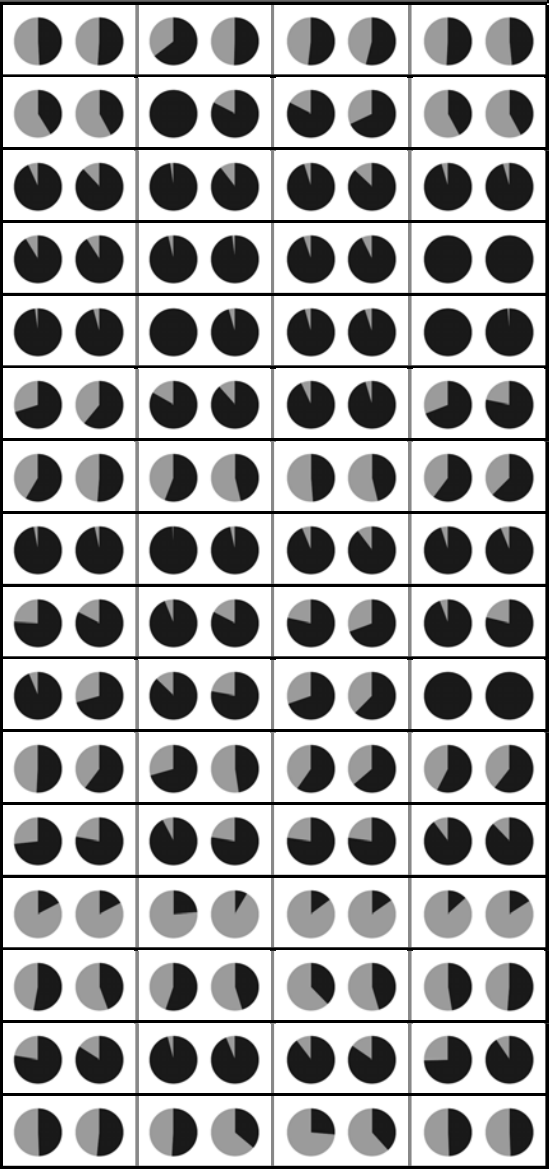

Show detected proteins only
Show all proteins
Filter by category:
GO: amino acid transport

Proteins found: 1424

Enter (or paste) list of ORFs

Find ORFs

Dot Plots Dot Plots

ORF	Fn n	Fn coverage	Fn prob	FnPg n	FnPg coverage	FnPg prob	FnSg n	FnSg coverage	FnSg prob	FnPgSg n	FnPgSg coverage	FnPgSg prob	Description
FN1170	125	68.4%	1.000	107	65.1%	1.000	113	73.7%	1.000	122	70.3%	1.000	AAL95366.1| Pyruvate-flavodoxin oxidoreductase
	119	67.5%	1.000	117	71.4%	1.000	118	78.0%	1.000	123	67.6%	1.000	
FN1171	37	76.1%	1.000	40	80.9%	1.000	37	86.7%	1.000	39	72.6%	1.000	AAL95367.1| Acetate kinase
	41	76.1%	1.000	43	80.4%	1.000	42	89.2%	1.000	37	73.4%	1.000	
FN1172	29	67.7%	1.000	25	58.8%	1.000	27	71.2%	1.000	30	76.3%	1.000	AAL95368.1| Phosphate acetyltransferase
	29	67.7%	1.000	24	62.9%	1.000	23	57.3%	1.000	27	76.3%	1.000	
FN1176	0	0.0%		4	39.6%	1.000	0	0.0%		0	0.0%		AAL95372.1| Hypothetical cytosolic protein
	0	0.0%		0	0.0%	0.000	0	0.0%		0	0.0%		
FN1179	1	2.1%	0.615	7	12.8%	1.000	1	2.2%	0.451	1	2.1%	0.588	AAL95375.1| ATP-dependent RNA helicase
	3	7.6%	1.000	0	0.0%	0.000	4	7.5%	0.927	0	0.0%	0.000	
FN1180	4	16.9%	1.000	3	18.1%	1.000	2	15.6%	0.986	2	9.9%	0.999	AAL95376.1| Hypothetical protein
	5	19.3%	1.000	0	0.0%	0.000	5	23.9%	1.000	1	6.6%	0.986	
FN1181	16	48.3%	1.000	18	50.3%	1.000	14	40.0%	1.000	18	66.0%	1.000	AAL95377.1| unknown
	12	39.0%	1.000	12	44.7%	1.000	15	48.0%	1.000	16	54.3%	1.000	
FN1182	4	7.2%	1.000	3	6.6%	1.000	1	1.7%	0.971	1	2.1%	0.887	AAL95378.1| Hypothetical protein
	5	7.4%	1.000	2	3.7%	0.940	5	8.3%	1.000	1	2.7%	0.327	
FN1183	3	12.4%	0.996	6	22.4%	1.000	6	28.0%	1.000	4	14.8%	1.000	AAL95379.1| Hypothetical cytosolic protein
	3	13.2%	0.990	4	13.6%	1.000	7	30.0%	1.000	3	10.8%	0.998	
FN1184	0	0.0%		3	18.1%	1.000	1	8.4%	0.985	1	5.4%	0.350	AAL95380.1| Hypothetical protein
	0	0.0%		0	0.0%	0.000	2	13.4%	0.999	0	0.0%	0.000	
FN1185	8	24.6%	1.000	4	11.5%	1.000	6	24.6%	1.000	9	28.2%	1.000	AAL95381.1| SIR2 family protein
	5	20.2%	1.000	11	33.3%	1.000	7	24.6%	1.000	8	31.0%	1.000	
FN1186	4	23.1%	1.000	7	33.4%	1.000	24	72.4%	1.000	6	33.4%	1.000	AAL95382.1| Amidohydrolase
	6	24.2%	1.000	26	80.5%	1.000	29	82.7%	1.000	3	13.4%	1.000	
FN1187	2	14.2%	0.999	3	14.2%	1.000	16	59.4%	1.000	10	44.8%	1.000	AAL95383.1| Amino acid-binding protein
	1	7.9%	0.982	18	69.5%	1.000	16	59.4%	1.000	10	51.5%	1.000	
FN1188	9	40.6%	1.000	4	19.4%	1.000	14	54.5%	1.000	8	58.8%	1.000	AAL95384.1| Hypothetical protein
	8	40.6%	1.000	14	54.5%	1.000	14	53.3%	1.000	8	32.7%	1.000	
FN1189	7	27.6%	1.000	7	25.9%	1.000	7	32.8%	1.000	9	56.9%	1.000	AAL95385.1| Hypothetical protein
	7	30.2%	1.000	13	68.1%	1.000	9	41.4%	1.000	9	46.6%	1.000	
FN1190	20	29.5%	1.000	18	28.2%	1.000	29	45.2%	1.000	22	31.6%	1.000	AAL95386.1| Probable cadmium-transporting ATPase
	25	35.8%	1.000	25	34.7%	1.000	31	45.0%	1.000	19	30.1%	1.000	

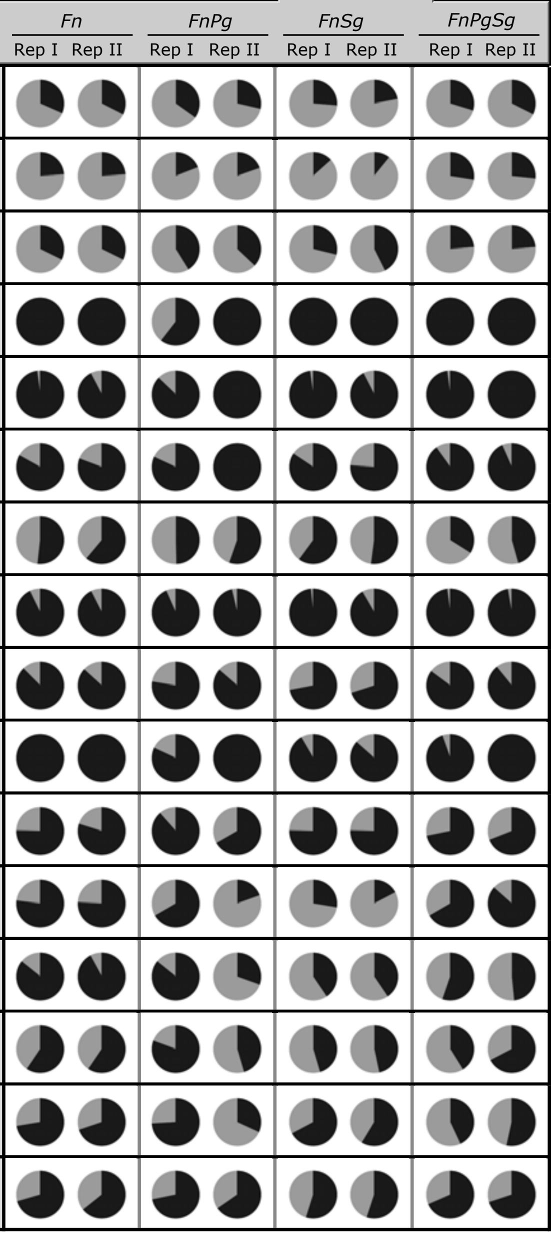

Show detected proteins only
Show all proteins
Filter by category:
GO: amino acid transport

Proteins found:
1424

Enter (or paste) list of ORFs

Find ORFs

Dot Plots Dot Plots

ORF	*Fn* n	*Fn* coverage	*Fn* prob	*FnPg* n	*FnPg* coverage	*FnPg* prob	*FnSg* n	*FnSg* coverage	*FnSg* prob	*FnPgSg* n	*FnPgSg* coverage	*FnPgSg* prob	Description
FN1191	7	28.8%	1.000	2	18.5%	0.960	13	56.4%	1.000	8	37.9%	1.000	AAL95387.1| unknown
	7	25.5%	1.000	8	35.0%	1.000	11	53.5%	1.000	6	21.8%	1.000	
FN1192	9	56.7%	1.000	7	47.8%	1.000	7	55.6%	1.000	11	80.0%	1.000	AAL95388.1| unknown
	9	55.6%	1.000	9	88.9%	1.000	7	55.6%	1.000	10	64.4%	1.000	
FN1198	10	24.8%	1.000	9	19.5%	1.000	16	35.7%	1.000	10	27.1%	1.000	AAL95394.1| Transporter
	7	16.7%	1.000	17	31.9%	1.000	21	42.1%	1.000	8	17.1%	1.000	
FN1200	2	9.7%	0.977	0	0.0%	0.000	25	72.6%	1.000	3	11.6%	1.000	AAL95396.1| Hypothetical protein
	2	9.7%	0.800	27	75.7%	1.000	25	71.8%	1.000	4	14.7%	1.000	
FN1201	2	9.7%	0.996	1	9.7%	0.915	1	9.7%	0.744	1	9.7%	0.429	AAL95397.1| unknown
	2	9.7%	0.994	2	9.7%	0.996	3	26.6%	0.996	0	0.0%	0.000	
FN1202	5	21.7%	1.000	7	24.4%	1.000	9	34.9%	1.000	10	35.7%	1.000	AAL95398.1| NH(3)-dependent NAD(+) synthetase
	2	5.8%	0.742	14	55.0%	1.000	6	24.4%	1.000	6	20.2%	1.000	
FN1203	2	11.1%	0.999	3	9.7%	1.000	5	19.4%	1.000	2	11.1%	0.993	AAL95399.1| GTP-binding protein
	4	19.7%	1.000	3	15.6%	1.000	5	22.8%	1.000	1	5.2%	0.967	
FN1204	3	20.4%	1.000	5	27.7%	1.000	6	37.0%	1.000	1	11.1%	0.989	AAL95400.1| Methyltransferase
	5	24.3%	1.000	5	30.6%	1.000	7	44.3%	1.000	1	3.0%	0.679	
FN1205	21	46.4%	1.000	8	30.0%	1.000	17	44.3%	1.000	19	49.4%	1.000	AAL95401.1| Protease
	19	47.5%	1.000	19	48.0%	1.000	14	43.3%	1.000	22	52.9%	1.000	
FN1206	2	10.5%	0.999	2	11.7%	0.890	1	4.9%	0.984	2	8.6%	0.655	AAL95402.1| Hemolysin
	4	10.5%	1.000	1	6.4%	0.307	2	10.5%	0.993	0	0.0%	0.000	
FN1208	6	12.3%	1.000	4	12.3%	0.999	2	6.3%	0.999	3	5.3%	0.999	AAL95404.1| 1-deoxyxylulose-5-phosphate synthase
	5	10.5%	1.000	6	14.7%	1.000	8	15.2%	1.000	1	1.5%	0.373	
FN1209	5	31.3%	1.000	2	21.2%	0.988	5	45.5%	1.000	5	38.4%	1.000	AAL95405.1| Hypothetical RNA binding protein
	6	41.4%	1.000	4	36.4%	1.000	5	31.3%	1.000	6	32.3%	1.000	
FN1210	16	26.6%	1.000	8	18.4%	1.000	12	20.2%	1.000	15	22.5%	1.000	AAL95406.1| Metal dependent hydrolase
	17	26.2%	1.000	18	29.6%	1.000	14	22.9%	1.000	19	30.8%	1.000	
FN1211	11	18.4%	1.000	3	7.8%	0.998	5	9.4%	1.000	7	14.3%	1.000	AAL95407.1| Cell division protein ftsI
	10	22.1%	1.000	6	11.0%	1.000	10	20.4%	1.000	3	5.3%	1.000	
FN1213	13	44.6%	1.000	5	26.3%	1.000	9	40.4%	1.000	11	33.3%	1.000	AAL95409.1| Hypothetical protein
	11	41.9%	1.000	13	38.5%	1.000	8	35.2%	1.000	8	25.1%	1.000	
FN1214	1	3.0%	0.981	0	0.0%	0.000	1	2.3%	0.801	6	18.6%	1.000	AAL95410.1| Fe-S oxidoreductase
	2	4.6%	0.939	1	3.9%	0.432	0	0.0%	0.000	4	9.0%	1.000	

Fn Rep I Rep II | *FnPg* Rep I Rep II | *FnSg* Rep I Rep II | *FnPgSg* Rep I Rep II

Show detected proteins only
Show all proteins
Filter by category:
GO: amino acid transport

Proteins found: 1424

Enter (or paste) list of ORFs

Find ORFs

Dot Plots Dot Plots

ORF	Fn n	Fn coverage	Fn prob	FnPg n	FnPg coverage	FnPg prob	FnSg n	FnSg coverage	FnSg prob	FnPgSg n	FnPgSg coverage	FnPgSg prob	Description
FN1216	5	44.8%	1.000	4	37.8%	1.000	5	50.3%	1.000	3	19.6%	0.999	AAL95412.1| RRF2 family protein
	5	31.5%	1.000	5	48.3%	1.000	6	44.1%	1.000	4	31.5%	1.000	
FN1217	7	30.1%	1.000	2	9.6%	0.988	8	44.3%	1.000	3	14.5%	0.999	AAL95413.1| Holliday junction DNA helicase ruvB
	8	31.9%	1.000	6	32.2%	1.000	10	37.3%	1.000	3	12.7%	0.961	
FN1218	1	4.0%	0.812	0	0.0%	0.000	4	22.0%	1.000	2	17.5%	0.992	AAL95414.1| unknown
	2	15.0%	0.783	3	17.0%	1.000	4	21.0%	1.000	0	0.0%	0.000	
FN1219	3	12.6%	1.000	4	25.2%	1.000	3	34.4%	1.000	5	30.5%	1.000	AAL95415.1| Hypothetical protein
	4	18.5%	1.000	5	24.5%	1.000	5	45.7%	1.000	5	30.5%	1.000	
FN1220	18	53.2%	1.000	17	53.8%	1.000	15	52.5%	1.000	20	51.3%	1.000	AAL95416.1| Cysteine synthase
	17	53.2%	1.000	18	63.9%	1.000	22	64.2%	1.000	19	55.4%	1.000	
FN1221	4	20.5%	1.000	4	14.9%	1.000	7	28.1%	1.000	7	26.4%	1.000	AAL95417.1| Hypothetical protein
	5	26.4%	1.000	11	41.0%	1.000	5	13.2%	1.000	7	20.8%	1.000	
FN1222	7	53.5%	1.000	4	31.8%	1.000	1	7.0%	0.501	4	30.2%	1.000	AAL95418.1| Hypothetical protein
	6	53.5%	1.000	6	53.5%	1.000	4	40.3%	1.000	6	36.4%	1.000	
FN1223	6	52.0%	1.000	3	37.7%	1.000	5	42.3%	1.000	7	52.0%	1.000	AAL95419.1| Oxygen-insensitive NAD(P)H nitroreductase
	6	52.0%	1.000	11	60.0%	1.000	6	42.9%	1.000	6	52.0%	1.000	
FN1224	23	71.7%	1.000	13	42.3%	1.000	18	57.0%	1.000	21	65.4%	1.000	AAL95420.1| 2-dehydro-3-deoxyphosphooctonate aldolase
	23	65.0%	1.000	23	68.5%	1.000	18	59.4%	1.000	19	65.0%	1.000	
FN1225	22	37.7%	1.000	13	24.3%	1.000	21	44.7%	1.000	28	51.3%	1.000	AAL95421.1| UDP-N-acetylmuramoyl-L-alanyl-D-glutamate--meso-lanthionine ..
	18	31.1%	1.000	28	43.5%	1.000	23	37.9%	1.000	26	47.4%	1.000	
FN1226	18	66.8%	1.000	10	37.6%	1.000	18	70.4%	1.000	15	56.2%	1.000	AAL95422.1| Uracil-DNA glycosylase
	17	66.8%	1.000	18	64.2%	1.000	21	64.2%	1.000	12	48.7%	1.000	
FN1229	3	17.8%	0.995	2	19.2%	0.483	0	0.0%		1	6.2%	0.967	AAL95425.1| Hypothetical protein
	3	12.3%	1.000	1	6.2%	0.964	0	0.0%		0	0.0%	0.000	
FN1230	14	58.6%	1.000	0	0.0%	0.000	0	0.0%		10	50.6%	1.000	AAL95426.1| Hypothetical cytosolic protein
	12	58.6%	1.000	1	6.8%	0.810	0	0.0%		8	45.7%	1.000	
FN1231	50	76.6%	1.000	27	56.1%	1.000	45	79.1%	1.000	61	81.7%	1.000	AAL95427.1| Inosine-5'-monophosphate dehydrogenase
	49	80.3%	1.000	56	85.0%	1.000	48	78.9%	1.000	57	80.9%	1.000	
FN1233	3	29.4%	0.999	0	0.0%	0.000	1	11.1%	0.983	2	14.4%	0.999	AAL95429.1| Putative NAD(P)H oxidoreductase
	3	22.2%	0.999	2	17.2%	0.993	3	25.0%	1.000	1	8.3%	0.690	
FN1234	4	15.1%	1.000	0	0.0%	0.000	2	11.9%	0.997	3	11.5%	0.979	AAL95430.1| Hypothetical protein
	3	7.3%	0.984	2	10.1%	0.780	5	20.6%	1.000	3	9.6%	0.987	

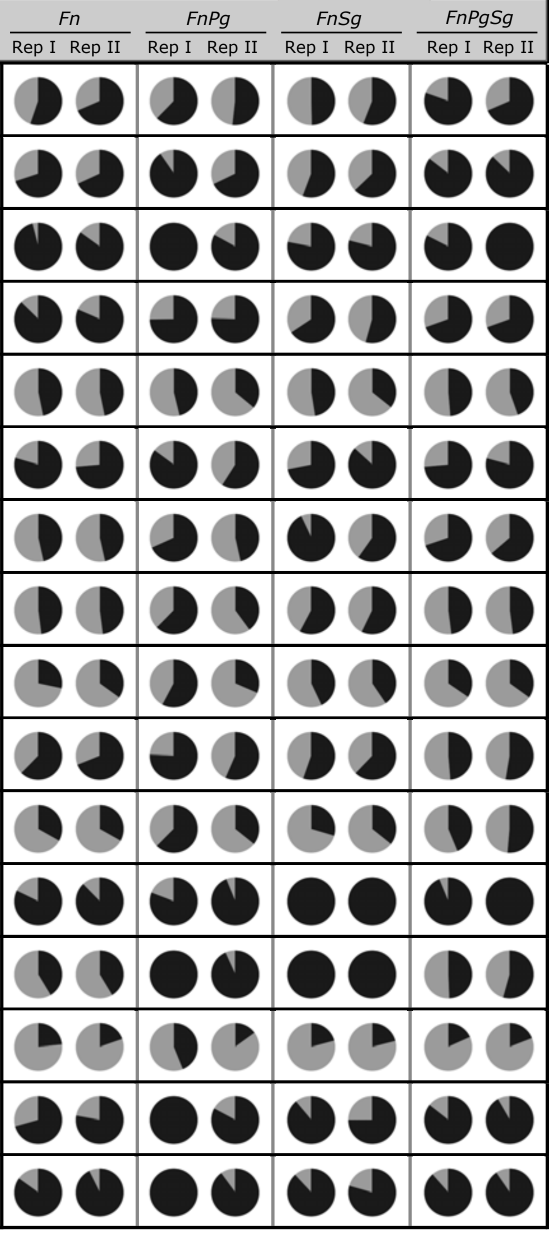

Show detected proteins only
Show all proteins
Filter by category:
GO: amino acid transport

Proteins found: 1424

Enter (or paste) list of ORFs

Find ORFs

Dot Plots Dot Plots

ORF	*Fn* n	*Fn* coverage	*Fn* prob	*FnPg* n	*FnPg* coverage	*FnPg* prob	*FnSg* n	*FnSg* coverage	*FnSg* prob	*FnPgSg* n	*FnPgSg* coverage	*FnPgSg* prob	Description
FN1235	4	22.4%	1.000	4	19.9%	1.000	13	50.9%	1.000	7	24.3%	1.000	AAL95431.1| Ankyrin repeat proteins
	2	11.7%	0.999	9	31.2%	1.000	10	35.4%	1.000	4	18.0%	1.000	
FN1237	10	20.0%	1.000	16	26.2%	1.000	26	40.3%	1.000	15	28.7%	1.000	AAL95433.1| Choline kinase
	12	23.4%	1.000	27	39.1%	1.000	32	42.3%	1.000	13	27.3%	1.000	
FN1238	2	5.8%	0.994	0	0.0%	0.000	1	2.5%	0.594	0	0.0%	0.000	AAL95434.1| Hypothetical protein
	2	5.8%	0.990	3	12.1%	0.995	3	9.6%	0.993	1	3.3%	0.986	
FN1239	1	3.3%	0.893	0	0.0%	0.000	0	0.0%	0.000	1	7.0%	0.632	AAL95435.1| Hypothetical protein
	0	0.0%	0.000	5	22.0%	1.000	2	7.5%	0.976	0	0.0%	0.000	
FN1240	4	21.7%	1.000	3	12.5%	1.000	3	15.4%	0.999	3	12.1%	1.000	AAL95436.1| Lipopolysaccharide core biosynthesis protein rfaY
	4	16.7%	1.000	3	15.0%	0.997	3	19.2%	0.993	3	10.8%	0.970	
FN1241	3	10.3%	1.000	4	19.3%	1.000	3	9.1%	1.000	2	4.5%	0.999	AAL95437.1| polysaccharide biosynthesis protein
	3	9.9%	0.999	3	14.8%	0.999	7	33.3%	1.000	1	4.5%	0.297	
FN1242	5	19.2%	1.000	2	6.4%	0.994	8	27.5%	1.000	4	13.9%	1.000	AAL95438.1| Polysaccharide deacetylase
	8	31.4%	1.000	8	29.4%	1.000	9	31.9%	1.000	3	13.9%	0.998	
FN1243	7	27.3%	1.000	1	3.5%	0.984	7	19.2%	1.000	6	22.4%	1.000	AAL95439.1| Glycosyl transferase
	4	12.2%	1.000	5	18.9%	1.000	6	19.2%	1.000	5	18.5%	1.000	
FN1244	5	22.8%	1.000	3	10.8%	1.000	8	31.6%	1.000	4	15.2%	1.000	AAL95440.1| Polysaccharide deacetylase
	5	21.2%	1.000	13	48.4%	1.000	12	44.8%	1.000	4	18.0%	1.000	
FN1245	9	22.0%	1.000	3	9.4%	1.000	3	9.4%	1.000	2	5.8%	0.669	AAL95441.1| Glycosyl transferase
	6	11.3%	1.000	4	8.1%	1.000	5	10.5%	1.000	1	2.4%	0.386	
FN1246	7	26.4%	1.000	2	11.1%	0.958	6	31.8%	1.000	2	8.4%	0.995	AAL95442.1| Lipooligosaccharide cholinephosphotransferase
	5	21.8%	1.000	6	23.4%	1.000	6	30.3%	1.000	3	14.6%	1.000	
FN1247	8	16.9%	1.000	4	11.1%	1.000	10	29.8%	1.000	3	7.7%	1.000	AAL95443.1| LOS biosynthesis enzyme LBGB
	7	19.0%	1.000	9	25.1%	1.000	17	41.2%	1.000	3	10.6%	1.000	
FN1248	3	15.7%	1.000	2	5.4%	0.999	0	0.0%		0	0.0%		AAL95444.1| Hypothetical cytosolic protein
	2	10.3%	0.971	0	0.0%	0.000	0	0.0%		0	0.0%		
FN1250	8	8.3%	1.000	4	6.5%	1.000	6	6.7%	1.000	5	8.0%	1.000	AAL95446.1| Guanine-hypoxanthine permease
	7	6.7%	1.000	6	6.7%	1.000	6	6.7%	1.000	8	8.3%	1.000	
FN1251	5	16.4%	1.000	1	2.3%	0.984	6	13.9%	1.000	6	15.5%	1.000	AAL95447.1| High-affinity iron permease
	5	15.9%	1.000	4	12.2%	1.000	3	7.6%	1.000	5	15.5%	1.000	
FN1252	22	86.8%	1.000	18	86.8%	1.000	19	86.8%	1.000	24	83.8%	1.000	AAL95448.1| 34 kDa membrane antigen precursor
	23	79.8%	1.000	21	86.8%	1.000	20	94.3%	1.000	22	83.8%	1.000	

Show detected proteins only
Show all proteins
Filter by category:
GO: amino acid transport

Proteins found:
1424

Enter (or paste) list of ORFs

Find ORFs

Dot Plots Dot Plots

ORF	*Fn* n	*Fn* coverage	*Fn* prob	*FnPg* n	*FnPg* coverage	*FnPg* prob	*FnSg* n	*FnSg* coverage	*FnSg* prob	*FnPgSg* n	*FnPgSg* coverage	*FnPgSg* prob	Description
FN1253	6	72.4%	1.000	4	72.4%	1.000	4	72.4%	1.000	4	72.4%	1.000	AAL95449.1| unknown
	6	72.4%	1.000	4	72.4%	1.000	4	72.4%	1.000	7	96.6%	1.000	
FN1254	10	33.3%	1.000	7	27.5%	1.000	6	27.5%	1.000	11	33.9%	1.000	AAL95450.1| Oxygen-insensitive NAD(P)H nitroreductase
	11	33.3%	1.000	9	33.3%	1.000	7	33.3%	1.000	11	33.3%	1.000	
FN1255	5	15.6%	1.000	1	8.7%	0.487	0	0.0%	0.000	2	8.4%	1.000	AAL95451.1| NagD protein
	5	22.5%	1.000	1	6.9%	0.513	1	4.7%	0.330	2	8.4%	0.995	
FN1256	3	4.2%	0.933	0	0.0%	0.000	0	0.0%		2	4.9%	0.994	AAL95452.1| C4-dicarboxylate transporter large subunit
	2	4.0%	0.684	3	9.1%	0.999	0	0.0%		3	7.5%	1.000	
FN1258	37	48.0%	1.000	26	48.3%	1.000	26	50.8%	1.000	33	45.9%	1.000	AAL95454.1| C4-dicarboxylate-binding protein
	35	43.5%	1.000	23	48.0%	1.000	23	47.7%	1.000	34	43.5%	1.000	
FN1259	1	8.6%	0.829	0	0.0%	0.000	1	6.6%	0.981	2	12.2%	0.633	AAL95455.1| hypothetical protein
	1	6.6%	0.982	1	6.6%	0.982	4	19.8%	1.000	1	3.6%	0.473	
FN1260	3	8.0%	0.985	1	3.3%	0.421	2	2.8%	0.965	3	8.0%	0.896	AAL95456.1| Sensory Transduction Protein Kinase
	2	2.8%	0.995	1	2.8%	0.774	1	2.8%	0.932	1	4.2%	0.664	
FN1261	2	9.0%	0.965	4	12.9%	1.000	1	6.0%	0.330	0	0.0%		AAL95457.1| Two-component response regulator
	3	18.9%	0.963	1	6.0%	0.751	2	16.3%	0.991	0	0.0%		
FN1262	3	5.2%	0.965	0	0.0%	0.000	3	11.4%	1.000	0	0.0%		AAL95458.1| Integral membrane protein
	2	2.7%	0.945	3	6.7%	1.000	3	6.7%	1.000	0	0.0%		
FN1263	8	27.2%	1.000	3	21.9%	1.000	5	27.2%	1.000	8	33.9%	1.000	AAL95459.1| Cobalt chelatase
	7	30.7%	1.000	4	27.2%	1.000	9	31.8%	1.000	7	26.5%	1.000	
FN1264	4	39.2%	1.000	1	19.2%	0.356	4	40.0%	1.000	3	30.8%	1.000	AAL95460.1| Hypothetical protein
	6	39.2%	1.000	2	11.7%	0.999	4	48.3%	1.000	3	30.8%	1.000	
FN1265	9	44.1%	1.000	6	41.6%	1.000	12	64.9%	1.000	7	35.1%	1.000	AAL95461.1| Outer membrane protein
	10	44.1%	1.000	9	42.6%	1.000	10	59.9%	1.000	8	40.1%	1.000	
FN1266	14	51.8%	1.000	9	30.9%	1.000	16	52.8%	1.000	14	47.5%	1.000	AAL95462.1| UTP--glucose-1-phosphate uridylyltransferase
	18	54.2%	1.000	20	61.1%	1.000	17	52.8%	1.000	14	47.5%	1.000	
FN1267	8	23.9%	1.000	5	30.2%	1.000	9	40.0%	1.000	8	33.2%	1.000	AAL95463.1| Hypothetical protein
	8	27.3%	1.000	10	37.6%	1.000	5	27.8%	1.000	7	23.9%	1.000	
FN1268	45	58.6%	1.000	28	45.6%	1.000	32	48.7%	1.000	42	58.3%	1.000	AAL95464.1| Methionyl-tRNA synthetase
	42	53.8%	1.000	42	58.3%	1.000	33	46.7%	1.000	39	61.3%	1.000	
FN1269	5	31.1%	1.000	0	0.0%	0.000	3	23.0%	0.998	2	8.6%	0.941	AAL95465.1| Hypothetical lipoprotein
	6	27.3%	1.000	4	18.7%	0.999	2	17.7%	0.995	3	19.6%	0.999	

Fn Rep I Rep II | *FnPg* Rep I Rep II | *FnSg* Rep I Rep II | *FnPgSg* Rep I Rep II

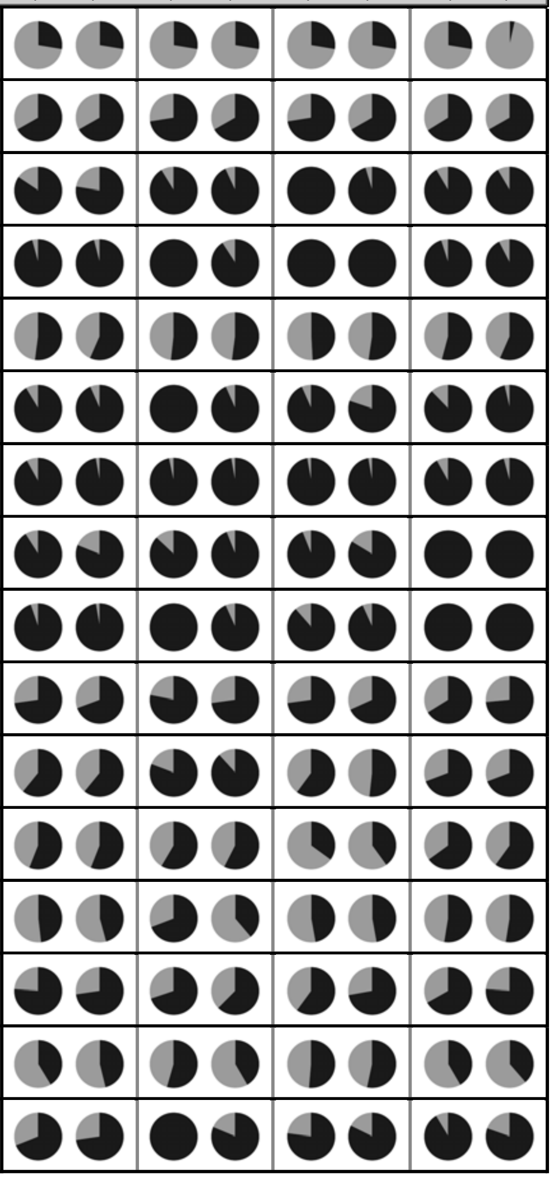

Show detected proteins only
Show all proteins
Filter by category:
GO: amino acid transport

Proteins found: 1424

Enter (or paste) list of ORFs

Find ORFs

Dot Plots Dot Plots

ORF	Fn n	Fn coverage	Fn prob	FnPg n	FnPg coverage	FnPg prob	FnSg n	FnSg coverage	FnSg prob	FnPgSg n	FnPgSg coverage	FnPgSg prob	Description
FN1270	6	48.9%	1.000	2	20.5%	1.000	3	42.0%	1.000	7	53.4%	1.000	AAL95466.1| Hypothetical cytosolic protein
	5	33.0%	1.000	5	51.1%	1.000	2	26.1%	1.000	8	69.3%	1.000	
FN1271	14	31.3%	1.000	6	8.7%	1.000	14	32.2%	1.000	14	36.3%	1.000	AAL95467.1| Protease IV
	15	33.6%	1.000	16	35.9%	1.000	12	27.1%	1.000	11	24.2%	1.000	
FN1273	14	43.6%	1.000	3	16.9%	0.999	13	45.0%	1.000	7	22.3%	1.000	AAL95469.1| Outer membrane protein tolC
	9	30.5%	1.000	17	47.0%	1.000	17	50.8%	1.000	7	22.8%	1.000	
FN1274	8	29.2%	1.000	3	7.3%	1.000	7	26.8%	1.000	9	29.7%	1.000	AAL95470.1| Acriflavin resistance protein E
	10	32.2%	1.000	7	21.6%	1.000	9	31.9%	1.000	5	16.5%	1.000	
FN1275	15	17.8%	1.000	5	8.4%	1.000	8	11.1%	1.000	9	11.9%	1.000	AAL95471.1| Acriflavin resistance protein B
	14	17.5%	1.000	11	12.7%	1.000	11	14.7%	1.000	8	9.6%	1.000	
FN1276	2	16.0%	0.962	6	37.4%	1.000	5	26.0%	1.000	4	25.2%	1.000	AAL95472.1| Hypothetical protein
	2	22.1%	0.999	6	48.1%	1.000	5	41.2%	1.000	5	37.4%	1.000	
FN1277	29	45.7%	1.000	23	43.6%	1.000	34	55.3%	1.000	32	57.0%	1.000	AAL95473.1| Aminoacyl-histidine dipeptidase
	29	48.4%	1.000	42	63.2%	1.000	30	58.6%	1.000	33	52.7%	1.000	
FN1278	1	5.4%	0.460	1	7.7%	0.981	1	7.7%	0.956	2	16.2%	0.993	AAL95474.1| Acetyltransferase
	1	7.7%	0.974	3	16.9%	0.995	6	39.2%	1.000	4	36.9%	1.000	
FN1279	16	47.9%	1.000	7	20.9%	1.000	10	28.5%	1.000	14	37.4%	1.000	AAL95475.1| Zinc metallohydrolase, glyoxalase II family
	18	58.6%	1.000	15	47.5%	1.000	11	31.0%	1.000	12	36.8%	1.000	
FN1280	4	7.9%	1.000	14	32.5%	1.000	17	36.6%	1.000	8	20.9%	1.000	AAL95476.1| Serine protease, V8 family
	6	14.0%	1.000	16	38.2%	1.000	19	37.8%	1.000	3	4.1%	1.000	
FN1281	4	10.6%	1.000	4	17.2%	1.000	7	21.6%	1.000	1	8.8%	0.739	AAL95477.1| Cysteine protease
	3	7.8%	1.000	7	23.8%	1.000	6	21.2%	1.000	2	7.8%	0.999	
FN1282	8	37.9%	1.000	7	24.1%	1.000	6	37.1%	1.000	8	37.9%	1.000	AAL95478.1| LSU ribosomal protein L17P
	9	44.0%	1.000	7	44.0%	1.000	6	37.1%	1.000	8	44.0%	1.000	
FN1283	33	68.1%	1.000	34	70.5%	1.000	29	68.7%	1.000	27	71.9%	1.000	AAL95479.1| DNA-directed RNA polymerase alpha chain
	34	68.1%	1.000	29	72.8%	1.000	26	64.3%	1.000	30	72.2%	1.000	
FN1284	27	57.9%	1.000	19	53.3%	1.000	22	70.8%	1.000	28	57.9%	1.000	AAL95480.1| SSU ribosomal protein S4P
	27	59.0%	1.000	21	62.1%	1.000	21	70.8%	1.000	26	53.3%	1.000	
FN1285	7	59.7%	1.000	10	62.0%	1.000	3	47.3%	1.000	7	59.7%	1.000	AAL95481.1| SSU ribosomal protein S11P
	5	47.3%	1.000	4	47.3%	1.000	5	47.3%	1.000	7	59.7%	1.000	
FN1286	8	39.8%	1.000	5	25.4%	1.000	5	27.1%	1.000	10	39.8%	1.000	AAL95482.1| SSU ribosomal protein S13P
	7	39.8%	1.000	6	41.5%	1.000	6	39.8%	1.000	10	39.8%	1.000	

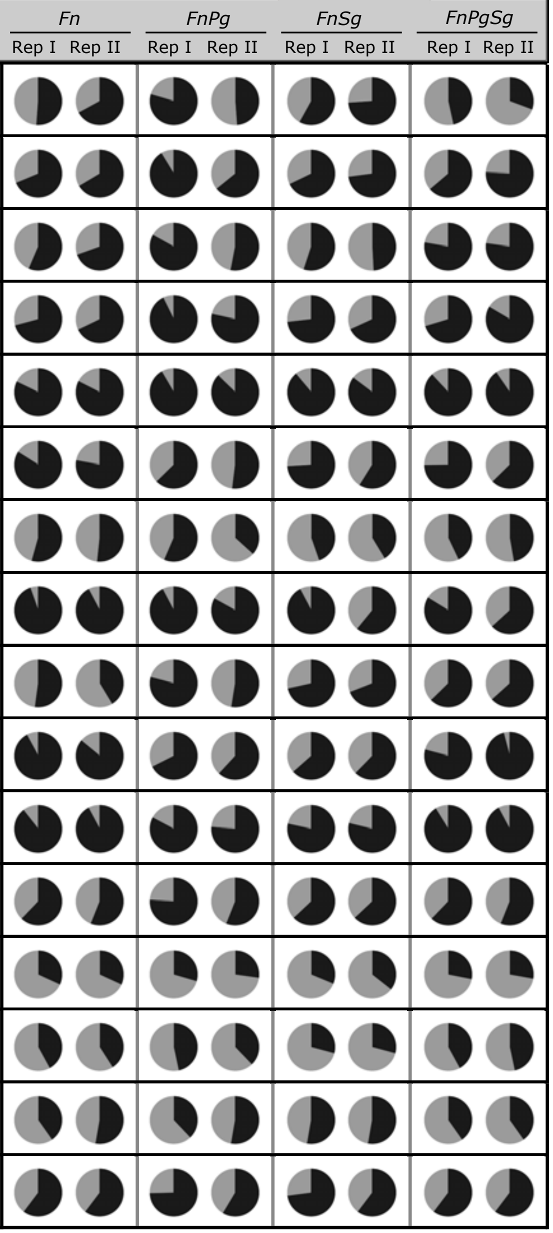

Show detected proteins only
Show all proteins
Filter by category:
GO: amino acid transport

Proteins found: 1424

Enter (or paste) list of ORFs

Find ORFs

Dot Plots Dot Plots

ORF	*Fn* n	*Fn* coverage	*Fn* prob	*FnPg* n	*FnPg* coverage	*FnPg* prob	*FnSg* n	*FnSg* coverage	*FnSg* prob	*FnPgSg* n	*FnPgSg* coverage	*FnPgSg* prob	Description
**FN1287**	3	41.5%	1.000	3	41.5%	1.000	3	41.5%	1.000	3	41.5%	1.000	AAL95483.1| Bacterial Protein Translation Initiation Factor 1 (IF -1)
	3	41.5%	1.000	3	41.5%	1.000	3	41.5%	1.000	3	41.5%	1.000	
**FN1290**	8	15.9%	1.000	11	32.1%	1.000	6	22.6%	1.000	3	12.9%	0.996	AAL95486.1| Hypothetical protein
	13	39.4%	1.000	4	13.5%	1.000	5	23.5%	1.000	7	19.7%	1.000	
**FN1293**	3	5.3%	1.000	0	0.0%		3	6.1%	0.723	2	5.3%	0.995	AAL95489.1| Hypothetical protein
	3	5.3%	1.000	0	0.0%		2	4.6%	0.997	3	7.4%	1.000	
**FN1296**	8	58.6%	1.000	0	0.0%		0	0.0%		7	52.7%	1.000	AAL95492.1| unknown
	9	65.1%	1.000	0	0.0%		0	0.0%		3	20.1%	1.000	
**FN1297**	10	33.1%	1.000	6	29.1%	1.000	10	42.1%	1.000	13	52.0%	1.000	AAL95493.1| Methionine aminopeptidase
	10	33.1%	1.000	11	39.4%	1.000	11	39.4%	1.000	13	46.1%	1.000	
**FN1298**	17	51.2%	1.000	18	47.4%	1.000	11	37.4%	1.000	14	50.7%	1.000	AAL95494.1| Adenylate kinase
	16	51.2%	1.000	18	58.3%	1.000	11	45.0%	1.000	14	51.2%	1.000	
**FN1299**	2	8.4%	0.979	1	5.8%	0.813	3	14.6%	0.919	1	5.8%	0.710	AAL95495.1| dTDP-glucose 4,6-dehydratase
	1	5.8%	0.979	5	20.1%	1.000	5	19.4%	1.000	2	10.7%	0.999	
**FN1301**	21	36.5%	1.000	15	25.0%	1.000	16	34.9%	1.000	18	39.1%	1.000	AAL95497.1| ABC transporter ATP-binding protein
	22	36.7%	1.000	11	20.0%	1.000	18	36.0%	1.000	20	30.6%	1.000	
**FN1302**	9	72.2%	1.000	7	68.1%	1.000	5	69.4%	1.000	7	59.7%	1.000	AAL95498.1| Hypothetical protein
	8	62.5%	1.000	7	80.6%	1.000	6	73.6%	1.000	9	81.9%	1.000	
**FN1303**	7	35.4%	1.000	5	23.4%	1.000	8	45.3%	1.000	9	41.7%	1.000	AAL95499.1| hypothetical cytosolic protein
	7	35.4%	1.000	8	47.9%	1.000	9	50.0%	1.000	9	41.7%	1.000	
**FN1304**	17	76.0%	1.000	10	38.3%	1.000	12	55.8%	1.000	16	75.3%	1.000	AAL95500.1| Single-strand DNA binding protein
	16	68.8%	1.000	11	44.8%	1.000	14	76.0%	1.000	14	75.3%	1.000	
**FN1305**	3	46.8%	1.000	4	40.5%	1.000	6	57.7%	1.000	3	40.5%	1.000	AAL95501.1| Hypothetical cytosolic protein
	5	46.8%	1.000	8	73.0%	1.000	5	50.5%	1.000	3	40.5%	1.000	
**FN1306**	24	41.9%	1.000	2	4.8%	0.476	34	47.2%	1.000	11	22.8%	1.000	AAL95502.1| Methyltransferase
	23	36.9%	1.000	29	44.3%	1.000	32	46.8%	1.000	12	20.3%	1.000	
**FN1309**	11	100.0%	1.000	10	100.0%	1.000	6	100.0%	1.000	9	100.0%	1.000	AAL95505.1| Hypothetical protein
	11	100.0%	1.000	5	100.0%	1.000	6	100.0%	1.000	10	100.0%	1.000	
**FN1311**	2	29.0%	0.999	0	0.0%	0.000	2	11.0%	0.991	0	0.0%	0.000	AAL95507.1| Biopolymer transport exbD protein
	3	48.0%	1.000	1	11.0%	0.983	2	11.0%	1.000	1	11.0%	0.982	
**FN1312**	7	26.2%	1.000	0	0.0%	0.000	2	14.4%	1.000	2	14.9%	0.943	AAL95508.1| Biopolymer transport exbB protein
	6	26.2%	1.000	2	11.9%	0.999	2	11.9%	0.996	2	11.9%	0.957	

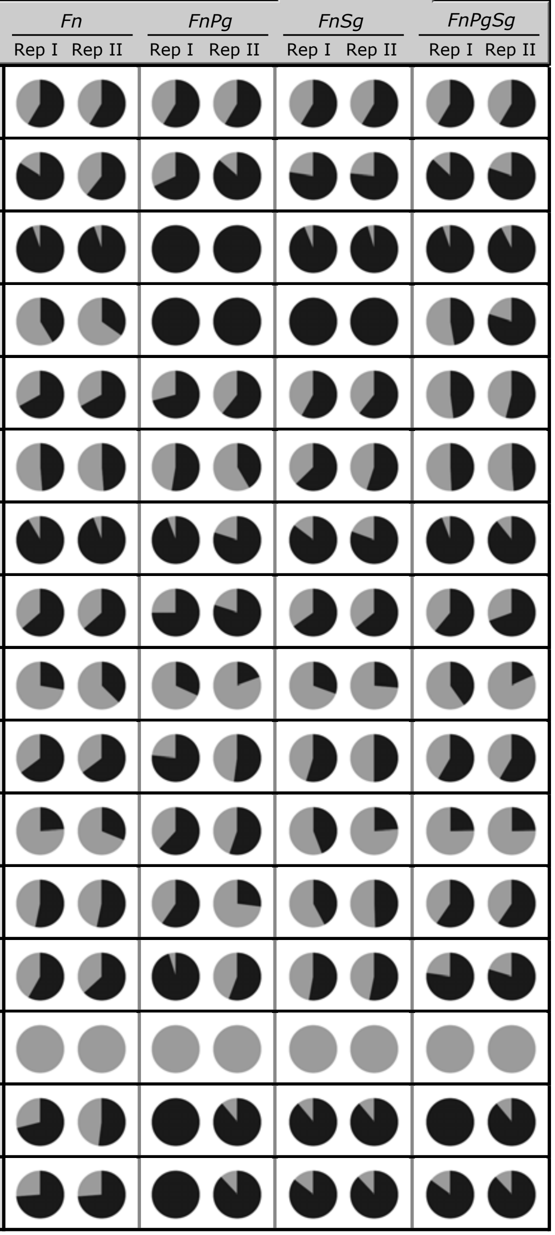

Show detected proteins only
Show all proteins
Filter by category:
GO: amino acid transport

Proteins found: 1424

Enter (or paste) list of ORFs

Find ORFs

Dot Plots Dot Plots

ORF	Fn n	Fn coverage	Fn prob	FnPg n	FnPg coverage	FnPg prob	FnSg n	FnSg coverage	FnSg prob	FnPgSg n	FnPgSg coverage	FnPgSg prob	Description
FN1313	19	54.6%	1.000	9	32.1%	1.000	23	59.1%	1.000	16	45.1%	1.000	AAL95509.1| Oligopeptide-binding protein oppA
	18	50.6%	1.000	18	53.4%	1.000	17	45.6%	1.000	13	44.5%	1.000	
FN1315	3	29.9%	0.998	0	0.0%	0.000	3	17.5%	1.000	2	17.5%	0.994	AAL95511.1| Hypothetical protein
	2	13.6%	1.000	1	13.6%	0.973	1	13.6%	0.985	1	13.6%	0.979	
FN1317	7	25.6%	1.000	6	17.0%	1.000	5	16.3%	1.000	2	10.4%	0.993	AAL95513.1| RNA polymerase sigma factor
	6	21.9%	1.000	5	24.1%	1.000	4	17.0%	1.000	0	0.0%	0.000	
FN1318	10	31.1%	1.000	6	25.4%	1.000	6	21.8%	1.000	11	36.3%	1.000	AAL95514.1| RNA polymerase sigma factor rpoD
	10	31.1%	1.000	10	33.2%	1.000	6	28.1%	1.000	12	36.0%	1.000	
FN1319	5	5.0%	1.000	5	7.3%	1.000	5	8.6%	1.000	4	5.8%	1.000	AAL95515.1| DNA primase
	7	10.3%	1.000	7	12.1%	1.000	12	16.4%	1.000	7	13.9%	1.000	
FN1320	34	62.4%	1.000	19	52.5%	1.000	24	60.4%	1.000	34	62.4%	1.000	AAL95516.1| Peptidyl-prolyl cis-trans isomerase
	33	64.6%	1.000	26	63.5%	1.000	24	64.0%	1.000	31	63.8%	1.000	
FN1321	49	68.4%	1.000	45	62.3%	1.000	47	73.6%	1.000	40	62.0%	1.000	AAL95517.1| Acetoacetate metabolism regulatory protein atoC
	44	62.5%	1.000	51	73.8%	1.000	47	70.4%	1.000	42	64.2%	1.000	
FN1322	5	17.1%	1.000	1	2.9%	0.933	7	13.9%	1.000	3	8.6%	0.995	AAL95518.1| Membrane metalloprotease
	6	19.5%	1.000	10	29.2%	1.000	8	20.1%	1.000	2	4.7%	0.995	
FN1323	8	29.8%	1.000	4	16.9%	1.000	3	10.2%	1.000	3	17.3%	1.000	AAL95519.1| Thymidylate kinase
	6	17.3%	1.000	6	29.3%	1.000	3	14.7%	1.000	6	23.6%	1.000	
FN1324	10	29.5%	1.000	8	25.1%	1.000	15	43.6%	1.000	6	21.8%	1.000	AAL95520.1| 1-deoxy-D-xylulose 5-phosphate reductoisomerase
	11	37.4%	1.000	15	45.6%	1.000	14	40.8%	1.000	8	21.5%	1.000	
FN1326	3	16.5%	1.000	1	4.8%	0.984	2	9.1%	0.936	2	10.9%	1.000	AAL95522.1| Undecaprenyl pyrophosphate synthetase
	3	16.5%	1.000	6	24.8%	1.000	3	13.0%	1.000	4	20.0%	1.000	
FN1327	2	11.1%	0.996	1	8.4%	0.757	6	31.3%	1.000	4	21.9%	0.999	AAL95523.1| Dimethylallyltransferase
	3	14.5%	0.996	2	7.4%	0.998	5	24.9%	1.000	2	7.4%	0.998	
FN1328	3	51.4%	1.000	1	34.3%	0.749	1	34.3%	0.821	3	51.4%	0.999	AAL95524.1| Exodeoxyribonuclease VII small subunit
	3	51.4%	1.000	1	34.3%	0.577	1	34.3%	0.985	3	47.1%	0.993	
FN1330	3	8.0%	0.702	0	0.0%	0.000	0	0.0%	0.000	1	5.7%	0.749	AAL95526.1| S-adenosylmethionine:tRNA ribosyltransferase-isomerase
	1	4.3%	0.242	3	15.7%	0.928	3	15.1%	0.709	2	8.8%	0.993	
FN1331	4	16.7%	1.000	1	5.4%	0.936	1	3.4%	0.970	5	19.2%	1.000	AAL95527.1| Methyltransferase
	4	20.6%	1.000	1	3.4%	0.259	1	3.4%	0.738	3	11.9%	1.000	
FN1332	15	36.2%	1.000	9	27.9%	1.000	5	17.3%	1.000	13	31.0%	1.000	AAL95528.1| Bacterial Peptide Chain Release Factor 1 (RF-1)
	13	33.4%	1.000	14	38.1%	1.000	8	25.8%	1.000	12	28.8%	1.000	

Show detected proteins only
Show all proteins
Filter by category:
GO: amino acid transport

Proteins found: 1424

Enter (or paste) list of ORFs

Find ORFs

Dot Plots Dot Plots

ORF	Fn n	Fn coverage	Fn prob	FnPg n	FnPg coverage	FnPg prob	FnSg n	FnSg coverage	FnSg prob	FnPgSg n	FnPgSg coverage	FnPgSg prob	Description
FN1333	2	18.1%	0.975	1	10.1%	0.966	4	23.9%	1.000	2	23.2%	0.988	AAL95529.1| Hypothetical protein
	2	18.1%	0.999	2	12.3%	1.000	8	52.2%	1.000	2	12.3%	0.990	
FN1334	8	23.4%	1.000	4	9.2%	1.000	3	6.2%	0.993	6	15.7%	1.000	AAL95530.1| N-acetylmuramoyl-L-alanine amidase
	6	13.0%	1.000	5	16.9%	1.000	4	13.6%	1.000	2	3.6%	1.000	
FN1335	7	55.3%	1.000	5	41.5%	1.000	7	54.3%	1.000	10	55.3%	1.000	AAL95531.1| Protein translocase subunit YajC
	7	55.3%	1.000	7	55.3%	1.000	8	81.9%	1.000	8	55.3%	1.000	
FN1336	4	27.9%	1.000	5	31.1%	1.000	2	27.9%	1.000	2	10.7%	1.000	AAL95532.1| Hypothetical protein
	5	37.7%	1.000	2	27.9%	0.994	4	28.7%	1.000	2	27.9%	0.989	
FN1337	5	18.1%	1.000	3	9.3%	1.000	0	0.0%	0.000	4	17.6%	1.000	AAL95533.1| unknown
	10	30.5%	1.000	3	13.1%	0.996	5	13.4%	1.000	2	12.3%	0.994	
FN1340	37	54.5%	1.000	37	51.9%	1.000	32	52.9%	1.000	31	54.8%	1.000	AAL95536.1| Glutamyl-tRNA synthetase
	32	52.7%	1.000	32	54.8%	1.000	36	58.3%	1.000	35	58.9%	1.000	
FN1341	6	20.5%	1.000	1	8.8%	0.983	3	13.6%	1.000	7	26.0%	1.000	AAL95537.1| Bacterial Peptide Chain Release Factor 2 (RF-2)
	4	13.3%	1.000	5	13.6%	1.000	4	10.1%	1.000	6	23.7%	1.000	
FN1343	5	28.3%	1.000	1	9.3%	0.419	6	28.3%	1.000	0	0.0%	0.000	AAL95539.1| seC-independent protein TATD
	2	7.8%	0.997	10	39.1%	1.000	6	28.3%	1.000	2	15.1%	0.988	
FN1345	2	12.0%	0.918	0	0.0%	0.000	1	5.8%	0.380	1	5.8%	0.562	AAL95541.1| 2-hydroxy-6-oxo-6-phenylhexa-2,4-dienoate hydrolase
	0	0.0%	0.000	0	0.0%	0.000	3	21.5%	1.000	1	5.8%	0.596	
FN1346	1	8.5%	0.933	0	0.0%	0.000	4	28.0%	1.000	0	0.0%		AAL95542.1| Hypothetical cytosolic protein
	1	8.5%	0.510	3	19.0%	1.000	5	28.0%	1.000	0	0.0%		
FN1347	5	27.5%	1.000	4	25.2%	1.000	3	16.5%	0.980	4	26.1%	1.000	AAL95543.1| Hypothetical cytosolic protein
	4	27.5%	1.000	5	30.3%	1.000	5	27.1%	1.000	4	26.1%	1.000	
FN1348	7	44.4%	1.000	3	13.5%	1.000	4	30.0%	1.000	7	44.4%	1.000	AAL95544.1| ABC transporter ATP-binding protein
	5	26.9%	1.000	7	40.8%	1.000	9	43.5%	1.000	3	20.2%	0.999	
FN1349	4	9.2%	1.000	1	6.5%	0.371	3	8.0%	1.000	3	10.2%	0.999	AAL95545.1| ABC transporter permease protein
	4	9.2%	1.000	3	9.7%	1.000	4	8.7%	1.000	2	6.5%	0.979	
FN1351	7	46.4%	1.000	5	46.4%	1.000	8	46.4%	1.000	5	37.1%	1.000	AAL95547.1| 15 kDa lipoprotein precursor
	6	37.1%	1.000	9	46.4%	1.000	9	46.4%	1.000	6	59.3%	1.000	
FN1352	13	61.6%	1.000	8	49.1%	1.000	11	55.4%	1.000	10	49.6%	1.000	AAL95548.1| ABC transporter ATP-binding protein
	10	51.8%	1.000	14	61.6%	1.000	12	54.9%	1.000	10	46.0%	1.000	
FN1353	5	5.0%	1.000	2	11.2%	0.872	4	6.2%	1.000	7	15.8%	1.000	AAL95549.1| ABC transporter permease protein
	4	8.8%	1.000	6	9.8%	1.000	7	20.5%	1.000	5	13.0%	1.000	

Fn: Rep I, Rep II | FnPg: Rep I, Rep II | FnSg: Rep I, Rep II | FnPgSg: Rep I, Rep II

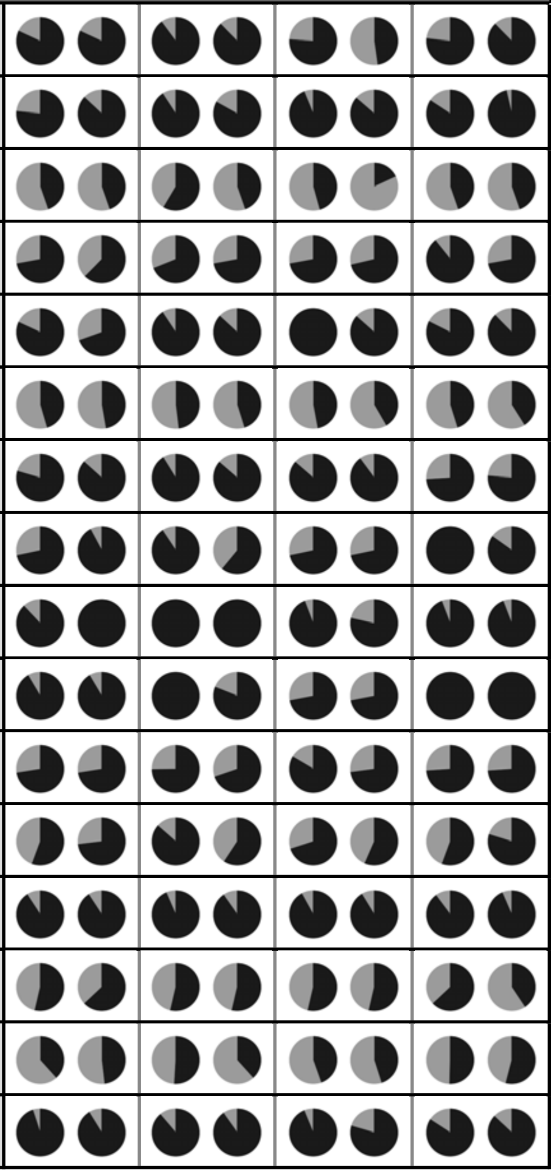

Show detected proteins only
Show all proteins
Filter by category:
GO: amino acid transport

Proteins found: 1424

Enter (or paste) list of ORFs

Find ORFs

Dot Plots Dot Plots

ORF	Fn n	Fn coverage	Fn prob	FnPg n	FnPg coverage	FnPg prob	FnSg n	FnSg coverage	FnSg prob	FnPgSg n	FnPgSg coverage	FnPgSg prob	Description
FN1354	9	26.2%	1.000	2	8.2%	0.987	7	22.2%	1.000	11	25.7%	1.000	AAL95550.1| ABC transporter permease protein
	9	23.8%	1.000	11	26.4%	1.000	8	23.6%	1.000	9	34.1%	1.000	
FN1355	3	15.1%	1.000	1	5.7%	0.456	2	11.7%	0.993	3	13.1%	0.948	AAL95551.1| Integral membrane protein
	4	22.5%	1.000	2	8.4%	1.000	0	0.0%	0.000	2	8.4%	0.999	
FN1358	2	11.2%	1.000	1	5.2%	0.984	4	15.9%	1.000	7	31.3%	1.000	AAL95554.1| Hypothetical protein
	2	11.2%	1.000	6	42.5%	1.000	9	33.9%	1.000	8	23.6%	1.000	
FN1359	20	39.0%	1.000	19	39.6%	1.000	41	62.4%	1.000	25	49.5%	1.000	AAL95555.1| Dipeptide-binding protein
	17	34.1%	1.000	45	63.6%	1.000	40	62.2%	1.000	21	46.1%	1.000	
FN1362	2	14.6%	0.996	7	30.0%	1.000	15	53.2%	1.000	0	0.0%	0.000	AAL95558.1| Dipeptide transport ATP-binding protein dppD
	2	10.7%	0.719	16	44.6%	1.000	14	41.2%	1.000	2	12.9%	0.990	
FN1363	4	24.4%	1.000	7	40.8%	1.000	13	62.4%	1.000	4	22.8%	1.000	AAL95559.1| Dipeptide transport ATP-binding protein dppF
	4	27.2%	1.000	12	49.2%	1.000	14	62.4%	1.000	3	18.4%	0.993	
FN1364	4	40.7%	1.000	5	27.1%	1.000	2	27.1%	0.992	5	40.7%	1.000	AAL95560.1| LSU ribosomal protein L32P
	4	42.4%	1.000	2	25.4%	1.000	2	25.4%	1.000	4	40.7%	1.000	
FN1365	21	61.8%	1.000	13	40.9%	1.000	21	63.5%	1.000	18	55.2%	1.000	AAL95561.1| GTP-binding protein
	18	55.2%	1.000	21	62.4%	1.000	20	51.9%	1.000	20	57.7%	1.000	
FN1366	21	62.5%	1.000	15	51.0%	1.000	18	68.5%	1.000	24	66.9%	1.000	AAL95562.1| Triosephosphate isomerase
	23	71.3%	1.000	25	76.5%	1.000	16	63.7%	1.000	27	70.5%	1.000	
FN1368	0	0.0%		0	0.0%		0	0.0%	0.000	1	4.4%	0.346	AAL95564.1| COMF operon protein 3
	0	0.0%		0	0.0%		3	14.2%	0.935	2	9.8%	0.760	
FN1371	1	7.0%	0.239	0	0.0%	0.000	1	7.0%	0.779	0	0.0%	0.000	AAL95567.1| Ribonuclease HII
	1	4.7%	0.817	3	14.4%	0.985	3	18.1%	0.999	1	6.0%	0.601	
FN1373	1	8.0%	0.675	1	8.0%	0.402	2	8.0%	0.997	2	15.4%	0.995	AAL95569.1| regulatory protein
	0	0.0%	0.000	0	0.0%	0.000	3	13.0%	1.000	1	8.0%	0.310	
FN1374	4	22.8%	1.000	2	15.7%	0.559	2	15.0%	0.997	4	19.5%	1.000	AAL95570.1| Transcriptional regulator
	4	17.6%	1.000	3	13.5%	0.998	5	24.3%	1.000	2	15.0%	0.946	
FN1375	4	10.4%	1.000	2	6.6%	0.990	6	19.4%	1.000	5	10.1%	1.000	AAL95571.1| Citrate-sodium symport
	6	12.3%	1.000	8	25.8%	1.000	8	31.1%	1.000	5	10.4%	1.000	
FN1376	37	65.6%	1.000	29	57.6%	1.000	33	79.2%	1.000	36	73.7%	1.000	AAL95572.1| Oxaloacetate decarboxylase alpha chain
	35	67.2%	1.000	34	81.2%	1.000	35	78.1%	1.000	31	62.7%	1.000	
FN1377	0	0.0%		0	0.0%	0.000	3	9.7%	0.994	2	5.7%	0.609	AAL95573.1| CITG protein
	0	0.0%		2	5.7%	0.980	2	9.0%	0.948	1	4.7%	0.978	

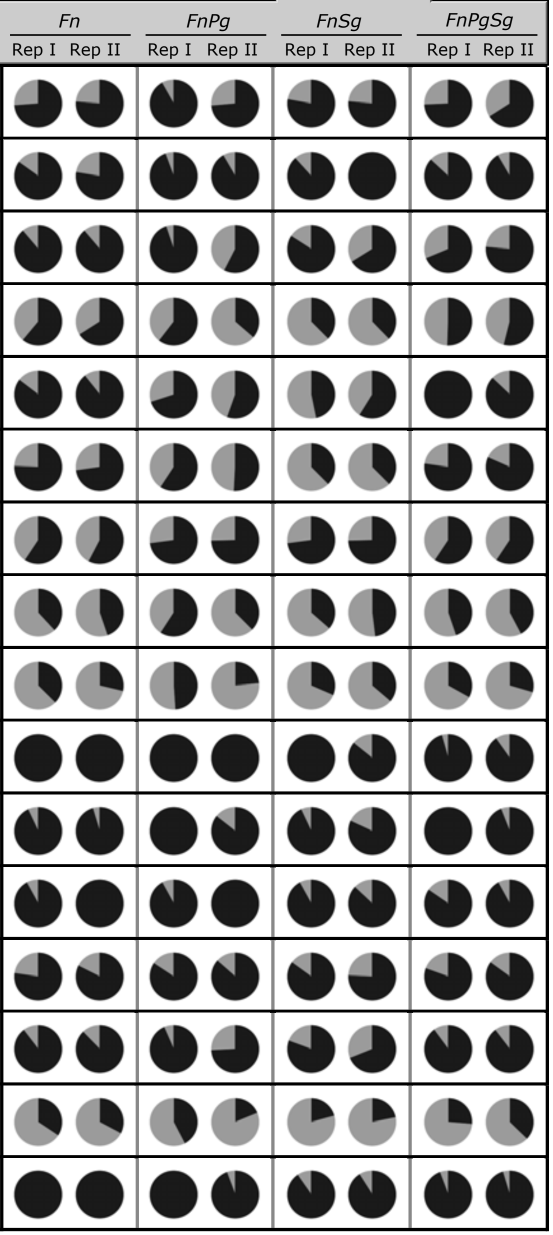

Show detected proteins only
Show all proteins
Filter by category:
GO: amino acid transport
Proteins found: 1424
Enter (or paste) list of ORFs
Find ORFs
Dot Plots Dot Plots

ORF	*Fn* n	*Fn* coverage	*Fn* prob	*FnPg* n	*FnPg* coverage	*FnPg* prob	*FnSg* n	*FnSg* coverage	*FnSg* prob	*FnPgSg* n	*FnPgSg* coverage	*FnPgSg* prob	Description
**FN1378**	4	42.6%	1.000	3	22.3%	1.000	3	31.9%	1.000	5	31.9%	1.000	AAL95574.1| Citrate lyase acyl carrier protein
	4	36.2%	1.000	7	46.8%	1.000	2	22.3%	1.000	5	31.9%	1.000	
**FN1379**	21	43.9%	1.000	10	33.8%	1.000	19	58.8%	1.000	16	43.9%	1.000	AAL95575.1| Citrate lyase beta chain
	19	46.3%	1.000	22	66.2%	1.000	20	62.8%	1.000	17	56.4%	1.000	
**FN1380**	32	54.8%	1.000	19	41.9%	1.000	32	63.4%	1.000	28	56.0%	1.000	AAL95576.1| Citrate lyase beta chain
	29	47.5%	1.000	35	66.9%	1.000	30	61.2%	1.000	29	47.3%	1.000	
**FN1381**	3	3.2%	1.000	0	0.0%	0.000	1	1.0%	0.364	2	1.8%	0.846	AAL95577.1| unknown
	3	3.1%	0.999	1	1.2%	0.276	1	1.0%	0.346	3	2.6%	0.770	
**FN1382**	2	5.0%	0.993	1	2.2%	0.983	2	5.2%	1.000	2	5.2%	0.900	AAL95578.1| ATPase
	2	5.2%	0.998	6	12.7%	1.000	4	11.4%	1.000	1	3.0%	0.328	
**FN1383**	1	0.9%	0.817	5	6.7%	1.000	9	9.4%	1.000	3	4.2%	0.834	AAL95579.1| DNA polymerase III alpha subunit
	2	3.1%	0.898	5	4.5%	1.000	9	7.9%	1.000	0	0.0%	0.000	
**FN1385**	4	7.8%	1.000	0	0.0%	0.000	3	6.2%	0.992	2	5.0%	0.978	AAL95581.1| Hypothetical protein
	5	10.2%	1.000	3	6.1%	1.000	6	12.6%	1.000	1	2.4%	0.459	
**FN1386**	4	6.3%	0.997	2	3.6%	0.982	9	12.7%	1.000	9	11.2%	1.000	AAL95582.1| SWF/SNF family helicase
	5	7.2%	1.000	9	13.2%	1.000	15	18.3%	1.000	6	8.2%	1.000	
**FN1391**	14	79.8%	1.000	8	47.2%	1.000	12	79.8%	1.000	14	69.7%	1.000	AAL95584.1| Acetyltransferase
	15	79.8%	1.000	12	68.5%	1.000	7	52.8%	1.000	15	69.7%	1.000	
**FN1392**	8	47.1%	1.000	8	36.8%	1.000	6	47.1%	1.000	11	47.1%	1.000	AAL95585.1| SSU ribosomal protein S16P
	8	47.1%	1.000	6	47.1%	1.000	6	47.1%	1.000	9	47.1%	1.000	
**FN1393**	15	29.7%	1.000	4	8.8%	1.000	14	25.2%	1.000	11	24.8%	1.000	AAL95586.1| Signal recognition particle, subunit FFH/SRP54
	19	29.5%	1.000	12	23.2%	1.000	14	25.7%	1.000	10	19.8%	1.000	
**FN1397**	17	54.3%	1.000	7	23.0%	1.000	13	47.4%	1.000	19	47.0%	1.000	AAL95590.1| Glutaminase
	21	57.9%	1.000	16	50.3%	1.000	15	51.0%	1.000	16	47.0%	1.000	
**FN1398**	15	17.7%	1.000	4	11.1%	1.000	8	16.6%	1.000	13	19.6%	1.000	AAL95591.1| Amino acid carrier protein alsT
	12	19.1%	1.000	9	18.7%	1.000	8	16.6%	1.000	15	21.3%	1.000	
**FN1399**	0	0.0%	0.000	1	3.5%	0.408	2	6.6%	0.629	0	0.0%		AAL95592.1| Hypothetical cytosolic protein
	1	3.5%	0.289	1	4.7%	0.274	3	12.6%	0.959	0	0.0%		
**FN1400**	0	0.0%	0.000	3	4.5%	0.978	2	2.3%	0.990	1	1.3%	0.616	AAL95593.1| serine/threonine kinase
	1	0.9%	0.357	3	3.3%	0.985	2	2.4%	0.988	4	4.9%	0.994	
**FN1406**	11	26.0%	1.000	5	12.7%	1.000	11	37.8%	1.000	12	26.0%	1.000	AAL95599.1| Histidine ammonia-lyase
	13	37.2%	1.000	22	49.3%	1.000	16	36.2%	1.000	12	24.7%	1.000	

Fn Rep I Rep II | *FnPg* Rep I Rep II | *FnSg* Rep I Rep II | *FnPgSg* Rep I Rep II

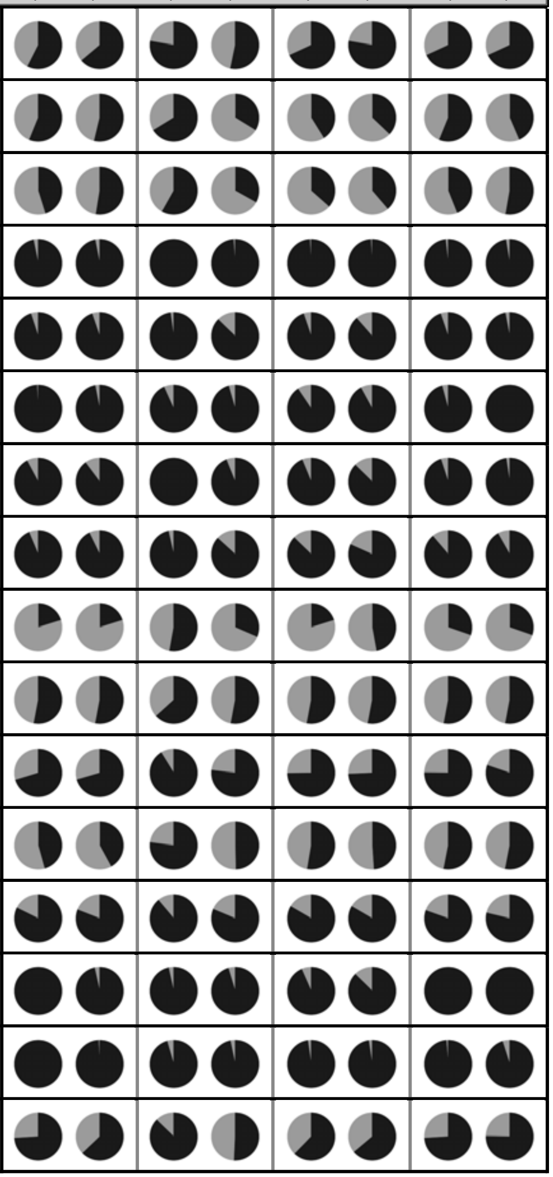

Show detected proteins only
Show all proteins
Filter by category:
GO: amino acid transport

Proteins found:
1424

Enter (or paste) list of ORFs

Find ORFs

Dot Plots Dot Plots

Fusobacterium nucleatum

ORF	*Fn* n	*Fn* coverage	*Fn* prob	*FnPg* n	*FnPg* coverage	*FnPg* prob	*FnSg* n	*FnSg* coverage	*FnSg* prob	*FnPgSg* n	*FnPgSg* coverage	*FnPgSg* prob	Description
FN1407	5	25.6%	1.000	2	9.6%	0.989	8	34.5%	1.000	4	16.4%	1.000	AAL95600.1| Glutamate formiminotransferase
	5	25.6%	1.000	12	51.2%	1.000	9	44.4%	1.000	4	13.0%	1.000	
FN1411	17	34.9%	1.000	22	44.1%	1.000	29	55.9%	1.000	18	31.2%	1.000	AAL95604.1| Threonine dehydratase
	17	34.7%	1.000	35	60.1%	1.000	30	58.7%	1.000	19	34.4%	1.000	
FN1412	1	1.8%	0.482	6	17.2%	1.000	3	9.1%	1.000	2	7.0%	0.992	AAL95605.1| 5-methylthioribose kinase
	0	0.0%	0.000	2	6.8%	0.992	4	9.4%	1.000	2	3.9%	0.823	
FN1413	1	8.3%	0.981	4	22.1%	1.000	0	0.0%	0.000	3	12.9%	0.989	AAL95606.1| Translation initiation factor EIF-2B subunit 1
	1	8.3%	0.982	2	8.6%	0.986	4	11.5%	1.000	1	3.2%	0.981	
FN1415	1	4.4%	0.721	4	18.2%	1.000	1	2.1%	0.880	2	3.9%	0.862	AAL95608.1| NADH-dependent butanol dehydrogenase A
	0	0.0%	0.000	6	17.9%	1.000	6	17.7%	1.000	1	2.1%	0.985	
FN1416	1	4.2%	0.350	2	7.5%	0.715	1	5.0%	0.725	4	12.3%	0.991	AAL95609.1| Transcriptional regulator, GntR family
	0	0.0%	0.000	3	7.9%	0.924	4	14.0%	1.000	3	9.4%	0.997	
FN1417	2	12.6%	0.999	5	18.0%	1.000	5	23.9%	1.000	3	12.2%	0.998	AAL95610.1| L-fuculose phosphate aldolase
	3	17.6%	1.000	7	37.8%	1.000	3	19.8%	1.000	2	11.7%	0.978	
FN1418	1	3.2%	0.446	3	10.7%	0.999	3	3.6%	0.905	2	5.1%	0.850	AAL95611.1| Transcriptional regulator, GntR family
	4	8.6%	0.999	2	6.5%	0.751	3	7.4%	0.889	2	4.8%	0.990	
FN1419	32	76.7%	1.000	54	86.8%	1.000	42	89.4%	1.000	45	85.8%	1.000	AAL95612.1| Methionine gamma-lyase
	32	77.5%	1.000	43	89.4%	1.000	40	87.1%	1.000	42	83.3%	1.000	
FN1420	1	1.8%	0.953	6	15.1%	1.000	2	5.6%	0.997	3	4.7%	0.999	AAL95613.1| NA+/H+ antiporter NHAC
	1	1.8%	0.967	4	5.8%	1.000	2	4.7%	0.990	2	4.7%	1.000	
FN1421	67	48.2%	1.000	107	65.0%	1.000	82	56.5%	1.000	86	59.6%	1.000	AAL95614.1| Pyruvate-flavodoxin oxidoreductase
	68	49.0%	1.000	98	69.1%	1.000	91	65.2%	1.000	81	59.3%	1.000	
FN1423	21	48.9%	1.000	12	32.6%	1.000	29	60.0%	1.000	27	62.2%	1.000	AAL95616.1| Flavoprotein
	23	45.9%	1.000	27	51.9%	1.000	31	60.7%	1.000	28	51.6%	1.000	
FN1424	35	60.1%	1.000	14	36.6%	1.000	34	64.0%	1.000	38	67.3%	1.000	AAL95617.1| ACYL-COA dehydrogenase, short-chain specific
	36	64.0%	1.000	31	60.8%	1.000	41	65.1%	1.000	34	66.0%	1.000	
FN1426	48	49.4%	1.000	9	15.4%	1.000	39	44.4%	1.000	31	40.6%	1.000	AAL95619.1| Serine protease
	49	48.9%	1.000	44	46.3%	1.000	48	46.0%	1.000	30	40.6%	1.000	
FN1427	2	14.3%	0.987	1	4.4%	0.387	2	9.2%	0.989	3	18.1%	1.000	AAL95620.1| Phenazine biosynthesis protein phzF
	2	14.3%	0.999	5	24.2%	1.000	2	8.2%	0.994	3	18.1%	0.997	
FN1429	3	15.8%	0.999	2	10.8%	0.879	1	3.8%	0.340	2	14.2%	0.997	AAL95622.1| Branched-chain amino acid transport ATP-binding protein livG
	1	10.4%	0.922	0	0.0%	0.000	0	0.0%	0.000	0	0.0%	0.000	

Fn Rep I Rep II | *FnPg* Rep I Rep II | *FnSg* Rep I Rep II | *FnPgSg* Rep I Rep II

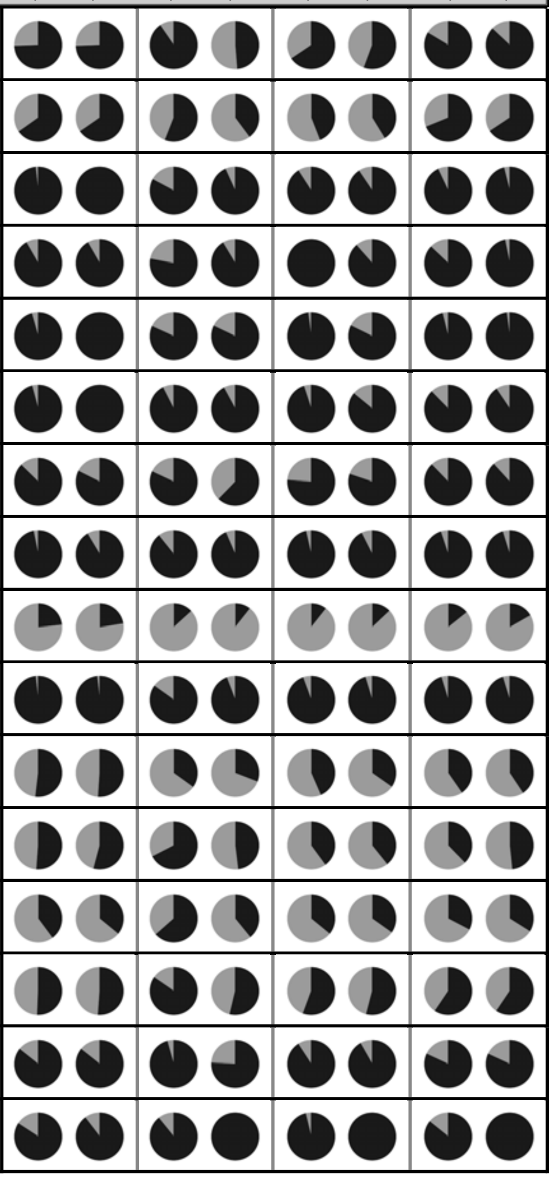

Show detected proteins only
Show all proteins
Filter by category:
GO: amino acid transport

Proteins found: 1424

Enter (or paste) list of ORFs

Find ORFs

Dot Plots Dot Plots

ORF	*Fn* n	*Fn* coverage	*Fn* prob	*FnPg* n	*FnPg* coverage	*FnPg* prob	*FnSg* n	*FnSg* coverage	*FnSg* prob	*FnPgSg* n	*FnPgSg* coverage	*FnPgSg* prob	Description
FN1432	14	48.0%	1.000	2	7.6%	0.744	2	9.4%	1.000	9	32.4%	1.000	AAL95625.1| Leucine-, isoleucine-, valine-, threonine-, and alanine-binding protein
	12	46.7%	1.000	2	8.9%	0.996	4	20.6%	1.000	10	34.5%	1.000	
FN1433	19	63.8%	1.000	20	66.9%	1.000	20	63.8%	1.000	23	75.8%	1.000	AAL95626.1| Short chain dehydrogenase
	22	73.8%	1.000	22	66.5%	1.000	23	73.1%	1.000	21	75.8%	1.000	
FN1434	31	43.4%	1.000	19	25.3%	1.000	32	51.1%	1.000	26	39.0%	1.000	AAL95627.1| Tetratricopeptide repeat family protein
	27	38.1%	1.000	30	43.2%	1.000	30	40.3%	1.000	25	36.8%	1.000	
FN1437	8	67.1%	1.000	6	45.9%	1.000	4	45.9%	1.000	9	67.1%	1.000	AAL95630.1| LSU ribosomal protein L28P
	8	56.5%	1.000	6	67.1%	1.000	7	56.5%	1.000	8	67.1%	1.000	
FN1439	12	39.6%	1.000	1	4.5%	0.525	4	18.4%	1.000	10	35.9%	1.000	AAL95632.1| Transcriptional regulator, DeoR family
	13	40.0%	1.000	7	32.2%	1.000	5	19.2%	1.000	8	27.8%	1.000	
FN1440	19	39.5%	1.000	8	18.8%	1.000	6	17.8%	1.000	17	44.6%	1.000	AAL95633.1| 1-phosphofructokinase
	17	36.3%	1.000	15	36.6%	1.000	10	24.5%	1.000	12	37.6%	1.000	
FN1441	24	25.6%	1.000	9	16.1%	1.000	17	28.0%	1.000	22	26.1%	1.000	AAL95634.1| PTS system, fructose-specific IIABC component
	22	25.6%	1.000	18	28.0%	1.000	17	24.4%	1.000	20	25.1%	1.000	
FN1444	36	68.0%	1.000	29	54.7%	1.000	33	63.9%	1.000	35	64.3%	1.000	AAL95637.1| GMP synthase (glutamine-hydrolyzing)
	34	69.3%	1.000	39	67.6%	1.000	41	69.1%	1.000	29	63.3%	1.000	
FN1445	13	19.9%	1.000	8	13.5%	1.000	8	16.8%	1.000	13	18.7%	1.000	AAL95638.1| DNA helicase
	16	23.3%	1.000	7	11.0%	1.000	7	13.8%	1.000	10	17.6%	1.000	
FN1448	1	3.8%	0.242	2	6.2%	0.979	0	0.0%	0.000	0	0.0%	0.000	AAL95641.1| Hypothetical cytosolic protein
	1	2.1%	0.254	1	4.1%	0.326	3	10.3%	0.998	1	4.1%	0.312	
FN1449	84	32.2%	1.000	20	10.7%	1.000	14	8.4%	1.000	50	24.4%	1.000	AAL95642.1| Fusobacterium outer membrane protein family
	79	36.5%	1.000	25	13.5%	1.000	15	9.0%	1.000	64	27.9%	1.000	
FN1450	4	9.6%	1.000	2	3.0%	1.000	6	13.2%	1.000	5	12.0%	1.000	AAL95643.1| Integral membrane protein
	4	9.6%	1.000	5	7.7%	1.000	1	3.0%	0.985	4	9.6%	1.000	
FN1451	20	57.5%	1.000	16	48.1%	1.000	30	73.9%	1.000	19	53.3%	1.000	AAL95644.1| Cell division protein ftsZ
	22	58.9%	1.000	27	73.1%	1.000	30	69.4%	1.000	17	39.2%	1.000	
FN1452	18	38.0%	1.000	8	25.1%	1.000	16	39.8%	1.000	15	35.3%	1.000	AAL95645.1| Cell division protein ftsA
	14	35.1%	1.000	18	46.3%	1.000	19	38.0%	1.000	16	33.1%	1.000	
FN1454	9	38.3%	1.000	1	9.4%	0.984	4	22.0%	1.000	9	40.4%	1.000	AAL95647.1| D-alanine--D-alanine ligase
	7	33.4%	1.000	10	35.2%	1.000	12	45.6%	1.000	9	39.0%	1.000	
FN1455	5	16.4%	1.000	7	13.9%	1.000	6	17.4%	1.000	3	7.5%	1.000	AAL95648.1| UDP-N-acetylenolpyruvoylglucosamine reductase
	6	16.4%	1.000	7	27.4%	1.000	7	23.8%	1.000	8	25.3%	1.000	

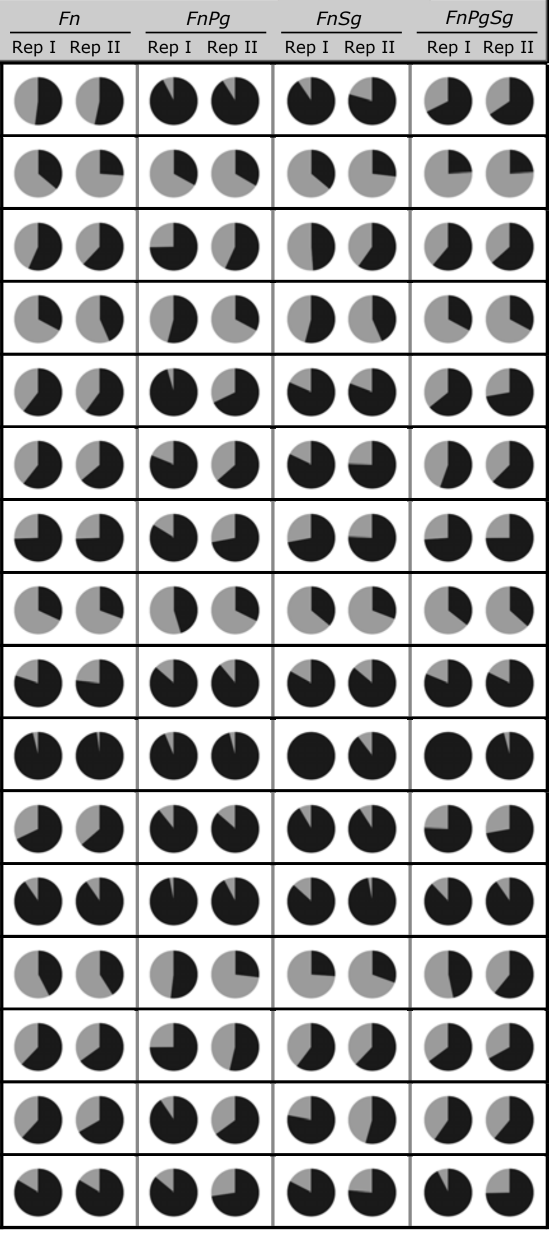

ORF	*Fn* n	*Fn* coverage	*Fn* prob	*FnPg* n	*FnPg* coverage	*FnPg* prob	*FnSg* n	*FnSg* coverage	*FnSg* prob	*FnPgSg* n	*FnPgSg* coverage	*FnPgSg* prob	Description
**FN1456**	16	34.0%	1.000	14	26.1%	1.000	15	32.5%	1.000	13	30.1%	1.000	AAL95649.1| UDP-N-acetylmuramate--alanine ligase
	16	36.3%	1.000	20	49.4%	1.000	20	42.3%	1.000	16	30.3%	1.000	
**FN1457**	9	30.0%	1.000	4	19.3%	1.000	16	41.7%	1.000	10	30.3%	1.000	AAL95650.1| UDP-N-acetylglucosamine-N-acetylmuramyl-Pentapeptide [illegible]
	10	34.2%	1.000	14	40.9%	1.000	12	34.7%	1.000	8	26.6%	1.000	
**FN1458**	18	41.4%	1.000	16	30.2%	1.000	16	38.3%	1.000	22	43.8%	1.000	AAL95651.1| UDP-N-acetylmuramoylalanine--D-glutamate ligase
	17	35.7%	1.000	13	34.4%	1.000	19	43.4%	1.000	14	33.5%	1.000	
**FN1459**	2	4.4%	1.000	1	4.4%	0.984	2	12.5%	0.989	0	0.0%	0.000	AAL95652.1| Phospho-N-acetylmuramoyl-pentapeptide-transferase
	3	9.1%	1.000	1	1.9%	0.451	2	9.1%	0.988	1	4.7%	0.737	
**FN1461**	14	27.2%	1.000	7	18.8%	1.000	5	11.9%	1.000	9	19.2%	1.000	AAL95654.1| Histidinol-phosphatase
	16	29.0%	1.000	9	22.5%	1.000	13	27.7%	1.000	7	16.0%	1.000	
**FN1462**	1	2.1%	0.580	0	0.0%	0.000	3	14.5%	0.712	0	0.0%		AAL95655.1| Transcriptional regulator, GntR family
	2	6.4%	0.508	2	8.7%	0.534	1	3.4%	0.572	0	0.0%		
**FN1463**	25	60.7%	1.000	19	51.1%	1.000	22	54.3%	1.000	21	55.0%	1.000	AAL95656.1| pyridoxine biosynthesis protein
	24	60.7%	1.000	20	57.5%	1.000	22	57.9%	1.000	23	55.0%	1.000	
**FN1464**	31	43.6%	1.000	19	31.6%	1.000	34	54.7%	1.000	27	45.1%	1.000	AAL95657.1| 1-deoxyxylulose-5-phosphate synthase
	30	39.6%	1.000	37	55.1%	1.000	36	60.9%	1.000	27	41.3%	1.000	
**FN1470**	20	54.3%	1.000	6	34.1%	1.000	3	12.1%	0.931	20	51.3%	1.000	AAL95663.1| Hypothetical protein
	17	44.4%	1.000	6	18.5%	1.000	6	19.9%	1.000	17	52.2%	1.000	
**FN1471**	3	8.7%	1.000	1	7.8%	0.513	3	11.4%	0.717	3	8.7%	0.992	AAL95664.1| LACI-family transcription regulator
	5	16.8%	1.000	1	2.7%	0.298	3	9.9%	0.993	0	0.0%	0.000	
**FN1472**	24	57.2%	1.000	4	20.5%	1.000	8	33.6%	1.000	17	45.3%	1.000	AAL95665.1| N-acetylneuraminate-binding protein
	21	56.9%	1.000	14	39.1%	1.000	7	33.6%	1.000	20	48.0%	1.000	
**FN1474**	3	15.1%	1.000	0	0.0%	0.000	0	0.0%	0.000	1	6.5%	0.969	AAL95667.1| N-acetylmannosamine kinase
	3	18.6%	1.000	2	10.0%	0.694	1	5.2%	0.360	0	0.0%	0.000	
**FN1475**	6	30.3%	1.000	3	12.8%	1.000	2	9.0%	0.782	9	44.8%	1.000	AAL95668.1| N-acetylneuraminate lyase
	8	48.6%	1.000	3	16.6%	1.000	3	16.6%	1.000	9	43.4%	1.000	
**FN1476**	6	34.8%	1.000	1	9.4%	0.975	1	5.4%	0.616	5	30.4%	1.000	AAL95669.1| N-acetylmannosamine-6-phosphate 2-epimerase
	7	55.4%	1.000	1	5.4%	0.255	0	0.0%	0.000	6	46.0%	1.000	
**FN1478**	1	21.6%	0.981	0	0.0%	0.000	3	23.4%	1.000	2	21.6%	1.000	AAL95671.1| Hypothetical protein
	2	23.4%	0.999	3	23.4%	1.000	2	23.4%	1.000	3	23.4%	1.000	
**FN1479**	5	19.1%	1.000	5	18.1%	1.000	4	17.6%	1.000	8	40.4%	1.000	AAL95672.1| Hypothetical protein
	6	23.9%	1.000	8	41.0%	1.000	7	31.9%	1.000	6	33.0%	1.000	

Fn Rep I Rep II | *FnPg* Rep I Rep II | *FnSg* Rep I Rep II | *FnPgSg* Rep I Rep II

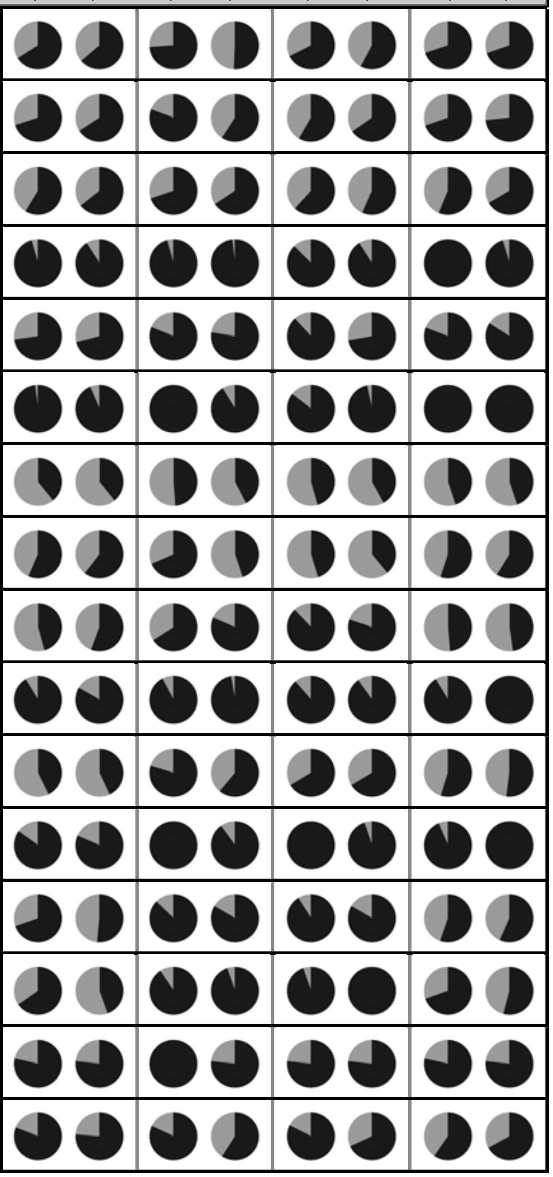

ORF	*Fn* n	*Fn* coverage	*Fn* prob	*FnPg* n	*FnPg* coverage	*FnPg* prob	*FnSg* n	*FnSg* coverage	*FnSg* prob	*FnPgSg* n	*FnPgSg* coverage	*FnPgSg* prob	Description
**FN1480**	6	19.8%	1.000	3	9.8%	1.000	11	26.7%	1.000	5	12.9%	1.000	AAL95673.1| MG2+ transporter MGTE
	5	16.7%	1.000	11	22.9%	1.000	14	32.7%	1.000	3	9.8%	0.996	
**FN1481**	4	16.1%	1.000	4	18.8%	1.000	3	11.5%	1.000	3	9.7%	0.882	AAL95674.1| Queuine tRNA-ribosyltransferase
	3	16.1%	0.995	5	27.9%	1.000	5	21.4%	1.000	1	7.0%	0.512	
**FN1482**	21	30.2%	1.000	11	16.3%	1.000	25	39.0%	1.000	9	13.7%	1.000	AAL95675.1| Guanosine-3',5'-bis (Diphosphate) 3'-pyrophosphohydrolase
	19	23.6%	1.000	27	36.0%	1.000	35	47.9%	1.000	11	14.2%	1.000	
**FN1483**	6	32.9%	1.000	6	38.2%	1.000	7	58.2%	1.000	10	51.8%	1.000	AAL95676.1| Adenine phosphoribosyltransferase
	5	32.9%	1.000	8	48.2%	1.000	8	49.4%	1.000	8	51.8%	1.000	
**FN1484**	5	22.4%	1.000	1	4.9%	0.981	3	11.8%	1.000	3	15.1%	1.000	AAL95677.1| Tetratricopeptide repeat family protein
	7	26.9%	1.000	2	8.2%	0.996	3	13.5%	0.999	3	11.8%	1.000	
**FN1485**	0	0.0%		2	18.8%	0.985	1	6.7%	0.952	2	9.9%	0.750	AAL95678.1| Transporter
	0	0.0%		1	5.8%	0.950	3	22.9%	1.000	1	6.7%	0.418	
**FN1486**	3	7.3%	1.000	1	3.8%	0.615	1	3.8%	0.817	5	16.9%	1.000	AAL95679.1| magnesium and cobalt efflux protein CorC
	5	17.4%	1.000	1	1.6%	0.822	5	12.7%	1.000	3	11.0%	0.965	
**FN1487**	18	56.2%	1.000	10	43.1%	1.000	16	51.0%	1.000	14	53.6%	1.000	AAL95681.1| Chorismate mutase
	18	56.2%	1.000	18	62.7%	1.000	13	52.9%	1.000	11	53.6%	1.000	
**FN1488**	1	9.9%	0.981	3	31.1%	1.000	2	18.7%	0.994	4	32.2%	1.000	AAL95682.1| Methylenetetrahydrofolate dehydrogenase (NADP+)
	1	9.9%	0.982	3	27.2%	1.000	3	23.0%	1.000	5	40.3%	1.000	
**FN1489**	12	25.6%	1.000	6	21.8%	1.000	10	34.7%	1.000	4	14.2%	1.000	AAL95683.1| Methionyl-tRNA formyltransferase
	12	32.5%	1.000	12	40.1%	1.000	11	31.2%	1.000	8	20.8%	1.000	
**FN1490**	4	30.9%	1.000	3	15.4%	1.000	8	51.7%	1.000	3	23.5%	1.000	AAL95684.1| putative regulatory protein
	4	22.1%	1.000	7	51.7%	1.000	6	35.6%	1.000	2	13.4%	0.993	
**FN1491**	2	17.3%	0.999	2	13.0%	0.997	3	22.2%	1.000	2	12.3%	0.997	AAL95685.1| PTS system, IIA component
	3	22.2%	1.000	3	22.2%	1.000	3	22.2%	1.000	2	13.6%	0.989	
**FN1492**	1	6.0%	0.507	0	0.0%		0	0.0%	0.000	1	3.0%	0.341	AAL95686.1| DNA repair protein recO
	0	0.0%	0.000	0	0.0%		4	27.0%	0.952	0	0.0%	0.000	
**FN1493**	7	26.0%	1.000	4	21.4%	0.999	3	20.8%	1.000	2	10.9%	0.996	AAL95687.1| Hypothetical protein
	6	26.0%	1.000	5	20.3%	1.000	3	10.9%	1.000	1	4.7%	0.985	
**FN1494**	9	58.1%	0.000	0	0.0%	0.000	5	21.0%	0.000	7	52.4%	0.000	AAL95680.1| Rod shape-determining protein mreC
	7	46.7%	0.000	6	41.0%	0.000	6	26.2%	0.000	5	35.2%	0.000	
**FN1496**	9	58.1%	1.000	0	0.0%	0.000	5	21.0%	1.000	7	52.4%	1.000	AAL95680.1| Rod shape-determining protein mreC
	7	46.7%	1.000	6	41.0%	1.000	6	26.2%	1.000	5	35.2%	1.000	

Fn Rep I Rep II | *FnPg* Rep I Rep II | *FnSg* Rep I Rep II | *FnPgSg* Rep I Rep II

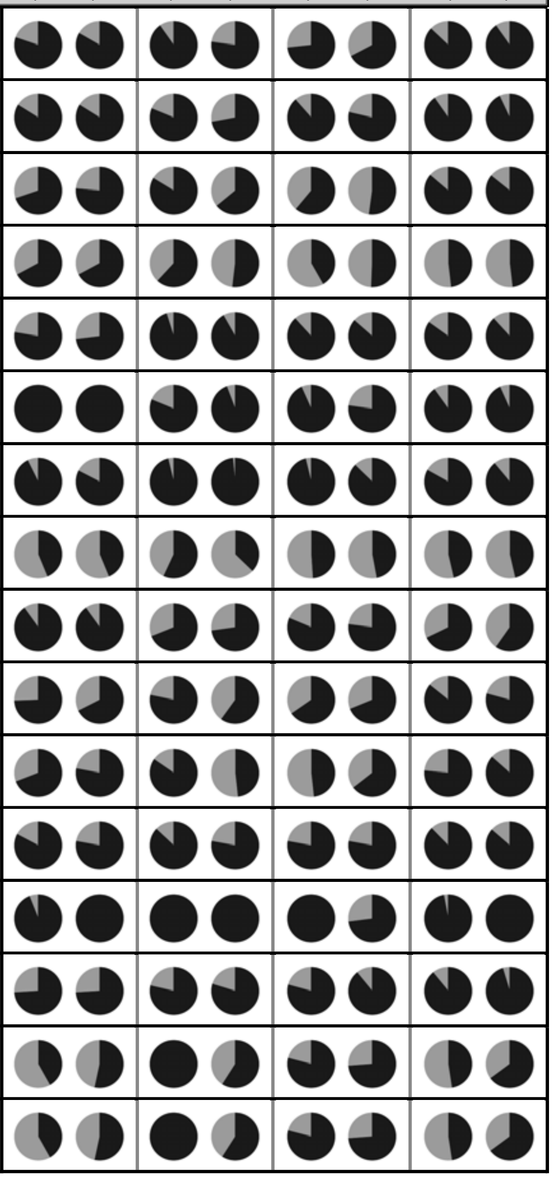

Show detected proteins only
Show all proteins
Filter by category:
GO: amino acid transport

Proteins found: 1424

Enter (or paste) list of ORFs

Find ORFs

Dot Plots Dot Plots

ORF	*Fn* n	*Fn* coverage	*Fn* prob	*FnPg* n	*FnPg* coverage	*FnPg* prob	*FnSg* n	*FnSg* coverage	*FnSg* prob	*FnPgSg* n	*FnPgSg* coverage	*FnPgSg* prob	Description
FN1499	11	25.3%	1.000	4	19.2%	1.000	7	21.3%	1.000	18	34.0%	1.000	AAL93625.1| Cell surface protein
	9	20.0%	1.000	20	44.1%	1.000	6	17.3%	1.000	22	36.7%	1.000	
FN1501	1	10.8%	0.399	2	10.8%	0.819	0	0.0%	0.000	0	0.0%	0.000	AAL93627.1| Nickel transport ATP-binding protein nikD
	1	5.4%	0.324	0	0.0%	0.000	1	6.2%	0.393	3	8.5%	0.897	
FN1504	30	46.6%	1.000	17	37.4%	1.000	26	60.7%	1.000	29	54.0%	1.000	AAL93630.1| Nickel-binding protein
	26	46.4%	1.000	24	50.2%	1.000	25	51.9%	1.000	22	46.7%	1.000	
FN1505	14	78.3%	1.000	10	68.8%	1.000	15	74.5%	1.000	14	73.9%	1.000	AAL93631.1| 6,7-dimethyl-8-ribityllumazine synthase
	13	73.9%	1.000	13	78.3%	1.000	13	73.9%	1.000	12	73.9%	1.000	
FN1506	5	18.7%	1.000	5	13.8%	1.000	1	3.8%	0.451	8	26.8%	1.000	AAL93632.1| Diaminohydroxyphosphoribosylaminopyrimidine deaminase
	2	7.3%	0.805	4	17.6%	1.000	6	19.8%	1.000	6	16.0%	1.000	
FN1507	2	13.5%	0.990	1	6.0%	0.416	2	10.0%	0.801	1	5.6%	0.702	AAL93633.1| Riboflavin synthase alpha chain
	1	6.0%	0.975	2	12.0%	0.985	4	23.1%	1.000	0	0.0%	0.000	
FN1508	17	45.1%	1.000	13	35.1%	1.000	18	51.1%	1.000	23	48.4%	1.000	AAL93634.1| GTP cyclohydrolase II
	16	47.1%	1.000	23	60.7%	1.000	22	57.4%	1.000	22	54.6%	1.000	
FN1512	7	16.7%	1.000	0	0.0%	0.000	0	0.0%		3	9.8%	1.000	AAL93638.1| hypothetical exported 24-amino acid repeat protein
	6	18.7%	1.000	1	3.0%	0.275	0	0.0%		3	9.1%	0.981	
FN1517	45	51.5%	1.000	19	25.1%	1.000	30	46.3%	1.000	40	47.5%	1.000	AAL93643.1| Leucyl-tRNA synthetase
	43	50.4%	1.000	48	59.8%	1.000	38	53.4%	1.000	37	49.1%	1.000	
FN1518	4	25.5%	1.000	1	6.4%	0.896	1	4.4%	0.966	2	12.7%	0.999	AAL93644.1| RNA polymerase sigma-H factor
	3	21.6%	1.000	1	4.4%	0.527	3	21.1%	1.000	1	3.9%	0.405	
FN1519	5	24.8%	1.000	2	6.0%	0.991	4	25.2%	1.000	5	24.4%	1.000	AAL93645.1| 23S rRNA methyltransferase
	4	24.4%	1.000	4	20.5%	1.000	5	24.8%	1.000	5	26.5%	1.000	
FN1520	14	43.7%	1.000	5	15.6%	1.000	10	34.0%	1.000	14	40.2%	1.000	AAL93646.1| UDP-N-acetylglucosamine 1-carboxyvinyltransferase
	17	44.9%	1.000	14	41.6%	1.000	12	36.2%	1.000	14	38.3%	1.000	
FN1521	6	13.8%	1.000	0	0.0%	0.000	0	0.0%		0	0.0%	0.000	AAL93647.1| Dipeptide transport system permease protein dppB
	6	13.8%	1.000	0	0.0%	0.000	0	0.0%		2	4.8%	0.992	
FN1523	34	47.3%	1.000	13	24.7%	1.000	21	38.6%	1.000	35	53.8%	1.000	AAL93649.1| Dipeptide-binding protein
	34	47.3%	1.000	24	45.4%	1.000	24	45.6%	1.000	35	54.0%	1.000	
FN1524	3	11.1%	1.000	0	0.0%		0	0.0%	0.000	0	0.0%		AAL93650.1| Dipeptide transport ATP-binding protein dppD
	2	6.9%	0.988	0	0.0%		1	12.6%	0.386	0	0.0%		
FN1525	4	18.4%	1.000	2	7.9%	0.880	0	0.0%		1	3.2%	0.816	AAL93651.1| Dipeptide transport ATP-binding protein dppF
	2	6.7%	0.996	0	0.0%	0.000	0	0.0%		4	9.4%	1.000	

Show detected proteins only
Show all proteins
Filter by category:
GO: amino acid transport

Proteins found: 1424

Enter (or paste) list of ORFs

Find ORFs

Dot Plots Dot Plots

ORF	*Fn* n	*Fn* coverage	*Fn* prob	*FnPg* n	*FnPg* coverage	*FnPg* prob	*FnSg* n	*FnSg* coverage	*FnSg* prob	*FnPgSg* n	*FnPgSg* coverage	*FnPgSg* prob	Description
**FN1526**	107	52.0%	1.000	67	41.2%	1.000	65	34.3%	1.000	121	59.4%	1.000	AAL93652.1| Fusobacterium outer membrane protein family
	101	50.5%	1.000	93	45.9%	1.000	74	43.0%	1.000	105	54.8%	1.000	
**FN1527**	12	46.5%	1.000	6	39.5%	1.000	6	48.1%	1.000	13	65.1%	1.000	AAL93653.1| Hypothetical protein
	13	46.5%	1.000	10	48.1%	1.000	9	48.8%	1.000	12	45.7%	1.000	
**FN1528**	21	67.8%	1.000	12	67.8%	1.000	7	34.7%	1.000	21	56.8%	1.000	AAL93654.1| Hypothetical protein
	21	67.8%	1.000	10	47.5%	1.000	11	50.0%	1.000	20	73.7%	1.000	
**FN1529**	13	45.5%	1.000	5	32.5%	1.000	6	39.0%	1.000	11	39.0%	1.000	AAL93655.1| Hypothetical protein
	10	39.0%	1.000	7	43.1%	1.000	3	23.6%	1.000	11	39.0%	1.000	
**FN1531**	3	11.5%	1.000	4	16.8%	1.000	4	23.0%	1.000	4	11.5%	1.000	AAL93657.1| murein hydrolase export regulator
	3	11.5%	0.998	5	17.6%	1.000	4	23.0%	1.000	3	11.5%	1.000	
**FN1533**	25	59.8%	1.000	30	70.3%	1.000	19	59.8%	1.000	28	69.3%	1.000	AAL93659.1| Electron transfer flavoprotein alpha-subunit
	24	59.8%	1.000	21	57.9%	1.000	20	59.8%	1.000	28	62.5%	1.000	
**FN1534**	22	58.7%	1.000	24	72.6%	1.000	18	79.9%	1.000	20	62.9%	1.000	AAL93660.1| Electron transfer flavoprotein beta-subunit
	22	58.7%	1.000	19	66.4%	1.000	17	75.7%	1.000	20	62.9%	1.000	
**FN1535**	39	61.6%	1.000	36	63.5%	1.000	42	85.4%	1.000	43	62.7%	1.000	AAL93661.1| Acyl-CoA dehydrogenase, short-chain specific
	43	63.0%	1.000	49	77.0%	1.000	40	70.6%	1.000	45	61.4%	1.000	
**FN1536**	29	56.0%	1.000	34	55.8%	1.000	34	69.1%	1.000	29	60.0%	1.000	AAL93662.1| (S)-2-hydroxy-acid oxidase chain D
	30	54.5%	1.000	34	66.3%	1.000	31	67.4%	1.000	32	56.8%	1.000	
**FN1537**	4	13.7%	1.000	4	10.6%	1.000	0	0.0%	0.000	2	9.3%	0.971	AAL93663.1| Arsenical pump-driving ATPase
	4	8.5%	1.000	1	3.1%	0.980	5	16.2%	1.000	2	6.7%	0.873	
**FN1538**	6	22.2%	1.000	5	14.6%	1.000	10	34.3%	1.000	5	20.7%	1.000	AAL93664.1| Arsenical pump-driving ATPase
	9	29.0%	1.000	9	31.6%	1.000	9	30.1%	1.000	2	6.1%	0.999	
**FN1539**	15	68.9%	1.000	18	77.0%	1.000	18	94.5%	1.000	16	80.3%	1.000	AAL93665.1| Iron-sulfur cluster-binding protein
	15	66.7%	1.000	17	90.7%	1.000	16	90.7%	1.000	18	80.3%	1.000	
**FN1540**	36	44.8%	1.000	39	44.8%	1.000	45	53.7%	1.000	32	49.4%	1.000	AAL93666.1| Iron-sulfur cluster-binding protein
	34	38.4%	1.000	50	61.8%	1.000	50	60.5%	1.000	31	46.9%	1.000	
**FN1544**	20	36.4%	1.000	26	51.5%	1.000	32	57.3%	1.000	26	68.2%	1.000	AAL93670.1| Probable electron transfer flavoprotein-quinone oxidoreductase ydiS
	20	42.5%	1.000	33	54.1%	1.000	31	59.4%	1.000	20	53.4%	1.000	
**FN1545**	6	70.2%	1.000	11	61.7%	1.000	8	70.2%	1.000	5	70.2%	1.000	AAL93671.1| Ferredoxin like protein
	6	70.2%	1.000	6	70.2%	1.000	5	58.5%	1.000	7	69.1%	1.000	
**FN1546**	71	67.0%	1.000	66	58.1%	1.000	59	65.5%	1.000	79	67.0%	1.000	AAL93672.1| Protein Translation Elongation Factor G (EF-G)
	72	67.4%	1.000	71	69.1%	1.000	63	66.7%	1.000	78	69.1%	1.000	

Fn Rep I Rep II | *FnPg* Rep I Rep II | *FnSg* Rep I Rep II | *FnPgSg* Rep I Rep II

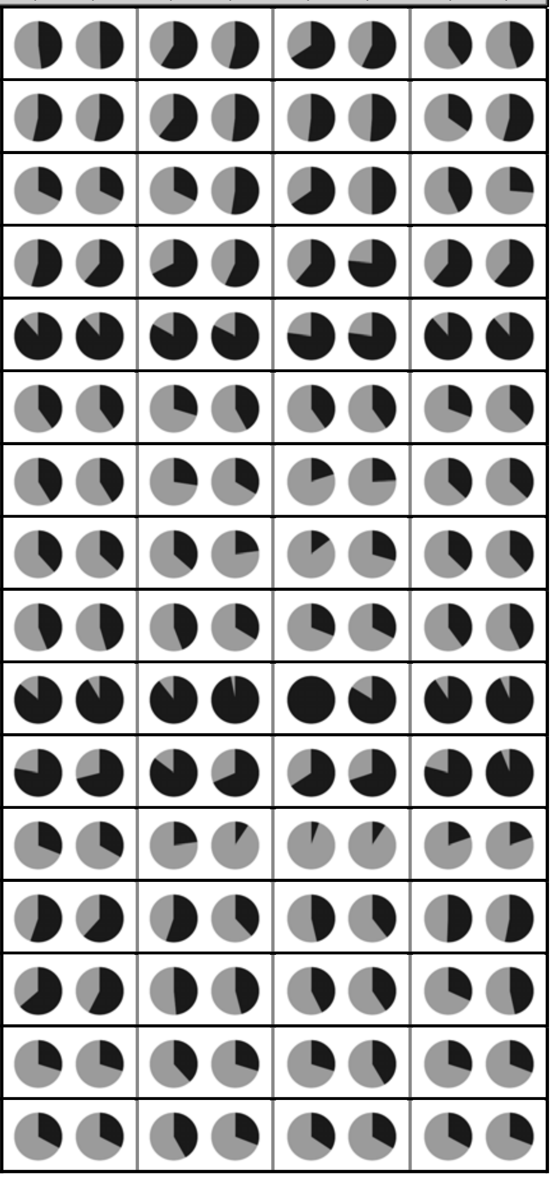

Show detected proteins only
Show all proteins
Filter by category:
GO: amino acid transport

Proteins found:
1424

Enter (or paste) list of ORFs
Find ORFs

Dot Plots Dot Plots

ORF	Fn n	Fn coverage	Fn prob	FnPg n	FnPg coverage	FnPg prob	FnSg n	FnSg coverage	FnSg prob	FnPgSg n	FnPgSg coverage	FnPgSg prob	Description
FN1547	9	17.0%	1.000	11	21.1%	1.000	12	20.9%	1.000	14	23.3%	1.000	AAL93673.1| PTS permease for N-acetylglucosamine and glucose
	11	18.4%	1.000	13	24.7%	1.000	14	26.4%	1.000	11	19.4%	1.000	
FN1548	7	21.7%	1.000	3	29.0%	1.000	5	37.7%	1.000	6	17.4%	1.000	AAL93674.1| Hypothetical protein
	7	21.7%	1.000	5	34.1%	1.000	5	29.0%	1.000	6	17.4%	1.000	
FN1549	25	59.9%	1.000	21	56.1%	1.000	22	63.6%	1.000	24	60.9%	1.000	AAL93675.1| Stomatin like protein
	26	61.2%	1.000	25	71.1%	1.000	20	59.2%	1.000	21	53.4%	1.000	
FN1552	3	8.0%	1.000	0	0.0%	0.000	2	7.6%	0.990	0	0.0%	0.000	AAL93678.1| abortive phage resistance protein
	5	13.7%	1.000	3	10.5%	0.999	3	11.5%	1.000	2	7.3%	0.996	
FN1553	6	11.9%	1.000	6	17.7%	1.000	5	15.8%	1.000	10	25.8%	1.000	AAL93679.1| abortive phage resistance protein
	7	13.0%	1.000	4	6.5%	1.000	10	29.2%	1.000	3	6.9%	1.000	
FN1554	55	35.7%	1.000	26	17.6%	1.000	26	25.9%	1.000	42	32.9%	1.000	AAL93680.1| Fusobacterium outer membrane protein family
	54	37.9%	1.000	36	31.4%	1.000	32	30.5%	1.000	42	33.2%	1.000	
FN1555	53	86.8%	1.000	54	84.5%	1.000	45	83.5%	1.000	56	75.4%	1.000	AAL93681.1| Protein Translation Elongation Factor Tu
	56	76.6%	1.000	50	88.8%	1.000	48	87.6%	1.000	56	76.6%	1.000	
FN1556	65	67.0%	1.000	51	64.5%	1.000	48	64.8%	1.000	79	73.7%	1.000	AAL93682.1| Protein Translation Elongation Factor G (EF-G)
	64	66.8%	1.000	58	68.5%	1.000	53	66.7%	1.000	76	75.2%	1.000	
FN1557	15	57.1%	1.000	15	57.1%	1.000	18	71.8%	1.000	27	62.8%	1.000	AAL93683.1| SSU ribosomal protein S7P
	16	57.1%	1.000	21	78.2%	1.000	15	67.3%	1.000	26	66.7%	1.000	
FN1558	2	22.1%	1.000	3	23.8%	1.000	2	31.1%	0.997	2	22.1%	1.000	AAL93684.1| SSU ribosomal protein S12P
	2	22.1%	1.000	3	37.7%	1.000	2	31.1%	0.997	4	39.3%	1.000	
FN1560	12	58.7%	1.000	6	34.7%	1.000	16	67.1%	1.000	12	57.5%	1.000	AAL93686.1| unknown
	16	60.5%	1.000	18	77.2%	1.000	17	76.0%	1.000	12	55.1%	1.000	
FN1562	5	12.3%	1.000	3	7.8%	1.000	6	25.4%	1.000	4	12.0%	1.000	AAL93688.1| Phospho-2-dehydro -3-deoxyheptonate aldolase
	5	16.8%	1.000	7	29.9%	1.000	7	34.1%	1.000	1	2.4%	0.629	
FN1576	0	0.0%	0.000	0	0.0%	0.000	3	19.7%	0.995	1	3.1%	0.503	AAL93691.1| ATPases and helicase subunits involved in DNA replication
	1	5.5%	0.951	1	3.8%	0.335	1	7.6%	0.318	0	0.0%	0.000	
FN1577	19	51.2%	1.000	12	41.2%	1.000	25	69.0%	1.000	19	56.7%	1.000	AAL93692.1| Rod shape-determining protein mreB
	18	52.0%	1.000	27	62.3%	1.000	24	61.4%	1.000	17	50.9%	1.000	
FN1579	21	39.1%	1.000	10	25.6%	1.000	20	44.0%	1.000	22	55.2%	1.000	AAL93694.1| Cysteinyl-tRNA synthetase
	19	41.0%	1.000	22	47.1%	1.000	21	45.0%	1.000	20	39.1%	1.000	
FN1580	4	19.9%	0.998	0	0.0%		1	5.6%	0.408	1	3.9%	0.396	AAL93695.1| 2-C-methyl-D-erythritol 4-phosphate cytidylyltransferase
	2	8.2%	0.986	0	0.0%		3	18.2%	0.996	1	4.8%	0.469	

Fn: Rep I, Rep II · FnPg: Rep I, Rep II · FnSg: Rep I, Rep II · FnPgSg: Rep I, Rep II

Show detected proteins only
Show all proteins
Filter by category:
GO: amino acid transport

Proteins found: 1424

Enter (or paste) list of ORFs

Find ORFs

Dot Plots Dot Plots

ORF	*Fn* n	*Fn* coverage	*Fn* prob	*FnPg* n	*FnPg* coverage	*FnPg* prob	*FnSg* n	*FnSg* coverage	*FnSg* prob	*FnPgSg* n	*FnPgSg* coverage	*FnPgSg* prob	Description
FN1581	13	21.2%	1.000	9	18.3%	1.000	18	28.0%	1.000	13	19.9%	1.000	AAL93696.1| DNA mismatch repair protein mutS
	13	22.1%	1.000	23	34.4%	1.000	25	38.9%	1.000	9	16.7%	1.000	
FN1582	1	2.4%	0.977	0	0.0%	0.000	5	11.2%	1.000	1	2.2%	0.445	AAL93697.1| Hypothetical protein
	3	9.6%	1.000	8	20.0%	1.000	6	12.9%	1.000	0	0.0%	0.000	
FN1586	0	0.0%		1	6.1%	0.754	4	8.3%	1.000	2	9.3%	0.998	AAL93701.1| O-succinylbenzoate-CoA synthase
	0	0.0%		10	27.7%	1.000	5	15.7%	1.000	0	0.0%	0.000	
FN1589	10	38.4%	1.000	3	18.3%	1.000	9	45.2%	1.000	10	49.3%	1.000	AAL93704.1| LexA repressor
	10	48.9%	1.000	7	40.6%	1.000	11	66.2%	1.000	5	26.0%	1.000	
FN1590	0	0.0%	0.000	0	0.0%	0.000	3	43.0%	1.000	1	27.8%	0.807	AAL93705.1| Hypothetical lipoprotein
	0	0.0%	0.000	4	40.1%	1.000	3	37.7%	1.000	1	18.1%	0.291	
FN1591	23	53.0%	1.000	17	44.4%	1.000	18	53.5%	1.000	28	56.9%	1.000	AAL93706.1| RNFB-related protein
	21	47.0%	1.000	15	46.2%	1.000	12	41.0%	1.000	28	56.9%	1.000	
FN1592	1	6.2%	0.981	0	0.0%	0.000	2	18.6%	0.933	1	6.2%	0.989	AAL93707.1| Na(+)-translocating NADH-quinone reductase subunit D
	1	6.2%	0.982	4	25.8%	1.000	3	24.7%	0.997	3	22.2%	0.999	
FN1594	9	30.5%	1.000	7	23.7%	1.000	5	24.3%	1.000	13	35.0%	1.000	AAL93709.1| Nitrogen fixation protein RNFG
	10	30.5%	1.000	8	35.0%	1.000	4	39.5%	1.000	12	35.0%	1.000	
FN1595	4	11.8%	1.000	3	11.8%	1.000	5	26.8%	1.000	4	11.8%	1.000	AAL93710.1| Na(+)-translocating NADH-quinone reductase subunit B
	4	11.8%	1.000	4	11.8%	1.000	5	19.1%	1.000	5	19.1%	1.000	
FN1596	40	75.5%	1.000	34	73.9%	1.000	34	73.5%	1.000	40	72.6%	1.000	AAL93711.1| Nitrogen fixation iron-sulphur protein RNFC
	40	77.1%	1.000	35	73.5%	1.000	35	73.5%	1.000	43	75.7%	1.000	
FN1597	5	22.0%	1.000	7	39.8%	1.000	0	0.0%	0.000	4	18.8%	0.968	AAL93712.1| Peptidyl-tRNA hydrolase
	6	22.0%	1.000	0	0.0%	0.000	1	9.4%	0.985	1	6.3%	0.762	
FN1600	3	22.3%	0.991	1	8.5%	0.921	3	18.6%	0.995	0	0.0%	0.000	AAL93715.1| tRNA pseudouridine synthase A
	1	8.5%	0.982	3	16.2%	1.000	3	16.2%	1.000	2	11.3%	0.992	
FN1601	2	3.9%	0.915	2	3.9%	1.000	3	12.0%	1.000	2	4.2%	0.994	AAL93716.1| Hypothetical cytosolic protein
	4	11.7%	1.000	2	7.8%	0.896	5	15.6%	1.000	0	0.0%	0.000	
FN1602	0	0.0%	0.000	0	0.0%	0.000	0	0.0%	0.000	2	12.8%	0.959	AAL93717.1| Hypothetical cytosolic protein
	1	7.7%	0.970	1	6.4%	0.534	2	21.8%	0.613	3	22.4%	0.999	
FN1603	11	21.2%	1.000	3	8.8%	1.000	3	5.3%	0.980	4	5.1%	1.000	AAL93718.1| 2',3'-cyclic nucleotide 3'-phosphodiesterase
	7	12.0%	1.000	1	1.5%	0.361	3	5.3%	0.998	5	9.5%	0.997	
FN1605	28	56.2%	1.000	25	44.7%	1.000	30	60.5%	1.000	30	58.1%	1.000	AAL93720.1| Adenylosuccinate synthetase
	28	56.2%	1.000	38	68.9%	1.000	30	60.9%	1.000	28	58.1%	1.000	

Columns *Fn*, *FnPg*, *FnSg*, *FnPgSg*: Rep I, Rep II

Show detected proteins only
Show all proteins
Filter by category:
GO: amino acid transport

Proteins found:
1424

Enter (or paste) list of ORFs

Find ORFs

Dot Plots Dot Plots

ORF	*Fn* n	*Fn* coverage	*Fn* prob	*FnPg* n	*FnPg* coverage	*FnPg* prob	*FnSg* n	*FnSg* coverage	*FnSg* prob	*FnPgSg* n	*FnPgSg* coverage	*FnPgSg* prob	Description
FN1606	9	11.1%	1.000	5	8.3%	1.000	9	13.1%	1.000	9	15.8%	1.000	AAL93721.1| 3-deoxy-D-manno-octulosonic-acid transferase
	12	15.5%	1.000	10	16.9%	1.000	12	16.1%	1.000	9	18.8%	1.000	
FN1607	6	29.8%	1.000	3	17.9%	0.997	5	26.6%	1.000	2	5.0%	0.997	AAL93722.1| Cytidylate kinase
	5	18.3%	1.000	7	33.0%	1.000	7	32.1%	1.000	4	18.3%	1.000	
FN1608	2	7.4%	0.988	0	0.0%	0.000	5	24.7%	1.000	2	10.9%	0.807	AAL93723.1| Ribosomal protein L11 methyltransferase
	0	0.0%	0.000	4	17.3%	1.000	4	16.7%	1.000	2	7.1%	0.673	
FN1609	10	28.5%	1.000	0	0.0%	0.000	3	9.9%	0.912	3	14.4%	0.977	AAL93724.1| Hypothetical protein
	7	23.2%	1.000	10	28.9%	1.000	3	8.7%	0.998	2	8.7%	0.997	
FN1610	9	43.9%	1.000	7	23.9%	1.000	9	38.6%	1.000	9	28.4%	1.000	AAL93725.1| 33 kDa chaperonin
	9	31.9%	1.000	11	43.2%	1.000	8	30.5%	1.000	8	34.4%	1.000	
FN1611	2	11.3%	0.994	1	7.5%	0.896	0	0.0%	0.000	1	5.7%	0.555	AAL93726.1| Competence protein
	3	13.2%	0.979	0	0.0%	0.000	1	4.4%	0.444	1	8.2%	0.340	
FN1613	7	27.2%	1.000	7	19.6%	1.000	6	31.3%	1.000	7	25.3%	1.000	AAL93728.1| Hypothetical protein
	8	23.4%	1.000	9	32.0%	1.000	9	33.2%	1.000	5	18.0%	1.000	
FN1614	13	23.9%	1.000	11	22.5%	1.000	7	13.5%	1.000	7	19.3%	1.000	AAL93729.1| MG(2+) chelatase family protein
	11	16.3%	1.000	11	22.9%	1.000	6	14.9%	1.000	9	12.5%	1.000	
FN1616	3	15.7%	1.000	1	10.5%	0.972	2	16.3%	1.000	6	26.1%	1.000	AAL93731.1| N utilization substance protein B
	3	15.7%	1.000	3	25.5%	1.000	3	25.5%	1.000	7	43.1%	1.000	
FN1618	4	14.0%	1.000	4	14.0%	1.000	8	31.3%	1.000	10	27.4%	1.000	AAL93733.1| Hypothetical protein
	5	27.4%	1.000	13	50.8%	1.000	12	31.8%	1.000	9	41.9%	1.000	
FN1619	6	56.6%	1.000	4	20.5%	1.000	6	56.6%	1.000	9	62.3%	1.000	AAL93734.1| Hypothetical cytosolic protein
	5	48.4%	1.000	6	56.6%	1.000	6	56.6%	1.000	10	70.5%	1.000	
FN1620	29	72.1%	1.000	21	59.1%	1.000	21	75.3%	1.000	31	70.4%	1.000	AAL93735.1| SSU ribosomal protein S2P
	28	69.2%	1.000	22	73.3%	1.000	20	65.2%	1.000	31	73.3%	1.000	
FN1621	42	67.3%	1.000	39	64.3%	1.000	32	70.0%	1.000	43	69.7%	1.000	AAL93736.1| Protein Translation Elongation Factor Ts
	41	71.0%	1.000	36	73.7%	1.000	33	70.0%	1.000	41	64.3%	1.000	
FN1622	18	62.8%	1.000	11	47.3%	1.000	9	36.4%	1.000	17	63.2%	1.000	AAL93737.1| Uridylate kinase
	20	58.6%	1.000	18	72.0%	1.000	12	51.0%	1.000	17	59.0%	1.000	
FN1623	12	52.6%	1.000	6	27.9%	1.000	8	47.9%	1.000	15	52.1%	1.000	AAL93738.1| Ribosome Recycling Factor (RRF)
	9	41.6%	1.000	11	47.9%	1.000	4	28.9%	1.000	11	52.1%	1.000	
FN1624	8	16.9%	1.000	2	5.9%	1.000	2	8.0%	0.999	6	16.9%	1.000	AAL93739.1| Protein translocase subunit secY
	7	13.6%	1.000	5	15.0%	1.000	4	11.3%	1.000	5	16.9%	1.000	

Show detected proteins only
Show all proteins
Filter by category:
GO: amino acid transport

Proteins found:
1424

Enter (or paste) list of ORFs

Find ORFs

Dot Plots Dot Plots

ORF	*Fn* n	*Fn* coverage	*Fn* prob	*FnPg* n	*FnPg* coverage	*FnPg* prob	*FnSg* n	*FnSg* coverage	*FnSg* prob	*FnPgSg* n	*FnPgSg* coverage	*FnPgSg* prob	Description
**FN1625**	15	32.1%	1.000	7	34.0%	1.000	7	25.8%	1.000	16	32.1%	1.000	AAL93740.1| LSU ribosomal protein L15P
	12	31.4%	1.000	9	37.7%	1.000	10	38.4%	1.000	15	49.7%	1.000	
**FN1626**	5	39.3%	1.000	1	23.0%	0.984	2	37.7%	0.988	7	39.3%	1.000	AAL93741.1| LSU ribosomal protein L30P
	5	39.3%	1.000	4	39.3%	1.000	4	39.3%	1.000	6	39.3%	1.000	
**FN1627**	8	47.6%	1.000	7	39.6%	1.000	11	61.0%	1.000	10	45.1%	1.000	AAL93742.1| SSU ribosomal protein S5P
	8	47.6%	1.000	10	58.5%	1.000	11	61.0%	1.000	12	52.4%	1.000	
**FN1628**	6	30.3%	1.000	5	30.3%	1.000	3	39.3%	1.000	8	48.4%	1.000	AAL93743.1| LSU ribosomal protein L18P
	6	30.3%	1.000	2	28.7%	1.000	1	21.3%	0.985	8	48.4%	1.000	
**FN1629**	22	65.0%	1.000	16	63.3%	1.000	12	55.4%	1.000	19	65.0%	1.000	AAL93744.1| LSU ribosomal protein L6P
	20	65.0%	1.000	17	72.9%	1.000	14	65.0%	1.000	20	65.0%	1.000	
**FN1630**	9	47.7%	1.000	5	28.8%	1.000	4	36.4%	1.000	7	43.2%	1.000	AAL93745.1| SSU ribosomal protein S8P
	9	47.7%	1.000	9	61.4%	1.000	6	43.2%	1.000	7	35.6%	1.000	
**FN1631**	3	28.4%	1.000	0	0.0%	0.000	3	28.4%	0.997	5	28.4%	1.000	AAL93746.1| SSU ribosomal protein S14P
	2	13.7%	0.999	3	15.8%	1.000	0	0.0%	0.000	5	28.4%	1.000	
**FN1632**	28	71.6%	1.000	13	43.7%	1.000	22	73.2%	1.000	24	69.9%	1.000	AAL93747.1| LSU ribosomal protein L5P
	26	71.6%	1.000	23	76.5%	1.000	18	70.5%	1.000	24	69.9%	1.000	
**FN1634**	6	66.7%	1.000	5	66.7%	1.000	3	66.7%	1.000	4	60.6%	1.000	AAL93749.1| LSU ribosomal protein L24P
	6	66.7%	1.000	2	60.6%	0.997	2	60.6%	0.997	4	60.6%	1.000	
**FN1635**	10	49.2%	1.000	3	35.2%	1.000	3	24.6%	1.000	8	43.4%	1.000	AAL93750.1| LSU ribosomal protein L14P
	9	43.4%	1.000	4	36.1%	1.000	4	36.1%	1.000	8	42.6%	1.000	
**FN1636**	7	57.8%	1.000	3	37.3%	1.000	10	60.2%	1.000	8	48.2%	1.000	AAL93751.1| SSU ribosomal protein S17P
	6	55.4%	1.000	9	60.2%	1.000	7	48.2%	1.000	6	48.2%	1.000	
**FN1637**	3	35.0%	1.000	1	21.7%	0.984	0	0.0%	0.000	6	68.3%	1.000	AAL93752.1| LSU ribosomal protein L29P
	4	46.7%	1.000	2	35.0%	0.988	1	21.7%	0.984	7	68.3%	1.000	
**FN1638**	7	35.0%	1.000	8	35.0%	1.000	6	35.0%	1.000	7	35.0%	1.000	AAL93753.1| LSU ribosomal protein L16P
	7	35.0%	1.000	8	45.5%	1.000	8	45.5%	1.000	7	35.0%	1.000	
**FN1639**	31	60.7%	1.000	27	57.1%	1.000	24	60.7%	1.000	34	60.7%	1.000	AAL93754.1| SSU ribosomal protein S3P
	32	60.7%	1.000	28	70.3%	1.000	24	60.7%	1.000	32	60.7%	1.000	
**FN1640**	11	57.7%	1.000	10	56.8%	1.000	6	53.2%	1.000	14	64.9%	1.000	AAL93755.1| LSU ribosomal protein L22P
	11	58.6%	1.000	8	53.2%	1.000	7	52.3%	1.000	15	64.0%	1.000	
**FN1641**	13	59.3%	1.000	7	49.5%	1.000	4	58.2%	1.000	11	58.2%	1.000	AAL93756.1| SSU ribosomal protein S19P
	13	59.3%	1.000	9	58.2%	1.000	8	58.2%	1.000	12	58.2%	1.000	

Fn Rep I Rep II | *FnPg* Rep I Rep II | *FnSg* Rep I Rep II | *FnPgSg* Rep I Rep II

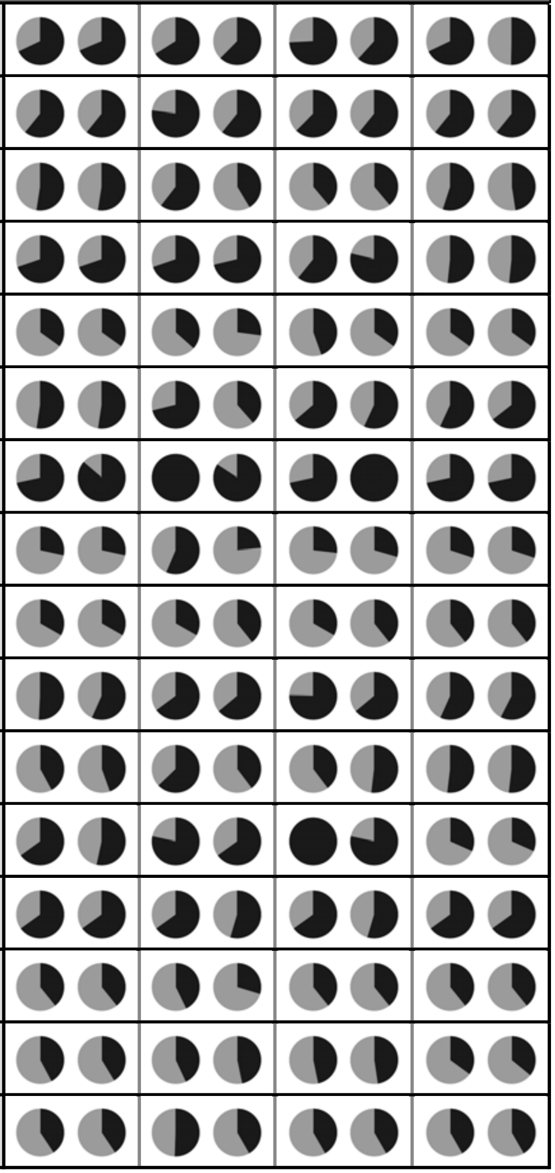

- Show detected proteins only
- Show all proteins
- Filter by category:
  GO: amino acid transport

Proteins found:
1424

Enter (or paste) list of ORFs

Find ORFs

Dot Plots Dot Plots

ORF	*Fn* n	*Fn* coverage	*Fn* prob	*FnPg* n	*FnPg* coverage	*FnPg* prob	*FnSg* n	*FnSg* coverage	*FnSg* prob	*FnPgSg* n	*FnPgSg* coverage	*FnPgSg* prob	Description
**FN1642**	22	45.3%	1.000	20	36.2%	1.000	22	53.6%	1.000	24	45.7%	1.000	AAL93757.1| LSU ribosomal protein L2P
	21	42.8%	1.000	16	47.8%	1.000	18	44.2%	1.000	26	51.8%	1.000	
**FN1643**	6	27.4%	1.000	11	44.2%	1.000	7	44.2%	1.000	8	42.1%	1.000	AAL93758.1| LSU ribosomal protein L23P
	6	27.4%	1.000	8	53.7%	1.000	4	30.5%	1.000	10	42.1%	1.000	
**FN1644**	20	54.1%	1.000	17	55.5%	1.000	14	49.3%	1.000	18	53.6%	1.000	AAL93759.1| LSU ribosomal protein L1E
	16	52.2%	1.000	15	53.1%	1.000	12	45.9%	1.000	17	53.6%	1.000	
**FN1645**	27	67.8%	1.000	27	69.7%	1.000	16	50.2%	1.000	26	67.8%	1.000	AAL93760.1| LSU ribosomal protein L3P
	25	67.8%	1.000	19	51.2%	1.000	17	66.4%	1.000	26	67.3%	1.000	
**FN1646**	15	56.3%	1.000	11	56.3%	1.000	12	56.3%	1.000	16	56.3%	1.000	AAL93761.1| SSU ribosomal protein S10P
	13	56.3%	1.000	11	56.3%	1.000	11	56.3%	1.000	15	56.3%	1.000	
**FN1647**	10	56.8%	1.000	8	56.8%	1.000	8	51.1%	1.000	13	83.5%	1.000	AAL93762.1| Hypothetical protein
	10	56.8%	1.000	8	51.1%	1.000	7	51.1%	1.000	12	67.6%	1.000	
**FN1652**	22	44.3%	1.000	12	21.6%	1.000	15	43.9%	1.000	21	46.7%	1.000	AAL93767.1| Oligopeptide-binding protein oppA
	22	43.5%	1.000	16	35.9%	1.000	18	49.3%	1.000	16	32.5%	1.000	
**FN1654**	9	14.7%	1.000	5	11.7%	1.000	29	52.9%	1.000	15	31.7%	1.000	AAL93769.1| Hypothetical protein
	9	15.4%	1.000	31	52.2%	1.000	35	54.5%	1.000	9	17.9%	1.000	
**FN1655**	18	35.5%	1.000	19	31.1%	1.000	25	44.7%	1.000	15	27.7%	1.000	AAL93770.1| Hypothetical cytosolic protein
	18	34.4%	1.000	19	35.9%	1.000	34	53.5%	1.000	13	25.0%	1.000	
**FN1656**	1	12.5%	0.813	0	0.0%	0.000	0	0.0%	0.000	4	25.0%	1.000	AAL93771.1| SSU ribosomal protein S18P
	1	12.5%	0.765	1	12.5%	0.862	1	12.5%	0.803	6	34.7%	1.000	
**FN1657**	13	66.7%	1.000	8	66.7%	1.000	5	58.1%	1.000	10	66.7%	1.000	AAL93772.1| SSU ribosomal protein S6P
	12	66.7%	1.000	5	47.6%	1.000	7	65.7%	1.000	13	66.7%	1.000	
**FN1658**	32	49.2%	1.000	15	28.4%	1.000	20	39.3%	1.000	31	51.7%	1.000	AAL93773.1| Prolyl-tRNA synthetase
	29	49.2%	1.000	24	47.1%	1.000	27	53.6%	1.000	32	54.3%	1.000	
**FN1660**	2	2.6%	0.985	0	0.0%	0.000	2	2.6%	0.989	0	0.0%	0.000	AAL93775.1| ATP-dependent DNA helicase recG
	3	7.7%	0.981	1	1.5%	0.364	4	6.2%	0.999	1	2.3%	0.393	
**FN1661**	8	32.1%	1.000	6	32.1%	1.000	5	33.3%	1.000	8	36.1%	1.000	AAL93776.1| Hypothetical cytosolic protein
	6	25.7%	1.000	10	45.8%	1.000	5	22.9%	1.000	5	16.9%	1.000	
**FN1662**	10	20.0%	1.000	8	21.3%	1.000	11	20.2%	1.000	13	24.6%	1.000	AAL93777.1| Hypothetical protein
	11	17.1%	1.000	12	26.9%	1.000	20	35.5%	1.000	10	25.4%	1.000	
**FN1663**	4	11.4%	1.000	0	0.0%	0.000	3	9.6%	0.999	5	12.1%	1.000	AAL93778.1| Hypothetical protein
	4	11.4%	1.000	5	13.4%	1.000	8	19.1%	1.000	4	11.2%	1.000	

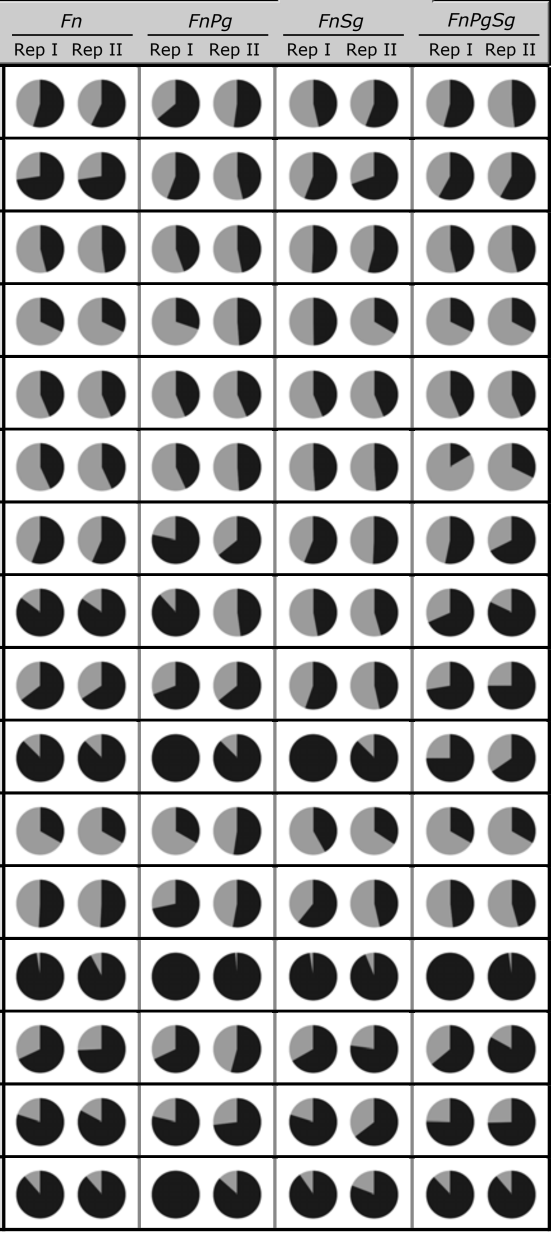

Show detected proteins only
Show all proteins
Filter by category:
GO: amino acid transport

Proteins found: 1424

Enter (or paste) list of ORFs
Find ORFs

Dot Plots Dot Plots

ORF	*Fn* n	*Fn* coverage	*Fn* prob	*FnPg* n	*FnPg* coverage	*FnPg* prob	*FnSg* n	*FnSg* coverage	*FnSg* prob	*FnPgSg* n	*FnPgSg* coverage	*FnPgSg* prob	Description
**FN1666**	1	6.8%	0.317	1	9.4%	0.265	1	11.1%	0.816	0	0.0%	0.000	AAL93781.1| Hypothetical protein
	3	27.4%	0.988	0	0.0%	0.000	0	0.0%	0.000	1	11.1%	0.986	
**FN1667**	0	0.0%	0.000	3	10.0%	1.000	1	3.8%	0.286	0	0.0%	0.000	AAL93782.1| dTDP-glucose 4,6-dehydratase
	2	8.0%	0.594	0	0.0%	0.000	3	8.5%	0.846	1	1.8%	0.308	
**FN1668**	0	0.0%		1	3.8%	0.278	1	6.6%	0.985	2	9.7%	0.912	AAL93783.1| Cholinephosphate cytidylyltransferase
	0	0.0%		4	15.5%	0.891	3	13.1%	0.998	1	3.8%	0.377	
**FN1670**	17	29.9%	1.000	8	14.5%	1.000	8	16.4%	1.000	14	22.9%	1.000	AAL93785.1| Choline kinase
	19	30.4%	1.000	12	24.2%	1.000	16	28.6%	1.000	10	15.9%	1.000	
**FN1671**	4	6.5%	1.000	2	5.6%	0.935	1	5.2%	0.297	2	7.9%	0.994	AAL93786.1| hypothetical exported protein
	3	10.6%	0.908	2	9.0%	0.751	1	5.6%	0.316	2	4.0%	0.922	
**FN1676**	0	0.0%		0	0.0%		0	0.0%	0.000	0	0.0%		AAL93791.1| Transposase
	0	0.0%		0	0.0%		3	11.0%	0.998	0	0.0%		
**FN1679**	30	67.0%	1.000	20	57.1%	1.000	30	72.4%	1.000	31	72.7%	1.000	AAL93794.1| LPS biosynthesis protein WbpG
	28	67.0%	1.000	37	78.0%	1.000	34	74.8%	1.000	26	67.6%	1.000	
**FN1683**	12	46.3%	1.000	10	34.5%	1.000	9	31.6%	1.000	9	45.2%	1.000	AAL93798.1| Acetyltransferase
	12	45.8%	1.000	7	22.0%	1.000	8	20.9%	1.000	6	29.4%	1.000	
**FN1684**	27	58.4%	1.000	25	57.8%	1.000	24	62.1%	1.000	31	68.1%	1.000	AAL93799.1| N-acetylneuraminate synthase
	29	63.0%	1.000	32	67.8%	1.000	29	67.2%	1.000	31	68.4%	1.000	
**FN1685**	15	40.4%	1.000	8	20.5%	1.000	9	26.4%	1.000	14	51.7%	1.000	AAL93800.1| dTDP-4-dehydrorhamnose reductase
	14	30.1%	1.000	12	30.5%	1.000	13	32.9%	1.000	13	35.6%	1.000	
**FN1686**	22	67.6%	1.000	16	55.2%	1.000	18	57.1%	1.000	24	67.6%	1.000	AAL93801.1| Spore coat polysaccharide biosynthesis protein spsF
	23	64.1%	1.000	18	54.8%	1.000	22	71.8%	1.000	21	64.9%	1.000	
**FN1687**	24	70.7%	1.000	17	54.1%	1.000	15	61.1%	1.000	21	70.7%	1.000	AAL93802.1| Gluconate 5-dehydrogenase
	22	67.8%	1.000	19	70.7%	1.000	20	66.7%	1.000	23	73.3%	1.000	
**FN1688**	11	27.6%	1.000	13	44.5%	1.000	8	43.4%	1.000	11	42.1%	1.000	AAL93803.1| Oxidoreductase
	11	31.0%	1.000	11	47.2%	1.000	11	44.5%	1.000	11	42.1%	1.000	
**FN1689**	31	66.1%	1.000	23	47.3%	1.000	22	58.0%	1.000	32	60.4%	1.000	AAL93804.1| UDP-N-acetylglucosamine 4,6-dehydratase
	34	62.5%	1.000	29	66.7%	1.000	25	73.2%	1.000	34	66.7%	1.000	
**FN1690**	16	41.2%	1.000	15	44.3%	1.000	12	34.0%	1.000	11	41.2%	1.000	AAL93805.1| Hypothetical protein
	18	47.7%	1.000	11	36.6%	1.000	15	44.7%	1.000	8	27.1%	1.000	
**FN1692**	16	37.3%	1.000	3	15.7%	1.000	10	29.5%	1.000	15	44.0%	1.000	AAL93807.1| Glycosyl transferase
	16	41.0%	1.000	13	40.3%	1.000	12	30.2%	1.000	12	36.9%	1.000	

Show detected proteins only
Show all proteins
Filter by category:
GO: amino acid transport

Proteins found: 1424

Enter (or paste) list of ORFs

Find ORFs

Dot Plots Dot Plots

ORF	*Fn*			*FnPg*			*FnSg*			*FnPgSg*			Description
	n	coverage	prob	n	coverage	prob	n	coverage	prob	n	coverage	prob	
FN1693	9	26.4%	1.000	4	19.2%	1.000	7	35.2%	1.000	12	53.9%	1.000	AAL93808.1| Hypothetical protein
	11	26.4%	1.000	6	26.9%	1.000	6	40.4%	1.000	7	31.6%	1.000	
FN1694	21	49.3%	1.000	15	42.1%	1.000	9	32.1%	1.000	19	58.6%	1.000	AAL93809.1| UDP-N-acetyl-D-quinovosamine 4-epimerase
	19	48.6%	1.000	13	35.9%	1.000	14	44.5%	1.000	18	52.8%	1.000	
FN1695	6	23.5%	1.000	3	15.2%	0.999	7	42.2%	1.000	7	26.1%	1.000	AAL93810.1| Probable quinovosaminephosphotransferae
	6	23.5%	1.000	4	30.0%	1.000	5	18.7%	1.000	7	37.4%	1.000	
FN1696	35	48.9%	1.000	23	36.4%	1.000	39	61.0%	1.000	29	47.6%	1.000	AAL93811.1| UDP-N-acetylglucosamine 4,6-dehydratase
	34	50.1%	1.000	35	53.9%	1.000	36	57.5%	1.000	27	44.6%	1.000	
FN1697	27	38.1%	1.000	11	28.7%	1.000	24	53.7%	1.000	18	42.4%	1.000	AAL93812.1| Hypothetical protein
	27	36.0%	1.000	22	43.3%	1.000	22	58.8%	1.000	18	42.4%	1.000	
FN1698	20	50.7%	1.000	14	35.2%	1.000	24	51.3%	1.000	20	47.0%	1.000	AAL93813.1| dTDP-4-dehydrorhamnose reductase
	19	45.6%	1.000	28	54.0%	1.000	25	52.3%	1.000	19	52.3%	1.000	
FN1700	2	16.3%	0.995	0	0.0%	0.000	3	25.0%	0.948	2	18.5%	0.852	AAL93815.1| Hypothetical protein
	6	25.0%	1.000	2	13.0%	0.990	1	8.2%	0.938	1	7.1%	0.307	
FN1701	10	25.2%	1.000	7	14.5%	1.000	14	29.6%	1.000	11	25.8%	1.000	AAL93816.1| ABC transporter ATP-binding protein
	11	25.4%	1.000	15	37.2%	1.000	15	37.0%	1.000	8	20.0%	1.000	
FN1703	17	36.1%	1.000	14	32.8%	1.000	25	50.6%	1.000	20	45.8%	1.000	AAL93818.1| ADP-L-glycero-D-manno-heptose-6-epimerase
	17	34.3%	1.000	30	55.4%	1.000	23	59.9%	1.000	19	41.0%	1.000	
FN1704	9	12.9%	1.000	3	3.9%	1.000	9	11.7%	1.000	4	6.4%	1.000	AAL93819.1| Serine protease
	11	16.7%	1.000	6	10.0%	1.000	8	10.4%	1.000	2	4.6%	0.993	
FN1707	4	11.0%	1.000	2	3.9%	0.855	0	0.0%		2	6.9%	0.999	AAL93822.1| Aldose 1-epimerase
	3	9.0%	1.000	0	0.0%	0.000	0	0.0%		2	9.3%	0.856	
FN1708	56	62.2%	1.000	39	48.6%	1.000	57	60.9%	1.000	47	59.9%	1.000	AAL93823.1| Polyribonucleotide nucleotidyltransferase
	58	66.7%	1.000	56	67.0%	1.000	54	62.4%	1.000	48	55.8%	1.000	
FN1711	4	10.8%	1.000	2	8.6%	0.910	0	0.0%	0.000	3	13.4%	0.987	AAL93826.1| Methyltransferase
	3	12.4%	1.000	2	5.7%	0.615	1	3.5%	0.625	3	12.7%	0.824	
FN1713	2	6.0%	0.733	1	3.4%	0.692	7	16.4%	1.000	1	1.7%	0.679	AAL93828.1| tRNA (Uracil-5-) -methyltransferase
	3	9.5%	0.955	5	15.7%	1.000	9	22.6%	1.000	2	5.6%	0.703	
FN1715	2	4.2%	0.826	0	0.0%	0.000	5	17.0%	1.000	1	2.3%	0.919	AAL93830.1| ATPase
	2	8.1%	0.989	3	8.8%	0.990	5	16.3%	1.000	0	0.0%	0.000	
FN1716	1	7.9%	0.889	0	0.0%	0.000	2	16.4%	0.875	1	3.7%	0.387	AAL93831.1| Hypothetical protein
	0	0.0%	0.000	1	10.1%	0.846	3	22.2%	0.979	0	0.0%	0.000	

Fn Rep I Rep II | *FnPg* Rep I Rep II | *FnSg* Rep I Rep II | *FnPgSg* Rep I Rep II

Show detected proteins only
Show all proteins
Filter by category:
GO: amino acid transport

Proteins found: 1424

Enter (or paste) list of ORFs

Find ORFs

Dot Plots Dot Plots

ORF	*Fn* n	*Fn* coverage	*Fn* prob	*FnPg* n	*FnPg* coverage	*FnPg* prob	*FnSg* n	*FnSg* coverage	*FnSg* prob	*FnPgSg* n	*FnPgSg* coverage	*FnPgSg* prob	Description
**FN1717**	13	22.8%	1.000	6	11.8%	1.000	7	12.8%	1.000	9	15.9%	1.000	AAL93832.1| NAD-dependent DNA ligase
	16	28.2%	1.000	6	12.4%	1.000	9	16.7%	1.000	7	15.7%	1.000	
**FN1718**	54	48.9%	1.000	28	40.4%	1.000	54	62.8%	1.000	49	50.2%	1.000	AAL93833.1| Protein translocase subunit secA
	58	52.7%	1.000	55	59.1%	1.000	53	62.8%	1.000	48	54.5%	1.000	
**FN1719**	33	70.3%	1.000	22	58.2%	1.000	27	70.3%	1.000	32	68.6%	1.000	AAL93834.1| Hypothetical protein
	34	70.3%	1.000	27	70.3%	1.000	30	70.3%	1.000	29	69.9%	1.000	
**FN1722**	3	15.5%	1.000	2	11.2%	0.997	3	25.0%	0.999	0	0.0%		AAL93837.1| Glucose inhibited division protein B
	4	18.5%	1.000	0	0.0%	0.000	3	14.7%	1.000	0	0.0%		
**FN1723**	17	24.5%	1.000	12	27.0%	1.000	22	33.6%	1.000	19	31.6%	1.000	AAL93838.1| Glucose inhibited division protein A
	21	27.5%	1.000	19	33.6%	1.000	20	35.1%	1.000	20	34.8%	1.000	
**FN1724**	3	12.4%	1.000	3	17.9%	1.000	4	17.9%	1.000	4	21.6%	1.000	AAL93839.1| Potassium uptake protein KtrA
	2	10.6%	1.000	9	41.7%	1.000	8	28.9%	1.000	3	14.7%	0.999	
**FN1727**	0	0.0%		0	0.0%	0.000	4	9.4%	0.919	3	9.8%	0.996	AAL93842.1| Chloride channel protein
	0	0.0%		1	3.3%	0.517	1	6.3%	0.299	3	8.6%	0.998	
**FN1728**	8	40.2%	1.000	7	46.3%	1.000	4	18.2%	1.000	4	25.7%	1.000	AAL93843.1| Pyrrolidone-carboxylate peptidase
	6	36.0%	1.000	10	46.7%	1.000	5	22.0%	1.000	3	21.0%	1.000	
**FN1730**	6	13.9%	1.000	5	7.5%	1.000	6	17.2%	1.000	1	2.9%	0.989	AAL93845.1| Para-aminobenzoate synthase component I
	6	10.2%	1.000	11	24.5%	1.000	10	22.1%	1.000	5	14.1%	1.000	
**FN1731**	2	3.4%	0.763	3	29.1%	1.000	4	33.0%	1.000	3	13.8%	0.952	AAL93846.1| Anthranilate synthase component II
	2	3.4%	0.737	3	22.2%	1.000	6	34.0%	1.000	2	3.4%	0.883	
**FN1732**	8	28.3%	1.000	5	15.1%	1.000	9	34.7%	1.000	7	30.6%	1.000	AAL93847.1| Hypothetical protein
	5	23.4%	1.000	13	55.1%	1.000	11	41.9%	1.000	5	22.6%	1.000	
**FN1733**	1	6.2%	0.975	2	19.4%	0.998	4	23.2%	1.000	1	6.2%	0.984	AAL93848.1| V-type sodium ATP synthase subunit D
	1	6.2%	0.979	3	18.0%	0.998	7	40.8%	1.000	2	10.0%	0.999	
**FN1734**	20	52.0%	1.000	20	40.8%	1.000	24	63.5%	1.000	20	46.3%	1.000	AAL93849.1| V-type sodium ATP synthase subunit B
	21	53.9%	1.000	28	66.2%	1.000	27	63.3%	1.000	24	53.3%	1.000	
**FN1735**	15	38.7%	1.000	12	36.4%	1.000	20	58.6%	1.000	14	53.4%	1.000	AAL93850.1| V-type sodium ATP synthase subunit A
	15	47.1%	1.000	22	62.8%	1.000	23	56.5%	1.000	11	36.1%	1.000	
**FN1736**	6	31.7%	1.000	4	27.2%	1.000	9	41.1%	1.000	4	22.8%	1.000	AAL93851.1| V-type sodium ATP synthase subunit A
	6	31.7%	1.000	13	59.4%	1.000	7	38.6%	1.000	4	20.3%	1.000	
**FN1737**	3	41.9%	1.000	3	40.0%	1.000	3	29.5%	1.000	1	15.2%	0.989	AAL93852.1| V-type sodium ATP synthase subunit G
	3	41.9%	1.000	3	36.2%	1.000	5	61.0%	1.000	2	31.4%	0.989	

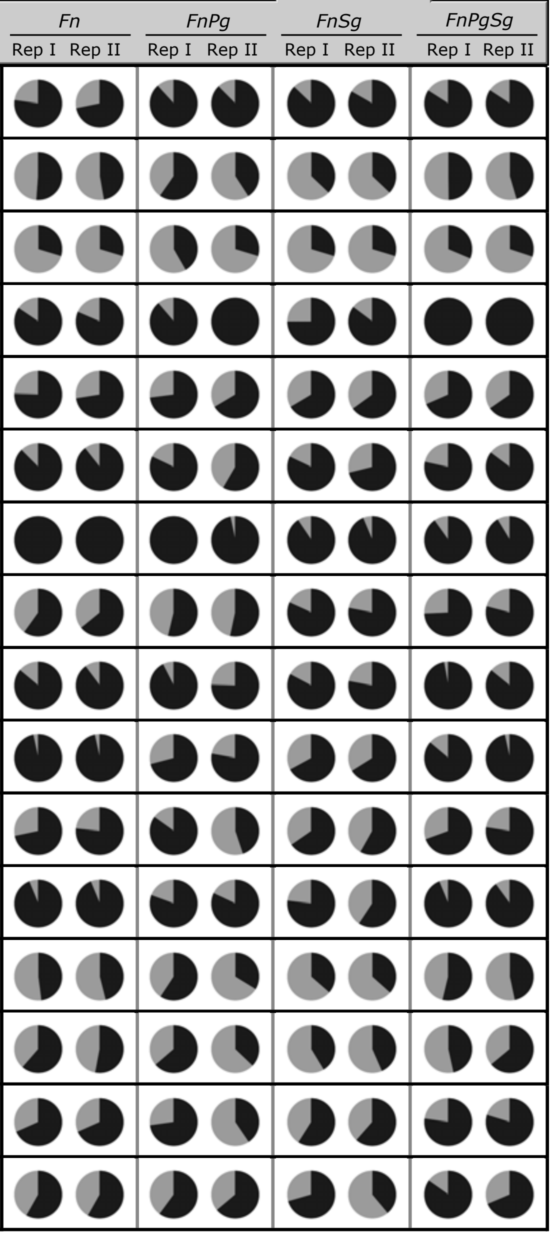

Show detected proteins only
Show all proteins
Filter by category:
GO: amino acid transport

Proteins found:
1424

Enter (or paste) list of ORFs
Find ORFs

Dot Plots Dot Plots

ORF	*Fn* n	*Fn* coverage	*Fn* prob	*FnPg* n	*FnPg* coverage	*FnPg* prob	*FnSg* n	*FnSg* coverage	*FnSg* prob	*FnPgSg* n	*FnPgSg* coverage	*FnPgSg* prob	Description
**FN1738**	7	21.3%	1.000	14	29.9%	1.000	17	44.0%	1.000	4	12.3%	1.000	AAL93853.1| V-type sodium ATP synthase subunit C
	7	18.0%	1.000	18	49.4%	1.000	22	59.6%	1.000	7	18.0%	1.000	
**FN1739**	2	21.3%	0.975	3	29.0%	1.000	3	29.0%	1.000	3	20.2%	1.000	AAL93854.1| V-type sodium ATP synthase subunit E
	3	28.4%	1.000	5	32.8%	1.000	2	21.3%	0.999	5	25.1%	1.000	
**FN1740**	1	11.9%	0.981	2	11.9%	0.991	2	23.1%	1.000	2	23.1%	0.996	AAL93855.1| V-type sodium ATP synthase subunit K
	1	11.9%	0.982	3	25.0%	1.000	3	39.4%	1.000	1	11.9%	0.986	
**FN1741**	4	11.0%	1.000	9	13.0%	1.000	12	26.6%	1.000	6	14.6%	1.000	AAL93856.1| V-type sodium ATP synthase subunit I
	6	11.4%	1.000	12	25.5%	1.000	11	24.8%	1.000	6	14.7%	1.000	
**FN1742**	2	25.0%	0.999	3	25.0%	1.000	2	25.0%	0.983	2	24.1%	0.989	AAL93857.1| V-type sodium ATP synthase subunit G
	1	8.3%	0.975	4	34.3%	1.000	0	0.0%	0.000	2	25.0%	0.998	
**FN1743**	1	4.5%	0.930	1	4.5%	0.863	1	4.5%	0.520	1	3.4%	0.910	AAL93858.1| Multidrug-efflux transporter 2 regulator
	1	8.2%	0.561	5	20.5%	1.000	2	7.5%	0.926	1	3.4%	0.951	
**FN1745**	3	15.2%	1.000	2	13.9%	0.849	9	34.3%	1.000	5	21.0%	1.000	AAL93860.1| Cystathionine gamma-synthase
	3	13.6%	1.000	8	30.1%	1.000	11	41.4%	1.000	3	12.9%	1.000	
**FN1746**	1	2.9%	0.928	1	4.3%	0.851	7	19.3%	1.000	0	0.0%	0.000	AAL93861.1| Cystathionine beta-lyase
	0	0.0%	0.000	5	12.6%	1.000	7	19.8%	1.000	2	6.7%	0.987	
**FN1752**	0	0.0%		0	0.0%	0.000	0	0.0%		3	15.0%	0.846	AAL93867.1| Regulatory protein TENI
	0	0.0%		1	4.4%	0.403	0	0.0%		1	7.8%	0.388	
**FN1754**	0	0.0%		3	21.8%	1.000	0	0.0%	0.000	1	6.2%	0.585	AAL93869.1| Thiazole biosynthesis protein thiG
	0	0.0%		1	11.7%	0.305	1	4.7%	0.419	0	0.0%	0.000	
**FN1762**	1	4.4%	0.889	0	0.0%	0.000	3	21.0%	0.984	1	8.1%	0.356	AAL93875.1| Protein yaaA
	2	4.4%	0.906	1	3.2%	0.656	2	13.7%	0.998	0	0.0%	0.000	
**FN1763**	7	31.0%	1.000	8	42.9%	1.000	10	55.4%	1.000	2	13.6%	1.000	AAL93876.1| Hypothetical cytosolic protein
	5	15.2%	1.000	10	52.7%	1.000	10	39.7%	1.000	3	19.6%	1.000	
**FN1764**	59	81.8%	1.000	40	71.7%	1.000	59	83.2%	1.000	46	76.3%	1.000	AAL93877.1| Enolase
	60	86.6%	1.000	70	92.6%	1.000	60	87.8%	1.000	47	77.2%	1.000	
**FN1765**	51	72.4%	1.000	24	49.9%	1.000	40	73.7%	1.000	43	65.5%	1.000	AAL93878.1| Pyruvate kinase
	46	70.9%	1.000	41	67.6%	1.000	37	70.9%	1.000	43	65.5%	1.000	
**FN1780**	8	32.4%	1.000	3	20.2%	1.000	6	30.0%	1.000	8	29.6%	1.000	AAL93879.1| Hypothetical protein
	9	32.4%	1.000	8	30.4%	1.000	5	25.5%	1.000	8	32.4%	1.000	
**FN1781**	41	31.6%	1.000	32	31.6%	1.000	41	41.0%	1.000	44	35.9%	1.000	AAL93880.1| LytB protein
	40	34.3%	1.000	45	38.5%	1.000	46	43.8%	1.000	45	36.6%	1.000	

Fn Rep I Rep II | *FnPg* Rep I Rep II | *FnSg* Rep I Rep II | *FnPgSg* Rep I Rep II

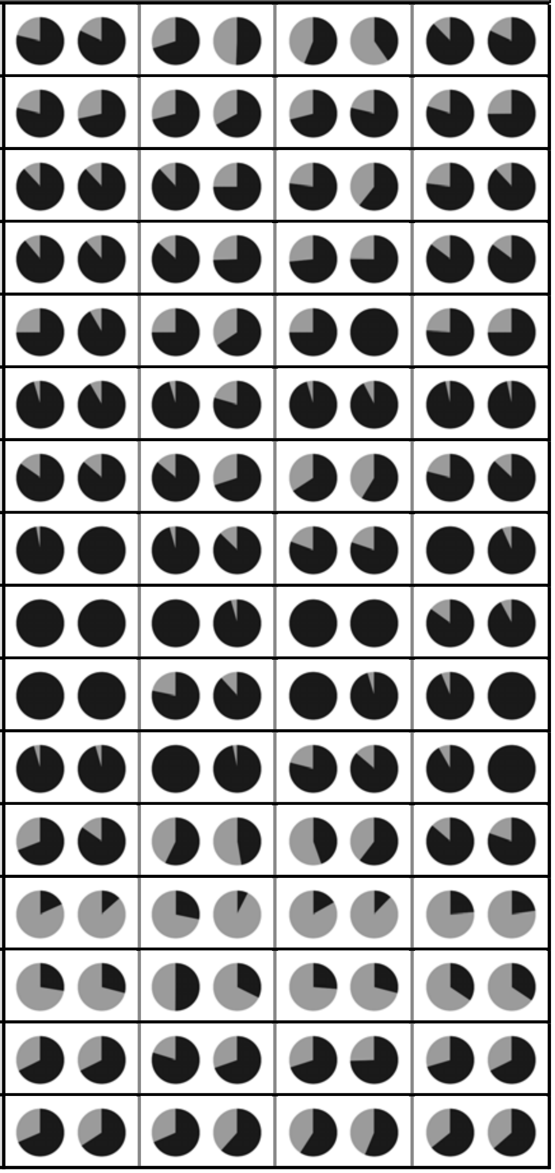

Show detected proteins only
Show all proteins
Filter by category:
GO: amino acid transport

Proteins found: 1424

Enter (or paste) list of ORFs

Find ORFs

Dot Plots Dot Plots

ORF	Fn n	Fn coverage	Fn prob	FnPg n	FnPg coverage	FnPg prob	FnSg n	FnSg coverage	FnSg prob	FnPgSg n	FnPgSg coverage	FnPgSg prob	Description
FN1783	1	9.5%	0.937	2	17.2%	0.477	1	9.5%	0.985	2	21.5%	0.995	AAL93882.1| Ethanolamine utilization protein eutJ
	1	5.5%	0.979	5	32.1%	1.000	3	19.3%	1.000	0	0.0%	0.000	
FN1784	5	27.8%	1.000	1	10.6%	0.266	5	27.8%	1.000	2	10.6%	0.999	AAL93883.1| unknown
	5	27.8%	1.000	4	27.2%	1.000	4	27.2%	1.000	3	19.2%	1.000	
FN1785	8	56.7%	1.000	1	6.4%	0.935	4	33.1%	1.000	7	41.4%	1.000	AAL93884.1| Hypothetical protein
	6	35.7%	1.000	3	14.0%	0.991	2	7.6%	0.991	8	42.0%	1.000	
FN1786	10	35.0%	1.000	3	14.9%	1.000	11	40.9%	1.000	13	53.6%	1.000	AAL93885.1| ADP-heptose synthase
	15	53.3%	1.000	12	44.6%	1.000	12	39.3%	1.000	14	50.2%	1.000	
FN1787	26	35.0%	1.000	8	14.2%	1.000	2	3.7%	0.998	20	36.5%	1.000	AAL93886.1| Tetratricopeptide repeat family protein
	22	37.3%	1.000	8	14.0%	1.000	8	11.3%	1.000	13	26.4%	1.000	
FN1788	9	38.8%	1.000	5	30.0%	1.000	8	31.2%	1.000	11	48.1%	1.000	AAL93887.1| 2C-methyl-D-erythritol 2,4-cyclodiphosphate synthase
	9	36.9%	1.000	11	48.1%	1.000	6	33.8%	1.000	7	28.1%	1.000	
FN1790	2	7.5%	0.998	3	13.9%	1.000	8	41.0%	1.000	6	34.1%	1.000	AAL93889.1| Cob(I)alamin adenosyltransferase
	3	15.6%	0.991	10	51.4%	1.000	9	47.4%	1.000	3	19.1%	1.000	
FN1791	0	0.0%		0	0.0%	0.000	3	17.1%	1.000	1	4.4%	0.580	AAL93890.1| Mutator MutT protein
	0	0.0%		2	9.1%	0.988	4	22.2%	1.000	0	0.0%	0.000	
FN1792	15	82.6%	1.000	11	75.2%	1.000	16	86.8%	1.000	22	100.0 %	1.000	AAL93891.1| Hypothetical protein
	14	75.2%	1.000	18	90.9%	1.000	16	86.8%	1.000	19	86.8%	1.000	
FN1793	18	36.4%	1.000	13	27.8%	1.000	29	43.2%	1.000	19	37.3%	1.000	AAL93892.1| Phosphoenolpyruvate-protein phosphotransferase
	15	28.2%	1.000	30	52.2%	1.000	27	48.5%	1.000	19	35.2%	1.000	
FN1794	8	82.8%	1.000	4	56.3%	1.000	5	73.6%	1.000	7	73.6%	1.000	AAL93893.1| Phosphocarrier protein HPr
	7	82.8%	1.000	5	82.8%	1.000	3	56.3%	1.000	8	73.6%	1.000	
FN1795	1	5.7%	0.946	1	5.7%	0.505	2	12.2%	0.998	1	6.5%	0.561	AAL93894.1| Hypothetical protein
	2	12.2%	0.998	1	5.7%	0.930	7	27.6%	1.000	0	0.0%	0.000	
FN1796	2	24.1%	0.983	0	0.0%		1	25.3%	0.937	1	15.2%	0.361	AAL93895.1| unknown
	3	25.3%	0.989	0	0.0%		5	49.4%	1.000	1	15.2%	0.447	
FN1797	11	37.0%	1.000	7	26.3%	1.000	13	44.7%	1.000	10	36.2%	1.000	AAL93896.1| Spermidine/putrescine transport ATP-binding protein potA
	10	37.5%	1.000	13	42.8%	1.000	11	39.4%	1.000	9	39.1%	1.000	
FN1798	2	10.6%	0.998	0	0.0%	0.000	3	14.4%	0.933	4	13.7%	1.000	AAL93897.1| Spermidine/putrescine transport system permease protein potB
	4	21.5%	1.000	4	21.5%	1.000	5	25.4%	1.000	4	18.3%	1.000	
FN1800	19	30.7%	1.000	10	19.3%	1.000	11	27.4%	1.000	14	29.9%	1.000	AAL93899.1| Peptidyl-prolyl cis-trans isomerase
	17	30.7%	1.000	12	27.4%	1.000	11	29.6%	1.000	13	28.5%	1.000	

Fn / FnPg / FnSg / FnPgSg: Rep I, Rep II

Show detected proteins only
Show all proteins
Filter by category:
GO: amino acid transport

Proteins found: 1424

Enter (or paste) list of ORFs

Find ORFs

Dot Plots Dot Plots

ORF	*Fn* n	*Fn* coverage	*Fn* prob	*FnPg* n	*FnPg* coverage	*FnPg* prob	*FnSg* n	*FnSg* coverage	*FnSg* prob	*FnPgSg* n	*FnPgSg* coverage	*FnPgSg* prob	Description
**FN1801**	2	13.3%	1.000	2	14.0%	1.000	3	14.0%	1.000	3	20.8%	1.000	AAL93900.1| Sodium/glutamate symport carrier protein
	3	14.0%	1.000	4	19.9%	1.000	2	13.3%	1.000	1	5.3%	0.986	
**FN1803**	3	14.3%	1.000	2	6.0%	0.777	4	25.8%	1.000	3	19.8%	1.000	AAL93902.1| Transcriptional regulator, TetR family
	2	6.0%	1.000	2	13.4%	0.998	4	24.0%	1.000	1	11.5%	0.769	
**FN1804**	4	7.2%	1.000	0	0.0%	0.000	13	27.8%	1.000	2	4.3%	0.743	AAL93903.1| Aminoacyl-histidine dipeptidase
	6	11.7%	1.000	7	16.5%	1.000	14	25.7%	1.000	2	6.6%	0.989	
**FN1807**	21	57.6%	1.000	16	54.9%	1.000	16	57.6%	1.000	20	57.6%	1.000	AAL93906.1| Hypothetical protein
	19	57.6%	1.000	17	57.6%	1.000	15	53.4%	1.000	18	57.6%	1.000	
**FN1808**	6	38.4%	1.000	2	18.4%	0.981	3	30.4%	1.000	8	38.4%	1.000	AAL93907.1| Hypothetical protein
	7	40.0%	1.000	3	30.4%	1.000	2	18.4%	0.999	6	28.0%	1.000	
**FN1809**	9	26.1%	1.000	3	15.5%	1.000	8	31.4%	1.000	9	35.7%	1.000	AAL93908.1| Iron/zinc/copper-binding protein
	8	30.0%	1.000	11	49.5%	1.000	6	27.2%	1.000	8	30.0%	1.000	
**FN1811**	14	68.6%	1.000	3	15.7%	1.000	6	44.5%	1.000	5	26.2%	1.000	AAL93910.1| Manganese transport system ATP-binding protein mntA
	12	46.3%	1.000	9	54.1%	1.000	7	51.1%	1.000	8	41.0%	1.000	
**FN1812**	16	58.6%	1.000	11	32.1%	1.000	10	36.1%	1.000	22	69.9%	1.000	AAL93911.1| Manganese-binding protein
	16	58.6%	1.000	11	39.1%	1.000	9	34.1%	1.000	18	55.6%	1.000	
**FN1813**	7	16.2%	1.000	1	5.6%	0.984	3	14.9%	0.996	6	28.1%	1.000	AAL93912.1| Manganese-binding protein
	7	19.5%	1.000	6	28.1%	1.000	3	11.9%	0.993	8	31.8%	1.000	
**FN1814**	7	32.3%	1.000	0	0.0%	0.000	4	27.6%	1.000	7	40.1%	1.000	AAL93913.1| Hypothetical protein
	6	32.3%	1.000	2	9.9%	1.000	2	13.0%	0.993	4	29.7%	1.000	
**FN1816**	4	17.2%	1.000	3	12.4%	0.999	2	8.2%	0.996	4	18.9%	1.000	AAL93915.1| unknown
	4	15.0%	1.000	5	22.3%	1.000	8	25.3%	1.000	3	15.5%	0.997	
**FN1817**	0	0.0%	0.000	3	2.0%	0.959	0	0.0%	0.000	12	6.9%	1.000	AAL93916.1| Hemolysin
	5	2.5%	0.993	8	4.8%	0.999	6	2.8%	0.999	4	2.0%	0.999	
**FN1819**	4	8.1%	1.000	1	3.4%	0.337	1	1.7%	0.333	1	1.7%	0.482	AAL93918.1| Export ABC transporter
	7	9.8%	1.000	0	0.0%	0.000	0	0.0%	0.000	1	2.2%	0.335	
**FN1820**	4	12.6%	1.000	1	2.3%	0.930	2	6.7%	0.572	2	4.1%	0.956	AAL93919.1| Export ABC transporter
	4	9.4%	1.000	1	2.8%	0.517	2	5.0%	0.660	0	0.0%	0.000	
**FN1822**	6	29.6%	1.000	1	5.3%	0.315	1	6.5%	0.290	0	0.0%		AAL93921.1| Flavodoxin
	6	34.9%	1.000	0	0.0%	0.000	0	0.0%	0.000	0	0.0%		
**FN1825**	2	14.5%	0.718	0	0.0%	0.000	1	9.0%	0.923	4	24.8%	1.000	AAL93924.1| Hypothetical protein
	2	17.2%	0.984	1	9.0%	0.983	1	9.0%	0.918	5	55.2%	1.000	

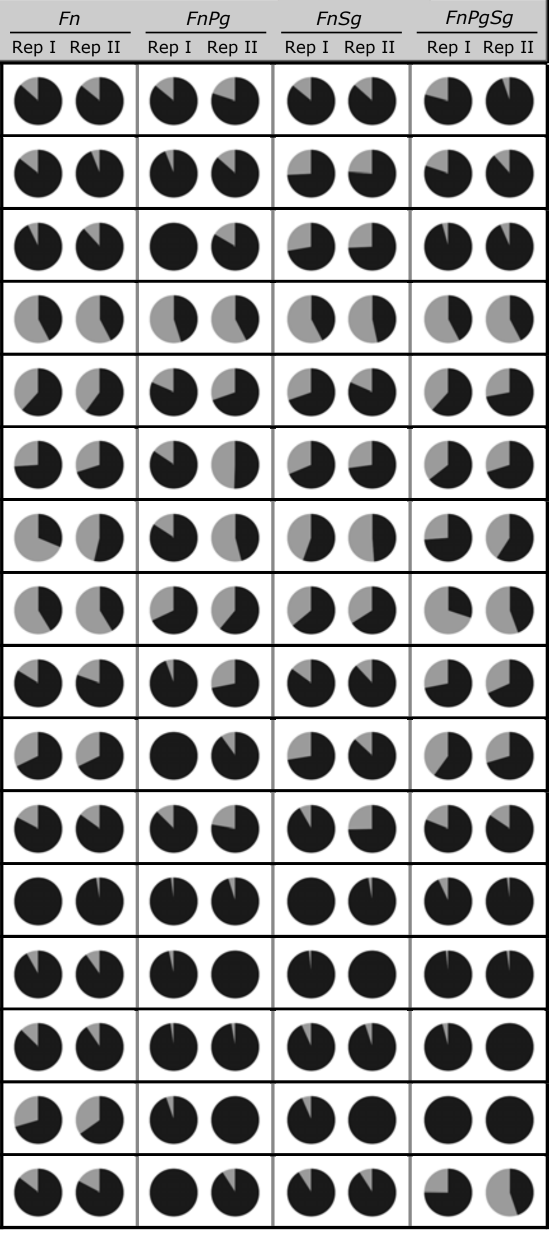

ORF	*Fn* n	*Fn* coverage	*Fn* prob	*FnPg* n	*FnPg* coverage	*FnPg* prob	*FnSg* n	*FnSg* coverage	*FnSg* prob	*FnPgSg* n	*FnPgSg* coverage	*FnPgSg* prob	Description
**FN1826**	13	37.8%	1.000	9	29.3%	1.000	9	23.9%	1.000	15	43.4%	1.000	AAL93925.1| Protease
	15	44.1%	1.000	12	32.0%	1.000	11	36.8%	1.000	13	37.6%	1.000	
**FN1827**	13	30.3%	1.000	9	27.6%	1.000	19	50.0%	1.000	15	36.1%	1.000	AAL93926.1| Replicative DNA helicase
	14	35.0%	1.000	20	50.4%	1.000	20	49.1%	1.000	13	30.9%	1.000	
**FN1828**	7	43.0%	1.000	6	36.2%	1.000	6	43.0%	1.000	8	36.2%	1.000	AAL93927.1| LSU ribosomal protein L9P
	11	47.7%	1.000	5	43.0%	1.000	6	43.0%	1.000	9	52.3%	1.000	
**FN1830**	14	28.7%	1.000	11	23.6%	1.000	19	37.8%	1.000	10	22.1%	1.000	AAL93929.1| DNA polymerase III subunits gamma and tau
	14	28.3%	1.000	19	35.7%	1.000	20	36.2%	1.000	7	19.6%	1.000	
**FN1831**	20	46.8%	1.000	16	37.5%	1.000	25	48.3%	1.000	16	34.5%	1.000	AAL93930.1| Nitrogen assimilation regulatory protein
	21	48.9%	1.000	23	48.9%	1.000	29	54.3%	1.000	13	31.0%	1.000	
**FN1832**	1	4.3%	0.750	0	0.0%	0.000	3	9.0%	0.999	3	16.7%	1.000	AAL93931.1| TonB protein
	2	11.5%	0.978	1	4.7%	0.947	2	4.7%	0.999	1	4.7%	0.330	
**FN1833**	2	25.4%	0.999	0	0.0%	0.000	2	20.2%	1.000	4	38.6%	1.000	AAL93932.1| Biopolymer transport exbD protein
	1	11.4%	0.982	4	24.6%	0.999	2	20.2%	0.998	3	24.6%	1.000	
**FN1834**	12	28.9%	1.000	6	18.2%	1.000	5	33.2%	1.000	7	27.3%	1.000	AAL93933.1| Biopolymer transport exbB protein
	11	28.9%	1.000	8	32.1%	1.000	4	11.8%	1.000	9	38.0%	1.000	
**FN1836**	36	36.3%	1.000	11	15.1%	1.000	19	25.7%	1.000	31	33.1%	1.000	AAL93935.1| Tetratricopeptide repeat family protein
	32	33.9%	1.000	27	30.2%	1.000	28	32.9%	1.000	26	27.9%	1.000	
**FN1838**	2	3.5%	0.999	1	3.5%	0.984	2	4.3%	1.000	3	5.5%	1.000	AAL93937.1| Glycerol uptake facilitator protein
	2	3.5%	1.000	4	26.8%	1.000	2	4.3%	0.998	2	3.5%	1.000	
**FN1839**	29	62.6%	1.000	33	57.5%	1.000	36	78.5%	1.000	32	57.1%	1.000	AAL93938.1| Glycerol kinase
	27	56.1%	1.000	44	78.5%	1.000	36	73.0%	1.000	36	61.4%	1.000	
**FN1840**	6	13.9%	1.000	15	29.2%	1.000	14	35.5%	1.000	12	31.6%	1.000	AAL93939.1| Dihydroxyacetone kinase
	6	13.9%	1.000	19	46.1%	1.000	12	35.5%	1.000	12	34.0%	1.000	
**FN1841**	5	35.1%	1.000	5	34.7%	1.000	4	26.2%	1.000	9	41.1%	1.000	AAL93940.1| Dihydroxyacetone kinase
	5	25.2%	1.000	4	20.3%	1.000	6	33.7%	1.000	9	49.0%	1.000	
**FN1842**	6	53.7%	1.000	4	43.4%	1.000	8	57.4%	1.000	7	53.7%	1.000	AAL93941.1| Dihydroxyacetone kinase phosphotransfer protein
	7	53.7%	1.000	9	53.7%	1.000	8	53.7%	1.000	6	43.4%	1.000	
**FN1843**	1	1.4%	0.227	3	8.3%	1.000	1	3.5%	0.981	2	5.0%	0.995	AAL93942.1| Surface antigen
	2	3.9%	0.674	0	0.0%	0.000	2	4.8%	0.990	0	0.0%	0.000	
**FN1844**	2	11.3%	0.987	0	0.0%	0.000	5	38.9%	1.000	1	11.3%	0.458	AAL93943.1| Ketoacyl reductase hetN
	4	23.0%	1.000	3	17.9%	0.997	2	15.2%	0.990	0	0.0%	0.000	

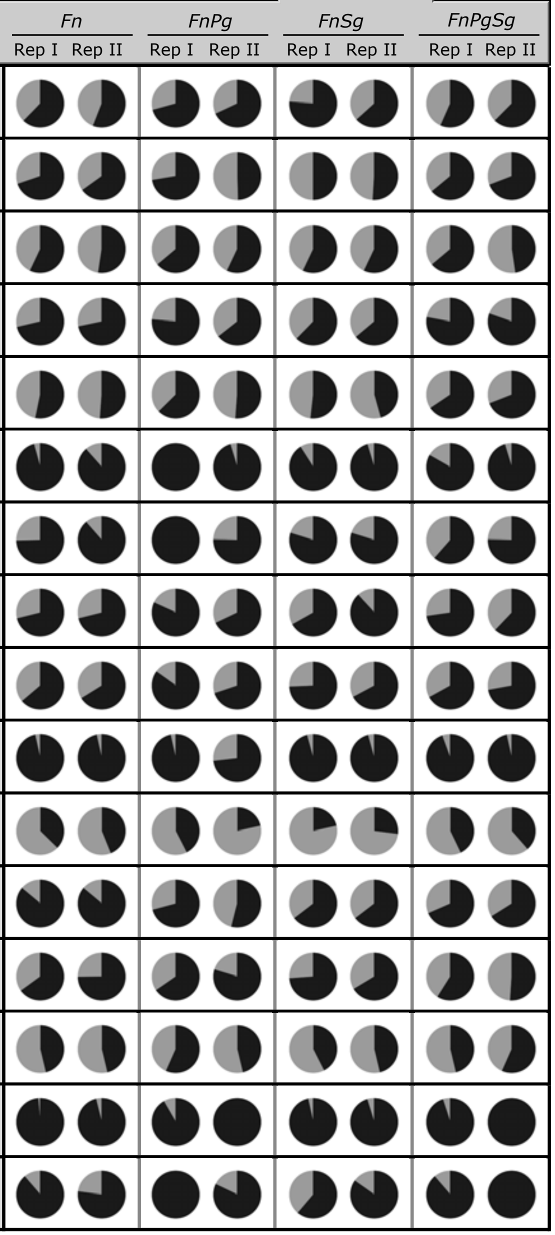

Show detected proteins only
Show all proteins
Filter by category:
GO: amino acid transport
Proteins found: 1424
Enter (or paste) list of ORFs
Find ORFs
Dot Plots Dot Plots

ORF	*Fn* n	*Fn* coverage	*Fn* prob	*FnPg* n	*FnPg* coverage	*FnPg* prob	*FnSg* n	*FnSg* coverage	*FnSg* prob	*FnPgSg* n	*FnPgSg* coverage	*FnPgSg* prob	Description
**FN1847**	6	18.0%	1.000	3	8.2%	1.000	8	23.5%	1.000	2	8.5%	0.993	AAL93946.1| DTDP-4-dehydrorhamnose 3,5-epimerase
	3	11.0%	0.999	14	47.6%	1.000	13	43.9%	1.000	0	0.0%	0.000	
**FN1848**	1	5.7%	0.292	1	5.7%	0.410	2	9.5%	0.641	1	6.1%	0.332	AAL93947.1| Metal dependent hydrolase
	0	0.0%	0.000	4	21.7%	0.996	2	10.3%	0.992	3	14.8%	0.991	
**FN1849**	6	25.0%	1.000	2	5.9%	0.750	12	33.5%	1.000	4	11.6%	0.971	AAL93948.1| Coenzyme F390 synthetase
	3	11.6%	1.000	11	29.7%	1.000	17	42.0%	1.000	2	5.4%	0.998	
**FN1850**	3	13.9%	0.994	1	8.4%	0.359	6	38.2%	1.000	1	3.9%	0.988	AAL93949.1| 3-oxoacyl-[acyl-carrier-protein] synthase III
	4	20.4%	1.000	11	49.5%	1.000	11	53.1%	1.000	2	3.9%	0.990	
**FN1851**	14	35.0%	1.000	11	30.4%	1.000	21	53.5%	1.000	13	42.6%	1.000	AAL93950.1| Ribonuclease PH
	16	38.9%	1.000	26	63.4%	1.000	20	49.1%	1.000	13	32.9%	1.000	
**FN1852**	7	54.8%	1.000	4	33.3%	1.000	5	34.1%	1.000	8	54.8%	1.000	AAL93951.1| unknown
	7	48.4%	1.000	6	34.1%	1.000	4	23.8%	1.000	7	40.5%	1.000	
**FN1853**	7	47.8%	1.000	5	18.4%	1.000	3	25.0%	1.000	3	24.3%	1.000	AAL93952.1| Methylaspartate mutase
	5	41.9%	1.000	4	25.0%	1.000	6	36.8%	1.000	3	24.3%	1.000	
**FN1854**	12	26.6%	1.000	6	19.7%	1.000	12	37.7%	1.000	11	30.3%	1.000	AAL93953.1| Methylaspartate mutase
	12	32.5%	1.000	12	36.1%	1.000	15	42.0%	1.000	6	18.2%	1.000	
**FN1855**	2	5.2%	0.982	2	9.5%	0.795	3	10.9%	0.996	2	5.6%	0.863	AAL93954.1| Methylaspartate mutase
	2	4.1%	0.996	3	8.9%	1.000	3	8.9%	1.000	1	2.7%	0.509	
**FN1856**	30	66.4%	1.000	30	74.2%	1.000	29	74.2%	1.000	29	68.7%	1.000	AAL93955.1| Butyrate-acetoacetate CoA-transferase subunit B
	25	62.7%	1.000	30	74.2%	1.000	27	68.7%	1.000	25	66.4%	1.000	
**FN1857**	31	90.3%	1.000	28	89.9%	1.000	28	92.2%	1.000	29	91.7%	1.000	AAL93956.1| Acetoacetate: butyrate/acetate coenzyme A transferase
	30	89.4%	1.000	30	95.9%	1.000	20	88.0%	1.000	29	88.0%	1.000	
**FN1858**	11	14.4%	1.000	5	8.3%	1.000	1	5.5%	0.411	13	14.4%	1.000	AAL93957.1| Short-chain fatty acids transporter
	12	14.4%	1.000	1	7.4%	0.318	1	2.0%	0.671	13	14.4%	1.000	
**FN1859**	40	62.0%	1.000	23	48.1%	1.000	56	78.5%	1.000	43	72.0%	1.000	AAL93958.1| Major outer membrane protein
	36	57.9%	1.000	57	84.2%	1.000	45	72.8%	1.000	45	60.1%	1.000	
**FN1860**	6	8.2%	1.000	1	3.4%	0.984	4	11.0%	1.000	5	7.8%	1.000	AAL93959.1| NA+/H+ antiporter NHAC
	5	8.2%	1.000	5	9.9%	1.000	4	8.0%	1.000	5	7.8%	1.000	
**FN1862**	21	62.4%	1.000	22	64.3%	1.000	14	43.7%	1.000	21	70.7%	1.000	AAL93961.1| L-beta-lysine 5,6-aminomutase beta subunit
	19	51.3%	1.000	22	78.3%	1.000	20	71.9%	1.000	20	67.3%	1.000	
**FN1863**	36	49.8%	1.000	37	52.1%	1.000	32	54.4%	1.000	41	53.3%	1.000	AAL93962.1| L-beta-lysine 5,6-aminomutase alpha subunit
	39	49.8%	1.000	35	59.5%	1.000	32	49.8%	1.000	44	56.9%	1.000	

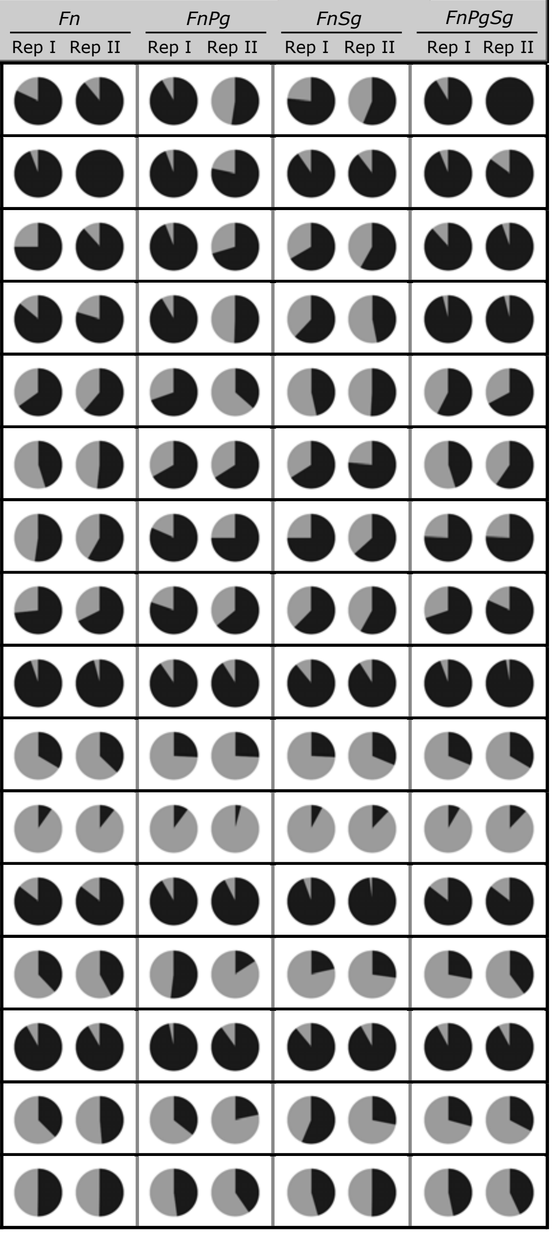

Show detected proteins only
Show all proteins
Filter by category:
GO: amino acid transport

Proteins found: 1424

Enter (or paste) list of ORFs

Find ORFs

Dot Plots Dot Plots

ORF	*Fn* n	*Fn* coverage	*Fn* prob	*FnPg* n	*FnPg* coverage	*FnPg* prob	*FnSg* n	*FnSg* coverage	*FnSg* prob	*FnPgSg* n	*FnPgSg* coverage	*FnPgSg* prob	Description
FN1864	7	17.7%	1.000	3	8.4%	0.999	3	10.5%	0.987	12	20.5%	1.000	AAL93963.1| DNA mismatch repair protein mutS
	4	10.1%	1.000	4	10.3%	1.000	3	8.8%	0.999	6	10.5%	1.000	
FN1865	3	11.2%	0.993	1	7.8%	0.425	1	4.4%	0.452	3	11.2%	0.999	AAL93964.1| Hypothetical protein
	2	4.7%	0.999	1	2.5%	0.362	0	0.0%	0.000	1	2.8%	0.379	
FN1866	52	83.1%	1.000	53	80.0%	1.000	43	83.3%	1.000	59	83.8%	1.000	AAL93965.1| Lysine 2,3-aminomutase
	51	81.6%	1.000	48	84.7%	1.000	44	80.0%	1.000	57	84.2%	1.000	
FN1867	34	61.2%	1.000	27	59.4%	1.000	27	69.9%	1.000	29	58.8%	1.000	AAL93966.1| Zn-dependent alcohol dehydrogenases and related dehydrogenases
	31	62.3%	1.000	28	65.2%	1.000	22	58.8%	1.000	29	61.2%	1.000	
FN1868	18	63.6%	1.000	14	43.8%	1.000	11	54.0%	1.000	16	54.8%	1.000	AAL93967.1| Hypothetical cytosolic protein
	17	63.6%	1.000	12	52.2%	1.000	9	36.8%	1.000	15	54.8%	1.000	
FN1869	9	62.5%	1.000	9	64.1%	1.000	7	62.5%	1.000	8	38.3%	1.000	AAL93968.1| Hypothetical protein
	8	62.5%	1.000	7	62.5%	1.000	7	62.5%	1.000	9	38.3%	1.000	
FN1870	1	6.6%	0.981	1	6.6%	0.983	1	8.3%	0.536	0	0.0%	0.000	AAL93969.1| unknown
	4	19.4%	1.000	0	0.0%	0.000	1	8.3%	0.492	3	14.0%	0.990	
FN1871	3	28.6%	1.000	0	0.0%	0.000	0	0.0%		0	0.0%	0.000	AAL93970.1| unknown
	3	28.6%	1.000	1	13.1%	0.622	0	0.0%		1	15.5%	0.944	
FN1872	3	22.6%	1.000	0	0.0%	0.000	2	20.2%	0.967	1	8.3%	0.433	AAL93971.1| unknown
	3	22.6%	1.000	1	11.9%	0.982	3	20.2%	0.999	0	0.0%	0.000	
FN1873	8	54.5%	1.000	5	39.3%	1.000	4	45.5%	1.000	6	45.5%	1.000	AAL93972.1| Bis(5'-nucleosyl)-tetraphosphatase
	8	76.8%	1.000	6	45.5%	1.000	6	45.5%	1.000	6	45.5%	1.000	
FN1874	8	40.3%	1.000	4	31.5%	1.000	4	26.2%	1.000	6	26.2%	1.000	AAL93973.1| Ribose 5-phosphate isomerase
	8	40.3%	1.000	6	40.3%	1.000	5	40.3%	1.000	6	40.3%	1.000	
FN1875	8	47.0%	1.000	5	35.4%	1.000	9	61.6%	1.000	10	47.0%	1.000	AAL93974.1| Peptidyl-prolyl cis-trans isomerase
	8	47.0%	1.000	10	61.6%	1.000	9	42.7%	1.000	10	47.0%	1.000	
FN1877	0	0.0%		1	3.8%	0.259	2	7.5%	0.982	3	15.6%	0.850	AAL93976.1| Guanine-hypoxanthine permease
	0	0.0%		4	16.5%	0.998	1	4.1%	0.410	1	7.0%	0.347	
FN1878	4	32.3%	1.000	1	9.1%	0.429	1	17.2%	0.985	3	18.2%	1.000	AAL93977.1| unknown
	4	32.3%	1.000	1	7.1%	0.445	1	7.1%	0.952	2	18.2%	0.999	
FN1879	2	18.9%	0.999	1	10.0%	0.984	1	7.8%	0.554	5	27.8%	1.000	AAL93978.1| SSU ribosomal protein S20P
	3	18.9%	1.000	1	10.0%	0.844	0	0.0%	0.000	5	27.8%	1.000	
FN1880	11	34.4%	1.000	10	52.1%	1.000	19	50.0%	1.000	9	38.5%	1.000	AAL93979.1| Oxygen-insensitive NAD(P)H nitroreductase
	13	44.8%	1.000	22	53.1%	1.000	17	49.0%	1.000	10	29.2%	1.000	

Show detected proteins only
Show all proteins
Filter by category:
GO: amino acid transport

Proteins found:
1424

Enter (or paste) list of ORFs
Find ORFs

Dot Plots Dot Plots

ORF	Fn n	Fn coverage	Fn prob	FnPg n	FnPg coverage	FnPg prob	FnSg n	FnSg coverage	FnSg prob	FnPgSg n	FnPgSg coverage	FnPgSg prob	Description
FN1881	2	26.4%	0.524	0	0.0%	0.000	3	17.8%	1.000	3	30.2%	1.000	AAL93980.1| Esterase
	0	0.0%	0.000	3	17.8%	1.000	3	17.8%	1.000	2	10.9%	1.000	
FN1884	5	72.9%	1.000	6	72.9%	1.000	0	0.0%		7	72.9%	1.000	AAL93983.1| unknown
	6	72.9%	1.000	1	18.6%	0.982	0	0.0%		6	72.9%	1.000	
FN1890	3	46.6%	1.000	4	46.6%	1.000	0	0.0%		4	46.6%	1.000	AAL93989.1| Hypothetical protein
	3	46.6%	1.000	1	13.8%	0.320	0	0.0%		3	46.6%	1.000	
FN1891	5	14.6%	1.000	4	9.6%	1.000	9	27.6%	1.000	6	19.9%	1.000	AAL93990.1| Glycerophosphoryl diester phosphodiesterase
	4	14.6%	1.000	12	29.9%	1.000	13	44.1%	1.000	6	18.8%	1.000	
FN1893	60	55.0%	1.000	29	25.2%	1.000	53	41.2%	1.000	50	39.5%	1.000	AAL93991.1| Fusobacterium outer membrane protein family
	59	52.1%	1.000	59	47.5%	1.000	48	39.2%	1.000	42	34.1%	1.000	
FN1895	2	16.5%	0.998	0	0.0%	0.000	1	21.1%	0.952	3	29.4%	1.000	AAL93994.1| Hypothetical protein
	2	16.5%	0.996	0	0.0%	0.000	0	0.0%	0.000	1	8.3%	0.626	
FN1898	19	38.3%	1.000	4	9.7%	1.000	16	34.2%	1.000	14	27.1%	1.000	AAL93997.1| Sugar transport ATP-binding protein
	15	33.4%	1.000	17	42.7%	1.000	22	49.7%	1.000	14	34.7%	1.000	
FN1899	17	50.2%	1.000	14	47.1%	1.000	17	64.7%	1.000	13	36.5%	1.000	AAL93998.1| Hypothetical lipoprotein
	18	61.3%	1.000	21	71.4%	1.000	18	61.3%	1.000	12	26.9%	1.000	
FN1901	1	8.3%	0.533	1	5.1%	0.729	2	16.6%	0.959	1	8.3%	0.989	AAL94000.1| Transcription regulator, CRP family
	1	8.3%	0.792	1	11.5%	0.730	4	29.5%	1.000	0	0.0%	0.000	
FN1902	7	39.7%	1.000	6	46.6%	1.000	8	47.1%	1.000	7	35.6%	1.000	AAL94001.1| Deoxycytidylate deaminase
	9	55.7%	1.000	11	67.8%	1.000	7	38.5%	1.000	6	29.3%	1.000	
FN1903	16	26.5%	1.000	7	11.1%	1.000	2	6.0%	0.981	31	48.3%	1.000	AAL94002.1| Coenzyme A disulfide reductase/ disulfide bond regulator domain
	17	30.1%	1.000	3	7.0%	0.987	1	2.6%	0.985	28	40.7%	1.000	
FN1905	0	0.0%	0.000	2	3.4%	0.598	2	2.0%	0.704	3	2.4%	0.822	AAL94004.1| outer membrane protein
	3	2.9%	0.920	1	1.3%	0.526	3	2.2%	0.939	1	0.5%	0.652	
FN1906	31	52.7%	1.000	26	55.6%	1.000	40	65.3%	1.000	41	63.4%	1.000	AAL94005.1| Cytosol aminopeptidase
	36	56.1%	1.000	51	73.8%	1.000	43	65.9%	1.000	31	50.4%	1.000	
FN1908	26	63.3%	1.000	24	55.5%	1.000	36	70.3%	1.000	22	55.2%	1.000	AAL94007.1| Glycerophosphoryl diester phosphodiesterase
	26	63.6%	1.000	34	68.3%	1.000	35	68.3%	1.000	21	59.1%	1.000	
FN1909	16	48.8%	1.000	15	47.6%	1.000	16	44.9%	1.000	13	41.9%	1.000	AAL94008.1| UDP-3-O-[3-hydroxymyristoyl] glucosamine N-acyltransferase
	20	57.5%	1.000	14	40.7%	1.000	14	42.8%	1.000	14	53.6%	1.000	
FN1910	19	38.9%	1.000	16	39.5%	1.000	10	31.8%	1.000	22	44.6%	1.000	AAL94009.1| periplasmic protein
	22	49.7%	1.000	13	42.7%	1.000	10	31.8%	1.000	25	55.4%	1.000	

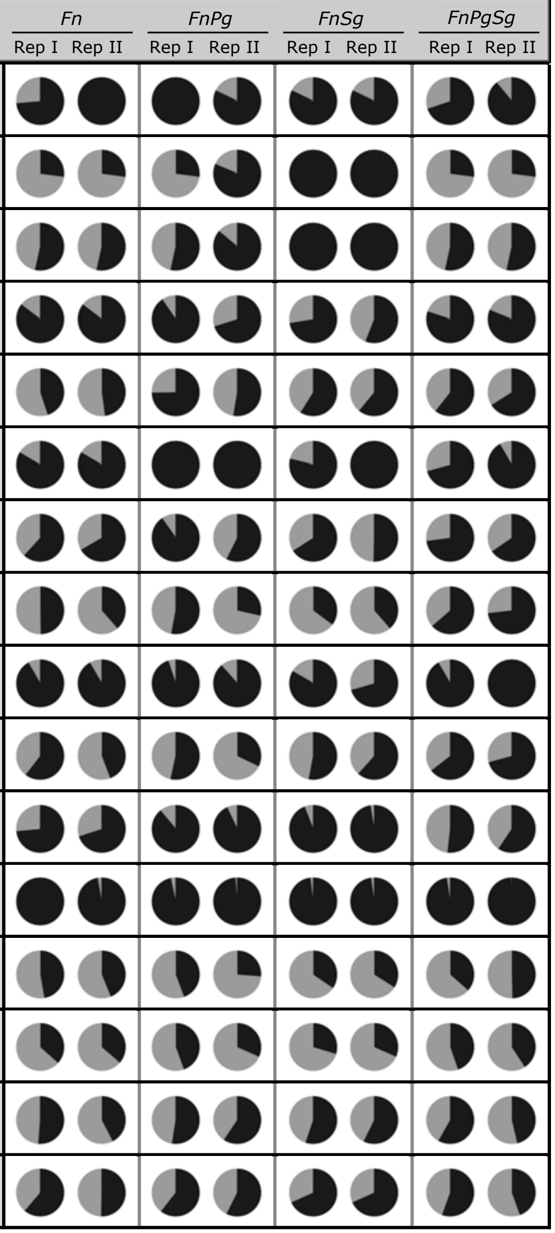

Show detected proteins only
Show all proteins
Filter by category:
GO: amino acid transport

Proteins found: 1424

Enter (or paste) list of ORFs

Find ORFs

Dot Plots Dot Plots

ORF	*Fn* n	*Fn* coverage	*Fn* prob	*FnPg* n	*FnPg* coverage	*FnPg* prob	*FnSg* n	*FnSg* coverage	*FnSg* prob	*FnPgSg* n	*FnPgSg* coverage	*FnPgSg* prob	Description
**FN1911**	67	66.5%	1.000	42	51.5%	1.000	66	71.4%	1.000	67	65.5%	1.000	AAL94010.1| Outer membrane protein
	66	66.4%	1.000	72	71.8%	1.000	58	69.2%	1.000	70	67.0%	1.000	
**FN1912**	11	11.1%	1.000	4	4.0%	1.000	7	7.6%	0.999	2	1.4%	0.993	AAL94011.1| Hypothetical protein
	10	10.6%	1.000	8	8.1%	1.000	7	5.3%	1.000	4	4.1%	1.000	
**FN1913**	18	40.2%	1.000	8	21.3%	1.000	18	36.8%	1.000	14	28.5%	1.000	AAL94012.1| hydrolase (HD superfamily)
	19	35.8%	1.000	19	36.6%	1.000	22	39.2%	1.000	17	36.2%	1.000	
**FN1914**	4	40.9%	1.000	5	61.7%	1.000	6	61.7%	1.000	3	39.1%	1.000	AAL94013.1| Anti-sigma F factor antagonist
	3	39.1%	1.000	5	45.2%	1.000	5	61.7%	1.000	3	39.1%	1.000	
**FN1917**	2	7.6%	0.998	2	4.0%	1.000	3	7.6%	1.000	2	6.6%	0.833	AAL94016.1| tRNA delta(2)-isopentenylpyrophosphate transferase
	3	8.9%	0.995	4	10.2%	1.000	2	4.0%	1.000	2	6.3%	0.972	
**FN1918**	15	32.5%	1.000	10	31.3%	1.000	19	44.4%	1.000	18	44.4%	1.000	AAL94017.1| SPO0B-associated GTP-binding protein
	14	32.0%	1.000	19	46.7%	1.000	21	48.1%	1.000	11	29.2%	1.000	
**FN1919**	11	43.8%	1.000	3	12.4%	1.000	4	16.9%	1.000	6	23.3%	1.000	AAL94018.1| Methyltransferase
	12	43.8%	1.000	4	22.1%	0.999	6	26.5%	1.000	3	20.5%	1.000	
**FN1920**	0	0.0%	0.000	3	13.1%	0.997	1	5.0%	0.659	0	0.0%		AAL94019.1| tRNA (5-methylaminomethyl-2-thiouridylate) -methyltransferase
	1	5.5%	0.944	1	5.5%	0.597	3	14.0%	0.996	0	0.0%		
**FN1922**	7	13.7%	1.000	4	6.8%	1.000	4	7.7%	1.000	2	2.7%	0.979	AAL94021.1| Hypothetical protein
	5	11.0%	1.000	8	14.0%	1.000	6	10.3%	1.000	5	8.8%	1.000	
**FN1923**	4	6.9%	1.000	0	0.0%		0	0.0%	0.000	1	2.4%	0.851	AAL94022.1| Adenine-specific methyltransferase
	1	2.8%	0.472	0	0.0%		3	7.3%	0.996	1	5.2%	0.406	
**FN1925**	0	0.0%		0	0.0%	0.000	0	0.0%		0	0.0%		AAL94024.1| Arsenical pump membrane protein
	0	0.0%		3	9.9%	0.979	0	0.0%		0	0.0%		
**FN1926**	12	36.3%	1.000	8	35.4%	1.000	13	46.6%	1.000	13	48.6%	1.000	AAL94025.1| Nitrogen regulatory IIA protein
	13	41.5%	1.000	18	51.4%	1.000	15	46.3%	1.000	14	47.3%	1.000	
**FN1927**	22	26.4%	1.000	23	30.1%	1.000	45	56.6%	1.000	20	28.0%	1.000	AAL94026.1| DEGV protein
	22	26.7%	1.000	49	60.8%	1.000	46	57.9%	1.000	23	28.4%	1.000	
**FN1928**	2	17.4%	1.000	1	8.2%	0.967	7	31.0%	1.000	5	31.5%	1.000	AAL94027.1| Transcriptional regulator, MerR family
	4	27.7%	1.000	8	38.6%	1.000	8	42.4%	1.000	4	22.3%	1.000	
**FN1929**	8	16.9%	1.000	4	17.9%	1.000	11	33.1%	1.000	10	26.6%	1.000	AAL94028.1| Competence-damage protein cinA
	9	21.1%	1.000	15	36.8%	1.000	17	43.3%	1.000	9	32.3%	1.000	
**FN1931**	3	3.8%	0.945	2	2.9%	0.927	1	2.1%	0.524	1	1.2%	0.381	AAL94030.1| Protease
	3	4.7%	0.980	4	7.8%	0.999	9	15.7%	1.000	0	0.0%	0.000	

Show detected proteins only
Show all proteins
Filter by category:
GO: amino acid transport

Proteins found: 1424

Enter (or paste) list of ORFs

Find ORFs

Dot Plots Dot Plots

ORF	*Fn* n	*Fn* coverage	*Fn* prob	*FnPg* n	*FnPg* coverage	*FnPg* prob	*FnSg* n	*FnSg* coverage	*FnSg* prob	*FnPgSg* n	*FnPgSg* coverage	*FnPgSg* prob	Description
**FN1933**	6	20.7%	1.000	3	7.2%	1.000	4	14.7%	1.000	9	27.1%	1.000	AAL94032.1| Hypothetical protein
	7	25.1%	1.000	4	14.4%	0.999	8	26.5%	1.000	5	16.4%	1.000	
**FN1935**	4	17.7%	1.000	2	10.5%	0.990	4	19.8%	1.000	3	15.6%	0.997	AAL94034.1| Adenine-specific methyltransferase
	5	23.1%	1.000	3	16.8%	1.000	4	20.7%	1.000	0	0.0%	0.000	
**FN1939**	7	26.3%	1.000	1	3.1%	0.547	3	9.9%	1.000	5	17.3%	1.000	AAL94038.1| Hypothetical protein
	7	24.1%	1.000	6	19.8%	1.000	5	17.0%	1.000	5	16.7%	1.000	
**FN1941**	27	33.7%	1.000	34	43.9%	1.000	59	60.2%	1.000	30	33.4%	1.000	AAL94040.1| ClpB protein
	27	39.8%	1.000	61	66.4%	1.000	66	67.6%	1.000	33	40.6%	1.000	
**FN1942**	3	17.9%	0.987	5	28.4%	1.000	2	11.8%	0.991	0	0.0%		AAL94041.1| putative DNA-binding protein
	0	0.0%	0.000	0	0.0%	0.000	3	17.5%	0.999	0	0.0%		
**FN1943**	62	71.2%	1.000	78	76.9%	1.000	68	86.1%	1.000	56	72.3%	1.000	AAL94042.1| Tryptophanase
	60	72.3%	1.000	73	80.7%	1.000	69	80.9%	1.000	53	63.5%	1.000	
**FN1948**	1	10.9%	0.962	2	22.7%	0.992	5	42.0%	1.000	0	0.0%	0.000	AAL94044.1| Hypothetical protein
	0	0.0%	0.000	5	39.5%	1.000	5	47.1%	1.000	1	12.6%	0.810	
**FN1949**	5	9.7%	1.000	6	14.1%	1.000	13	29.4%	1.000	2	8.7%	0.759	AAL94045.1| Xaa-Pro dipeptidase
	4	6.7%	1.000	14	24.7%	1.000	14	30.5%	1.000	3	8.2%	0.999	
**FN1950**	23	25.3%	1.000	0	0.0%	0.000	0	0.0%	0.000	2	10.5%	0.905	AAL94046.1| Serine protease
	19	21.2%	1.000	1	21.4%	0.991	1	18.7%	0.961	3	13.5%	0.991	
**FN1951**	3	10.9%	0.986	1	20.0%	0.630	1	5.1%	0.282	0	0.0%		AAL94047.1| ATPase associated with chromosome architecture/replication
	3	20.6%	0.999	2	10.9%	0.778	3	17.7%	0.993	0	0.0%		
**FN1956**	1	10.5%	0.979	1	11.3%	0.652	5	20.2%	1.000	1	10.5%	0.891	AAL94052.1| Hypothetical protein
	1	10.5%	0.982	5	20.2%	1.000	5	33.9%	1.000	2	19.4%	0.990	
**FN1964**	8	13.7%	1.000	8	16.5%	1.000	6	14.8%	1.000	9	16.5%	1.000	AAL94054.1| O-linked GLCNAC transferase
	11	21.9%	1.000	5	9.7%	1.000	9	17.9%	1.000	6	10.2%	1.000	
**FN1965**	12	31.9%	1.000	10	22.0%	1.000	9	37.1%	1.000	13	42.0%	1.000	AAL94055.1| Tetratricopeptide repeat family protein
	12	31.6%	1.000	15	44.6%	1.000	13	44.6%	1.000	10	35.7%	1.000	
**FN1966**	7	51.4%	1.000	4	35.7%	1.000	6	51.4%	1.000	7	51.4%	1.000	AAL94056.1| Hypothetical protein
	7	51.4%	1.000	5	37.1%	1.000	6	51.4%	1.000	6	50.0%	1.000	
**FN1970**	4	19.6%	0.999	0	0.0%	0.000	0	0.0%	0.000	3	8.6%	0.928	AAL94060.1| Hemin-binding periplasmic protein hmuT precursor
	3	8.6%	0.871	2	8.9%	0.732	1	3.2%	0.343	1	3.2%	0.886	
**FN1971**	8	18.9%	1.000	1	2.1%	0.534	3	7.6%	1.000	11	19.9%	1.000	AAL94061.1| Hemin receptor
	11	16.7%	1.000	13	20.4%	1.000	4	9.4%	1.000	12	22.2%	1.000	

Fn Rep I Rep II | *FnPg* Rep I Rep II | *FnSg* Rep I Rep II | *FnPgSg* Rep I Rep II

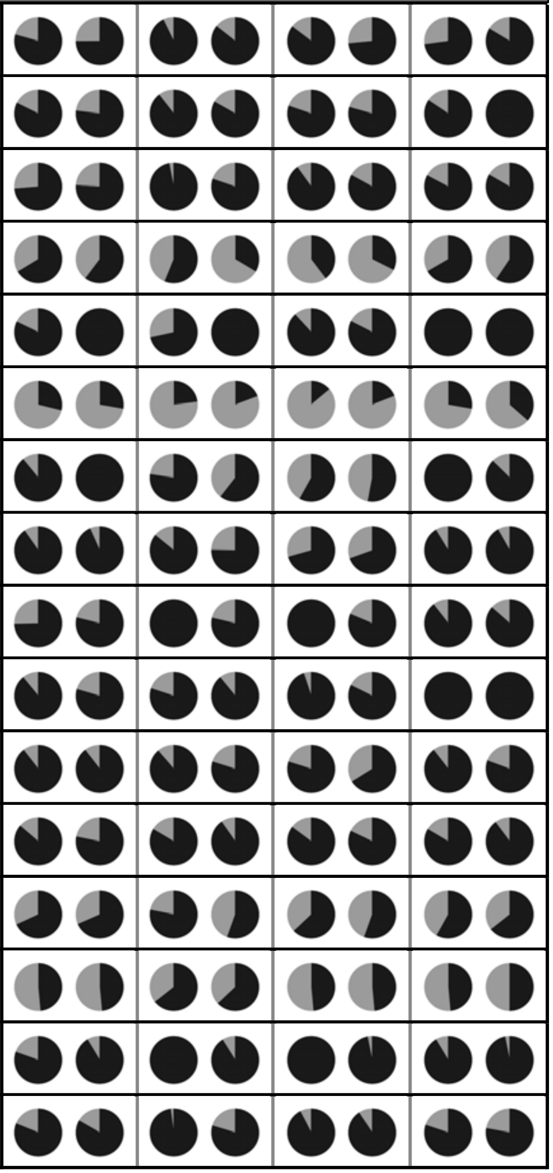

Show detected proteins only
Show all proteins
Filter by category:
GO: amino acid transport

Proteins found: 1424

Enter (or paste) list of ORFs

Find ORFs

Dot Plots Dot Plots

ORF	*Fn* n	*Fn* coverage	*Fn* prob	*FnPg* n	*FnPg* coverage	*FnPg* prob	*FnSg* n	*FnSg* coverage	*FnSg* prob	*FnPgSg* n	*FnPgSg* coverage	*FnPgSg* prob	Description
FN1972	5	36.9%	1.000	2	13.1%	0.993	6	60.7%	1.000	3	38.5%	1.000	AAL94062.1| unknown
	5	32.0%	1.000	7	45.1%	1.000	7	62.3%	1.000	4	45.1%	1.000	
FN1973	5	30.5%	1.000	8	41.4%	1.000	4	27.3%	1.000	11	49.2%	1.000	AAL94063.1| Translation initiation inhibitor
	5	35.9%	1.000	4	27.3%	1.000	3	21.9%	1.000	10	41.4%	1.000	
FN1974	3	2.3%	1.000	1	3.1%	0.375	1	1.3%	0.974	2	2.3%	0.999	AAL94064.1| DNA/RNA helicase (DEAD/DEAH BOX family)
	7	8.3%	1.000	2	3.7%	0.845	5	7.2%	1.000	1	1.5%	0.878	
FN1975	27	54.0%	1.000	11	24.4%	1.000	23	54.4%	1.000	23	48.9%	1.000	AAL94065.1| ATP-dependent RNA helicase
	27	55.9%	1.000	21	45.1%	1.000	22	50.6%	1.000	26	53.4%	1.000	
FN1976	10	45.8%	1.000	5	26.1%	0.998	14	65.8%	1.000	8	34.8%	1.000	AAL94066.1| 4-amino-4-deoxychorismate lyase
	12	42.3%	1.000	14	55.8%	1.000	13	58.7%	1.000	12	43.5%	1.000	
FN1977	4	8.5%	1.000	4	15.2%	1.000	4	11.8%	1.000	4	8.3%	0.995	AAL94067.1| Cell cycle protein MesJ
	4	10.3%	0.998	2	6.5%	0.906	8	22.5%	1.000	1	3.3%	0.444	
FN1978	30	38.1%	1.000	7	14.6%	1.000	21	32.1%	1.000	27	34.5%	1.000	AAL94068.1| Cell division protein ftsH
	30	39.5%	1.000	22	31.9%	1.000	21	32.4%	1.000	26	35.9%	1.000	
FN1979	7	42.4%	1.000	3	42.4%	1.000	1	10.6%	0.984	7	49.4%	1.000	AAL94069.1| SSU ribosomal protein S15P
	4	41.2%	1.000	3	41.2%	1.000	1	10.6%	0.983	9	49.4%	1.000	
FN1980	0	0.0%		0	0.0%	0.000	3	10.0%	0.983	2	8.7%	0.994	AAL94070.1| Transporter
	0	0.0%		2	6.5%	0.989	2	10.0%	0.990	3	11.1%	0.988	
FN1983	24	66.5%	1.000	15	48.9%	1.000	16	76.6%	1.000	28	66.5%	1.000	AAL94073.1| Alkyl hydroperoxide reductase C22 protein
	23	62.2%	1.000	17	60.6%	1.000	14	76.6%	1.000	27	66.5%	1.000	
FN1984	35	57.6%	1.000	27	53.9%	1.000	25	54.5%	1.000	36	59.6%	1.000	AAL94074.1| Thioredoxin reductase
	33	55.0%	1.000	34	56.1%	1.000	27	50.8%	1.000	34	60.9%	1.000	
FN1985	15	39.4%	1.000	5	14.1%	1.000	14	37.4%	1.000	12	37.0%	1.000	AAL94075.1| Inner membrane protein
	14	36.1%	1.000	19	48.2%	1.000	14	36.3%	1.000	14	42.3%	1.000	
FN1986	40	81.5%	1.000	22	51.2%	1.000	28	73.2%	1.000	26	63.4%	1.000	AAL94076.1| Hypothetical protein
	40	78.6%	1.000	28	77.1%	1.000	21	58.3%	1.000	27	67.3%	1.000	
FN1987	4	15.7%	1.000	4	15.3%	1.000	1	5.6%	0.827	5	14.8%	1.000	AAL94077.1| Transcriptional regulator, GntR family
	3	14.8%	0.995	1	4.6%	0.452	4	14.4%	1.000	3	10.2%	1.000	
FN1988	18	43.7%	1.000	22	47.6%	1.000	54	75.0%	1.000	20	46.7%	1.000	AAL94078.1| Tyrosine phenol-lyase
	17	39.3%	1.000	44	71.3%	1.000	43	72.0%	1.000	21	48.3%	1.000	
FN1989	0	0.0%		0	0.0%	0.000	3	5.5%	1.000	0	0.0%		AAL94079.1| Sodium-dependent tyrosine transporter
	0	0.0%		4	5.5%	1.000	4	5.5%	1.000	0	0.0%		

Fn Rep I Rep II | *FnPg* Rep I Rep II | *FnSg* Rep I Rep II | *FnPgSg* Rep I Rep II

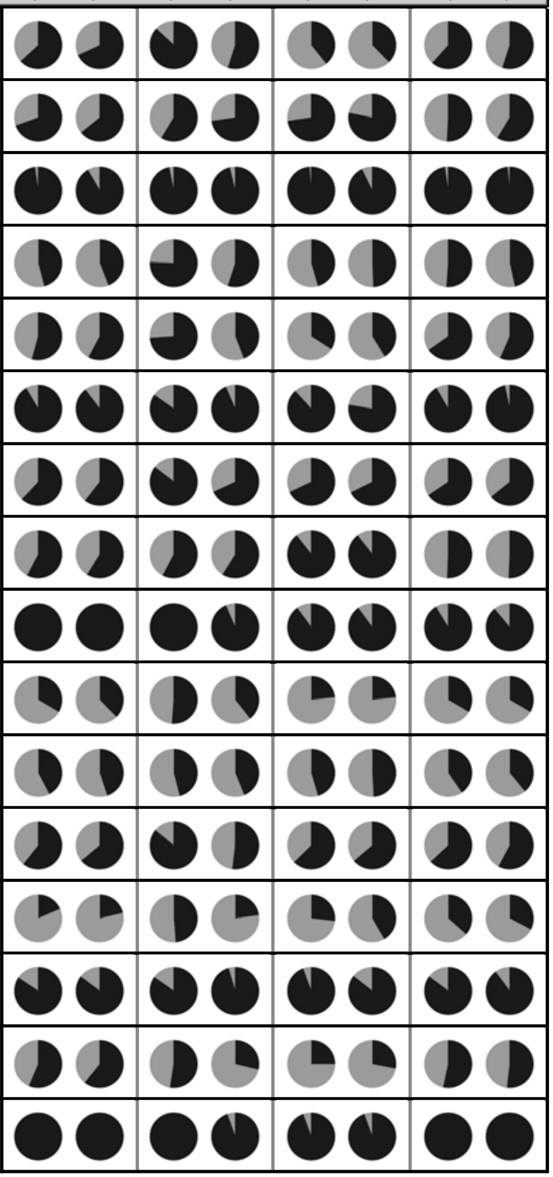

Show detected proteins only
Show all proteins
Filter by category:
GO: amino acid transport

Proteins found: 1424

Enter (or paste) list of ORFs

Find ORFs

Dot Plots Dot Plots

ORF	*Fn* n	*Fn* coverage	*Fn* prob	*FnPg* n	*FnPg* coverage	*FnPg* prob	*FnSg* n	*FnSg* coverage	*FnSg* prob	*FnPgSg* n	*FnPgSg* coverage	*FnPgSg* prob	Description
FN1991	38	74.0%	1.000	29	59.2%	1.000	35	58.3%	1.000	31	62.6%	1.000	AAL94081.1| Glucosamine-1-phosphate acetyltransferase
	35	72.2%	1.000	35	74.7%	1.000	34	66.6%	1.000	29	62.6%	1.000	
FN1992	22	69.3%	1.000	20	60.8%	1.000	23	70.6%	1.000	26	77.2%	1.000	AAL94082.1| Ribose-phosphate pyrophosphokinase
	21	51.9%	1.000	23	69.6%	1.000	22	61.7%	1.000	22	59.2%	1.000	
FN1993	3	20.3%	1.000	0	0.0%	0.000	3	24.4%	0.860	2	14.7%	0.735	AAL94083.1| SUA5 protein
	1	7.4%	0.885	5	27.6%	1.000	4	24.9%	1.000	4	27.2%	1.000	
FN1994	7	34.7%	1.000	6	27.8%	1.000	7	40.3%	1.000	9	50.7%	1.000	AAL94084.1| Hypothetical protein
	7	34.7%	1.000	7	50.0%	1.000	4	27.1%	0.999	6	45.8%	1.000	
FN1995	4	14.3%	1.000	1	5.5%	0.388	2	6.3%	0.857	5	16.9%	1.000	AAL94085.1| Hypothetical protein
	5	21.5%	1.000	4	19.0%	0.983	2	5.9%	0.921	4	13.9%	1.000	
FN2001	3	25.3%	1.000	0	0.0%	0.000	5	31.7%	1.000	2	12.4%	0.997	AAL94091.1| Hypothetical protein
	3	18.3%	1.000	5	30.6%	1.000	6	32.3%	1.000	2	12.4%	0.998	
FN2002	0	0.0%	0.000	0	0.0%	0.000	0	0.0%	0.000	1	3.5%	0.564	AAL94092.1| Permease
	1	6.0%	0.362	2	3.5%	0.490	3	12.8%	0.833	0	0.0%	0.000	
FN2007	8	54.3%	1.000	4	13.6%	1.000	4	18.6%	1.000	4	18.6%	1.000	AAL94097.1| Glutathione peroxidase
	6	44.7%	1.000	3	18.1%	1.000	3	18.6%	1.000	5	31.7%	1.000	
FN2008	1	9.6%	0.981	0	0.0%	0.000	3	28.3%	1.000	2	13.3%	0.882	AAL94098.1| Glycine betaine transport ATP-binding protein
	3	16.2%	0.998	6	26.7%	1.000	2	15.4%	0.999	1	9.6%	0.962	
FN2009	1	2.7%	0.958	1	7.0%	0.471	4	17.5%	1.000	2	9.2%	0.824	AAL94099.1| Glycine betaine transport system permease protein
	0	0.0%	0.000	5	20.5%	1.000	6	18.1%	1.000	0	0.0%	0.000	
FN2011	37	35.2%	1.000	15	21.6%	1.000	16	21.1%	1.000	27	30.7%	1.000	AAL94101.1| Valyl-tRNA synthetase
	36	35.6%	1.000	23	26.5%	1.000	31	37.3%	1.000	30	34.4%	1.000	
FN2013	3	19.1%	0.793	2	6.2%	0.999	4	16.0%	1.000	1	6.2%	0.954	AAL94103.1| GTP-binding protein
	1	8.8%	0.374	4	20.6%	0.942	3	13.4%	1.000	1	6.2%	0.421	
FN2014	37	40.5%	1.000	18	27.6%	1.000	25	36.3%	1.000	36	43.1%	1.000	AAL94104.1| ATP-dependent protease La
	36	43.0%	1.000	34	43.9%	1.000	33	40.2%	1.000	35	42.4%	1.000	
FN2015	26	58.9%	1.000	14	23.2%	1.000	27	64.5%	1.000	20	43.7%	1.000	AAL94105.1| ATP-dependent clp protease ATP-binding subunit clpX
	27	60.3%	1.000	26	65.5%	1.000	23	38.8%	1.000	22	52.2%	1.000	
FN2016	7	35.2%	1.000	1	6.7%	0.342	5	23.3%	1.000	7	32.6%	1.000	AAL94106.1| ATP-dependent Clp protease proteolytic subunit
	8	38.9%	1.000	7	27.5%	1.000	3	14.5%	1.000	8	32.1%	1.000	
FN2017	49	69.5%	1.000	41	61.3%	1.000	37	69.5%	1.000	55	67.1%	1.000	AAL94107.1| Trigger factor, ppiase
	54	68.8%	1.000	48	73.2%	1.000	41	72.7%	1.000	51	66.7%	1.000	

Show detected proteins only
Show all proteins
Filter by category:
GO: amino acid transport

Proteins found: 1424

Enter (or paste) list of ORFs

Find ORFs

Dot Plots Dot Plots

ORF	*Fn* n	*Fn* coverage	*Fn* prob	*FnPg* n	*FnPg* coverage	*FnPg* prob	*FnSg* n	*FnSg* coverage	*FnSg* prob	*FnPgSg* n	*FnPgSg* coverage	*FnPgSg* prob	Description	*Fn* Rep I	*Fn* Rep II	*FnPg* Rep I	*FnPg* Rep II	*FnSg* Rep I	*FnSg* Rep II	*FnPgSg* Rep I	*FnPgSg* Rep II
**FN2018**	12	25.0%	1.000	4	10.3%	1.000	4	11.3%	1.000	11	23.9%	1.000	AAL94108.1| Single-stranded-DNA-specific exonuclease recJ								
	14	24.8%	1.000	4	11.3%	1.000	9	24.5%	1.000	10	21.6%	1.000									
**FN2019**	6	47.5%	1.000	9	54.2%	1.000	2	26.7%	0.958	5	35.8%	1.000	AAL94109.1| Ribosome-binding factor A								
	6	44.2%	1.000	1	12.5%	0.967	4	30.0%	1.000	6	48.3%	1.000									
**FN2020**	43	54.0%	1.000	32	41.0%	1.000	39	52.8%	1.000	39	47.4%	1.000	AAL94110.1| Bacterial Protein Translation Initiation Factor 2 (IF -2)								
	44	52.0%	1.000	49	63.5%	1.000	48	59.4%	1.000	38	45.3%	1.000									
**FN2022**	20	43.1%	1.000	15	39.2%	1.000	20	45.9%	1.000	18	42.3%	1.000	AAL94112.1| N utilization substance protein A								
	20	43.4%	1.000	18	48.7%	1.000	24	50.1%	1.000	18	42.6%	1.000									
**FN2023**	6	37.2%	1.000	5	17.9%	1.000	6	21.8%	1.000	5	26.3%	1.000	AAL94113.1| Hypothetical cytosolic protein								
	5	37.2%	1.000	4	17.3%	1.000	8	21.8%	1.000	4	24.4%	1.000									
**FN2030**	14	16.2%	1.000	7	10.4%	1.000	13	17.7%	1.000	22	18.9%	1.000	AAL94115.1| Inorganic pyrophosphatase								
	14	14.5%	1.000	11	14.3%	1.000	13	15.9%	1.000	21	21.8%	1.000									
**FN2031**	3	12.5%	1.000	3	12.2%	1.000	2	13.1%	0.997	7	18.8%	1.000	AAL94116.1| Thiamine biosynthesis lipoprotein apbE								
	5	22.8%	1.000	2	4.4%	0.977	3	16.6%	1.000	6	17.2%	1.000									
**FN2033**	6	37.8%	1.000	5	37.8%	1.000	1	12.4%	0.985	1	9.2%	0.512	AAL94118.1| Guanylate kinase								
	6	37.3%	1.000	1	12.4%	0.804	4	36.8%	1.000	2	14.1%	0.761									
**FN2034**	6	16.4%	1.000	5	19.2%	1.000	7	21.9%	1.000	11	31.8%	1.000	AAL94119.1| Protein yicC								
	9	22.9%	1.000	8	31.5%	1.000	9	24.0%	1.000	11	34.9%	1.000									
**FN2035**	81	54.1%	1.000	57	44.0%	1.000	77	54.3%	1.000	71	44.9%	1.000	AAL94120.1| DNA-directed RNA polymerase beta' chain								
	71	47.6%	1.000	79	52.7%	1.000	71	55.7%	1.000	70	45.0%	1.000									
**FN2036**	70	51.4%	1.000	36	35.1%	1.000	62	55.4%	1.000	55	46.0%	1.000	AAL94121.1| DNA-directed RNA polymerase beta chain								
	66	47.9%	1.000	57	48.8%	1.000	67	54.5%	1.000	61	52.4%	1.000									
**FN2037**	13	72.1%	1.000	8	57.4%	1.000	8	65.6%	1.000	15	82.0%	1.000	AAL94122.1| LSU ribosomal protein L12P (L7/L12)								
	11	74.6%	1.000	9	65.6%	1.000	10	75.4%	1.000	14	72.1%	1.000									
**FN2038**	25	71.8%	1.000	17	60.6%	1.000	16	65.9%	1.000	26	73.5%	1.000	AAL94123.1| LSU ribosomal protein L10P								
	23	70.6%	1.000	19	54.1%	1.000	20	71.8%	1.000	26	70.6%	1.000									
**FN2039**	22	49.4%	1.000	16	38.3%	1.000	15	51.1%	1.000	21	46.4%	1.000	AAL94124.1| LSU ribosomal protein L1P								
	21	41.7%	1.000	13	46.0%	1.000	15	51.5%	1.000	24	44.7%	1.000									
**FN2040**	7	32.6%	1.000	3	25.5%	1.000	3	22.7%	1.000	7	32.6%	1.000	AAL94125.1| LSU ribosomal protein L11P								
	6	32.6%	1.000	4	32.6%	1.000	3	22.7%	1.000	6	32.6%	1.000									
**FN2041**	9	55.4%	1.000	12	59.6%	1.000	9	38.9%	1.000	9	49.2%	1.000	AAL94126.1| Transcription antitermination protein nusG								
	9	51.8%	1.000	10	46.6%	1.000	10	40.9%	1.000	9	49.2%	1.000									

Show detected proteins only
Show all proteins
Filter by category:
GO: amino acid transport

Proteins found:
1424

Enter (or paste) list of ORFs

Find ORFs

Dot Plots Dot Plots

ORF	*Fn* n	*Fn* coverage	*Fn* prob	*FnPg* n	*FnPg* coverage	*FnPg* prob	*FnSg* n	*FnSg* coverage	*FnSg* prob	*FnPgSg* n	*FnPgSg* coverage	*FnPgSg* prob	Description
**FN2045**	2	10.6%	0.995	1	13.4%	0.981	3	31.7%	1.000	1	9.2%	0.661	AAL94129.1| Ferric uptake regulation protein
	0	0.0%	0.000	3	26.1%	1.000	4	31.0%	1.000	0	0.0%	0.000	
**FN2046**	1	8.1%	0.975	1	9.4%	0.984	6	47.7%	1.000	0	0.0%	0.000	AAL94130.1| Acetyltransferase
	1	7.4%	0.967	6	32.9%	1.000	4	24.2%	1.000	1	8.1%	0.986	
**FN2047**	67	55.5%	1.000	10	11.3%	1.000	29	30.2%	1.000	27	24.8%	1.000	AAL94131.1| Fusobacterium outer membrane protein family
	65	48.8%	1.000	31	28.0%	1.000	32	33.6%	1.000	32	27.4%	1.000	
**FN2048**	15	70.2%		0	0.0%		9	70.2%		16	70.2%		AAL94132.1| Outer membrane protein
	13	70.2%		12	70.2%		9	70.2%		13	52.3%		
**FN2049**	9	54.9%		8	45.1%		7	48.8%		8	54.9%		AAL94133.1| unknown
	8	48.8%		7	54.9%		5	45.1%		7	45.1%		
**FN2050**	11	47.6%		6	42.9%		9	58.7%		12	53.2%		AAL94134.1| Hypothetical membrane-spanning protein
	10	46.8%		10	58.7%		7	53.2%		12	52.4%		
**FN2051**	13	57.0%		12	54.7%		10	58.7%		11	56.4%		AAL94135.1| unknown
	12	57.0%		10	58.7%		9	54.2%		10	56.4%		
**FN2052**	14	39.5%		9	39.5%		8	39.5%		14	39.5%		AAL94136.1| unknown
	13	39.5%		9	39.5%		10	39.5%		14	39.5%		
**FN2053**	7	13.9%	1.000	4	11.9%	1.000	6	13.9%	1.000	5	11.9%	1.000	AAL94137.1| Serine/threonine sodium symporter
	6	11.9%	1.000	6	13.9%	1.000	6	13.9%	1.000	6	11.9%	1.000	
**FN2054**	27	45.8%	1.000	18	43.5%	1.000	25	52.0%	1.000	28	51.8%	1.000	AAL94138.1| Glucose-6-phosphate isomerase
	28	47.8%	1.000	25	56.7%	1.000	20	46.4%	1.000	25	54.7%	1.000	
**FN2058**	69	46.8%	1.000	34	26.3%	1.000	36	26.0%	1.000	53	39.7%	1.000	AAL94142.1| Fusobacterium outer membrane protein family
	57	40.2%	1.000	61	42.7%	1.000	49	36.1%	1.000	54	41.5%	1.000	
**FN2059**	15	70.2%		0	0.0%		9	70.2%		16	70.2%		AAL94143.1| Outer membrane protein
	13	70.2%		12	70.2%		9	70.2%		13	52.3%		
**FN2060**	9	54.9%		8	45.1%		7	48.8%		8	54.9%		AAL94144.1| unknown
	8	48.8%		7	54.9%		5	45.1%		7	45.1%		
**FN2061**	11	47.6%		6	42.9%		9	58.7%		12	53.2%		AAL94145.1| Hypothetical membrane-spanning protein
	10	46.8%		10	58.7%		7	53.2%		12	52.4%		
**FN2062**	13	57.0%		12	54.7%		10	58.7%		11	56.4%		AAL94146.1| unknown
	12	57.0%		10	58.7%		9	54.2%		10	56.4%		
**FN2063**	14	39.5%		9	39.5%		8	39.5%		14	39.5%		AAL94147.1| unknown
	13	39.5%		9	39.5%		10	39.5%		14	39.5%		

Fn Rep I / Rep II, *FnPg* Rep I / Rep II, *FnSg* Rep I / Rep II, *FnPgSg* Rep I / Rep II

Show detected proteins only
Show all proteins
Filter by category:
GO: amino acid transport

Proteins found: 1424

Enter (or paste) list of ORFs

Find ORFs

Dot Plots Dot Plots

Fn Coverage

Fusobacterium nucleatum

Hackett Laboratory UW

Fn Summary Table | FnPg vs Fn | FnSg vs Fn | FnPgSg vs Fn | FnPgSg vs FnPg | FnSg vs FnPg | FnPgSg vs FnSg | Fn Coverage

ORF	*Fn* n	*Fn* coverage	*Fn* prob	*FnPg* n	*FnPg* coverage	*FnPg* prob	*FnSg* n	*FnSg* coverage	*FnSg* prob	*FnPgSg* n	*FnPgSg* coverage	*FnPgSg* prob	Description
**FN2067**	4	26.2%	1.000	0	0.0%	0.000	1	17.1%	0.940	5	29.3%	1.000	AAL94151.1| Thiol:disulfide interchange protein tlpA
	3	25.6%	1.000	2	24.4%	0.921	6	43.3%	1.000	6	29.3%	1.000	
**FN2068**	5	12.7%	1.000	5	14.6%	1.000	7	18.3%	1.000	4	9.4%	0.999	AAL94152.1| dGTP triphosphohydrolase
	4	9.7%	1.000	6	10.3%	1.000	13	26.5%	1.000	1	1.6%	0.492	
**FN2069**	0	0.0%	0.000	1	2.2%	0.420	0	0.0%	0.000	1	6.0%	0.782	AAL94153.1| Amino acid carrier protein alsT
	3	13.1%	0.668	1	3.1%	0.920	2	9.5%	0.747	0	0.0%	0.000	
**FN2070**	9	18.9%	1.000	1	3.5%	0.967	6	14.5%	1.000	3	8.4%	0.997	AAL94154.1| Cobyric acid synthase
	6	19.6%	1.000	7	21.8%	1.000	9	22.0%	1.000	2	4.7%	0.989	
**FN2073**	8	52.0%	1.000	5	27.1%	1.000	11	47.5%	1.000	4	20.9%	1.000	AAL94157.1| Adenine phosphoribosyltransferase
	7	40.7%	1.000	10	58.8%	1.000	12	53.1%	1.000	6	27.1%	1.000	
**FN2074**	0	0.0%	0.000	1	1.8%	0.984	1	1.5%	0.338	2	5.2%	0.886	AAL94158.1| BslIM
	1	2.4%	0.982	3	6.1%	0.856	2	2.5%	0.958	4	5.2%	0.957	
**FN2075**	14	35.9%	1.000	8	15.2%	1.000	4	19.4%	1.000	9	28.2%	1.000	AAL94159.1| Hypothetical protein
	14	37.9%	1.000	8	24.6%	1.000	10	24.6%	1.000	11	41.1%	1.000	
**FN2076**	1	14.5%	0.979	1	14.5%	0.776	0	0.0%		0	0.0%	0.000	AAL94160.1| MunI regulatory protein
	1	14.5%	0.967	0	0.0%	0.000	0	0.0%		3	40.8%	1.000	
**FN2078**	3	11.1%	0.983	1	2.9%	0.716	0	0.0%	0.000	1	2.9%	0.430	AAL94162.1| Transcriptional regulator, DeoR family
	1	2.9%	0.979	3	11.8%	0.999	3	12.1%	0.999	1	2.9%	0.967	
**FN2081**	6	21.7%	1.000	2	10.0%	0.819	0	0.0%		1	4.7%	0.635	AAL94165.1| ABC transporter substrate-binding protein
	6	27.4%	1.000	0	0.0%	0.000	0	0.0%		0	0.0%	0.000	
**FN2082**	54	64.7%	1.000	37	55.3%	1.000	43	67.3%	1.000	47	58.1%	1.000	AAL94166.1| Formate--tetrahydrofolate ligase
	42	64.7%	1.000	50	68.0%	1.000	43	69.1%	1.000	48	58.5%	1.000	
**FN2093**	8	60.3%	1.000	3	36.4%	1.000	4	16.6%	1.000	8	42.4%	1.000	AAL94177.1| General secretion pathway protein G
	8	60.3%	1.000	4	22.5%	1.000	4	16.6%	1.000	7	42.4%	1.000	
**FN2098**	2	16.3%	0.999	3	16.7%	0.997	4	27.2%	1.000	3	17.9%	1.000	AAL94182.1| MRP-family nucleotide-binding protein
	1	5.4%	0.788	7	43.6%	1.000	7	38.1%	1.000	1	5.4%	0.657	
**FN2100**	10	32.9%	1.000	5	14.2%	1.000	6	25.0%	1.000	9	25.2%	1.000	AAL94184.1| Hypothetical protein
	11	38.7%	1.000	7	24.8%	1.000	8	27.4%	1.000	8	27.6%	1.000	
**FN2102**	9	22.7%	1.000	5	17.0%	1.000	5	15.7%	1.000	5	11.7%	1.000	AAL94186.1| ABC transporter ATP-binding protein
	9	19.7%	1.000	5	15.5%	1.000	7	20.1%	1.000	5	8.6%	1.000	
**FN2103**	33	76.3%	1.000	29	76.0%	1.000	32	81.5%	1.000	35	81.5%	1.000	AAL94187.1| tricarboxylate-binding protein
	31	76.3%	1.000	29	78.9%	1.000	30	76.3%	1.000	32	76.3%	1.000	

Fn Rep I Rep II | *FnPg* Rep I Rep II | *FnSg* Rep I Rep II | *FnPgSg* Rep I Rep II

Show detected proteins only
Show all proteins
Filter by category:
GO: amino acid transport

Proteins found: 1424

Enter (or paste) list of ORFs

Find ORFs

Dot Plots Dot Plots

ORF	*Fn* n	*Fn* coverage	*Fn* prob	*FnPg* n	*FnPg* coverage	*FnPg* prob	*FnSg* n	*FnSg* coverage	*FnSg* prob	*FnPgSg* n	*FnPgSg* coverage	*FnPgSg* prob	Description
FN2105	5	8.5%	1.000	1	4.7%	0.661	2	2.4%	0.953	5	12.1%	1.000	AAL94189.1| tricarboxylate transport membrane protein RctA
	3	4.7%	0.999	4	11.9%	1.000	2	8.5%	0.990	4	8.5%	1.000	
FN2106	17	18.3%	1.000	15	19.3%	1.000	12	17.6%	1.000	14	21.0%	1.000	AAL94190.1| Transporter
	16	18.3%	1.000	13	18.7%	1.000	11	17.4%	1.000	15	18.3%	1.000	
FN2107	16	37.5%	1.000	6	16.7%	1.000	4	17.5%	1.000	14	32.4%	1.000	AAL94191.1| Galactokinase
	16	42.7%	1.000	7	21.6%	1.000	5	15.7%	1.000	14	32.1%	1.000	
FN2108	10	26.1%	1.000	4	10.8%	1.000	7	20.2%	1.000	12	27.9%	1.000	AAL94192.1| Galactose-1-phosphate uridylyltransferase
	12	26.9%	1.000	7	18.5%	1.000	9	27.7%	1.000	11	27.9%	1.000	
FN2109	10	37.4%	1.000	11	45.0%	1.000	11	35.6%	1.000	15	60.5%	1.000	AAL94193.1| UDP-glucose 4-epimerase
	13	43.8%	1.000	14	32.2%	1.000	14	42.2%	1.000	9	32.5%	1.000	
FN2116	12	55.4%	1.000	2	23.8%	0.990	4	28.0%	1.000	4	26.8%	1.000	AAL94200.1| Hypothetical exported 24-amino acid repeat protein
	13	57.1%	1.000	6	41.7%	1.000	3	14.9%	1.000	4	14.3%	1.000	
FN2117	7	32.0%	1.000	0	0.0%	0.000	1	2.9%	0.361	2	7.8%	0.999	AAL94201.1| Hypothetical exported 24-amino acid repeat protein
	4	15.2%	1.000	4	14.3%	1.000	4	15.6%	1.000	2	7.4%	0.524	
FN2118	10	33.9%	1.000	0	0.0%	0.000	1	4.5%	0.985	9	35.5%	1.000	AAL94202.1| Hypothetical exported 24-amino acid repeat protein
	11	33.9%	1.000	3	11.4%	1.000	3	11.4%	1.000	6	23.3%	1.000	
FN2119	17	35.2%	1.000	0	0.0%	0.000	4	15.7%	1.000	7	19.2%	1.000	AAL94203.1| Hypothetical exported 24-amino acid repeat protein
	10	19.8%	1.000	15	32.8%	1.000	9	25.1%	1.000	6	14.8%	1.000	
FN2120	7	30.2%	1.000	1	4.2%	0.896	0	0.0%	0.000	2	16.9%	1.000	AAL94204.1| Hypothetical exported 24-amino acid repeat protein
	8	34.4%	1.000	2	16.9%	1.000	1	10.1%	0.956	2	10.6%	0.992	
FN2121	16	50.0%	1.000	6	31.3%	1.000	6	22.2%	1.000	14	50.0%	1.000	AAL94205.1| Hypothetical exported 24-amino acid repeat protein
	16	50.0%	1.000	10	43.0%	1.000	1	7.4%	0.947	16	50.4%	1.000	
FN2122	40	53.0%	1.000	32	47.4%	1.000	52	70.4%	1.000	47	59.6%	1.000	AAL94206.1| Phenylalanyl-tRNA synthetase beta chain
	46	58.9%	1.000	50	69.8%	1.000	47	62.0%	1.000	44	55.6%	1.000	
FN2123	15	36.1%	1.000	12	42.9%	1.000	18	63.9%	1.000	15	40.8%	1.000	AAL94207.1| Phenylalanyl-tRNA synthetase alpha chain
	18	40.8%	1.000	18	66.3%	1.000	19	66.0%	1.000	14	38.5%	1.000	
FN2125	27	31.4%	1.000	15	18.1%	1.000	15	21.1%	1.000	26	31.6%	1.000	AAL94209.1| DNA gyrase subunit A
	30	31.2%	1.000	21	32.1%	1.000	30	35.3%	1.000	24	30.6%	1.000	
FN2126	22	32.9%	1.000	8	13.5%	1.000	8	17.4%	1.000	26	35.4%	1.000	AAL94210.1| DNA gyrase subunit B
	23	32.4%	1.000	15	24.7%	1.000	14	28.5%	1.000	23	35.1%	1.000	
FN2128	3	7.3%	1.000	2	3.3%	0.988	1	2.4%	0.902	2	5.4%	0.820	AAL94212.1| RECF protein
	2	4.9%	0.963	1	3.5%	0.285	0	0.0%	0.000	2	6.0%	0.882	

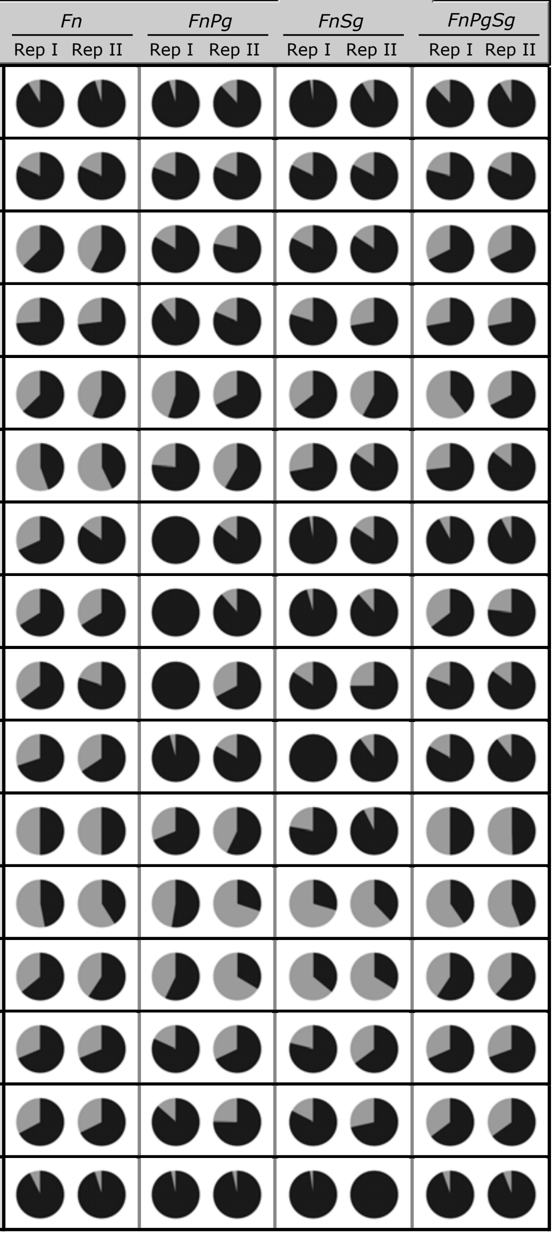

Show detected proteins only
Show all proteins
Filter by category:
GO: amino acid transport
Proteins found: 1424
Enter (or paste) list of ORFs
Find ORFs
Dot Plots
Dot Plots
