# Supplementary material for: Proteomics of Fusobacterium nucleatum within a model developing oral microbial community
Source: Microbiologyopen. 2014 Aug 25;3(5):729–51. doi: 10.1002/mbo3.204 (PMC4234264; doi:10.1002/mbo3.204)
Supplement: Table S3 — A more detailed presentation of the quantitative data for this comparison, including P-values, q-values, raw and normalized spectral counts, abundance ratios, and protein descriptors. [file mbo30003-0729-sd5.pdf]

FnPgSg vs Fn

Fusobacterium nucleatum

Hackett Laboratory UW

Fn Summary Table | FnPg vs Fn | FnSg vs Fn | FnPgSg vs Fn | FnPgSg vs FnPg | FnSg vs FnPg | FnPgSg vs FnSg | Fn Coverage

Protein	*FnPgSg* vs *Fn* Log2 Ratio	Log2 Sum	*q*-Value	*p*-Value	Raw *FnPgSg*	Raw *Fn*	Normalized *FnPgSg*	Normalized *Fn*	Description	Log2 Ratios (-6 -4 -2 0 2 4 6)
**FN0001**	-1.995	6.873	5.464e-4	1.117e-3	7	22	7.0000	24.9009	AAL94214.1| Chromosomal replication initiator protein dnaA	
					3	15	3.8437	18.3350		
**FN0004**	0.743	12.991	1.429e-2	6.168e-2	145	66	145.0000	74.7027	AAL94217.1| Inner membrane protein	
					69	53	88.4055	64.7838		
**FN0005**	0.049	11.579	1.256e-1	7.273e-1	60	41	60.0000	46.4062	AAL94218.1| Jag protein	
					41	51	52.5308	62.3391		
**FN0006**	-0.914	7.902	1.152e-3	2.895e-3	11	17	11.0000	19.2416	AAL94219.1| Thiophene and furan oxidation protein THDF	
					9	19	11.5312	23.2244		
**FN0007**	-1.690	7.762	1.687e-4	2.265e-4	10	23	10.0000	26.0328	AAL94220.1| Glucose inhibited division protein A	
					5	22	6.4062	26.8914		
**FN0017**						6		6.7912	AAL94230.1| Hypothetical protein	
						7		8.5563		
**FN0018**	-0.591	10.897	1.44e-4	1.826e-4	34	45	34.0000	50.9337	AAL94231.1| Hypothetical protein	
					29	46	37.1559	56.2274		
**FN0019**	0.483	4.232							AAL94232.1| Transcription-repair coupling factor	
					4	3	5.1250	3.6670		
**FN0021**									AAL94234.1| 4-diphosphocytidyl-2-C-methyl-D-erythritol kinase	
						5		6.1117		
**FN0022**	-0.906	11.279	2.08e-3	6.345e-3	28	59	28.0000	66.7797	AAL94235.1| Hypothetical protein	
					35	57	44.8434	69.6731		
**FN0023**									AAL94236.1| Short-chain fatty acids transporter	
					10		12.8124			
**FN0024**	-1.508	11.036	3.217e-4	5.553e-4	30	75	30.0000	84.8894	AAL94237.1| Hypothetical exported 24-amino acid repeat protein	
					19	57	24.3435	69.6731		
**FN0025**	-2.155	8.484				36		40.7469	AAL94238.1| Hypothetical exported 24-amino acid repeat protein	
					7	32	8.9687	39.1147		
**FN0026**	0.872	9.200	1.2e-2	5.066e-2	40	9	40.0000	10.1867	AAL94239.1| Hypothetical exported 24-amino acid repeat protein	
					20	21	25.6248	25.6690		
**FN0029**	-0.920	5.564			5	7	5.0000	7.9230	AAL94242.1| Flavodoxin	
						9		11.0010		
**FN0030**	-0.029	11.568	1.437e-1	8.494e-1	63	53	63.0000	59.9885	AAL94243.1| 5-nitroimidazole antibiotic resistance protein	
					36	42	46.1246	51.3381		

○ Show detected proteins only
○ Show all proteins
☐ Filter by category:
GO: amino acid transport

Proteins found: 1297

Enter (or paste) list of ORFs

Find ORFs

Test	Cutoff
q-Value	.005
p-Value	

	Signif	Direction	Applies To
red	yes	+	ratios, bars
yellow	no	n/a	bars
green	yes	–	ratios, bars
pink	yes	+	*p*- , *q*-Values
light green	yes	–	

Dot Plots Dot Plots

FnPgSg vs Fn	Fusobacterium nucleatum	Hackett Laboratory	UW

Protein	*FnPgSg* vs *Fn* Log$_2$ Ratio	Log$_2$ Sum	*q*-Value	*p*-Value	Raw *FnPgSg*	Raw *Fn*	Normalized *FnPgSg*	Normalized *Fn*	Description
FN0031	0.497	6.119	3.703e-2	1.839e-1	7	7	7.0000	7.9230	AAL94244.1| unknown
					10	5	12.8124	6.1117	
FN0033	-1.022	11.959	2.845e-8	7.664e-10	45	79	45.0000	89.4169	AAL94246.1| unknown
					34	74	43.5621	90.4528	
FN0034	0.619	10.066	1.553e-3	4.415e-3	44	24	44.0000	27.1646	AAL94247.1| unknown
					29	21	37.1559	25.6690	
FN0039						8		9.0549	AAL94252.1| DNA primase (bacterial type) and small primase-like proteins
						9		11.0010	
FN0040	-0.097	17.664	1.467e-2	6.352e-2	424	402	424.0000	455.0073	AAL94253.1| Asparaginyl-tRNA synthetase
					357	399	457.4025	487.7119	
FN0041	-0.402	6.068	5.601e-2	2.941e-1	4	8	4.0000	9.0549	AAL94254.1| unknown
					8	8	10.2499	9.7787	
FN0043	-1.806	5.806			4	15	4.0000	16.9779	AAL94256.1| Hypothetical exported 24-amino acid repeat protein
						9		11.0010	
FN0045						8		9.0549	AAL94258.1| Shikimate 5-dehydrogenase
						8		9.7787	
FN0046	1.136	5.579				5		5.6593	AAL94259.1| 3-dehydroquinate dehydratase
					8	3	10.2499	3.6670	
FN0047	-0.392	12.565	8.829e-4	2.036e-3	68	82	68.0000	92.8124	AAL94260.1| Exodeoxyribonuclease III
					53	70	67.9057	85.5635	
FN0048	-1.375	9.318	4.649e-4	9.091e-4	16	33	16.0000	37.3513	AAL94261.1| 4-nitrophenylphosphatase
					12	36	15.3749	44.0041	
FN0049									AAL94262.1| Hypothetical protein
						3		3.6670	
FN0050	-0.308	20.596	2.719e-2	1.28e-1	906	1157	906.0000	1309.5608	AAL94263.1| Fumarate reductase flavoprotein subunit
					1059	1221	1356.8326	1492.4717	
FN0052	-0.988	10.152	5.96e-5	5.203e-5	21	44	21.0000	49.8018	AAL94265.1| Arsenate reductase
					21	37	26.9060	45.2264	
FN0054	0.180	15.065	7.739e-4	1.728e-3	193	153	193.0000	173.1744	AAL94267.1| Tyrosyl-tRNA synthetase
					157	143	201.1546	174.7940	
FN0058	-0.885	14.203	1.496e-5	5.701e-6	106	171	106.0000	193.5479	AAL94271.1| Cysteine desulfhydrase
					75	147	96.0930	179.6833	

Log$_2$ Ratios: -6 -4 -2 0 2 4 6

- Show detected proteins only
- Show all proteins
- Filter by category: GO: amino acid transport

Proteins found: 1297

Enter (or paste) list of ORFs

Find ORFs

Test	Cutoff
q-Value	.005
p-Value	

	Signif	Direction	Applies To
red	yes	+	ratios, bars
yellow	no	n/a	bars
green	yes	−	ratios, bars
light red	yes	+	*p*- , *q*-Values
light green	yes	−	

Dot Plots | Dot Plots

Protein	*FnPgSg* vs *Fn* Log$_2$ Ratio	Log$_2$ Sum	*q*-Value	*p*-Value	Raw *FnPgSg*	Raw *Fn*	Normalized *FnPgSg*	Normalized *Fn*	Description
**FN0059**	-0.329	10.946	5.812e-3	2.184e-2	37	48	37.0000	54.3292	AAL94272.1| NifU protein
					33	37	42.2809	45.2264	
**FN0060**	-0.809	9.321	1.62e-5	6.499e-6	19	30	19.0000	33.9558	AAL94273.1| D-alanyl-D-alanine carboxypeptidase
					15	27	19.2186	33.0031	
**FN0061**	0.017	12.506	1.065e-1	6.032e-1	74	67	74.0000	75.8345	AAL94274.1| Thermostable carboxypeptidase 1
					62	62	79.4368	75.7848	
**FN0062**	0.342	7.177	3.453e-2	1.697e-1	13	7	13.0000	7.9230	AAL94275.1| Hypothetical cytosolic protein
					11	11	14.0936	13.4457	
**FN0065**	-0.753	14.005	4.603e-4	8.975e-4	95	157	95.0000	177.7019	AAL94278.1| Transcription accessory protein (S1 RNA binding domain)
					80	127	102.4992	155.2366	
**FN0067**	-0.530	17.426	2.072e-5	9.503e-6	341	434	341.0000	491.2268	AAL94280.1| Isoleucyl-tRNA synthetase
					279	423	357.4658	517.0479	
**FN0069**	-0.085	14.640	7.328e-2	3.959e-1	163	159	163.0000	179.9656	AAL94282.1| Glycyl-tRNA synthetase alpha chain
					115	122	147.3425	149.1249	
**FN0070**	-0.461	17.173	1.237e-4	1.47e-4	326	390	326.0000	441.4250	AAL94283.1| Glycyl-tRNA synthetase beta chain
					257	377	329.2785	460.8205	
**FN0071**									AAL94284.1| GTP cyclohydrolase I
						8		9.7787	
**FN0072**	-0.484	12.097	7.448e-5	7.088e-5	53	67	53.0000	75.8345	AAL94285.1| 2-amino-4-hydroxy-6-hydroxymethyldihydropteridine pyrophosphokinase
					46	66	58.9370	80.6741	
**FN0073**	-1.025	9.482	1.825e-5	7.944e-6	17	35	17.0000	39.6151	AAL94286.1| Dihydropteroate synthase
					16	30	20.4998	36.6701	
**FN0074**	-0.710	7.629			11	21	11.0000	23.7690	AAL94287.1| Ethanolamine utilization protein eutS
						10		12.2234	
**FN0077**									AAL94290.1| Ethanolamine two-component sensor kinase
						6		7.3340	
**FN0078**	-0.209	5.540			5		5.0000		AAL94291.1| Ethanolamine utilization protein eutA
					6	6	7.6874	7.3340	
**FN0079**	-1.622	11.369	8.866e-5	9.085e-5	33	86	33.0000	97.3399	AAL94292.1| Ethanolamine ammonia-lyase heavy chain
					20	68	25.6248	83.1188	
**FN0080**	-0.932	12.585	9.368e-4	2.197e-3	52	106	52.0000	119.9770	AAL94293.1| Ethanolamine ammonia-lyase light chain
					48	79	61.4995	96.5645	

Log$_2$ Ratios: -6 -4 -2 0 2 4 6

Show detected proteins only
Show all proteins
Filter by category:
GO: amino acid transport

Proteins found: 1297

Enter (or paste) list of ORFs

Find ORFs

Test	Cutoff
q-Value	.005
p-Value	

	Signif	Direction	Applies To
(red)	yes	+	ratios, bars
(yellow)	no	n/a	bars
(green)	yes	−	ratios, bars
(pink)	yes	+	*p*-, *q*-Values
(light green)	yes	−	

Dot Plots Dot Plots

Protein	*FnPgSg* vs Fn: Log_2 Ratio	Log_2 Sum	*q*-Value	*p*-Value	Raw: *FnPgSg*	Raw: *Fn*	Normalized: *FnPgSg*	Normalized: *Fn*	Description	Log_2 Ratios (-6 -4 -2 0 2 4 6)
FN0081	-1.614	11.503	3.069e-5	1.767e-5	27	78	27.0000	88.2850	AAL94294.1| Ethanolamine utilization protein eutL	
					27	82	34.5935	100.2315		
FN0082	0.534	8.032			21		21.0000		AAL94295.1| Ethanolamine utilization protein eutM	
					14	11	17.9374	13.4457		
FN0083	-0.929	13.177	1.214e-3	3.115e-3	78	132	78.0000	149.4054	AAL94296.1| Ethanolamine utilization protein eutM precursor	
					48	95	61.4995	116.1219		
FN0084	-0.929	9.620	4.369e-4	8.395e-4	24	36	24.0000	40.7469	AAL94297.1| Acetaldehyde dehydrogenase (acetylating)	
					13	30	16.6561	36.6701		
FN0088									AAL94301.1| Hypothetical protein	
						4		4.8893		
FN0089					4		4.0000		AAL94302.1| Ethanolamine permease	
FN0092	-1.780	8.204	3.482e-5	2.242e-5	7	26	7.0000	29.4283	AAL94305.1| NADPH-dependent butanol dehydrogenase	
					9	28	11.5312	34.2254		
FN0093	-0.043	16.685	1.195e-1	6.867e-1	305	323	305.0000	365.5904	AAL94306.1| Thioredoxin	
					261	240	334.4035	293.3605		
FN0100	-0.431	9.723	2.228e-2	1.019e-1	27	24	27.0000	27.1646	AAL94309.1| Flavodoxins/hemoproteins	
					18	33	23.0623	40.3371		
FN0102	-0.826	6.687	7.66e-3	3.016e-2	5	12	5.0000	13.5823	AAL94311.1| Ribonucleoside-diphosphate reductase alpha chain	
					8	11	10.2499	13.4457		
FN0103	-1.146	5.146			4	7	4.0000	7.9230	AAL94312.1| Ribonucleoside-diphosphate reductase beta chain	
						8		9.7787		
FN0106	0.122	13.189	3.395e-2	1.665e-1	94	87	94.0000	98.4717	AAL94315.1| Hypothetical protein	
					84	71	107.6241	86.7858		
FN0108	0.104	6.236			9	4	9.0000	4.5274	AAL94317.1| Microcin C7 self-immunity protein mccF	
						10		12.2234		
FN0110	-0.076	17.160	6.415e-2	3.414e-1	342	352	342.0000	398.4144	AAL94319.1| Seryl-tRNA synthetase	
					315	317	403.5904	387.4804		
FN0113	-1.470	9.373	3.872e-5	2.807e-5	13	39	13.0000	44.1425	AAL94322.1| Heat-inducible transcription repressor hrcA	
					14	34	17.9374	41.5594		
FN0114	0.309	14.087	1.204e-2	5.086e-2	163	109	163.0000	123.3726	AAL94323.1| GrpE protein	
					102	93	130.6864	113.6772		

Show detected proteins only
Show all proteins
Filter by category:
GO: amino acid transport

Proteins found: 1297

Enter (or paste) list of ORFs

Find ORFs

Test	Cutoff
q-Value	.005
p-Value	

	Signif	Direction	Applies To
(red)	yes	+	ratios, bars
(yellow)	no	n/a	bars
(green)	yes	–	ratios, bars
(pink)	yes	+	*p*- , *q*-Values
(light green)	yes	–	

Dot Plots Dot Plots

Protein	*FnPgSg* vs *Fn* Log$_2$ Ratio	Log$_2$ Sum	*q*-Value	*p*-Value	Raw *FnPgSg*	Raw *Fn*	Normalized *FnPgSg*	Normalized *Fn*	Description	Log$_2$ Ratios (-6 -4 -2 0 2 4 6)
FN0116	0.016	19.808	1.367e-1	8.016e-1	1009	797	1009.0000	902.0916	AAL94325.1| Chaperone protein dnaK	
					716	821	917.3674	1003.5375		
FN0117									AAL94326.1| O6-methylguanine-DNA methyltransferase	
						5		6.1117		
FN0118	-0.308	12.617	5.011e-6	1.143e-6	72	77	72.0000	87.1531	AAL94327.1| Chaperone protein dnaJ	
					55	73	70.4682	89.2305		
FN0119						10		11.3186	AAL94328.1| Flavodoxin	
						9		11.0010		
FN0123						5		5.6593	AAL94332.1| ATPase	
						6		7.3340		
FN0128	-1.639	8.215	3.046e-4	5.156e-4	8	30	8.0000	33.9558	AAL94334.1| Spermidine/putrescine-binding protein	
					9	22	11.5312	26.8914		
FN0129						3		3.3956	AAL94335.1| Urease accessory protein ureG	
FN0130	-0.159	8.012			24	15	24.0000	16.9779	AAL94336.1| ABC transporter ATP-binding protein	
					5		6.4062			
FN0132	0.425	6.174			12		12.0000		AAL93916.1| Hemolysin	
					6	6	7.6874	7.3340		
FN0147	-0.837	10.087	3.929e-6	8.249e-7	25	39	25.0000	44.1425	AAL94353.1| PLSX protein	
					19	36	24.3435	44.0041		
FN0148	-0.054	15.945	3.576e-2	1.767e-1	242	234	242.0000	264.8550	AAL94354.1| 3-oxoacyl-[acyl-carrier-protein] synthase III	
					196	202	251.1229	246.9118		
FN0149	-0.142	15.778	8.342e-2	4.585e-1	190	252	190.0000	285.2285	AAL94355.1| Malonyl-CoA-[acyl-carrier-protein] transacylase	
					204	174	261.3728	212.6864		
FN0150	-0.199	12.000	1.166e-2	4.904e-2	58	65	58.0000	73.5708	AAL94356.1| Acyl carrier protein	
					48	52	61.4995	63.5614		
FN0151	-0.016	19.835	1.398e-1	8.225e-1	893	825	893.0000	933.7836	AAL94357.1| 3-oxoacyl-[acyl-carrier-protein] synthase	
					804	827	1030.1165	1010.8715		
FN0152	0.444	4.915				4		4.5274	AAL94358.1| Ribonuclease III	
					5	4	6.4062	4.8893		
FN0154	-0.307	5.666				7		7.9230	AAL94360.1| Ribonuclease G	
					5		6.4062			

Show detected proteins only
Show all proteins
Filter by category:
GO: amino acid transport

Proteins found: 1297

Enter (or paste) list of ORFs

Find ORFs

Test	Cutoff
q-Value	.005
p-Value	

	Signif	Direction	Applies To
red	yes	+	ratios, bars
yellow	no	n/a	bars
green	yes	–	ratios, bars
pink	yes	+	*p*-, *q*-Values
light green	yes	–	

Dot Plots Dot Plots

Protein	*FnPgSg* vs Fn				Raw		Normalized		Description	Log_2 Ratios (-6 -4 -2 0 2 4 6)
	Log_2 Ratio	Log_2 Sum	*q*-Value	*p*-Value	*FnPgSg*	*Fn*	*FnPgSg*	*Fn*		
**FN0155**	-0.070	6.627			13	9	13.0000	10.1867	AAL94361.1| Hypothetical protein	
					5		6.4062			
**FN0156**	0.307	7.947	1.625e-4	2.15e-4	17	12	17.0000	13.5823	AAL94362.1| Phosphopantetheine adenylyltransferase	
					14	12	17.9374	14.6680		
**FN0157**						7		7.9230	AAL94363.1| DNA repair protein RadA	
						6		7.3340		
**FN0158**	-0.730	8.802	5.307e-3	1.963e-2	20	20	20.0000	22.6372	AAL94364.1| DNA-binding protein	
					10	26	12.8124	31.7807		
**FN0164**	-1.297	15.126	3.319e-4	5.799e-4	117	243	117.0000	275.0417	AAL94370.1| Anhydro-N-acetylmuramyl-tripeptide amidase	
					97	260	124.2802	317.8072		
**FN0170**	0.137	13.551	1.406e-3	3.848e-3	117	95	117.0000	107.5266	AAL94376.1| GTP-binding protein	
					88	83	112.7491	101.4538		
**FN0173**	0.284	11.663	6.328e-3	2.416e-2	68	48	68.0000	54.3292	AAL94379.1| Hypothetical protein	
					45	40	57.6558	48.8934		
**FN0174**	0.849	13.478	3.396e-3	1.143e-2	165	65	165.0000	73.5708	AAL94380.1| Enoyl-[acyl-carrier-protein] reductase	
					95	70	121.7177	85.5635		
**FN0175**	-0.214	7.848				17		19.2416	AAL94381.1| Cell division inhibitor MinC	
					11	11	14.0936	13.4457		
**FN0176**	0.209	15.019	2.682e-2	1.26e-1	174	158	174.0000	178.8337	AAL94382.1| Cell division inhibitor MinD	
					170	131	217.8107	160.1260		
**FN0177**	-0.050	7.349	1.285e-1	7.461e-1	11	10	11.0000	11.3186	AAL94383.1| Cell division inhibitor MinE	
					11	12	14.0936	14.6680		
**FN0178**	-0.978	12.024	3.175e-5	1.88e-5	42	78	42.0000	88.2850	AAL94384.1| UNC-44 ankyrins	
					39	76	49.9683	92.8975		
**FN0179**	-0.461	9.357	1.498e-2	6.506e-2	27	25	27.0000	28.2965	AAL94385.1| Ankyrin repeat proteins	
					13	26	16.6561	31.7807		
**FN0180**					8		8.0000		AAL94386.1| Tetratricopeptide repeat family protein	
**FN0181**	-0.669	8.172	3.035e-2	1.456e-1	9	13	9.0000	14.7142	AAL94387.1| Hypothetical protein	
					14	23	17.9374	28.1137		
**FN0182**	-0.020	11.915	1.551e-1	9.294e-1	76	49	76.0000	55.4611	AAL94388.1| Sarcosine oxidase alpha subunit	
					37	57	47.4059	69.6731		

Show detected proteins only
Show all proteins
Filter by category:
GO: amino acid transport

Proteins found: 1297

Enter (or paste) list of ORFs

Find ORFs

Test	Cutoff
q-Value	.005
p-Value	

Color	Signif	Direction	Applies To
red	yes	+	ratios, bars
yellow	no	n/a	bars
green	yes	−	ratios, bars
light red	yes	+	*p*- , *q*-Values
light green	yes	−	*p*- , *q*-Values

Dot Plots Dot Plots

Protein	*FnPgSg* vs *Fn*				Raw		Normalized		Description
	Log_2 Ratio	Log_2 Sum	*q*-Value	*p*-Value	*FnPgSg*	*Fn*	*FnPgSg*	*Fn*	
**FN0183**	0.222	13.861	4.094e-2	2.068e-1	152	97	152.0000	109.7903	AAL94389.1| Glycerol-3-phosphate dehydrogenase
					87	95	111.4678	116.1219	
**FN0185**	-0.703	7.422	3.725e-2	1.852e-1	9	9	9.0000	10.1867	AAL94391.1| Hypothetical protein
					9	19	11.5312	23.2244	
**FN0188**						13		14.7142	AAL94394.1| Peptide methionine sulfoxide reductase
**FN0189**					9		9.0000		AAL94395.1| Two-component response regulator yesN
					4		5.1250		
**FN0190**									AAL94396.1| Two-component sensor kinase yesM
						7		8.5563	
**FN0191**	-0.956	7.924	5.339e-3	1.977e-2	7	20	7.0000	22.6372	AAL94397.1| helix-turn-helix DNA-binding protein
					12	17	15.3749	20.7797	
**FN0192**	-1.560	11.963	2.817e-4	4.648e-4	39	88	39.0000	99.6036	AAL94398.1| Dipeptide-binding protein
					27	96	34.5935	117.3442	
**FN0197**									AAL94403.1| Methyltransferase
						4		4.8893	
**FN0198**	0.119	5.211	1.087e-1	6.173e-1	5	6	5.0000	6.7912	AAL94404.1| Transcriptional regulatory protein
					6	4	7.6874	4.8893	
**FN0199**	0.034	10.353	1.476e-1	8.765e-1	45	34	45.0000	38.4832	AAL94405.1| Hypothetical protein
					22	27	28.1873	33.0031	
**FN0200**	-1.580	20.140	6.631e-7	7.146e-8	609	1665	609.0000	1884.5452	AAL94406.1| Biotin carboxyl carrier protein of glutaconyl-COA decarboxylase
					495	1499	634.2135	1832.2809	
**FN0202**	0.046	20.246	1.346e-1	7.871e-1	1335	1032	1335.0000	1168.0784	AAL94408.1| Glutaconate CoA-transferase subunit A
					727	840	931.4611	1026.7618	
**FN0203**	-0.570	20.287	9.913e-4	2.364e-3	833	1114	833.0000	1260.8909	AAL94409.1| Glutaconate CoA-transferase subunit B
					799	1223	1023.7103	1494.9163	
**FN0204**	-0.211	21.405	4.17e-3	1.466e-2	1462	1600	1462.0000	1810.9743	AAL94410.1| Glutaconyl-CoA decarboxylase A subunit
					1277	1452	1636.1428	1774.8312	
**FN0206**	-0.994	11.813	1.258e-6	1.822e-7	44	74	44.0000	83.7576	AAL94412.1| Activator of (R)-2-hydroxyglutaryl-CoA dehydratase
					32	70	40.9997	85.5635	
**FN0207**	-0.338	18.825	8.945e-5	9.205e-5	620	681	620.0000	770.7959	AAL94413.1| (R)-2-hydroxyglutaryl-CoA dehydratase alpha-subunit
					462	623	591.9326	761.5150	

Log_2 Ratios: -6 -4 -2 0 2 4 6

Show detected proteins only
Show all proteins
Filter by category:
GO: amino acid transport

Proteins found: 1297

Enter (or paste) list of ORFs

Find ORFs

Test	Cutoff
q-Value	.005
p-Value	

Signif	Direction	Applies To
yes	+	ratios, bars
no	n/a	bars
yes	−	ratios, bars
yes	+	*p*- , *q*-Values
yes	−	

Dot Plots Dot Plots

FnPgSg vs Fn

Fusobacterium nucleatum

Hackett Laboratory UW

Protein	*FnPgSg vs Fn* Log$_2$ Ratio	Log$_2$ Sum	*q*-Value	*p*-Value	Raw *FnPgSg*	Raw *Fn*	Normalized *FnPgSg*	Normalized *Fn*	Description
FN0208	-0.233	17.852	4.31e-4	8.251e-4	435	461	435.0000	521.7870	AAL94414.1| (R)-2-hydroxyglutaryl-CoA dehydratase beta-subunit
					361	436	462.5274	532.9383	
FN0209	-0.455	17.713	3.089e-5	1.788e-5	383	486	383.0000	550.0834	AAL94415.1| Hypothetical cytosolic protein
					319	438	408.7154	535.3830	
FN0212	-0.593	8.361	5.343e-3	1.979e-2	18	21	18.0000	23.7690	AAL94418.1| Hypothetical protein
					9	17	11.5312	20.7797	
FN0218	-1.149	12.740	1.827e-4	2.538e-4	56	114	56.0000	129.0319	AAL94424.1| Anthranilate synthase component II
					43	96	55.0933	117.3442	
FN0219	0.986	5.344				4		4.5274	AAL94425.1| Autolysin response regulator
					7		8.9687		
FN0221	0.683	11.721	9.094e-3	3.691e-2	87	40	87.0000	45.2744	AAL94427.1| Carbon starvation protein A
					47	38	60.2183	46.4487	
FN0224	-0.636	9.062	7.015e-3	2.723e-2	23	25	23.0000	28.2965	AAL94430.1| Excinuclease ABC subunit B
					11	24	14.0936	29.3361	
FN0226	-0.441	11.765	3.279e-6	6.51e-7	50	61	50.0000	69.0434	AAL94432.1| Pyridoxal phosphate biosynthetic protein pdxA
					40	56	51.2496	68.4508	
FN0233	-0.444	12.009	2.958e-3	9.715e-3	55	61	55.0000	69.0434	AAL94439.1| Hypothetical protein
					43	66	55.0933	80.6741	
FN0234						12		13.5823	AAL94440.1| unknown
						14		17.1127	
FN0236	-0.360	15.082	2.733e-3	8.803e-3	152	172	152.0000	194.6797	AAL94442.1| ABC transporter substrate-binding protein
					138	186	176.8110	227.3544	
FN0238	-0.547	10.368	8.482e-3	3.398e-2	23	42	23.0000	47.5381	AAL94444.1| Hypothetical protein
					29	33	37.1559	40.3371	
FN0240	-0.155	12.854	3.066e-2	1.474e-1	76	87	76.0000	98.4717	AAL94446.1| Thymidylate synthase
					68	68	87.1243	83.1188	
FN0241	-1.298	7.633	8.746e-3	3.524e-2	9	25	9.0000	28.2965	AAL94447.1| Dihydrofolate reductase
					7	13	8.9687	15.8904	
FN0242	-1.748	10.070	2.019e-5	9.157e-6	14	50	14.0000	56.5929	AAL94448.1| Trk system potassium uptake protein trkA
					17	52	21.7811	63.5614	
FN0243	-2.031	6.746				10		11.3186	AAL94449.1| Poly(A) polymerase
					4	25	5.1250	30.5584	

Log$_2$ Ratios: -6 -4 -2 0 2 4 6

Show detected proteins only
Show all proteins
Filter by category:
GO: amino acid transport

Proteins found: 1297

Enter (or paste) list of ORFs

Find ORFs

Test	Cutoff
q-Value	.005
p-Value	

	Signif	Direction	Applies To
red	yes	+	ratios, bars
yellow	no	n/a	bars
green	yes	–	ratios, bars
light red	yes	+	*p*- , *q*-Values
light green	yes	–	

Dot Plots Dot Plots

Protein	FnPgSg vs Fn Log$_2$ Ratio	Log$_2$ Sum	*q*-Value	*p*-Value	Raw *FnPgSg*	Raw *Fn*	Normalized *FnPgSg*	Normalized *Fn*	Description
FN0244						16		18.1097	AAL94450.1| COP associated protein
						20		24.4467	
FN0245	-1.235	6.405			6	12	6.0000	13.5823	AAL94451.1| Copper-exporting ATPase
						12		14.6680	
FN0247	-0.127	15.249	9.125e-2	5.071e-1	229	183	229.0000	207.1302	AAL94453.1| Hypothetical cytosolic protein
					116	168	148.6238	205.3524	
FN0248					9		9.0000		AAL94454.1| Hypothetical Exported Protein
FN0249	-0.813	14.879	2.894e-5	1.594e-5	121	210	121.0000	237.6904	AAL94455.1| unknown
					110	182	140.9363	222.4651	
FN0250	0.063	14.226	5.06e-2	2.621e-1	142	113	142.0000	127.9001	AAL94456.1| unknown
					110	117	140.9363	143.0133	
FN0251	-0.104	15.702	1.066e-1	6.041e-1	175	195	175.0000	220.7125	AAL94457.1| Hypothetical membrane-spanning Protein
					211	211	270.3415	257.9128	
FN0252	-0.338	16.247	1.241e-3	3.212e-3	231	270	231.0000	305.6019	AAL94458.1| unknown
					207	263	265.2166	321.4742	
FN0253	0.155	15.816	2.243e-2	1.027e-1	234	198	234.0000	224.1081	AAL94459.1| Outer membrane protein
					213	189	272.9040	231.0214	
FN0254	-1.120	18.754	4.687e-4	9.187e-4	373	851	373.0000	963.2120	AAL94460.1| Fusobacterium outer membrane protein family
					413	816	529.1519	997.4258	
FN0258	-1.444	6.159				16		18.1097	AAL94464.1| Zinc-transporting ATPase
					4	8	5.1250	9.7787	
FN0259					11		11.0000		AAL94465.1| Zinc-transporting ATPase
					12		15.3749		
FN0260									AAL94466.1| Transcriptional repressor smtB
						5		6.1117	
FN0261	-1.556	8.822	3.891e-5	2.839e-5	12	31	12.0000	35.0876	AAL94467.1| Pyruvate formate-lyase activating enzyme
					10	31	12.8124	37.8924	
FN0262	-0.198	24.438	9.858e-4	2.347e-3	4443	4406	4443.0000	4986.9705	AAL94468.1| Formate acetyltransferase
					3482	4274	4461.2757	5224.2620	
FN0263	-1.266	15.895	2.808e-5	1.515e-5	180	358	180.0000	405.2055	AAL94469.1| Peptidyl-prolyl cis-trans isomerase
					108	295	138.3739	360.5890	

Log$_2$ Ratios: -6 -4 -2 0 2 4 6

Show detected proteins only
Show all proteins
Filter by category:
GO: amino acid transport

Proteins found: 1297

Enter (or paste) list of ORFs

Find ORFs

Test	Cutoff
q-Value	.005
p-Value	

Signif	Direction	Applies To
yes	+	ratios, bars
no	n/a	bars
yes	–	ratios, bars
yes	+	*p*- , *q*-Values
yes	–	

Dot Plots Dot Plots

Protein	*FnPgSg* vs *Fn* Log$_2$ Ratio	Log$_2$ Sum	*q*-Value	*p*-Value	Raw *FnPgSg*	Raw *Fn*	Normalized *FnPgSg*	Normalized *Fn*	Description
**FN0264**	-0.264	16.639	2.791e-3	9.033e-3	273	314	273.0000	355.4037	AAL94470.1| Hypothetical protein
					242	282	310.0599	344.6986	
**FN0265**	-2.080	7.250			6	20	6.0000	22.6372	AAL94471.1| Cell division protein ftsX
						23		28.1137	
**FN0266**	-2.100	5.985				14		15.8460	AAL94472.1| membrane protein related to metalloendopeptidase
					3	14	3.8437	17.1127	
**FN0267**						7		7.9230	AAL94473.1| ATP-NAD kinase
						9		11.0010	
**FN0268**	-2.006	5.891				10		11.3186	AAL94474.1| DNA repair protein recN
					3	16	3.8437	19.5574	
**FN0270**	-1.136	5.516	2.84e-4	4.698e-4	4	8	4.0000	9.0549	AAL94476.1| GTP-binding protein era
					4	9	5.1250	11.0010	
**FN0271**	-0.923	4.923			4	8	4.0000	9.0549	AAL94477.1| Enoyl-CoA hydratase
						5		6.1117	
**FN0272**									AAL94478.1| Acetoacetate: butyrate/acetate coenzyme A transferase
						5		6.1117	
**FN0276**	-1.749	5.749			4		4.0000		AAL94482.1| Sodium-dependent phosphate transporter
						11		13.4457	
**FN0277**	-0.579	6.914	1.193e-2	5.032e-2	9	14	9.0000	15.8460	AAL94483.1| Hypothetical protein
					7	9	8.9687	11.0010	
**FN0278**	0.277	16.373	2.187e-2	9.985e-2	362	230	362.0000	260.3276	AAL94484.1| Xaa-His dipeptidase
					218	220	279.3102	268.9138	
**FN0279**	-0.558	11.420	6.44e-6	1.66e-6	44	55	44.0000	62.2522	AAL94485.1| Lipoprotein
					33	53	42.2809	64.7838	
**FN0280**	-0.804	13.341	7.714e-6	2.203e-6	76	119	76.0000	134.6912	AAL94486.1| Hypothetical protein
					61	110	78.1556	134.4569	
**FN0281**	-1.928	10.273	2.801e-5	1.509e-5	13	64	13.0000	72.4390	AAL94487.1| DNA polymerase III alpha subunit
					18	53	23.0623	64.7838	
**FN0282**	-0.165	10.616	8.002e-2	4.372e-1	30	32	30.0000	36.2195	AAL94488.1| Hypothetical protein
					35	39	44.8434	47.6711	
**FN0283**	-1.932	7.291							AAL94489.1| tRNA (Guanine-N1) - methyltransferase
					5	20	6.4062	24.4467	

Log$_2$ Ratios: -6 -4 -2 0 2 4 6

Show detected proteins only
Show all proteins
Filter by category:
GO: amino acid transport

Proteins found: 1297

Enter (or paste) list of ORFs

Find ORFs

Test	Cutoff
q-Value	.005
p-Value	

	Signif	Direction	Applies To
red	yes	+	ratios, bars
yellow	no	n/a	bars
green	yes	−	ratios, bars
pink	yes	+	*p*- , *q*-Values
light green	yes	−	*p*- , *q*-Values

Dot Plots Dot Plots

Protein	FnPgSg vs Fn Log$_2$ Ratio	Log$_2$ Sum	*q*-Value	*p*-Value	Raw *FnPgSg*	Raw *Fn*	Normalized *FnPgSg*	Normalized *Fn*	Description
FN0284	0.337	7.950	7.893e-2	4.305e-1	11	15	11.0000	16.9779	AAL94490.1| 16S rRNA processing protein rimM
					19	9	24.3435	11.0010	
FN0285	1.943	6.300			22	4	22.0000	4.5274	AAL94491.1| RNA binding protein
					10		12.8124		
FN0287	-0.675	6.428	2.962e-4	4.968e-4	7	11	7.0000	12.4504	AAL94493.1| Dimethyladenosine transferase
					6	9	7.6874	11.0010	
FN0288	0.338	10.201	3.316e-2	1.618e-1	31	28	31.0000	31.6921	AAL94494.1| Hypoxanthine-guanine phosphoribosyltransferase
					36	24	46.1246	29.3361	
FN0290									AAL94496.1| Hemolysin
					3		3.8437		
FN0291	0.270	4.019			5		5.0000		AAL94497.1| Hemolysin
					3	3	3.8437	3.6670	
FN0294	-0.403	15.495	3.238e-4	5.605e-4	188	212	188.0000	239.9541	AAL94500.1| Transketolase subunit A
					145	208	185.7797	254.2458	
FN0295	-0.486	14.228	1.671e-3	4.845e-3	106	146	106.0000	165.2514	AAL94501.1| Transketolase
					100	133	128.1239	162.5706	
FN0296	0.648	11.491	2.681e-3	8.596e-3	60	39	60.0000	44.1425	AAL94502.1| Hypothetical cytosolic protein
					58	34	74.3119	41.5594	
FN0297	-0.481	6.366				6		6.7912	AAL94503.1| ATPase associated with chromosome architecture/replication
					6	12	7.6874	14.6680	
FN0298	-0.036	15.012	9.846e-2	5.526e-1	181	173	181.0000	195.8116	AAL94504.1| Histidyl-tRNA synthetase
					139	141	178.0923	172.3493	
FN0299	-0.090	16.188	1.752e-2	7.775e-2	262	239	262.0000	270.5143	AAL94505.1| Aspartyl-tRNA synthetase
					209	240	267.7790	293.3605	
FN0305	-0.100	3.649			3		3.0000		AAL94511.1| Iron(III) dicitrate-binding protein
					3	3	3.8437	3.6670	
FN0307									AAL94513.1| Iron(III) dicitrate transport ATP-binding protein fecE
					3		3.8437		
FN0308	-0.343	16.795	1.812e-2	8.08e-2	354	347	354.0000	392.7551	AAL94514.1| Iron(III)-binding protein
					191	300	244.7167	366.7007	
FN0309	-0.359	6.220			5		5.0000		AAL94515.1| Iron(III)-transport system permease protein sfuB
					8	8	10.2499	9.7787	

Log$_2$ Ratios: -6 -4 -2 0 2 4 6

Show detected proteins only
Show all proteins
Filter by category:
GO: amino acid transport

Proteins found: 1297

Enter (or paste) list of ORFs

Find ORFs

Test	Cutoff
q-Value	.005
p-Value	

	Signif	Direction	Applies To
red	yes	+	ratios, bars
yellow	no	n/a	bars
green	yes	−	ratios, bars
light red	yes	+	*p*-, *q*-Values
light green	yes	−	

Dot Plots Dot Plots

Protein	FnPgSg vs Fn Log_2 Ratio	Log_2 Sum	*q*-Value	*p*-Value	Raw *FnPgSg*	Raw *Fn*	Normalized *FnPgSg*	Normalized *Fn*	Description	Log_2 Ratios -6 -4 -2 0 2 4 6
FN0310	-0.601	13.803	3.806e-3	1.311e-2	116	123	116.0000	139.2187	AAL94516.1| Iron(III)-transport ATP-binding protein sfuC	
					61	127	78.1556	155.2366		
FN0311	-0.226	13.517	1.285e-2	5.469e-2	90	100	90.0000	113.1859	AAL94517.1| Anaerobic ribonucleoside-triphosphate reductase	
					86	99	110.1866	121.0112		
FN0313	-2.327	6.970			5	13	5.0000	14.7142	AAL94519.1| 16S rRNA m(5)C 967 methyltransferase	
						29		35.4477		
FN0316	-0.223	9.825	4.806e-2	2.474e-1	25	24	25.0000	27.1646	AAL94522.1| Hypothetical protein	
					24	31	30.7497	37.8924		
FN0317	-0.857	6.522	1.105e-2	4.61e-2	4	12	4.0000	13.5823	AAL94523.1| Tryptophan synthase beta chain	
					8	10	10.2499	12.2234		
FN0319	-0.766	5.481				10		11.3186	AAL94525.1| Citrate (pro-3S)-lyase ligase	
					4	5	5.1250	6.1117		
FN0320	0.484	9.799	1.113e-5	3.673e-6	36	23	36.0000	26.0328	AAL94526.1| Hypothetical cytosolic protein	
					27	20	34.5935	24.4467		
FN0321	0.075	13.499	8.253e-2	4.529e-1	103	101	103.0000	114.3178	AAL94527.1| Heat shock protein htpG	
					92	78	117.8740	95.3422		
FN0322	0.111	21.126	3.666e-2	1.818e-1	1688	1315	1688.0000	1488.3945	AAL94528.1| Fructose-bisphosphate aldolase	
					1136	1164	1455.4880	1422.7985		
FN0325	1.139	13.220	2.875e-3	9.372e-3	122	58	122.0000	65.6478	AAL94529.1| LSU ribosomal protein L20P	
					131	54	167.8424	66.0061		
FN0326	1.302	7.274			16	7	16.0000	7.9230	AAL94530.1| LSU ribosomal protein L35P	
					18		23.0623			
FN0327	-1.855	10.992	1.798e-4	2.48e-4	18	75	18.0000	84.8894	AAL94531.1| Bacterial Protein Translation Initiation Factor 3 (IF-3)	
					23	71	29.4685	86.7858		
FN0329	-0.651	17.760	3.448e-4	6.117e-4	347	528	347.0000	597.6215	AAL94533.1| LSU ribosomal protein L13P	
					316	477	404.8717	583.0540		
FN0330	0.826	14.085	1.409e-4	1.774e-4	187	81	187.0000	91.6806	AAL94534.1| SSU ribosomal protein S9P	
					128	87	163.9986	106.3432		
FN0331	-0.216	11.866	1.345e-6	2.051e-7	57	58	57.0000	65.6478	AAL94535.1| Hypothetical protein	
					44	54	56.3745	66.0061		
FN0332					7		7.0000		AAL94536.1| Magnesium and cobalt transport protein corA	

Show detected proteins only
Show all proteins
Filter by category:
GO: amino acid transport

Proteins found: 1297

Enter (or paste) list of ORFs

Find ORFs

Test	Cutoff
q-Value	.005
p-Value	

	Signif	Direction	Applies To
red	yes	+	ratios, bars
yellow	no	n/a	bars
green	yes	−	ratios, bars
light red	yes	+	*p*- , *q*-Values
light green	yes	−	*p*- , *q*-Values

Dot Plots Dot Plots

Protein	FnPgSg vs Fn Log2 Ratio	Log2 Sum	q-Value	p-Value	Raw FnPgSg	Raw Fn	Normalized FnPgSg	Normalized Fn	Description
FN0334	-0.088	14.104	2.244e-3	6.939e-3	128	123	128.0000	139.2187	AAL94538.1| Aspartate/aromatic aminotransferase
					101	110	129.4052	134.4569	
FN0335	-0.800	19.873	1.018e-4	1.121e-4	764	1093	764.0000	1237.1218	AAL94539.1| Outer membrane porin F
					563	1103	721.3378	1348.2361	
FN0336	-0.757	12.400	1.222e-3	3.141e-3	67	76	67.0000	86.0213	AAL94540.1| Hypothetical protein
					36	86	46.1246	105.1209	
FN0341	0.452	12.443	1.446e-3	4.002e-3	90	62	90.0000	70.1753	AAL94545.1| transport protein
					66	47	84.5618	57.4498	
FN0342	0.166	10.903	8.716e-2	4.817e-1	44	46	44.0000	52.0655	AAL94546.1| Peptidyl-prolyl cis-trans isomerase
					38	25	48.6871	30.5584	
FN0343						4		4.5274	AAL94547.1| Hypothetical protein
FN0344									AAL94548.1| Methyltransferase
						9		11.0010	
FN0347	-1.941	11.232	2.077e-4	3.076e-4	27	78	27.0000	88.2850	AAL94551.1| Phosphatidylserine decarboxylase
					18	85	23.0623	103.8985	
FN0348	-0.598	15.785	1.01e-3	2.422e-3	203	279	203.0000	315.7886	AAL94552.1| Nicotinate phosphoribosyltransferase
					143	220	183.2172	268.9138	
FN0349	0.087	6.557			10	8	10.0000	9.0549	AAL94553.1| D-Tyr-tRNATyr deacylase
						8		9.7787	
FN0351	-0.271	14.174	1.764e-2	7.835e-2	122	117	122.0000	132.4275	AAL94555.1| unknown
					98	136	125.5615	166.2376	
FN0352	-0.787	10.231	4.061e-3	1.419e-2	31	47	31.0000	53.1974	AAL94556.1| NA+/H+ antiporter NHAC
					17	31	21.7811	37.8924	
FN0355	-0.606	14.529	2.716e-4	4.432e-4	116	170	116.0000	192.4160	AAL94558.1| S-adenosylmethionine synthetase
					104	153	133.2489	187.0173	
FN0356	-1.801	8.752	2.524e-7	1.913e-8	12	35	12.0000	39.6151	AAL94559.1| Lactoylglutathione lyase
					8	31	10.2499	37.8924	
FN0357	-0.138	7.580	1.187e-1	6.818e-1	11	17	11.0000	19.2416	AAL94560.1| ATP synthase epsilon chain, sodium ion specific
					12	8	15.3749	9.7787	
FN0358	-0.992	17.088	2.477e-7	1.823e-8	268	470	268.0000	531.9737	AAL94561.1| ATP synthase beta chain, sodium ion specific
					204	426	261.3728	520.7149	

Log2 Ratios: -6 -4 -2 0 2 4 6

Show detected proteins only
Show all proteins
Filter by category:
GO: amino acid transport

Proteins found: 1297

Enter (or paste) list of ORFs

Find ORFs

Test	Cutoff
q-Value	.005
p-Value	

	Signif	Direction	Applies To
red	yes	+	ratios, bars
yellow	no	n/a	bars
green	yes	−	ratios, bars
light red	yes	+	p- , q-Values
light green	yes	−	

Dot Plots Dot Plots

Protein	FnPgSg vs Fn				Raw		Normalized		Description	Log_2 Ratios
	Log_2 Ratio	Log_2 Sum	*q*-Value	*p*-Value	*FnPgSg*	*Fn*	*FnPgSg*	*Fn*		-6 -4 -2 0 2 4 6
FN0359	-0.584	11.809	1.868e-4	2.623e-4	53	68	53.0000	76.9664	AAL94562.1| ATP synthase gamma chain, sodium ion specific	
					35	57	44.8434	69.6731		
FN0360	0.041	13.439	8.798e-2	4.868e-1	101	95	101.0000	107.5266	AAL94563.1| ATP synthase alpha chain, sodium ion specific	
					88	82	112.7491	100.2315		
FN0361	-0.550	10.085	2.28e-4	3.525e-4	25	37	25.0000	41.8788	AAL94564.1| ATP synthase delta chain, sodium ion specific	
					23	31	29.4685	37.8924		
FN0362	-0.536	10.276	3.621e-2	1.792e-1	29	49	29.0000	55.4611	AAL94565.1| ATP synthase B chain, sodium ion specific	
					23	24	29.4685	29.3361		
FN0364	1.071	4.820			9		9.0000		AAL94567.1| ATP synthase A chain, sodium ion specific	
					5	3	6.4062	3.6670		
FN0366	0.100	16.101	4.609e-2	2.361e-1	253	228	253.0000	258.0638	AAL94569.1| Phosphoglucosamine mutase	
					231	208	295.9663	254.2458		
FN0368	0.078	12.788	7.982e-2	4.36e-1	78	69	78.0000	78.0983	AAL94571.1| Adenylosuccinate lyase	
					74	70	94.8117	85.5635		
FN0370	-1.004	9.526	2.752e-3	8.88e-3	14	28	14.0000	31.6921	AAL94573.1| Signal peptidase I	
					19	37	24.3435	45.2264		
FN0371	0.167	12.681	6.273e-2	3.331e-1	100	66	100.0000	74.7027	AAL94574.1| Hypothetical protein	
					56	64	71.7494	78.2295		
FN0374						3		3.3956	AAL94577.1| Single-stranded-DNA-specific exonuclease recJ	
						4		4.8893		
FN0375	0.529	17.403	1.105e-3	2.734e-3	466	297	466.0000	336.1621	AAL94578.1| Iron(III)-binding protein	
					417	292	534.2768	356.9220		
FN0376	-0.708	11.289	6.328e-5	5.649e-5	36	59	36.0000	66.7797	AAL94579.1| Iron(III)-transport ATP-binding protein sfuC	
					33	50	42.2809	61.1168		
FN0377	1.124	8.044			39		39.0000		AAL94580.1| Iron(III)-transport system permease protein sfuB	
					7	9	8.9687	11.0010		
FN0378	0.286	6.371	8.949e-2	4.962e-1	6	7	6.0000	7.9230	AAL94581.1| UDP-glucose 4-epimerase	
					11	7	14.0936	8.5563		
FN0379	-0.107	6.985	1.231e-1	7.105e-1	14	12	14.0000	13.5823	AAL94582.1| Hypothetical protein	
					6	8	7.6874	9.7787		
FN0380	-1.707	7.592				26		29.4283	AAL94583.1| unknown	
					6	17	7.6874	20.7797		

Show detected proteins only
Show all proteins
Filter by category:
GO: amino acid transport

Proteins found: 1297

Enter (or paste) list of ORFs

Find ORFs

Test	Cutoff
q-Value	.005
p-Value	

	Signif	Direction	Applies To
red	yes	+	ratios, bars
yellow	no	n/a	bars
green	yes	−	ratios, bars
pink	yes	+	*p*- , *q*-Values
light green	yes	−	

Dot Plots Dot Plots

Protein	FnPgSg vs Fn: Log_2 Ratio	Log_2 Sum	*q*-Value	*p*-Value	Raw *FnPgSg*	Raw *Fn*	Normalized *FnPgSg*	Normalized *Fn*	Description
FN0381	0.286	8.570	4.251e-3	1.502e-2	20	15	20.0000	16.9779	AAL94584.1| unknown
					18	15	23.0623	18.3350	
FN0382						8		9.0549	AAL94585.1| Hypothetical protein
						8		9.7787	
FN0383						7		7.9230	AAL94586.1| Lipopolysaccharide N-acetylglucosaminyltransferase
						10		12.2234	
FN0384	-1.709	8.942	4.412e-4	8.5e-4	13	32	13.0000	36.2195	AAL94587.1| Hypothetical protein
					9	36	11.5312	44.0041	
FN0386						12		13.5823	AAL94589.1| Hypothetical protein
						11		13.4457	
FN0387	-1.823	12.522	3.938e-5	2.918e-5	38	121	38.0000	136.9549	AAL94590.1| Fusobacterium outer membrane protein family
					34	124	43.5621	151.5696	
FN0390	1.213	11.769	2.133e-3	6.538e-3	103	34	103.0000	38.4832	AAL94593.1| Hypothetical protein
					60	32	76.8744	39.1147	
FN0391	-0.312	7.965	2.99e-3	9.845e-3	13	16	13.0000	18.1097	AAL94594.1| Hydrolase (HAD superfamily)
					12	14	15.3749	17.1127	
FN0392	-1.162	6.915	6.901e-6	1.857e-6	7	15	7.0000	16.9779	AAL94595.1| Oxygen-independent coproporphyrinogen III oxidase
					6	13	7.6874	15.8904	
FN0393	-0.799	5.478	6.439e-5	5.787e-5	5	8	5.0000	9.0549	AAL94596.1| Polysaccharide deacetylase
					4	7	5.1250	8.5563	
FN0394	-0.889	6.059			6	11	6.0000	12.4504	AAL94597.1| Outer membrane protein
						8		9.7787	
FN0396	0.388	23.786	1.779e-4	2.444e-4	4210	2994	4210.0000	3388.7857	AAL94599.1| Dipeptide-binding protein
					3505	2667	4490.7442	3259.9688	
FN0397	0.292	11.044	9.168e-3	3.727e-2	53	41	53.0000	46.4062	AAL94600.1| Dipeptide transport system permease protein dppB
					38	30	48.6871	36.6701	
FN0398	-0.506	11.735	2.89e-3	9.437e-3	48	56	48.0000	63.3841	AAL94601.1| Dipeptide transport system permease protein dppC
					39	62	49.9683	75.7848	
FN0399	-0.367	15.250	9.994e-3	4.12e-2	149	178	149.0000	201.4709	AAL94602.1| Dipeptide transport ATP-binding protein dppD
					155	202	198.5921	246.9118	
FN0400	-1.174	17.112	1.348e-4	1.662e-4	227	498	227.0000	563.6658	AAL94603.1| Dipeptide transport ATP-binding protein dppF
					214	464	274.1852	567.1637	

Log_2 Ratios: -6 -4 -2 0 2 4 6

Show detected proteins only
Show all proteins
Filter by category:
GO: amino acid transport

Proteins found: 1297

Enter (or paste) list of ORFs

Find ORFs

Test	Cutoff
q-Value	.005
p-Value	

	Signif	Direction	Applies To
red	yes	+	ratios, bars
yellow	no	n/a	bars
green	yes	−	ratios, bars
pink	yes	+	*p*- , *q*-Values
light green	yes	−	

Dot Plots | Dot Plots

Protein	*FnPgSg vs Fn* Log$_2$ Ratio	Log$_2$ Sum	*q*-Value	*p*-Value	Raw *FnPgSg*	Raw *Fn*	Normalized *FnPgSg*	Normalized *Fn*	Description
FN0405	0.742	13.733	4.952e-3	1.804e-2	175	86	175.0000	97.3399	AAL94608.1| Tryptophanyl-tRNA synthetase
					99	68	126.8427	83.1188	
FN0406	-0.395	11.956	2.87e-3	9.355e-3	60	64	60.0000	72.4390	AAL94609.1| Alanine racemase, biosynthetic
					39	59	49.9683	72.1178	
FN0407	0.167	12.951	3.356e-2	1.642e-1	104	75	104.0000	84.8894	AAL94610.1| Hypothetical protein
					66	68	84.5618	83.1188	
FN0408	-0.372	12.560	6.445e-3	2.47e-2	61	86	61.0000	97.3399	AAL94611.1| Acetyl-coenzyme A carboxylase carboxyl transferase subunit beta
					59	65	75.5931	79.4518	
FN0409	0.047	17.998	1.012e-1	5.699e-1	475	453	475.0000	512.7321	AAL94612.1| Acetyl-coenzyme A carboxylase carboxyl transferase subunit alpha
					441	404	565.0266	493.8235	
FN0410	-0.330	13.814	1.17e-5	3.94e-6	109	120	109.0000	135.8231	AAL94613.1| 6-phosphofructokinase
					82	109	105.0616	133.2346	
FN0411									AAL94614.1| putative alpha helix protein
						8		9.7787	
FN0412									AAL94615.1| Recombination protein recR
						10		12.2234	
FN0413	0.236	3.764			4	3	4.0000	3.3956	AAL94616.1| unknown
FN0414					6		6.0000		AAL94617.1| ATP-dependent helicase HEPA
FN0416	-1.240	7.959	1.825e-3	5.409e-3	9	18	9.0000	20.3735	AAL94619.1| Type III restriction-modification system methylation subunit
					9	23	11.5312	28.1137	
FN0417	-2.222	9.230	1.308e-4	1.591e-4	15	46	15.0000	52.0655	AAL94620.1| Type III restriction-modification system restriction subunit
					6	44	7.6874	53.7828	
FN0418					4		4.0000		AAL94621.1| Uracil phosphoribosyltransferase
FN0419	-0.696	6.305			5	10	5.0000	11.3186	AAL94622.1| Aspartate carbamoyltransferase
					7		8.9687		
FN0420	-2.171	5.341			3	12	3.0000	13.5823	AAL94623.1| Dihydroorotase
						11		13.4457	
FN0421	-1.542	7.350	3.29e-5	2.008e-5	6	18	6.0000	20.3735	AAL94624.1| Carbamoyl-phosphate synthase small chain
					7	19	8.9687	23.2244	

Log$_2$ Ratios: -6 -4 -2 0 2 4 6

Show detected proteins only
Show all proteins
Filter by category:
GO: amino acid transport

Proteins found: 1297

Enter (or paste) list of ORFs

Find ORFs

Test	Cutoff
q-Value	.005
p-Value	

	Signif	Direction	Applies To
red	yes	+	ratios, bars
yellow	no	n/a	bars
green	yes	−	ratios, bars
light red	yes	+	*p*- , *q*-Values
light green	yes	−	*p*- , *q*-Values

Dot Plots Dot Plots

Protein	*FnPgSg* vs *Fn*				Raw		Normalized		Description
	Log_2 Ratio	Log_2 Sum	*q*-Value	*p*-Value	*FnPgSg*	*Fn*	*FnPgSg*	*Fn*	
**FN0422**	-1.107	13.354	2.957e-4	4.957e-4	69	125	69.0000	141.4824	AAL94625.1| Carbamoyl-phosphate synthase large chain
					55	130	70.4682	158.9036	
**FN0423**	-0.906	5.929	1.149e-2	4.821e-2	5	7	5.0000	7.9230	AAL94626.1| Dihydroorotate dehydrogenase electron transfer subunit
					5	11	6.4062	13.4457	
**FN0424**	-0.650	3.820			3	4	3.0000	4.5274	AAL94627.1| Dihydroorotate dehydrogenase
						4		4.8893	
**FN0426**	0.490	8.271	2.317e-2	1.066e-1	25	10	25.0000	11.3186	AAL94629.1| Orotidine 5'-phosphate decarboxylase
					13	15	16.6561	18.3350	
**FN0427**	-0.336	11.021	1.975e-3	5.97e-3	44	43	44.0000	48.6699	AAL94630.1| Orotate phosphoribosyltransferase
					29	44	37.1559	53.7828	
**FN0430**	-0.602	15.497	3.365e-4	5.911e-4	171	223	171.0000	252.4045	AAL94633.1| LSU ribosomal protein L19P
					139	227	178.0923	277.4702	
**FN0435**	-0.657	10.570	3.348e-2	1.637e-1	48	39	48.0000	44.1425	AAL94634.1| Purine nucleoside phosphorylase
					11	44	14.0936	53.7828	
**FN0436**	-0.470	9.107	3.133e-2	1.512e-1	13	24	13.0000	27.1646	AAL94635.1| regulator of kinase autophosphorylation inhibitor
					21	23	26.9060	28.1137	
**FN0437**	-0.598	6.054	4.03e-2	2.03e-1	3	8	3.0000	9.0549	AAL94636.1| kinase autophosphorylation inhibitor KipI
					8	9	10.2499	11.0010	
**FN0438**									AAL94637.1| transporter protein
						9		11.0010	
**FN0439**	-0.011	11.486	1.562e-1	9.369e-1	58	41	58.0000	46.4062	AAL94638.1| Lactam utilization protein LAMB
					38	50	48.6871	61.1168	
**FN0445**	0.077	4.875	7.806e-2	4.251e-1	6	5	6.0000	5.6593	AAL94641.1| Hypothetical protein
					4	4	5.1250	4.8893	
**FN0446**	-0.567	7.518	1.677e-4	2.247e-4	12	14	12.0000	15.8460	AAL94642.1| Hypothetical protein
					8	14	10.2499	17.1127	
**FN0447**	-0.221	4.221			4	5	4.0000	5.6593	AAL94643.1| NIFS protein
						3		3.6670	
**FN0448**									AAL94644.1| DNA-repair protein
					3		3.8437		
**FN0450**	-0.980	9.525	1.959e-2	8.824e-2	22	22	22.0000	24.9009	AAL94646.1| ABC transporter ATP-binding protein
					13	42	16.6561	51.3381	

Log_2 Ratios: -6 -4 -2 0 2 4 6

Show detected proteins only
Show all proteins
Filter by category:
GO: amino acid transport

Proteins found: 1297

Enter (or paste) list of ORFs

Find ORFs

Test	Cutoff
q-Value	.005
p-Value	

	Signif	Direction	Applies To
red	yes	+	ratios, bars
yellow	no	n/a	bars
green	yes	−	ratios, bars
pink	yes	+	*p*- , *q*-Values
light green	yes	−	*p*- , *q*-Values

Dot Plots Dot Plots

Protein	*FnPgSg* vs *Fn* Log$_2$ Ratio	Log$_2$ Sum	*q*-Value	*p*-Value	Raw *FnPgSg*	Raw *Fn*	Normalized *FnPgSg*	Normalized *Fn*	Description
FN0451	-0.864	5.579				10		11.3186	AAL94647.1| Hypothetical protein
					4	6	5.1250	7.3340	
FN0452	0.015	18.716	9.886e-2	5.552e-1	663	592	663.0000	670.0605	AAL94648.1| Glucosamine--fructose-6-phosphate aminotransferase (isomerizing)
					512	520	655.9946	635.6145	
FN0453	-0.220	15.291	4.987e-3	1.819e-2	198	195	198.0000	220.7125	AAL94649.1| Xaa-Pro aminopeptidase
					135	173	172.9673	211.4640	
FN0454	-0.507	12.316	4.712e-5	3.749e-5	57	77	57.0000	87.1531	AAL94650.1| Aldehyde dehydrogenase B
					49	68	62.7807	83.1188	
FN0455	1.180	16.037	2.472e-3	7.79e-3	331	150	331.0000	169.7788	AAL94651.1| Rubrerythrin
					351	143	449.7150	174.7940	
FN0456	-1.123	11.157	2.866e-5	1.568e-5	34	62	34.0000	70.1753	AAL94652.1| Hypothetical cytosolic protein
					24	58	30.7497	70.8955	
FN0458	0.645	4.714				4		4.5274	AAL94654.1| Hypothetical Exported Protein
					5	3	6.4062	3.6670	
FN0459						8		9.0549	AAL94655.1| Hypothetical exported 24-amino acid repeat protein
						8		9.7787	
FN0460	-1.497	10.305	6.626e-5	6.025e-5	18	57	18.0000	64.5160	AAL94656.1| Delta-aminolevulinic acid dehydratase
					19	45	24.3435	55.0051	
FN0461	-0.409	15.695	3.45e-4	6.121e-4	187	227	187.0000	256.9320	AAL94657.1| Probable sigma(54) modulation protein
					166	224	212.6857	273.8032	
FN0462	-1.400	9.521	1.37e-6	2.122e-7	18	40	18.0000	45.2744	AAL94658.1| DNA mismatch repair protein mutL
					12	35	15.3749	42.7817	
FN0465	-1.256	14.564	4.782e-4	9.431e-4	104	195	104.0000	220.7125	AAL94661.1| Hypothetical protein
					76	213	97.3742	260.3575	
FN0466	-0.060	16.914	1.128e-1	6.434e-1	295	323	295.0000	365.5904	AAL94662.1| Lysyl-tRNA synthetase
					307	288	393.3405	352.0326	
FN0470	-0.264	17.849	9.081e-3	3.685e-2	427	430	427.0000	486.6993	AAL94666.1| Putative efflux pump component MtrF
					359	473	459.9650	578.1647	
FN0472	1.747	24.324	1.612e-8	2.726e-10	8444	2271	8444.0000	2570.4517	AAL94668.1| Flavodoxin
					6518	1990	8351.1187	2432.4477	
FN0474									AAL94670.1| Acriflavin resistance protein B
						6		7.3340	

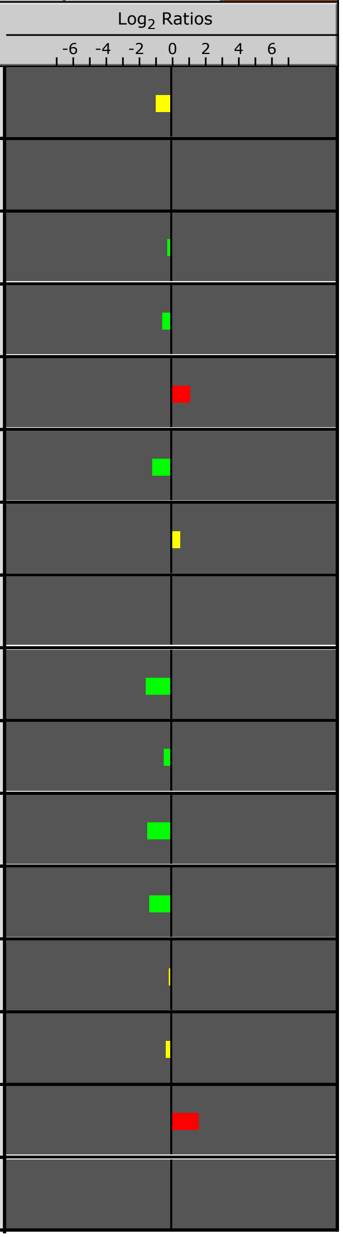

Show detected proteins only
Show all proteins
Filter by category:
GO: amino acid transport

Proteins found: 1297

Enter (or paste) list of ORFs

Find ORFs

Test	Cutoff
q-Value	.005
p-Value	

	Signif	Direction	Applies To
red	yes	+	ratios, bars
yellow	no	n/a	bars
green	yes	−	ratios, bars
pink	yes	+	*p*- , *q*-Values
light green	yes	−	*p*- , *q*-Values

Dot Plots Dot Plots

Protein	FnPgSg vs Fn Log2 Ratio	Log2 Sum	q-Value	p-Value	Raw FnPgSg	Raw Fn	Normalized FnPgSg	Normalized Fn	Description
FN0475	-2.051	9.760	1.464e-4	1.867e-4	11	53	11.0000	59.9885	AAL94671.1| MIAB protein
					14	49	17.9374	59.8944	
FN0476	0.349	12.079	2.206e-3	6.801e-3	69	49	69.0000	55.4611	AAL94672.1| Transcription termination factor rho
					62	50	79.4368	61.1168	
FN0477	-2.734	9.591	5.255e-6	1.223e-6	10	65	10.0000	73.5708	AAL94673.1| Cell wall endopeptidase family M23/M37
					9	57	11.5312	69.6731	
FN0478	-0.326	6.924	2.915e-2	1.388e-1	12	11	12.0000	12.4504	AAL94674.1| GcpE protein
					6	10	7.6874	12.2234	
FN0481						11		12.4504	AAL94677.1| unknown
						17		20.7797	
FN0482	0.455	10.977	2.631e-2	1.232e-1	59	44	59.0000	49.8018	AAL94678.1| LSU ribosomal protein L31P
					36	22	46.1246	26.8914	
FN0483	-0.298	13.739	1.011e-3	2.428e-3	111	120	111.0000	135.8231	AAL94679.1| Uracil phosphoribosyltransferase
					78	101	99.9367	123.4559	
FN0484	-1.179	5.179			4	8	4.0000	9.0549	AAL94680.1| Lipase
FN0487	-0.002	20.199	1.601e-1	9.651e-1	1065	933	1065.0000	1056.0244	AAL94683.1| 2-hydroxyglutarate dehydrogenase
					880	932	1127.4907	1139.2167	
FN0488	0.232	23.279	6.43e-3	2.463e-2	3228	2530	3228.0000	2863.6031	AAL94684.1| NAD-specific glutamate dehydrogenase
					2879	2474	3688.6884	3024.0580	
FN0489	0.489	9.576	9.597e-3	3.936e-2	36	25	36.0000	28.2965	AAL94685.1| Prolipoprotein diacylglyceryl transferase
					23	15	29.4685	18.3350	
FN0490									AAL94686.1| Integral membrane protein
						4		4.8893	
FN0491	-0.523	10.891	3.977e-2	1.999e-1	33	61	33.0000	69.0434	AAL94687.1| Alanine racemase
					31	29	39.7184	35.4477	
FN0493	-2.267	6.556	9.329e-4	2.185e-3	5	16	5.0000	18.1097	AAL94689.1| Hypothetical protein
					3	20	3.8437	24.4467	
FN0494	-0.350	17.923	1.919e-3	5.765e-3	409	464	409.0000	525.1826	AAL94690.1| Short chain dehydrogenase
					370	491	474.0586	600.1667	
FN0495	0.423	24.896	3.67e-5	2.497e-5	6337	4420	6337.0000	5002.8166	AAL94691.1| Acetyl-CoA acetyltransferase
					5153	3801	6602.2269	4646.0973	

Log2 Ratios: -6 -4 -2 0 2 4 6

Show detected proteins only
Show all proteins
Filter by category:
GO: amino acid transport

Proteins found: 1297

Enter (or paste) list of ORFs

Find ORFs

Test	Cutoff
q-Value	.005
p-Value	

	Signif	Direction	Applies To
red	yes	+	ratios, bars
yellow	no	n/a	bars
green	yes	−	ratios, bars
pink	yes	+	p-, q-Values
light green	yes	−	

Dot Plots Dot Plots

Protein	*FnPgSg* vs *Fn* Log$_2$ Ratio	Log$_2$ Sum	*q*-Value	*p*-Value	Raw *FnPgSg*	Raw *Fn*	Normalized *FnPgSg*	Normalized *Fn*	Description	Log$_2$ Ratios (-6 -4 -2 0 2 4 6)
**FN0496**									AAL94692.1| unknown	
						6		7.3340		
**FN0497**									AAL94693.1| Plasmid addiction system poison protein	
					5		6.4062			
**FN0501**	-0.572	15.318	1.268e-3	3.314e-3	156	199	156.0000	225.2399	AAL94697.1| Ornithine decarboxylase	
					137	219	175.5298	267.6915		
**FN0502**	1.159	7.811	1.78e-5	7.62e-6	23	8	23.0000	9.0549	AAL94698.1| Phosphoheptose isomerase	
					17	9	21.7811	11.0010		
**FN0503**	-0.137	11.289	2.524e-2	1.175e-1	48	43	48.0000	48.6699	AAL94699.1| Transcriptional regulatory protein, LYSR family	
					37	46	47.4059	56.2274		
**FN0504**									AAL94700.1| Arginine permease	
					3		3.8437			
**FN0505**	-0.498	9.628	3.89e-4	7.23e-4	23	31	23.0000	35.0876	AAL94701.1| Anthranilate synthase component II	
					19	26	24.3435	31.7807		
**FN0506**	0.209	15.637	4.281e-2	2.173e-1	279	182	279.0000	205.9983	AAL94702.1| Arginyl-tRNA synthetase	
					161	175	206.2796	213.9087		
**FN0511**	-0.866	9.704	2.724e-3	8.769e-3	21	30	21.0000	33.9558	AAL94707.1| D-lactate dehydrogenase	
					17	36	21.7811	44.0041		
**FN0512**	0.175	15.036	4.996e-3	1.823e-2	196	145	196.0000	164.1195	AAL94708.1| Flavoprotein	
					151	148	193.4672	180.9057		
**FN0513**	0.391	10.668	3.591e-5	2.388e-5	45	32	45.0000	36.2195	AAL94709.1| Flavodoxin	
					37	28	47.4059	34.2254		
**FN0515**						4		4.5274	AAL94711.1| Acriflavin resistance protein D	
						5		6.1117		
**FN0519**	-1.396	5.281				6		6.7912	AAL94715.1| Hypothetical exported 24-amino acid repeat protein	
					3	11	3.8437	13.4457		
**FN0522**	-0.444	4.329				6		6.7912	AAL94718.1| Exonuclease SBCC	
					3	3	3.8437	3.6670		
**FN0523**						15		16.9779	AAL94719.1| Exonuclease SBCD	
						20		24.4467		
**FN0524**					17		17.0000		AAL94720.1| DNA helicase II	
					13		16.6561			

Show detected proteins only
Show all proteins
Filter by category:
GO: amino acid transport

Proteins found: 1297

Enter (or paste) list of ORFs

Find ORFs

Test	Cutoff
q-Value	.005
p-Value	

	Signif	Direction	Applies To
red	yes	+	ratios, bars
yellow	no	n/a	bars
green	yes	−	ratios, bars
light red	yes	+	*p*- , *q*-Values
light green	yes	−	

Dot Plots Dot Plots

Protein	*FnPgSg* vs *Fn* Log$_2$ Ratio	Log$_2$ Sum	*q*-Value	*p*-Value	Raw *FnPgSg*	Raw *Fn*	Normalized *FnPgSg*	Normalized *Fn*	Description
FN0525	-0.997	14.733	2.544e-5	1.291e-5	108	196	108.0000	221.8444	AAL94721.1| Penicillin-binding protein
					98	200	125.5615	244.4671	
FN0526	-0.169	10.723	3.901e-2	1.955e-1	34	36	34.0000	40.7469	AAL94722.1| Florfenicol resistance protein
					34	38	43.5621	46.4487	
FN0527	-0.976	7.656	7.51e-4	1.665e-3	10	19	10.0000	21.5053	AAL94723.1| Alanyl-tRNA synthetase
					8	15	10.2499	18.3350	
FN0528	-0.907	20.473	7.938e-5	7.754e-5	951	1436	951.0000	1625.3495	AAL94724.1| Cold shock protein
					633	1373	811.0246	1678.2667	
FN0535	1.124	10.896	7.88e-5	7.674e-5	61	22	61.0000	24.9009	AAL94731.1| Hypothetical protein
					53	28	67.9057	34.2254	
FN0536	0.227	15.611	3.896e-5	2.846e-5	242	184	242.0000	208.2620	AAL94732.1| DNA polymerase III, beta chain
					189	168	242.1543	205.3524	
FN0540	-0.795	10.965	1.277e-4	1.538e-4	32	49	32.0000	55.4611	AAL94736.1| Glutamate-1-semialdehyde 2,1-aminomutase
					28	51	35.8747	62.3391	
FN0541						6		6.7912	AAL94737.1| polysaccharide deacetylase
						15		18.3350	
FN0542	0.228	7.946			17	17	17.0000	19.2416	AAL94738.1| Beta 1,4 glucosyltransferase
						8		9.7787	
FN0543	-0.931	10.280	3.401e-5	2.141e-5	28	45	28.0000	50.9337	AAL94739.1| Lipopolysaccharide heptosyltransferase-1
					18	38	23.0623	46.4487	
FN0546						6		6.7912	AAL94742.1| Lipopolysaccharide core biosynthesis protein rfaQ
FN0547	-0.841	17.021	1.858e-3	5.534e-3	326	425	326.0000	481.0401	AAL94743.1| RecA protein
					171	405	219.0919	495.0459	
FN0549									AAL94745.1| O-sialoglycoprotein endopeptidase
						3		3.6670	
FN0550					18		18.0000		AAL94746.1| hypothetical Protein
FN0552	-1.197	6.812			7	10	7.0000	11.3186	AAL94748.1| Serine racemase
						17		20.7797	
FN0553	-0.155	12.692	3.125e-2	1.508e-1	85	75	85.0000	84.8894	AAL94749.1| D-serine dehydratase
					54	71	69.1869	86.7858	

Log$_2$ Ratios: -6 -4 -2 0 2 4 6

Show detected proteins only
Show all proteins
Filter by category:
GO: amino acid transport

Proteins found: 1297

Enter (or paste) list of ORFs

Find ORFs

Test	Cutoff
q-Value	.005
p-Value	

	Signif	Direction	Applies To
red	yes	+	ratios, bars
yellow	no	n/a	bars
green	yes	–	ratios, bars
pink	yes	+	*p*-, *q*-Values
light green	yes	–	*p*-, *q*-Values

Dot Plots Dot Plots

Protein	*FnPgSg* vs *Fn* Log$_2$ Ratio	Log$_2$ Sum	*q*-Value	*p*-Value	Raw *FnPgSg*	Raw *Fn*	Normalized *FnPgSg*	Normalized *Fn*	Description
FN0554	-0.705	3.875			3		3.0000		AAL94750.1| D-serine permease
						4		4.8893	
FN0556	-2.016	10.209	3.889e-5	2.836e-5	15	65	15.0000	73.5708	AAL94752.1| unknown
					15	53	19.2186	64.7838	
FN0557	-0.995	10.065	2.426e-3	7.614e-3	31	46	31.0000	52.0655	AAL94753.1| unknown
					12	33	15.3749	40.3371	
FN0558	-1.116	10.697	1.296e-4	1.57e-4	31	52	31.0000	58.8567	AAL94754.1| TraT complement resistance protein precursor
					19	50	24.3435	61.1168	
FN0559	0.204	13.574	4.672e-2	2.397e-1	100	90	100.0000	101.8673	AAL94755.1| Phosphoglucomutase
					107	85	137.0926	103.8985	
FN0561	-0.851	10.190	1.578e-5	6.215e-6	24	39	24.0000	44.1425	AAL94757.1| Proline synthetase associated protein
					21	39	26.9060	47.6711	
FN0562	0.267	14.159	8.121e-3	3.229e-2	157	98	157.0000	110.9222	AAL94758.1| Hypothetical cytosolic protein
					109	111	139.6551	135.6792	
FN0563	-1.470	7.713	5.619e-4	1.157e-3	11	21	11.0000	23.7690	AAL94759.1| putative tRNA (5-methylaminomethyl-2-thiouridylate) -methyltransferase
					5	20	6.4062	24.4467	
FN0574	0.236	3.764			4	3	4.0000	3.3956	AAL94770.1| Hypothetical cytosolic protein
FN0576	-1.044	11.064	9.375e-4	2.199e-3	26	58	26.0000	65.6478	AAL94772.1| hypothetical protein
					30	55	38.4372	67.2285	
FN0577									AAL94773.1| Hypothetical protein
						4		4.8893	
FN0579	-0.091	17.086	9.155e-3	3.72e-2	350	341	350.0000	385.9639	AAL94775.1| Hypothetical cytosolic protein
					291	314	372.8407	383.8133	
FN0580						21		23.7690	AAL94776.1| Penicillin-binding protein
						24		29.3361	
FN0581	-1.448	6.807				19		21.5053	AAL94777.1| Lipoprotein releasing system transmembrane protein lolE
					5	11	6.4062	13.4457	
FN0582	-1.230	9.787	1.291e-3	3.398e-3	26	34	26.0000	38.4832	AAL94778.1| Lipoprotein releasing system ATP-binding protein lolD
					10	43	12.8124	52.5604	
FN0583						7		7.9230	AAL94779.1| Hypothetical Exported Protein

Log$_2$ Ratios: -6 -4 -2 0 2 4 6

Show detected proteins only
Show all proteins
Filter by category:
GO: amino acid transport

Proteins found: 1297

Enter (or paste) list of ORFs

Find ORFs

Test	Cutoff
q-Value	.005
p-Value	

	Signif	Direction	Applies To
red	yes	+	ratios, bars
yellow	no	n/a	bars
green	yes	−	ratios, bars
pink	yes	+	*p*- , *q*-Values
light green	yes	−	

Dot Plots Dot Plots

Protein	*FnPgSg* vs *Fn* Log_2 Ratio	Log_2 Sum	*q*-Value	*p*-Value	Raw *FnPgSg*	Raw *Fn*	Normalized *FnPgSg*	Normalized *Fn*	Description
FN0585	-1.050	5.050			4	6	4.0000	6.7912	AAL94781.1| Two-component response regulator czcR
						8		9.7787	
FN0586	-0.720	4.720			4	3	4.0000	3.3956	AAL94782.1| Two-component sensor kinase czcS
						8		9.7787	
FN0590	-1.114	6.380	3.959e-3	1.375e-2	6	14	6.0000	15.8460	AAL94786.1| N-acyl-L-amino acid amidohydrolase
					5	9	6.4062	11.0010	
FN0592	-0.765	12.472	9.214e-4	2.15e-3	49	84	49.0000	95.0762	AAL94788.1| ATP-dependent DNA helicase pcrA
					52	83	66.6245	101.4538	
FN0593	0.036	10.928	1.128e-1	6.434e-1	42	36	42.0000	40.7469	AAL94789.1| UDP-3-O-[3-hydroxymyristoyl] N-acetylglucosamine deacetylase
					37	38	47.4059	46.4487	
FN0594	0.014	5.986			8	7	8.0000	7.9230	AAL94790.1| (3R)-hydroxymyristoyl-[acyl carrier protein] dehydratase
FN0595	0.824	9.288	5.432e-2	2.84e-1	14	17	14.0000	19.2416	AAL94791.1| Acyl-[acyl-carrier-protein]-UDP-N-acetylglucosamine O-acyltransferase
					41	15	52.5308	18.3350	
FN0596	-1.328	8.498			12	23	12.0000	26.0328	AAL94792.1| Hypothetical protein
						28		34.2254	
FN0597	-1.240	9.009	1.167e-3	2.945e-3	18	26	18.0000	29.4283	AAL94793.1| Lipid-A-disaccharide synthase
					9	33	11.5312	40.3371	
FN0598	-0.350	9.446	1.566e-2	6.843e-2	25	30	25.0000	33.9558	AAL94794.1| Phospholipid-lipopolysaccharide ABC transporter
					17	21	21.7811	25.6690	
FN0600	0.162	12.539	5.949e-2	3.142e-1	94	63	94.0000	71.3071	AAL94796.1| Hypothetical protein
					54	61	69.1869	74.5625	
FN0601						31		35.0876	AAL94797.1| Hypothetical exported 24-amino acid repeat protein
						27		33.0031	
FN0602	-0.483	14.163	1.868e-4	2.623e-4	110	148	110.0000	167.5151	AAL94798.1| Hypothetical protein
					93	125	119.1553	152.7919	
FN0603									AAL94799.1| Transcriptional regulatory protein, LYSR family
						4		4.8893	
FN0605									AAL94801.1| Aspartate aminotransferase
					5		6.4062		
FN0608	0.080	10.922	2.224e-2	1.017e-1	47	39	47.0000	44.1425	AAL94804.1| Exoribonuclease II
					34	34	43.5621	41.5594	

Log_2 Ratios (axis: -6 -4 -2 0 2 4 6)

Show detected proteins only
Show all proteins
Filter by category:
GO: amino acid transport

Proteins found: 1297

Enter (or paste) list of ORFs

Find ORFs

Test	Cutoff
q-Value	.005
p-Value	

Signif	Direction	Applies To
yes	+	ratios, bars
no	n/a	bars
yes	–	ratios, bars
yes	+	*p*- , *q*-Values
yes	–	

Dot Plots Dot Plots

Protein	*FnPgSg* vs *Fn* Log_2 Ratio	Log_2 Sum	*q*-Value	*p*-Value	Raw *FnPgSg*	Raw *Fn*	Normalized *FnPgSg*	Normalized *Fn*	Description
FN0610	-0.543	15.636	2.075e-4	3.071e-4	201	248	201.0000	280.7010	AAL94806.1| unknown
					135	216	172.9673	264.0245	
FN0611	-0.494	17.949	1.756e-3	5.151e-3	471	541	471.0000	612.3357	AAL94807.1| Threonyl-tRNA synthetase
					294	476	376.6844	581.8317	
FN0612	-0.449	13.835	3.803e-2	1.897e-1	66	107	66.0000	121.1089	AAL94808.1| Hypothetical protein
					110	132	140.9363	161.3483	
FN0616	-0.494	11.072	1.783e-2	7.93e-2	50	53	50.0000	59.9885	AAL94812.1| Hypothetical protein
					22	41	28.1873	50.1158	
FN0617	-0.472	15.195	9.75e-5	1.048e-4	170	200	170.0000	226.3718	AAL94813.1| DNA polymerase III, beta chain
					124	188	158.8737	229.7991	
FN0618	-1.074	13.277	4.123e-5	3.105e-5	72	128	72.0000	144.8779	AAL94814.1| Spermidine/putrescine-binding protein
					51	118	65.3432	144.2356	
FN0619	-0.843	8.477				22		24.9009	AAL94815.1| Small-conductance mechanosensitive channel
					11	21	14.0936	25.6690	
FN0621	0.032	11.512	1.478e-1	8.778e-1	67	48	67.0000	54.3292	AAL94817.1| 4-hydroxybutyrate coenzyme A transferase
					33	43	42.2809	52.5604	
FN0622	-0.586	4.529	1.697e-4	2.285e-4	4	5	4.0000	5.6593	AAL94818.1| 8-oxoguanine DNA glycosylase
					3	5	3.8437	6.1117	
FN0625	0.628	10.033	2.237e-2	1.024e-1	51	19	51.0000	21.5053	AAL94821.1| Aspartate aminotransferase
					23	25	29.4685	30.5584	
FN0627	0.549	15.693	1.368e-4	1.7e-4	284	158	284.0000	178.8337	AAL94823.1| Glucosamine--fructose-6-phosphate aminotransferase (isomerizing)
					213	165	272.9040	201.6854	
FN0628	1.666	6.825	6.355e-4	1.355e-3	20	3	20.0000	3.3956	AAL94824.1| Glucosamine--fructose-6-phosphate aminotransferase (isomerizing)
					14	7	17.9374	8.5563	
FN0629	1.101	9.400	3.787e-4	6.97e-4	39	13	39.0000	14.7142	AAL94825.1| PTS system, IID component
					29	17	37.1559	20.7797	
FN0630	0.839	8.337			34		34.0000		AAL94826.1| PTS system, IIC component
					11	11	14.0936	13.4457	
FN0631	-0.712	7.927	6.122e-3	2.322e-2	9	18	9.0000	20.3735	AAL94827.1| PTS system, IIB component
					12	16	15.3749	19.5574	
FN0633	-0.507	5.963	5.275e-2	2.747e-1	3	8	3.0000	9.0549	AAL94829.1| Replication protein
					8	8	10.2499	9.7787	

Log_2 Ratios: -6 -4 -2 0 2 4 6

Show detected proteins only
Show all proteins
Filter by category:
GO: amino acid transport

Proteins found: 1297

Enter (or paste) list of ORFs

Find ORFs

Test	Cutoff
q-Value	.005
p-Value	

Signif	Direction	Applies To
yes	+	ratios, bars
no	n/a	bars
yes	–	ratios, bars
yes	+	*p*- , *q*-Values
yes	–	

Dot Plots Dot Plots

Protein	*FnPgSg* vs Fn Log$_2$ Ratio	Log$_2$ Sum	*q*-Value	*p*-Value	Raw *FnPgSg*	Raw *Fn*	Normalized *FnPgSg*	Normalized *Fn*	Description	Log$_2$ Ratios (-6 -4 -2 0 2 4 6)
**FN0634**	-0.464	13.823	1.253e-3	3.257e-3	105	117	105.0000	132.4275	AAL94830.1| GTP-binding protein TypA/BipA	
					78	123	99.9367	150.3473		
**FN0636**						6		6.7912	AAL94832.1| Hypothetical protein	
**FN0637**	-0.561	5.319	7.934e-3	3.142e-2	4	6	4.0000	6.7912	AAL94833.1| Hypothetical exported 24-amino acid repeat protein	
					5	7	6.4062	8.5563		
**FN0643**	-0.252	7.818	3.311e-2	1.615e-1	16	16	16.0000	18.1097	AAL94839.1| hypothetical DNA-binding protein	
					9	12	11.5312	14.6680		
**FN0644**	-0.400	10.754	3.796e-2	1.893e-1	48	39	48.0000	44.1425	AAL94840.1| Uroporphyrin-III C-methyltransferase	
					19	42	24.3435	51.3381		
**FN0645**	-0.577	5.907	9.526e-3	3.902e-2	5	7	5.0000	7.9230	AAL94841.1| Porphobilinogen deaminase	
					6	9	7.6874	11.0010		
**FN0646**					11		11.0000		AAL94842.1| Glutamyl-tRNA reductase	
**FN0647**					5		5.0000		AAL94843.1| transcriptional regulator	
**FN0652**	-0.162	22.228	2.716e-2	1.279e-1	2302	2050	2302.0000	2320.3108	AAL94848.1| Glyceraldehyde 3-phosphate dehydrogenase	
					1474	1937	1888.5469	2367.6639		
**FN0653**	-1.204	10.051	6.921e-4	1.509e-3	16	42	16.0000	47.5381	AAL94849.1| unknown	
					21	42	26.9060	51.3381		
**FN0654**	-0.605	18.012	6.725e-4	1.457e-3	366	532	366.0000	602.1490	AAL94850.1| Phosphoglycerate kinase	
					365	545	467.6524	666.1729		
**FN0655**	-0.599	11.889	1.606e-2	7.04e-2	36	54	36.0000	61.1204	AAL94851.1| unknown	
					50	74	64.0620	90.4528		
**FN0656**	-1.318	11.420	1.331e-3	3.554e-3	33	64	33.0000	72.4390	AAL94852.1| Hypothetical protein	
					26	76	33.3122	92.8975		
**FN0657**						9		10.1867	AAL94853.1| Acetyltransferase	
**FN0658**	0.052	14.536	4.079e-2	2.059e-1	155	139	155.0000	157.3284	AAL94854.1| ABC transporter substrate-binding protein	
					124	119	158.8737	145.4579		
**FN0660**	-0.653	6.461	3.459e-3	1.168e-2	6	10	6.0000	11.3186	AAL94856.1| ABC transporter ATP-binding protein	
					7	10	8.9687	12.2234		

Show detected proteins only
Show all proteins
Filter by category:
GO: amino acid transport

Proteins found: 1297

Enter (or paste) list of ORFs

Find ORFs

Test	Cutoff
q-Value	.005
p-Value	

	Signif	Direction	Applies To
red	yes	+	ratios, bars
yellow	no	n/a	bars
green	yes	−	ratios, bars
pink	yes	+	*p*- , *q*-Values
light green	yes	−	

Dot Plots | Dot Plots

Protein	*FnPgSg* vs Fn Log$_2$ Ratio	Log$_2$ Sum	*q*-Value	*p*-Value	Raw *FnPgSg*	Raw *Fn*	Normalized *FnPgSg*	Normalized *Fn*	Description
FN0662	0.263	10.716	1.425e-2	6.149e-2	45	37	45.0000	41.8788	AAL94858.1| Formiminoglutamase
					35	27	44.8434	33.0031	
FN0664	-0.587	14.000	2.851e-3	9.277e-3	123	126	123.0000	142.6142	AAL94860.1| 2-nitropropane dioxygenase
					67	140	85.8430	171.1270	
FN0666						9		10.1867	AAL94862.1| Hypothetical protein
						10		12.2234	
FN0668	-1.596	8.436	1.109e-3	2.747e-3	15	28	15.0000	31.6921	AAL94864.1| High-affinity zinc uptake system protein znuA precursor
					5	27	6.4062	33.0031	
FN0672									AAL94868.1| ATPase
					9		11.5312		
FN0675	-0.047	20.137	9.545e-2	5.335e-1	974	936	974.0000	1059.4200	AAL94871.1| 60 kDa chaperonin GROEL
					889	919	1139.0219	1123.3263	
FN0676	-1.245	14.012	5.316e-4	1.079e-3	76	193	76.0000	218.4488	AAL94872.1| 10 kDa chaperonin GROES
					71	145	90.9680	177.2386	
FN0677	-1.849	10.827	1.647e-5	6.684e-6	18	76	18.0000	86.0213	AAL94873.1| Hypothetical protein
					21	62	26.9060	75.7848	
FN0678	-0.784	11.988	9.635e-5	1.029e-4	51	70	51.0000	79.2301	AAL94874.1| Ser/Thr protein kinase
					36	72	46.1246	88.0082	
FN0679	-2.037	5.922				16		18.1097	AAL94875.1| GTPase
					3	11	3.8437	13.4457	
FN0680					6		6.0000		AAL94876.1| Ribulose-phosphate 3-epimerase
					4		5.1250		
FN0681	-0.629	15.534	5.92e-4	1.237e-3	176	227	176.0000	256.9320	AAL94877.1| Transcriptional regulator, MarR family
					136	233	174.2486	284.8042	
FN0682	-0.398	8.618	4.682e-2	2.403e-1	23	24	23.0000	27.1646	AAL94878.1| Fibronectin-binding protein-like protein A
					9	15	11.5312	18.3350	
FN0684					3		3.0000		AAL94880.1| Prismane protein
FN0685	-1.816	8.059	3.693e-5	2.531e-5	11	25	11.0000	28.2965	AAL94881.1| Sodium/pantothenate symporter
					5	27	6.4062	33.0031	
FN0688	-2.434	8.604	1.698e-8	3.432e-10	8	40	8.0000	45.2744	AAL94884.1| Hypothetical protein
					7	38	8.9687	46.4487	

Log$_2$ Ratios: -6 -4 -2 0 2 4 6

Show detected proteins only
Show all proteins
Filter by category:
GO: amino acid transport

Proteins found: 1297

Enter (or paste) list of ORFs

Find ORFs

Test	Cutoff
q-Value	.005
p-Value	

	Signif	Direction	Applies To
red	yes	+	ratios, bars
yellow	no	n/a	bars
green	yes	−	ratios, bars
pink	yes	+	*p*- , *q*-Values
light green	yes	−	

Dot Plots Dot Plots

Protein	FnPgSg vs Fn				Raw		Normalized		Description	Log_2 Ratios
	Log_2 Ratio	Log_2 Sum	*q*-Value	*p*-Value	*FnPgSg*	*Fn*	*FnPgSg*	*Fn*		-6 -4 -2 0 2 4 6
FN0689	-1.277	14.954	3.587e-5	2.382e-5	120	234	120.0000	264.8550	AAL94885.1| Hypothetical protein	
					85	237	108.9054	289.6935		
FN0692	-2.211	6.096				12		13.5823	AAL94888.1| Nitrogen regulation protein NIFR3	
					3	18	3.8437	22.0020		
FN0693	-1.515	8.434			11	21	11.0000	23.7690	AAL94889.1| DNA mismatch repair protein mutS	
						32		39.1147		
FN0694	-0.881	11.895	4.819e-4	9.525e-4	41	67	41.0000	75.8345	AAL94890.1| S-layer protein	
					39	75	49.9683	91.6752		
FN0695	-0.332	9.455	4.54e-2	2.321e-1	28	32	28.0000	36.2195	AAL94891.1| ABC transporter ATP-binding protein	
					15	19	19.2186	23.2244		
FN0697	-0.279	15.429	1.298e-3	3.428e-3	202	210	202.0000	237.6904	AAL94893.1| Alanyl-tRNA synthetase	
					140	184	179.3735	224.9097		
FN0699	-0.586	13.346	2.026e-3	6.156e-3	82	101	82.0000	114.3178	AAL94895.1| Protein translocase subunit secD	
					66	111	84.5618	135.6792		
FN0700	-1.124	10.813	1.864e-3	5.557e-3	28	48	28.0000	54.3292	AAL94896.1| Protein translocase subunit secF	
					23	58	29.4685	70.8955		
FN0701	-0.241	15.049	4.958e-4	9.89e-4	176	182	176.0000	205.9983	AAL94897.1| Methyltransferase	
					127	159	162.7174	194.3513		
FN0705	-0.928	13.173	3.658e-5	2.481e-5	65	123	65.0000	139.2187	AAL94901.1| DNA polymerase I	
					58	103	74.3119	125.9006		
FN0706	-0.664	5.834			6	6	6.0000	6.7912	AAL94902.1| Hypothetical cytosolic protein	
						10		12.2234		
FN0707	-1.492	7.564	1.176e-3	2.978e-3	10	17	10.0000	19.2416	AAL94903.1| Riboflavin kinase	
					5	22	6.4062	26.8914		
FN0710	-0.639	11.457	1.889e-3	5.652e-3	44	64	44.0000	72.4390	AAL94906.1| Hypothetical protein	
					32	49	40.9997	59.8944		
FN0711	-0.279	6.543	6.685e-2	3.574e-1	6	8	6.0000	9.0549	AAL94907.1| Phosphopantothenate--cysteine ligase	
					9	10	11.5312	12.2234		
FN0715	0.854	14.829	4.094e-3	1.433e-2	264	113	264.0000	127.9001	AAL94911.1| Hypothetical protein	
					152	103	194.7484	125.9006		
FN0716	-1.301	10.767	1.141e-3	2.855e-3	25	65	25.0000	73.5708	AAL94912.1| hypothetical protein	
					22	47	28.1873	57.4498		

Show detected proteins only
Show all proteins
Filter by category:
GO: amino acid transport

Proteins found: 1297

Enter (or paste) list of ORFs

Find ORFs

Test	Cutoff
q-Value	.005
p-Value	

	Signif	Direction	Applies To
red	yes	+	ratios, bars
yellow	no	n/a	bars
green	yes	–	ratios, bars
pink	yes	+	*p*- , *q*-Values
light green	yes	–	

Dot Plots Dot Plots

Protein	FnPgSg vs Fn Log2 Ratio	Log2 Sum	q-Value	p-Value	Raw FnPgSg	Raw Fn	Normalized FnPgSg	Normalized Fn	Description
FN0717	-0.553	5.197			5		5.0000		AAL94913.1| Ribosomal small subunit pseudouridine synthase A
						6		7.3340	
FN0719						9		10.1867	AAL94915.1| Hypothetical cytosolic protein
						8		9.7787	
FN0720	-1.880	12.850	7.657e-5	7.375e-5	46	139	46.0000	157.3284	AAL94916.1| Protein Translation Elongation Factor P (EF-P)
					34	141	43.5621	172.3493	
FN0721	-1.131	11.511	2.317e-4	3.61e-4	32	70	32.0000	79.2301	AAL94917.1| Hypothetical protein
					32	66	40.9997	80.6741	
FN0722	-1.872	5.872			4	14	4.0000	15.8460	AAL94918.1| WD-repeat family protein
						11		13.4457	
FN0724	0.344	10.574	1.666e-2	7.341e-2	38	31	38.0000	35.0876	AAL94920.1| Flavodoxin
					39	28	49.9683	34.2254	
FN0725	0.016	6.642	1.604e-1	9.67e-1	6	10	6.0000	11.3186	AAL94921.1| Molybdopterin biosynthesis MoeB protein
					11	7	14.0936	8.5563	
FN0728	0.298	10.116	9.09e-4	2.113e-3	38	25	38.0000	28.2965	AAL94924.1| Hypothetical protein
					28	26	35.8747	31.7807	
FN0729	-0.521	14.939	6.743e-4	1.462e-3	146	197	146.0000	222.9762	AAL94925.1| Phosphoglycerate mutase
					117	165	149.9050	201.6854	
FN0731	-0.059	8.739	7.61e-2	4.131e-1	20	20	20.0000	22.6372	AAL94927.1| Hypothetical protein
					16	16	20.4998	19.5574	
FN0733	0.483	14.007	3.863e-5	2.793e-5	147	100	147.0000	113.1859	AAL94929.1| Peptidase T
					122	85	156.3112	103.8985	
FN0734						9		10.1867	AAL94930.1| Fe-S oxidoreductase
						9		11.0010	
FN0735	-0.054	13.439	1.183e-1	6.795e-1	89	99	89.0000	112.0540	AAL94931.1| Cell surface protein
					92	84	117.8740	102.6762	
FN0736					5		5.0000		AAL94932.1| Methyltransferase
					8		10.2499		
FN0737					15		15.0000		AAL94933.1| Hypothetical protein
					10		12.8124		
FN0738	-1.700	10.360	5.852e-5	5.076e-5	21	55	21.0000	62.2522	AAL94934.1| Hypothetical exported 24-amino acid repeat protein
					15	56	19.2186	68.4508	

Log2 Ratios: -6 -4 -2 0 2 4 6

Show detected proteins only
Show all proteins
Filter by category:
GO: amino acid transport

Proteins found: 1297

Enter (or paste) list of ORFs

Find ORFs

Test	Cutoff
q-Value	.005
p-Value	

Signif	Direction	Applies To
yes	+	ratios, bars
no	n/a	bars
yes	−	ratios, bars
yes	+	p-, q-Values
yes	−	

Dot Plots Dot Plots

Protein	*FnPgSg* vs *Fn* Log$_2$ Ratio	Log$_2$ Sum	*q*-Value	*p*-Value	Raw *FnPgSg*	Raw *Fn*	Normalized *FnPgSg*	Normalized *Fn*	Description	Log$_2$ Ratios (-6 -4 -2 0 2 4 6)
FN0739	0.591	16.351	9.206e-5	9.607e-5	338	196	338.0000	221.8444	AAL94935.1| Formiminotetrahydrofolate cyclodeaminase	
					290	204	371.5594	249.3564		
FN0740	-0.616	17.012	1.28e-4	1.542e-4	308	401	308.0000	453.8754	AAL94936.1| Imidazolonepropionase	
					218	365	279.3102	446.1525		
FN0741	0.288	18.624	2.784e-3	9.005e-3	659	525	659.0000	594.2259	AAL94937.1| Glutamate formiminotransferase	
					582	455	745.6814	556.1627		
FN0742	-0.460	11.802	2.817e-2	1.334e-1	66	72	66.0000	81.4938	AAL94938.1| unknown	
					28	48	35.8747	58.6721		
FN0743	0.211	7.022	5.456e-3	2.028e-2	13	9	13.0000	10.1867	AAL94939.1| ATP-dependent helicase, DinG family	
					9	9	11.5312	11.0010		
FN0745	-0.765	14.194	3.351e-3	1.126e-2	114	135	114.0000	152.8010	AAL94941.1| metal dependent phosphohydrolase	
					75	167	96.0930	204.1300		
FN0746						9		10.1867	AAL94942.1| Hypothetical Metal-Binding Protein	
FN0749	-0.864	5.579				10		11.3186	AAL94945.1| Hypothetical protein	
					4	6	5.1250	7.3340		
FN0750	-1.869	8.204	2.312e-4	3.598e-4	9	31	9.0000	35.0876	AAL94946.1| Hypothetical protein	
					7	25	8.9687	30.5584		
FN0751	-0.623	8.276	2.707e-3	8.698e-3	13	17	13.0000	19.2416	AAL94947.1| L-asparaginase I	
					12	20	15.3749	24.4467		
FN0752	-1.588	6.788	1.331e-4	1.632e-4	7	16	7.0000	18.1097	AAL94948.1| Proline iminopeptidase	
					4	15	5.1250	18.3350		
FN0753	1.007	12.778	5.188e-4	1.047e-3	121	44	121.0000	49.8018	AAL94949.1| Glutamyl-tRNA(Gln) amidotransferase subunit B	
					91	56	116.5928	68.4508		
FN0754	-0.034	13.692	1.354e-1	7.925e-1	130	102	130.0000	115.4496	AAL94950.1| Glutamyl-tRNA(Gln) amidotransferase subunit A	
					76	96	97.3742	117.3442		
FN0755	-0.741	11.029	1.173e-3	2.969e-3	31	58	31.0000	65.6478	AAL94951.1| Glutamyl-tRNA(Gln) amidotransferase subunit C	
					31	43	39.7184	52.5604		
FN0758	-0.951	14.496	1.389e-4	1.738e-4	111	179	111.0000	202.6028	AAL94954.1| Rod shape-determining protein mreB	
					84	180	107.6241	220.0204		
FN0761	-0.342	7.659	4.446e-2	2.267e-1	15	11	15.0000	12.4504	AAL94957.1| Bvg accessory factor	
					8	16	10.2499	19.5574		

Show detected proteins only
Show all proteins
Filter by category:
GO: amino acid transport

Proteins found: 1297

Enter (or paste) list of ORFs

Find ORFs

Test	Cutoff
q-Value	.005
p-Value	

Signif	Direction	Applies To
yes	+	ratios, bars
no	n/a	bars
yes	−	ratios, bars
yes	+	*p*-, *q*-Values
yes	−	*p*-, *q*-Values

Dot Plots | Dot Plots

Protein	*FnPgSg vs Fn* Log_2 Ratio	Log_2 Sum	*q*-Value	*p*-Value	Raw *FnPgSg*	Raw *Fn*	Normalized *FnPgSg*	Normalized *Fn*	Description
**FN0765**	-1.411	5.411			4	8	4.0000	9.0549	AAL94961.1| tRNA (5-methylaminomethyl -2-thiouridylate) -methyltransferase
						10		12.2234	
**FN0766**	0.488	8.871				15		16.9779	AAL94962.1| Large-conductance mechanosensitive channel
					20	16	25.6248	19.5574	
**FN0768**	-1.399	5.399			4	10	4.0000	11.3186	AAL94964.1| Hemin receptor
						8		9.7787	
**FN0771**									AAL94967.1| Oxygen-independent coproporphyrinogen III oxidase
						14		17.1127	
**FN0772**						7		7.9230	AAL94968.1| Flavodoxin
						3		3.6670	
**FN0774**	-0.436	14.423	9.202e-3	3.744e-2	105	148	105.0000	167.5151	AAL94970.1| Hypothetical cytosolic protein
					117	145	149.9050	177.2386	
**FN0775**	-0.699	16.200	1.849e-6	3.037e-7	218	313	218.0000	354.2719	AAL94971.1| Aspartyl aminopeptidase
					166	282	212.6857	344.6986	
**FN0776**	-0.808	12.351	1.605e-3	4.605e-3	58	75	58.0000	84.8894	AAL94972.1| Aspartate-ammonia ligase
					40	87	51.2496	106.3432	
**FN0777**	0.357	12.533	1.327e-2	5.67e-2	91	50	91.0000	56.5929	AAL94973.1| GTP-binding protein lepA
					65	65	83.2806	79.4518	
**FN0778**	-1.087	8.721				27		30.5602	AAL94974.1| Methyltransferase
					11	24	14.0936	29.3361	
**FN0783**	0.931	23.698	4.028e-3	1.405e-2	5918	2445	5918.0000	2767.3951	AAL94979.1| acyl-CoA dehydrogenase
					3331	2107	4267.8086	2575.4609	
**FN0784**	-0.082	22.427	9.113e-2	5.064e-1	1997	2170	1997.0000	2456.1339	AAL94980.1| Electron transfer flavoprotein beta-subunit
					2044	1988	2618.8534	2430.0030	
**FN0785**	-0.348	21.263	1.182e-3	2.999e-3	1458	1670	1458.0000	1890.2044	AAL94981.1| Electron transfer flavoprotein alpha-subunit
					1057	1381	1354.2701	1688.0453	
**FN0788**	0.016	8.675	1.563e-1	9.377e-1	24	15	24.0000	16.9779	AAL94984.1| unknown
					13	19	16.6561	23.2244	
**FN0790**	-1.717	6.361			5	15	5.0000	16.9779	AAL94986.1| Xylose repressor
						13		15.8904	
**FN0791**	-0.404	18.068	1.095e-3	2.699e-3	459	558	459.0000	631.5773	AAL94987.1| Histidine ammonia-lyase
					353	470	452.2775	574.4977	

Log_2 Ratios: -6 -4 -2 0 2 4 6

Show detected proteins only
Show all proteins
Filter by category:
GO: amino acid transport

Proteins found: 1297

Enter (or paste) list of ORFs

Find ORFs

Test	Cutoff
q-Value	.005
p-Value	

Color	Signif	Direction	Applies To
red	yes	+	ratios, bars
yellow	no	n/a	bars
green	yes	–	ratios, bars
pink	yes	+	*p*-, *q*-Values
light green	yes	–	*p*-, *q*-Values

Dot Plots Dot Plots

Protein	FnPgSg vs Fn: Log_2 Ratio	Log_2 Sum	*q*-Value	*p*-Value	Raw: *FnPgSg*	Raw: *Fn*	Normalized: *FnPgSg*	Normalized: *Fn*	Description
FN0792	-0.341	19.854	2.672e-4	4.339e-4	823	1002	823.0000	1134.1227	AAL94988.1| Urocanate hydratase
					708	865	907.1175	1057.3202	
FN0793	0.585	9.049				17		19.2416	AAL94989.1| Sodium/glutamate symport carrier protein
					22	15	28.1873	18.3350	
FN0794	-0.428	6.043			7	8	7.0000	9.0549	AAL94990.1| Hypothetical protein
						8		9.7787	
FN0796	-0.552	14.481	1.323e-4	1.618e-4	128	168	128.0000	190.1523	AAL94992.1| Pyruvate,phosphate dikinase
					95	144	121.7177	176.0163	
FN0798	0.280	7.582	1.017e-1	5.729e-1	10	6	10.0000	6.7912	AAL94994.1| Fructose-1,6-bisphosphatase
					16	15	20.4998	18.3350	
FN0799	-0.172	7.573			13	14	13.0000	15.8460	AAL94995.1| Isoamylase
						11		13.4457	
FN0800	1.398	11.810	1.159e-3	2.917e-3	110	35	110.0000	39.6151	AAL94996.1| Amino acid-binding protein
					66	28	84.5618	34.2254	
FN0801						9		10.1867	AAL94997.1| Amino acid transport ATP-binding protein
						9		11.0010	
FN0803	-1.139	12.385	3.573e-3	1.215e-2	55	114	55.0000	129.0319	AAL94999.1| Cytochrome C-TYPE biogenesis protein ccdA
					34	72	43.5621	88.0082	
FN0805	-0.640	4.583			4		4.0000		AAL95001.1| Hypothetical protein
					3	5	3.8437	6.1117	
FN0806	-0.680	13.041	1.185e-3	3.01e-3	81	92	81.0000	104.1310	AAL95002.1| SpoIID homolog
					50	105	64.0620	128.3452	
FN0807	-0.330	9.925	4.398e-2	2.24e-1	30	38	30.0000	43.0106	AAL95003.1| 3-deoxy-manno-octulosonate cytidylyltransferase
					20	22	25.6248	26.8914	
FN0808	-0.392	11.325	4.92e-3	1.79e-2	50	55	50.0000	62.2522	AAL95004.1| Phosphoglycerate mutase
					30	44	38.4372	53.7828	
FN0809	-1.127	8.045			11	23	11.0000	26.0328	AAL95005.1| 23S rRNA methyltransferase
						18		22.0020	
FN0810	-1.644	9.813	1.044e-4	1.167e-4	16	44	16.0000	49.8018	AAL95006.1| Low-specificity threonine aldolase
					14	46	17.9374	56.2274	
FN0813	-0.317	6.632	9.265e-2	5.159e-1	14	11	14.0000	12.4504	AAL95009.1| Transcriptional regulator, TetR family
					3	8	3.8437	9.7787	

Log_2 Ratios: -6 -4 -2 0 2 4 6

Show detected proteins only
Show all proteins
Filter by category:
GO: amino acid transport

Proteins found: 1297

Enter (or paste) list of ORFs

Find ORFs

Test	Cutoff
q-Value	.005
p-Value	

Color	Signif	Direction	Applies To
red	yes	+	ratios, bars
yellow	no	n/a	bars
green	yes	−	ratios, bars
light red	yes	+	*p*- , *q*-Values
light green	yes	−	

Dot Plots Dot Plots

Protein	FnPgSg vs Fn Log2 Ratio	Log2 Sum	q-Value	p-Value	Raw FnPgSg	Raw Fn	Normalized FnPgSg	Normalized Fn	Description
FN0814	-0.729	16.413	3.378e-5	2.113e-5	241	322	241.0000	364.4586	AAL95010.1| Propionate CoA-transferase
					170	324	217.8107	396.0367	
FN0815	0.171	11.483	1.807e-2	8.055e-2	61	47	61.0000	53.1974	AAL95011.1| Propionate permease
					41	39	52.5308	47.6711	
FN0816	-1.830	14.326	1.691e-5	6.994e-6	70	228	70.0000	258.0638	AAL95012.1| dehydrogenase with MaoC-like domain
					64	231	81.9993	282.3595	
FN0818	-0.030	19.677	1.22e-1	7.032e-1	852	761	852.0000	861.3447	AAL95014.1| DNA-binding protein HU
					749	809	959.6483	988.8694	
FN0819	-0.003	12.089	1.633e-1	9.878e-1	55	53	55.0000	59.9885	AAL95015.1| Tetratricopeptide repeat family protein
					60	59	76.8744	72.1178	
FN0820	-0.244	14.313	7.001e-3	2.717e-2	120	134	120.0000	151.6691	AAL95016.1| Mercuric reductase
					111	130	142.2176	158.9036	
FN0821	-1.142	12.084	2.269e-4	3.499e-4	40	79	40.0000	89.4169	AAL95017.1| Hypothetical protein
					38	87	48.6871	106.3432	
FN0823	0.170	8.375	8.063e-2	4.41e-1	22	12	22.0000	13.5823	AAL95019.1| GTP-binding protein hflX
					13	17	16.6561	20.7797	
FN0825	-1.529	11.525	7.45e-5	7.091e-5	37	83	37.0000	93.9443	AAL95021.1| Hypothetical cytosolic protein
					21	74	26.9060	90.4528	
FN0826	-0.526	10.521	1.786e-3	5.264e-3	28	37	28.0000	41.8788	AAL95022.1| periplasmic component of efflux system
					28	41	35.8747	50.1158	
FN0827	-1.579	9.833	4.789e-3	1.733e-2	17	35	17.0000	39.6151	AAL95023.1| ABC transporter ATP-binding protein
					14	53	17.9374	64.7838	
FN0828	-1.647	6.817			6	17	6.0000	19.2416	AAL95024.1| ABC transporter permease protein
						15		18.3350	
FN0830	-1.340	8.606	4.172e-3	1.467e-2	12	22	12.0000	24.9009	AAL95026.1| Hypothetical protein
					10	31	12.8124	37.8924	
FN0832	0.226	12.551	1.779e-2	7.913e-2	92	65	92.0000	73.5708	AAL95028.1| Hypothetical protein
					59	57	75.5931	69.6731	
FN0833	-2.045	9.517	7.314e-4	1.613e-3	10	41	10.0000	46.4062	AAL95029.1| Hypothetical protein
					13	52	16.6561	63.5614	
FN0834	-0.963	9.550	1.062e-5	3.442e-6	20	33	20.0000	37.3513	AAL95030.1| Hypothetical Exported Protein
					15	32	19.2186	39.1147	

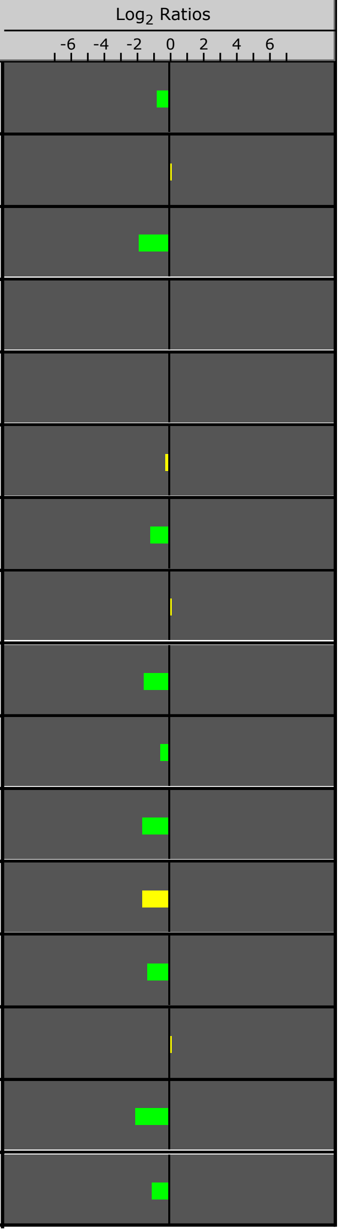

Show detected proteins only
Show all proteins
Filter by category:
GO: amino acid transport

Proteins found: 1297

Enter (or paste) list of ORFs

Find ORFs

Test	Cutoff
q-Value	.005
p-Value	

Signif	Direction	Applies To
yes	+	ratios, bars
no	n/a	bars
yes	−	ratios, bars
yes	+	p- , q-Values
yes	−	

Dot Plots Dot Plots

Protein	*FnPgSg vs Fn* Log_2 Ratio	Log_2 Sum	*q*-Value	*p*-Value	Raw *FnPgSg*	Raw *Fn*	Normalized *FnPgSg*	Normalized *Fn*	Description
FN0836	0.384	8.006	7.241e-2	3.906e-1	11	14	11.0000	15.8460	AAL95032.1| Hypothetical protein
					20	10	25.6248	12.2234	
FN0846	-1.032	12.029	1.323e-3	3.524e-3	43	91	43.0000	102.9992	AAL95042.1| Hypothetical Exported Protein
					37	67	47.4059	81.8965	
FN0847	-1.892	7.222	1.49e-5	5.662e-6	5	20	5.0000	22.6372	AAL95043.1| TPR-repeat-containing proteins
					6	20	7.6874	24.4467	
FN0849	-0.787	9.278	3.527e-3	1.196e-2	20	33	20.0000	37.3513	AAL95045.1| 8-amino-7-oxononanoate synthase
					14	23	17.9374	28.1137	
FN0850	-1.330	7.402	1.025e-4	1.134e-4	10	17	10.0000	19.2416	AAL95046.1| Hypothetical cytosolic protein
					5	18	6.4062	22.0020	
FN0853	-0.782	10.665	6.092e-6	1.522e-6	32	48	32.0000	54.3292	AAL95049.1| Glycogen synthase
					23	42	29.4685	51.3381	
FN0854	0.226	11.489	3.804e-2	1.898e-1	66	39	66.0000	44.1425	AAL95050.1| Glucose-1-phosphate adenylyltransferase
					39	45	49.9683	55.0051	
FN0855	-0.839	13.027	4.245e-7	3.817e-8	70	109	70.0000	123.3726	AAL95051.1| Glucose-1-phosphate adenylyltransferase
					52	99	66.6245	121.0112	
FN0856	-0.627	11.514	7.566e-3	2.973e-2	55	55	55.0000	62.2522	AAL95052.1| 1,4-alpha-glucan branching enzyme
					25	59	32.0310	72.1178	
FN0857	-0.346	14.955	3.947e-3	1.37e-2	165	192	165.0000	217.3169	AAL95053.1| Glycogen phosphorylase
					118	151	151.1863	184.5727	
FN0858	-2.182	7.017	4.324e-4	8.285e-4	3	18	3.0000	20.3735	AAL95054.1| 4-alpha-glucanotransferase
					6	23	7.6874	28.1137	
FN0865	-0.763	9.598	1.47e-2	6.369e-2	12	36	12.0000	40.7469	AAL95061.1| unknown
					24	26	30.7497	31.7807	
FN0867	-0.731	13.510	4.801e-5	3.853e-5	78	126	78.0000	142.6142	AAL95063.1| Long-chain-fatty-acid--CoA ligase
					70	111	89.6868	135.6792	
FN0868						8		9.0549	AAL95064.1| ATPases of the PP superfamily
						5		6.1117	
FN0869	-1.027	4.197			3		3.0000		AAL95065.1| Hydrolase (HAD superfamily)
						5		6.1117	
FN0870	0.817	6.040			10		10.0000		AAL95066.1| Rhodanese-related sulfurtransferases
					9	5	11.5312	6.1117	

Log_2 Ratios: -6 -4 -2 0 2 4 6

Show detected proteins only
Show all proteins
Filter by category:
GO: amino acid transport

Proteins found: 1297

Enter (or paste) list of ORFs

Find ORFs

Test	Cutoff
q-Value	.005
p-Value	

	Signif	Direction	Applies To
red	yes	+	ratios, bars
yellow	no	n/a	bars
green	yes	–	ratios, bars
pink	yes	+	*p*- , *q*-Values
light green	yes	–	

Dot Plots Dot Plots

Protein	*FnPgSg* vs *Fn* Log$_2$ Ratio	Log$_2$ Sum	*q*-Value	*p*-Value	Raw *FnPgSg*	Raw *Fn*	Normalized *FnPgSg*	Normalized *Fn*	Description
FN0871	-0.535	6.875			9	9	9.0000	10.1867	AAL95067.1| 3-dehydroquinate synthase
						13		15.8904	
FN0873	-0.043	8.609	1.209e-1	6.963e-1	21	16	21.0000	18.1097	AAL95069.1| Protease IV
					14	18	17.9374	22.0020	
FN0875					5		5.0000		AAL95071.1| 23S rRNA methyltransferase
FN0878	0.358	8.934	1.732e-2	7.67e-2	27	14	27.0000	15.8460	AAL95074.1| Transcriptional regulator, GntR family
					18	19	23.0623	23.2244	
FN0885									AAL95081.1| Hemin-binding periplasmic protein hmuT precursor
						10		12.2234	
FN0886	-2.221	5.391			3	15	3.0000	16.9779	AAL95082.1| Hemin receptor
						9		11.0010	
FN0887	-0.665	11.445	1.368e-3	3.7e-3	48	57	48.0000	64.5160	AAL95083.1| Oligoendopeptidase F
					28	56	35.8747	68.4508	
FN0888	-0.277	9.804	4.042e-2	2.037e-1	21	29	21.0000	32.8239	AAL95084.1| Uracil permease
					26	27	33.3122	33.0031	
FN0889						9		10.1867	AAL95085.1| hypothetical protein
						7		8.5563	
FN0893	-0.745	7.623	5.864e-3	2.207e-2	14	17	14.0000	19.2416	AAL95089.1| Hypothetical protein
					6	14	7.6874	17.1127	
FN0896	-0.412	8.220	1.307e-2	5.572e-2	12	19	12.0000	21.5053	AAL95092.1| Hypothetical protein
					14	15	17.9374	18.3350	
FN0900						4		4.5274	AAL95096.1| Metal dependent hydrolase
						5		6.1117	
FN0901									AAL95097.1| DNA polymerase, bacteriophage-type
						5		6.1117	
FN0902					6		6.0000		AAL95098.1| 5-formyltetrahydrofolate cyclo-ligase
					8		10.2499		
FN0903	0.458	7.418	3.434e-3	1.158e-2	14	10	14.0000	11.3186	AAL95099.1| Polysialic acid capsule expression protein kpsF
					13	9	16.6561	11.0010	
FN0905	-0.658	7.805	1.363e-4	1.691e-4	11	17	11.0000	19.2416	AAL95101.1| Hypothetical protein
					10	15	12.8124	18.3350	

Log$_2$ Ratios: -6 -4 -2 0 2 4 6

Show detected proteins only
Show all proteins
Filter by category:
GO: amino acid transport

Proteins found: 1297

Enter (or paste) list of ORFs

Find ORFs

Test	Cutoff
q-Value	.005
p-Value	

	Signif	Direction	Applies To
red	yes	+	ratios, bars
yellow	no	n/a	bars
green	yes	−	ratios, bars
pink	yes	+	*p*-, *q*-Values
light green	yes	−	*p*-, *q*-Values

Dot Plots Dot Plots

Protein	FnPgSg vs Fn Log$_2$ Ratio	Log$_2$ Sum	*q*-Value	*p*-Value	Raw *FnPgSg*	Raw *Fn*	Normalized *FnPgSg*	Normalized *Fn*	Description
FN0906	-1.134	8.049	5.811e-4	1.208e-3	13	21	13.0000	23.7690	AAL95102.1| Glycerol-3-phosphate dehydrogenase [NAD(P)+]
					7	20	8.9687	24.4467	
FN0908	-0.797	8.726	1.811e-3	5.357e-3	12	22	12.0000	24.9009	AAL95104.1| Tpl protein
					15	24	19.2186	29.3361	
FN0910						3		3.3956	AAL95106.1| Nicotinate-nucleotide--dimethylbenzimidazole phosphoribosyltransferase
						9		11.0010	
FN0911	-0.519	6.133			7	8	7.0000	9.0549	AAL95107.1| Alpha-ribazole-5'-phosphate phosphatase
						9		11.0010	
FN0913	-1.458	4.628			3	7	3.0000	7.9230	AAL95109.1| Cobinamide kinase
						7		8.5563	
FN0915	0.092	9.413	1.574e-3	4.491e-3	27	22	27.0000	24.9009	AAL95111.1| PTS system, N-acetylglucosamine-specific IIA component
					21	21	26.9060	25.6690	
FN0916	0.625	15.448	5.657e-3	2.116e-2	301	141	301.0000	159.5921	AAL95112.1| Hypothetical Exported Protein
					175	148	224.2169	180.9057	
FN0917	-1.390	5.275				7		7.9230	AAL95113.1| Hypothetical protein
					3	10	3.8437	12.2234	
FN0920	-0.352	5.842			7		7.0000		AAL95116.1| Protease HTPX
					5	7	6.4062	8.5563	
FN0921	0.422	6.319	4.465e-2	2.278e-1	13	5	13.0000	5.6593	AAL95117.1| Hypothetical protein
					6	8	7.6874	9.7787	
FN0922					3		3.0000		AAL95118.1| Homoserine kinase
FN0924									AAL95120.1| Hypothetical protein
					3		3.8437		
FN0925	-0.431	6.553	2.228e-2	1.019e-1	9	8	9.0000	9.0549	AAL95121.1| Hypothetical protein
					6	11	7.6874	13.4457	
FN0926	0.381	5.908			10	6	10.0000	6.7912	AAL95122.1| GTP pyrophosphokinase
					6		7.6874		
FN0928	-0.420	5.552			8	7	8.0000	7.9230	AAL95124.1| O-sialoglycoprotein endopeptidase
					3		3.8437		
FN0929									AAL95125.1| ATP/GTP hydrolase
					6		7.6874		

Log$_2$ Ratios: -6 -4 -2 0 2 4 6

Show detected proteins only
Show all proteins
Filter by category:
GO: amino acid transport

Proteins found: 1297

Enter (or paste) list of ORFs

Find ORFs

Test	Cutoff
q-Value	.005
p-Value	

	Signif	Direction	Applies To
red	yes	+	ratios, bars
yellow	no	n/a	bars
green	yes	−	ratios, bars
pink	yes	+	*p*-, *q*-Values
light green	yes	−	

Dot Plots Dot Plots

Protein	*FnPgSg vs Fn* Log$_2$ Ratio	Log$_2$ Sum	*q*-Value	*p*-Value	Raw *FnPgSg*	Raw *Fn*	Normalized *FnPgSg*	Normalized *Fn*	Description
FN0930						16		18.1097	AAL95126.1| Glycerol-3-phosphate cytidylyltransferase
						14		17.1127	
FN0932	-1.612	6.563	8.808e-5	8.998e-5	6	16	6.0000	18.1097	AAL95128.1| Hypothetical protein
					4	13	5.1250	15.8904	
FN0933					3		3.0000		AAL95129.1| 3-phosphoshikimate 1-carboxyvinyltransferase
FN0934						8		9.0549	AAL95130.1| Chorismate synthase
						6		7.3340	
FN0940					10		10.0000		AAL95136.1| Hypothetical protein
					9		11.5312		
FN0941	0.493	9.225	3.183e-3	1.059e-2	26	17	26.0000	19.2416	AAL95137.1| Gamma-glutamyltranspeptidase
					25	18	32.0310	22.0020	
FN0943	-0.898	5.187	1.816e-4	2.518e-4	5	7	5.0000	7.9230	AAL95139.1| Sensory Transduction Protein Kinase
					3	7	3.8437	8.5563	
FN0947	0.067	13.138	1.119e-1	6.376e-1	88	71	88.0000	80.3620	AAL95143.1| Hypothetical protein
					83	86	106.3429	105.1209	
FN0949	-0.722	15.710	1.517e-4	1.958e-4	185	251	185.0000	284.0966	AAL95145.1| DNA helicase
					137	254	175.5298	310.4732	
FN0951						16		18.1097	AAL95147.1| Precorrin-3B C17-methyltransferase
						13		15.8904	
FN0952									AAL95148.1| Cobalamin biosynthesis protein G
						6		7.3340	
FN0957	0.546	8.300	2.589e-2	1.21e-1	16	13	16.0000	14.7142	AAL95153.1| Precorrin-4 C11-methyltransferase
					21	12	26.9060	14.6680	
FN0958						23		26.0328	AAL95154.1| unknown
						18		22.0020	
FN0959	-1.205	8.846	2.769e-3	8.945e-3	18	23	18.0000	26.0328	AAL95155.1| Precorrin-2 C20-methyltransferase
					8	32	10.2499	39.1147	
FN0961						4		4.5274	AAL95157.1| Hypothetical protein
						6		7.3340	
FN0962	-1.249	9.795	3.758e-5	2.628e-5	22	38	22.0000	43.0106	AAL95158.1| Hypothetical cytosolic protein
					13	40	16.6561	48.8934	

Log$_2$ Ratios: -6 -4 -2 0 2 4 6

Show detected proteins only
Show all proteins
Filter by category:
GO: amino acid transport

Proteins found: 1297

Enter (or paste) list of ORFs

Find ORFs

Test	Cutoff
q-Value	.005
p-Value	

Signif	Direction	Applies To
yes	+	ratios, bars
no	n/a	bars
yes	–	ratios, bars
yes	+	*p*-, *q*-Values
yes	–	

Dot Plots Dot Plots

Protein	FnPgSg vs Fn: Log_2 Ratio	Log_2 Sum	*q*-Value	*p*-Value	Raw: *FnPgSg*	Raw: *Fn*	Normalized: *FnPgSg*	Normalized: *Fn*	Description
FN0964									AAL95160.1| Precorrin-8W decarboxylase
					5		6.4062		
FN0965	0.503	10.266	2.95e-2	1.408e-1	31	24	31.0000	27.1646	AAL95161.1| D-3-phosphoglycerate dehydrogenase
					41	26	52.5308	31.7807	
FN0967					3		3.0000		AAL95163.1| CbiD protein
FN0970	-0.337	8.543	4.225e-3	1.49e-2	19	20	19.0000	22.6372	AAL95166.1| Precorrin-8X methylmutase
					12	17	15.3749	20.7797	
FN0976	0.098	10.525	2.476e-2	1.149e-1	41	31	41.0000	35.0876	AAL95172.1| Hypothetical protein
					30	32	38.4372	39.1147	
FN0977	0.906	7.263			16	8	16.0000	9.0549	AAL95173.1| Cobyric acid synthase
					14		17.9374		
FN0981	0.225	13.832	1.476e-2	6.401e-2	142	98	142.0000	110.9222	AAL95177.1| Phosphoribosylamine--glycine ligase
					93	92	119.1553	112.4549	
FN0982	-0.629	15.961	6.087e-4	1.282e-3	223	272	223.0000	307.8656	AAL95178.1| Phosphoribosylaminoimidazolecarboxamide formyltransferase
					143	262	183.2172	320.2519	
FN0983	-0.751	13.862	3.496e-5	2.26e-5	101	135	101.0000	152.8010	AAL95179.1| Hypothetical protein
					68	134	87.1243	163.7930	
FN0984	-1.455	7.790	2.059e-4	3.036e-4	9	23	9.0000	26.0328	AAL95180.1| Tetracenomycin polyketide synthesis O-methyltransferase tcmP
					7	19	8.9687	23.2244	
FN0985	0.822	7.069	5.329e-3	1.973e-2	18	10	18.0000	11.3186	AAL95181.1| Phosphoribosylglycinamide formyltransferase
					10	5	12.8124	6.1117	
FN0986	0.300	14.458	9.588e-3	3.932e-2	151	119	151.0000	134.6912	AAL95182.1| Phosphoribosylformylglycinamidine cyclo-ligase
					142	111	181.9360	135.6792	
FN0987	-0.848	13.270	1.14e-3	2.853e-3	70	106	70.0000	119.9770	AAL95183.1| Amidophosphoribosyltransferase
					61	120	78.1556	146.6803	
FN0988	0.276	16.528	9.733e-4	2.308e-3	351	243	351.0000	275.0417	AAL95184.1| Phosphoribosylamidoimidazole-succinocarboxamide synthase
					254	232	325.4348	283.5818	
FN0989	0.366	14.907	2.992e-3	9.854e-3	184	141	184.0000	159.5921	AAL95185.1| Phosphoribosylaminoimidazole carboxylase catalytic subunit
					167	122	213.9670	149.1249	
FN0990	-0.210	20.455	1.551e-3	4.408e-3	1063	1164	1063.0000	1317.4838	AAL95186.1| Phosphoribosylformylglycinamidine synthase
					910	1032	1165.9279	1261.4502	

Log_2 Ratios: -6 -4 -2 0 2 4 6

Show detected proteins only
Show all proteins
Filter by category:
GO: amino acid transport

Proteins found: 1297

Enter (or paste) list of ORFs

Find ORFs

Test	Cutoff
q-Value	.005
p-Value	

	Signif	Direction	Applies To
red	yes	+	ratios, bars
yellow	no	n/a	bars
green	yes	−	ratios, bars
light red	yes	+	*p*- , *q*-Values
light green	yes	−	*p*- , *q*-Values

Dot Plots Dot Plots

Protein	*FnPgSg* vs *Fn* Log$_2$ Ratio	Log$_2$ Sum	*q*-Value	*p*-Value	Raw *FnPgSg*	Raw *Fn*	Normalized *FnPgSg*	Normalized *Fn*	Description
FN0991	0.894	9.003	2.917e-3	9.545e-3	31	11	31.0000	12.4504	AAL95187.1| CDP-diacylglycerol--serine O-phosphatidyltransferase
					24	17	30.7497	20.7797	
FN0992	-0.556	9.721	1.545e-3	4.381e-3	21	32	21.0000	36.2195	AAL95188.1| ADP-heptose:LPS heptosyltransferase II
					21	28	26.9060	34.2254	
FN0994	0.167	14.088	6.077e-4	1.279e-3	140	112	140.0000	126.7682	AAL95190.1| Hypothetical protein
					109	100	139.6551	122.2336	
FN0997	-0.204	9.873	5.1e-2	2.645e-1	34	30	34.0000	33.9558	AAL95193.1| Hypothetical protein
					18	26	23.0623	31.7807	
FN0998	-0.900	13.567	7.919e-4	1.777e-3	69	132	69.0000	149.4054	AAL95194.1| Dipeptide-binding protein
					72	124	92.2492	151.5696	
FN0999	-1.794	7.434	1.731e-3	5.06e-3	9	26	9.0000	29.4283	AAL95195.1| Deblocking aminopeptidase
					4	16	5.1250	19.5574	
FN1000	-0.673	11.477	3.788e-4	6.974e-4	41	63	41.0000	71.3071	AAL95196.1| Biotin synthase
					34	52	43.5621	63.5614	
FN1001	-0.383	9.074	1.276e-2	5.425e-2	24	22	24.0000	24.9009	AAL95197.1| Dethiobiotin synthetase
					13	23	16.6561	28.1137	
FN1002	-0.790	11.096	8.906e-4	2.059e-3	34	59	34.0000	66.7797	AAL95198.1| Adenosylmethionine-8-amino -7-oxononanoate aminotransferase
					29	46	37.1559	56.2274	
FN1003	0.334	10.456	1.132e-2	4.74e-2	47	32	47.0000	36.2195	AAL95199.1| Outer membrane protein P1 precursor
					29	25	37.1559	30.5584	
FN1004	-0.465	7.544	4.058e-2	2.047e-1	13	10	13.0000	11.3186	AAL95200.1| Transcriptional regulator, TetR family
					8	17	10.2499	20.7797	
FN1005	-1.648	11.090	2.823e-5	1.529e-5	22	78	22.0000	88.2850	AAL95201.1| Hypothetical protein
					24	63	30.7497	77.0071	
FN1008						4		4.5274	AAL95204.1| Hypothetical protein
FN1009						8		9.0549	AAL95205.1| Hypothetical protein
						3		3.6670	
FN1010	0.075	14.592	3.787e-2	1.888e-1	165	128	165.0000	144.8779	AAL95206.1| Hypothetical cytosolic protein
					123	132	157.5925	161.3483	
FN1011	-1.140	5.855				7		7.9230	AAL95207.1| MGPA protein
					4	12	5.1250	14.6680	

Log$_2$ Ratios: -6 -4 -2 0 2 4 6

Show detected proteins only
Show all proteins
Filter by category:
GO: amino acid transport

Proteins found: 1297

Enter (or paste) list of ORFs

Find ORFs

Test	Cutoff
q-Value	.005
p-Value	

Signif	Direction	Applies To
yes	+	ratios, bars
no	n/a	bars
yes	−	ratios, bars
yes	+	*p*- , *q*-Values
yes	−	

Dot Plots Dot Plots

Protein	*FnPgSg* vs Fn: Log_2 Ratio	Log_2 Sum	*q*-Value	*p*-Value	Raw *FnPgSg*	Raw *Fn*	Normalized *FnPgSg*	Normalized *Fn*	Description
FN1012	-0.881	7.661	8.138e-4	1.838e-3	12	19	12.0000	21.5053	AAL95208.1| HPR(Ser) kinase
					7	14	8.9687	17.1127	
FN1014	-1.798	7.064	2.864e-3	9.327e-3	6	23	6.0000	26.0328	AAL95210.1| Folylpolyglutamate synthase
					5	14	6.4062	17.1127	
FN1015	-0.513	8.696	2.865e-2	1.361e-1	20	16	20.0000	18.1097	AAL95211.1| 5'-methylthioadenosine nucleosidase
					11	25	14.0936	30.5584	
FN1016	-1.958	7.224	1.7e-7	8.462e-9	6	21	6.0000	23.7690	AAL95212.1| Lipid A biosynthesis lauroyl acyltransferase
					5	20	6.4062	24.4467	
FN1017	-0.389	13.554	1.469e-3	4.093e-3	93	104	93.0000	117.7133	AAL95213.1| Hypothetical Exported Protein
					77	109	98.6554	133.2346	
FN1019	1.629	22.153	4.008e-4	7.532e-4	3484	1077	3484.0000	1219.0121	AAL95215.1| 3-hydroxybutyryl-CoA dehydrogenase
					3209	1012	4111.4974	1237.0035	
FN1020	0.845	16.396	4.69e-3	1.689e-2	331	195	331.0000	220.7125	AAL95216.1| 3-hydroxybutyryl-CoA dehydratase
					356	178	456.1212	217.5757	
FN1022	-1.792	9.007	6.154e-3	2.337e-2	9	26	9.0000	29.4283	AAL95218.1| Calcium-transporting ATPase
					12	45	15.3749	55.0051	
FN1023					7		7.0000		AAL95219.1| 5-Nitroimidazole antibiotic resistance protein
FN1024	-0.164	20.387	8.373e-2	4.604e-1	1379	1079	1379.0000	1221.2758	AAL95220.1| DNA-binding protein HU
					651	1029	834.0869	1257.7832	
FN1025	0.114	7.736	6.586e-2	3.515e-1	15	14	15.0000	15.8460	AAL95221.1| Guanine-hypoxanthine permease
					12	10	15.3749	12.2234	
FN1026						14		15.8460	AAL95222.1| tRNA pseudouridine synthase A
						6		7.3340	
FN1028	-0.275	10.505	5.765e-2	3.036e-1	36	46	36.0000	52.0655	AAL95224.1| Deoxyuridine 5'-triphosphate nucleotidohydrolase
					26	26	33.3122	31.7807	
FN1029	-0.888	12.803	6.058e-4	1.274e-3	73	106	73.0000	119.9770	AAL95225.1| Zinc protease
					40	90	51.2496	110.0102	
FN1030	1.236	4.764			8	3	8.0000	3.3956	AAL95226.1| Hypothetical membrane-spanning protein
FN1031	-1.211	5.096				6		6.7912	AAL95227.1| Hypothetical membrane-spanning protein
					3	9	3.8437	11.0010	

Log_2 Ratios

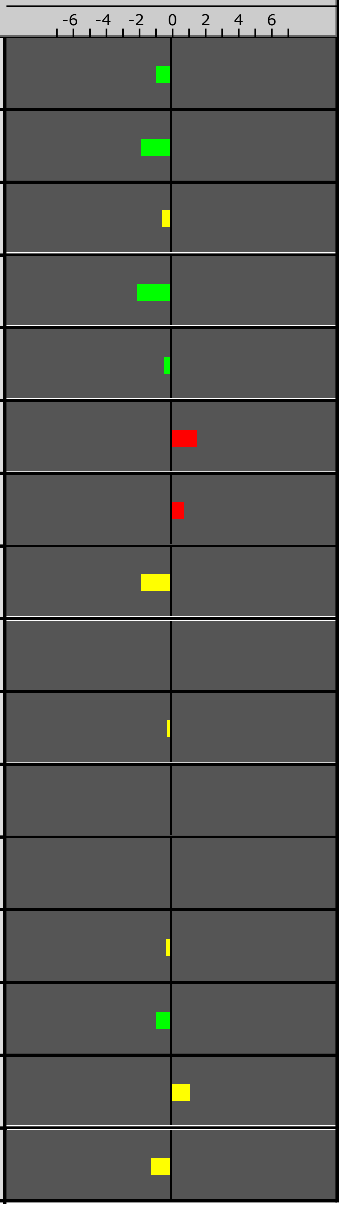

Show detected proteins only
Show all proteins
Filter by category:
GO: amino acid transport

Proteins found: 1297

Enter (or paste) list of ORFs

Find ORFs

Test	Cutoff
q-Value	.005
p-Value	

	Signif	Direction	Applies To
red	yes	+	ratios, bars
yellow	no	n/a	bars
green	yes	−	ratios, bars
pink	yes	+	*p*- , *q*-Values
light green	yes	−	

Dot Plots Dot Plots

Protein	*FnPgSg vs Fn* Log$_2$ Ratio	Log$_2$ Sum	*q*-Value	*p*-Value	Raw *FnPgSg*	Raw *Fn*	Normalized *FnPgSg*	Normalized *Fn*	Description
**FN1033**	-1.756	10.912	9.872e-4	2.351e-3	26	81	26.0000	91.6806	AAL95229.1| Methyltransferase
					17	57	21.7811	69.6731	
**FN1034**					4		4.0000		AAL95230.1| Transcriptional regulator, TetR family
**FN1037**					3		3.0000		AAL95233.1| Hypothetical cytosolic protein
**FN1041**					8		8.0000		AAL95237.1| Acetyltransferase
					7		8.9687		
**FN1042**	-2.360	11.283	1.743e-4	2.372e-4	21	107	21.0000	121.1089	AAL95238.1| S1 RNA binding domain
					18	86	23.0623	105.1209	
**FN1045**						7		7.9230	AAL95241.1| Hypothetical protein
						4		4.8893	
**FN1048**					3		3.0000		AAL95244.1| Hypothetical membrane-spanning protein
**FN1050**						7		7.9230	AAL95246.1| Lactoylglutathione lyase
**FN1055**	-0.421	10.944	7.886e-3	3.12e-2	37	40	37.0000	45.2744	AAL95251.1| Cysteine synthase
					31	47	39.7184	57.4498	
**FN1057**						8		9.0549	AAL95253.1| Diamine acetyltransferase
						6		7.3340	
**FN1060**	-0.041	9.198	1.263e-1	7.317e-1	26	24	26.0000	27.1646	AAL95256.1| hypothetical cytosolic protein
					17	18	21.7811	22.0020	
**FN1062**	0.379	13.417	1.426e-3	3.923e-3	113	80	113.0000	90.5487	AAL95258.1| Hydrolase
					98	76	125.5615	92.8975	
**FN1063**	-1.399	5.399			4	10	4.0000	11.3186	AAL95259.1| N-acyl-L-amino acid amidohydrolase
						8		9.7787	
**FN1066**	0.018	6.103	1.388e-1	8.16e-1	9	7	9.0000	7.9230	AAL95262.1| Exodeoxyribonuclease VII large subunit
					6	7	7.6874	8.5563	
**FN1067**	-1.263	11.258	4.003e-4	7.519e-4	28	75	28.0000	84.8894	AAL95263.1| Tetratricopeptide repeat family protein
					28	56	35.8747	68.4508	
**FN1069**	-0.952	11.939	2.083e-4	3.089e-4	44	73	44.0000	82.6257	AAL95265.1| DNA topoisomerase I
					36	75	46.1246	91.6752	

Log$_2$ Ratios: -6 -4 -2 0 2 4 6

Show detected proteins only
Show all proteins
Filter by category:
GO: amino acid transport

Proteins found: 1297

Enter (or paste) list of ORFs

Find ORFs

Test	Cutoff
q-Value	.005
p-Value	

	Signif	Direction	Applies To
red	yes	+	ratios, bars
yellow	no	n/a	bars
green	yes	–	ratios, bars
pink	yes	+	*p*- , *q*-Values
light green	yes	–	

Dot Plots Dot Plots

Protein	FnPgSg vs Fn Log2 Ratio	Log2 Sum	q-Value	p-Value	Raw FnPgSg	Raw Fn	Normalized FnPgSg	Normalized Fn	Description
FN1070	-0.598	8.041	3.333e-3	1.118e-2	11	18	11.0000	20.3735	AAL95266.1| Glucose inhibited division protein A
					12	16	15.3749	19.5574	
FN1071	0.295	4.875			6		6.0000		AAL95267.1| Integrase/recombinase
						4		4.8893	
FN1072	-1.230	10.601	5.007e-5	4.1e-5	22	57	22.0000	64.5160	AAL95268.1| GTP-binding protein
					23	46	29.4685	56.2274	
FN1073	-1.682	5.567				11		12.4504	AAL95269.1| Hypothetical protein
					3	10	3.8437	12.2234	
FN1074	-0.890	9.292	1.448e-3	4.01e-3	15	30	15.0000	33.9558	AAL95270.1| Signal recognition particle receptor FtsY
					17	28	21.7811	34.2254	
FN1077	2.394	8.366			41	7	41.0000	7.9230	AAL95273.1| Hypothetical protein
					33		42.2809		
FN1078	-0.160	14.734	5.6e-2	2.94e-1	133	142	133.0000	160.7240	AAL95274.1| Hypothetical exported 24-amino acid repeat protein
					140	154	179.3735	188.2397	
FN1079	0.561	17.838	1.771e-3	5.207e-3	638	364	638.0000	411.9967	AAL95275.1| Neutrophil-activating protein A
					420	315	538.1206	385.0357	
FN1080	-0.224	5.175	3.174e-2	1.536e-1	6	5	6.0000	5.6593	AAL95276.1| Export ABC transporter
					4	6	5.1250	7.3340	
FN1081	-0.891	9.327	1.83e-3	5.426e-3	18	34	18.0000	38.4832	AAL95277.1| unknown
					15	25	19.2186	30.5584	
FN1082					4		4.0000		AAL95278.1| unknown
FN1084	-0.039	13.144	1.443e-1	8.537e-1	75	97	75.0000	109.7903	AAL95280.1| unknown
					88	68	112.7491	83.1188	
FN1085	0.163	11.123	7.055e-2	3.794e-1	41	40	41.0000	45.2744	AAL95281.1| 4-methyl-5(B-hydroxyethyl)-thiazole monophosphate biosynthesis enzyme
					46	36	58.9370	44.0041	
FN1086	0.388	6.489	5.178e-2	2.69e-1	14	6	14.0000	6.7912	AAL95282.1| Transporter
					6	8	7.6874	9.7787	
FN1088	0.379	9.704	3.74e-3	1.283e-2	30	21	30.0000	23.7690	AAL95284.1| NADH oxidase
					28	22	35.8747	26.8914	
FN1089	-0.071	9.804	1.148e-1	6.566e-1	34	25	34.0000	28.2965	AAL95285.1| ATP-binding protein (contains P-loop)
					19	27	24.3435	33.0031	

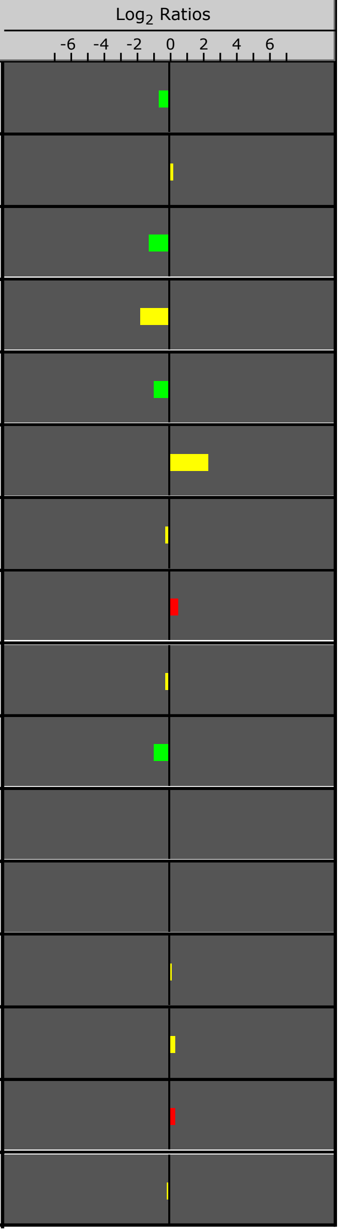

Show detected proteins only
Show all proteins
Filter by category:
GO: amino acid transport

Proteins found: 1297

Enter (or paste) list of ORFs
Find ORFs

Test	Cutoff
q-Value	.005
p-Value	

	Signif	Direction	Applies To
red	yes	+	ratios, bars
yellow	no	n/a	bars
green	yes	−	ratios, bars
pink	yes	+	p-, q-Values
light green	yes	−	p-, q-Values

Dot Plots | Dot Plots

FnPgSg vs Fn

Fusobacterium nucleatum

Hackett Laboratory UW

Fn Summary Table | FnPg vs Fn | FnSg vs Fn | FnPgSg vs Fn | FnPgSg vs FnPg | FnSg vs FnPg | FnPgSg vs FnSg | Fn Coverage

Protein	*FnPgSg* vs *Fn* Log$_2$ Ratio	Log$_2$ Sum	*q*-Value	*p*-Value	Raw *FnPgSg*	Raw *Fn*	Normalized *FnPgSg*	Normalized *Fn*	Description
FN1091	-0.748	8.207	3.136e-4	5.364e-4	15	21	15.0000	23.7690	AAL95287.1| Sigma factor sigB regulation protein rsbU
					9	17	11.5312	20.7797	
FN1092	-0.497	9.991	4.951e-2	2.558e-1	14	27	14.0000	30.5602	AAL95288.1| Hypothetical protein
					31	37	39.7184	45.2264	
FN1093	-0.599	12.248	8.443e-6	2.533e-6	57	75	57.0000	84.8894	AAL95289.1| Hypothetical protein
					44	71	56.3745	86.7858	
FN1096	-0.245	10.688	7.222e-2	3.895e-1	49	36	49.0000	40.7469	AAL95292.1| Hypothetical protein
					20	39	25.6248	47.6711	
FN1097	-2.213	10.020	2.738e-3	8.822e-3	12	47	12.0000	53.1974	AAL95293.1| Hypothetical protein
					14	70	17.9374	85.5635	
FN1101	-0.916	4.086			3	5	3.0000	5.6593	AAL95297.1| ATPase
FN1102						28		31.6921	AAL95298.1| tRNA 2'phosphotransferase
						32		39.1147	
FN1103	-2.179	11.110	1.714e-5	7.156e-6	16	85	16.0000	96.2080	AAL95299.1| Excinuclease ABC subunit A
					22	85	28.1873	103.8985	
FN1104					5		5.0000		AAL95300.1| Holliday junction DNA helicase ruvA
FN1105	-0.344	12.794	7.461e-3	2.925e-2	74	76	74.0000	86.0213	AAL95301.1| Hypothetical protein
					59	85	75.5931	103.8985	
FN1106	-1.220	11.864	3.345e-4	5.862e-4	39	88	39.0000	99.6036	AAL95302.1| L-serine dehydratase
					32	71	40.9997	86.7858	
FN1111	-0.122	6.457			9		9.0000		AAL95307.1| Dipeptide-binding protein
					7	8	8.9687	9.7787	
FN1117	0.673	12.012	2.563e-3	8.139e-3	79	37	79.0000	41.8788	AAL95313.1| LSU ribosomal protein L21P
					65	49	83.2806	59.8944	
FN1119	0.519	14.977	6.182e-4	1.308e-3	230	142	230.0000	160.7240	AAL95315.1| LSU ribosomal protein L27P
					156	114	199.8734	139.3462	
FN1120	-0.154	14.141	5.19e-2	2.697e-1	128	109	128.0000	123.3726	AAL95316.1| Phosphoenolpyruvate carboxykinase (ATP)
					99	131	126.8427	160.1260	
FN1121	0.155	12.426	3.124e-2	1.507e-1	72	67	72.0000	75.8345	AAL95317.1| hypothetical cytosolic protein
					66	53	84.5618	64.7838	

Log$_2$ Ratios: -6 -4 -2 0 2 4 6

Show detected proteins only
Show all proteins
Filter by category:
GO: amino acid transport

Proteins found: 1297

Enter (or paste) list of ORFs

Find ORFs

Test	Cutoff
q-Value	.005
p-Value	

Signif	Direction	Applies To
yes	+	ratios, bars
no	n/a	bars
yes	−	ratios, bars
yes	+	*p*-, *q*-Values
yes	−	

Dot Plots Dot Plots

Protein	*FnPgSg* vs *Fn*				Raw		Normalized		Description
	Log_2 Ratio	Log_2 Sum	*q*-Value	*p*-Value	*FnPgSg*	*Fn*	*FnPgSg*	*Fn*	
**FN1122**	-0.692	14.885	3.039e-3	1.003e-2	143	219	143.0000	247.8771	AAL95318.1| Long-chain-fatty-acid--CoA ligase
					102	159	130.6864	194.3513	
**FN1123**	0.332	9.587	7.741e-2	4.211e-1	43	21	43.0000	23.7690	AAL95319.1| Thioredoxin-like protein
					15	21	19.2186	25.6690	
**FN1124**	-0.247	17.032	5.061e-3	1.852e-2	312	356	312.0000	402.9418	AAL95320.1| Outer membrane porin F
					281	323	360.0283	394.8144	
**FN1125**	0.472	10.810	1.275e-3	3.34e-3	46	29	46.0000	32.8239	AAL95321.1| LemA protein
					42	32	53.8121	39.1147	
**FN1127**	-0.015	10.554	1.511e-1	9.013e-1	40	30	40.0000	33.9558	AAL95323.1| Hypothetical membrane-spanning protein
					29	36	37.1559	44.0041	
**FN1128**	-0.418	15.119	4.669e-4	9.141e-4	156	193	156.0000	218.4488	AAL95324.1| Acylamino-acid-releasing enzyme
					133	178	170.4048	217.5757	
**FN1129**	-1.290	7.362	1.808e-4	2.501e-4	10	16	10.0000	18.1097	AAL95325.1| Chromosome partition protein smc
					5	18	6.4062	22.0020	
**FN1130**	-0.816	6.816			8	13	8.0000	14.7142	AAL95326.1| Tetraacyldisaccharide 4'-kinase
						11		13.4457	
**FN1131**	-1.512	4.682			3		3.0000		AAL95327.1| Hypothetical protein
						7		8.5563	
**FN1133**	0.110	9.557	7.076e-2	3.807e-1	25	24	25.0000	27.1646	AAL95329.1| N-acetylglucosamine-6-phosphate deacetylase
					25	21	32.0310	25.6690	
**FN1134**	-0.962	9.877	3.515e-4	6.284e-4	26	40	26.0000	45.2744	AAL95330.1| Hypothetical cytosolic protein
					14	33	17.9374	40.3371	
**FN1135**	-0.346	14.305	2.104e-4	3.139e-4	123	142	123.0000	160.7240	AAL95331.1| Phosphonates-binding protein
					101	131	129.4052	160.1260	
**FN1136**	0.355	6.632	3.615e-5	2.421e-5	11	8	11.0000	9.0549	AAL95332.1| Phosphonates transport ATP-binding protein phnC
					9	7	11.5312	8.5563	
**FN1137**									AAL95333.1| Phosphonates transport system permease protein phnE
					3		3.8437		
**FN1138**	0.417	19.352	2.834e-2	1.343e-1	1147	644	1147.0000	728.9172	AAL95334.1| Hypothetical cytosolic protein
					580	562	743.1189	686.9526	
**FN1139**	-2.980	9.223	1e-6	1.261e-7	11	63	11.0000	71.3071	AAL95335.1| Activator of (R)-2-hydroxyglutaryl-CoA dehydratase
					5	54	6.4062	66.0061	

Log_2 Ratios: -6 -4 -2 0 2 4 6

Show detected proteins only
Show all proteins
Filter by category:
GO: amino acid transport

Proteins found: 1297

Enter (or paste) list of ORFs

Find ORFs

Test	Cutoff
q-Value	.005
p-Value	

Color	Signif	Direction	Applies To
red	yes	+	ratios, bars
yellow	no	n/a	bars
green	yes	−	ratios, bars
pink	yes	+	*p*- , *q*-Values
light green	yes	−	*p*- , *q*-Values

Dot Plots Dot Plots

Protein	*FnPgSg vs Fn* Log$_2$ Ratio	Log$_2$ Sum	*q*-Value	*p*-Value	Raw *FnPgSg*	Raw *Fn*	Normalized *FnPgSg*	Normalized *Fn*	Description
**FN1140**						7		7.9230	AAL95336.1| hypothetical protein
						8		9.7787	
**FN1142**	-2.110	5.280			3	11	3.0000	12.4504	AAL95338.1| Oxygen-independent coproporphyrinogen III oxidase
						11		13.4457	
**FN1143**	0.012	12.924	1.573e-1	9.45e-1	104	72	104.0000	81.4938	AAL95339.1| Glucosamine-6-phosphate isomerase
					57	77	73.0306	94.1198	
**FN1144**	-0.252	15.409	1.186e-3	3.015e-3	199	210	199.0000	237.6904	AAL95340.1| Hypothetical Exported Protein
					143	178	183.2172	217.5757	
**FN1145**	-0.030	8.799	1.475e-1	8.757e-1	20	15	20.0000	16.9779	AAL95341.1| Oligoendopeptidase F
					17	21	21.7811	25.6690	
**FN1146**	-0.320	9.059	1.974e-2	8.9e-2	17	24	17.0000	27.1646	AAL95342.1| Hypothetical exported 24-amino acid repeat protein
					19	20	24.3435	24.4467	
**FN1147**	1.021	9.901	9.502e-3	3.89e-2	33	20	33.0000	22.6372	AAL95343.1| Hypothetical protein
					43	17	55.0933	20.7797	
**FN1148**	-0.087	10.711	5.158e-2	2.678e-1	41	40	41.0000	45.2744	AAL95344.1| Serine/threonine sodium symporter
					30	32	38.4372	39.1147	
**FN1149**	-1.099	6.764	5.055e-3	1.849e-2	4	14	4.0000	15.8460	AAL95345.1| ATP-dependent nuclease subunit A
					8	12	10.2499	14.6680	
**FN1150**	-0.739	5.455							AAL95346.1| unknown
					4	7	5.1250	8.5563	
**FN1152**	-0.040	14.224	9.982e-2	5.613e-1	146	128	146.0000	144.8779	AAL95348.1| Aspartate aminotransferase
					99	111	126.8427	135.6792	
**FN1153**	0.032	10.393	1.326e-1	7.737e-1	37	36	37.0000	40.7469	AAL95349.1| Hypothetical protein
					29	26	37.1559	31.7807	
**FN1154**									AAL95350.1| Ribonuclease BN
						7		8.5563	
**FN1155**						7		7.9230	AAL95351.1| Cell division protein ftsI
						11		13.4457	
**FN1157**	0.980	7.065	1.114e-2	4.655e-2	12	7	12.0000	7.9230	AAL95353.1| Polypeptide deformylase
					16	7	20.4998	8.5563	
**FN1159**	-0.035	12.863	1.354e-1	7.929e-1	77	68	77.0000	76.9664	AAL95355.1| Fructose-1,6-bisphosphatase
					73	80	93.5305	97.7868	

Log$_2$ Ratios: -6 -4 -2 0 2 4 6

Show detected proteins only
Show all proteins
Filter by category:
GO: amino acid transport

Proteins found: 1297

Enter (or paste) list of ORFs

Find ORFs

Test	Cutoff
q-Value	.005
p-Value	

	Signif	Direction	Applies To
red	yes	+	ratios, bars
yellow	no	n/a	bars
green	yes	–	ratios, bars
pink	yes	+	*p*- , *q*-Values
light green	yes	–	

Dot Plots Dot Plots

Protein	*FnPgSg* vs *Fn*				Raw		Normalized		Description	Log_2 Ratios
	Log_2 Ratio	Log_2 Sum	*q*-Value	*p*-Value	*FnPgSg*	*Fn*	*FnPgSg*	*Fn*		-6 -4 -2 0 2 4 6
**FN1160**	0.227	6.107	1.842e-2	8.233e-2	9	6	9.0000	6.7912	AAL95356.1| SWF/SNF family helicase	
					7	7	8.9687	8.5563		
**FN1161**	-1.389	6.104				14		15.8460	AAL95357.1| Glutamate racemase	
					4	9	5.1250	11.0010		
**FN1162**									AAL95358.1| Hydroxyacylglutathione hydrolase	
						5		6.1117		
**FN1163**	0.429	12.174	1.094e-3	2.695e-3	77	56	77.0000	63.3841	AAL95359.1| Thioredoxin reductase	
					63	44	80.7181	53.7828		
**FN1164**	-1.552	6.911				17		19.2416	AAL95360.1| Glucokinase	
					5	15	6.4062	18.3350		
**FN1165**	0.707	24.471	1.037e-4	1.155e-4	6372	3391	6372.0000	3838.1337	AAL95361.1| D-galactose-binding protein	
					4643	3036	5948.7947	3711.0106		
**FN1166**	-0.912	14.665	6.242e-4	1.324e-3	112	179	112.0000	202.6028	AAL95362.1| Galactoside transport ATP-binding protein mglA	
					96	196	122.9990	239.5778		
**FN1167**	-0.858	9.773	4.028e-2	2.029e-1	26	52	26.0000	58.8567	AAL95363.1| Galactoside transport system permease protein mglC	
					14	17	17.9374	20.7797		
**FN1169**	-0.641	16.326	5.206e-5	4.348e-5	217	330	217.0000	373.5135	AAL95365.1| L-lactate dehydrogenase	
					189	280	242.1543	342.2539		
**FN1170**	-0.301	24.218	6.627e-4	1.43e-3	3813	4375	3813.0000	4951.8829	AAL95366.1| Pyruvate-flavodoxin oxidoreductase	
					3237	3972	4147.3721	4855.1166		
**FN1171**	-0.180	19.288	7.07e-3	2.748e-2	767	793	767.0000	897.5641	AAL95367.1| Acetate kinase	
					575	659	736.7127	805.5191		
**FN1172**	-0.131	18.873	1.678e-2	7.399e-2	704	627	704.0000	709.6756	AAL95368.1| Phosphate acetyltransferase	
					484	606	620.1199	740.7353		
**FN1179**									AAL95375.1| ATP-dependent RNA helicase	
						4		4.8893		
**FN1180**						10		11.3186	AAL95376.1| Hypothetical protein	
						7		8.5563		
**FN1181**	-0.603	12.939	1.358e-3	3.661e-3	81	99	81.0000	112.0540	AAL95377.1| unknown	
					49	87	62.7807	106.3432		
**FN1182**						8		9.0549	AAL95378.1| Hypothetical protein	
						9		11.0010		

Show detected proteins only
Show all proteins
Filter by category:
GO: amino acid transport

Proteins found: 1297

Enter (or paste) list of ORFs

Find ORFs

Test	Cutoff
q-Value	.005
p-Value	

	Signif	Direction	Applies To
red	yes	+	ratios, bars
yellow	no	n/a	bars
green	yes	−	ratios, bars
pink	yes	+	*p*- , *q*-Values
light green	yes	−	

Dot Plots Dot Plots

Protein	*FnPgSg vs Fn* Log$_2$ Ratio	Log$_2$ Sum	*q*-Value	*p*-Value	Raw *FnPgSg*	Raw *Fn*	Normalized *FnPgSg*	Normalized *Fn*	Description
FN1183	-0.372	5.250	3.84e-2	1.919e-1	7	7	7.0000	7.9230	AAL95379.1| Hypothetical cytosolic protein
					3	5	3.8437	6.1117	
FN1185	-0.022	9.796	1.28e-1	7.429e-1	31	25	31.0000	28.2965	AAL95381.1| SIR2 family protein
					22	26	28.1873	31.7807	
FN1186	0.571	8.672	1.738e-2	7.7e-2	30	12	30.0000	13.5823	AAL95382.1| Amidohydrolase
					15	16	19.2186	19.5574	
FN1187					38		38.0000		AAL95383.1| Amino acid-binding protein
					24		30.7497		
FN1188	-0.774	11.803	1.582e-3	4.52e-3	53	69	53.0000	78.0983	AAL95384.1| Hypothetical protein
					30	64	38.4372	78.2295	
FN1189	-0.844	11.917	9.974e-5	1.086e-4	48	77	48.0000	87.1531	AAL95385.1| Hypothetical protein
					35	65	44.8434	79.4518	
FN1190	-0.773	13.486	2.781e-5	1.491e-5	87	121	87.0000	136.9549	AAL95386.1| Probable cadmium-transporting ATPase
					60	117	76.8744	143.0133	
FN1191	-1.310	9.382	1.205e-4	1.418e-4	20	33	20.0000	37.3513	AAL95387.1| unknown
					10	36	12.8124	44.0041	
FN1192	0.899	17.594	2.076e-4	3.074e-4	642	298	642.0000	337.2940	AAL95388.1| unknown
					447	257	572.7140	314.1402	
FN1198	-0.032	9.001	8.343e-2	4.585e-1	23	21	23.0000	23.7690	AAL95394.1| Transporter
					17	18	21.7811	22.0020	
FN1200					5		5.0000		AAL95396.1| Hypothetical protein
					15		19.2186		
FN1202	0.633	6.991			14	8	14.0000	9.0549	AAL95398.1| NH(3)-dependent NAD(+) synthetase
					11		14.0936		
FN1203									AAL95399.1| GTP-binding protein
						10		12.2234	
FN1204						6		6.7912	AAL95400.1| Methyltransferase
						10		12.2234	
FN1205	-0.383	12.326	4.428e-3	1.578e-2	55	69	55.0000	78.0983	AAL95401.1| Protease
					55	70	70.4682	85.5635	
FN1206									AAL95402.1| Hemolysin
						8		9.7787	

Log$_2$ Ratios: -6 -4 -2 0 2 4 6

Show detected proteins only
Show all proteins
Filter by category:
GO: amino acid transport

Proteins found: 1297

Enter (or paste) list of ORFs

Find ORFs

Test	Cutoff
q-Value	.005
p-Value	

	Signif	Direction	Applies To
red	yes	+	ratios, bars
yellow	no	n/a	bars
green	yes	−	ratios, bars
pink	yes	+	*p*- , *q*-Values
light green	yes	−	

Dot Plots Dot Plots

Protein	FnPgSg vs Fn Log2 Ratio	Log2 Sum	q-Value	p-Value	Raw FnPgSg	Raw Fn	Normalized FnPgSg	Normalized Fn	Description
FN1208	-0.991	5.635			5	10	5.0000	11.3186	AAL95404.1| 1-deoxyxylulose-5-phosphate synthase
						7		8.5563	
FN1209	-0.479	11.848	3.301e-2	1.609e-1	67	77	67.0000	87.1531	AAL95405.1| Hypothetical RNA binding protein
					28	46	35.8747	56.2274	
FN1210	-1.246	11.463	5.322e-3	1.97e-2	28	55	28.0000	62.2522	AAL95406.1| Metal dependent hydrolase
					32	83	40.9997	101.4538	
FN1211	-0.425	8.138	4.291e-2	2.179e-1	20	16	20.0000	18.1097	AAL95407.1| Cell division protein ftsI
					7	17	8.9687	20.7797	
FN1213	-0.785	11.575	2.858e-4	4.737e-4	47	59	47.0000	66.7797	AAL95409.1| Hypothetical protein
					29	64	37.1559	78.2295	
FN1214					6		6.0000		AAL95410.1| Fe-S oxidoreductase
					5		6.4062		
FN1216	-2.043	7.499	2.413e-3	7.568e-3	3	18	3.0000	20.3735	AAL95412.1| RRF2 family protein
					8	28	10.2499	34.2254	
FN1217	-2.308	6.597	1.333e-3	3.563e-3	5	16	5.0000	18.1097	AAL95413.1| Holliday junction DNA helicase ruvB
					3	21	3.8437	25.6690	
FN1219	0.422	7.821	3.929e-2	1.971e-1	22	10	22.0000	11.3186	AAL95415.1| Hypothetical protein
					10	12	12.8124	14.6680	
FN1220	0.018	15.272	1.344e-1	7.861e-1	212	183	212.0000	207.1302	AAL95416.1| Cysteine synthase
					147	154	188.3422	188.2397	
FN1221	0.092	8.185	8.491e-2	4.678e-1	16	13	16.0000	14.7142	AAL95417.1| Hypothetical protein
					15	15	19.2186	18.3350	
FN1222	-1.412	8.774	5.603e-4	1.153e-3	9	29	9.0000	32.8239	AAL95418.1| Hypothetical protein
					13	29	16.6561	35.4477	
FN1223	0.552	12.301	1.365e-4	1.694e-4	90	54	90.0000	61.1204	AAL95419.1| Oxygen-insensitive NAD(P)H nitroreductase
					64	46	81.9993	56.2274	
FN1224	-0.646	13.620	8.024e-4	1.807e-3	100	113	100.0000	127.9001	AAL95420.1| 2-dehydro-3-deoxyphosphooctonate aldolase
					62	125	79.4368	152.7919	
FN1225	0.346	13.875	9.908e-3	4.08e-2	147	82	147.0000	92.8124	AAL95421.1| UDP-N-acetylmuramoyl-L-alanyl-D-glutamate--meso-lanthionine ligase
					101	102	129.4052	124.6782	
FN1226	-0.774	13.590	2.599e-7	2.069e-8	84	127	84.0000	143.7461	AAL95422.1| Uracil-DNA glycosylase
					67	120	85.8430	146.6803	

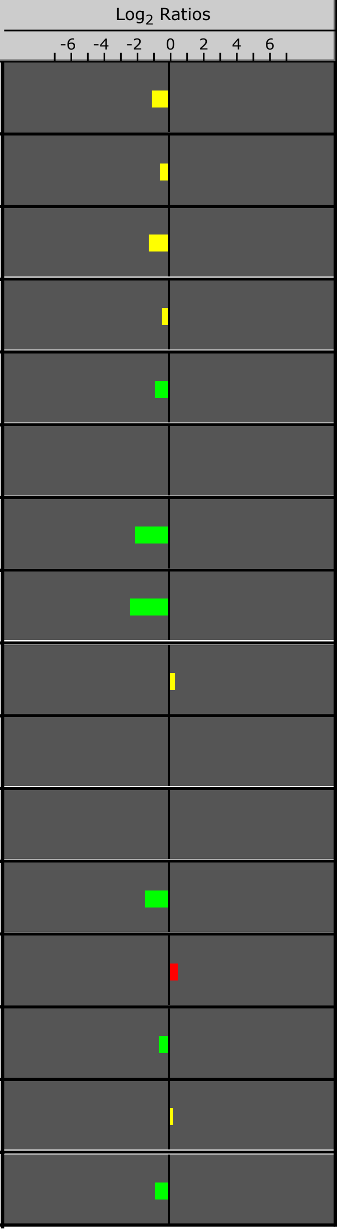

Show detected proteins only
Show all proteins
Filter by category:
GO: amino acid transport

Proteins found: 1297

Enter (or paste) list of ORFs
Find ORFs

Test	Cutoff
q-Value	.005
p-Value	

	Signif	Direction	Applies To
red	yes	+	ratios, bars
yellow	no	n/a	bars
green	yes	−	ratios, bars
pink	yes	+	p- , q-Values
light green	yes	−	

Dot Plots Dot Plots

Protein	*FnPgSg* vs *Fn* Log$_2$ Ratio	Log$_2$ Sum	*q*-Value	*p*-Value	Raw *FnPgSg*	Raw *Fn*	Normalized *FnPgSg*	Normalized *Fn*	Description
FN1229						5		5.6593	AAL95425.1| Hypothetical protein
						7		8.5563	
FN1230	0.003	11.544	1.62e-1	9.785e-1	62	50	62.0000	56.5929	AAL95426.1| Hypothetical cytosolic protein
					37	43	47.4059	52.5604	
FN1231	0.337	18.219	1.457e-3	4.047e-3	592	431	592.0000	487.8312	AAL95427.1| Inosine-5'-monophosphate dehydrogenase
					507	405	649.5884	495.0459	
FN1233						3		3.3956	AAL95429.1| Putative NAD(P)H oxidoreductase
						4		4.8893	
FN1234	-1.190	6.887	2.012e-5	9.116e-6	8	15	8.0000	16.9779	AAL95430.1| Hypothetical protein
					5	13	6.4062	15.8904	
FN1235	0.553	6.525			13	7	13.0000	7.9230	AAL95431.1| Ankyrin repeat proteins
					8		10.2499		
FN1237	0.144	9.322	1.478e-2	6.409e-2	25	22	25.0000	24.9009	AAL95433.1| Choline kinase
					22	19	28.1873	23.2244	
FN1240	-2.083	8.300	2.962e-5	1.66e-5	7	30	7.0000	33.9558	AAL95436.1| Lipopolysaccharide core biosynthesis protein rfaY
					8	32	10.2499	39.1147	
FN1241						8		9.0549	AAL95437.1| polysaccharide biosynthesis protein
						4		4.8893	
FN1242	-0.936	5.887	2.737e-3	8.818e-3	6	8	6.0000	9.0549	AAL95438.1| Polysaccharide deacetylase
					4	10	5.1250	12.2234	
FN1243	-0.940	6.429	2.902e-3	9.485e-3	7	13	7.0000	14.7142	AAL95439.1| Glycosyl transferase
					5	9	6.4062	11.0010	
FN1244	-1.254	6.278	2.128e-3	6.519e-3	5	10	5.0000	11.3186	AAL95440.1| Polysaccharide deacetylase
					5	13	6.4062	15.8904	
FN1245						22		24.9009	AAL95441.1| Glycosyl transferase
						11		13.4457	
FN1246	-2.234	6.119				19		21.5053	AAL95442.1| Lipooligosaccharide cholinephosphotransferase
					3	12	3.8437	14.6680	
FN1247	-1.668	7.452	5.921e-4	1.237e-3	11	19	11.0000	21.5053	AAL95443.1| LOS biosynthesis enzyme LBGB
					3	21	3.8437	25.6690	
FN1248						5		5.6593	AAL95444.1| Hypothetical cytosolic protein

Log$_2$ Ratios: -6 -4 -2 0 2 4 6

Show detected proteins only
Show all proteins
Filter by category:
GO: amino acid transport

Proteins found: 1297

Enter (or paste) list of ORFs

Find ORFs

Test	Cutoff
q-Value	.005
p-Value	

	Signif	Direction	Applies To
red	yes	+	ratios, bars
yellow	no	n/a	bars
green	yes	–	ratios, bars
pink	yes	+	*p*- , *q*-Values
light green	yes	–	

Dot Plots Dot Plots

FnPgSg vs Fn

Fusobacterium nucleatum

Hackett Laboratory | UW

Protein	FnPgSg vs Fn Log$_2$ Ratio	Log$_2$ Sum	*q*-Value	*p*-Value	Raw *FnPgSg*	Raw *Fn*	Normalized *FnPgSg*	Normalized *Fn*	Description
FN1250	-1.028	10.069	5.004e-4	1.001e-3	19	46	19.0000	52.0655	AAL95446.1| Guanine-hypoxanthine permease
					21	34	26.9060	41.5594	
FN1251	-0.127	6.344	8.453e-2	4.654e-1	7	8	7.0000	9.0549	AAL95447.1| High-affinity iron permease
					8	8	10.2499	9.7787	
FN1252	-0.402	18.857	4.009e-3	1.397e-2	667	638	667.0000	722.1260	AAL95448.1| 34 kDa membrane antigen precursor
					415	705	531.7144	861.7465	
FN1253	-0.279	16.411	8.377e-3	3.349e-2	240	268	240.0000	303.3382	AAL95449.1| unknown
					231	284	295.9663	347.1433	
FN1254	0.021	13.541	1.426e-1	8.416e-1	120	103	120.0000	116.5815	AAL95450.1| Oxygen-insensitive NAD(P)H nitroreductase
					78	82	99.9367	100.2315	
FN1255						15		16.9779	AAL95451.1| NagD protein
						14		17.1127	
FN1256	-0.558	4.443				5		5.6593	AAL95452.1| C4-dicarboxylate transporter large subunit
					3		3.8437		
FN1258	-0.263	20.103	6.578e-5	5.964e-5	987	1049	987.0000	1187.3200	AAL95454.1| C4-dicarboxylate-binding protein
					742	931	950.6797	1137.9944	
FN1260	-0.594	3.764			3	4	3.0000	4.5274	AAL95456.1| Sensory Transduction Protein Kinase
FN1261									AAL95457.1| Two-component response regulator
						4		4.8893	
FN1262						5		5.6593	AAL95458.1| Integral membrane protein
FN1263	-0.555	8.405	3.136e-6	6.101e-7	15	20	15.0000	22.6372	AAL95459.1| Cobalt chelatase
					12	18	15.3749	22.0020	
FN1264	-1.171	7.990	3.768e-3	1.295e-2	11	25	11.0000	28.2965	AAL95460.1| Hypothetical protein
					8	16	10.2499	19.5574	
FN1265	-0.502	12.394	7.692e-3	3.031e-2	49	83	49.0000	93.9443	AAL95461.1| Outer membrane protein
					58	66	74.3119	80.6741	
FN1266	-0.561	14.103	1.174e-3	2.972e-3	98	140	98.0000	158.4603	AAL95462.1| UTP--glucose-1-phosphate uridylyltransferase
					94	134	120.4365	163.7930	
FN1267	-0.318	10.057	2.622e-7	2.119e-8	29	32	29.0000	36.2195	AAL95463.1| Hypothetical protein
					23	30	29.4685	36.6701	

Log$_2$ Ratios: -6 -4 -2 0 2 4 6

Show detected proteins only
Show all proteins
Filter by category:
GO: amino acid transport

Proteins found: 1297

Enter (or paste) list of ORFs

Find ORFs

Test	Cutoff
q-Value	.005
p-Value	

	Signif	Direction	Applies To
red	yes	+	ratios, bars
yellow	no	n/a	bars
green	yes	−	ratios, bars
pink	yes	+	*p*- , *q*-Values
light green	yes	−	

Dot Plots Dot Plots

Protein	FnPgSg vs Fn Log$_2$ Ratio	Log$_2$ Sum	*q*-Value	*p*-Value	Raw *FnPgSg*	Raw *Fn*	Normalized *FnPgSg*	Normalized *Fn*	Description
FN1268	-0.610	15.583	4.232e-3	1.493e-2	187	272	187.0000	307.8656	AAL95464.1| Methionyl-tRNA synthetase
					134	196	171.6861	239.5778	
FN1269	-1.997	5.882				12		13.5823	AAL95465.1| Hypothetical lipoprotein
					3	14	3.8437	17.1127	
FN1270	0.528	14.416	4.406e-4	8.485e-4	177	115	177.0000	130.1638	AAL95466.1| Hypothetical cytosolic protein
					139	95	178.0923	116.1219	
FN1271	-0.310	11.294	4.592e-3	1.648e-2	49	50	49.0000	56.5929	AAL95467.1| Protease IV
					32	45	40.9997	55.0051	
FN1273	-1.363	9.239	1.301e-2	5.546e-2	14	47	14.0000	53.1974	AAL95469.1| Outer membrane protein tolC
					13	21	16.6561	25.6690	
FN1274	-1.745	9.340	4.319e-4	8.273e-4	15	37	15.0000	41.8788	AAL95470.1| Acriflavin resistance protein E
					10	42	12.8124	51.3381	
FN1275	-1.300	10.297	7.017e-5	6.519e-5	26	53	26.0000	59.9885	AAL95471.1| Acriflavin resistance protein B
					15	42	19.2186	51.3381	
FN1276					6		6.0000		AAL95472.1| Hypothetical protein
					7		8.9687		
FN1277	0.072	14.785	6.748e-2	3.611e-1	169	133	169.0000	150.5372	AAL95473.1| Aminoacyl-histidine dipeptidase
					137	145	175.5298	177.2386	
FN1278									AAL95474.1| Acetyltransferase
					9		11.5312		
FN1279	-0.736	11.827	8.152e-4	1.842e-3	46	64	46.0000	72.4390	AAL95475.1| Zinc metallohydrolase, glyoxalase II family
					37	68	47.4059	83.1188	
FN1280	0.286	7.234	1.051e-2	4.36e-2	13	11	13.0000	12.4504	AAL95476.1| Serine protease, V8 family
					11	8	14.0936	9.7787	
FN1281						21		23.7690	AAL95477.1| Cysteine protease
						13		15.8904	
FN1282	0.704	12.404	1.554e-5	6.064e-6	97	49	97.0000	55.4611	AAL95478.1| LSU ribosomal protein L17P
					71	49	90.9680	59.8944	
FN1283	1.182	18.566	6.679e-6	1.76e-6	947	383	947.0000	433.5020	AAL95479.1| DNA-directed RNA polymerase alpha chain
					726	322	930.1798	393.5920	
FN1284	-0.356	18.980	3.92e-5	2.886e-5	628	705	628.0000	797.9606	AAL95480.1| SSU ribosomal protein S4P
					502	678	643.1822	828.7435	

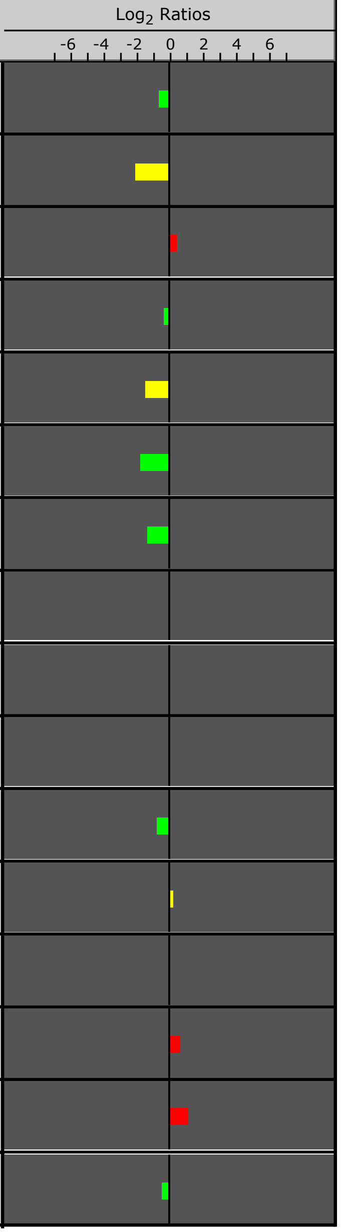

Show detected proteins only
Show all proteins
Filter by category:
GO: amino acid transport

Proteins found: 1297

Enter (or paste) list of ORFs

Find ORFs

Test	Cutoff
q-Value	.005
p-Value	

	Signif	Direction	Applies To
red	yes	+	ratios, bars
yellow	no	n/a	bars
green	yes	−	ratios, bars
light red	yes	+	*p*- , *q*-Values
light green	yes	−	

Dot Plots Dot Plots

Protein	FnPgSg vs Fn Log2 Ratio	Log2 Sum	q-Value	p-Value	Raw FnPgSg	Raw Fn	Normalized FnPgSg	Normalized Fn	Description
FN1285	0.344	13.440	1.377e-3	3.737e-3	112	80	112.0000	90.5487	AAL95481.1| SSU ribosomal protein S11P
					98	79	125.5615	96.5645	
FN1286	-1.897	15.240	3.128e-6	6.078e-7	118	323	118.0000	365.5904	AAL95482.1| SSU ribosomal protein S13P
					67	322	85.8430	393.5920	
FN1287	-2.396	8.287	3.07e-5	1.768e-5	9	36	9.0000	40.7469	AAL95483.1| Bacterial Protein Translation Initiation Factor 1 (IF-1)
					5	33	6.4062	40.3371	
FN1290	-1.483	8.099	2.174e-4	3.29e-4	7	23	7.0000	26.0328	AAL95486.1| Hypothetical protein
					10	24	12.8124	29.3361	
FN1293	-0.693	5.408				6		6.7912	AAL95489.1| Hypothetical protein
					4	8	5.1250	9.7787	
FN1296	-1.566	7.856	5.053e-3	1.849e-2	10	29	10.0000	32.8239	AAL95492.1| unknown
					6	16	7.6874	19.5574	
FN1297	0.451	10.532	6.444e-3	2.469e-2	40	29	40.0000	32.8239	AAL95493.1| Methionine aminopeptidase
					39	27	49.9683	33.0031	
FN1298	-0.430	13.144	1.609e-2	7.057e-2	64	99	64.0000	112.0540	AAL95494.1| Adenylate kinase
					78	89	99.9367	108.7879	
FN1301	-0.404	12.985	8.358e-4	1.9e-3	72	88	72.0000	99.6036	AAL95497.1| ABC transporter ATP-binding protein
					66	88	84.5618	107.5655	
FN1302	0.595	17.439	1.779e-3	5.238e-3	566	349	566.0000	395.0188	AAL95498.1| Hypothetical protein
					367	238	470.2149	290.9159	
FN1303	-0.764	12.525	5.782e-5	4.994e-5	64	85	64.0000	96.2080	AAL95499.1| hypothetical cytosolic protein
					42	85	53.8121	103.8985	
FN1304	-0.135	14.308	1.864e-2	8.348e-2	145	133	145.0000	150.5372	AAL95500.1| Single-strand DNA binding protein
					99	121	126.8427	147.9026	
FN1305	-0.462	9.965	2.969e-2	1.419e-1	18	31	18.0000	35.0876	AAL95501.1| Hypothetical cytosolic protein
					28	32	35.8747	39.1147	
FN1306	-1.696	11.192	1.299e-5	4.587e-6	23	75	23.0000	84.8894	AAL95502.1| Methyltransferase
					24	73	30.7497	89.2305	
FN1309	0.143	16.647	2.402e-2	1.11e-1	312	272	312.0000	307.8656	AAL95505.1| Hypothetical protein
					282	247	361.3095	301.9169	
FN1311									AAL95507.1| Biopolymer transport exbD protein
						8		9.7787	

Log2 Ratios: -6 -4 -2 0 2 4 6

Show detected proteins only
Show all proteins
Filter by category:
GO: amino acid transport

Proteins found: 1297

Enter (or paste) list of ORFs

Find ORFs

Test	Cutoff
q-Value	.005
p-Value	

Color	Signif	Direction	Applies To
red	yes	+	ratios, bars
yellow	no	n/a	bars
green	yes	−	ratios, bars
pink	yes	+	p-, q-Values
light green	yes	−	p-, q-Values

Dot Plots | Dot Plots

Log$_2$ Ratios: -6 -4 -2 0 2 4 6

Protein	*FnPgSg* vs *Fn* Log$_2$ Ratio	Log$_2$ Sum	*q*-Value	*p*-Value	Raw *FnPgSg*	Raw *Fn*	Normalized *FnPgSg*	Normalized *Fn*	Description
FN1312						44		49.8018	AAL95508.1| Biopolymer transport exbB protein
						52		63.5614	
FN1313	-0.522	12.653	1.252e-2	5.31e-2	84	90	84.0000	101.8673	AAL95509.1| Oligopeptide-binding protein oppA
					39	74	49.9683	90.4528	
FN1315						7		7.9230	AAL95511.1| Hypothetical protein
FN1317						13		14.7142	AAL95513.1| RNA polymerase sigma factor
						14		17.1127	
FN1318	-0.272	10.274	9.167e-3	3.726e-2	32	37	32.0000	41.8788	AAL95514.1| RNA polymerase sigma factor rpoD
					25	29	32.0310	35.4477	
FN1319	-0.848	6.655	8.414e-4	1.916e-3	6	13	6.0000	14.7142	AAL95515.1| DNA primase
					7	10	8.9687	12.2234	
FN1320	-0.838	16.608	3.695e-4	6.744e-4	205	385	205.0000	435.7657	AAL95516.1| Peptidyl-prolyl cis-trans isomerase
					209	335	267.7790	409.4824	
FN1321	0.034	19.388	1.389e-1	8.162e-1	969	752	969.0000	851.1579	AAL95517.1| Acetoacetate metabolism regulatory protein atoC
					552	643	707.2442	785.9617	
FN1322	-1.666	6.310			5	14	5.0000	15.8460	AAL95518.1| Membrane metalloprotease
						13		15.8904	
FN1323	-0.909	6.954	2.423e-3	7.603e-3	6	14	6.0000	15.8460	AAL95519.1| Thymidylate kinase
					8	12	10.2499	14.6680	
FN1324	-0.681	9.203	7.383e-3	2.889e-2	14	23	14.0000	26.0328	AAL95520.1| 1-deoxy-D-xylulose 5-phosphate reductoisomerase
					19	29	24.3435	35.4477	
FN1326	-0.660	6.990				11		12.4504	AAL95522.1| Undecaprenyl pyrophosphate synthetase
					7	13	8.9687	15.8904	
FN1327	1.125	4.875			8		8.0000		AAL95523.1| Dimethylallyltransferase
						3		3.6670	
FN1328	-1.790	5.733	3.431e-4	6.073e-4	4	11	4.0000	12.4504	AAL95524.1| Exodeoxyribonuclease VII small subunit
					3	12	3.8437	14.6680	
FN1330						3		3.3956	AAL95526.1| S-adenosylmethionine:tRNA ribosyltransferase-isomerase
FN1331	0.309	6.206	9.769e-2	5.477e-1	14	5	14.0000	5.6593	AAL95527.1| Methyltransferase
					4	8	5.1250	9.7787	

Show detected proteins only
Show all proteins
Filter by category:
GO: amino acid transport

Proteins found: 1297

Enter (or paste) list of ORFs

Find ORFs

Test	Cutoff
q-Value	.005
p-Value	

	Signif	Direction	Applies To
red	yes	+	ratios, bars
yellow	no	n/a	bars
green	yes	−	ratios, bars
pink	yes	+	*p*- , *q*-Values
light green	yes	−	

Dot Plots Dot Plots

Protein	*FnPgSg* vs *Fn* Log$_2$ Ratio	Log$_2$ Sum	*q*-Value	*p*-Value	Raw *FnPgSg*	Raw *Fn*	Normalized *FnPgSg*	Normalized *Fn*	Description	Log$_2$ Ratios (-6 -4 -2 0 2 4 6)
FN1332	-0.278	11.697	8.455e-3	3.385e-2	47	57	47.0000	64.5160	AAL95528.1| Bacterial Peptide Chain Release Factor 1 (RF-1)	
					45	51	57.6558	62.3391		
FN1334	-0.454	7.624			12	15	12.0000	16.9779	AAL95530.1| N-acetylmuramoyl-L-alanine amidase	
						13		15.8904		
FN1335	1.254	14.366	4.279e-3	1.514e-2	176	95	176.0000	107.5266	AAL95531.1| Protein translocase subunit YajC	
					213	66	272.9040	80.6741		
FN1336						21		23.7690	AAL95532.1| Hypothetical protein	
						23		28.1137		
FN1337	-2.390	7.560			6	21	6.0000	23.7690	AAL95533.1| unknown	
						32		39.1147		
FN1340	0.070	16.993	9.03e-2	5.012e-1	403	288	403.0000	325.9754	AAL95536.1| Glutamyl-tRNA synthetase	
					263	310	336.9660	378.9240		
FN1341	0.410	7.913	2.967e-2	1.418e-1	14	13	14.0000	14.7142	AAL95537.1| Bacterial Peptide Chain Release Factor 2 (RF-2)	
					17	10	21.7811	12.2234		
FN1343						13		14.7142	AAL95539.1| seC-independent protein TATD	
FN1347	-1.168	8.025	8.866e-4	2.047e-3	10	19	10.0000	21.5053	AAL95543.1| Hypothetical cytosolic protein	
					9	22	11.5312	26.8914		
FN1348	-0.313	8.818	1.086e-1	6.168e-1	33	17	33.0000	19.2416	AAL95544.1| ABC transporter ATP-binding protein	
					4	23	5.1250	28.1137		
FN1349	-2.237	8.237			8	31	8.0000	35.0876	AAL95545.1| ABC transporter permease protein	
						33		40.3371		
FN1351	-1.101	10.178	6.715e-3	2.589e-2	17	33	17.0000	37.3513	AAL95547.1| 15 kDa lipoprotein precursor	
					23	51	29.4685	62.3391		
FN1352	-0.191	11.812	1.493e-2	6.484e-2	61	56	61.0000	63.3841	AAL95548.1| ABC transporter ATP-binding protein	
					40	53	51.2496	64.7838		
FN1353	-0.576	7.133	5.372e-2	2.804e-1	13	18	13.0000	20.3735	AAL95549.1| ABC transporter permease protein	
					5	7	6.4062	8.5563		
FN1354	-0.511	9.426	7.899e-3	3.126e-2	26	24	26.0000	27.1646	AAL95550.1| ABC transporter permease protein	
					14	29	17.9374	35.4477		
FN1355	-1.125	4.295			3	4	3.0000	4.5274	AAL95551.1| Integral membrane protein	
						7		8.5563		

Show detected proteins only
Show all proteins
Filter by category:
GO: amino acid transport

Proteins found: 1297

Enter (or paste) list of ORFs

Find ORFs

Test	Cutoff
q-Value	.005
p-Value	

Signif	Direction	Applies To
yes	+	ratios, bars
no	n/a	bars
yes	−	ratios, bars
yes	+	*p*-, *q*-Values
yes	−	

Dot Plots Dot Plots

Protein	*FnPgSg* vs *Fn* Log$_2$ Ratio	Log$_2$ Sum	*q*-Value	*p*-Value	Raw *FnPgSg*	Raw *Fn*	Normalized *FnPgSg*	Normalized *Fn*	Description
**FN1358**					38		38.0000		AAL95554.1| Hypothetical protein
					26		33.3122		
**FN1359**	-0.764	13.669	1.43e-3	3.94e-3	97	116	97.0000	131.2956	AAL95555.1| Dipeptide-binding protein
					61	136	78.1556	166.2376	
**FN1363**	-1.279	5.568	1.554e-2	6.78e-2	5	6	5.0000	6.7912	AAL95559.1| Dipeptide transport ATP-binding protein dppF
					3	12	3.8437	14.6680	
**FN1364**	-0.834	12.373	1.019e-3	2.451e-3	54	79	54.0000	89.4169	AAL95560.1| LSU ribosomal protein L32P
					43	86	55.0933	105.1209	
**FN1365**	0.213	14.951	5.136e-3	1.885e-2	186	156	186.0000	176.5700	AAL95561.1| GTP-binding protein
					154	126	197.3109	154.0143	
**FN1366**	0.931	16.145	5.722e-5	4.925e-5	350	190	350.0000	215.0532	AAL95562.1| Triosephosphate isomerase
					307	143	393.3405	174.7940	
**FN1374**	-0.455	6.795			9	11	9.0000	12.4504	AAL95570.1| Transcriptional regulator
						10		12.2234	
**FN1375**	-0.308	9.394	4.839e-3	1.754e-2	21	24	21.0000	27.1646	AAL95571.1| Citrate-sodium symport
					20	25	25.6248	30.5584	
**FN1376**	-1.002	17.319	3.112e-4	5.309e-4	319	510	319.0000	577.2481	AAL95572.1| Oxaloacetate decarboxylase alpha chain
					197	464	252.4042	567.1637	
**FN1378**	-0.500	9.927	1.515e-3	4.27e-3	23	31	23.0000	35.0876	AAL95574.1| Citrate lyase acyl carrier protein
					23	32	29.4685	39.1147	
**FN1379**	-0.144	14.985	3.171e-2	1.534e-1	185	179	185.0000	202.6028	AAL95575.1| Citrate lyase beta chain
					123	144	157.5925	176.0163	
**FN1380**	-0.006	17.151	1.519e-1	9.066e-1	359	344	359.0000	389.3595	AAL95576.1| Citrate lyase beta chain
					314	307	402.3092	375.2570	
**FN1381**	-0.293	4.178				4		4.5274	AAL95577.1| unknown
					3	4	3.8437	4.8893	
**FN1383**					3		3.0000		AAL95579.1| DNA polymerase III alpha subunit
**FN1385**						6		6.7912	AAL95581.1| Hypothetical protein
						8		9.7787	
**FN1386**	1.058	6.478	6.956e-3	2.696e-2	17	4	17.0000	4.5274	AAL95582.1| SWF/SNF family helicase
					8	7	10.2499	8.5563	

Log$_2$ Ratios: -6 -4 -2 0 2 4 6

Show detected proteins only
Show all proteins
Filter by category:
GO: amino acid transport

Proteins found: 1297

Enter (or paste) list of ORFs

Find ORFs

Test	Cutoff
q-Value	.005
p-Value	

Signif	Direction	Applies To
yes	+	ratios, bars
no	n/a	bars
yes	–	ratios, bars
yes	+	*p*-, *q*-Values
yes	–	

Dot Plots | Dot Plots

Protein	*FnPgSg* vs *Fn* Log$_2$ Ratio	Log$_2$ Sum	*q*-Value	*p*-Value	Raw *FnPgSg*	Raw *Fn*	Normalized *FnPgSg*	Normalized *Fn*	Description
FN1391	-1.264	12.297	8.207e-4	1.858e-3	39	110	39.0000	124.5045	AAL95584.1| Acetyltransferase
					41	78	52.5308	95.3422	
FN1392	-0.204	15.130	3.254e-3	1.087e-2	185	181	185.0000	204.8665	AAL95585.1| SSU ribosomal protein S16P
					131	165	167.8424	201.6854	
FN1393	-1.034	10.766	3.196e-4	5.504e-4	25	58	25.0000	65.6478	AAL95586.1| Signal recognition particle, subunit FFH/SRP54
					26	44	33.3122	53.7828	
FN1397	-0.464	14.574	8.85e-3	3.573e-2	157	161	157.0000	182.2293	AAL95590.1| Glutaminase
					85	151	108.9054	184.5727	
FN1398	0.635	14.217	5.686e-3	2.129e-2	171	119	171.0000	134.6912	AAL95591.1| Amino acid carrier protein alsT
					135	71	172.9673	86.7858	
FN1400									AAL95593.1| serine/threonine kinase
					7		8.9687		
FN1406	-0.021	11.536	1.483e-1	8.813e-1	48	43	48.0000	48.6699	AAL95599.1| Histidine ammonia-lyase
					47	50	60.2183	61.1168	
FN1407	0.617	7.439	6.913e-2	3.709e-1	7	8	7.0000	9.0549	AAL95600.1| Glutamate formiminotransferase
					20	10	25.6248	12.2234	
FN1411	0.339	14.464	4.53e-2	2.315e-1	210	112	210.0000	126.7682	AAL95604.1| Threonine dehydratase
					100	115	128.1239	140.5686	
FN1413					3		3.0000		AAL95606.1| Translation initiation factor EIF-2B subunit 1
FN1416					5		5.0000		AAL95609.1| Transcriptional regulator, GntR family
					3		3.8437		
FN1417	0.032	4.612			5		5.0000		AAL95610.1| L-fuculose phosphate aldolase
						4		4.8893	
FN1418									AAL95611.1| Transcriptional regulator, GntR family
						4		4.8893	
FN1419	2.033	20.268	1.133e-6	1.531e-7	2312	475	2312.0000	537.6330	AAL95612.1| Methionine gamma-lyase
					1743	469	2233.2004	573.2754	
FN1420					14		14.0000		AAL95613.1| NA+/H+ antiporter NHAC
FN1421	0.210	20.155	2.186e-3	6.729e-3	1134	842	1134.0000	953.0252	AAL95614.1| Pyruvate-flavodoxin oxidoreductase
					929	864	1190.2714	1056.0979	

Log$_2$ Ratios: -6 -4 -2 0 2 4 6

Show detected proteins only
Show all proteins
Filter by category:
GO: amino acid transport

Proteins found: 1297

Enter (or paste) list of ORFs

Find ORFs

Test	Cutoff
q-Value	.005
p-Value	

	Signif	Direction	Applies To
red	yes	+	ratios, bars
yellow	no	n/a	bars
green	yes	–	ratios, bars
pink	yes	+	*p*- , *q*-Values
light green	yes	–	

Dot Plots Dot Plots

Protein	FnPgSg vs Fn: Log_2 Ratio	Log_2 Sum	*q*-Value	*p*-Value	Raw: *FnPgSg*	Raw: *Fn*	Normalized: *FnPgSg*	Normalized: *Fn*	Description
FN1423	0.330	15.111	3.541e-3	1.202e-2	227	155	227.0000	175.4381	AAL95616.1| Flavoprotein
					152	131	194.7484	160.1260	
FN1424	0.207	17.125	5.448e-2	2.85e-1	478	329	478.0000	372.3816	AAL95617.1| ACYL-COA dehydrogenase, short-chain specific
					261	271	334.4035	331.2529	
FN1426	-1.863	16.445	4.383e-5	3.382e-5	130	496	130.0000	561.4020	AAL95619.1| Serine protease
					143	473	183.2172	578.1647	
FN1427					6		6.0000		AAL95620.1| Phenazine biosynthesis protein phzF
					4		5.1250		
FN1429						7		7.9230	AAL95622.1| Branched-chain amino acid transport ATP-binding protein livG
FN1432	-1.094	10.343	1.523e-3	4.298e-3	25	52	25.0000	58.8567	AAL95625.1| Leucine-, isoleucine-, valine-, threonine-, and alanine-binding protein
					19	38	24.3435	46.4487	
FN1433	0.426	16.104	3.541e-3	1.202e-2	336	193	336.0000	218.4488	AAL95626.1| Short chain dehydrogenase
					218	196	279.3102	239.5778	
FN1434	-0.705	13.570	1.409e-4	1.774e-4	92	117	92.0000	132.4275	AAL95627.1| Tetratricopeptide repeat family protein
					63	122	80.7181	149.1249	
FN1437	-0.930	15.037	9.446e-5	9.988e-5	117	215	117.0000	243.3497	AAL95630.1| LSU ribosomal protein L28P
					116	215	148.6238	262.8021	
FN1439	-1.963	11.632	1.338e-3	3.582e-3	34	81	34.0000	91.6806	AAL95632.1| Transcriptional regulator, DeoR family
					18	107	23.0623	130.7899	
FN1440	-1.580	12.489	4.442e-4	8.573e-4	48	128	48.0000	144.8779	AAL95633.1| 1-phosphofructokinase
					31	96	39.7184	117.3442	
FN1441	-0.637	14.817	4.58e-5	3.599e-5	129	195	129.0000	220.7125	AAL95634.1| PTS system, fructose-specific IIABC component
					112	166	143.4988	202.9077	
FN1444	-0.271	17.212	1.343e-3	3.603e-3	375	362	375.0000	409.7329	AAL95637.1| GMP synthase (glutamine-hydrolyzing)
					261	365	334.4035	446.1525	
FN1445	-1.001	10.911	3.582e-3	1.218e-2	30	46	30.0000	52.0655	AAL95638.1| DNA helicase
					25	59	32.0310	72.1178	
FN1449	-1.634	17.044	1.317e-4	1.608e-4	165	560	165.0000	633.8410	AAL95642.1| Fusobacterium outer membrane protein family
					197	541	252.4042	661.2835	
FN1450	-1.110	7.929	2.511e-7	1.888e-8	11	20	11.0000	22.6372	AAL95643.1| Integral membrane protein
					8	19	10.2499	23.2244	

Log_2 Ratios: -6 -4 -2 0 2 4 6

Show detected proteins only
Show all proteins
Filter by category:
GO: amino acid transport

Proteins found: 1297

Enter (or paste) list of ORFs

Find ORFs

Test	Cutoff
q-Value	.005
p-Value	

	Signif	Direction	Applies To
red	yes	+	ratios, bars
yellow	no	n/a	bars
green	yes	−	ratios, bars
pink	yes	+	*p*-, *q*-Values
light green	yes	−	*p*-, *q*-Values

Dot Plots Dot Plots

Protein	*FnPgSg* vs *Fn* Log$_2$ Ratio	Log$_2$ Sum	*q*-Value	*p*-Value	Raw *FnPgSg*	Raw *Fn*	Normalized *FnPgSg*	Normalized *Fn*	Description
FN1451	-0.363	14.291	5.929e-3	2.236e-2	110	139	110.0000	157.3284	AAL95644.1| Cell division protein ftsZ
					109	134	139.6551	163.7930	
FN1452	-0.615	12.755	3.825e-3	1.319e-2	78	103	78.0000	116.5815	AAL95645.1| Cell division protein ftsA
					44	73	56.3745	89.2305	
FN1454	-0.302	9.682	5.884e-3	2.216e-2	26	26	26.0000	29.4283	AAL95647.1| D-alanine--D-alanine ligase
					20	28	25.6248	34.2254	
FN1455	-0.511	7.759	2.654e-2	1.245e-1	8	17	8.0000	19.2416	AAL95648.1| UDP-N-acetylenolpyruvoylglucosamine reductase
					13	13	16.6561	15.8904	
FN1456	-0.648	11.640	7.203e-4	1.583e-3	39	58	39.0000	65.6478	AAL95649.1| UDP-N-acetylmuramate--alanine ligase
					40	62	51.2496	75.7848	
FN1457	-1.513	10.302	3.401e-6	6.872e-7	19	51	19.0000	57.7248	AAL95650.1| UDP-N-acetylglucosamine-N-acetylmuramyl-Pentapeptide pyrophosphoryl-undecaprenol N-[illegible]
					18	51	23.0623	62.3391	
FN1458	-0.832	11.491	4.331e-5	3.325e-5	42	66	42.0000	74.7027	AAL95651.1| UDP-N-acetylmuramoylalanine--D-glutamate ligase
					30	56	38.4372	68.4508	
FN1459									AAL95652.1| Phospho-N-acetylmuramoyl-pentapeptide-transferase
						12		14.6680	
FN1461	-1.278	9.567	2.113e-4	3.16e-4	20	38	20.0000	43.0106	AAL95654.1| Histidinol-phosphatase
					12	35	15.3749	42.7817	
FN1463	0.642	16.865	5.185e-5	4.321e-5	448	236	448.0000	267.1187	AAL95656.1| pyridoxine biosynthesis protein
					324	234	415.1216	286.0265	
FN1464	-0.738	14.970	2.936e-4	4.909e-4	125	209	125.0000	236.5585	AAL95657.1| 1-deoxyxylulose-5-phosphate synthase
					119	185	152.4675	226.1321	
FN1470	-0.032	12.261	1.353e-1	7.919e-1	63	69	63.0000	78.0983	AAL95663.1| Hypothetical protein
					59	52	75.5931	63.5614	
FN1471	-1.454	6.098			5	8	5.0000	9.0549	AAL95664.1| LACI-family transcription regulator
						15		18.3350	
FN1472	-1.228	14.605	1.337e-3	3.577e-3	105	239	105.0000	270.5143	AAL95665.1| N-acetylneuraminate-binding protein
					79	174	101.2179	212.6864	
FN1474						8		9.0549	AAL95667.1| N-acetylmannosamine kinase
						7		8.5563	
FN1475	0.996	12.046	9.879e-4	2.354e-3	103	36	103.0000	40.7469	AAL95668.1| N-acetylneuraminate lyase
					63	42	80.7181	51.3381	

Log$_2$ Ratios: -6 -4 -2 0 2 4 6

Show detected proteins only
Show all proteins
Filter by category:
GO: amino acid transport

Proteins found: 1297

Enter (or paste) list of ORFs

Find ORFs

Test	Cutoff
q-Value	.005
p-Value	

	Signif	Direction	Applies To
red	yes	+	ratios, bars
yellow	no	n/a	bars
green	yes	−	ratios, bars
pink	yes	+	*p*- , *q*-Values
light green	yes	−	

Dot Plots Dot Plots

Protein	*FnPgSg vs Fn* Log$_2$ Ratio	Log$_2$ Sum	*q*-Value	*p*-Value	Raw *FnPgSg*	Raw *Fn*	Normalized *FnPgSg*	Normalized *Fn*	Description
FN1476	-0.949	7.937	1.144e-7	4.624e-9	11	19	11.0000	21.5053	AAL95669.1| N-acetylmannosamine-6-phosphate 2-epimerase
					9	18	11.5312	22.0020	
FN1478									AAL95671.1| Hypothetical protein
					9		11.5312		
FN1479	-0.809	9.479	2.012e-3	6.102e-3	25	29	25.0000	32.8239	AAL95672.1| Hypothetical protein
					12	31	15.3749	37.8924	
FN1480	-1.505	6.871	1.481e-3	4.139e-3	9	16	9.0000	18.1097	AAL95673.1| MG2+ transporter MGTE
					3	15	3.8437	18.3350	
FN1481	-0.802	4.802			4	8	4.0000	9.0549	AAL95674.1| Queuine tRNA-ribosyltransferase
					4			4.8893	
FN1482	-1.728	9.836	1.616e-3	4.647e-3	14	40	14.0000	45.2744	AAL95675.1| Guanosine-3',5'-bis (Diphosphate) 3'-pyrophosphohydrolase
					15	53	19.2186	64.7838	
FN1483	0.263	11.227	1.932e-3	5.818e-3	56	39	56.0000	44.1425	AAL95676.1| Adenine phosphoribosyltransferase
					40	37	51.2496	45.2264	
FN1484	-0.856	6.553	8.42e-3	3.369e-2	8	9	8.0000	10.1867	AAL95677.1| Tetratricopeptide repeat family protein
					5	13	6.4062	15.8904	
FN1486	-0.555	4.844	4.252e-3	1.502e-2	5	5	5.0000	5.6593	AAL95679.1| magnesium and cobalt efflux protein CorC
					3	6	3.8437	7.3340	
FN1487	-0.519	15.287	6.114e-3	2.319e-2	197	207	197.0000	234.2948	AAL95681.1| Chorismate mutase
					107	200	137.0926	244.4671	
FN1488					11		11.0000		AAL95682.1| Methylenetetrahydrofolate dehydrogenase (NADP+)
					10		12.8124		
FN1489	-1.776	7.690	5.003e-4	1.001e-3	4	20	4.0000	22.6372	AAL95683.1| Methionyl-tRNA formyltransferase
					9	25	11.5312	30.5584	
FN1490	-0.449	6.063			7	5	7.0000	5.6593	AAL95684.1| putative regulatory protein
						11		13.4457	
FN1491									AAL95685.1| PTS system, IIA component
						16		19.5574	
FN1493						23		26.0328	AAL95687.1| Hypothetical protein
						14		17.1127	
FN1494	-1.044	8.533	8.809e-7	1.048e-7	14	24	14.0000	27.1646	AAL95680.1| Rod shape-determining protein mreC
					10	23	12.8124	28.1137	

Log$_2$ Ratios: -6 -4 -2 0 2 4 6

Show detected proteins only
Show all proteins
Filter by category:
GO: amino acid transport

Proteins found: 1297

Enter (or paste) list of ORFs

Find ORFs

Test	Cutoff
q-Value	.005
p-Value	

	Signif	Direction	Applies To
red	yes	+	ratios, bars
yellow	no	n/a	bars
green	yes	−	ratios, bars
light red	yes	+	*p*-, *q*-Values
light green	yes	−	*p*-, *q*-Values

Dot Plots Dot Plots

Protein	FnPgSg vs Fn Log2 Ratio	Log2 Sum	q-Value	p-Value	Raw FnPgSg	Raw Fn	Normalized FnPgSg	Normalized Fn	Description
FN1496	-1.044	8.533	8.809e-7	1.048e-7	14	24	14.0000	27.1646	AAL95680.1| Rod shape-determining protein mreC
					10	23	12.8124	28.1137	
FN1499	0.793	12.833	1.634e-3	4.711e-3	98	65	98.0000	73.5708	AAL93625.1| Cell surface protein
					99	46	126.8427	56.2274	
FN1501									AAL93627.1| Nickel transport ATP-binding protein nikD
					3		3.8437		
FN1504	-0.874	14.292	9.725e-5	1.044e-4	117	179	117.0000	202.6028	AAL93630.1| Nickel-binding protein
					72	148	92.2492	180.9057	
FN1505	-0.776	15.260	2.495e-4	3.975e-4	158	215	158.0000	243.3497	AAL93631.1| 6,7-dimethyl-8-ribityllumazine synthase
					113	225	144.7801	275.0255	
FN1506	0.717	7.719			18	10	18.0000	11.3186	AAL93632.1| Diaminohydroxyphosphoribosylaminopyrimidine deaminase
					15		19.2186		
FN1508	0.624	13.320	1.54e-4	1.998e-4	123	77	123.0000	87.1531	AAL93634.1| GTP cyclohydrolase II
					100	62	128.1239	75.7848	
FN1512	-1.700	5.249	5.512e-4	1.129e-3	3	11	3.0000	12.4504	AAL93638.1| hypothetical exported 24-amino acid repeat protein
					3	8	3.8437	9.7787	
FN1517	-0.240	15.926	1.464e-2	6.337e-2	235	262	235.0000	296.5470	AAL93643.1| Leucyl-tRNA synthetase
					175	201	224.2169	245.6894	
FN1518						9		10.1867	AAL93644.1| RNA polymerase sigma-H factor
						6		7.3340	
FN1519	-0.052	9.057	8.335e-2	4.58e-1	21	21	21.0000	23.7690	AAL93645.1| 23S rRNA methyltransferase
					19	19	24.3435	23.2244	
FN1520	0.821	12.750	1.369e-3	3.704e-3	122	52	122.0000	58.8567	AAL93646.1| UDP-N-acetylglucosamine 1-carboxyvinyltransferase
					77	54	98.6554	66.0061	
FN1521						15		16.9779	AAL93647.1| Dipeptide transport system permease protein dppB
						16		19.5574	
FN1523	-0.519	16.339	1.033e-4	1.148e-4	253	299	253.0000	338.4258	AAL93649.1| Dipeptide-binding protein
					178	287	228.0606	350.8103	
FN1524						5		5.6593	AAL93650.1| Dipeptide transport ATP-binding protein dppD
FN1525	0.442	5.443				5		5.6593	AAL93651.1| Dipeptide transport ATP-binding protein dppF
					6		7.6874		

Log2 Ratios: -6 -4 -2 0 2 4 6

Show detected proteins only
Show all proteins
Filter by category:
GO: amino acid transport

Proteins found: 1297

Enter (or paste) list of ORFs
Find ORFs

Test	Cutoff
q-Value	.005
p-Value	

Color	Signif	Direction	Applies To
red	yes	+	ratios, bars
yellow	no	n/a	bars
green	yes	–	ratios, bars
pink	yes	+	p- , q-Values
light green	yes	–	p- , q-Values

Dot Plots Dot Plots

Protein	FnPgSg vs Fn Log2 Ratio	Log2 Sum	q-Value	p-Value	Raw FnPgSg	Raw Fn	Normalized FnPgSg	Normalized Fn	Description
FN1526	-0.638	22.065	3.842e-4	7.109e-4	1491	2415	1491.0000	2733.4394	AAL93652.1| Fusobacterium outer membrane protein family
					1457	2039	1866.7659	2492.3421	
FN1527	-0.874	14.420	1.588e-4	2.084e-4	97	164	97.0000	185.6249	AAL93653.1| Hypothetical protein
					95	176	121.7177	215.1310	
FN1528	-0.950	14.337	1.05e-3	2.551e-3	84	173	84.0000	195.8116	AAL93654.1| Hypothetical protein
					96	167	122.9990	204.1300	
FN1529	-0.438	12.752	2.029e-2	9.178e-2	53	79	53.0000	89.4169	AAL93655.1| Hypothetical protein
					70	85	89.6868	103.8985	
FN1531	0.821	10.195	1.847e-2	8.262e-2	59	25	59.0000	28.2965	AAL93657.1| murein hydrolase export regulator
					25	19	32.0310	23.2244	
FN1533	0.285	18.751	1.004e-4	1.097e-4	744	534	744.0000	604.4127	AAL93659.1| Electron transfer flavoprotein alpha-subunit
					564	490	722.6190	598.9444	
FN1534	0.052	19.332	8.7e-2	4.807e-1	868	657	868.0000	743.6313	AAL93660.1| Electron transfer flavoprotein beta-subunit
					614	697	786.6810	851.9678	
FN1535	0.258	20.294	7.127e-4	1.563e-3	1208	873	1208.0000	988.1129	AAL93661.1| Acyl-CoA dehydrogenase, short-chain specific
					993	888	1272.2708	1085.4339	
FN1536	0.403	17.709	1.128e-4	1.295e-4	539	369	539.0000	417.6560	AAL93662.1| (S)-2-hydroxy-acid oxidase chain D
					410	317	525.3082	387.4804	
FN1537						4		4.5274	AAL93663.1| Arsenical pump-driving ATPase
						11		13.4457	
FN1538	-0.655	6.995			9	11	9.0000	12.4504	AAL93664.1| Arsenical pump-driving ATPase
						13		15.8904	
FN1539	-0.327	15.290	2.81e-4	4.633e-4	187	205	187.0000	232.0311	AAL93665.1| Iron-sulfur cluster-binding protein
					133	177	170.4048	216.3534	
FN1540	-0.732	14.837	7.582e-5	7.271e-5	122	205	122.0000	232.0311	AAL93666.1| Iron-sulfur cluster-binding protein
					112	171	143.4988	209.0194	
FN1544	-0.117	15.005	4.955e-2	2.56e-1	156	162	156.0000	183.3611	AAL93670.1| Probable electron transfer flavoprotein-quinone oxidoreductase ydiS
					150	159	192.1859	194.3513	
FN1545	-0.225	9.202	7.222e-2	3.895e-1	18	28	18.0000	31.6921	AAL93671.1| Ferredoxin like protein
					21	17	26.9060	20.7797	
FN1546	0.606	20.143	2.58e-4	4.147e-4	1330	730	1330.0000	826.2570	AAL93672.1| Protein Translation Elongation Factor G (EF-G)
					1034	751	1324.8016	917.9740	

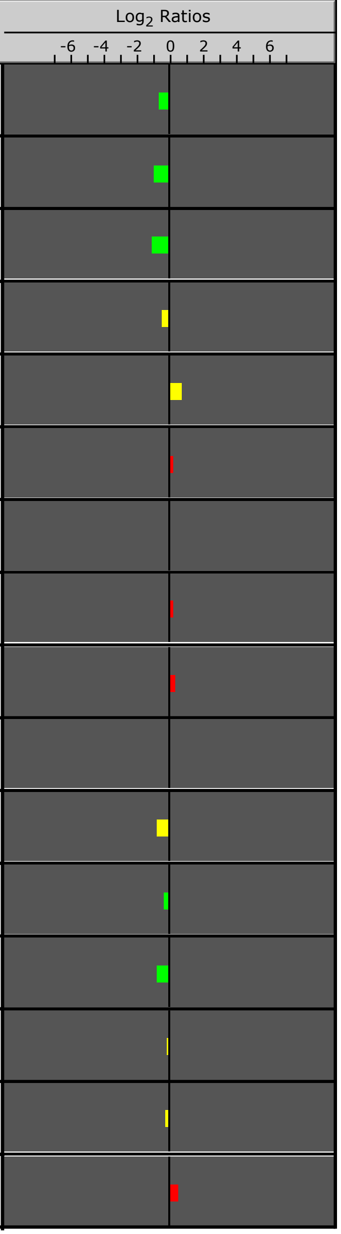

Show detected proteins only
Show all proteins
Filter by category:
GO: amino acid transport

Proteins found: 1297

Enter (or paste) list of ORFs

Find ORFs

Test	Cutoff
q-Value	.005
p-Value	

	Signif	Direction	Applies To
red	yes	+	ratios, bars
yellow	no	n/a	bars
green	yes	–	ratios, bars
pink	yes	+	p- , q-Values
light green	yes	–	

Dot Plots Dot Plots

Protein	*FnPgSg* vs *Fn* Log$_2$ Ratio	Log$_2$ Sum	*q*-Value	*p*-Value	Raw *FnPgSg*	Raw *Fn*	Normalized *FnPgSg*	Normalized *Fn*	Description
FN1547	-0.316	14.795	3.208e-5	1.915e-5	151	165	151.0000	186.7567	AAL93673.1| PTS permease for N-acetylglucosamine and glucose
					118	155	151.1863	189.4620	
FN1548	0.477	10.804	3.204e-2	1.553e-1	37	32	37.0000	36.2195	AAL93674.1| Hypothetical protein
					49	29	62.7807	35.4477	
FN1549	0.343	19.753	2.978e-3	9.795e-3	1019	667	1019.0000	754.9499	AAL93675.1| Stomatin like protein
					857	748	1098.0222	914.3070	
FN1552						12		13.5823	AAL93678.1| abortive phage resistance protein
						12		14.6680	
FN1553	-0.038	8.779	1.476e-1	8.762e-1	26	17	26.0000	19.2416	AAL93679.1| abortive phage resistance protein
					12	19	15.3749	23.2244	
FN1554	-0.953	16.471	3.379e-4	5.945e-4	195	337	195.0000	381.4365	AAL93680.1| Fusobacterium outer membrane protein family
					186	374	238.3105	457.1535	
FN1555	0.494	24.054	7.898e-6	2.287e-6	5050	3198	5050.0000	3619.6849	AAL93681.1| Protein Translation Elongation Factor Tu
					3790	2792	4855.8975	3412.7607	
FN1556	0.097	20.934	2.3e-2	1.057e-1	1392	1210	1392.0000	1369.5493	AAL93682.1| Protein Translation Elongation Factor G (EF-G)
					1199	1119	1536.2061	1367.7934	
FN1557	2.045	14.967	9.541e-5	1.014e-4	381	79	381.0000	89.4169	AAL93683.1| SSU ribosomal protein S7P
					270	71	345.9346	86.7858	
FN1558									AAL93684.1| SSU ribosomal protein S12P
					83		106.3429		
FN1560	-0.656	13.653	1.924e-4	2.739e-4	86	133	86.0000	150.5372	AAL93686.1| unknown
					74	110	94.8117	134.4569	
FN1562	-1.550	6.194			5	14	5.0000	15.8460	AAL93688.1| Phospho-2-dehydro-3-deoxyheptonate aldolase
						11		13.4457	
FN1577	-0.079	14.568	3.613e-2	1.788e-1	152	134	152.0000	151.6691	AAL93692.1| Rod shape-determining protein mreB
					118	138	151.1863	168.6823	
FN1579	-0.459	12.170	4.335e-3	1.539e-2	62	78	62.0000	88.2850	AAL93694.1| Cysteinyl-tRNA synthetase
					42	58	53.8121	70.8955	
FN1580						6		6.7912	AAL93695.1| 2-C-methyl-D-erythritol 4-phosphate cytidylyltransferase
FN1581	-0.610	10.586	4.59e-3	1.647e-2	34	37	34.0000	41.8788	AAL93696.1| DNA mismatch repair protein mutS
					23	45	29.4685	55.0051	

Log$_2$ Ratios: -6 -4 -2 0 2 4 6

Show detected proteins only
Show all proteins
Filter by category:
GO: amino acid transport

Proteins found: 1297

Enter (or paste) list of ORFs

Find ORFs

Test	Cutoff
q-Value	.005
p-Value	

	Signif	Direction	Applies To
red	yes	+	ratios, bars
yellow	no	n/a	bars
green	yes	−	ratios, bars
pink	yes	+	*p*- , *q*-Values
light green	yes	−	

Dot Plots Dot Plots

Protein	*FnPgSg* vs Fn Log$_2$ Ratio	Log$_2$ Sum	*q*-Value	*p*-Value	Raw *FnPgSg*	Raw *Fn*	Normalized *FnPgSg*	Normalized *Fn*	Description
FN1582									AAL93697.1| Hypothetical protein
						11		13.4457	
FN1589	-1.276	9.956	2.001e-3	6.064e-3	20	37	20.0000	41.8788	AAL93704.1| LexA repressor
					16	46	20.4998	56.2274	
FN1591	-0.438	15.917	1.34e-4	1.648e-4	202	247	202.0000	279.5692	AAL93706.1| RNFB-related protein
					176	245	225.4981	299.4722	
FN1592									AAL93707.1| Na(+)-translocating NADH-quinone reductase subunit D
					12		15.3749		
FN1594	0.815	12.225	3.051e-5	1.748e-5	90	49	90.0000	55.4611	AAL93709.1| Nitrogen fixation protein RNFG
					73	40	93.5305	48.8934	
FN1595	0.443	14.395	9.011e-4	2.09e-3	177	121	177.0000	136.9549	AAL93710.1| Na(+)-translocating NADH-quinone reductase subunit B
					129	94	165.2799	114.8995	
FN1596	-0.538	19.910	1.288e-3	3.388e-3	748	1055	748.0000	1194.1112	AAL93711.1| Nitrogen fixation iron-sulphur protein RNFC
					702	980	899.4301	1197.8888	
FN1597	-1.886	5.886			4	11	4.0000	12.4504	AAL93712.1| Peptidyl-tRNA hydrolase
						14		17.1127	
FN1600						6		6.7912	AAL93715.1| tRNA pseudouridine synthase A
FN1601									AAL93716.1| Hypothetical cytosolic protein
						4		4.8893	
FN1602									AAL93717.1| Hypothetical cytosolic protein
					4		5.1250		
FN1603	-1.777	7.171	2.058e-4	3.034e-4	4	22	4.0000	24.9009	AAL93718.1| 2',3'-cyclic nucleotide 3'-phosphodiesterase
					7	16	8.9687	19.5574	
FN1605	-0.533	16.299	3.651e-4	6.636e-4	239	314	239.0000	355.4037	AAL93720.1| Adenylosuccinate synthetase
					182	268	233.1856	327.5859	
FN1606	-1.934	9.616	5.688e-6	1.373e-6	12	46	12.0000	52.0655	AAL93721.1| 3-deoxy-D-manno-octulosonic-acid transferase
					13	47	16.6561	57.4498	
FN1607	-1.077	7.792				22		24.9009	AAL93722.1| Cytidylate kinase
					8	15	10.2499	18.3350	
FN1609	-3.843	7.843			4	55	4.0000	62.2522	AAL93724.1| Hypothetical protein
						43		52.5604	

Log$_2$ Ratios: -6 -4 -2 0 2 4 6

Show detected proteins only
Show all proteins
Filter by category:
GO: amino acid transport

Proteins found: 1297

Enter (or paste) list of ORFs

Find ORFs

Test	Cutoff
q-Value	.005
p-Value	

	Signif	Direction	Applies To
red	yes	+	ratios, bars
yellow	no	n/a	bars
green	yes	–	ratios, bars
light red	yes	+	*p*-, *q*-Values
light green	yes	–	

Dot Plots Dot Plots

Protein	*FnPgSg* vs *Fn* Log₂ Ratio	Log₂ Sum	*q*-Value	*p*-Value	Raw *FnPgSg*	Raw *Fn*	Normalized *FnPgSg*	Normalized *Fn*	Description
FN1610	-0.837	8.135	3.9e-4	7.255e-4	11	18	11.0000	20.3735	AAL93725.1| 33 kDa chaperonin
					11	20	14.0936	24.4467	
FN1611									AAL93726.1| Competence protein
						3		3.6670	
FN1613	-0.131	8.589	3.769e-2	1.877e-1	17	19	17.0000	21.5053	AAL93728.1| Hypothetical protein
					16	16	20.4998	19.5574	
FN1614	-0.923	9.380	6.385e-3	2.443e-2	17	25	17.0000	28.2965	AAL93729.1| MG(2+) chelatase family protein
					16	35	20.4998	42.7817	
FN1616	1.703	9.788	1.199e-2	5.059e-2	74	14	74.0000	15.8460	AAL93731.1| N utilization substance protein B
					26	14	33.3122	17.1127	
FN1618	0.513	10.595	1.399e-3	3.82e-3	44	29	44.0000	32.8239	AAL93733.1| Hypothetical protein
					39	27	49.9683	33.0031	
FN1619	1.804	14.923	1.135e-3	2.836e-3	282	90	282.0000	101.8673	AAL93734.1| Hypothetical cytosolic protein
					294	71	376.6844	86.7858	
FN1620	-0.186	18.736	7.55e-3	2.966e-2	624	589	624.0000	666.6649	AAL93735.1| SSU ribosomal protein S2P
					480	608	614.9949	743.1800	
FN1621	-0.264	21.085	1.658e-2	7.3e-2	1181	1389	1181.0000	1572.1521	AAL93736.1| Protein Translation Elongation Factor Ts
					1203	1388	1541.3311	1696.6017	
FN1622	-0.173	15.566	1.593e-2	6.974e-2	224	207	224.0000	234.2948	AAL93737.1| Uridylate kinase
					149	191	190.9047	233.4661	
FN1623	-0.657	12.842	3.933e-3	1.364e-2	66	107	66.0000	121.1089	AAL93738.1| Ribosome Recycling Factor (RRF)
					55	77	70.4682	94.1198	
FN1624	-0.341	12.388	6.462e-4	1.384e-3	66	70	66.0000	79.2301	AAL93739.1| Protein translocase subunit secY
					50	70	64.0620	85.5635	
FN1625	-0.387	14.085	1.329e-3	3.546e-3	114	140	114.0000	158.4603	AAL93740.1| LSU ribosomal protein L15P
					91	117	116.5928	143.0133	
FN1626	-0.900	11.987	7.562e-5	7.244e-5	51	75	51.0000	84.8894	AAL93741.1| LSU ribosomal protein L30P
					33	73	42.2809	89.2305	
FN1627	0.508	18.203	1.979e-2	8.923e-2	791	397	791.0000	449.3480	AAL93742.1| SSU ribosomal protein S5P
					405	386	518.9020	471.8215	
FN1628	0.274	15.172	3.376e-2	1.653e-1	242	177	242.0000	200.3390	AAL93743.1| LSU ribosomal protein L18P
					141	122	180.6548	149.1249	

Log₂ Ratios: -6 -4 -2 0 2 4 6

Show detected proteins only
Show all proteins
Filter by category:
GO: amino acid transport

Proteins found: 1297

Enter (or paste) list of ORFs

Find ORFs

Test	Cutoff
q-Value	.005
p-Value	

Signif	Direction	Applies To
yes	+	ratios, bars
no	n/a	bars
yes	−	ratios, bars
yes	+	*p*-, *q*-Values
yes	−	

Dot Plots Dot Plots

Protein	*FnPgSg vs Fn* Log$_2$ Ratio	Log$_2$ Sum	*q*-Value	*p*-Value	Raw *FnPgSg*	Raw *Fn*	Normalized *FnPgSg*	Normalized *Fn*	Description
FN1629	-1.235	17.092	1.41e-5	5.192e-6	217	524	217.0000	593.0941	AAL93744.1| LSU ribosomal protein L6P
					211	453	270.3415	553.7180	
FN1630	0.382	14.101	6.248e-2	3.316e-1	204	108	204.0000	122.2408	AAL93745.1| SSU ribosomal protein S8P
					77	90	98.6554	110.0102	
FN1631	1.383	8.384			27	10	27.0000	11.3186	AAL93746.1| SSU ribosomal protein S14P
					25		32.0310		
FN1632	-0.245	17.018	1.921e-3	5.772e-3	326	367	326.0000	415.3922	AAL93747.1| LSU ribosomal protein L5P
					268	309	343.3722	377.7017	
FN1634	0.349	10.575	2.107e-2	9.573e-2	51	32	51.0000	36.2195	AAL93749.1| LSU ribosomal protein L24P
					29	27	37.1559	33.0031	
FN1635	0.958	11.759	4.306e-3	1.526e-2	68	39	68.0000	44.1425	AAL93750.1| LSU ribosomal protein L14P
					75	33	96.0930	40.3371	
FN1636	-1.114	11.264	2.879e-3	9.391e-3	29	76	29.0000	86.0213	AAL93751.1| SSU ribosomal protein S17P
					30	49	38.4372	59.8944	
FN1637	-0.289	10.697	1.072e-1	6.077e-1	34	18	34.0000	20.3735	AAL93752.1| LSU ribosomal protein L29P
					31	57	39.7184	69.6731	
FN1638	-0.088	16.494	2.375e-2	1.096e-1	292	264	292.0000	298.8108	AAL93753.1| LSU ribosomal protein L16P
					232	268	297.2476	327.5859	
FN1639	0.286	19.945	1.71e-2	7.562e-2	1241	778	1241.0000	880.5863	AAL93754.1| SSU ribosomal protein S3P
					763	768	977.5857	938.7537	
FN1640	1.630	15.969	1.477e-3	4.125e-3	380	128	380.0000	144.8779	AAL93755.1| LSU ribosomal protein L22P
					399	117	511.2145	143.0133	
FN1641	-0.028	15.694	1.364e-1	7.991e-1	246	225	246.0000	254.6683	AAL93756.1| SSU ribosomal protein S19P
					164	172	210.1233	210.2417	
FN1642	-1.107	16.915	2.324e-4	3.626e-4	233	429	233.0000	485.5675	AAL93757.1| LSU ribosomal protein L2P
					192	447	245.9980	546.3840	
FN1643	-0.010	12.933	1.377e-1	8.084e-1	84	78	84.0000	88.2850	AAL93758.1| LSU ribosomal protein L23P
					72	73	92.2492	89.2305	
FN1644	0.360	20.141	3.73e-5	2.586e-5	1202	862	1202.0000	975.6624	AAL93759.1| LSU ribosomal protein L1E
					963	755	1233.8336	922.8633	
FN1645	0.481	18.801	1.256e-4	1.503e-4	764	528	764.0000	597.6215	AAL93760.1| LSU ribosomal protein L3P
					650	447	832.8056	546.3840	

Log$_2$ Ratios: -6 -4 -2 0 2 4 6

Show detected proteins only
Show all proteins
Filter by category:
GO: amino acid transport

Proteins found: 1297

Enter (or paste) list of ORFs

Find ORFs

Test	Cutoff
q-Value	.005
p-Value	

	Signif	Direction	Applies To
red	yes	+	ratios, bars
yellow	no	n/a	bars
green	yes	–	ratios, bars
pink	yes	+	*p*- , *q*-Values
light green	yes	–	

Dot Plots Dot Plots

Protein	*FnPgSg* vs Fn				Raw		Normalized		Description
	Log_2 Ratio	Log_2 Sum	*q*-Value	*p*-Value	*FnPgSg*	*Fn*	*FnPgSg*	*Fn*	
FN1646	-0.451	15.567	4.517e-3	1.616e-2	163	223	163.0000	252.4045	AAL93761.1| SSU ribosomal protein S10P
					167	215	213.9670	262.8021	
FN1647	0.273	17.449	3.025e-2	1.451e-1	530	384	530.0000	434.6338	AAL93762.1| Hypothetical protein
					312	274	399.7467	334.9199	
FN1652	-1.164	14.047	4.821e-5	3.877e-5	88	167	88.0000	189.0204	AAL93767.1| Oligopeptide-binding protein oppA
					67	164	85.8430	200.4630	
FN1654	0.302	9.947	1.47e-2	6.368e-2	39	23	39.0000	26.0328	AAL93769.1| Hypothetical protein
					24	25	30.7497	30.5584	
FN1655	-1.198	11.651	1.272e-6	1.857e-7	39	74	39.0000	83.7576	AAL93770.1| Hypothetical cytosolic protein
					28	72	35.8747	88.0082	
FN1656					21		21.0000		AAL93771.1| SSU ribosomal protein S18P
					27		34.5935		
FN1657	-1.602	13.685	6.451e-5	5.802e-5	60	188	60.0000	212.7895	AAL93772.1| SSU ribosomal protein S6P
					56	153	71.7494	187.0173	
FN1658	0.034	15.275	1.224e-1	7.062e-1	189	188	189.0000	212.7895	AAL93773.1| Prolyl-tRNA synthetase
					167	148	213.9670	180.9057	
FN1660									AAL93775.1| ATP-dependent DNA helicase recG
						4		4.8893	
FN1661	-0.035	10.939	1.472e-1	8.735e-1	44	49	44.0000	55.4611	AAL93776.1| Hypothetical cytosolic protein
					34	28	43.5621	34.2254	
FN1662	-0.570	9.943	6.345e-3	2.424e-2	31	33	31.0000	37.3513	AAL93777.1| Hypothetical protein
					16	32	20.4998	39.1147	
FN1663	0.687	7.648	8.592e-6	2.604e-6	18	10	18.0000	11.3186	AAL93778.1| Hypothetical protein
					14	9	17.9374	11.0010	
FN1666									AAL93781.1| Hypothetical protein
						3		3.6670	
FN1670	-1.431	10.553	5.101e-5	4.216e-5	28	52	28.0000	58.8567	AAL93785.1| Choline kinase
					15	56	19.2186	68.4508	
FN1671						5		5.6593	AAL93786.1| hypothetical exported protein
						4		4.8893	
FN1679	0.061	16.695	9.283e-2	5.17e-1	354	258	354.0000	292.0196	AAL93794.1| LPS biosynthesis protein WbpG
					243	283	311.3412	345.9209	

Log_2 Ratios: -6 -4 -2 0 2 4 6

Show detected proteins only
Show all proteins
Filter by category:
GO: amino acid transport

Proteins found: 1297

Enter (or paste) list of ORFs

Find ORFs

Test	Cutoff
q-Value	.005
p-Value	

	Signif	Direction	Applies To
red	yes	+	ratios, bars
yellow	no	n/a	bars
green	yes	−	ratios, bars
pink	yes	+	*p*-, *q*-Values
light green	yes	−	

Dot Plots Dot Plots

Protein	*FnPgSg vs Fn* Log2 Ratio	Log2 Sum	*q*-Value	*p*-Value	Raw *FnPgSg*	Raw *Fn*	Normalized *FnPgSg*	Normalized *Fn*	Description
FN1683	-1.101	12.498	1.248e-3	3.237e-3	68	91	68.0000	102.9992	AAL93798.1| Acetyltransferase
					28	98	35.8747	119.7889	
FN1684	0.149	16.554	8.54e-3	3.426e-2	311	257	311.0000	290.8878	AAL93799.1| N-acetylneuraminate synthase
					267	244	342.0909	298.2499	
FN1685	-0.397	11.898	1.175e-2	4.947e-2	50	54	50.0000	61.1204	AAL93800.1| dTDP-4-dehydrorhamnose reductase
					45	66	57.6558	80.6741	
FN1686	0.065	15.493	1.181e-1	6.782e-1	256	182	256.0000	205.9983	AAL93801.1| Spore coat polysaccharide biosynthesis protein spsF
					143	175	183.2172	213.9087	
FN1687	-0.132	15.420	7.712e-2	4.193e-1	222	221	222.0000	250.1408	AAL93802.1| Gluconate 5-dehydrogenase
					139	154	178.0923	188.2397	
FN1688	0.776	12.314	4.389e-5	3.388e-5	92	51	92.0000	57.7248	AAL93803.1| Oxidoreductase
					74	42	94.8117	51.3381	
FN1689	-0.178	18.102	1.501e-3	4.217e-3	494	484	494.0000	547.8197	AAL93804.1| UDP-N-acetylglucosamine 4,6-dehydratase
					393	475	503.5271	580.6094	
FN1690	-1.672	10.587	3.332e-5	2.057e-5	26	60	26.0000	67.9115	AAL93805.1| Hypothetical protein
					14	59	17.9374	72.1178	
FN1692	-0.883	11.575	7.625e-3	3e-2	57	71	57.0000	80.3620	AAL93807.1| Glycosyl transferase
					19	57	24.3435	69.6731	
FN1693	-0.112	8.271	1.084e-1	6.151e-1	21	15	21.0000	16.9779	AAL93808.1| Hypothetical protein
					10	16	12.8124	19.5574	
FN1694	-0.393	13.401	3.491e-5	2.253e-5	88	108	88.0000	122.2408	AAL93809.1| UDP-N-acetyl-D-quinovosamine 4-epimerase
					73	95	93.5305	116.1219	
FN1695	0.292	10.167	2.494e-2	1.159e-1	43	25	43.0000	28.2965	AAL93810.1| Probable quinovosaminephosphotransferae
					25	27	32.0310	33.0031	
FN1696	-0.144	14.664	1.379e-2	5.92e-2	163	147	163.0000	166.3833	AAL93811.1| UDP-N-acetylglucosamine 4,6-dehydratase
					112	141	143.4988	172.3493	
FN1697	-1.167	13.721	6.157e-4	1.301e-3	68	136	68.0000	153.9328	AAL93812.1| Hypothetical protein
					68	159	87.1243	194.3513	
FN1698	-0.074	12.875	1.006e-1	5.662e-1	78	88	78.0000	99.6036	AAL93813.1| dTDP-4-dehydrorhamnose reductase
					71	64	90.9680	78.2295	
FN1700									AAL93815.1| Hypothetical protein
						9		11.0010	

Log2 Ratios: -6 -4 -2 0 2 4 6

Show detected proteins only
Show all proteins
Filter by category:
GO: amino acid transport

Proteins found: 1297

Enter (or paste) list of ORFs

Find ORFs

Test	Cutoff
q-Value	.005
p-Value	

	Signif	Direction	Applies To
red	yes	+	ratios, bars
yellow	no	n/a	bars
green	yes	−	ratios, bars
pink	yes	+	*p*- , *q*-Values
light green	yes	−	

Dot Plots Dot Plots

Protein	*FnPgSg* vs *Fn* Log$_2$ Ratio	Log$_2$ Sum	*q*-Value	*p*-Value	Raw *FnPgSg*	Raw *Fn*	Normalized *FnPgSg*	Normalized *Fn*	Description
FN1701	-1.255	10.774	1.84e-5	8.057e-6	26	57	26.0000	64.5160	AAL93816.1| ABC transporter ATP-binding protein
					22	53	28.1873	64.7838	
FN1703	-0.918	13.513	3.033e-5	1.73e-5	74	133	74.0000	150.5372	AAL93818.1| ADP-L-glycero-D-manno-heptose-6-epimerase
					65	120	83.2806	146.6803	
FN1704	-1.512	7.127			7	18	7.0000	20.3735	AAL93819.1| Serine protease
						16		19.5574	
FN1707						7		7.9230	AAL93822.1| Aldose 1-epimerase
						4		4.8893	
FN1708	-0.504	18.437	5.877e-6	1.441e-6	488	634	488.0000	717.5986	AAL93823.1| Polyribonucleotide nucleotidyltransferase
					400	574	512.4958	701.6206	
FN1711	-2.157	5.706	2.347e-6	4.111e-7	3	14	3.0000	15.8460	AAL93826.1| Methyltransferase
					3	12	3.8437	14.6680	
FN1713									AAL93828.1| tRNA (Uracil-5-) -methyltransferase
						6		7.3340	
FN1717	-2.442	9.822	2.502e-4	3.989e-4	13	57	13.0000	64.5160	AAL93832.1| NAD-dependent DNA ligase
					10	62	12.8124	75.7848	
FN1718	-0.953	17.908	2.743e-4	4.488e-4	372	569	372.0000	644.0277	AAL93833.1| Protein translocase subunit secA
					266	602	340.8097	735.8460	
FN1719	-0.715	16.583	3.33e-5	2.054e-5	261	341	261.0000	385.9639	AAL93834.1| Hypothetical protein
					178	341	228.0606	416.8164	
FN1722						7		7.9230	AAL93837.1| Glucose inhibited division protein B
						4		4.8893	
FN1723	-0.389	13.184	8.235e-3	3.282e-2	93	86	93.0000	97.3399	AAL93838.1| Glucose inhibited division protein A
					59	101	75.5931	123.4559	
FN1724	-0.577	6.699			9	11	9.0000	12.4504	AAL93839.1| Potassium uptake protein KtrA
					6		7.6874		
FN1727					4		4.0000		AAL93842.1| Chloride channel protein
					4		5.1250		
FN1728	-1.893	6.917	2.063e-6	3.481e-7	5	18	5.0000	20.3735	AAL93843.1| Pyrrolidone-carboxylate peptidase
					5	18	6.4062	22.0020	
FN1730	-1.332	7.217				18		20.3735	AAL93845.1| Para-aminobenzoate synthase component I
					6	15	7.6874	18.3350	

Log$_2$ Ratios: -6 -4 -2 0 2 4 6

Show detected proteins only
Show all proteins
Filter by category:
GO: amino acid transport

Proteins found: 1297

Enter (or paste) list of ORFs

Find ORFs

Test	Cutoff
q-Value	.005
p-Value	

	Signif	Direction	Applies To
red	yes	+	ratios, bars
yellow	no	n/a	bars
green	yes	−	ratios, bars
pink	yes	+	*p*- , *q*-Values
light green	yes	−	

Dot Plots Dot Plots

Protein	*FnPgSg vs Fn* Log$_2$ Ratio	Log$_2$ Sum	*q*-Value	*p*-Value	Raw *FnPgSg*	Raw *Fn*	Normalized *FnPgSg*	Normalized *Fn*	Description
FN1731					16		16.0000		AAL93846.1| Anthranilate synthase component II
FN1732	-0.305	8.862	6.336e-2	3.367e-1	26	24	26.0000	27.1646	AAL93847.1| Hypothetical protein
					10	17	12.8124	20.7797	
FN1734	0.282	13.552	2.984e-2	1.427e-1	102	86	102.0000	97.3399	AAL93849.1| V-type sodium ATP synthase subunit B
					109	83	139.6551	101.4538	
FN1735	-0.552	11.297	1.147e-3	2.875e-3	38	49	38.0000	55.4611	AAL93850.1| V-type sodium ATP synthase subunit A
					35	54	44.8434	66.0061	
FN1736	-0.692	6.687	3.585e-4	6.464e-4	7	12	7.0000	13.5823	AAL93851.1| V-type sodium ATP synthase subunit A
					7	10	8.9687	12.2234	
FN1737						5		5.6593	AAL93852.1| V-type sodium ATP synthase subunit G
						9		11.0010	
FN1738	-0.713	8.155	3.759e-3	1.291e-2	11	22	11.0000	24.9009	AAL93853.1| V-type sodium ATP synthase subunit C
					12	15	15.3749	18.3350	
FN1739	1.461	6.684			17		17.0000		AAL93854.1| V-type sodium ATP synthase subunit E
					13	5	16.6561	6.1117	
FN1741	-1.416	6.966	4.968e-5	4.052e-5	6	15	6.0000	16.9779	AAL93856.1| V-type sodium ATP synthase subunit I
					6	16	7.6874	19.5574	
FN1745	0.611	5.434	2.23e-2	1.021e-1	6	4	6.0000	4.5274	AAL93860.1| Cystathionine gamma-synthase
					8	5	10.2499	6.1117	
FN1752					3		3.0000		AAL93867.1| Regulatory protein TENI
FN1763	-0.243	7.602				16		18.1097	AAL93876.1| Hypothetical cytosolic protein
					10	10	12.8124	12.2234	
FN1764	-0.224	23.234	2.337e-4	3.655e-4	2954	3002	2954.0000	3397.8406	AAL93877.1| Enolase
					2231	2773	2858.4452	3389.5364	
FN1765	-0.880	19.371	5.355e-5	4.511e-5	563	933	563.0000	1056.0244	AAL93878.1| Pyruvate kinase
					508	964	650.8696	1178.3314	
FN1780	-0.124	9.621	7.404e-2	4.005e-1	23	28	23.0000	31.6921	AAL93879.1| Hypothetical protein
					24	22	30.7497	26.8914	
FN1781	-0.570	18.459	1.946e-4	2.787e-4	496	666	496.0000	753.8181	AAL93880.1| LytB protein
					382	580	489.4335	708.9546	

Log$_2$ Ratios

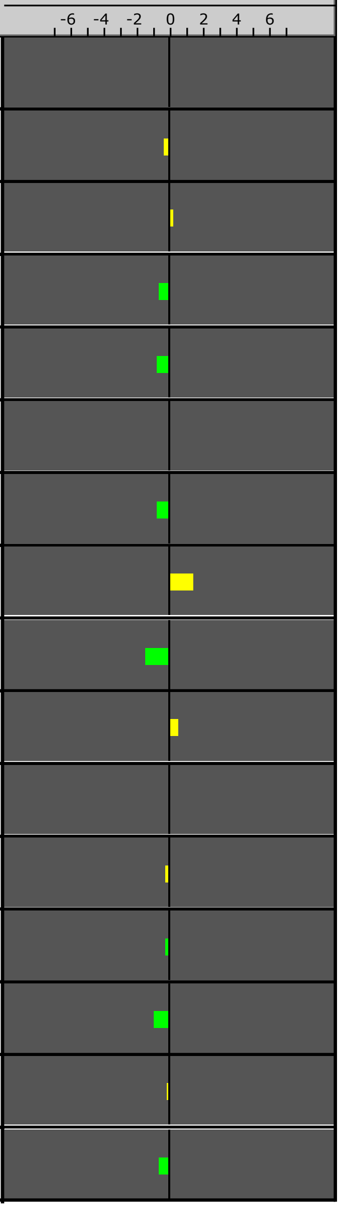

Show detected proteins only
Show all proteins
Filter by category:
GO: amino acid transport

Proteins found: 1297

Enter (or paste) list of ORFs

Find ORFs

Test	Cutoff
q-Value	.005
p-Value	

	Signif	Direction	Applies To
(red)	yes	+	ratios, bars
(yellow)	no	n/a	bars
(green)	yes	−	ratios, bars
(light red)	yes	+	*p*- , *q*-Values
(light green)	yes	−	

Dot Plots | Dot Plots

Protein	*FnPgSg vs Fn* Log$_2$ Ratio	Log$_2$ Sum	*q*-Value	*p*-Value	Raw *FnPgSg*	Raw *Fn*	Normalized *FnPgSg*	Normalized *Fn*	Description
FN1784	-2.082	7.441				22		24.9009	AAL93883.1| unknown
					5	24	6.4062	29.3361	
FN1785	0.622	10.161	4.805e-2	2.473e-1	25	32	25.0000	36.2195	AAL93884.1| Hypothetical protein
					46	15	58.9370	18.3350	
FN1786	-0.317	11.942	6.397e-4	1.366e-3	56	60	56.0000	67.9115	AAL93885.1| ADP-heptose synthase
					44	59	56.3745	72.1178	
FN1787	-1.042	11.114	1.322e-3	3.521e-3	40	60	40.0000	67.9115	AAL93886.1| Tetratricopeptide repeat family protein
					20	55	25.6248	67.2285	
FN1788	0.133	9.007	7.27e-2	3.924e-1	27	21	27.0000	23.7690	AAL93887.1| 2C-methyl-D-erythritol 2,4-cyclodiphosphate synthase
					16	16	20.4998	19.5574	
FN1790	1.328	5.077			12		12.0000		AAL93889.1| Cob(I)alamin adenosyltransferase
					5	3	6.4062	3.6670	
FN1792	2.062	24.088	8.106e-9	5.46e-11	8681	1765	8681.0000	1997.7310	AAL93891.1| Hypothetical protein
					6695	1746	8577.8981	2134.1978	
FN1793	-0.653	13.634	8.342e-5	8.319e-5	85	131	85.0000	148.2735	AAL93892.1| Phosphoenolpyruvate-protein phosphotransferase
					74	110	94.8117	134.4569	
FN1794	-0.325	14.614	2.967e-2	1.418e-1	110	148	110.0000	167.5151	AAL93893.1| Phosphocarrier protein HPr
					135	153	172.9673	187.0173	
FN1796									AAL93895.1| unknown
						5		6.1117	
FN1797	-1.666	11.284	1.595e-5	6.328e-6	33	74	33.0000	83.7576	AAL93896.1| Spermidine/putrescine transport ATP-binding protein potA
					18	77	23.0623	94.1198	
FN1798	-1.273	6.225			6		6.0000		AAL93897.1| Spermidine/putrescine transport system permease protein potB
					4	11	5.1250	13.4457	
FN1800	-0.311	14.924	9.349e-3	3.815e-2	177	172	177.0000	194.6797	AAL93899.1| Peptidyl-prolyl cis-trans isomerase
					109	162	139.6551	198.0184	
FN1801	-0.652	6.267			7		7.0000		AAL93900.1| Sodium/glutamate symport carrier protein
						9		11.0010	
FN1803	1.473	7.445			22	7	22.0000	7.9230	AAL93902.1| Transcriptional regulator, TetR family
FN1804						11		12.4504	AAL93903.1| Aminoacyl-histidine dipeptidase
						10		12.2234	

Log$_2$ Ratios: -6 -4 -2 0 2 4 6

Show detected proteins only
Show all proteins
Filter by category:
GO: amino acid transport

Proteins found: 1297

Enter (or paste) list of ORFs

Find ORFs

Test	Cutoff
q-Value	.005
p-Value	

	Signif	Direction	Applies To
red	yes	+	ratios, bars
yellow	no	n/a	bars
green	yes	−	ratios, bars
pink	yes	+	*p*- , *q*-Values
light green	yes	−	

Dot Plots | Dot Plots

Protein	*FnPgSg* vs Fn Log_2 Ratio	Log_2 Sum	*q*-Value	*p*-Value	Raw *FnPgSg*	Raw *Fn*	Normalized *FnPgSg*	Normalized *Fn*	Description
FN1807	0.029	17.351	5.788e-2	3.049e-1	407	348	407.0000	393.8869	AAL93906.1| Hypothetical protein
					327	340	418.9653	415.5941	
FN1808	-1.913	11.762	3.078e-4	5.231e-4	30	93	30.0000	105.2629	AAL93907.1| Hypothetical protein
					24	101	30.7497	123.4559	
FN1809	0.257	8.951	5.993e-2	3.168e-1	23	23	23.0000	26.0328	AAL93908.1| Iron/zinc/copper-binding protein
					20	12	25.6248	14.6680	
FN1811	-1.648	10.922	3.729e-5	2.584e-5	19	73	19.0000	82.6257	AAL93910.1| Manganese transport system ATP-binding protein mntA
					24	60	30.7497	73.3401	
FN1812	-0.884	14.299	1.088e-4	1.234e-4	95	167	95.0000	189.0204	AAL93911.1| Manganese-binding protein
					89	161	114.0303	196.7960	
FN1813	-1.315	9.374	1.684e-4	2.261e-4	16	34	16.0000	38.4832	AAL93912.1| Manganese-binding protein
					13	35	16.6561	42.7817	
FN1814	-1.431	8.650	2.654e-3	8.491e-3	18	29	18.0000	32.8239	AAL93913.1| Hypothetical protein
					5	27	6.4062	33.0031	
FN1816	-0.306	5.795	3.338e-2	1.631e-1	7	6	7.0000	6.7912	AAL93915.1| unknown
					5	8	6.4062	9.7787	
FN1817	0.425	6.174			12		12.0000		AAL93916.1| Hemolysin
					6	6	7.6874	7.3340	
FN1819						8		9.0549	AAL93918.1| Export ABC transporter
						12		14.6680	
FN1820						8		9.0549	AAL93919.1| Export ABC transporter
						6		7.3340	
FN1822						16		18.1097	AAL93921.1| Flavodoxin
						10		12.2234	
FN1825					10		10.0000		AAL93924.1| Hypothetical protein
					7		8.9687		
FN1826	-0.410	11.630	5.195e-3	1.912e-2	49	52	49.0000	58.8567	AAL93925.1| Protease
					38	58	48.6871	70.8955	
FN1827	-0.895	10.851	6.268e-3	2.389e-2	31	42	31.0000	47.5381	AAL93926.1| Replicative DNA helicase
					25	57	32.0310	69.6731	
FN1828	-1.285	11.923	1.042e-4	1.164e-4	35	79	35.0000	89.4169	AAL93927.1| LSU ribosomal protein L9P
					35	86	44.8434	105.1209	

Log_2 Ratios: -6 -4 -2 0 2 4 6

Show detected proteins only
Show all proteins
Filter by category:
GO: amino acid transport

Proteins found: 1297

Enter (or paste) list of ORFs

Find ORFs

Test	Cutoff
q-Value	.005
p-Value	

Signif	Direction	Applies To
yes	+	ratios, bars
no	n/a	bars
yes	−	ratios, bars
yes	+	*p*-, *q*-Values
yes	−	

Dot Plots Dot Plots

Protein	*FnPgSg vs Fn* Log2 Ratio	Log2 Sum	*q*-Value	*p*-Value	Raw *FnPgSg*	Raw *Fn*	Normalized *FnPgSg*	Normalized *Fn*	Description
FN1830	-0.965	8.788	1.069e-4	1.205e-4	16	26	16.0000	29.4283	AAL93929.1| DNA polymerase III subunits gamma and tau
					11	24	14.0936	29.3361	
FN1831	-0.796	11.183	3.185e-3	1.06e-2	45	55	45.0000	62.2522	AAL93930.1| Nitrogen assimilation regulatory protein
					22	53	28.1873	64.7838	
FN1832					4		4.0000		AAL93931.1| TonB protein
FN1833					24		24.0000		AAL93932.1| Biopolymer transport exbD protein
					15		19.2186		
FN1834	-0.833	10.984	2.285e-4	3.536e-4	29	50	29.0000	56.5929	AAL93933.1| Biopolymer transport exbB protein
					30	52	38.4372	63.5614	
FN1836	-0.525	13.016	6.48e-5	5.838e-5	80	100	80.0000	113.1859	AAL93935.1| Tetratricopeptide repeat family protein
					56	86	71.7494	105.1209	
FN1838					11		11.0000		AAL93937.1| Glycerol uptake facilitator protein
FN1839	1.061	15.629	1.862e-3	5.55e-3	284	135	284.0000	152.8010	AAL93938.1| Glycerol kinase
					286	130	366.4345	158.9036	
FN1840	1.679	11.320	5.464e-7	5.367e-8	90	24	90.0000	27.1646	AAL93939.1| Dihydroxyacetone kinase
					71	24	90.9680	29.3361	
FN1841	-0.747	8.326	2.398e-2	1.108e-1	11	14	11.0000	15.8460	AAL93940.1| Dihydroxyacetone kinase
					13	25	16.6561	30.5584	
FN1842	0.943	10.274	6.731e-4	1.459e-3	54	20	54.0000	22.6372	AAL93941.1| Dihydroxyacetone kinase phosphotransfer protein
					34	23	43.5621	28.1137	
FN1844									AAL93943.1| Ketoacyl reductase hetN
						10		12.2234	
FN1847						11		12.4504	AAL93946.1| DTDP-4-dehydrorhamnose 3,5-epimerase
						4		4.8893	
FN1848									AAL93947.1| Metal dependent hydrolase
					4		5.1250		
FN1849	-2.749	6.749			4	27	4.0000	30.5602	AAL93948.1| Coenzyme F390 synthetase
						19		23.2244	
FN1850						5		5.6593	AAL93949.1| 3-oxoacyl-[acyl-carrier-protein] synthase III
						7		8.5563	

Log2 Ratios: -6 -4 -2 0 2 4 6

Show detected proteins only
Show all proteins
Filter by category:
GO: amino acid transport

Proteins found: 1297

Enter (or paste) list of ORFs

Find ORFs

Test	Cutoff
q-Value	.005
p-Value	

Color	Signif	Direction	Applies To
red	yes	+	ratios, bars
yellow	no	n/a	bars
green	yes	−	ratios, bars
light red	yes	+	*p*- , *q*-Values
light green	yes	−	*p*- , *q*-Values

Dot Plots Dot Plots

Protein	*FnPgSg vs Fn* Log_2 Ratio	Log_2 Sum	*q*-Value	*p*-Value	Raw *FnPgSg*	Raw *Fn*	Normalized *FnPgSg*	Normalized *Fn*	Description
**FN1851**	-0.557	11.361	7.598e-5	7.293e-5	41	57	41.0000	64.5160	AAL93950.1| Ribonuclease PH
					34	49	43.5621	59.8944	
**FN1852**	0.067	10.712	6.879e-2	3.689e-1	39	34	39.0000	38.4832	AAL93951.1| unknown
					35	34	44.8434	41.5594	
**FN1853**	-1.173	6.196	1.402e-3	3.832e-3	5	13	5.0000	14.7142	AAL93952.1| Methylaspartate mutase
					5	9	6.4062	11.0010	
**FN1854**	-0.915	10.472	6.436e-7	6.825e-8	28	45	28.0000	50.9337	AAL93953.1| Methylaspartate mutase
					21	43	26.9060	52.5604	
**FN1856**	-0.236	23.088	9.181e-3	3.733e-2	2988	2855	2988.0000	3231.4573	AAL93955.1| Butyrate-acetoacetate CoA-transferase subunit B
					1963	2657	2515.0730	3247.7454	
**FN1857**	0.190	18.976	2.879e-2	1.369e-1	687	633	687.0000	716.4667	AAL93956.1| Acetoacetate: butyrate/acetate coenzyme A transferase
					661	514	846.8993	628.2805	
**FN1858**	0.610	12.869	5.854e-3	2.203e-2	92	60	92.0000	67.9115	AAL93957.1| Short-chain fatty acids transporter
					95	59	121.7177	72.1178	
**FN1859**	-1.875	22.425	5.322e-4	1.08e-3	1120	4473	1120.0000	5062.8051	AAL93958.1| Major outer membrane protein
					1060	3294	1358.1138	4026.3732	
**FN1860**	0.578	9.807	2.499e-2	1.162e-1	27	26	27.0000	29.4283	AAL93959.1| NA+/H+ antiporter NHAC
					36	16	46.1246	19.5574	
**FN1862**	0.075	15.728	4.34e-2	2.207e-1	249	211	249.0000	238.8222	AAL93961.1| L-beta-lysine 5,6-aminomutase beta subunit
					179	176	229.3419	215.1310	
**FN1863**	0.824	17.264	5.428e-4	1.107e-3	483	246	483.0000	278.4373	AAL93962.1| L-beta-lysine 5,6-aminomutase alpha subunit
					447	260	572.7140	317.8072	
**FN1864**	-0.129	8.808	9.99e-2	5.618e-1	20	24	20.0000	27.1646	AAL93963.1| DNA mismatch repair protein mutS
					16	14	20.4998	17.1127	
**FN1865**	-0.594	3.764			3	4	3.0000	4.5274	AAL93964.1| Hypothetical protein
**FN1866**	-0.041	21.135	7.773e-2	4.231e-1	1420	1326	1420.0000	1500.8450	AAL93965.1| Lysine 2,3-aminomutase
					1227	1291	1572.0808	1578.0351	
**FN1867**	0.438	19.764	1.161e-3	2.926e-3	1165	736	1165.0000	833.0482	AAL93966.1| Zn-dependent alcohol dehydrogenases and related dehydrogenases
					805	645	1031.3978	788.4064	
**FN1868**	-0.191	14.985	7.034e-3	2.731e-2	168	179	168.0000	202.6028	AAL93967.1| Hypothetical cytosolic protein
					132	149	169.1236	182.1280	

Log_2 Ratios: -6 -4 -2 0 2 4 6

Show detected proteins only
Show all proteins
Filter by category:
GO: amino acid transport

Proteins found: 1297

Enter (or paste) list of ORFs

Find ORFs

Test	Cutoff
q-Value	.005
p-Value	

Signif	Direction	Applies To
yes	+	ratios, bars
no	n/a	bars
yes	−	ratios, bars
yes	+	*p*-, *q*-Values
yes	−	

Dot Plots Dot Plots

Protein	*FnPgSg vs Fn* Log2 Ratio	Log2 Sum	*q*-Value	*p*-Value	Raw *FnPgSg*	Raw *Fn*	Normalized *FnPgSg*	Normalized *Fn*	Description
FN1869	-0.375	15.226	4.489e-3	1.604e-2	167	180	167.0000	203.7346	AAL93968.1| Hypothetical protein
					138	198	176.8110	242.0224	
FN1870	-0.932	4.817							AAL93969.1| unknown
					3	6	3.8437	7.3340	
FN1871						9		10.1867	AAL93970.1| unknown
						9		11.0010	
FN1872						17		19.2416	AAL93971.1| unknown
						19		23.2244	
FN1873	-0.124	11.905	5.623e-2	2.954e-1	52	57	52.0000	64.5160	AAL93972.1| Bis(5'-nucleosyl)-tetraphosphatase
					52	53	66.6245	64.7838	
FN1874	-1.083	10.169	9.898e-5	1.073e-4	21	43	21.0000	48.6699	AAL93973.1| Ribose 5-phosphate isomerase
					20	41	25.6248	50.1158	
FN1875	-0.055	13.845	1.35e-1	7.898e-1	133	131	133.0000	148.2735	AAL93974.1| Peptidyl-prolyl cis-trans isomerase
					82	81	105.0616	99.0092	
FN1877					7		7.0000		AAL93976.1| Guanine-hypoxanthine permease
FN1878	-1.230	5.874			5	11	5.0000	12.4504	AAL93977.1| unknown
						9		11.0010	
FN1879	-0.567	10.101			25		25.0000		AAL93978.1| SSU ribosomal protein S20P
					23	33	29.4685	40.3371	
FN1880	0.261	11.039	6.04e-2	3.195e-1	62	33	62.0000	37.3513	AAL93979.1| Oxygen-insensitive NAD(P)H nitroreductase
					30	38	38.4372	46.4487	
FN1881					6		6.0000		AAL93980.1| Esterase
FN1884	0.857	12.449	9.932e-3	4.091e-2	77	55	77.0000	62.2522	AAL93983.1| unknown
					97	40	124.2802	48.8934	
FN1890	1.438	7.041	7.304e-4	1.61e-3	16	8	16.0000	9.0549	AAL93989.1| Hypothetical protein
					17	4	21.7811	4.8893	
FN1891	0.151	7.369	1.013e-1	5.703e-1	13	14	13.0000	15.8460	AAL93990.1| Glycerophosphoryl diester phosphodiesterase
					11	7	14.0936	8.5563	
FN1893	-1.052	18.021	3.598e-4	6.499e-4	309	648	309.0000	733.4446	AAL93991.1| Fusobacterium outer membrane protein family
					318	615	407.4341	751.7363	

Log2 Ratios: -6 -4 -2 0 2 4 6

Show detected proteins only
Show all proteins
Filter by category:
GO: amino acid transport

Proteins found: 1297

Enter (or paste) list of ORFs

Find ORFs

Test	Cutoff
q-Value	.005
p-Value	

Colour	Signif	Direction	Applies To
red	yes	+	ratios, bars
yellow	no	n/a	bars
green	yes	−	ratios, bars
light red	yes	+	*p*- , *q*-Values
light green	yes	−	*p*- , *q*-Values

Dot Plots Dot Plots

Protein	FnPgSg vs Fn: Log_2 Ratio	FnPgSg vs Fn: Log_2 Sum	FnPgSg vs Fn: *q*-Value	FnPgSg vs Fn: *p*-Value	Raw: *FnPgSg*	Raw: *Fn*	Normalized: *FnPgSg*	Normalized: *Fn*	Description
**FN1895**					5		5.0000		AAL93994.1| Hypothetical protein
**FN1898**	-0.600	10.928	5.152e-3	1.893e-2	32	55	32.0000	62.2522	AAL93997.1| Sugar transport ATP-binding protein
					31	38	39.7184	46.4487	
**FN1899**	-0.635	14.521	4.938e-4	9.835e-4	109	181	109.0000	204.8665	AAL93998.1| Hypothetical lipoprotein
					107	145	137.0926	177.2386	
**FN1902**	-0.214	9.710	3.108e-2	1.498e-1	23	27	23.0000	30.5602	AAL94001.1| Deoxycytidylate deaminase
					24	26	30.7497	31.7807	
**FN1903**	1.093	12.958	4.744e-5	3.787e-5	126	55	126.0000	62.2522	AAL94002.1| Coenzyme A disulfide reductase/ disulfide bond regulator domain
					105	49	134.5301	59.8944	
**FN1905**	-0.290	3.460			3		3.0000		AAL94004.1| outer membrane protein
						3		3.6670	
**FN1906**	-0.001	16.361	1.635e-1	9.896e-1	320	257	320.0000	290.8878	AAL94005.1| Cytosol aminopeptidase
					203	237	260.0916	289.6935	
**FN1908**	-0.372	15.435	6.722e-3	2.592e-2	192	233	192.0000	263.7231	AAL94007.1| Glycerophosphoryl diester phosphodiesterase
					139	176	178.0923	215.1310	
**FN1909**	-0.228	14.218	5.994e-4	1.256e-3	127	129	127.0000	146.0098	AAL94008.1| UDP-3-O-[3-hydroxymyristoyl] glucosamine N-acyltransferase
					100	125	128.1239	152.7919	
**FN1910**	0.532	17.313	1.498e-3	4.204e-3	454	257	454.0000	290.8878	AAL94009.1| periplasmic protein
					403	311	516.3395	380.1463	
**FN1911**	0.105	21.007	3.143e-2	1.518e-1	1561	1318	1561.0000	1491.7901	AAL94010.1| Outer membrane protein
					1131	1070	1449.0818	1307.8990	
**FN1912**	-2.460	7.175				25		28.2965	AAL94011.1| Hypothetical protein
					4	23	5.1250	28.1137	
**FN1913**	-0.991	12.146	2.015e-4	2.936e-4	43	77	43.0000	87.1531	AAL94012.1| hydrolase (HD superfamily)
					41	84	52.5308	102.6762	
**FN1914**	-0.623	12.497	1.233e-3	3.184e-3	70	76	70.0000	86.0213	AAL94013.1| Anti-sigma F factor antagonist
					41	84	52.5308	102.6762	
**FN1917**									AAL94016.1| tRNA delta(2)-isopentenylpyrophosphate transferase
						3		3.6670	
**FN1918**	0.288	12.341	2.08e-2	9.434e-2	90	59	90.0000	66.7797	AAL94017.1| SPO0B-associated GTP-binding protein
					54	52	69.1869	63.5614	

Log_2 Ratios: -6 -4 -2 0 2 4 6

Show detected proteins only
Show all proteins
Filter by category:
GO: amino acid transport

Proteins found: 1297

Enter (or paste) list of ORFs

Find ORFs

Test	Cutoff
q-Value	.005
p-Value	

	Signif	Direction	Applies To
red	yes	+	ratios, bars
yellow	no	n/a	bars
green	yes	−	ratios, bars
light red	yes	+	*p*- , *q*-Values
light green	yes	−	

Dot Plots Dot Plots

Protein	FnPgSg vs Fn				Raw		Normalized		Description
	Log_2 Ratio	Log_2 Sum	*q*-Value	*p*-Value	*FnPgSg*	*Fn*	*FnPgSg*	*Fn*	
FN1919	-2.787	8.571	1.538e-5	5.96e-6	11	43	11.0000	48.6699	AAL94018.1| Methyltransferase
					3	44	3.8437	53.7828	
FN1922	-0.377	6.262				9		10.1867	AAL94021.1| Hypothetical protein
					6	8	7.6874	9.7787	
FN1923						4		4.5274	AAL94022.1| Adenine-specific methyltransferase
FN1926	0.306	11.621	2.064e-2	9.352e-2	71	46	71.0000	52.0655	AAL94025.1| Nitrogen regulatory IIA protein
					42	40	53.8121	48.8934	
FN1927	-0.585	12.934	1.919e-3	5.766e-3	74	104	74.0000	117.7133	AAL94026.1| DEGV protein
					55	81	70.4682	99.0092	
FN1928	0.186	7.409			15		15.0000		AAL94027.1| Transcriptional regulator, MerR family
					10	10	12.8124	12.2234	
FN1929	-1.330	10.628	1.076e-2	4.475e-2	22	38	22.0000	43.0106	AAL94028.1| Competence-damage protein cinA
					22	68	28.1873	83.1188	
FN1931						4		4.5274	AAL94030.1| Protease
						5		6.1117	
FN1933	-0.152	7.611	5.258e-2	2.737e-1	15	12	15.0000	13.5823	AAL94032.1| Hypothetical protein
					9	13	11.5312	15.8904	
FN1935	-1.754	4.924			3	6	3.0000	6.7912	AAL94034.1| Adenine-specific methyltransferase
						11		13.4457	
FN1939	-0.314	7.208	2.067e-2	9.369e-2	9	11	9.0000	12.4504	AAL94038.1| Hypothetical protein
					10	12	12.8124	14.6680	
FN1941	-0.193	13.273	7.688e-3	3.029e-2	99	94	99.0000	106.3947	AAL94040.1| ClpB protein
					68	87	87.1243	106.3432	
FN1942						4		4.5274	AAL94041.1| putative DNA-binding protein
FN1943	-0.290	19.679	1.37e-3	3.709e-3	828	929	828.0000	1051.4970	AAL94042.1| Tryptophanase
					647	797	828.9619	974.2014	
FN1949	-1.370	5.255				10		11.3186	AAL94045.1| Xaa-Pro dipeptidase
					3	7	3.8437	8.5563	
FN1950	-3.486	8.201				55		62.2522	AAL94046.1| Serine protease
					4	43	5.1250	52.5604	

Log_2 Ratios: -6 -4 -2 0 2 4 6

Show detected proteins only
Show all proteins
Filter by category:
GO: amino acid transport

Proteins found: 1297

Enter (or paste) list of ORFs

Find ORFs

Test	Cutoff
q-Value	.005
p-Value	

	Signif	Direction	Applies To
red	yes	+	ratios, bars
yellow	no	n/a	bars
green	yes	−	ratios, bars
pink	yes	+	*p*- , *q*-Values
light green	yes	−	

Dot Plots Dot Plots

Protein	*FnPgSg* vs *Fn* Log$_2$ Ratio	Log$_2$ Sum	*q*-Value	*p*-Value	Raw *FnPgSg*	Raw *Fn*	Normalized *FnPgSg*	Normalized *Fn*	Description
FN1951						3		3.3956	AAL94047.1| ATPase associated with chromosome architecture/replication
						8		9.7787	
FN1964	-0.805	8.642	6.811e-3	2.631e-2	20	24	20.0000	27.1646	AAL94054.1| O-linked GLCNAC transferase
					8	21	10.2499	25.6690	
FN1965	0.256	10.490	2.682e-2	1.26e-1	47	30	47.0000	33.9558	AAL94055.1| Tetratricopeptide repeat family protein
					28	29	35.8747	35.4477	
FN1966	-0.059	11.533	1.202e-1	6.915e-1	49	42	49.0000	47.5381	AAL94056.1| Hypothetical protein
					45	52	57.6558	63.5614	
FN1970	1.396	6.219			14	4	14.0000	4.5274	AAL94060.1| Hemin-binding periplasmic protein hmuT precursor
						5		6.1117	
FN1971	0.109	8.767	9.539e-2	5.332e-1	19	15	19.0000	16.9779	AAL94061.1| Hemin receptor
					19	19	24.3435	23.2244	
FN1972	0.231	7.335	4.225e-2	2.142e-1	16	11	16.0000	12.4504	AAL94062.1| unknown
					9	9	11.5312	11.0010	
FN1973	2.612	12.252	3.902e-5	2.856e-5	166	24	166.0000	27.1646	AAL94063.1| Translation initiation inhibitor
					140	24	179.3735	29.3361	
FN1974						6		6.7912	AAL94064.1| DNA/RNA helicase (DEAD/DEAH BOX family)
						8		9.7787	
FN1975	-0.653	14.244	1.97e-4	2.837e-4	103	164	103.0000	185.6249	AAL94065.1| ATP-dependent RNA helicase
					93	134	119.1553	163.7930	
FN1976	-1.202	9.819	9.477e-4	2.23e-3	14	46	14.0000	52.0655	AAL94066.1| 4-amino-4-deoxychorismate lyase
					20	32	25.6248	39.1147	
FN1977	-0.923	4.923			4	8	4.0000	9.0549	AAL94067.1| Cell cycle protein MesJ
					5			6.1117	
FN1978	-0.599	14.873	1.937e-4	2.766e-4	129	178	129.0000	201.4709	AAL94068.1| Cell division protein ftsH
					119	184	152.4675	224.9097	
FN1979	1.316	14.456	1.183e-3	3.004e-3	204	75	204.0000	84.8894	AAL94069.1| SSU ribosomal protein S15P
					210	86	269.0603	105.1209	
FN1980									AAL94070.1| Transporter
					3		3.8437		
FN1983	0.554	18.002	2.665e-3	8.536e-3	564	380	564.0000	430.1064	AAL94073.1| Alkyl hydroperoxide reductase C22 protein
					529	340	677.7757	415.5941	

Log$_2$ Ratios: -6 -4 -2 0 2 4 6

Show detected proteins only
Show all proteins
Filter by category:
GO: amino acid transport

Proteins found: 1297

Enter (or paste) list of ORFs

Find ORFs

Test	Cutoff
q-Value	.005
p-Value	

	Signif	Direction	Applies To
red	yes	+	ratios, bars
yellow	no	n/a	bars
green	yes	−	ratios, bars
pink	yes	+	*p*-, *q*-Values
light green	yes	−	

Dot Plots Dot Plots

Protein	*FnPgSg* vs *Fn* Log$_2$ Ratio	Log$_2$ Sum	*q*-Value	*p*-Value	Raw *FnPgSg*	Raw *Fn*	Normalized *FnPgSg*	Normalized *Fn*	Description	Log$_2$ Ratios (-6 -4 -2 0 2 4 6)
FN1984	0.514	16.887	2.091e-3	6.385e-3	376	276	376.0000	312.3931	AAL94074.1| Thioredoxin reductase	
					356	221	456.1212	270.1361		
FN1985	-0.717	12.352	1.767e-3	5.194e-3	50	72	50.0000	81.4938	AAL94075.1| Inner membrane protein	
					49	85	62.7807	103.8985		
FN1986	-0.356	15.861	2.635e-3	8.419e-3	234	248	234.0000	280.7010	AAL94076.1| Hypothetical protein	
					154	222	197.3109	271.3585		
FN1987	-0.545	4.834	1.357e-3	3.658e-3	5	6	5.0000	6.7912	AAL94077.1| Transcriptional regulator, GntR family	
					3	5	3.8437	6.1117		
FN1988	0.987	12.239	3.608e-4	6.525e-4	92	43	92.0000	48.6699	AAL94078.1| Tyrosine phenol-lyase	
					81	41	103.7804	50.1158		
FN1991	-0.018	16.999	9.589e-2	5.363e-1	349	326	349.0000	368.9860	AAL94081.1| Glucosamine-1-phosphate acetyltransferase	
					289	294	370.2782	359.3666		
FN1992	-0.264	15.227	2.353e-4	3.693e-4	173	187	173.0000	211.6576	AAL94082.1| Ribose-phosphate pyrophosphokinase	
					144	178	184.4985	217.5757		
FN1993	0.179	5.180				5		5.6593	AAL94083.1| SUA5 protein	
					5		6.4062			
FN1994	-0.324	11.019	5.567e-3	2.076e-2	43	49	43.0000	55.4611	AAL94084.1| Hypothetical protein	
					30	38	38.4372	46.4487		
FN1995	1.009	7.110	3.173e-2	1.535e-1	9	6	9.0000	6.7912	AAL94085.1| Hypothetical protein	
					19	8	24.3435	9.7787		
FN2001						9		10.1867	AAL94091.1| Hypothetical protein	
						8		9.7787		
FN2007	-1.867	6.961	1.598e-3	4.581e-3	4	15	4.0000	16.9779	AAL94097.1| Glutathione peroxidase	
					6	21	7.6874	25.6690		
FN2008									AAL94098.1| Glycine betaine transport ATP-binding protein	
						3		3.6670		
FN2011	-0.958	14.186	2.145e-4	3.228e-4	87	171	87.0000	193.5479	AAL94101.1| Valyl-tRNA synthetase	
					85	153	108.9054	187.0173		
FN2013						5		5.6593	AAL94103.1| GTP-binding protein	
FN2014	-0.887	15.552	2.858e-5	1.561e-5	152	275	152.0000	311.2612	AAL94104.1| ATP-dependent protease La	
					133	233	170.4048	284.8042		

Show detected proteins only
Show all proteins
Filter by category:
GO: amino acid transport

Proteins found: 1297

Enter (or paste) list of ORFs

Find ORFs

Test	Cutoff
q-Value	.005
p-Value	

Color	Signif	Direction	Applies To
red	yes	+	ratios, bars
yellow	no	n/a	bars
green	yes	–	ratios, bars
pink	yes	+	*p*- , *q*-Values
light green	yes	–	

Dot Plots Dot Plots

FnPgSg vs Fn

Fusobacterium nucleatum

Hackett Laboratory UW

Fn Summary Table | FnPg vs Fn | FnSg vs Fn | FnPgSg vs Fn | FnPgSg vs FnPg | FnSg vs FnPg | FnPgSg vs FnSg | Fn Coverage

Protein	*FnPgSg* vs Fn				Raw		Normalized		Description
	Log_2 Ratio	Log_2 Sum	*q*-Value	*p*-Value	*FnPgSg*	*Fn*	*FnPgSg*	*Fn*	
FN2015	-0.687	13.402	3.541e-4	6.352e-4	73	108	73.0000	122.2408	AAL94105.1| ATP-dependent clp protease ATP-binding subunit clpX
					71	116	90.9680	141.7909	
FN2016	-0.190	10.782	3.211e-2	1.557e-1	35	36	35.0000	40.7469	AAL94106.1| ATP-dependent Clp protease proteolytic subunit
					34	40	43.5621	48.8934	
FN2017	-0.023	19.307	1.39e-1	8.169e-1	711	728	711.0000	823.9933	AAL94107.1| Trigger factor, ppiase
					692	654	886.6177	799.4074	
FN2018	-0.729	10.204	3.476e-4	6.186e-4	29	36	29.0000	40.7469	AAL94108.1| Single-stranded-DNA-specific exonuclease recJ
					19	39	24.3435	47.6711	
FN2019	-0.674	10.230	1.126e-2	4.709e-2	19	32	19.0000	36.2195	AAL94109.1| Ribosome-binding factor A
					28	42	35.8747	51.3381	
FN2020	-0.211	16.009	6.937e-5	6.416e-5	243	242	243.0000	273.9099	AAL94110.1| Bacterial Protein Translation Initiation Factor 2 (IF-2)
					183	228	234.4668	278.6925	
FN2022	-0.503	12.765	7.657e-6	2.177e-6	71	88	71.0000	99.6036	AAL94112.1| N utilization substance protein A
					54	81	69.1869	99.0092	
FN2023	0.064	6.716	1.182e-1	6.787e-1	12	8	12.0000	9.0549	AAL94113.1| Hypothetical cytosolic protein
					7	9	8.9687	11.0010	
FN2030	-0.842	15.443	1.972e-4	2.842e-4	150	265	150.0000	299.9426	AAL94115.1| Inorganic pyrophosphatase
					129	217	165.2799	265.2468	
FN2031	-0.397	7.612	5.555e-2	2.913e-1	9	10	9.0000	11.3186	AAL94116.1| Thiamine biosynthesis lipoprotein apbE
					12	17	15.3749	20.7797	
FN2033						16		18.1097	AAL94118.1| Guanylate kinase
						12		14.6680	
FN2034	0.512	9.490	4.057e-3	1.417e-2	32	17	32.0000	19.2416	AAL94119.1| Protein yicC
					25	21	32.0310	25.6690	
FN2035	-0.320	17.851	1.616e-4	2.134e-4	418	490	418.0000	554.6109	AAL94120.1| DNA-directed RNA polymerase beta' chain
					353	435	452.2775	531.7159	
FN2036	-0.543	17.386	4.911e-4	9.764e-4	327	469	327.0000	530.8418	AAL94121.1| DNA-directed RNA polymerase beta chain
					280	383	358.7470	468.1545	
FN2037	2.706	21.921	2.355e-5	1.146e-5	4959	682	4959.0000	771.9278	AAL94122.1| LSU ribosomal protein L12P (L7/L12)
					4077	645	5223.6132	788.4064	
FN2038	0.235	16.942	3.202e-2	1.552e-1	383	242	383.0000	273.9099	AAL94123.1| LSU ribosomal protein L10P
					302	311	386.9343	380.1463	

Log_2 Ratios: -6 -4 -2 0 2 4 6

- Show detected proteins only
- Show all proteins
- Filter by category:
GO: amino acid transport

Proteins found: 1297

Enter (or paste) list of ORFs

Find ORFs

Test	Cutoff
q-Value	.005
p-Value	

	Signif	Direction	Applies To
red	yes	+	ratios, bars
yellow	no	n/a	bars
green	yes	−	ratios, bars
pink	yes	+	*p*- , *q*-Values
light green	yes	−	

Dot Plots Dot Plots

Protein	*FnPgSg* vs *Fn* Log$_2$ Ratio	Log$_2$ Sum	*q*-Value	*p*-Value	Raw *FnPgSg*	Raw *Fn*	Normalized *FnPgSg*	Normalized *Fn*	Description
FN2039	-0.291	16.964	2.548e-4	4.082e-4	316	349	316.0000	395.0188	AAL94124.1| LSU ribosomal protein L1P
					258	324	330.5598	396.0367	
FN2040	0.868	14.519	2.292e-4	3.552e-4	223	114	223.0000	129.0319	AAL94125.1| LSU ribosomal protein L11P
					149	80	190.9047	97.7868	
FN2041	-0.606	10.671	3.983e-4	7.467e-4	36	47	36.0000	53.1974	AAL94126.1| Transcription antitermination protein nusG
					23	38	29.4685	46.4487	
FN2047	-2.902	16.351	6.366e-8	2.144e-9	91	685	91.0000	775.3234	AAL94131.1| Fusobacterium outer membrane protein family
					94	659	120.4365	805.5191	
FN2048	0.155	15.816	2.243e-2	1.027e-1	234	198	234.0000	224.1081	AAL94132.1| Outer membrane protein
					213	189	272.9040	231.0214	
FN2049	-0.338	16.247	1.241e-3	3.212e-3	231	270	231.0000	305.6019	AAL94133.1| unknown
					207	263	265.2166	321.4742	
FN2050	-0.104	15.702	1.066e-1	6.041e-1	175	195	175.0000	220.7125	AAL94134.1| Hypothetical membrane-spanning protein
					211	211	270.3415	257.9128	
FN2051	0.063	14.226	5.06e-2	2.621e-1	142	113	142.0000	127.9001	AAL94135.1| unknown
					110	117	140.9363	143.0133	
FN2052	-0.813	14.879	2.894e-5	1.594e-5	121	210	121.0000	237.6904	AAL94136.1| unknown
					110	182	140.9363	222.4651	
FN2053	0.822	12.787	1.662e-3	4.812e-3	98	61	98.0000	69.0434	AAL94137.1| Serine/threonine sodium symporter
					98	47	125.5615	57.4498	
FN2054	-0.004	15.531	1.536e-1	9.185e-1	227	195	227.0000	220.7125	AAL94138.1| Glucose-6-phosphate isomerase
					162	176	207.5608	215.1310	
FN2058	-0.596	16.919	8.122e-5	8.007e-5	269	374	269.0000	423.3152	AAL94142.1| Fusobacterium outer membrane protein family
					237	362	303.6537	442.4855	
FN2059	0.155	15.816	2.243e-2	1.027e-1	234	198	234.0000	224.1081	AAL94143.1| Outer membrane protein
					213	189	272.9040	231.0214	
FN2060	-0.338	16.247	1.241e-3	3.212e-3	231	270	231.0000	305.6019	AAL94144.1| unknown
					207	263	265.2166	321.4742	
FN2061	-0.104	15.702	1.066e-1	6.041e-1	175	195	175.0000	220.7125	AAL94145.1| Hypothetical membrane-spanning protein
					211	211	270.3415	257.9128	
FN2062	0.063	14.226	5.06e-2	2.621e-1	142	113	142.0000	127.9001	AAL94146.1| unknown
					110	117	140.9363	143.0133	

Log$_2$ Ratios: -6 -4 -2 0 2 4 6

Show detected proteins only
Show all proteins
Filter by category:
GO: amino acid transport

Proteins found: 1297

Enter (or paste) list of ORFs

Find ORFs

Test	Cutoff
q-Value	.005
p-Value	

	Signif	Direction	Applies To
red	yes	+	ratios, bars
yellow	no	n/a	bars
green	yes	−	ratios, bars
light red	yes	+	*p*-, *q*-Values
light green	yes	−	

Dot Plots Dot Plots

Protein	*FnPgSg* vs *Fn*				Raw		Normalized		Description
	Log_2 Ratio	Log_2 Sum	*q*-Value	*p*-Value	*FnPgSg*	*Fn*	*FnPgSg*	*Fn*	
FN2063	-0.813	14.879	2.894e-5	1.594e-5	121	210	121.0000	237.6904	AAL94147.1| unknown
					110	182	140.9363	222.4651	
FN2067	0.925	6.547	1.012e-2	4.178e-2	10	7	10.0000	7.9230	AAL94151.1| Thiol:disulfide interchange protein tlpA
					13	5	16.6561	6.1117	
FN2068	-0.690	4.690			4	6	4.0000	6.7912	AAL94152.1| dGTP triphosphohydrolase
						5		6.1117	
FN2069									AAL94153.1| Amino acid carrier protein alsT
						4		4.8893	
FN2070	-2.188	6.188			4	16	4.0000	18.1097	AAL94154.1| Cobyric acid synthase
						15		18.3350	
FN2073	-0.859	7.618	1.318e-3	3.504e-3	8	15	8.0000	16.9779	AAL94157.1| Adenine phosphoribosyltransferase
					10	17	12.8124	20.7797	
FN2074									AAL94158.1| BslIM
					4		5.1250		
FN2075	-0.932	10.677	8.5e-4	1.941e-3	24	48	24.0000	54.3292	AAL94159.1| Hypothetical protein
					27	47	34.5935	57.4498	
FN2076									AAL94160.1| MunI regulatory protein
					3		3.8437		
FN2078						4		4.5274	AAL94162.1| Transcriptional regulator, DeoR family
FN2081						20		22.6372	AAL94165.1| ABC transporter substrate-binding protein
						17		20.7797	
FN2082	0.006	20.575	1.619e-1	9.778e-1	1557	1045	1557.0000	1182.7926	AAL94166.1| Formate--tetrahydrofolate ligase
					740	1073	948.1172	1311.5660	
FN2093	-0.781	10.254	4.272e-3	1.511e-2	20	41	20.0000	46.4062	AAL94177.1| General secretion pathway protein G
					26	37	33.3122	45.2264	
FN2098					3		3.0000		AAL94182.1| MRP-family nucleotide-binding protein
FN2100	-0.577	11.371	6.915e-6	1.863e-6	42	56	42.0000	63.3841	AAL94184.1| Hypothetical protein
					33	51	42.2809	62.3391	
FN2102	-1.320	7.629	1.419e-3	3.898e-3	5	22	5.0000	24.9009	AAL94186.1| ABC transporter ATP-binding protein
					10	16	12.8124	19.5574	

Log_2 Ratios: -6 -4 -2 0 2 4 6

Show detected proteins only
Show all proteins
Filter by category:
GO: amino acid transport

Proteins found: 1297

Enter (or paste) list of ORFs

Find ORFs

Test	Cutoff
q-Value	.005
p-Value	

	Signif	Direction	Applies To
red	yes	+	ratios, bars
yellow	no	n/a	bars
green	yes	−	ratios, bars
pink	yes	+	*p*-, *q*-Values
light green	yes	−	

Dot Plots Dot Plots

Protein	FnPgSg vs Fn Log$_2$ Ratio	Log$_2$ Sum	q-Value	p-Value	Raw FnPgSg	Raw Fn	Normalized FnPgSg	Normalized Fn	Description
FN2103	-0.354	19.448	2.946e-4	4.93e-4	788	873	788.0000	988.1129	AAL94187.1| tricarboxylate-binding protein
					553	756	708.5254	924.0856	
FN2105	0.010	7.103	1.571e-1	9.434e-1	12	12	12.0000	13.5823	AAL94189.1| tricarboxylate transport membrane protein RctA
					9	8	11.5312	9.7787	
FN2106	-0.334	14.087	9.885e-4	2.356e-3	112	131	112.0000	148.2735	AAL94190.1| Transporter
					96	121	122.9990	147.9026	
FN2107	-0.627	12.192	4.277e-3	1.513e-2	55	66	55.0000	74.7027	AAL94191.1| Galactokinase
					43	78	55.0933	95.3422	
FN2108	-0.390	10.391	1.343e-2	5.75e-2	32	32	32.0000	36.2195	AAL94192.1| Galactose-1-phosphate uridylyltransferase
					25	39	32.0310	47.6711	
FN2109	0.186	11.674	2.964e-3	9.74e-3	63	45	63.0000	50.9337	AAL94193.1| UDP-glucose 4-epimerase
					46	46	58.9370	56.2274	
FN2116	-3.826	8.850	9.781e-5	1.053e-4	5	76	5.0000	86.0213	AAL94200.1| Hypothetical exported 24-amino acid repeat protein
					5	62	6.4062	75.7848	
FN2117						23		26.0328	AAL94201.1| Hypothetical exported 24-amino acid repeat protein
						12		14.6680	
FN2118	-1.537	9.837	1.799e-4	2.482e-4	15	50	15.0000	56.5929	AAL94202.1| Hypothetical exported 24-amino acid repeat protein
					16	38	20.4998	46.4487	
FN2119	-0.938	9.237	1.852e-4	2.59e-4	15	32	15.0000	36.2195	AAL94203.1| Hypothetical exported 24-amino acid repeat protein
					16	26	20.4998	31.7807	
FN2120						24		27.1646	AAL94204.1| Hypothetical exported 24-amino acid repeat protein
						30		36.6701	
FN2121	-1.244	14.071	1.016e-5	3.242e-6	91	171	91.0000	193.5479	AAL94205.1| Hypothetical exported 24-amino acid repeat protein
					62	172	79.4368	210.2417	
FN2122	-0.165	16.415	1.034e-2	4.28e-2	297	280	297.0000	316.9205	AAL94206.1| Phenylalanyl-tRNA synthetase beta chain
					204	253	261.3728	309.2509	
FN2123	-0.650	12.469	1.363e-3	3.681e-3	60	77	60.0000	87.1531	AAL94207.1| Phenylalanyl-tRNA synthetase alpha chain
					47	83	60.2183	101.4538	
FN2125	-1.679	13.325	1.06e-5	3.434e-6	53	154	53.0000	174.3063	AAL94209.1| DNA gyrase subunit A
					47	154	60.2183	188.2397	
FN2126	-0.407	13.107	3.654e-4	6.646e-4	85	95	85.0000	107.5266	AAL94210.1| DNA gyrase subunit B
					61	89	78.1556	108.7879	

Log$_2$ Ratios: -6 -4 -2 0 2 4 6

Show detected proteins only
Show all proteins
Filter by category:
GO: amino acid transport

Proteins found: 1297

Enter (or paste) list of ORFs

Find ORFs

Test	Cutoff
q-Value	.005
p-Value	

	Signif	Direction	Applies To
red	yes	+	ratios, bars
yellow	no	n/a	bars
green	yes	–	ratios, bars
pink	yes	+	p-, q-Values
light green	yes	–	p-, q-Values

Dot Plots Dot Plots

Protein	*FnPgSg* vs *Fn*				Raw		Normalized		Description	Log_2 Ratios
	Log_2 Ratio	Log_2 Sum	*q*-Value	*p*-Value	*FnPgSg*	*Fn*	*FnPgSg*	*Fn*		-6 -4 -2 0 2 4 6
FN2128						3		3.3956	AAL94212.1| RECF protein	

Show detected proteins only
Show all proteins
Filter by category:
GO: amino acid transport

Proteins found: 1297

Enter (or paste) list of ORFs

Find ORFs

Test	Cutoff
q-Value	.005
p-Value	

	Signif	Direction	Applies To
	yes	+	ratios, bars
	no	n/a	bars
	yes	–	ratios, bars
	yes	+	*p*- , *q*-Values
	yes	–	

Dot Plots Dot Plots
