# Supplementary material for: Proteomics of Fusobacterium nucleatum within a model developing oral microbial community
Source: Microbiologyopen. 2014 Aug 25;3(5):729–51. doi: 10.1002/mbo3.204 (PMC4234264; doi:10.1002/mbo3.204)
Supplement: Table S4 — See description for Table S3. [file mbo30003-0729-sd6.pdf]

Fusobacterium nucleatum

Protein	FnPg vs Fn Log$_2$ Ratio	Log$_2$ Sum	*q*-Value	*p*-Value	Raw *FnPg*	Raw *Fn*	Normalized *FnPg*	Normalized *Fn*	Description
FN0001	-0.861	8.211	5.373e-2	9.891e-2	15	22	20.5521	26.7235	AAL94214.1| Chromosomal replication initiator protein dnaA
					5	15	5.0000	19.6771	
FN0004	-0.611	11.841				66		80.1706	AAL94217.1| Inner membrane protein
					49	53	49.0000	69.5257	
FN0005	-2.353	9.381	1.156e-3	5.965e-4	5	41	6.8507	49.8030	AAL94218.1| Jag protein
					16	51	16.0000	66.9021	
FN0006	-0.118	8.902				17		20.6500	AAL94219.1| Thiophene and furan oxidation protein THDF
					21	19	21.0000	24.9243	
FN0007	-1.490	8.166	7.004e-4	2.603e-4	6	23	8.2208	27.9382	AAL94220.1| Glucose inhibited division protein A
					12	22	12.0000	28.8597	
FN0008									AAL94221.1| Quinolinate synthetase A
					13		13.0000		
FN0009									AAL94222.1| L-aspartate oxidase
					29		29.0000		
FN0017						6		7.2882	AAL94230.1| Hypothetical protein
						7		9.1826	
FN0018	-1.322	10.369				45		54.6618	AAL94231.1| Hypothetical protein
					23	46	23.0000	60.3430	
FN0019									AAL94232.1| Transcription-repair coupling factor
						3		3.9354	
FN0021									AAL94234.1| 4-diphosphocytidyl-2-C-methyl-D-erythritol kinase
						5		6.5590	
FN0022	0.356	12.744	2.373e-1	6.936e-1	128	59	175.3779	71.6677	AAL94235.1| Hypothetical protein
					12	57	12.0000	74.7729	
FN0023									AAL94236.1| Short-chain fatty acids transporter
					11		11.0000		
FN0024	-2.410	10.338	2.211e-4	4.451e-5	6	75	8.2208	91.1030	AAL94237.1| Hypothetical exported 24-amino acid repeat protein
					23	57	23.0000	74.7729	
FN0025	-2.614	8.229				36		43.7294	AAL94238.1| Hypothetical exported 24-amino acid repeat protein
					7	32	7.0000	41.9778	
FN0026						9		10.9324	AAL94239.1| Hypothetical exported 24-amino acid repeat protein
						21		27.5479	

Log$_2$ Ratios

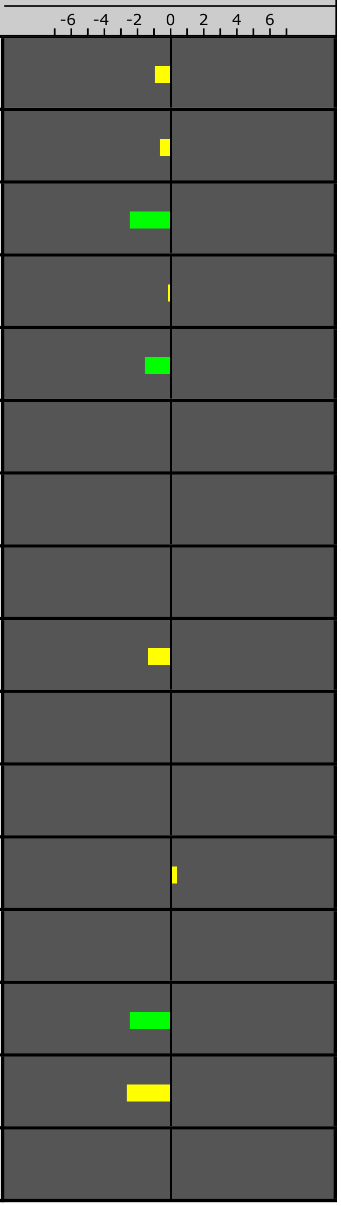

Show detected proteins only
Show all proteins
Filter by category:
GO: amino acid transport

Proteins found: 1338

Enter (or paste) list of ORFs

Find ORFs

Test	Cutoff
q-Value	.005
p-Value	

	Signif	Direction	Applies To
(red)	yes	+	ratios, bars
(yellow)	no	n/a	bars
(green)	yes	−	ratios, bars
(pink)	yes	+	*p*- , *q*-Values
(light green)	yes	−	

Dot Plots Dot Plots

Protein	FnPg vs Fn: Log_2 Ratio	Log_2 Sum	*q*-Value	*p*-Value	Raw *FnPg*	Raw *Fn*	Normalized *FnPg*	Normalized *Fn*	Description
FN0029						7		8.5029	AAL94242.1| Flavodoxin
						9		11.8062	
FN0030	0.941	12.742	2.833e-2	4.197e-2	63	53	86.3188	64.3794	AAL94243.1| 5-nitroimidazole antibiotic resistance protein
					143	42	143.0000	55.0958	
FN0031	1.926	7.752	6.48e-3	6.475e-3	25	7	34.2535	8.5029	AAL94244.1| unknown
					23	5	23.0000	6.5590	
FN0033	-0.537	12.649	5.583e-2	1.044e-1	65	79	89.0591	95.9618	AAL94246.1| unknown
					44	74	44.0000	97.0736	
FN0034	0.033	9.683	2.871e-1	8.979e-1	27	24	36.9938	29.1529	AAL94247.1| unknown
					21	21	21.0000	27.5479	
FN0038					77		105.5008		AAL94251.1| unknown
FN0039	0.032	6.887				8		9.7176	AAL94252.1| DNA primase (bacterial type) and small primase-like proteins
					11	9	11.0000	11.8062	
FN0040	0.040	18.005	2.345e-1	6.828e-1	341	402	467.2177	488.3119	AAL94253.1| Asparaginyl-tRNA synthetase
					573	399	573.0000	523.4103	
FN0041	-0.530	6.145				8		9.7176	AAL94254.1| unknown
					7	8	7.0000	10.4944	
FN0043	-0.908	6.908				15		18.2206	AAL94256.1| Hypothetical exported 24-amino acid repeat protein
					8	9	8.0000	11.8062	
FN0045						8		9.7176	AAL94258.1| Shikimate 5-dehydrogenase
						8		10.4944	
FN0046	0.453	5.099			5	5	6.8507	6.0735	AAL94259.1| 3-dehydroquinate dehydratase
						3		3.9354	
FN0047	-0.754	12.407	6.949e-3	7.143e-3	50	82	68.5070	99.6059	AAL94260.1| Exodeoxyribonuclease III
					45	70	45.0000	91.8264	
FN0048	-1.806	9.090	4.751e-4	1.424e-4	8	33	10.9611	40.0853	AAL94261.1| 4-nitrophenylphosphatase
					14	36	14.0000	47.2250	
FN0049	2.461	6.414			9		12.3313		AAL94262.1| Hypothetical protein
					31	3	31.0000	3.9354	
FN0050	-2.087	19.021	5.604e-5	5.869e-6	194	1157	265.8071	1405.4151	AAL94263.1| Fumarate reductase flavoprotein subunit
					442	1221	442.0000	1601.7142	

Log_2 Ratios: -6 -4 -2 0 2 4 6

Show detected proteins only
Show all proteins
Filter by category:
GO: amino acid transport

Proteins found: 1338

Enter (or paste) list of ORFs

Find ORFs

Test	Cutoff
q-Value	.005
p-Value	

	Signif	Direction	Applies To
(red)	yes	+	ratios, bars
(yellow)	no	n/a	bars
(green)	yes	−	ratios, bars
(pink)	yes	+	*p*- , *q*-Values
(light green)	yes	−	

Dot Plots Dot Plots

FnPg vs Fn

Fusobacterium nucleatum

Hackett Laboratory UW

Fn Summary Table | FnPg vs Fn | FnSg vs Fn | FnPgSg vs Fn | FnPgSg vs FnPg | FnSg vs FnPg | FnPgSg vs FnSg | Fn Coverage

Protein	FnPg vs Fn: Log_2 Ratio	Log_2 Sum	*q*-Value	*p*-Value	Raw *FnPg*	Raw *Fn*	Normalized *FnPg*	Normalized *Fn*	Description
**FN0052**	-1.633	9.711			12	44	16.4417	53.4471	AAL94265.1| Arsenate reductase
						37		48.5368	
**FN0054**	-0.656	14.433	4.46e-2	7.663e-2	54	153	73.9876	185.8501	AAL94267.1| Tyrosyl-tRNA synthetase
					163	143	163.0000	187.5882	
**FN0058**	-0.140	15.152	1.373e-1	3.386e-1	153	171	209.6314	207.7148	AAL94271.1| Cysteine desulfhydrase
					154	147	154.0000	192.8354	
**FN0059**	-1.187	10.292	1.566e-3	9.088e-4	16	48	21.9222	58.3059	AAL94272.1| NifU protein
					25	37	25.0000	48.5368	
**FN0060**	-1.856	8.478	1.087e-3	5.368e-4	5	30	6.8507	36.4412	AAL94273.1| D-alanyl-D-alanine carboxypeptidase
					13	27	13.0000	35.4187	
**FN0061**	0.092	12.784	2.844e-1	8.859e-1	20	67	27.4028	81.3853	AAL94274.1| Thermostable carboxypeptidase 1
					146	62	146.0000	81.3319	
**FN0062**	-0.934	6.104				7		8.5029	AAL94275.1| Hypothetical cytosolic protein
					6	11	6.0000	14.4299	
**FN0065**	-1.267	13.695	4.057e-4	1.097e-4	50	157	68.5070	190.7089	AAL94278.1| Transcription accessory protein (S1 RNA binding domain)
					80	127	80.0000	166.5993	
**FN0067**	-0.988	17.171	2.064e-3	1.353e-3	234	434	320.6127	527.1825	AAL94280.1| Isoleucyl-tRNA synthetase
					225	423	225.0000	554.8936	
**FN0069**	0.373	15.301	6.215e-2	1.221e-1	198	159	271.2877	193.1383	AAL94282.1| Glycyl-tRNA synthetase alpha chain
					186	122	186.0000	160.0402	
**FN0070**	-0.301	17.538	4.828e-2	8.539e-2	241	390	330.2037	473.7354	AAL94283.1| Glycyl-tRNA synthetase beta chain
					456	377	456.0000	494.5506	
**FN0071**	-0.392	6.392							AAL94284.1| GTP cyclohydrolase I
					8	8	8.0000	10.4944	
**FN0072**	0.159	12.943	2.316e-1	6.721e-1	42	67	57.5459	81.3853	AAL94285.1| 2-amino-4-hydroxy-6-hydroxymethyldihydropteridine pyrophosphokinase
					130	66	130.0000	86.5791	
**FN0073**	-0.702	10.009	6.226e-2	1.224e-1	9	35	12.3313	42.5147	AAL94286.1| Dihydropteroate synthase
					38	30	38.0000	39.3542	
**FN0074**	-0.272	8.272				21		25.5088	AAL94287.1| Ethanolamine utilization protein eutS
					16	10	16.0000	13.1181	
**FN0076**									AAL94289.1| Ethanolamine two-component response regulator
					4		4.0000		

Log_2 Ratios: -6 -4 -2 0 2 4 6

- Show detected proteins only
- Show all proteins
- Filter by category: GO: amino acid transport

Proteins found: 1338

Enter (or paste) list of ORFs

Find ORFs

Test	Cutoff
***q*-Value**	.005
p-Value	

	Signif	Direction	Applies To
(red)	yes	+	ratios, bars
(yellow)	no	n/a	bars
(green)	yes	–	ratios, bars
(pink)	yes	+	*p*- , *q*-Values
(light green)	yes	–	

Dot Plots Dot Plots

FnPg vs Fn

Fusobacterium nucleatum

Hackett Laboratory UW

Fn Summary Table | FnPg vs Fn | FnSg vs Fn | FnPgSg vs Fn | FnPgSg vs FnPg | FnSg vs FnPg | FnPgSg vs FnSg | Fn Coverage

Protein	*FnPg vs Fn* Log$_2$ Ratio	Log$_2$ Sum	*q*-Value	*p*-Value	Raw *FnPg*	Raw *Fn*	Normalized *FnPg*	Normalized *Fn*	Description
**FN0077**	-1.392	4.561							AAL94290.1| Ethanolamine two-component sensor kinase
					3	6	3.0000	7.8708	
**FN0078**	1.882	7.835			19		26.0327		AAL94291.1| Ethanolamine utilization protein eutA
					32	6	32.0000	7.8708	
**FN0079**	0.842	14.037	1.25e-1	3.005e-1	49	86	67.1369	104.4647	AAL94292.1| Ethanolamine ammonia-lyase heavy chain
					280	68	280.0000	89.2028	
**FN0080**	0.902	14.623	4.69e-4	1.392e-4	163	106	223.3328	128.7589	AAL94293.1| Ethanolamine ammonia-lyase light chain
					211	79	211.0000	103.6326	
**FN0081**	1.042	14.363	7.517e-2	1.565e-1	80	78	109.6112	94.7471	AAL94294.1| Ethanolamine utilization protein eutL
					307	82	307.0000	107.5680	
**FN0082**	1.056	8.758							AAL94295.1| Ethanolamine utilization protein eutM
					30	11	30.0000	14.4299	
**FN0083**	1.151	15.460	6.376e-2	1.263e-1	126	132	172.6376	160.3412	AAL94296.1| Ethanolamine utilization protein eutM precursor
					460	95	460.0000	124.6215	
**FN0084**	0.119	10.872	4.019e-2	6.639e-2	33	36	45.2146	43.7294	AAL94297.1| Acetaldehyde dehydrogenase (acetylating)
					45	30	45.0000	39.3542	
**FN0086**					7		9.5910		AAL94299.1| Hypothetical protein
**FN0087**					13		17.8118		AAL94300.1| Ethanolamine utilization protein eutN
**FN0088**									AAL94301.1| Hypothetical protein
						4		5.2472	
**FN0090**					9		12.3313		AAL94303.1| Ethanolamine utilization protein eutQ
					6		6.0000		
**FN0091**					6		8.2208		AAL94304.1| Phosphoserine phosphatase
**FN0092**	0.492	10.680	1.89e-1	5.192e-1	11	26	15.0715	31.5824	AAL94305.1| NADPH-dependent butanol dehydrogenase
					81	28	81.0000	36.7305	
**FN0093**	-0.842	16.090	4.414e-3	3.817e-3	153	323	209.6314	392.3501	AAL94306.1| Thioredoxin
					185	240	185.0000	314.8333	
**FN0100**	-1.317	9.040	1.103e-2	1.274e-2	11	24	15.0715	29.1529	AAL94309.1| Flavodoxins/hemoproteins
					14	33	14.0000	43.2896	

Log$_2$ Ratios: -6 -4 -2 0 2 4 6

Show detected proteins only
Show all proteins
Filter by category:
GO: amino acid transport

Proteins found: 1338

Enter (or paste) list of ORFs

Find ORFs

Test	Cutoff
***q*-Value**	.005
p-Value	

	Signif	Direction	Applies To
red	yes	+	ratios, bars
yellow	no	n/a	bars
green	yes	–	ratios, bars
pink	yes	+	*p*- , *q*-Values
light green	yes	–	

Dot Plots Dot Plots

Protein	FnPg vs Fn: Log_2 Ratio	Log_2 Sum	*q*-Value	*p*-Value	Raw *FnPg*	Raw *Fn*	Normalized *FnPg*	Normalized *Fn*	Description
FN0102						12		14.5765	AAL94311.1| Ribonucleoside-diphosphate reductase alpha chain
						11		14.4299	
FN0103						7		8.5029	AAL94312.1| Ribonucleoside-diphosphate reductase beta chain
						8		10.4944	
FN0106	0.481	13.751	4.514e-3	3.942e-3	93	87	127.4230	105.6794	AAL94315.1| Hypothetical protein
					150	71	150.0000	93.1382	
FN0108	1.611	7.947	4.839e-3	4.367e-3	16	4	21.9222	4.8588	AAL94317.1| Microcin C7 self-immunity protein mccF
					33	10	33.0000	13.1181	
FN0110	0.226	17.666	1.41e-1	3.503e-1	442	352	605.6018	427.5766	AAL94319.1| Seryl-tRNA synthetase
					381	317	381.0000	415.8423	
FN0111									AAL94320.1| unknown
					5		5.0000		
FN0113	0.647	11.693	1.201e-2	1.411e-2	46	39	63.0264	47.3735	AAL94322.1| Heat-inducible transcription repressor hrcA
					81	34	81.0000	44.6014	
FN0114	-0.198	13.784	6.152e-3	6.025e-3	83	109	113.7216	132.4030	AAL94323.1| GrpE protein
					108	93	108.0000	121.9979	
FN0116	0.278	20.274	1.647e-1	4.297e-1	604	797	827.5645	968.1209	AAL94325.1| Chaperone protein dnaK
					1653	821	1653.0000	1076.9921	
FN0117	1.094	6.521							AAL94326.1| O6-methylguanine-DNA methyltransferase
					14	5	14.0000	6.5590	
FN0118	-0.984	12.145	2.653e-3	1.89e-3	29	77	39.7341	93.5324	AAL94327.1| Chaperone protein dnaJ
					56	73	56.0000	95.7618	
FN0119	1.972	9.137				10		12.1471	AAL94328.1| Flavodoxin
					47	9	47.0000	11.8062	
FN0123	-0.480	5.124				5		6.0735	AAL94332.1| ATPase
					5	6	5.0000	7.8708	
FN0127					4		5.4806		AAL94333.1| Fe-S oxidoreductase
FN0128	-0.982	9.077	3.37e-3	2.639e-3	11	30	15.0715	36.4412	AAL94334.1| Spermidine/putrescine-binding protein
					18	22	18.0000	28.8597	
FN0129						3		3.6441	AAL94335.1| Urease accessory protein ureG

Log_2 Ratios: -6 -4 -2 0 2 4 6

Show detected proteins only
Show all proteins
Filter by category:
GO: amino acid transport

Proteins found: 1338

Enter (or paste) list of ORFs

Find ORFs

Test	Cutoff
q-Value	.005
p-Value	

Colour	Signif	Direction	Applies To
red	yes	+	ratios, bars
yellow	no	n/a	bars
green	yes	−	ratios, bars
pink	yes	+	*p*- , *q*-Values
light green	yes	−	

Dot Plots Dot Plots

Protein	FnPg vs Fn Log$_2$ Ratio	FnPg vs Fn Log$_2$ Sum	FnPg vs Fn *q*-Value	FnPg vs Fn *p*-Value	Raw *FnPg*	Raw *Fn*	Normalized *FnPg*	Normalized *Fn*	Description	Log$_2$ Ratios (-6 -4 -2 0 2 4 6)
FN0130	-1.187	7.187				15		18.2206	AAL94336.1| ABC transporter ATP-binding protein	
					8		8.0000			
FN0132	-0.378	5.575			3		4.1104		AAL93916.1| Hemolysin	
					8	6	8.0000	7.8708		
FN0147	-0.966	10.162	1.213e-2	1.427e-2	12	39	16.4417	47.3735	AAL94353.1| PLSX protein	
					32	36	32.0000	47.2250		
FN0148	-0.177	16.026	5.895e-2	1.129e-1	159	234	217.8522	284.2413	AAL94354.1| 3-oxoacyl-[acyl-carrier-protein] synthase III	
					268	202	268.0000	264.9847		
FN0149	-0.210	15.914	1.751e-1	4.672e-1	219	252	300.0606	306.1060	AAL94355.1| Malonyl-CoA-[acyl-carrier-protein] transacylase	
					162	174	162.0000	228.2541		
FN0150	-0.746	11.656	8.561e-4	3.661e-4	29	65	39.7341	78.9559	AAL94356.1| Acyl carrier protein	
					48	52	48.0000	68.2139		
FN0151	-0.793	19.262	5.296e-2	9.695e-2	205	825	280.8787	1002.1326	AAL94357.1| 3-oxoacyl-[acyl-carrier-protein] synthase	
					924	827	924.0000	1084.8630		
FN0152	0.663	5.337				4		4.8588	AAL94358.1| Ribonuclease III	
					8	4	8.0000	5.2472		
FN0154						7		8.5029	AAL94360.1| Ribonuclease G	
FN0155						9		10.9324	AAL94361.1| Hypothetical protein	
FN0156	-0.497	7.347	1.051e-1	2.418e-1	4	12	5.4806	14.5765	AAL94362.1| Phosphopantetheine adenylyltransferase	
					16	12	16.0000	15.7417		
FN0157						7		8.5029	AAL94363.1| DNA repair protein RadA	
						6		7.8708		
FN0158	-0.530	9.206	3.161e-2	4.837e-2	12	20	16.4417	24.2941	AAL94364.1| DNA-binding protein	
					24	26	24.0000	34.1069		
FN0164	-0.769	15.858	1.169e-3	6.076e-4	155	243	212.3717	295.1736	AAL94370.1| Anhydro-N-acetylmuramyl-tripeptide amidase	
					161	260	161.0000	341.0694		
FN0170	0.112	13.730	2.35e-1	6.85e-1	63	95	86.3188	115.3971	AAL94376.1| GTP-binding protein	
					156	83	156.0000	108.8798		
FN0173	-0.778	10.805	8.21e-2	1.76e-1	7	48	9.5910	58.3059	AAL94379.1| Hypothetical protein	
					55	40	55.0000	52.4722		

Show detected proteins only
Show all proteins
Filter by category:
GO: amino acid transport

Proteins found: 1338

Enter (or paste) list of ORFs

Find ORFs

Test	Cutoff
q-Value	.005
p-Value	

	Signif	Direction	Applies To
red	yes	+	ratios, bars
yellow	no	n/a	bars
green	yes	−	ratios, bars
pink	yes	+	*p*-, *q*-Values
light green	yes	−	

Dot Plots Dot Plots

Protein	FnPg vs Fn: Log_2 Ratio	Log_2 Sum	*q*-Value	*p*-Value	Raw *FnPg*	Raw *Fn*	Normalized *FnPg*	Normalized *Fn*	Description
FN0174	-0.124	12.708	7.411e-2	1.536e-1	56	65	76.7278	78.9559	AAL94380.1| Enoyl-[acyl-carrier-protein] reductase
					80	70	80.0000	91.8264	
FN0175	-0.146	8.119	1.763e-1	4.719e-1	10	17	13.7014	20.6500	AAL94381.1| Cell division inhibitor MinC
					18	11	18.0000	14.4299	
FN0176	0.146	15.160	7.733e-2	1.624e-1	134	158	183.5987	191.9236	AAL94382.1| Cell division inhibitor MinD
					219	131	219.0000	171.8465	
FN0177	-1.762	5.841			3	10	4.1104	12.1471	AAL94383.1| Cell division inhibitor MinE
						12		15.7417	
FN0178	-1.229	11.977	5.813e-4	1.931e-4	35	78	47.9549	94.7471	AAL94384.1| UNC-44 ankyrins
					35	76	35.0000	99.6972	
FN0179	1.260	11.281	9.709e-2	2.186e-1	93	25	127.4230	30.3677	AAL94385.1| Ankyrin repeat proteins
					27	26	27.0000	34.1069	
FN0181	0.337	9.382	1.942e-1	5.371e-1	11	13	15.0715	15.7912	AAL94387.1| Hypothetical protein
					43	23	43.0000	30.1715	
FN0182	1.741	13.880	9.767e-2	2.203e-1	35	49	47.9549	59.5206	AAL94388.1| Sarcosine oxidase alpha subunit
					401	57	401.0000	74.7729	
FN0183	0.669	14.512	1.728e-1	4.589e-1	34	97	46.5848	117.8265	AAL94389.1| Glycerol-3-phosphate dehydrogenase
					339	95	339.0000	124.6215	
FN0185	-0.190	8.139	2.186e-1	6.238e-1	12	9	16.4417	10.9324	AAL94391.1| Hypothetical protein
					15	19	15.0000	24.9243	
FN0188						13		15.7912	AAL94394.1| Peptide methionine sulfoxide reductase
FN0189									AAL94395.1| Two-component response regulator yesN
					20		20.0000		
FN0190	-0.711	5.687			6		8.2208		AAL94396.1| Two-component sensor kinase yesM
					3	7	3.0000	9.1826	
FN0191	0.135	9.220	2.069e-1	5.817e-1	14	20	19.1820	24.2941	AAL94397.1| helix-turn-helix DNA-binding protein
					32	17	32.0000	22.3007	
FN0192	-2.863	10.863				88		106.8941	AAL94398.1| Dipeptide-binding protein
					16	96	16.0000	125.9333	
FN0197									AAL94403.1| Methyltransferase
						4		5.2472	

Show detected proteins only
Show all proteins
Filter by category:
GO: amino acid transport

Proteins found: 1338

Enter (or paste) list of ORFs

Find ORFs

Test	Cutoff
q-Value	.005
p-Value	

	Signif	Direction	Applies To
(red)	yes	+	ratios, bars
(yellow)	no	n/a	bars
(green)	yes	–	ratios, bars
(pink)	yes	+	*p*- , *q*-Values
(light green)	yes	–	

Dot Plots Dot Plots

Protein	FnPg vs Fn				Raw		Normalized		Description
	Log_2 Ratio	Log_2 Sum	*q*-Value	*p*-Value	*FnPg*	*Fn*	*FnPg*	*Fn*	
FN0198						6		7.2882	AAL94404.1| Transcriptional regulatory protein
						4		5.2472	
FN0199	0.589	11.112	1.742e-3	1.054e-3	39	34	53.4355	41.3000	AAL94405.1| Hypothetical protein
					62	27	62.0000	35.4187	
FN0200	-1.336	20.587	1.877e-2	2.497e-2	942	1665	1290.6717	2022.4859	AAL94406.1| Biotin carboxyl carrier protein of glutaconyl-COA decarboxylase
					289	1499	289.0000	1966.3961	
FN0202	-0.590	19.813	2.697e-3	1.932e-3	585	1032	801.5318	1253.5768	AAL94408.1| Glutaconate CoA-transferase subunit A
					763	840	763.0000	1101.9164	
FN0203	0.084	21.144	2.282e-1	6.59e-1	1361	1114	1864.7603	1353.1827	AAL94409.1| Glutaconate CoA-transferase subunit B
					1270	1223	1270.0000	1604.3379	
FN0204	-0.493	21.327	1.772e-2	2.309e-2	833	1600	1141.3265	1943.5299	AAL94410.1| Glutaconyl-CoA decarboxylase A subunit
					1593	1452	1593.0000	1904.7413	
FN0206	-0.444	12.567	1.01e-1	2.3e-1	69	74	94.5396	89.8883	AAL94412.1| Activator of (R)-2-hydroxyglutaryl-CoA dehydratase
					39	70	39.0000	91.8264	
FN0207	-0.327	19.040	1.127e-1	2.635e-1	632	681	865.9284	827.2149	AAL94413.1| (R)-2-hydroxyglutaryl-CoA dehydratase alpha-subunit
					445	623	445.0000	817.2547	
FN0208	-0.455	17.834	2.923e-3	2.161e-3	280	461	383.6392	559.9796	AAL94414.1| (R)-2-hydroxyglutaryl-CoA dehydratase beta-subunit
					442	436	442.0000	571.9471	
FN0209	-0.386	17.986	7.596e-4	2.975e-4	339	486	464.4774	590.3472	AAL94415.1| Hypothetical cytosolic protein
					427	438	427.0000	574.5707	
FN0212	-0.009	9.149	3.045e-1	9.76e-1	23	21	31.5132	25.5088	AAL94418.1| Hypothetical protein
					16	17	16.0000	22.3007	
FN0214					3		4.1104		AAL94420.1| Crossover junction endodeoxyribonuclease ruvC
FN0218	0.516	14.609	6.593e-2	1.321e-1	173	114	237.0342	138.4765	AAL94424.1| Anthranilate synthase component II
					141	96	141.0000	125.9333	
FN0219	0.304	4.866				4		4.8588	AAL94425.1| Autolysin response regulator
					6		6.0000		
FN0221	0.220	11.462	3.049e-2	4.613e-2	45	40	61.6563	48.5882	AAL94427.1| Carbon starvation protein A
					53	38	53.0000	49.8486	
FN0224	-0.910	8.992	1.518e-2	1.886e-2	16	25	21.9222	30.3677	AAL94430.1| Excinuclease ABC subunit B
					11	24	11.0000	31.4833	

Log_2 Ratios: -6 -4 -2 0 2 4 6

Show detected proteins only
Show all proteins
Filter by category:
GO: amino acid transport

Proteins found: 1338

Enter (or paste) list of ORFs

Find ORFs

Test	Cutoff
q-Value	.005
p-Value	

	Signif	Direction	Applies To
red	yes	+	ratios, bars
yellow	no	n/a	bars
green	yes	−	ratios, bars
pink	yes	+	*p*- , *q*-Values
light green	yes	−	

Dot Plots Dot Plots

Protein	FnPg vs Fn: Log_2 Ratio	Log_2 Sum	*q*-Value	*p*-Value	Raw *FnPg*	Raw *Fn*	Normalized *FnPg*	Normalized *Fn*	Description
FN0225									AAL94431.1| Gluconate permease
					4		4.0000		
FN0226	-0.258	12.152	4.659e-3	4.129e-3	47	61	64.3966	74.0971	AAL94432.1| Pyridoxal phosphate biosynthetic protein pdxA
					59	56	59.0000	73.4611	
FN0227									AAL94433.1| Hypothetical protein
					4		4.0000		
FN0233	-0.178	12.478	1.701e-1	4.491e-1	65	61	89.0591	74.0971	AAL94439.1| Hypothetical protein
					53	66	53.0000	86.5791	
FN0234	-0.582	7.501				12		14.5765	AAL94440.1| unknown
					11	14	11.0000	18.3653	
FN0235					16		21.9222		AAL94441.1| ABC transporter ATP-binding protein
FN0236	0.731	16.378	1.67e-1	4.38e-1	486	172	665.8880	208.9295	AAL94442.1| ABC transporter substrate-binding protein
					86	186	86.0000	243.9958	
FN0237					3		4.1104		AAL94443.1| ABC transporter permease protein
FN0238	-0.136	10.982	2.095e-1	5.91e-1	40	42	54.8056	51.0177	AAL94444.1| Hypothetical protein
					31	33	31.0000	43.2896	
FN0240	-0.078	13.135	1.549e-1	3.959e-1	64	87	87.6890	105.6794	AAL94446.1| Thymidylate synthase
					97	68	97.0000	89.2028	
FN0241	0.165	9.300	2.215e-1	6.345e-1	14	25	19.1820	30.3677	AAL94447.1| Dihydrofolate reductase
					34	13	34.0000	17.0535	
FN0242	-0.011	12.011				50		60.7353	AAL94448.1| Trk system potassium uptake protein trkA
					64	52	64.0000	68.2139	
FN0243	0.210	9.190				10		12.1471	AAL94449.1| Poly(A) polymerase
					26	25	26.0000	32.7951	
FN0244	-1.191	7.835				16		19.4353	AAL94450.1| COP associated protein
					10	20	10.0000	26.2361	
FN0245	-0.922	6.922				12		14.5765	AAL94451.1| Copper-exporting ATPase
					8	12	8.0000	15.7417	
FN0247	-1.109	14.471	1.669e-2	2.132e-2	41	183	56.1757	222.2912	AAL94453.1| Hypothetical cytosolic protein
					149	168	149.0000	220.3833	

Log_2 Ratios: -6 -4 -2 0 2 4 6

Show detected proteins only
Show all proteins
Filter by category:
GO: amino acid transport

Proteins found: 1338

Enter (or paste) list of ORFs

Find ORFs

Test	Cutoff
q-Value	.005
p-Value	

	Signif	Direction	Applies To
red	yes	+	ratios, bars
yellow	no	n/a	bars
green	yes	−	ratios, bars
pink	yes	+	*p*-, *q*-Values
light green	yes	−	

Dot Plots Dot Plots

Protein	FnPg vs Fn Log$_2$ Ratio	Log$_2$ Sum	*q*-Value	*p*-Value	Raw *FnPg*	Raw *Fn*	Normalized *FnPg*	Normalized *Fn*	Description
FN0248									AAL94454.1| Hypothetical Exported Protein
					42		42.0000		
FN0249	-1.626	14.269	5.543e-3	5.222e-3	89	210	121.9424	255.0883	AAL94455.1| unknown
					38	182	38.0000	238.7486	
FN0250	-0.589	13.778	8.361e-2	1.803e-1	106	113	145.2348	137.2618	AAL94456.1| unknown
					48	117	48.0000	153.4812	
FN0251	-0.361	15.648	1.844e-1	5.025e-1	240	195	328.8336	236.8677	AAL94457.1| Hypothetical membrane-spanning Protein
					71	211	71.0000	276.7909	
FN0252	1.100	17.889	6.173e-2	1.209e-1	753	270	1031.7153	327.9707	AAL94458.1| unknown
					411	263	411.0000	345.0048	
FN0253	-0.332	15.532				198		240.5118	AAL94459.1| Outer membrane protein
					194	189	194.0000	247.9312	
FN0254	-0.864	19.214	3.416e-3	2.688e-3	352	851	482.2892	1033.7150	AAL94460.1| Fusobacterium outer membrane protein family
					674	816	674.0000	1070.4331	
FN0258	-1.074	6.733	3.329e-2	5.181e-2	6	16	8.2208	19.4353	AAL94464.1| Zinc-transporting ATPase
					6	8	6.0000	10.4944	
FN0260									AAL94466.1| Transcriptional repressor smtB
						5		6.5590	
FN0261	-1.252	9.331			12	31	16.4417	37.6559	AAL94467.1| Pyruvate formate-lyase activating enzyme
						31		40.6660	
FN0262	-1.824	23.015	4.137e-3	3.483e-3	1745	4406	2390.8940	5351.9956	AAL94468.1| Formate acetyltransferase
					704	4274	704.0000	5606.6557	
FN0263	-0.370	16.995	1.843e-1	5.019e-1	386	358	528.8740	434.8648	AAL94469.1| Peptidyl-prolyl cis-trans isomerase
					107	295	107.0000	386.9826	
FN0264	-0.911	16.196	1.619e-2	2.05e-2	96	314	131.5334	381.4178	AAL94470.1| Hypothetical protein
					268	282	268.0000	369.9291	
FN0265	-1.308	8.227				20		24.2941	AAL94471.1| Cell division protein ftsX
					11	23	11.0000	30.1715	
FN0266	-1.145	7.145				14		17.0059	AAL94472.1| membrane protein related to metalloendopeptidase
					8	14	8.0000	18.3653	
FN0267	-0.759	5.929				7		8.5029	AAL94473.1| ATP-NAD kinase
					6	9	6.0000	11.8062	

Log$_2$ Ratios: -6 -4 -2 0 2 4 6

Show detected proteins only
Show all proteins
Filter by category:
GO: amino acid transport

Proteins found: 1338

Enter (or paste) list of ORFs

Find ORFs

Test	Cutoff
q-Value	.005
p-Value	

	Signif	Direction	Applies To
red	yes	+	ratios, bars
yellow	no	n/a	bars
green	yes	−	ratios, bars
pink	yes	+	*p*- , *q*-Values
light green	yes	−	

Dot Plots Dot Plots

Protein	FnPg vs Fn Log_2 Ratio	Log_2 Sum	*q*-Value	*p*-Value	Raw *FnPg*	Raw *Fn*	Normalized *FnPg*	Normalized *Fn*	Description
FN0268	-0.137	7.964			11	10	15.0715	12.1471	AAL94474.1| DNA repair protein recN
						16		20.9889	
FN0270						8		9.7176	AAL94476.1| GTP-binding protein era
						9		11.8062	
FN0271	0.783	6.832				8		9.7176	AAL94477.1| Enoyl-CoA hydratase
					14	5	14.0000	6.5590	
FN0272	-0.713	4.713							AAL94478.1| Acetoacetate: butyrate/acetate coenzyme A transferase
					4	5	4.0000	6.5590	
FN0273									AAL94479.1| Butyrate-acetoacetate CoA-transferase subunit B
					16		16.0000		
FN0276	1.319	9.021							AAL94482.1| Sodium-dependent phosphate transporter
					36	11	36.0000	14.4299	
FN0277	-0.304	7.393	7.804e-2	1.644e-1	9	14	12.3313	17.0059	AAL94483.1| Hypothetical protein
					11	9	11.0000	11.8062	
FN0278	-0.096	16.203	9.868e-2	2.233e-1	209	230	286.3592	279.3824	AAL94484.1| Xaa-His dipeptidase
					245	220	245.0000	288.5972	
FN0279	0.946	13.128	1.364e-1	3.36e-1	26	55	35.6236	66.8088	AAL94485.1| Lipoprotein
					227	53	227.0000	69.5257	
FN0280	-0.987	13.362	4.062e-2	6.736e-2	21	119	28.7729	144.5500	AAL94486.1| Hypothetical protein
					117	110	117.0000	144.2986	
FN0281	-0.491	11.913	9.66e-2	2.172e-1	21	64	28.7729	77.7412	AAL94487.1| DNA polymerase III alpha subunit
					76	53	76.0000	69.5257	
FN0282	-0.017	10.968	2.942e-1	9.293e-1	27	32	36.9938	38.8706	AAL94488.1| Hypothetical protein
					52	39	52.0000	51.1604	
FN0283	-1.392	8.035							AAL94489.1| tRNA (Guanine-N1) -methyltransferase
					10	20	10.0000	26.2361	
FN0284	1.792	9.609				15		18.2206	AAL94490.1| 16S rRNA processing protein rimM
					52	9	52.0000	11.8062	
FN0285	2.474	7.035				4		4.8588	AAL94491.1| RNA binding protein
					27		27.0000		
FN0287	0.047	7.354				11		13.3618	AAL94493.1| Dimethyladenosine transferase
					13	9	13.0000	11.8062	

Log_2 Ratios: -6 -4 -2 0 2 4 6

Show detected proteins only
Show all proteins
Filter by category:
GO: amino acid transport

Proteins found: 1338

Enter (or paste) list of ORFs

Find ORFs

Test	Cutoff
q-Value	.005
p-Value	

	Signif	Direction	Applies To
red	yes	+	ratios, bars
yellow	no	n/a	bars
green	yes	−	ratios, bars
pink	yes	+	*p*-, *q*-Values
light green	yes	−	

Dot Plots Dot Plots

Protein	FnPg vs Fn: Log_2 Ratio	Log_2 Sum	*q*-Value	*p*-Value	Raw *FnPg*	Raw *Fn*	Normalized *FnPg*	Normalized *Fn*	Description
FN0288	0.806	10.873	8.985e-2	1.977e-1	23	28	31.5132	34.0118	AAL94494.1| Hypoxanthine-guanine phosphoribosyltransferase
					83	24	83.0000	31.4833	
FN0291	1.345	5.298							AAL94497.1| Hemolysin
					10	3	10.0000	3.9354	
FN0294	-0.806	15.296	8.268e-3	9.034e-3	136	212	186.3390	257.5177	AAL94500.1| Transketolase subunit A
					117	208	117.0000	272.8555	
FN0295	0.025	14.942	2.939e-1	9.281e-1	170	146	232.9238	177.3471	AAL94501.1| Transketolase
					125	133	125.0000	174.4701	
FN0296	0.286	11.332	1.34e-1	3.283e-1	30	39	41.1042	47.3735	AAL94502.1| Hypothetical cytosolic protein
					71	34	71.0000	44.6014	
FN0297	0.274	7.325	2.215e-1	6.344e-1	5	6	6.8507	7.2882	AAL94503.1| ATPase associated with chromosome architecture/replication
					21	12	21.0000	15.7417	
FN0298	0.398	15.651	1.757e-1	4.697e-1	94	173	128.7931	210.1442	AAL94504.1| Histidyl-tRNA synthetase
					392	141	392.0000	184.9645	
FN0299	-0.529	15.954	6.556e-2	1.311e-1	96	239	131.5334	290.3148	AAL94505.1| Aspartyl-tRNA synthetase
					288	240	288.0000	314.8333	
FN0305									AAL94511.1| Iron(III) dicitrate-binding protein
						3		3.9354	
FN0308	-0.517	16.825	1.128e-3	5.716e-4	191	347	261.6967	421.5031	AAL94514.1| Iron(III)-binding protein
					308	300	308.0000	393.5416	
FN0309									AAL94515.1| Iron(III)-transport system permease protein sfuB
						8		10.4944	
FN0310	-0.888	13.719	1.399e-2	1.701e-2	83	123	113.7216	149.4089	AAL94516.1| Iron(III)-transport ATP-binding protein sfuC
					57	127	57.0000	166.5993	
FN0311	-0.932	13.015	1.996e-2	2.706e-2	29	100	39.7341	121.4706	AAL94517.1| Anaerobic ribonucleoside-triphosphate reductase
					92	99	92.0000	129.8687	
FN0313	-0.943	8.558				13		15.7912	AAL94519.1| 16S rRNA m(5)C 967 methyltransferase
					14	29	14.0000	38.0424	
FN0314									AAL94520.1| Caffeoyl-CoA O-methyltransferase
					8		8.0000		
FN0315									AAL94521.1| Transcriptional regulator, AraC family
					10		10.0000		

Log₂ Ratios: -6 -4 -2 0 2 4 6

◉ Show detected proteins only
○ Show all proteins
☐ Filter by category:
GO: amino acid transport

Proteins found: 1338

Enter (or paste) list of ORFs

Find ORFs

Test	Cutoff
q-Value	.005
p-Value	

	Signif	Direction	Applies To
red	yes	+	ratios, bars
yellow	no	n/a	bars
green	yes	−	ratios, bars
light red	yes	+	*p*- , *q*-Values
light green	yes	−	*p*- , *q*-Values

Dot Plots Dot Plots

Protein	FnPg vs Fn Log_2 Ratio	Log_2 Sum	*q*-Value	*p*-Value	Raw *FnPg*	Raw *Fn*	Normalized *FnPg*	Normalized *Fn*	Description
FN0316	-0.584	9.667			17	24	23.2924	29.1529	AAL94522.1| Hypothetical protein
						31		40.6660	
FN0317	0.441	8.024	1.622e-1	4.21e-1	7	12	9.5910	14.5765	AAL94523.1| Tryptophan synthase beta chain
					28	10	28.0000	13.1181	
FN0319	-0.418	6.033				10		12.1471	AAL94525.1| Citrate (pro-3S)-lyase ligase
					7	5	7.0000	6.5590	
FN0320	-0.089	9.430	1.812e-1	4.901e-1	16	23	21.9222	27.9382	AAL94526.1| Hypothetical cytosolic protein
					29	20	29.0000	26.2361	
FN0321	0.268	13.895	1.544e-1	3.944e-1	70	101	95.9098	122.6853	AAL94527.1| Heat shock protein htpG
					175	78	175.0000	102.3208	
FN0322	0.176	21.395	1.898e-1	5.221e-1	934	1315	1279.7106	1597.3387	AAL94528.1| Fructose-bisphosphate aldolase
					2250	1164	2250.0000	1526.9413	
FN0325	-4.103	8.182			3	58	4.1104	70.4530	AAL94529.1| LSU ribosomal protein L20P
						54		70.8375	
FN0326						7		8.5029	AAL94530.1| LSU ribosomal protein L35P
FN0327	-2.002	11.049				75		91.1030	AAL94531.1| Bacterial Protein Translation Initiation Factor 3 (IF-3)
					23	71	23.0000	93.1382	
FN0329	-0.359	18.255	9.469e-2	2.116e-1	472	528	646.7060	641.3649	AAL94533.1| LSU ribosomal protein L13P
					341	477	341.0000	625.7311	
FN0330	-1.152	12.311	2.453e-3	1.701e-3	45	81	61.6563	98.3912	AAL94534.1| SSU ribosomal protein S9P
					34	87	34.0000	114.1271	
FN0331	-1.077	11.208	3.941e-2	6.465e-2	8	58	10.9611	70.4530	AAL94535.1| Hypothetical protein
					56	54	56.0000	70.8375	
FN0332									AAL94536.1| Magnesium and cobalt transport protein corA
					7		7.0000		
FN0333									AAL94537.1| Glycerol uptake operon antiterminator regulatory protein
					8		8.0000		
FN0334	-0.173	14.224	1.787e-1	4.807e-1	69	123	94.5396	149.4089	AAL94538.1| Aspartate/aromatic aminotransferase
					166	110	166.0000	144.2986	
FN0335	-0.593	20.284	5.665e-2	1.066e-1	932	1093	1276.9703	1327.6739	AAL94539.1| Outer membrane porin F
					563	1103	563.0000	1446.9212	

Log_2 Ratios: -6 -4 -2 0 2 4 6

Show detected proteins only
Show all proteins
Filter by category:
GO: amino acid transport

Proteins found: 1338

Enter (or paste) list of ORFs

Find ORFs

Test	Cutoff
q-Value	.005
p-Value	

	Signif	Direction	Applies To
red	yes	+	ratios, bars
yellow	no	n/a	bars
green	yes	−	ratios, bars
pink	yes	+	*p*- , *q*-Values
light green	yes	−	

Dot Plots Dot Plots

Protein	FnPg vs Fn Log_2 Ratio	Log_2 Sum	*q*-Value	*p*-Value	Raw *FnPg*	Raw *Fn*	Normalized *FnPg*	Normalized *Fn*	Description
FN0336	-1.726	11.635				76		92.3177	AAL94540.1| Hypothetical protein
					31	86	31.0000	112.8153	
FN0341	-0.776	11.420				62		75.3118	AAL94545.1| transport protein
					40	47	40.0000	61.6548	
FN0342	-0.221	10.720	2.038e-1	5.707e-1	38	46	52.0653	55.8765	AAL94546.1| Peptidyl-prolyl cis-trans isomerase
					24	25	24.0000	32.7951	
FN0343						4		4.8588	AAL94547.1| Hypothetical protein
FN0344									AAL94548.1| Methyltransferase
						9		11.8062	
FN0347	-1.193	12.183	1.878e-2	2.499e-2	14	78	19.1820	94.7471	AAL94551.1| Phosphatidylserine decarboxylase
					71	85	71.0000	111.5034	
FN0348	-0.459	16.128	5.352e-2	9.837e-2	120	279	164.4168	338.9030	AAL94552.1| Nicotinate phosphoribosyltransferase
					292	220	292.0000	288.5972	
FN0349	1.092	7.766	3.465e-2	5.461e-2	11	8	15.0715	9.7176	AAL94553.1| D-Tyr-tRNATyr deacylase
					28	8	28.0000	10.4944	
FN0351	-0.512	14.137	1.493e-1	3.774e-1	140	117	191.8196	142.1206	AAL94555.1| unknown
					33	136	33.0000	178.4055	
FN0352	0.044	11.266	2.911e-1	9.157e-1	21	47	28.7729	57.0912	AAL94556.1| NA+/H+ antiporter NHAC
					72	31	72.0000	40.6660	
FN0355	-0.213	15.127	1.534e-1	3.911e-1	93	170	127.4230	206.5001	AAL94558.1| S-adenosylmethionine synthetase
					224	153	224.0000	200.7062	
FN0356	-0.716	10.040	4.226e-2	7.109e-2	26	35	35.6236	42.5147	AAL94559.1| Lactoylglutathione lyase
					15	31	15.0000	40.6660	
FN0357	-0.326	7.596	1.831e-1	4.972e-1	5	17	6.8507	20.6500	AAL94560.1| ATP synthase epsilon chain, sodium ion specific
					18	8	18.0000	10.4944	
FN0358	-0.929	17.355	1.835e-7	2.97e-9	212	470	290.4696	570.9119	AAL94561.1| ATP synthase beta chain, sodium ion specific
					303	426	303.0000	558.8290	
FN0359	-0.634	11.962	2.378e-2	3.367e-2	47	68	64.3966	82.6000	AAL94562.1| ATP synthase gamma chain, sodium ion specific
					37	57	37.0000	74.7729	
FN0360	0.202	13.804	2.111e-3	1.401e-3	96	95	131.5334	115.3971	AAL94563.1| ATP synthase alpha chain, sodium ion specific
					125	82	125.0000	107.5680	

Log_2 Ratios: -6 -4 -2 0 2 4 6

Show detected proteins only
Show all proteins
Filter by category:
GO: amino acid transport

Proteins found: 1338

Enter (or paste) list of ORFs

Find ORFs

Test	Cutoff
q-Value	.005
p-Value	

Color	Signif	Direction	Applies To
red	yes	+	ratios, bars
yellow	no	n/a	bars
green	yes	−	ratios, bars
pink	yes	+	*p*- , *q*-Values
light green	yes	−	

Dot Plots Dot Plots

Protein	*FnPg vs Fn* Log$_2$ Ratio	Log$_2$ Sum	*q*-Value	*p*-Value	Raw *FnPg*	Raw *Fn*	Normalized *FnPg*	Normalized *Fn*	Description
**FN0361**	-0.808	10.031	2.108e-2	2.895e-2	24	37	32.8834	44.9441	AAL94564.1| ATP synthase delta chain, sodium ion specific
					16	31	16.0000	40.6660	
**FN0362**	-0.554	10.462				49		59.5206	AAL94565.1| ATP synthase B chain, sodium ion specific
					31	24	31.0000	31.4833	
**FN0364**	1.724	5.677							AAL94567.1| ATP synthase A chain, sodium ion specific
					13	3	13.0000	3.9354	
**FN0366**	-0.510	15.695	5.096e-3	4.671e-3	127	228	174.0078	276.9530	AAL94569.1| Phosphoglucosamine mutase
					212	208	212.0000	272.8555	
**FN0368**	0.324	13.237	1.183e-1	2.803e-1	59	69	80.8383	83.8147	AAL94571.1| Adenylosuccinate lyase
					139	70	139.0000	91.8264	
**FN0370**	-0.675	10.059	6.105e-2	1.189e-1	10	28	13.7014	34.0118	AAL94573.1| Signal peptidase I
					38	37	38.0000	48.5368	
**FN0371**	-1.635	11.082	9.831e-3	1.113e-2	32	66	43.8445	80.1706	AAL94574.1| Hypothetical protein
					9	64	9.0000	83.9555	
**FN0374**	1.120	5.425	2.593e-2	3.754e-2	9	3	12.3313	3.6441	AAL94577.1| Single-stranded-DNA-specific exonuclease recJ
					7	4	7.0000	5.2472	
**FN0375**	0.509	17.587	7.141e-3	7.429e-3	353	297	483.6594	360.7677	AAL94578.1| Iron(III)-binding protein
					575	292	575.0000	383.0471	
**FN0376**	0.466	12.667	1.783e-2	2.329e-2	61	59	83.5785	71.6677	AAL94579.1| Iron(III)-transport ATP-binding protein sfuC
					106	50	106.0000	65.5903	
**FN0377**									AAL94580.1| Iron(III)-transport system permease protein sfuB
						9		11.8062	
**FN0378**						7		8.5029	AAL94581.1| UDP-glucose 4-epimerase
						7		9.1826	
**FN0379**	-0.651	6.644	4.499e-2	7.757e-2	8	12	10.9611	14.5765	AAL94582.1| Hypothetical protein
					5	8	5.0000	10.4944	
**FN0380**	-2.167	7.337				26		31.5824	AAL94583.1| unknown
					6	17	6.0000	22.3007	
**FN0381**						15		18.2206	AAL94584.1| unknown
						15		19.6771	
**FN0382**						8		9.7176	AAL94585.1| Hypothetical protein
						8		10.4944	

Log$_2$ Ratios: -6 -4 -2 0 2 4 6

Show detected proteins only
Show all proteins
Filter by category:
GO: amino acid transport

Proteins found: 1338

Enter (or paste) list of ORFs

Find ORFs

Test	Cutoff
q-Value	.005
p-Value	

	Signif	Direction	Applies To
red	yes	+	ratios, bars
yellow	no	n/a	bars
green	yes	−	ratios, bars
pink	yes	+	*p*- , *q*-Values
light green	yes	−	

Dot Plots | Dot Plots

Protein	FnPg vs Fn: Log_2 Ratio	FnPg vs Fn: Log_2 Sum	FnPg vs Fn: *q*-Value	FnPg vs Fn: *p*-Value	Raw: *FnPg*	Raw: *Fn*	Normalized: *FnPg*	Normalized: *Fn*	Description
FN0383						7		8.5029	AAL94586.1| Lipopolysaccharide N-acetylglucosaminyltransferase
						10		13.1181	
FN0384	-3.389	7.467			3	32	4.1104	38.8706	AAL94587.1| Hypothetical protein
						36		47.2250	
FN0385									AAL94588.1| Hypothetical protein
					5		5.0000		
FN0386						12		14.5765	AAL94589.1| Hypothetical protein
						11		14.4299	
FN0387	-2.777	11.772	7.82e-5	9.377e-6	14	121	19.1820	146.9795	AAL94590.1| Fusobacterium outer membrane protein family
					26	124	26.0000	162.6639	
FN0390	1.070	11.829	5.147e-2	9.318e-2	40	34	54.8056	41.3000	AAL94593.1| Hypothetical protein
					120	32	120.0000	41.9778	
FN0391	0.598	9.079	1.758e-1	4.7e-1	6	16	8.2208	19.4353	AAL94594.1| Hydrolase (HAD superfamily)
					49	14	49.0000	18.3653	
FN0392	-1.819	6.462				15		18.2206	AAL94595.1| Oxygen-independent coproporphyrinogen III oxidase
					5	13	5.0000	17.0535	
FN0393	0.932	7.413	1.252e-2	1.484e-2	11	8	15.0715	9.7176	AAL94596.1| Polysaccharide deacetylase
					21	7	21.0000	9.1826	
FN0394	0.531	7.684	1.818e-1	4.925e-1	4	11	5.4806	13.3618	AAL94597.1| Outer membrane protein
					29	8	29.0000	10.4944	
FN0396	0.402	24.004	5.688e-4	1.869e-4	3549	2994	4862.6263	3636.8304	AAL94599.1| Dipeptide-binding protein
					4566	2667	4566.0000	3498.5847	
FN0397	-0.052	10.905				41		49.8030	AAL94600.1| Dipeptide transport system permease protein dppB
					43	30	43.0000	39.3542	
FN0398	-1.058	11.387	8.884e-4	3.919e-4	29	56	39.7341	68.0235	AAL94601.1| Dipeptide transport system permease protein dppC
					32	62	32.0000	81.3319	
FN0399	-0.543	15.278	1.413e-2	1.723e-2	98	178	134.2737	216.2177	AAL94602.1| Dipeptide transport ATP-binding protein dppD
					196	202	196.0000	264.9847	
FN0400	-0.684	17.806	3.158e-3	2.414e-3	306	498	419.2628	604.9237	AAL94603.1| Dipeptide transport ATP-binding protein dppF
					336	464	336.0000	608.6776	
FN0405	-0.284	12.911	1.443e-1	3.61e-1	38	86	52.0653	104.4647	AAL94608.1| Tryptophanyl-tRNA synthetase
					107	68	107.0000	89.2028	

Log_2 Ratios: -6 -4 -2 0 2 4 6

Show detected proteins only
Show all proteins
Filter by category:
GO: amino acid transport

Proteins found: 1338

Enter (or paste) list of ORFs

Find ORFs

Test	Cutoff
q-Value	.005
p-Value	

Colour	Signif	Direction	Applies To
red	yes	+	ratios, bars
yellow	no	n/a	bars
green	yes	–	ratios, bars
light red	yes	+	*p*- , *q*-Values
light green	yes	–	*p*- , *q*-Values

Dot Plots Dot Plots

Protein	FnPg vs Fn				Raw		Normalized		Description
	Log_2 Ratio	Log_2 Sum	*q*-Value	*p*-Value	*FnPg*	*Fn*	*FnPg*	*Fn*	
**FN0406**	-1.164	11.391	1.616e-7	2.24e-9	25	64	34.2535	77.7412	AAL94609.1| Alanine racemase, biosynthetic
					35	59	35.0000	77.3965	
**FN0407**	-2.102	10.887				75		91.1030	AAL94610.1| Hypothetical protein
					21	68	21.0000	89.2028	
**FN0408**	-0.747	12.388	5.938e-2	1.142e-1	19	86	26.0327	104.4647	AAL94611.1| Acetyl-coenzyme A carboxylase carboxyl transferase subunit beta
					87	65	87.0000	85.2673	
**FN0409**	-1.080	17.074	1.773e-3	1.08e-3	219	453	300.0606	550.2619	AAL94612.1| Acetyl-coenzyme A carboxylase carboxyl transferase subunit alpha
					211	404	211.0000	529.9693	
**FN0410**	-0.698	13.649	2.14e-4	4.225e-5	62	120	84.9487	145.7647	AAL94613.1| 6-phosphofructokinase
					93	109	93.0000	142.9868	
**FN0411**									AAL94614.1| putative alpha helix protein
						8		10.4944	
**FN0412**									AAL94615.1| Recombination protein recR
						10		13.1181	
**FN0413**	0.938	4.669			8	3	10.9611	3.6441	AAL94616.1| unknown
					3		3.0000		
**FN0416**	-1.077	8.326			9	18	12.3313	21.8647	AAL94619.1| Type III restriction-modification system methylation subunit
						23		30.1715	
**FN0417**	-1.788	9.867			12	46	16.4417	55.8765	AAL94620.1| Type III restriction-modification system restriction subunit
						44		57.7194	
**FN0418**									AAL94621.1| Uracil phosphoribosyltransferase
					67		67.0000		
**FN0419**	0.272	7.477			9	10	12.3313	12.1471	AAL94622.1| Aspartate carbamoyltransferase
					17		17.0000		
**FN0420**	1.054	8.770	1.263e-1	3.048e-1	6	12	8.2208	14.5765	AAL94623.1| Dihydroorotase
					52	11	52.0000	14.4299	
**FN0421**	0.774	9.870				18		21.8647	AAL94624.1| Carbamoyl-phosphate synthase small chain
					40	19	40.0000	24.9243	
**FN0422**	-0.715	13.950	9.791e-2	2.211e-1	20	125	27.4028	151.8383	AAL94625.1| Carbamoyl-phosphate synthase large chain
					169	130	169.0000	170.5347	
**FN0423**	0.481	7.519				7		8.5029	AAL94626.1| Dihydroorotate dehydrogenase electron transfer subunit
					16	11	16.0000	14.4299	

Log_2 Ratios: -6 -4 -2 0 2 4 6

Show detected proteins only
Show all proteins
Filter by category:
GO: amino acid transport

Proteins found: 1338

Enter (or paste) list of ORFs

Find ORFs

Test	Cutoff
q-Value	.005
p-Value	

	Signif	Direction	Applies To
red	yes	+	ratios, bars
yellow	no	n/a	bars
green	yes	−	ratios, bars
pink	yes	+	*p*- , *q*-Values
light green	yes	−	

Dot Plots Dot Plots

Protein	FnPg vs Fn Log_2 Ratio	Log_2 Sum	*q*-Value	*p*-Value	Raw *FnPg*	Raw *Fn*	Normalized *FnPg*	Normalized *Fn*	Description
FN0424	0.993	5.667	1.049e-1	2.411e-1	3	4	4.1104	4.8588	AAL94627.1| Dihydroorotate dehydrogenase
					16	4	16.0000	5.2472	
FN0426	1.087	9.071	1.221e-1	2.916e-1	7	10	9.5910	12.1471	AAL94629.1| Orotidine 5'-phosphate decarboxylase
					58	15	58.0000	19.6771	
FN0427	-0.092	11.470	1.324e-1	3.233e-1	41	43	56.1757	52.2324	AAL94630.1| Orotate phosphoribosyltransferase
					47	44	47.0000	57.7194	
FN0430	-1.332	14.971	4.591e-3	4.041e-3	51	223	69.8771	270.8795	AAL94633.1| LSU ribosomal protein L19P
					156	227	156.0000	297.7798	
FN0435	0.230	11.661	2.416e-1	7.102e-1	17	39	23.2924	47.3735	AAL94634.1| Purine nucleoside phosphorylase
					100	44	100.0000	57.7194	
FN0436	1.228	11.009	3.199e-2	4.913e-2	35	24	47.9549	29.1529	AAL94635.1| regulator of kinase autophosphorylation inhibitor
					91	23	91.0000	30.1715	
FN0437	0.279	7.134	2.359e-1	6.883e-1	3	8	4.1104	9.7176	AAL94636.1| kinase autophosphorylation inhibitor KipI
					22	9	22.0000	11.8062	
FN0438									AAL94637.1| transporter protein
						9		11.8062	
FN0439	0.336	12.037	2.36e-1	6.889e-1	10	41	13.7014	49.8030	AAL94638.1| Lactam utilization protein LAMB
					132	50	132.0000	65.5903	
FN0445						5		6.0735	AAL94641.1| Hypothetical protein
						4		5.2472	
FN0446	-1.008	7.281	6.665e-5	7.553e-6	7	14	9.5910	17.0059	AAL94642.1| Hypothetical protein
					8	14	8.0000	18.3653	
FN0447	0.262	4.908				5		6.0735	AAL94643.1| NIFS protein
					6	3	6.0000	3.9354	
FN0450	-1.177	9.532	4.1e-2	6.82e-2	14	22	19.1820	26.7235	AAL94646.1| ABC transporter ATP-binding protein
					17	42	17.0000	55.0958	
FN0451	-0.516	6.131				10		12.1471	AAL94647.1| Hypothetical protein
					7	6	7.0000	7.8708	
FN0452	-0.429	18.476	1.721e-1	4.565e-1	647	592	886.4805	719.1061	AAL94648.1| Glucosamine--fructose-6-phosphate aminotransferase (isomerizing)
					154	520	154.0000	682.1387	
FN0453	0.787	16.502	9.092e-3	1.012e-2	255	195	349.3857	236.8677	AAL94649.1| Xaa-Pro aminopeptidase
					451	173	451.0000	226.9423	

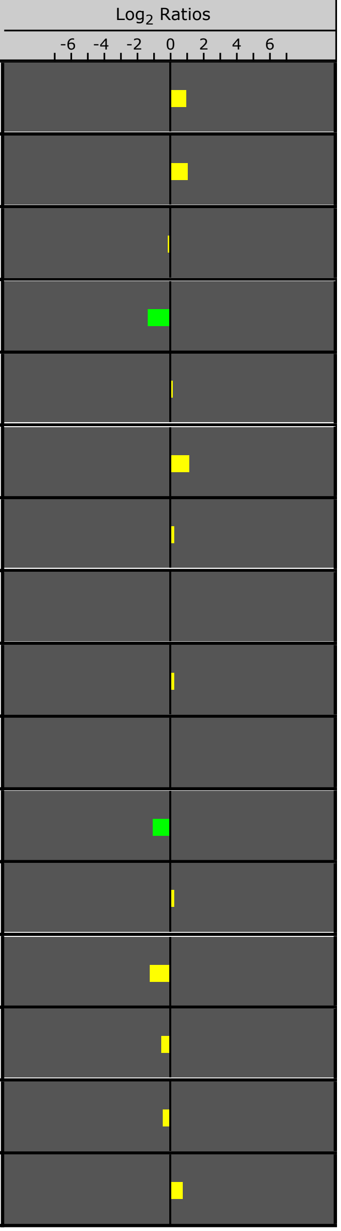

Show detected proteins only
Show all proteins
Filter by category:
GO: amino acid transport

Proteins found: 1338

Enter (or paste) list of ORFs
Find ORFs

Test	Cutoff
q-Value	.005
p-Value	

	Signif	Direction	Applies To
red	yes	+	ratios, bars
yellow	no	n/a	bars
green	yes	−	ratios, bars
pink	yes	+	*p*- , *q*-Values
light green	yes	−	

Dot Plots | Dot Plots

Protein	*FnPg vs Fn* Log_2 Ratio	Log_2 Sum	*q*-Value	*p*-Value	Raw *FnPg*	Raw *Fn*	Normalized *FnPg*	Normalized *Fn*	Description
FN0454	0.855	13.882	7.932e-2	1.68e-1	69	77	94.5396	93.5324	AAL94650.1| Aldehyde dehydrogenase B
					236	68	236.0000	89.2028	
FN0455	1.074	16.135	4.143e-3	3.49e-3	315	150	431.5941	182.2059	AAL94651.1| Rubrerythrin
					347	143	347.0000	187.5882	
FN0456	-2.394	10.091	2.686e-4	6.088e-5	13	62	17.8118	75.3118	AAL94652.1| Hypothetical cytosolic protein
					11	58	11.0000	76.0847	
FN0458	1.533	5.806	2.136e-2	2.941e-2	12	4	16.4417	4.8588	AAL94654.1| Hypothetical Exported Protein
					9	3	9.0000	3.9354	
FN0459						8		9.7176	AAL94655.1| Hypothetical exported 24-amino acid repeat protein
						8		10.4944	
FN0460	-0.196	11.810				57		69.2383	AAL94656.1| Delta-aminolevulinic acid dehydratase
					56	45	56.0000	59.0312	
FN0461	0.389	16.697	1.504e-1	3.809e-1	381	227	522.0233	275.7383	AAL94657.1| Probable sigma(54) modulation protein
					224	224	224.0000	293.8444	
FN0462	0.045	11.170	2.852e-1	8.896e-1	23	40	31.5132	48.5882	AAL94658.1| DNA mismatch repair protein mutL
					66	35	66.0000	45.9132	
FN0465	0.270	16.294	2.351e-1	6.853e-1	377	195	516.5427	236.8677	AAL94661.1| Hypothetical protein
					106	213	106.0000	279.4145	
FN0466	0.383	17.561	5.203e-2	9.458e-2	428	323	586.4199	392.3501	AAL94662.1| Lysyl-tRNA synthetase
					418	288	418.0000	377.7999	
FN0470	-0.034	18.282	2.706e-1	8.272e-1	343	430	469.9580	522.3237	AAL94666.1| Putative efflux pump component MtrF
					646	473	646.0000	620.4839	
FN0472	1.659	24.440	5.982e-3	5.795e-3	7225	2271	9899.2604	2758.5978	AAL94668.1| Flavodoxin
					7052	1990	7052.0000	2610.4925	
FN0474									AAL94670.1| Acriflavin resistance protein B
						6		7.8708	
FN0475	-1.548	10.467				53		64.3794	AAL94671.1| MIAB protein
					22	49	22.0000	64.2785	
FN0476	-0.244	11.690	1.564e-1	4.011e-1	26	49	35.6236	59.5206	AAL94672.1| Transcription termination factor rho
					70	50	70.0000	65.5903	
FN0477	-2.372	10.157	4.722e-6	2.484e-7	10	65	13.7014	78.9559	AAL94673.1| Cell wall endopeptidase family M23/M37
					16	57	16.0000	74.7729	

Log_2 Ratios: -6 -4 -2 0 2 4 6

Show detected proteins only
Show all proteins
Filter by category:
GO: amino acid transport

Proteins found: 1338

Enter (or paste) list of ORFs

Find ORFs

Test	Cutoff
q-Value	.005
p-Value	

	Signif	Direction	Applies To
red	yes	+	ratios, bars
yellow	no	n/a	bars
green	yes	−	ratios, bars
pink	yes	+	*p*- , *q*-Values
light green	yes	−	

Dot Plots Dot Plots

Protein	*FnPg vs Fn* Log$_2$ Ratio	Log$_2$ Sum	*q*-Value	*p*-Value	Raw *FnPg*	Raw *Fn*	Normalized *FnPg*	Normalized *Fn*	Description
FN0478	1.361	8.814				11		13.3618	AAL94674.1| GcpE protein
					34	10	34.0000	13.1181	
FN0481	-0.697	7.616				11		13.3618	AAL94677.1| unknown
					11	17	11.0000	22.3007	
FN0482	-2.778	7.948				44		53.4471	AAL94678.1| LSU ribosomal protein L31P
					6	22	6.0000	28.8597	
FN0483	-1.016	13.224	8.106e-3	8.797e-3	34	120	46.5848	145.7647	AAL94679.1| Uracil phosphoribosyltransferase
					91	101	91.0000	132.4923	
FN0484	-0.959	5.603				8		9.7176	AAL94680.1| Lipase
					5		5.0000		
FN0487	-0.121	20.283	2.279e-1	6.579e-1	548	933	750.8366	1133.3209	AAL94683.1| 2-hydroxyglutarate dehydrogenase
					1416	932	1416.0000	1222.6025	
FN0488	0.305	23.556	1.465e-1	3.683e-1	1959	2530	2684.1040	3073.2067	AAL94684.1| NAD-specific glutamate dehydrogenase
					5124	2474	5124.0000	3245.4062	
FN0489	-1.186	8.105				25		30.3677	AAL94685.1| Prolipoprotein diacylglyceryl transferase
					11	15	11.0000	19.6771	
FN0490									AAL94686.1| Integral membrane protein
						4		5.2472	
FN0491	1.570	13.188	5.768e-2	1.094e-1	59	61	80.8383	74.0971	AAL94687.1| Alanine racemase
					252	29	252.0000	38.0424	
FN0493	-0.264	8.762	1.677e-1	4.405e-1	19	16	26.0327	19.4353	AAL94689.1| Hypothetical protein
					12	20	12.0000	26.2361	
FN0494	-1.417	17.059	1.5e-4	2.529e-5	136	464	186.3390	563.6237	AAL94690.1| Short chain dehydrogenase
					266	491	266.0000	644.0964	
FN0495	1.116	25.792	5.159e-5	5.034e-6	8224	4420	11268.0301	5369.0015	AAL94691.1| Acetyl-CoA acetyltransferase
					11175	3801	11175.000[illegible]	4986.1718	
FN0496									AAL94692.1| unknown
						6		7.8708	
FN0501	0.635	16.730	9.468e-2	2.115e-1	183	199	250.7356	241.7265	AAL94697.1| Ornithine decarboxylase
					571	219	571.0000	287.2854	
FN0502	1.032	7.887				8		9.7176	AAL94698.1| Phosphoheptose isomerase
					22	9	22.0000	11.8062	

Log$_2$ Ratios: -6 -4 -2 0 2 4 6

Show detected proteins only
Show all proteins
Filter by category:
GO: amino acid transport

Proteins found: 1338

Enter (or paste) list of ORFs

Find ORFs

Test	Cutoff
q-Value	.005
p-Value	

	Signif	Direction	Applies To
red	yes	+	ratios, bars
yellow	no	n/a	bars
green	yes	–	ratios, bars
pink	yes	+	*p*- , *q*-Values
light green	yes	–	

Dot Plots Dot Plots

Protein	FnPg vs Fn Log_2 Ratio	Log_2 Sum	*q*-Value	*p*-Value	Raw *FnPg*	Raw *Fn*	Normalized *FnPg*	Normalized *Fn*	Description
FN0503	-0.592	11.037	4.431e-2	7.591e-2	18	43	24.6625	52.2324	AAL94699.1| Transcriptional regulatory protein, LYSR family
					50	46	50.0000	60.3430	
FN0505	0.947	11.277	7.569e-2	1.579e-1	28	31	38.3639	37.6559	AAL94701.1| Anthranilate synthase component II
					100	26	100.0000	34.1069	
FN0506	-0.638	14.994	2.51e-2	3.606e-2	134	182	183.5987	221.0765	AAL94702.1| Arginyl-tRNA synthetase
					106	175	106.0000	229.5659	
FN0511	0.404	11.177	2.162e-1	6.149e-1	10	30	13.7014	36.4412	AAL94707.1| D-lactate dehydrogenase
					97	36	97.0000	47.2250	
FN0512	0.714	15.779	4.243e-4	1.178e-4	223	145	305.5412	176.1324	AAL94708.1| Flavoprotein
					302	148	302.0000	194.1472	
FN0513	-1.712	8.768	1.029e-3	4.909e-4	11	32	15.0715	38.8706	AAL94709.1| Flavodoxin
					8	28	8.0000	36.7305	
FN0515	-0.928	4.098				4		4.8588	AAL94711.1| Acriflavin resistance protein D
					3	5	3.0000	6.5590	
FN0516									AAL94712.1| Acriflavin resistance protein E
					4		4.0000		
FN0517									AAL94713.1| Outer membrane protein tolC
					5		5.0000		
FN0519	0.080	6.962	2.602e-1	7.842e-1	8	6	10.9611	7.2882	AAL94715.1| Hypothetical exported 24-amino acid repeat protein
					12	11	12.0000	14.4299	
FN0522	0.833	5.810				6		7.2882	AAL94718.1| Exonuclease SBCC
					10	3	10.0000	3.9354	
FN0523	-2.435	6.514			3	15	4.1104	18.2206	AAL94719.1| Exonuclease SBCD
						20		26.2361	
FN0524					5		6.8507		AAL94720.1| DNA helicase II
					9		9.0000		
FN0525	-1.036	14.898	3.154e-2	4.824e-2	38	196	52.0653	238.0824	AAL94721.1| Penicillin-binding protein
					192	200	192.0000	262.3611	
FN0526	0.310	11.406				36		43.7294	AAL94722.1| Florfenicol resistance protein
					58	38	58.0000	49.8486	
FN0527	0.950	9.787	6.384e-3	6.342e-3	26	19	35.6236	23.0794	AAL94723.1| Alanyl-tRNA synthetase
					47	15	47.0000	19.6771	

Log_2 Ratios: -6 -4 -2 0 2 4 6

Show detected proteins only
Show all proteins
Filter by category:
GO: amino acid transport

Proteins found: 1338

Enter (or paste) list of ORFs
Find ORFs

Test	Cutoff
q-Value	.005
p-Value	

	Signif	Direction	Applies To
red	yes	+	ratios, bars
yellow	no	n/a	bars
green	yes	−	ratios, bars
pink	yes	+	*p*- , *q*-Values
light green	yes	−	

Dot Plots Dot Plots

Protein	FnPg vs Fn				Raw		Normalized		Description
	Log_2 Ratio	Log_2 Sum	*q*-Value	*p*-Value	*FnPg*	*Fn*	*FnPg*	*Fn*	
FN0528	-2.764	18.819	1.531e-3	8.813e-4	340	1436	465.8475	1744.3181	AAL94724.1| Cold shock protein
					56	1373	56.0000	1801.1086	
FN0535	0.648	10.623	3.787e-3	3.085e-3	39	22	53.4355	26.7235	AAL94731.1| Hypothetical protein
					46	28	46.0000	36.7305	
FN0536	-0.352	15.236	7.724e-2	1.622e-1	94	184	128.7931	223.5059	AAL94732.1| DNA polymerase III, beta chain
					219	168	219.0000	220.3833	
FN0540	-0.944	11.020	4.112e-2	6.849e-2	10	49	13.7014	59.5206	AAL94736.1| Glutamate-1-semialdehyde 2,1-aminomutase
					52	51	52.0000	66.9021	
FN0541						6		7.2882	AAL94737.1| polysaccharide deacetylase
						15		19.6771	
FN0542	0.849	8.771	1.668e-1	4.372e-1	3	17	4.1104	20.6500	AAL94738.1| Beta 1,4 glucosyltransferase
					52	8	52.0000	10.4944	
FN0543	-1.094	10.321	2.493e-2	3.574e-2	8	45	10.9611	54.6618	AAL94739.1| Lipopolysaccharide heptosyltransferase-1
					38	38	38.0000	49.8486	
FN0544									AAL94740.1| ADP-heptose:LPS heptosyltransferase II
					3		3.0000		
FN0546	-0.281	5.451				6		7.2882	AAL94742.1| Lipopolysaccharide core biosynthesis protein rfaQ
					6		6.0000		
FN0547	-1.334	16.732	7.476e-3	7.902e-3	88	425	120.5723	516.2501	AAL94743.1| RecA protein
					295	405	295.0000	531.2811	
FN0549									AAL94745.1| O-sialoglycoprotein endopeptidase
						3		3.9354	
FN0550					34		46.5848		AAL94746.1| hypothetical Protein
					16		16.0000		
FN0552	0.538	8.751	4.343e-2	7.384e-2	19	10	26.0327	12.1471	AAL94748.1| Serine racemase
					24	17	24.0000	22.3007	
FN0553	0.711	13.762	9.265e-2	2.057e-1	64	75	87.6890	91.1030	AAL94749.1| D-serine dehydratase
					214	71	214.0000	93.1382	
FN0554	1.694	6.477			8		10.9611		AAL94750.1| D-serine permease
					23	4	23.0000	5.2472	
FN0556	-0.194	12.234	2.387e-1	6.989e-1	75	65	102.7605	78.9559	AAL94752.1| unknown
					27	53	27.0000	69.5257	

Log_2 Ratios: -6 -4 -2 0 2 4 6

Show detected proteins only
Show all proteins
Filter by category:
GO: amino acid transport

Proteins found: 1338

Enter (or paste) list of ORFs

Find ORFs

Test	Cutoff
q-Value	.005
p-Value	

	Signif	Direction	Applies To
red	yes	+	ratios, bars
yellow	no	n/a	bars
green	yes	−	ratios, bars
light red	yes	+	*p*-, *q*-Values
light green	yes	−	*p*-, *q*-Values

Dot Plots Dot Plots

Protein	*FnPg* vs *Fn*				Raw		Normalized		Description
	Log_2 Ratio	Log_2 Sum	*q*-Value	*p*-Value	*FnPg*	*Fn*	*FnPg*	*Fn*	
**FN0557**	1.582	12.845	4.794e-2	8.458e-2	59	46	80.8383	55.8765	AAL94753.1| unknown
					216	33	216.0000	43.2896	
**FN0558**	2.171	14.188				52		63.1647	AAL94754.1| TraT complement resistance protein precursor
					290	50	290.0000	65.5903	
**FN0559**	0.991	14.564	4.928e-2	8.779e-2	105	90	143.8647	109.3236	AAL94755.1| Phosphoglucomutase
					295	85	295.0000	111.5034	
**FN0561**	-0.125	11.120	1.507e-1	3.821e-1	28	39	38.3639	47.3735	AAL94757.1| Proline synthetase associated protein
					52	39	52.0000	51.1604	
**FN0562**	-0.332	13.764	6.792e-2	1.372e-1	60	98	82.2084	119.0412	AAL94758.1| Hypothetical cytosolic protein
					128	111	128.0000	145.6104	
**FN0563**	-1.631	7.755	9.49e-3	1.066e-2	10	21	13.7014	25.5088	AAL94759.1| putative tRNA (5-methylaminomethyl-2-thiouridylate) -methyltransferase
					3	20	3.0000	26.2361	
**FN0574**						3		3.6441	AAL94770.1| Hypothetical cytosolic protein
**FN0576**	-0.794	11.518	4.822e-3	4.344e-3	25	58	34.2535	70.4530	AAL94772.1| hypothetical protein
					48	55	48.0000	72.1493	
**FN0577**	1.068	5.851							AAL94773.1| Hypothetical protein
					11	4	11.0000	5.2472	
**FN0579**	1.502	18.883	2.34e-2	3.3e-2	1109	341	1519.4851	414.2148	AAL94775.1| Hypothetical cytosolic protein
					821	314	821.0000	411.9069	
**FN0580**	-2.833	6.833				21		25.5088	AAL94776.1| Penicillin-binding protein
					4	24	4.0000	31.4833	
**FN0581**	-1.229	7.229				19		23.0794	AAL94777.1| Lipoprotein releasing system transmembrane protein lolE
					8	11	8.0000	14.4299	
**FN0582**	-1.440	9.780				34		41.3000	AAL94778.1| Lipoprotein releasing system ATP-binding protein lolD
					18	43	18.0000	56.4076	
**FN0583**	1.304	7.480				7		8.5029	AAL94779.1| Hypothetical Exported Protein
					21		21.0000		
**FN0585**	0.893	7.198	1.115e-1	2.601e-1	19	6	26.0327	7.2882	AAL94781.1| Two-component response regulator czcR
					7	8	7.0000	10.4944	
**FN0586**	0.879	6.522				3		3.6441	AAL94782.1| Two-component sensor kinase czcS
					13	8	13.0000	10.4944	

Log_2 Ratios: -6 -4 -2 0 2 4 6

Show detected proteins only
Show all proteins
Filter by category:
GO: amino acid transport

Proteins found: 1338

Enter (or paste) list of ORFs

Find ORFs

Test	Cutoff
q-Value	.005
p-Value	

	Signif	Direction	Applies To
(red)	yes	+	ratios, bars
(yellow)	no	n/a	bars
(green)	yes	−	ratios, bars
(pink)	yes	+	*p*- , *q*-Values
(light green)	yes	−	

Dot Plots Dot Plots

Protein	FnPg vs Fn Log2 Ratio	FnPg vs Fn Log2 Sum	FnPg vs Fn *q*-Value	FnPg vs Fn *p*-Value	Raw FnPg	Raw Fn	Normalized FnPg	Normalized Fn	Description
FN0590	0.905	8.602	1.142e-1	2.679e-1	8	14	10.9611	17.0059	AAL94786.1| N-acyl-L-amino acid amidohydrolase
					43	9	43.0000	11.8062	
FN0592	-0.593	12.849	8.665e-4	3.742e-4	51	84	69.8771	102.0353	AAL94788.1| ATP-dependent DNA helicase pcrA
					70	83	70.0000	108.8798	
FN0593	-0.011	11.085	2.828e-1	8.792e-1	32	36	43.8445	43.7294	AAL94789.1| UDP-3-O-[3-hydroxymyristoyl] N-acetylglucosamine deacetylase
					49	38	49.0000	49.8486	
FN0594	4.420	10.596				7		8.5029	AAL94790.1| (3R)-hydroxymyristoyl-[acyl carrier protein] dehydratase
					182		182.0000		
FN0595	0.421	9.089				17		20.6500	AAL94791.1| Acyl-[acyl-carrier-protein]-UDP-N-acetylglucosamine O-acyltransferase
					27	15	27.0000	19.6771	
FN0596	-0.887	9.143	1.623e-2	2.057e-2	8	23	10.9611	27.9382	AAL94792.1| Hypothetical protein
					24	28	24.0000	36.7305	
FN0597	-0.057	10.396	2.393e-1	7.015e-1	27	26	36.9938	31.5824	AAL94793.1| Lipid-A-disaccharide synthase
					35	33	35.0000	43.2896	
FN0598	-1.214	8.786	2.897e-3	2.133e-3	7	30	9.5910	36.4412	AAL94794.1| Phospholipid-lipopolysaccharide ABC transporter
					18	21	18.0000	27.5479	
FN0600	-0.134	12.447	2.673e-1	8.134e-1	18	63	24.6625	76.5265	AAL94796.1| Hypothetical protein
					118	61	118.0000	80.0201	
FN0601						31		37.6559	AAL94797.1| Hypothetical exported 24-amino acid repeat protein
						27		35.4187	
FN0602	-0.450	14.400	1.856e-2	2.459e-2	107	148	146.6050	179.7765	AAL94798.1| Hypothetical protein
					105	125	105.0000	163.9757	
FN0603	0.608	5.392							AAL94799.1| Transcriptional regulatory protein, LYSR family
					8	4	8.0000	5.2472	
FN0608	1.321	12.367	3.34e-2	5.204e-2	56	39	76.7278	47.3735	AAL94804.1| Exoribonuclease II
					153	34	153.0000	44.6014	
FN0609									AAL94805.1| Small protein B
					6		6.0000		
FN0610	-0.386	15.997	1.266e-1	3.055e-1	93	248	127.4230	301.2471	AAL94806.1| unknown
					320	216	320.0000	283.3499	
FN0611	0.008	18.655	2.588e-1	7.784e-1	461	541	631.6345	657.1561	AAL94807.1| Threonyl-tRNA synthetase
					657	476	657.0000	624.4193	

Log2 Ratios: -6 -4 -2 0 2 4 6

Show detected proteins only
Show all proteins
Filter by category: GO: amino acid transport

Proteins found: 1338

Enter (or paste) list of ORFs

Find ORFs

Test	Cutoff
q-Value	.005
p-Value	

Color	Signif	Direction	Applies To
red	yes	+	ratios, bars
yellow	no	n/a	bars
green	yes	−	ratios, bars
pink	yes	+	*p*- , *q*-Values
light green	yes	−	

Dot Plots Dot Plots

Protein	*FnPg vs Fn* Log$_2$ Ratio	Log$_2$ Sum	*q*-Value	*p*-Value	Raw *FnPg*	Raw *Fn*	Normalized *FnPg*	Normalized *Fn*	Description
**FN0612**	-1.943	12.544	1.296e-3	7.08e-4	13	107	17.8118	129.9736	AAL94808.1| Hypothetical protein
					61	132	61.0000	173.1583	
**FN0615**					3		4.1104		AAL94811.1| Export ABC transporter
**FN0616**	-3.077	8.692				53		64.3794	AAL94812.1| Hypothetical protein
					7	41	7.0000	53.7840	
**FN0617**	0.513	16.384	1.465e-1	3.681e-1	380	200	520.6531	242.9412	AAL94813.1| DNA polymerase III, beta chain
					178	188	178.0000	246.6194	
**FN0618**	-0.465	14.090	1.212e-1	2.89e-1	40	128	54.8056	155.4824	AAL94814.1| Spermidine/putrescine-binding protein
					170	118	170.0000	154.7930	
**FN0619**	-1.035	8.489	3.386e-2	5.301e-2	4	22	5.4806	26.7235	AAL94815.1| Small-conductance mechanosensitive channel
					21	21	21.0000	27.5479	
**FN0621**	-0.074	11.610	2.679e-1	8.158e-1	54	48	73.9876	58.3059	AAL94817.1| 4-hydroxybutyrate coenzyme A transferase
					35	43	35.0000	56.4076	
**FN0622**	2.520	7.838	4.561e-2	7.908e-2	12	5	16.4417	6.0735	AAL94818.1| 8-oxoguanine DNA glycosylase
					56	5	56.0000	6.5590	
**FN0625**	-0.443	9.165			15	19	20.5521	23.0794	AAL94821.1| Aspartate aminotransferase
						25		32.7951	
**FN0627**	-1.099	14.249	1.614e-2	2.042e-2	37	158	50.6952	191.9236	AAL94823.1| Glucosamine--fructose-6-phosphate aminotransferase (isomerizing)
					140	165	140.0000	216.4479	
**FN0628**						3		3.6441	AAL94824.1| Glucosamine--fructose-6-phosphate aminotransferase (isomerizing)
						7		9.1826	
**FN0629**	-0.251	8.251				13		15.7912	AAL94825.1| PTS system, IID component
					16	17	16.0000	22.3007	
**FN0630**									AAL94826.1| PTS system, IIC component
						11		14.4299	
**FN0631**	-0.334	8.509				18		21.8647	AAL94827.1| PTS system, IIB component
					17	16	17.0000	20.9889	
**FN0633**	0.570	7.244				8		9.7176	AAL94829.1| Replication protein
					15	8	15.0000	10.4944	
**FN0634**	-0.481	14.010	1.474e-1	3.711e-1	131	117	179.4883	142.1206	AAL94830.1| GTP-binding protein TypA/BipA
					38	123	38.0000	161.3520	

Log$_2$ Ratios: -6 -4 -2 0 2 4 6

Show detected proteins only
Show all proteins
Filter by category:
GO: amino acid transport

Proteins found: 1338

Enter (or paste) list of ORFs

Find ORFs

Test	Cutoff
q-Value	.005
p-Value	

	Signif	Direction	Applies To
red	yes	+	ratios, bars
yellow	no	n/a	bars
green	yes	–	ratios, bars
pink	yes	+	*p*- , *q*-Values
light green	yes	–	

Dot Plots Dot Plots

Protein	FnPg vs Fn				Raw		Normalized		Description	Log_2 Ratios
	Log_2 Ratio	Log_2 Sum	*q*-Value	*p*-Value	*FnPg*	*Fn*	*FnPg*	*Fn*		-6 -4 -2 0 2 4 6
FN0636						6		7.2882	AAL94832.1| Hypothetical protein	
FN0637						6		7.2882	AAL94833.1| Hypothetical exported 24-amino acid repeat protein	
						7		9.1826		
FN0643	0.764	9.037	6.087e-2	1.184e-1	29	16	39.7341	19.4353	AAL94839.1| hypothetical DNA-binding protein	
					20	12	20.0000	15.7417		
FN0644	-0.006	11.352	2.955e-1	9.351e-1	38	39	52.0653	47.3735	AAL94840.1| Uroporphyrin-III C-methyltransferase	
					50	42	50.0000	55.0958		
FN0645	-0.441	6.247	1.22e-1	2.915e-1	8	7	10.9611	8.5029	AAL94841.1| Porphobilinogen deaminase	
					4	9	4.0000	11.8062		
FN0649					6		8.2208		AAL94845.1| Exoenzymes regulatory protein aepA precursor	
FN0652	1.042	23.635	5.399e-2	9.959e-2	2352	2050	3222.5689	2490.1477	AAL94848.1| Glyceraldehyde 3-phosphate dehydrogenase	
					7137	1937	7137.0000	2540.9668		
FN0653	0.280	11.739	1.59e-1	4.101e-1	32	42	43.8445	51.0177	AAL94849.1| unknown	
					85	42	85.0000	55.0958		
FN0654	-0.077	18.744	2.383e-1	6.975e-1	368	532	504.2115	646.2237	AAL94850.1| Phosphoglycerate kinase	
					786	545	786.0000	714.9339		
FN0655	-2.886	9.805				54		65.5941	AAL94851.1| unknown	
					11	74	11.0000	97.0736		
FN0656						64		77.7412	AAL94852.1| Hypothetical protein	
						76		99.6972		
FN0657	1.009	7.910				9		10.9324	AAL94853.1| Acetyltransferase	
					22		22.0000			
FN0658	-1.362	13.326	3.023e-4	7.136e-5	47	139	64.3966	168.8442	AAL94854.1| ABC transporter substrate-binding protein	
					62	119	62.0000	156.1048		
FN0660	-0.313	7.005	6.954e-2	1.414e-1	9	10	12.3313	12.1471	AAL94856.1| ABC transporter ATP-binding protein	
					8	10	8.0000	13.1181		
FN0662	0.384	11.041	2.322e-1	6.744e-1	5	37	6.8507	44.9441	AAL94858.1| Formiminoglutamase	
					98	27	98.0000	35.4187		
FN0664	-1.329	13.462	7.1e-3	7.367e-3	73	126	100.0202	153.0530	AAL94860.1| 2-nitropropane dioxygenase	
					34	140	34.0000	183.6527		

Show detected proteins only
Show all proteins
Filter by category:
GO: amino acid transport

Proteins found: 1338

Enter (or paste) list of ORFs

Find ORFs

Test	Cutoff
q-Value	.005
p-Value	

	Signif	Direction	Applies To
(red)	yes	+	ratios, bars
(yellow)	no	n/a	bars
(green)	yes	–	ratios, bars
(pink)	yes	+	*p*- , *q*-Values
(light green)	yes	–	

Dot Plots Dot Plots

Protein	FnPg vs Fn Log$_2$ Ratio	Log$_2$ Sum	*q*-Value	*p*-Value	Raw *FnPg*	Raw *Fn*	Normalized *FnPg*	Normalized *Fn*	Description	Log$_2$ Ratios (-6 -4 -2 0 2 4 6)
FN0666						9		10.9324	AAL94862.1| Hypothetical protein	
						10		13.1181		
FN0668	-0.885	9.350	3.602e-2	5.742e-2	7	28	9.5910	34.0118	AAL94864.1| High-affinity zinc uptake system protein znuA precursor	
					28	27	28.0000	35.4187		
FN0672									AAL94868.1| ATPase	
					8		8.0000			
FN0675	0.293	20.681	2.319e-1	6.732e-1	331	936	453.5163	1136.9650	AAL94871.1| 60 kDa chaperonin GROEL	
					2417	919	2417.0000	1205.5490		
FN0676	-2.238	13.222				193		234.4383	AAL94872.1| 10 kDa chaperonin GROES	
					45	145	45.0000	190.2118		
FN0677	-0.452	12.428	7.458e-2	1.549e-1	62	76	84.9487	92.3177	AAL94873.1| Hypothetical protein	
					42	62	42.0000	81.3319		
FN0678	-2.001	10.975	3.84e-3	3.143e-3	5	70	6.8507	85.0294	AAL94874.1| Ser/Thr protein kinase	
					38	72	38.0000	94.4500		
FN0679	-0.047	8.116	2.976e-1	9.447e-1	21	16	28.7729	19.4353	AAL94875.1| GTPase	
					4	11	4.0000	14.4299		
FN0680									AAL94876.1| Ribulose-phosphate 3-epimerase	
					8		8.0000			
FN0681	0.072	16.439	2.266e-1	6.533e-1	260	227	356.2364	275.7383	AAL94877.1| Transcriptional regulator, MarR family	
					255	233	255.0000	305.6506		
FN0682	-0.287	8.933	1.441e-1	3.602e-1	19	24	26.0327	29.1529	AAL94878.1| Fibronectin-binding protein-like protein A	
					14	15	14.0000	19.6771		
FN0684					8		10.9611		AAL94880.1| Prismane protein	
					36		36.0000			
FN0685	1.715	11.795				25		30.3677	AAL94881.1| Sodium/pantothenate symporter	
					108	27	108.0000	35.4187		
FN0688	-0.814	10.428				40		48.5882	AAL94884.1| Hypothetical protein	
					28	38	28.0000	49.8486		
FN0689	-1.400	15.034	7.674e-3	8.181e-3	42	234	57.5459	284.2413	AAL94885.1| Hypothetical protein	
					168	237	168.0000	310.8979		
FN0692						12		14.5765	AAL94888.1| Nitrogen regulation protein NIFR3	
						18		23.6125		

Show detected proteins only
Show all proteins
Filter by category:
GO: amino acid transport

Proteins found: 1338

Enter (or paste) list of ORFs

Find ORFs

Test	Cutoff
q-Value	.005
p-Value	

	Signif	Direction	Applies To
(red)	yes	+	ratios, bars
(yellow)	no	n/a	bars
(green)	yes	–	ratios, bars
(pink)	yes	+	*p*- , *q*-Values
(light green)	yes	–	

Dot Plots Dot Plots

Protein	*FnPg* vs *Fn* Log$_2$ Ratio	Log$_2$ Sum	*q*-Value	*p*-Value	Raw *FnPg*	Raw *Fn*	Normalized *FnPg*	Normalized *Fn*	Description
**FN0693**	-0.715	9.438			15	21	20.5521	25.5088	AAL94889.1| DNA mismatch repair protein mutS
						32		41.9778	
**FN0694**	-1.205	11.775	2.013e-3	1.303e-3	27	67	36.9938	81.3853	AAL94890.1| S-layer protein
					41	75	41.0000	98.3854	
**FN0695**	-0.255	9.735	1.841e-1	5.011e-1	12	32	16.4417	38.8706	AAL94891.1| ABC transporter ATP-binding protein
					37	19	37.0000	24.9243	
**FN0697**	-0.888	15.023	2.426e-3	1.676e-3	114	210	156.1959	255.0883	AAL94893.1| Alanyl-tRNA synthetase
					112	184	112.0000	241.3722	
**FN0699**	-2.041	12.095	2.411e-4	5.118e-5	33	101	45.2146	122.6853	AAL94895.1| Protein translocase subunit secD
					20	111	20.0000	145.6104	
**FN0700**	-1.970	10.170	8.457e-4	3.581e-4	17	48	23.2924	58.3059	AAL94896.1| Protein translocase subunit secF
					11	58	11.0000	76.0847	
**FN0701**	-0.323	15.171	7.1e-4	2.664e-4	120	182	164.4168	221.0765	AAL94897.1| Methyltransferase
					179	159	179.0000	208.5770	
**FN0705**	-0.948	13.356	6.438e-3	6.416e-3	39	123	53.4355	149.4089	AAL94901.1| DNA polymerase I
					94	103	94.0000	135.1159	
**FN0706**						6		7.2882	AAL94902.1| Hypothetical cytosolic protein
						10		13.1181	
**FN0707**	-0.919	8.340	2.628e-2	3.818e-2	14	17	19.1820	20.6500	AAL94903.1| Riboflavin kinase
					7	22	7.0000	28.8597	
**FN0710**	-0.325	11.975	3.353e-2	5.231e-2	47	64	64.3966	77.7412	AAL94906.1| Hypothetical protein
					49	49	49.0000	64.2785	
**FN0711**	0.574	7.601				8		9.7176	AAL94907.1| Phosphopantothenate--cysteine ligase
					17	10	17.0000	13.1181	
**FN0714**					7		9.5910		AAL94910.1| NADH oxidase
					7		7.0000		
**FN0715**	0.137	14.316	2.68e-1	8.164e-1	177	113	242.5148	137.2618	AAL94911.1| Hypothetical protein
					57	103	57.0000	135.1159	
**FN0716**	-0.970	11.302	2.889e-2	4.303e-2	40	65	54.8056	78.9559	AAL94912.1| hypothetical protein
					17	47	17.0000	61.6548	
**FN0717**									AAL94913.1| Ribosomal small subunit pseudouridine synthase A
						6		7.8708	

Log$_2$ Ratios: -6 -4 -2 0 2 4 6

Show detected proteins only
Show all proteins
Filter by category:
GO: amino acid transport

Proteins found: 1338

Enter (or paste) list of ORFs
Find ORFs

Test	Cutoff
q-Value	.005
p-Value	

Signif	Direction	Applies To
yes	+	ratios, bars
no	n/a	bars
yes	−	ratios, bars
yes	+	*p*- , *q*-Values
yes	−	

Dot Plots Dot Plots

Protein	FnPg vs Fn Log$_2$ Ratio	Log$_2$ Sum	*q*-Value	*p*-Value	Raw *FnPg*	Raw *Fn*	Normalized *FnPg*	Normalized *Fn*	Description
FN0719						9		10.9324	AAL94915.1| Hypothetical cytosolic protein
						8		10.4944	
FN0720	-1.271	13.663	1.901e-4	3.531e-5	45	139	61.6563	168.8442	AAL94916.1| Protein Translation Elongation Factor P (EF-P)
					85	141	85.0000	184.9645	
FN0721	-2.523	10.323	1.122e-3	5.666e-4	5	70	6.8507	85.0294	AAL94917.1| Hypothetical protein
					23	66	23.0000	86.5791	
FN0722	-0.674	7.275	4.278e-2	7.23e-2	10	14	13.7014	17.0059	AAL94918.1| WD-repeat family protein
					6	11	6.0000	14.4299	
FN0723									AAL94919.1| Hypothetical protein
					4		4.0000		
FN0724	0.527	10.961	1.99e-3	1.28e-3	41	31	56.1757	37.6559	AAL94920.1| Flavodoxin
					51	28	51.0000	36.7305	
FN0725	0.639	7.469	1.276e-1	3.087e-1	6	10	8.2208	12.1471	AAL94921.1| Molybdopterin biosynthesis MoeB protein
					25	7	25.0000	9.1826	
FN0728	0.390	10.411	3.142e-3	2.396e-3	31	25	42.4743	30.3677	AAL94924.1| Hypothetical protein
					42	26	42.0000	34.1069	
FN0729	-0.595	15.069	5.641e-2	1.06e-1	153	197	209.6314	239.2971	AAL94925.1| Phosphoglycerate mutase
					92	165	92.0000	216.4479	
FN0731	0.969	9.971	6.08e-2	1.182e-1	45	20	61.6563	24.2941	AAL94927.1| Hypothetical protein
					27	16	27.0000	20.9889	
FN0733	-0.641	13.087	1.256e-2	1.49e-2	66	100	90.4292	121.4706	AAL94929.1| Peptidase T
					59	85	59.0000	111.5034	
FN0734						9		10.9324	AAL94930.1| Fe-S oxidoreductase
						9		11.8062	
FN0735	0.018	13.715	3.04e-1	9.736e-1	136	99	186.3390	120.2559	AAL94931.1| Cell surface protein
					47	84	47.0000	110.1916	
FN0736					5		6.8507		AAL94932.1| Methyltransferase
					6		6.0000		
FN0738	-0.806	11.458	7.814e-4	3.119e-4	33	55	45.2146	66.8088	AAL94934.1| Hypothetical exported 24-amino acid repeat protein
					35	56	35.0000	73.4611	
FN0739	-0.513	15.451	1.985e-3	1.275e-3	125	196	171.2675	238.0824	AAL94935.1| Formiminotetrahydrofolate cyclodeaminase
					183	204	183.0000	267.6083	

Log$_2$ Ratios: -6 -4 -2 0 2 4 6

Show detected proteins only
Show all proteins
Filter by category:
GO: amino acid transport

Proteins found: 1338

Enter (or paste) list of ORFs

Find ORFs

Test	Cutoff
q-Value	.005
p-Value	

	Signif	Direction	Applies To
red	yes	+	ratios, bars
yellow	no	n/a	bars
green	yes	−	ratios, bars
pink	yes	+	*p*- , *q*-Values
light green	yes	−	*p*- , *q*-Values

Dot Plots Dot Plots

FnPg vs Fn

Fusobacterium nucleatum

Hackett Laboratory UW

Fn Summary Table | FnPg vs Fn | FnSg vs Fn | FnPgSg vs Fn | FnPgSg vs FnPg | FnSg vs FnPg | FnPgSg vs FnSg | Fn Coverage

Protein	FnPg vs Fn Log$_2$ Ratio	Log$_2$ Sum	*q*-Value	*p*-Value	Raw FnPg	Raw Fn	Normalized FnPg	Normalized Fn	Description	Log$_2$ Ratios (-6 -4 -2 0 2 4 6)
FN0740	-0.223	17.608	2.153e-1	6.116e-1	147	401	201.4106	487.0972	AAL94936.1| Imidazolonepropionase	
					626	365	626.0000	478.8089		
FN0741	-0.254	18.286	1.884e-1	5.171e-1	206	525	282.2488	637.7208	AAL94937.1| Glutamate formiminotransferase	
					753	455	753.0000	596.8714		
FN0742	-0.400	12.066				72		87.4588	AAL94938.1| unknown	
					57	48	57.0000	62.9667		
FN0743						9		10.9324	AAL94939.1| ATP-dependent helicase, DinG family	
						9		11.8062		
FN0745	-0.804	14.359	2.971e-2	4.461e-2	47	135	64.3966	163.9853	AAL94941.1| metal dependent phosphohydrolase	
					155	167	155.0000	219.0715		
FN0746	0.470	7.371			17	9	23.2924	10.9324	AAL94942.1| Hypothetical Metal-Binding Protein	
					7		7.0000			
FN0749						10		12.1471	AAL94945.1| Hypothetical protein	
						6		7.8708		
FN0750	-0.814	9.463	6.976e-3	7.184e-3	11	31	15.0715	37.6559	AAL94946.1| Hypothetical protein	
					25	25	25.0000	32.7951		
FN0751	-0.553	8.549	4.505e-2	7.772e-2	8	17	10.9611	20.6500	AAL94947.1| L-asparaginase I	
					21	20	21.0000	26.2361		
FN0752	-0.408	8.171	1.659e-1	4.341e-1	4	16	5.4806	19.4353	AAL94948.1| Proline iminopeptidase	
					24	15	24.0000	19.6771		
FN0753	0.623	12.598	6.089e-3	5.94e-3	74	44	101.3903	53.4471	AAL94949.1| Glutamyl-tRNA(Gln) amidotransferase subunit B	
					94	56	94.0000	73.4611		
FN0754	-0.166	13.764	1.542e-1	3.937e-1	64	102	87.6890	123.9000	AAL94950.1| Glutamyl-tRNA(Gln) amidotransferase subunit A	
					135	96	135.0000	125.9333		
FN0755	-0.990	10.984	4.575e-3	4.019e-3	24	58	32.8834	70.4530	AAL94951.1| Glutamyl-tRNA(Gln) amidotransferase subunit C	
					31	43	31.0000	56.4076		
FN0758	-0.636	15.014	6.197e-4	2.131e-4	97	179	132.9036	217.4324	AAL94954.1| Rod shape-determining protein mreB	
					159	180	159.0000	236.1249		
FN0761	1.146	9.350				11		13.3618	AAL94957.1| Bvg accessory factor	
					38	16	38.0000	20.9889		
FN0765	-0.706	6.321				8		9.7176	AAL94961.1| tRNA (5-methylaminomethyl -2-thiouridylate) -methyltransferase	
					7	10	7.0000	13.1181		

Show detected proteins only
Show all proteins
Filter by category:
GO: amino acid transport

Proteins found: 1338

Enter (or paste) list of ORFs

Find ORFs

Test	Cutoff
q-Value	.005
p-Value	

	Signif	Direction	Applies To
red	yes	+	ratios, bars
yellow	no	n/a	bars
green	yes	−	ratios, bars
pink	yes	+	*p*- , *q*-Values
light green	yes	−	

Dot Plots Dot Plots

Protein	FnPg vs Fn: Log_2 Ratio	FnPg vs Fn: Log_2 Sum	FnPg vs Fn: *q*-Value	FnPg vs Fn: *p*-Value	Raw: *FnPg*	Raw: *Fn*	Normalized: *FnPg*	Normalized: *Fn*	Description
FN0766						15		18.2206	AAL94962.1| Large-conductance mechanosensitive channel
						16		20.9889	
FN0768	-1.501	5.501				10		12.1471	AAL94964.1| Hemin receptor
					4	8	4.0000	10.4944	
FN0771									AAL94967.1| Oxygen-independent coproporphyrinogen III oxidase
						14		18.3653	
FN0772						7		8.5029	AAL94968.1| Flavodoxin
						3		3.9354	
FN0774	-1.485	13.578	9.811e-4	4.556e-4	60	148	82.2084	179.7765	AAL94970.1| Hypothetical cytosolic protein
					50	145	50.0000	190.2118	
FN0775	-0.365	16.737	1.277e-1	3.09e-1	126	313	172.6376	380.2030	AAL94971.1| Aspartyl aminopeptidase
					410	282	410.0000	369.9291	
FN0776	-0.544	12.818	9.877e-3	1.119e-2	48	75	65.7667	91.1030	AAL94972.1| Aspartate-ammonia ligase
					75	87	75.0000	114.1271	
FN0777	-0.718	11.661	1.643e-2	2.09e-2	29	50	39.7341	60.7353	AAL94973.1| GTP-binding protein lepA
					49	65	49.0000	85.2673	
FN0778	-1.013	9.000	3.391e-2	5.311e-2	5	27	6.8507	32.7971	AAL94974.1| Methyltransferase
					25	24	25.0000	31.4833	
FN0779									AAL94975.1| Putative GTPases (G3E family)
					5		5.0000		
FN0783	0.114	23.085	1.388e-1	3.433e-1	2527	2445	3462.3434	2969.9567	AAL94979.1| acyl-CoA dehydrogenase
					2744	2107	2744.0000	2763.9737	
FN0784	-0.300	22.413	8.082e-2	1.723e-1	1903	2170	2607.3761	2635.9125	AAL94980.1| Electron transfer flavoprotein beta-subunit
					1653	1988	1653.0000	2607.8689	
FN0785	0.019	21.833	2.747e-1	8.446e-1	1553	1670	2127.8272	2028.5594	AAL94981.1| Electron transfer flavoprotein alpha-subunit
					1763	1381	1763.0000	1811.6031	
FN0788	0.597	9.459	5.714e-3	5.441e-3	25	15	34.2535	18.2206	AAL94984.1| unknown
					31	19	31.0000	24.9243	
FN0790	-1.542	6.739	1.755e-3	1.065e-3	3	15	4.1104	18.2206	AAL94986.1| Xylose repressor
					8	13	8.0000	17.0535	
FN0791	-0.142	18.534	1.267e-1	3.059e-1	367	558	502.8413	677.8061	AAL94987.1| Histidine ammonia-lyase
					670	470	670.0000	616.5485	

Log_2 Ratios: -6 -4 -2 0 2 4 6

Show detected proteins only
Show all proteins
Filter by category:
GO: amino acid transport

Proteins found: 1338

Enter (or paste) list of ORFs

Find ORFs

Test	Cutoff
q-Value	.005
p-Value	

	Signif	Direction	Applies To
(red)	yes	+	ratios, bars
(yellow)	no	n/a	bars
(green)	yes	−	ratios, bars
(pink)	yes	+	*p*- , *q*-Values
(light green)	yes	−	

Dot Plots Dot Plots

Protein	FnPg vs Fn Log$_2$ Ratio	Log$_2$ Sum	*q*-Value	*p*-Value	Raw *FnPg*	Raw *Fn*	Normalized *FnPg*	Normalized *Fn*	Description	Log$_2$ Ratios (-6 -4 -2 0 2 4 6)
FN0792	0.041	20.440	2.873e-1	8.985e-1	573	1002	785.0901	1217.1356	AAL94988.1| Urocanate hydratase	
					1635	865	1635.0000	1134.7116		
FN0793	-0.749	7.919				17		20.6500	AAL94989.1| Sodium/glutamate symport carrier protein	
					12	15	12.0000	19.6771		
FN0794	0.570	7.244				8		9.7176	AAL94990.1| Hypothetical protein	
					15	8	15.0000	10.4944		
FN0796	-0.165	15.072	1.399e-1	3.468e-1	104	168	142.4945	204.0706	AAL94992.1| Pyruvate,phosphate dikinase	
					208	144	208.0000	188.9000		
FN0798	-0.232	7.274	2.163e-1	6.154e-1	8	6	10.9611	7.2882	AAL94994.1| Fructose-1,6-bisphosphatase	
					12	15	12.0000	19.6771		
FN0799	0.417	8.365	1.651e-1	4.315e-1	8	14	10.9611	17.0059	AAL94995.1| Isoamylase	
					31	11	31.0000	14.4299		
FN0800	-0.203	10.414	2.558e-1	7.663e-1	5	35	6.8507	42.5147	AAL94996.1| Amino acid-binding protein	
					62	28	62.0000	36.7305		
FN0801	0.663	7.677				9		10.9324	AAL94997.1| Amino acid transport ATP-binding protein	
					18	9	18.0000	11.8062		
FN0802									AAL94998.1| Amino acid transport system permease protein	
					6		6.0000			
FN0803	-1.108	12.619	9.426e-3	1.057e-2	46	114	63.0264	138.4765	AAL94999.1| Cytochrome C-TYPE biogenesis protein ccdA	
					45	72	45.0000	94.4500		
FN0805	1.098	6.525			11		15.0715		AAL95001.1| Hypothetical protein	
					13	5	13.0000	6.5590		
FN0806	-0.933	12.992	3.472e-3	2.747e-3	45	92	61.6563	111.7530	AAL95002.1| SpoIID homolog	
					69	105	69.0000	137.7396		
FN0807	-0.123	10.335	2.081e-1	5.857e-1	24	38	32.8834	46.1588	AAL95003.1| 3-deoxy-manno-octulosonate cytidylyltransferase	
					36	22	36.0000	28.8597		
FN0808	0.194	12.115	7.773e-2	1.635e-1	58	55	79.4681	66.8088	AAL95004.1| Phosphoglycerate mutase	
					63	44	63.0000	57.7194		
FN0809	-1.688	7.688				23		27.9382	AAL95005.1| 23S rRNA methyltransferase	
					8	18	8.0000	23.6125		
FN0810	-0.040	11.620	2.449e-1	7.231e-1	45	44	61.6563	53.4471	AAL95006.1| Low-specificity threonine aldolase	
					49	46	49.0000	60.3430		

Show detected proteins only
Show all proteins
Filter by category:
GO: amino acid transport

Proteins found: 1338

Enter (or paste) list of ORFs

Find ORFs

Test	Cutoff
q-Value	.005
p-Value	

Color	Signif	Direction	Applies To
red	yes	+	ratios, bars
yellow	no	n/a	bars
green	yes	–	ratios, bars
pink	yes	+	*p*- , *q*-Values
light green	yes	–	

Dot Plots Dot Plots

Protein	*FnPg vs Fn* Log$_2$ Ratio	Log$_2$ Sum	*q*-Value	*p*-Value	Raw *FnPg*	Raw *Fn*	Normalized *FnPg*	Normalized *Fn*	Description
FN0813	1.746	8.898				11		13.3618	AAL95009.1| Transcriptional regulator, TetR family
					40	8	40.0000	10.4944	
FN0814	-3.939	13.406	4.364e-6	2.23e-7	33	322	45.2146	391.1354	AAL95010.1| Propionate CoA-transferase
					8	324	8.0000	425.0249	
FN0815						47		57.0912	AAL95011.1| Propionate permease
						39		51.1604	
FN0816	-2.404	13.956			40	228	54.8056	276.9530	AAL95012.1| dehydrogenase with MaoC-like domain
						231		303.0270	
FN0818	-0.807	19.104	2.353e-2	3.323e-2	567	761	776.8693	924.3914	AAL95014.1| DNA-binding protein HU
					358	809	358.0000	1061.2505	
FN0819	-0.455	11.840	4.324e-2	7.337e-2	47	53	64.3966	64.3794	AAL95015.1| Tetratricopeptide repeat family protein
					39	59	39.0000	77.3965	
FN0820	-0.033	14.729	2.787e-1	8.615e-1	94	134	128.7931	162.7706	AAL95016.1| Mercuric reductase
					197	130	197.0000	170.5347	
FN0821	-0.301	13.129	1.883e-1	5.166e-1	96	79	131.5334	95.9618	AAL95017.1| Hypothetical protein
					39	87	39.0000	114.1271	
FN0823	1.149	9.558	2.902e-2	4.329e-2	21	12	28.7729	14.5765	AAL95019.1| GTP-binding protein hflX
					53	17	53.0000	22.3007	
FN0825	-1.068	12.189	1.567e-2	1.965e-2	20	83	27.4028	100.8206	AAL95021.1| Hypothetical cytosolic protein
					67	74	67.0000	97.0736	
FN0826	-1.809	9.442	4.137e-4	1.131e-4	14	37	19.1820	44.9441	AAL95022.1| periplasmic component of efflux system
					9	41	9.0000	53.7840	
FN0827	-2.166	9.449	9.592e-3	1.08e-2	8	35	10.9611	42.5147	AAL95023.1| ABC transporter ATP-binding protein
					14	53	14.0000	69.5257	
FN0828						17		20.6500	AAL95024.1| ABC transporter permease protein
						15		19.6771	
FN0830	1.059	11.207	1.03e-3	4.918e-4	55	22	75.3577	26.7235	AAL95026.1| Hypothetical protein
					65	31	65.0000	40.6660	
FN0831					13		17.8118		AAL95027.1| Hemin receptor
					6		6.0000		
FN0832	0.110	12.638	2.816e-1	8.741e-1	105	65	143.8647	78.9559	AAL95028.1| Hypothetical protein
					22	57	22.0000	74.7729	

Log$_2$ Ratios: -6 -4 -2 0 2 4 6

Show detected proteins only
Show all proteins
Filter by category:
GO: amino acid transport

Proteins found: 1338

Enter (or paste) list of ORFs

Find ORFs

Test	Cutoff
q-Value	.005
p-Value	

	Signif	Direction	Applies To
red	yes	+	ratios, bars
yellow	no	n/a	bars
green	yes	−	ratios, bars
pink	yes	+	*p*- , *q*-Values
light green	yes	−	

Dot Plots Dot Plots

Protein	FnPg vs Fn Log$_2$ Ratio	Log$_2$ Sum	*q*-Value	*p*-Value	Raw *FnPg*	Raw *Fn*	Normalized *FnPg*	Normalized *Fn*	Description
FN0833	-3.561	8.205				41		49.8030	AAL95029.1| Hypothetical protein
					5	52	5.0000	68.2139	
FN0834	-0.508	10.209	1.577e-1	4.055e-1	37	33	50.6952	40.0853	AAL95030.1| Hypothetical Exported Protein
					7	32	7.0000	41.9778	
FN0835					27		36.9938		AAL95031.1| Hypothetical protein
					4		4.0000		
FN0836	1.061	8.887	5.62e-2	1.054e-1	32	14	43.8445	17.0059	AAL95032.1| Hypothetical protein
					19	10	19.0000	13.1181	
FN0846	0.094	13.359	1.674e-1	4.394e-1	83	91	113.7216	110.5383	AAL95042.1| Hypothetical Exported Protein
					98	67	98.0000	87.8910	
FN0847	-1.659	7.659				20		24.2941	AAL95043.1| TPR-repeat-containing proteins
					8	20	8.0000	26.2361	
FN0848					5		6.8507		AAL95044.1| Hypothetical protein
FN0849	0.764	11.033	3.726e-3	3.018e-3	44	33	60.2862	40.0853	AAL95045.1| 8-amino-7-oxononanoate synthase
					59	23	59.0000	30.1715	
FN0850	0.248	9.184	1.255e-1	3.022e-1	15	17	20.5521	20.6500	AAL95046.1| Hypothetical cytosolic protein
					32	18	32.0000	23.6125	
FN0853	0.961	12.611	6.882e-3	7.045e-3	91	48	124.6827	58.3059	AAL95049.1| Glycogen synthase
					96	42	96.0000	55.0958	
FN0854	1.630	13.097	4.36e-3	3.751e-3	139	39	190.4494	47.3735	AAL95050.1| Glucose-1-phosphate adenylyltransferase
					139	45	139.0000	59.0312	
FN0855	0.488	14.558	6.521e-2	1.302e-1	102	109	139.7543	132.4030	AAL95051.1| Glucose-1-phosphate adenylyltransferase
					228	99	228.0000	129.8687	
FN0856	1.224	13.568	1.528e-4	2.598e-5	124	55	169.8973	66.8088	AAL95052.1| 1,4-alpha-glucan branching enzyme
					167	59	167.0000	77.3965	
FN0857	0.317	15.822	9.16e-2	2.026e-1	155	192	212.3717	233.2236	AAL95053.1| Glycogen phosphorylase
					325	151	325.0000	198.0826	
FN0858	1.039	10.442	1.199e-3	6.356e-4	35	18	47.9549	21.8647	AAL95054.1| 4-alpha-glucanotransferase
					59	23	59.0000	30.1715	
FN0865	2.292	12.857	9.565e-2	2.144e-1	17	36	23.2924	43.7294	AAL95061.1| unknown
					358	26	358.0000	34.1069	

Log$_2$ Ratios: -6 -4 -2 0 2 4 6

Show detected proteins only
Show all proteins
Filter by category:
GO: amino acid transport

Proteins found: 1338

Enter (or paste) list of ORFs

Find ORFs

Test	Cutoff
q-Value	.005
p-Value	

	Signif	Direction	Applies To
red	yes	+	ratios, bars
yellow	no	n/a	bars
green	yes	−	ratios, bars
pink	yes	+	*p*- , *q*-Values
light green	yes	−	

Dot Plots Dot Plots

Protein	FnPg vs Fn: Log_2 Ratio	Log_2 Sum	*q*-Value	*p*-Value	Raw *FnPg*	Raw *Fn*	Normalized *FnPg*	Normalized *Fn*	Description
FN0867	-2.181	12.264	1.015e-4	1.38e-5	24	126	32.8834	153.0530	AAL95063.1| Long-chain-fatty-acid--CoA ligase
					33	111	33.0000	145.6104	
FN0868						8		9.7176	AAL95064.1| ATPases of the PP superfamily
						5		6.5590	
FN0869	-0.713	4.713							AAL95065.1| Hydrolase (HAD superfamily)
					4	5	4.0000	6.5590	
FN0870									AAL95066.1| Rhodanese-related sulfurtransferases
						5		6.5590	
FN0871	0.193	7.807				9		10.9324	AAL95067.1| 3-dehydroquinate synthase
					16	13	16.0000	17.0535	
FN0873	1.534	10.390	7.542e-2	1.571e-1	18	16	24.6625	19.4353	AAL95069.1| Protease IV
					100	18	100.0000	23.6125	
FN0874									AAL95070.1| Phosphohydrolase (MUTT/NUDIX family protein)
					11		11.0000		
FN0878	-0.755	8.025	4.545e-2	7.868e-2	5	14	6.8507	17.0059	AAL95074.1| Transcriptional regulator, GntR family
					18	19	18.0000	24.9243	
FN0885									AAL95081.1| Hemin-binding periplasmic protein hmuT precursor
						10		13.1181	
FN0886						15		18.2206	AAL95082.1| Hemin receptor
						9		11.8062	
FN0887	-1.252	11.062	2.731e-3	1.965e-3	16	57	21.9222	69.2383	AAL95083.1| Oligoendopeptidase F
					38	56	38.0000	73.4611	
FN0888	-1.032	9.253	3.171e-3	2.428e-3	15	29	20.5521	35.2265	AAL95084.1| Uracil permease
					14	27	14.0000	35.4187	
FN0889	1.569	8.230	9.02e-2	1.987e-1	37	9	50.6952	10.9324	AAL95085.1| hypothetical protein
					9	7	9.0000	9.1826	
FN0893	-1.479	7.093				17		20.6500	AAL95089.1| Hypothetical protein
					7	14	7.0000	18.3653	
FN0896	-0.599	8.237	6.117e-2	1.193e-1	6	19	8.2208	23.0794	AAL95092.1| Hypothetical protein
					20	15	20.0000	19.6771	
FN0898									AAL95094.1| Hypothetical protein
					5		5.0000		

Log_2 Ratios: -6 -4 -2 0 2 4 6

Show detected proteins only
Show all proteins
Filter by category:
GO: amino acid transport

Proteins found: 1338

Enter (or paste) list of ORFs

Find ORFs

Test	Cutoff
q-Value	.005
p-Value	

Signif	Direction	Applies To
yes	+	ratios, bars
no	n/a	bars
yes	–	ratios, bars
yes	+	*p*- , *q*-Values
yes	–	

Dot Plots Dot Plots

Protein	FnPg vs Fn Log_2 Ratio	Log_2 Sum	*q*-Value	*p*-Value	Raw *FnPg*	Raw *Fn*	Normalized *FnPg*	Normalized *Fn*	Description	Log_2 Ratios (-6 -4 -2 0 2 4 6)
FN0900	-0.025	5.001	2.996e-1	9.537e-1	6	4	8.2208	4.8588	AAL95096.1| Metal dependent hydrolase	
					3	5	3.0000	6.5590		
FN0901	0.456	5.883							AAL95097.1| DNA polymerase, bacteriophage-type	
					9	5	9.0000	6.5590		
FN0903	0.985	8.149	1.034e-2	1.181e-2	20	10	27.4028	12.1471	AAL95099.1| Polysialic acid capsule expression protein kpsF	
					20	9	20.0000	11.8062		
FN0904									AAL95100.1| NAD(FAD)-utilizing dehydrogenases	
					4		4.0000			
FN0905						17		20.6500	AAL95101.1| Hypothetical protein	
						15		19.6771		
FN0906	-0.153	9.234	8.966e-2	1.971e-1	15	21	20.5521	25.5088	AAL95102.1| Glycerol-3-phosphate dehydrogenase [NAD(P)+]	
					26	20	26.0000	26.2361		
FN0908	-0.956	8.770				22		26.7235	AAL95104.1| Tpl protein	
					15	24	15.0000	31.4833		
FN0909					7		9.5910		AAL95105.1| DNA repair protein radC	
FN0910	0.050	5.950				3		3.6441	AAL95106.1| Nicotinate-nucleotide--dimethylbenzimidazole phosphoribosyltransferase	
					8	9	8.0000	11.8062		
FN0911	-0.258	6.598				8		9.7176	AAL95107.1| Alpha-ribazole-5'-phosphate phosphatase	
					9	9	9.0000	11.8062		
FN0912									AAL95108.1| Cobalamin [5'-phosphate] synthase	
					19		19.0000			
FN0913	0.177	6.466				7		8.5029	AAL95109.1| Cobinamide kinase	
					10	7	10.0000	9.1826		
FN0915	0.563	10.087	1.446e-1	3.619e-1	14	22	19.1820	26.7235	AAL95111.1| PTS system, N-acetylglucosamine-specific IIA component	
					61	21	61.0000	27.5479		
FN0916	-0.426	14.600	6.671e-2	1.341e-1	70	141	95.9098	171.2736	AAL95112.1| Hypothetical Exported Protein	
					176	148	176.0000	194.1472		
FN0917	-1.434	5.434				7		8.5029	AAL95113.1| Hypothetical protein	
					4	10	4.0000	13.1181		
FN0920	0.123	6.521							AAL95116.1| Protease HTPX	
					10	7	10.0000	9.1826		

Show detected proteins only
Show all proteins
Filter by category:
GO: amino acid transport

Proteins found: 1338

Enter (or paste) list of ORFs

Find ORFs

Test	Cutoff
q-Value	.005
p-Value	

Color	Signif	Direction	Applies To
red	yes	+	ratios, bars
yellow	no	n/a	bars
green	yes	–	ratios, bars
pink	yes	+	*p*- , *q*-Values
light green	yes	–	

Dot Plots Dot Plots

Protein	FnPg vs Fn Log2 Ratio	FnPg vs Fn Log2 Sum	FnPg vs Fn *q*-Value	FnPg vs Fn *p*-Value	Raw *FnPg*	Raw *Fn*	Normalized *FnPg*	Normalized *Fn*	Description
**FN0921**	2.442	8.542				5		6.0735	AAL95117.1| Hypothetical protein
					45	8	45.0000	10.4944	
**FN0923**									AAL95119.1| Cardiolipin synthetase
					4		4.0000		
**FN0925**	-1.009	6.179				8		9.7176	AAL95121.1| Hypothetical protein
					6	11	6.0000	14.4299	
**FN0926**	2.552	8.283			31	6	42.4743	7.2882	AAL95122.1| GTP pyrophosphokinase
					43		43.0000		
**FN0928**	0.234	6.410				7		8.5029	AAL95124.1| O-sialoglycoprotein endopeptidase
					10		10.0000		
**FN0929**					12		16.4417		AAL95125.1| ATP/GTP hydrolase
					21		21.0000		
**FN0930**	-1.586	6.895	6.277e-3	6.195e-3	7	16	9.5910	19.4353	AAL95126.1| Glycerol-3-phosphate cytidylyltransferase
					3	14	3.0000	18.3653	
**FN0932**	-0.282	8.096				16		19.4353	AAL95128.1| Hypothetical protein
					15	13	15.0000	17.0535	
**FN0934**	1.256	7.529				8		9.7176	AAL95130.1| Chorismate synthase
					21	6	21.0000	7.8708	
**FN0938**									AAL95134.1| Hypothetical protein
					47		47.0000		
**FN0940**									AAL95136.1| Hypothetical protein
					41		41.0000		
**FN0941**	1.593	10.529	3.764e-3	3.06e-3	42	17	57.5459	20.6500	AAL95137.1| Gamma-glutamyltranspeptidase
					76	18	76.0000	23.6125	
**FN0943**	1.943	8.232				7		8.5029	AAL95139.1| Sensory Transduction Protein Kinase
					34	7	34.0000	9.1826	
**FN0944**									AAL95140.1| Na+ driven multidrug efflux pump
					4		4.0000		
**FN0947**	-0.292	12.982	1.46e-1	3.666e-1	80	71	109.6112	86.2441	AAL95143.1| Hypothetical protein
					53	86	53.0000	112.8153	
**FN0949**	-1.101	15.535	5.008e-2	8.975e-2	196	251	268.5474	304.8913	AAL95145.1| DNA helicase
					29	254	29.0000	333.1985	

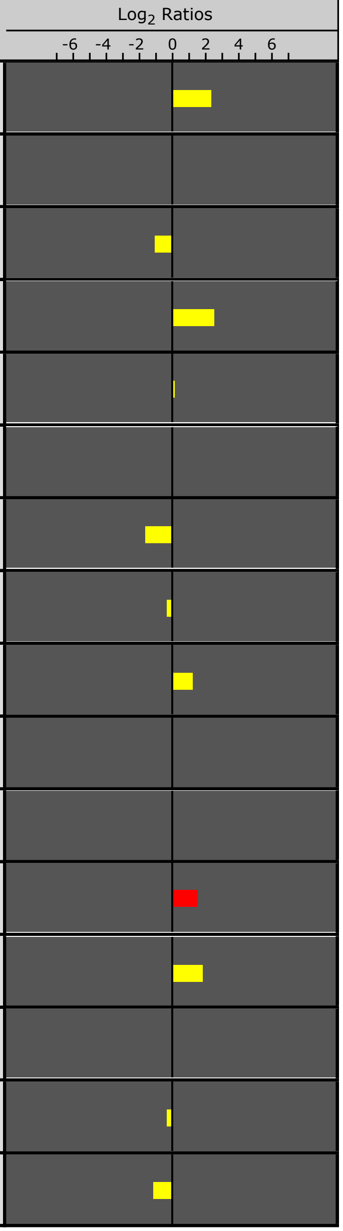

Show detected proteins only
Show all proteins
Filter by category:
GO: amino acid transport

Proteins found: 1338

Enter (or paste) list of ORFs

Find ORFs

Test	Cutoff
q-Value	.005
p-Value	

	Signif	Direction	Applies To
red	yes	+	ratios, bars
yellow	no	n/a	bars
green	yes	−	ratios, bars
pink	yes	+	*p*- , *q*-Values
light green	yes	−	

Dot Plots Dot Plots

Protein	FnPg vs Fn: Log_2 Ratio	FnPg vs Fn: Log_2 Sum	FnPg vs Fn: *q*-Value	FnPg vs Fn: *p*-Value	Raw: *FnPg*	Raw: *Fn*	Normalized: *FnPg*	Normalized: *Fn*	Description
FN0951	-0.699	7.680	6.285e-2	1.24e-1	4	16	5.4806	19.4353	AAL95147.1| Precorrin-3B C17-methyltransferase
					17	13	17.0000	17.0535	
FN0952									AAL95148.1| Cobalamin biosynthesis protein G
						6		7.8708	
FN0957	0.610	8.568	5.36e-2	9.857e-2	22	13	30.1431	15.7912	AAL95153.1| Precorrin-4 C11-methyltransferase
					18	12	18.0000	15.7417	
FN0958	0.088	9.464			20	23	27.4028	27.9382	AAL95154.1| unknown
						18		23.6125	
FN0959	-1.417	8.838	6.722e-3	6.814e-3	14	23	19.1820	27.9382	AAL95155.1| Precorrin-2 C20-methyltransferase
					7	32	7.0000	41.9778	
FN0961	1.043	6.383	5.123e-2	9.257e-2	6	4	8.2208	4.8588	AAL95157.1| Hypothetical protein
					18	6	18.0000	7.8708	
FN0962	-0.529	10.719	3.717e-3	3.008e-3	28	38	38.3639	46.1588	AAL95158.1| Hypothetical cytosolic protein
					30	40	30.0000	52.4722	
FN0964					5		6.8507		AAL95160.1| Precorrin-8W decarboxylase
					6		6.0000		
FN0965	0.981	10.948	4.022e-3	3.349e-3	51	24	69.8771	29.1529	AAL95161.1| D-3-phosphoglycerate dehydrogenase
					55	26	55.0000	34.1069	
FN0966									AAL95162.1| Precorrin-6Y C5,15-methyltransferase (decarboxylating)
					3		3.0000		
FN0967					7		9.5910		AAL95163.1| CbiD protein
FN0970	-0.918	8.166			9	20	12.3313	24.2941	AAL95166.1| Precorrin-8X methylmutase
						17		22.3007	
FN0972					3		4.1104		AAL95168.1| Cobyrinic acid a,c-diamide synthase
					22		22.0000		
FN0974									AAL95170.1| Lactoylglutathione lyase
					4		4.0000		
FN0976	0.212	10.842	8.715e-3	9.619e-3	33	31	45.2146	37.6559	AAL95172.1| Hypothetical protein
					47	32	47.0000	41.9778	
FN0977	0.466	7.027			5	8	6.8507	9.7176	AAL95173.1| Cobyric acid synthase
					20		20.0000		

Log_2 Ratios: -6 -4 -2 0 2 4 6

Show detected proteins only
Show all proteins
Filter by category:
GO: amino acid transport

Proteins found: 1338

Enter (or paste) list of ORFs

Find ORFs

Test	Cutoff
q-Value	.005
p-Value	

Signif	Direction	Applies To
yes	+	ratios, bars
no	n/a	bars
yes	−	ratios, bars
yes	+	*p*- , *q*-Values
yes	−	

Dot Plots Dot Plots

Protein	FnPg vs Fn Log2 Ratio	Log2 Sum	*q*-Value	*p*-Value	Raw *FnPg*	Raw *Fn*	Normalized *FnPg*	Normalized *Fn*	Description
FN0981	-0.541	13.269	9.925e-2	2.25e-1	29	98	39.7341	119.0412	AAL95177.1| Phosphoribosylamine--glycine ligase
					125	92	125.0000	120.6861	
FN0982	-0.982	15.812	3.046e-3	2.291e-3	101	272	138.3841	330.4001	AAL95178.1| Phosphoribosylaminoimidazolecarboxamide formyltransferase
					203	262	203.0000	343.6930	
FN0983	-0.487	14.330	1.85e-2	2.448e-2	104	135	142.4945	163.9853	AAL95179.1| Hypothetical protein
					100	134	100.0000	175.7819	
FN0984	0.262	9.711	6.168e-2	1.208e-1	20	23	27.4028	27.9382	AAL95180.1| Tetracenomycin polyketide synthesis O-methyltransferase tcmP
					36	19	36.0000	24.9243	
FN0985	-0.225	6.225				10		12.1471	AAL95181.1| Phosphoribosylglycinamide formyltransferase
					8	5	8.0000	6.5590	
FN0986	0.244	14.606	2.176e-1	6.201e-1	64	119	87.6890	144.5500	AAL95182.1| Phosphoribosylformylglycinamidine cyclo-ligase
					256	111	256.0000	145.6104	
FN0987	-0.757	13.564	8.981e-2	1.976e-1	17	106	23.2924	128.7589	AAL95183.1| Amidophosphoribosyltransferase
					146	120	146.0000	157.4166	
FN0988	-0.446	16.009	5.721e-2	1.081e-1	116	243	158.9362	295.1736	AAL95184.1| Phosphoribosylamidoimidazole-succinocarboxamide synthase
					281	232	281.0000	304.3388	
FN0989	0.155	14.899	8.33e-2	1.794e-1	121	141	165.7869	171.2736	AAL95185.1| Phosphoribosylaminoimidazole carboxylase catalytic subunit
					203	122	203.0000	160.0402	
FN0990	-0.490	20.379	9.099e-2	2.009e-1	411	1164	563.1275	1413.9180	AAL95186.1| Phosphoribosylformylglycinamidine synthase
					1408	1032	1408.0000	1353.7830	
FN0991	0.655	8.967	1.629e-2	2.067e-2	22	11	30.1431	13.3618	AAL95187.1| CDP-diacylglycerol--serine O-phosphatidyltransferase
					26	17	26.0000	22.3007	
FN0992	-1.170	9.310	1.187e-2	1.391e-2	7	32	9.5910	38.8706	AAL95188.1| ADP-heptose:LPS heptosyltransferase II
					24	28	24.0000	36.7305	
FN0994	-0.521	13.603	1.042e-1	2.392e-1	33	112	45.2146	136.0471	AAL95190.1| Hypothetical protein
					141	100	141.0000	131.1805	
FN0997	0.196	10.477	2.442e-1	7.205e-1	13	30	17.8118	36.4412	AAL95193.1| Hypothetical protein
					63	26	63.0000	34.1069	
FN0998	-0.464	14.207	8.798e-2	1.924e-1	52	132	71.2473	160.3412	AAL95194.1| Dipeptide-binding protein
					163	124	163.0000	162.6639	
FN0999	1.169	10.601	1.08e-1	2.5e-1	14	26	19.1820	31.5824	AAL95195.1| Deblocking aminopeptidase
					99	16	99.0000	20.9889	

Log2 Ratios: -6 -4 -2 0 2 4 6

Show detected proteins only
Show all proteins
Filter by category:
GO: amino acid transport

Proteins found: 1338

Enter (or paste) list of ORFs
Find ORFs

Test	Cutoff
q-Value	.005
p-Value	

	Signif	Direction	Applies To
red	yes	+	ratios, bars
yellow	no	n/a	bars
green	yes	−	ratios, bars
pink	yes	+	*p*- , *q*-Values
light green	yes	−	

Dot Plots Dot Plots

Protein	*FnPg* vs *Fn* Log_2 Ratio	Log_2 Sum	*q*-Value	*p*-Value	Raw *FnPg*	Raw *Fn*	Normalized *FnPg*	Normalized *Fn*	Description
**FN1000**	-1.723	10.632			16	63	21.9222	76.5265	AAL95196.1| Biotin synthase
						52		68.2139	
**FN1001**	0.363	10.024	1.78e-1	4.78e-1	14	22	19.1820	26.7235	AAL95197.1| Dethiobiotin synthetase
					54	23	54.0000	30.1715	
**FN1002**	0.038	12.127	2.916e-1	9.176e-1	69	59	94.5396	71.6677	AAL95198.1| Adenosylmethionine-8-amino -7-oxononanoate aminotransferase
					41	46	41.0000	60.3430	
**FN1003**	2.452	12.779	1.031e-1	2.361e-1	6	32	8.2208	38.8706	AAL95199.1| Outer membrane protein P1 precursor
					384	25	384.0000	32.7951	
**FN1004**	-0.043	8.170	2.818e-1	8.748e-1	12	10	16.4417	12.1471	AAL95200.1| Transcriptional regulator, TetR family
					17	17	17.0000	22.3007	
**FN1005**	-1.383	11.558				78		94.7471	AAL95201.1| Hypothetical protein
					34	63	34.0000	82.6437	
**FN1006**									AAL95202.1| Acetyltransferase
					21		21.0000		
**FN1008**						4		4.8588	AAL95204.1| Hypothetical protein
**FN1009**						8		9.7176	AAL95205.1| Hypothetical protein
						3		3.9354	
**FN1010**	0.010	14.731	3.047e-1	9.77e-1	73	128	100.0202	155.4824	AAL95206.1| Hypothetical cytosolic protein
					231	132	231.0000	173.1583	
**FN1011**	-0.119	7.081	2.358e-1	6.877e-1	9	7	12.3313	8.5029	AAL95207.1| MGPA protein
					10	12	10.0000	15.7417	
**FN1012**	0.540	9.286	1.597e-2	2.014e-2	25	19	34.2535	23.0794	AAL95208.1| HPR(Ser) kinase
					26	14	26.0000	18.3653	
**FN1014**	-0.074	8.992				23		27.9382	AAL95210.1| Folylpolyglutamate synthase
					22	14	22.0000	18.3653	
**FN1015**	0.151	9.565				16		19.4353	AAL95211.1| 5'-methylthioadenosine nucleosidase
					29	25	29.0000	32.7951	
**FN1016**	-0.913	8.474	4.779e-2	8.424e-2	4	21	5.4806	25.5088	AAL95212.1| Lipid A biosynthesis lauroyl acyltransferase
					22	20	22.0000	26.2361	
**FN1017**	-0.810	13.336	2.757e-2	4.055e-2	34	104	46.5848	126.3294	AAL95213.1| Hypothetical Exported Protein
					107	109	107.0000	142.9868	

Log_2 Ratios: -6 -4 -2 0 2 4 6

Show detected proteins only
Show all proteins
Filter by category:
GO: amino acid transport

Proteins found: 1338

Enter (or paste) list of ORFs
Find ORFs

Test	Cutoff
q-Value	.005
p-Value	

	Signif	Direction	Applies To
red	yes	+	ratios, bars
yellow	no	n/a	bars
green	yes	−	ratios, bars
pink	yes	+	*p*- , *q*-Values
light green	yes	−	

Dot Plots Dot Plots

Protein	FnPg vs Fn Log2 Ratio	FnPg vs Fn Log2 Sum	FnPg vs Fn *q*-Value	FnPg vs Fn *p*-Value	Raw *FnPg*	Raw *Fn*	Normalized *FnPg*	Normalized *Fn*	Description
FN1019	1.988	22.716	7.01e-4	2.607e-4	3572	1077	4894.1395	1308.2386	AAL95215.1| 3-hydroxybutyryl-CoA dehydrogenase
					5563	1012	5563.0000	1327.5469	
FN1020	1.393	17.148	8.937e-5	1.135e-5	460	195	630.2643	236.8677	AAL95216.1| 3-hydroxybutyryl-CoA dehydratase
					605	178	605.0000	233.5013	
FN1022						26		31.5824	AAL95218.1| Calcium-transporting ATPase
						45		59.0312	
FN1023									AAL95219.1| 5-Nitroimidazole antibiotic resistance protein
					17		17.0000		
FN1024	-0.259	20.496	5.749e-2	1.089e-1	934	1079	1279.7106	1310.6680	AAL95220.1| DNA-binding protein HU
					943	1029	943.0000	1349.8476	
FN1025	0.257	8.083				14		17.0059	AAL95221.1| Guanine-hypoxanthine permease
					18	10	18.0000	13.1181	
FN1026						14		17.0059	AAL95222.1| tRNA pseudouridine synthase A
						6		7.8708	
FN1028	-0.690	10.293	4.263e-2	7.194e-2	21	46	28.7729	55.8765	AAL95224.1| Deoxyuridine 5'-triphosphate nucleotidohydrolase
					27	26	27.0000	34.1069	
FN1029	-0.677	13.217	6.642e-4	2.383e-4	55	106	75.3577	128.7589	AAL95225.1| Zinc protease
					79	90	79.0000	118.0625	
FN1030	2.719	6.451				3		3.6441	AAL95226.1| Hypothetical membrane-spanning protein
					24		24.0000		
FN1031	0.330	6.840				6		7.2882	AAL95227.1| Hypothetical membrane-spanning protein
					12	9	12.0000	11.8062	
FN1033	-0.493	12.379	2.38e-2	3.371e-2	46	81	63.0264	98.3912	AAL95229.1| Methyltransferase
					60	57	60.0000	74.7729	
FN1037									AAL95233.1| Hypothetical cytosolic protein
					15		15.0000		
FN1041					5		6.8507		AAL95237.1| Acetyltransferase
					16		16.0000		
FN1042	-2.915	10.932	2.608e-4	5.805e-5	14	107	19.1820	129.9736	AAL95238.1| S1 RNA binding domain
					13	86	13.0000	112.8153	
FN1045						7		8.5029	AAL95241.1| Hypothetical protein
						4		5.2472	

Log2 Ratios: -6 -4 -2 0 2 4 6

Show detected proteins only
Show all proteins
Filter by category:
GO: amino acid transport

Proteins found: 1338

Enter (or paste) list of ORFs

Find ORFs

Test	Cutoff
q-Value	.005
p-Value	

	Signif	Direction	Applies To
red	yes	+	ratios, bars
yellow	no	n/a	bars
green	yes	−	ratios, bars
pink	yes	+	*p*-, *q*-Values
light green	yes	−	

Dot Plots Dot Plots

Protein	FnPg vs Fn Log2 Ratio	Log2 Sum	q-Value	p-Value	Raw FnPg	Raw Fn	Normalized FnPg	Normalized Fn	Description
FN1050	0.963	7.139			22	7	30.1431	8.5029	AAL95246.1| Lactoylglutathione lyase
					3		3.0000		
FN1053					8		10.9611		AAL95249.1| Hypothetical protein
FN1055	0.210	11.779	1.959e-1	5.433e-1	31	40	42.4743	48.5882	AAL95251.1| Cysteine synthase
					85	47	85.0000	61.6548	
FN1057						8		9.7176	AAL95253.1| Diamine acetyltransferase
						6		7.8708	
FN1060	0.259	9.702	2.678e-2	3.909e-2	22	24	30.1431	29.1529	AAL95256.1| hypothetical cytosolic protein
					33	18	33.0000	23.6125	
FN1062	-0.585	12.657	2.795e-2	4.127e-2	60	80	82.2084	97.1765	AAL95258.1| Hydrolase
					49	76	49.0000	99.6972	
FN1063	1.195	8.197	1.172e-1	2.768e-1	5	10	6.8507	12.1471	AAL95259.1| N-acyl-L-amino acid amidohydrolase
					45	8	45.0000	10.4944	
FN1066	-0.017	6.272	3.01e-1	9.601e-1	4	7	5.4806	8.5029	AAL95262.1| Exodeoxyribonuclease VII large subunit
					12	7	12.0000	9.1826	
FN1067	-1.650	11.075	8.144e-4	3.35e-4	12	75	16.4417	91.1030	AAL95263.1| Tetratricopeptide repeat family protein
					36	56	36.0000	73.4611	
FN1068					3		4.1104		AAL95264.1| Smf protein
					16		16.0000		
FN1069	-0.652	12.442	4.102e-4	1.116e-4	46	73	63.0264	88.6736	AAL95265.1| DNA topoisomerase I
					56	75	56.0000	98.3854	
FN1070	-1.251	7.591				18		21.8647	AAL95266.1| Glucose inhibited division protein A
					9	16	9.0000	20.9889	
FN1071									AAL95267.1| Integrase/recombinase
						4		5.2472	
FN1072	-0.740	11.295	5.045e-3	4.61e-3	34	57	46.5848	69.2383	AAL95268.1| GTP-binding protein
					31	46	31.0000	60.3430	
FN1073	-1.142	6.312				11		13.3618	AAL95269.1| Hypothetical protein
					6	10	6.0000	13.1181	
FN1074	-0.644	9.742	1.966e-2	2.653e-2	13	30	17.8118	36.4412	AAL95270.1| Signal recognition particle receptor FtsY
					29	28	29.0000	36.7305	

Log2 Ratios: -6 -4 -2 0 2 4 6

Show detected proteins only
Show all proteins
Filter by category: GO: amino acid transport

Proteins found: 1338

Enter (or paste) list of ORFs

Find ORFs

Test	Cutoff
q-Value	.005
p-Value	

Signif	Direction	Applies To
yes	+	ratios, bars
no	n/a	bars
yes	−	ratios, bars
yes	+	p-, q-Values
yes	−	

Dot Plots Dot Plots

Protein	FnPg vs Fn Log$_2$ Ratio	Log$_2$ Sum	q-Value	p-Value	Raw FnPg	Raw Fn	Normalized FnPg	Normalized Fn	Description
FN1077	2.041	8.217				7		8.5029	AAL95273.1| Hypothetical protein
					35		35.0000		
FN1078	0.316	15.414	5.335e-2	9.794e-2	195	142	267.1773	172.4883	AAL95274.1| Hypothetical exported 24-amino acid repeat protein
					199	154	199.0000	202.0180	
FN1079	0.523	18.003	1.197e-1	2.843e-1	267	364	365.8273	442.1531	AAL95275.1| Neutrophil-activating protein A
					863	315	863.0000	413.2187	
FN1080	-0.972	4.632	3.24e-3	2.504e-3	3	5	4.1104	6.0735	AAL95276.1| Export ABC transporter
					3	6	3.0000	7.8708	
FN1081	-0.113	10.309			25	34	34.2535	41.3000	AAL95277.1| unknown
						25		32.7951	
FN1084	-1.661	11.727	1.27e-3	6.878e-4	31	97	42.4743	117.8265	AAL95280.1| unknown
					23	68	23.0000	89.2028	
FN1085	1.380	12.544	3.034e-3	2.278e-3	101	40	138.3841	48.5882	AAL95281.1| 4-methyl-5(B-hydroxyethyl)-thiazole monophosphate biosynthesis enzyme
					111	36	111.0000	47.2250	
FN1086	-0.345	5.960				6		7.2882	AAL95282.1| Transporter
					7	8	7.0000	10.4944	
FN1088	-0.324	9.206	8.026e-2	1.707e-1	12	21	16.4417	25.5088	AAL95284.1| NADH oxidase
					27	22	27.0000	28.8597	
FN1089	2.082	12.161	1.92e-2	2.572e-2	69	25	94.5396	30.3677	AAL95285.1| ATP-binding protein (contains P-loop)
					184	27	184.0000	35.4187	
FN1091	-1.787	7.371	8.297e-4	3.461e-4	5	21	6.8507	25.5088	AAL95287.1| Sigma factor sigB regulation protein rsbU
					7	17	7.0000	22.3007	
FN1092	0.197	10.888	2.661e-1	8.086e-1	6	27	8.2208	32.7971	AAL95288.1| Hypothetical protein
					85	37	85.0000	48.5368	
FN1093	-1.444	11.607	7.89e-4	3.171e-4	29	75	39.7341	91.1030	AAL95289.1| Hypothetical protein
					28	71	28.0000	93.1382	
FN1094									AAL95290.1| Dolichol-phosphate mannosyltransferase
					33		33.0000		
FN1095					8		10.9611		AAL95291.1| unknown
FN1096	-0.169	10.967	2.421e-1	7.121e-1	47	36	64.3966	43.7294	AAL95292.1| Hypothetical protein
					20	39	20.0000	51.1604	

Log$_2$ Ratios: -6 -4 -2 0 2 4 6

Show detected proteins only
Show all proteins
Filter by category:
GO: amino acid transport

Proteins found: 1338

Enter (or paste) list of ORFs

Find ORFs

Test	Cutoff
q-Value	.005
p-Value	

	Signif	Direction	Applies To
red	yes	+	ratios, bars
yellow	no	n/a	bars
green	yes	−	ratios, bars
pink	yes	+	p- , q-Values
light green	yes	−	p- , q-Values

Dot Plots Dot Plots

Protein	FnPg vs Fn: Log_2 Ratio	Log_2 Sum	*q*-Value	*p*-Value	Raw *FnPg*	Raw *Fn*	Normalized *FnPg*	Normalized *Fn*	Description
FN1097	-1.064	11.373			26	47	35.6236	57.0912	AAL95293.1| Hypothetical protein
						70		91.8264	
FN1101						5		6.0735	AAL95297.1| ATPase
FN1102						28		34.0118	AAL95298.1| tRNA 2'phosphotransferase
						32		41.9778	
FN1103	-1.735	11.758	2.231e-4	4.514e-5	23	85	31.5132	103.2500	AAL95299.1| Excinuclease ABC subunit A
					33	85	33.0000	111.5034	
FN1105	-0.290	13.052	1.489e-1	3.76e-1	83	76	113.7216	92.3177	AAL95301.1| Hypothetical protein
					53	85	53.0000	111.5034	
FN1106	-0.657	12.631	1.085e-3	5.357e-4	51	88	69.8771	106.8941	AAL95302.1| L-serine dehydratase
					57	71	57.0000	93.1382	
FN1111									AAL95307.1| Dipeptide-binding protein
						8		10.4944	
FN1117	-0.613	10.930	8.015e-2	1.704e-1	39	37	53.4355	44.9441	AAL95313.1| LSU ribosomal protein L21P
					18	49	18.0000	64.2785	
FN1119	-0.859	13.803	3.573e-2	5.682e-2	96	142	131.5334	172.4883	AAL95315.1| LSU ribosomal protein L27P
					46	114	46.0000	149.5458	
FN1120	0.799	15.297	6.978e-3	7.186e-3	166	109	227.4432	132.4030	AAL95316.1| Phosphoenolpyruvate carboxykinase (ATP)
					302	131	302.0000	171.8465	
FN1121	-0.372	12.103	4.579e-2	7.952e-2	34	67	46.5848	81.3853	AAL95317.1| hypothetical cytosolic protein
					70	53	70.0000	69.5257	
FN1122	-2.018	13.763	2.365e-3	1.621e-3	41	219	56.1757	266.0207	AAL95318.1| Long-chain-fatty-acid--CoA ligase
					61	159	61.0000	208.5770	
FN1123	-2.046	7.413	5.593e-5	5.847e-6	5	21	6.8507	25.5088	AAL95319.1| Thioredoxin-like protein
					6	21	6.0000	27.5479	
FN1124	-1.027	16.457	1.293e-3	7.057e-4	133	356	182.2286	432.4354	AAL95320.1| Outer membrane porin F
					238	323	238.0000	423.7131	
FN1125	0.845	11.386	1.311e-1	3.194e-1	18	29	24.6625	35.2265	AAL95321.1| LemA protein
					114	32	114.0000	41.9778	
FN1127	1.087	11.860	2.91e-2	4.344e-2	83	30	113.7216	36.4412	AAL95323.1| Hypothetical membrane-spanning protein
					64	36	64.0000	47.2250	

Log_2 Ratios: -6 -4 -2 0 2 4 6

Show detected proteins only
Show all proteins
Filter by category:
GO: amino acid transport

Proteins found: 1338

Enter (or paste) list of ORFs

Find ORFs

Test	Cutoff
q-Value	.005
p-Value	

	Signif	Direction	Applies To
red	yes	+	ratios, bars
yellow	no	n/a	bars
green	yes	−	ratios, bars
pink	yes	+	*p*- , *q*-Values
light green	yes	−	*p*- , *q*-Values

Dot Plots Dot Plots

Protein	FnPg vs Fn Log_2 Ratio	Log_2 Sum	*q*-Value	*p*-Value	Raw *FnPg*	Raw *Fn*	Normalized *FnPg*	Normalized *Fn*	Description
**FN1128**	0.334	16.075	1.092e-1	2.535e-1	270	193	369.9378	234.4383	AAL95324.1| Acylamino-acid-releasing enzyme
					220	178	220.0000	233.5013	
**FN1129**	-1.843	7.013				16		19.4353	AAL95325.1| Chromosome partition protein smc
					6	18	6.0000	23.6125	
**FN1130**	-1.596	6.239				13		15.7912	AAL95326.1| Tetraacyldisaccharide 4'-kinase
					5	11	5.0000	14.4299	
**FN1131**	0.502	6.899							AAL95327.1| Hypothetical protein
					13	7	13.0000	9.1826	
**FN1133**	0.535	10.186	1.525e-1	3.881e-1	14	24	19.1820	29.1529	AAL95329.1| N-acetylglucosamine-6-phosphate deacetylase
					63	21	63.0000	27.5479	
**FN1134**	-0.750	10.293	1.882e-2	2.506e-2	26	40	35.6236	48.5882	AAL95330.1| Hypothetical cytosolic protein
					19	33	19.0000	43.2896	
**FN1135**	0.232	15.087	2.28e-1	6.583e-1	225	142	308.2815	172.4883	AAL95331.1| Phosphonates-binding protein
					96	131	96.0000	171.8465	
**FN1136**	1.345	7.825				8		9.7176	AAL95332.1| Phosphonates transport ATP-binding protein phnC
					24	7	24.0000	9.1826	
**FN1138**	-0.268	18.871	1.05e-1	2.414e-1	349	644	478.1788	782.2708	AAL95334.1| Hypothetical cytosolic protein
					784	562	784.0000	737.2346	
**FN1139**	0.086	12.492	2.847e-1	8.874e-1	20	63	27.4028	76.5265	AAL95335.1| Activator of (R)-2-hydroxyglutaryl-CoA dehydratase
					129	54	129.0000	70.8375	
**FN1140**	1.396	7.892				7		8.5029	AAL95336.1| hypothetical protein
					25	8	25.0000	10.4944	
**FN1142**	-1.475	6.119				11		13.3618	AAL95338.1| Oxygen-independent coproporphyrinogen III oxidase
					5	11	5.0000	14.4299	
**FN1143**	0.301	13.417	2.046e-1	5.737e-1	41	72	56.1757	87.4588	AAL95339.1| Glucosamine-6-phosphate isomerase
					176	77	176.0000	101.0090	
**FN1144**	-2.024	13.841	1.351e-3	7.472e-4	22	210	30.1431	255.0883	AAL95340.1| Hypothetical Exported Protein
					90	178	90.0000	233.5013	
**FN1145**	-0.160	8.872	2.423e-1	7.13e-1	8	15	10.9611	18.2206	AAL95341.1| Oligoendopeptidase F
					30	21	30.0000	27.5479	
**FN1146**	-1.752	7.831			6	24	8.2208	29.1529	AAL95342.1| Hypothetical exported 24-amino acid repeat protein
						20		26.2361	

Log_2 Ratios: -6 -4 -2 0 2 4 6

Show detected proteins only
Show all proteins
Filter by category:
GO: amino acid transport

Proteins found: 1338

Enter (or paste) list of ORFs

Find ORFs

Test	Cutoff
q-Value	.005
p-Value	

	Signif	Direction	Applies To
red	yes	+	ratios, bars
yellow	no	n/a	bars
green	yes	–	ratios, bars
pink	yes	+	*p*- , *q*-Values
light green	yes	–	*p*- , *q*-Values

Dot Plots Dot Plots

Protein	FnPg vs Fn Log$_2$ Ratio	Log$_2$ Sum	*q*-Value	*p*-Value	Raw *FnPg*	Raw *Fn*	Normalized *FnPg*	Normalized *Fn*	Description
FN1147						20		24.2941	AAL95343.1| Hypothetical protein
						17		22.3007	
FN1148	0.121	11.123	2.843e-1	8.857e-1	4	40	5.4806	48.5882	AAL95344.1| Serine/threonine sodium symporter
					93	32	93.0000	41.9778	
FN1149	-0.897	7.170	1.237e-4	1.925e-5	7	14	9.5910	17.0059	AAL95345.1| ATP-dependent nuclease subunit A
					8	12	8.0000	15.7417	
FN1150	-1.199	5.199							AAL95346.1| unknown
					4	7	4.0000	9.1826	
FN1152	-0.607	13.861	5.176e-2	9.389e-2	45	128	61.6563	155.4824	AAL95348.1| Aspartate aminotransferase
					136	111	136.0000	145.6104	
FN1153						36		43.7294	AAL95349.1| Hypothetical protein
						26		34.1069	
FN1154	1.123	7.521							AAL95350.1| Ribonuclease BN
					20	7	20.0000	9.1826	
FN1155						7		8.5029	AAL95351.1| Cell division protein ftsI
						11		14.4299	
FN1157						7		8.5029	AAL95353.1| Polypeptide deformylase
						7		9.1826	
FN1159	0.507	13.609	4.817e-2	8.513e-2	77	68	105.5008	82.6000	AAL95355.1| Fructose-1,6-bisphosphatase
					161	80	161.0000	104.9444	
FN1160	-1.457	4.627				6		7.2882	AAL95356.1| SWF/SNF family helicase
					3	7	3.0000	9.1826	
FN1161	-2.264	5.434				14		17.0059	AAL95357.1| Glutamate racemase
					3	9	3.0000	11.8062	
FN1162	1.679	7.106							AAL95358.1| Hydroxyacylglutathione hydrolase
					21	5	21.0000	6.5590	
FN1163	0.016	11.964	3.015e-1	9.621e-1	30	56	41.1042	68.0235	AAL95359.1| Thioredoxin reductase
					86	44	86.0000	57.7194	
FN1164	-1.042	7.626	4.611e-5	4.151e-6	7	17	9.5910	20.6500	AAL95360.1| Glucokinase
					10	15	10.0000	19.6771	
FN1165	-0.474	23.494	3.39e-4	8.379e-5	2224	3391	3047.1910	4119.0688	AAL95361.1| D-galactose-binding protein
					2786	3036	2786.0000	3982.6408	

Log$_2$ Ratios: -6 -4 -2 0 2 4 6

Show detected proteins only
Show all proteins
Filter by category:
GO: amino acid transport

Proteins found: 1338

Enter (or paste) list of ORFs

Find ORFs

Test	Cutoff
q-Value	.005
p-Value	

	Signif	Direction	Applies To
red	yes	+	ratios, bars
yellow	no	n/a	bars
green	yes	−	ratios, bars
pink	yes	+	*p*- , *q*-Values
light green	yes	−	

Dot Plots Dot Plots

Protein	FnPg vs Fn: Log_2 Ratio	Log_2 Sum	*q*-Value	*p*-Value	Raw *FnPg*	Raw *Fn*	Normalized *FnPg*	Normalized *Fn*	Description
**FN1166**	-0.484	15.297	6.935e-3	7.123e-3	128	179	175.3779	217.4324	AAL95362.1| Galactoside transport ATP-binding protein mglA
					164	196	164.0000	257.1138	
**FN1167**						52		63.1647	AAL95363.1| Galactoside transport system permease protein mglC
						17		22.3007	
**FN1169**	-0.356	16.815	4.448e-2	7.633e-2	260	330	356.2364	400.8531	AAL95365.1| L-lactate dehydrogenase
					244	280	244.0000	367.3055	
**FN1170**	0.506	25.229	7.054e-2	1.441e-1	6878	4375	9423.8219	5314.3397	AAL95366.1| Pyruvate-flavodoxin oxidoreductase
					5525	3972	5525.0000	5210.4906	
**FN1171**	0.209	19.881	1.436e-1	3.589e-1	936	793	1282.4509	963.2620	AAL95367.1| Acetate kinase
					830	659	830.0000	864.4797	
**FN1172**	-0.630	18.578	1.192e-4	1.8e-5	348	627	476.8087	761.6208	AAL95368.1| Phosphate acetyltransferase
					529	606	529.0000	794.9540	
**FN1176**					4		5.4806		AAL95372.1| Hypothetical cytosolic protein
**FN1179**	1.870	6.653			14		19.1820		AAL95375.1| ATP-dependent RNA helicase
						4		5.2472	
**FN1180**	0.361	7.191			10	10	13.7014	12.1471	AAL95376.1| Hypothetical protein
						7		9.1826	
**FN1181**	1.353	15.098	1.258e-1	3.03e-1	404	99	553.5365	120.2559	AAL95377.1| unknown
					45	87	45.0000	114.1271	
**FN1182**	0.348	7.204			10	8	13.7014	9.7176	AAL95378.1| Hypothetical protein
						9		11.8062	
**FN1183**	1.050	6.875	1.941e-2	2.608e-2	14	7	19.1820	8.5029	AAL95379.1| Hypothetical cytosolic protein
					12	5	12.0000	6.5590	
**FN1184**					4		5.4806		AAL95380.1| Hypothetical protein
**FN1185**	0.578	10.599	1.313e-1	3.2e-1	52	25	71.2473	30.3677	AAL95381.1| SIR2 family protein
					25	26	25.0000	34.1069	
**FN1186**	4.221	12.526	4.992e-2	8.934e-2	82	12	112.3515	14.5765	AAL95382.1| Amidohydrolase
					551	16	551.0000	20.9889	
**FN1187**					7		9.5910		AAL95383.1| Amino acid-binding protein
					93		93.0000		

Log_2 Ratios: -6 -4 -2 0 2 4 6

Show detected proteins only
Show all proteins
Filter by category:
GO: amino acid transport

Proteins found: 1338

Enter (or paste) list of ORFs

Find ORFs

Test	Cutoff
q-Value	.005
p-Value	

	Signif	Direction	Applies To
red	yes	+	ratios, bars
yellow	no	n/a	bars
green	yes	−	ratios, bars
pink	yes	+	*p*- , *q*-Values
light green	yes	−	

Dot Plots Dot Plots

Protein	FnPg vs Fn Log$_2$ Ratio	Log$_2$ Sum	*q*-Value	*p*-Value	Raw *FnPg*	Raw *Fn*	Normalized *FnPg*	Normalized *Fn*	Description
FN1188	-0.936	11.845	5.604e-2	1.05e-1	10	69	13.7014	83.8147	AAL95384.1| Hypothetical protein
					74	64	74.0000	83.9555	
FN1189	-0.482	12.482	1.891e-3	1.186e-3	46	77	63.0264	93.5324	AAL95385.1| Hypothetical protein
					65	65	65.0000	85.2673	
FN1190	-0.937	13.526	2.184e-5	1.409e-6	54	121	73.9876	146.9795	AAL95386.1| Probable cadmium-transporting ATPase
					83	117	83.0000	153.4812	
FN1191	0.044	10.940				33		40.0853	AAL95387.1| unknown
					45	36	45.0000	47.2250	
FN1192	0.086	16.985	2.075e-1	5.839e-1	227	298	311.0217	361.9825	AAL95388.1| unknown
					431	257	431.0000	337.1340	
FN1198	0.382	9.619	6.09e-2	1.185e-1	19	21	26.0327	25.5088	AAL95394.1| Transporter
					38	18	38.0000	23.6125	
FN1200									AAL95396.1| Hypothetical protein
					456		456.0000		
FN1202	1.492	8.053			18	8	24.6625	9.7176	AAL95398.1| NH(3)-dependent NAD(+) synthetase
					30		30.0000		
FN1203	-0.717	6.710			8		10.9611		AAL95399.1| GTP-binding protein
					5	10	5.0000	13.1181	
FN1204	0.388	7.090	7.563e-2	1.577e-1	10	6	13.7014	7.2882	AAL95400.1| Methyltransferase
					13	10	13.0000	13.1181	
FN1205	-0.683	12.230	6.338e-2	1.254e-1	20	69	27.4028	83.8147	AAL95401.1| Protease
					82	70	82.0000	91.8264	
FN1206									AAL95402.1| Hemolysin
						8		10.4944	
FN1208	-0.395	6.435	3.627e-2	5.794e-2	6	10	8.2208	12.1471	AAL95404.1| 1-deoxyxylulose-5-phosphate synthase
					8	7	8.0000	9.1826	
FN1209	-2.359	10.173				77		93.5324	AAL95405.1| Hypothetical RNA binding protein
					15	46	15.0000	60.3430	
FN1210	-1.499	11.414	1.487e-2	1.838e-2	22	55	30.1431	66.8088	AAL95406.1| Metal dependent hydrolase
					32	83	32.0000	108.8798	
FN1211	-1.699	7.067	5.425e-4	1.742e-4	5	16	6.8507	19.4353	AAL95407.1| Cell division protein ftsI
					6	17	6.0000	22.3007	

Log$_2$ Ratios: -6 -4 -2 0 2 4 6

Show detected proteins only
Show all proteins
Filter by category:
GO: amino acid transport

Proteins found: 1338

Enter (or paste) list of ORFs

Find ORFs

Test	Cutoff
q-Value	.005
p-Value	

	Signif	Direction	Applies To
red	yes	+	ratios, bars
yellow	no	n/a	bars
green	yes	−	ratios, bars
pink	yes	+	*p*- , *q*-Values
light green	yes	−	

Dot Plots Dot Plots

Protein	FnPg vs Fn Log$_2$ Ratio	FnPg vs Fn Log$_2$ Sum	FnPg vs Fn *q*-Value	FnPg vs Fn *p*-Value	Raw FnPg	Raw Fn	Normalized FnPg	Normalized Fn	Description	Log$_2$ Ratios (-6 -4 -2 0 2 4 6)
FN1213	-1.264	11.300	7.2e-3	7.517e-3	13	59	17.8118	71.6677	AAL95409.1| Hypothetical protein	
					47	64	47.0000	83.9555		
FN1216	-1.368	8.377	1.593e-2	2.007e-2	10	18	13.7014	21.8647	AAL95412.1| RRF2 family protein	
					9	28	9.0000	36.7305		
FN1217	-1.747	7.361				16		19.4353	AAL95413.1| Holliday junction DNA helicase ruvB	
					7	21	7.0000	27.5479		
FN1218									AAL95414.1| unknown	
					13		13.0000			
FN1219	0.350	7.953	5.396e-2	9.952e-2	15	10	20.5521	12.1471	AAL95415.1| Hypothetical protein	
					15	12	15.0000	15.7417		
FN1220	-0.155	15.303	5.815e-2	1.107e-1	127	183	174.0078	222.2912	AAL95416.1| Cysteine synthase	
					207	154	207.0000	202.0180		
FN1221	0.131	8.428	2.718e-1	8.321e-1	5	13	6.8507	15.7912	AAL95417.1| Hypothetical protein	
					32	15	32.0000	19.6771		
FN1222	-1.403	8.987	3.455e-4	8.614e-5	10	29	13.7014	35.2265	AAL95418.1| Hypothetical protein	
					14	29	14.0000	38.0424		
FN1223	0.428	12.381	5.721e-2	1.081e-1	74	54	101.3903	65.5941	AAL95419.1| Oxygen-insensitive NAD(P)H nitroreductase	
					68	46	68.0000	60.3430		
FN1224	-0.623	13.846	5.546e-2	1.034e-1	42	113	57.5459	137.2618	AAL95420.1| 2-dehydro-3-deoxyphosphooctonate aldolase	
					138	125	138.0000	163.9757		
FN1225	-0.240	13.494	1.81e-1	4.893e-1	45	82	61.6563	99.6059	AAL95421.1| UDP-N-acetylmuramoyl-L-alanyl-D-glutamate--meso-lanthionine ligase	
					136	102	136.0000	133.8041		
FN1226	-1.125	13.443	2.289e-2	3.208e-2	24	127	32.8834	154.2677	AAL95422.1| Uracil-DNA glycosylase	
					110	120	110.0000	157.4166		
FN1229						5		6.0735	AAL95425.1| Hypothetical protein	
						7		9.1826		
FN1230						50		60.7353	AAL95426.1| Hypothetical cytosolic protein	
						43		56.4076		
FN1231	0.451	18.536	1.967e-1	5.46e-1	166	431	227.4432	523.5384	AAL95427.1| Inosine-5'-monophosphate dehydrogenase	
					1214	405	1214.0000	531.2811		
FN1233						3		3.6441	AAL95429.1| Putative NAD(P)H oxidoreductase	
						4		5.2472		

Show detected proteins only
Show all proteins
Filter by category:
GO: amino acid transport

Proteins found: 1338

Enter (or paste) list of ORFs

Find ORFs

Test	Cutoff
q-Value	.005
p-Value	

Color	Signif	Direction	Applies To
red	yes	+	ratios, bars
yellow	no	n/a	bars
green	yes	–	ratios, bars
pink	yes	+	*p*- , *q*-Values
light green	yes	–	*p*- , *q*-Values

Dot Plots Dot Plots

Protein	FnPg vs Fn Log_2 Ratio	Log_2 Sum	*q*-Value	*p*-Value	Raw *FnPg*	Raw *Fn*	Normalized *FnPg*	Normalized *Fn*	Description	Log_2 Ratios (-6 -4 -2 0 2 4 6)
FN1234						15		18.2206	AAL95430.1| Hypothetical protein	
						13		17.0535		
FN1235	1.403	7.579			8	7	10.9611	8.5029	AAL95431.1| Ankyrin repeat proteins	
					34		34.0000			
FN1237	1.575	10.956	1.626e-3	9.569e-4	51	22	69.8771	26.7235	AAL95433.1| Choline kinase	
					84	19	84.0000	24.9243		
FN1238									AAL95434.1| Hypothetical protein	
					5		5.0000			
FN1239									AAL95435.1| Hypothetical protein	
					12		12.0000			
FN1240	-1.829	8.757	4.347e-4	1.226e-4	11	30	15.0715	36.4412	AAL95436.1| Lipopolysaccharide core biosynthesis protein rfaY	
					7	32	7.0000	41.9778		
FN1241	-0.139	5.668	2.521e-1	7.514e-1	7	8	9.5910	9.7176	AAL95437.1| polysaccharide biosynthesis protein	
					4	4	4.0000	5.2472		
FN1242	0.657	7.683				8		9.7176	AAL95438.1| Polysaccharide deacetylase	
					18	10	18.0000	13.1181		
FN1243	0.021	7.594				13		15.7912	AAL95439.1| Glycosyl transferase	
					14	9	14.0000	11.8062		
FN1244	0.587	8.322	1.743e-1	4.643e-1	5	10	6.8507	12.1471	AAL95440.1| Polysaccharide deacetylase	
					37	13	37.0000	17.0535		
FN1245	-2.175	6.550	1.589e-2	2.001e-2	3	22	4.1104	26.7235	AAL95441.1| Glycosyl transferase	
					5	11	5.0000	14.4299		
FN1246	-0.372	8.186				19		23.0794	AAL95442.1| Lipooligosaccharide cholinephosphotransferase	
					15	12	15.0000	15.7417		
FN1247	-0.619	8.705	3.85e-2	6.267e-2	8	19	10.9611	23.0794	AAL95443.1| LOS biosynthesis enzyme LBGB	
					22	21	22.0000	27.5479		
FN1248						5		6.0735	AAL95444.1| Hypothetical cytosolic protein	
FN1250	-1.376	9.925	1.084e-3	5.351e-4	10	46	13.7014	55.8765	AAL95446.1| Guanine-hypoxanthine permease	
					25	34	25.0000	44.6014		
FN1251	-0.530	6.145				8		9.7176	AAL95447.1| High-affinity iron permease	
					7	8	7.0000	10.4944		

Show detected proteins only
Show all proteins
Filter by category:
GO: amino acid transport

Proteins found: 1338

Enter (or paste) list of ORFs

Find ORFs

Test	Cutoff
q-Value	.005
p-Value	

	Signif	Direction	Applies To
red	yes	+	ratios, bars
yellow	no	n/a	bars
green	yes	–	ratios, bars
pink	yes	+	*p*- , *q*-Values
light green	yes	–	

Dot Plots Dot Plots

Protein	FnPg vs Fn Log$_2$ Ratio	Log$_2$ Sum	*q*-Value	*p*-Value	Raw *FnPg*	Raw *Fn*	Normalized *FnPg*	Normalized *Fn*	Description
FN1252	-1.973	17.489	1.155e-3	5.951e-4	151	638	206.8911	774.9826	AAL95448.1| 34 kDa membrane antigen precursor
					226	705	226.0000	924.8227	
FN1253	-0.833	16.061	6.623e-2	1.328e-1	48	268	65.7667	325.5413	AAL95449.1| unknown
					326	284	326.0000	372.5527	
FN1254	-0.817	12.908	2.362e-2	3.338e-2	30	103	41.1042	125.1147	AAL95450.1| Oxygen-insensitive NAD(P)H nitroreductase
					91	82	91.0000	107.5680	
FN1255						15		18.2206	AAL95451.1| NagD protein
						14		18.3653	
FN1256	-0.603	4.603				5		6.0735	AAL95452.1| C4-dicarboxylate transporter large subunit
					4		4.0000		
FN1258	-0.787	19.783	2.008e-3	1.297e-3	595	1049	815.2332	1274.2268	AAL95454.1| C4-dicarboxylate-binding protein
					631	931	631.0000	1221.2907	
FN1260						4		4.8588	AAL95456.1| Sensory Transduction Protein Kinase
FN1261	1.385	6.168			10		13.7014		AAL95457.1| Two-component response regulator
						4		5.2472	
FN1262	1.719	6.924				5		6.0735	AAL95458.1| Integral membrane protein
					20		20.0000		
FN1263	-0.934	8.230	4.534e-3	3.968e-3	11	20	15.0715	24.2941	AAL95459.1| Cobalt chelatase
					10	18	10.0000	23.6125	
FN1264						25		30.3677	AAL95460.1| Hypothetical protein
						16		20.9889	
FN1265	0.033	13.133	2.501e-1	7.434e-1	64	83	87.6890	100.8206	AAL95461.1| Outer membrane protein
					104	66	104.0000	86.5791	
FN1266	-0.752	14.116	4.763e-3	4.265e-3	63	140	86.3188	170.0589	AAL95462.1| UTP--glucose-1-phosphate uridylyltransferase
					119	134	119.0000	175.7819	
FN1267	0.132	10.711	1.759e-1	4.702e-1	37	32	50.6952	38.8706	AAL95463.1| Hypothetical protein
					35	30	35.0000	39.3542	
FN1268	-0.507	15.890	1.852e-2	2.451e-2	155	272	212.3717	330.4001	AAL95464.1| Methionyl-tRNA synthetase
					201	196	201.0000	257.1138	
FN1269	-2.042	6.042				12		14.5765	AAL95465.1| Hypothetical lipoprotein
					4	14	4.0000	18.3653	

Log$_2$ Ratios: -6 -4 -2 0 2 4 6

Show detected proteins only
Show all proteins
Filter by category:
GO: amino acid transport

Proteins found: 1338

Enter (or paste) list of ORFs

Find ORFs

Test	Cutoff
q-Value	.005
p-Value	

	Signif	Direction	Applies To
red	yes	+	ratios, bars
yellow	no	n/a	bars
green	yes	−	ratios, bars
pink	yes	+	*p*- , *q*-Values
light green	yes	−	

Dot Plots Dot Plots

Protein	FnPg vs Fn Log2 Ratio	Log2 Sum	q-Value	p-Value	Raw FnPg	Raw Fn	Normalized FnPg	Normalized Fn	Description
FN1270	-2.654	11.438				115		139.6912	AAL95466.1| Hypothetical cytosolic protein
					21	95	21.0000	124.6215	
FN1271	-0.989	10.819	3.867e-2	6.304e-2	9	50	12.3313	60.7353	AAL95467.1| Protease IV
					48	45	48.0000	59.0312	
FN1273	0.174	10.980	2.748e-1	8.449e-1	4	47	5.4806	57.0912	AAL95469.1| Outer membrane protein tolC
					90	21	90.0000	27.5479	
FN1274	-2.178	9.111	8.321e-4	3.478e-4	3	37	4.1104	44.9441	AAL95470.1| Acriflavin resistance protein E
					18	42	18.0000	55.0958	
FN1275	-1.966	9.836	2.602e-4	5.783e-5	7	53	9.5910	64.3794	AAL95471.1| Acriflavin resistance protein B
					21	42	21.0000	55.0958	
FN1276					13		17.8118		AAL95472.1| Hypothetical protein
					18		18.0000		
FN1277	0.370	15.287	1.416e-1	3.524e-1	107	133	146.6050	161.5559	AAL95473.1| Aminoacyl-histidine dipeptidase
					308	145	308.0000	190.2118	
FN1278									AAL95474.1| Acetyltransferase
					3		3.0000		
FN1279	-0.542	12.225	3.008e-3	2.25e-3	37	64	50.6952	77.7412	AAL95475.1| Zinc metallohydrolase, glyoxalase II family
					64	68	64.0000	89.2028	
FN1280	2.279	9.432	9.521e-5	1.248e-5	40	11	54.8056	13.3618	AAL95476.1| Serine protease, V8 family
					61	8	61.0000	10.4944	
FN1281	-0.305	8.518	8.875e-2	1.946e-1	12	21	16.4417	25.5088	AAL95477.1| Cysteine protease
					18	13	18.0000	17.0535	
FN1282	0.471	12.375	8.354e-2	1.801e-1	80	49	109.6112	59.5206	AAL95478.1| LSU ribosomal protein L17P
					62	49	62.0000	64.2785	
FN1283	-0.083	17.505	1.086e-1	2.515e-1	287	383	393.2301	465.2325	AAL95479.1| DNA-directed RNA polymerase alpha chain
					445	322	445.0000	422.4013	
FN1284	-0.803	18.736	3.716e-2	5.98e-2	525	705	719.3234	856.3679	AAL95480.1| SSU ribosomal protein S4P
					281	678	281.0000	889.4040	
FN1285	1.408	14.708	1.326e-1	3.242e-1	373	80	511.0622	97.1765	AAL95481.1| SSU ribosomal protein S11P
					22	79	22.0000	103.6326	
FN1286	-2.940	14.401	1.158e-4	1.712e-5	41	323	56.1757	392.3501	AAL95482.1| SSU ribosomal protein S13P
					50	322	50.0000	422.4013	

Log2 Ratios: -6 -4 -2 0 2 4 6

Show detected proteins only
Show all proteins
Filter by category:
GO: amino acid transport

Proteins found: 1338

Enter (or paste) list of ORFs
Find ORFs

Test	Cutoff
q-Value	.005
p-Value	

	Signif	Direction	Applies To
red	yes	+	ratios, bars
yellow	no	n/a	bars
green	yes	−	ratios, bars
pink	yes	+	p-, q-Values
light green	yes	−	

Dot Plots Dot Plots

Protein	*FnPg* vs *Fn* Log$_2$ Ratio	Log$_2$ Sum	*q*-Value	*p*-Value	Raw *FnPg*	Raw *Fn*	Normalized *FnPg*	Normalized *Fn*	Description
**FN1287**	-1.021	9.866	2.957e-2	4.434e-2	24	36	32.8834	43.7294	AAL95483.1| Bacterial Protein Translation Initiation Factor 1 (IF-1)
					10	33	10.0000	43.2896	
**FN1290**	0.645	10.431	1.852e-1	5.055e-1	62	23	84.9487	27.9382	AAL95486.1| Hypothetical protein
					8	24	8.0000	31.4833	
**FN1293**						6		7.2882	AAL95489.1| Hypothetical protein
						8		10.4944	
**FN1296**						29		35.2265	AAL95492.1| unknown
						16		20.9889	
**FN1297**	-0.841	9.444	4.337e-3	3.722e-3	12	29	16.4417	35.2265	AAL95493.1| Methionine aminopeptidase
					23	27	23.0000	35.4187	
**FN1298**	-0.719	13.058	7.377e-3	7.764e-3	62	99	84.9487	120.2559	AAL95494.1| Adenylate kinase
					59	89	59.0000	116.7507	
**FN1299**									AAL95495.1| dTDP-glucose 4,6-dehydratase
					6		6.0000		
**FN1301**	-0.775	12.818	4.601e-2	8.006e-2	70	88	95.9098	106.8941	AAL95497.1| ABC transporter ATP-binding protein
					34	88	34.0000	115.4389	
**FN1302**	0.143	17.191	2.688e-1	8.195e-1	489	349	669.9984	423.9325	AAL95498.1| Hypothetical protein
					143	238	143.0000	312.2097	
**FN1303**	-0.634	12.859	6.586e-4	2.35e-4	55	85	75.3577	103.2500	AAL95499.1| hypothetical cytosolic protein
					63	85	63.0000	111.5034	
**FN1304**	-0.297	14.350	3.374e-2	5.275e-2	83	133	113.7216	161.5559	AAL95500.1| Single-strand DNA binding protein
					147	121	147.0000	158.7284	
**FN1305**	0.390	11.020	1.948e-3	1.24e-3	36	31	49.3250	37.6559	AAL95501.1| Hypothetical cytosolic protein
					55	32	55.0000	41.9778	
**FN1306**	0.930	14.022				75		91.1030	AAL95502.1| Methyltransferase
					178	73	178.0000	95.7618	
**FN1309**	-1.262	15.446	2.089e-3	1.378e-3	121	272	165.7869	330.4001	AAL95505.1| Hypothetical protein
					107	247	107.0000	324.0159	
**FN1311**									AAL95507.1| Biopolymer transport exbD protein
						8		10.4944	
**FN1312**						44		53.4471	AAL95508.1| Biopolymer transport exbB protein
						52		68.2139	

Log$_2$ Ratios: -6 -4 -2 0 2 4 6

Show detected proteins only
Show all proteins
Filter by category:
GO: amino acid transport

Proteins found: 1338

Enter (or paste) list of ORFs

Find ORFs

Test	Cutoff
q-Value	.005
p-Value	

	Signif	Direction	Applies To
red	yes	+	ratios, bars
yellow	no	n/a	bars
green	yes	–	ratios, bars
pink	yes	+	*p*- , *q*-Values
light green	yes	–	

Dot Plots Dot Plots

Protein	FnPg vs Fn Log$_2$ Ratio	Log$_2$ Sum	*q*-Value	*p*-Value	Raw *FnPg*	Raw *Fn*	Normalized *FnPg*	Normalized *Fn*	Description
FN1313	-0.725	12.654	8.857e-4	3.897e-4	51	90	69.8771	109.3236	AAL95509.1| Oligopeptide-binding protein oppA
					55	74	55.0000	97.0736	
FN1315						7		8.5029	AAL95511.1| Hypothetical protein
FN1317	-0.425	7.763	6.64e-2	1.333e-1	12	13	16.4417	15.7912	AAL95513.1| RNA polymerase sigma factor
					9	14	9.0000	18.3653	
FN1318	-0.535	10.215	1.379e-2	1.671e-2	17	37	23.2924	44.9441	AAL95514.1| RNA polymerase sigma factor rpoD
					34	29	34.0000	38.0424	
FN1319	-0.561	7.146	7.728e-3	8.256e-3	7	13	9.5910	15.7912	AAL95515.1| DNA primase
					10	10	10.0000	13.1181	
FN1320	-1.481	16.169	1.26e-4	1.988e-5	116	385	158.9362	467.6619	AAL95516.1| Peptidyl-prolyl cis-trans isomerase
					166	335	166.0000	439.4548	
FN1321	0.002	19.560	3.006e-1	9.58e-1	641	752	878.2596	913.4591	AAL95517.1| Acetoacetate metabolism regulatory protein atoC
					881	643	881.0000	843.4908	
FN1322	0.997	9.177				14		17.0059	AAL95518.1| Membrane metalloprotease
					34	13	34.0000	17.0535	
FN1323	-0.817	7.250	2.046e-4	3.943e-5	7	14	9.5910	17.0059	AAL95519.1| Thymidylate kinase
					9	12	9.0000	15.7417	
FN1324	0.097	10.185	2.672e-1	8.128e-1	15	23	20.5521	27.9382	AAL95520.1| 1-deoxy-D-xylulose 5-phosphate reductoisomerase
					50	29	50.0000	38.0424	
FN1326	-0.927	6.927				11		13.3618	AAL95522.1| Undecaprenyl pyrophosphate synthetase
					8	13	8.0000	17.0535	
FN1327									AAL95523.1| Dimethylallyltransferase
						3		3.9354	
FN1328						11		13.3618	AAL95524.1| Exodeoxyribonuclease VII small subunit
						12		15.7417	
FN1330	-0.281	3.451				3		3.6441	AAL95526.1| S-adenosylmethionine:tRNA ribosyltransferase-isomerase
					3		3.0000		
FN1331						5		6.0735	AAL95527.1| Methyltransferase
						8		10.4944	
FN1332	-0.954	11.223	1.642e-6	5.352e-8	25	57	34.2535	69.2383	AAL95528.1| Bacterial Peptide Chain Release Factor 1 (RF-1)
					36	51	36.0000	66.9021	

Log$_2$ Ratios: -6 -4 -2 0 2 4 6

Show detected proteins only
Show all proteins
Filter by category:
GO: amino acid transport

Proteins found: 1338

Enter (or paste) list of ORFs

Find ORFs

Test	Cutoff
q-Value	.005
p-Value	

	Signif	Direction	Applies To
red	yes	+	ratios, bars
yellow	no	n/a	bars
green	yes	−	ratios, bars
pink	yes	+	*p*-, *q*-Values
light green	yes	−	

Dot Plots Dot Plots

Protein	FnPg vs Fn Log2 Ratio	Log2 Sum	q-Value	p-Value	Raw FnPg	Raw Fn	Normalized FnPg	Normalized Fn	Description
FN1334	0.666	8.947	1.665e-1	4.361e-1	35	15	47.9549	18.2206	AAL95530.1| N-acetylmuramoyl-L-alanine amidase
					8	13	8.0000	17.0535	
FN1335	-0.510	12.806	3.382e-2	5.293e-2	40	95	54.8056	115.3971	AAL95531.1| Protein translocase subunit YajC
					87	66	87.0000	86.5791	
FN1336	-0.345	9.253			16	21	21.9222	25.5088	AAL95532.1| Hypothetical protein
						23		30.1715	
FN1337	-2.173	7.980	6.754e-3	6.861e-3	8	21	10.9611	25.5088	AAL95533.1| unknown
					4	32	4.0000	41.9778	
FN1340	-0.495	16.632	5.847e-2	1.116e-1	259	288	354.8662	349.8354	AAL95536.1| Glutamyl-tRNA synthetase
					182	310	182.0000	406.6596	
FN1341	-0.268	7.438				13		15.7912	AAL95537.1| Bacterial Peptide Chain Release Factor 2 (RF-2)
					12	10	12.0000	13.1181	
FN1343	1.511	9.473				13		15.7912	AAL95539.1| seC-independent protein TATD
					45		45.0000		
FN1346									AAL95542.1| Hypothetical cytosolic protein
					6		6.0000		
FN1347	-1.806	7.591	1.867e-3	1.164e-3	5	19	6.8507	23.0794	AAL95543.1| Hypothetical cytosolic protein
					8	22	8.0000	28.8597	
FN1348	-1.177	8.158	1.437e-2	1.76e-2	4	17	5.4806	20.6500	AAL95544.1| ABC transporter ATP-binding protein
					17	23	17.0000	30.1715	
FN1349	-2.532	8.146				31		37.6559	AAL95545.1| ABC transporter permease protein
					7	33	7.0000	43.2896	
FN1351	-0.416	11.067	8.498e-2	1.841e-1	33	33	45.2146	40.0853	AAL95547.1| 15 kDa lipoprotein precursor
					35	51	35.0000	66.9021	
FN1352	-0.521	11.686	3.2e-3	2.46e-3	32	56	43.8445	68.0235	AAL95548.1| ABC transporter ATP-binding protein
					52	53	52.0000	69.5257	
FN1353	-0.149	7.764				18		21.8647	AAL95549.1| ABC transporter permease protein
					14	7	14.0000	9.1826	
FN1354	-0.116	10.024				24		29.1529	AAL95550.1| ABC transporter permease protein
					31	29	31.0000	38.0424	
FN1355						4		4.8588	AAL95551.1| Integral membrane protein
						7		9.1826	

Log2 Ratios: -6 -4 -2 0 2 4 6

Show detected proteins only
Show all proteins
Filter by category:
GO: amino acid transport

Proteins found: 1338

Enter (or paste) list of ORFs

Find ORFs

Test	Cutoff
q-Value	.005
p-Value	

	Signif	Direction	Applies To
(red)	yes	+	ratios, bars
(yellow)	no	n/a	bars
(green)	yes	−	ratios, bars
(pink)	yes	+	p- , q-Values
(light green)	yes	−	

Dot Plots Dot Plots

Protein	FnPg vs Fn Log$_2$ Ratio	Log$_2$ Sum	*q*-Value	*p*-Value	Raw *FnPg*	Raw *Fn*	Normalized *FnPg*	Normalized *Fn*	Description	Log$_2$ Ratios (-6 -4 -2 0 2 4 6)
FN1358									AAL95554.1| Hypothetical protein	
					48		48.0000			
FN1359	1.675	16.313	6.126e-2	1.195e-1	167	116	228.8134	140.9059	AAL95555.1| Dipeptide-binding protein	
					791	136	791.0000	178.4055		
FN1362					25		34.2535		AAL95558.1| Dipeptide transport ATP-binding protein dppD	
					70		70.0000			
FN1363	1.759	8.809	3.939e-2	6.462e-2	16	6	21.9222	7.2882	AAL95559.1| Dipeptide transport ATP-binding protein dppF	
					56	12	56.0000	15.7417		
FN1364	-1.345	12.067			30	79	41.1042	95.9618	AAL95560.1| LSU ribosomal protein L32P	
						86		112.8153		
FN1365	-0.595	14.347	8.129e-2	1.736e-1	43	156	58.9160	189.4942	AAL95561.1| GTP-binding protein	
					176	126	176.0000	165.2875		
FN1366	-0.402	15.015	2.211e-2	3.071e-2	99	190	135.6438	230.7942	AAL95562.1| Triosephosphate isomerase	
					181	143	181.0000	187.5882		
FN1371									AAL95567.1| Ribonuclease HII	
					4		4.0000			
FN1374	-0.919	6.534				11		13.3618	AAL95570.1| Transcriptional regulator	
					7	10	7.0000	13.1181		
FN1375	1.676	11.582				24		29.1529	AAL95571.1| Citrate-sodium symport	
					99	25	99.0000	32.7951		
FN1376	-0.022	18.502	2.977e-1	9.453e-1	282	510	386.3794	619.5002	AAL95572.1| Oxaloacetate decarboxylase alpha chain	
					823	464	823.0000	608.6776		
FN1378	-0.796	9.834	7.855e-2	1.658e-1	5	31	6.8507	37.6559	AAL95574.1| Citrate lyase acyl carrier protein	
					39	32	39.0000	41.9778		
FN1379	0.395	15.728	2.098e-1	5.92e-1	60	179	82.2084	217.4324	AAL95575.1| Citrate lyase beta chain	
					452	144	452.0000	188.9000		
FN1380	0.460	17.821	2.113e-1	5.973e-1	81	344	110.9813	417.8589	AAL95576.1| Citrate lyase beta chain	
					1018	307	1018.0000	402.7242		
FN1381						4		4.8588	AAL95577.1| unknown	
						4		5.2472		
FN1382									AAL95578.1| ATPase	
					13		13.0000			

Protein	FnPg vs Fn Log_2 Ratio	Log_2 Sum	*q*-Value	*p*-Value	Raw *FnPg*	Raw *Fn*	Normalized *FnPg*	Normalized *Fn*	Description
FN1383					9		12.3313		AAL95579.1| DNA polymerase III alpha subunit
					5		5.0000		
FN1385	-1.567	4.737				6		7.2882	AAL95581.1| Hypothetical protein
					3	8	3.0000	10.4944	
FN1386	0.648	6.271				4		4.8588	AAL95582.1| SWF/SNF family helicase
					11	7	11.0000	9.1826	
FN1391	-1.043	12.722	2.913e-3	2.15e-3	50	110	68.5070	133.6177	AAL95584.1| Acetyltransferase
					46	78	46.0000	102.3208	
FN1392	-0.672	14.866	9.864e-2	2.232e-1	167	181	228.8134	219.8618	AAL95585.1| SSU ribosomal protein S16P
					45	165	45.0000	216.4479	
FN1393	-2.009	9.995	1.006e-3	4.741e-4	5	58	6.8507	70.4530	AAL95586.1| Signal recognition particle, subunit FFH/SRP54
					25	44	25.0000	57.7194	
FN1397	-1.469	13.773	4.132e-3	3.477e-3	33	161	45.2146	195.5677	AAL95590.1| Glutaminase
					97	151	97.0000	198.0826	
FN1398	-1.087	12.699	3.05e-2	4.617e-2	16	119	21.9222	144.5500	AAL95591.1| Amino acid carrier protein alsT
					90	71	90.0000	93.1382	
FN1406	0.209	11.969	2.406e-1	7.065e-1	22	43	30.1431	52.2324	AAL95599.1| Histidine ammonia-lyase
					106	50	106.0000	65.5903	
FN1407	1.487	8.513				8		9.7176	AAL95600.1| Glutamate formiminotransferase
					32	10	32.0000	13.1181	
FN1411	1.414	15.743	2.48e-2	3.551e-2	196	112	268.5474	136.0471	AAL95604.1| Threonine dehydratase
					496	115	496.0000	150.8576	
FN1412					20		27.4028		AAL95605.1| 5-methylthioribose kinase
FN1413					10		13.7014		AAL95606.1| Translation initiation factor EIF-2B subunit 1
FN1415					11		15.0715		AAL95608.1| NADH-dependent butanol dehydrogenase A
					7		7.0000		
FN1416									AAL95609.1| Transcriptional regulator, GntR family
					24		24.0000		
FN1417	1.041	5.824			7		9.5910		AAL95610.1| L-fuculose phosphate aldolase
					12	4	12.0000	5.2472	

Log_2 Ratios: -6 -4 -2 0 2 4 6

Show detected proteins only
Show all proteins
Filter by category:
GO: amino acid transport

Proteins found: 1338

Enter (or paste) list of ORFs

Find ORFs

Test	Cutoff
q-Value	.005
p-Value	

	Signif	Direction	Applies To
red	yes	+	ratios, bars
yellow	no	n/a	bars
green	yes	–	ratios, bars
pink	yes	+	*p*- , *q*-Values
light green	yes	–	

Dot Plots Dot Plots

Protein	*FnPg* vs *Fn*				Raw		Normalized		Description
	Log_2 Ratio	Log_2 Sum	*q*-Value	*p*-Value	*FnPg*	*Fn*	*FnPg*	*Fn*	
**FN1418**	-0.352	4.431			3		4.1104		AAL95611.1| Transcriptional regulator, GntR family
						4		5.2472	
**FN1419**	3.256	21.695	2.928e-2	4.378e-2	6075	475	8323.5996	576.9855	AAL95612.1| Methionine gamma-lyase
					3065	469	3065.0000	615.2367	
**FN1421**	1.019	21.168	5.457e-2	1.011e-1	2193	842	3004.7167	1022.7826	AAL95614.1| Pyruvate-flavodoxin oxidoreductase
					1365	864	1365.0000	1133.3998	
**FN1423**	-0.853	14.131	4.087e-2	6.792e-2	36	155	49.3250	188.2795	AAL95616.1| Flavoprotein
					150	131	150.0000	171.8465	
**FN1424**	-1.272	15.849	1.827e-2	2.406e-2	45	329	61.6563	399.6383	AAL95617.1| ACYL-COA dehydrogenase, short-chain specific
					251	271	251.0000	355.4992	
**FN1426**	-2.115	16.397	2.673e-3	1.909e-3	44	496	60.2862	602.4943	AAL95619.1| Serine protease
					222	473	222.0000	620.4839	
**FN1429**						7		8.5029	AAL95622.1| Branched-chain amino acid transport ATP-binding protein livG
**FN1432**						52		63.1647	AAL95625.1| Leucine-, isoleucine-, valine-, threonine-, and alanine-binding protein
						38		49.8486	
**FN1433**	0.148	16.031	1.611e-2	2.037e-2	202	193	276.7682	234.4383	AAL95626.1| Short chain dehydrogenase
					268	196	268.0000	257.1138	
**FN1434**	-0.562	13.916	3.737e-2	6.026e-2	53	117	72.6174	142.1206	AAL95627.1| Tetratricopeptide repeat family protein
					132	122	132.0000	160.0402	
**FN1437**	-1.261	14.910	4.208e-3	3.566e-3	110	215	150.7154	261.1618	AAL95630.1| LSU ribosomal protein L28P
					76	215	76.0000	282.0381	
**FN1439**	-2.376	11.423				81		98.3912	AAL95632.1| Transcriptional regulator, DeoR family
					23	107	23.0000	140.3632	
**FN1440**	-1.976	12.297	1.173e-3	6.117e-4	23	128	31.5132	155.4824	AAL95633.1| 1-phosphofructokinase
					40	96	40.0000	125.9333	
**FN1441**	-2.055	13.602	1.297e-3	7.084e-4	20	195	27.4028	236.8677	AAL95634.1| PTS system, fructose-specific IIABC component
					82	166	82.0000	217.7597	
**FN1444**	0.031	17.717	2.477e-1	7.34e-1	374	362	512.4323	439.7237	AAL95637.1| GMP synthase (glutamine-hydrolyzing)
					426	365	426.0000	478.8089	
**FN1445**	-1.799	10.317	3.063e-3	2.309e-3	17	46	23.2924	55.8765	AAL95638.1| DNA helicase
					15	59	15.0000	77.3965	

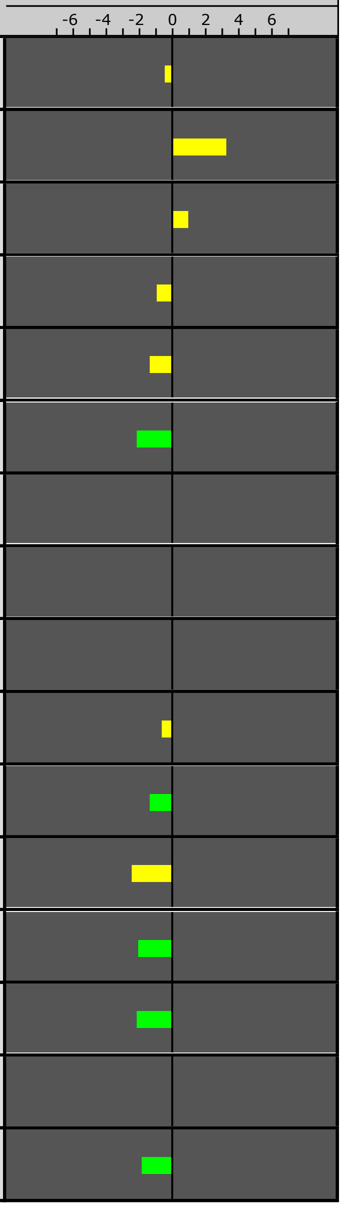

Show detected proteins only
Show all proteins
Filter by category:
GO: amino acid transport

Proteins found: 1338

Enter (or paste) list of ORFs

Find ORFs

Test	Cutoff
q-Value	.005
p-Value	

	Signif	Direction	Applies To
red	yes	+	ratios, bars
yellow	no	n/a	bars
green	yes	−	ratios, bars
light red	yes	+	*p*- , *q*-Values
light green	yes	−	

Dot Plots Dot Plots

Protein	FnPg vs Fn Log2 Ratio	Log2 Sum	q-Value	p-Value	Raw FnPg	Raw Fn	Normalized FnPg	Normalized Fn	Description
FN1449	-3.578	15.303	3.516e-7	7.114e-9	55	560	75.3577	680.2355	AAL95642.1| Fusobacterium outer membrane protein family
					41	541	41.0000	709.6867	
FN1450	-0.451	8.791				20		24.2941	AAL95643.1| Integral membrane protein
					18	19	18.0000	24.9243	
FN1451	-0.039	14.819	7.148e-2	1.465e-1	120	139	164.4168	168.8442	AAL95644.1| Cell division protein ftsZ
					171	134	171.0000	175.7819	
FN1452	0.036	13.611	2.74e-1	8.416e-1	96	103	131.5334	125.1147	AAL95645.1| Cell division protein ftsA
					95	73	95.0000	95.7618	
FN1454	-0.702	9.486				26		31.5824	AAL95647.1| D-alanine--D-alanine ligase
					21	28	21.0000	36.7305	
FN1455	0.157	8.630	1.9e-1	5.228e-1	19	17	26.0327	20.6500	AAL95648.1| UDP-N-acetylenolpyruvoylglucosamine reductase
					16	13	16.0000	17.0535	
FN1456	-0.507	11.985	4.608e-3	4.063e-3	40	58	54.8056	70.4530	AAL95649.1| UDP-N-acetylmuramate--alanine ligase
					52	62	52.0000	81.3319	
FN1457	-0.631	11.388	8.193e-2	1.755e-1	14	51	19.1820	61.9500	AAL95650.1| UDP-N-acetylglucosamine-N-acetylmuramyl-Pentapeptide pyrophosphoryl-undecaprenol N-
					64	51	64.0000	66.9021	
FN1458	0.050	12.577	2.882e-1	9.029e-1	84	66	115.0917	80.1706	AAL95651.1| UDP-N-acetylmuramoylalanine--D-glutamate ligase
					44	56	44.0000	73.4611	
FN1459									AAL95652.1| Phospho-N-acetylmuramoyl-pentapeptide-transferase
						12		15.7417	
FN1461	-0.759	10.290	5.368e-8	4.345e-10	20	38	27.4028	46.1588	AAL95654.1| Histidinol-phosphatase
					27	35	27.0000	45.9132	
FN1463	0.182	16.609	1.215e-1	2.9e-1	285	236	390.4899	286.6707	AAL95656.1| pyridoxine biosynthesis protein
					283	234	283.0000	306.9624	
FN1464	-0.124	15.788	2.528e-1	7.543e-1	91	209	124.6827	253.8736	AAL95657.1| 1-deoxyxylulose-5-phosphate synthase
					331	185	331.0000	242.6840	
FN1470	-2.269	10.228	3.663e-4	9.386e-5	15	69	20.5521	83.8147	AAL95663.1| Hypothetical protein
					11	52	11.0000	68.2139	
FN1471						8		9.7176	AAL95664.1| LACI-family transcription regulator
						15		19.6771	
FN1472	-3.274	12.762	4.674e-4	1.384e-4	7	239	9.5910	290.3148	AAL95665.1| N-acetylneuraminate-binding protein
					44	174	44.0000	228.2541	

Log2 Ratios: -6 -4 -2 0 2 4 6

Show detected proteins only
Show all proteins
Filter by category:
GO: amino acid transport

Proteins found: 1338

Enter (or paste) list of ORFs

Find ORFs

Test	Cutoff
q-Value	.005
p-Value	

Signif	Direction	Applies To
yes	+	ratios, bars
no	n/a	bars
yes	–	ratios, bars
yes	+	p- , q-Values
yes	–	

Dot Plots Dot Plots

Protein	FnPg vs Fn Log_2 Ratio	Log_2 Sum	*q*-Value	*p*-Value	Raw *FnPg*	Raw *Fn*	Normalized *FnPg*	Normalized *Fn*	Description
FN1474						8		9.7176	AAL95667.1| N-acetylmannosamine kinase
						7		9.1826	
FN1475	-1.481	9.773	5.317e-3	4.94e-3	20	36	27.4028	43.7294	AAL95668.1| N-acetylneuraminate lyase
					8	42	8.0000	55.0958	
FN1476						19		23.0794	AAL95669.1| N-acetylmannosamine-6-phosphate 2-epimerase
						18		23.6125	
FN1478									AAL95671.1| Hypothetical protein
					23		23.0000		
FN1479	-0.899	9.593	1.151e-2	1.341e-2	10	29	13.7014	35.2265	AAL95672.1| Hypothetical protein
					27	31	27.0000	40.6660	
FN1480	-0.025	8.554	2.755e-1	8.479e-1	12	16	16.4417	19.4353	AAL95673.1| MG2+ transporter MGTE
					22	15	22.0000	19.6771	
FN1481	0.921	6.728	7.301e-3	7.657e-3	9	8	12.3313	9.7176	AAL95674.1| Queuine tRNA-ribosyltransferase
					16	4	16.0000	5.2472	
FN1482	-0.212	11.556	1.174e-1	2.775e-1	35	40	47.9549	48.5882	AAL95675.1| Guanosine-3',5'-bis (Diphosphate) 3'-pyrophosphohydrolase
					54	53	54.0000	69.5257	
FN1483	-0.713	10.454	9.174e-4	4.111e-4	23	39	31.5132	47.3735	AAL95676.1| Adenine phosphoribosyltransferase
					27	37	27.0000	48.5368	
FN1484						9		10.9324	AAL95677.1| Tetratricopeptide repeat family protein
						13		17.0535	
FN1486						5		6.0735	AAL95679.1| magnesium and cobalt efflux protein CorC
						6		7.8708	
FN1487	-1.415	14.595	4.163e-3	3.513e-3	45	207	61.6563	251.4442	AAL95681.1| Chorismate mutase
					131	200	131.0000	262.3611	
FN1488					22		30.1431		AAL95682.1| Methylenetetrahydrofolate dehydrogenase (NADP+)
					12		12.0000		
FN1489	-0.585	9.086	2.168e-2	2.997e-2	11	20	15.0715	24.2941	AAL95683.1| Methionyl-tRNA formyltransferase
					23	25	23.0000	32.7951	
FN1490	0.744	7.459	5.827e-2	1.111e-1	9	5	12.3313	6.0735	AAL95684.1| putative regulatory protein
					22	11	22.0000	14.4299	
FN1491	-1.070	7.713							AAL95685.1| PTS system, IIA component
					10	16	10.0000	20.9889	

Log_2 Ratios: -6 -4 -2 0 2 4 6

Show detected proteins only
Show all proteins
Filter by category:
GO: amino acid transport

Proteins found: 1338

Enter (or paste) list of ORFs

Find ORFs

Test	Cutoff
q-Value	.005
p-Value	

Color	Signif	Direction	Applies To
red	yes	+	ratios, bars
yellow	no	n/a	bars
green	yes	−	ratios, bars
pink	yes	+	*p*- , *q*-Values
light green	yes	−	

Dot Plots Dot Plots

Protein	FnPg vs Fn Log$_2$ Ratio	Log$_2$ Sum	*q*-Value	*p*-Value	Raw *FnPg*	Raw *Fn*	Normalized *FnPg*	Normalized *Fn*	Description
FN1493	-1.101	7.965	1.442e-2	1.768e-2	7	23	9.5910	27.9382	AAL95687.1| Hypothetical protein
					12	14	12.0000	18.3653	
FN1494	-1.569	8.212				24		29.1529	AAL95680.1| Rod shape-determining protein mreC
					10	23	10.0000	30.1715	
FN1496	-1.569	8.212				24		29.1529	AAL95680.1| Rod shape-determining protein mreC
					10	23	10.0000	30.1715	
FN1499	-0.356	11.888	1.919e-1	5.294e-1	13	65	17.8118	78.9559	AAL93625.1| Cell surface protein
					91	46	91.0000	60.3430	
FN1504	-1.567	13.803	5.632e-5	5.926e-6	43	179	58.9160	217.4324	AAL93630.1| Nickel-binding protein
					80	148	80.0000	194.1472	
FN1505	-0.020	16.220	2.836e-1	8.827e-1	172	215	235.6641	261.1618	AAL93631.1| 6,7-dimethyl-8-ribityllumazine synthase
					313	225	313.0000	295.1562	
FN1506	0.684	7.889			19	10	26.0327	12.1471	AAL93632.1| Diaminohydroxyphosphoribosylaminopyrimidine deaminase
					13		13.0000		
FN1508	0.227	13.127	1.712e-1	4.533e-1	53	77	72.6174	93.5324	AAL93634.1| GTP cyclohydrolase II
					132	62	132.0000	81.3319	
FN1512						11		13.3618	AAL93638.1| hypothetical exported 24-amino acid repeat protein
						8		10.4944	
FN1517	-0.545	15.824	4.167e-2	6.973e-2	102	262	139.7543	318.2530	AAL93643.1| Leucyl-tRNA synthetase
					259	201	259.0000	263.6729	
FN1518						9		10.9324	AAL93644.1| RNA polymerase sigma-H factor
						6		7.8708	
FN1519	-0.956	8.357				21		25.5088	AAL93645.1| 23S rRNA methyltransferase
					13	19	13.0000	24.9243	
FN1520	-1.151	10.981	2.418e-2	3.44e-2	9	52	12.3313	63.1647	AAL93646.1| UDP-N-acetylglucosamine 1-carboxyvinyltransferase
					48	54	48.0000	70.8375	
FN1521						15		18.2206	AAL93647.1| Dipeptide transport system permease protein dppB
						16		20.9889	
FN1523	-2.022	15.040	1.623e-5	9.748e-7	68	299	93.1695	363.1972	AAL93649.1| Dipeptide-binding protein
					89	287	89.0000	376.4881	
FN1524						5		6.0735	AAL93650.1| Dipeptide transport ATP-binding protein dppD

Log$_2$ Ratios: -6 -4 -2 0 2 4 6

Show detected proteins only
Show all proteins
Filter by category:
GO: amino acid transport

Proteins found: 1338

Enter (or paste) list of ORFs

Find ORFs

Test	Cutoff
q-Value	.005
p-Value	

	Signif	Direction	Applies To
(red)	yes	+	ratios, bars
(yellow)	no	n/a	bars
(green)	yes	–	ratios, bars
(pink)	yes	+	*p*- , *q*-Values
(light green)	yes	–	*p*- , *q*-Values

Dot Plots Dot Plots

Protein	FnPg vs Fn: Log_2 Ratio	Log_2 Sum	*q*-Value	*p*-Value	Raw: *FnPg*	Raw: *Fn*	Normalized: *FnPg*	Normalized: *Fn*	Description
FN1525						5		6.0735	AAL93651.1| Dipeptide transport ATP-binding protein dppF
FN1526	-0.352	22.555	1.721e-1	4.564e-1	2501	2415	3426.7198	2933.5155	AAL93652.1| Fusobacterium outer membrane protein family
					968	2039	968.0000	2674.7710	
FN1527	-2.812	12.685	4.914e-4	1.512e-4	25	164	34.2535	199.2118	AAL93653.1| Hypothetical protein
					27	176	27.0000	230.8777	
FN1528	-1.987	13.504	7.129e-4	2.683e-4	52	173	71.2473	210.1442	AAL93654.1| Hypothetical protein
					37	167	37.0000	219.0715	
FN1529	-0.496	12.897	1.244e-1	2.989e-1	84	79	115.0917	95.9618	AAL93655.1| Hypothetical protein
					32	85	32.0000	111.5034	
FN1531	0.267	9.845	1.848e-1	5.038e-1	15	25	20.5521	30.3677	AAL93657.1| murein hydrolase export regulator
					46	19	46.0000	24.9243	
FN1533	0.427	19.096	1.955e-1	5.418e-1	1043	534	1429.0559	648.6531	AAL93659.1| Electron transfer flavoprotein alpha-subunit
					307	490	307.0000	642.7846	
FN1534	0.680	20.164	1.196e-1	2.84e-1	1502	657	2057.9500	798.0620	AAL93660.1| Electron transfer flavoprotein beta-subunit
					686	697	686.0000	914.3283	
FN1535	1.049	21.288	4.761e-3	4.263e-3	1883	873	2579.9733	1060.4385	AAL93661.1| Acyl-CoA dehydrogenase, short-chain specific
					2023	888	2023.0000	1164.8831	
FN1536	1.713	19.223	2.984e-3	2.224e-3	905	369	1239.9766	448.2266	AAL93662.1| (S)-2-hydroxy-acid oxidase chain D
					1593	317	1593.0000	415.8423	
FN1537	-0.815	5.724			4	4	5.4806	4.8588	AAL93663.1| Arsenical pump-driving ATPase
						11		14.4299	
FN1538	-0.071	7.783	2.556e-1	7.654e-1	8	11	10.9611	13.3618	AAL93664.1| Arsenical pump-driving ATPase
					18	13	18.0000	17.0535	
FN1539	0.182	16.003	1.694e-1	4.465e-1	246	205	337.0544	249.0148	AAL93665.1| Iron-sulfur cluster-binding protein
					209	177	209.0000	232.1895	
FN1540	1.067	16.840	2.35e-2	3.318e-2	450	205	616.5629	249.0148	AAL93666.1| Iron-sulfur cluster-binding protein
					375	171	375.0000	224.3187	
FN1544	1.827	17.153	4.206e-2	7.062e-2	762	162	1044.0466	196.7824	AAL93670.1| Probable electron transfer flavoprotein-quinone oxidoreductase ydiS
					394	159	394.0000	208.5770	
FN1545	2.272	11.903	7.697e-2	1.614e-1	173	28	237.0342	34.0118	AAL93671.1| Ferredoxin like protein
					35	17	35.0000	22.3007	

Log_2 Ratios: -6 -4 -2 0 2 4 6

Show detected proteins only
Show all proteins
Filter by category:
GO: amino acid transport

Proteins found: 1338

Enter (or paste) list of ORFs

Find ORFs

Test	Cutoff
q-Value	.005
p-Value	

	Signif	Direction	Applies To
red	yes	+	ratios, bars
yellow	no	n/a	bars
green	yes	−	ratios, bars
pink	yes	+	*p*- , *q*-Values
light green	yes	−	

Dot Plots Dot Plots

Protein	FnPg vs Fn Log2 Ratio	Log2 Sum	q-Value	p-Value	Raw FnPg	Raw Fn	Normalized FnPg	Normalized Fn	Description
FN1546	1.071	20.811	7.679e-2	1.609e-1	2138	730	2929.3590	886.7355	AAL93672.1| Protein Translation Elongation Factor G (EF-G)
					1003	751	1003.0000	985.1658	
FN1547	-0.720	14.595	5.234e-3	4.838e-3	76	165	104.1306	200.4265	AAL93673.1| PTS permease for N-acetylglucosamine and glucose
					141	155	141.0000	203.3298	
FN1548	-0.490	10.041	8.847e-4	3.888e-4	21	32	28.7729	38.8706	AAL93674.1| Hypothetical protein
					26	29	26.0000	38.0424	
FN1549	-0.602	19.011	6.144e-3	6.014e-3	362	667	495.9906	810.2090	AAL93675.1| Stomatin like protein
					684	748	684.0000	981.2303	
FN1552	-1.922	5.922				12		14.5765	AAL93678.1| abortive phage resistance protein
					4	12	4.0000	15.7417	
FN1553	-1.121	7.900	2.257e-3	1.526e-3	8	17	10.9611	20.6500	AAL93679.1| abortive phage resistance protein
					10	19	10.0000	24.9243	
FN1554	-0.887	16.740	1.18e-3	6.182e-4	158	337	216.4821	409.3560	AAL93680.1| Fusobacterium outer membrane protein family
					270	374	270.0000	490.6152	
FN1555	-0.096	23.668	1.81e-2	2.377e-2	2528	3198	3463.7135	3884.6305	AAL93681.1| Protein Translation Elongation Factor Tu
					3600	2792	3600.0000	3662.5603	
FN1556	-0.929	20.112	1.178e-3	6.165e-4	621	1210	850.8568	1469.7945	AAL93682.1| Protein Translation Elongation Factor G (EF-G)
					692	1119	692.0000	1467.9101	
FN1557	0.863	13.989	7.109e-7	1.726e-8	124	79	169.8973	95.9618	AAL93683.1| SSU ribosomal protein S7P
					174	71	174.0000	93.1382	
FN1560	-0.560	13.953	1.565e-1	4.016e-1	12	133	16.4417	161.5559	AAL93686.1| unknown
					191	110	191.0000	144.2986	
FN1562	-1.156	6.793	8.204e-3	8.941e-3	3	14	4.1104	17.0059	AAL93688.1| Phospho-2-dehydro-3-deoxyheptonate aldolase
					10	11	10.0000	14.4299	
FN1577	0.437	15.288	1.17e-1	2.763e-1	112	134	153.4557	162.7706	AAL93692.1| Rod shape-determining protein mreB
					312	138	312.0000	181.0291	
FN1579	-0.033	12.799	2.884e-1	9.037e-1	43	78	58.9160	94.7471	AAL93694.1| Cysteinyl-tRNA synthetase
					108	58	108.0000	76.0847	
FN1580						6		7.2882	AAL93695.1| 2-C-methyl-D-erythritol 4-phosphate cytidylyltransferase
FN1581	-0.576	10.825	1.57e-2	1.97e-2	21	37	28.7729	44.9441	AAL93696.1| DNA mismatch repair protein mutS
					41	45	41.0000	59.0312	

Log2 Ratios: -6 -4 -2 0 2 4 6

Show detected proteins only
Show all proteins
Filter by category: GO: amino acid transport

Proteins found: 1338

Enter (or paste) list of ORFs

Find ORFs

Test	Cutoff
q-Value	.005
p-Value	

Color	Signif	Direction	Applies To
red	yes	+	ratios, bars
yellow	no	n/a	bars
green	yes	−	ratios, bars
pink	yes	+	p-, q-Values
light green	yes	−	p-, q-Values

Dot Plots Dot Plots

Protein	FnPg vs Fn Log$_2$ Ratio	Log$_2$ Sum	*q*-Value	*p*-Value	Raw *FnPg*	Raw *Fn*	Normalized *FnPg*	Normalized *Fn*	Description	Log$_2$ Ratios (-6 -4 -2 0 2 4 6)
FN1582	0.608	8.310							AAL93697.1| Hypothetical protein	
					22	11	22.0000	14.4299		
FN1586									AAL93701.1| O-succinylbenzoate-CoA synthase	
					18		18.0000			
FN1589	-1.771	9.665	2.161e-3	1.443e-3	5	37	6.8507	44.9441	AAL93704.1| LexA repressor	
					24	46	24.0000	60.3430		
FN1590									AAL93705.1| Hypothetical lipoprotein	
					5		5.0000			
FN1591	-0.869	15.690	1.049e-3	5.071e-4	141	247	193.1897	300.0324	AAL93706.1| RNFB-related protein	
					147	245	147.0000	321.3923		
FN1592									AAL93707.1| Na(+)-translocating NADH-quinone reductase subunit D	
					13		13.0000			
FN1594	-0.519	11.095	2.673e-2	3.9e-2	22	49	30.1431	59.5206	AAL93709.1| Nitrogen fixation protein RNFG	
					48	40	48.0000	52.4722		
FN1595	0.081	14.238	2.867e-1	8.961e-1	35	121	47.9549	146.9795	AAL93710.1| Na(+)-translocating NADH-quinone reductase subunit B	
					238	94	238.0000	123.3097		
FN1596	-0.888	19.764	2.767e-2	4.074e-2	722	1055	989.2410	1281.5151	AAL93711.1| Nitrogen fixation iron-sulphur protein RNFC	
					398	980	398.0000	1285.5692		
FN1597	0.789	8.764			20	11	27.4028	13.3618	AAL93712.1| Peptidyl-tRNA hydrolase	
						14		18.3653		
FN1600	0.594	6.325				6		7.2882	AAL93715.1| tRNA pseudouridine synthase A	
					11		11.0000			
FN1601									AAL93716.1| Hypothetical cytosolic protein	
						4		5.2472		
FN1603	-2.122	7.031			4	22	5.4806	26.7235	AAL93718.1| 2',3'-cyclic nucleotide 3'-phosphodiesterase	
						16		20.9889		
FN1605	-0.300	16.736	7.979e-2	1.693e-1	169	314	231.5536	381.4178	AAL93720.1| Adenylosuccinate synthetase	
					364	268	364.0000	351.5638		
FN1606	-1.640	10.113	4.178e-4	1.15e-4	10	46	13.7014	55.8765	AAL93721.1| 3-deoxy-D-manno-octulosonic-acid transferase	
					24	47	24.0000	61.6548		
FN1607	-0.404	8.668	4.08e-2	6.775e-2	11	22	15.0715	26.7235	AAL93722.1| Cytidylate kinase	
					20	15	20.0000	19.6771		

Show detected proteins only
Show all proteins
Filter by category:
GO: amino acid transport

Proteins found: 1338

Enter (or paste) list of ORFs

Find ORFs

Test	Cutoff
q-Value	.005
p-Value	

	Signif	Direction	Applies To
red	yes	+	ratios, bars
yellow	no	n/a	bars
green	yes	−	ratios, bars
pink	yes	+	*p*- , *q*-Values
light green	yes	−	*p*- , *q*-Values

Dot Plots Dot Plots

Protein	FnPg vs Fn Log$_2$ Ratio	Log$_2$ Sum	*q*-Value	*p*-Value	Raw *FnPg*	Raw *Fn*	Normalized *FnPg*	Normalized *Fn*	Description
FN1608									AAL93723.1| Ribosomal protein L11 methyltransferase
					8		8.0000		
FN1609	-2.360	9.530				55		66.8088	AAL93724.1| Hypothetical protein
					12	43	12.0000	56.4076	
FN1610	0.674	9.850	5.91e-4	1.98e-4	29	18	39.7341	21.8647	AAL93725.1| 33 kDa chaperonin
					37	20	37.0000	26.2361	
FN1611									AAL93726.1| Competence protein
						3		3.9354	
FN1613	-0.193	8.730	2.094e-2	2.87e-2	15	19	20.5521	23.0794	AAL93728.1| Hypothetical protein
					18	16	18.0000	20.9889	
FN1614	-0.398	10.108	6.677e-2	1.343e-1	24	25	32.8834	30.3677	AAL93729.1| MG(2+) chelatase family protein
					25	35	25.0000	45.9132	
FN1616	-0.145	8.145				14		17.0059	AAL93731.1| N utilization substance protein B
					16	14	16.0000	18.3653	
FN1618	0.013	10.298	3.024e-1	9.667e-1	17	29	23.2924	35.2265	AAL93733.1| Hypothetical protein
					48	27	48.0000	35.4187	
FN1619	1.079	14.402	5.791e-3	5.542e-3	134	90	183.5987	109.3236	AAL93734.1| Hypothetical cytosolic protein
					244	71	244.0000	93.1382	
FN1620	-0.686	18.440	3.672e-2	5.888e-2	468	589	641.2254	715.4620	AAL93735.1| SSU ribosomal protein S2P
					299	608	299.0000	797.5776	
FN1621	-0.815	20.738	1.155e-2	1.346e-2	923	1389	1264.6391	1687.2269	AAL93736.1| Protein Translation Elongation Factor Ts
					730	1388	730.0000	1820.7857	
FN1622	-1.405	14.538	1.889e-3	1.184e-3	53	207	72.6174	251.4442	AAL93737.1| Uridylate kinase
					117	191	117.0000	250.5548	
FN1623	-1.639	12.064	1.382e-3	7.695e-4	22	107	30.1431	129.9736	AAL93738.1| Ribosome Recycling Factor (RRF)
					44	77	44.0000	101.0090	
FN1624	-2.007	10.926				70		85.0294	AAL93739.1| Protein translocase subunit secY
					22	70	22.0000	91.8264	
FN1625	-1.012	13.664	1.985e-2	2.686e-2	85	140	116.4619	170.0589	AAL93740.1| LSU ribosomal protein L15P
					44	117	44.0000	153.4812	
FN1626	-3.376	9.716				75		91.1030	AAL93741.1| LSU ribosomal protein L30P
					9	73	9.0000	95.7618	

Log$_2$ Ratios: -6 -4 -2 0 2 4 6

Show detected proteins only
Show all proteins
Filter by category:
GO: amino acid transport

Proteins found: 1338

Enter (or paste) list of ORFs

Find ORFs

Test	Cutoff
q-Value	.005
p-Value	

Signif	Direction	Applies To
yes	+	ratios, bars
no	n/a	bars
yes	−	ratios, bars
yes	+	*p*- , *q*-Values
yes	−	

Dot Plots Dot Plots

Protein	FnPg vs Fn Log$_2$ Ratio	FnPg vs Fn Log$_2$ Sum	FnPg vs Fn *q*-Value	FnPg vs Fn *p*-Value	Raw *FnPg*	Raw *Fn*	Normalized *FnPg*	Normalized *Fn*	Description
FN1627	-0.912	16.987	2.226e-4	4.499e-5	193	397	264.4370	482.2384	AAL93742.1| SSU ribosomal protein S5P
					261	386	261.0000	506.3568	
FN1628	0.419	15.521			183	177	250.7356	215.0030	AAL93743.1| LSU ribosomal protein L18P
						122		160.0402	
FN1629	-0.876	17.655	3.913e-2	6.405e-2	369	524	505.5816	636.5061	AAL93744.1| LSU ribosomal protein L6P
					165	453	165.0000	594.2478	
FN1630	-0.713	13.210	1.009e-3	4.763e-4	57	108	78.0980	131.1883	AAL93745.1| SSU ribosomal protein S8P
					74	90	74.0000	118.0625	
FN1631	-1.281	5.924				10		12.1471	AAL93746.1| SSU ribosomal protein S14P
					5		5.0000		
FN1632	-1.015	16.452	7.678e-4	3.029e-4	152	367	208.2613	445.7972	AAL93747.1| LSU ribosomal protein L5P
					213	309	213.0000	405.3478	
FN1634	0.794	11.224			47	32	64.3966	38.8706	AAL93749.1| LSU ribosomal protein L24P
						27		35.4187	
FN1635	-1.370	9.635	6.473e-5	7.232e-6	11	39	15.0715	47.3735	AAL93750.1| LSU ribosomal protein L14P
					20	33	20.0000	43.2896	
FN1636	-1.670	10.912	5.613e-3	5.312e-3	6	76	8.2208	92.3177	AAL93751.1| SSU ribosomal protein S17P
					41	49	41.0000	64.2785	
FN1637						18		21.8647	AAL93752.1| LSU ribosomal protein L29P
						57		74.7729	
FN1638	-2.223	14.563	2.355e-4	4.924e-5	73	264	100.0202	320.6824	AAL93753.1| LSU ribosomal protein L16P
					44	268	44.0000	351.5638	
FN1639	-0.602	19.260	3.975e-2	6.54e-2	619	778	848.1166	945.0414	AAL93754.1| SSU ribosomal protein S3P
					438	768	438.0000	1007.4665	
FN1640	-0.669	13.873	4.407e-2	7.534e-2	98	128	134.2737	155.4824	AAL93755.1| LSU ribosomal protein L22P
					60	117	60.0000	153.4812	
FN1641	-0.944	14.982	3.583e-2	5.703e-2	147	225	201.4106	273.3089	AAL93756.1| SSU ribosomal protein S19P
					58	172	58.0000	225.6305	
FN1642	-1.187	17.039	2.304e-2	3.236e-2	285	429	390.4899	521.1090	AAL93757.1| LSU ribosomal protein L2P
					96	447	96.0000	586.3770	
FN1643	0.230	13.378	2.394e-1	7.017e-1	131	78	179.4883	94.7471	AAL93758.1| LSU ribosomal protein L23P
					44	73	44.0000	95.7618	

Log$_2$ Ratios: -6 -4 -2 0 2 4 6

Show detected proteins only
Show all proteins
Filter by category:
GO: amino acid transport

Proteins found: 1338

Enter (or paste) list of ORFs

Find ORFs

Test	Cutoff
q-Value	.005
p-Value	

	Signif	Direction	Applies To
red	yes	+	ratios, bars
yellow	no	n/a	bars
green	yes	–	ratios, bars
pink	yes	+	*p*- , *q*-Values
light green	yes	–	

Dot Plots Dot Plots

Protein	FnPg vs Fn Log$_2$ Ratio	FnPg vs Fn Log$_2$ Sum	FnPg vs Fn *q*-Value	FnPg vs Fn *p*-Value	Raw *FnPg*	Raw *Fn*	Normalized *FnPg*	Normalized *Fn*	Description
FN1644	-0.619	19.366	1.84e-2	2.429e-2	594	862	813.8631	1047.0768	AAL93759.1| LSU ribosomal protein L1E
					513	755	513.0000	990.4130	
FN1645	-1.468	17.056	7.871e-3	8.459e-3	248	528	339.7947	641.3649	AAL93760.1| LSU ribosomal protein L3P
					104	447	104.0000	586.3770	
FN1646	-0.665	15.556	5.081e-2	9.155e-2	180	223	246.6252	270.8795	AAL93761.1| SSU ribosomal protein S10P
					102	215	102.0000	282.0381	
FN1647	-0.316	17.063	2.085e-1	5.874e-1	409	384	560.3872	466.4472	AAL93762.1| Hypothetical protein
					103	274	103.0000	359.4346	
FN1652	-1.992	13.422	1.418e-4	2.337e-5	38	167	52.0653	202.8559	AAL93767.1| Oligopeptide-binding protein oppA
					53	164	53.0000	215.1361	
FN1654	0.914	10.762	9.644e-2	2.167e-1	20	23	27.4028	27.9382	AAL93769.1| Hypothetical protein
					87	25	87.0000	32.7951	
FN1655	-0.122	12.930	2.315e-1	6.717e-1	82	74	112.3515	89.8883	AAL93770.1| Hypothetical cytosolic protein
					57	72	57.0000	94.4500	
FN1657	-0.914	14.576	6.547e-2	1.308e-1	145	188	198.6703	228.3648	AAL93772.1| SSU ribosomal protein S6P
					29	153	29.0000	200.7062	
FN1658	-1.402	14.043	1.551e-3	8.967e-4	59	188	80.8383	228.3648	AAL93773.1| Prolyl-tRNA synthetase
					79	148	79.0000	194.1472	
FN1660									AAL93775.1| ATP-dependent DNA helicase recG
						4		5.2472	
FN1661	-0.541	10.636	5.53e-2	1.03e-1	22	49	30.1431	59.5206	AAL93776.1| Hypothetical cytosolic protein
					36	28	36.0000	36.7305	
FN1662	-0.791	9.927	1.872e-2	2.488e-2	12	33	16.4417	40.0853	AAL93777.1| Hypothetical protein
					31	32	31.0000	41.9778	
FN1663	-0.412	6.752				10		12.1471	AAL93778.1| Hypothetical protein
					9	9	9.0000	11.8062	
FN1666									AAL93781.1| Hypothetical protein
						3		3.9354	
FN1667					3		4.1104		AAL93782.1| dTDP-glucose 4,6-dehydratase
FN1668									AAL93783.1| Cholinephosphate cytidylyltransferase
					5		5.0000		

Log$_2$ Ratios: -6 -4 -2 0 2 4 6

Show detected proteins only
Show all proteins
Filter by category:
GO: amino acid transport

Proteins found: 1338

Enter (or paste) list of ORFs

Find ORFs

Test	Cutoff
q-Value	.005
p-Value	

	Signif	Direction	Applies To
red	yes	+	ratios, bars
yellow	no	n/a	bars
green	yes	–	ratios, bars
pink	yes	+	*p*- , *q*-Values
light green	yes	–	

Dot Plots Dot Plots

Protein	FnPg vs Fn Log$_2$ Ratio	Log$_2$ Sum	*q*-Value	*p*-Value	Raw *FnPg*	Raw *Fn*	Normalized *FnPg*	Normalized *Fn*	Description
FN1670	-1.175	11.013	1.198e-3	6.347e-4	23	52	31.5132	63.1647	AAL93785.1| Choline kinase
					29	56	29.0000	73.4611	
FN1671						5		6.0735	AAL93786.1| hypothetical exported protein
						4		5.2472	
FN1679	0.384	17.222	8.534e-2	1.851e-1	249	258	341.1648	313.3942	AAL93794.1| LPS biosynthesis protein WbpG
					552	283	552.0000	371.2409	
FN1683	-1.996	11.806	1.071e-4	1.503e-5	16	91	21.9222	110.5383	AAL93798.1| Acetyltransferase
					38	98	38.0000	128.5569	
FN1684	0.315	16.924	1.126e-1	2.633e-1	358	257	490.5101	312.1795	AAL93799.1| N-acetylneuraminate synthase
					296	244	296.0000	320.0805	
FN1685	-0.127	12.373	2.538e-1	7.584e-1	74	54	101.3903	65.5941	AAL93800.1| dTDP-4-dehydrorhamnose reductase
					38	66	38.0000	86.5791	
FN1686	0.226	15.857	2.062e-1	5.79e-1	270	182	369.9378	221.0765	AAL93801.1| Spore coat polysaccharide biosynthesis protein spsF
					157	175	157.0000	229.5659	
FN1687	-0.251	15.505	7.081e-2	1.448e-1	147	221	201.4106	268.4501	AAL93802.1| Gluconate 5-dehydrogenase
					194	154	194.0000	202.0180	
FN1688	1.263	13.004	9.311e-2	2.07e-1	167	51	228.8134	61.9500	AAL93803.1| Oxidoreductase
					52	42	52.0000	55.0958	
FN1689	-0.982	17.502	4.804e-5	4.446e-6	241	484	330.2037	587.9178	AAL93804.1| UDP-N-acetylglucosamine 4,6-dehydratase
					283	475	283.0000	623.1075	
FN1690	-0.698	11.765	3.028e-2	4.573e-2	45	60	61.6563	72.8824	AAL93805.1| Hypothetical protein
					31	59	31.0000	77.3965	
FN1692	-1.382	11.280	6.028e-4	2.042e-4	21	71	28.7729	86.2441	AAL93807.1| Glycosyl transferase
					33	57	33.0000	74.7729	
FN1693	-0.295	8.291	1.261e-1	3.04e-1	8	15	10.9611	18.2206	AAL93808.1| Hypothetical protein
					21	16	21.0000	20.9889	
FN1694	-1.263	12.735	6.092e-3	5.943e-3	53	108	72.6174	131.1883	AAL93809.1| UDP-N-acetyl-D-quinovosamine 4-epimerase
					34	95	34.0000	124.6215	
FN1695	-1.327	8.752	3.036e-3	2.28e-3	6	25	8.2208	30.3677	AAL93810.1| Probable quinovosaminephosphotransferae
					18	27	18.0000	35.4187	
FN1696	-0.164	14.848	1.569e-1	4.028e-1	93	147	127.4230	178.5618	AAL93811.1| UDP-N-acetylglucosamine 4,6-dehydratase
					197	141	197.0000	184.9645	

Log$_2$ Ratios: -6 -4 -2 0 2 4 6

Show detected proteins only
Show all proteins
Filter by category:
GO: amino acid transport

Proteins found: 1338

Enter (or paste) list of ORFs

Find ORFs

Test	Cutoff
q-Value	.005
p-Value	

	Signif	Direction	Applies To
red	yes	+	ratios, bars
yellow	no	n/a	bars
green	yes	−	ratios, bars
pink	yes	+	*p*-, *q*-Values
light green	yes	−	

Dot Plots Dot Plots

Protein	FnPg vs Fn Log$_2$ Ratio	Log$_2$ Sum	*q*-Value	*p*-Value	Raw *FnPg*	Raw *Fn*	Normalized *FnPg*	Normalized *Fn*	Description
FN1697	-1.012	14.080	1.832e-2	2.416e-2	36	136	49.3250	165.2000	AAL93812.1| Hypothetical protein
					136	159	136.0000	208.5770	
FN1698	0.660	13.812	6.211e-2	1.22e-1	77	88	105.5008	106.8941	AAL93813.1| dTDP-4-dehydrorhamnose reductase
					196	64	196.0000	83.9555	
FN1700									AAL93815.1| Hypothetical protein
						9		11.8062	
FN1701	-0.897	11.336	3.078e-3	2.325e-3	23	57	31.5132	69.2383	AAL93816.1| ABC transporter ATP-binding protein
					43	53	43.0000	69.5257	
FN1703	-0.139	14.496	2.479e-1	7.349e-1	56	133	76.7278	161.5559	AAL93818.1| ADP-L-glycero-D-manno-heptose-6-epimerase
					213	120	213.0000	157.4166	
FN1704	-1.213	7.629	9.445e-3	1.06e-2	4	18	5.4806	21.8647	AAL93819.1| Serine protease
					13	16	13.0000	20.9889	
FN1707						7		8.5029	AAL93822.1| Aldose 1-epimerase
						4		5.2472	
FN1708	-0.861	18.285	2.669e-5	1.839e-6	294	634	402.8211	770.1237	AAL93823.1| Polyribonucleotide nucleotidyltransferase
					436	574	436.0000	752.9762	
FN1711						14		17.0059	AAL93826.1| Methyltransferase
						12		15.7417	
FN1713	0.483	6.436							AAL93828.1| tRNA (Uracil-5-) -methyltransferase
					11	6	11.0000	7.8708	
FN1715									AAL93830.1| ATPase
					3		3.0000		
FN1717	-2.038	10.431	1.683e-4	2.964e-5	18	57	24.6625	69.2383	AAL93832.1| NAD-dependent DNA ligase
					12	62	12.0000	81.3319	
FN1718	-1.083	17.982	2.449e-4	5.252e-5	284	569	389.1197	691.1678	AAL93833.1| Protein translocase subunit secA
					310	602	310.0000	789.7068	
FN1719	-0.539	16.963	1.975e-3	1.266e-3	238	341	326.0933	414.2148	AAL93834.1| Hypothetical protein
					267	341	267.0000	447.3256	
FN1722						7		8.5029	AAL93837.1| Glucose inhibited division protein B
						4		5.2472	
FN1723	-1.008	12.769	4.075e-3	3.41e-3	40	86	54.8056	104.4647	AAL93838.1| Glucose inhibited division protein A
					63	101	63.0000	132.4923	

Log$_2$ Ratios: -6 -4 -2 0 2 4 6

Show detected proteins only
Show all proteins
Filter by category:
GO: amino acid transport

Proteins found: 1338

Enter (or paste) list of ORFs

Find ORFs

Test	Cutoff
q-Value	.005
p-Value	

Color	Signif	Direction	Applies To
red	yes	+	ratios, bars
yellow	no	n/a	bars
green	yes	−	ratios, bars
pink	yes	+	*p*- , *q*-Values
light green	yes	−	

Dot Plots Dot Plots

Protein	*FnPg vs Fn* Log$_2$ Ratio	Log$_2$ Sum	*q*-Value	*p*-Value	Raw *FnPg*	Raw *Fn*	Normalized *FnPg*	Normalized *Fn*	Description
FN1724	0.576	8.057			5	11	6.8507	13.3618	AAL93839.1| Potassium uptake protein KtrA
					33		33.0000		
FN1728	-0.386	8.629	1.038e-3	4.983e-4	13	18	17.8118	21.8647	AAL93843.1| Pyrrolidone-carboxylate peptidase
					17	18	17.0000	23.6125	
FN1730	-0.644	8.109	2.431e-2	3.463e-2	7	18	9.5910	21.8647	AAL93845.1| Para-aminobenzoate synthase component I
					17	15	17.0000	19.6771	
FN1731					7		9.5910		AAL93846.1| Anthranilate synthase component II
					8		8.0000		
FN1732	0.941	10.311	6.894e-2	1.399e-1	21	24	28.7729	29.1529	AAL93847.1| Hypothetical protein
					70	17	70.0000	22.3007	
FN1733									AAL93848.1| V-type sodium ATP synthase subunit D
					8		8.0000		
FN1734	0.411	13.885	1.689e-3	1.008e-3	99	86	135.6438	104.4647	AAL93849.1| V-type sodium ATP synthase subunit B
					148	83	148.0000	108.8798	
FN1735	0.738	12.791	8.608e-2	1.872e-1	47	49	64.3966	59.5206	AAL93850.1| V-type sodium ATP synthase subunit A
					153	54	153.0000	70.8375	
FN1736	1.129	8.712	3.761e-2	6.075e-2	15	12	20.5521	14.5765	AAL93851.1| V-type sodium ATP synthase subunit A
					40	10	40.0000	13.1181	
FN1737	-0.822	5.498	5.269e-2	9.624e-2	3	5	4.1104	6.0735	AAL93852.1| V-type sodium ATP synthase subunit G
					6	9	6.0000	11.8062	
FN1738	1.946	11.018	3.79e-2	6.138e-2	94	22	128.7931	26.7235	AAL93853.1| V-type sodium ATP synthase subunit C
					50	15	50.0000	19.6771	
FN1739	0.306	5.733			6		8.2208		AAL93854.1| V-type sodium ATP synthase subunit E
					8	5	8.0000	6.5590	
FN1740									AAL93855.1| V-type sodium ATP synthase subunit K
					185		185.0000		
FN1741	0.670	9.257	8.139e-3	8.846e-3	20	15	27.4028	18.2206	AAL93856.1| V-type sodium ATP synthase subunit I
					35	16	35.0000	20.9889	
FN1742					9		12.3313		AAL93857.1| V-type sodium ATP synthase subunit G
					8		8.0000		
FN1743									AAL93858.1| Multidrug-efflux transporter 2 regulator
					8		8.0000		

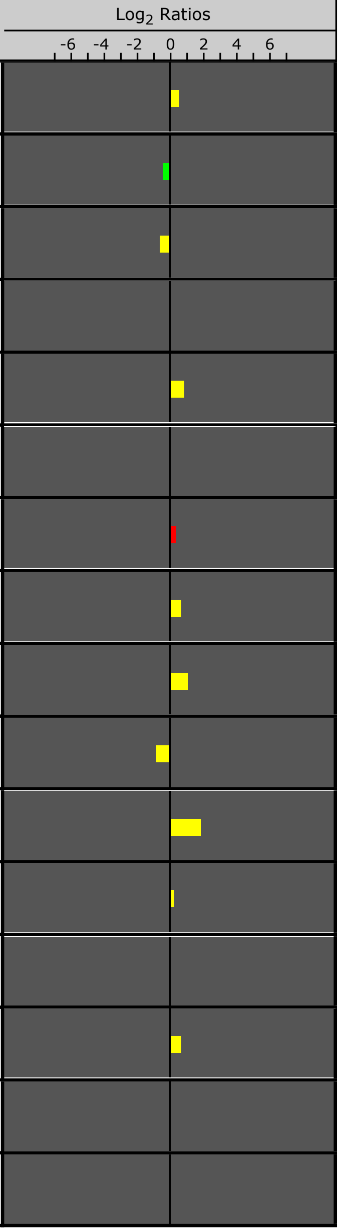

○ Show detected proteins only
○ Show all proteins
☐ Filter by category:
GO: amino acid transport

Proteins found: 1338

Enter (or paste) list of ORFs

Find ORFs

Test	Cutoff
q-Value	.005
p-Value	

	Signif	Direction	Applies To
red	yes	+	ratios, bars
yellow	no	n/a	bars
green	yes	−	ratios, bars
pink	yes	+	*p*- , *q*-Values
light green	yes	−	

Dot Plots Dot Plots

Protein	FnPg vs Fn: Log_2 Ratio	Log_2 Sum	*q*-Value	*p*-Value	Raw: *FnPg*	Raw: *Fn*	Normalized: *FnPg*	Normalized: *Fn*	Description
FN1745	2.072	7.098				4		4.8588	AAL93860.1| Cystathionine gamma-synthase
					24	5	24.0000	6.5590	
FN1746									AAL93861.1| Cystathionine beta-lyase
					7		7.0000		
FN1754					11		15.0715		AAL93869.1| Thiazole biosynthesis protein thiG
FN1763	1.959	10.009	1.247e-2	1.477e-2	34	16	46.5848	19.4353	AAL93876.1| Hypothetical cytosolic protein
					80	10	80.0000	13.1181	
FN1764	0.187	23.848	2.596e-1	7.817e-1	926	3002	1268.7495	3646.5481	AAL93877.1| Enolase
					7022	2773	7022.0000	3637.6360	
FN1765	-1.291	19.164	6.487e-3	6.485e-3	207	933	283.6189	1133.3209	AAL93878.1| Pyruvate kinase
					696	964	696.0000	1264.5803	
FN1780	-1.339	8.610	5.36e-3	4.992e-3	5	28	6.8507	34.0118	AAL93879.1| Hypothetical protein
					18	22	18.0000	28.8597	
FN1781	-0.691	18.542	1.231e-2	1.454e-2	433	666	593.2706	808.9943	AAL93880.1| LytB protein
					379	580	379.0000	760.8471	
FN1783									AAL93882.1| Ethanolamine utilization protein eutJ
					11		11.0000		
FN1784	-1.541	8.185				22		26.7235	AAL93883.1| unknown
					10	24	10.0000	31.4833	
FN1785	-1.171	8.572				32		38.8706	AAL93884.1| Hypothetical protein
					13	15	13.0000	19.6771	
FN1786	-1.607	10.856	5.276e-3	4.89e-3	9	60	12.3313	72.8824	AAL93885.1| ADP-heptose synthase
					37	59	37.0000	77.3965	
FN1787	-2.139	10.221	8.616e-4	3.704e-4	16	60	21.9222	72.8824	AAL93886.1| Tetratricopeptide repeat family protein
					11	55	11.0000	72.1493	
FN1788	0.089	9.167	2.6e-1	7.834e-1	12	21	16.4417	25.5088	AAL93887.1| 2C-methyl-D-erythritol 2,4-cyclodiphosphate synthase
					33	16	33.0000	20.9889	
FN1790	3.150	7.103			5		6.8507		AAL93889.1| Cob(I)alamin adenosyltransferase
					63	3	63.0000	3.9354	
FN1792	0.744	22.973	7.001e-2	1.427e-1	3669	1765	5027.0431	2143.9565	AAL93891.1| Hypothetical protein
					2401	1746	2401.0000	2290.4120	

Log_2 Ratios: -6 -4 -2 0 2 4 6

Show detected proteins only
Show all proteins
Filter by category: GO: amino acid transport

Proteins found: 1338

Enter (or paste) list of ORFs

Find ORFs

Test	Cutoff
q-Value	.005
p-Value	

	Signif	Direction	Applies To
red	yes	+	ratios, bars
yellow	no	n/a	bars
green	yes	−	ratios, bars
pink	yes	+	*p*-, *q*-Values
light green	yes	−	

Dot Plots | Dot Plots

Protein	FnPg vs Fn				Raw		Normalized		Description
	Log_2 Ratio	Log_2 Sum	*q*-Value	*p*-Value	*FnPg*	*Fn*	*FnPg*	*Fn*	
**FN1793**	0.340	14.830	2.264e-1	6.524e-1	38	131	52.0653	159.1265	AAL93892.1| Phosphoenolpyruvate-protein phosphotransferase
					332	110	332.0000	144.2986	
**FN1794**	-2.237	12.907	4.652e-4	1.373e-4	29	148	39.7341	179.7765	AAL93893.1| Phosphocarrier protein HPr
					41	153	41.0000	200.7062	
**FN1796**									AAL93895.1| unknown
						5		6.5590	
**FN1797**	-0.965	12.188	1.807e-2	2.372e-2	21	74	28.7729	89.8883	AAL93896.1| Spermidine/putrescine transport ATP-binding protein potA
					69	77	69.0000	101.0090	
**FN1798**	-0.529	7.173							AAL93897.1| Spermidine/putrescine transport system permease protein potB
					10	11	10.0000	14.4299	
**FN1800**	-1.164	14.275	1.624e-3	9.55e-4	57	172	78.0980	208.9295	AAL93899.1| Peptidyl-prolyl cis-trans isomerase
					110	162	110.0000	212.5125	
**FN1801**	1.297	8.419							AAL93900.1| Sodium/glutamate symport carrier protein
					29	9	29.0000	11.8062	
**FN1803**						7		8.5029	AAL93902.1| Transcriptional regulator, TetR family
**FN1804**	0.733	8.186				11		13.3618	AAL93903.1| Aminoacyl-histidine dipeptidase
					22	10	22.0000	13.1181	
**FN1807**	-0.405	17.120	4.755e-4	1.426e-4	251	348	343.9051	422.7178	AAL93906.1| Hypothetical protein
					312	340	312.0000	446.0138	
**FN1808**	-3.354	10.524				93		112.9677	AAL93907.1| Hypothetical protein
					12	101	12.0000	132.4923	
**FN1809**	0.046	8.944	2.993e-1	9.525e-1	3	23	4.1104	27.9382	AAL93908.1| Iron/zinc/copper-binding protein
					41	12	41.0000	15.7417	
**FN1811**	-1.950	10.824	6.988e-4	2.593e-4	9	73	12.3313	88.6736	AAL93910.1| Manganese transport system ATP-binding protein mntA
					31	60	31.0000	78.7083	
**FN1812**	-0.764	14.623	7.852e-2	1.657e-1	148	167	202.7807	202.8559	AAL93911.1| Manganese-binding protein
					41	161	41.0000	211.2006	
**FN1813**	-2.125	8.768				34		41.3000	AAL93912.1| Manganese-binding protein
					10	35	10.0000	45.9132	
**FN1814**						29		35.2265	AAL93913.1| Hypothetical protein
						27		35.4187	

Log_2 Ratios: -6 -4 -2 0 2 4 6

Show detected proteins only
Show all proteins
Filter by category:
GO: amino acid transport

Proteins found: 1338

Enter (or paste) list of ORFs

Find ORFs

Test	Cutoff
q-Value	.005
p-Value	

	Signif	Direction	Applies To
red	yes	+	ratios, bars
yellow	no	n/a	bars
green	yes	–	ratios, bars
pink	yes	+	*p*- , *q*-Values
light green	yes	–	

Dot Plots Dot Plots

Protein	FnPg vs Fn Log$_2$ Ratio	Log$_2$ Sum	*q*-Value	*p*-Value	Raw *FnPg*	Raw *Fn*	Normalized *FnPg*	Normalized *Fn*	Description	Log$_2$ Ratios (-6 -4 -2 0 2 4 6)
FN1816	0.093	6.397	2.275e-1	6.566e-1	8	6	10.9611	7.2882	AAL93915.1| unknown	
					8	8	8.0000	10.4944		
FN1817	-0.378	5.575			3		4.1104		AAL93916.1| Hemolysin	
					8	6	8.0000	7.8708		
FN1819						8		9.7176	AAL93918.1| Export ABC transporter	
						12		15.7417		
FN1820						8		9.7176	AAL93919.1| Export ABC transporter	
						6		7.8708		
FN1822						16		19.4353	AAL93921.1| Flavodoxin	
						10		13.1181		
FN1826	-1.242	11.001	8.325e-4	3.482e-4	24	52	32.8834	63.1647	AAL93925.1| Protease	
					26	58	26.0000	76.0847		
FN1827	-0.457	11.493	5.739e-2	1.087e-1	26	42	35.6236	51.0177	AAL93926.1| Replicative DNA helicase	
					56	57	56.0000	74.7729		
FN1828	-0.343	13.069	1.914e-1	5.278e-1	99	79	135.6438	95.9618	AAL93927.1| LSU ribosomal protein L9P	
					29	86	29.0000	112.8153		
FN1830	0.319	10.276	1.633e-1	4.251e-1	18	26	24.6625	31.5824	AAL93929.1| DNA polymerase III subunits gamma and tau	
					54	24	54.0000	31.4833		
FN1831	0.505	12.687	8.971e-3	9.957e-3	77	55	105.5008	66.8088	AAL93930.1| Nitrogen assimilation regulatory protein	
					88	53	88.0000	69.5257		
FN1834	-1.228	10.793	3.924e-4	1.042e-4	19	50	26.0327	60.7353	AAL93933.1| Biopolymer transport exbB protein	
					29	52	29.0000	68.2139		
FN1836	-1.370	12.374	6.756e-4	2.451e-4	26	100	35.6236	121.4706	AAL93935.1| Tetratricopeptide repeat family protein	
					55	86	55.0000	112.8153		
FN1839	1.968	16.740	3.183e-2	4.881e-2	286	135	391.8600	163.9853	AAL93938.1| Glycerol kinase	
					917	130	917.0000	170.5347		
FN1840	1.484	11.328	4.759e-3	4.259e-3	53	24	72.6174	29.1529	AAL93939.1| Dihydroxyacetone kinase	
					97	24	97.0000	31.4833		
FN1841	-0.413	8.863	1.55e-1	3.963e-1	20	14	27.4028	17.0059	AAL93940.1| Dihydroxyacetone kinase	
					10	25	10.0000	32.7951		
FN1842	-0.169	9.365	2.024e-1	5.658e-1	12	20	16.4417	24.2941	AAL93941.1| Dihydroxyacetone kinase phosphotransfer protein	
					32	23	32.0000	30.1715		

Show detected proteins only
Show all proteins
Filter by category:
GO: amino acid transport

Proteins found: 1338

Enter (or paste) list of ORFs

Find ORFs

Test	Cutoff
q-Value	.005
p-Value	

	Signif	Direction	Applies To
(red)	yes	+	ratios, bars
(yellow)	no	n/a	bars
(green)	yes	–	ratios, bars
(pink)	yes	+	*p*-, *q*-Values
(light green)	yes	–	

Dot Plots Dot Plots

Protein	FnPg vs Fn: Log_2 Ratio	Log_2 Sum	*q*-Value	*p*-Value	Raw *FnPg*	Raw *Fn*	Normalized *FnPg*	Normalized *Fn*	Description
**FN1843**					3		4.1104		AAL93942.1| Surface antigen
**FN1844**	-1.392	6.035							AAL93943.1| Ketoacyl reductase hetN
					5	10	5.0000	13.1181	
**FN1847**	0.729	7.165	1.321e-1	3.225e-1	5	11	6.8507	13.3618	AAL93946.1| DTDP-4-dehydrorhamnose 3,5-epimerase
					24	4	24.0000	5.2472	
**FN1849**	-0.266	9.436				27		32.7971	AAL93948.1| Coenzyme F390 synthetase
					24	19	24.0000	24.9243	
**FN1850**	2.654	8.516				5		6.0735	AAL93949.1| 3-oxoacyl-[acyl-carrier-protein] synthase III
					48	7	48.0000	9.1826	
**FN1851**	0.490	12.612	1.857e-1	5.072e-1	23	57	31.5132	69.2383	AAL93950.1| Ribonuclease PH
					156	49	156.0000	64.2785	
**FN1852**	-0.404	10.445	8.45e-2	1.828e-1	16	34	21.9222	41.3000	AAL93951.1| unknown
					43	34	43.0000	44.6014	
**FN1853**	0.477	8.050	1.379e-1	3.407e-1	20	13	27.4028	15.7912	AAL93952.1| Methylaspartate mutase
					11	9	11.0000	11.8062	
**FN1854**	-0.562	11.028	1.92e-2	2.572e-2	33	45	45.2146	54.6618	AAL93953.1| Methylaspartate mutase
					30	43	30.0000	56.4076	
**FN1855**									AAL93954.1| Methylaspartate mutase
					4		4.0000		
**FN1856**	-0.155	23.372	4.956e-2	8.848e-2	2099	2855	2875.9235	3467.9863	AAL93955.1| Butyrate-acetoacetate CoA-transferase subunit B
					3369	2657	3369.0000	3485.4666	
**FN1857**	0.801	19.791	1.135e-4	1.655e-5	897	633	1229.0154	768.9090	AAL93956.1| Acetoacetate: butyrate/acetate coenzyme A transferase
					1285	514	1285.0000	674.2679	
**FN1858**	-0.870	11.593			30	60	41.1042	72.8824	AAL93957.1| Short-chain fatty acids transporter
						59		77.3965	
**FN1859**	-0.003	24.501	3.09e-1	9.971e-1	443	4473	606.9720	5433.3809	AAL93958.1| Major outer membrane protein
					9128	3294	9128.0000	4321.0866	
**FN1860**	1.392	10.825				26		31.5824	AAL93959.1| NA+/H+ antiporter NHAC
					69	16	69.0000	20.9889	
**FN1862**	-0.052	15.804	2.805e-1	8.691e-1	232	211	317.8724	256.3030	AAL93961.1| L-beta-lysine 5,6-aminomutase beta subunit
					152	176	152.0000	230.8777	

Log_2 Ratios: -6 -4 -2 0 2 4 6

Show detected proteins only
Show all proteins
Filter by category:
GO: amino acid transport

Proteins found: 1338

Enter (or paste) list of ORFs

Find ORFs

Test	Cutoff
q-Value	.005
p-Value	

	Signif	Direction	Applies To
red	yes	+	ratios, bars
yellow	no	n/a	bars
green	yes	−	ratios, bars
pink	yes	+	*p*- , *q*-Values
light green	yes	−	

Dot Plots Dot Plots

Fn Summary Table | FnPg vs Fn | FnSg vs Fn | FnPgSg vs Fn | FnPgSg vs FnPg | FnSg vs FnPg | FnPgSg vs FnSg | Fn Coverage

Protein	FnPg vs Fn Log$_2$ Ratio	FnPg vs Fn Log$_2$ Sum	FnPg vs Fn *q*-Value	FnPg vs Fn *p*-Value	Raw *FnPg*	Raw *Fn*	Normalized *FnPg*	Normalized *Fn*	Description
FN1863	1.287	17.930	1.008e-1	2.297e-1	957	246	1311.2238	298.8177	AAL93962.1| L-beta-lysine 5,6-aminomutase alpha subunit
					250	260	250.0000	341.0694	
FN1864	-0.800	8.341	8.261e-2	1.774e-1	17	24	23.2924	29.1529	AAL93963.1| DNA mismatch repair protein mutS
					4	14	4.0000	18.3653	
FN1865						4		4.8588	AAL93964.1| Hypothetical protein
FN1866	-0.479	20.901	5.418e-2	1.001e-1	1117	1326	1530.4462	1610.7004	AAL93965.1| Lysine 2,3-aminomutase
					840	1291	840.0000	1693.5406	
FN1867	0.241	19.771	2.013e-1	5.62e-1	1058	736	1449.6080	894.0238	AAL93966.1| Zn-dependent alcohol dehydrogenases and related dehydrogenases
					607	645	607.0000	846.1144	
FN1868	-0.951	14.428	3.495e-3	2.773e-3	96	179	131.5334	217.4324	AAL93967.1| Hypothetical cytosolic protein
					82	149	82.0000	195.4590	
FN1869	-0.432	15.372	7.765e-2	1.633e-1	172	180	235.6641	218.6471	AAL93968.1| Hypothetical protein
					119	198	119.0000	259.7374	
FN1870									AAL93969.1| unknown
						6		7.8708	
FN1871						9		10.9324	AAL93970.1| unknown
						9		11.8062	
FN1872						17		20.6500	AAL93971.1| unknown
						19		24.9243	
FN1873	-1.080	11.153	5.665e-4	1.858e-4	26	57	35.6236	69.2383	AAL93972.1| Bis(5'-nucleosyl)-tetraphosphatase
					30	53	30.0000	69.5257	
FN1874	-1.394	10.062	5.462e-3	5.12e-3	9	43	12.3313	52.2324	AAL93973.1| Ribose 5-phosphate isomerase
					28	41	28.0000	53.7840	
FN1875	0.043	14.147	2.717e-1	8.319e-1	112	131	153.4557	159.1265	AAL93974.1| Peptidyl-prolyl cis-trans isomerase
					120	81	120.0000	106.2562	
FN1878						11		13.3618	AAL93977.1| unknown
						9		11.8062	
FN1879									AAL93978.1| SSU ribosomal protein S20P
						33		43.2896	
FN1880	1.807	12.789	2.521e-6	1.122e-7	118	33	161.6765	40.0853	AAL93979.1| Oxygen-insensitive NAD(P)H nitroreductase
					153	38	153.0000	49.8486	

Log$_2$ Ratios

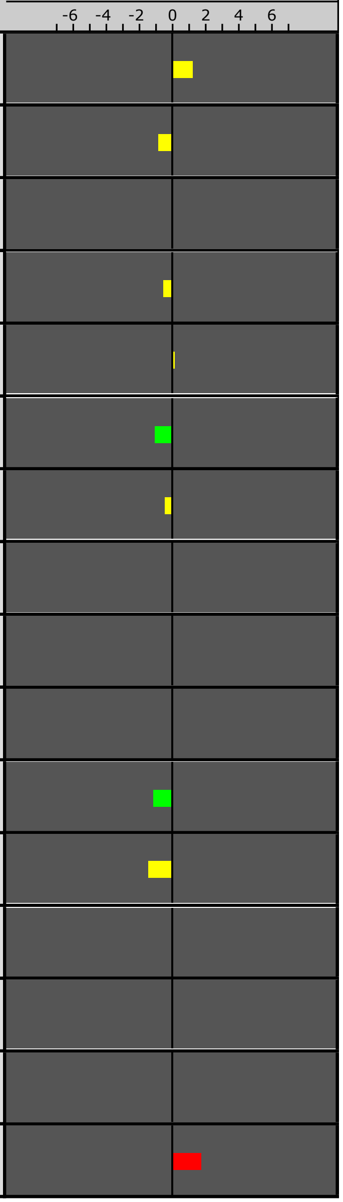

Show detected proteins only
Show all proteins
Filter by category:
GO: amino acid transport

Proteins found: 1338

Enter (or paste) list of ORFs

Find ORFs

Test	Cutoff
q-Value	.005
p-Value	

	Signif	Direction	Applies To
red	yes	+	ratios, bars
yellow	no	n/a	bars
green	yes	−	ratios, bars
pink	yes	+	*p*- , *q*-Values
light green	yes	−	

Dot Plots Dot Plots

Protein	FnPg vs Fn Log$_2$ Ratio	FnPg vs Fn Log$_2$ Sum	FnPg vs Fn *q*-Value	FnPg vs Fn *p*-Value	Raw *FnPg*	Raw *Fn*	Normalized *FnPg*	Normalized *Fn*	Description
FN1881									AAL93980.1| Esterase
					10		10.0000		
FN1884	-0.444	11.353			32	55	43.8445	66.8088	AAL93983.1| unknown
						40		52.4722	
FN1890	2.010	7.817			22	8	30.1431	9.7176	AAL93989.1| Hypothetical protein
						4		5.2472	
FN1891	0.939	8.361	1.275e-1	3.085e-1	6	14	8.2208	17.0059	AAL93990.1| Glycerophosphoryl diester phosphodiesterase
					42	7	42.0000	9.1826	
FN1893	-0.583	18.694	1.202e-4	1.828e-5	389	648	532.9844	787.1296	AAL93991.1| Fusobacterium outer membrane protein family
					531	615	531.0000	806.7602	
FN1898	-1.157	10.576	1.509e-2	1.872e-2	9	55	12.3313	66.8088	AAL93997.1| Sugar transport ATP-binding protein
					40	38	40.0000	49.8486	
FN1899	0.060	15.420	2.563e-1	7.682e-1	123	181	168.5272	219.8618	AAL93998.1| Hypothetical lipoprotein
					259	145	259.0000	190.2118	
FN1902	-0.028	10.100	2.452e-1	7.242e-1	26	27	35.6236	32.7971	AAL94001.1| Deoxycytidylate deaminase
					30	26	30.0000	34.1069	
FN1903	-2.264	9.805	3.089e-3	2.337e-3	17	55	23.2924	66.8088	AAL94002.1| Coenzyme A disulfide reductase/ disulfide bond regulator domain
					4	49	4.0000	64.2785	
FN1905									AAL94004.1| outer membrane protein
						3		3.9354	
FN1906	0.592	17.159	8.944e-2	1.965e-1	222	257	304.1710	312.1795	AAL94005.1| Cytosol aminopeptidase
					635	237	635.0000	310.8979	
FN1908	0.384	16.395	2.985e-2	4.49e-2	213	233	291.8398	283.0265	AAL94007.1| Glycerophosphoryl diester phosphodiesterase
					379	176	379.0000	230.8777	
FN1909	-0.951	13.699	1.873e-3	1.17e-3	51	129	69.8771	156.6971	AAL94008.1| UDP-3-O-[3-hydroxymyristoyl] glucosamine N-acyltransferase
					96	125	96.0000	163.9757	
FN1910	-1.075	15.909	2.784e-3	2.019e-3	148	257	202.7807	312.1795	AAL94009.1| periplasmic protein
					139	311	139.0000	407.9714	
FN1911	0.539	21.645	2.035e-2	2.771e-2	1822	1318	2496.3948	1600.9828	AAL94010.1| Outer membrane protein
					1869	1070	1869.0000	1403.6316	
FN1912	-1.505	8.335	6.81e-4	2.483e-4	9	25	12.3313	30.3677	AAL94011.1| Hypothetical protein
					9	23	9.0000	30.1715	

Log$_2$ Ratios: -6 -4 -2 0 2 4 6

Show detected proteins only
Show all proteins
Filter by category:
GO: amino acid transport

Proteins found: 1338

Enter (or paste) list of ORFs

Find ORFs

Test	Cutoff
q-Value	.005
p-Value	

	Signif	Direction	Applies To
red	yes	+	ratios, bars
yellow	no	n/a	bars
green	yes	−	ratios, bars
pink	yes	+	*p*- , *q*-Values
light green	yes	−	

Dot Plots Dot Plots

Protein	FnPg vs Fn Log$_2$ Ratio	Log$_2$ Sum	q-Value	p-Value	Raw FnPg	Raw Fn	Normalized FnPg	Normalized Fn	Description
FN1913	-0.697	12.644	4.043e-2	6.692e-2	64	77	87.6890	93.5324	AAL94012.1| hydrolase (HD superfamily)
					38	84	38.0000	110.1916	
FN1914	0.655	13.979	1.845e-1	5.025e-1	213	76	291.8398	92.3177	AAL94013.1| Anti-sigma F factor antagonist
					27	84	27.0000	110.1916	
FN1917	0.608	4.561							AAL94016.1| tRNA delta(2)-isopentenylpyrophosphate transferase
					6	3	6.0000	3.9354	
FN1918	-0.558	11.698	7.937e-2	1.682e-1	19	59	26.0327	71.6677	AAL94017.1| SPO0B-associated GTP-binding protein
					69	52	69.0000	68.2139	
FN1919	-2.878	8.684	3.404e-5	2.617e-6	8	43	10.9611	52.2324	AAL94018.1| Methyltransferase
					4	44	4.0000	57.7194	
FN1920					7		9.5910		AAL94019.1| tRNA (5-methylaminomethyl-2-thiouridylate) -methyltransferase
FN1922	0.598	7.441	2.103e-6	7.977e-8	12	9	16.4417	10.9324	AAL94021.1| Hypothetical protein
					16	8	16.0000	10.4944	
FN1923						4		4.8588	AAL94022.1| Adenine-specific methyltransferase
FN1925									AAL94024.1| Arsenical pump membrane protein
					6		6.0000		
FN1926	0.597	12.116	1.235e-1	2.962e-1	32	46	43.8445	55.8765	AAL94025.1| Nitrogen regulatory IIA protein
					120	40	120.0000	52.4722	
FN1927	0.658	14.381	9.963e-2	2.262e-1	78	104	106.8709	126.3294	AAL94026.1| DEGV protein
					260	81	260.0000	106.2562	
FN1928	1.094	8.521							AAL94027.1| Transcriptional regulator, MerR family
					28	10	28.0000	13.1181	
FN1929	-0.664	11.498	1.087e-1	2.519e-1	12	38	16.4417	46.1588	AAL94028.1| Competence-damage protein cinA
					69	68	69.0000	89.2028	
FN1931	0.294	5.321				4		4.8588	AAL94030.1| Protease
					7	5	7.0000	6.5590	
FN1933	-1.191	6.775	1.782e-3	1.088e-3	5	12	6.8507	14.5765	AAL94032.1| Hypothetical protein
					7	13	7.0000	17.0535	
FN1935	-0.856	6.026				6		7.2882	AAL94034.1| Adenine-specific methyltransferase
					6	11	6.0000	14.4299	

Log$_2$ Ratios: -6 -4 -2 0 2 4 6

Show detected proteins only
Show all proteins
Filter by category:
GO: amino acid transport

Proteins found: 1338

Enter (or paste) list of ORFs

Find ORFs

Test	Cutoff
q-Value	.005
p-Value	

	Signif	Direction	Applies To
red	yes	+	ratios, bars
yellow	no	n/a	bars
green	yes	−	ratios, bars
pink	yes	+	p- , q-Values
light green	yes	−	

Dot Plots Dot Plots

Protein	FnPg vs Fn Log$_2$ Ratio	Log$_2$ Sum	*q*-Value	*p*-Value	Raw *FnPg*	Raw *Fn*	Normalized *FnPg*	Normalized *Fn*	Description
FN1939	0.385	8.111				11		13.3618	AAL94038.1| Hypothetical protein
					19	12	19.0000	15.7417	
FN1941	1.481	15.151	1.812e-4	3.292e-5	239	94	327.4634	114.1824	AAL94040.1| ClpB protein
					310	87	310.0000	114.1271	
FN1942	1.174	5.735			8	4	10.9611	4.8588	AAL94041.1| putative DNA-binding protein
FN1943	2.132	22.304	3.643e-3	2.929e-3	4000	929	5480.5594	1128.4621	AAL94042.1| Tryptophanase
					4049	797	4049.0000	1045.5088	
FN1948									AAL94044.1| Hypothetical protein
					7		7.0000		
FN1949	2.365	9.195	2.717e-2	3.982e-2	24	10	32.8834	12.1471	AAL94045.1| Xaa-Pro dipeptidase
					77	7	77.0000	9.1826	
FN1950						55		66.8088	AAL94046.1| Serine protease
						43		56.4076	
FN1951						3		3.6441	AAL94047.1| ATPase associated with chromosome architecture/replication
						8		10.4944	
FN1956									AAL94052.1| Hypothetical protein
					15		15.0000		
FN1964	-0.609	9.042	5.524e-5	5.711e-6	14	24	19.1820	29.1529	AAL94054.1| O-linked GLCNAC transferase
					18	21	18.0000	27.5479	
FN1965	0.486	10.923	3.23e-2	4.977e-2	44	30	60.2862	36.4412	AAL94055.1| Tetratricopeptide repeat family protein
					44	29	44.0000	38.0424	
FN1966	-0.851	10.944	7.543e-3	7.998e-3	30	42	41.1042	51.0177	AAL94056.1| Hypothetical protein
					25	52	25.0000	68.2139	
FN1970						4		4.8588	AAL94060.1| Hemin-binding periplasmic protein hmuT precursor
						5		6.5590	
FN1971	0.778	9.641				15		18.2206	AAL94061.1| Hemin receptor
					37	19	37.0000	24.9243	
FN1972	2.277	9.584				11		13.3618	AAL94062.1| unknown
					61	9	61.0000	11.8062	
FN1973	2.182	12.026	1.656e-3	9.815e-4	111	24	152.0855	29.1529	AAL94063.1| Translation initiation inhibitor
					123	24	123.0000	31.4833	

Log$_2$ Ratios: -6 -4 -2 0 2 4 6

Show detected proteins only
Show all proteins
Filter by category:
GO: amino acid transport

Proteins found: 1338

Enter (or paste) list of ORFs

Find ORFs

Test	Cutoff
q-Value	.005
p-Value	

	Signif	Direction	Applies To
red	yes	+	ratios, bars
yellow	no	n/a	bars
green	yes	−	ratios, bars
pink	yes	+	*p*- , *q*-Values
light green	yes	−	

Dot Plots Dot Plots

Protein	FnPg vs Fn: Log_2 Ratio	Log_2 Sum	*q*-Value	*p*-Value	Raw: FnPg	Raw: Fn	Normalized: FnPg	Normalized: Fn	Description
FN1974						6		7.2882	AAL94064.1| DNA/RNA helicase (DEAD/DEAH BOX family)
						8		10.4944	
FN1975	-1.400	13.702	4.849e-4	1.476e-4	49	164	67.1369	199.2118	AAL94065.1| ATP-dependent RNA helicase
					75	134	75.0000	175.7819	
FN1976	-0.800	10.426	7.915e-2	1.675e-1	6	46	8.2208	55.8765	AAL94066.1| 4-amino-4-deoxychorismate lyase
					48	32	48.0000	41.9778	
FN1977	0.237	6.286			7	8	9.5910	9.7176	AAL94067.1| Cell cycle protein MesJ
						5		6.5590	
FN1978	-1.935	13.741	2.614e-3	1.852e-3	18	178	24.6625	216.2177	AAL94068.1| Cell division protein ftsH
					95	184	95.0000	241.3722	
FN1979	-2.888	10.456	2.983e-4	7.006e-5	15	75	20.5521	91.1030	AAL94069.1| SSU ribosomal protein S15P
					7	86	7.0000	112.8153	
FN1983	-1.070	16.582	6.53e-4	2.317e-4	139	380	190.4494	461.5884	AAL94073.1| Alkyl hydroperoxide reductase C22 protein
					242	340	242.0000	446.0138	
FN1984	0.205	16.782	1.926e-1	5.319e-1	348	276	476.8087	335.2589	AAL94074.1| Thioredoxin reductase
					244	221	244.0000	289.9090	
FN1985	-0.583	12.690	1.336e-1	3.273e-1	13	72	17.8118	87.4588	AAL94075.1| Inner membrane protein
					115	85	115.0000	111.5034	
FN1986	-0.000	16.421	3.094e-1	9.99e-1	255	248	349.3857	301.2471	AAL94076.1| Hypothetical protein
					243	222	243.0000	291.2208	
FN1987	2.122	7.705			22	6	30.1431	7.2882	AAL94077.1| Transcriptional regulator, GntR family
						5		6.5590	
FN1988	3.596	15.052	3.588e-2	5.713e-2	221	43	302.8009	52.2324	AAL94078.1| Tyrosine phenol-lyase
					979	41	979.0000	53.7840	
FN1989									AAL94079.1| Sodium-dependent tyrosine transporter
					16		16.0000		
FN1991	0.141	17.361	4.567e-2	7.922e-2	334	326	457.6267	395.9942	AAL94081.1| Glucosamine-1-phosphate acetyltransferase
					404	294	404.0000	385.6708	
FN1992	0.581	16.276	1.639e-2	2.083e-2	219	187	300.0606	227.1501	AAL94082.1| Ribose-phosphate pyrophosphokinase
					389	178	389.0000	233.5013	
FN1993	-0.018	5.187				5		6.0735	AAL94083.1| SUA5 protein
					6		6.0000		

Log_2 Ratios: -6 -4 -2 0 2 4 6

Show detected proteins only
Show all proteins
Filter by category:
GO: amino acid transport

Proteins found: 1338

Enter (or paste) list of ORFs

Find ORFs

Test	Cutoff
q-Value	.005
p-Value	

Signif	Direction	Applies To
yes	+	ratios, bars
no	n/a	bars
yes	–	ratios, bars
yes	+	*p*-, *q*-Values
yes	–	

Dot Plots | Dot Plots

Protein	FnPg vs Fn Log$_2$ Ratio	FnPg vs Fn Log$_2$ Sum	FnPg vs Fn *q*-Value	FnPg vs Fn *p*-Value	Raw *FnPg*	Raw *Fn*	Normalized *FnPg*	Normalized *Fn*	Description
FN1994	-0.089	11.457	2.786e-1	8.611e-1	59	49	80.8383	59.5206	AAL94084.1| Hypothetical protein
					22	38	22.0000	49.8486	
FN1995	-1.152	5.152				6		7.2882	AAL94085.1| Hypothetical protein
					4	8	4.0000	10.4944	
FN2001	0.901	7.743				9		10.9324	AAL94091.1| Hypothetical protein
					20	8	20.0000	10.4944	
FN2007	-0.841	8.192	5.876e-2	1.124e-1	15	15	20.5521	18.2206	AAL94097.1| Glutathione peroxidase
					5	21	5.0000	27.5479	
FN2008	2.271	6.224							AAL94098.1| Glycine betaine transport ATP-binding protein
					19	3	19.0000	3.9354	
FN2009									AAL94099.1| Glycine betaine transport system permease protein
					8		8.0000		
FN2011	-1.337	14.011	5.679e-3	5.396e-3	37	171	50.6952	207.7148	AAL94101.1| Valyl-tRNA synthetase
					111	153	111.0000	200.7062	
FN2013	-0.281	4.924				5		6.0735	AAL94103.1| GTP-binding protein
					5		5.0000		
FN2014	-1.417	15.225	3.257e-5	2.448e-6	77	275	105.5008	334.0442	AAL94104.1| ATP-dependent protease La
					134	233	134.0000	305.6506	
FN2015	-0.748	13.545	2.051e-3	1.34e-3	64	108	87.6890	131.1883	AAL94105.1| ATP-dependent clp protease ATP-binding subunit clpX
					81	116	81.0000	152.1694	
FN2016	-0.730	10.446				36		43.7294	AAL94106.1| ATP-dependent Clp protease proteolytic subunit
					29	40	29.0000	52.4722	
FN2017	-0.572	18.961	3.324e-2	5.171e-2	543	728	743.9859	884.3061	AAL94107.1| Trigger factor, ppiase
					428	654	428.0000	857.9206	
FN2018	-1.837	9.299	1.959e-3	1.25e-3	15	36	20.5521	43.7294	AAL94108.1| Single-stranded-DNA-specific exonuclease recJ
					6	39	6.0000	51.1604	
FN2019	-0.242	10.866			29	32	39.7341	38.8706	AAL94109.1| Ribosome-binding factor A
						42		55.0958	
FN2020	-0.269	16.155	7.182e-3	7.491e-3	190	242	260.3266	293.9589	AAL94110.1| Bacterial Protein Translation Initiation Factor 2 (IF-2)
					232	228	232.0000	299.0916	
FN2022	-1.021	12.451	1.198e-8	4.849e-11	38	88	52.0653	106.8941	AAL94112.1| N utilization substance protein A
					53	81	53.0000	106.2562	

Log$_2$ Ratios: -6 -4 -2 0 2 4 6

Show detected proteins only
Show all proteins
Filter by category:
GO: amino acid transport

Proteins found: 1338

Enter (or paste) list of ORFs

Find ORFs

Test	Cutoff
q-Value	.005
p-Value	

	Signif	Direction	Applies To
red	yes	+	ratios, bars
yellow	no	n/a	bars
green	yes	–	ratios, bars
pink	yes	+	*p*- , *q*-Values
light green	yes	–	*p*- , *q*-Values

Dot Plots Dot Plots

Protein	FnPg vs Fn Log$_2$ Ratio	FnPg vs Fn Log$_2$ Sum	FnPg vs Fn *q*-Value	FnPg vs Fn *p*-Value	Raw *FnPg*	Raw *Fn*	Normalized *FnPg*	Normalized *Fn*	Description
FN2023	0.590	7.446	1.739e-1	4.628e-1	20	8	27.4028	9.7176	AAL94113.1| Hypothetical cytosolic protein
					5	9	5.0000	11.8062	
FN2030	-2.984	13.505	3.26e-5	2.451e-6	18	265	24.6625	321.8971	AAL94115.1| Inorganic pyrophosphatase
					52	217	52.0000	284.6617	
FN2031	-1.652	6.561			4	10	5.4806	12.1471	AAL94116.1| Thiamine biosynthesis lipoprotein apbE
						17		22.3007	
FN2033	0.225	8.498			15	16	20.5521	19.4353	AAL94118.1| Guanylate kinase
						12		15.7417	
FN2034	-0.476	8.706	9.662e-2	2.172e-1	18	17	24.6625	20.6500	AAL94119.1| Protein yicC
					10	21	10.0000	27.5479	
FN2035	-0.356	18.018	2.15e-2	2.966e-2	375	490	513.8024	595.2060	AAL94120.1| DNA-directed RNA polymerase beta' chain
					397	435	397.0000	570.6353	
FN2036	-0.901	17.232	3.329e-3	2.596e-3	163	469	223.3328	569.6972	AAL94121.1| DNA-directed RNA polymerase beta chain
					351	383	351.0000	502.4214	
FN2037	0.290	19.709	2.004e-1	5.588e-1	1107	682	1516.7448	828.4296	AAL94122.1| LSU ribosomal protein L12P (L7/L12)
					531	645	531.0000	846.1144	
FN2038	-0.837	16.073	4.147e-2	6.929e-2	216	242	295.9502	293.9589	AAL94123.1| LSU ribosomal protein L10P
					97	311	97.0000	407.9714	
FN2039	-0.613	16.846	1.035e-3	4.962e-4	214	349	293.2099	423.9325	AAL94124.1| LSU ribosomal protein L1P
					262	324	262.0000	425.0249	
FN2040	-1.158	12.697	7.93e-3	8.544e-3	57	114	78.0980	138.4765	AAL94125.1| LSU ribosomal protein L11P
					31	80	31.0000	104.9444	
FN2041	0.560	12.042	1.587e-1	4.09e-1	91	47	124.6827	57.0912	AAL94126.1| Transcription antitermination protein nusG
					33	38	33.0000	49.8486	
FN2045									AAL94129.1| Ferric uptake regulation protein
					18		18.0000		
FN2046									AAL94130.1| Acetyltransferase
					24		24.0000		
FN2047	-3.129	16.328	1.148e-6	3.253e-8	54	685	73.9876	832.0738	AAL94131.1| Fusobacterium outer membrane protein family
					120	659	120.0000	864.4797	
FN2048	-0.332	15.532				198		240.5118	AAL94132.1| Outer membrane protein
					194	189	194.0000	247.9312	

Log$_2$ Ratios: -6 -4 -2 0 2 4 6

Show detected proteins only
Show all proteins
Filter by category:
GO: amino acid transport

Proteins found: 1338

Enter (or paste) list of ORFs

Find ORFs

Test	Cutoff
q-Value	.005
p-Value	

Color	Signif	Direction	Applies To
red	yes	+	ratios, bars
yellow	no	n/a	bars
green	yes	–	ratios, bars
pink	yes	+	*p*- , *q*-Values
light green	yes	–	

Dot Plots Dot Plots

Protein	FnPg vs Fn Log$_2$ Ratio	FnPg vs Fn Log$_2$ Sum	FnPg vs Fn *q*-Value	FnPg vs Fn *p*-Value	Raw *FnPg*	Raw *Fn*	Normalized *FnPg*	Normalized *Fn*	Description
**FN2049**	1.100	17.889	6.173e-2	1.209e-1	753	270	1031.7153	327.9707	AAL94133.1| unknown
					411	263	411.0000	345.0048	
**FN2050**	-0.361	15.648	1.844e-1	5.025e-1	240	195	328.8336	236.8677	AAL94134.1| Hypothetical membrane-spanning protein
					71	211	71.0000	276.7909	
**FN2051**	-0.589	13.778	8.361e-2	1.803e-1	106	113	145.2348	137.2618	AAL94135.1| unknown
					48	117	48.0000	153.4812	
**FN2052**	-1.626	14.269	5.543e-3	5.222e-3	89	210	121.9424	255.0883	AAL94136.1| unknown
					38	182	38.0000	238.7486	
**FN2053**	-0.050	12.119	2.835e-1	8.823e-1	30	61	41.1042	74.0971	AAL94137.1| Serine/threonine sodium symporter
					90	47	90.0000	61.6548	
**FN2054**	-0.665	15.074	8.638e-4	3.721e-4	100	195	137.0140	236.8677	AAL94138.1| Glucose-6-phosphate isomerase
					158	176	158.0000	230.8777	
**FN2058**	0.479	18.198	1.417e-1	3.525e-1	683	374	935.8055	454.3001	AAL94142.1| Fusobacterium outer membrane protein family
					359	362	359.0000	474.8735	
**FN2059**	-0.332	15.532				198		240.5118	AAL94143.1| Outer membrane protein
					194	189	194.0000	247.9312	
**FN2060**	1.100	17.889	6.173e-2	1.209e-1	753	270	1031.7153	327.9707	AAL94144.1| unknown
					411	263	411.0000	345.0048	
**FN2061**	-0.361	15.648	1.844e-1	5.025e-1	240	195	328.8336	236.8677	AAL94145.1| Hypothetical membrane-spanning protein
					71	211	71.0000	276.7909	
**FN2062**	-0.589	13.778	8.361e-2	1.803e-1	106	113	145.2348	137.2618	AAL94146.1| unknown
					48	117	48.0000	153.4812	
**FN2063**	-1.626	14.269	5.543e-3	5.222e-3	89	210	121.9424	255.0883	AAL94147.1| unknown
					38	182	38.0000	238.7486	
**FN2067**						7		8.5029	AAL94151.1| Thiol:disulfide interchange protein tlpA
						5		6.5590	
**FN2068**	1.581	7.165	9.753e-3	1.102e-2	12	6	16.4417	7.2882	AAL94152.1| dGTP triphosphohydrolase
					25	5	25.0000	6.5590	
**FN2069**									AAL94153.1| Amino acid carrier protein alsT
						4		5.2472	
**FN2070**	-0.830	7.749				16		19.4353	AAL94154.1| Cobyric acid synthase
					11	15	11.0000	19.6771	

Log$_2$ Ratios: -6 -4 -2 0 2 4 6

○ Show detected proteins only
○ Show all proteins
☐ Filter by category:
GO: amino acid transport

Proteins found: 1338

Enter (or paste) list of ORFs

Find ORFs

Test	Cutoff
q-Value	.005
p-Value	

	Signif	Direction	Applies To
red	yes	+	ratios, bars
yellow	no	n/a	bars
green	yes	−	ratios, bars
pink	yes	+	*p*- , *q*-Values
light green	yes	−	

Dot Plots | Dot Plots

Protein	FnPg vs Fn Log$_2$ Ratio	Log$_2$ Sum	*q*-Value	*p*-Value	Raw *FnPg*	Raw *Fn*	Normalized *FnPg*	Normalized *Fn*	Description
FN2073	0.466	9.147	1.814e-1	4.908e-1	8	15	10.9611	18.2206	AAL94157.1| Adenine phosphoribosyltransferase
					45	17	45.0000	22.3007	
FN2075	-1.552	10.261	2.351e-6	9.778e-8	16	48	21.9222	58.3059	AAL94159.1| Hypothetical protein
					19	47	19.0000	61.6548	
FN2078	-0.696	3.866				4		4.8588	AAL94162.1| Transcriptional regulator, DeoR family
					3		3.0000		
FN2081						20		24.2941	AAL94165.1| ABC transporter substrate-binding protein
						17		22.3007	
FN2082	0.066	20.838	2.795e-1	8.648e-1	599	1045	820.7138	1269.3680	AAL94166.1| Formate--tetrahydrofolate ligase
					1981	1073	1981.0000	1407.5671	
FN2093	-0.816	10.423	5.559e-2	1.038e-1	32	41	43.8445	49.8030	AAL94177.1| General secretion pathway protein G
					12	37	12.0000	48.5368	
FN2098					3		4.1104		AAL94182.1| MRP-family nucleotide-binding protein
					9		9.0000		
FN2100	-1.098	11.054	2.063e-3	1.352e-3	19	56	26.0327	68.0235	AAL94184.1| Hypothetical protein
					37	51	37.0000	66.9021	
FN2102	-0.535	8.617	5.344e-2	9.817e-2	16	22	21.9222	26.7235	AAL94186.1| ABC transporter ATP-binding protein
					11	16	11.0000	20.9889	
FN2103	-0.584	19.422	1.5e-2	1.858e-2	598	873	819.3436	1060.4385	AAL94187.1| tricarboxylate-binding protein
					550	756	550.0000	991.7248	
FN2105	-0.841	6.455				12		14.5765	AAL94189.1| tricarboxylate transport membrane protein RctA
					7	8	7.0000	10.4944	
FN2106	0.480	15.105	1.619e-1	4.202e-1	247	131	338.4245	159.1265	AAL94190.1| Transporter
					105	121	105.0000	158.7284	
FN2107	-1.519	11.505	5.936e-3	5.733e-3	37	66	50.6952	80.1706	AAL94191.1| Galactokinase
					13	78	13.0000	102.3208	
FN2108	-0.937	10.048	5.403e-3	5.046e-3	19	32	26.0327	38.8706	AAL94192.1| Galactose-1-phosphate uridylyltransferase
					21	39	21.0000	51.1604	
FN2109	0.357	12.048	5.849e-2	1.117e-1	63	45	86.3188	54.6618	AAL94193.1| UDP-glucose 4-epimerase
					61	46	61.0000	60.3430	
FN2116	-1.796	11.084				76		92.3177	AAL94200.1| Hypothetical exported 24-amino acid repeat protein
					25	62	25.0000	81.3319	

Log$_2$ Ratios: -6 -4 -2 0 2 4 6

Show detected proteins only
Show all proteins
Filter by category:
GO: amino acid transport

Proteins found: 1338

Enter (or paste) list of ORFs

Find ORFs

Test	Cutoff
q-Value	.005
p-Value	

	Signif	Direction	Applies To
red	yes	+	ratios, bars
yellow	no	n/a	bars
green	yes	−	ratios, bars
pink	yes	+	*p*-, *q*-Values
light green	yes	−	

Dot Plots Dot Plots

Protein	FnPg vs Fn: Log_2 Ratio	Log_2 Sum	*q*-Value	*p*-Value	Raw: *FnPg*	Raw: *Fn*	Normalized: *FnPg*	Normalized: *Fn*	Description
FN2117	-1.864	7.034				23		27.9382	AAL94201.1| Hypothetical exported 24-amino acid repeat protein
					6	12	6.0000	15.7417	
FN2118	-2.982	8.596				50		60.7353	AAL94202.1| Hypothetical exported 24-amino acid repeat protein
					7	38	7.0000	49.8486	
FN2119	0.059	10.437				32		38.8706	AAL94203.1| Hypothetical exported 24-amino acid repeat protein
					38	26	38.0000	34.1069	
FN2120						24		29.1529	AAL94204.1| Hypothetical exported 24-amino acid repeat protein
						30		39.3542	
FN2121	-1.562	13.957	1.544e-2	1.929e-2	94	171	128.7931	207.7148	AAL94205.1| Hypothetical exported 24-amino acid repeat protein
					18	172	18.0000	225.6305	
FN2122	0.322	17.106	6.198e-2	1.216e-1	356	280	487.7698	340.1177	AAL94206.1| Phenylalanyl-tRNA synthetase beta chain
					352	253	352.0000	331.8867	
FN2123	-0.043	13.279	2.912e-1	9.161e-1	39	77	53.4355	93.5324	AAL94207.1| Phenylalanyl-tRNA synthetase alpha chain
					143	83	143.0000	108.8798	
FN2125	-1.221	13.987	1.167e-2	1.362e-2	89	154	121.9424	187.0648	AAL94209.1| DNA gyrase subunit A
					45	154	45.0000	202.0180	
FN2126	-1.937	11.781	3.32e-4	8.134e-5	26	95	35.6236	115.3971	AAL94210.1| DNA gyrase subunit B
					25	89	25.0000	116.7507	
FN2128						3		3.6441	AAL94212.1| RECF protein

Log₂ Ratios: -6 -4 -2 0 2 4 6

Show detected proteins only
Show all proteins
Filter by category:
GO: amino acid transport

Proteins found: 1338

Enter (or paste) list of ORFs

Find ORFs

Test	Cutoff
q-Value	.005
p-Value	

Color	Signif	Direction	Applies To
red	yes	+	ratios, bars
yellow	no	n/a	bars
green	yes	−	ratios, bars
pink	yes	+	*p*-, *q*-Values
light green	yes	−	*p*-, *q*-Values

Dot Plots Dot Plots
