# Supplementary material for: Proteomics of Fusobacterium nucleatum within a model developing oral microbial community
Source: Microbiologyopen. 2014 Aug 25;3(5):729–51. doi: 10.1002/mbo3.204 (PMC4234264; doi:10.1002/mbo3.204)
Supplement: Table S5 — See description for Table S3. [file mbo30003-0729-sd7.pdf]

FnSg vs FnPg

Fusobacterium nucleatum

Hackett Laboratory UW

Fn Summary Table | FnPg vs Fn | FnSg vs Fn | FnPgSg vs Fn | FnPgSg vs FnPg | FnSg vs FnPg | FnPgSg vs FnSg | Fn Coverage

Protein	*FnSg* vs *FnPg* Log$_2$ Ratio	Log$_2$ Sum	*q*-Value	*p*-Value	Raw *FnPg*	Raw *FnSg*	Normalized *FnPg*	Normalized *FnSg*	Description
FN0001	0.140	7.675	3.783e-1	7.944e-1	15	10	21.9089	13.0162	AAL94214.1| Chromosomal replication initiator protein dnaA
					5	17	5.3301	17.0000	
FN0004	-0.430	10.984				35		45.5566	AAL94217.1| Inner membrane protein
					49	32	52.2349	32.0000	
FN0005	0.807	8.020	9.688e-2	8.044e-2	5	12	7.3030	15.6194	AAL94218.1| Jag protein
					16	27	17.0563	27.0000	
FN0006	0.389	9.358				22		28.6356	AAL94219.1| Thiophene and furan oxidation protein THDF
					21	30	22.3864	30.0000	
FN0007	0.497	7.357	1.598e-1	2.137e-1	6	8	8.7636	10.4129	AAL94220.1| Glucose inhibited division protein A
					12	20	12.7922	20.0000	
FN0008	-0.559	7.026				6		7.8097	AAL94221.1| Quinolinate synthetase A
					13	11	13.8583	11.0000	
FN0009	-0.306	9.594							AAL94222.1| L-aspartate oxidase
					29	25	30.9146	25.0000	
FN0017						4		5.2065	AAL94230.1| Hypothetical protein
						7		7.0000	
FN0018	-0.018	9.214				18		23.4291	AAL94231.1| Hypothetical protein
					23	25	24.5184	25.0000	
FN0019						6		7.8097	AAL94232.1| Transcription-repair coupling factor
						4		4.0000	
FN0022	-2.975	10.310	1.469e-1	1.814e-1	128	8	186.9562	10.4129	AAL94235.1| Hypothetical protein
					12	15	12.7922	15.0000	
FN0023	0.355	7.459							AAL94236.1| Short-chain fatty acids transporter
					11	15	11.7262	15.0000	
FN0024	0.113	8.227			6		8.7636		AAL94237.1| Hypothetical exported 24-amino acid repeat protein
					23	18	24.5184	18.0000	
FN0025	0.100	5.900							AAL94238.1| Hypothetical exported 24-amino acid repeat protein
					7	8	7.4621	8.0000	
FN0030	-0.171	13.696	2.765e-1	4.991e-1	63	90	92.0175	117.1455	AAL94243.1| 5-nitroimidazole antibiotic resistance protein
					143	100	152.4408	100.0000	
FN0031	-0.287	9.576	1.584e-1	2.104e-1	25	20	36.5149	26.0323	AAL94244.1| unknown
					23	24	24.5184	24.0000	

Log$_2$ Ratios: -6 -4 -2 0 2 4 6

Show detected proteins only
Show all proteins
Filter by category:
GO: amino acid transport

Proteins found: 1313

Enter (or paste) list of ORFs

Find ORFs

Test	Cutoff
q-Value	.005
p-Value	

Color	Signif	Direction	Applies To
red	yes	+	ratios, bars
yellow	no	n/a	bars
green	yes	−	ratios, bars
pink	yes	+	*p*- , *q*-Values
light green	yes	−	

Dot Plots | Dot Plots

FnSg vs FnPg

Fusobacterium nucleatum

Hackett Laboratory UW

Fn Summary Table | FnPg vs Fn | FnSg vs Fn | FnPgSg vs Fn | FnPgSg vs FnPg | FnSg vs FnPg | FnPgSg vs FnSg | Fn Coverage

Protein	*FnSg* vs *FnPg* Log$_2$ Ratio	Log$_2$ Sum	*q*-Value	*p*-Value	Raw *FnPg*	Raw *FnSg*	Normalized *FnPg*	Normalized *FnSg*	Description
FN0033	0.147	12.444	3.222e-1	6.206e-1	65	60	94.9387	78.0970	AAL94246.1| unknown
					44	79	46.9048	79.0000	
FN0034	-0.732	9.168	1.017e-1	8.763e-2	27	14	39.4361	18.2226	AAL94247.1| unknown
					21	19	22.3864	19.0000	
FN0038					77		112.4659		AAL94251.1| unknown
FN0039	0.489	7.593				13		16.9210	AAL94252.1| DNA primase (bacterial type) and small primase-like proteins
					11	16	11.7262	16.0000	
FN0040	0.402	18.632	2.871e-2	8.462e-3	341	549	498.0631	714.5875	AAL94253.1| Asparaginyl-tRNA synthetase
					573	751	610.8290	751.0000	
FN0041									AAL94254.1| unknown
					7		7.4621		
FN0043									AAL94256.1| Hypothetical exported 24-amino acid repeat protein
					8		8.5282		
FN0045									AAL94258.1| Shikimate 5-dehydrogenase
						7		7.0000	
FN0046					5		7.3030		AAL94259.1| 3-dehydroquinate dehydratase
FN0047	0.064	11.902	3.588e-1	7.305e-1	50	48	73.0298	62.4776	AAL94260.1| Exodeoxyribonuclease III
					45	64	47.9709	64.0000	
FN0048	0.902	8.370	7.266e-3	1.077e-3	8	19	11.6848	24.7307	AAL94261.1| 4-nitrophenylphosphatase
					14	25	14.9243	25.0000	
FN0049	1.366	10.425	1.414e-2	2.841e-3	9	40	13.1454	52.0647	AAL94262.1| Hypothetical protein
					31	67	33.0466	67.0000	
FN0050	-0.820	16.299	8.04e-2	5.463e-2	194	151	283.3555	196.5441	AAL94263.1| Fumarate reductase flavoprotein subunit
					442	231	471.1805	231.0000	
FN0052					12		17.5271		AAL94265.1| Arsenate reductase
FN0054	0.352	14.314	1.918e-1	2.926e-1	54	124	78.8722	161.4005	AAL94267.1| Tyrosyl-tRNA synthetase
					163	161	173.7611	161.0000	
FN0058	0.454	15.651	2.212e-1	3.627e-1	153	116	223.4711	150.9875	AAL94271.1| Cysteine desulfhydrase
					154	380	164.1670	380.0000	

Log$_2$ Ratios: -6 -4 -2 0 2 4 6

- Show detected proteins only
- Show all proteins
- Filter by category: GO: amino acid transport

Proteins found: 1313

Enter (or paste) list of ORFs

Find ORFs

Test	Cutoff
q-Value	.005
p-Value	

	Signif	Direction	Applies To
red	yes	+	ratios, bars
yellow	no	n/a	bars
green	yes	–	ratios, bars
pink	yes	+	*p*- , *q*-Values
light green	yes	–	*p*- , *q*-Values

Dot Plots Dot Plots

Protein	*FnSg vs FnPg* Log$_2$ Ratio	Log$_2$ Sum	*q*-Value	*p*-Value	Raw *FnPg*	Raw *FnSg*	Normalized *FnPg*	Normalized *FnSg*	Description
FN0059	0.914	10.203	1.057e-1	9.419e-2	16	24	23.3695	31.2388	AAL94272.1| NifU protein
					25	63	26.6505	63.0000	
FN0060	-0.596	6.211			5		7.3030		AAL94273.1| D-alanyl-D-alanine carboxypeptidase
					13	7	13.8583	7.0000	
FN0061	-0.086	12.974	4.062e-1	8.929e-1	20	60	29.2119	78.0970	AAL94274.1| Thermostable carboxypeptidase 1
					146	96	155.6388	96.0000	
FN0062									AAL94275.1| Hypothetical cytosolic protein
					6		6.3961		
FN0063									AAL94276.1| unknown
						24		24.0000	
FN0065	0.372	12.985	1.016e-1	8.749e-2	50	66	73.0298	85.9067	AAL94278.1| Transcription accessory protein (S1 RNA binding domain)
					80	119	85.2815	119.0000	
FN0066									AAL94279.1| Two component system histidine kinase
						5		5.0000	
FN0067	-0.288	16.080	1.432e-1	1.712e-1	234	180	341.7793	234.2910	AAL94280.1| Isoleucyl-tRNA synthetase
					225	242	239.8543	242.0000	
FN0069	-0.392	15.467	1.169e-1	1.137e-1	198	161	289.1979	209.5603	AAL94282.1| Glycyl-tRNA synthetase alpha chain
					186	162	198.2796	162.0000	
FN0070	0.368	17.790	7.383e-2	4.585e-2	241	432	352.0035	562.2984	AAL94283.1| Glycyl-tRNA synthetase beta chain
					456	519	486.1048	519.0000	
FN0071	0.095	6.280				7		9.1113	AAL94284.1| GTP cyclohydrolase I
					8		8.5282		
FN0072	0.461	13.747	1.498e-1	1.899e-1	42	100	61.3450	130.1617	AAL94285.1| 2-amino-4-hydroxy-6-hydroxymethyldihydropteridine pyrophosphokinase
					130	145	138.5825	145.0000	
FN0073	0.174	9.665	3.632e-1	7.446e-1	9	15	13.1454	19.5242	AAL94286.1| Dihydropteroate synthase
					38	41	40.5087	41.0000	
FN0074	0.038	8.222				10		13.0162	AAL94287.1| Ethanolamine utilization protein eutS
					16	22	17.0563	22.0000	
FN0076									AAL94289.1| Ethanolamine two-component response regulator
					4		4.2641		
FN0077	2.023	5.378							AAL94290.1| Ethanolamine two-component sensor kinase
					3	13	3.1981	13.0000	

Log$_2$ Ratios: -6 -4 -2 0 2 4 6

Show detected proteins only
Show all proteins
Filter by category:
GO: amino acid transport

Proteins found: 1313

Enter (or paste) list of ORFs

Find ORFs

Test	Cutoff
q-Value	.005
p-Value	

Color	Signif	Direction	Applies To
red	yes	+	ratios, bars
yellow	no	n/a	bars
green	yes	−	ratios, bars
pink	yes	+	*p*- , *q*-Values
light green	yes	−	*p*- , *q*-Values

Dot Plots Dot Plots

FnSg vs FnPg | # *Fusobacterium nucleatum* | **Hackett Laboratory** UW

Fn Summary Table | FnPg vs Fn | FnSg vs Fn | FnPgSg vs Fn | FnPgSg vs FnPg | FnSg vs FnPg | FnPgSg vs FnSg | Fn Coverage

Protein	*FnSg* vs *FnPg* Log$_2$ Ratio	Log$_2$ Sum	*q*-Value	*p*-Value	Raw *FnPg*	Raw *FnSg*	Normalized *FnPg*	Normalized *FnSg*	Description
FN0078	-0.445	9.457	4.438e-2	1.819e-2	19	18	27.7513	23.4291	AAL94291.1| Ethanolamine utilization protein eutA
					32	22	34.1126	22.0000	
FN0079	0.165	15.228	3.665e-1	7.552e-1	49	169	71.5692	219.9732	AAL94292.1| Ethanolamine ammonia-lyase heavy chain
					280	195	298.4854	195.0000	
FN0080	-0.723	14.987	3.075e-3	2.684e-4	163	118	238.0771	153.5908	AAL94293.1| Ethanolamine ammonia-lyase light chain
					211	127	224.9301	127.0000	
FN0081	0.308	15.898	2.664e-1	4.741e-1	80	172	116.8476	223.8780	AAL94294.1| Ethanolamine utilization protein eutL
					307	326	327.2679	326.0000	
FN0082	0.287	10.285				20		26.0323	AAL94295.1| Ethanolamine utilization protein eutM
					30	52	31.9806	52.0000	
FN0083	-0.436	16.359	2.349e-1	3.977e-1	126	164	184.0350	213.4651	AAL94296.1| Ethanolamine utilization protein eutM precursor
					460	285	490.3689	285.0000	
FN0084	-0.329	10.846	8.358e-2	5.918e-2	33	25	48.1997	32.5404	AAL94297.1| Acetaldehyde dehydrogenase (acetylating)
					45	44	47.9709	44.0000	
FN0086					7		10.2242		AAL94299.1| Hypothetical protein
FN0087	-1.925	6.569			13		18.9877		AAL94300.1| Ethanolamine utilization protein eutN
						5		5.0000	
FN0088									AAL94301.1| Hypothetical protein
						9		9.0000	
FN0090	-0.576	6.001	1.706e-1	2.397e-1	9	7	13.1454	9.1113	AAL94303.1| Ethanolamine utilization protein eutQ
					6	4	6.3961	4.0000	
FN0091					6		8.7636		AAL94304.1| Phosphoserine phosphatase
FN0092	0.338	11.695	2.975e-1	5.529e-1	11	48	16.0666	62.4776	AAL94305.1| NADPH-dependent butanol dehydrogenase
					81	67	86.3476	67.0000	
FN0093	-1.072	14.361	1.411e-3	6.286e-5	153	83	223.4711	108.0342	AAL94306.1| Thioredoxin
					185	92	197.2136	92.0000	
FN0100	-0.214	7.693	1.124e-1	1.056e-1	11	9	16.0666	11.7145	AAL94309.1| Flavodoxins/hemoproteins
					14	15	14.9243	15.0000	
FN0106	-0.403	14.014	2.89e-2	8.559e-3	93	82	135.8354	106.7326	AAL94315.1| Hypothetical protein
					150	117	159.9029	117.0000	

Log$_2$ Ratios: -6 -4 -2 0 2 4 6

Show detected proteins only
Show all proteins
Filter by category:
GO: amino acid transport

Proteins found: 1313

Enter (or paste) list of ORFs

Find ORFs

Test	Cutoff
q-Value	.005
p-Value	

Signif	Direction	Applies To
yes	+	ratios, bars
no	n/a	bars
yes	−	ratios, bars
yes	+	*p*- , *q*-Values
yes	−	

Dot Plots Dot Plots

Protein	FnSg vs FnPg Log$_2$ Ratio	Log$_2$ Sum	*q*-Value	*p*-Value	Raw *FnPg*	Raw *FnSg*	Normalized *FnPg*	Normalized *FnSg*	Description
FN0108	-0.124	9.619	2.944e-1	5.448e-1	16	19	23.3695	24.7307	AAL94317.1| Microcin C7 self-immunity protein mccF
					33	29	35.1786	29.0000	
FN0110	-0.357	17.720	1.514e-1	1.94e-1	442	303	645.5832	394.3898	AAL94319.1| Seryl-tRNA synthetase
					381	427	406.1533	427.0000	
FN0111									AAL94320.1| unknown
					5		5.3301		
FN0113	1.001	13.526	1.855e-3	1.012e-4	46	114	67.1874	148.3843	AAL94322.1| Heat-inducible transcription repressor hrcA
					81	159	86.3476	159.0000	
FN0114	0.123	13.893	3.326e-1	6.506e-1	83	71	121.2294	92.4148	AAL94323.1| GrpE protein
					108	165	115.1301	165.0000	
FN0116	0.517	21.255	1.923e-1	2.94e-1	604	902	882.1997	1174.0581	AAL94325.1| Chaperone protein dnaK
					1653	2611	1762.1299	2611.0000	
FN0117	0.593	8.392				10		13.0162	AAL94326.1| O6-methylguanine-DNA methyltransferase
					14	32	14.9243	32.0000	
FN0118	0.540	11.886	3.846e-2	1.406e-2	29	51	42.3573	66.3824	AAL94327.1| Chaperone protein dnaJ
					56	82	59.6971	82.0000	
FN0119	0.245	11.539				29		37.7469	AAL94328.1| Flavodoxin
					47	81	50.1029	81.0000	
FN0123	0.474	5.303				6		7.8097	AAL94332.1| ATPase
					5	7	5.3301	7.0000	
FN0127	0.209	5.302			4	5	5.8424	6.5081	AAL94333.1| Fe-S oxidoreductase
						7		7.0000	
FN0128	-0.006	8.274	4.311e-1	9.893e-1	11	7	16.0666	9.1113	AAL94334.1| Spermidine/putrescine-binding protein
					18	26	19.1883	26.0000	
FN0130	1.094	7.279				8		10.4129	AAL94336.1| ABC transporter ATP-binding protein
					8	26	8.5282	26.0000	
FN0132	-0.105	5.275			3		4.3818		AAL93916.1| Hemolysin
					8	6	8.5282	6.0000	
FN0147	0.313	9.694	1.912e-1	2.911e-1	12	27	17.5271	35.1436	AAL94353.1| PLSX protein
					32	29	34.1126	29.0000	
FN0148	-0.026	16.008	4.133e-1	9.196e-1	159	248	232.2347	322.8009	AAL94354.1| 3-oxoacyl-[acyl-carrier-protein] synthase III
					268	186	285.6932	186.0000	

Log$_2$ Ratios: -6 -4 -2 0 2 4 6

Show detected proteins only
Show all proteins
Filter by category:
GO: amino acid transport

Proteins found: 1313

Enter (or paste) list of ORFs

Find ORFs

Test	Cutoff
q-Value	.005
p-Value	

	Signif	Direction	Applies To
red	yes	+	ratios, bars
yellow	no	n/a	bars
green	yes	−	ratios, bars
pink	yes	+	*p*-, *q*-Values
light green	yes	−	*p*-, *q*-Values

Dot Plots Dot Plots

Protein	*FnSg* vs *FnPg* Log_2 Ratio	Log_2 Sum	*q*-Value	*p*-Value	Raw *FnPg*	Raw *FnSg*	Normalized *FnPg*	Normalized *FnSg*	Description
FN0149	-1.021	14.867	7.968e-2	5.358e-2	219	115	319.8704	149.6859	AAL94355.1| Malonyl-CoA-[acyl-carrier-protein] transacylase
					162	93	172.6951	93.0000	
FN0150	-0.325	10.769	6.703e-2	3.818e-2	29	32	42.3573	41.6517	AAL94356.1| Acyl carrier protein
					48	33	51.1689	33.0000	
FN0151	0.132	18.786	3.797e-1	7.991e-1	205	389	299.4221	506.3288	AAL94357.1| 3-oxoacyl-[acyl-carrier-protein] synthase
					924	901	985.0018	901.0000	
FN0152	-0.092	6.092							AAL94358.1| Ribonuclease III
					8	8	8.5282	8.0000	
FN0153									AAL94359.1| Oxygen-independent coproporphyrinogen III oxidase
						13		13.0000	
FN0155									AAL94361.1| Hypothetical protein
						5		5.0000	
FN0156	0.301	7.335	3.425e-1	6.801e-1	4	4	5.8424	5.2065	AAL94362.1| Phosphopantetheine adenylyltransferase
					16	23	17.0563	23.0000	
FN0157						6		7.8097	AAL94363.1| DNA repair protein RadA
						7		7.0000	
FN0158	1.153	10.013	6.506e-3	8.764e-4	12	36	17.5271	46.8582	AAL94364.1| DNA-binding protein
					24	49	25.5845	49.0000	
FN0161									AAL94367.1| RNA-directed DNA polymerase
						3		3.0000	
FN0164	0.530	15.804	1.25e-1	1.294e-1	155	278	226.3923	361.8494	AAL94370.1| Anhydro-N-acetylmuramyl-tripeptide amidase
					161	213	171.6291	213.0000	
FN0170	0.594	14.620	9.347e-2	7.553e-2	63	179	92.0175	232.9894	AAL94376.1| GTP-binding protein
					156	157	166.2990	157.0000	
FN0173	0.796	11.007	1.436e-1	1.723e-1	7	55	10.2242	71.5889	AAL94379.1| Hypothetical protein
					55	48	58.6311	48.0000	
FN0174	0.030	12.799	1.465e-1	1.801e-1	56	65	81.7933	84.6051	AAL94380.1| Enoyl-[acyl-carrier-protein] reductase
					80	86	85.2815	86.0000	
FN0175	1.054	9.211	6.296e-3	8.271e-4	10	27	14.6060	35.1436	AAL94381.1| Cell division inhibitor MinC
					18	35	19.1883	35.0000	
FN0176	0.638	16.129	3.194e-3	2.913e-4	134	272	195.7198	354.0397	AAL94382.1| Cell division inhibitor MinD
					219	314	233.4582	314.0000	

Log_2 Ratios: -6 -4 -2 0 2 4 6

Show detected proteins only
Show all proteins
Filter by category:
GO: amino acid transport

Proteins found: 1313

Enter (or paste) list of ORFs

Find ORFs

Test	Cutoff
q-Value	.005
p-Value	

Signif	Direction	Applies To
yes	+	ratios, bars
no	n/a	bars
yes	−	ratios, bars
yes	+	*p*- , *q*-Values
yes	−	

Dot Plots Dot Plots

Protein	*FnSg* vs *FnPg*				Raw		Normalized		Description	Log_2 Ratios
	Log_2 Ratio	Log_2 Sum	*q*-Value	*p*-Value	*FnPg*	*FnSg*	*FnPg*	*FnSg*		-6 -4 -2 0 2 4 6
**FN0177**					3		4.3818		AAL94383.1| Cell division inhibitor MinE	
**FN0178**	-0.296	10.637	1.257e-1	1.31e-1	35	30	51.1208	39.0485	AAL94384.1| UNC-44 ankyrins	
					35	33	37.3107	33.0000		
**FN0179**	-1.460	11.266	1.493e-1	1.882e-1	93	16	135.8354	20.8259	AAL94385.1| Ankyrin repeat proteins	
					27	39	28.7825	39.0000		
**FN0180**						4		5.2065	AAL94386.1| Tetratricopeptide repeat family protein	
						6		6.0000		
**FN0181**	0.168	10.072	3.454e-1	6.89e-1	11	25	16.0666	32.5404	AAL94387.1| Hypothetical protein	
					43	37	45.8388	37.0000		
**FN0182**	1.358	17.164	6.972e-2	4.109e-2	35	474	51.1208	616.9663	AAL94388.1| Sarcosine oxidase alpha subunit	
					401	610	427.4737	610.0000		
**FN0183**	0.853	16.220	1.397e-1	1.623e-1	34	274	49.6602	356.6429	AAL94389.1| Glycerol-3-phosphate dehydrogenase	
					339	386	361.3805	386.0000		
**FN0185**	0.274	8.408	8.045e-3	1.272e-3	12	15	17.5271	19.5242	AAL94391.1| Hypothetical protein	
					15	21	15.9903	21.0000		
**FN0189**	-0.627	8.202				12		15.6194	AAL94395.1| Two-component response regulator yesN	
					20	12	21.3204	12.0000		
**FN0190**	0.175	5.336	3.371e-1	6.64e-1	6	5	8.7636	6.5081	AAL94396.1| Two-component sensor kinase yesM	
					3	7	3.1981	7.0000		
**FN0191**	0.470	10.009	1.254e-1	1.304e-1	14	35	20.4483	45.5566	AAL94397.1| helix-turn-helix DNA-binding protein	
					32	30	34.1126	30.0000		
**FN0192**	0.331	8.516				13		16.9210	AAL94398.1| Dipeptide-binding protein	
					16	26	17.0563	26.0000		
**FN0197**									AAL94403.1| Methyltransferase	
						5		5.0000		
**FN0199**	0.187	12.073	9.129e-2	7.165e-2	39	50	56.9632	65.0808	AAL94405.1| Hypothetical protein	
					62	75	66.0932	75.0000		
**FN0200**	-0.797	18.638	2.076e-1	3.302e-1	942	385	1375.8810	501.1224	AAL94406.1| Biotin carboxyl carrier protein of glutaconyl-COA decarboxylase	
					289	468	308.0796	468.0000		
**FN0202**	-0.376	19.031	1.858e-2	4.165e-3	585	454	854.4484	590.9339	AAL94408.1| Glutaconate CoA-transferase subunit A	
					763	694	813.3727	694.0000		

Show detected proteins only
Show all proteins
Filter by category:
GO: amino acid transport

Proteins found: 1313

Enter (or paste) list of ORFs

Find ORFs

Test	Cutoff
q-Value	.005
p-Value	

	Signif	Direction	Applies To
(red)	yes	+	ratios, bars
(yellow)	no	n/a	bars
(green)	yes	–	ratios, bars
(pink)	yes	+	*p*- , *q*-Values
(light green)	yes	–	*p*- , *q*-Values

Dot Plots Dot Plots

Protein	FnSg vs FnPg Log$_2$ Ratio	Log$_2$ Sum	*q*-Value	*p*-Value	Raw *FnPg*	Raw *FnSg*	Normalized *FnPg*	Normalized *FnSg*	Description
FN0203	-0.666	20.747	6.635e-2	3.747e-2	1361	730	1987.8705	950.1801	AAL94409.1| Glutaconate CoA-transferase subunit B
					1270	1156	1353.8445	1156.0000	
FN0204	0.181	21.199	1.987e-1	3.098e-1	833	1129	1216.6760	1469.5251	AAL94410.1| Glutaconyl-CoA decarboxylase A subunit
					1593	1835	1698.1687	1835.0000	
FN0206	-0.719	11.587	1.542e-1	2.005e-1	69	38	100.7811	49.4614	AAL94412.1| Activator of (R)-2-hydroxyglutaryl-CoA dehydratase
					39	37	41.5748	37.0000	
FN0207	-0.238	18.659	2.657e-1	4.725e-1	632	444	923.0963	577.9178	AAL94413.1| (R)-2-hydroxyglutaryl-CoA dehydratase alpha-subunit
					445	607	474.3786	607.0000	
FN0208	-0.277	17.287	5.334e-2	2.581e-2	280	254	408.9667	330.6106	AAL94414.1| (R)-2-hydroxyglutaryl-CoA dehydratase beta-subunit
					442	396	471.1805	396.0000	
FN0209	-0.218	17.566	1.837e-1	2.717e-1	339	248	495.1419	322.8009	AAL94415.1| Hypothetical cytosolic protein
					427	494	455.1902	494.0000	
FN0212	-0.484	8.841	1.657e-1	2.279e-1	23	14	33.5937	18.2226	AAL94418.1| Hypothetical protein
					16	18	17.0563	18.0000	
FN0214					3		4.3818		AAL94420.1| Crossover junction endodeoxyribonuclease ruvC
FN0218	-1.480	13.829	4.689e-2	2.01e-2	173	48	252.6830	62.4776	AAL94424.1| Anthranilate synthase component II
					141	82	150.3087	82.0000	
FN0219	1.130	6.485							AAL94425.1| Autolysin response regulator
					6	14	6.3961	14.0000	
FN0221	-0.801	11.066	1.214e-2	2.271e-3	45	27	65.7268	35.1436	AAL94427.1| Carbon starvation protein A
					53	35	56.4990	35.0000	
FN0224	-0.500	7.766	1.859e-1	2.773e-1	16	6	23.3695	7.8097	AAL94430.1| Excinuclease ABC subunit B
					11	17	11.7262	17.0000	
FN0225	-0.507	3.677							AAL94431.1| Gluconate permease
					4	3	4.2641	3.0000	
FN0226	-0.776	11.303	3.264e-3	3.059e-4	47	26	68.6480	33.8420	AAL94432.1| Pyridoxal phosphate biosynthetic protein pdxA
					59	43	62.8951	43.0000	
FN0227	0.230	4.414							AAL94433.1| Hypothetical protein
					4	5	4.2641	5.0000	
FN0233	-0.557	11.929	1.174e-1	1.145e-1	65	43	94.9387	55.9695	AAL94439.1| Hypothetical protein
					53	47	56.4990	47.0000	

Log$_2$ Ratios: -6 -4 -2 0 2 4 6

Show detected proteins only
Show all proteins
Filter by category: GO: amino acid transport

Proteins found: 1313

Enter (or paste) list of ORFs

Find ORFs

Test	Cutoff
q-Value	.005
p-Value	

	Signif	Direction	Applies To
red	yes	+	ratios, bars
yellow	no	n/a	bars
green	yes	−	ratios, bars
pink	yes	+	*p*- , *q*-Values
light green	yes	−	

Dot Plots Dot Plots

Protein	FnSg vs FnPg Log$_2$ Ratio	Log$_2$ Sum	q-Value	p-Value	Raw FnPg	Raw FnSg	Normalized FnPg	Normalized FnSg	Description
FN0234	0.272	7.375				11		14.3178	AAL94440.1| unknown
					11	14	11.7262	14.0000	
FN0235					16		23.3695		AAL94441.1| ABC transporter ATP-binding protein
FN0236	-1.954	15.340	1.515e-1	1.943e-1	486	76	709.8494	98.9229	AAL94442.1| ABC transporter substrate-binding protein
					86	108	91.6777	108.0000	
FN0237					3		4.3818		AAL94443.1| ABC transporter permease protein
FN0238	-0.897	10.134	8.445e-2	6.049e-2	40	17	58.4238	22.1275	AAL94444.1| Hypothetical protein
					31	27	33.0466	27.0000	
FN0240	-0.719	12.523	2.403e-2	6.248e-3	64	55	93.4781	71.5889	AAL94446.1| Thymidylate synthase
					97	48	103.4039	48.0000	
FN0241	0.229	9.879	2.275e-1	3.785e-1	14	28	20.4483	36.4453	AAL94447.1| Dihydrofolate reductase
					34	30	36.2447	30.0000	
FN0242	0.947	13.131				116		150.9875	AAL94448.1| Trk system potassium uptake protein trkA
					64	112	68.2252	112.0000	
FN0243	0.276	9.862				17		22.1275	AAL94449.1| Poly(A) polymerase
					26	45	27.7165	45.0000	
FN0244	-0.449	6.379				6		7.8097	AAL94450.1| COP associated protein
					10		10.6602		
FN0245	0.592	6.777				9		11.7145	AAL94451.1| Copper-exporting ATPase
					8	14	8.5282	14.0000	
FN0247	0.819	14.365	8.486e-2	6.112e-2	41	149	59.8844	193.9409	AAL94453.1| Hypothetical cytosolic protein
					149	192	158.8369	192.0000	
FN0248	0.180	11.149				28		36.4453	AAL94454.1| Hypothetical Exported Protein
					42	65	44.7728	65.0000	
FN0249	-0.190	12.637	3.647e-1	7.496e-1	89	81	129.9930	105.4309	AAL94455.1| unknown
					38	44	40.5087	44.0000	
FN0250	-0.406	12.967	2.74e-1	4.926e-1	106	81	154.8231	105.4309	AAL94456.1| unknown
					48	50	51.1689	50.0000	
FN0251	-2.135	13.336	1.251e-1	1.297e-1	240	40	350.5429	52.0647	AAL94457.1| Hypothetical membrane-spanning Protein
					71	45	75.6874	45.0000	

Log$_2$ Ratios: -6 -4 -2 0 2 4 6

Show detected proteins only
Show all proteins
Filter by category:
GO: amino acid transport

Proteins found: 1313

Enter (or paste) list of ORFs

Find ORFs

Test	Cutoff
q-Value	.005
p-Value	

	Signif	Direction	Applies To
red	yes	+	ratios, bars
yellow	no	n/a	bars
green	yes	−	ratios, bars
pink	yes	+	p- , q-Values
light green	yes	−	p- , q-Values

Dot Plots Dot Plots

Protein	*FnSg* vs *FnPg* Log$_2$ Ratio	Log$_2$ Sum	*q*-Value	*p*-Value	Raw *FnPg*	Raw *FnSg*	Normalized *FnPg*	Normalized *FnSg*	Description
**FN0252**	-0.525	18.648	1.974e-1	3.071e-1	753	430	1099.8284	559.6951	AAL94458.1| unknown
					411	509	438.1339	509.0000	
**FN0253**	0.792	16.177				180		234.2910	AAL94459.1| Outer membrane protein
					194	482	206.8077	482.0000	
**FN0254**	-0.171	18.364	2.091e-1	3.337e-1	352	444	514.1296	577.9178	AAL94460.1| Fusobacterium outer membrane protein family
					674	517	718.4970	517.0000	
**FN0258**	-0.115	5.730			6		8.7636		AAL94464.1| Zinc-transporting ATPase
					6	7	6.3961	7.0000	
**FN0261**	-0.588	7.675			12	11	17.5271	14.3178	AAL94467.1| Pyruvate formate-lyase activating enzyme
						9		9.0000	
**FN0262**	-0.511	20.865	2.411e-1	4.134e-1	1745	878	2548.7391	1142.8193	AAL94468.1| Formate acetyltransferase
					704	1173	750.4776	1173.0000	
**FN0263**	-1.480	15.329	1.508e-1	1.925e-1	386	106	563.7899	137.9714	AAL94469.1| Peptidyl-prolyl cis-trans isomerase
					107	105	114.0641	105.0000	
**FN0264**	-0.049	15.420	4.096e-1	9.058e-1	96	211	140.2172	274.6411	AAL94470.1| Hypothetical protein
					268	137	285.6932	137.0000	
**FN0265**	-0.455	6.649				7		9.1113	AAL94471.1| Cell division protein ftsX
					11	8	11.7262	8.0000	
**FN0266**									AAL94472.1| membrane protein related to metalloendopeptidase
					8		8.5282		
**FN0267**	0.323	5.677							AAL94473.1| ATP-NAD kinase
					6	8	6.3961	8.0000	
**FN0268**	0.386	8.398			11		16.0666		AAL94474.1| DNA repair protein recN
						21		21.0000	
**FN0270**						4		5.2065	AAL94476.1| GTP-binding protein era
						5		5.0000	
**FN0271**	-0.690	7.110				5		6.5081	AAL94477.1| Enoyl-CoA hydratase
					14	12	14.9243	12.0000	
**FN0272**	1.301	5.486				10		13.0162	AAL94478.1| Acetoacetate: butyrate/acetate coenzyme A transferase
					4	8	4.2641	8.0000	
**FN0273**	-1.074	7.111				4		5.2065	AAL94479.1| Butyrate-acetoacetate CoA-transferase subunit B
					16	11	17.0563	11.0000	

Log$_2$ Ratios: -6 -4 -2 0 2 4 6

Show detected proteins only
Show all proteins
Filter by category:
GO: amino acid transport

Proteins found: 1313

Enter (or paste) list of ORFs

Find ORFs

Test	Cutoff
q-Value	.005
p-Value	

	Signif	Direction	Applies To
red	yes	+	ratios, bars
yellow	no	n/a	bars
green	yes	–	ratios, bars
pink	yes	+	*p*-, *q*-Values
light green	yes	–	

Dot Plots Dot Plots

Protein	FnSg vs FnPg Log2 Ratio	Log2 Sum	q-Value	p-Value	Raw FnPg	Raw FnSg	Normalized FnPg	Normalized FnSg	Description
FN0276	0.609	11.134				50		65.0808	AAL94482.1| Sodium-dependent phosphate transporter
					36	52	38.3767	52.0000	
FN0277	-0.190	7.083	2.604e-1	4.596e-1	9	6	13.1454	7.8097	AAL94483.1| Hypothetical protein
					11	14	11.7262	14.0000	
FN0278	-0.464	15.828	2.93e-2	8.761e-3	209	158	305.2645	205.6554	AAL94484.1| Xaa-His dipeptidase
					245	205	261.1747	205.0000	
FN0279	0.615	14.873	1.932e-1	2.964e-1	26	175	37.9755	227.7829	AAL94485.1| Lipoprotein
					227	201	241.9864	201.0000	
FN0280	0.199	12.759	3.492e-1	7.006e-1	21	71	30.6725	92.4148	AAL94486.1| Hypothetical protein
					117	86	124.7243	86.0000	
FN0281	1.332	12.939	2.707e-2	7.662e-3	21	87	30.6725	113.2406	AAL94487.1| DNA polymerase III alpha subunit
					76	168	81.0175	168.0000	
FN0282	0.154	11.290	2.31e-1	3.874e-1	27	45	39.4361	58.5727	AAL94488.1| Hypothetical protein
					52	47	55.4330	47.0000	
FN0283	-0.244	6.584							AAL94489.1| tRNA (Guanine-N1) -methyltransferase
					10	9	10.6602	9.0000	
FN0284	-0.125	11.460				32		41.6517	AAL94490.1| 16S rRNA processing protein rimM
					52	60	55.4330	60.0000	
FN0285									AAL94491.1| RNA binding protein
					27		28.7825		
FN0287	-0.507	7.079				5		6.5081	AAL94493.1| Dimethyladenosine transferase
					13	13	13.8583	13.0000	
FN0288	-0.132	11.731	3.674e-1	7.582e-1	23	41	33.5937	53.3663	AAL94494.1| Hypoxanthine-guanine phosphoribosyltransferase
					83	58	88.4796	58.0000	
FN0291	-1.092	5.736							AAL94497.1| Hemolysin
					10	5	10.6602	5.0000	
FN0294	-1.095	13.579	4.651e-2	1.98e-2	136	71	198.6410	92.4148	AAL94500.1| Transketolase subunit A
					117	59	124.7243	59.0000	
FN0295	-0.929	14.223	9.08e-2	7.081e-2	170	71	248.3012	92.4148	AAL94501.1| Transketolase
					125	108	133.2524	108.0000	
FN0296	-0.120	11.682	3.291e-1	6.404e-1	30	43	43.8179	55.9695	AAL94502.1| Hypothetical cytosolic protein
					71	54	75.6874	54.0000	

Log2 Ratios: -6 -4 -2 0 2 4 6

Show detected proteins only
Show all proteins
Filter by category:
GO: amino acid transport

Proteins found: 1313

Enter (or paste) list of ORFs

Find ORFs

Test	Cutoff
q-Value	.005
p-Value	

Color	Signif	Direction	Applies To
red	yes	+	ratios, bars
yellow	no	n/a	bars
green	yes	−	ratios, bars
pink	yes	+	p- , q-Values
light green	yes	−	

Dot Plots Dot Plots

Protein	*FnSg* vs *FnPg* Log$_2$ Ratio	Log$_2$ Sum	*q*-Value	*p*-Value	Raw *FnPg*	Raw *FnSg*	Normalized *FnPg*	Normalized *FnSg*	Description
FN0297	0.890	8.674	8.369e-2	5.936e-2	5	20	7.3030	26.0323	AAL94503.1| ATPase associated with chromosome architecture/replication
					21	29	22.3864	29.0000	
FN0298	0.068	16.301	4.025e-1	8.796e-1	94	225	137.2960	292.8637	AAL94504.1| Histidyl-tRNA synthetase
					392	289	417.8796	289.0000	
FN0299	0.263	15.873	2.449e-1	4.227e-1	96	198	140.2172	257.7201	AAL94505.1| Aspartyl-tRNA synthetase
					288	279	307.0136	279.0000	
FN0308	0.763	17.256	1.419e-3	6.376e-5	191	417	278.9737	542.7741	AAL94514.1| Iron(III)-binding protein
					308	488	328.3339	488.0000	
FN0310	-0.860	12.155	1.081e-1	9.826e-2	83	44	121.2294	57.2711	AAL94516.1| Iron(III)-transport ATP-binding protein sfuC
					57	43	60.7631	43.0000	
FN0311	1.210	13.477	2.582e-2	7.09e-3	29	132	42.3573	171.8134	AAL94517.1| Anaerobic ribonucleoside-triphosphate reductase
					92	153	98.0738	153.0000	
FN0313	0.816	8.615				25		32.5404	AAL94519.1| 16S rRNA m(5)C 967 methyltransferase
					14	20	14.9243	20.0000	
FN0314	0.280	6.465				9		11.7145	AAL94520.1| Caffeoyl-CoA O-methyltransferase
					8	9	8.5282	9.0000	
FN0315	-0.369	6.459				5		6.5081	AAL94521.1| Transcriptional regulator, AraC family
					10	10	10.6602	10.0000	
FN0316	-1.304	7.964			17	7	24.8301	9.1113	AAL94522.1| Hypothetical protein
						11		11.0000	
FN0317	0.956	9.605	7.221e-2	4.392e-2	7	29	10.2242	37.7469	AAL94523.1| Tryptophan synthase beta chain
					28	40	29.8485	40.0000	
FN0319	0.357	6.156				7		9.1113	AAL94525.1| Citrate (pro-3S)-lyase ligase
					7	10	7.4621	10.0000	
FN0320	-1.219	8.306	9.754e-3	1.653e-3	16	11	23.3695	14.3178	AAL94526.1| Hypothetical cytosolic protein
					29	9	30.9146	9.0000	
FN0321	0.621	14.969	8.387e-2	5.963e-2	70	140	102.2417	182.2263	AAL94527.1| Heat shock protein htpG
					175	262	186.5534	262.0000	
FN0322	0.160	21.915	2.94e-1	5.436e-1	934	1816	1364.1962	2363.7357	AAL94528.1| Fructose-bisphosphate aldolase
					2250	1839	2398.5434	1839.0000	
FN0325	-0.132	4.132			3		4.3818		AAL94529.1| LSU ribosomal protein L20P
						4		4.0000	

Show detected proteins only
Show all proteins
Filter by category:
GO: amino acid transport

Proteins found: 1313

Enter (or paste) list of ORFs

Find ORFs

Test	Cutoff
q-Value	.005
p-Value	

Color	Signif	Direction	Applies To
red	yes	+	ratios, bars
yellow	no	n/a	bars
green	yes	−	ratios, bars
pink	yes	+	*p*- , *q*-Values
light green	yes	−	

Dot Plots Dot Plots

Protein	FnSg vs FnPg Log2 Ratio	Log2 Sum	q-Value	p-Value	Raw FnPg	Raw FnSg	Normalized FnPg	Normalized FnSg	Description
FN0327	0.255	9.487				15		19.5242	AAL94531.1| Bacterial Protein Translation Initiation Factor 3 (IF-3)
					23	39	24.5184	39.0000	
FN0329	-0.590	17.490	1.37e-1	1.557e-1	472	243	689.4011	316.2928	AAL94533.1| LSU ribosomal protein L13P
					341	383	363.5126	383.0000	
FN0330	-0.705	10.639	1.334e-1	1.474e-1	45	35	65.7268	45.5566	AAL94534.1| SSU ribosomal protein S9P
					34	17	36.2447	17.0000	
FN0331	0.878	11.193	1.185e-1	1.166e-1	8	57	11.6848	74.1921	AAL94535.1| Hypothetical protein
					56	57	59.6971	57.0000	
FN0332	0.740	6.539				13		16.9210	AAL94536.1| Magnesium and cobalt transport protein corA
					7	8	7.4621	8.0000	
FN0333	-0.137	6.047				5		6.5081	AAL94537.1| Glycerol uptake operon antiterminator regulatory protein
					8	9	8.5282	9.0000	
FN0334	0.788	15.023	3.433e-2	1.159e-2	69	168	100.7811	218.6716	AAL94538.1| Aspartate/aromatic aminotransferase
					166	261	176.9592	261.0000	
FN0335	1.121	20.996	2.564e-2	7.002e-3	932	1798	1361.2750	2340.3066	AAL94539.1| Outer membrane porin F
					563	1925	600.1689	1925.0000	
FN0336	0.303	10.396				35		45.5566	AAL94540.1| Hypothetical protein
					31	36	33.0466	36.0000	
FN0341	-2.181	8.648				6		7.8097	AAL94545.1| transport protein
					40	11	42.6408	11.0000	
FN0342	-0.612	10.071	1.795e-1	2.61e-1	38	30	55.5026	39.0485	AAL94546.1| Peptidyl-prolyl cis-trans isomerase
					24	14	25.5845	14.0000	
FN0344									AAL94548.1| Methyltransferase
						3		3.0000	
FN0347	0.519	11.693	1.886e-1	2.841e-1	14	49	20.4483	63.7792	AAL94551.1| Phosphatidylserine decarboxylase
					71	74	75.6874	74.0000	
FN0348	0.069	15.922	3.841e-1	8.143e-1	120	233	175.2715	303.2767	AAL94552.1| Nicotinate phosphoribosyltransferase
					292	207	311.2776	207.0000	
FN0349	0.720	9.762	9.009e-2	6.962e-2	11	22	16.0666	28.6356	AAL94553.1| D-Tyr-tRNATyr deacylase
					28	47	29.8485	47.0000	
FN0351	-1.971	11.839	1.408e-1	1.652e-1	140	27	204.4834	35.1436	AAL94555.1| unknown
					33	26	35.1786	26.0000	

Log2 Ratios: -6 -4 -2 0 2 4 6

Show detected proteins only
Show all proteins
Filter by category:
GO: amino acid transport

Proteins found: 1313

Enter (or paste) list of ORFs
Find ORFs

Test	Cutoff
q-Value	.005
p-Value	

Color	Signif	Direction	Applies To
red	yes	+	ratios, bars
yellow	no	n/a	bars
green	yes	–	ratios, bars
pink	yes	+	p- , q-Values
light green	yes	–	

Dot Plots Dot Plots

Protein	FnSg vs FnPg Log2 Ratio	Log2 Sum	q-Value	p-Value	Raw FnPg	Raw FnSg	Normalized FnPg	Normalized FnSg	Description
FN0352	-0.330	11.164	2.681e-1	4.782e-1	21	38	30.6725	49.4614	AAL94556.1| NA+/H+ antiporter NHAC
					72	36	76.7534	36.0000	
FN0355	0.172	15.270	2.916e-1	5.374e-1	93	189	135.8354	246.0055	AAL94558.1| S-adenosylmethionine synthetase
					224	176	238.7883	176.0000	
FN0356	-1.566	7.942			26	7	37.9755	9.1113	AAL94559.1| Lactoylglutathione lyase
					15		15.9903		
FN0357	-0.124	7.331	3.745e-1	7.816e-1	5	11	7.3030	14.3178	AAL94560.1| ATP synthase epsilon chain, sodium ion specific
					18	10	19.1883	10.0000	
FN0358	0.581	17.192	6.231e-2	3.35e-2	212	420	309.6462	546.6790	AAL94561.1| ATP synthase beta chain, sodium ion specific
					303	400	323.0038	400.0000	
FN0359	0.365	11.877	1.394e-1	1.615e-1	47	57	68.6480	74.1921	AAL94562.1| ATP synthase gamma chain, sodium ion specific
					37	65	39.4427	65.0000	
FN0360	0.285	14.476	7.053e-2	4.199e-2	96	140	140.2172	182.2263	AAL94563.1| ATP synthase alpha chain, sodium ion specific
					125	151	133.2524	151.0000	
FN0361	0.049	9.456	4.111e-1	9.113e-1	24	13	35.0543	16.9210	AAL94564.1| ATP synthase delta chain, sodium ion specific
					16	37	17.0563	37.0000	
FN0362	0.489	10.582				29		37.7469	AAL94565.1| ATP synthase B chain, sodium ion specific
					31	55	33.0466	55.0000	
FN0364									AAL94567.1| ATP synthase A chain, sodium ion specific
					13		13.8583		
FN0366	0.094	15.463	2.964e-1	5.5e-1	127	193	185.4956	251.2120	AAL94569.1| Phosphoglucosamine mutase
					212	188	225.9961	188.0000	
FN0368	-0.854	12.891	8.103e-2	5.551e-2	59	42	86.1751	54.6679	AAL94571.1| Adenylosuccinate lyase
					139	75	148.1767	75.0000	
FN0370	-0.097	9.472	3.919e-1	8.415e-1	10	25	14.6060	32.5404	AAL94573.1| Signal peptidase I
					38	19	40.5087	19.0000	
FN0371	-1.266	8.366			32	9	46.7391	11.7145	AAL94574.1| Hypothetical protein
					9		9.5942		
FN0374	0.957	7.687			9		13.1454		AAL94577.1| Single-stranded-DNA-specific exonuclease recJ
					7	20	7.4621	20.0000	
FN0375	0.592	18.873	3.983e-2	1.494e-2	353	740	515.5902	963.1963	AAL94578.1| Iron(III)-binding protein
					575	738	612.9611	738.0000	

Log2 Ratios: -6 -4 -2 0 2 4 6

Show detected proteins only
Show all proteins
Filter by category:
GO: amino acid transport

Proteins found: 1313

Enter (or paste) list of ORFs

Find ORFs

Test	Cutoff
q-Value	.005
p-Value	

Signif	Direction	Applies To
yes	+	ratios, bars
no	n/a	bars
yes	–	ratios, bars
yes	+	p- , q-Values
yes	–	

Dot Plots Dot Plots

Protein	*FnSg* vs *FnPg* Log$_2$ Ratio	Log$_2$ Sum	*q*-Value	*p*-Value	Raw *FnPg*	Raw *FnSg*	Normalized *FnPg*	Normalized *FnSg*	Description
**FN0376**	0.684	14.002	6.587e-3	8.96e-4	61	115	89.0963	149.6859	AAL94579.1| Iron(III)-transport ATP-binding protein sfuC
					106	175	112.9980	175.0000	
**FN0377**									AAL94580.1| Iron(III)-transport system permease protein sfuB
						5		5.0000	
**FN0378**						3		3.9048	AAL94581.1| UDP-glucose 4-epimerase
						11		11.0000	
**FN0379**	-0.504	5.674			8		11.6848		AAL94582.1| Hypothetical protein
					5	6	5.3301	6.0000	
**FN0380**									AAL94583.1| unknown
					6		6.3961		
**FN0384**	0.071	4.334			3	4	4.3818	5.2065	AAL94587.1| Hypothetical protein
						4		4.0000	
**FN0385**									AAL94588.1| Hypothetical protein
					5		5.3301		
**FN0387**	0.178	9.358	3.829e-1	8.102e-1	14	5	20.4483	6.5081	AAL94590.1| Fusobacterium outer membrane protein family
					26	48	27.7165	48.0000	
**FN0390**	-0.310	12.774	2.522e-1	4.404e-1	40	64	58.4238	83.3035	AAL94593.1| Hypothetical protein
					120	67	127.9223	67.0000	
**FN0391**	-0.025	9.836	4.26e-1	9.689e-1	6	23	8.7636	29.9372	AAL94594.1| Hydrolase (HAD superfamily)
					49	30	52.2349	30.0000	
**FN0392**	-0.021	4.808				5		6.5081	AAL94595.1| Oxygen-independent coproporphyrinogen III oxidase
					5	4	5.3301	4.0000	
**FN0393**	0.683	9.213	7.93e-2	5.305e-2	11	29	16.0666	37.7469	AAL94596.1| Polysaccharide deacetylase
					21	24	22.3864	24.0000	
**FN0394**	-1.012	7.388			4	7	5.8424	9.1113	AAL94597.1| Outer membrane protein
					29		30.9146		
**FN0396**	0.238	24.828	1.754e-1	2.514e-1	3549	5397	5183.6534	7024.8246	AAL94599.1| Dipeptide-binding protein
					4566	4827	4867.4440	4827.0000	
**FN0397**	0.037	11.074				40		52.0647	AAL94600.1| Dipeptide transport system permease protein dppB
					43	42	45.8388	42.0000	
**FN0398**	0.556	11.070	3.375e-2	1.123e-2	29	38	42.3573	49.4614	AAL94601.1| Dipeptide transport system permease protein dppC
					32	63	34.1126	63.0000	

Log$_2$ Ratios: -6 -4 -2 0 2 4 6

Show detected proteins only
Show all proteins
Filter by category:
GO: amino acid transport

Proteins found: 1313

Enter (or paste) list of ORFs

Find ORFs

Test	Cutoff
q-Value	.005
p-Value	

	Signif	Direction	Applies To
red	yes	+	ratios, bars
yellow	no	n/a	bars
green	yes	−	ratios, bars
pink	yes	+	*p*- , *q*-Values
light green	yes	−	

Dot Plots Dot Plots

Protein	*FnSg* vs *FnPg* Log$_2$ Ratio	Log$_2$ Sum	*q*-Value	*p*-Value	Raw *FnPg*	Raw *FnSg*	Normalized *FnPg*	Normalized *FnSg*	Description
FN0399	0.307	15.227	1.549e-1	2.022e-1	98	138	143.1384	179.6231	AAL94602.1| Dipeptide transport ATP-binding protein dppD
					196	256	208.9398	256.0000	
FN0400	0.197	17.503	1.169e-1	1.136e-1	306	331	446.9422	430.8351	AAL94603.1| Dipeptide transport ATP-binding protein dppF
					336	492	358.1825	492.0000	
FN0405	-0.009	12.802	4.281e-1	9.774e-1	38	58	55.5026	75.4938	AAL94608.1| Tryptophanyl-tRNA synthetase
					107	93	114.0641	93.0000	
FN0406	-0.352	10.060	1.482e-1	1.851e-1	25	16	36.5149	20.8259	AAL94609.1| Alanine racemase, biosynthetic
					35	37	37.3107	37.0000	
FN0407	0.367	9.336				19		24.7307	AAL94610.1| Hypothetical protein
					21	33	22.3864	33.0000	
FN0408	0.618	12.444	1.478e-1	1.84e-1	19	76	27.7513	98.9229	AAL94611.1| Acetyl-coenzyme A carboxylase carboxyl transferase subunit beta
					87	86	92.7437	86.0000	
FN0409	0.093	16.272	2.99e-1	5.569e-1	219	219	319.8704	285.0540	AAL94612.1| Acetyl-coenzyme A carboxylase carboxyl transferase subunit alpha
					211	296	224.9301	296.0000	
FN0410	0.234	13.369	4.817e-2	2.113e-2	62	80	90.5569	104.1293	AAL94613.1| 6-phosphofructokinase
					93	119	99.1398	119.0000	
FN0411						13		16.9210	AAL94614.1| putative alpha helix protein
FN0413					8		11.6848		AAL94616.1| unknown
					3		3.1981		
FN0414									AAL94617.1| ATP-dependent helicase HEPA
						4		4.0000	
FN0416					9		13.1454		AAL94619.1| Type III restriction-modification system methylation subunit
FN0417	-0.810	7.453			12		17.5271		AAL94620.1| Type III restriction-modification system restriction subunit
						10		10.0000	
FN0418	0.413	12.730				77		100.2245	AAL94621.1| Uracil phosphoribosyltransferase
					67	90	71.4233	90.0000	
FN0419	-0.812	7.121	3.494e-2	1.199e-2	9	6	13.1454	7.8097	AAL94622.1| Aspartate carbamoyltransferase
					17	10	18.1223	10.0000	
FN0420	0.271	10.280	3.414e-1	6.767e-1	6	38	8.7636	49.4614	AAL94623.1| Dihydroorotase
					52	28	55.4330	28.0000	

Log$_2$ Ratios: -6 -4 -2 0 2 4 6

Show detected proteins only
Show all proteins
Filter by category:
GO: amino acid transport

Proteins found: 1313

Enter (or paste) list of ORFs

Find ORFs

Test	Cutoff
q-Value	.005
p-Value	

	Signif	Direction	Applies To
red	yes	+	ratios, bars
yellow	no	n/a	bars
green	yes	−	ratios, bars
pink	yes	+	*p*- , *q*-Values
light green	yes	−	

Dot Plots | Dot Plots

FnSg vs FnPg

Fusobacterium nucleatum

Hackett Laboratory UW

Fn Summary Table | FnPg vs Fn | FnSg vs Fn | FnPgSg vs Fn | FnPgSg vs FnPg | FnSg vs FnPg | FnPgSg vs FnSg | Fn Coverage

Protein	*FnSg* vs *FnPg* Log$_2$ Ratio	Log$_2$ Sum	*q*-Value	*p*-Value	Raw *FnPg*	Raw *FnSg*	Normalized *FnPg*	Normalized *FnSg*	Description
FN0421	-0.441	10.388				26		33.8420	AAL94624.1| Carbamoyl-phosphate synthase small chain
					40	29	42.6408	29.0000	
FN0422	0.866	14.286	1.306e-1	1.411e-1	20	158	29.2119	205.6554	AAL94625.1| Carbamoyl-phosphate synthase large chain
					169	176	180.1573	176.0000	
FN0423	0.781	8.966				22		28.6356	AAL94626.1| Dihydroorotate dehydrogenase electron transfer subunit
					16	30	17.0563	30.0000	
FN0424	0.995	7.839	8.033e-2	5.452e-2	3	19	4.3818	24.7307	AAL94627.1| Dihydroorotate dehydrogenase
					16	18	17.0563	18.0000	
FN0426	0.582	10.924	2.025e-1	3.186e-1	7	46	10.2242	59.8744	AAL94629.1| Orotidine 5'-phosphate decarboxylase
					58	48	61.8291	48.0000	
FN0427	0.006	11.569	4.234e-1	9.585e-1	41	38	59.8844	49.4614	AAL94630.1| Orotate phosphoribosyltransferase
					47	61	50.1029	61.0000	
FN0430	0.631	14.454	9.84e-2	8.267e-2	51	159	74.4904	206.9570	AAL94633.1| LSU ribosomal protein L19P
					156	166	166.2990	166.0000	
FN0435	-0.178	11.898	3.704e-1	7.682e-1	17	47	24.8301	61.1760	AAL94634.1| Purine nucleoside phosphorylase
					100	55	106.6019	55.0000	
FN0436	0.105	12.527	3.77e-1	7.9e-1	35	41	51.1208	53.3663	AAL94635.1| regulator of kinase autophosphorylation inhibitor
					91	106	97.0078	106.0000	
FN0437	0.528	8.125	2.155e-1	3.488e-1	3	17	4.3818	22.1275	AAL94636.1| kinase autophosphorylation inhibitor KipI
					22	18	23.4524	18.0000	
FN0439	-0.376	12.182	3.377e-1	6.657e-1	10	32	14.6060	41.6517	AAL94638.1| Lactam utilization protein LAMB
					132	78	140.7145	78.0000	
FN0445									AAL94641.1| Hypothetical protein
						6		6.0000	
FN0446	1.035	7.493	5.234e-2	2.483e-2	7	18	10.2242	23.4291	AAL94642.1| Hypothetical protein
					8	15	8.5282	15.0000	
FN0447	1.424	6.778				11		14.3178	AAL94643.1| NIFS protein
					6	20	6.3961	20.0000	
FN0450	0.695	9.234	9.329e-2	7.519e-2	14	18	20.4483	23.4291	AAL94646.1| ABC transporter ATP-binding protein
					17	39	18.1223	39.0000	
FN0451	0.197	5.996				7		9.1113	AAL94647.1| Hypothetical protein
					7	8	7.4621	8.0000	

Log$_2$ Ratios: -6 -4 -2 0 2 4 6

Show detected proteins only
Show all proteins
Filter by category:
GO: amino acid transport

Proteins found: 1313

Enter (or paste) list of ORFs

Find ORFs

Test	Cutoff
q-Value	.005
p-Value	

	Signif	Direction	Applies To
red	yes	+	ratios, bars
yellow	no	n/a	bars
green	yes	−	ratios, bars
pink	yes	+	*p*- , *q*-Values
light green	yes	−	

Dot Plots Dot Plots

Protein	*FnSg* vs *FnPg* Log$_2$ Ratio	Log$_2$ Sum	*q*-Value	*p*-Value	Raw *FnPg*	Raw *FnSg*	Normalized *FnPg*	Normalized *FnSg*	Description
FN0452	-1.408	16.823	1.636e-1	2.228e-1	647	136	945.0053	177.0199	AAL94648.1| Glucosamine--fructose-6-phosphate aminotransferase (isomerizing)
					154	241	164.1670	241.0000	
FN0453	-0.491	16.982	5.095e-2	2.353e-2	255	219	372.4518	285.0540	AAL94649.1| Xaa-Pro aminopeptidase
					451	322	480.7747	322.0000	
FN0454	0.562	15.484	1.351e-1	1.513e-1	69	173	100.7811	225.1797	AAL94650.1| Aldehyde dehydrogenase B
					236	295	251.5805	295.0000	
FN0455	0.644	18.038	1.388e-2	2.762e-3	315	547	460.0876	711.9843	AAL94651.1| Rubrerythrin
					347	585	369.9087	585.0000	
FN0456	-0.148	7.733	3.022e-1	5.655e-1	13	9	18.9877	11.7145	AAL94652.1| Hypothetical cytosolic protein
					11	16	11.7262	16.0000	
FN0458	-0.725	6.798	1.092e-1	1.002e-1	12	8	17.5271	10.4129	AAL94654.1| Hypothetical Exported Protein
					9	6	9.5942	6.0000	
FN0460	-2.071	9.728				8		10.4129	AAL94656.1| Delta-aminolevulinic acid dehydratase
					56	18	59.6971	18.0000	
FN0461	-0.836	16.434	1.356e-1	1.524e-1	381	177	556.4869	230.3861	AAL94657.1| Probable sigma(54) modulation protein
					224	215	238.7883	215.0000	
FN0462	1.025	12.425	4.38e-2	1.773e-2	23	98	33.5937	127.5584	AAL94658.1| DNA mismatch repair protein mutL
					66	84	70.3573	84.0000	
FN0465	-0.882	15.866	2.019e-1	3.172e-1	377	166	550.6445	216.0683	AAL94661.1| Hypothetical protein
					106	144	112.9980	144.0000	
FN0466	-0.036	18.093	3.916e-1	8.402e-1	428	446	625.1349	580.5210	AAL94662.1| Lysyl-tRNA synthetase
					418	464	445.5961	464.0000	
FN0469									AAL94665.1| Copper homeostasis protein cutC
						4		4.0000	
FN0470	-0.376	18.056	9.883e-2	8.33e-2	343	310	500.9843	403.5011	AAL94666.1| Putative efflux pump component MtrF
					646	513	688.6485	513.0000	
FN0472	-0.628	25.655	6.442e-2	3.553e-2	7225	4554	10552.802	5927.5618	AAL94668.1| Flavodoxin
					7052	5764	7517.5680	5764.0000	
FN0474						4		5.2065	AAL94670.1| Acriflavin resistance protein B
FN0475	0.458	9.562				28		36.4453	AAL94671.1| MIAB protein
					22	28	23.4524	28.0000	

Log$_2$ Ratios: -6 -4 -2 0 2 4 6

Show detected proteins only
Show all proteins
Filter by category:
GO: amino acid transport

Proteins found: 1313

Enter (or paste) list of ORFs

Find ORFs

Test	Cutoff
q-Value	.005
p-Value	

	Signif	Direction	Applies To
red	yes	+	ratios, bars
yellow	no	n/a	bars
green	yes	−	ratios, bars
pink	yes	+	*p*- , *q*-Values
light green	yes	−	

Dot Plots Dot Plots

FnSg vs FnPg | # *Fusobacterium nucleatum* | **Hackett Laboratory** | UW

Fn Summary Table | FnPg vs Fn | FnSg vs Fn | FnPgSg vs Fn | FnPgSg vs FnPg | FnSg vs FnPg | FnPgSg vs FnSg | Fn Coverage

Protein	*FnSg* vs *FnPg* Log$_2$ Ratio	Log$_2$ Sum	*q*-Value	*p*-Value	Raw *FnPg*	Raw *FnSg*	Normalized *FnPg*	Normalized *FnSg*	Description	Log$_2$ Ratios (-6 -4 -2 0 2 4 6)
**FN0476**	0.553	12.183	9.89e-2	8.341e-2	26	57	37.9755	74.1921	AAL94672.1| Transcription termination factor rho	
					70	91	74.6213	91.0000		
**FN0477**	1.108	9.077	4.597e-3	5.034e-4	10	24	14.6060	31.2388	AAL94673.1| Cell wall endopeptidase family M23/M37	
					16	37	17.0563	37.0000		
**FN0478**	-0.145	10.214				25		32.5404	AAL94674.1| GcpE protein	
					34	33	36.2447	33.0000		
**FN0479**						9		11.7145	AAL94675.1| RNA polymerase sigma-E factor	
						25		25.0000		
**FN0480**									AAL94676.1| unknown	
						4		4.0000		
**FN0481**	-0.311	6.792				3		3.9048	AAL94677.1| unknown	
					11	15	11.7262	15.0000		
**FN0482**									AAL94678.1| LSU ribosomal protein L31P	
					6		6.3961			
**FN0483**	-0.269	12.123	2.504e-1	4.361e-1	34	52	49.6602	67.6841	AAL94679.1| Uracil phosphoribosyltransferase	
					91	54	97.0078	54.0000		
**FN0484**	0.773	5.602				7		9.1113	AAL94680.1| Lipase	
					5		5.3301			
**FN0487**	0.108	20.456	3.514e-1	7.074e-1	548	1078	800.4063	1403.1427	AAL94683.1| 2-hydroxyglutarate dehydrogenase	
					1416	1087	1509.4833	1087.0000		
**FN0488**	0.163	24.209	3.054e-1	5.741e-1	1959	4005	2861.3066	5212.9743	AAL94684.1| NAD-specific glutamate dehydrogenase	
					5124	4107	5462.2828	4107.0000		
**FN0489**	-0.382	6.722							AAL94685.1| Prolipoprotein diacylglyceryl transferase	
					11	9	11.7262	9.0000		
**FN0491**	0.155	15.097	3.622e-1	7.414e-1	59	123	86.1751	160.0988	AAL94687.1| Alanine racemase	
					252	235	268.6369	235.0000		
**FN0493**	-0.507	8.176	1.956e-1	3.031e-1	19	15	27.7513	19.5242	AAL94689.1| Hypothetical protein	
					12	9	12.7922	9.0000		
**FN0494**	-0.915	14.912	3.853e-2	1.411e-2	136	69	198.6410	89.8115	AAL94690.1| Short chain dehydrogenase	
					266	166	283.5611	166.0000		
**FN0495**	-0.333	26.760	6.737e-4	1.257e-5	8224	7402	12011.937[illegible]	9634.5658	AAL94691.1| Acetyl-CoA acetyltransferase	
					11175	9364	11912.765[illegible]	9364.0000		

- Show detected proteins only
- Show all proteins
- Filter by category: GO: amino acid transport

Proteins found: 1313

Enter (or paste) list of ORFs

Find ORFs

Test	Cutoff
q-Value	.005
p-Value	

	Signif	Direction	Applies To
red	yes	+	ratios, bars
yellow	no	n/a	bars
green	yes	−	ratios, bars
light red	yes	+	*p*- , *q*-Values
light green	yes	−	

Dot Plots | Dot Plots

FnSg vs FnPg

Fusobacterium nucleatum

Hackett Laboratory | UW

Fn Summary Table | FnPg vs Fn | FnSg vs Fn | FnPgSg vs Fn | FnPgSg vs FnPg | FnSg vs FnPg | FnPgSg vs FnSg | Fn Coverage

Protein	*FnSg* vs *FnPg* Log$_2$ Ratio	Log$_2$ Sum	*q*-Value	*p*-Value	Raw *FnPg*	Raw *FnSg*	Normalized *FnPg*	Normalized *FnSg*	Description
FN0501	0.449	17.998	1.558e-1	2.044e-1	183	420	267.2890	546.6790	AAL94697.1| Ornithine decarboxylase
					571	649	608.6970	649.0000	
FN0502	-0.028	9.075							AAL94698.1| Phosphoheptose isomerase
					22	23	23.4524	23.0000	
FN0503	0.369	10.998	1.683e-1	2.34e-1	18	39	26.2907	50.7630	AAL94699.1| Transcriptional regulatory protein, LYSR family
					50	52	53.3010	52.0000	
FN0505	-1.240	11.169	1.138e-1	1.081e-1	28	18	40.8967	23.4291	AAL94701.1| Anthranilate synthase component II
					100	39	106.6019	39.0000	
FN0506	-0.647	13.894	1.081e-1	9.817e-2	134	67	195.7198	87.2083	AAL94702.1| Arginyl-tRNA synthetase
					106	110	112.9980	110.0000	
FN0511	0.189	11.954	3.714e-1	7.714e-1	10	58	14.6060	75.4938	AAL94707.1| D-lactate dehydrogenase
					97	59	103.4039	59.0000	
FN0512	-0.489	16.189	2.394e-3	1.65e-4	223	171	325.7128	222.5764	AAL94708.1| Flavoprotein
					302	239	321.9378	239.0000	
FN0513	0.555	7.795	1.212e-1	1.219e-1	11	17	16.0666	22.1275	AAL94709.1| Flavodoxin
					8	14	8.5282	14.0000	
FN0515	1.249	4.604				4		5.2065	AAL94711.1| Acriflavin resistance protein D
					3	10	3.1981	10.0000	
FN0516									AAL94712.1| Acriflavin resistance protein E
					4		4.2641		
FN0517	0.979	5.808				10		13.0162	AAL94713.1| Outer membrane protein tolC
					5	8	5.3301	8.0000	
FN0519	-0.466	6.760	1.258e-1	1.313e-1	8	9	11.6848	11.7145	AAL94715.1| Hypothetical exported 24-amino acid repeat protein
					12	6	12.7922	6.0000	
FN0522	0.631	7.459				10		13.0162	AAL94718.1| Exonuclease SBCC
					10	20	10.6602	20.0000	
FN0523	0.823	5.086			3	5	4.3818	6.5081	AAL94719.1| Exonuclease SBCD
						9		9.0000	
FN0524	0.131	6.289	3.37e-1	6.635e-1	5	5	7.3030	6.5081	AAL94720.1| DNA helicase II
					9	12	9.5942	12.0000	
FN0525	0.887	14.933	9.659e-2	8.002e-2	38	219	55.5026	285.0540	AAL94721.1| Penicillin-binding protein
					192	196	204.6757	196.0000	

Log$_2$ Ratios: -6 -4 -2 0 2 4 6

Show detected proteins only
Show all proteins
Filter by category: GO: amino acid transport

Proteins found: 1313

Enter (or paste) list of ORFs

Find ORFs

Test	Cutoff
q-Value	.005
p-Value	

Signif	Direction	Applies To
yes	+	ratios, bars
no	n/a	bars
yes	−	ratios, bars
yes	+	*p*-, *q*-Values
yes	−	

Dot Plots | Dot Plots

Protein	*FnSg* vs *FnPg* Log$_2$ Ratio	Log$_2$ Sum	*q*-Value	*p*-Value	Raw *FnPg*	Raw *FnSg*	Normalized *FnPg*	Normalized *FnSg*	Description
FN0526	-0.035	11.865				42		54.6679	AAL94722.1| Florfenicol resistance protein
					58	66	61.8291	66.0000	
FN0527	0.168	11.089	2.08e-1	3.312e-1	26	33	37.9755	42.9533	AAL94723.1| Alanyl-tRNA synthetase
					47	56	50.1029	56.0000	
FN0528	-3.178	13.061	1.321e-1	1.446e-1	340	28	496.6025	36.4453	AAL94724.1| Cold shock protein
					56	25	59.6971	25.0000	
FN0535	-0.792	10.664	1.084e-2	1.924e-3	39	24	56.9632	31.2388	AAL94731.1| Hypothetical protein
					46	30	49.0369	30.0000	
FN0536	0.062	15.130	3.784e-1	7.947e-1	94	159	137.2960	206.9570	AAL94732.1| DNA polymerase III, beta chain
					219	180	233.4582	180.0000	
FN0540	0.419	10.679	2.342e-1	3.957e-1	10	32	14.6060	41.6517	AAL94736.1| Glutamate-1-semialdehyde 2,1-aminomutase
					52	52	55.4330	52.0000	
FN0541						6		7.8097	AAL94737.1| polysaccharide deacetylase
						11		11.0000	
FN0542	0.192	9.997	3.784e-1	7.949e-1	3	31	4.3818	40.3501	AAL94738.1| Beta 1,4 glucosyltransferase
					52	28	55.4330	28.0000	
FN0543	-0.511	8.900	2.444e-1	4.214e-1	8	12	11.6848	15.6194	AAL94739.1| Lipopolysaccharide heptosyltransferase-1
					38	21	40.5087	21.0000	
FN0544									AAL94740.1| ADP-heptose:LPS heptosyltransferase II
					3		3.1981		
FN0546									AAL94742.1| Lipopolysaccharide core biosynthesis protein rfaQ
					6		6.3961		
FN0547	0.900	16.482	6.395e-2	3.507e-2	88	304	128.5324	395.6914	AAL94743.1| RecA protein
					295	431	314.4757	431.0000	
FN0549									AAL94745.1| O-sialoglycoprotein endopeptidase
						13		13.0000	
FN0550	-1.381	8.739	1.179e-1	1.155e-1	34	12	49.6602	15.6194	AAL94746.1| hypothetical Protein
					16	10	17.0563	10.0000	
FN0552	0.058	9.532	3.897e-1	8.338e-1	19	15	27.7513	19.5242	AAL94748.1| Serine racemase
					24	36	25.5845	36.0000	
FN0553	0.524	15.182	1.491e-1	1.876e-1	64	144	93.4781	187.4328	AAL94749.1| D-serine dehydratase
					214	275	228.1281	275.0000	

Log$_2$ Ratios: -6 -4 -2 0 2 4 6

◉ Show detected proteins only
○ Show all proteins
☐ Filter by category: GO: amino acid transport

Proteins found: 1313

Enter (or paste) list of ORFs

Find ORFs

Test	Cutoff
q-Value	.005
p-Value	

	Signif	Direction	Applies To
red	yes	+	ratios, bars
yellow	no	n/a	bars
green	yes	–	ratios, bars
pink	yes	+	*p*- , *q*-Values
light green	yes	–	

Dot Plots | Dot Plots

Protein	FnSg vs FnPg Log$_2$ Ratio	Log$_2$ Sum	*q*-Value	*p*-Value	Raw *FnPg*	Raw *FnSg*	Normalized *FnPg*	Normalized *FnSg*	Description
**FN0554**	0.286	8.642	2.214e-1	3.633e-1	8	17	11.6848	22.1275	AAL94750.1| D-serine permease
					23	22	24.5184	22.0000	
**FN0555**						6		7.8097	AAL94751.1| Transcriptional regulator, MerR family
**FN0556**	-0.517	11.707	2.519e-1	4.396e-1	75	42	109.5447	54.6679	AAL94752.1| unknown
					27	42	28.7825	42.0000	
**FN0557**	0.637	15.248	1.458e-1	1.784e-1	59	242	86.1751	314.9912	AAL94753.1| unknown
					216	177	230.2602	177.0000	
**FN0558**	-1.486	15.058				105		136.6697	AAL94754.1| TraT complement resistance protein precursor
					290	84	309.1456	84.0000	
**FN0559**	-0.272	15.468	2.57e-1	4.516e-1	105	151	153.3625	196.5441	AAL94755.1| Phosphoglucomutase
					295	191	314.4757	191.0000	
**FN0560**									AAL94756.1| Oxygen-independent coproporphyrinogen III oxidase
						6		6.0000	
**FN0561**	-0.774	10.406	4.26e-2	1.684e-2	28	21	40.8967	27.3339	AAL94757.1| Proline synthetase associated protein
					52	29	55.4330	29.0000	
**FN0562**	-0.100	13.516	3.412e-1	6.762e-1	60	93	87.6357	121.0503	AAL94758.1| Hypothetical cytosolic protein
					128	88	136.4505	88.0000	
**FN0563**	0.351	6.660	2.811e-1	5.104e-1	10	9	14.6060	11.7145	AAL94759.1| putative tRNA (5-methylaminomethyl-2-thiouridylate) -methyltransferase
					3	11	3.1981	11.0000	
**FN0576**	0.959	11.867	6.031e-2	3.168e-2	25	81	36.5149	105.4309	AAL94772.1| hypothetical protein
					48	65	51.1689	65.0000	
**FN0577**	0.351	7.455				13		16.9210	AAL94773.1| Hypothetical protein
					11	13	11.7262	13.0000	
**FN0579**	-0.950	19.619	8.566e-2	6.238e-2	1109	588	1619.8004	765.3505	AAL94775.1| Hypothetical cytosolic protein
					821	526	875.2018	526.0000	
**FN0580**	1.308	5.492				7		9.1113	AAL94776.1| Penicillin-binding protein
					4	12	4.2641	12.0000	
**FN0581**	0.873	7.058				12		15.6194	AAL94777.1| Lipoprotein releasing system transmembrane protein lolE
					8		8.5282		
**FN0582**	1.053	9.578				32		41.6517	AAL94778.1| Lipoprotein releasing system ATP-binding protein lolD
					18	38	19.1883	38.0000	

Log$_2$ Ratios: -6 -4 -2 0 2 4 6

Show detected proteins only
Show all proteins
Filter by category:
GO: amino acid transport

Proteins found: 1313

Enter (or paste) list of ORFs

Find ORFs

Test	Cutoff
q-Value	.005
p-Value	

	Signif	Direction	Applies To
red	yes	+	ratios, bars
yellow	no	n/a	bars
green	yes	–	ratios, bars
pink	yes	+	*p*- , *q*-Values
light green	yes	–	

Dot Plots Dot Plots

Protein	*FnSg* vs *FnPg* Log$_2$ Ratio	Log$_2$ Sum	*q*-Value	*p*-Value	Raw *FnPg*	Raw *FnSg*	Normalized *FnPg*	Normalized *FnSg*	Description
FN0583	-0.893	8.076				7		9.1113	AAL94779.1| Hypothetical Exported Protein
					21	15	22.3864	15.0000	
FN0585	-0.859	7.417	1.832e-1	2.703e-1	19	8	27.7513	10.4129	AAL94781.1| Two-component response regulator czcR
					7	9	7.4621	9.0000	
FN0586	-0.420	7.165				9		11.7145	AAL94782.1| Two-component sensor kinase czcS
					13	9	13.8583	9.0000	
FN0590	0.468	10.160	2.207e-1	3.614e-1	8	25	11.6848	32.5404	AAL94786.1| N-acyl-L-amino acid amidohydrolase
					43	47	45.8388	47.0000	
FN0592	0.335	12.775	1.947e-1	3.006e-1	51	93	74.4904	121.0503	AAL94788.1| ATP-dependent DNA helicase pcrA
					70	67	74.6213	67.0000	
FN0593	-0.301	10.957	3.108e-2	9.699e-3	32	31	46.7391	40.3501	AAL94789.1| UDP-3-O-[3-hydroxymyristoyl] N-acetylglucosamine deacetylase
					49	40	52.2349	40.0000	
FN0594	-0.715	14.485				91		118.4471	AAL94790.1| (3R)-hydroxymyristoyl-[acyl carrier protein] dehydratase
					182	118	194.0155	118.0000	
FN0595	-1.261	8.433				10		13.0162	AAL94791.1| Acyl-[acyl-carrier-protein]-UDP-N-acetylglucosamine O-acyltransferase
					27	11	28.7825	11.0000	
FN0596	0.221	8.661	2.788e-1	5.048e-1	8	18	11.6848	23.4291	AAL94792.1| Hypothetical protein
					24	20	25.5845	20.0000	
FN0597	-0.266	10.258	1.98e-2	4.58e-3	27	26	39.4361	33.8420	AAL94793.1| Lipid-A-disaccharide synthase
					35	30	37.3107	30.0000	
FN0598	0.452	8.208	1.233e-1	1.26e-1	7	14	10.2242	18.2226	AAL94794.1| Phospholipid-lipopolysaccharide ABC transporter
					18	22	19.1883	22.0000	
FN0600	0.130	12.627	3.857e-1	8.196e-1	18	61	26.2907	79.3986	AAL94796.1| Hypothetical protein
					118	87	125.7903	87.0000	
FN0602	-0.600	13.535	6.163e-2	3.287e-2	107	63	156.2837	82.0018	AAL94798.1| Hypothetical protein
					105	95	111.9320	95.0000	
FN0603	0.626	6.810				11		14.3178	AAL94799.1| Transcriptional regulatory protein, LYSR family
					8	12	8.5282	12.0000	
FN0608	0.310	14.182	1.945e-1	2.999e-1	56	118	81.7933	153.5908	AAL94804.1| Exoribonuclease II
					153	150	163.1009	150.0000	
FN0609	0.782	6.137							AAL94805.1| Small protein B
					6	11	6.3961	11.0000	

Log$_2$ Ratios

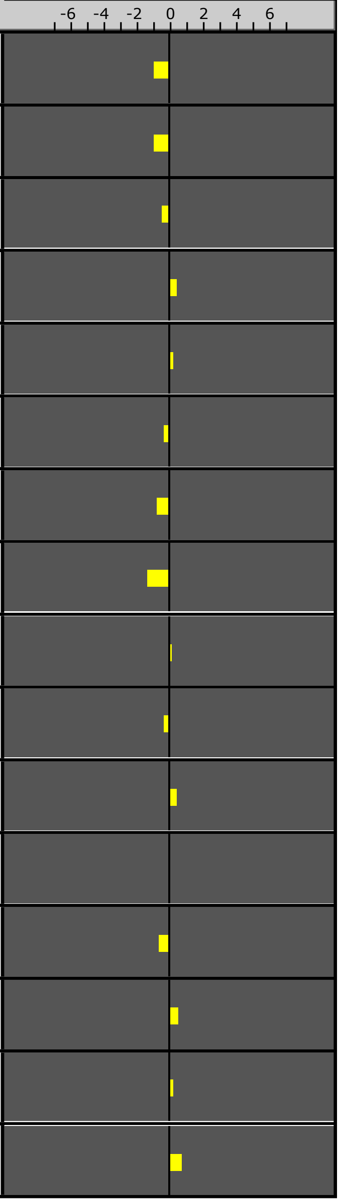

Show detected proteins only
Show all proteins
Filter by category:
GO: amino acid transport

Proteins found: 1313

Enter (or paste) list of ORFs

Find ORFs

Test	Cutoff
q-Value	.005
p-Value	

	Signif	Direction	Applies To
red	yes	+	ratios, bars
yellow	no	n/a	bars
green	yes	−	ratios, bars
pink	yes	+	*p*- , *q*-Values
light green	yes	−	

Dot Plots Dot Plots

Protein	*FnSg vs FnPg* Log$_2$ Ratio	Log$_2$ Sum	*q*-Value	*p*-Value	Raw *FnPg*	Raw *FnSg*	Normalized *FnPg*	Normalized *FnSg*	Description
FN0610	-0.320	15.476	2.7e-1	4.828e-1	93	153	135.8354	199.1473	AAL94806.1| unknown
					320	183	341.1262	183.0000	
FN0611	0.320	19.168	3.881e-2	1.429e-2	461	612	673.3345	796.5893	AAL94807.1| Threonyl-tRNA synthetase
					657	918	700.3747	918.0000	
FN0612	0.387	11.172	2.415e-1	4.143e-1	13	36	18.9877	46.8582	AAL94808.1| Hypothetical protein
					61	63	65.0272	63.0000	
FN0614									AAL94810.1| Export ABC transporter
						3		3.0000	
FN0615					3		4.3818		AAL94811.1| Export ABC transporter
FN0616									AAL94812.1| Hypothetical protein
					7		7.4621		
FN0617	-1.889	15.192	9.783e-2	8.183e-2	380	73	555.0263	95.0180	AAL94813.1| DNA polymerase III, beta chain
					178	106	189.7514	106.0000	
FN0618	-0.326	13.483	2.987e-1	5.562e-1	40	90	58.4238	117.1455	AAL94814.1| Spermidine/putrescine-binding protein
					170	74	181.2233	74.0000	
FN0619	-0.085	7.554	4.027e-1	8.801e-1	4	12	5.8424	15.6194	AAL94815.1| Small-conductance mechanosensitive channel
					21	11	22.3864	11.0000	
FN0621	-0.431	11.289	1.955e-1	3.027e-1	54	37	78.8722	48.1598	AAL94817.1| 4-hydroxybutyrate coenzyme A transferase
					35	38	37.3107	38.0000	
FN0622	1.262	11.804	4.144e-2	1.602e-2	12	57	17.5271	74.1921	AAL94818.1| 8-oxoguanine DNA glycosylase
					56	111	59.6971	111.0000	
FN0625	-0.547	8.360			15		21.9089		AAL94821.1| Aspartate aminotransferase
						15		15.0000	
FN0626									AAL94822.1| Hypothetical cytosolic protein
						12		12.0000	
FN0627	0.231	13.566	3.026e-1	5.665e-1	37	95	54.0420	123.6536	AAL94823.1| Glucosamine--fructose-6-phosphate aminotransferase (isomerizing)
					140	115	149.2427	115.0000	
FN0628						5		6.5081	AAL94824.1| Glucosamine--fructose-6-phosphate aminotransferase (isomerizing)
						7		7.0000	
FN0629	0.595	8.780				25		32.5404	AAL94825.1| PTS system, IID component
					16	19	17.0563	19.0000	

Log$_2$ Ratios: -6 -4 -2 0 2 4 6

Show detected proteins only
Show all proteins
Filter by category:
GO: amino acid transport

Proteins found: 1313

Enter (or paste) list of ORFs

Find ORFs

Test	Cutoff
q-Value	.005
p-Value	

	Signif	Direction	Applies To
red	yes	+	ratios, bars
yellow	no	n/a	bars
green	yes	–	ratios, bars
pink	yes	+	*p*- , *q*-Values
light green	yes	–	

Dot Plots Dot Plots

Protein	FnSg vs FnPg Log$_2$ Ratio	Log$_2$ Sum	*q*-Value	*p*-Value	Raw *FnPg*	Raw *FnSg*	Normalized *FnPg*	Normalized *FnSg*	Description
FN0631	-0.070	8.289				15		19.5242	AAL94827.1| PTS system, IIB component
					17	15	18.1223	15.0000	
FN0633	-0.513	7.485				8		10.4129	AAL94829.1| Replication protein
					15	12	15.9903	12.0000	
FN0634	-1.658	12.056	1.414e-1	1.665e-1	131	28	191.3380	36.4453	AAL94830.1| GTP-binding protein TypA/BipA
					38	37	40.5087	37.0000	
FN0643	-0.635	9.351	1.48e-1	1.846e-1	29	10	42.3573	13.0162	AAL94839.1| hypothetical DNA-binding protein
					20	28	21.3204	28.0000	
FN0644	-0.629	10.902	4.995e-2	2.264e-2	38	21	55.5026	27.3339	AAL94840.1| Uroporphyrin-III C-methyltransferase
					50	43	53.3010	43.0000	
FN0645	-0.410	5.580			8		11.6848		AAL94841.1| Porphobilinogen deaminase
					4	6	4.2641	6.0000	
FN0646									AAL94842.1| Glutamyl-tRNA reductase
						3		3.0000	
FN0649					6		8.7636		AAL94845.1| Exoenzymes regulatory protein aepA precursor
FN0651									AAL94847.1| Ribosomal large subunit pseudouridine synthase D
						6		6.0000	
FN0652	0.643	25.505	9.371e-2	7.596e-2	2352	5960	3435.3206	7757.6347	AAL94848.1| Glyceraldehyde 3-phosphate dehydrogenase
					7137	9493	7608.1796	9493.0000	
FN0653	0.659	12.862	8.06e-2	5.492e-2	32	69	46.7391	89.8115	AAL94849.1| unknown
					85	127	90.6116	127.0000	
FN0654	1.001	19.852	7.062e-3	1.02e-3	368	1156	537.4991	1504.6687	AAL94850.1| Phosphoglycerate kinase
					786	1248	837.8912	1248.000	
FN0655	0.524	7.628				19		24.7307	AAL94851.1| unknown
					11	9	11.7262	9.0000	
FN0657	1.251	10.355				42		54.6679	AAL94853.1| Acetyltransferase
					22	57	23.4524	57.0000	
FN0658	-0.254	11.894	2.477e-3	1.78e-4	47	43	68.6480	55.9695	AAL94854.1| ABC transporter substrate-binding protein
					62	57	66.0932	57.0000	
FN0660	-0.419	6.456	1.751e-1	2.506e-1	9	4	13.1454	5.2065	AAL94856.1| ABC transporter ATP-binding protein
					8	11	8.5282	11.0000	

Log$_2$ Ratios: -6 -4 -2 0 2 4 6

Show detected proteins only
Show all proteins
Filter by category:
GO: amino acid transport

Proteins found: 1313

Enter (or paste) list of ORFs

Find ORFs

Test	Cutoff
q-Value	.005
p-Value	

	Signif	Direction	Applies To
red	yes	+	ratios, bars
yellow	no	n/a	bars
green	yes	–	ratios, bars
pink	yes	+	*p*- , *q*-Values
light green	yes	–	*p*- , *q*-Values

Dot Plots Dot Plots

Protein	*FnSg* vs *FnPg* Log$_2$ Ratio	Log$_2$ Sum	*q*-Value	*p*-Value	Raw *FnPg*	Raw *FnSg*	Normalized *FnPg*	Normalized *FnSg*	Description
FN0662	0.198	11.806	3.768e-1	7.893e-1	5	47	7.3030	61.1760	AAL94858.1| Formiminoglutamase
					98	67	104.4699	67.0000	
FN0664	1.016	13.333	1.705e-1	2.394e-1	73	46	106.6235	59.8744	AAL94860.1| 2-nitropropane dioxygenase
					34	229	36.2447	229.0000	
FN0666						4		5.2065	AAL94862.1| Hypothetical protein
						7		7.0000	
FN0668	0.016	8.665	4.265e-1	9.708e-1	7	15	10.2242	19.5242	AAL94864.1| High-affinity zinc uptake system protein znuA precursor
					28	21	29.8485	21.0000	
FN0672	0.958	7.142				17		22.1275	AAL94868.1| ATPase
					8	11	8.5282	11.0000	
FN0675	1.259	22.417	7.412e-2	4.62e-2	331	2016	483.4571	2624.0590	AAL94871.1| 60 kDa chaperonin GROEL
					2417	4698	2576.5686	4698.0000	
FN0676	-0.177	10.991				36		46.8582	AAL94872.1| 10 kDa chaperonin GROES
					45	38	47.9709	38.0000	
FN0677	0.032	12.193	4.123e-1	9.158e-1	62	51	90.5569	66.3824	AAL94873.1| Hypothetical protein
					42	72	44.7728	72.0000	
FN0678	1.260	10.418	6.35e-2	3.463e-2	5	48	7.3030	62.4776	AAL94874.1| Ser/Thr protein kinase
					38	52	40.5087	52.0000	
FN0679	-0.957	7.297			21		30.6725		AAL94875.1| GTPase
					4	9	4.2641	9.0000	
FN0680	0.608	6.793							AAL94876.1| Ribulose-phosphate 3-epimerase
					8	13	8.5282	13.0000	
FN0681	-0.117	16.579	2.764e-1	4.986e-1	260	212	379.7548	275.9427	AAL94877.1| Transcriptional regulator, MarR family
					255	325	271.8349	325.0000	
FN0682	0.639	9.469	6.915e-2	4.046e-2	19	28	27.7513	36.4453	AAL94878.1| Fibronectin-binding protein-like protein A
					14	30	14.9243	30.0000	
FN0684	-0.796	8.495	1.86e-1	2.775e-1	8	16	11.6848	20.8259	AAL94880.1| Prismane protein
					36	8	38.3767	8.0000	
FN0685	-0.486	13.208				81		105.4309	AAL94881.1| Sodium/pantothenate symporter
					108	59	115.1301	59.0000	
FN0688	1.295	11.094				58		75.4938	AAL94884.1| Hypothetical protein
					28	71	29.8485	71.0000	

Log$_2$ Ratios: -6 -4 -2 0 2 4 6

Show detected proteins only
Show all proteins
Filter by category:
GO: amino acid transport

Proteins found: 1313

Enter (or paste) list of ORFs

Find ORFs

Test	Cutoff
q-Value	.005
p-Value	

Signif	Direction	Applies To
yes	+	ratios, bars
no	n/a	bars
yes	−	ratios, bars
yes	+	*p*- , *q*-Values
yes	−	

Dot Plots Dot Plots

Protein	FnSg vs FnPg Log$_2$ Ratio	Log$_2$ Sum	*q*-Value	*p*-Value	Raw *FnPg*	Raw *FnSg*	Normalized *FnPg*	Normalized *FnSg*	Description
FN0689	0.695	14.514	1.458e-1	1.783e-1	42	196	61.3450	255.1168	AAL94885.1| Hypothetical protein
					168	134	179.0912	134.0000	
FN0692						4		5.2065	AAL94888.1| Nitrogen regulation protein NIFR3
						8		8.0000	
FN0693					15		21.9089		AAL94889.1| DNA mismatch repair protein mutS
FN0694	0.664	11.419	5.857e-4	8.19e-6	27	49	39.4361	63.7792	AAL94890.1| S-layer protein
					41	68	43.7068	68.0000	
FN0695	0.749	10.414	7.742e-2	5.045e-2	12	39	17.5271	50.7630	AAL94891.1| ABC transporter ATP-binding protein
					37	45	39.4427	45.0000	
FN0697	0.234	14.553	1.472e-1	1.822e-1	114	117	166.5079	152.2891	AAL94893.1| Alanyl-tRNA synthetase
					112	184	119.3942	184.0000	
FN0699	-0.498	9.741	1.948e-1	3.009e-1	33	24	48.1997	31.2388	AAL94895.1| Protein translocase subunit secD
					20	18	21.3204	18.0000	
FN0700	-1.237	7.147	8.815e-2	6.643e-2	17	5	24.8301	6.5081	AAL94896.1| Protein translocase subunit secF
					11	9	11.7262	9.0000	
FN0701	-0.565	14.468	2.414e-3	1.68e-4	120	88	175.2715	114.5423	AAL94897.1| Methyltransferase
					179	133	190.8175	133.0000	
FN0705	0.395	12.987	1.301e-1	1.401e-1	39	75	56.9632	97.6212	AAL94901.1| DNA polymerase I
					94	109	100.2058	109.0000	
FN0706									AAL94902.1| Hypothetical cytosolic protein
						4		4.0000	
FN0707	-0.758	6.848	1.654e-1	2.271e-1	14	5	20.4483	6.5081	AAL94903.1| Riboflavin kinase
					7	10	7.4621	10.0000	
FN0710	-0.226	11.609	1.391e-1	1.607e-1	47	41	68.6480	53.3663	AAL94906.1| Hypothetical protein
					49	50	52.2349	50.0000	
FN0711	0.499	8.858				14		18.2226	AAL94907.1| Phosphopantothenate--cysteine ligase
					17	33	18.1223	33.0000	
FN0714	0.579	6.868	8.837e-2	6.68e-2	7	8	10.2242	10.4129	AAL94910.1| NADH oxidase
					7	16	7.4621	16.0000	
FN0715	-0.108	14.529	3.952e-1	8.53e-1	177	120	258.5254	156.1940	AAL94911.1| Hypothetical protein
					57	140	60.7631	140.0000	

Log$_2$ Ratios: -6 -4 -2 0 2 4 6

Show detected proteins only
Show all proteins
Filter by category:
GO: amino acid transport

Proteins found: 1313

Enter (or paste) list of ORFs

Find ORFs

Test	Cutoff
q-Value	.005
p-Value	

	Signif	Direction	Applies To
red	yes	+	ratios, bars
yellow	no	n/a	bars
green	yes	−	ratios, bars
pink	yes	+	*p*- , *q*-Values
light green	yes	−	

Dot Plots Dot Plots

Protein	FnSg vs FnPg				Raw		Normalized		Description
	Log_2 Ratio	Log_2 Sum	*q*-Value	*p*-Value	*FnPg*	*FnSg*	*FnPg*	*FnSg*	
FN0716	0.735	11.251	1.181e-1	1.158e-1	40	51	58.4238	66.3824	AAL94912.1| hypothetical protein
					17	61	18.1223	61.0000	
FN0717									AAL94913.1| Ribosomal small subunit pseudouridine synthase A
						5		5.0000	
FN0720	0.104	12.681	3.747e-1	7.823e-1	45	40	65.7268	52.0647	AAL94916.1| Protein Translation Elongation Factor P (EF-P)
					85	116	90.6116	116.0000	
FN0721	1.207	9.191	5.226e-2	2.476e-2	5	28	7.3030	36.4453	AAL94917.1| Hypothetical protein
					23	37	24.5184	37.0000	
FN0722	0.199	6.984	3.202e-1	6.151e-1	10	7	14.6060	9.1113	AAL94918.1| WD-repeat family protein
					6	15	6.3961	15.0000	
FN0723	0.230	4.414							AAL94919.1| Hypothetical protein
					4	5	4.2641	5.0000	
FN0724	0.198	11.870	2.208e-1	3.618e-1	41	40	59.8844	52.0647	AAL94920.1| Flavodoxin
					51	79	54.3670	79.0000	
FN0725	0.942	9.234	8.216e-2	5.711e-2	6	20	8.7636	26.0323	AAL94921.1| Molybdopterin biosynthesis MoeB protein
					25	42	26.6505	42.0000	
FN0728	0.044	11.030	2.072e-2	4.912e-3	31	36	45.2785	46.8582	AAL94924.1| Hypothetical protein
					42	46	44.7728	46.0000	
FN0729	-0.672	13.985	1.529e-1	1.974e-1	153	92	223.4711	119.7487	AAL94925.1| Phosphoglycerate mutase
					92	82	98.0738	82.0000	
FN0731	-1.416	9.709	8.692e-2	6.439e-2	45	18	65.7268	23.4291	AAL94927.1| Hypothetical protein
					27	12	28.7825	12.0000	
FN0733	-0.640	11.992	7.913e-2	5.28e-2	66	34	96.3993	44.2550	AAL94929.1| Peptidase T
					59	58	62.8951	58.0000	
FN0734						3		3.9048	AAL94930.1| Fe-S oxidoreductase
						4		4.0000	
FN0735	-1.836	12.081	1.241e-1	1.275e-1	136	32	198.6410	41.6517	AAL94931.1| Cell surface protein
					47	28	50.1029	28.0000	
FN0736	1.465	7.017	7.327e-3	1.095e-3	5	16	7.3030	20.8259	AAL94932.1| Methyltransferase
					6	17	6.3961	17.0000	
FN0737									AAL94933.1| Hypothetical protein
					28		29.8485		

Log_2 Ratios: -6 -4 -2 0 2 4 6

Show detected proteins only
Show all proteins
Filter by category:
GO: amino acid transport

Proteins found: 1313

Enter (or paste) list of ORFs

Find ORFs

Test	Cutoff
q-Value	.005
p-Value	

	Signif	Direction	Applies To
red	yes	+	ratios, bars
yellow	no	n/a	bars
green	yes	–	ratios, bars
pink	yes	+	*p*- , *q*-Values
light green	yes	–	

Dot Plots Dot Plots

FnSg vs FnPg

Fusobacterium nucleatum

Hackett Laboratory UW

Fn Summary Table | FnPg vs Fn | FnSg vs Fn | FnPgSg vs Fn | FnPgSg vs FnPg | FnSg vs FnPg | FnPgSg vs FnSg | Fn Coverage

Protein	*FnSg* vs *FnPg* Log$_2$ Ratio	Log$_2$ Sum	*q*-Value	*p*-Value	Raw *FnPg*	Raw *FnSg*	Normalized *FnPg*	Normalized *FnSg*	Description
FN0738	-0.137	10.699	3.267e-1	6.335e-1	33	39	48.1997	50.7630	AAL94934.1| Hypothetical exported 24-amino acid repeat protein
					35	27	37.3107	27.0000	
FN0739	-0.219	14.903	1.404e-1	1.64e-1	125	144	182.5744	187.4328	AAL94935.1| Formiminotetrahydrofolate cyclodeaminase
					183	137	195.0815	137.0000	
FN0740	0.658	18.227	1.348e-1	1.506e-1	147	625	214.7075	813.5104	AAL94936.1| Imidazolonepropionase
					626	578	667.3281	578.0000	
FN0741	0.216	18.432	3.115e-1	5.908e-1	206	551	300.8827	717.1907	AAL94937.1| Glutamate formiminotransferase
					753	565	802.7125	565.0000	
FN0742	-0.176	11.674				35		45.5566	AAL94938.1| unknown
					57	62	60.7631	62.0000	
FN0743						3		3.9048	AAL94939.1| ATP-dependent helicase, DinG family
						6		6.0000	
FN0745	0.700	14.439	1.084e-1	9.875e-2	47	179	68.6480	232.9894	AAL94941.1| metal dependent phosphohydrolase
					155	147	165.2330	147.0000	
FN0746	-0.231	7.795	3.577e-1	7.271e-1	17	5	24.8301	6.5081	AAL94942.1| Hypothetical Metal-Binding Protein
					7	21	7.4621	21.0000	
FN0750	1.496	10.329	3.169e-3	2.864e-4	11	48	16.0666	62.4776	AAL94946.1| Hypothetical protein
					25	58	26.6505	58.0000	
FN0751	0.864	9.045	4.183e-2	1.629e-2	8	20	11.6848	26.0323	AAL94947.1| L-asparaginase I
					21	36	22.3864	36.0000	
FN0752	0.512	8.460	2.058e-1	3.26e-1	4	16	5.8424	20.8259	AAL94948.1| Proline iminopeptidase
					24	24	25.5845	24.0000	
FN0753	-0.738	12.667	7.267e-4	1.671e-5	74	46	108.0841	59.8744	AAL94949.1| Glutamyl-tRNA(Gln) amidotransferase subunit B
					94	65	100.2058	65.0000	
FN0754	-0.254	13.528	2.054e-1	3.252e-1	64	63	93.4781	82.0018	AAL94950.1| Glutamyl-tRNA(Gln) amidotransferase subunit A
					135	117	143.9126	117.0000	
FN0755	-0.064	10.115	2.386e-1	4.074e-1	24	27	35.0543	35.1436	AAL94951.1| Glutamyl-tRNA(Gln) amidotransferase subunit C
					31	30	33.0466	30.0000	
FN0758	0.513	15.076	8.518e-2	6.162e-2	97	202	141.6778	262.9265	AAL94954.1| Rod shape-determining protein mreB
					159	181	169.4971	181.0000	
FN0761	-1.076	9.604				18		23.4291	AAL94957.1| Bvg accessory factor
					38	15	40.5087	15.0000	

Log$_2$ Ratios

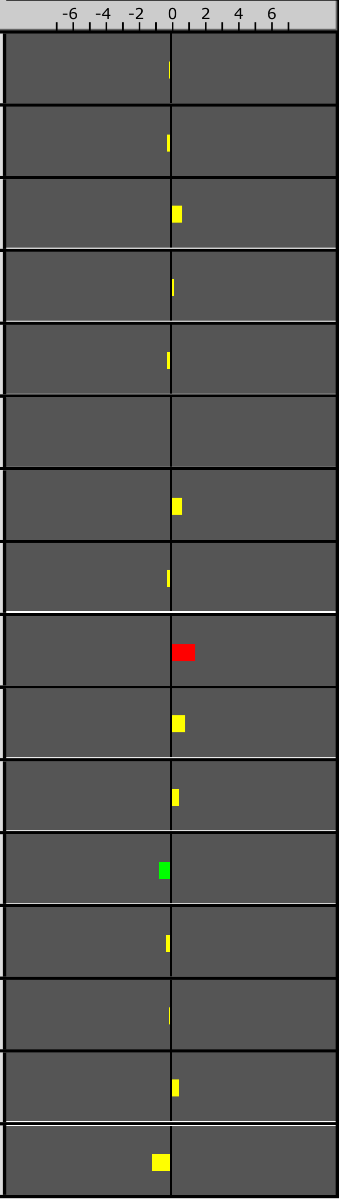

Show detected proteins only
Show all proteins
Filter by category:
GO: amino acid transport

Proteins found: 1313

Enter (or paste) list of ORFs

Find ORFs

Test	Cutoff
q-Value	.005
p-Value	

	Signif	Direction	Applies To
red	yes	+	ratios, bars
yellow	no	n/a	bars
green	yes	−	ratios, bars
pink	yes	+	*p*- , *q*-Values
light green	yes	−	*p*- , *q*-Values

Dot Plots Dot Plots

FnSg vs FnPg

Fusobacterium nucleatum

Hackett Laboratory UW

Fn Summary Table | FnPg vs Fn | FnSg vs Fn | FnPgSg vs Fn | FnPgSg vs FnPg | FnSg vs FnPg | FnPgSg vs FnSg | Fn Coverage

Protein	*FnSg* vs *FnPg* Log_2 Ratio	Log_2 Sum	*q*-Value	*p*-Value	Raw *FnPg*	Raw *FnSg*	Normalized *FnPg*	Normalized *FnSg*	Description
FN0765	-1.315	4.485							AAL94961.1| tRNA (5-methylaminomethyl -2-thiouridylate) -methyltransferase
					7	3	7.4621	3.0000	
FN0768									AAL94964.1| Hemin receptor
					4		4.2641		
FN0771						3		3.9048	AAL94967.1| Oxygen-independent coproporphyrinogen III oxidase
						7		7.0000	
FN0774	-1.602	10.676	4.351e-2	1.752e-2	60	18	87.6357	23.4291	AAL94970.1| Hypothetical cytosolic protein
					50	23	53.3010	23.0000	
FN0775	-0.126	16.431	3.637e-1	7.462e-1	126	229	184.0350	298.0702	AAL94971.1| Aspartyl aminopeptidase
					410	271	437.0679	271.0000	
FN0776	0.545	13.004	2.901e-3	2.384e-4	48	86	70.1086	111.9390	AAL94972.1| Aspartate-ammonia ligase
					75	107	79.9514	107.0000	
FN0777	0.754	11.881	1.469e-2	2.998e-3	29	68	42.3573	88.5099	AAL94973.1| GTP-binding protein lepA
					49	71	52.2349	71.0000	
FN0778	0.571	8.742	1.696e-1	2.373e-1	5	18	7.3030	23.4291	AAL94974.1| Methyltransferase
					25	27	26.6505	27.0000	
FN0779	0.819	5.648				6		7.8097	AAL94975.1| Putative GTPases (G3E family)
					5	11	5.3301	11.0000	
FN0783	-0.310	23.073	8.181e-2	5.661e-2	2527	1871	3690.9248	2435.3246	AAL94979.1| acyl-CoA dehydrogenase
					2744	2900	2925.1569	2900.0000	
FN0784	-0.025	22.273	4.104e-1	9.089e-1	1903	1910	2779.5132	2486.0876	AAL94980.1| Electron transfer flavoprotein beta-subunit
					1653	1976	1762.1299	1976.0000	
FN0785	-0.085	21.951	2.939e-1	5.434e-1	1553	1307	2268.3048	1701.2128	AAL94981.1| Electron transfer flavoprotein alpha-subunit
					1763	2208	1879.3920	2208.0000	
FN0788	-0.113	10.128	2.482e-1	4.307e-1	25	21	36.5149	27.3339	AAL94984.1| unknown
					31	37	33.0466	37.0000	
FN0790	0.937	6.318	3.515e-2	1.213e-2	3	9	4.3818	11.7145	AAL94986.1| Xylose repressor
					8	13	8.5282	13.0000	
FN0791	-0.105	18.471	3.015e-1	5.636e-1	367	513	536.0385	667.7293	AAL94987.1| Histidine ammonia-lyase
					670	495	714.2329	495.0000	
FN0792	0.197	20.863	2.865e-1	5.241e-1	573	1095	836.9212	1425.2701	AAL94988.1| Urocanate hydratase
					1635	1532	1742.9415	1532.0000	

Log_2 Ratios: -6 -4 -2 0 2 4 6

Show detected proteins only
Show all proteins
Filter by category:
GO: amino acid transport

Proteins found: 1313

Enter (or paste) list of ORFs

Find ORFs

Test	Cutoff
q-Value	.005
p-Value	

	Signif	Direction	Applies To
red	yes	+	ratios, bars
yellow	no	n/a	bars
green	yes	−	ratios, bars
pink	yes	+	*p*- , *q*-Values
light green	yes	−	

Dot Plots Dot Plots

Protein	FnSg vs FnPg Log2 Ratio	Log2 Sum	q-Value	p-Value	Raw FnPg	Raw FnSg	Normalized FnPg	Normalized FnSg	Description
FN0793									AAL94989.1| Sodium/glutamate symport carrier protein
					12		12.7922		
FN0794	0.547	8.545				19		24.7307	AAL94990.1| Hypothetical protein
					15	22	15.9903	22.0000	
FN0796	-0.052	15.039	3.704e-1	7.68e-1	104	144	151.9019	187.4328	AAL94992.1| Pyruvate,phosphate dikinase
					208	173	221.7320	173.0000	
FN0798	1.523	8.750	1.185e-1	1.167e-1	8	41	11.6848	53.3663	AAL94994.1| Fructose-1,6-bisphosphatase
					12	17	12.7922	17.0000	
FN0799	0.025	8.992	4.223e-1	9.542e-1	8	15	11.6848	19.5242	AAL94995.1| Isoamylase
					31	26	33.0466	26.0000	
FN0800	0.336	10.731	3.194e-1	6.127e-1	5	32	7.3030	41.6517	AAL94996.1| Amino acid-binding protein
					62	51	66.0932	51.0000	
FN0801	0.243	8.768				18		23.4291	AAL94997.1| Amino acid transport ATP-binding protein
					18	22	19.1883	22.0000	
FN0802									AAL94998.1| Amino acid transport system permease protein
					6		6.3961		
FN0803	-0.571	11.124	6.377e-2	3.49e-2	46	25	67.1874	32.5404	AAL94999.1| Cytochrome C-TYPE biogenesis protein ccdA
					45	45	47.9709	45.0000	
FN0805	-0.132	7.675	1.304e-1	1.407e-1	11	11	16.0666	14.3178	AAL95001.1| Hypothetical protein
					13	13	13.8583	13.0000	
FN0806	0.123	12.367	1.288e-1	1.374e-1	45	62	65.7268	80.7002	AAL95002.1| SpoIID homolog
					69	71	73.5553	71.0000	
FN0807	-1.344	9.052	1.066e-3	3.371e-5	24	13	35.0543	16.9210	AAL95003.1| 3-deoxy-manno-octulosonate cytidylyltransferase
					36	12	38.3767	12.0000	
FN0808	0.115	12.609	2.173e-1	3.531e-1	58	68	84.7145	88.5099	AAL95004.1| Phosphoglycerate mutase
					63	76	67.1592	76.0000	
FN0809	0.908	7.092							AAL95005.1| 23S rRNA methyltransferase
					8	16	8.5282	16.0000	
FN0810	0.068	11.832	4.141e-1	9.228e-1	45	12	65.7268	15.6194	AAL95006.1| Low-specificity threonine aldolase
					49	108	52.2349	108.0000	
FN0811						4		5.2065	AAL95007.1| Hypothetical protein
						5		5.0000	

Log2 Ratios: -6 -4 -2 0 2 4 6

Show detected proteins only
Show all proteins
Filter by category:
GO: amino acid transport

Proteins found: 1313

Enter (or paste) list of ORFs

Find ORFs

Test	Cutoff
q-Value	.005
p-Value	

Signif	Direction	Applies To
yes	+	ratios, bars
no	n/a	bars
yes	−	ratios, bars
yes	+	p- , q-Values
yes	−	

Dot Plots Dot Plots

Protein	*FnSg* vs *FnPg* Log$_2$ Ratio	Log$_2$ Sum	*q*-Value	*p*-Value	Raw *FnPg*	Raw *FnSg*	Normalized *FnPg*	Normalized *FnSg*	Description
FN0813	0.526	11.354				49		63.7792	AAL95009.1| Transcriptional regulator, TetR family
					40	59	42.6408	59.0000	
FN0814	-0.998	8.654	1.97e-1	3.062e-1	33	8	48.1997	10.4129	AAL95010.1| Propionate CoA-transferase
					8	18	8.5282	18.0000	
FN0816					40		58.4238		AAL95012.1| dehydrogenase with MaoC-like domain
FN0818	-1.239	17.242	9.136e-2	7.177e-2	567	224	828.1576	291.5621	AAL95014.1| DNA-binding protein HU
					358	221	381.6349	221.0000	
FN0819	0.016	11.585	4.201e-1	9.459e-1	47	38	68.6480	49.4614	AAL95015.1| Tetratricopeptide repeat family protein
					39	62	41.5748	62.0000	
FN0820	-0.228	14.652	2.305e-1	3.862e-1	94	91	137.2960	118.4471	AAL95016.1| Mercuric reductase
					197	178	210.0058	178.0000	
FN0821	-1.605	11.407	1.204e-1	1.202e-1	96	29	140.2172	37.7469	AAL95017.1| Hypothetical protein
					39	22	41.5748	22.0000	
FN0823	1.308	12.200	8.822e-3	1.439e-3	21	79	30.6725	102.8277	AAL95019.1| GTP-binding protein hflX
					53	113	56.4990	113.0000	
FN0824						4		5.2065	AAL95020.1| hypothetical cytosolic protein
FN0825	-0.782	10.524	1.472e-1	1.822e-1	20	25	29.2119	32.5404	AAL95021.1| Hypothetical cytosolic protein
					67	26	71.4233	26.0000	
FN0826	0.340	8.158	2.634e-1	4.668e-1	14	20	20.4483	26.0323	AAL95022.1| periplasmic component of efflux system
					9	12	9.5942	12.0000	
FN0827	1.573	9.040	6.343e-3	8.378e-4	8	27	11.6848	35.1436	AAL95023.1| ABC transporter ATP-binding protein
					14	44	14.9243	44.0000	
FN0828						6		7.8097	AAL95024.1| ABC transporter permease protein
						4		4.0000	
FN0830	0.165	12.616	1.271e-1	1.338e-1	55	59	80.3328	76.7954	AAL95026.1| Hypothetical protein
					65	91	69.2913	91.0000	
FN0831					13		18.9877		AAL95027.1| Hemin receptor
					6		6.3961		
FN0832	-1.597	11.335	1.593e-1	2.126e-1	105	18	153.3625	23.4291	AAL95028.1| Hypothetical protein
					22	35	23.4524	35.0000	

Log$_2$ Ratios: -6 -4 -2 0 2 4 6

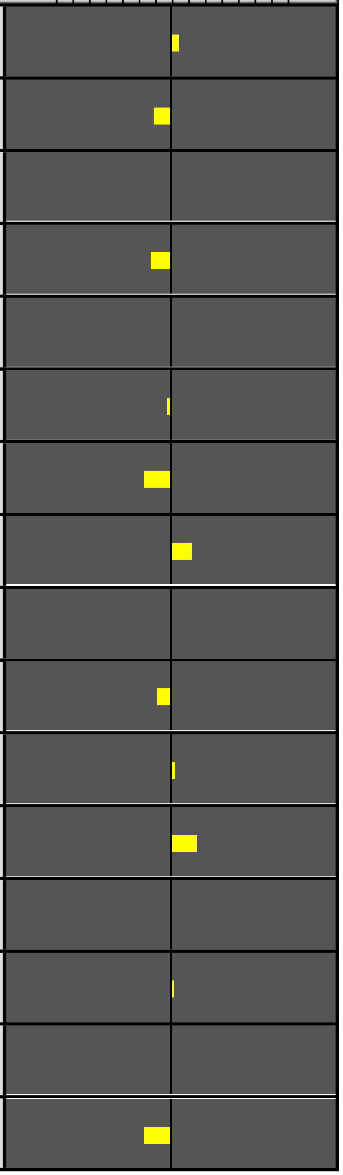

Show detected proteins only
Show all proteins
Filter by category:
GO: amino acid transport

Proteins found: 1313

Enter (or paste) list of ORFs

Find ORFs

Test	Cutoff
q-Value	.005
p-Value	

	Signif	Direction	Applies To
red	yes	+	ratios, bars
yellow	no	n/a	bars
green	yes	−	ratios, bars
pink	yes	+	*p*- , *q*-Values
light green	yes	−	

Dot Plots Dot Plots

Protein	*FnSg* vs *FnPg* Log$_2$ Ratio	Log$_2$ Sum	*q*-Value	*p*-Value	Raw *FnPg*	Raw *FnSg*	Normalized *FnPg*	Normalized *FnSg*	Description	Log$_2$ Ratios (-6 -4 -2 0 2 4 6)
FN0833	2.462	7.291				29		37.7469	AAL95029.1| Hypothetical protein	
					5	21	5.3301	21.0000		
FN0834	-1.275	8.610	1.84e-1	2.724e-1	37	8	54.0420	10.4129	AAL95030.1| Hypothetical Exported Protein	
					7	15	7.4621	15.0000		
FN0835					27		39.4361		AAL95031.1| Hypothetical protein	
					4		4.2641			
FN0836	-1.130	9.002	1.078e-1	9.769e-2	32	12	46.7391	15.6194	AAL95032.1| Hypothetical protein	
					19	15	20.2544	15.0000		
FN0837									AAL95033.1| Integrase/recombinase	
						8		8.0000		
FN0846	-0.348	13.288	3.225e-2	1.035e-2	83	74	121.2294	96.3196	AAL95042.1| Hypothetical Exported Protein	
					98	81	104.4699	81.0000		
FN0847	0.475	6.660				9		11.7145	AAL95043.1| TPR-repeat-containing proteins	
					8	12	8.5282	12.0000		
FN0848					5		7.3030		AAL95044.1| Hypothetical protein	
FN0849	-0.199	11.782	7.91e-2	5.276e-2	44	39	64.2662	50.7630	AAL95045.1| 8-amino-7-oxononanoate synthase	
					59	60	62.8951	60.0000		
FN0850	0.764	10.380	3.355e-2	1.111e-2	15	37	21.9089	48.1598	AAL95046.1| Hypothetical cytosolic protein	
					32	47	34.1126	47.0000		
FN0853	-0.045	13.711	3.647e-1	7.496e-1	91	96	132.9142	124.9552	AAL95049.1| Glycogen synthase	
					96	103	102.3379	103.0000		
FN0854	-0.137	14.775	2.379e-1	4.056e-1	139	114	203.0228	148.3843	AAL95050.1| Glucose-1-phosphate adenylyltransferase	
					139	171	148.1767	171.0000		
FN0855	0.616	15.846	5.671e-2	2.86e-2	102	238	148.9807	309.7847	AAL95051.1| Glucose-1-phosphate adenylyltransferase	
					228	291	243.0524	291.0000		
FN0856	-0.253	14.724	4.334e-2	1.739e-2	124	124	181.1138	161.4005	AAL95052.1| 1,4-alpha-glucan branching enzyme	
					167	140	178.0252	140.0000		
FN0857	0.426	16.750	8.631e-2	6.341e-2	155	304	226.3923	395.6914	AAL95053.1| Glycogen phosphorylase	
					325	374	346.4563	374.0000		
FN0858	0.125	11.791	3.54e-1	7.155e-1	35	31	51.1208	40.3501	AAL95054.1| 4-alpha-glucanotransferase	
					59	84	62.8951	84.0000		

Show detected proteins only
Show all proteins
Filter by category:
GO: amino acid transport

Proteins found: 1313

Enter (or paste) list of ORFs

Find ORFs

Test	Cutoff
q-Value	.005
p-Value	

Signif	Direction	Applies To
yes	+	ratios, bars
no	n/a	bars
yes	–	ratios, bars
yes	+	*p*-, *q*-Values
yes	–	

Dot Plots Dot Plots

Protein	*FnSg* vs *FnPg* Log$_2$ Ratio	Log$_2$ Sum	*q*-Value	*p*-Value	Raw *FnPg*	Raw *FnSg*	Normalized *FnPg*	Normalized *FnSg*	Description
**FN0865**	0.081	15.415	4.125e-1	9.168e-1	17	172	24.8301	223.8780	AAL95061.1| unknown
					358	206	381.6349	206.0000	
**FN0867**	0.670	10.938	2.326e-2	5.917e-3	24	39	35.0543	50.7630	AAL95063.1| Long-chain-fatty-acid--CoA ligase
					33	61	35.1786	61.0000	
**FN0868**									AAL95064.1| ATPases of the PP superfamily
						8		8.0000	
**FN0869**	0.953	5.137				5		6.5081	AAL95065.1| Hydrolase (HAD superfamily)
					4	10	4.2641	10.0000	
**FN0871**	-0.713	7.471				6		7.8097	AAL95067.1| 3-dehydroquinate synthase
					16	13	17.0563	13.0000	
**FN0873**	-0.259	11.850	3.409e-1	6.754e-1	18	50	26.2907	65.0808	AAL95069.1| Protease IV
					100	46	106.6019	46.0000	
**FN0874**	-1.230	5.874							AAL95070.1| Phosphohydrolase (MUTT/NUDIX family protein)
					11	5	11.7262	5.0000	
**FN0875**						6		7.8097	AAL95071.1| 23S rRNA methyltransferase
**FN0878**	0.937	8.392	6.663e-2	3.776e-2	5	19	7.3030	24.7307	AAL95074.1| Transcriptional regulator, GntR family
					18	26	19.1883	26.0000	
**FN0884**						3		3.9048	AAL95079.1| Hemin transport system permease protein hmuU
**FN0887**	0.209	10.204	2.359e-1	4.001e-1	16	26	23.3695	33.8420	AAL95083.1| Oligoendopeptidase F
					38	40	40.5087	40.0000	
**FN0888**					15		21.9089		AAL95084.1| Uracil permease
					14		14.9243		
**FN0889**	-1.822	8.162			37		54.0420		AAL95085.1| hypothetical protein
					9	9	9.5942	9.0000	
**FN0892**						10		13.0162	AAL95088.1| Phosphoserine phosphatase
						5		5.0000	
**FN0893**	0.908	6.707							AAL95089.1| Hypothetical protein
					7	14	7.4621	14.0000	
**FN0896**	-0.444	7.378	2.198e-1	3.593e-1	6	7	8.7636	9.1113	AAL95092.1| Hypothetical protein
					20	13	21.3204	13.0000	

Log$_2$ Ratios: -6 -4 -2 0 2 4 6

Show detected proteins only
Show all proteins
Filter by category:
GO: amino acid transport

Proteins found: 1313

Enter (or paste) list of ORFs

Find ORFs

Test	Cutoff
q-Value	.005
p-Value	

	Signif	Direction	Applies To
red	yes	+	ratios, bars
yellow	no	n/a	bars
green	yes	–	ratios, bars
pink	yes	+	*p*- , *q*-Values
light green	yes	–	

Dot Plots | Dot Plots

Fusobacterium nucleatum

Hackett Laboratory UW

Protein	*FnSg* vs *FnPg* Log$_2$ Ratio	Log$_2$ Sum	*q*-Value	*p*-Value	Raw *FnPg*	Raw *FnSg*	Normalized *FnPg*	Normalized *FnSg*	Description
**FN0898**	0.373	5.202				6		7.8097	AAL95094.1| Hypothetical protein
					5	6	5.3301	6.0000	
**FN0900**	1.742	6.902			6		8.7636		AAL95096.1| Metal dependent hydrolase
					3	20	3.1981	20.0000	
**FN0901**	0.597	7.121				10		13.0162	AAL95097.1| DNA polymerase, bacteriophage-type
					9	16	9.5942	16.0000	
**FN0903**	-0.003	9.315	4.299e-1	9.845e-1	20	18	29.2119	23.4291	AAL95099.1| Polysialic acid capsule expression protein kpsF
					20	27	21.3204	27.0000	
**FN0904**									AAL95100.1| NAD(FAD)-utilizing dehydrogenases
					4		4.2641		
**FN0906**	0.573	9.839	1.33e-2	2.589e-3	15	26	21.9089	33.8420	AAL95102.1| Glycerol-3-phosphate dehydrogenase [NAD(P)+]
					26	40	27.7165	40.0000	
**FN0908**	-0.058	7.940				9		11.7145	AAL95104.1| Tpl protein
					15	19	15.9903	19.0000	
**FN0909**					7		10.2242		AAL95105.1| DNA repair protein radC
**FN0910**	-0.402	5.782				3		3.9048	AAL95106.1| Nicotinate-nucleotide--dimethylbenzimidazole phosphoribosyltransferase
					8	9	8.5282	9.0000	
**FN0911**	-0.506	6.018				5		6.5081	AAL95107.1| Alpha-ribazole-5'-phosphate phosphatase
					9	7	9.5942	7.0000	
**FN0912**	-1.114	7.566				9		11.7145	AAL95108.1| Cobalamin [5'-phosphate] synthase
					19	7	20.2544	7.0000	
**FN0913**	-0.414	6.414							AAL95109.1| Cobinamide kinase
					10	8	10.6602	8.0000	
**FN0915**	0.493	11.328	1.881e-1	2.829e-1	14	54	20.4483	70.2873	AAL95111.1| PTS system, N-acetylglucosamine-specific IIA component
					61	50	65.0272	50.0000	
**FN0916**	0.368	14.727	1.485e-1	1.858e-1	70	153	102.2417	199.1473	AAL95112.1| Hypothetical Exported Protein
					176	175	187.6194	175.0000	
**FN0917**	1.614	5.799				7		9.1113	AAL95113.1| Hypothetical protein
					4	17	4.2641	17.0000	
**FN0920**									AAL95116.1| Protease HTPX
					10		10.6602		

Log$_2$ Ratios: -6 -4 -2 0 2 4 6

Show detected proteins only
Show all proteins
Filter by category:
GO: amino acid transport

Proteins found: 1313

Enter (or paste) list of ORFs

Find ORFs

Test	Cutoff
q-Value	.005
p-Value	

	Signif	Direction	Applies To
red	yes	+	ratios, bars
yellow	no	n/a	bars
green	yes	−	ratios, bars
pink	yes	+	*p*- , *q*-Values
light green	yes	−	*p*- , *q*-Values

Dot Plots Dot Plots

Protein	FnSg vs FnPg Log$_2$ Ratio	Log$_2$ Sum	*q*-Value	*p*-Value	Raw *FnPg*	Raw *FnSg*	Normalized *FnPg*	Normalized *FnSg*	Description
FN0921	-0.604	10.565				27		35.1436	AAL95117.1| Hypothetical protein
					45	28	47.9709	28.0000	
FN0923									AAL95119.1| Cardiolipin synthetase
					4		4.2641		
FN0925	0.660	6.014				4		5.2065	AAL95121.1| Hypothetical protein
					6	15	6.3961	15.0000	
FN0926	-0.223	10.796	1e-3	2.979e-5	31	30	45.2785	39.0485	AAL95122.1| GTP pyrophosphokinase
					43	39	45.8388	39.0000	
FN0928	-0.423	6.406				3		3.9048	AAL95124.1| O-sialoglycoprotein endopeptidase
					10	12	10.6602	12.0000	
FN0929	0.453	9.091	2.564e-2	7.001e-3	12	22	17.5271	28.6356	AAL95125.1| ATP/GTP hydrolase
					21	26	22.3864	26.0000	
FN0930	-0.425	5.068			7		10.2242		AAL95126.1| Glycerol-3-phosphate cytidylyltransferase
					3	5	3.1981	5.0000	
FN0932	0.159	8.158				9		11.7145	AAL95128.1| Hypothetical protein
					15	24	15.9903	24.0000	
FN0934	0.517	9.486				30		39.0485	AAL95130.1| Chorismate synthase
					21	25	22.3864	25.0000	
FN0938	-0.128	11.165				42		54.6679	AAL95134.1| Hypothetical protein
					47	37	50.1029	37.0000	
FN0940	-1.050	9.850				14		18.2226	AAL95136.1| Hypothetical protein
					41	24	43.7068	24.0000	
FN0941	-0.610	11.697	5.384e-2	2.631e-2	42	44	61.3450	57.2711	AAL95137.1| Gamma-glutamyltranspeptidase
					76	36	81.0175	36.0000	
FN0943	-0.567	9.792				13		16.9210	AAL95139.1| Sensory Transduction Protein Kinase
					34	32	36.2447	32.0000	
FN0944									AAL95140.1| Na+ driven multidrug efflux pump
					4		4.2641		
FN0947	-0.858	12.017	1.148e-1	1.099e-1	80	32	116.8476	41.6517	AAL95143.1| Hypothetical protein
					53	54	56.4990	54.0000	
FN0949	-1.411	13.207	1.839e-1	2.72e-1	196	44	286.2767	57.2711	AAL95145.1| DNA helicase
					29	62	30.9146	62.0000	

Show detected proteins only
Show all proteins
Filter by category:
GO: amino acid transport

Proteins found: 1313

Enter (or paste) list of ORFs

Find ORFs

Test	Cutoff
q-Value	.005
p-Value	

	Signif	Direction	Applies To
red	yes	+	ratios, bars
yellow	no	n/a	bars
green	yes	−	ratios, bars
pink	yes	+	*p*- , *q*-Values
light green	yes	−	

Dot Plots Dot Plots

Protein	FnSg vs FnPg Log$_2$ Ratio	Log$_2$ Sum	*q*-Value	*p*-Value	Raw *FnPg*	Raw *FnSg*	Normalized *FnPg*	Normalized *FnSg*	Description
FN0951	-0.775	6.390			4		5.8424		AAL95147.1| Precorrin-3B C17-methyltransferase
					17	7	18.1223	7.0000	
FN0957	-1.418	7.945	3.972e-2	1.487e-2	22	4	32.1331	5.2065	AAL95153.1| Precorrin-4 C11-methyltransferase
					18	14	19.1883	14.0000	
FN0958	-2.061	7.676			20		29.2119		AAL95154.1| unknown
						7		7.0000	
FN0959	-1.481	6.125			14		20.4483		AAL95155.1| Precorrin-2 C20-methyltransferase
					7	5	7.4621	5.0000	
FN0961	0.896	8.505			6		8.7636		AAL95157.1| Hypothetical protein
					18	26	19.1883	26.0000	
FN0962	-1.359	9.016	5.476e-3	6.561e-4	28	8	40.8967	10.4129	AAL95158.1| Hypothetical cytosolic protein
					30	18	31.9806	18.0000	
FN0964	0.321	5.873	2.515e-2	6.763e-3	5	7	7.3030	9.1113	AAL95160.1| Precorrin-8W decarboxylase
					6	8	6.3961	8.0000	
FN0965	-0.112	12.001	3.106e-1	5.882e-1	51	57	74.4904	74.1921	AAL95161.1| D-3-phosphoglycerate dehydrogenase
					55	49	58.6311	49.0000	
FN0966	0.645	3.999							AAL95162.1| Precorrin-6Y C5,15-methyltransferase (decarboxylating)
					3	5	3.1981	5.0000	
FN0967	-0.769	5.939			7		10.2242		AAL95163.1| CbiD protein
						6		6.0000	
FN0970	-0.290	7.143			9	5	13.1454	6.5081	AAL95166.1| Precorrin-8X methylmutase
						15		15.0000	
FN0971						4		5.2065	AAL95167.1| hypothetical cytosolic protein
FN0972	0.608	8.205	2.417e-1	4.149e-1	3	8	4.3818	10.4129	AAL95168.1| Cobyrinic acid a,c-diamide synthase
					22	32	23.4524	32.0000	
FN0974									AAL95170.1| Lactoylglutathione lyase
					4		4.2641		
FN0976	0.144	11.383	2.891e-1	5.31e-1	33	32	48.1997	41.6517	AAL95172.1| Hypothetical protein
					47	67	50.1029	67.0000	
FN0977	1.438	9.116	2.056e-2	4.855e-3	5	35	7.3030	45.5566	AAL95173.1| Cobyric acid synthase
					20	32	21.3204	32.0000	

Log$_2$ Ratios: -6 -4 -2 0 2 4 6

Show detected proteins only
Show all proteins
Filter by category:
GO: amino acid transport

Proteins found: 1313

Enter (or paste) list of ORFs

Find ORFs

Test	Cutoff
q-Value	.005
p-Value	

	Signif	Direction	Applies To
red	yes	+	ratios, bars
yellow	no	n/a	bars
green	yes	−	ratios, bars
pink	yes	+	*p*- , *q*-Values
light green	yes	−	

Dot Plots Dot Plots

Protein	*FnSg* vs *FnPg* Log$_2$ Ratio	Log$_2$ Sum	*q*-Value	*p*-Value	Raw *FnPg*	Raw *FnSg*	Normalized *FnPg*	Normalized *FnSg*	Description	Log$_2$ Ratios (-6 -4 -2 0 2 4 6)
FN0981	-0.504	12.408	2.381e-1	4.061e-1	29	59	42.3573	76.7954	AAL95177.1| Phosphoribosylamine--glycine ligase	
					125	47	133.2524	47.0000		
FN0982	0.265	15.280	1.408e-1	1.651e-1	101	180	147.5201	234.2910	AAL95178.1| Phosphoribosylaminoimidazolecarboxamide formyltransferase	
					203	203	216.4019	203.0000		
FN0983	0.355	14.383	8.978e-2	6.911e-2	104	115	151.9019	149.6859	AAL95179.1| Hypothetical protein	
					100	181	106.6019	181.0000		
FN0984	0.873	11.030	5.226e-3	6.098e-4	20	49	29.2119	63.7792	AAL95180.1| Tetracenomycin polyketide synthesis O-methyltransferase tcmP	
					36	60	38.3767	60.0000		
FN0985	1.102	7.287				12		15.6194	AAL95181.1| Phosphoribosylglycinamide formyltransferase	
					8	21	8.5282	21.0000		
FN0986	0.231	15.265	3.259e-1	6.311e-1	64	119	93.4781	154.8924	AAL95182.1| Phosphoribosylformylglycinamidine cyclo-ligase	
					256	275	272.9009	275.0000		
FN0987	0.779	13.771	1.482e-1	1.851e-1	17	112	24.8301	145.7811	AAL95183.1| Amidophosphoribosyltransferase	
					146	164	155.6388	164.0000		
FN0988	0.060	15.807	3.888e-1	8.305e-1	116	156	169.4291	203.0522	AAL95184.1| Phosphoribosylamidoimidazole-succinocarboxamide synthase	
					281	286	299.5514	286.0000		
FN0989	0.050	15.288	3.92e-1	8.416e-1	121	116	176.7321	150.9875	AAL95185.1| Phosphoribosylaminoimidazole carboxylase catalytic subunit	
					203	256	216.4019	256.0000		
FN0990	0.834	20.908	7.738e-2	5.039e-2	411	1412	600.3047	1837.8826	AAL95186.1| Phosphoribosylformylglycinamidine synthase	
					1408	1907	1500.9551	1907.0000		
FN0991	-0.090	9.717	2.554e-1	4.478e-1	22	24	32.1331	31.2388	AAL95187.1| CDP-diacylglycerol--serine O-phosphatidyltransferase	
					26	25	27.7165	25.0000		
FN0992	0.892	9.216	6.184e-2	3.307e-2	7	28	10.2242	36.4453	AAL95188.1| ADP-heptose:LPS heptosyltransferase II	
					24	30	25.5845	30.0000		
FN0994	0.040	13.306	4.174e-1	9.355e-1	33	60	48.1997	78.0970	AAL95190.1| Hypothetical protein	
					141	126	150.3087	126.0000		
FN0997	0.649	11.507	1.465e-1	1.803e-1	13	60	18.9877	78.0970	AAL95193.1| Hypothetical protein	
					63	57	67.1592	57.0000		
FN0998	1.402	15.330	2.05e-2	4.831e-3	52	248	75.9510	322.8009	AAL95194.1| Dipeptide-binding protein	
					163	337	173.7611	337.0000		
FN0999	-0.636	11.318	2.471e-1	4.279e-1	14	30	20.4483	39.0485	AAL95195.1| Deblocking aminopeptidase	
					99	42	105.5359	42.0000		

Show detected proteins only
Show all proteins
Filter by category:
GO: amino acid transport

Proteins found: 1313

Enter (or paste) list of ORFs

Find ORFs

Test	Cutoff
q-Value	.005
p-Value	

	Signif	Direction	Applies To
red	yes	+	ratios, bars
yellow	no	n/a	bars
green	yes	−	ratios, bars
pink	yes	+	*p*-, *q*-Values
light green	yes	−	

Dot Plots Dot Plots

Protein	FnSg vs FnPg Log$_2$ Ratio	Log$_2$ Sum	q-Value	p-Value	Raw FnPg	Raw FnSg	Normalized FnPg	Normalized FnSg	Description
FN1000					16		23.3695		AAL95196.1| Biotin synthase
FN1001	0.163	10.734	3.475e-1	6.953e-1	14	31	20.4483	40.3501	AAL95197.1| Dethiobiotin synthetase
					54	47	57.5650	47.0000	
FN1002	-1.471	10.879	8.808e-2	6.632e-2	69	17	100.7811	22.1275	AAL95198.1| Adenosylmethionine-8-amino -7-oxononanoate aminotransferase
					41	30	43.7068	30.0000	
FN1003	-0.787	14.629	2.781e-1	5.028e-1	6	84	8.7636	109.3358	AAL95199.1| Outer membrane protein P1 precursor
					384	133	409.3514	133.0000	
FN1004	0.234	8.546	3.101e-1	5.867e-1	12	23	17.5271	29.9372	AAL95200.1| Transcriptional regulator, TetR family
					17	12	18.1223	12.0000	
FN1005	0.414	10.773				45		58.5727	AAL95201.1| Hypothetical protein
					34	38	36.2447	38.0000	
FN1006	-0.900	8.070							AAL95202.1| Acetyltransferase
					21	12	22.3864	12.0000	
FN1010	-0.769	14.158	1.418e-1	1.676e-1	73	90	106.6235	117.1455	AAL95206.1| Hypothetical cytosolic protein
					231	90	246.2505	90.0000	
FN1011	-0.006	7.141	4.221e-1	9.536e-1	9	9	13.1454	11.7145	AAL95207.1| MGPA protein
					10	12	10.6602	12.0000	
FN1012	0.290	10.301	1.471e-1	1.82e-1	25	25	36.5149	32.5404	AAL95208.1| HPR(Ser) kinase
					26	46	27.7165	46.0000	
FN1014	0.492	9.595				33		42.9533	AAL95210.1| Folylpolyglutamate synthase
					22	23	23.4524	23.0000	
FN1015	-1.341	8.560				8		10.4129	AAL95211.1| 5'-methylthioadenosine nucleosidase
					29	14	30.9146	14.0000	
FN1016	1.182	8.927	5.703e-2	2.886e-2	4	28	5.8424	36.4453	AAL95212.1| Lipid A biosynthesis lauroyl acyltransferase
					22	30	23.4524	30.0000	
FN1017	-0.078	12.632	3.919e-1	8.413e-1	34	70	49.6602	91.1132	AAL95213.1| Hypothetical Exported Protein
					107	64	114.0641	64.0000	
FN1019	-0.646	24.242	7.914e-3	1.238e-3	3572	2651	5217.2471	3450.5855	AAL95215.1| 3-hydroxybutyryl-CoA dehydrogenase
					5563	3671	5930.2652	3671.0000	
FN1020	-0.377	18.349	1.128e-1	1.064e-1	460	478	671.8739	622.1727	AAL95216.1| 3-hydroxybutyryl-CoA dehydratase
					605	392	644.9417	392.0000	

Log$_2$ Ratios: -6 -4 -2 0 2 4 6

Show detected proteins only
Show all proteins
Filter by category:
GO: amino acid transport

Proteins found: 1313

Enter (or paste) list of ORFs

Find ORFs

Test	Cutoff
q-Value	.005
p-Value	

Signif	Direction	Applies To
yes	+	ratios, bars
no	n/a	bars
yes	−	ratios, bars
yes	+	p- , q-Values
yes	−	

Dot Plots Dot Plots

Protein	*FnSg* vs *FnPg* Log$_2$ Ratio	Log$_2$ Sum	*q*-Value	*p*-Value	Raw *FnPg*	Raw *FnSg*	Normalized *FnPg*	Normalized *FnSg*	Description
**FN1023**	0.351	8.711				24		31.2388	AAL95219.1| 5-Nitroimidazole antibiotic resistance protein
					17	15	18.1223	15.0000	
**FN1024**	-0.080	20.340	3.402e-1	6.731e-1	934	726	1364.1962	944.9736	AAL95220.1| DNA-binding protein HU
					943	1296	1005.2562	1296.0000	
**FN1025**	-0.940	7.584							AAL95221.1| Guanine-hypoxanthine permease
					18	10	19.1883	10.0000	
**FN1026**									AAL95222.1| tRNA pseudouridine synthase A
						6		6.0000	
**FN1028**	-0.206	9.581	2.31e-1	3.874e-1	21	25	30.6725	32.5404	AAL95224.1| Deoxyuridine 5'-triphosphate nucleotidohydrolase
					27	19	28.7825	19.0000	
**FN1029**	-0.699	12.026	5.539e-4	7.104e-6	55	41	80.3328	53.3663	AAL95225.1| Zinc protease
					79	48	84.2155	48.0000	
**FN1030**	-0.312	9.043				14		18.2226	AAL95226.1| Hypothetical membrane-spanning protein
					24	23	25.5845	23.0000	
**FN1031**	-0.218	7.137							AAL95227.1| Hypothetical membrane-spanning protein
					12	11	12.7922	11.0000	
**FN1033**	-0.270	11.800	1.251e-1	1.298e-1	46	49	67.1874	63.7792	AAL95229.1| Methyltransferase
					60	45	63.9612	45.0000	
**FN1034**						5		6.5081	AAL95230.1| Transcriptional regulator, TetR family
						13		13.0000	
**FN1035**									AAL95231.1| Iron-sulfur flavoprotein
						3		3.0000	
**FN1036**						3		3.9048	AAL95232.1| Hypothetical protein
						4		4.0000	
**FN1037**	0.102	8.100				11		14.3178	AAL95233.1| Hypothetical cytosolic protein
					15	20	15.9903	20.0000	
**FN1041**	0.452	7.665	1.624e-1	2.198e-1	5	11	7.3030	14.3178	AAL95237.1| Acetyltransferase
					16	19	17.0563	19.0000	
**FN1042**	0.070	8.271	3.84e-1	8.14e-1	14	10	20.4483	13.0162	AAL95238.1| S1 RNA binding domain
					13	23	13.8583	23.0000	
**FN1048**						3		3.9048	AAL95244.1| Hypothetical membrane-spanning protein

Log$_2$ Ratios: -6 -4 -2 0 2 4 6

Show detected proteins only
Show all proteins
Filter by category:
GO: amino acid transport

Proteins found: 1313

Enter (or paste) list of ORFs

Find ORFs

Test	Cutoff
q-Value	.005
p-Value	

	Signif	Direction	Applies To
red	yes	+	ratios, bars
yellow	no	n/a	bars
green	yes	−	ratios, bars
pink	yes	+	*p*- , *q*-Values
light green	yes	−	

Dot Plots Dot Plots

Protein	FnSg vs FnPg: Log_2 Ratio	Log_2 Sum	*q*-Value	*p*-Value	Raw: *FnPg*	Raw: *FnSg*	Normalized: *FnPg*	Normalized: *FnSg*	Description
**FN1050**					22		32.1331		AAL95246.1| Lactoylglutathione lyase
					3		3.1981		
**FN1053**					8		11.6848		AAL95249.1| Hypothetical protein
**FN1055**	0.082	12.254	3.757e-1	7.855e-1	31	59	45.2785	76.7954	AAL95251.1| Cysteine synthase
					85	67	90.6116	67.0000	
**FN1057**						17		22.1275	AAL95253.1| Diamine acetyltransferase
						5		5.0000	
**FN1060**	0.353	10.498	1.031e-2	1.79e-3	22	33	32.1331	42.9533	AAL95256.1| hypothetical cytosolic protein
					33	43	35.1786	43.0000	
**FN1062**	-0.849	11.407	7.556e-2	4.801e-2	60	22	87.6357	28.6356	AAL95258.1| Hydrolase
					49	49	52.2349	49.0000	
**FN1063**	0.090	9.667	4.069e-1	8.958e-1	5	16	7.3030	20.8259	AAL95259.1| N-acyl-L-amino acid amidohydrolase
					45	38	47.9709	38.0000	
**FN1066**	0.919	7.358	5.128e-2	2.383e-2	4	14	5.8424	18.2226	AAL95262.1| Exodeoxyribonuclease VII large subunit
					12	17	12.7922	17.0000	
**FN1067**	1.468	11.077	6.324e-3	8.334e-4	12	65	17.5271	84.6051	AAL95263.1| Tetratricopeptide repeat family protein
					36	70	38.3767	70.0000	
**FN1068**	0.658	7.502	1.579e-1	2.093e-1	3	16	4.3818	20.8259	AAL95264.1| Smf protein
					16	13	17.0563	13.0000	
**FN1069**	-0.636	11.338	7.169e-2	4.331e-2	46	22	67.1874	28.6356	AAL95265.1| DNA topoisomerase I
					56	53	59.6971	53.0000	
**FN1070**	-0.052	6.472				5		6.5081	AAL95266.1| Glucose inhibited division protein A
					9	12	9.5942	12.0000	
**FN1071**									AAL95267.1| Integrase/recombinase
						4		4.0000	
**FN1072**	-0.766	9.973	7.671e-2	4.95e-2	34	12	49.6602	15.6194	AAL95268.1| GTP-binding protein
					31	33	33.0466	33.0000	
**FN1073**	0.556	5.911				6		7.8097	AAL95269.1| Hypothetical protein
					6	11	6.3961	11.0000	
**FN1074**	0.373	9.655	1.244e-1	1.284e-1	13	22	18.9877	28.6356	AAL95270.1| Signal recognition particle receptor FtsY
					29	36	30.9146	36.0000	

Log_2 Ratios: -6 -4 -2 0 2 4 6

Show detected proteins only
Show all proteins
Filter by category: GO: amino acid transport

Proteins found: 1313

Enter (or paste) list of ORFs

Find ORFs

Test	Cutoff
q-Value	.005
p-Value	

	Signif	Direction	Applies To
red	yes	+	ratios, bars
yellow	no	n/a	bars
green	yes	–	ratios, bars
pink	yes	+	*p*- , *q*-Values
light green	yes	–	

Dot Plots Dot Plots

Protein	*FnSg* vs *FnPg* Log$_2$ Ratio	Log$_2$ Sum	*q*-Value	*p*-Value	Raw *FnPg*	Raw *FnSg*	Normalized *FnPg*	Normalized *FnSg*	Description	Log$_2$ Ratios (-6 -4 -2 0 2 4 6)
**FN1075**									AAL95271.1| Hypothetical protein	
						7		7.0000		
**FN1077**	-1.808	8.636				11		14.3178	AAL95273.1| Hypothetical protein	
					35	7	37.3107	7.0000		
**FN1078**	-0.405	15.509	8.587e-2	6.27e-2	195	144	284.8161	187.4328	AAL95274.1| Hypothetical exported 24-amino acid repeat protein	
					199	188	212.1378	188.0000		
**FN1079**	0.447	19.158	1.624e-1	2.199e-1	267	748	389.9790	973.6092	AAL95275.1| Neutrophil-activating protein A	
					863	812	919.9746	812.0000		
**FN1080**	1.682	5.526	2.047e-2	4.82e-3	3	11	4.3818	14.3178	AAL95276.1| Export ABC transporter	
					3	10	3.1981	10.0000		
**FN1081**					25		36.5149		AAL95277.1| unknown	
**FN1084**	-0.983	9.267	8.285e-2	5.811e-2	31	11	45.2785	14.3178	AAL95280.1| unknown	
					23	21	24.5184	21.0000		
**FN1085**	-0.378	13.731	5.469e-2	2.697e-2	101	85	147.5201	110.6374	AAL95281.1| 4-methyl-5(B-hydroxyethyl)-thiazole monophosphate biosynthesis enzyme	
					111	94	118.3281	94.0000		
**FN1086**	1.459	7.258				20		26.0323	AAL95282.1| Transporter	
					7	15	7.4621	15.0000		
**FN1088**	0.677	9.744	4.406e-2	1.794e-2	12	30	17.5271	39.0485	AAL95284.1| NADH oxidase	
					27	35	28.7825	35.0000		
**FN1089**	0.747	15.175	5.694e-2	2.879e-2	69	164	100.7811	213.4651	AAL95285.1| ATP-binding protein (contains P-loop)	
					184	285	196.1475	285.0000		
**FN1091**	0.944	6.713	7.9e-2	5.261e-2	5	8	7.3030	10.4129	AAL95287.1| Sigma factor sigB regulation protein rsbU	
					7	18	7.4621	18.0000		
**FN1092**	0.711	11.981	1.841e-1	2.727e-1	6	62	8.7636	80.7002	AAL95288.1| Hypothetical protein	
					85	82	90.6116	82.0000		
**FN1093**	-0.487	9.861	1.025e-1	8.896e-2	29	15	42.3573	19.5242	AAL95289.1| Hypothetical protein	
					28	32	29.8485	32.0000		
**FN1094**	-0.782	9.491				13		16.9210	AAL95290.1| Dolichol-phosphate mannosyltransferase	
					33	24	35.1786	24.0000		
**FN1095**					8		11.6848		AAL95291.1| unknown	

Show detected proteins only
Show all proteins
Filter by category:
GO: amino acid transport

Proteins found: 1313

Enter (or paste) list of ORFs

Find ORFs

Test	Cutoff
q-Value	.005
p-Value	

	Signif	Direction	Applies To
red	yes	+	ratios, bars
yellow	no	n/a	bars
green	yes	−	ratios, bars
pink	yes	+	*p*- , *q*-Values
light green	yes	−	

Dot Plots | Dot Plots

Protein	*FnSg* vs *FnPg* Log$_2$ Ratio	Log$_2$ Sum	*q*-Value	*p*-Value	Raw *FnPg*	Raw *FnSg*	Normalized *FnPg*	Normalized *FnSg*	Description
FN1096	-0.741	10.242	1.873e-1	2.807e-1	47	16	68.6480	20.8259	AAL95292.1| Hypothetical protein
					20	33	21.3204	33.0000	
FN1097	-2.247	8.247			26		37.9755		AAL95293.1| Hypothetical protein
						8		8.0000	
FN1101									AAL95297.1| ATPase
						8		8.0000	
FN1102									AAL95298.1| tRNA 2'phosphotransferase
						10		10.0000	
FN1103	0.896	11.103	2.65e-2	7.402e-3	23	43	33.5937	55.9695	AAL95299.1| Excinuclease ABC subunit A
					33	72	35.1786	72.0000	
FN1105	-0.962	11.985	1.112e-1	1.035e-1	83	34	121.2294	44.2550	AAL95301.1| Hypothetical protein
					53	47	56.4990	47.0000	
FN1106	-0.118	12.041	2.593e-1	4.571e-1	51	55	74.4904	71.5889	AAL95302.1| L-serine dehydratase
					57	53	60.7631	53.0000	
FN1111						11		14.3178	AAL95307.1| Dipeptide-binding protein
						17		17.0000	
FN1117	-1.259	9.243	1.266e-1	1.329e-1	39	16	56.9632	20.8259	AAL95313.1| LSU ribosomal protein L21P
					18	11	19.1883	11.0000	
FN1119	-1.638	11.491	1.031e-1	8.995e-2	96	16	140.2172	20.8259	AAL95315.1| LSU ribosomal protein L27P
					46	40	49.0369	40.0000	
FN1120	-0.049	16.232	3.515e-1	7.078e-1	166	207	242.4589	269.4346	AAL95316.1| Phosphoenolpyruvate carboxykinase (ATP)
					302	276	321.9378	276.0000	
FN1121	0.209	12.124	1.857e-1	2.765e-1	34	52	49.6602	67.6841	AAL95317.1| hypothetical cytosolic protein
					70	76	74.6213	76.0000	
FN1122	0.184	12.114	2.358e-1	3.999e-1	41	66	59.8844	85.9067	AAL95318.1| Long-chain-fatty-acid--CoA ligase
					61	56	65.0272	56.0000	
FN1123	0.496	6.048	2.256e-1	3.737e-1	5	11	7.3030	14.3178	AAL95319.1| Thioredoxin-like protein
					6	5	6.3961	5.0000	
FN1124	0.763	16.378	1.047e-2	1.829e-3	133	286	194.2592	372.2623	AAL95320.1| Outer membrane porin F
					238	388	253.7126	388.0000	
FN1125	0.357	12.772	2.829e-1	5.15e-1	18	84	26.2907	109.3358	AAL95321.1| LemA protein
					114	80	121.5262	80.0000	

Log$_2$ Ratios: -6 -4 -2 0 2 4 6

Show detected proteins only
Show all proteins
Filter by category:
GO: amino acid transport

Proteins found: 1313

Enter (or paste) list of ORFs

Find ORFs

Test	Cutoff
q-Value	.005
p-Value	

Signif	Direction	Applies To
yes	+	ratios, bars
no	n/a	bars
yes	−	ratios, bars
yes	+	*p*-, *q*-Values
yes	−	

Dot Plots Dot Plots

Protein	*FnSg* vs *FnPg* Log$_2$ Ratio	Log$_2$ Sum	*q*-Value	*p*-Value	Raw *FnPg*	Raw *FnSg*	Normalized *FnPg*	Normalized *FnSg*	Description
FN1127	-0.497	12.635	1.423e-1	1.688e-1	83	54	121.2294	70.2873	AAL95323.1| Hypothetical membrane-spanning protein
					64	64	68.2252	64.0000	
FN1128	-0.734	15.860	9.162e-2	7.223e-2	270	140	394.3608	182.2263	AAL95324.1| Acylamino-acid-releasing enzyme
					220	196	234.5242	196.0000	
FN1129	0.341	5.696				4		5.2065	AAL95325.1| Chromosome partition protein smc
					6	11	6.3961	11.0000	
FN1130	1.045	5.874							AAL95326.1| Tetraacyldisaccharide 4'-kinase
					5	11	5.3301	11.0000	
FN1131	-0.985	6.600							AAL95327.1| Hypothetical protein
					13	7	13.8583	7.0000	
FN1133	-0.495	10.411	2.427e-1	4.173e-1	14	27	20.4483	35.1436	AAL95329.1| N-acetylglucosamine-6-phosphate deacetylase
					63	27	67.1592	27.0000	
FN1134	-1.864	7.864			26		37.9755		AAL95330.1| Hypothetical cytosolic protein
					19	8	20.2544	8.0000	
FN1135	-0.761	14.742	1.829e-1	2.695e-1	225	107	328.6340	139.2730	AAL95331.1| Phosphonates-binding protein
					96	115	102.3379	115.0000	
FN1136	0.062	9.417				18		23.4291	AAL95332.1| Phosphonates transport ATP-binding protein phnC
					24	30	25.5845	30.0000	
FN1138	-0.414	18.374	1.623e-1	2.196e-1	349	491	509.7478	639.0937	AAL95334.1| Hypothetical cytosolic protein
					784	371	835.7591	371.0000	
FN1139	0.924	13.686	1.044e-1	9.191e-2	20	107	29.2119	139.2730	AAL95335.1| Activator of (R)-2-hydroxyglutaryl-CoA dehydratase
					129	177	137.5165	177.0000	
FN1140	-0.276	9.196				10		13.0162	AAL95336.1| hypothetical protein
					25	31	26.6505	31.0000	
FN1142	0.309	5.137				4		5.2065	AAL95338.1| Oxygen-independent coproporphyrinogen III oxidase
					5	8	5.3301	8.0000	
FN1143	1.064	14.967	6.498e-2	3.608e-2	41	244	59.8844	317.5944	AAL95339.1| Glucosamine-6-phosphate isomerase
					176	200	187.6194	200.0000	
FN1144	1.001	13.002	6.259e-2	3.377e-2	22	107	32.1331	139.2730	AAL95340.1| Hypothetical Exported Protein
					90	117	95.9417	117.0000	
FN1145	-0.101	8.796	3.956e-1	8.544e-1	8	9	11.6848	11.7145	AAL95341.1| Oligoendopeptidase F
					30	29	31.9806	29.0000	

Log$_2$ Ratios: -6 -4 -2 0 2 4 6

Show detected proteins only
Show all proteins
Filter by category:
GO: amino acid transport

Proteins found: 1313

Enter (or paste) list of ORFs

Find ORFs

Test	Cutoff
q-Value	.005
p-Value	

Signif	Direction	Applies To
yes	+	ratios, bars
no	n/a	bars
yes	–	ratios, bars
yes	+	*p*- , *q*-Values
yes	–	

Dot Plots Dot Plots

Protein	FnSg vs FnPg Log_2 Ratio	Log_2 Sum	*q*-Value	*p*-Value	Raw *FnPg*	Raw *FnSg*	Normalized *FnPg*	Normalized *FnSg*	Description
FN1146					6		8.7636		AAL95342.1| Hypothetical exported 24-amino acid repeat protein
FN1148	0.111	11.539	4.044e-1	8.864e-1	4	41	5.8424	53.3663	AAL95344.1| Serine/threonine sodium symporter
					93	60	99.1398	60.0000	
FN1149	0.693	7.151	2.336e-3	1.566e-4	7	11	10.2242	14.3178	AAL95345.1| ATP-dependent nuclease subunit A
					8	16	8.5282	16.0000	
FN1150	0.715	4.900							AAL95346.1| unknown
					4	7	4.2641	7.0000	
FN1152	-0.055	13.383	4.011e-1	8.743e-1	45	79	65.7268	102.8277	AAL95348.1| Aspartate aminotransferase
					136	100	144.9786	100.0000	
FN1154	-1.063	7.766				8		10.4129	AAL95350.1| Ribonuclease BN
					20	10	21.3204	10.0000	
FN1155						3		3.9048	AAL95351.1| Cell division protein ftsI
						5		5.0000	
FN1156						10		13.0162	AAL95352.1| Primosomal protein N'
						5		5.0000	
FN1159	0.294	14.595	1.371e-1	1.56e-1	77	137	112.4659	178.3215	AAL95355.1| Fructose-1,6-bisphosphatase
					161	170	171.6291	170.0000	
FN1160	1.333	4.687				7		9.1113	AAL95356.1| SWF/SNF family helicase
					3	7	3.1981	7.0000	
FN1161	1.278	4.632				5		6.5081	AAL95357.1| Glutamate racemase
					3	9	3.1981	9.0000	
FN1162	-1.558	7.411				4		5.2065	AAL95358.1| Hydroxyacylglutathione hydrolase
					21	10	22.3864	10.0000	
FN1163	-0.514	11.650	1.768e-1	2.547e-1	30	46	43.8179	59.8744	AAL95359.1| Thioredoxin reductase
					86	35	91.6777	35.0000	
FN1164	0.278	7.046	1.106e-1	1.026e-1	7	11	10.2242	14.3178	AAL95360.1| Glucokinase
					10	11	10.6602	11.0000	
FN1165	0.612	23.817	5.094e-2	2.352e-2	2224	4182	3248.3644	5443.3605	AAL95361.1| D-galactose-binding protein
					2786	4062	2969.9297	4062.0000	
FN1166	0.377	15.375	1.558e-3	7.615e-5	128	178	186.9562	231.6877	AAL95362.1| Galactoside transport ATP-binding protein mglA
					164	238	174.8272	238.0000	

Log_2 Ratios: -6 -4 -2 0 2 4 6

Show detected proteins only
Show all proteins
Filter by category:
GO: amino acid transport

Proteins found: 1313

Enter (or paste) list of ORFs

Find ORFs

Test	Cutoff
q-Value	.005
p-Value	

	Signif	Direction	Applies To
red	yes	+	ratios, bars
yellow	no	n/a	bars
green	yes	−	ratios, bars
pink	yes	+	*p*-, *q*-Values
light green	yes	−	

Dot Plots Dot Plots

Protein	FnSg vs FnPg Log2 Ratio	Log2 Sum	q-Value	p-Value	Raw FnPg	Raw FnSg	Normalized FnPg	Normalized FnSg	Description	Log2 Ratios
FN1167						8		10.4129	AAL95363.1| Galactoside transport system permease protein mglC	
						13		13.0000		
FN1169	0.278	16.921	1.73e-1	2.456e-1	260	245	379.7548	318.8961	AAL95365.1| L-lactate dehydrogenase	
					244	457	260.1087	457.0000		
FN1170	-0.142	25.777	3.064e-1	5.768e-1	6878	5624	10045.975	7320.2915	AAL95366.1| Pyruvate-flavodoxin oxidoreductase	
					5525	7117	5889.7565	7117.0000		
FN1171	-0.222	20.052	2.133e-1	3.435e-1	936	673	1367.1174	875.9879	AAL95367.1| Acetate kinase	
					830	1055	884.7960	1055.0000		
FN1172	0.030	18.163	3.338e-1	6.54e-1	348	397	508.2872	516.7418	AAL95368.1| Phosphate acetyltransferase	
					529	578	563.9242	578.0000		
FN1176					4		5.8424		AAL95372.1| Hypothetical cytosolic protein	
FN1179	-1.769	6.939			14		20.4483		AAL95375.1| ATP-dependent RNA helicase	
						6		6.0000		
FN1180	-1.061	6.676			10		14.6060		AAL95376.1| Hypothetical protein	
						7		7.0000		
FN1181	-2.344	14.291	1.54e-1	2.001e-1	404	42	590.0806	54.6679	AAL95377.1| unknown	
					45	71	47.9709	71.0000		
FN1182	-1.284	6.453			10		14.6060		AAL95378.1| Hypothetical protein	
						6		6.0000		
FN1183	-0.176	7.933	3.097e-1	5.857e-1	14	8	20.4483	10.4129	AAL95379.1| Hypothetical cytosolic protein	
					12	19	12.7922	19.0000		
FN1184					4		5.8424		AAL95380.1| Hypothetical protein	
FN1185	-0.689	10.673	1.81e-1	2.649e-1	52	22	75.9510	28.6356	AAL95381.1| SIR2 family protein	
					25	35	26.6505	35.0000		
FN1186	0.240	17.171	3.387e-1	6.688e-1	82	308	119.7688	400.8979	AAL95382.1| Amidohydrolase	
					551	434	587.3766	434.0000		
FN1187	0.650	12.196	2.121e-1	3.408e-1	7	85	10.2242	110.6374	AAL95383.1| Amino acid-binding protein	
					93	61	99.1398	61.0000		
FN1188	1.162	12.256	7.563e-2	4.809e-2	10	87	14.6060	113.2406	AAL95384.1| Hypothetical protein	
					74	96	78.8854	96.0000		

Log2 Ratios axis: -6 -4 -2 0 2 4 6

Protein	*FnSg* vs *FnPg* Log$_2$ Ratio	Log$_2$ Sum	*q*-Value	*p*-Value	Raw *FnPg*	Raw *FnSg*	Normalized *FnPg*	Normalized *FnSg*	Description	Log$_2$ Ratios -6 -4 -2 0 2 4 6
**FN1189**	-0.118	12.067	2.978e-1	5.538e-1	46	59	67.1874	76.7954	AAL95385.1| Hypothetical protein	
					65	49	69.2913	49.0000		
**FN1190**	1.038	13.812	8.211e-5	4.721e-7	54	135	78.8722	175.7182	AAL95386.1| Probable cadmium-transporting ATPase	
					83	168	88.4796	168.0000		
**FN1191**	1.187	12.355				104		135.3681	AAL95387.1| unknown	
					45	83	47.9709	83.0000		
**FN1192**	0.791	18.046	2.599e-2	7.173e-3	227	451	331.5552	587.0291	AAL95388.1| unknown	
					431	782	459.4543	782.0000		
**FN1198**	0.898	11.084	1.113e-2	2.001e-3	19	47	27.7513	61.1760	AAL95394.1| Transporter	
					38	66	40.5087	66.0000		
**FN1200**	-0.742	17.108				253		329.3090	AAL95396.1| Hypothetical protein	
					456	252	486.1048	252.0000		
**FN1201**									AAL95397.1| unknown	
						7		7.0000		
**FN1202**	-0.197	9.533	2.819e-1	5.125e-1	18	26	26.2907	33.8420	AAL95398.1| NH(3)-dependent NAD(+) synthetase	
					30	17	31.9806	17.0000		
**FN1203**	0.305	6.482	2.465e-1	4.265e-1	8	10	11.6848	13.0162	AAL95399.1| GTP-binding protein	
					5	8	5.3301	8.0000		
**FN1204**	0.124	7.786	2.531e-1	4.425e-1	10	10	14.6060	13.0162	AAL95400.1| Methyltransferase	
					13	18	13.8583	18.0000		
**FN1205**	-0.078	11.654	4.044e-1	8.864e-1	20	58	29.2119	75.4938	AAL95401.1| Protease	
					82	35	87.4136	35.0000		
**FN1208**	0.975	7.199			6		8.7636		AAL95404.1| 1-deoxyxylulose-5-phosphate synthase	
					8	17	8.5282	17.0000		
**FN1209**	0.487	8.485				16		20.8259	AAL95405.1| Hypothetical RNA binding protein	
					15	24	15.9903	24.0000		
**FN1210**	0.467	10.567	1.481e-1	1.847e-1	22	45	32.1331	58.5727	AAL95406.1| Metal dependent hydrolase	
					32	33	34.1126	33.0000		
**FN1211**	1.001	6.553	6.418e-2	3.53e-2	5	8	7.3030	10.4129	AAL95407.1| Cell division protein ftsI	
					6	17	6.3961	17.0000		
**FN1213**	0.842	11.063	8.008e-2	5.416e-2	13	46	18.9877	59.8744	AAL95409.1| Hypothetical protein	
					47	64	50.1029	64.0000		

Show detected proteins only
Show all proteins
Filter by category:
GO: amino acid transport

Proteins found: 1313

Enter (or paste) list of ORFs

Find ORFs

Test	Cutoff
q-Value	.005
p-Value	

	Signif	Direction	Applies To
red	yes	+	ratios, bars
yellow	no	n/a	bars
green	yes	−	ratios, bars
pink	yes	+	*p*- , *q*-Values
light green	yes	−	

Dot Plots Dot Plots

Protein	FnSg vs FnPg Log$_2$ Ratio	Log$_2$ Sum	q-Value	p-Value	Raw FnPg	Raw FnSg	Normalized FnPg	Normalized FnSg	Description
FN1216	0.487	7.681	7.205e-2	4.373e-2	10	13	14.6060	16.9210	AAL95412.1| RRF2 family protein
					9	17	9.5942	17.0000	
FN1217	1.625	7.424				20		26.0323	AAL95413.1| Holliday junction DNA helicase ruvB
					7	20	7.4621	20.0000	
FN1218	0.374	7.959				13		16.9210	AAL95414.1| unknown
					13	19	13.8583	19.0000	
FN1219	-0.275	8.213	1.35e-1	1.511e-1	15	11	21.9089	14.3178	AAL95415.1| Hypothetical protein
					15	17	15.9903	17.0000	
FN1220	0.289	15.621	1.709e-1	2.404e-1	127	150	185.4956	195.2425	AAL95416.1| Cysteine synthase
					207	301	220.6660	301.0000	
FN1221	0.497	9.241	2.284e-1	3.808e-1	5	28	7.3030	36.4453	AAL95417.1| Hypothetical protein
					32	22	34.1126	22.0000	
FN1222	-1.562	6.206			10		14.6060		AAL95418.1| Hypothetical protein
					14	5	14.9243	5.0000	
FN1223	-1.625	11.368	2.843e-2	8.322e-3	74	25	108.0841	32.5404	AAL95419.1| Oxygen-insensitive NAD(P)H nitroreductase
					68	26	72.4893	26.0000	
FN1224	-0.398	13.009	2.364e-1	4.016e-1	42	47	61.3450	61.1760	AAL95420.1| 2-dehydro-3-deoxyphosphooctonate aldolase
					138	97	147.1107	97.0000	
FN1225	-0.187	13.251	3.249e-1	6.283e-1	45	60	65.7268	78.0970	AAL95421.1| UDP-N-acetylmuramoyl-L-alanyl-D-glutamate--meso-lanthionine ligase
					136	107	144.9786	107.0000	
FN1226	1.490	13.992	2.328e-2	5.925e-3	24	195	35.0543	253.8152	AAL95422.1| Uracil-DNA glycosylase
					110	174	117.2621	174.0000	
FN1231	0.471	19.642	2.362e-1	4.012e-1	166	788	242.4589	1025.6739	AAL95427.1| Inosine-5'-monophosphate dehydrogenase
					1214	1104	1294.1474	1104.0000	
FN1233									AAL95429.1| Putative NAD(P)H oxidoreductase
						6		6.0000	
FN1234									AAL95430.1| Hypothetical protein
						21		21.0000	
FN1235	0.365	9.531	2.504e-1	4.362e-1	8	29	11.6848	37.7469	AAL95431.1| Ankyrin repeat proteins
					34	24	36.2447	24.0000	
FN1237	0.712	13.428	6.986e-3	9.988e-4	51	112	74.4904	145.7811	AAL95433.1| Choline kinase
					84	123	89.5456	123.0000	

Log$_2$ Ratios: -6 -4 -2 0 2 4 6

Show detected proteins only
Show all proteins
Filter by category:
GO: amino acid transport

Proteins found: 1313

Enter (or paste) list of ORFs

Find ORFs

Test	Cutoff
q-Value	.005
p-Value	

Signif	Direction	Applies To
yes	+	ratios, bars
no	n/a	bars
yes	–	ratios, bars
yes	+	p- , q-Values
yes	–	

Dot Plots Dot Plots

Protein	FnSg vs FnPg Log$_2$ Ratio	Log$_2$ Sum	q-Value	p-Value	Raw FnPg	Raw FnSg	Normalized FnPg	Normalized FnSg	Description
FN1238	-0.414	4.414							AAL95434.1| Hypothetical protein
					5	4	5.3301	4.0000	
FN1239									AAL95435.1| Hypothetical protein
					12		12.7922		
FN1240	-0.769	6.344	1.319e-1	1.439e-1	11	6	16.0666	7.8097	AAL95436.1| Lipopolysaccharide core biosynthesis protein rfaY
					7	6	7.4621	6.0000	
FN1241	0.629	6.343	1.099e-1	1.014e-1	7	8	10.2242	10.4129	AAL95437.1| polysaccharide biosynthesis protein
					4	12	4.2641	12.0000	
FN1242	0.448	8.972				21		27.3339	AAL95438.1| Polysaccharide deacetylase
					18	25	19.1883	25.0000	
FN1243	-0.255	7.544				10		13.0162	AAL95439.1| Glycosyl transferase
					14	12	14.9243	12.0000	
FN1244	0.185	9.278	3.677e-1	7.594e-1	5	17	7.3030	22.1275	AAL95440.1| Polysaccharide deacetylase
					37	31	39.4427	31.0000	
FN1245	0.609	5.168	4.202e-3	4.429e-4	3	6	4.3818	7.8097	AAL95441.1| Glycosyl transferase
					5	7	5.3301	7.0000	
FN1246	0.231	8.229				15		19.5242	AAL95442.1| Lipooligosaccharide cholinephosphotransferase
					15	18	15.9903	18.0000	
FN1247	0.968	9.238	7.704e-2	4.994e-2	8	19	11.6848	24.7307	AAL95443.1| LOS biosynthesis enzyme LBGB
					22	44	23.4524	44.0000	
FN1250	0.040	8.774	4.033e-1	8.823e-1	10	18	14.6060	23.4291	AAL95446.1| Guanine-hypoxanthine permease
					25	19	26.6505	19.0000	
FN1251	0.555	6.354				13		16.9210	AAL95447.1| High-affinity iron permease
					7	5	7.4621	5.0000	
FN1252	0.786	16.487	5.381e-2	2.628e-2	151	361	220.5499	469.8836	AAL95448.1| 34 kDa membrane antigen precursor
					226	326	240.9204	326.0000	
FN1253	0.102	15.514	3.977e-1	8.62e-1	48	186	70.1086	242.1007	AAL95449.1| unknown
					326	206	347.5223	206.0000	
FN1254	-0.219	12.056	3.057e-1	5.75e-1	30	53	43.8179	68.9857	AAL95450.1| Oxygen-insensitive NAD(P)H nitroreductase
					91	52	97.0078	52.0000	
FN1256									AAL95452.1| C4-dicarboxylate transporter large subunit
					4		4.2641		

Log$_2$ Ratios: -6 -4 -2 0 2 4 6

Show detected proteins only
Show all proteins
Filter by category:
GO: amino acid transport

Proteins found: 1313

Enter (or paste) list of ORFs

Find ORFs

Test	Cutoff
q-Value	.005
p-Value	

Signif	Direction	Applies To
yes	+	ratios, bars
no	n/a	bars
yes	–	ratios, bars
yes	+	p- , q-Values
yes	–	

Dot Plots Dot Plots

Protein	*FnSg* vs *FnPg* Log$_2$ Ratio	Log$_2$ Sum	*q*-Value	*p*-Value	Raw *FnPg*	Raw *FnSg*	Normalized *FnPg*	Normalized *FnSg*	Description
FN1258	0.230	19.411	1.1e-1	1.015e-1	595	741	869.0543	964.4979	AAL95454.1| C4-dicarboxylate-binding protein
					631	844	672.6582	844.0000	
FN1259									AAL95455.1| hypothetical protein
						9		9.0000	
FN1261					10		14.6060		AAL95457.1| Two-component response regulator
FN1262	-0.313	8.516				21		27.3339	AAL95458.1| Integral membrane protein
					20	7	21.3204	7.0000	
FN1263	0.775	8.255	1.612e-2	3.402e-3	11	19	16.0666	24.7307	AAL95459.1| Cobalt chelatase
					10	21	10.6602	21.0000	
FN1264						12		15.6194	AAL95460.1| Hypothetical protein
						17		17.0000	
FN1265	-0.241	13.109	1.249e-1	1.294e-1	64	76	93.4781	98.9229	AAL95461.1| Outer membrane protein
					104	74	110.8660	74.0000	
FN1266	0.026	13.574	4.062e-1	8.932e-1	63	99	92.0175	128.8600	AAL95462.1| UTP--glucose-1-phosphate uridylyltransferase
					119	94	126.8563	94.0000	
FN1267	-0.455	10.572	1.202e-1	1.199e-1	37	32	54.0420	41.6517	AAL95463.1| Hypothetical protein
					35	25	37.3107	25.0000	
FN1268	-0.563	15.004	6.929e-4	1.394e-5	155	120	226.3923	156.1940	AAL95464.1| Methionyl-tRNA synthetase
					201	142	214.2699	142.0000	
FN1269	1.095	5.280				7		9.1113	AAL95465.1| Hypothetical lipoprotein
					4		4.2641		
FN1270	-0.934	8.035				9		11.7145	AAL95466.1| Hypothetical cytosolic protein
					21		22.3864		
FN1271	0.718	10.732	1.407e-1	1.649e-1	9	49	13.1454	63.7792	AAL95467.1| Protease IV
					48	42	51.1689	42.0000	
FN1273	0.357	11.696	3.223e-1	6.208e-1	4	51	5.8424	66.3824	AAL95469.1| Outer membrane protein tolC
					90	64	95.9417	64.0000	
FN1274	0.927	8.045	1.006e-1	8.603e-2	3	16	4.3818	20.8259	AAL95470.1| Acriflavin resistance protein E
					18	24	19.1883	24.0000	
FN1275	0.393	8.448	1.715e-1	2.418e-1	7	16	10.2242	20.8259	AAL95471.1| Acriflavin resistance protein B
					21	22	22.3864	22.0000	

Log$_2$ Ratios: -6 -4 -2 0 2 4 6

Show detected proteins only
Show all proteins
Filter by category:
GO: amino acid transport

Proteins found: 1313

Enter (or paste) list of ORFs

Find ORFs

Test	Cutoff
q-Value	.005
p-Value	

	Signif	Direction	Applies To
red	yes	+	ratios, bars
yellow	no	n/a	bars
green	yes	−	ratios, bars
pink	yes	+	*p*- , *q*-Values
light green	yes	−	

Dot Plots Dot Plots

FnSg vs FnPg

Fusobacterium nucleatum

Hackett Laboratory UW

Fn Summary Table | FnPg vs Fn | FnSg vs Fn | FnPgSg vs Fn | FnPgSg vs FnPg | FnSg vs FnPg | FnPgSg vs FnSg | Fn Coverage

Protein	FnSg vs FnPg Log$_2$ Ratio	Log$_2$ Sum	*q*-Value	*p*-Value	Raw *FnPg*	Raw *FnSg*	Normalized *FnPg*	Normalized *FnSg*	Description	Log$_2$ Ratios -6 -4 -2 0 2 4 6
FN1276	0.028	8.537	3.846e-1	8.16e-1	13	13	18.9877	16.9210	AAL95472.1| Hypothetical protein	
					18	22	19.1883	22.0000		
FN1277	-0.103	15.739	3.686e-1	7.621e-1	107	160	156.2837	208.2587	AAL95473.1| Aminoacyl-histidine dipeptidase	
					308	243	328.3339	243.0000		
FN1278	1.782	5.137							AAL95474.1| Acetyltransferase	
					3	11	3.1981	11.0000		
FN1279	-0.348	11.520	6.965e-2	4.101e-2	37	40	54.0420	52.0647	AAL95475.1| Zinc metallohydrolase, glyoxalase II family	
					64	44	68.2252	44.0000		
FN1280	0.758	12.654	2.161e-2	5.248e-3	40	89	58.4238	115.8439	AAL95476.1| Serine protease, V8 family	
					61	93	65.0272	93.0000		
FN1281	0.934	9.331	1.248e-3	4.666e-5	12	27	17.5271	35.1436	AAL95477.1| Cysteine protease	
					18	35	19.1883	35.0000		
FN1282	-0.221	12.809	2.555e-1	4.482e-1	80	66	116.8476	85.9067	AAL95478.1| LSU ribosomal protein L17P	
					62	71	66.0932	71.0000		
FN1283	0.506	18.113	7.992e-2	5.392e-2	287	404	419.1909	525.8531	AAL95479.1| DNA-directed RNA polymerase alpha chain	
					445	743	474.3786	743.0000		
FN1284	-0.404	17.713	2.38e-1	4.058e-1	525	324	766.8126	421.7238	AAL95480.1| SSU ribosomal protein S4P	
					281	384	299.5514	384.0000		
FN1285	-3.654	12.646	1.466e-1	1.806e-1	373	17	544.8021	22.1275	AAL95481.1| SSU ribosomal protein S11P	
					22	23	23.4524	23.0000		
FN1286	0.784	12.429	2.419e-2	6.321e-3	41	66	59.8844	85.9067	AAL95482.1| SSU ribosomal protein S13P	
					50	109	53.3010	109.0000		
FN1287	-1.206	7.823	1.397e-1	1.624e-1	24	6	35.0543	7.8097	AAL95483.1| Bacterial Protein Translation Initiation Factor 1 (IF-1)	
					10	12	10.6602	12.0000		
FN1290	-1.581	9.681	1.784e-1	2.584e-1	62	17	90.5569	22.1275	AAL95486.1| Hypothetical protein	
					8	11	8.5282	11.0000		
FN1293						4		5.2065	AAL95489.1| Hypothetical protein	
FN1297	0.580	9.368	2.583e-2	7.093e-3	12	26	17.5271	33.8420	AAL95493.1| Methionine aminopeptidase	
					23	29	24.5184	29.0000		
FN1298	-0.692	11.831	1.109e-1	1.031e-1	62	53	90.5569	68.9857	AAL95494.1| Adenylate kinase	
					59	26	62.8951	26.0000		

Show detected proteins only
Show all proteins
Filter by category:
GO: amino acid transport

Proteins found: 1313

Enter (or paste) list of ORFs

Find ORFs

Test	Cutoff
q-Value	.005
p-Value	

	Signif	Direction	Applies To
red	yes	+	ratios, bars
yellow	no	n/a	bars
green	yes	–	ratios, bars
pink	yes	+	*p*- , *q*-Values
light green	yes	–	

Dot Plots Dot Plots

Protein	*FnSg* vs *FnPg* Log$_2$ Ratio	Log$_2$ Sum	*q*-Value	*p*-Value	Raw *FnPg*	Raw *FnSg*	Normalized *FnPg*	Normalized *FnSg*	Description
FN1299	-0.068	5.287				4		5.2065	AAL95495.1| dTDP-glucose 4,6-dehydratase
					6	7	6.3961	7.0000	
FN1301	-0.475	11.753	2.3e-1	3.849e-1	70	42	102.2417	54.6679	AAL95497.1| ABC transporter ATP-binding protein
					34	45	36.2447	45.0000	
FN1302	-2.002	15.517	1.294e-1	1.387e-1	489	81	714.2312	105.4309	AAL95498.1| Hypothetical protein
					143	111	152.4408	111.0000	
FN1303	-0.165	12.244	1.421e-1	1.683e-1	55	55	80.3328	71.5889	AAL95499.1| hypothetical cytosolic protein
					63	60	67.1592	60.0000	
FN1304	-0.228	14.009	1.296e-1	1.391e-1	83	87	121.2294	113.2406	AAL95500.1| Single-strand DNA binding protein
					147	124	156.7048	124.0000	
FN1305	-0.424	11.170	1.855e-1	2.762e-1	36	46	52.5814	59.8744	AAL95501.1| Hypothetical cytosolic protein
					55	23	58.6311	23.0000	
FN1306	0.100	15.236				135		175.7182	AAL95502.1| Methyltransferase
					178	231	189.7514	231.0000	
FN1309	-0.074	14.294	3.691e-1	7.639e-1	121	87	176.7321	113.2406	AAL95505.1| Hypothetical protein
					107	163	114.0641	163.0000	
FN1313	0.047	12.160	3.583e-1	7.289e-1	51	58	74.4904	75.4938	AAL95509.1| Oligopeptide-binding protein oppA
					55	62	58.6311	62.0000	
FN1315						6		7.8097	AAL95511.1| Hypothetical protein
FN1317	-0.341	7.182	1.95e-1	3.013e-1	12	8	17.5271	10.4129	AAL95513.1| RNA polymerase sigma factor
					9	11	9.5942	11.0000	
FN1318	-0.420	9.445	1.354e-1	1.52e-1	17	22	24.8301	28.6356	AAL95514.1| RNA polymerase sigma factor rpoD
					34	17	36.2447	17.0000	
FN1319	1.201	7.970	1.214e-1	1.221e-1	7	10	10.2242	13.0162	AAL95515.1| DNA primase
					10	35	10.6602	35.0000	
FN1320	0.117	14.990	2.657e-1	4.725e-1	116	168	169.4291	218.6716	AAL95516.1| Peptidyl-prolyl cis-trans isomerase
					166	157	176.9592	157.0000	
FN1321	0.311	20.057	2.495e-2	6.668e-3	641	849	936.2417	1105.0725	AAL95517.1| Acetoacetate metabolism regulatory protein atoC
					881	1221	939.1630	1221.0000	
FN1322	-0.832	9.528				19		24.7307	AAL95518.1| Membrane metalloprotease
					34	16	36.2447	16.0000	

Log$_2$ Ratios: -6 -4 -2 0 2 4 6

Show detected proteins only
Show all proteins
Filter by category:
GO: amino acid transport

Proteins found: 1313

Enter (or paste) list of ORFs

Find ORFs

Test	Cutoff
q-Value	.005
p-Value	

Signif	Direction	Applies To
yes	+	ratios, bars
no	n/a	bars
yes	−	ratios, bars
yes	+	*p*- , *q*-Values
yes	−	

Dot Plots Dot Plots

Protein	FnSg vs FnPg Log$_2$ Ratio	Log$_2$ Sum	q-Value	p-Value	Raw FnPg	Raw FnSg	Normalized FnPg	Normalized FnSg	Description
FN1323	-0.420	6.197	2.669e-3	2.034e-4	7	6	10.2242	7.8097	AAL95519.1| Thymidylate kinase
					9	7	9.5942	7.0000	
FN1324	0.333	10.799	2.213e-1	3.63e-1	15	39	21.9089	50.7630	AAL95520.1| 1-deoxy-D-xylulose 5-phosphate reductoisomerase
					50	44	53.3010	44.0000	
FN1326	-0.285	5.900							AAL95522.1| Undecaprenyl pyrophosphate synthetase
					8	7	8.5282	7.0000	
FN1327						11		14.3178	AAL95523.1| Dimethylallyltransferase
						18		18.0000	
FN1330	0.323	3.677							AAL95526.1| S-adenosylmethionine:tRNA ribosyltransferase-isomerase
					3	4	3.1981	4.0000	
FN1332	-0.219	10.234	1.362e-1	1.538e-1	25	21	36.5149	27.3339	AAL95528.1| Bacterial Peptide Chain Release Factor 1 (RF-1)
					36	37	38.3767	37.0000	
FN1333						7		9.1113	AAL95529.1| Hypothetical protein
						18		18.0000	
FN1334	-2.289	7.508	1.342e-1	1.492e-1	35	4	51.1208	5.2065	AAL95530.1| N-acetylmuramoyl-L-alanine amidase
					8	7	8.5282	7.0000	
FN1335	-0.725	11.755	1.196e-1	1.187e-1	40	18	58.4238	23.4291	AAL95531.1| Protein translocase subunit YajC
					87	68	92.7437	68.0000	
FN1336	-0.739	8.354			16		23.3695		AAL95532.1| Hypothetical protein
						14		14.0000	
FN1337	-0.188	5.803			8		11.6848		AAL95533.1| unknown
					4	7	4.2641	7.0000	
FN1340	-0.186	16.136	3.042e-1	5.707e-1	259	173	378.2942	225.1797	AAL95536.1| Glutamyl-tRNA synthetase
					182	278	194.0155	278.0000	
FN1341	-0.191	7.163				8		10.4129	AAL95537.1| Bacterial Peptide Chain Release Factor 2 (RF-2)
					12	12	12.7922	12.0000	
FN1343	-0.776	10.392				20		26.0323	AAL95539.1| seC-independent protein TATD
					45	30	47.9709	30.0000	
FN1345									AAL95541.1| 2-hydroxy-6-oxo-6-phenylhexa-2,4-dienoate hydrolase
						4		4.0000	
FN1346	1.590	6.945				15		19.5242	AAL95542.1| Hypothetical cytosolic protein
					6	19	6.3961	19.0000	

Log$_2$ Ratios: -6 -4 -2 0 2 4 6

Show detected proteins only
Show all proteins
Filter by category:
GO: amino acid transport

Proteins found: 1313

Enter (or paste) list of ORFs

Find ORFs

Test	Cutoff
q-Value	.005
p-Value	

Color	Signif	Direction	Applies To
red	yes	+	ratios, bars
yellow	no	n/a	bars
green	yes	–	ratios, bars
pink	yes	+	p- , q-Values
light green	yes	–	p- , q-Values

Dot Plots Dot Plots

Protein	FnSg vs FnPg Log$_2$ Ratio	Log$_2$ Sum	*q*-Value	*p*-Value	Raw *FnPg*	Raw *FnSg*	Normalized *FnPg*	Normalized *FnSg*	Description
FN1347	-0.229	5.740	6.766e-2	3.884e-2	5	5	7.3030	6.5081	AAL95543.1| Hypothetical cytosolic protein
					8	7	8.5282	7.0000	
FN1348	1.438	8.604	2.334e-2	5.951e-3	4	23	5.8424	29.9372	AAL95544.1| ABC transporter ATP-binding protein
					17	35	18.1223	35.0000	
FN1349	0.357	6.156				7		9.1113	AAL95545.1| ABC transporter permease protein
					7	10	7.4621	10.0000	
FN1351	-0.016	10.820	4.085e-1	9.017e-1	33	35	48.1997	45.5566	AAL95547.1| 15 kDa lipoprotein precursor
					35	39	37.3107	39.0000	
FN1352	0.047	11.397	3.625e-1	7.424e-1	32	35	46.7391	45.5566	AAL95548.1| ABC transporter ATP-binding protein
					52	60	55.4330	60.0000	
FN1353	-0.056	7.743				9		11.7145	AAL95549.1| ABC transporter permease protein
					14	17	14.9243	17.0000	
FN1354	-0.583	9.510				17		22.1275	AAL95550.1| ABC transporter permease protein
					31	22	33.0466	22.0000	
FN1358	-0.455	10.899				22		28.6356	AAL95554.1| Hypothetical protein
					48	46	51.1689	46.0000	
FN1359	0.621	18.794	1.536e-1	1.993e-1	167	760	243.9194	989.2286	AAL95555.1| Dipeptide-binding protein
					791	683	843.2212	683.0000	
FN1362	0.703	12.295	7.088e-2	4.238e-2	25	76	36.5149	98.9229	AAL95558.1| Dipeptide transport ATP-binding protein dppD
					70	82	74.6213	82.0000	
FN1363	0.947	11.699	5.358e-2	2.605e-2	16	70	23.3695	91.1132	AAL95559.1| Dipeptide transport ATP-binding protein dppF
					56	69	59.6971	69.0000	
FN1364					30		43.8179		AAL95560.1| LSU ribosomal protein L32P
FN1365	0.750	14.686	1.07e-1	9.628e-2	43	136	62.8056	177.0199	AAL95561.1| GTP-binding protein
					176	244	187.6194	244.0000	
FN1366	-0.376	14.422	1.007e-1	8.614e-2	99	83	144.5990	108.0342	AAL95562.1| Triosephosphate isomerase
					181	152	192.9495	152.0000	
FN1368									AAL95564.1| COMF operon protein 3
						4		4.0000	
FN1371	0.230	4.414							AAL95567.1| Ribonuclease HII
					4	5	4.2641	5.0000	

Log$_2$ Ratios: -6 -4 -2 0 2 4 6

Show detected proteins only
Show all proteins
Filter by category:
GO: amino acid transport

Proteins found: 1313

Enter (or paste) list of ORFs

Find ORFs

Test	Cutoff
q-Value	.005
p-Value	

	Signif	Direction	Applies To
red	yes	+	ratios, bars
yellow	no	n/a	bars
green	yes	–	ratios, bars
pink	yes	+	*p*- , *q*-Values
light green	yes	–	

Dot Plots Dot Plots

Protein	*FnSg* vs *FnPg* Log$_2$ Ratio	Log$_2$ Sum	*q*-Value	*p*-Value	Raw *FnPg*	Raw *FnSg*	Normalized *FnPg*	Normalized *FnSg*	Description
FN1373									AAL95569.1| regulatory protein
						5		5.0000	
FN1374	0.100	5.900							AAL95570.1| Transcriptional regulator
					7	8	7.4621	8.0000	
FN1375	-1.210	12.234				44		57.2711	AAL95571.1| Citrate-sodium symport
					99	34	105.5359	34.0000	
FN1376	0.724	19.389	8.708e-2	6.465e-2	282	647	411.8879	842.1459	AAL95572.1| Oxaloacetate decarboxylase alpha chain
					823	1288	877.3339	1288.0000	
FN1377						9		11.7145	AAL95573.1| CITG protein
FN1378	0.229	9.451			5	22	7.3030	28.6356	AAL95574.1| Citrate lyase acyl carrier protein
					39		41.5748		
FN1379	0.559	16.866	2.039e-1	3.218e-1	60	328	87.6357	426.9302	AAL95575.1| Citrate lyase beta chain
					452	412	481.8407	412.0000	
FN1380	0.404	18.871	3.009e-1	5.619e-1	81	431	118.3082	560.9967	AAL95576.1| Citrate lyase beta chain
					1018	1032	1085.2076	1032.0000	
FN1382	-0.985	6.600							AAL95578.1| ATPase
					13	7	13.8583	7.0000	
FN1383	0.715	7.130	9.362e-2	7.579e-2	9	11	13.1454	14.3178	AAL95579.1| DNA polymerase III alpha subunit
					5	16	5.3301	16.0000	
FN1385	1.796	5.150				4		5.2065	AAL95581.1| Hypothetical protein
					3	17	3.1981	17.0000	
FN1386	1.199	8.302				16		20.8259	AAL95582.1| SWF/SNF family helicase
					11	33	11.7262	33.0000	
FN1391	-0.662	11.201	7.426e-2	4.638e-2	50	37	73.0298	48.1598	AAL95584.1| Acetyltransferase
					46	29	49.0369	29.0000	
FN1392	-1.173	13.206	1.734e-1	2.466e-1	167	48	243.9194	62.4776	AAL95585.1| SSU ribosomal protein S16P
					45	67	47.9709	67.0000	
FN1393	1.375	9.546	3.124e-2	9.79e-3	5	30	7.3030	39.0485	AAL95586.1| Signal recognition particle, subunit FFH/SRP54
					25	49	26.6505	49.0000	
FN1397	-0.513	11.976	1.751e-1	2.505e-1	33	44	48.1997	57.2711	AAL95590.1| Glutaminase
					97	49	103.4039	49.0000	

Log$_2$ Ratios: -6 -4 -2 0 2 4 6

Show detected proteins only
Show all proteins
Filter by category:
GO: amino acid transport

Proteins found: 1313

Enter (or paste) list of ORFs

Find ORFs

Test	Cutoff
q-Value	.005
p-Value	

	Signif	Direction	Applies To
red	yes	+	ratios, bars
yellow	no	n/a	bars
green	yes	−	ratios, bars
pink	yes	+	*p*-, *q*-Values
light green	yes	−	

Dot Plots Dot Plots

Protein	FnSg vs FnPg				Raw		Normalized		Description
	Log_2 Ratio	Log_2 Sum	*q*-Value	*p*-Value	*FnPg*	*FnSg*	*FnPg*	*FnSg*	
**FN1398**	-0.419	11.378	2.868e-1	5.25e-1	16	34	23.3695	44.2550	AAL95591.1| Amino acid carrier protein alsT
					90	45	95.9417	45.0000	
**FN1399**									AAL95592.1| Hypothetical cytosolic protein
						3		3.0000	
**FN1400**					3		4.3818		AAL95593.1| serine/threonine kinase
					3		3.1981		
**FN1406**	0.065	12.427	4.069e-1	8.956e-1	22	59	32.1331	76.7954	AAL95599.1| Histidine ammonia-lyase
					106	75	112.9980	75.0000	
**FN1407**	-0.042	10.142				34		44.2550	AAL95600.1| Glutamate formiminotransferase
					32	22	34.1126	22.0000	
**FN1411**	0.940	18.282	3.584e-2	1.251e-2	196	594	286.2767	773.1602	AAL95604.1| Threonine dehydratase
					496	791	528.7456	791.0000	
**FN1412**	-1.714	8.023			20	6	29.2119	7.8097	AAL95605.1| 5-methylthioribose kinase
						10		10.0000	
**FN1413**	-1.868	5.868			10		14.6060		AAL95606.1| Translation initiation factor EIF-2B subunit 1
						4		4.0000	
**FN1415**	0.144	7.257			11		16.0666		AAL95608.1| NADH-dependent butanol dehydrogenase A
					7	13	7.4621	13.0000	
**FN1416**	-2.677	6.677							AAL95609.1| Transcriptional regulator, GntR family
					24	4	25.5845	4.0000	
**FN1417**	-0.201	6.848	2.65e-1	4.708e-1	7	10	10.2242	13.0162	AAL95610.1| L-fuculose phosphate aldolase
					12	7	12.7922	7.0000	
**FN1418**	-0.344	3.919			3	3	4.3818	3.9048	AAL95611.1| Transcriptional regulator, GntR family
						3		3.0000	
**FN1419**	-0.834	24.301	1.532e-1	1.982e-1	6075	2104	8873.1176	2738.6012	AAL95612.1| Methionine gamma-lyase
					3065	4072	3267.3491	4072.0000	
**FN1420**					44		64.2662		AAL95613.1| NA+/H+ antiporter NHAC
					16		17.0563		
**FN1421**	-0.421	21.950	2.063e-1	3.271e-1	2193	1356	3203.0859	1764.9921	AAL95614.1| Pyruvate-flavodoxin oxidoreductase
					1365	1714	1455.1163	1714.0000	
**FN1423**	1.226	14.689	4.488e-2	1.858e-2	36	186	52.5814	242.1007	AAL95616.1| Flavoprotein
					150	255	159.9029	255.0000	

Log_2 Ratios: -6 -4 -2 0 2 4 6

Show detected proteins only
Show all proteins
Filter by category:
GO: amino acid transport

Proteins found: 1313

Enter (or paste) list of ORFs

Find ORFs

Test	Cutoff
q-Value	.005
p-Value	

	Signif	Direction	Applies To
red	yes	+	ratios, bars
yellow	no	n/a	bars
green	yes	–	ratios, bars
pink	yes	+	*p*- , *q*-Values
light green	yes	–	

Dot Plots Dot Plots

FnSg vs FnPg | # *Fusobacterium nucleatum* | **Hackett Laboratory** | UW

Fn Summary Table | FnPg vs Fn | FnSg vs Fn | FnPgSg vs Fn | FnPgSg vs FnPg | FnSg vs FnPg | FnPgSg vs FnSg | Fn Coverage

Protein	*FnSg* vs *FnPg* Log$_2$ Ratio	Log$_2$ Sum	*q*-Value	*p*-Value	Raw *FnPg*	Raw *FnSg*	Normalized *FnPg*	Normalized *FnSg*	Description
**FN1424**	1.643	16.405	1.887e-2	4.262e-3	45	362	65.7268	471.1852	AAL95617.1| ACYL-COA dehydrogenase, short-chain specific
					251	570	267.5708	570.0000	
**FN1426**	1.070	15.537	7.141e-2	4.299e-2	44	232	64.2662	301.9750	AAL95619.1| Serine protease
					222	330	236.6563	330.0000	
**FN1427**									AAL95620.1| Phenazine biosynthesis protein phzF
					10		10.6602		
**FN1432**									AAL95625.1| Leucine-, isoleucine-, valine-, threonine-, and alanine-binding protein
						4		4.0000	
**FN1433**	-0.581	15.783	8.306e-4	2.149e-5	202	150	295.0403	195.2425	AAL95626.1| Short chain dehydrogenase
					268	193	285.6932	193.0000	
**FN1434**	0.447	13.985	1.195e-1	1.186e-1	53	114	77.4116	148.3843	AAL95627.1| Tetratricopeptide repeat family protein
					132	149	140.7145	149.0000	
**FN1437**	-0.852	12.982	1.088e-1	9.949e-2	110	56	160.6655	72.8905	AAL95630.1| LSU ribosomal protein L28P
					76	61	81.0175	61.0000	
**FN1439**	-1.461	7.770				6		7.8097	AAL95632.1| Transcriptional regulator, DeoR family
					23	10	24.5184	10.0000	
**FN1440**	-0.941	9.564	4.847e-2	2.137e-2	23	9	33.5937	11.7145	AAL95633.1| 1-phosphofructokinase
					40	28	42.6408	28.0000	
**FN1441**	1.148	12.880	4.152e-2	1.608e-2	20	111	29.2119	144.4794	AAL95634.1| PTS system, fructose-specific IIABC component
					82	114	87.4136	114.0000	
**FN1444**	-0.356	17.577	4.469e-2	1.842e-2	374	274	546.2627	356.6429	AAL95637.1| GMP synthase (glutamine-hydrolyzing)
					426	425	454.1242	425.0000	
**FN1445**	-0.401	8.302	1.329e-1	1.462e-1	17	13	24.8301	16.9210	AAL95638.1| DNA helicase
					15	14	15.9903	14.0000	
**FN1448**									AAL95641.1| Hypothetical cytosolic protein
						4		4.0000	
**FN1449**	-1.330	10.579	6.598e-2	3.709e-2	55	21	80.3328	27.3339	AAL95642.1| Fusobacterium outer membrane protein family
					41	22	43.7068	22.0000	
**FN1450**	0.976	9.500				29		37.7469	AAL95643.1| Integral membrane protein
					18		19.1883		
**FN1451**	0.625	15.589	2.416e-3	1.684e-4	120	204	175.2715	265.5298	AAL95644.1| Cell division protein ftsZ
					171	286	182.2893	286.0000	

Log$_2$ Ratios: -6 -4 -2 0 2 4 6

- Show detected proteins only
- Show all proteins
- Filter by category: GO: amino acid transport

Proteins found: 1313

Enter (or paste) list of ORFs

Find ORFs

Test	Cutoff
q-Value	.005
p-Value	

Color	Signif	Direction	Applies To
red	yes	+	ratios, bars
yellow	no	n/a	bars
green	yes	−	ratios, bars
pink	yes	+	*p*-, *q*-Values
light green	yes	−	

Dot Plots | Dot Plots

Protein	*FnSg* vs *FnPg* Log$_2$ Ratio	Log$_2$ Sum	*q*-Value	*p*-Value	Raw *FnPg*	Raw *FnSg*	Normalized *FnPg*	Normalized *FnSg*	Description
FN1452	0.325	14.157	8.838e-2	6.682e-2	96	108	140.2172	140.5746	AAL95645.1| Cell division protein ftsA
					95	162	101.2718	162.0000	
FN1454	0.113	9.082				8		10.4129	AAL95647.1| D-alanine--D-alanine ligase
					21	38	22.3864	38.0000	
FN1455	-0.170	8.801	2.695e-1	4.816e-1	19	16	27.7513	20.8259	AAL95648.1| UDP-N-acetylenolpyruvoylglucosamine reductase
					16	19	17.0563	19.0000	
FN1456	0.191	11.853	6.143e-2	3.269e-2	40	53	58.4238	68.9857	AAL95649.1| UDP-N-acetylmuramate--alanine ligase
					52	61	55.4330	61.0000	
FN1457	0.203	11.144	3.468e-1	6.931e-1	14	50	20.4483	65.0808	AAL95650.1| UDP-N-acetylglucosamine-N-acetylmuramyl-Pentapeptide pyrophosphoryl-undecaprenol N- [illegible]
					64	37	68.2252	37.0000	
FN1458	-0.401	12.411	2.487e-1	4.318e-1	84	38	122.6900	49.4614	AAL95651.1| UDP-N-acetylmuramoylalanine--D-glutamate ligase
					44	79	46.9048	79.0000	
FN1461	-0.451	9.264	1.945e-1	2.999e-1	20	8	29.2119	10.4129	AAL95654.1| Histidinol-phosphatase
					27	32	28.7825	32.0000	
FN1462						3		3.9048	AAL95655.1| Transcriptional regulator, GntR family
FN1463	0.222	17.197	1.422e-1	1.686e-1	285	332	416.2697	432.1367	AAL95656.1| pyridoxine biosynthesis protein
					283	405	301.6835	405.0000	
FN1464	0.648	16.496	1.192e-1	1.179e-1	91	302	132.9142	393.0882	AAL95657.1| 1-deoxyxylulose-5-phosphate synthase
					331	368	352.8524	368.0000	
FN1470	-1.764	6.380	5.236e-2	2.485e-2	15	3	21.9089	3.9048	AAL95663.1| Hypothetical protein
					11	6	11.7262	6.0000	
FN1471						3		3.9048	AAL95664.1| LACI-family transcription regulator
						4		4.0000	
FN1472	-0.154	9.518	3.807e-1	8.026e-1	7	21	10.2242	27.3339	AAL95665.1| N-acetylneuraminate-binding protein
					44	24	46.9048	24.0000	
FN1475	-0.916	7.560			20		29.2119		AAL95668.1| N-acetylneuraminate lyase
					8	10	8.5282	10.0000	
FN1478	-0.235	8.996				16		20.8259	AAL95671.1| Hypothetical protein
					23		24.5184		
FN1479	0.140	9.019	3.305e-1	6.446e-1	10	16	14.6060	20.8259	AAL95672.1| Hypothetical protein
					27	27	28.7825	27.0000	

Log$_2$ Ratios: -6 -4 -2 0 2 4 6

Show detected proteins only
Show all proteins
Filter by category: GO: amino acid transport

Proteins found: 1313

Enter (or paste) list of ORFs

Find ORFs

Test	Cutoff
q-Value	.005
p-Value	

	Signif	Direction	Applies To
red	yes	+	ratios, bars
yellow	no	n/a	bars
green	yes	−	ratios, bars
pink	yes	+	*p*- , *q*-Values
light green	yes	−	

Dot Plots Dot Plots

Protein	*FnSg* vs *FnPg* Log$_2$ Ratio	Log$_2$ Sum	*q*-Value	*p*-Value	Raw *FnPg*	Raw *FnSg*	Normalized *FnPg*	Normalized *FnSg*	Description
FN1480	0.763	9.477	5.914e-3	7.434e-4	12	25	17.5271	32.5404	AAL95673.1| MG2+ transporter MGTE
					22	37	23.4524	37.0000	
FN1481	-0.602	7.232	1.709e-1	2.403e-1	9	3	13.1454	3.9048	AAL95674.1| Queuine tRNA-ribosyltransferase
					16	16	17.0563	16.0000	
FN1482	0.879	12.407	3.005e-2	9.148e-3	35	66	51.1208	85.9067	AAL95675.1| Guanosine-3',5'-bis (Diphosphate) 3'-pyrophosphohydrolase
					54	114	57.5650	114.0000	
FN1483	-0.344	9.581	4.053e-2	1.54e-2	23	17	33.5937	22.1275	AAL95676.1| Adenine phosphoribosyltransferase
					27	27	28.7825	27.0000	
FN1484						4		5.2065	AAL95677.1| Tetratricopeptide repeat family protein
						10		10.0000	
FN1485									AAL95678.1| Transporter
						4		4.0000	
FN1486									AAL95679.1| magnesium and cobalt efflux protein CorC
						5		5.0000	
FN1487	0.443	13.807	1.471e-1	1.82e-1	45	110	65.7268	143.1778	AAL95681.1| Chorismate mutase
					131	136	139.6485	136.0000	
FN1488	-0.402	8.577			22		32.1331		AAL95682.1| Methylenetetrahydrofolate dehydrogenase (NADP+)
					12	17	12.7922	17.0000	
FN1489	0.216	8.901	1.864e-1	2.785e-1	11	17	16.0666	22.1275	AAL95683.1| Methionyl-tRNA formyltransferase
					23	25	24.5184	25.0000	
FN1490	0.828	9.215	1.076e-1	9.738e-2	9	33	13.1454	42.9533	AAL95684.1| putative regulatory protein
					22	22	23.4524	22.0000	
FN1491	0.777	7.605				15		19.5242	AAL95685.1| PTS system, IIA component
					10	17	10.6602	17.0000	
FN1492									AAL95686.1| DNA repair protein recO
						4		4.0000	
FN1493	-1.000	6.049	8.03e-3	1.268e-3	7	5	10.2242	6.5081	AAL95687.1| Hypothetical protein
					12	5	12.7922	5.0000	
FN1494	0.033	6.861				6		7.8097	AAL95680.1| Rod shape-determining protein mreC
					10	14	10.6602	14.0000	
FN1496	0.033	6.861				6		7.8097	AAL95680.1| Rod shape-determining protein mreC
					10	14	10.6602	14.0000	

Log$_2$ Ratios: -6 -4 -2 0 2 4 6

Show detected proteins only
Show all proteins
Filter by category:
GO: amino acid transport

Proteins found: 1313

Enter (or paste) list of ORFs

Find ORFs

Test	Cutoff
q-Value	.005
p-Value	

Signif	Direction	Applies To
yes	+	ratios, bars
no	n/a	bars
yes	−	ratios, bars
yes	+	*p*- , *q*-Values
yes	−	

Dot Plots Dot Plots

Protein	*FnSg* vs *FnPg* Log$_2$ Ratio	Log$_2$ Sum	*q*-Value	*p*-Value	Raw *FnPg*	Raw *FnSg*	Normalized *FnPg*	Normalized *FnSg*	Description
FN1499	-1.272	10.444	1.659e-1	2.285e-1	13	20	18.9877	26.0323	AAL93625.1| Cell surface protein
					91	22	97.0078	22.0000	
FN1504	0.694	13.114	1.224e-1	1.242e-1	43	121	62.8056	157.4956	AAL93630.1| Nickel-binding protein
					80	82	85.2815	82.0000	
FN1505	-0.443	15.941	6.778e-2	3.897e-2	172	183	251.2224	238.1958	AAL93631.1| 6,7-dimethyl-8-ribityllumazine synthase
					313	192	333.6640	192.0000	
FN1506	-1.209	7.549			19		27.7513		AAL93632.1| Diaminohydroxyphosphoribosylaminopyrimidine deaminase
					13	9	13.8583	9.0000	
FN1507									AAL93633.1| Riboflavin synthase alpha chain
						8		8.0000	
FN1508	-0.792	12.746	1.003e-1	8.55e-2	53	33	77.4116	42.9533	AAL93634.1| GTP cyclohydrolase II
					132	83	140.7145	83.0000	
FN1517	-0.411	15.052	1.736e-1	2.47e-1	102	115	148.9807	149.6859	AAL93643.1| Leucyl-tRNA synthetase
					259	170	276.0990	170.0000	
FN1518									AAL93644.1| RNA polymerase sigma-H factor
						6		6.0000	
FN1519	0.452	8.038				13		16.9210	AAL93645.1| 23S rRNA methyltransferase
					13	21	13.8583	21.0000	
FN1520	0.100	10.114	3.942e-1	8.496e-1	9	23	13.1454	29.9372	AAL93646.1| UDP-N-acetylglucosamine 1-carboxyvinyltransferase
					48	39	51.1689	39.0000	
FN1523	-0.203	13.000	1.072e-1	9.664e-2	68	72	99.3205	93.7164	AAL93649.1| Dipeptide-binding protein
					89	75	94.8757	75.0000	
FN1526	-2.198	20.190	1.055e-1	9.383e-2	2501	332	3652.9493	432.1367	AAL93652.1| Fusobacterium outer membrane protein family
					968	589	1031.9067	589.0000	
FN1527	-0.218	9.840	1.995e-1	3.118e-1	25	17	36.5149	22.1275	AAL93653.1| Hypothetical protein
					27	34	28.7825	34.0000	
FN1528	0.007	11.708	4.299e-1	9.843e-1	52	33	75.9510	42.9533	AAL93654.1| Hypothetical protein
					37	73	39.4427	73.0000	
FN1529	-2.247	10.339	1.041e-1	9.151e-2	84	20	122.6900	26.0323	AAL93655.1| Hypothetical protein
					32	7	34.1126	7.0000	
FN1531	0.663	10.961	9.296e-2	7.46e-2	15	41	21.9089	53.3663	AAL93657.1| murein hydrolase export regulator
					46	59	49.0369	59.0000	

Log$_2$ Ratios: -6 -4 -2 0 2 4 6

Show detected proteins only
Show all proteins
Filter by category:
GO: amino acid transport

Proteins found: 1313

Enter (or paste) list of ORFs

Find ORFs

Test	Cutoff
q-Value	.005
p-Value	

Signif	Direction	Applies To
yes	+	ratios, bars
no	n/a	bars
yes	−	ratios, bars
yes	+	*p*- , *q*-Values
yes	−	

Dot Plots Dot Plots

Protein	*FnSg* vs *FnPg* Log$_2$ Ratio	Log$_2$ Sum	*q*-Value	*p*-Value	Raw *FnPg*	Raw *FnSg*	Normalized *FnPg*	Normalized *FnSg*	Description
**FN1533**	-0.991	18.717	1.852e-1	2.753e-1	1043	329	1523.4011	428.2318	AAL93659.1| Electron transfer flavoprotein alpha-subunit
					307	503	327.2679	503.0000	
**FN1534**	-0.777	20.252	1.727e-1	2.449e-1	1502	651	2193.8144	847.3524	AAL93660.1| Electron transfer flavoprotein beta-subunit
					686	860	731.2892	860.0000	
**FN1535**	-0.565	21.956	3.373e-2	1.122e-2	1883	1107	2750.3013	1440.8895	AAL93661.1| Acyl-CoA dehydrogenase, short-chain specific
					2023	1875	2156.5570	1875.0000	
**FN1536**	-0.292	20.828	1.193e-1	1.182e-1	905	806	1321.8389	1049.1029	AAL93662.1| (S)-2-hydroxy-acid oxidase chain D
					1593	1417	1698.1687	1417.0000	
**FN1537**	-0.225	4.868			4		5.8424		AAL93663.1| Arsenical pump-driving ATPase
						5		5.0000	
**FN1538**	0.783	8.680	4.243e-2	1.672e-2	8	17	11.6848	22.1275	AAL93664.1| Arsenical pump-driving ATPase
					18	31	19.1883	31.0000	
**FN1539**	-0.510	15.861	1.176e-1	1.149e-1	246	162	359.3065	210.8619	AAL93665.1| Iron-sulfur cluster-binding protein
					209	198	222.7980	198.0000	
**FN1540**	-0.281	17.811	1.944e-1	2.997e-1	450	355	657.2680	462.0739	AAL93666.1| Iron-sulfur cluster-binding protein
					375	408	399.7572	408.0000	
**FN1544**	-0.315	18.849	2.807e-1	5.095e-1	762	428	1112.9738	557.0919	AAL93670.1| Probable electron transfer flavoprotein-quinone oxidoreductase ydiS
					394	675	420.0116	675.0000	
**FN1545**	-1.939	12.421	1.475e-1	1.832e-1	173	32	252.6830	41.6517	AAL93671.1| Ferredoxin like protein
					35	34	37.3107	34.0000	
**FN1546**	-0.526	21.541	2.201e-1	3.6e-1	2138	1041	3122.7532	1354.9828	AAL93672.1| Protein Translation Elongation Factor G (EF-G)
					1003	1557	1069.2173	1557.0000	
**FN1547**	-0.143	13.916	2.21e-1	3.623e-1	76	95	111.0053	123.6536	AAL93673.1| PTS permease for N-acetylglucosamine and glucose
					141	113	150.3087	113.0000	
**FN1548**	0.120	9.855	3.86e-1	8.207e-1	21	38	30.6725	49.4614	AAL93674.1| Hypothetical protein
					26	14	27.7165	14.0000	
**FN1549**	0.348	18.942	1.557e-1	2.042e-1	362	750	528.7356	976.2124	AAL93675.1| Stomatin like protein
					684	625	729.1572	625.0000	
**FN1552**	1.078	5.262							AAL93678.1| abortive phage resistance protein
					4	9	4.2641	9.0000	
**FN1553**	0.978	7.942	1.243e-1	1.279e-1	8	10	11.6848	13.0162	AAL93679.1| abortive phage resistance protein
					10	31	10.6602	31.0000	

Log$_2$ Ratios: -6 -4 -2 0 2 4 6

Show detected proteins only
Show all proteins
Filter by category:
GO: amino acid transport

Proteins found: 1313

Enter (or paste) list of ORFs

Find ORFs

Test	Cutoff
q-Value	.005
p-Value	

	Signif	Direction	Applies To
red	yes	+	ratios, bars
yellow	no	n/a	bars
green	yes	−	ratios, bars
pink	yes	+	*p*- , *q*-Values
light green	yes	−	

Dot Plots Dot Plots

Fusobacterium nucleatum

Protein	FnSg vs FnPg: Log_2 Ratio	Log_2 Sum	*q*-Value	*p*-Value	Raw: *FnPg*	Raw: *FnSg*	Normalized: *FnPg*	Normalized: *FnSg*	Description
FN1554	-0.451	15.586	8.303e-2	5.837e-2	158	114	230.7741	148.3843	AAL93680.1| Fusobacterium outer membrane protein family
					270	231	287.8252	231.0000	
FN1555	0.168	23.924	2.456e-3	1.746e-4	2528	3291	3692.3854	4283.6201	AAL93681.1| Protein Translation Elongation Factor Tu
					3600	4174	3837.6694	4174.0000	
FN1556	-0.413	18.954	9.26e-2	7.395e-2	621	372	907.0298	484.2014	AAL93682.1| Protein Translation Elongation Factor G (EF-G)
					692	751	737.6853	751.0000	
FN1557	-0.358	14.678	1.171e-3	4.066e-5	124	113	181.1138	147.0827	AAL93683.1| SSU ribosomal protein S7P
					174	139	185.4874	139.0000	
FN1558					128		186.9562		AAL93684.1| SSU ribosomal protein S12P
					32		34.1126		
FN1560	1.286	14.864	8.326e-2	5.871e-2	12	200	17.5271	260.3233	AAL93686.1| unknown
					191	279	203.6097	279.0000	
FN1562	1.011	6.833	4.761e-2	2.068e-2	3	11	4.3818	14.3178	AAL93688.1| Phospho-2-dehydro-3-deoxyheptonate aldolase
					10	16	10.6602	16.0000	
FN1576						5		6.5081	AAL93691.1| ATPases and helicase subunits involved in DNA replication
FN1577	0.696	16.606	7.488e-2	4.715e-2	112	325	163.5867	423.0254	AAL93692.1| Rod shape-determining protein mreB
					312	381	332.5980	381.0000	
FN1579	0.292	13.243	1.894e-1	2.863e-1	43	76	62.8056	98.9229	AAL93694.1| Cysteinyl-tRNA synthetase
					108	119	115.1301	119.0000	
FN1580									AAL93695.1| 2-C-methyl-D-erythritol 4-phosphate cytidylyltransferase
						3		3.0000	
FN1581	0.985	11.419	3.48e-2	1.189e-2	21	67	30.6725	87.2083	AAL93696.1| DNA mismatch repair protein mutS
					41	60	43.7068	60.0000	
FN1582	-0.040	9.063				12		15.6194	AAL93697.1| Hypothetical protein
					22	30	23.4524	30.0000	
FN1586	-0.890	7.635				9		11.7145	AAL93701.1| O-succinylbenzoate-CoA synthase
					18	9	19.1883	9.0000	
FN1589	0.973	9.052	8.254e-2	5.765e-2	5	25	7.3030	32.5404	AAL93704.1| LexA repressor
					24	32	25.5845	32.0000	
FN1590	-0.431	4.397				3		3.9048	AAL93705.1| Hypothetical lipoprotein
					5	4	5.3301	4.0000	

Log_2 Ratios: -6 -4 -2 0 2 4 6

Show detected proteins only
Show all proteins
Filter by category:
GO: amino acid transport

Proteins found: 1313

Enter (or paste) list of ORFs

Find ORFs

Test	Cutoff
q-Value	.005
p-Value	

Color	Signif	Direction	Applies To
red	yes	+	ratios, bars
yellow	no	n/a	bars
green	yes	–	ratios, bars
pink	yes	+	*p*-, *q*-Values
light green	yes	–	

Dot Plots Dot Plots

Protein	*FnSg* vs *FnPg* Log$_2$ Ratio	Log$_2$ Sum	*q*-Value	*p*-Value	Raw *FnPg*	Raw *FnSg*	Normalized *FnPg*	Normalized *FnSg*	Description
FN1591	0.041	15.046	3.943e-1	8.498e-1	141	173	205.9440	225.1797	AAL93706.1| RNFB-related protein
					147	148	156.7048	148.0000	
FN1592	-1.208	6.378							AAL93707.1| Na(+)-translocating NADH-quinone reductase subunit D
					13	6	13.8583	6.0000	
FN1594	-0.252	10.508	2.101e-1	3.36e-1	22	23	32.1331	29.9372	AAL93709.1| Nitrogen fixation protein RNFG
					48	40	51.1689	40.0000	
FN1595	-0.471	14.033	2.854e-1	5.215e-1	35	76	51.1208	98.9229	AAL93710.1| Na(+)-translocating NADH-quinone reductase subunit B
					238	121	253.7126	121.0000	
FN1596	-0.833	18.228	1.435e-1	1.721e-1	722	359	1054.5499	467.2803	AAL93711.1| Nitrogen fixation iron-sulphur protein RNFC
					398	363	424.2757	363.0000	
FN1597					20		29.2119		AAL93712.1| Peptidyl-tRNA hydrolase
FN1600	-0.796	6.307				5		6.5081	AAL93715.1| tRNA pseudouridine synthase A
					11	7	11.7262	7.0000	
FN1601						5		6.5081	AAL93716.1| Hypothetical cytosolic protein
						12		12.0000	
FN1603	-0.344	4.749			4	4	5.8424	5.2065	AAL93718.1| 2',3'-cyclic nucleotide 3'-phosphodiesterase
						4		4.0000	
FN1605	-0.006	16.615	4.282e-1	9.776e-1	169	240	246.8406	312.3880	AAL93720.1| Adenylosuccinate synthetase
					364	320	388.0310	320.0000	
FN1606	0.749	9.406	4.704e-2	2.022e-2	10	25	14.6060	32.5404	AAL93721.1| 3-deoxy-D-manno-octulosonic-acid transferase
					24	35	25.5845	35.0000	
FN1607	0.062	8.510	3.944e-1	8.503e-1	11	10	16.0666	13.0162	AAL93722.1| Cytidylate kinase
					20	26	21.3204	26.0000	
FN1608	0.087	6.271				7		9.1113	AAL93723.1| Ribosomal protein L11 methyltransferase
					8	9	8.5282	9.0000	
FN1609	-1.523	5.832				3		3.9048	AAL93724.1| Hypothetical protein
					12	5	12.7922	5.0000	
FN1610	-0.161	10.547	3.236e-2	1.041e-2	29	27	42.3573	35.1436	AAL93725.1| 33 kDa chaperonin
					37	38	39.4427	38.0000	
FN1613	-0.112	8.610	3.461e-1	6.909e-1	15	10	21.9089	13.0162	AAL93728.1| Hypothetical protein
					18	25	19.1883	25.0000	

Log$_2$ Ratios: -6 -4 -2 0 2 4 6

Show detected proteins only
Show all proteins
Filter by category:
GO: amino acid transport

Proteins found: 1313

Enter (or paste) list of ORFs

Find ORFs

Test	Cutoff
q-Value	.005
p-Value	

	Signif	Direction	Applies To
red	yes	+	ratios, bars
yellow	no	n/a	bars
green	yes	–	ratios, bars
pink	yes	+	*p*- , *q*-Values
light green	yes	–	

Dot Plots | Dot Plots

Protein	*FnSg* vs *FnPg* Log$_2$ Ratio	Log$_2$ Sum	*q*-Value	*p*-Value	Raw *FnPg*	Raw *FnSg*	Normalized *FnPg*	Normalized *FnSg*	Description
**FN1614**	-0.920	8.975	2.841e-2	8.311e-3	24	12	35.0543	15.6194	AAL93729.1| MG(2+) chelatase family protein
					25	17	26.6505	17.0000	
**FN1616**	-0.507	7.677							AAL93731.1| N utilization substance protein B
					16	12	17.0563	12.0000	
**FN1618**	0.500	10.996	1.242e-1	1.277e-1	17	38	24.8301	49.4614	AAL93733.1| Hypothetical protein
					48	58	51.1689	58.0000	
**FN1619**	0.010	15.674	4.265e-1	9.71e-1	134	129	195.7198	167.9085	AAL93734.1| Hypothetical cytosolic protein
					244	291	260.1087	291.0000	
**FN1620**	-0.455	17.483	1.904e-1	2.889e-1	468	296	683.5587	385.2785	AAL93735.1| SSU ribosomal protein S2P
					299	346	318.7398	346.0000	
**FN1621**	-0.957	19.151	7.665e-2	4.943e-2	923	379	1348.1296	493.3127	AAL93736.1| Protein Translation Elongation Factor Ts
					730	602	778.1941	602.0000	
**FN1622**	0.105	13.423	3.79e-1	7.969e-1	53	51	77.4116	66.3824	AAL93737.1| Uridylate kinase
					117	151	124.7243	151.0000	
**FN1623**	-1.182	9.427	2.799e-2	8.104e-3	22	16	32.1331	20.8259	AAL93738.1| Ribosome Recycling Factor (RRF)
					44	14	46.9048	14.0000	
**FN1624**	-0.304	8.800							AAL93739.1| Protein translocase subunit secY
					22	19	23.4524	19.0000	
**FN1625**	-0.240	12.596	3.144e-1	5.987e-1	85	59	124.1506	76.7954	AAL93740.1| LSU ribosomal protein L15P
					44	68	46.9048	68.0000	
**FN1626**	-0.940	5.584							AAL93741.1| LSU ribosomal protein L30P
					9	5	9.5942	5.0000	
**FN1627**	-0.117	16.142	1.286e-1	1.369e-1	193	184	281.8949	239.4974	AAL93742.1| SSU ribosomal protein S5P
					261	277	278.2310	277.0000	
**FN1628**	-4.097	12.028			183	12	267.2890	15.6194	AAL93743.1| LSU ribosomal protein L18P
**FN1629**	-0.844	16.119	1.659e-1	2.283e-1	369	167	538.9597	217.3700	AAL93744.1| LSU ribosomal protein L6P
					165	181	175.8932	181.0000	
**FN1630**	-0.217	12.465	6.395e-2	3.507e-2	57	58	83.2539	75.4938	AAL93745.1| SSU ribosomal protein S8P
					74	64	78.8854	64.0000	
**FN1631**	-0.449	4.379				3		3.9048	AAL93746.1| SSU ribosomal protein S14P
					5		5.3301		

Log$_2$ Ratios: -6 -4 -2 0 2 4 6

Show detected proteins only
Show all proteins
Filter by category:
GO: amino acid transport

Proteins found: 1313

Enter (or paste) list of ORFs

Find ORFs

Test	Cutoff
q-Value	.005
p-Value	

	Signif	Direction	Applies To
red	yes	+	ratios, bars
yellow	no	n/a	bars
green	yes	−	ratios, bars
pink	yes	+	*p*- , *q*-Values
light green	yes	−	

Dot Plots Dot Plots

Protein	*FnSg* vs *FnPg* Log$_2$ Ratio	Log$_2$ Sum	*q*-Value	*p*-Value	Raw *FnPg*	Raw *FnSg*	Normalized *FnPg*	Normalized *FnSg*	Description	Log$_2$ Ratios (-6 -4 -2 0 2 4 6)
**FN1632**	0.107	15.729	2.263e-1	3.754e-1	152	208	222.0105	270.7362	AAL93747.1| LSU ribosomal protein L5P	
					213	213	227.0621	213.0000		
**FN1634**	-2.020	10.182			47	13	68.6480	16.9210	AAL93749.1| LSU ribosomal protein L24P	
**FN1635**	-0.675	7.774	3.498e-2	1.201e-2	11	8	16.0666	10.4129	AAL93750.1| LSU ribosomal protein L14P	
					20	13	21.3204	13.0000		
**FN1636**	1.554	10.981	3.977e-2	1.49e-2	6	60	8.7636	78.0970	AAL93751.1| SSU ribosomal protein S17P	
					41	76	43.7068	76.0000		
**FN1638**	-1.226	11.299	9.84e-2	8.267e-2	73	22	106.6235	28.6356	AAL93753.1| LSU ribosomal protein L16P	
					44	37	46.9048	37.0000		
**FN1639**	-0.471	18.371	1.656e-1	2.276e-1	619	355	904.1086	462.0739	AAL93754.1| SSU ribosomal protein S3P	
					438	527	466.9164	527.0000		
**FN1640**	-1.408	11.980	8.823e-2	6.657e-2	98	30	143.1384	39.0485	AAL93755.1| LSU ribosomal protein L22P	
					60	39	63.9612	39.0000		
**FN1641**	-2.874	11.348	9.194e-2	7.279e-2	147	9	214.7075	11.7145	AAL93756.1| SSU ribosomal protein S19P	
					58	26	61.8291	26.0000		
**FN1642**	-0.375	15.662	3.009e-1	5.621e-1	285	175	416.2697	227.7829	AAL93757.1| LSU ribosomal protein L2P	
					96	172	102.3379	172.0000		
**FN1643**	-1.746	12.047	1.294e-1	1.385e-1	131	30	191.3380	39.0485	AAL93758.1| LSU ribosomal protein L23P	
					44	32	46.9048	32.0000		
**FN1644**	0.352	19.284	1.219e-1	1.231e-1	594	721	867.5937	938.4655	AAL93759.1| LSU ribosomal protein L1E	
					513	867	546.8679	867.0000		
**FN1645**	-1.309	14.463	1.333e-1	1.471e-1	248	76	362.2277	98.9229	AAL93760.1| LSU ribosomal protein L3P	
					104	92	110.8660	92.0000		
**FN1646**	-0.808	14.268	1.432e-1	1.714e-1	180	84	262.9072	109.3358	AAL93761.1| SSU ribosomal protein S10P	
					102	103	108.7340	103.0000		
**FN1647**	-1.585	15.347	1.509e-1	1.925e-1	409	82	597.3836	106.7326	AAL93762.1| Hypothetical protein	
					103	129	109.8000	129.0000		
**FN1652**	0.383	11.998	3.084e-2	9.571e-3	38	60	55.5026	78.0970	AAL93767.1| Oligopeptide-binding protein oppA	
					53	68	56.4990	68.0000		
**FN1654**	1.045	12.905	6.716e-2	3.832e-2	20	98	29.2119	127.5584	AAL93769.1| Hypothetical protein	
					87	124	92.7437	124.0000		

Show detected proteins only
Show all proteins
Filter by category:
GO: amino acid transport

Proteins found: 1313

Enter (or paste) list of ORFs

Find ORFs

Test	Cutoff
***q*-Value**	.005
p-Value	

	Signif	Direction	Applies To
(red)	yes	+	ratios, bars
(yellow)	no	n/a	bars
(green)	yes	−	ratios, bars
(pink)	yes	+	*p*- , *q*-Values
(light green)	yes	−	

Dot Plots Dot Plots

Protein	*FnSg* vs *FnPg* Log$_2$ Ratio	Log$_2$ Sum	*q*-Value	*p*-Value	Raw *FnPg*	Raw *FnSg*	Normalized *FnPg*	Normalized *FnSg*	Description
**FN1655**	1.019	14.012	8.593e-2	6.28e-2	82	96	119.7688	124.9552	AAL93770.1| Hypothetical cytosolic protein
					57	241	60.7631	241.0000	
**FN1657**	-1.150	12.696	1.914e-1	2.915e-1	145	41	211.7863	53.3663	AAL93772.1| SSU ribosomal protein S6P
					29	56	30.9146	56.0000	
**FN1658**	0.548	13.374	1.277e-2	2.44e-3	59	90	86.1751	117.1455	AAL93773.1| Prolyl-tRNA synthetase
					79	132	84.2155	132.0000	
**FN1660**									AAL93775.1| ATP-dependent DNA helicase recG
						5		5.0000	
**FN1661**	-0.723	9.557	1.101e-2	1.97e-3	22	19	32.1331	24.7307	AAL93776.1| Hypothetical cytosolic protein
					36	18	38.3767	18.0000	
**FN1662**	0.783	10.104	4.415e-2	1.8e-2	12	30	17.5271	39.0485	AAL93777.1| Hypothetical protein
					31	48	33.0466	48.0000	
**FN1663**	0.096	6.620				5		6.5081	AAL93778.1| Hypothetical protein
					9	14	9.5942	14.0000	
**FN1667**	-0.547	3.716			3		4.3818		AAL93782.1| dTDP-glucose 4,6-dehydratase
						3		3.0000	
**FN1668**	-0.829	3.999							AAL93783.1| Cholinephosphate cytidylyltransferase
					5	3	5.3301	3.0000	
**FN1670**	-0.004	10.019	4.282e-1	9.776e-1	23	21	33.5937	27.3339	AAL93785.1| Choline kinase
					29	37	30.9146	37.0000	
**FN1676**									AAL93791.1| Transposase
						3		3.0000	
**FN1679**	0.299	18.089	1.499e-1	1.9e-1	249	423	363.6883	550.5838	AAL93794.1| LPS biosynthesis protein WbpG
					552	621	588.4426	621.0000	
**FN1683**	0.552	10.547	8.934e-2	6.837e-2	16	42	23.3695	54.6679	AAL93798.1| Acetyltransferase
					38	39	40.5087	39.0000	
**FN1684**	-0.793	16.631	7.831e-2	5.166e-2	358	146	522.8932	190.0360	AAL93799.1| N-acetylneuraminate synthase
					296	294	315.5417	294.0000	
**FN1685**	-0.455	11.975	2.275e-1	3.784e-1	74	41	108.0841	53.3663	AAL93800.1| dTDP-4-dehydrorhamnose reductase
					38	55	40.5087	55.0000	
**FN1686**	-0.213	16.055	3.157e-1	6.022e-1	270	168	394.3608	218.6716	AAL93801.1| Spore coat polysaccharide biosynthesis protein spsF
					157	266	167.3650	266.0000	

Log$_2$ Ratios: -6 -4 -2 0 2 4 6

Show detected proteins only
Show all proteins
Filter by category:
GO: amino acid transport

Proteins found: 1313

Enter (or paste) list of ORFs

Find ORFs

Test	Cutoff
q-Value	.005
p-Value	

	Signif	Direction	Applies To
red	yes	+	ratios, bars
yellow	no	n/a	bars
green	yes	–	ratios, bars
pink	yes	+	*p*- , *q*-Values
light green	yes	–	

Dot Plots Dot Plots

Protein	*FnSg* vs *FnPg* Log$_2$ Ratio	Log$_2$ Sum	*q*-Value	*p*-Value	Raw *FnPg*	Raw *FnSg*	Normalized *FnPg*	Normalized *FnSg*	Description
FN1687	-0.183	15.256	1.884e-1	2.837e-1	147	117	214.7075	152.2891	AAL93802.1| Gluconate 5-dehydrogenase
					194	219	206.8077	219.0000	
FN1688	-1.666	12.786	1.371e-1	1.559e-1	167	31	243.9194	40.3501	AAL93803.1| Oxidoreductase
					52	54	55.4330	54.0000	
FN1689	-0.291	16.414	5.993e-2	3.134e-2	241	226	352.0035	294.1653	AAL93804.1| UDP-N-acetylglucosamine 4,6-dehydratase
					283	240	301.6835	240.0000	
FN1690	-0.231	11.021	2.826e-1	5.143e-1	45	27	65.7268	35.1436	AAL93805.1| Hypothetical protein
					31	49	33.0466	49.0000	
FN1692	0.605	10.687	1.438e-3	6.615e-5	21	37	30.6725	48.1598	AAL93807.1| Glycosyl transferase
					33	52	35.1786	52.0000	
FN1693	-0.547	7.634	1.491e-1	1.877e-1	8	11	11.6848	14.3178	AAL93808.1| Hypothetical protein
					21	9	22.3864	9.0000	
FN1694	-0.437	11.220	1.945e-1	3.001e-1	53	33	77.4116	42.9533	AAL93809.1| UDP-N-acetyl-D-quinovosamine 4-epimerase
					34	41	36.2447	41.0000	
FN1695	0.507	8.117	1.551e-1	2.027e-1	6	19	8.7636	24.7307	AAL93810.1| Probable quinovosaminephosphotransferae
					18	15	19.1883	15.0000	
FN1696	0.407	15.275	9.183e-2	7.259e-2	93	161	135.8354	209.5603	AAL93811.1| UDP-N-acetylglucosamine 4,6-dehydratase
					197	249	210.0058	249.0000	
FN1697	0.276	13.528	2.76e-1	4.976e-1	36	100	52.5814	130.1617	AAL93812.1| Hypothetical protein
					136	109	144.9786	109.0000	
FN1698	0.576	15.233	8.787e-2	6.597e-2	77	183	112.4659	238.1958	AAL93813.1| dTDP-4-dehydrorhamnose reductase
					196	241	208.9398	241.0000	
FN1700						3		3.9048	AAL93815.1| Hypothetical protein
FN1701	0.680	11.303	1.601e-2	3.37e-3	23	44	33.5937	57.2711	AAL93816.1| ABC transporter ATP-binding protein
					43	70	45.8388	70.0000	
FN1703	0.128	14.669	3.682e-1	7.608e-1	56	144	81.7933	187.4328	AAL93818.1| ADP-L-glycero-D-manno-heptose-6-epimerase
					213	150	227.0621	150.0000	
FN1704	0.401	7.002	1.814e-1	2.657e-1	4	10	5.8424	13.0162	AAL93819.1| Serine protease
					13	13	13.8583	13.0000	
FN1708	0.212	17.821	1.129e-2	2.045e-3	294	406	429.4151	528.4563	AAL93823.1| Polyribonucleotide nucleotidyltransferase
					436	507	464.7844	507.0000	

Log$_2$ Ratios: -6 -4 -2 0 2 4 6

Show detected proteins only
Show all proteins
Filter by category:
GO: amino acid transport

Proteins found: 1313

Enter (or paste) list of ORFs

Find ORFs

Test	Cutoff
q-Value	.005
p-Value	

	Signif	Direction	Applies To
(red)	yes	+	ratios, bars
(yellow)	no	n/a	bars
(green)	yes	−	ratios, bars
(pink)	yes	+	*p*- , *q*-Values
(light green)	yes	−	

Dot Plots Dot Plots

Protein	FnSg vs FnPg Log$_2$ Ratio	Log$_2$ Sum	*q*-Value	*p*-Value	Raw *FnPg*	Raw *FnSg*	Normalized *FnPg*	Normalized *FnSg*	Description	Log$_2$ Ratios (-6 -4 -2 0 2 4 6)
FN1713	0.705	7.808				14		18.2226	AAL93828.1| tRNA (Uracil-5-) -methyltransferase	
					11	20	11.7262	20.0000		
FN1715	1.695	5.050				9		11.7145	AAL93830.1| ATPase	
					3	9	3.1981	9.0000		
FN1716									AAL93831.1| Hypothetical protein	
						5		5.0000		
FN1717	-0.475	8.102	1.956e-1	3.031e-1	18	7	26.2907	9.1113	AAL93832.1| NAD-dependent DNA ligase	
					12	19	12.7922	19.0000		
FN1718	-0.001	17.082	4.314e-1	9.907e-1	284	284	414.8091	369.6591	AAL93833.1| Protein translocase subunit secA	
					310	375	330.4660	375.0000		
FN1719	0.530	17.139	6.164e-2	3.289e-2	238	405	347.6217	527.1547	AAL93834.1| Hypothetical protein	
					267	386	284.6271	386.0000		
FN1722						3		3.9048	AAL93837.1| Glucose inhibited division protein B	
						4		4.0000		
FN1723	0.233	12.178	1.456e-1	1.778e-1	40	65	58.4238	84.6051	AAL93838.1| Glucose inhibited division protein A	
					63	63	67.1592	63.0000		
FN1724	0.432	9.250	2.942e-1	5.443e-1	5	11	7.3030	14.3178	AAL93839.1| Potassium uptake protein KtrA	
					33	43	35.1786	43.0000		
FN1727						5		6.5081	AAL93842.1| Chloride channel protein	
FN1728	-0.935	7.493	6.035e-4	8.891e-6	13	8	18.9877	10.4129	AAL93843.1| Pyrrolidone-carboxylate peptidase	
					17	9	18.1223	9.0000		
FN1730	0.876	8.526	3.813e-2	1.386e-2	7	20	10.2242	26.0323	AAL93845.1| Para-aminobenzoate synthase component I	
					17	26	18.1223	26.0000		
FN1731	-0.441	6.017	3.786e-2	1.37e-2	7	6	10.2242	7.8097	AAL93846.1| Anthranilate synthase component II	
					8	6	8.5282	6.0000		
FN1732	0.240	11.677	2.866e-1	5.244e-1	21	41	30.6725	53.3663	AAL93847.1| Hypothetical protein	
					70	71	74.6213	71.0000		
FN1733	0.983	7.168				9		11.7145	AAL93848.1| V-type sodium ATP synthase subunit D	
					8	22	8.5282	22.0000		
FN1734	0.324	14.804	4.872e-3	5.482e-4	99	151	144.5990	196.5441	AAL93849.1| V-type sodium ATP synthase subunit B	
					148	182	157.7709	182.0000		

Show detected proteins only
Show all proteins
Filter by category:
GO: amino acid transport

Proteins found: 1313

Enter (or paste) list of ORFs

Find ORFs

Test	Cutoff
q-Value	.005
p-Value	

	Signif	Direction	Applies To
red	yes	+	ratios, bars
yellow	no	n/a	bars
green	yes	−	ratios, bars
light red	yes	+	*p*- , *q*-Values
light green	yes	−	

Dot Plots Dot Plots

Protein	FnSg vs FnPg				Raw		Normalized		Description
	Log_2 Ratio	Log_2 Sum	*q*-Value	*p*-Value	*FnPg*	*FnSg*	*FnPg*	*FnSg*	
FN1735	0.965	14.678	5.033e-2	2.297e-2	47	184	68.6480	239.4974	AAL93850.1| V-type sodium ATP synthase subunit A
					153	213	163.1009	213.0000	
FN1736	0.829	10.854	4.839e-2	2.131e-2	15	42	21.9089	54.6679	AAL93851.1| V-type sodium ATP synthase subunit A
					40	60	42.6408	60.0000	
FN1737	1.553	6.413	3.046e-3	2.633e-4	3	12	4.3818	15.6194	AAL93852.1| V-type sodium ATP synthase subunit G
					6	16	6.3961	16.0000	
FN1738	-0.128	13.021	3.688e-1	7.629e-1	94	61	137.2960	79.3986	AAL93853.1| V-type sodium ATP synthase subunit C
					50	95	53.3010	95.0000	
FN1739	-0.410	5.814			6	5	8.7636	6.5081	AAL93854.1| V-type sodium ATP synthase subunit E
					8		8.5282		
FN1740	-0.791	14.457							AAL93855.1| V-type sodium ATP synthase subunit K
					185	114	197.2136	114.0000	
FN1741	0.162	10.274	1.494e-1	1.884e-1	20	28	29.2119	36.4453	AAL93856.1| V-type sodium ATP synthase subunit I
					35	38	37.3107	38.0000	
FN1742					9		13.1454		AAL93857.1| V-type sodium ATP synthase subunit G
					8		8.5282		
FN1743									AAL93858.1| Multidrug-efflux transporter 2 regulator
					8		8.5282		
FN1745	-0.070	9.284				19		24.7307	AAL93860.1| Cystathionine gamma-synthase
					24	24	25.5845	24.0000	
FN1746	0.768	6.567				8		10.4129	AAL93861.1| Cystathionine beta-lyase
					7	15	7.4621	15.0000	
FN1754					11		16.0666		AAL93869.1| Thiazole biosynthesis protein thiG
FN1762						4		5.2065	AAL93875.1| Protein yaaA
FN1763	0.049	12.201	3.905e-1	8.363e-1	34	55	49.6602	71.5889	AAL93876.1| Hypothetical cytosolic protein
					80	68	85.2815	68.0000	
FN1764	0.776	24.995	1.441e-1	1.735e-1	926	5881	1352.5114	7654.8070	AAL93877.1| Enolase
					7022	7477	7485.5874	7477.0000	
FN1765	0.370	18.426	2.014e-1	3.161e-1	207	535	302.3433	696.3649	AAL93878.1| Pyruvate kinase
					696	653	741.9494	653.0000	

Log_2 Ratios: -6 -4 -2 0 2 4 6

Show detected proteins only
Show all proteins
Filter by category:
GO: amino acid transport

Proteins found: 1313

Enter (or paste) list of ORFs

Find ORFs

Test	Cutoff
q-Value	.005
p-Value	

Color	Signif	Direction	Applies To
red	yes	+	ratios, bars
yellow	no	n/a	bars
green	yes	−	ratios, bars
pink	yes	+	*p*-, *q*-Values
light green	yes	−	

Dot Plots Dot Plots

Protein	*FnSg* vs *FnPg* Log$_2$ Ratio	Log$_2$ Sum	*q*-Value	*p*-Value	Raw *FnPg*	Raw *FnSg*	Normalized *FnPg*	Normalized *FnSg*	Description	Log$_2$ Ratios
FN1780	0.065	7.520	4.014e-1	8.754e-1	5	9	7.3030	11.7145	AAL93879.1| Hypothetical protein	
					18	16	19.1883	16.0000		
FN1781	0.027	18.062	4.073e-1	8.973e-1	433	374	632.4378	486.8046	AAL93880.1| LytB protein	
					379	569	404.0213	569.0000		
FN1783	-0.552	6.552							AAL93882.1| Ethanolamine utilization protein eutJ	
					11	8	11.7262	8.0000		
FN1784	1.075	7.903				13		16.9210	AAL93883.1| unknown	
					10	28	10.6602	28.0000		
FN1785	0.047	7.632				11		14.3178	AAL93884.1| Hypothetical protein	
					13		13.8583			
FN1786	0.740	10.174	1.092e-1	1.002e-1	9	36	13.1454	46.8582	AAL93885.1| ADP-heptose synthase	
					37	41	39.4427	41.0000		
FN1787	-0.226	8.040			16		23.3695		AAL93886.1| Tetratricopeptide repeat family protein	
					11	15	11.7262	15.0000		
FN1788	-0.594	8.846	1.461e-1	1.792e-1	12	13	17.5271	16.9210	AAL93887.1| 2C-methyl-D-erythritol 2,4-cyclodiphosphate synthase	
					33	18	35.1786	18.0000		
FN1790	0.381	10.817	3.163e-1	6.041e-1	5	23	7.3030	29.9372	AAL93889.1| Cob(I)alamin adenosyltransferase	
					63	67	67.1592	67.0000		
FN1791						3		3.9048	AAL93890.1| Mutator MutT protein	
						5		5.0000		
FN1792	-0.988	22.914	1.031e-1	8.992e-2	3669	1257	5358.9249	1636.1320	AAL93891.1| Hypothetical protein	
					2401	2356	2559.5123	2356.0000		
FN1793	0.279	15.633	3.318e-1	6.484e-1	38	201	55.5026	261.6249	AAL93892.1| Phosphoenolpyruvate-protein phosphotransferase	
					332	235	353.9184	235.0000		
FN1794	0.097	10.952	3.806e-1	8.022e-1	29	50	42.3573	65.0808	AAL93893.1| Phosphocarrier protein HPr	
					41	27	43.7068	27.0000		
FN1795									AAL93894.1| Hypothetical protein	
						12		12.0000		
FN1796									AAL93895.1| unknown	
						8		8.0000		
FN1797	0.568	11.975	1.291e-1	1.38e-1	21	68	30.6725	88.5099	AAL93896.1| Spermidine/putrescine transport ATP-binding protein potA	
					69	66	73.5553	66.0000		

Log$_2$ Ratios axis: -6 -4 -2 0 2 4 6

Show detected proteins only
Show all proteins
Filter by category:
GO: amino acid transport

Proteins found: 1313

Enter (or paste) list of ORFs

Find ORFs

Test	Cutoff
q-Value	.005
p-Value	

	Signif	Direction	Applies To
red	yes	+	ratios, bars
yellow	no	n/a	bars
green	yes	–	ratios, bars
pink	yes	+	*p*- , *q*-Values
light green	yes	–	

Dot Plots Dot Plots

Protein	FnSg vs FnPg Log$_2$ Ratio	Log$_2$ Sum	*q*-Value	*p*-Value	Raw *FnPg*	Raw *FnSg*	Normalized *FnPg*	Normalized *FnSg*	Description
FN1798	-0.526	6.303				6		7.8097	AAL93897.1| Spermidine/putrescine transport system permease protein potB
					10	7	10.6602	7.0000	
FN1800	1.122	14.418	2.047e-2	4.821e-3	57	141	83.2539	183.5279	AAL93899.1| Peptidyl-prolyl cis-trans isomerase
					110	253	117.2621	253.0000	
FN1801	-0.248	9.652				20		26.0323	AAL93900.1| Sodium/glutamate symport carrier protein
					29		30.9146		
FN1803						6		7.8097	AAL93902.1| Transcriptional regulator, TetR family
						12		12.0000	
FN1804	0.435	9.539				18		23.4291	AAL93903.1| Aminoacyl-histidine dipeptidase
					22	40	23.4524	40.0000	
FN1807	-0.674	16.225	3.372e-3	3.296e-4	251	186	366.6095	242.1007	AAL93906.1| Hypothetical protein
					312	196	332.5980	196.0000	
FN1808	-0.712	6.642				6		7.8097	AAL93907.1| Hypothetical protein
					12		12.7922		
FN1809	-0.495	8.680	3.109e-1	5.889e-1	3	17	4.3818	22.1275	AAL93908.1| Iron/zinc/copper-binding protein
					41	12	43.7068	12.0000	
FN1811	-0.288	8.771	2.86e-1	5.229e-1	9	16	13.1454	20.8259	AAL93910.1| Manganese transport system ATP-binding protein mntA
					31	17	33.0466	17.0000	
FN1812	-1.699	12.344	1.422e-1	1.687e-1	148	30	216.1681	39.0485	AAL93911.1| Manganese-binding protein
					41	41	43.7068	41.0000	
FN1813	-0.343	6.485				6		7.8097	AAL93912.1| Manganese-binding protein
					10	9	10.6602	9.0000	
FN1814						8		10.4129	AAL93913.1| Hypothetical protein
FN1816	1.470	8.145			8		11.6848		AAL93915.1| unknown
					8	28	8.5282	28.0000	
FN1817	-0.105	5.275			3		4.3818		AAL93916.1| Hemolysin
					8	6	8.5282	6.0000	
FN1826	0.127	10.071	1.876e-1	2.815e-1	24	25	35.0543	32.5404	AAL93925.1| Protease
					26	36	27.7165	36.0000	
FN1827	1.020	12.239	6.817e-3	9.539e-4	26	83	37.9755	108.0342	AAL93926.1| Replicative DNA helicase
					56	90	59.6971	90.0000	

Log$_2$ Ratios: -6 -4 -2 0 2 4 6

Show detected proteins only
Show all proteins
Filter by category:
GO: amino acid transport

Proteins found: 1313

Enter (or paste) list of ORFs

Find ORFs

Test	Cutoff
q-Value	.005
p-Value	

	Signif	Direction	Applies To
red	yes	+	ratios, bars
yellow	no	n/a	bars
green	yes	−	ratios, bars
pink	yes	+	*p*- , *q*-Values
light green	yes	−	

Dot Plots Dot Plots

Protein	*FnSg* vs *FnPg* Log$_2$ Ratio	Log$_2$ Sum	*q*-Value	*p*-Value	Raw *FnPg*	Raw *FnSg*	Normalized *FnPg*	Normalized *FnSg*	Description
**FN1828**	-1.269	11.642	1.606e-1	2.157e-1	99	26	144.5990	33.8420	AAL93927.1| LSU ribosomal protein L9P
					29	39	30.9146	39.0000	
**FN1830**	0.512	11.292	1.423e-1	1.689e-1	18	55	26.2907	71.5889	AAL93929.1| DNA polymerase III subunits gamma and tau
					54	48	57.5650	48.0000	
**FN1831**	0.260	13.637	5.632e-2	2.828e-2	77	93	112.4659	121.0503	AAL93930.1| Nitrogen assimilation regulatory protein
					88	126	93.8097	126.0000	
**FN1832**						6		7.8097	AAL93931.1| TonB protein
**FN1833**									AAL93932.1| Biopolymer transport exbD protein
					17		18.1223		
**FN1834**	-0.241	9.508	1.259e-1	1.314e-1	19	22	27.7513	28.6356	AAL93933.1| Biopolymer transport exbB protein
					29	21	30.9146	21.0000	
**FN1836**	0.289	11.477	1.511e-1	1.931e-1	26	40	37.9755	52.0647	AAL93935.1| Tetratricopeptide repeat family protein
					55	66	58.6311	66.0000	
**FN1838**									AAL93937.1| Glycerol uptake facilitator protein
					32		34.1126		
**FN1839**	0.522	19.415	1.45e-1	1.761e-1	286	914	417.7303	1189.6775	AAL93938.1| Glycerol kinase
					917	814	977.5397	814.0000	
**FN1840**	-0.255	12.741	1.327e-1	1.457e-1	53	58	77.4116	75.4938	AAL93939.1| Dihydroxyacetone kinase
					97	76	103.4039	76.0000	
**FN1841**	-0.945	7.690	1.431e-1	1.711e-1	20	9	29.2119	11.7145	AAL93940.1| Dihydroxyacetone kinase
					10	9	10.6602	9.0000	
**FN1842**	0.171	9.552	3.228e-1	6.224e-1	12	17	17.5271	22.1275	AAL93941.1| Dihydroxyacetone kinase phosphotransfer protein
					32	36	34.1126	36.0000	
**FN1843**					3		4.3818		AAL93942.1| Surface antigen
**FN1844**	0.966	5.794				8		10.4129	AAL93943.1| Ketoacyl reductase hetN
					5		5.3301		
**FN1847**	0.414	8.493	2.27e-1	3.773e-1	5	16	7.3030	20.8259	AAL93946.1| DTDP-4-dehydrorhamnose 3,5-epimerase
					24	23	25.5845	23.0000	
**FN1848**									AAL93947.1| Metal dependent hydrolase
					5		5.3301		

Log$_2$ Ratios: -6 -4 -2 0 2 4 6

Show detected proteins only
Show all proteins
Filter by category:
GO: amino acid transport

Proteins found: 1313

Enter (or paste) list of ORFs

Find ORFs

Test	Cutoff
q-Value	.005
p-Value	

	Signif	Direction	Applies To
red	yes	+	ratios, bars
yellow	no	n/a	bars
green	yes	−	ratios, bars
pink	yes	+	*p*- , *q*-Values
light green	yes	−	

Dot Plots Dot Plots

Protein	*FnSg* vs *FnPg* Log$_2$ Ratio	Log$_2$ Sum	*q*-Value	*p*-Value	Raw *FnPg*	Raw *FnSg*	Normalized *FnPg*	Normalized *FnSg*	Description
**FN1849**	0.368	9.722				20		26.0323	AAL93948.1| Coenzyme F390 synthetase
					24	40	25.5845	40.0000	
**FN1850**	-0.055	11.300				25		32.5404	AAL93949.1| 3-oxoacyl-[acyl-carrier-protein] synthase III
					48	66	51.1689	66.0000	
**FN1851**	0.678	13.965	1.604e-1	2.152e-1	23	129	33.5937	167.9085	AAL93950.1| Ribonuclease PH
					156	152	166.2990	152.0000	
**FN1852**	-0.392	9.834	2.384e-1	4.069e-1	16	29	23.3695	37.7469	AAL93951.1| unknown
					43	15	45.8388	15.0000	
**FN1853**	-0.788	7.923	1.487e-1	1.865e-1	20	9	29.2119	11.7145	AAL93952.1| Methylaspartate mutase
					11	12	11.7262	12.0000	
**FN1854**	-0.123	10.528	3.29e-1	6.402e-1	33	22	48.1997	28.6356	AAL93953.1| Methylaspartate mutase
					30	45	31.9806	45.0000	
**FN1855**	0.517	4.702				4		5.2065	AAL93954.1| Methylaspartate mutase
					4	7	4.2641	7.0000	
**FN1856**	0.234	23.635	1.666e-1	2.3e-1	2099	2497	3065.7899	3250.1366	AAL93955.1| Butyrate-acetoacetate CoA-transferase subunit B
					3369	4579	3591.4189	4579.0000	
**FN1857**	0.178	20.954	2.462e-2	6.517e-3	897	1208	1310.1542	1572.3528	AAL93956.1| Acetoacetate: butyrate/acetate coenzyme A transferase
					1285	1459	1369.8348	1459.0000	
**FN1858**					30		43.8179		AAL93957.1| Short-chain fatty acids transporter
**FN1859**	1.191	25.873	1.008e-1	8.631e-2	443	11444	647.0438	14895.699	AAL93958.1| Major outer membrane protein
					9128	8794	9730.6240	8794.0000	
**FN1860**	-1.559	10.843				23		29.9372	AAL93959.1| NA+/H+ antiporter NHAC
					69	20	73.5553	20.0000	
**FN1862**	-1.125	14.812	9.372e-2	7.598e-2	232	82	338.8582	106.7326	AAL93961.1| L-beta-lysine 5,6-aminomutase beta subunit
					152	123	162.0349	123.0000	
**FN1863**	-1.845	17.556	1.401e-1	1.632e-1	957	183	1397.7899	238.1958	AAL93962.1| L-beta-lysine 5,6-aminomutase alpha subunit
					250	225	266.5048	225.0000	
**FN1864**	-1.469	6.256	1.605e-1	2.155e-1	17	5	24.8301	6.5081	AAL93963.1| DNA mismatch repair protein mutS
					4	4	4.2641	4.0000	
**FN1866**	-0.412	20.194	1.69e-1	2.359e-1	1117	722	1631.4852	939.7672	AAL93965.1| Lysine 2,3-aminomutase
					840	959	895.4562	959.0000	

Log$_2$ Ratios: -6 -4 -2 0 2 4 6

Show detected proteins only
Show all proteins
Filter by category:
GO: amino acid transport

Proteins found: 1313

Enter (or paste) list of ORFs

Find ORFs

Test	Cutoff
q-Value	.005
p-Value	

	Signif	Direction	Applies To
(red)	yes	+	ratios, bars
(yellow)	no	n/a	bars
(green)	yes	−	ratios, bars
(pink)	yes	+	*p*- , *q*-Values
(light green)	yes	−	

Dot Plots Dot Plots

Protein	*FnSg* vs *FnPg* Log$_2$ Ratio	Log$_2$ Sum	*q*-Value	*p*-Value	Raw *FnPg*	Raw *FnSg*	Normalized *FnPg*	Normalized *FnSg*	Description
**FN1867**	-1.540	18.656	8.989e-2	6.929e-2	1058	281	1545.3100	365.7543	AAL93966.1| Zn-dependent alcohol dehydrogenases and related dehydrogenases
					607	388	647.0737	388.0000	
**FN1868**	-0.572	13.089	1.043e-1	9.186e-2	96	60	140.2172	78.0970	AAL93967.1| Hypothetical cytosolic protein
					82	75	87.4136	75.0000	
**FN1869**	-1.008	14.117	9.304e-2	7.475e-2	172	63	251.2224	82.0018	AAL93968.1| Hypothetical protein
					119	106	126.8563	106.0000	
**FN1872**									AAL93971.1| unknown
						5		5.0000	
**FN1873**	-1.221	9.036	2.383e-3	1.633e-4	26	10	37.9755	13.0162	AAL93972.1| Bis(5'-nucleosyl)-tetraphosphatase
					30	17	31.9806	17.0000	
**FN1874**	-0.061	8.791	3.998e-1	8.696e-1	9	14	13.1454	18.2226	AAL93973.1| Ribose 5-phosphate isomerase
					28	23	29.8485	23.0000	
**FN1875**	0.544	14.919	2.095e-2	4.997e-3	112	159	163.5867	206.9570	AAL93974.1| Peptidyl-prolyl cis-trans isomerase
					120	218	127.9223	218.0000	
**FN1877**									AAL93976.1| Guanine-hypoxanthine permease
					6		6.3961		
**FN1880**	-0.560	14.219	3.269e-2	1.06e-2	118	101	172.3503	131.4633	AAL93979.1| Oxygen-insensitive NAD(P)H nitroreductase
					153	96	163.1009	96.0000	
**FN1881**	0.309	7.137				8		10.4129	AAL93980.1| Esterase
					10	16	10.6602	16.0000	
**FN1884**					32		46.7391		AAL93983.1| unknown
**FN1890**					22		32.1331		AAL93989.1| Hypothetical protein
**FN1891**	0.377	9.862	2.765e-1	4.99e-1	6	25	8.7636	32.5404	AAL93990.1| Glycerophosphoryl diester phosphodiesterase
					42	37	44.7728	37.0000	
**FN1893**	-0.312	17.983	1.168e-2	2.149e-3	389	336	568.1716	437.3432	AAL93991.1| Fusobacterium outer membrane protein family
					531	476	566.0562	476.0000	
**FN1898**	1.111	10.714	5.218e-2	2.468e-2	9	38	13.1454	49.4614	AAL93997.1| Sugar transport ATP-binding protein
					40	71	42.6408	71.0000	
**FN1899**	0.364	16.028	1.089e-1	9.967e-2	123	230	179.6532	299.3718	AAL93998.1| Hypothetical lipoprotein
					259	287	276.0990	287.0000	

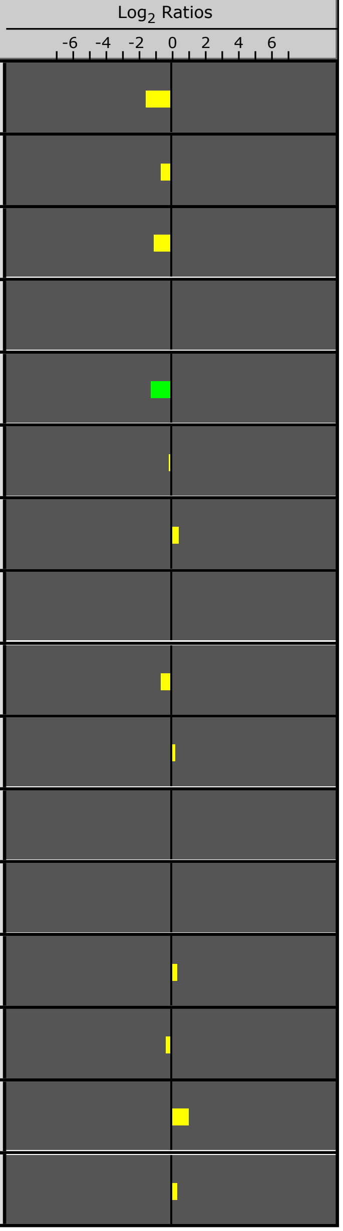

Show detected proteins only
Show all proteins
Filter by category:
GO: amino acid transport

Proteins found: 1313

Enter (or paste) list of ORFs

Find ORFs

Test	Cutoff
q-Value	.005
p-Value	

	Signif	Direction	Applies To
(red)	yes	+	ratios, bars
(yellow)	no	n/a	bars
(green)	yes	–	ratios, bars
(pink)	yes	+	*p*- , *q*-Values
(light green)	yes	–	*p*- , *q*-Values

Dot Plots Dot Plots

FnSg vs FnPg

Fusobacterium nucleatum

Hackett Laboratory UW

Fn Summary Table | FnPg vs Fn | FnSg vs Fn | FnPgSg vs Fn | FnPgSg vs FnPg | FnSg vs FnPg | FnPgSg vs FnSg | Fn Coverage

Protein	*FnSg* vs *FnPg* Log$_2$ Ratio	Log$_2$ Sum	*q*-Value	*p*-Value	Raw *FnPg*	Raw *FnSg*	Normalized *FnPg*	Normalized *FnSg*	Description
FN1901									AAL94000.1| Transcription regulator, CRP family
						8		8.0000	
FN1902	-0.516	9.741	6.493e-2	3.603e-2	26	23	37.9755	29.9372	AAL94001.1| Deoxycytidylate deaminase
					30	19	31.9806	19.0000	
FN1903					17		24.8301		AAL94002.1| Coenzyme A disulfide reductase/ disulfide bond regulator domain
					4		4.2641		
FN1905									AAL94004.1| outer membrane protein
						4		4.0000	
FN1906	0.312	18.247	2.136e-1	3.442e-1	222	408	324.2522	531.0596	AAL94005.1| Cytosol aminopeptidase
					635	712	676.9222	712.0000	
FN1908	0.161	17.125	1.713e-1	2.414e-1	213	330	311.1068	429.5335	AAL94007.1| Glycerophosphoryl diester phosphodiesterase
					379	370	404.0213	370.0000	
FN1909	-0.231	12.702	1.614e-1	2.176e-1	51	52	74.4904	67.6841	AAL94008.1| UDP-3-O-[3-hydroxymyristoyl] glucosamine N-acyltransferase
					96	83	102.3379	83.0000	
FN1910	-0.793	14.225	6.115e-2	3.244e-2	148	107	216.1681	139.2730	AAL94009.1| periplasmic protein
					139	71	148.1767	71.0000	
FN1911	-0.583	21.785	4.635e-2	1.968e-2	1822	1320	2661.2050	1718.1339	AAL94010.1| Outer membrane protein
					1869	1389	1992.3900	1389.0000	
FN1912	0.023	7.038	4.131e-1	9.189e-1	9	7	13.1454	9.1113	AAL94011.1| Hypothetical protein
					9	14	9.5942	14.0000	
FN1913	-0.024	12.108	4.204e-1	9.471e-1	64	49	93.4781	63.7792	AAL94012.1| hydrolase (HD superfamily)
					38	68	40.5087	68.0000	
FN1914	-0.943	13.875	2.323e-1	3.908e-1	213	69	311.1068	89.8115	AAL94013.1| Anti-sigma F factor antagonist
					27	87	28.7825	87.0000	
FN1917	1.025	6.379				10		13.0162	AAL94016.1| tRNA delta(2)-isopentenylpyrophosphate transferase
					6		6.3961		
FN1918	0.884	12.210	7.514e-2	4.748e-2	19	73	27.7513	95.0180	AAL94017.1| SPO0B-associated GTP-binding protein
					69	92	73.5553	92.0000	
FN1919	0.215	6.205	3.322e-1	6.495e-1	8	5	11.6848	6.5081	AAL94018.1| Methyltransferase
					4	12	4.2641	12.0000	
FN1920	-1.354	5.354			7		10.2242		AAL94019.1| tRNA (5-methylaminomethyl -2-thiouridylate) -methyltransferase
						4		4.0000	

Log$_2$ Ratios: -6 -4 -2 0 2 4 6

Show detected proteins only
Show all proteins
Filter by category:
GO: amino acid transport

Proteins found: 1313

Enter (or paste) list of ORFs

Find ORFs

Test	Cutoff
q-Value	.005
p-Value	

Signif	Direction	Applies To
yes	+	ratios, bars
no	n/a	bars
yes	–	ratios, bars
yes	+	*p*- , *q*-Values
yes	–	

Dot Plots Dot Plots

Protein	*FnSg* vs *FnPg* Log$_2$ Ratio	Log$_2$ Sum	*q*-Value	*p*-Value	Raw *FnPg*	Raw *FnSg*	Normalized *FnPg*	Normalized *FnSg*	Description
**FN1922**	-0.761	7.463	8.127e-6	2.336e-8	12	8	17.5271	10.4129	AAL94021.1| Hypothetical protein
					16	10	17.0563	10.0000	
**FN1923**									AAL94022.1| Adenine-specific methyltransferase
						3		3.0000	
**FN1925**									AAL94024.1| Arsenical pump membrane protein
					6		6.3961		
**FN1926**	-0.156	12.741	3.582e-1	7.286e-1	32	59	46.7391	76.7954	AAL94025.1| Nitrogen regulatory IIA protein
					120	80	127.9223	80.0000	
**FN1927**	0.955	16.177	5.265e-2	2.513e-2	78	272	113.9264	354.0397	AAL94026.1| DEGV protein
					260	404	277.1650	404.0000	
**FN1928**	-0.663	9.137				9		11.7145	AAL94027.1| Transcriptional regulator, MerR family
					28	26	29.8485	26.0000	
**FN1929**	0.847	11.866	1.253e-1	1.302e-1	12	46	17.5271	59.8744	AAL94028.1| Competence-damage protein cinA
					69	104	73.5553	104.0000	
**FN1931**	0.801	6.600							AAL94030.1| Protease
					7	13	7.4621	13.0000	
**FN1933**	0.522	6.291	2.27e-1	3.773e-1	5	4	7.3030	5.2065	AAL94032.1| Hypothetical protein
					7	16	7.4621	16.0000	
**FN1935**	0.477	5.832				6		7.8097	AAL94034.1| Adenine-specific methyltransferase
					6	10	6.3961	10.0000	
**FN1939**	-0.760	7.920				13		16.9210	AAL94038.1| Hypothetical protein
					19	7	20.2544	7.0000	
**FN1941**	0.513	17.330	7.283e-2	4.464e-2	239	314	349.0823	408.7076	AAL94040.1| ClpB protein
					310	561	330.4660	561.0000	
**FN1942**	-1.547	5.547			8		11.6848		AAL94041.1| putative DNA-binding protein
						4		4.0000	
**FN1943**	-0.408	24.213	8.632e-2	6.342e-2	4000	2836	5842.3820	3691.3846	AAL94042.1| Tryptophanase
					4049	3965	4316.3121	3965.0000	
**FN1948**	0.350	6.149				10		13.0162	AAL94044.1| Hypothetical protein
					7	6	7.4621	6.0000	
**FN1949**	0.180	11.924	3.175e-1	6.074e-1	24	52	35.0543	67.6841	AAL94045.1| Xaa-Pro dipeptidase
					77	65	82.0835	65.0000	

Log$_2$ Ratios: -6 -4 -2 0 2 4 6

Show detected proteins only
Show all proteins
Filter by category:
GO: amino acid transport

Proteins found: 1313

Enter (or paste) list of ORFs

Find ORFs

Test	Cutoff
q-Value	.005
p-Value	

Colour	Signif	Direction	Applies To
red	yes	+	ratios, bars
yellow	no	n/a	bars
green	yes	−	ratios, bars
pink	yes	+	*p*- , *q*-Values
light green	yes	−	

Dot Plots Dot Plots

Protein	FnSg vs FnPg Log2 Ratio	Log2 Sum	*q*-Value	*p*-Value	Raw *FnPg*	Raw *FnSg*	Normalized *FnPg*	Normalized *FnSg*	Description
FN1951									AAL94047.1| ATPase associated with chromosome architecture/replication
						4		4.0000	
FN1956	-0.298	7.700				10		13.0162	AAL94052.1| Hypothetical protein
					15	13	15.9903	13.0000	
FN1964	-0.516	8.101	3.728e-2	1.334e-2	14	9	20.4483	11.7145	AAL94054.1| O-linked GLCNAC transferase
					18	16	19.1883	16.0000	
FN1965	-0.338	11.256	1.657e-1	2.279e-1	44	43	64.2662	55.9695	AAL94055.1| Tetratricopeptide repeat family protein
					44	32	46.9048	32.0000	
FN1966	1.158	11.435	6.287e-3	8.25e-4	30	67	43.8179	87.2083	AAL94056.1| Hypothetical protein
					25	70	26.6505	70.0000	
FN1971	-2.347	8.257				5		6.5081	AAL94061.1| Hemin receptor
					37	9	39.4427	9.0000	
FN1972	-0.837	11.209				16		20.8259	AAL94062.1| unknown
					61	52	65.0272	52.0000	
FN1973	-1.255	13.137	3.793e-3	3.849e-4	111	56	162.1261	72.8905	AAL94063.1| Translation initiation inhibitor
					123	50	131.1204	50.0000	
FN1974									AAL94064.1| DNA/RNA helicase (DEAD/DEAH BOX family)
						7		7.0000	
FN1975	0.665	13.152	5.242e-2	2.491e-2	49	107	71.5692	139.2730	AAL94065.1| ATP-dependent RNA helicase
					75	101	79.9514	101.0000	
FN1976	1.211	11.021	6.886e-2	4.014e-2	6	62	8.7636	80.7002	AAL94066.1| 4-amino-4-deoxychorismate lyase
					48	58	51.1689	58.0000	
FN1977	-0.068	6.640			7	5	10.2242	6.5081	AAL94067.1| Cell cycle protein MesJ
						13		13.0000	
FN1978	0.577	12.567	1.734e-1	2.467e-1	18	64	26.2907	83.3035	AAL94068.1| Cell division protein ftsH
					95	107	101.2718	107.0000	
FN1979					15		21.9089		AAL94069.1| SSU ribosomal protein S15P
					7		7.4621		
FN1980						4		5.2065	AAL94070.1| Transporter
FN1983	-0.019	15.679	4.017e-1	8.765e-1	139	186	203.0228	242.1007	AAL94073.1| Alkyl hydroperoxide reductase C22 protein
					242	213	257.9767	213.0000	

Log2 Ratios: -6 -4 -2 0 2 4 6

Show detected proteins only
Show all proteins
Filter by category:
GO: amino acid transport

Proteins found: 1313

Enter (or paste) list of ORFs

Find ORFs

Test	Cutoff
q-Value	.005
p-Value	

Color	Signif	Direction	Applies To
red	yes	+	ratios, bars
yellow	no	n/a	bars
green	yes	−	ratios, bars
light red	yes	+	*p*- , *q*-Values
light green	yes	−	

Dot Plots Dot Plots

Protein	*FnSg* vs *FnPg* Log_2 Ratio	Log_2 Sum	*q*-Value	*p*-Value	Raw *FnPg*	Raw *FnSg*	Normalized *FnPg*	Normalized *FnSg*	Description	Log_2 Ratios
**FN1984**	-0.796	16.375	1.125e-1	1.059e-1	348	154	508.2872	200.4490	AAL94074.1| Thioredoxin reductase	
					244	242	260.1087	242.0000		
**FN1985**	0.335	12.626	3.078e-1	5.806e-1	13	65	18.9877	84.6051	AAL94075.1| Inner membrane protein	
					115	94	122.5922	94.0000		
**FN1986**	-0.445	16.160	1.209e-1	1.211e-1	255	222	372.4518	288.9589	AAL94076.1| Hypothetical protein	
					243	175	259.0427	175.0000		
**FN1987**	-2.421	7.591			22		32.1331		AAL94077.1| Transcriptional regulator, GntR family	
						6		6.0000		
**FN1988**	1.109	19.941	6.103e-2	3.232e-2	221	1391	322.7916	1810.5486	AAL94078.1| Tyrosine phenol-lyase	
					979	1137	1043.6329	1137.0000		
**FN1989**	-0.530	7.654				12		15.6194	AAL94079.1| Sodium-dependent tyrosine transporter	
					16	8	17.0563	8.0000		
**FN1991**	-0.317	17.369	3.052e-2	9.394e-3	334	260	487.8389	338.4203	AAL94081.1| Glucosamine-1-phosphate acetyltransferase	
					404	399	430.6718	399.0000		
**FN1992**	0.073	17.114	3.394e-1	6.708e-1	219	340	319.8704	442.5496	AAL94082.1| Ribose-phosphate pyrophosphokinase	
					389	330	414.6815	330.0000		
**FN1993**	-0.230	5.124				3		3.9048	AAL94083.1| SUA5 protein	
					6	7	6.3961	7.0000		
**FN1994**	-1.145	10.407	1.637e-1	2.231e-1	59	35	86.1751	45.5566	AAL94084.1| Hypothetical protein	
					22	4	23.4524	4.0000		
**FN1995**									AAL94085.1| Hypothetical protein	
					4		4.2641			
**FN2001**	-0.007	8.821				18		23.4291	AAL94091.1| Hypothetical protein	
					20	19	21.3204	19.0000		
**FN2002**									AAL94092.1| Permease	
						3		3.0000		
**FN2007**	-1.055	6.480	1.705e-1	2.394e-1	15	7	21.9089	9.1113	AAL94097.1| Glutathione peroxidase	
					5	4	5.3301	4.0000		
**FN2008**	-1.960	6.720				4		5.2065	AAL94098.1| Glycine betaine transport ATP-binding protein	
					19		20.2544			
**FN2009**	-0.109	6.075				6		7.8097	AAL94099.1| Glycine betaine transport system permease protein	
					8	8	8.5282	8.0000		

Log_2 Ratios axis: -6 -4 -2 0 2 4 6

Show detected proteins only
Show all proteins
Filter by category:
GO: amino acid transport

Proteins found: 1313

Enter (or paste) list of ORFs

Find ORFs

Test	Cutoff
q-Value	.005
p-Value	

	Signif	Direction	Applies To
red	yes	+	ratios, bars
yellow	no	n/a	bars
green	yes	−	ratios, bars
pink	yes	+	*p*- , *q*-Values
light green	yes	−	

Dot Plots Dot Plots

Protein	FnSg vs FnPg Log$_2$ Ratio	Log$_2$ Sum	*q*-Value	*p*-Value	Raw *FnPg*	Raw *FnSg*	Normalized *FnPg*	Normalized *FnSg*	Description
FN2011	0.571	13.430	1.852e-1	2.754e-1	37	60	54.0420	78.0970	AAL94101.1| Valyl-tRNA synthetase
					111	178	118.3281	178.0000	
FN2013	0.231	5.059				5		6.5081	AAL94103.1| GTP-binding protein
					5	6	5.3301	6.0000	
FN2014	0.278	14.270	7.903e-2	5.265e-2	77	128	112.4659	166.6069	AAL94104.1| ATP-dependent protease La
					134	143	142.8466	143.0000	
FN2015	0.357	13.338	5.169e-2	2.421e-2	64	97	93.4781	126.2568	AAL94105.1| ATP-dependent clp protease ATP-binding subunit clpX
					81	104	86.3476	104.0000	
FN2016	-0.927	8.974				15		19.5242	AAL94106.1| ATP-dependent Clp protease proteolytic subunit
					29	13	30.9146	13.0000	
FN2017	-0.681	17.893	1.044e-1	9.202e-2	543	273	793.1034	355.3413	AAL94107.1| Trigger factor, ppiase
					428	424	456.2563	424.0000	
FN2018	0.021	7.667	4.257e-1	9.677e-1	15	9	21.9089	11.7145	AAL94108.1| Single-stranded-DNA-specific exonuclease recJ
					6	17	6.3961	17.0000	
FN2019	-2.820	7.990			29		42.3573		AAL94109.1| Ribosome-binding factor A
						6		6.0000	
FN2020	0.271	16.342	1.647e-1	2.254e-1	190	196	277.5131	255.1168	AAL94110.1| Bacterial Protein Translation Initiation Factor 2 (IF-2)
					232	378	247.3165	378.0000	
FN2022	0.690	12.305	3.297e-3	3.129e-4	38	72	55.5026	93.7164	AAL94112.1| N utilization substance protein A
					53	87	56.4990	87.0000	
FN2023	0.044	8.265	4.195e-1	9.433e-1	20	12	29.2119	15.6194	AAL94113.1| Hypothetical cytosolic protein
					5	20	5.3301	20.0000	
FN2030	0.288	10.993	2.211e-1	3.625e-1	18	39	26.2907	50.7630	AAL94115.1| Inorganic pyrophosphatase
					52	49	55.4330	49.0000	
FN2031	-0.547	4.547			4		5.8424		AAL94116.1| Thiamine biosynthesis lipoprotein apbE
						4		4.0000	
FN2033	-0.753	8.154			15		21.9089		AAL94118.1| Guanylate kinase
						13		13.0000	
FN2034	-0.285	8.130	2.835e-1	5.167e-1	18	11	26.2907	14.3178	AAL94119.1| Protein yicC
					10	16	10.6602	16.0000	
FN2035	0.157	18.004	1.824e-1	2.683e-1	375	453	547.7233	589.6323	AAL94120.1| DNA-directed RNA polymerase beta' chain
					397	493	423.2097	493.0000	

Log$_2$ Ratios: -6 -4 -2 0 2 4 6

Show detected proteins only
Show all proteins
Filter by category:
GO: amino acid transport

Proteins found: 1313

Enter (or paste) list of ORFs

Find ORFs

Test	Cutoff
q-Value	.005
p-Value	

	Signif	Direction	Applies To
red	yes	+	ratios, bars
yellow	no	n/a	bars
green	yes	−	ratios, bars
pink	yes	+	*p*- , *q*-Values
light green	yes	−	

Dot Plots Dot Plots

Fusobacterium nucleatum

Hackett Laboratory UW

Protein	FnSg vs FnPg Log$_2$ Ratio	Log$_2$ Sum	*q*-Value	*p*-Value	Raw *FnPg*	Raw *FnSg*	Normalized *FnPg*	Normalized *FnSg*	Description
FN2036	0.508	17.024	6.91e-2	4.04e-2	163	318	238.0771	413.9141	AAL94121.1| DNA-directed RNA polymerase beta chain
					351	457	374.1728	457.0000	
FN2037	-0.812	19.372	1.645e-1	2.249e-1	1107	315	1616.8792	410.0092	AAL94122.1| LSU ribosomal protein L12P (L7/L12)
					531	833	566.0562	833.0000	
FN2038	-0.612	14.809	2.041e-1	3.221e-1	216	103	315.4886	134.0665	AAL94123.1| LSU ribosomal protein L10P
					97	140	103.4039	140.0000	
FN2039	-0.039	16.379	3.701e-1	7.671e-1	214	252	312.5674	328.0074	AAL94124.1| LSU ribosomal protein L1P
					262	248	279.2971	248.0000	
FN2040	-1.479	10.245	9.707e-2	8.072e-2	57	19	83.2539	24.7307	AAL94125.1| LSU ribosomal protein L11P
					31	17	33.0466	17.0000	
FN2041	-1.700	11.086	1.258e-1	1.312e-1	91	19	132.9142	24.7307	AAL94126.1| Transcription antitermination protein nusG
					33	27	35.1786	27.0000	
FN2045	-0.556	7.969				7		9.1113	AAL94129.1| Ferric uptake regulation protein
					18	17	19.1883	17.0000	
FN2046	-0.555	8.799				16		20.8259	AAL94130.1| Acetyltransferase
					24	14	25.5845	14.0000	
FN2047	0.301	13.685	1.497e-1	1.896e-1	54	92	78.8722	119.7487	AAL94131.1| Fusobacterium outer membrane protein family
					120	135	127.9223	135.0000	
FN2048	0.792	16.177				180		234.2910	AAL94132.1| Outer membrane protein
					194	482	206.8077	482.0000	
FN2049	-0.525	18.648	1.974e-1	3.071e-1	753	430	1099.8284	559.6951	AAL94133.1| unknown
					411	509	438.1339	509.0000	
FN2050	-2.135	13.336	1.251e-1	1.297e-1	240	40	350.5429	52.0647	AAL94134.1| Hypothetical membrane-spanning protein
					71	45	75.6874	45.0000	
FN2051	-0.406	12.967	2.74e-1	4.926e-1	106	81	154.8231	105.4309	AAL94135.1| unknown
					48	50	51.1689	50.0000	
FN2052	-0.190	12.637	3.647e-1	7.496e-1	89	81	129.9930	105.4309	AAL94136.1| unknown
					38	44	40.5087	44.0000	
FN2053	-0.362	11.892	2.323e-1	3.908e-1	30	49	43.8179	63.7792	AAL94137.1| Serine/threonine sodium symporter
					90	45	95.9417	45.0000	
FN2054	-0.452	14.142	1.419e-2	2.855e-3	100	96	146.0595	124.9552	AAL94138.1| Glucose-6-phosphate isomerase
					158	105	168.4310	105.0000	

Log$_2$ Ratios: -6 -4 -2 0 2 4 6

Show detected proteins only
Show all proteins
Filter by category:
GO: amino acid transport

Proteins found: 1313

Enter (or paste) list of ORFs

Find ORFs

Test	Cutoff
q-Value	.005
p-Value	

	Signif	Direction	Applies To
red	yes	+	ratios, bars
yellow	no	n/a	bars
green	yes	−	ratios, bars
pink	yes	+	*p*- , *q*-Values
light green	yes	−	

Dot Plots Dot Plots

Protein	FnSg vs FnPg Log$_2$ Ratio	Log$_2$ Sum	*q*-Value	*p*-Value	Raw FnPg	Raw FnSg	Normalized FnPg	Normalized FnSg	Description
FN2058	-1.728	17.134	9.199e-2	7.287e-2	683	155	997.5867	201.7506	AAL94142.1| Fusobacterium outer membrane protein family
					359	215	382.7009	215.0000	
FN2059	0.792	16.177				180		234.2910	AAL94143.1| Outer membrane protein
					194	482	206.8077	482.0000	
FN2060	-0.525	18.648	1.974e-1	3.071e-1	753	430	1099.8284	559.6951	AAL94144.1| unknown
					411	509	438.1339	509.0000	
FN2061	-2.135	13.336	1.251e-1	1.297e-1	240	40	350.5429	52.0647	AAL94145.1| Hypothetical membrane-spanning protein
					71	45	75.6874	45.0000	
FN2062	-0.406	12.967	2.74e-1	4.926e-1	106	81	154.8231	105.4309	AAL94146.1| unknown
					48	50	51.1689	50.0000	
FN2063	-0.190	12.637	3.647e-1	7.496e-1	89	81	129.9930	105.4309	AAL94147.1| unknown
					38	44	40.5087	44.0000	
FN2067									AAL94151.1| Thiol:disulfide interchange protein tlpA
						6		6.0000	
FN2068	-0.139	8.791	3.744e-1	7.812e-1	12	7	17.5271	9.1113	AAL94152.1| dGTP triphosphohydrolase
					25	31	26.6505	31.0000	
FN2070	0.150	7.253				10		13.0162	AAL94154.1| Cobyric acid synthase
					11	13	11.7262	13.0000	
FN2073	1.261	11.058	5.149e-2	2.403e-2	8	43	11.6848	55.9695	AAL94157.1| Adenine phosphoribosyltransferase
					45	87	47.9709	87.0000	
FN2074									AAL94158.1| BslIM
					3		3.1981		
FN2075	-0.222	8.673	3.026e-1	5.664e-1	16	8	23.3695	10.4129	AAL94159.1| Hypothetical protein
					19	27	20.2544	27.0000	
FN2078	1.323	4.677							AAL94162.1| Transcriptional regulator, DeoR family
					3	8	3.1981	8.0000	
FN2082	0.129	21.217	3.573e-1	7.257e-1	599	1156	874.8967	1504.6687	AAL94166.1| Formate--tetrahydrofolate ligase
					1981	1761	2111.7842	1761.0000	
FN2093	-1.092	8.699	1.561e-1	2.051e-1	32	13	46.7391	16.9210	AAL94177.1| General secretion pathway protein G
					12	11	12.7922	11.0000	
FN2098	0.662	6.272	9.451e-2	7.707e-2	3	7	4.3818	9.1113	AAL94182.1| MRP-family nucleotide-binding protein
					9	13	9.5942	13.0000	

Log$_2$ Ratios: -6 -4 -2 0 2 4 6

Show detected proteins only
Show all proteins
Filter by category:
GO: amino acid transport

Proteins found: 1313

Enter (or paste) list of ORFs

Find ORFs

Test	Cutoff
q-Value	.005
p-Value	

	Signif	Direction	Applies To
	yes	+	ratios, bars
	no	n/a	bars
	yes	−	ratios, bars
	yes	+	*p*- , *q*-Values
	yes	−	

Dot Plots Dot Plots

Protein	*FnSg* vs *FnPg* Log$_2$ Ratio	Log$_2$ Sum	*q*-Value	*p*-Value	Raw *FnPg*	Raw *FnSg*	Normalized *FnPg*	Normalized *FnSg*	Description
**FN2100**	-0.254	9.886	1.564e-1	2.059e-1	19	21	27.7513	27.3339	AAL94184.1| Hypothetical protein
					37	29	39.4427	29.0000	
**FN2102**	0.540	8.807	1.079e-1	9.792e-2	16	20	23.3695	26.0323	AAL94186.1| ABC transporter ATP-binding protein
					11	25	11.7262	25.0000	
**FN2103**	0.094	19.117	3.212e-1	6.177e-1	598	651	873.4361	847.3524	AAL94187.1| tricarboxylate-binding protein
					550	711	586.3106	711.0000	
**FN2105**									AAL94189.1| tricarboxylate transport membrane protein RctA
					7		7.4621		
**FN2106**	-1.009	14.761	1.525e-1	1.965e-1	247	96	360.7671	124.9552	AAL94190.1| Transporter
					105	110	111.9320	110.0000	
**FN2107**	-1.441	8.730	1.394e-1	1.617e-1	37	10	54.0420	13.0162	AAL94191.1| Galactokinase
					13	12	13.8583	12.0000	
**FN2108**	-0.407	8.889	4.741e-2	2.051e-2	19	16	27.7513	20.8259	AAL94192.1| Galactose-1-phosphate uridylyltransferase
					21	17	22.3864	17.0000	
**FN2109**	-0.530	12.060	7.629e-2	4.895e-2	63	39	92.0175	50.7630	AAL94193.1| UDP-glucose 4-epimerase
					61	58	65.0272	58.0000	
**FN2116**	-1.156	8.316				13		16.9210	AAL94200.1| Hypothetical exported 24-amino acid repeat protein
					25	7	26.6505	7.0000	
**FN2117**	0.493	5.847							AAL94201.1| Hypothetical exported 24-amino acid repeat protein
					6	9	6.3961	9.0000	
**FN2118**	-0.315	5.485							AAL94202.1| Hypothetical exported 24-amino acid repeat protein
					7	6	7.4621	6.0000	
**FN2119**	-1.170	9.511				10		13.0162	AAL94203.1| Hypothetical exported 24-amino acid repeat protein
					38	23	40.5087	23.0000	
**FN2121**	-2.588	9.992			94	10	137.2960	13.0162	AAL94205.1| Hypothetical exported 24-amino acid repeat protein
					18		19.1883		
**FN2122**	-0.355	17.257	1.096e-1	1.008e-1	356	275	519.9720	357.9446	AAL94206.1| Phenylalanyl-tRNA synthetase beta chain
					352	342	375.2388	342.0000	
**FN2123**	0.160	13.580	3.568e-1	7.243e-1	39	66	56.9632	85.9067	AAL94207.1| Phenylalanyl-tRNA synthetase alpha chain
					143	148	152.4408	148.0000	
**FN2125**	-0.254	12.696	3.099e-1	5.864e-1	89	57	129.9930	74.1921	AAL94209.1| DNA gyrase subunit A
					45	75	47.9709	75.0000	

Log$_2$ Ratios: -6 -4 -2 0 2 4 6

Show detected proteins only
Show all proteins
Filter by category: GO: amino acid transport

Proteins found: 1313

Enter (or paste) list of ORFs

Find ORFs

Test	Cutoff
q-Value	.005
p-Value	

Signif	Direction	Applies To
yes	+	ratios, bars
no	n/a	bars
yes	−	ratios, bars
yes	+	*p*-, *q*-Values
yes	−	

Dot Plots Dot Plots

Protein	*FnSg* vs *FnPg* Log$_2$ Ratio	Log$_2$ Sum	*q*-Value	*p*-Value	Raw *FnPg*	Raw *FnSg*	Normalized *FnPg*	Normalized *FnSg*	Description	Log$_2$ Ratios (-6 -4 -2 0 2 4 6)
FN2126	-0.272	9.756	2.109e-1	3.38e-1	26	15	37.9755	19.5242	AAL94210.1| DNA gyrase subunit B	
					25	34	26.6505	34.0000		

Show detected proteins only
Show all proteins
Filter by category:
GO: amino acid transport

Proteins found: 1313

Enter (or paste) list of ORFs

Find ORFs

Test	Cutoff
q-Value	.005
p-Value	

	Signif	Direction	Applies To
red	yes	+	ratios, bars
yellow	no	n/a	bars
green	yes	−	ratios, bars
pink	yes	+	*p*- , *q*-Values
light green	yes	−	*p*- , *q*-Values

Dot Plots Dot Plots
