# Supplementary material for: Proteomics of Fusobacterium nucleatum within a model developing oral microbial community
Source: Microbiologyopen. 2014 Aug 25;3(5):729–51. doi: 10.1002/mbo3.204 (PMC4234264; doi:10.1002/mbo3.204)
Supplement: Table S6 — See description for Table S3. [file mbo30003-0729-sd8.pdf]

Protein	*FnPgSg vs FnSg* Log$_2$ Ratio	Log$_2$ Sum	*q*-Value	*p*-Value	Raw *FnPgSg*	Raw *FnSg*	Normalized *FnPgSg*	Normalized *FnSg*	Description
**FN0001**	-1.275	6.541	7.573e-4	1.334e-3	7	10	8.0083	13.0162	AAL94214.1| Chromosomal replication initiator protein dnaA
					3	17	4.3974	17.0000	
**FN0004**	1.784	12.338	4.439e-3	1.286e-2	145	35	165.8869	45.5566	AAL94217.1| Inner membrane protein
					69	32	101.1401	32.0000	
**FN0005**	1.595	10.422	8.613e-5	7.124e-5	60	12	68.6428	15.6194	AAL94218.1| Jag protein
					41	27	60.0977	27.0000	
**FN0006**	-1.186	8.562	7.973e-6	1.958e-6	11	22	12.5845	28.6356	AAL94219.1| Thiophene and furan oxidation protein THDF
					9	30	13.1922	30.0000	
**FN0007**	-0.696	7.157	2.992e-2	1.242e-1	10	8	11.4405	10.4129	AAL94220.1| Glucose inhibited division protein A
					5	20	7.3290	20.0000	
**FN0008**						6		7.8097	AAL94221.1| Quinolinate synthetase A
						11		11.0000	
**FN0009**									AAL94222.1| L-aspartate oxidase
						25		25.0000	
**FN0017**						4		5.2065	AAL94230.1| Hypothetical protein
						7		7.0000	
**FN0018**	0.749	9.945	1.226e-4	1.125e-4	34	18	38.8976	23.4291	AAL94231.1| Hypothetical protein
					29	25	42.5082	25.0000	
**FN0019**	-0.010	5.114				6		7.8097	AAL94232.1| Transcription-repair coupling factor
					4	4	5.8632	4.0000	
**FN0022**	1.713	9.048	4.072e-3	1.141e-2	28	8	32.0333	10.4129	AAL94235.1| Hypothetical protein
					35	15	51.3030	15.0000	
**FN0023**	-0.033	7.781							AAL94236.1| Short-chain fatty acids transporter
					10	15	14.6580	15.0000	
**FN0024**	0.788	9.128			30		34.3214		AAL94237.1| Hypothetical exported 24-amino acid repeat protein
					19	18	27.8502	18.0000	
**FN0025**	0.359	6.359							AAL94238.1| Hypothetical exported 24-amino acid repeat protein
					7	8	10.2606	8.0000	
**FN0026**					40		45.7619		AAL94239.1| Hypothetical exported 24-amino acid repeat protein
					20		29.3160		
**FN0029**					5		5.7202		AAL94242.1| Flavodoxin

Log$_2$ Ratios: -6 -4 -2 0 2 4 6

Show detected proteins only
Show all proteins
Filter by category:
GO: amino acid transport

Proteins found: 1344

Enter (or paste) list of ORFs

Find ORFs

Test	Cutoff
q-Value	.005
p-Value	

	Signif	Direction	Applies To
red	yes	+	ratios, bars
yellow	no	n/a	bars
green	yes	−	ratios, bars
pink	yes	+	*p*- , *q*-Values
light green	yes	−	

Dot Plots Dot Plots

Protein	*FnPgSg* vs *FnSg* Log$_2$ Ratio	Log$_2$ Sum	*q*-Value	*p*-Value	Raw *FnPgSg*	Raw *FnSg*	Normalized *FnPgSg*	Normalized *FnSg*	Description
**FN0030**	-0.799	12.726	5.533e-4	8.615e-4	63	90	72.0750	117.1455	AAL94243.1| 5-nitroimidazole antibiotic resistance protein
					36	100	52.7687	100.0000	
**FN0031**	-1.142	8.147	1.66e-3	3.674e-3	7	20	8.0083	26.0323	AAL94244.1| unknown
					10	24	14.6580	24.0000	
**FN0033**	-0.633	11.958	1.07e-6	1.34e-7	45	60	51.4821	78.0970	AAL94246.1| unknown
					34	79	49.8372	79.0000	
**FN0034**	1.319	9.755	6.41e-4	1.067e-3	44	14	50.3381	18.2226	AAL94247.1| unknown
					29	19	42.5082	19.0000	
**FN0039**						13		16.9210	AAL94252.1| DNA primase (bacterial type) and small primase-like proteins
						16		16.0000	
**FN0040**	-0.539	18.495	1.488e-5	5.633e-6	424	549	485.0761	714.5875	AAL94253.1| Asparaginyl-tRNA synthetase
					357	751	523.2901	751.0000	
**FN0041**					4		4.5762		AAL94254.1| unknown
					8		11.7264		
**FN0043**					4		4.5762		AAL94256.1| Hypothetical exported 24-amino acid repeat protein
**FN0045**									AAL94258.1| Shikimate 5-dehydrogenase
						7		7.0000	
**FN0046**									AAL94259.1| 3-dehydroquinate dehydratase
					8		11.7264		
**FN0047**	0.298	12.263	7.296e-5	5.701e-5	68	48	77.7952	62.4776	AAL94260.1| Exodeoxyribonuclease III
					53	64	77.6873	64.0000	
**FN0048**	-0.470	8.802	1.982e-5	9.147e-6	16	19	18.3048	24.7307	AAL94261.1| 4-nitrophenylphosphatase
					12	25	17.5896	25.0000	
**FN0049**						40		52.0647	AAL94262.1| Hypothetical protein
						67		67.0000	
**FN0050**	2.598	18.078	2.221e-3	5.272e-3	906	151	1036.5069	196.5441	AAL94263.1| Fumarate reductase flavoprotein subunit
					1059	231	1552.2807	231.0000	
**FN0052**					21		24.0250		AAL94265.1| Arsenate reductase
					21		30.7818		
**FN0054**	0.484	15.149	1.567e-4	1.577e-4	193	124	220.8011	161.4005	AAL94267.1| Tyrosyl-tRNA synthetase
					157	161	230.1304	161.0000	

Log$_2$ Ratios: -6 -4 -2 0 2 4 6

Show detected proteins only
Show all proteins
Filter by category:
GO: amino acid transport

Proteins found: 1344

Enter (or paste) list of ORFs

Find ORFs

Test	Cutoff
q-Value	.005
p-Value	

Color	Signif	Direction	Applies To
red	yes	+	ratios, bars
yellow	no	n/a	bars
green	yes	−	ratios, bars
light red	yes	+	*p*- , *q*-Values
light green	yes	−	

Dot Plots Dot Plots

Protein	*FnPgSg vs FnSg* Log_2 Ratio	Log_2 Sum	*q*-Value	*p*-Value	Raw *FnPgSg*	Raw *FnSg*	Normalized *FnPgSg*	Normalized *FnSg*	Description
**FN0058**	-1.200	14.906	2.644e-2	1.08e-1	106	116	121.2690	150.9875	AAL94271.1| Cysteine desulfhydrase
					75	380	109.9349	380.0000	
**FN0059**	-0.055	11.061	1.66e-1	8.61e-1	37	24	42.3298	31.2388	AAL94272.1| NifU protein
					33	63	48.3714	63.0000	
**FN0060**	1.643	7.258			19		21.7369		AAL94273.1| D-alanyl-D-alanine carboxypeptidase
					15	7	21.9870	7.0000	
**FN0061**	0.012	12.899	1.725e-1	9.02e-1	74	60	84.6595	78.0970	AAL94274.1| Thermostable carboxypeptidase 1
					62	96	90.8795	96.0000	
**FN0062**					13		14.8726		AAL94275.1| Hypothetical cytosolic protein
					11		16.1238		
**FN0063**									AAL94276.1| unknown
						24		24.0000	
**FN0065**	0.141	13.499	7.609e-2	3.562e-1	95	66	108.6845	85.9067	AAL94278.1| Transcription accessory protein (S1 RNA binding domain)
					80	119	117.2639	119.0000	
**FN0066**									AAL94279.1| Two component system histidine kinase
						5		5.0000	
**FN0067**	0.746	16.538	2.253e-5	1.099e-5	341	180	390.1202	234.2910	AAL94280.1| Isoleucyl-tRNA synthetase
					279	242	408.9578	242.0000	
**FN0069**	-0.066	15.009	1.227e-1	6.048e-1	163	161	186.4797	209.5603	AAL94282.1| Glycyl-tRNA synthetase alpha chain
					115	162	168.5668	162.0000	
**FN0070**	-0.528	17.629	5.561e-4	8.676e-4	326	432	372.9594	562.2984	AAL94283.1| Glycyl-tRNA synthetase beta chain
					257	519	376.7102	519.0000	
**FN0071**						7		9.1113	AAL94284.1| GTP cyclohydrolase I
**FN0072**	-1.103	13.105	8.499e-5	6.994e-5	53	100	60.6345	130.1617	AAL94285.1| 2-amino-4-hydroxy-6-hydroxymethyldihydropteridine pyrophosphokinase
					46	145	67.4267	145.0000	
**FN0073**	-0.496	9.342	5.559e-2	2.512e-1	17	15	19.4488	19.5242	AAL94286.1| Dihydropteroate synthase
					16	41	23.4528	41.0000	
**FN0074**	-0.476	7.784			11	10	12.5845	13.0162	AAL94287.1| Ethanolamine utilization protein eutS
						22		22.0000	
**FN0077**									AAL94290.1| Ethanolamine two-component sensor kinase
						13		13.0000	

Log_2 Ratios: -6 -4 -2 0 2 4 6

Show detected proteins only
Show all proteins
Filter by category:
GO: amino acid transport

Proteins found: 1344

Enter (or paste) list of ORFs

Find ORFs

Test	Cutoff
q-Value	.005
p-Value	

	Signif	Direction	Applies To
(red)	yes	+	ratios, bars
(yellow)	no	n/a	bars
(green)	yes	–	ratios, bars
(pink)	yes	+	*p*- , *q*-Values
(light green)	yes	–	

Dot Plots Dot Plots

Protein	*FnPgSg vs FnSg* Log$_2$ Ratio	Log$_2$ Sum	*q*-Value	*p*-Value	Raw *FnPgSg*	Raw *FnSg*	Normalized *FnPgSg*	Normalized *FnSg*	Description
**FN0078**	-1.646	7.365	7.87e-5	6.302e-5	5	18	5.7202	23.4291	AAL94291.1| Ethanolamine utilization protein eutA
					6	22	8.7948	22.0000	
**FN0079**	-2.629	12.764	5.808e-5	4.252e-5	33	169	37.7536	219.9732	AAL94292.1| Ethanolamine ammonia-lyase heavy chain
					20	195	29.3160	195.0000	
**FN0080**	-1.112	13.153	5.42e-4	8.365e-4	52	118	59.4905	153.5908	AAL94293.1| Ethanolamine ammonia-lyase light chain
					48	127	70.3583	127.0000	
**FN0081**	-2.964	13.242	1.679e-3	3.728e-3	27	172	30.8893	223.8780	AAL94294.1| Ethanolamine utilization protein eutL
					27	326	39.5766	326.0000	
**FN0082**	-0.809	9.763	2.699e-2	1.106e-1	21	20	24.0250	26.0323	AAL94295.1| Ethanolamine utilization protein eutM
					14	52	20.5212	52.0000	
**FN0083**	-1.643	14.280	1.247e-3	2.574e-3	78	164	89.2357	213.4651	AAL94296.1| Ethanolamine utilization protein eutM precursor
					48	285	70.3583	285.0000	
**FN0084**	-0.719	9.798	4.306e-3	1.232e-2	24	25	27.4571	32.5404	AAL94297.1| Acetaldehyde dehydrogenase (acetylating)
					13	44	19.0554	44.0000	
**FN0087**									AAL94300.1| Ethanolamine utilization protein eutN
						5		5.0000	
**FN0088**									AAL94301.1| Hypothetical protein
						9		9.0000	
**FN0089**					4		4.5762		AAL94302.1| Ethanolamine permease
**FN0090**						7		9.1113	AAL94303.1| Ethanolamine utilization protein eutQ
						4		4.0000	
**FN0092**	-2.611	9.423	1.505e-6	2e-7	7	48	8.0083	62.4776	AAL94305.1| NADPH-dependent butanol dehydrogenase
					9	67	13.1922	67.0000	
**FN0093**	1.871	15.159	1.909e-5	8.535e-6	305	83	348.9344	108.0342	AAL94306.1| Thioredoxin
					261	92	382.5734	92.0000	
**FN0100**	1.100	8.579	1.371e-4	1.308e-4	27	9	30.8893	11.7145	AAL94309.1| Flavodoxins/hemoproteins
					18	15	26.3844	15.0000	
**FN0102**					5		5.7202		AAL94311.1| Ribonucleoside-diphosphate reductase alpha chain
					8		11.7264		
**FN0103**					4		4.5762		AAL94312.1| Ribonucleoside-diphosphate reductase beta chain

Log$_2$ Ratios: -6 -4 -2 0 2 4 6

Show detected proteins only
Show all proteins
Filter by category:
GO: amino acid transport

Proteins found: 1344

Enter (or paste) list of ORFs

Find ORFs

Test	Cutoff
q-Value	.005
p-Value	

	Signif	Direction	Applies To
red	yes	+	ratios, bars
yellow	no	n/a	bars
green	yes	−	ratios, bars
pink	yes	+	*p*- , *q*-Values
light green	yes	−	

Dot Plots Dot Plots

FnPgSg vs FnSg

Fusobacterium nucleatum

Hackett Laboratory | UW

Fn Summary Table | FnPg vs Fn | FnSg vs Fn | FnPgSg vs Fn | FnPgSg vs FnPg | FnSg vs FnPg | FnPgSg vs FnSg | Fn Coverage

Protein	*FnPgSg* vs *FnSg* Log$_2$ Ratio	Log$_2$ Sum	*q*-Value	*p*-Value	Raw *FnPgSg*	Raw *FnSg*	Normalized *FnPgSg*	Normalized *FnSg*	Description
**FN0106**	0.044	13.655	1.123e-1	5.472e-1	94	82	107.5405	106.7326	AAL94315.1| Hypothetical protein
					84	117	123.1271	117.0000	
**FN0108**	-1.384	8.112			9	19	10.2964	24.7307	AAL94317.1| Microcin C7 self-immunity protein mccF
						29		29.0000	
**FN0110**	0.054	17.418	1.069e-1	5.177e-1	342	303	391.2642	394.3898	AAL94319.1| Seryl-tRNA synthetase
					315	427	461.7266	427.0000	
**FN0113**	-3.118	11.409	3.382e-6	5.898e-7	13	114	14.8726	148.3843	AAL94322.1| Heat-inducible transcription repressor hrcA
					14	159	20.5212	159.0000	
**FN0114**	0.384	14.400	3.79e-2	1.627e-1	163	71	186.4797	92.4148	AAL94323.1| GrpE protein
					102	165	149.5115	165.0000	
**FN0116**	-0.780	20.992	3.583e-2	1.525e-1	1009	902	1154.3438	1174.0581	AAL94325.1| Chaperone protein dnaK
					716	2611	1049.5118	2611.0000	
**FN0117**						10		13.0162	AAL94326.1| O6-methylguanine-DNA methyltransferase
						32		32.0000	
**FN0118**	0.135	12.562	4.615e-2	2.036e-1	72	51	82.3714	66.3824	AAL94327.1| Chaperone protein dnaJ
					55	82	80.6189	82.0000	
**FN0119**						29		37.7469	AAL94328.1| Flavodoxin
						81		81.0000	
**FN0123**						6		7.8097	AAL94332.1| ATPase
						7		7.0000	
**FN0127**						5		6.5081	AAL94333.1| Fe-S oxidoreductase
						7		7.0000	
**FN0128**	-0.652	7.616	6.216e-2	2.842e-1	8	7	9.1524	9.1113	AAL94334.1| Spermidine/putrescine-binding protein
					9	26	13.1922	26.0000	
**FN0130**	-0.066	8.307	1.747e-1	9.157e-1	24	8	27.4571	10.4129	AAL94336.1| ABC transporter ATP-binding protein
					5	26	7.3290	26.0000	
**FN0132**	0.908	6.078			12		13.7286		AAL93916.1| Hemolysin
					6	6	8.7948	6.0000	
**FN0147**	-0.184	9.822	2.855e-2	1.178e-1	25	27	28.6012	35.1436	AAL94353.1| PLSX protein
					19	29	27.8502	29.0000	
**FN0148**	0.149	16.131	1.1e-1	5.344e-1	242	248	276.8595	322.8009	AAL94354.1| 3-oxoacyl-[acyl-carrier-protein] synthase III
					196	186	287.2965	186.0000	

Log$_2$ Ratios: -6 -4 -2 0 2 4 6

Show detected proteins only
Show all proteins
Filter by category:
GO: amino acid transport

Proteins found: 1344

Enter (or paste) list of ORFs

Find ORFs

Test	Cutoff
q-Value	.005
p-Value	

Signif	Direction	Applies To
yes	+	ratios, bars
no	n/a	bars
yes	−	ratios, bars
yes	+	*p*- , *q*-Values
yes	−	

Dot Plots | Dot Plots

Protein	*FnPgSg vs FnSg* Log$_2$ Ratio	Log$_2$ Sum	*q*-Value	*p*-Value	Raw *FnPgSg*	Raw *FnSg*	Normalized *FnPgSg*	Normalized *FnSg*	Description
**FN0149**	1.089	14.935	1.855e-3	4.21e-3	190	115	217.3690	149.6859	AAL94355.1| Malonyl-CoA-[acyl-carrier-protein] transacylase
					204	93	299.0229	93.0000	
**FN0150**	0.873	11.317	2.254e-4	2.589e-4	58	32	66.3547	41.6517	AAL94356.1| Acyl carrier protein
					48	33	70.3583	33.0000	
**FN0151**	0.645	19.562	9.456e-3	3.276e-2	893	389	1021.6343	506.3288	AAL94357.1| 3-oxoacyl-[acyl-carrier-protein] synthase
					804	901	1178.5021	901.0000	
**FN0152**	-0.126	5.874							AAL94358.1| Ribonuclease III
					5	8	7.3290	8.0000	
**FN0153**									AAL94359.1| Oxygen-independent coproporphyrinogen III oxidase
						13		13.0000	
**FN0154**									AAL94360.1| Ribonuclease G
					5		7.3290		
**FN0155**	1.151	5.795			13		14.8726		AAL94361.1| Hypothetical protein
					5	5	7.3290	5.0000	
**FN0156**	0.503	8.139	7.204e-2	3.354e-1	17	4	19.4488	5.2065	AAL94362.1| Phosphopantetheine adenylyltransferase
					14	23	20.5212	23.0000	
**FN0157**						6		7.8097	AAL94363.1| DNA repair protein RadA
						7		7.0000	
**FN0158**	-1.353	9.813	4.684e-4	6.842e-4	20	36	22.8809	46.8582	AAL94364.1| DNA-binding protein
					10	49	14.6580	49.0000	
**FN0161**									AAL94367.1| RNA-directed DNA polymerase
						3		3.0000	
**FN0164**	-1.058	15.276	1.113e-2	3.991e-2	117	278	133.8535	361.8494	AAL94370.1| Anhydro-N-acetylmuramyl-tripeptide amidase
					97	213	142.1825	213.0000	
**FN0170**	-0.569	14.645	1.633e-2	6.229e-2	117	179	133.8535	232.9894	AAL94376.1| GTP-binding protein
					88	157	128.9903	157.0000	
**FN0173**	0.266	12.069	4.161e-2	1.811e-1	68	55	77.7952	71.5889	AAL94379.1| Hypothetical protein
					45	48	65.9609	48.0000	
**FN0174**	0.943	13.772	4.162e-3	1.176e-2	165	65	188.7678	84.6051	AAL94380.1| Enoyl-[acyl-carrier-protein] reductase
					95	86	139.2509	86.0000	
**FN0175**	-1.121	9.143				27		35.1436	AAL94381.1| Cell division inhibitor MinC
					11	35	16.1238	35.0000	

Log$_2$ Ratios: -6 -4 -2 0 2 4 6

Show detected proteins only
Show all proteins
Filter by category:
GO: amino acid transport

Proteins found: 1344

Enter (or paste) list of ORFs

Find ORFs

Test	Cutoff
q-Value	.005
p-Value	

	Signif	Direction	Applies To
red	yes	+	ratios, bars
yellow	no	n/a	bars
green	yes	−	ratios, bars
pink	yes	+	*p*- , *q*-Values
light green	yes	−	

Dot Plots Dot Plots

Protein	FnPgSg vs FnSg Log$_2$ Ratio	Log$_2$ Sum	*q*-Value	*p*-Value	Raw *FnPgSg*	Raw *FnSg*	Normalized *FnPgSg*	Normalized *FnSg*	Description
FN0176	-0.576	16.192	7.056e-4	1.212e-3	174	272	199.0642	354.0397	AAL94382.1| Cell division inhibitor MinD
					170	314	249.1858	314.0000	
FN0177					11		12.5845		AAL94383.1| Cell division inhibitor MinE
					11		16.1238		
FN0178	0.546	10.888	1.39e-3	2.937e-3	42	30	48.0500	39.0485	AAL94384.1| UNC-44 ankyrins
					39	33	57.1661	33.0000	
FN0179	-0.260	9.545	9.701e-2	4.647e-1	27	16	30.8893	20.8259	AAL94385.1| Ankyrin repeat proteins
					13	39	19.0554	39.0000	
FN0180	0.708	5.680			8	4	9.1524	5.2065	AAL94386.1| Tetratricopeptide repeat family protein
						6		6.0000	
FN0181	-1.174	9.065	1.618e-3	3.554e-3	9	25	10.2964	32.5404	AAL94387.1| Hypothetical protein
					14	37	20.5212	37.0000	
FN0182	-3.119	15.402	1.447e-5	5.349e-6	76	474	86.9476	616.9663	AAL94388.1| Sarcosine oxidase alpha subunit
					37	610	54.2345	610.0000	
FN0183	-1.301	15.772	4.587e-5	3.095e-5	152	274	173.8952	356.6429	AAL94389.1| Glycerol-3-phosphate dehydrogenase
					87	386	127.5245	386.0000	
FN0185	-0.787	7.895	3.582e-4	4.845e-4	9	15	10.2964	19.5242	AAL94391.1| Hypothetical protein
					9	21	13.1922	21.0000	
FN0189	-0.773	6.802	4.787e-3	1.42e-2	9	12	10.2964	15.6194	AAL94395.1| Two-component response regulator yesN
					4	12	5.8632	12.0000	
FN0190						5		6.5081	AAL94396.1| Two-component sensor kinase yesM
						7		7.0000	
FN0191	-1.562	8.917	2.196e-3	5.195e-3	7	35	8.0083	45.5566	AAL94397.1| helix-turn-helix DNA-binding protein
					12	30	17.5896	30.0000	
FN0192	0.972	9.819	7.334e-4	1.277e-3	39	13	44.6178	16.9210	AAL94398.1| Dipeptide-binding protein
					27	26	39.5766	26.0000	
FN0197									AAL94403.1| Methyltransferase
						5		5.0000	
FN0198					5		5.7202		AAL94404.1| Transcriptional regulatory protein
					6		8.7948		
FN0199	-0.742	11.518	3.161e-3	8.198e-3	45	50	51.4821	65.0808	AAL94405.1| Hypothetical protein
					22	75	32.2476	75.0000	

Log$_2$ Ratios: -6 -4 -2 0 2 4 6

Show detected proteins only
Show all proteins
Filter by category:
GO: amino acid transport

Proteins found: 1344

Enter (or paste) list of ORFs

Find ORFs

Test	Cutoff
q-Value	.005
p-Value	

	Signif	Direction	Applies To
red	yes	+	ratios, bars
yellow	no	n/a	bars
green	yes	–	ratios, bars
light red	yes	+	*p*- , *q*-Values
light green	yes	–	

Dot Plots Dot Plots

Protein	FnPgSg vs FnSg Log$_2$ Ratio	Log$_2$ Sum	q-Value	p-Value	Raw FnPgSg	Raw FnSg	Normalized FnPgSg	Normalized FnSg	Description	Log$_2$ Ratios (-6 -4 -2 0 2 4 6)
FN0200	0.553	18.395	8.884e-6	2.354e-6	609	385	696.7248	501.1224	AAL94406.1| Biotin carboxyl carrier protein of glutaconyl-COA decarboxylase	
					495	468	725.5703	468.0000		
FN0202	1.013	19.668	4.71e-3	1.39e-2	1335	454	1527.3032	590.9339	AAL94408.1| Glutaconate CoA-transferase subunit A	
					727	694	1065.6356	694.0000		
FN0203	0.012	20.093	1.755e-1	9.207e-1	833	730	952.9915	950.1801	AAL94409.1| Glutaconate CoA-transferase subunit B	
					799	1156	1171.1731	1156.0000		
FN0204	0.101	21.482	7.827e-2	3.674e-1	1462	1129	1672.5972	1469.5251	AAL94410.1| Glutaconyl-CoA decarboxylase A subunit	
					1277	1835	1871.8248	1835.0000		
FN0206	0.170	11.038	5.194e-2	2.327e-1	44	38	50.3381	49.4614	AAL94412.1| Activator of (R)-2-hydroxyglutaryl-CoA dehydratase	
					32	37	46.9056	37.0000		
FN0207	0.227	18.648	1.91e-4	2.051e-4	620	444	709.3094	577.9178	AAL94413.1| (R)-2-hydroxyglutaryl-CoA dehydratase alpha-subunit	
					462	607	677.1990	607.0000		
FN0208	0.499	17.509	8.199e-4	1.491e-3	435	254	497.6606	330.6106	AAL94414.1| (R)-2-hydroxyglutaryl-CoA dehydratase beta-subunit	
					361	396	529.1533	396.0000		
FN0209	0.149	17.497	9.177e-2	4.371e-1	383	248	438.1701	322.8009	AAL94415.1| Hypothetical cytosolic protein	
					319	494	467.5898	494.0000		
FN0212	-0.101	8.257	1.233e-1	6.084e-1	18	14	20.5929	18.2226	AAL94418.1| Hypothetical protein	
					9	18	13.1922	18.0000		
FN0218	-0.185	12.164	4.958e-2	2.207e-1	56	48	64.0667	62.4776	AAL94424.1| Anthranilate synthase component II	
					43	82	63.0293	82.0000		
FN0219	-0.448	7.166							AAL94425.1| Autolysin response regulator	
					7	14	10.2606	14.0000		
FN0221	1.264	11.528	4.095e-3	1.15e-2	87	27	99.5321	35.1436	AAL94427.1| Carbon starvation protein A	
					47	35	68.8925	35.0000		
FN0224	0.774	8.040	1.765e-2	6.82e-2	23	6	26.3131	7.8097	AAL94430.1| Excinuclease ABC subunit B	
					11	17	16.1238	17.0000		
FN0225									AAL94431.1| Gluconate permease	
						3		3.0000		
FN0226	0.592	11.120	1.971e-3	4.537e-3	50	26	57.2024	33.8420	AAL94432.1| Pyridoxal phosphate biosynthetic protein pdxA	
					40	43	58.6319	43.0000		
FN0227									AAL94433.1| Hypothetical protein	
						5		5.0000		

Show detected proteins only
Show all proteins
Filter by category:
GO: amino acid transport

Proteins found: 1344

Enter (or paste) list of ORFs

Find ORFs

Test	Cutoff
q-Value	.005
p-Value	

Color	Signif	Direction	Applies To
red	yes	+	ratios, bars
yellow	no	n/a	bars
green	yes	–	ratios, bars
pink	yes	+	p- , q-Values
light green	yes	–	p- , q-Values

Dot Plots Dot Plots

Protein	*FnPgSg vs FnSg* Log$_2$ Ratio	Log$_2$ Sum	*q*-Value	*p*-Value	Raw *FnPgSg*	Raw *FnSg*	Normalized *FnPgSg*	Normalized *FnSg*	Description	Log$_2$ Ratios (-6 -4 -2 0 2 4 6)
**FN0233**	0.291	11.663	6.601e-3	2.126e-2	55	43	62.9226	55.9695	AAL94439.1| Hypothetical protein	
					43	47	63.0293	47.0000		
**FN0234**						11		14.3178	AAL94440.1| unknown	
						14		14.0000		
**FN0236**	0.862	14.248	6.066e-4	9.851e-4	152	76	173.8952	98.9229	AAL94442.1| ABC transporter substrate-binding protein	
					138	108	202.2802	108.0000		
**FN0238**	0.486	9.723	2.967e-2	1.23e-1	23	17	26.3131	22.1275	AAL94444.1| Hypothetical protein	
					29	27	42.5082	27.0000		
**FN0240**	0.642	12.446	3.401e-3	8.998e-3	76	55	86.9476	71.5889	AAL94446.1| Thymidylate synthase	
					68	48	99.6743	48.0000		
**FN0241**	-1.693	8.416	6.776e-4	1.148e-3	9	28	10.2964	36.4453	AAL94447.1| Dihydrofolate reductase	
					7	30	10.2606	30.0000		
**FN0242**	-2.684	11.394	8.481e-4	1.565e-3	14	116	16.0167	150.9875	AAL94448.1| Trk system potassium uptake protein trkA	
					17	112	24.9186	112.0000		
**FN0243**	-2.517	7.621				17		22.1275	AAL94449.1| Poly(A) polymerase	
					4	45	5.8632	45.0000		
**FN0244**						6		7.8097	AAL94450.1| COP associated protein	
**FN0245**	-0.905	6.464			6	9	6.8643	11.7145	AAL94451.1| Copper-exporting ATPase	
						14		14.0000		
**FN0247**	0.163	15.347	9.41e-2	4.493e-1	229	149	261.9868	193.9409	AAL94453.1| Hypothetical cytosolic protein	
					116	192	170.0326	192.0000		
**FN0248**	-2.300	9.029			9	28	10.2964	36.4453	AAL94454.1| Hypothetical Exported Protein	
						65		65.0000		
**FN0249**	1.004	13.451	5.799e-3	1.815e-2	121	81	138.4297	105.4309	AAL94455.1| unknown	
					110	44	161.2378	44.0000		
**FN0250**	1.058	13.619	4.575e-3	1.338e-2	142	81	162.4547	105.4309	AAL94456.1| unknown	
					110	50	161.2378	50.0000		
**FN0251**	2.392	13.594	2.816e-3	7.096e-3	175	40	200.2083	52.0647	AAL94457.1| Hypothetical membrane-spanning Protein	
					211	45	309.2835	45.0000		
**FN0252**	-0.913	17.211	3.001e-5	1.622e-5	231	430	264.2749	559.6951	AAL94458.1| unknown	
					207	509	303.4203	509.0000		

Show detected proteins only
Show all proteins
Filter by category:
GO: amino acid transport

Proteins found: 1344

Enter (or paste) list of ORFs

Find ORFs

Test	Cutoff
q-Value	.005
p-Value	

Colour	Signif	Direction	Applies To
red	yes	+	ratios, bars
yellow	no	n/a	bars
green	yes	−	ratios, bars
pink	yes	+	*p*- , *q*-Values
light green	yes	−	*p*- , *q*-Values

Dot Plots Dot Plots

Protein	*FnPgSg* vs *FnSg* Log$_2$ Ratio	Log$_2$ Sum	*q*-Value	*p*-Value	Raw *FnPgSg*	Raw *FnSg*	Normalized *FnPgSg*	Normalized *FnSg*	Description
FN0253	-0.305	16.664	8.723e-2	4.134e-1	234	180	267.7071	234.2910	AAL94459.1| Outer membrane protein
					213	482	312.2151	482.0000	
FN0254	-0.085	18.108	1.214e-1	5.977e-1	373	444	426.7297	577.9178	AAL94460.1| Fusobacterium outer membrane protein family
					413	517	605.3748	517.0000	
FN0258	-0.256	5.359							AAL94464.1| Zinc-transporting ATPase
					4	7	5.8632	7.0000	
FN0259					11		12.5845		AAL94465.1| Zinc-transporting ATPase
					12		17.5896		
FN0261	0.284	7.370	4.485e-2	1.971e-1	12	11	13.7286	14.3178	AAL94467.1| Pyruvate formate-lyase activating enzyme
					10	9	14.6580	9.0000	
FN0262	2.137	22.492	8.649e-11	5.458e-13	4443	878	5083.0024	1142.8193	AAL94468.1| Formate acetyltransferase
					3482	1173	5103.9107	1173.0000	
FN0263	0.584	14.433	4.604e-3	1.349e-2	180	106	205.9285	137.9714	AAL94469.1| Peptidyl-prolyl cis-trans isomerase
					108	105	158.3062	105.0000	
FN0264	0.696	16.067	1.196e-2	4.339e-2	273	211	312.3249	274.6411	AAL94470.1| Hypothetical protein
					242	137	354.7233	137.0000	
FN0265	-0.318	5.876			6	7	6.8643	9.1113	AAL94471.1| Cell division protein ftsX
						8		8.0000	
FN0266									AAL94472.1| membrane protein related to metalloendopeptidase
					3		4.3974		
FN0267									AAL94473.1| ATP-NAD kinase
						8		8.0000	
FN0268	-2.256	6.529							AAL94474.1| DNA repair protein recN
					3	21	4.3974	21.0000	
FN0270	0.033	4.735	1.521e-1	7.763e-1	4	4	4.5762	5.2065	AAL94476.1| GTP-binding protein era
					4	5	5.8632	5.0000	
FN0271	-1.016	5.404			4	5	4.5762	6.5081	AAL94477.1| Enoyl-CoA hydratase
						12		12.0000	
FN0272						10		13.0162	AAL94478.1| Acetoacetate: butyrate/acetate coenzyme A transferase
						8		8.0000	
FN0273						4		5.2065	AAL94479.1| Butyrate-acetoacetate CoA-transferase subunit B
						11		11.0000	

Log$_2$ Ratios: -6 -4 -2 0 2 4 6

Show detected proteins only
Show all proteins
Filter by category:
GO: amino acid transport

Proteins found: 1344

Enter (or paste) list of ORFs

Find ORFs

Test	Cutoff
q-Value	.005
p-Value	

Signif	Direction	Applies To
yes	+	ratios, bars
no	n/a	bars
yes	–	ratios, bars
yes	+	*p*- , *q*-Values
yes	–	

Dot Plots Dot Plots

Protein	FnPgSg vs FnSg Log_2 Ratio	Log_2 Sum	*q*-Value	*p*-Value	Raw *FnPgSg*	Raw *FnSg*	Normalized *FnPgSg*	Normalized *FnSg*	Description
FN0276	-3.677	8.066			4	50	4.5762	65.0808	AAL94482.1| Sodium-dependent phosphate transporter
						52		52.0000	
FN0277	-0.085	6.808	1.476e-1	7.491e-1	9	6	10.2964	7.8097	AAL94483.1| Hypothetical protein
					7	14	10.2606	14.0000	
FN0278	0.837	16.201	3.591e-3	9.653e-3	362	158	414.1451	205.6554	AAL94484.1| Xaa-His dipeptidase
					218	205	319.5441	205.0000	
FN0279	-2.119	13.369	1.953e-4	2.115e-4	44	175	50.3381	227.7829	AAL94485.1| Lipoprotein
					33	201	48.3714	201.0000	
FN0280	-0.017	12.941	1.276e-1	6.33e-1	76	71	86.9476	92.4148	AAL94486.1| Hypothetical protein
					61	86	89.4137	86.0000	
FN0281	-2.769	11.502	1.689e-3	3.756e-3	13	87	14.8726	113.2406	AAL94487.1| DNA polymerase III alpha subunit
					18	168	26.3844	168.0000	
FN0282	-0.302	11.142	3.537e-2	1.503e-1	30	45	34.3214	58.5727	AAL94488.1| Hypothetical protein
					35	47	51.3030	47.0000	
FN0283	-0.296	6.044							AAL94489.1| tRNA (Guanine-N1) -methyltransferase
					5	9	7.3290	9.0000	
FN0284	-1.330	10.005	2.036e-3	4.722e-3	11	32	12.5845	41.6517	AAL94490.1| 16S rRNA processing protein rimM
					19	60	27.8502	60.0000	
FN0285					22		25.1690		AAL94491.1| RNA binding protein
					10		14.6580		
FN0287	-0.215	6.357	1.081e-1	5.241e-1	7	5	8.0083	6.5081	AAL94493.1| Dimethyladenosine transferase
					6	13	8.7948	13.0000	
FN0288	-0.336	11.262	2.47e-2	1.002e-1	31	41	35.4655	53.3663	AAL94494.1| Hypoxanthine-guanine phosphoribosyltransferase
					36	58	52.7687	58.0000	
FN0290									AAL94496.1| Hemolysin
					3		4.3974		
FN0291	0.017	4.661			5		5.7202		AAL94497.1| Hemolysin
					3	5	4.3974	5.0000	
FN0294	1.498	13.983	4.768e-4	7.007e-4	188	71	215.0809	92.4148	AAL94500.1| Transketolase subunit A
					145	59	212.5408	59.0000	
FN0295	0.418	13.712	3.996e-3	1.113e-2	106	71	121.2690	92.4148	AAL94501.1| Transketolase
					100	108	146.5799	108.0000	

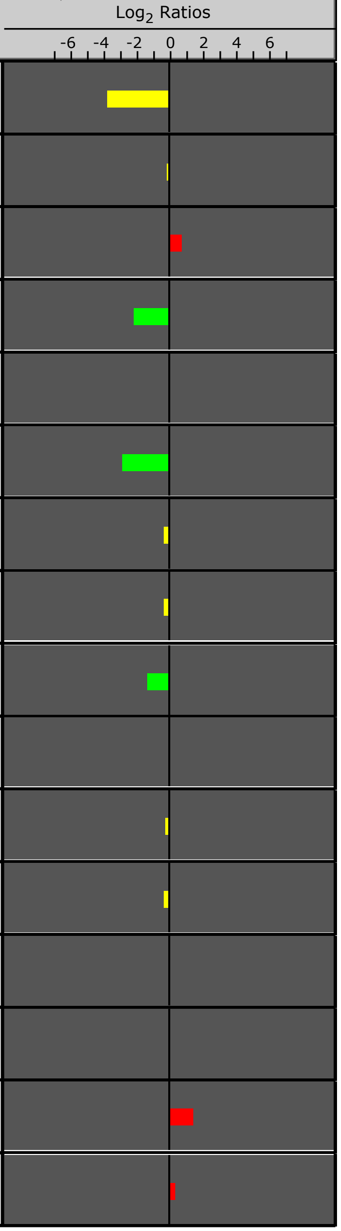

Show detected proteins only
Show all proteins
Filter by category:
GO: amino acid transport

Proteins found: 1344

Enter (or paste) list of ORFs

Find ORFs

Test	Cutoff
q-Value	.005
p-Value	

	Signif	Direction	Applies To
red	yes	+	ratios, bars
yellow	no	n/a	bars
green	yes	–	ratios, bars
pink	yes	+	*p*- , *q*-Values
light green	yes	–	*p*- , *q*-Values

Dot Plots Dot Plots

Protein	*FnPgSg* vs *FnSg* Log$_2$ Ratio	Log$_2$ Sum	*q*-Value	*p*-Value	Raw *FnPgSg*	Raw *FnSg*	Normalized *FnPgSg*	Normalized *FnSg*	Description
**FN0296**	0.483	12.045	5.828e-3	1.827e-2	60	43	68.6428	55.9695	AAL94502.1| Hypothetical cytosolic protein
					58	54	85.0163	54.0000	
**FN0297**	-1.646	7.919				20		26.0323	AAL94503.1| ATPase associated with chromosome architecture/replication
					6	29	8.7948	29.0000	
**FN0298**	-0.502	15.867	7.901e-8	2.493e-9	181	225	207.0726	292.8637	AAL94504.1| Histidyl-tRNA synthetase
					139	289	203.7460	289.0000	
**FN0299**	0.175	16.311	3.052e-3	7.844e-3	262	198	299.7404	257.7201	AAL94505.1| Aspartyl-tRNA synthetase
					209	279	306.3519	279.0000	
**FN0305**					3		3.4321		AAL94511.1| Iron(III) dicitrate-binding protein
					3		4.3974		
**FN0307**									AAL94513.1| Iron(III) dicitrate transport ATP-binding protein fecE
					3		4.3974		
**FN0308**	-0.590	17.429	3.99e-3	1.11e-2	354	417	404.9928	542.7741	AAL94514.1| Iron(III)-binding protein
					191	488	279.9675	488.0000	
**FN0309**					5		5.7202		AAL94515.1| Iron(III)-transport system permease protein sfuB
					8		11.7264		
**FN0310**	1.147	12.443	4.279e-3	1.222e-2	116	44	132.7095	57.2711	AAL94516.1| Iron(III)-transport ATP-binding protein sfuC
					61	43	89.4137	43.0000	
**FN0311**	-0.504	14.183	8.717e-4	1.621e-3	90	132	102.9643	171.8134	AAL94517.1| Anaerobic ribonucleoside-triphosphate reductase
					86	153	126.0587	153.0000	
**FN0313**	-2.199	7.231			5	25	5.7202	32.5404	AAL94519.1| 16S rRNA m(5)C 967 methyltransferase
						20		20.0000	
**FN0314**						9		11.7145	AAL94520.1| Caffeoyl-CoA O-methyltransferase
						9		9.0000	
**FN0315**						5		6.5081	AAL94521.1| Transcriptional regulator, AraC family
						10		10.0000	
**FN0316**	1.665	8.325	5.114e-4	7.712e-4	25	7	28.6012	9.1113	AAL94522.1| Hypothetical protein
					24	11	35.1792	11.0000	
**FN0317**	-2.254	8.308	2.366e-4	2.776e-4	4	29	4.5762	37.7469	AAL94523.1| Tryptophan synthase beta chain
					8	40	11.7264	40.0000	
**FN0319**	-0.705	5.808				7		9.1113	AAL94525.1| Citrate (pro-3S)-lyase ligase
					4	10	5.8632	10.0000	

Log$_2$ Ratios: -6 -4 -2 0 2 4 6

Show detected proteins only
Show all proteins
Filter by category: GO: amino acid transport

Proteins found: 1344

Enter (or paste) list of ORFs

Find ORFs

Test	Cutoff
q-Value	.005
p-Value	

	Signif	Direction	Applies To
red	yes	+	ratios, bars
yellow	no	n/a	bars
green	yes	−	ratios, bars
light red	yes	+	*p*- , *q*-Values
light green	yes	−	*p*- , *q*-Values

Dot Plots Dot Plots

Protein	*FnPgSg vs FnSg* Log$_2$ Ratio	Log$_2$ Sum	*q*-Value	*p*-Value	Raw *FnPgSg*	Raw *FnSg*	Normalized *FnPgSg*	Normalized *FnSg*	Description
**FN0320**	1.792	8.879	1.383e-4	1.325e-4	36	11	41.1857	14.3178	AAL94526.1| Hypothetical cytosolic protein
					27	9	39.5766	9.0000	
**FN0321**	-0.814	14.776	6.964e-3	2.27e-2	103	140	117.8369	182.2263	AAL94527.1| Heat shock protein htpG
					92	262	134.8535	262.0000	
**FN0322**	-0.225	21.849	3.356e-2	1.415e-1	1688	1816	1931.1519	2363.7357	AAL94528.1| Fructose-bisphosphate aldolase
					1136	1839	1665.1472	1839.0000	
**FN0325**	5.373	9.373			122		139.5738		AAL94529.1| LSU ribosomal protein L20P
					131	4	192.0196	4.0000	
**FN0326**					16		18.3048		AAL94530.1| LSU ribosomal protein L35P
					18		26.3844		
**FN0327**	-0.108	9.634	1.507e-1	7.677e-1	18	15	20.5929	19.5242	AAL94531.1| Bacterial Protein Translation Initiation Factor 3 (IF-3)
					23	39	33.7134	39.0000	
**FN0329**	0.299	17.198	7.56e-3	2.512e-2	347	243	396.9844	316.2928	AAL94533.1| LSU ribosomal protein L13P
					316	383	463.1924	383.0000	
**FN0330**	2.682	12.617	1.481e-5	5.583e-6	187	35	213.9369	45.5566	AAL94534.1| SSU ribosomal protein S9P
					128	17	187.6222	17.0000	
**FN0331**	-0.016	12.055	1.707e-1	8.905e-1	57	57	65.2107	74.1921	AAL94535.1| Hypothetical protein
					44	57	64.4951	57.0000	
**FN0332**	-0.638	6.641			7	13	8.0083	16.9210	AAL94536.1| Magnesium and cobalt transport protein corA
						8		8.0000	
**FN0333**						5		6.5081	AAL94537.1| Glycerol uptake operon antiterminator regulatory protein
						9		9.0000	
**FN0334**	-0.704	15.108	2.038e-3	4.73e-3	128	168	146.4381	218.6716	AAL94538.1| Aspartate/aromatic aminotransferase
					101	261	148.0457	261.0000	
**FN0335**	-1.328	20.789	8.524e-4	1.576e-3	764	1798	874.0522	2340.3066	AAL94539.1| Outer membrane porin F
					563	1925	825.2446	1925.0000	
**FN0336**	0.666	11.366	9.5e-3	3.294e-2	67	35	76.6512	45.5566	AAL94540.1| Hypothetical protein
					36	36	52.7687	36.0000	
**FN0341**	3.408	9.875	2.655e-6	4.131e-7	90	6	102.9643	7.8097	AAL94545.1| transport protein
					66	11	96.7427	11.0000	
**FN0342**	0.999	10.458	9.333e-3	3.225e-2	44	30	50.3381	39.0485	AAL94546.1| Peptidyl-prolyl cis-trans isomerase
					38	14	55.7003	14.0000	

Log$_2$ Ratios: -6 -4 -2 0 2 4 6

Show detected proteins only
Show all proteins
Filter by category:
GO: amino acid transport

Proteins found: 1344

Enter (or paste) list of ORFs

Find ORFs

Test	Cutoff
q-Value	.005
p-Value	

	Signif	Direction	Applies To
(red)	yes	+	ratios, bars
(yellow)	no	n/a	bars
(green)	yes	−	ratios, bars
(light red)	yes	+	*p*-, *q*-Values
(light green)	yes	−	

Dot Plots Dot Plots

Protein	FnPgSg vs FnSg Log$_2$ Ratio	Log$_2$ Sum	*q*-Value	*p*-Value	Raw *FnPgSg*	Raw *FnSg*	Normalized *FnPgSg*	Normalized *FnSg*	Description
FN0344									AAL94548.1| Methyltransferase
						3		3.0000	
FN0347	-1.266	10.946	1.861e-4	1.979e-4	27	49	30.8893	63.7792	AAL94551.1| Phosphatidylserine decarboxylase
					18	74	26.3844	74.0000	
FN0348	-0.208	15.783	6.699e-2	3.091e-1	203	233	232.2416	303.2767	AAL94552.1| Nicotinate phosphoribosyltransferase
					143	207	209.6092	207.0000	
FN0349	-1.725	8.757			10	22	11.4405	28.6356	AAL94553.1| D-Tyr-tRNATyr deacylase
						47		47.0000	
FN0351	2.212	12.080	7.791e-6	1.885e-6	122	27	139.5738	35.1436	AAL94555.1| unknown
					98	26	143.6483	26.0000	
FN0352	-0.501	10.333	1.264e-2	4.63e-2	31	38	35.4655	49.4614	AAL94556.1| NA+/H+ antiporter NHAC
					17	36	24.9186	36.0000	
FN0355	-0.566	14.877	1.074e-2	3.819e-2	116	189	132.7095	246.0055	AAL94558.1| S-adenosylmethionine synthetase
					104	176	152.4431	176.0000	
FN0356	0.482	6.858			12	7	13.7286	9.1113	AAL94559.1| Lactoylglutathione lyase
					8		11.7264		
FN0357	0.311	7.519	4.074e-2	1.768e-1	11	11	12.5845	14.3178	AAL94560.1| ATP synthase epsilon chain, sodium ion specific
					12	10	17.5896	10.0000	
FN0358	-0.644	17.129	8.106e-3	2.734e-2	268	420	306.6047	546.6790	AAL94561.1| ATP synthase beta chain, sodium ion specific
					204	400	299.0229	400.0000	
FN0359	-0.314	11.927	4.047e-3	1.132e-2	53	57	60.6345	74.1921	AAL94562.1| ATP synthase gamma chain, sodium ion specific
					35	65	51.3030	65.0000	
FN0360	-0.446	14.314	3.751e-3	1.022e-2	101	140	115.5488	182.2263	AAL94563.1| ATP synthase alpha chain, sodium ion specific
					88	151	128.9903	151.0000	
FN0361	0.209	9.714	1.088e-1	5.281e-1	25	13	28.6012	16.9210	AAL94564.1| ATP synthase delta chain, sodium ion specific
					23	37	33.7134	37.0000	
FN0362	-0.471	10.599	2.038e-2	8.06e-2	29	29	33.1774	37.7469	AAL94565.1| ATP synthase B chain, sodium ion specific
					23	55	33.7134	55.0000	
FN0364					9		10.2964		AAL94567.1| ATP synthase A chain, sodium ion specific
					5		7.3290		
FN0366	0.516	16.073	2.89e-3	7.328e-3	253	193	289.4440	251.2120	AAL94569.1| Phosphoglucosamine mutase
					231	188	338.5995	188.0000	

Log$_2$ Ratios: -6 -4 -2 0 2 4 6

Show detected proteins only
Show all proteins
Filter by category:
GO: amino acid transport

Proteins found: 1344

Enter (or paste) list of ORFs

Find ORFs

Test	Cutoff
q-Value	.005
p-Value	

	Signif	Direction	Applies To
red	yes	+	ratios, bars
yellow	no	n/a	bars
green	yes	−	ratios, bars
pink	yes	+	*p*- , *q*-Values
light green	yes	−	

Dot Plots Dot Plots

Protein	FnPgSg vs FnSg Log$_2$ Ratio	Log$_2$ Sum	*q*-Value	*p*-Value	Raw *FnPgSg*	Raw *FnSg*	Normalized *FnPgSg*	Normalized *FnSg*	Description
FN0368	0.609	12.646	2.347e-3	5.658e-3	78	42	89.2357	54.6679	AAL94571.1| Adenylosuccinate lyase
					74	75	108.4691	75.0000	
FN0370	-0.233	9.143	1.014e-1	4.882e-1	14	25	16.0167	32.5404	AAL94573.1| Signal peptidase I
					19	19	27.8502	19.0000	
FN0371	3.068	10.169			100	9	114.4047	11.7145	AAL94574.1| Hypothetical protein
					56		82.0847		
FN0374									AAL94577.1| Single-stranded-DNA-specific exonuclease recJ
						20		20.0000	
FN0375	-0.572	18.893	5.766e-3	1.801e-2	466	740	533.1261	963.1963	AAL94578.1| Iron(III)-binding protein
					417	738	611.2380	738.0000	
FN0376	-1.858	12.828	2.183e-4	2.474e-4	36	115	41.1857	149.6859	AAL94579.1| Iron(III)-transport ATP-binding protein sfuC
					33	175	48.3714	175.0000	
FN0377	2.456	7.100			39		44.6178		AAL94580.1| Iron(III)-transport system permease protein sfuB
					7	5	10.2606	5.0000	
FN0378	0.625	6.421	6.098e-2	2.782e-1	6	3	6.8643	3.9048	AAL94581.1| UDP-glucose 4-epimerase
					11	11	16.1238	11.0000	
FN0379	1.048	6.218			14		16.0167		AAL94582.1| Hypothetical protein
					6	6	8.7948	6.0000	
FN0380									AAL94583.1| unknown
					6		8.7948		
FN0381					20		22.8809		AAL94584.1| unknown
					18		26.3844		
FN0384	1.608	6.013	2.007e-5	9.367e-6	13	4	14.8726	5.2065	AAL94587.1| Hypothetical protein
					9	4	13.1922	4.0000	
FN0387	0.776	10.312	4.616e-2	2.036e-1	38	5	43.4738	6.5081	AAL94590.1| Fusobacterium outer membrane protein family
					34	48	49.8372	48.0000	
FN0390	0.453	12.917	1.121e-2	4.025e-2	103	64	117.8369	83.3035	AAL94593.1| Hypothetical protein
					60	67	87.9479	67.0000	
FN0391	-0.885	8.926	3.102e-4	4.034e-4	13	23	14.8726	29.9372	AAL94594.1| Hydrolase (HAD superfamily)
					12	30	17.5896	30.0000	
FN0392	0.677	5.464	5.732e-3	1.788e-2	7	5	8.0083	6.5081	AAL94595.1| Oxygen-independent coproporphyrinogen III oxidase
					6	4	8.7948	4.0000	

Log$_2$ Ratios: -6 -4 -2 0 2 4 6

Show detected proteins only
Show all proteins
Filter by category:
GO: amino acid transport

Proteins found: 1344

Enter (or paste) list of ORFs

Find ORFs

Test	Cutoff
q-Value	.005
p-Value	

Signif	Direction	Applies To
yes	+	ratios, bars
no	n/a	bars
yes	–	ratios, bars
yes	+	*p*-, *q*-Values
yes	–	

Dot Plots Dot Plots

Protein	FnPgSg vs FnSg Log$_2$ Ratio	Log$_2$ Sum	*q*-Value	*p*-Value	Raw *FnPgSg*	Raw *FnSg*	Normalized *FnPgSg*	Normalized *FnSg*	Description
FN0393	-2.414	7.482	3.101e-3	8.002e-3	5	29	5.7202	37.7469	AAL94596.1| Polysaccharide deacetylase
					4	24	5.8632	24.0000	
FN0394	-0.409	5.967			6	7	6.8643	9.1113	AAL94597.1| Outer membrane protein
FN0396	-0.252	24.814	5.178e-2	2.319e-1	4210	5397	4816.4394	7024.8246	AAL94599.1| Dipeptide-binding protein
					3505	4827	5137.6241	4827.0000	
FN0397	0.307	11.418	7.009e-3	2.287e-2	53	40	60.6345	52.0647	AAL94600.1| Dipeptide transport system permease protein dppB
					38	42	55.7003	42.0000	
FN0398	-0.005	11.622	1.823e-1	9.645e-1	48	38	54.9143	49.4614	AAL94601.1| Dipeptide transport system permease protein dppC
					39	63	57.1661	63.0000	
FN0399	-0.132	15.402	1.068e-1	5.174e-1	149	138	170.4631	179.6231	AAL94602.1| Dipeptide transport ATP-binding protein dppD
					155	256	227.1988	256.0000	
FN0400	-0.687	17.013	2.663e-4	3.311e-4	227	331	259.6988	430.8351	AAL94603.1| Dipeptide transport ATP-binding protein dppF
					214	492	313.6809	492.0000	
FN0405	1.035	13.828	3.145e-3	8.145e-3	175	58	200.2083	75.4938	AAL94608.1| Tryptophanyl-tRNA synthetase
					99	93	145.1141	93.0000	
FN0406	1.121	10.829	8.129e-4	1.473e-3	60	16	68.6428	20.8259	AAL94609.1| Alanine racemase, biosynthetic
					39	37	57.1661	37.0000	
FN0407	1.902	11.604	3.189e-4	4.188e-4	104	19	118.9809	24.7307	AAL94610.1| Hypothetical protein
					66	33	96.7427	33.0000	
FN0408	-0.243	12.819	1.574e-2	5.965e-2	61	76	69.7869	98.9229	AAL94611.1| Acetyl-coenzyme A carboxylase carboxyl transferase subunit beta
					59	86	86.4821	86.0000	
FN0409	1.034	17.399	9.59e-4	1.833e-3	475	219	543.4225	285.0540	AAL94612.1| Acetyl-coenzyme A carboxylase carboxyl transferase subunit alpha
					441	296	646.4172	296.0000	
FN0410	0.134	13.738	2.035e-2	8.044e-2	109	80	124.7012	104.1293	AAL94613.1| 6-phosphofructokinase
					82	119	120.1955	119.0000	
FN0411						13		16.9210	AAL94614.1| putative alpha helix protein
FN0413					4		4.5762		AAL94616.1| unknown
FN0414	0.779	4.779			6		6.8643		AAL94617.1| ATP-dependent helicase HEPA
						4		4.0000	

Show detected proteins only
Show all proteins
Filter by category:
GO: amino acid transport

Proteins found: 1344

Enter (or paste) list of ORFs

Find ORFs

Test	Cutoff
q-Value	.005
p-Value	

Signif	Direction	Applies To
yes	+	ratios, bars
no	n/a	bars
yes	–	ratios, bars
yes	+	*p*- , *q*-Values
yes	–	

Dot Plots Dot Plots

Protein	FnPgSg vs FnSg Log_2 Ratio	Log_2 Sum	*q*-Value	*p*-Value	Raw *FnPgSg*	Raw *FnSg*	Normalized *FnPgSg*	Normalized *FnSg*	Description
FN0416					9		10.2964		AAL94619.1| Type III restriction-modification system methylation subunit
					9		13.1922		
FN0417	0.376	7.020			15		17.1607		AAL94620.1| Type III restriction-modification system restriction subunit
					6	10	8.7948	10.0000	
FN0418	-4.377	8.766			4	77	4.5762	100.2245	AAL94621.1| Uracil phosphoribosyltransferase
						90		90.0000	
FN0419	-0.156	6.153	1.149e-1	5.614e-1	5	6	5.7202	7.8097	AAL94622.1| Aspartate carbamoyltransferase
					7	10	10.2606	10.0000	
FN0420	-3.496	7.055			3	38	3.4321	49.4614	AAL94623.1| Dihydroorotase
						28		28.0000	
FN0421	-1.876	8.072	3.952e-5	2.469e-5	6	26	6.8643	33.8420	AAL94624.1| Carbamoyl-phosphate synthase small chain
					7	29	10.2606	29.0000	
FN0422	-1.258	13.894	5.972e-4	9.625e-4	69	158	78.9393	205.6554	AAL94625.1| Carbamoyl-phosphate synthase large chain
					55	176	80.6189	176.0000	
FN0423	-2.168	7.580	3.677e-7	3.48e-8	5	22	5.7202	28.6356	AAL94626.1| Dihydroorotate dehydrogenase electron transfer subunit
					5	30	7.3290	30.0000	
FN0424	-2.638	6.196			3	19	3.4321	24.7307	AAL94627.1| Dihydroorotate dehydrogenase
						18		18.0000	
FN0426	-1.179	10.328	4.122e-4	5.785e-4	25	46	28.6012	59.8744	AAL94629.1| Orotidine 5'-phosphate decarboxylase
					13	48	19.0554	48.0000	
FN0427	-0.251	11.324	1.969e-2	7.742e-2	44	38	50.3381	49.4614	AAL94630.1| Orotate phosphoribosyltransferase
					29	61	42.5082	61.0000	
FN0430	0.099	15.184	7.447e-2	3.479e-1	171	159	195.6321	206.9570	AAL94633.1| LSU ribosomal protein L19P
					139	166	203.7460	166.0000	
FN0435	-0.710	11.011	3.245e-2	1.362e-1	48	47	54.9143	61.1760	AAL94634.1| Purine nucleoside phosphorylase
					11	55	16.1238	55.0000	
FN0436	-1.804	10.829	8.356e-3	2.832e-2	13	41	14.8726	53.3663	AAL94635.1| regulator of kinase autophosphorylation inhibitor
					21	106	30.7818	106.0000	
FN0437	-1.404	7.249	2.962e-3	7.557e-3	3	17	3.4321	22.1275	AAL94636.1| kinase autophosphorylation inhibitor KipI
					8	18	11.7264	18.0000	
FN0439	0.029	11.834	1.751e-1	9.185e-1	58	32	66.3547	41.6517	AAL94638.1| Lactam utilization protein LAMB
					38	78	55.7003	78.0000	

Show detected proteins only
Show all proteins
Filter by category:
GO: amino acid transport

Proteins found: 1344

Enter (or paste) list of ORFs

Find ORFs

Test	Cutoff
q-Value	.005
p-Value	

Signif	Direction	Applies To
yes	+	ratios, bars
no	n/a	bars
yes	−	ratios, bars
yes	+	*p*- , *q*-Values
yes	−	

Dot Plots Dot Plots

Protein	FnPgSg vs FnSg Log_2 Ratio	Log_2 Sum	*q*-Value	*p*-Value	Raw *FnPgSg*	Raw *FnSg*	Normalized *FnPgSg*	Normalized *FnSg*	Description
FN0445	0.085	5.255			6		6.8643		AAL94641.1| Hypothetical protein
					4	6	5.8632	6.0000	
FN0446	-0.594	7.934	1.861e-2	7.25e-2	12	18	13.7286	23.4291	AAL94642.1| Hypothetical protein
					8	15	11.7264	15.0000	
FN0447	-1.907	6.295			4	11	4.5762	14.3178	AAL94643.1| NIFS protein
						20		20.0000	
FN0448									AAL94644.1| DNA-repair protein
					3		4.3974		
FN0450	-0.497	9.431	3.205e-2	1.343e-1	22	18	25.1690	23.4291	AAL94646.1| ABC transporter ATP-binding protein
					13	39	19.0554	39.0000	
FN0451	-0.545	5.649				7		9.1113	AAL94647.1| Hypothetical protein
					4	8	5.8632	8.0000	
FN0452	1.852	17.267	8.434e-5	6.921e-5	663	136	758.5034	177.0199	AAL94648.1| Glucosamine--fructose-6-phosphate aminotransferase (isomerizing)
					512	241	750.4889	241.0000	
FN0453	-0.516	15.975	4.527e-4	6.539e-4	198	219	226.5214	285.0540	AAL94649.1| Xaa-Pro aminopeptidase
					135	322	197.8828	322.0000	
FN0454	-1.924	14.121	1.148e-3	2.325e-3	57	173	65.2107	225.1797	AAL94650.1| Aldehyde dehydrogenase B
					49	295	71.8241	295.0000	
FN0455	-0.538	18.144	3.535e-3	9.458e-3	331	547	378.6797	711.9843	AAL94651.1| Rubrerythrin
					351	585	514.4953	585.0000	
FN0456	1.418	9.004	1.978e-5	9.115e-6	34	9	38.8976	11.7145	AAL94652.1| Hypothetical cytosolic protein
					24	16	35.1792	16.0000	
FN0458	-0.163	5.910				8		10.4129	AAL94654.1| Hypothetical Exported Protein
					5	6	7.3290	6.0000	
FN0460	0.770	8.427	5.348e-3	1.637e-2	18	8	20.5929	10.4129	AAL94656.1| Delta-aminolevulinic acid dehydratase
					19	18	27.8502	18.0000	
FN0461	0.038	15.636	1.155e-1	5.651e-1	187	177	213.9369	230.3861	AAL94657.1| Probable sigma(54) modulation protein
					166	215	243.3226	215.0000	
FN0462	-2.470	10.980	2.488e-3	6.106e-3	18	98	20.5929	127.5584	AAL94658.1| DNA mismatch repair protein mutL
					12	84	17.5896	84.0000	
FN0465	-0.644	14.340	1.392e-2	5.177e-2	104	166	118.9809	216.0683	AAL94661.1| Hypothetical protein
					76	144	111.4007	144.0000	

Show detected proteins only
Show all proteins
Filter by category:
GO: amino acid transport

Proteins found: 1344

Enter (or paste) list of ORFs

Find ORFs

Test	Cutoff
q-Value	.005
p-Value	

	Signif	Direction	Applies To
red	yes	+	ratios, bars
yellow	no	n/a	bars
green	yes	−	ratios, bars
pink	yes	+	*p*- , *q*-Values
light green	yes	−	

Dot Plots Dot Plots

Protein	FnPgSg vs FnSg Log$_2$ Ratio	Log$_2$ Sum	*q*-Value	*p*-Value	Raw *FnPgSg*	Raw *FnSg*	Normalized *FnPgSg*	Normalized *FnSg*	Description
FN0466	-0.408	17.650	9.611e-3	3.34e-2	295	446	337.4940	580.5210	AAL94662.1| Lysyl-tRNA synthetase
					307	464	450.0002	464.0000	
FN0469									AAL94665.1| Copper homeostasis protein cutC
						4		4.0000	
FN0470	0.147	17.827	4.969e-2	2.213e-1	427	310	488.5082	403.5011	AAL94666.1| Putative efflux pump component MtrF
					359	513	526.2217	513.0000	
FN0472	0.717	25.743	1.814e-7	9.116e-9	8444	4554	9660.3359	5927.5618	AAL94668.1| Flavodoxin
					6518	5764	9554.0753	5764.0000	
FN0474						4		5.2065	AAL94670.1| Acriflavin resistance protein B
FN0475	-0.961	9.059	1.569e-3	3.417e-3	11	28	12.5845	36.4453	AAL94671.1| MIAB protein
					14	28	20.5212	28.0000	
FN0476	0.040	12.776	1.414e-1	7.123e-1	69	57	78.9393	74.1921	AAL94672.1| Transcription termination factor rho
					62	91	90.8795	91.0000	
FN0477	-1.470	8.715	3.416e-4	4.569e-4	10	24	11.4405	31.2388	AAL94673.1| Cell wall endopeptidase family M23/M37
					9	37	13.1922	37.0000	
FN0478	-1.541	8.528	4.136e-4	5.812e-4	12	25	13.7286	32.5404	AAL94674.1| GcpE protein
					6	33	8.7948	33.0000	
FN0479						9		11.7145	AAL94675.1| RNA polymerase sigma-E factor
						25		25.0000	
FN0480									AAL94676.1| unknown
						4		4.0000	
FN0481						3		3.9048	AAL94677.1| unknown
						15		15.0000	
FN0482					59		67.4988		AAL94678.1| LSU ribosomal protein L31P
					36		52.7687		
FN0483	0.988	12.842	4.783e-5	3.29e-5	111	52	126.9893	67.6841	AAL94679.1| Uracil phosphoribosyltransferase
					78	54	114.3323	54.0000	
FN0484	-0.994	5.382			4	7	4.5762	9.1113	AAL94680.1| Lipase
FN0487	0.010	20.575	1.767e-1	9.282e-1	1065	1078	1218.4104	1403.1427	AAL94683.1| 2-hydroxyglutarate dehydrogenase
					880	1087	1289.9028	1087.0000	

Log$_2$ Ratios: -6 -4 -2 0 2 4 6

Show detected proteins only
Show all proteins
Filter by category:
GO: amino acid transport

Proteins found: 1344

Enter (or paste) list of ORFs

Find ORFs

Test	Cutoff
q-Value	.005
p-Value	

	Signif	Direction	Applies To
red	yes	+	ratios, bars
yellow	no	n/a	bars
green	yes	−	ratios, bars
pink	yes	+	*p*- , *q*-Values
light green	yes	−	*p*- , *q*-Values

Dot Plots Dot Plots

Protein	*FnPgSg vs FnSg* Log$_2$ Ratio	Log$_2$ Sum	*q*-Value	*p*-Value	Raw *FnPgSg*	Raw *FnSg*	Normalized *FnPgSg*	Normalized *FnSg*	Description
**FN0488**	-0.236	24.136	2.745e-2	1.127e-1	3228	4005	3692.9849	5212.9743	AAL94684.1| NAD-specific glutamate dehydrogenase
					2879	4107	4220.0342	4107.0000	
**FN0489**	2.057	8.397			36		41.1857		AAL94685.1| Prolipoprotein diacylglyceryl transferase
					23	9	33.7134	9.0000	
**FN0491**	-2.248	13.004	2.198e-3	5.202e-3	33	123	37.7536	160.0988	AAL94687.1| Alanine racemase
					31	235	45.4398	235.0000	
**FN0493**	-1.495	6.173	1.472e-2	5.518e-2	5	15	5.7202	19.5242	AAL94689.1| Hypothetical protein
					3	9	4.3974	9.0000	
**FN0494**	1.982	15.979	3.216e-5	1.793e-5	409	69	467.9154	89.8115	AAL94690.1| Short chain dehydrogenase
					370	166	542.3455	166.0000	
**FN0495**	-0.360	26.067	8.608e-6	2.227e-6	6337	7402	7249.8281	9634.5658	AAL94691.1| Acetyl-CoA acetyltransferase
					5153	9364	7553.2602	9364.0000	
**FN0497**									AAL94693.1| Plasmid addiction system poison protein
					5		7.3290		
**FN0501**	-1.656	16.791	3.928e-4	5.439e-4	156	420	178.4714	546.6790	AAL94697.1| Ornithine decarboxylase
					137	649	200.8144	649.0000	
**FN0502**	0.155	9.203			23		26.3131		AAL94698.1| Phosphoheptose isomerase
					17	23	24.9186	23.0000	
**FN0503**	0.087	11.453	4.988e-4	7.451e-4	48	39	54.9143	50.7630	AAL94699.1| Transcriptional regulatory protein, LYSR family
					37	52	54.2345	52.0000	
**FN0504**									AAL94700.1| Arginine permease
					3		4.3974		
**FN0505**	-0.205	9.723	8.974e-2	4.265e-1	23	18	26.3131	23.4291	AAL94701.1| Anthranilate synthase component II
					19	39	27.8502	39.0000	
**FN0506**	1.493	14.740	1.575e-3	3.434e-3	279	67	319.1892	87.2083	AAL94702.1| Arginyl-tRNA synthetase
					161	110	235.9936	110.0000	
**FN0511**	-1.458	10.684	1.359e-3	2.857e-3	21	58	24.0250	75.4938	AAL94707.1| D-lactate dehydrogenase
					17	59	24.9186	59.0000	
**FN0512**	-0.051	15.650	4.334e-2	1.896e-1	196	171	224.2333	222.5764	AAL94708.1| Flavoprotein
					151	239	221.3356	239.0000	
**FN0513**	1.549	9.899	2.193e-4	2.491e-4	45	17	51.4821	22.1275	AAL94709.1| Flavodoxin
					37	14	54.2345	14.0000	

Log$_2$ Ratios: -6 -4 -2 0 2 4 6

- Show detected proteins only
- Show all proteins
- Filter by category: GO: amino acid transport

Proteins found: 1344

Enter (or paste) list of ORFs

Find ORFs

Test	Cutoff
q-Value	.005
p-Value	

Color	Signif	Direction	Applies To
red	yes	+	ratios, bars
yellow	no	n/a	bars
green	yes	–	ratios, bars
pink	yes	+	*p*- , *q*-Values
light green	yes	–	*p*- , *q*-Values

Dot Plots Dot Plots

Protein	FnPgSg vs FnSg: Log_2 Ratio	Log_2 Sum	*q*-Value	*p*-Value	Raw *FnPgSg*	Raw *FnSg*	Normalized *FnPgSg*	Normalized *FnSg*	Description
FN0515						4		5.2065	AAL94711.1| Acriflavin resistance protein D
						10		10.0000	
FN0517						10		13.0162	AAL94713.1| Outer membrane protein tolC
						8		8.0000	
FN0519	-1.010	5.284				9		11.7145	AAL94715.1| Hypothetical exported 24-amino acid repeat protein
					3	6	4.3974	6.0000	
FN0522	-1.908	6.182				10		13.0162	AAL94718.1| Exonuclease SBCC
					3	20	4.3974	20.0000	
FN0523						5		6.5081	AAL94719.1| Exonuclease SBCD
						9		9.0000	
FN0524	1.057	7.477	3.061e-3	7.872e-3	17	5	19.4488	6.5081	AAL94720.1| DNA helicase II
					13	12	19.0554	12.0000	
FN0525	-0.848	14.972	6.896e-3	2.242e-2	108	219	123.5571	285.0540	AAL94721.1| Penicillin-binding protein
					98	196	143.6483	196.0000	
FN0526	-0.443	11.386	4.394e-3	1.267e-2	34	42	38.8976	54.6679	AAL94722.1| Florfenicol resistance protein
					34	66	49.8372	66.0000	
FN0527	-2.095	9.163	1.056e-3	2.084e-3	10	33	11.4405	42.9533	AAL94723.1| Alanyl-tRNA synthetase
					8	56	11.7264	56.0000	
FN0528	5.036	14.918	2e-4	2.185e-4	951	28	1087.9890	36.4453	AAL94724.1| Cold shock protein
					633	25	927.8505	25.0000	
FN0535	1.268	11.141	2.193e-4	2.491e-4	61	24	69.7869	31.2388	AAL94731.1| Hypothetical protein
					53	30	77.6873	30.0000	
FN0536	0.517	15.709	9.179e-4	1.732e-3	242	159	276.8595	206.9570	AAL94732.1| DNA polymerase III, beta chain
					189	180	277.0359	180.0000	
FN0540	-0.270	10.828	1.773e-2	6.856e-2	32	32	36.6095	41.6517	AAL94736.1| Glutamate-1-semialdehyde 2,1-aminomutase
					28	52	41.0424	52.0000	
FN0541						6		7.8097	AAL94737.1| polysaccharide deacetylase
						11		11.0000	
FN0542	-0.813	9.376			17	31	19.4488	40.3501	AAL94738.1| Beta 1,4 glucosyltransferase
						28		28.0000	
FN0543	0.674	9.063	1.376e-3	2.901e-3	28	12	32.0333	15.6194	AAL94739.1| Lipopolysaccharide heptosyltransferase-1
					18	21	26.3844	21.0000	

Show detected proteins only
Show all proteins
Filter by category:
GO: amino acid transport

Proteins found: 1344

Enter (or paste) list of ORFs

Find ORFs

Test	Cutoff
q-Value	.005
p-Value	

Signif	Direction	Applies To
yes	+	ratios, bars
no	n/a	bars
yes	–	ratios, bars
yes	+	*p*- , *q*-Values
yes	–	

Dot Plots Dot Plots

Protein	*FnPgSg vs FnSg* Log_2 Ratio	Log_2 Sum	*q*-Value	*p*-Value	Raw *FnPgSg*	Raw *FnSg*	Normalized *FnPgSg*	Normalized *FnSg*	Description	Log_2 Ratios (-6 … 6)
**FN0547**	-0.407	16.976	1.557e-2	5.891e-2	326	304	372.9594	395.6914	AAL94743.1| RecA protein	
					171	431	250.6516	431.0000		
**FN0549**									AAL94745.1| O-sialoglycoprotein endopeptidase	
						13		13.0000		
**FN0550**	0.685	8.043			18	12	20.5929	15.6194	AAL94746.1| hypothetical Protein	
						10		10.0000		
**FN0552**	-1.794	7.797			7	15	8.0083	19.5242	AAL94748.1| Serine racemase	
						36		36.0000		
**FN0553**	-1.390	14.316	3.49e-3	9.303e-3	85	144	97.2440	187.4328	AAL94749.1| D-serine dehydratase	
					54	275	79.1531	275.0000		
**FN0554**	-2.684	6.243			3	17	3.4321	22.1275	AAL94750.1| D-serine permease	
						22		22.0000		
**FN0555**						6		7.8097	AAL94751.1| Transcriptional regulator, MerR family	
**FN0556**	-1.304	9.886	1.066e-3	2.11e-3	15	42	17.1607	54.6679	AAL94752.1| unknown	
					15	42	21.9870	42.0000		
**FN0557**	-3.213	12.672	3.976e-3	1.105e-2	31	242	35.4655	314.9912	AAL94753.1| unknown	
					12	177	17.5896	177.0000		
**FN0558**	-1.801	11.770	4.484e-3	1.303e-2	31	105	35.4655	136.6697	AAL94754.1| TraT complement resistance protein precursor	
					19	84	27.8502	84.0000		
**FN0559**	-0.515	14.682	5.467e-3	1.683e-2	100	151	114.4047	196.5441	AAL94755.1| Phosphoglucomutase	
					107	191	156.8405	191.0000		
**FN0560**									AAL94756.1| Oxygen-independent coproporphyrinogen III oxidase	
						6		6.0000		
**FN0561**	0.048	9.680	8.862e-2	4.206e-1	24	21	27.4571	27.3339	AAL94757.1| Proline synthetase associated protein	
					21	29	30.7818	29.0000		
**FN0562**	0.699	14.114	1.092e-3	2.181e-3	157	93	179.6154	121.0503	AAL94758.1| Hypothetical cytosolic protein	
					109	88	159.7720	88.0000		
**FN0563**	-0.190	6.821	8.957e-2	4.256e-1	11	9	12.5845	11.7145	AAL94759.1| putative tRNA (5-methylaminomethyl-2-thiouridylate) -methyltransferase	
					5	11	7.3290	11.0000		
**FN0574**					4		4.5762		AAL94770.1| Hypothetical cytosolic protein	

Show detected proteins only
Show all proteins
Filter by category:
GO: amino acid transport

Proteins found: 1344

Enter (or paste) list of ORFs

Find ORFs

Test	Cutoff
q-Value	.005
p-Value	

	Signif	Direction	Applies To
red	yes	+	ratios, bars
yellow	no	n/a	bars
green	yes	−	ratios, bars
pink	yes	+	*p*- , *q*-Values
light green	yes	−	*p*- , *q*-Values

Dot Plots Dot Plots

Protein	*FnPgSg vs FnSg* Log$_2$ Ratio	Log$_2$ Sum	*q*-Value	*p*-Value	Raw *FnPgSg*	Raw *FnSg*	Normalized *FnPgSg*	Normalized *FnSg*	Description
FN0576	-1.209	11.617	6.242e-3	1.987e-2	26	81	29.7452	105.4309	AAL94772.1| hypothetical protein
					30	65	43.9740	65.0000	
FN0577						13		16.9210	AAL94773.1| Hypothetical protein
						13		13.0000	
FN0579	-0.643	18.026	1.183e-2	4.285e-2	350	588	400.4166	765.3505	AAL94775.1| Hypothetical cytosolic protein
					291	526	426.5474	526.0000	
FN0580						7		9.1113	AAL94776.1| Penicillin-binding protein
						12		12.0000	
FN0581	-1.092	6.839				12		15.6194	AAL94777.1| Lipoprotein releasing system transmembrane protein lolE
					5		7.3290		
FN0582	-0.843	9.788	7.256e-3	2.388e-2	26	32	29.7452	41.6517	AAL94778.1| Lipoprotein releasing system ATP-binding protein lolD
					10	38	14.6580	38.0000	
FN0583						7		9.1113	AAL94779.1| Hypothetical Exported Protein
						15		15.0000	
FN0585	-1.085	5.473			4	8	4.5762	10.4129	AAL94781.1| Two-component response regulator czcR
						9		9.0000	
FN0586	-1.178	5.567			4	9	4.5762	11.7145	AAL94782.1| Two-component sensor kinase czcS
						9		9.0000	
FN0590	-2.486	8.141	1.893e-3	4.316e-3	6	25	6.8643	32.5404	AAL94786.1| N-acyl-L-amino acid amidohydrolase
					5	47	7.3290	47.0000	
FN0592	-0.508	12.602	3.992e-2	1.727e-1	49	93	56.0583	121.0503	AAL94788.1| ATP-dependent DNA helicase pcrA
					52	67	76.2215	67.0000	
FN0593	0.348	11.005	3.27e-3	8.556e-3	42	31	48.0500	40.3501	AAL94789.1| UDP-3-O-[3-hydroxymyristoyl] N-acetylglucosamine deacetylase
					37	40	54.2345	40.0000	
FN0594	-3.691	10.080			8	91	9.1524	118.4471	AAL94790.1| (3R)-hydroxymyristoyl-[acyl carrier protein] dehydratase
						118		118.0000	
FN0595	1.664	8.836	3.179e-2	1.33e-1	14	10	16.0167	13.0162	AAL94791.1| Acyl-[acyl-carrier-protein]-UDP-N-acetylglucosamine O-acyltransferase
					41	11	60.0977	11.0000	
FN0596	-0.661	8.220			12	18	13.7286	23.4291	AAL94792.1| Hypothetical protein
						20		20.0000	
FN0597	-0.918	9.075	1.111e-3	2.232e-3	18	26	20.5929	33.8420	AAL94793.1| Lipid-A-disaccharide synthase
					9	30	13.1922	30.0000	

Log$_2$ Ratios: -6 -4 -2 0 2 4 6

Show detected proteins only
Show all proteins
Filter by category:
GO: amino acid transport

Proteins found: 1344

Enter (or paste) list of ORFs

Find ORFs

Test	Cutoff
q-Value	.005
p-Value	

	Signif	Direction	Applies To
red	yes	+	ratios, bars
yellow	no	n/a	bars
green	yes	−	ratios, bars
pink	yes	+	*p*- , *q*-Values
light green	yes	−	

Dot Plots Dot Plots

Protein	FnPgSg vs FnSg Log$_2$ Ratio	Log$_2$ Sum	*q*-Value	*p*-Value	Raw *FnPgSg*	Raw *FnSg*	Normalized *FnPgSg*	Normalized *FnSg*	Description
FN0598	0.412	9.072	2.045e-3	4.748e-3	25	14	28.6012	18.2226	AAL94794.1| Phospholipid-lipopolysaccharide ABC transporter
					17	22	24.9186	22.0000	
FN0600	0.166	12.923	6.675e-2	3.078e-1	94	61	107.5405	79.3986	AAL94796.1| Hypothetical protein
					54	87	79.1531	87.0000	
FN0602	0.567	13.502	1.503e-4	1.489e-4	110	63	125.8452	82.0018	AAL94798.1| Hypothetical protein
					93	95	136.3193	95.0000	
FN0603						11		14.3178	AAL94799.1| Transcriptional regulatory protein, LYSR family
						12		12.0000	
FN0605									AAL94801.1| Aspartate aminotransferase
					5		7.3290		
FN0608	-1.551	12.941	4.05e-8	1.022e-9	47	118	53.7702	153.5908	AAL94804.1| Exoribonuclease II
					34	150	49.8372	150.0000	
FN0609									AAL94805.1| Small protein B
						11		11.0000	
FN0610	0.163	15.319	2.146e-2	8.568e-2	201	153	229.9535	199.1473	AAL94806.1| unknown
					135	183	197.8828	183.0000	
FN0611	-0.822	18.665	2.054e-4	2.268e-4	471	612	538.8463	796.5893	AAL94807.1| Threonyl-tRNA synthetase
					294	918	430.9448	918.0000	
FN0612	1.108	12.667	2.04e-2	8.069e-2	66	36	75.5071	46.8582	AAL94808.1| Hypothetical protein
					110	63	161.2378	63.0000	
FN0614									AAL94810.1| Export ABC transporter
						3		3.0000	
FN0616					50		57.2024		AAL94812.1| Hypothetical protein
					22		32.2476		
FN0617	0.904	14.207	8.709e-6	2.273e-6	170	73	194.4881	95.0180	AAL94813.1| DNA polymerase III, beta chain
					124	106	181.7590	106.0000	
FN0618	-0.283	12.874	5.864e-2	2.664e-1	72	90	82.3714	117.1455	AAL94814.1| Spermidine/putrescine-binding protein
					51	74	74.7557	74.0000	
FN0619	0.277	7.746				12		15.6194	AAL94815.1| Small-conductance mechanosensitive channel
					11	11	16.1238	11.0000	
FN0621	0.537	11.395	2.307e-2	9.287e-2	67	37	76.6512	48.1598	AAL94817.1| 4-hydroxybutyrate coenzyme A transferase
					33	38	48.3714	38.0000	

Log$_2$ Ratios: -6 -4 -2 0 2 4 6

Show detected proteins only
Show all proteins
Filter by category:
GO: amino acid transport

Proteins found: 1344

Enter (or paste) list of ORFs

Find ORFs

Test	Cutoff
q-Value	.005
p-Value	

	Signif	Direction	Applies To
red	yes	+	ratios, bars
yellow	no	n/a	bars
green	yes	–	ratios, bars
pink	yes	+	*p*- , *q*-Values
light green	yes	–	

Dot Plots Dot Plots

Protein	*FnPgSg vs FnSg* Log2 Ratio	Log2 Sum	*q*-Value	*p*-Value	Raw *FnPgSg*	Raw *FnSg*	Normalized *FnPgSg*	Normalized *FnSg*	Description
FN0622	-4.367	8.699	1.66e-3	3.674e-3	4	57	4.5762	74.1921	AAL94818.1| 8-oxoguanine DNA glycosylase
					3	111	4.3974	111.0000	
FN0625	1.618	9.431			51		58.3464		AAL94821.1| Aspartate aminotransferase
					23	15	33.7134	15.0000	
FN0626									AAL94822.1| Hypothetical cytosolic protein
						12		12.0000	
FN0627	1.417	15.214	4.805e-7	4.851e-8	284	95	324.9095	123.6536	AAL94823.1| Glucosamine--fructose-6-phosphate aminotransferase (isomerizing)
					213	115	312.2151	115.0000	
FN0628	1.684	7.195	1.338e-4	1.266e-4	20	5	22.8809	6.5081	AAL94824.1| Glucosamine--fructose-6-phosphate aminotransferase (isomerizing)
					14	7	20.5212	7.0000	
FN0629	0.757	10.133	5.895e-3	1.853e-2	39	25	44.6178	32.5404	AAL94825.1| PTS system, IID component
					29	19	42.5082	19.0000	
FN0630					34		38.8976		AAL94826.1| PTS system, IIC component
					11		16.1238		
FN0631	-0.308	7.911	5.295e-2	2.379e-1	9	15	10.2964	19.5242	AAL94827.1| PTS system, IIB component
					12	15	17.5896	15.0000	
FN0633	-0.564	6.408	5.091e-2	2.275e-1	3	8	3.4321	10.4129	AAL94829.1| Replication protein
					8	12	11.7264	12.0000	
FN0634	1.675	12.072	3.101e-5	1.7e-5	105	28	120.1250	36.4453	AAL94830.1| GTP-binding protein TypA/BipA
					78	37	114.3323	37.0000	
FN0637					4		4.5762		AAL94833.1| Hypothetical exported 24-amino acid repeat protein
					5		7.3290		
FN0643	-0.381	8.335	7.704e-2	3.611e-1	16	10	18.3048	13.0162	AAL94839.1| hypothetical DNA-binding protein
					9	28	13.1922	28.0000	
FN0644	0.235	10.507	1.079e-1	5.229e-1	48	21	54.9143	27.3339	AAL94840.1| Uroporphyrin-III C-methyltransferase
					19	43	27.8502	43.0000	
FN0645	0.275	5.444			5		5.7202		AAL94841.1| Porphobilinogen deaminase
					6	6	8.7948	6.0000	
FN0646	2.069	5.239			11		12.5845		AAL94842.1| Glutamyl-tRNA reductase
						3		3.0000	
FN0647					5		5.7202		AAL94843.1| transcriptional regulator

Log2 Ratios: -6 -4 -2 0 2 4 6

Show detected proteins only
Show all proteins
Filter by category:
GO: amino acid transport

Proteins found: 1344

Enter (or paste) list of ORFs

Find ORFs

Test	Cutoff
q-Value	.005
p-Value	

	Signif	Direction	Applies To
red	yes	+	ratios, bars
yellow	no	n/a	bars
green	yes	−	ratios, bars
pink	yes	+	*p*- , *q*-Values
light green	yes	−	

Dot Plots | Dot Plots

Protein	*FnPgSg* vs *FnSg* Log2 Ratio	Log2 Sum	*q*-Value	*p*-Value	Raw *FnPgSg*	Raw *FnSg*	Normalized *FnPgSg*	Normalized *FnSg*	Description
FN0651									AAL94847.1| Ribosomal large subunit pseudouridine synthase D
						6		6.0000	
FN0652	-1.847	24.301	4.383e-4	6.266e-4	2302	5960	2633.5970	7757.6347	AAL94848.1| Glyceraldehyde 3-phosphate dehydrogenase
					1474	9493	2160.5871	9493.0000	
FN0653	-2.143	11.378	1.208e-3	2.477e-3	16	69	18.3048	89.8115	AAL94849.1| unknown
					21	127	30.7818	127.0000	
FN0654	-1.529	19.324	2.485e-4	2.983e-4	366	1156	418.7213	1504.6687	AAL94850.1| Phosphoglycerate kinase
					365	1248	535.0165	1248.0000	
FN0655	1.763	9.915	4.916e-3	1.47e-2	36	19	41.1857	24.7307	AAL94851.1| unknown
					50	9	73.2899	9.0000	
FN0656					33		37.7536		AAL94852.1| Hypothetical protein
					26		38.1108		
FN0657						42		54.6679	AAL94853.1| Acetyltransferase
						57		57.0000	
FN0658	1.668	13.308	4.392e-6	8.367e-7	155	43	177.3273	55.9695	AAL94854.1| ABC transporter substrate-binding protein
					124	57	181.7590	57.0000	
FN0660	0.080	6.117	1.598e-1	8.224e-1	6	4	6.8643	5.2065	AAL94856.1| ABC transporter ATP-binding protein
					7	11	10.2606	11.0000	
FN0662	-0.318	11.685	2.059e-3	4.791e-3	45	47	51.4821	61.1760	AAL94858.1| Formiminoglutamase
					35	67	51.3030	67.0000	
FN0664	-0.274	14.075	1.307e-1	6.503e-1	123	46	140.7178	59.8744	AAL94860.1| 2-nitropropane dioxygenase
					67	229	98.2085	229.0000	
FN0666						4		5.2065	AAL94862.1| Hypothetical protein
						7		7.0000	
FN0668	-0.727	7.955	1.69e-2	6.487e-2	15	15	17.1607	19.5242	AAL94864.1| High-affinity zinc uptake system protein znuA precursor
					5	21	7.3290	21.0000	
FN0672	-0.328	7.772				17		22.1275	AAL94868.1| ATPase
					9	11	13.1922	11.0000	
FN0675	-1.599	22.077	7.717e-3	2.578e-2	974	2016	1114.3021	2624.0590	AAL94871.1| 60 kDa chaperonin GROEL
					889	4698	1303.0950	4698.0000	
FN0676	1.171	11.985	2.937e-4	3.755e-4	76	36	86.9476	46.8582	AAL94872.1| 10 kDa chaperonin GROES
					71	38	104.0717	38.0000	

Log2 Ratios: -6 -4 -2 0 2 4 6

Show detected proteins only
Show all proteins
Filter by category:
GO: amino acid transport

Proteins found: 1344

Enter (or paste) list of ORFs

Find ORFs

Test	Cutoff
q-Value	.005
p-Value	

	Signif	Direction	Applies To
red	yes	+	ratios, bars
yellow	no	n/a	bars
green	yes	−	ratios, bars
pink	yes	+	*p*- , *q*-Values
light green	yes	−	

Dot Plots Dot Plots

Protein	FnPgSg vs FnSg Log$_2$ Ratio	Log$_2$ Sum	*q*-Value	*p*-Value	Raw *FnPgSg*	Raw *FnSg*	Normalized *FnPgSg*	Normalized *FnSg*	Description
**FN0677**	-1.430	10.796	9.163e-5	7.743e-5	18	51	20.5929	66.3824	AAL94873.1| Hypothetical protein
					21	72	30.7818	72.0000	
**FN0678**	-0.043	11.635	1.3e-1	6.463e-1	51	48	58.3464	62.4776	AAL94874.1| Ser/Thr protein kinase
					36	52	52.7687	52.0000	
**FN0679**	-1.033	5.307							AAL94875.1| GTPase
					3	9	4.3974	9.0000	
**FN0680**	-1.031	6.370			6		6.8643		AAL94876.1| Ribulose-phosphate 3-epimerase
					4	13	5.8632	13.0000	
**FN0681**	-0.585	15.877	2.382e-3	5.769e-3	176	212	201.3523	275.9427	AAL94877.1| Transcriptional regulator, MarR family
					136	325	199.3486	325.0000	
**FN0682**	-0.750	9.358	8.597e-3	2.928e-2	23	28	26.3131	36.4453	AAL94878.1| Fibronectin-binding protein-like protein A
					9	30	13.1922	30.0000	
**FN0684**	-2.070	5.628			3	16	3.4321	20.8259	AAL94880.1| Prismane protein
						8		8.0000	
**FN0685**	-3.046	9.677	4.206e-3	1.193e-2	11	81	12.5845	105.4309	AAL94881.1| Sodium/pantothenate symporter
					5	59	7.3290	59.0000	
**FN0688**	-2.916	9.474	1.716e-5	7.086e-6	8	58	9.1524	75.4938	AAL94884.1| Hypothetical protein
					7	71	10.2606	71.0000	
**FN0689**	-0.571	14.637	3.858e-2	1.661e-1	120	196	137.2857	255.1168	AAL94885.1| Hypothetical protein
					85	134	124.5929	134.0000	
**FN0692**	-0.587	4.860				4		5.2065	AAL94888.1| Nitrogen regulation protein NIFR3
					3	8	4.3974	8.0000	
**FN0693**					11		12.5845		AAL94889.1| DNA mismatch repair protein mutS
**FN0694**	-0.341	11.743	4.346e-3	1.248e-2	41	49	46.9059	63.7792	AAL94890.1| S-layer protein
					39	68	57.1661	68.0000	
**FN0695**	-0.826	10.337	9.512e-4	1.814e-3	28	39	32.0333	50.7630	AAL94891.1| ABC transporter ATP-binding protein
					15	45	21.9870	45.0000	
**FN0697**	0.376	15.163	2.452e-3	5.992e-3	202	117	231.0976	152.2891	AAL94893.1| Alanyl-tRNA synthetase
					140	184	205.2118	184.0000	
**FN0699**	1.952	11.196	1.933e-4	2.085e-4	82	24	93.8119	31.2388	AAL94895.1| Protein translocase subunit secD
					66	18	96.7427	18.0000	

Log$_2$ Ratios: -6 -4 -2 0 2 4 6

Show detected proteins only
Show all proteins
Filter by category:
GO: amino acid transport

Proteins found: 1344

Enter (or paste) list of ORFs

Find ORFs

Test	Cutoff
q-Value	.005
p-Value	

	Signif	Direction	Applies To
red	yes	+	ratios, bars
yellow	no	n/a	bars
green	yes	−	ratios, bars
pink	yes	+	*p*-, *q*-Values
light green	yes	−	

Dot Plots Dot Plots

Protein	*FnPgSg vs FnSg* Log$_2$ Ratio	Log$_2$ Sum	*q*-Value	*p*-Value	Raw *FnPgSg*	Raw *FnSg*	Normalized *FnPgSg*	Normalized *FnSg*	Description
**FN0700**	2.084	7.994	3.144e-6	5.274e-7	28	5	32.0333	6.5081	AAL94896.1| Protein translocase subunit secF
					23	9	33.7134	9.0000	
**FN0701**	0.647	14.550	8.241e-5	6.706e-5	176	88	201.3523	114.5423	AAL94897.1| Methyltransferase
					127	133	186.1564	133.0000	
**FN0705**	-0.375	13.007	1.004e-3	1.947e-3	65	75	74.3631	97.6212	AAL94901.1| DNA polymerase I
					58	109	85.0163	109.0000	
**FN0706**	0.779	4.779			6		6.8643		AAL94902.1| Hypothetical cytosolic protein
						4		4.0000	
**FN0707**	0.185	6.275	1.028e-1	4.958e-1	10	5	11.4405	6.5081	AAL94903.1| Riboflavin kinase
					5	10	7.3290	10.0000	
**FN0710**	-0.088	11.295	1.796e-2	6.956e-2	44	41	50.3381	53.3663	AAL94906.1| Hypothetical protein
					32	50	46.9056	50.0000	
**FN0711**	-1.353	8.005	8.191e-3	2.767e-2	6	14	6.8643	18.2226	AAL94907.1| Phosphopantothenate--cysteine ligase
					9	33	13.1922	33.0000	
**FN0714**						8		10.4129	AAL94910.1| NADH oxidase
						16		16.0000	
**FN0715**	0.825	15.246	4.6e-3	1.348e-2	264	120	302.0285	156.1940	AAL94911.1| Hypothetical protein
					152	140	222.8014	140.0000	
**FN0716**	-1.066	10.920	1.651e-5	6.648e-6	25	51	28.6012	66.3824	AAL94912.1| hypothetical protein
					22	61	32.2476	61.0000	
**FN0717**	0.194	4.838			5		5.7202		AAL94913.1| Ribosomal small subunit pseudouridine synthase A
						5		5.0000	
**FN0720**	-0.714	12.072	4.008e-2	1.736e-1	46	40	52.6262	52.0647	AAL94916.1| Protein Translation Elongation Factor P (EF-P)
					34	116	49.8372	116.0000	
**FN0721**	0.185	10.583	4.316e-2	1.888e-1	32	28	36.6095	36.4453	AAL94917.1| Hypothetical protein
					32	37	46.9056	37.0000	
**FN0722**	-1.397	5.786			4	7	4.5762	9.1113	AAL94918.1| WD-repeat family protein
						15		15.0000	
**FN0723**									AAL94919.1| Hypothetical protein
						5		5.0000	
**FN0724**	-0.381	11.687	3.506e-2	1.488e-1	38	40	43.4738	52.0647	AAL94920.1| Flavodoxin
					39	79	57.1661	79.0000	

Log$_2$ Ratios: -6 -4 -2 0 2 4 6

Show detected proteins only
Show all proteins
Filter by category:
GO: amino acid transport

Proteins found: 1344

Enter (or paste) list of ORFs

Find ORFs

Test	Cutoff
q-Value	.005
p-Value	

	Signif	Direction	Applies To
(red)	yes	+	ratios, bars
(yellow)	no	n/a	bars
(green)	yes	−	ratios, bars
(pink)	yes	+	*p*- , *q*-Values
(light green)	yes	−	

Dot Plots | Dot Plots

Protein	FnPgSg vs FnSg Log$_2$ Ratio	Log$_2$ Sum	*q*-Value	*p*-Value	Raw *FnPgSg*	Raw *FnSg*	Normalized *FnPgSg*	Normalized *FnSg*	Description
FN0725	-1.565	8.611	3.401e-3	8.996e-3	6	20	6.8643	26.0323	AAL94921.1| Molybdopterin biosynthesis MoeB protein
					11	42	16.1238	42.0000	
FN0728	-0.136	10.938	2.482e-3	6.088e-3	38	36	43.4738	46.8582	AAL94924.1| Hypothetical protein
					28	46	41.0424	46.0000	
FN0729	0.747	14.060	2.999e-3	7.674e-3	146	92	167.0309	119.7487	AAL94925.1| Phosphoglycerate mutase
					117	82	171.4984	82.0000	
FN0731	0.387	8.681	4.482e-2	1.97e-1	20	18	22.8809	23.4291	AAL94927.1| Hypothetical protein
					16	12	23.4528	12.0000	
FN0733	1.763	13.115	3.459e-6	6.111e-7	147	34	168.1750	44.2550	AAL94929.1| Peptidase T
					122	58	178.8274	58.0000	
FN0734						3		3.9048	AAL94930.1| Fe-S oxidoreductase
						4		4.0000	
FN0735	1.765	12.009	7.314e-4	1.272e-3	89	32	101.8202	41.6517	AAL94931.1| Cell surface protein
					92	28	134.8535	28.0000	
FN0736	-1.116	7.366	1.799e-3	4.056e-3	5	16	5.7202	20.8259	AAL94932.1| Methyltransferase
					8	17	11.7264	17.0000	
FN0737					15		17.1607		AAL94933.1| Hypothetical protein
					10		14.6580		
FN0738	-0.757	9.805	2.537e-2	1.032e-1	21	39	24.0250	50.7630	AAL94934.1| Hypothetical exported 24-amino acid repeat protein
					15	27	21.9870	27.0000	
FN0739	1.323	16.007	3.31e-5	1.872e-5	338	144	386.6880	187.4328	AAL94935.1| Formiminotetrahydrofolate cyclodeaminase
					290	137	425.0816	137.0000	
FN0740	-1.050	17.835	4.295e-3	1.228e-2	308	625	352.3666	813.5104	AAL94936.1| Imidazolonepropionase
					218	578	319.5441	578.0000	
FN0741	0.326	18.975	7.731e-3	2.584e-2	659	551	753.9272	717.1907	AAL94937.1| Glutamate formiminotransferase
					582	565	853.0948	565.0000	
FN0742	0.116	11.614	1.398e-1	7.029e-1	66	35	75.5071	45.5566	AAL94938.1| unknown
					28	62	41.0424	62.0000	
FN0743	1.503	6.119	4.719e-5	3.226e-5	13	3	14.8726	3.9048	AAL94939.1| ATP-dependent helicase, DinG family
					9	6	13.1922	6.0000	
FN0745	-0.661	14.479	1.661e-2	6.357e-2	114	179	130.4214	232.9894	AAL94941.1| metal dependent phosphohydrolase
					75	147	109.9349	147.0000	

Log$_2$ Ratios: -6 -4 -2 0 2 4 6

Show detected proteins only
Show all proteins
Filter by category:
GO: amino acid transport

Proteins found: 1344

Enter (or paste) list of ORFs

Find ORFs

Test	Cutoff
q-Value	.005
p-Value	

	Signif	Direction	Applies To
red	yes	+	ratios, bars
yellow	no	n/a	bars
green	yes	−	ratios, bars
pink	yes	+	*p*- , *q*-Values
light green	yes	−	

Dot Plots Dot Plots

Protein	*FnPgSg vs FnSg* Log$_2$ Ratio	Log$_2$ Sum	*q*-Value	*p*-Value	Raw *FnPgSg*	Raw *FnSg*	Normalized *FnPgSg*	Normalized *FnSg*	Description	Log$_2$ Ratios (-6 -4 -2 0 2 4 6)
**FN0746**						5		6.5081	AAL94942.1| Hypothetical Metal-Binding Protein	
						21		21.0000		
**FN0749**									AAL94945.1| Hypothetical protein	
					4		5.8632			
**FN0750**	-2.551	9.274	5.349e-5	3.806e-5	9	48	10.2964	62.4776	AAL94946.1| Hypothetical protein	
					7	58	10.2606	58.0000		
**FN0751**	-0.934	8.976	4.039e-3	1.129e-2	13	20	14.8726	26.0323	AAL94947.1| L-asparaginase I	
					12	36	17.5896	36.0000		
**FN0752**	-1.692	7.280	3.767e-5	2.284e-5	7	16	8.0083	20.8259	AAL94948.1| Proline iminopeptidase	
					4	24	5.8632	24.0000		
**FN0753**	1.122	13.051	3.642e-7	3.4e-8	121	46	138.4297	59.8744	AAL94949.1| Glutamyl-tRNA(Gln) amidotransferase subunit B	
					91	65	133.3877	65.0000		
**FN0754**	0.386	13.660	2.115e-2	8.418e-2	130	63	148.7262	82.0018	AAL94950.1| Glutamyl-tRNA(Gln) amidotransferase subunit A	
					76	117	111.4007	117.0000		
**FN0755**	0.313	10.364	1.69e-2	6.487e-2	31	27	35.4655	35.1436	AAL94951.1| Glutamyl-tRNA(Gln) amidotransferase subunit C	
					31	30	45.4398	30.0000		
**FN0758**	-0.828	14.761	7.804e-3	2.615e-2	111	202	126.9893	262.9265	AAL94954.1| Rod shape-determining protein mreB	
					84	181	123.1271	181.0000		
**FN0761**	-0.412	8.116	3.712e-2	1.588e-1	15	18	17.1607	23.4291	AAL94957.1| Bvg accessory factor	
					8	15	11.7264	15.0000		
**FN0765**	0.609	3.779			4		4.5762		AAL94961.1| tRNA (5-methylaminomethyl -2-thiouridylate) -methyltransferase	
						3		3.0000		
**FN0766**									AAL94962.1| Large-conductance mechanosensitive channel	
					20		29.3160			
**FN0768**					4		4.5762		AAL94964.1| Hemin receptor	
**FN0771**						3		3.9048	AAL94967.1| Oxygen-independent coproporphyrinogen III oxidase	
						7		7.0000		
**FN0774**	2.651	11.725	1.671e-3	3.706e-3	105	18	120.1250	23.4291	AAL94970.1| Hypothetical cytosolic protein	
					117	23	171.4984	23.0000		
**FN0775**	-0.208	16.097	4.753e-3	1.406e-2	218	229	249.4023	298.0702	AAL94971.1| Aspartyl aminopeptidase	
					166	271	243.3226	271.0000		

Show detected proteins only
Show all proteins
Filter by category:
GO: amino acid transport

Proteins found: 1344

Enter (or paste) list of ORFs

Find ORFs

Test	Cutoff
q-Value	.005
p-Value	

	Signif	Direction	Applies To
red	yes	+	ratios, bars
yellow	no	n/a	bars
green	yes	−	ratios, bars
pink	yes	+	*p*- , *q*-Values
light green	yes	−	*p*- , *q*-Values

Dot Plots Dot Plots

Protein	*FnPgSg vs FnSg* Log$_2$ Ratio	Log$_2$ Sum	*q*-Value	*p*-Value	Raw *FnPgSg*	Raw *FnSg*	Normalized *FnPgSg*	Normalized *FnSg*	Description
**FN0776**	-0.809	12.740	1.938e-5	8.774e-6	58	86	66.3547	111.9390	AAL94972.1| Aspartate-ammonia ligase
					40	107	58.6319	107.0000	
**FN0777**	0.322	12.957	6.432e-3	2.06e-2	91	68	104.1083	88.5099	AAL94973.1| GTP-binding protein lepA
					65	71	95.2769	71.0000	
**FN0778**	-0.645	8.667				18		23.4291	AAL94974.1| Methyltransferase
					11	27	16.1238	27.0000	
**FN0779**						6		7.8097	AAL94975.1| Putative GTPases (G3E family)
						11		11.0000	
**FN0783**	1.127	23.890	3.135e-3	8.112e-3	5918	1871	6770.4723	2435.3246	AAL94979.1| acyl-CoA dehydrogenase
					3331	2900	4882.5752	2900.0000	
**FN0784**	0.243	22.490	3.765e-2	1.615e-1	1997	1910	2284.6626	2486.0876	AAL94980.1| Electron transfer flavoprotein beta-subunit
					2044	1976	2996.0924	1976.0000	
**FN0785**	-0.281	21.584	2.38e-2	9.609e-2	1458	1307	1668.0211	1701.2128	AAL94981.1| Electron transfer flavoprotein alpha-subunit
					1057	2208	1549.3491	2208.0000	
**FN0788**	-0.468	9.547	1.429e-2	5.336e-2	24	21	27.4571	27.3339	AAL94984.1| unknown
					13	37	19.0554	37.0000	
**FN0790**	-1.111	6.143			5	9	5.7202	11.7145	AAL94986.1| Xylose repressor
						13		13.0000	
**FN0791**	-0.157	18.209	6.807e-2	3.146e-1	459	513	525.1177	667.7293	AAL94987.1| Histidine ammonia-lyase
					353	495	517.4269	495.0000	
**FN0792**	-0.579	20.481	3.913e-5	2.429e-5	823	1095	941.5510	1425.2701	AAL94988.1| Urocanate hydratase
					708	1532	1037.7854	1532.0000	
**FN0793**									AAL94989.1| Sodium/glutamate symport carrier protein
					22		32.2476		
**FN0794**	-1.545	7.548			7	19	8.0083	24.7307	AAL94990.1| Hypothetical protein
						22		22.0000	
**FN0796**	-0.335	14.652	5.547e-4	8.644e-4	128	144	146.4381	187.4328	AAL94992.1| Pyruvate,phosphate dikinase
					95	173	139.2509	173.0000	
**FN0798**	-1.012	9.262	4.355e-2	1.907e-1	10	41	11.4405	53.3663	AAL94994.1| Fructose-1,6-bisphosphatase
					16	17	23.4528	17.0000	
**FN0799**	-0.614	8.403			13	15	14.8726	19.5242	AAL94995.1| Isoamylase
						26		26.0000	

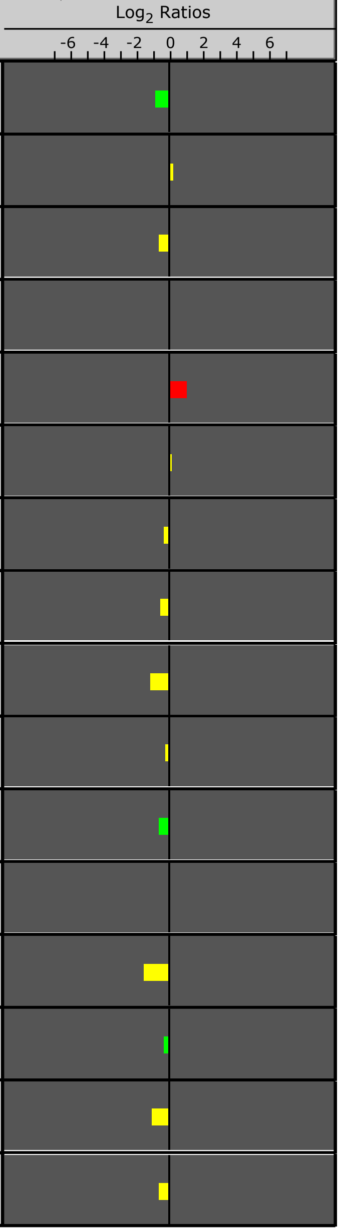

Show detected proteins only

Show all proteins

Filter by category:

GO: amino acid transport

Proteins found: 1344

Enter (or paste) list of ORFs

Find ORFs

Test	Cutoff
q-Value	.005
p-Value	

	Signif	Direction	Applies To
(red)	yes	+	ratios, bars
(yellow)	no	n/a	bars
(green)	yes	−	ratios, bars
(pink)	yes	+	*p*- , *q*-Values
(light green)	yes	−	

Dot Plots Dot Plots

Protein	FnPgSg vs FnSg Log$_2$ Ratio	Log$_2$ Sum	*q*-Value	*p*-Value	Raw *FnPgSg*	Raw *FnSg*	Normalized *FnPgSg*	Normalized *FnSg*	Description
FN0800	1.264	12.332	1.287e-3	2.673e-3	110	32	125.8452	41.6517	AAL94996.1| Amino acid-binding protein
					66	51	96.7427	51.0000	
FN0801						18		23.4291	AAL94997.1| Amino acid transport ATP-binding protein
						22		22.0000	
FN0803	0.540	11.094	4.987e-3	1.498e-2	55	25	62.9226	32.5404	AAL94999.1| Cytochrome C-TYPE biogenesis protein ccdA
					34	45	49.8372	45.0000	
FN0805	-1.606	5.937	1.328e-4	1.252e-4	4	11	4.5762	14.3178	AAL95001.1| Hypothetical protein
					3	13	4.3974	13.0000	
FN0806	0.130	12.620	6.766e-2	3.125e-1	81	62	92.6678	80.7002	AAL95002.1| SpoIID homolog
					50	71	73.2899	71.0000	
FN0807	1.138	8.846	1.433e-4	1.392e-4	30	13	34.3214	16.9210	AAL95003.1| 3-deoxy-manno-octulosonate cytidylyltransferase
					20	12	29.3160	12.0000	
FN0808	-0.701	12.023	5.939e-4	9.545e-4	50	68	57.2024	88.5099	AAL95004.1| Phosphoglycerate mutase
					30	76	43.9740	76.0000	
FN0809	-0.346	7.654			11		12.5845		AAL95005.1| 23S rRNA methyltransferase
						16		16.0000	
FN0810	-1.671	10.229	4.747e-2	2.101e-1	16	12	18.3048	15.6194	AAL95006.1| Low-specificity threonine aldolase
					14	108	20.5212	108.0000	
FN0811						4		5.2065	AAL95007.1| Hypothetical protein
						5		5.0000	
FN0813	-2.588	9.291	1.507e-4	1.494e-4	14	49	16.0167	63.7792	AAL95009.1| Transcriptional regulator, TetR family
					3	59	4.3974	59.0000	
FN0814	4.207	11.864	3.637e-5	2.161e-5	241	8	275.7154	10.4129	AAL95010.1| Propionate CoA-transferase
					170	18	249.1858	18.0000	
FN0815					61		69.7869		AAL95011.1| Propionate permease
					41		60.0977		
FN0816					70		80.0833		AAL95012.1| dehydrogenase with MaoC-like domain
					64		93.8111		
FN0818	2.016	18.019	2.237e-5	1.089e-5	852	224	974.7284	291.5621	AAL95014.1| DNA-binding protein HU
					749	221	1097.8832	221.0000	
FN0819	0.437	12.038	1.701e-2	6.535e-2	55	38	62.9226	49.4614	AAL95015.1| Tetratricopeptide repeat family protein
					60	62	87.9479	62.0000	

Log$_2$ Ratios: -6 -4 -2 0 2 4 6

Show detected proteins only
Show all proteins
Filter by category:
GO: amino acid transport

Proteins found: 1344

Enter (or paste) list of ORFs

Find ORFs

Test	Cutoff
q-Value	.005
p-Value	

	Signif	Direction	Applies To
red	yes	+	ratios, bars
yellow	no	n/a	bars
green	yes	−	ratios, bars
pink	yes	+	*p*- , *q*-Values
light green	yes	−	*p*- , *q*-Values

Dot Plots Dot Plots

Protein	FnPgSg vs FnSg Log$_2$ Ratio	Log$_2$ Sum	*q*-Value	*p*-Value	Raw *FnPgSg*	Raw *FnSg*	Normalized *FnPgSg*	Normalized *FnSg*	Description	Log$_2$ Ratios (-6 -4 -2 0 2 4 6)
**FN0820**	0.017	14.440	1.768e-1	9.29e-1	120	91	137.2857	118.4471	AAL95016.1| Mercuric reductase	
					111	178	162.7036	178.0000		
**FN0821**	0.764	10.566	4.059e-3	1.136e-2	40	29	45.7619	37.7469	AAL95017.1| Hypothetical protein	
					38	22	55.7003	22.0000		
**FN0823**	-2.287	11.221	8.633e-6	2.239e-6	22	79	25.1690	102.8277	AAL95019.1| GTP-binding protein hflX	
					13	113	19.0554	113.0000		
**FN0824**						4		5.2065	AAL95020.1| hypothetical cytosolic protein	
**FN0825**	0.321	10.063	2.875e-2	1.187e-1	37	25	42.3298	32.5404	AAL95021.1| Hypothetical cytosolic protein	
					21	26	30.7818	26.0000		
**FN0826**	0.942	9.440	4.821e-3	1.433e-2	28	20	32.0333	26.0323	AAL95022.1| periplasmic component of efflux system	
					28	12	41.0424	12.0000		
**FN0827**	-0.986	9.627	1.874e-3	4.262e-3	17	27	19.4488	35.1436	AAL95023.1| ABC transporter ATP-binding protein	
					14	44	20.5212	44.0000		
**FN0828**	0.217	5.341			6	6	6.8643	7.8097	AAL95024.1| ABC transporter permease protein	
						4		4.0000		
**FN0830**	-2.563	10.218	3.223e-4	4.247e-4	12	59	13.7286	76.7954	AAL95026.1| Hypothetical protein	
					10	91	14.6580	91.0000		
**FN0832**	1.714	11.452	1.427e-4	1.383e-4	92	18	105.2524	23.4291	AAL95028.1| Hypothetical protein	
					59	35	86.4821	35.0000		
**FN0833**	-0.946	8.807	1.439e-2	5.376e-2	10	29	11.4405	37.7469	AAL95029.1| Hypothetical protein	
					13	21	19.0554	21.0000		
**FN0834**	0.820	8.155	1.883e-3	4.288e-3	20	8	22.8809	10.4129	AAL95030.1| Hypothetical Exported Protein	
					15	15	21.9870	15.0000		
**FN0836**	0.453	8.325	7.05e-2	3.273e-1	11	12	12.5845	15.6194	AAL95032.1| Hypothetical protein	
					20	15	29.3160	15.0000		
**FN0837**									AAL95033.1| Integrase/recombinase	
						8		8.0000		
**FN0846**	-0.778	12.163	1.024e-3	2e-3	43	74	49.1940	96.3196	AAL95042.1| Hypothetical Exported Protein	
					37	81	54.2345	81.0000		
**FN0847**	-0.708	6.427	4.613e-3	1.353e-2	5	9	5.7202	11.7145	AAL95043.1| TPR-repeat-containing proteins	
					6	12	8.7948	12.0000		

Show detected proteins only
Show all proteins
Filter by category:
GO: amino acid transport

Proteins found: 1344

Enter (or paste) list of ORFs

Find ORFs

Test	Cutoff
q-Value	.005
p-Value	

	Signif	Direction	Applies To
red	yes	+	ratios, bars
yellow	no	n/a	bars
green	yes	−	ratios, bars
pink	yes	+	*p*- , *q*-Values
light green	yes	−	

Dot Plots Dot Plots

Protein	FnPgSg vs FnSg: Log_2 Ratio	Log_2 Sum	*q*-Value	*p*-Value	Raw: *FnPgSg*	Raw: *FnSg*	Normalized: *FnPgSg*	Normalized: *FnSg*	Description
FN0849	-1.352	10.231	4.431e-4	6.356e-4	20	39	22.8809	50.7630	AAL95045.1| 8-amino-7-oxononanoate synthase
					14	60	20.5212	60.0000	
FN0850	-2.342	8.803	3.762e-5	2.279e-5	10	37	11.4405	48.1598	AAL95046.1| Hypothetical cytosolic protein
					5	47	7.3290	47.0000	
FN0853	-1.697	11.969	6.011e-4	9.718e-4	32	96	36.6095	124.9552	AAL95049.1| Glycogen synthase
					23	103	33.7134	103.0000	
FN0854	-1.267	13.371	5.758e-5	4.202e-5	66	114	75.5071	148.3843	AAL95050.1| Glucose-1-phosphate adenylyltransferase
					39	171	57.1661	171.0000	
FN0855	-1.942	14.519	3.09e-5	1.691e-5	70	238	80.0833	309.7847	AAL95051.1| Glucose-1-phosphate adenylyltransferase
					52	291	76.2215	291.0000	
FN0856	-1.598	12.873	7.824e-5	6.253e-5	55	124	62.9226	161.4005	AAL95052.1| 1,4-alpha-glucan branching enzyme
					25	140	36.6450	140.0000	
FN0857	-1.089	16.087	3.235e-6	5.507e-7	165	304	188.7678	395.6914	AAL95053.1| Glycogen phosphorylase
					118	374	172.9642	374.0000	
FN0858	-3.346	8.570	6.341e-3	2.025e-2	3	31	3.4321	40.3501	AAL95054.1| 4-alpha-glucanotransferase
					6	84	8.7948	84.0000	
FN0865	-3.136	12.360	3.176e-6	5.356e-7	12	172	13.7286	223.8780	AAL95061.1| unknown
					24	206	35.1792	206.0000	
FN0867	0.779	12.388	2.152e-4	2.424e-4	78	39	89.2357	50.7630	AAL95063.1| Long-chain-fatty-acid--CoA ligase
					70	61	102.6059	61.0000	
FN0868									AAL95064.1| ATPases of the PP superfamily
						8		8.0000	
FN0869	-1.266	4.824			3	5	3.4321	6.5081	AAL95065.1| Hydrolase (HAD superfamily)
						10		10.0000	
FN0870					10		11.4405		AAL95066.1| Rhodanese-related sulfurtransferases
					9		13.1922		
FN0871	-0.015	6.743			9	6	10.2964	7.8097	AAL95067.1| 3-dehydroquinate synthase
						13		13.0000	
FN0873	-1.318	10.273	3.073e-3	7.913e-3	21	50	24.0250	65.0808	AAL95069.1| Protease IV
					14	46	20.5212	46.0000	
FN0874									AAL95070.1| Phosphohydrolase (MUTT/NUDIX family protein)
						5		5.0000	

Log_2 Ratios: -6 -4 -2 0 2 4 6

Show detected proteins only
Show all proteins
Filter by category:
GO: amino acid transport

Proteins found: 1344

Enter (or paste) list of ORFs

Find ORFs

Test	Cutoff
q-Value	.005
p-Value	

Colour	Signif	Direction	Applies To
red	yes	+	ratios, bars
yellow	no	n/a	bars
green	yes	−	ratios, bars
pink	yes	+	*p*-, *q*-Values
light green	yes	−	*p*-, *q*-Values

Dot Plots Dot Plots

Protein	FnPgSg vs FnSg Log$_2$ Ratio	Log$_2$ Sum	*q*-Value	*p*-Value	Raw *FnPgSg*	Raw *FnSg*	Normalized *FnPgSg*	Normalized *FnSg*	Description
FN0875	-0.449	5.481			5	6	5.7202	7.8097	AAL95071.1| 23S rRNA methyltransferase
FN0878	0.175	9.505	2.076e-2	8.236e-2	27	19	30.8893	24.7307	AAL95074.1| Transcriptional regulator, GntR family
					18	26	26.3844	26.0000	
FN0884						3		3.9048	AAL95079.1| Hemin transport system permease protein hmuU
FN0886					3		3.4321		AAL95082.1| Hemin receptor
FN0887	0.378	10.791	1.65e-2	6.304e-2	48	26	54.9143	33.8420	AAL95083.1| Oligoendopeptidase F
					28	40	41.0424	40.0000	
FN0888					21		24.0250		AAL95084.1| Uracil permease
					26		38.1108		
FN0889									AAL95085.1| hypothetical protein
						9		9.0000	
FN0892						10		13.0162	AAL95088.1| Phosphoserine phosphatase
						5		5.0000	
FN0893	-0.174	7.440			14		16.0167		AAL95089.1| Hypothetical protein
					6	14	8.7948	14.0000	
FN0896	0.631	7.565	1.248e-2	4.56e-2	12	7	13.7286	9.1113	AAL95092.1| Hypothetical protein
					14	13	20.5212	13.0000	
FN0898						6		7.8097	AAL95094.1| Hypothetical protein
						6		6.0000	
FN0900									AAL95096.1| Metal dependent hydrolase
						20		20.0000	
FN0901						10		13.0162	AAL95097.1| DNA polymerase, bacteriophage-type
						16		16.0000	
FN0902					6		6.8643		AAL95098.1| 5-formyltetrahydrofolate cyclo-ligase
					8		11.7264		
FN0903	-0.524	8.788	7.871e-4	1.408e-3	14	18	16.0167	23.4291	AAL95099.1| Polysialic acid capsule expression protein kpsF
					13	27	19.0554	27.0000	
FN0905					11		12.5845		AAL95101.1| Hypothetical protein
					10		14.6580		

Log$_2$ Ratios: -6 -4 -2 0 2 4 6

Show detected proteins only
Show all proteins
Filter by category:
GO: amino acid transport

Proteins found: 1344

Enter (or paste) list of ORFs

Find ORFs

Test	Cutoff
q-Value	.005
p-Value	

Signif	Direction	Applies To
yes	+	ratios, bars
no	n/a	bars
yes	−	ratios, bars
yes	+	*p*- , *q*-Values
yes	−	

Dot Plots Dot Plots

Protein	FnPgSg vs FnSg Log2 Ratio	Log2 Sum	*q*-Value	*p*-Value	Raw *FnPgSg*	Raw *FnSg*	Normalized *FnPgSg*	Normalized *FnSg*	Description
FN0906	-1.555	8.858	7.235e-5	5.64e-5	13	26	14.8726	33.8420	AAL95102.1| Glycerol-3-phosphate dehydrogenase [NAD(P)+]
					7	40	10.2606	40.0000	
FN0908	0.218	8.099	9.648e-2	4.619e-1	12	9	13.7286	11.7145	AAL95104.1| Tpl protein
					15	19	21.9870	19.0000	
FN0910						3		3.9048	AAL95106.1| Nicotinate-nucleotide--dimethylbenzimidazole phosphoribosyltransferase
						9		9.0000	
FN0911	0.246	5.757			7	5	8.0083	6.5081	AAL95107.1| Alpha-ribazole-5'-phosphate phosphatase
						7		7.0000	
FN0912						9		11.7145	AAL95108.1| Cobalamin [5'-phosphate] synthase
						7		7.0000	
FN0913	-1.221	4.779			3		3.4321		AAL95109.1| Cobinamide kinase
						8		8.0000	
FN0915	-0.964	10.857	5.083e-3	1.535e-2	27	54	30.8893	70.2873	AAL95111.1| PTS system, N-acetylglucosamine-specific IIA component
					21	50	30.7818	50.0000	
FN0916	0.683	15.778	5.564e-3	1.721e-2	301	153	344.3583	199.1473	AAL95112.1| Hypothetical Exported Protein
					175	175	256.5148	175.0000	
FN0917	-1.570	5.843				7		9.1113	AAL95113.1| Hypothetical protein
					3	17	4.3974	17.0000	
FN0920					7		8.0083		AAL95116.1| Protease HTPX
					5		7.3290		
FN0921	-1.416	8.545	2.97e-4	3.811e-4	13	27	14.8726	35.1436	AAL95117.1| Hypothetical protein
					6	28	8.7948	28.0000	
FN0922					3		3.4321		AAL95118.1| Homoserine kinase
FN0924									AAL95120.1| Hypothetical protein
					3		4.3974		
FN0925	-0.082	6.592	1.654e-1	8.574e-1	9	4	10.2964	5.2065	AAL95121.1| Hypothetical protein
					6	15	8.7948	15.0000	
FN0926	-1.947	8.625	5.59e-5	4.037e-5	10	30	11.4405	39.0485	AAL95122.1| GTP pyrophosphokinase
					6	39	8.7948	39.0000	
FN0928	-0.231	5.752	1.363e-1	6.826e-1	8	3	9.1524	3.9048	AAL95124.1| O-sialoglycoprotein endopeptidase
					3	12	4.3974	12.0000	

Log2 Ratios: -6 -4 -2 0 2 4 6

Show detected proteins only
Show all proteins
Filter by category:
GO: amino acid transport

Proteins found: 1344

Enter (or paste) list of ORFs

Find ORFs

Test	Cutoff
q-Value	.005
p-Value	

	Signif	Direction	Applies To
red	yes	+	ratios, bars
yellow	no	n/a	bars
green	yes	−	ratios, bars
pink	yes	+	*p*- , *q*-Values
light green	yes	−	*p*- , *q*-Values

Dot Plots Dot Plots

Protein	*FnPgSg vs FnSg* Log$_2$ Ratio	Log$_2$ Sum	*q*-Value	*p*-Value	Raw *FnPgSg*	Raw *FnSg*	Normalized *FnPgSg*	Normalized *FnSg*	Description
**FN0929**	-1.635	7.908				22		28.6356	AAL95125.1| ATP/GTP hydrolase
					6	26	8.7948	26.0000	
**FN0930**									AAL95126.1| Glycerol-3-phosphate cytidylyltransferase
						5		5.0000	
**FN0932**	-1.489	6.828	1.288e-2	4.732e-2	6	9	6.8643	11.7145	AAL95128.1| Hypothetical protein
					4	24	5.8632	24.0000	
**FN0933**					3		3.4321		AAL95129.1| 3-phosphoshikimate 1-carboxyvinyltransferase
**FN0934**						30		39.0485	AAL95130.1| Chorismate synthase
						25		25.0000	
**FN0938**						42		54.6679	AAL95134.1| Hypothetical protein
						37		37.0000	
**FN0940**	-0.777	8.022	3.662e-3	9.905e-3	10	14	11.4405	18.2226	AAL95136.1| Hypothetical protein
					9	24	13.1922	24.0000	
**FN0941**	-0.490	10.596	2.751e-2	1.13e-1	26	44	29.7452	57.2711	AAL95137.1| Gamma-glutamyltranspeptidase
					25	36	36.6450	36.0000	
**FN0943**	-2.274	6.951	6.423e-3	2.057e-2	5	13	5.7202	16.9210	AAL95139.1| Sensory Transduction Protein Kinase
					3	32	4.3974	32.0000	
**FN0947**	1.217	12.376	2.641e-4	3.269e-4	88	32	100.6762	41.6517	AAL95143.1| Hypothetical protein
					83	54	121.6613	54.0000	
**FN0949**	1.790	13.586	6.204e-6	1.333e-6	185	44	211.6488	57.2711	AAL95145.1| DNA helicase
					137	62	200.8144	62.0000	
**FN0951**									AAL95147.1| Precorrin-3B C17-methyltransferase
						7		7.0000	
**FN0957**	1.354	7.881	5.589e-3	1.731e-2	16	4	18.3048	5.2065	AAL95153.1| Precorrin-4 C11-methyltransferase
					21	14	30.7818	14.0000	
**FN0958**									AAL95154.1| unknown
						7		7.0000	
**FN0959**	1.692	6.336			18		20.5929		AAL95155.1| Precorrin-2 C20-methyltransferase
					8	5	11.7264	5.0000	
**FN0961**									AAL95157.1| Hypothetical protein
						26		26.0000	

Log$_2$ Ratios: -6 -4 -2 0 2 4 6

Show detected proteins only
Show all proteins
Filter by category:
GO: amino acid transport

Proteins found: 1344

Enter (or paste) list of ORFs

Find ORFs

Test	Cutoff
q-Value	.005
p-Value	

	Signif	Direction	Applies To
red	yes	+	ratios, bars
yellow	no	n/a	bars
green	yes	−	ratios, bars
pink	yes	+	*p*- , *q*-Values
light green	yes	−	

Dot Plots Dot Plots

Protein	*FnPgSg* vs *FnSg* Log$_2$ Ratio	Log$_2$ Sum	*q*-Value	*p*-Value	Raw *FnPgSg*	Raw *FnSg*	Normalized *FnPgSg*	Normalized *FnSg*	Description
FN0962	0.638	8.295	9.328e-3	3.223e-2	22	8	25.1690	10.4129	AAL95158.1| Hypothetical cytosolic protein
					13	18	19.0554	18.0000	
FN0964	-0.223	5.970				7		9.1113	AAL95160.1| Precorrin-8W decarboxylase
					5	8	7.3290	8.0000	
FN0965	-0.366	11.523	5.008e-2	2.233e-1	31	57	35.4655	74.1921	AAL95161.1| D-3-phosphoglycerate dehydrogenase
					41	49	60.0977	49.0000	
FN0966									AAL95162.1| Precorrin-6Y C5,15-methyltransferase (decarboxylating)
						5		5.0000	
FN0967	-0.806	4.364			3		3.4321		AAL95163.1| CbiD protein
						6		6.0000	
FN0970	0.871	7.724	8.098e-3	2.731e-2	19	5	21.7369	6.5081	AAL95166.1| Precorrin-8X methylmutase
					12	15	17.5896	15.0000	
FN0971						4		5.2065	AAL95167.1| hypothetical cytosolic protein
FN0972						8		10.4129	AAL95168.1| Cobyrinic acid a,c-diamide synthase
						32		32.0000	
FN0976	-0.258	11.269	6.759e-2	3.121e-1	41	32	46.9059	41.6517	AAL95172.1| Hypothetical protein
					30	67	43.9740	67.0000	
FN0977	-0.998	9.556	4.871e-3	1.453e-2	16	35	18.3048	45.5566	AAL95173.1| Cobyric acid synthase
					14	32	20.5212	32.0000	
FN0981	1.271	13.175	2.399e-4	2.832e-4	142	59	162.4547	76.7954	AAL95177.1| Phosphoribosylamine--glycine ligase
					93	47	136.3193	47.0000	
FN0982	0.088	15.633	8.976e-2	4.266e-1	223	180	255.1226	234.2910	AAL95178.1| Phosphoribosylaminoimidazolecarboxamide formyltransferase
					143	203	209.6092	203.0000	
FN0983	-0.620	14.119	1.557e-3	3.386e-3	101	115	115.5488	149.6859	AAL95179.1| Hypothetical protein
					68	181	99.6743	181.0000	
FN0984	-2.590	9.313	3.545e-5	2.077e-5	9	49	10.2964	63.7792	AAL95180.1| Tetracenomycin polyketide synthesis O-methyltransferase tcmP
					7	60	10.2606	60.0000	
FN0985	-0.055	8.334	1.523e-1	7.774e-1	18	12	20.5929	15.6194	AAL95181.1| Phosphoribosylglycinamide formyltransferase
					10	21	14.6580	21.0000	
FN0986	-0.175	15.321	1.11e-1	5.399e-1	151	119	172.7512	154.8924	AAL95182.1| Phosphoribosylformylglycinamidine cyclo-ligase
					142	275	208.1434	275.0000	

Log$_2$ Ratios

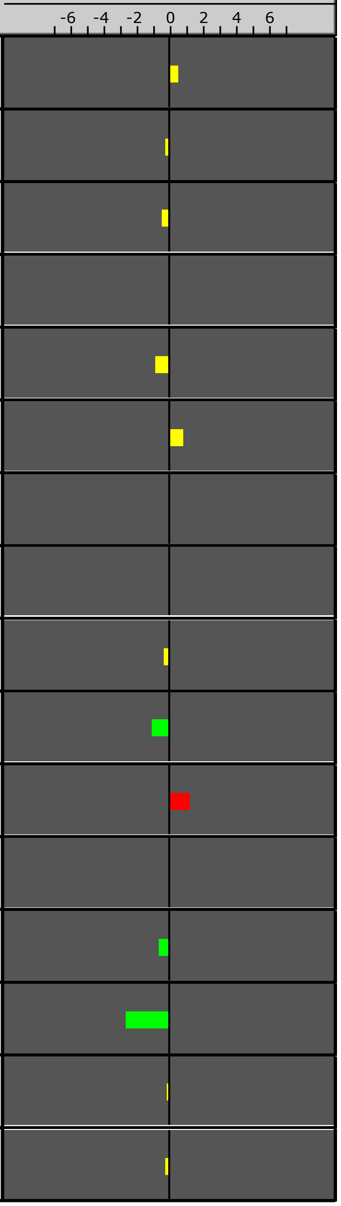

Show detected proteins only
Show all proteins
Filter by category:
GO: amino acid transport

Proteins found: 1344

Enter (or paste) list of ORFs

Find ORFs

Test	Cutoff
q-Value	.005
p-Value	

	Signif	Direction	Applies To
red	yes	+	ratios, bars
yellow	no	n/a	bars
green	yes	–	ratios, bars
pink	yes	+	*p*- , *q*-Values
light green	yes	–	

Dot Plots Dot Plots

Protein	FnPgSg vs FnSg Log_2 Ratio	Log_2 Sum	*q*-Value	*p*-Value	Raw *FnPgSg*	Raw *FnSg*	Normalized *FnPgSg*	Normalized *FnSg*	Description
FN0987	-0.870	13.680	1.512e-4	1.5e-4	70	112	80.0833	145.7811	AAL95183.1| Amidophosphoribosyltransferase
					61	164	89.4137	164.0000	
FN0988	0.662	16.530	2.478e-3	6.075e-3	351	156	401.5606	203.0522	AAL95184.1| Phosphoribosylamidoimidazole-succinocarboxamide synthase
					254	286	372.3128	286.0000	
FN0989	0.162	15.499	1.027e-1	4.95e-1	184	116	210.5047	150.9875	AAL95185.1| Phosphoribosylaminoimidazole carboxylase catalytic subunit
					167	256	244.7884	256.0000	
FN0990	-0.554	21.187	4.339e-5	2.86e-5	1063	1412	1216.1223	1837.8826	AAL95186.1| Phosphoribosylformylglycinamidine synthase
					910	1907	1333.8767	1907.0000	
FN0991	0.329	9.956	8.242e-3	2.788e-2	31	24	35.4655	31.2388	AAL95187.1| CDP-diacylglycerol--serine O-phosphatidyltransferase
					24	25	35.1792	25.0000	
FN0992	-0.278	9.830	1.901e-2	7.43e-2	21	28	24.0250	36.4453	AAL95188.1| ADP-heptose:LPS heptosyltransferase II
					21	30	30.7818	30.0000	
FN0994	0.649	13.995	7.483e-3	2.481e-2	140	60	160.1666	78.0970	AAL95190.1| Hypothetical protein
					109	126	159.7720	126.0000	
FN0997	-1.049	11.106	2.015e-3	4.662e-3	34	60	38.8976	78.0970	AAL95193.1| Hypothetical protein
					18	57	26.3844	57.0000	
FN0998	-1.839	14.893	1.011e-5	2.998e-6	69	248	78.9393	322.8009	AAL95194.1| Dipeptide-binding protein
					72	337	105.5375	337.0000	
FN0999	-2.326	8.355	1.002e-5	2.944e-6	9	30	10.2964	39.0485	AAL95195.1| Deblocking aminopeptidase
					4	42	5.8632	42.0000	
FN1000					41		46.9059		AAL95196.1| Biotin synthase
					34		49.8372		
FN1001	-0.909	9.988	4.845e-4	7.161e-4	24	31	27.4571	40.3501	AAL95197.1| Dethiobiotin synthetase
					13	47	19.0554	47.0000	
FN1002	0.643	10.051	1.64e-3	3.618e-3	34	17	38.8976	22.1275	AAL95198.1| Adenosylmethionine-8-amino-7-oxononanoate aminotransferase
					29	30	42.5082	30.0000	
FN1003	-1.332	12.510	3.394e-4	4.533e-4	47	84	53.7702	109.3358	AAL95199.1| Outer membrane protein P1 precursor
					29	133	42.5082	133.0000	
FN1004	-0.657	8.123	5.251e-2	2.356e-1	13	23	14.8726	29.9372	AAL95200.1| Transcriptional regulator, TetR family
					8	12	11.7264	12.0000	
FN1005	-0.678	10.509	1.288e-2	4.73e-2	22	45	25.1690	58.5727	AAL95201.1| Hypothetical protein
					24	38	35.1792	38.0000	

Log_2 Ratios: -6 -4 -2 0 2 4 6

Show detected proteins only
Show all proteins
Filter by category:
GO: amino acid transport

Proteins found: 1344

Enter (or paste) list of ORFs

Find ORFs

Test	Cutoff
q-Value	.005
p-Value	

Signif	Direction	Applies To
yes	+	ratios, bars
no	n/a	bars
yes	−	ratios, bars
yes	+	*p*- , *q*-Values
yes	−	

Dot Plots Dot Plots

Protein	*FnPgSg vs FnSg* Log₂ Ratio	Log₂ Sum	*q*-Value	*p*-Value	Raw *FnPgSg*	Raw *FnSg*	Normalized *FnPgSg*	Normalized *FnSg*	Description
FN1006									AAL95202.1| Acetyltransferase
						12		12.0000	
FN1010	0.833	14.222	6.179e-4	1.013e-3	165	90	188.7678	117.1455	AAL95206.1| Hypothetical cytosolic protein
					123	90	180.2932	90.0000	
FN1011	-1.016	6.119				9		11.7145	AAL95207.1| MGPA protein
					4	12	5.8632	12.0000	
FN1012	-1.711	8.880	1.899e-3	4.333e-3	12	25	13.7286	32.5404	AAL95208.1| HPR(Ser) kinase
					7	46	10.2606	46.0000	
FN1014	-2.216	7.871	6.417e-3	2.054e-2	6	33	6.8643	42.9533	AAL95210.1| Folylpolyglutamate synthase
					5	23	7.3290	23.0000	
FN1015	0.676	7.895	7.405e-3	2.448e-2	20	8	22.8809	10.4129	AAL95211.1| 5'-methylthioadenosine nucleosidase
					11	14	16.1238	14.0000	
FN1016	-2.227	7.881	4.995e-4	7.466e-4	6	28	6.8643	36.4453	AAL95212.1| Lipid A biosynthesis lauroyl acyltransferase
					5	30	7.3290	30.0000	
FN1017	0.499	13.054	7.066e-3	2.31e-2	93	70	106.3964	91.1132	AAL95213.1| Hypothetical Exported Protein
					77	64	112.8665	64.0000	
FN1019	0.287	23.883	8.102e-3	2.732e-2	3484	2651	3985.8610	3450.5855	AAL95215.1| 3-hydroxybutyryl-CoA dehydrogenase
					3209	3671	4703.7477	3671.0000	
FN1020	-0.171	17.801	1.036e-1	5.001e-1	331	478	378.6797	622.1727	AAL95216.1| 3-hydroxybutyryl-CoA dehydratase
					356	392	521.8243	392.0000	
FN1022					9		10.2964		AAL95218.1| Calcium-transporting ATPase
					12		17.5896		
FN1023	-1.530	7.533			7	24	8.0083	31.2388	AAL95219.1| 5-Nitroimidazole antibiotic resistance protein
						15		15.0000	
FN1024	0.176	20.436	1.063e-1	5.144e-1	1379	726	1577.6413	944.9736	AAL95220.1| DNA-binding protein HU
					651	1296	954.2349	1296.0000	
FN1025	0.797	7.441			15		17.1607		AAL95221.1| Guanine-hypoxanthine permease
					12	10	17.5896	10.0000	
FN1026									AAL95222.1| tRNA pseudouridine synthase A
						6		6.0000	
FN1028	0.622	9.997	9.951e-3	3.482e-2	36	25	41.1857	32.5404	AAL95224.1| Deoxyuridine 5'-triphosphate nucleotidohydrolase
					26	19	38.1108	19.0000	

Log₂ Ratios

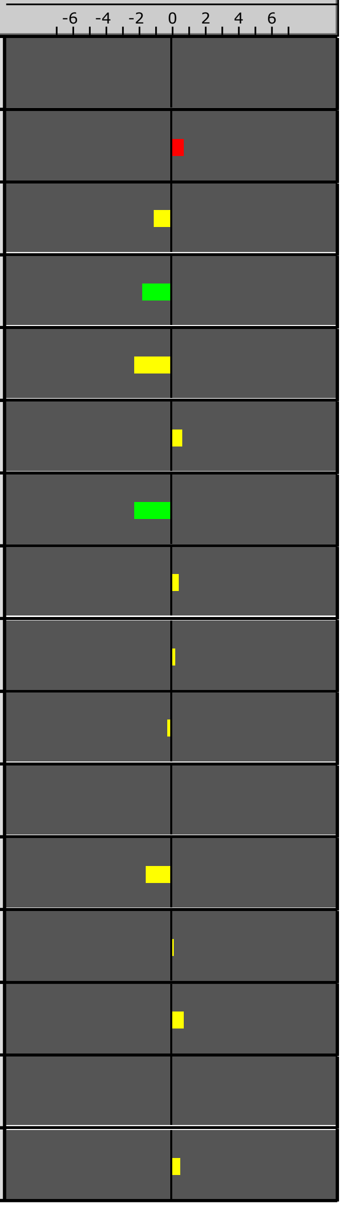

Show detected proteins only
Show all proteins
Filter by category:
GO: amino acid transport

Proteins found: 1344

Enter (or paste) list of ORFs

Find ORFs

Test	Cutoff
q-Value	.005
p-Value	

Color	Signif	Direction	Applies To
red	yes	+	ratios, bars
yellow	no	n/a	bars
green	yes	–	ratios, bars
pink	yes	+	*p*- , *q*-Values
light green	yes	–	*p*- , *q*-Values

Dot Plots | Dot Plots

Protein	*FnPgSg vs FnSg* Log$_2$ Ratio	Log$_2$ Sum	*q*-Value	*p*-Value	Raw *FnPgSg*	Raw *FnSg*	Normalized *FnPgSg*	Normalized *FnSg*	Description
FN1029	0.488	11.815	1.641e-2	6.267e-2	73	41	83.5155	53.3663	AAL95225.1| Zinc protease
					40	48	58.6319	48.0000	
FN1030	-1.171	7.560			8	14	9.1524	18.2226	AAL95226.1| Hypothetical membrane-spanning protein
						23		23.0000	
FN1031	-1.323	5.596							AAL95227.1| Hypothetical membrane-spanning protein
					3	11	4.3974	11.0000	
FN1033	-0.993	10.538	4.381e-3	1.262e-2	26	49	29.7452	63.7792	AAL95229.1| Methyltransferase
					17	45	24.9186	45.0000	
FN1034	-1.092	5.480			4	5	4.5762	6.5081	AAL95230.1| Transcriptional regulator, TetR family
						13		13.0000	
FN1035									AAL95231.1| Iron-sulfur flavoprotein
						3		3.0000	
FN1036						3		3.9048	AAL95232.1| Hypothetical protein
						4		4.0000	
FN1037	-2.322	5.880			3	11	3.4321	14.3178	AAL95233.1| Hypothetical cytosolic protein
						20		20.0000	
FN1041	-0.779	7.337	4.189e-3	1.186e-2	8	11	9.1524	14.3178	AAL95237.1| Acetyltransferase
					7	19	10.2606	19.0000	
FN1042	0.485	8.826	2.127e-2	8.474e-2	21	10	24.0250	13.0162	AAL95238.1| S1 RNA binding domain
					18	23	26.3844	23.0000	
FN1048	-0.186	3.744			3	3	3.4321	3.9048	AAL95244.1| Hypothetical membrane-spanning protein
FN1055	-0.712	11.624	6.778e-4	1.149e-3	37	59	42.3298	76.7954	AAL95251.1| Cysteine synthase
					31	67	45.4398	67.0000	
FN1057						17		22.1275	AAL95253.1| Diamine acetyltransferase
						5		5.0000	
FN1060	-0.653	10.198	8.286e-4	1.513e-3	26	33	29.7452	42.9533	AAL95256.1| hypothetical cytosolic protein
					17	43	24.9186	43.0000	
FN1062	1.814	12.371	3.726e-5	2.245e-5	113	22	129.2774	28.6356	AAL95258.1| Hydrolase
					98	49	143.6483	49.0000	
FN1063	-2.684	7.073			4	16	4.5762	20.8259	AAL95259.1| N-acyl-L-amino acid amidohydrolase
						38		38.0000	

Show detected proteins only
Show all proteins
Filter by category:
GO: amino acid transport

Proteins found: 1344

Enter (or paste) list of ORFs

Find ORFs

Test	Cutoff
q-Value	.005
p-Value	

	Signif	Direction	Applies To
red	yes	+	ratios, bars
yellow	no	n/a	bars
green	yes	−	ratios, bars
light red	yes	+	*p*- , *q*-Values
light green	yes	−	*p*- , *q*-Values

Dot Plots Dot Plots

Protein	*FnPgSg vs FnSg* Log$_2$ Ratio	Log$_2$ Sum	*q*-Value	*p*-Value	Raw *FnPgSg*	Raw *FnSg*	Normalized *FnPgSg*	Normalized *FnSg*	Description
**FN1066**	-0.884	7.393	2.037e-5	9.641e-6	9	14	10.2964	18.2226	AAL95262.1| Exodeoxyribonuclease VII large subunit
					6	17	8.7948	17.0000	
**FN1067**	-1.081	11.464	3.276e-4	4.341e-4	28	65	32.0333	84.6051	AAL95263.1| Tetratricopeptide repeat family protein
					28	70	41.0424	70.0000	
**FN1068**						16		20.8259	AAL95264.1| Smf protein
						13		13.0000	
**FN1069**	0.337	11.039	5.03e-2	2.244e-1	44	22	50.3381	28.6356	AAL95265.1| DNA topoisomerase I
					36	53	52.7687	53.0000	
**FN1070**	0.705	7.125	9.987e-3	3.497e-2	11	5	12.5845	6.5081	AAL95266.1| Glucose inhibited division protein A
					12	12	17.5896	12.0000	
**FN1071**	0.779	4.779			6		6.8643		AAL95267.1| Integrase/recombinase
						4		4.0000	
**FN1072**	0.276	9.483	8.59e-2	4.065e-1	22	12	25.1690	15.6194	AAL95268.1| GTP-binding protein
					23	33	33.7134	33.0000	
**FN1073**	-1.097	5.370				6		7.8097	AAL95269.1| Hypothetical protein
					3	11	4.3974	11.0000	
**FN1074**	-0.619	9.409	3.889e-3	1.073e-2	15	22	17.1607	28.6356	AAL95270.1| Signal recognition particle receptor FtsY
					17	36	24.9186	36.0000	
**FN1075**									AAL95271.1| Hypothetical protein
						7		7.0000	
**FN1077**	2.160	8.988	2.331e-4	2.717e-4	41	11	46.9059	14.3178	AAL95273.1| Hypothetical protein
					33	7	48.3714	7.0000	
**FN1078**	-0.071	15.034	1.212e-1	5.969e-1	133	144	152.1583	187.4328	AAL95274.1| Hypothetical exported 24-amino acid repeat protein
					140	188	205.2118	188.0000	
**FN1079**	-0.408	19.196	3.785e-3	1.035e-2	638	748	729.9022	973.6092	AAL95275.1| Neutrophil-activating protein A
					420	812	615.6354	812.0000	
**FN1080**	-0.934	6.274	5.287e-3	1.613e-2	6	11	6.8643	14.3178	AAL95276.1| Export ABC transporter
					4	10	5.8632	10.0000	
**FN1081**					18		20.5929		AAL95277.1| unknown
					15		21.9870		
**FN1082**					4		4.5762		AAL95278.1| unknown

Show detected proteins only
Show all proteins
Filter by category:
GO: amino acid transport

Proteins found: 1344

Enter (or paste) list of ORFs

Find ORFs

Test	Cutoff
q-Value	.005
p-Value	

	Signif	Direction	Applies To
red	yes	+	ratios, bars
yellow	no	n/a	bars
green	yes	–	ratios, bars
light red	yes	+	*p*- , *q*-Values
light green	yes	–	

Dot Plots Dot Plots

Protein	*FnPgSg vs FnSg* Log$_2$ Ratio	Log$_2$ Sum	*q*-Value	*p*-Value	Raw *FnPgSg*	Raw *FnSg*	Normalized *FnPgSg*	Normalized *FnSg*	Description
**FN1084**	2.604	10.889	2.093e-3	4.89e-3	75	11	85.8036	14.3178	AAL95280.1| unknown
					88	21	128.9903	21.0000	
**FN1085**	-0.840	12.514	7.008e-4	1.201e-3	41	85	46.9059	110.6374	AAL95281.1| 4-methyl-5(B-hydroxyethyl)-thiazole monophosphate biosynthesis enzyme
					46	94	67.4267	94.0000	
**FN1086**	-0.726	7.992	2.121e-2	8.449e-2	14	20	16.0167	26.0323	AAL95282.1| Transporter
					6	15	8.7948	15.0000	
**FN1088**	0.025	10.446	1.533e-1	7.834e-1	30	30	34.3214	39.0485	AAL95284.1| NADH oxidase
					28	35	41.0424	35.0000	
**FN1089**	-2.901	13.022	8.523e-4	1.576e-3	34	164	38.8976	213.4651	AAL95285.1| ATP-binding protein (contains P-loop)
					19	285	27.8502	285.0000	
**FN1091**	0.095	7.752	1.414e-1	7.125e-1	15	8	17.1607	10.4129	AAL95287.1| Sigma factor sigB regulation protein rsbU
					9	18	13.1922	18.0000	
**FN1092**	-1.405	11.288	3.51e-3	9.371e-3	14	62	16.0167	80.7002	AAL95288.1| Hypothetical protein
					31	82	45.4398	82.0000	
**FN1093**	1.332	10.706	8.764e-4	1.633e-3	57	15	65.2107	19.5242	AAL95289.1| Hypothetical protein
					44	32	64.4951	32.0000	
**FN1094**						13		16.9210	AAL95290.1| Dolichol-phosphate mannosyltransferase
						24		24.0000	
**FN1096**	0.666	10.166	3.181e-2	1.332e-1	49	16	56.0583	20.8259	AAL95292.1| Hypothetical protein
					20	33	29.3160	33.0000	
**FN1097**	1.098	7.098			12		13.7286		AAL95293.1| Hypothetical protein
					14	8	20.5212	8.0000	
**FN1101**	-1.221	4.779			3		3.4321		AAL95297.1| ATPase
						8		8.0000	
**FN1102**									AAL95298.1| tRNA 2'phosphotransferase
						10		10.0000	
**FN1103**	-1.340	10.659	5.227e-4	7.95e-4	16	43	18.3048	55.9695	AAL95299.1| Excinuclease ABC subunit A
					22	72	32.2476	72.0000	
**FN1104**					5		5.7202		AAL95300.1| Holliday junction DNA helicase ruvA
**FN1105**	0.907	11.931	7.415e-7	8.423e-8	74	34	84.6595	44.2550	AAL95301.1| Hypothetical protein
					59	47	86.4821	47.0000	

Log$_2$ Ratios: -6 -4 -2 0 2 4 6

Show detected proteins only
Show all proteins
Filter by category:
GO: amino acid transport

Proteins found: 1344

Enter (or paste) list of ORFs
Find ORFs

Test	Cutoff
q-Value	.005
p-Value	

	Signif	Direction	Applies To
red	yes	+	ratios, bars
yellow	no	n/a	bars
green	yes	−	ratios, bars
light red	yes	+	*p*- , *q*-Values
light green	yes	−	

Dot Plots Dot Plots

Protein	*FnPgSg vs FnSg* Log$_2$ Ratio	Log$_2$ Sum	*q*-Value	*p*-Value	Raw *FnPgSg*	Raw *FnSg*	Normalized *FnPgSg*	Normalized *FnSg*	Description
**FN1106**	-0.445	11.477	1.421e-2	5.298e-2	39	55	44.6178	71.5889	AAL95302.1| L-serine dehydratase
					32	53	46.9056	53.0000	
**FN1111**	-0.607	7.330	2.488e-3	6.108e-3	9	11	10.2964	14.3178	AAL95307.1| Dipeptide-binding protein
					7	17	10.2606	17.0000	
**FN1117**	2.544	10.529	1.757e-5	7.374e-6	79	16	90.3797	20.8259	AAL95313.1| LSU ribosomal protein L21P
					65	11	95.2769	11.0000	
**FN1119**	3.015	12.869	2.552e-5	1.297e-5	230	16	263.1309	20.8259	AAL95315.1| LSU ribosomal protein L27P
					156	40	228.6646	40.0000	
**FN1120**	-0.904	15.279	1.126e-5	3.526e-6	128	207	146.4381	269.4346	AAL95316.1| Phosphoenolpyruvate carboxykinase (ATP)
					99	276	145.1141	276.0000	
**FN1121**	0.318	12.651	5.013e-3	1.509e-2	72	52	82.3714	67.6841	AAL95317.1| hypothetical cytosolic protein
					66	76	96.7427	76.0000	
**FN1122**	1.142	13.439	4.401e-4	6.299e-4	143	66	163.5988	85.9067	AAL95318.1| Long-chain-fatty-acid--CoA ligase
					102	56	149.5115	56.0000	
**FN1123**	1.882	8.425	1.103e-2	3.946e-2	43	11	49.1940	14.3178	AAL95319.1| Thioredoxin-like protein
					15	5	21.9870	5.0000	
**FN1124**	0.016	17.157	1.577e-1	8.097e-1	312	286	356.9428	372.2623	AAL95320.1| Outer membrane porin F
					281	388	411.8894	388.0000	
**FN1125**	-0.730	12.400	5.505e-3	1.698e-2	46	84	52.6262	109.3358	AAL95321.1| LemA protein
					42	80	61.5635	80.0000	
**FN1127**	-0.605	11.533	1.751e-4	1.824e-4	40	54	45.7619	70.2873	AAL95323.1| Hypothetical membrane-spanning protein
					29	64	42.5082	64.0000	
**FN1128**	-0.018	15.108	1.414e-1	7.122e-1	156	140	178.4714	182.2263	AAL95324.1| Acylamino-acid-releasing enzyme
					133	196	194.9512	196.0000	
**FN1129**	0.212	6.249	1.142e-1	5.575e-1	10	4	11.4405	5.2065	AAL95325.1| Chromosome partition protein smc
					5	11	7.3290	11.0000	
**FN1130**	-0.265	6.654			8		9.1524		AAL95326.1| Tetraacyldisaccharide 4'-kinase
						11		11.0000	
**FN1131**	-1.028	4.586			3		3.4321		AAL95327.1| Hypothetical protein
						7		7.0000	
**FN1133**	0.070	9.985	1.316e-1	6.553e-1	25	27	28.6012	35.1436	AAL95329.1| N-acetylglucosamine-6-phosphate deacetylase
					25	27	36.6450	27.0000	

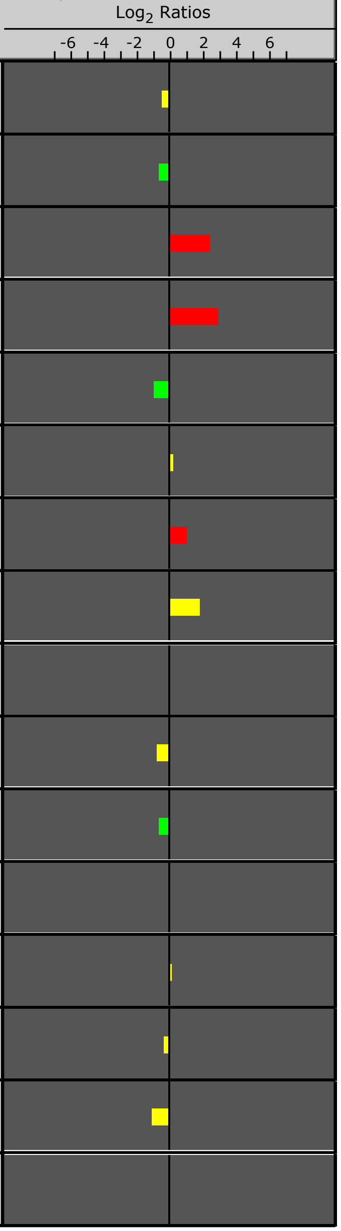

Show detected proteins only
Show all proteins
Filter by category:
GO: amino acid transport

Proteins found: 1344

Enter (or paste) list of ORFs

Find ORFs

Test	Cutoff
q-Value	.005
p-Value	

	Signif	Direction	Applies To
red	yes	+	ratios, bars
yellow	no	n/a	bars
green	yes	−	ratios, bars
pink	yes	+	*p*- , *q*-Values
light green	yes	−	*p*- , *q*-Values

Dot Plots Dot Plots

Protein	FnPgSg vs FnSg Log$_2$ Ratio	Log$_2$ Sum	*q*-Value	*p*-Value	Raw *FnPgSg*	Raw *FnSg*	Normalized *FnPgSg*	Normalized *FnSg*	Description
**FN1134**	1.652	7.652			26		29.7452		AAL95330.1| Hypothetical cytosolic protein
					14	8	20.5212	8.0000	
**FN1135**	0.184	14.164	2.162e-2	8.642e-2	123	107	140.7178	139.2730	AAL95331.1| Phosphonates-binding protein
					101	115	148.0457	115.0000	
**FN1136**	-1.052	8.428	2.163e-3	5.096e-3	11	18	12.5845	23.4291	AAL95332.1| Phosphonates transport ATP-binding protein phnC
					9	30	13.1922	30.0000	
**FN1137**									AAL95333.1| Phosphonates transport system permease protein phnE
					3		4.3974		
**FN1138**	1.098	19.059	4.851e-3	1.445e-2	1147	491	1312.2223	639.0937	AAL95334.1| Hypothetical cytosolic protein
					580	371	850.1632	371.0000	
**FN1139**	-3.989	10.621	4.891e-4	7.255e-4	11	107	12.5845	139.2730	AAL95335.1| Activator of (R)-2-hydroxyglutaryl-CoA dehydratase
					5	177	7.3290	177.0000	
**FN1140**						10		13.0162	AAL95336.1| hypothetical protein
						31		31.0000	
**FN1142**	-0.944	4.502			3	4	3.4321	5.2065	AAL95338.1| Oxygen-independent coproporphyrinogen III oxidase
						8		8.0000	
**FN1143**	-1.354	14.678	4.952e-3	1.485e-2	104	244	118.9809	317.5944	AAL95339.1| Glucosamine-6-phosphate isomerase
					57	200	83.5505	200.0000	
**FN1144**	0.771	14.774	6.158e-5	4.586e-5	199	107	227.6654	139.2730	AAL95340.1| Hypothetical Exported Protein
					143	117	209.6092	117.0000	
**FN1145**	0.231	8.926	1.092e-1	5.303e-1	20	9	22.8809	11.7145	AAL95341.1| Oligoendopeptidase F
					17	29	24.9186	29.0000	
**FN1146**					17		19.4488		AAL95342.1| Hypothetical exported 24-amino acid repeat protein
					19		27.8502		
**FN1147**					33		37.7536		AAL95343.1| Hypothetical protein
					43		63.0293		
**FN1148**	-0.319	11.331	2.232e-3	5.304e-3	41	41	46.9059	53.3663	AAL95344.1| Serine/threonine sodium symporter
					30	60	43.9740	60.0000	
**FN1149**	-0.895	6.949	1.095e-2	3.909e-2	4	11	4.5762	14.3178	AAL95345.1| ATP-dependent nuclease subunit A
					8	16	11.7264	16.0000	
**FN1150**	-0.256	5.359							AAL95346.1| unknown
					4	7	5.8632	7.0000	

Log$_2$ Ratios: -6 -4 -2 0 2 4 6

Show detected proteins only
Show all proteins
Filter by category:
GO: amino acid transport

Proteins found: 1344

Enter (or paste) list of ORFs

Find ORFs

Test	Cutoff
q-Value	.005
p-Value	

	Signif	Direction	Applies To
red	yes	+	ratios, bars
yellow	no	n/a	bars
green	yes	−	ratios, bars
pink	yes	+	*p*- , *q*-Values
light green	yes	−	

Dot Plots Dot Plots

Protein	*FnPgSg vs FnSg* Log$_2$ Ratio	Log$_2$ Sum	*q*-Value	*p*-Value	Raw *FnPgSg*	Raw *FnSg*	Normalized *FnPgSg*	Normalized *FnSg*	Description
FN1152	0.622	13.950	1.393e-3	2.945e-3	146	79	167.0309	102.8277	AAL95348.1| Aspartate aminotransferase
					99	100	145.1141	100.0000	
FN1153					37		42.3298		AAL95349.1| Hypothetical protein
					29		42.5082		
FN1154						8		10.4129	AAL95350.1| Ribonuclease BN
						10		10.0000	
FN1155						3		3.9048	AAL95351.1| Cell division protein ftsI
						5		5.0000	
FN1156						10		13.0162	AAL95352.1| Primosomal protein N'
						5		5.0000	
FN1157					12		13.7286		AAL95353.1| Polypeptide deformylase
					16		23.4528		
FN1159	-0.836	14.052	1.714e-4	1.773e-4	77	137	88.0916	178.3215	AAL95355.1| Fructose-1,6-bisphosphatase
					73	170	107.0033	170.0000	
FN1160	0.352	6.372	1.012e-2	3.554e-2	9	7	10.2964	9.1113	AAL95356.1| SWF/SNF family helicase
					7	7	10.2606	7.0000	
FN1161	-0.403	5.507				5		6.5081	AAL95357.1| Glutamate racemase
					4	9	5.8632	9.0000	
FN1162						4		5.2065	AAL95358.1| Hydroxyacylglutathione hydrolase
						10		10.0000	
FN1163	0.927	12.063	3.219e-3	8.388e-3	77	46	88.0916	59.8744	AAL95359.1| Thioredoxin reductase
					63	35	92.3453	35.0000	
FN1164	-0.788	6.536				11		14.3178	AAL95360.1| Glucokinase
					5	11	7.3290	11.0000	
FN1165	0.568	24.997	2.725e-3	6.819e-3	6372	4182	7289.8698	5443.3605	AAL95361.1| D-galactose-binding protein
					4643	4062	6805.7029	4062.0000	
FN1166	-0.805	14.946	1.643e-5	6.592e-6	112	178	128.1333	231.6877	AAL95362.1| Galactoside transport ATP-binding protein mglA
					96	238	140.7167	238.0000	
FN1167	1.102	8.201	4.179e-3	1.182e-2	26	8	29.7452	10.4129	AAL95363.1| Galactoside transport system permease protein mglC
					14	13	20.5212	13.0000	
FN1169	-0.563	16.637	1.318e-2	4.862e-2	217	245	248.2583	318.8961	AAL95365.1| L-lactate dehydrogenase
					189	457	277.0359	457.0000	

Log$_2$ Ratios: -6 -4 -2 0 2 4 6

Show detected proteins only
Show all proteins
Filter by category:
GO: amino acid transport

Proteins found: 1344

Enter (or paste) list of ORFs

Find ORFs

Test	Cutoff
q-Value	.005
p-Value	

Signif	Direction	Applies To
yes	+	ratios, bars
no	n/a	bars
yes	–	ratios, bars
yes	+	*p*- , *q*-Values
yes	–	

Dot Plots Dot Plots

Protein	*FnPgSg vs FnSg* Log$_2$ Ratio	Log$_2$ Sum	*q*-Value	*p*-Value	Raw *FnPgSg*	Raw *FnSg*	Normalized *FnPgSg*	Normalized *FnSg*	Description
FN1170	-0.665	24.970	2.019e-5	9.477e-6	3813	5624	4362.2526	7320.2915	AAL95366.1| Pyruvate-flavodoxin oxidoreductase
					3237	7117	4744.7901	7117.0000	
FN1171	-0.167	19.664	3.173e-2	1.328e-1	767	673	877.4843	875.9879	AAL95367.1| Acetate kinase
					575	1055	842.8342	1055.0000	
FN1172	0.469	18.661	7.401e-4	1.293e-3	704	397	805.4093	516.7418	AAL95368.1| Phosphate acetyltransferase
					484	578	709.4465	578.0000	
FN1179									AAL95375.1| ATP-dependent RNA helicase
						6		6.0000	
FN1180									AAL95376.1| Hypothetical protein
						7		7.0000	
FN1181	0.388	12.335	1.265e-2	4.632e-2	81	42	92.6678	54.6679	AAL95377.1| unknown
					49	71	71.8241	71.0000	
FN1182									AAL95378.1| Hypothetical protein
						6		6.0000	
FN1183	-1.245	6.511	9.701e-3	3.377e-2	7	8	8.0083	10.4129	AAL95379.1| Hypothetical cytosolic protein
					3	19	4.3974	19.0000	
FN1185	0.090	10.073	7.936e-2	3.729e-1	31	22	35.4655	28.6356	AAL95381.1| SIR2 family protein
					22	35	32.2476	35.0000	
FN1186	-3.890	13.521	1.311e-5	4.501e-6	30	308	34.3214	400.8979	AAL95382.1| Amidohydrolase
					15	434	21.9870	434.0000	
FN1187	-1.126	11.721	1.251e-2	4.572e-2	38	85	43.4738	110.6374	AAL95383.1| Amino acid-binding protein
					24	61	35.1792	61.0000	
FN1188	-1.000	12.418	2.38e-4	2.799e-4	53	87	60.6345	113.2406	AAL95384.1| Hypothetical protein
					30	96	43.9740	96.0000	
FN1189	-0.244	11.706	6.726e-2	3.104e-1	48	59	54.9143	76.7954	AAL95385.1| Hypothetical protein
					35	49	51.3030	49.0000	
FN1190	-0.874	13.976	1.307e-5	4.48e-6	87	135	99.5321	175.7182	AAL95386.1| Probable cadmium-transporting ATPase
					60	168	87.9479	168.0000	
FN1191	-2.540	11.001	3.237e-3	8.446e-3	20	104	22.8809	135.3681	AAL95387.1| unknown
					10	83	14.6580	83.0000	
FN1192	0.022	18.859	1.68e-1	8.733e-1	642	451	734.4784	587.0291	AAL95388.1| unknown
					447	782	655.2120	782.0000	

Log$_2$ Ratios: -6 -4 -2 0 2 4 6

Show detected proteins only
Show all proteins
Filter by category:
GO: amino acid transport

Proteins found: 1344

Enter (or paste) list of ORFs

Find ORFs

Test	Cutoff
q-Value	.005
p-Value	

	Signif	Direction	Applies To
red	yes	+	ratios, bars
yellow	no	n/a	bars
green	yes	−	ratios, bars
pink	yes	+	*p*- , *q*-Values
light green	yes	−	*p*- , *q*-Values

Dot Plots Dot Plots

Protein	*FnPgSg vs FnSg* Log$_2$ Ratio	Log$_2$ Sum	*q*-Value	*p*-Value	Raw *FnPgSg*	Raw *FnSg*	Normalized *FnPgSg*	Normalized *FnSg*	Description
FN1198	-1.312	10.670	5.405e-5	3.859e-5	23	47	26.3131	61.1760	AAL95394.1| Transporter
					17	66	24.9186	66.0000	
FN1200	-4.391	11.975	5.214e-4	7.921e-4	5	253	5.7202	329.3090	AAL95396.1| Hypothetical protein
					15	252	21.9870	252.0000	
FN1201									AAL95397.1| unknown
						7		7.0000	
FN1202	-0.662	8.674	3.534e-2	1.502e-1	14	26	16.0167	33.8420	AAL95398.1| NH(3)-dependent NAD(+) synthetase
					11	17	16.1238	17.0000	
FN1203						10		13.0162	AAL95399.1| GTP-binding protein
						8		8.0000	
FN1204						10		13.0162	AAL95400.1| Methyltransferase
						18		18.0000	
FN1205	0.378	11.953	5.801e-2	2.633e-1	55	58	62.9226	75.4938	AAL95401.1| Protease
					55	35	80.6189	35.0000	
FN1208	-1.571	6.604			5		5.7202		AAL95404.1| 1-deoxyxylulose-5-phosphate synthase
						17		17.0000	
FN1209	1.393	10.365	1.062e-2	3.767e-2	67	16	76.6512	20.8259	AAL95405.1| Hypothetical RNA binding protein
					28	24	41.0424	24.0000	
FN1210	-0.214	10.820	1.025e-1	4.939e-1	28	45	32.0333	58.5727	AAL95406.1| Metal dependent hydrolase
					32	33	46.9056	33.0000	
FN1211	0.274	7.827	1.073e-1	5.2e-1	20	8	22.8809	10.4129	AAL95407.1| Cell division protein ftsI
					7	17	10.2606	17.0000	
FN1213	-0.364	11.542	5.795e-3	1.813e-2	47	46	53.7702	59.8744	AAL95409.1| Hypothetical protein
					29	64	42.5082	64.0000	
FN1214					6		6.8643		AAL95410.1| Fe-S oxidoreductase
					5		7.3290		
FN1216	-1.162	7.006	8.666e-3	2.956e-2	3	13	3.4321	16.9210	AAL95412.1| RRF2 family protein
					8	17	11.7264	17.0000	
FN1217	-2.186	6.863	7.813e-4	1.393e-3	5	20	5.7202	26.0323	AAL95413.1| Holliday junction DNA helicase ruvB
					3	20	4.3974	20.0000	
FN1218						13		16.9210	AAL95414.1| unknown
						19		19.0000	

Log$_2$ Ratios: -6 -4 -2 0 2 4 6

Show detected proteins only
Show all proteins
Filter by category:
GO: amino acid transport

Proteins found: 1344

Enter (or paste) list of ORFs

Find ORFs

Test	Cutoff
q-Value	.005
p-Value	

	Signif	Direction	Applies To
red	yes	+	ratios, bars
yellow	no	n/a	bars
green	yes	−	ratios, bars
pink	yes	+	*p*-, *q*-Values
light green	yes	−	

Dot Plots Dot Plots

Protein	FnPgSg vs FnSg Log2 Ratio	Log2 Sum	q-Value	p-Value	Raw FnPgSg	Raw FnSg	Normalized FnPgSg	Normalized FnSg	Description
FN1219	0.347	8.285	5.689e-2	2.576e-1	22	11	25.1690	14.3178	AAL95415.1| Hypothetical protein
					10	17	14.6580	17.0000	
FN1220	-0.116	15.794	1.186e-1	5.823e-1	212	150	242.5380	195.2425	AAL95416.1| Cysteine synthase
					147	301	215.4724	301.0000	
FN1221	-0.537	9.201	2.795e-2	1.15e-1	16	28	18.3048	36.4453	AAL95417.1| Hypothetical protein
					15	22	21.9870	22.0000	
FN1222	1.553	6.197			9		10.2964		AAL95418.1| Hypothetical protein
					13	5	19.0554	5.0000	
FN1223	1.749	11.492	7.912e-6	1.933e-6	90	25	102.9643	32.5404	AAL95419.1| Oxygen-insensitive NAD(P)H nitroreductase
					64	26	93.8111	26.0000	
FN1224	0.376	12.987	2.757e-2	1.133e-1	100	47	114.4047	61.1760	AAL95420.1| 2-dehydro-3-deoxyphosphooctonate aldolase
					62	97	90.8795	97.0000	
FN1225	0.773	13.837	6.241e-4	1.028e-3	147	60	168.1750	78.0970	AAL95421.1| UDP-N-acetylmuramoyl-L-alanyl-D-glutamate--meso-lanthionine ligase
					101	107	148.0457	107.0000	
FN1226	-1.139	14.343	4.941e-3	1.48e-2	84	195	96.1000	253.8152	AAL95422.1| Uracil-DNA glycosylase
					67	174	98.2085	174.0000	
FN1230					62		70.9309		AAL95426.1| Hypothetical cytosolic protein
					37		54.2345		
FN1231	-0.584	19.529	3.977e-5	2.495e-5	592	788	677.2760	1025.6739	AAL95427.1| Inosine-5'-monophosphate dehydrogenase
					507	1104	743.1599	1104.0000	
FN1233									AAL95429.1| Putative NAD(P)H oxidoreductase
						6		6.0000	
FN1234	-1.350	7.435			8		9.1524		AAL95430.1| Hypothetical protein
					5	21	7.3290	21.0000	
FN1235	-1.215	8.682	5.925e-3	1.866e-2	13	29	14.8726	37.7469	AAL95431.1| Ankyrin repeat proteins
					8	24	11.7264	24.0000	
FN1237	-2.143	11.997	3.259e-4	4.31e-4	25	112	28.6012	145.7811	AAL95433.1| Choline kinase
					22	123	32.2476	123.0000	
FN1238									AAL95434.1| Hypothetical protein
						4		4.0000	
FN1240	0.515	6.090	1.651e-2	6.312e-2	7	6	8.0083	7.8097	AAL95436.1| Lipopolysaccharide core biosynthesis protein rfaY
					8	6	11.7264	6.0000	

Log2 Ratios: -6 -4 -2 0 2 4 6

Show detected proteins only
Show all proteins
Filter by category:
GO: amino acid transport

Proteins found: 1344

Enter (or paste) list of ORFs

Find ORFs

Test	Cutoff
q-Value	.005
p-Value	

	Signif	Direction	Applies To
red	yes	+	ratios, bars
yellow	no	n/a	bars
green	yes	−	ratios, bars
pink	yes	+	p-, q-Values
light green	yes	−	p-, q-Values

Dot Plots Dot Plots

Protein	FnPgSg vs FnSg Log$_2$ Ratio	Log$_2$ Sum	*q*-Value	*p*-Value	Raw *FnPgSg*	Raw *FnSg*	Normalized *FnPgSg*	Normalized *FnSg*	Description
FN1241						8		10.4129	AAL95437.1| polysaccharide biosynthesis protein
						12		12.0000	
FN1242	-2.040	7.380	2.034e-5	9.613e-6	6	21	6.8643	27.3339	AAL95438.1| Polysaccharide deacetylase
					4	25	5.8632	25.0000	
FN1243	-0.706	6.584	4.124e-5	2.651e-5	7	10	8.0083	13.0162	AAL95439.1| Glycosyl transferase
					5	12	7.3290	12.0000	
FN1244	-2.025	7.437	1.644e-3	3.627e-3	5	17	5.7202	22.1275	AAL95440.1| Polysaccharide deacetylase
					5	31	7.3290	31.0000	
FN1245						6		7.8097	AAL95441.1| Glycosyl transferase
						7		7.0000	
FN1246	-2.093	6.366				15		19.5242	AAL95442.1| Lipooligosaccharide cholinephosphotransferase
					3	18	4.3974	18.0000	
FN1247	-2.017	8.189	4.351e-3	1.25e-2	11	19	12.5845	24.7307	AAL95443.1| LOS biosynthesis enzyme LBGB
					3	44	4.3974	44.0000	
FN1250	0.308	9.122	3.567e-2	1.518e-1	19	18	21.7369	23.4291	AAL95446.1| Guanine-hypoxanthine permease
					21	19	30.7818	19.0000	
FN1251	-0.152	6.757	1.525e-1	7.785e-1	7	13	8.0083	16.9210	AAL95447.1| High-affinity iron permease
					8	5	11.7264	5.0000	
FN1252	0.785	18.058	1.531e-3	3.314e-3	667	361	763.0796	469.8836	AAL95448.1| 34 kDa membrane antigen precursor
					415	326	608.3064	326.0000	
FN1253	0.452	16.068	4.431e-3	1.282e-2	240	186	274.5714	242.1007	AAL95449.1| unknown
					231	206	338.5995	206.0000	
FN1254	1.056	12.894	2.61e-4	3.21e-4	120	53	137.2857	68.9857	AAL95450.1| Oxygen-insensitive NAD(P)H nitroreductase
					78	52	114.3323	52.0000	
FN1256									AAL95452.1| C4-dicarboxylate transporter large subunit
					3		4.3974		
FN1258	0.294	19.935	2.606e-3	6.457e-3	987	741	1129.1747	964.4979	AAL95454.1| C4-dicarboxylate-binding protein
					742	844	1087.6226	844.0000	
FN1259									AAL95455.1| hypothetical protein
						9		9.0000	
FN1260					3		3.4321		AAL95456.1| Sensory Transduction Protein Kinase

Log$_2$ Ratios: -6 -4 -2 0 2 4 6

Show detected proteins only
Show all proteins
Filter by category:
GO: amino acid transport

Proteins found: 1344

Enter (or paste) list of ORFs

Find ORFs

Test	Cutoff
q-Value	.005
p-Value	

	Signif	Direction	Applies To
red	yes	+	ratios, bars
yellow	no	n/a	bars
green	yes	−	ratios, bars
pink	yes	+	*p*- , *q*-Values
light green	yes	−	*p*- , *q*-Values

Dot Plots Dot Plots

Protein	FnPgSg vs FnSg Log$_2$ Ratio	Log$_2$ Sum	*q*-Value	*p*-Value	Raw *FnPgSg*	Raw *FnSg*	Normalized *FnPgSg*	Normalized *FnSg*	Description
FN1262						21		27.3339	AAL95458.1| Integral membrane protein
						7		7.0000	
FN1263	-0.396	8.634	4.722e-3	1.395e-2	15	19	17.1607	24.7307	AAL95459.1| Cobalt chelatase
					12	21	17.5896	21.0000	
FN1264	-0.424	7.631	2.497e-4	3.004e-4	11	12	12.5845	15.6194	AAL95460.1| Hypothetical protein
					8	17	11.7264	17.0000	
FN1265	-0.294	12.574	4.543e-2	2e-1	49	76	56.0583	98.9229	AAL95461.1| Outer membrane protein
					58	74	85.0163	74.0000	
FN1266	0.165	13.765	6.992e-2	3.243e-1	98	99	112.1166	128.8600	AAL95462.1| UTP--glucose-1-phosphate uridylyltransferase
					94	94	137.7851	94.0000	
FN1267	0.005	10.122	1.85e-1	9.817e-1	29	32	33.1774	41.6517	AAL95463.1| Hypothetical protein
					23	25	33.7134	25.0000	
FN1268	0.461	14.901	1.65e-4	1.688e-4	187	120	213.9369	156.1940	AAL95464.1| Methionyl-tRNA synthetase
					134	142	196.4170	142.0000	
FN1269	-1.051	5.324				7		9.1113	AAL95465.1| Hypothetical lipoprotein
					3		4.3974		
FN1270	4.116	11.216			177	9	202.4964	11.7145	AAL95466.1| Hypothetical cytosolic protein
					139		203.7460		
FN1271	-0.039	11.411	1.637e-1	8.465e-1	49	49	56.0583	63.7792	AAL95467.1| Protease IV
					32	42	46.9056	42.0000	
FN1273	-1.894	10.159	3.127e-7	2.423e-8	14	51	16.0167	66.3824	AAL95469.1| Outer membrane protein tolC
					13	64	19.0554	64.0000	
FN1274	-0.494	8.478	8.989e-4	1.686e-3	15	16	17.1607	20.8259	AAL95470.1| Acriflavin resistance protein E
					10	24	14.6580	24.0000	
FN1275	0.273	9.113	3.324e-2	1.4e-1	26	16	29.7452	20.8259	AAL95471.1| Acriflavin resistance protein B
					15	22	21.9870	22.0000	
FN1276	-1.184	7.380	7.721e-4	1.37e-3	6	13	6.8643	16.9210	AAL95472.1| Hypothetical protein
					7	22	10.2606	22.0000	
FN1277	-0.195	15.440	1.632e-2	6.224e-2	169	160	193.3440	208.2587	AAL95473.1| Aminoacyl-histidine dipeptidase
					137	243	200.8144	243.0000	
FN1278	0.262	7.181							AAL95474.1| Acetyltransferase
					9	11	13.1922	11.0000	

Log$_2$ Ratios: -6 -4 -2 0 2 4 6

Show detected proteins only
Show all proteins
Filter by category:
GO: amino acid transport

Proteins found: 1344

Enter (or paste) list of ORFs

Find ORFs

Test	Cutoff
q-Value	.005
p-Value	

	Signif	Direction	Applies To
red	yes	+	ratios, bars
yellow	no	n/a	bars
green	yes	−	ratios, bars
light red	yes	+	*p*- , *q*-Values
light green	yes	−	*p*- , *q*-Values

Dot Plots Dot Plots

Protein	*FnPgSg vs FnSg* Log$_2$ Ratio	Log$_2$ Sum	*q*-Value	*p*-Value	Raw *FnPgSg*	Raw *FnSg*	Normalized *FnPgSg*	Normalized *FnSg*	Description
FN1279	0.154	11.326	2.489e-2	1.01e-1	46	40	52.6262	52.0647	AAL95475.1| Zinc metallohydrolase, glyoxalase II family
					37	44	54.2345	44.0000	
FN1280	-2.752	10.660	5.523e-4	8.591e-4	13	89	14.8726	115.8439	AAL95476.1| Serine protease, V8 family
					11	93	16.1238	93.0000	
FN1281						27		35.1436	AAL95477.1| Cysteine protease
						35		35.0000	
FN1282	0.455	13.042	1.408e-3	2.984e-3	97	66	110.9726	85.9067	AAL95478.1| LSU ribosomal protein L17P
					71	71	104.0717	71.0000	
FN1283	0.759	19.378	2.37e-3	5.732e-3	947	404	1083.4129	525.8531	AAL95479.1| DNA-directed RNA polymerase alpha chain
					726	743	1064.1698	743.0000	
FN1284	0.852	18.160	1.686e-5	6.882e-6	628	324	718.4617	421.7238	AAL95480.1| SSU ribosomal protein S4P
					502	384	735.8309	384.0000	
FN1285	2.590	11.582	1.366e-4	1.301e-4	112	17	128.1333	22.1275	AAL95481.1| SSU ribosomal protein S11P
					98	23	143.6483	23.0000	
FN1286	0.259	13.472	4.274e-2	1.867e-1	118	66	134.9976	85.9067	AAL95482.1| SSU ribosomal protein S13P
					67	109	98.2085	109.0000	
FN1287	-0.169	6.448	1.021e-1	4.92e-1	9	6	10.2964	7.8097	AAL95483.1| Bacterial Protein Translation Initiation Factor 1 (IF-1)
					5	12	7.3290	12.0000	
FN1290	-0.547	7.552	4.987e-2	2.222e-1	7	17	8.0083	22.1275	AAL95486.1| Hypothetical protein
					10	11	14.6580	11.0000	
FN1293	0.171	4.932				4		5.2065	AAL95489.1| Hypothetical protein
					4		5.8632		
FN1296					10		11.4405		AAL95492.1| unknown
					6		8.7948		
FN1297	0.712	10.659	2.065e-3	4.807e-3	40	26	45.7619	33.8420	AAL95493.1| Methionine aminopeptidase
					39	29	57.1661	29.0000	
FN1298	0.982	12.121	1.02e-2	3.586e-2	64	53	73.2190	68.9857	AAL95494.1| Adenylate kinase
					78	26	114.3323	26.0000	
FN1299						4		5.2065	AAL95495.1| dTDP-glucose 4,6-dehydratase
						7		7.0000	
FN1301	0.846	12.124	3.104e-4	4.039e-4	72	42	82.3714	54.6679	AAL95497.1| ABC transporter ATP-binding protein
					66	45	96.7427	45.0000	

Log$_2$ Ratios: -6 -4 -2 0 2 4 6

Show detected proteins only
Show all proteins
Filter by category:
GO: amino acid transport

Proteins found: 1344

Enter (or paste) list of ORFs

Find ORFs

Test	Cutoff
q-Value	.005
p-Value	

	Signif	Direction	Applies To
red	yes	+	ratios, bars
yellow	no	n/a	bars
green	yes	−	ratios, bars
pink	yes	+	*p*- , *q*-Values
light green	yes	−	

Dot Plots Dot Plots

Protein	*FnPgSg vs FnSg* Log$_2$ Ratio	Log$_2$ Sum	*q*-Value	*p*-Value	Raw *FnPgSg*	Raw *FnSg*	Normalized *FnPgSg*	Normalized *FnSg*	Description
FN1302	2.453	15.969	4.184e-4	5.898e-4	566	81	647.5308	105.4309	AAL95498.1| Hypothetical protein
					367	111	537.9481	111.0000	
FN1303	0.035	12.114	1.474e-1	7.479e-1	64	55	73.2190	71.5889	AAL95499.1| hypothetical cytosolic protein
					42	60	61.5635	60.0000	
FN1304	0.391	14.171	1.726e-3	3.858e-3	145	87	165.8869	113.2406	AAL95500.1| Single-strand DNA binding protein
					99	124	145.1141	124.0000	
FN1305	-0.427	10.319	8.954e-2	4.254e-1	18	46	20.5929	59.8744	AAL95501.1| Hypothetical cytosolic protein
					28	23	41.0424	23.0000	
FN1306	-2.726	12.610	7.839e-4	1.4e-3	23	135	26.3131	175.7182	AAL95502.1| Methyltransferase
					24	231	35.1792	231.0000	
FN1309	1.479	15.699	4.567e-5	3.076e-5	312	87	356.9428	113.2406	AAL95505.1| Hypothetical protein
					282	163	413.3552	163.0000	
FN1313	0.157	12.363	1.121e-1	5.463e-1	84	58	96.1000	75.4938	AAL95509.1| Oligopeptide-binding protein oppA
					39	62	57.1661	62.0000	
FN1315						6		7.8097	AAL95511.1| Hypothetical protein
FN1317						8		10.4129	AAL95513.1| RNA polymerase sigma factor
						11		11.0000	
FN1318	0.683	9.707	7.783e-3	2.606e-2	32	22	36.6095	28.6356	AAL95514.1| RNA polymerase sigma factor rpoD
					25	17	36.6450	17.0000	
FN1319	-1.487	7.683	2.276e-2	9.15e-2	6	10	6.8643	13.0162	AAL95515.1| DNA primase
					7	35	10.2606	35.0000	
FN1320	0.526	15.632	7.282e-3	2.398e-2	205	168	234.5297	218.6716	AAL95516.1| Peptidyl-prolyl cis-trans isomerase
					209	157	306.3519	157.0000	
FN1321	-0.279	20.089	2.344e-2	9.448e-2	969	849	1108.5819	1105.0725	AAL95517.1| Acetoacetate metabolism regulatory protein atoC
					552	1221	809.1208	1221.0000	
FN1322	-1.832	6.864			5	19	5.7202	24.7307	AAL95518.1| Membrane metalloprotease
						16		16.0000	
FN1323	0.328	6.105	5.967e-2	2.716e-1	6	6	6.8643	7.8097	AAL95519.1| Thymidylate kinase
					8	7	11.7264	7.0000	
FN1324	-1.111	10.021	8.493e-4	1.568e-3	14	39	16.0167	50.7630	AAL95520.1| 1-deoxy-D-xylulose 5-phosphate reductoisomerase
					19	44	27.8502	44.0000	

Log$_2$ Ratios

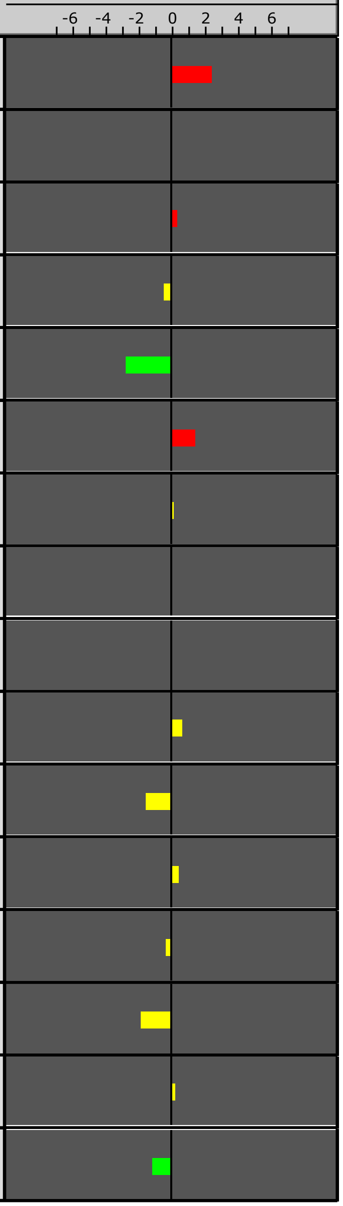

Show detected proteins only
Show all proteins
Filter by category:
GO: amino acid transport

Proteins found: 1344

Enter (or paste) list of ORFs

Find ORFs

Test	Cutoff
q-Value	.005
p-Value	

	Signif	Direction	Applies To
red	yes	+	ratios, bars
yellow	no	n/a	bars
green	yes	−	ratios, bars
light red	yes	+	*p*- , *q*-Values
light green	yes	−	

Dot Plots Dot Plots

Protein	FnPgSg vs FnSg Log$_2$ Ratio	Log$_2$ Sum	*q*-Value	*p*-Value	Raw *FnPgSg*	Raw *FnSg*	Normalized *FnPgSg*	Normalized *FnSg*	Description
FN1326	0.552	6.166							AAL95522.1| Undecaprenyl pyrophosphate synthetase
					7	7	10.2606	7.0000	
FN1327	-0.820	7.208			8	11	9.1524	14.3178	AAL95523.1| Dimethylallyltransferase
						18		18.0000	
FN1328					4		4.5762		AAL95524.1| Exodeoxyribonuclease VII small subunit
					3		4.3974		
FN1330									AAL95526.1| S-adenosylmethionine:tRNA ribosyltransferase-isomerase
						4		4.0000	
FN1331					14		16.0167		AAL95527.1| Methyltransferase
					4		5.8632		
FN1332	0.896	10.911	6.166e-4	1.009e-3	47	21	53.7702	27.3339	AAL95528.1| Bacterial Peptide Chain Release Factor 1 (RF-1)
					45	37	65.9609	37.0000	
FN1333						7		9.1113	AAL95529.1| Hypothetical protein
						18		18.0000	
FN1334	1.170	6.389			12	4	13.7286	5.2065	AAL95530.1| N-acetylmuramoyl-L-alanine amidase
						7		7.0000	
FN1335	2.490	13.519	1.696e-3	3.778e-3	176	18	201.3523	23.4291	AAL95531.1| Protein translocase subunit YajC
					213	68	312.2151	68.0000	
FN1336									AAL95532.1| Hypothetical protein
						14		14.0000	
FN1337	-0.028	5.586			6		6.8643		AAL95533.1| unknown
						7		7.0000	
FN1340	0.751	16.700	6.195e-4	1.016e-3	403	173	461.0511	225.1797	AAL95536.1| Glutamyl-tRNA synthetase
					263	278	385.5050	278.0000	
FN1341	0.869	7.842	9.887e-3	3.455e-2	14	8	16.0167	10.4129	AAL95537.1| Bacterial Peptide Chain Release Factor 2 (RF-2)
					17	12	24.9186	12.0000	
FN1343						20		26.0323	AAL95539.1| seC-independent protein TATD
						30		30.0000	
FN1345									AAL95541.1| 2-hydroxy-6-oxo-6-phenylhexa-2,4-dienoate hydrolase
						4		4.0000	
FN1346						15		19.5242	AAL95542.1| Hypothetical cytosolic protein
						19		19.0000	

Log$_2$ Ratios: -6 -4 -2 0 2 4 6

Show detected proteins only
Show all proteins
Filter by category:
GO: amino acid transport

Proteins found: 1344

Enter (or paste) list of ORFs

Find ORFs

Test	Cutoff
q-Value	.005
p-Value	

	Signif	Direction	Applies To
red	yes	+	ratios, bars
yellow	no	n/a	bars
green	yes	–	ratios, bars
pink	yes	+	*p*- , *q*-Values
light green	yes	–	

Dot Plots Dot Plots

Protein	FnPgSg vs FnSg Log_2 Ratio	Log_2 Sum	*q*-Value	*p*-Value	Raw *FnPgSg*	Raw *FnSg*	Normalized *FnPgSg*	Normalized *FnSg*	Description
FN1347	0.867	6.378	5.785e-4	9.184e-4	10	5	11.4405	6.5081	AAL95543.1| Hypothetical cytosolic protein
					9	7	13.1922	7.0000	
FN1348	-0.574	9.468	7.142e-2	3.322e-1	33	23	37.7536	29.9372	AAL95544.1| ABC transporter ATP-binding protein
					4	35	5.8632	35.0000	
FN1349	-0.062	6.451			8	7	9.1524	9.1113	AAL95545.1| ABC transporter permease protein
						10		10.0000	
FN1351	-0.670	10.134	7.207e-3	2.367e-2	17	35	19.4488	45.5566	AAL95547.1| 15 kDa lipoprotein precursor
					23	39	33.7134	39.0000	
FN1352	0.283	11.727	1.938e-2	7.597e-2	61	35	69.7869	45.5566	AAL95548.1| ABC transporter ATP-binding protein
					40	60	58.6319	60.0000	
FN1353	-0.371	7.316	5.968e-2	2.716e-1	13	9	14.8726	11.7145	AAL95549.1| ABC transporter permease protein
					5	17	7.3290	17.0000	
FN1354	0.188	9.115	7.149e-2	3.325e-1	26	17	29.7452	22.1275	AAL95550.1| ABC transporter permease protein
					14	22	20.5212	22.0000	
FN1355					3		3.4321		AAL95551.1| Integral membrane protein
FN1358	0.128	10.572	1.125e-1	5.481e-1	38	22	43.4738	28.6356	AAL95554.1| Hypothetical protein
					26	46	38.1108	46.0000	
FN1359	-3.061	16.354	1.608e-3	3.526e-3	97	760	110.9726	989.2286	AAL95555.1| Dipeptide-binding protein
					61	683	89.4137	683.0000	
FN1362						76		98.9229	AAL95558.1| Dipeptide transport ATP-binding protein dppD
						82		82.0000	
FN1363	-3.984	8.662	7.381e-4	1.288e-3	5	70	5.7202	91.1132	AAL95559.1| Dipeptide transport ATP-binding protein dppF
					3	69	4.3974	69.0000	
FN1364					54		61.7786		AAL95560.1| LSU ribosomal protein L32P
					43		63.0293		
FN1365	0.059	15.494	1.367e-1	6.849e-1	186	136	212.7928	177.0199	AAL95561.1| GTP-binding protein
					154	244	225.7330	244.0000	
FN1366	1.709	15.755	1.438e-5	5.293e-6	350	83	400.4166	108.0342	AAL95562.1| Triosephosphate isomerase
					307	152	450.0002	152.0000	
FN1368									AAL95564.1| COMF operon protein 3
						4		4.0000	

Log_2 Ratios: -6 -4 -2 0 2 4 6

Show detected proteins only
Show all proteins
Filter by category:
GO: amino acid transport

Proteins found: 1344

Enter (or paste) list of ORFs

Find ORFs

Test	Cutoff
q-Value	.005
p-Value	

	Signif	Direction	Applies To
red	yes	+	ratios, bars
yellow	no	n/a	bars
green	yes	−	ratios, bars
pink	yes	+	*p*-, *q*-Values
light green	yes	−	*p*-, *q*-Values

Dot Plots Dot Plots

Protein	*FnPgSg vs FnSg* Log$_2$ Ratio	Log$_2$ Sum	*q*-Value	*p*-Value	Raw *FnPgSg*	Raw *FnSg*	Normalized *FnPgSg*	Normalized *FnSg*	Description
FN1371									AAL95567.1| Ribonuclease HII
						5		5.0000	
FN1373									AAL95569.1| regulatory protein
						5		5.0000	
FN1374	0.364	6.364			9		10.2964		AAL95570.1| Transcriptional regulator
						8		8.0000	
FN1375	-0.775	10.249	1.654e-2	6.324e-2	21	44	24.0250	57.2711	AAL95571.1| Citrate-sodium symport
					20	34	29.3160	34.0000	
FN1376	-1.704	18.409	3.514e-3	9.385e-3	319	647	364.9511	842.1459	AAL95572.1| Oxaloacetate decarboxylase alpha chain
					197	1288	288.7623	1288.0000	
FN1377						9		11.7145	AAL95573.1| CITG protein
FN1378	0.068	9.747			23	22	26.3131	28.6356	AAL95574.1| Citrate lyase acyl carrier protein
					23		33.7134		
FN1379	-1.098	16.327	2.599e-5	1.329e-5	185	328	211.6488	426.9302	AAL95575.1| Citrate lyase beta chain
					123	412	180.2932	412.0000	
FN1380	-0.871	18.404	1.935e-2	7.586e-2	359	431	410.7130	560.9967	AAL95576.1| Citrate lyase beta chain
					314	1032	460.2608	1032.0000	
FN1381									AAL95577.1| unknown
					3		4.3974		
FN1382									AAL95578.1| ATPase
						7		7.0000	
FN1383	-2.143	5.701			3	11	3.4321	14.3178	AAL95579.1| DNA polymerase III alpha subunit
						16		16.0000	
FN1385						4		5.2065	AAL95581.1| Hypothetical protein
						17		17.0000	
FN1386	-0.788	8.713	1.139e-2	4.104e-2	17	16	19.4488	20.8259	AAL95582.1| SWF/SNF family helicase
					8	33	11.7264	33.0000	
FN1391	0.441	10.980	2.531e-2	1.029e-1	39	37	44.6178	48.1598	AAL95584.1| Acetyltransferase
					41	29	60.0977	29.0000	
FN1392	1.640	13.674	9.612e-5	8.249e-5	185	48	211.6488	62.4776	AAL95585.1| SSU ribosomal protein S16P
					131	67	192.0196	67.0000	

Log$_2$ Ratios: -6 -4 -2 0 2 4 6

Show detected proteins only
Show all proteins
Filter by category:
GO: amino acid transport

Proteins found: 1344

Enter (or paste) list of ORFs

Find ORFs

Test	Cutoff
q-Value	.005
p-Value	

	Signif	Direction	Applies To
red	yes	+	ratios, bars
yellow	no	n/a	bars
green	yes	−	ratios, bars
pink	yes	+	*p*- , *q*-Values
light green	yes	−	

Dot Plots Dot Plots

Protein	FnPgSg vs FnSg: Log_2 Ratio	Log_2 Sum	*q*-Value	*p*-Value	Raw: *FnPgSg*	Raw: *FnSg*	Normalized: *FnPgSg*	Normalized: *FnSg*	Description
**FN1393**	-0.400	10.520	1.032e-2	3.637e-2	25	30	28.6012	39.0485	AAL95586.1| Signal recognition particle, subunit FFH/SRP54
					26	49	38.1108	49.0000	
**FN1397**	1.517	12.981	2.961e-3	7.552e-3	157	44	179.6154	57.2711	AAL95590.1| Glutaminase
					85	49	124.5929	49.0000	
**FN1398**	2.140	13.100	2.006e-7	1.127e-8	171	34	195.6321	44.2550	AAL95591.1| Amino acid carrier protein alsT
					135	45	197.8828	45.0000	
**FN1399**									AAL95592.1| Hypothetical cytosolic protein
						3		3.0000	
**FN1400**									AAL95593.1| serine/threonine kinase
					7		10.2606		
**FN1406**	-0.294	12.198	1.098e-2	3.923e-2	48	59	54.9143	76.7954	AAL95599.1| Histidine ammonia-lyase
					47	75	68.8925	75.0000	
**FN1407**	-0.828	9.272	3.635e-2	1.551e-1	7	34	8.0083	44.2550	AAL95600.1| Glutamate formiminotransferase
					20	22	29.3160	22.0000	
**FN1411**	-2.016	17.207	1.444e-4	1.406e-4	210	594	240.2499	773.1602	AAL95604.1| Threonine dehydratase
					100	791	146.5799	791.0000	
**FN1412**						6		7.8097	AAL95605.1| 5-methylthioribose kinase
						10		10.0000	
**FN1413**	-0.221	3.779			3		3.4321		AAL95606.1| Translation initiation factor EIF-2B subunit 1
						4		4.0000	
**FN1415**									AAL95608.1| NADH-dependent butanol dehydrogenase A
						13		13.0000	
**FN1416**	0.339	4.339			5		5.7202		AAL95609.1| Transcriptional regulator, GntR family
					3	4	4.3974	4.0000	
**FN1417**	-0.807	5.839			5	10	5.7202	13.0162	AAL95610.1| L-fuculose phosphate aldolase
						7		7.0000	
**FN1418**						3		3.9048	AAL95611.1| Transcriptional regulator, GntR family
						3		3.0000	
**FN1419**	-0.389	23.078	3.057e-2	1.273e-1	2312	2104	2645.0375	2738.6012	AAL95612.1| Methionine gamma-lyase
					1743	4072	2554.8870	4072.0000	
**FN1420**					14		16.0167		AAL95613.1| NA+/H+ antiporter NHAC

Show detected proteins only
Show all proteins
Filter by category:
GO: amino acid transport

Proteins found: 1344

Enter (or paste) list of ORFs

Find ORFs

Test	Cutoff
q-Value	.005
p-Value	

Signif	Direction	Applies To
yes	+	ratios, bars
no	n/a	bars
yes	−	ratios, bars
yes	+	*p*- , *q*-Values
yes	−	

Dot Plots Dot Plots

Protein	*FnPgSg vs FnSg* Log$_2$ Ratio	Log$_2$ Sum	*q*-Value	*p*-Value	Raw *FnPgSg*	Raw *FnSg*	Normalized *FnPgSg*	Normalized *FnSg*	Description
FN1421	-0.388	21.141	1.186e-5	3.823e-6	1134	1356	1297.3497	1764.9921	AAL95614.1| Pyruvate-flavodoxin oxidoreductase
					929	1714	1361.7269	1714.0000	
FN1423	-0.043	15.872	1.138e-1	5.554e-1	227	186	259.6988	242.1007	AAL95616.1| Flavoprotein
					152	255	222.8014	255.0000	
FN1424	-0.164	17.884	7.677e-2	3.597e-1	478	362	546.8546	471.1852	AAL95617.1| ACYL-COA dehydrogenase, short-chain specific
					261	570	382.5734	570.0000	
FN1426	-0.819	15.789	9.156e-4	1.726e-3	130	232	148.7262	301.9750	AAL95619.1| Serine protease
					143	330	209.6092	330.0000	
FN1427					6		6.8643		AAL95620.1| Phenazine biosynthesis protein phzF
					4		5.8632		
FN1432	2.819	6.819			25		28.6012		AAL95625.1| Leucine-, isoleucine-, valine-, threonine-, and alanine-binding protein
					19	4	27.8502	4.0000	
FN1433	0.859	16.060	1.59e-3	3.476e-3	336	150	384.3999	195.2425	AAL95626.1| Short chain dehydrogenase
					218	193	319.5441	193.0000	
FN1434	-0.590	13.843	5.628e-4	8.827e-4	92	114	105.2524	148.3843	AAL95627.1| Tetratricopeptide repeat family protein
					63	149	92.3453	149.0000	
FN1437	1.182	13.312	1.097e-3	2.193e-3	117	56	133.8535	72.8905	AAL95630.1| LSU ribosomal protein L28P
					116	61	170.0326	61.0000	
FN1439	1.874	8.183	2.529e-3	6.227e-3	34	6	38.8976	7.8097	AAL95632.1| Transcriptional regulator, DeoR family
					18	10	26.3844	10.0000	
FN1440	1.337	9.961	1.364e-3	2.869e-3	48	9	54.9143	11.7145	AAL95633.1| 1-phosphofructokinase
					31	28	45.4398	28.0000	
FN1441	0.270	14.298	1.316e-2	4.852e-2	129	111	147.5821	144.4794	AAL95634.1| PTS system, fructose-specific IIABC component
					112	114	164.1694	114.0000	
FN1444	0.054	17.275	1.14e-1	5.564e-1	375	274	429.0178	356.6429	AAL95637.1| GMP synthase (glutamine-hydrolyzing)
					261	425	382.5734	425.0000	
FN1445	1.199	9.100	9.208e-6	2.511e-6	30	13	34.3214	16.9210	AAL95638.1| DNA helicase
					25	14	36.6450	14.0000	
FN1448									AAL95641.1| Hypothetical cytosolic protein
						4		4.0000	
FN1449	3.275	12.524	2.129e-3	4.994e-3	165	21	188.7678	27.3339	AAL95642.1| Fusobacterium outer membrane protein family
					197	22	288.7623	22.0000	

Log$_2$ Ratios: -6 -4 -2 0 2 4 6

Show detected proteins only
Show all proteins
Filter by category:
GO: amino acid transport

Proteins found: 1344

Enter (or paste) list of ORFs

Find ORFs

Test	Cutoff
q-Value	.005
p-Value	

	Signif	Direction	Applies To
red	yes	+	ratios, bars
yellow	no	n/a	bars
green	yes	−	ratios, bars
pink	yes	+	*p*-, *q*-Values
light green	yes	−	

Dot Plots Dot Plots

Protein	*FnPgSg vs FnSg* Log$_2$ Ratio	Log$_2$ Sum	*q*-Value	*p*-Value	Raw *FnPgSg*	Raw *FnSg*	Normalized *FnPgSg*	Normalized *FnSg*	Description
FN1450	-1.635	8.842			11	29	12.5845	37.7469	AAL95643.1| Integral membrane protein
					8		11.7264		
FN1451	-0.949	15.265	1.036e-4	9.063e-5	110	204	125.8452	265.5298	AAL95644.1| Cell division protein ftsZ
					109	286	159.7720	286.0000	
FN1452	-0.977	13.505	2.168e-4	2.45e-4	78	108	89.2357	140.5746	AAL95645.1| Cell division protein ftsA
					44	162	64.4951	162.0000	
FN1454	0.287	9.481	1.131e-1	5.516e-1	26	8	29.7452	10.4129	AAL95647.1| D-alanine--D-alanine ligase
					20	38	29.3160	38.0000	
FN1455	-0.498	8.134	3.193e-2	1.337e-1	8	16	9.1524	20.8259	AAL95648.1| UDP-N-acetylenolpyruvoylglucosamine reductase
					13	19	19.0554	19.0000	
FN1456	-0.332	11.712	1.046e-2	3.699e-2	39	53	44.6178	68.9857	AAL95649.1| UDP-N-acetylmuramate--alanine ligase
					40	61	58.6319	61.0000	
FN1457	-1.085	10.262	1.183e-2	4.286e-2	19	50	21.7369	65.0808	AAL95650.1| UDP-N-acetylglucosamine-N-acetylmuramyl-Pentapeptide pyrophosphoryl-undecaprenol N-[illegible]
					18	37	26.3844	37.0000	
FN1458	-0.481	11.529	2.93e-2	1.213e-1	42	38	48.0500	49.4614	AAL95651.1| UDP-N-acetylmuramoylalanine--D-glutamate ligase
					30	79	43.9740	79.0000	
FN1461	-0.068	8.745	1.704e-1	8.883e-1	20	8	22.8809	10.4129	AAL95654.1| Histidinol-phosphatase
					12	32	17.5896	32.0000	
FN1462						3		3.9048	AAL95655.1| Transcriptional regulator, GntR family
FN1463	0.238	17.657	9.733e-4	1.869e-3	448	332	512.5332	432.1367	AAL95656.1| pyridoxine biosynthesis protein
					324	405	474.9187	405.0000	
FN1464	-1.262	15.882	8.35e-6	2.115e-6	125	302	143.0059	393.0882	AAL95657.1| 1-deoxyxylulose-5-phosphate synthase
					119	368	174.4300	368.0000	
FN1470	4.001	8.617	2.536e-4	3.074e-4	63	3	72.0750	3.9048	AAL95663.1| Hypothetical protein
					59	6	86.4821	6.0000	
FN1471	0.533	4.499			5	3	5.7202	3.9048	AAL95664.1| LACI-family transcription regulator
						4		4.0000	
FN1472	2.200	11.564	1.217e-7	4.607e-9	105	21	120.1250	27.3339	AAL95665.1| N-acetylneuraminate-binding protein
					79	24	115.7981	24.0000	
FN1475	3.394	10.037			103		117.8369		AAL95668.1| N-acetylneuraminate lyase
					63	10	92.3453	10.0000	

Log$_2$ Ratios: -6 -4 -2 0 2 4 6

Show detected proteins only
Show all proteins
Filter by category:
GO: amino acid transport

Proteins found: 1344

Enter (or paste) list of ORFs

Find ORFs

Test	Cutoff
q-Value	.005
p-Value	

	Signif	Direction	Applies To
(red)	yes	+	ratios, bars
(yellow)	no	n/a	bars
(green)	yes	–	ratios, bars
(pink)	yes	+	*p*-, *q*-Values
(light green)	yes	–	

Dot Plots | Dot Plots

Protein	*FnPgSg* vs *FnSg* Log$_2$ Ratio	Log$_2$ Sum	*q*-Value	*p*-Value	Raw *FnPgSg*	Raw *FnSg*	Normalized *FnPgSg*	Normalized *FnSg*	Description
FN1476					11		12.5845		AAL95669.1| N-acetylmannosamine-6-phosphate 2-epimerase
					9		13.1922		
FN1478	-0.659	8.102				16		20.8259	AAL95671.1| Hypothetical protein
					9		13.1922		
FN1479	-0.050	9.109	1.613e-1	8.319e-1	25	16	28.6012	20.8259	AAL95672.1| Hypothetical protein
					12	27	17.5896	27.0000	
FN1480	-2.243	7.997	3.816e-5	2.332e-5	9	25	10.2964	32.5404	AAL95673.1| MG2+ transporter MGTE
					3	37	4.3974	37.0000	
FN1481	-1.121	5.509			4	3	4.5762	3.9048	AAL95674.1| Queuine tRNA-ribosyltransferase
						16		16.0000	
FN1482	-2.395	10.891	8.523e-4	1.576e-3	14	66	16.0167	85.9067	AAL95675.1| Guanosine-3',5'-bis (Diphosphate) 3'-pyrophosphohydrolase
					15	114	21.9870	114.0000	
FN1483	1.321	10.557	9.241e-6	2.527e-6	56	17	64.0667	22.1275	AAL95676.1| Adenine phosphoribosyltransferase
					40	27	58.6319	27.0000	
FN1484	0.116	5.969	1.375e-1	6.898e-1	8	4	9.1524	5.2065	AAL95677.1| Tetratricopeptide repeat family protein
					5	10	7.3290	10.0000	
FN1485									AAL95678.1| Transporter
						4		4.0000	
FN1486	0.017	4.661			5		5.7202		AAL95679.1| magnesium and cobalt efflux protein CorC
					3	5	4.3974	5.0000	
FN1487	0.453	14.703	2.011e-2	7.932e-2	197	110	225.3773	143.1778	AAL95681.1| Chorismate mutase
					107	136	156.8405	136.0000	
FN1488	-0.320	7.855			11		12.5845		AAL95682.1| Methylenetetrahydrofolate dehydrogenase (NADP+)
					10	17	14.6580	17.0000	
FN1489	-1.407	7.710	2.615e-3	6.485e-3	4	17	4.5762	22.1275	AAL95683.1| Methionyl-tRNA formyltransferase
					9	25	13.1922	25.0000	
FN1490	-2.020	8.023			7	33	8.0083	42.9533	AAL95684.1| putative regulatory protein
						22		22.0000	
FN1491						15		19.5242	AAL95685.1| PTS system, IIA component
						17		17.0000	
FN1492									AAL95686.1| DNA repair protein recO
						4		4.0000	

Log$_2$ Ratios: -6 -4 -2 0 2 4 6

- Show detected proteins only
- Show all proteins
- Filter by category: GO: amino acid transport

Proteins found: 1344

Enter (or paste) list of ORFs

Find ORFs

Test	Cutoff
q-Value	.005
p-Value	

Color	Signif	Direction	Applies To
red	yes	+	ratios, bars
yellow	no	n/a	bars
green	yes	−	ratios, bars
light red	yes	+	*p*- , *q*-Values
light green	yes	−	

Dot Plots | Dot Plots

Protein	FnPgSg vs FnSg: Log_2 Ratio	Log_2 Sum	*q*-Value	*p*-Value	Raw: *FnPgSg*	Raw: *FnSg*	Normalized: *FnPgSg*	Normalized: *FnSg*	Description
**FN1493**						5		6.5081	AAL95687.1| Hypothetical protein
						5		5.0000	
**FN1494**	0.492	7.386	2.162e-2	8.641e-2	14	6	16.0167	7.8097	AAL95680.1| Rod shape-determining protein mreC
					10	14	14.6580	14.0000	
**FN1496**	0.492	7.386	2.162e-2	8.641e-2	14	6	16.0167	7.8097	AAL95680.1| Rod shape-determining protein mreC
					10	14	14.6580	14.0000	
**FN1499**	2.421	11.593	8.024e-4	1.446e-3	98	20	112.1166	26.0323	AAL93625.1| Cell surface protein
					99	22	145.1141	22.0000	
**FN1501**									AAL93627.1| Nickel transport ATP-binding protein nikD
					3		4.3974		
**FN1504**	-0.001	13.807	1.875e-1	9.983e-1	117	121	133.8535	157.4956	AAL93630.1| Nickel-binding protein
					72	82	105.5375	82.0000	
**FN1505**	-0.313	15.185	1.25e-2	4.571e-2	158	183	180.7595	238.1958	AAL93631.1| 6,7-dimethyl-8-ribityllumazine synthase
					113	192	165.6352	192.0000	
**FN1506**	1.242	7.582			18		20.5929		AAL93632.1| Diaminohydroxyphosphoribosylaminopyrimidine deaminase
					15	9	21.9870	9.0000	
**FN1507**									AAL93633.1| Riboflavin synthase alpha chain
						8		8.0000	
**FN1508**	1.190	13.143	2.274e-3	5.431e-3	123	33	140.7178	42.9533	AAL93634.1| GTP cyclohydrolase II
					100	83	146.5799	83.0000	
**FN1512**					3		3.4321		AAL93638.1| hypothetical exported 24-amino acid repeat protein
					3		4.3974		
**FN1517**	0.717	15.358	4.067e-5	2.59e-5	235	115	268.8511	149.6859	AAL93643.1| Leucyl-tRNA synthetase
					175	170	256.5148	170.0000	
**FN1518**									AAL93644.1| RNA polymerase sigma-H factor
						6		6.0000	
**FN1519**	0.452	8.942	2.137e-3	5.019e-3	21	13	24.0250	16.9210	AAL93645.1| 23S rRNA methyltransferase
					19	21	27.8502	21.0000	
**FN1520**	1.873	12.087	3.96e-4	5.496e-4	122	23	139.5738	29.9372	AAL93646.1| UDP-N-acetylglucosamine 1-carboxyvinyltransferase
					77	39	112.8665	39.0000	
**FN1523**	1.706	14.503	1.385e-5	4.947e-6	253	72	289.4440	93.7164	AAL93649.1| Dipeptide-binding protein
					178	75	260.9122	75.0000	

Log_2 Ratios: -6 -4 -2 0 2 4 6

Show detected proteins only
Show all proteins
Filter by category:
GO: amino acid transport

Proteins found: 1344

Enter (or paste) list of ORFs

Find ORFs

Test	Cutoff
q-Value	.005
p-Value	

	Signif	Direction	Applies To
red	yes	+	ratios, bars
yellow	no	n/a	bars
green	yes	−	ratios, bars
pink	yes	+	*p*- , *q*-Values
light green	yes	−	

Dot Plots | Dot Plots

Protein	FnPgSg vs FnSg: Log_2 Ratio	Log_2 Sum	*q*-Value	*p*-Value	Raw: *FnPgSg*	Raw: *FnSg*	Normalized: *FnPgSg*	Normalized: *FnSg*	Description
FN1525									AAL93651.1| Dipeptide transport ATP-binding protein dppF
					6		8.7948		
FN1526	1.911	19.903	4.134e-4	5.808e-4	1491	332	1705.7746	432.1367	AAL93652.1| Fusobacterium outer membrane protein family
					1457	589	2135.6686	589.0000	
FN1527	2.156	11.778	2.965e-4	3.802e-4	97	17	110.9726	22.1275	AAL93653.1| Hypothetical protein
					95	34	139.2509	34.0000	
FN1528	1.030	12.745	3.81e-3	1.044e-2	84	33	96.1000	42.9533	AAL93654.1| Hypothetical protein
					96	73	140.7167	73.0000	
FN1529	2.305	10.397	2.849e-3	7.201e-3	53	20	60.6345	26.0323	AAL93655.1| Hypothetical protein
					70	7	102.6059	7.0000	
FN1531	-0.110	11.514	1.356e-1	6.788e-1	59	41	67.4988	53.3663	AAL93657.1| murein hydrolase export regulator
					25	59	36.6450	59.0000	
FN1533	0.849	18.575	1.527e-4	1.522e-4	744	329	851.1712	428.2318	AAL93659.1| Electron transfer flavoprotein alpha-subunit
					564	503	826.7104	503.0000	
FN1534	0.149	19.624	1.102e-2	3.942e-2	868	651	993.0331	847.3524	AAL93660.1| Electron transfer flavoprotein beta-subunit
					614	860	900.0003	860.0000	
FN1535	-0.225	21.166	3.56e-2	1.514e-1	1208	1107	1382.0092	1440.8895	AAL93661.1| Acyl-CoA dehydrogenase, short-chain specific
					993	1875	1455.5380	1875.0000	
FN1536	-1.018	19.518	3.619e-3	9.751e-3	539	806	616.6415	1049.1029	AAL93662.1| (S)-2-hydroxy-acid oxidase chain D
					410	1417	600.9774	1417.0000	
FN1537									AAL93663.1| Arsenical pump-driving ATPase
						5		5.0000	
FN1538	-1.367	8.095			9	17	10.2964	22.1275	AAL93664.1| Arsenical pump-driving ATPase
						31		31.0000	
FN1539	0.000	15.351	1.875e-1	9.985e-1	187	162	213.9369	210.8619	AAL93665.1| Iron-sulfur cluster-binding protein
					133	198	194.9512	198.0000	
FN1540	-1.518	16.012	7.272e-5	5.677e-5	122	355	139.5738	462.0739	AAL93666.1| Iron-sulfur cluster-binding protein
					112	408	164.1694	408.0000	
FN1544	-1.629	16.905	3.492e-4	4.696e-4	156	428	178.4714	557.0919	AAL93670.1| Probable electron transfer flavoprotein-quinone oxidoreductase ydiS
					150	675	219.8698	675.0000	
FN1545	-0.558	9.924	5.84e-3	1.832e-2	18	32	20.5929	41.6517	AAL93671.1| Ferredoxin like protein
					21	34	30.7818	34.0000	

Log$_2$ Ratios: -6 -4 -2 0 2 4 6

Show detected proteins only
Show all proteins
Filter by category:
GO: amino acid transport

Proteins found: 1344

Enter (or paste) list of ORFs

Find ORFs

Test	Cutoff
q-Value	.005
p-Value	

Color	Signif	Direction	Applies To
red	yes	+	ratios, bars
yellow	no	n/a	bars
green	yes	−	ratios, bars
pink	yes	+	*p*- , *q*-Values
light green	yes	−	

Dot Plots Dot Plots

Protein	*FnPgSg vs FnSg* Log$_2$ Ratio	Log$_2$ Sum	*q*-Value	*p*-Value	Raw *FnPgSg*	Raw *FnSg*	Normalized *FnPgSg*	Normalized *FnSg*	Description
**FN1546**	0.061	21.076	7.716e-2	3.617e-1	1330	1041	1521.5830	1354.9828	AAL93672.1| Protein Translation Elongation Factor G (EF-G)
					1034	1557	1515.6358	1557.0000	
**FN1547**	0.547	14.320	3.02e-4	3.895e-4	151	95	172.7512	123.6536	AAL93673.1| PTS permease for N-acetylglucosamine and glucose
					118	113	172.9642	113.0000	
**FN1548**	0.847	10.823	2.627e-2	1.073e-1	37	38	42.3298	49.4614	AAL93674.1| Hypothetical protein
					49	14	71.8241	14.0000	
**FN1549**	0.597	19.887	7.166e-3	2.351e-2	1019	750	1165.7843	976.2124	AAL93675.1| Stomatin like protein
					857	625	1256.1894	625.0000	
**FN1552**									AAL93678.1| abortive phage resistance protein
						9		9.0000	
**FN1553**	0.105	9.025	1.563e-1	8.012e-1	26	10	29.7452	13.0162	AAL93679.1| abortive phage resistance protein
					12	31	17.5896	31.0000	
**FN1554**	0.386	15.521	2.281e-2	9.171e-2	195	114	223.0892	148.3843	AAL93680.1| Fusobacterium outer membrane protein family
					186	231	272.6385	231.0000	
**FN1555**	0.422	24.514	3.167e-5	1.753e-5	5050	3291	5777.4392	4283.6201	AAL93681.1| Protein Translation Elongation Factor Tu
					3790	4174	5555.3767	4174.0000	
**FN1556**	1.439	19.980	9.46e-5	8.076e-5	1392	372	1592.5139	484.2014	AAL93682.1| Protein Translation Elongation Factor G (EF-G)
					1199	751	1757.4925	751.0000	
**FN1557**	1.540	15.860	1.154e-4	1.039e-4	381	113	435.8820	147.0827	AAL93683.1| SSU ribosomal protein S7P
					270	139	395.7656	139.0000	
**FN1558**									AAL93684.1| SSU ribosomal protein S12P
					83		121.6613		
**FN1560**	-1.383	14.767	9.93e-6	2.895e-6	86	200	98.3881	260.3233	AAL93686.1| unknown
					74	279	108.4691	279.0000	
**FN1562**	-1.406	6.438			5	11	5.7202	14.3178	AAL93688.1| Phospho-2-dehydro-3-deoxyheptonate aldolase
						16		16.0000	
**FN1576**						5		6.5081	AAL93691.1| ATPases and helicase subunits involved in DNA replication
**FN1577**	-1.213	16.089	2.63e-4	3.247e-4	152	325	173.8952	423.0254	AAL93692.1| Rod shape-determining protein mreB
					118	381	172.9642	381.0000	
**FN1579**	-0.718	12.817	1.064e-3	2.105e-3	62	76	70.9309	98.9229	AAL93694.1| Cysteinyl-tRNA synthetase
					42	119	61.5635	119.0000	

Log$_2$ Ratios: -6 -4 -2 0 2 4 6

Show detected proteins only
Show all proteins
Filter by category:
GO: amino acid transport

Proteins found: 1344

Enter (or paste) list of ORFs

Find ORFs

Test	Cutoff
q-Value	.005
p-Value	

	Signif	Direction	Applies To
red	yes	+	ratios, bars
yellow	no	n/a	bars
green	yes	–	ratios, bars
pink	yes	+	*p*- , *q*-Values
light green	yes	–	

Dot Plots Dot Plots

Protein	FnPgSg vs FnSg Log_2 Ratio	Log_2 Sum	*q*-Value	*p*-Value	Raw *FnPgSg*	Raw *FnSg*	Normalized *FnPgSg*	Normalized *FnSg*	Description
**FN1580**									AAL93695.1| 2-C-methyl-D-erythritol 4-phosphate cytidylyltransferase
						3		3.0000	
**FN1581**	-1.020	11.384	5.226e-3	1.589e-2	34	67	38.8976	87.2083	AAL93696.1| DNA mismatch repair protein mutS
					23	60	33.7134	60.0000	
**FN1582**						12		15.6194	AAL93697.1| Hypothetical protein
						30		30.0000	
**FN1586**						9		11.7145	AAL93701.1| O-succinylbenzoate-CoA synthase
						9		9.0000	
**FN1589**	-0.478	9.546	2.575e-7	1.687e-8	20	25	22.8809	32.5404	AAL93704.1| LexA repressor
					16	32	23.4528	32.0000	
**FN1590**						3		3.9048	AAL93705.1| Hypothetical lipoprotein
						4		4.0000	
**FN1591**	0.390	15.478	1.912e-2	7.478e-2	202	173	231.0976	225.1797	AAL93706.1| RNFB-related protein
					176	148	257.9806	148.0000	
**FN1592**	1.552	6.722							AAL93707.1| Na(+)-translocating NADH-quinone reductase subunit D
					12	6	17.5896	6.0000	
**FN1594**	1.586	11.842	4.133e-5	2.661e-5	90	23	102.9643	29.9372	AAL93709.1| Nitrogen fixation protein RNFG
					73	40	107.0033	40.0000	
**FN1595**	0.832	14.394	1.055e-4	9.263e-5	177	76	202.4964	98.9229	AAL93710.1| Na(+)-translocating NADH-quinone reductase subunit B
					129	121	189.0880	121.0000	
**FN1596**	1.183	18.578	2.504e-4	3.016e-4	748	359	855.7474	467.2803	AAL93711.1| Nitrogen fixation iron-sulphur protein RNFC
					702	363	1028.9906	363.0000	
**FN1597**					4		4.5762		AAL93712.1| Peptidyl-tRNA hydrolase
**FN1600**						5		6.5081	AAL93715.1| tRNA pseudouridine synthase A
						7		7.0000	
**FN1601**						5		6.5081	AAL93716.1| Hypothetical cytosolic protein
						12		12.0000	
**FN1602**									AAL93717.1| Hypothetical cytosolic protein
					4		5.8632		
**FN1603**	0.688	5.094	4.226e-2	1.843e-1	4	4	4.5762	5.2065	AAL93718.1| 2',3'-cyclic nucleotide 3'-phosphodiesterase
					7	4	10.2606	4.0000	

Log_2 Ratios: -6 -4 -2 0 2 4 6

Show detected proteins only
Show all proteins
Filter by category:
GO: amino acid transport

Proteins found: 1344

Enter (or paste) list of ORFs
Find ORFs

Test	Cutoff
q-Value	.005
p-Value	

	Signif	Direction	Applies To
red	yes	+	ratios, bars
yellow	no	n/a	bars
green	yes	−	ratios, bars
pink	yes	+	*p*-, *q*-Values
light green	yes	−	

Dot Plots Dot Plots

Protein	FnPgSg vs FnSg Log$_2$ Ratio	Log$_2$ Sum	*q*-Value	*p*-Value	Raw *FnPgSg*	Raw *FnSg*	Normalized *FnPgSg*	Normalized *FnSg*	Description
FN1605	-0.227	16.382	1.356e-5	4.77e-6	239	240	273.4273	312.3880	AAL93720.1| Adenylosuccinate synthetase
					182	320	266.7753	320.0000	
FN1606	-1.043	9.113	2.985e-4	3.836e-4	12	25	13.7286	32.5404	AAL93721.1| 3-deoxy-D-manno-octulosonic-acid transferase
					13	35	19.0554	35.0000	
FN1607	-0.734	7.838				10		13.0162	AAL93722.1| Cytidylate kinase
					8	26	11.7264	26.0000	
FN1608						7		9.1113	AAL93723.1| Ribosomal protein L11 methyltransferase
						9		9.0000	
FN1609	0.040	4.349			4	3	4.5762	3.9048	AAL93724.1| Hypothetical protein
						5		5.0000	
FN1610	-1.349	9.036	1.228e-5	4.038e-6	11	27	12.5845	35.1436	AAL93725.1| 33 kDa chaperonin
					11	38	16.1238	38.0000	
FN1613	0.174	8.672	1.115e-1	5.429e-1	17	10	19.4488	13.0162	AAL93728.1| Hypothetical protein
					16	25	23.4528	25.0000	
FN1614	0.395	8.451	5.26e-3	1.603e-2	17	12	19.4488	15.6194	AAL93729.1| MG(2+) chelatase family protein
					16	17	23.4528	17.0000	
FN1616	2.355	9.525			74		84.6595		AAL93731.1| N utilization substance protein B
					26	12	38.1108	12.0000	
FN1618	0.001	11.496	1.87e-1	9.948e-1	44	38	50.3381	49.4614	AAL93733.1| Hypothetical protein
					39	58	57.1661	58.0000	
FN1619	0.716	16.400	6.592e-3	2.122e-2	282	129	322.6214	167.9085	AAL93734.1| Hypothetical cytosolic protein
					294	291	430.9448	291.000	
FN1620	0.955	17.983	4.72e-5	3.227e-5	624	296	713.8856	385.2785	AAL93735.1| SSU ribosomal protein S2P
					480	346	703.5833	346.0000	
FN1621	1.508	19.702	1.141e-3	2.309e-3	1181	379	1351.1199	493.3127	AAL93736.1| Protein Translation Elongation Factor Ts
					1203	602	1763.3557	602.0000	
FN1622	1.127	14.655	3.045e-3	7.82e-3	224	51	256.2666	66.3824	AAL93737.1| Uridylate kinase
					149	151	218.4040	151.0000	
FN1623	2.164	10.409	3.734e-6	6.834e-7	66	16	75.5071	20.8259	AAL93738.1| Ribosome Recycling Factor (RRF)
					55	14	80.6189	14.0000	
FN1624	1.969	10.465			66		75.5071		AAL93739.1| Protein translocase subunit secY
					50	19	73.2899	19.0000	

Log$_2$ Ratios: -6 -4 -2 0 2 4 6

Show detected proteins only
Show all proteins
Filter by category:
GO: amino acid transport

Proteins found: 1344

Enter (or paste) list of ORFs
Find ORFs

Test	Cutoff
q-Value	.005
p-Value	

Color	Signif	Direction	Applies To
red	yes	+	ratios, bars
yellow	no	n/a	bars
green	yes	−	ratios, bars
pink	yes	+	*p*- , *q*-Values
light green	yes	−	*p*- , *q*-Values

Dot Plots Dot Plots

Protein	FnPgSg vs FnSg Log$_2$ Ratio	Log$_2$ Sum	*q*-Value	*p*-Value	Raw *FnPgSg*	Raw *FnSg*	Normalized *FnPgSg*	Normalized *FnSg*	Description
FN1625	0.865	13.221	6.324e-5	4.749e-5	114	59	130.4214	76.7954	AAL93740.1| LSU ribosomal protein L15P
					91	68	133.3877	68.0000	
FN1626	3.416	8.060			51		58.3464		AAL93741.1| LSU ribosomal protein L30P
					33	5	48.3714	5.0000	
FN1627	1.537	17.562	4.071e-3	1.141e-2	791	184	904.9415	239.4974	AAL93742.1| SSU ribosomal protein S5P
					405	277	593.6484	277.0000	
FN1628	3.952	11.883			242	12	276.8595	15.6194	AAL93743.1| LSU ribosomal protein L18P
					141		206.6776		
FN1629	0.485	15.761	4.258e-3	1.213e-2	217	167	248.2583	217.3700	AAL93744.1| LSU ribosomal protein L6P
					211	181	309.2835	181.0000	
FN1630	1.312	13.560	1.543e-2	5.831e-2	204	58	233.3857	75.4938	AAL93745.1| SSU ribosomal protein S8P
					77	64	112.8665	64.0000	
FN1631	3.112	7.043			27	3	30.8893	3.9048	AAL93746.1| SSU ribosomal protein S14P
					25		36.6450		
FN1632	0.663	16.499	9.545e-4	1.822e-3	326	208	372.9594	270.7362	AAL93747.1| LSU ribosomal protein L5P
					268	213	392.8340	213.0000	
FN1634	1.575	9.737			51	13	58.3464	16.9210	AAL93749.1| LSU ribosomal protein L24P
					29		42.5082		
FN1635	3.003	10.102	1.381e-3	2.914e-3	68	8	77.7952	10.4129	AAL93750.1| LSU ribosomal protein L14P
					75	13	109.9349	13.0000	
FN1636	-0.998	11.537	5.464e-4	8.462e-4	29	60	33.1774	78.0970	AAL93751.1| SSU ribosomal protein S17P
					30	76	43.9740	76.0000	
FN1637					34		38.8976		AAL93752.1| LSU ribosomal protein L29P
					31		45.4398		
FN1638	3.360	13.433	1.988e-8	3.763e-10	292	22	334.0618	28.6356	AAL93753.1| LSU ribosomal protein L16P
					232	37	340.0653	37.0000	
FN1639	1.360	19.260	1.117e-3	2.248e-3	1241	355	1419.7628	462.0739	AAL93754.1| SSU ribosomal protein S3P
					763	527	1118.4043	527.0000	
FN1640	3.707	14.280	8.917e-4	1.669e-3	380	30	434.7380	39.0485	AAL93755.1| LSU ribosomal protein L22P
					399	39	584.8536	39.0000	
FN1641	3.790	12.264	8.772e-5	7.307e-5	246	9	281.4357	11.7145	AAL93756.1| SSU ribosomal protein S19P
					164	26	240.3910	26.0000	

Log$_2$ Ratios: -6 -4 -2 0 2 4 6

Show detected proteins only
Show all proteins
Filter by category:
GO: amino acid transport

Proteins found: 1344

Enter (or paste) list of ORFs

Find ORFs

Test	Cutoff
q-Value	.005
p-Value	

	Signif	Direction	Applies To
red	yes	+	ratios, bars
yellow	no	n/a	bars
green	yes	−	ratios, bars
pink	yes	+	*p*- , *q*-Values
light green	yes	−	

Dot Plots Dot Plots

Protein	FnPgSg vs FnSg Log$_2$ Ratio	Log$_2$ Sum	*q*-Value	*p*-Value	Raw *FnPgSg*	Raw *FnSg*	Normalized *FnPgSg*	Normalized *FnSg*	Description
FN1642	0.455	15.741	5.236e-3	1.593e-2	233	175	266.5630	227.7829	AAL93757.1| LSU ribosomal protein L2P
					192	172	281.4333	172.0000	
FN1643	1.505	11.806	9.626e-6	2.727e-6	84	30	96.1000	39.0485	AAL93758.1| LSU ribosomal protein L23P
					72	32	105.5375	32.0000	
FN1644	0.626	20.263	2.405e-5	1.199e-5	1202	721	1375.1449	938.4655	AAL93759.1| LSU ribosomal protein L1E
					963	867	1411.5641	867.0000	
FN1645	3.258	16.412	5.771e-5	4.215e-5	764	76	874.0522	98.9229	AAL93760.1| LSU ribosomal protein L3P
					650	92	952.7691	92.0000	
FN1646	1.022	14.483	2.781e-3	6.99e-3	163	84	186.4797	109.3358	AAL93761.1| SSU ribosomal protein S10P
					167	103	244.7884	103.0000	
FN1647	2.174	15.936	1.044e-3	2.05e-3	530	82	606.3451	106.7326	AAL93762.1| Hypothetical protein
					312	129	457.3292	129.0000	
FN1652	0.445	12.827	1.013e-3	1.971e-3	88	60	100.6762	78.0970	AAL93767.1| Oligopeptide-binding protein oppA
					67	68	98.2085	68.0000	
FN1654	-1.656	12.293	2.333e-5	1.151e-5	39	98	44.6178	127.5584	AAL93769.1| Hypothetical protein
					24	124	35.1792	124.0000	
FN1655	-2.095	12.936	7.488e-3	2.483e-2	39	96	44.6178	124.9552	AAL93770.1| Hypothetical cytosolic protein
					28	241	41.0424	241.0000	
FN1656					21		24.0250		AAL93771.1| SSU ribosomal protein S18P
					27		39.5766		
FN1657	0.463	12.009	4.037e-3	1.128e-2	60	41	68.6428	53.3663	AAL93772.1| SSU ribosomal protein S6P
					56	56	82.0847	56.0000	
FN1658	0.888	14.810	1.659e-4	1.699e-4	189	90	216.2250	117.1455	AAL93773.1| Prolyl-tRNA synthetase
					167	132	244.7884	132.0000	
FN1660									AAL93775.1| ATP-dependent DNA helicase recG
						5		5.0000	
FN1661	1.229	10.064	4.444e-4	6.38e-4	44	19	50.3381	24.7307	AAL93776.1| Hypothetical cytosolic protein
					34	18	49.8372	18.0000	
FN1662	-0.563	10.324	6.141e-3	1.949e-2	31	30	35.4655	39.0485	AAL93777.1| Hypothetical protein
					16	48	23.4528	48.0000	
FN1663	1.003	7.720	5.653e-3	1.756e-2	18	5	20.5929	6.5081	AAL93778.1| Hypothetical protein
					14	14	20.5212	14.0000	

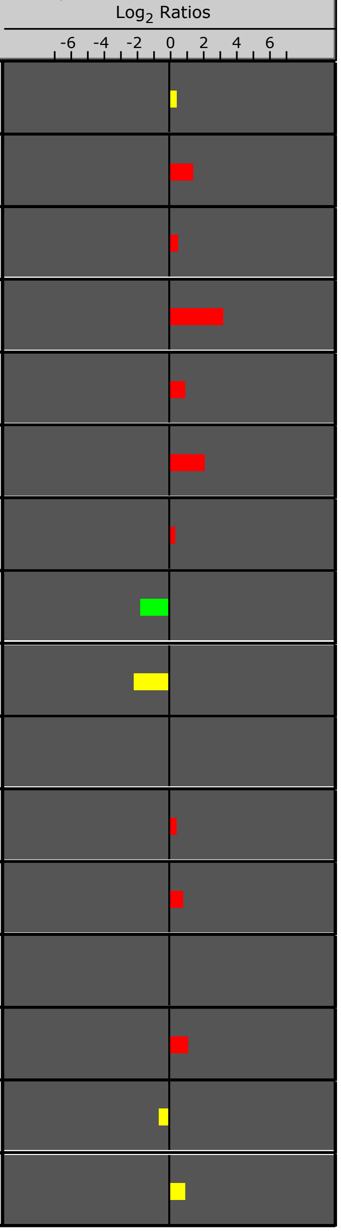

Show detected proteins only
Show all proteins
Filter by category:
GO: amino acid transport

Proteins found: 1344

Enter (or paste) list of ORFs

Find ORFs

Test	Cutoff
q-Value	.005
p-Value	

	Signif	Direction	Applies To
red	yes	+	ratios, bars
yellow	no	n/a	bars
green	yes	−	ratios, bars
pink	yes	+	*p*- , *q*-Values
light green	yes	−	

Dot Plots Dot Plots

Protein	FnPgSg vs FnSg: Log_2 Ratio	Log_2 Sum	*q*-Value	*p*-Value	Raw: *FnPgSg*	Raw: *FnSg*	Normalized: *FnPgSg*	Normalized: *FnSg*	Description	Log_2 Ratios (-6 -4 -2 0 2 4 6)
**FN1667**									AAL93782.1| dTDP-glucose 4,6-dehydratase	
						3		3.0000		
**FN1668**									AAL93783.1| Cholinephosphate cytidylyltransferase	
						3		3.0000		
**FN1670**	-0.252	9.763	5.485e-2	2.474e-1	28	21	32.0333	27.3339	AAL93785.1| Choline kinase	
					15	37	21.9870	37.0000		
**FN1676**									AAL93791.1| Transposase	
						3		3.0000		
**FN1679**	-0.622	17.766	2.498e-4	3.005e-4	354	423	404.9928	550.5838	AAL93794.1| LPS biosynthesis protein WbpG	
					243	621	356.1891	621.0000		
**FN1683**	0.343	11.442	7.21e-2	3.357e-1	68	42	77.7952	54.6679	AAL93798.1| Acetyltransferase	
					28	39	41.0424	39.0000		
**FN1684**	0.626	16.464	5.469e-3	1.683e-2	311	146	355.7987	190.0360	AAL93799.1| N-acetylneuraminate synthase	
					267	294	391.3682	294.0000		
**FN1685**	0.185	11.704	1.554e-2	5.879e-2	50	41	57.2024	53.3663	AAL93800.1| dTDP-4-dehydrorhamnose reductase	
					45	55	65.9609	55.0000		
**FN1686**	0.052	15.894	1.496e-1	7.61e-1	256	168	292.8761	218.6716	AAL93801.1| Spore coat polysaccharide biosynthesis protein spsF	
					143	266	209.6092	266.0000		
**FN1687**	0.302	15.375	3.05e-2	1.269e-1	222	117	253.9785	152.2891	AAL93802.1| Gluconate 5-dehydrogenase	
					139	219	203.7460	219.0000		
**FN1688**	1.180	12.300	2.947e-4	3.771e-4	92	31	105.2524	40.3501	AAL93803.1| Oxidoreductase	
					74	54	108.4691	54.0000		
**FN1689**	1.095	17.218	1.834e-4	1.941e-4	494	226	565.1594	294.1653	AAL93804.1| UDP-N-acetylglucosamine 4,6-dehydratase	
					393	240	576.0589	240.0000		
**FN1690**	-0.743	10.046	5.163e-3	1.566e-2	26	27	29.7452	35.1436	AAL93805.1| Hypothetical protein	
					14	49	20.5212	49.0000		
**FN1692**	-0.106	11.186	1.502e-1	7.645e-1	57	37	65.2107	48.1598	AAL93807.1| Glycosyl transferase	
					19	52	27.8502	52.0000		
**FN1693**	0.730	7.817	1.56e-2	5.904e-2	21	11	24.0250	14.3178	AAL93808.1| Hypothetical protein	
					10	9	14.6580	9.0000		
**FN1694**	1.307	12.090	2.917e-5	1.558e-5	88	33	100.6762	42.9533	AAL93809.1| UDP-N-acetyl-D-quinovosamine 4-epimerase	
					73	41	107.0033	41.0000		

Show detected proteins only
Show all proteins
Filter by category:
GO: amino acid transport

Proteins found: 1344

Enter (or paste) list of ORFs

Find ORFs

Test	Cutoff
q-Value	.005
p-Value	

	Signif	Direction	Applies To
red	yes	+	ratios, bars
yellow	no	n/a	bars
green	yes	−	ratios, bars
pink	yes	+	*p*- , *q*-Values
light green	yes	−	

Dot Plots | Dot Plots

Protein	FnPgSg vs FnSg Log$_2$ Ratio	Log$_2$ Sum	*q*-Value	*p*-Value	Raw *FnPgSg*	Raw *FnSg*	Normalized *FnPgSg*	Normalized *FnSg*	Description
FN1695	1.111	9.736	1.35e-3	2.833e-3	43	19	49.1940	24.7307	AAL93810.1| Probable quinovosaminephosphotransferae
					25	15	36.6450	15.0000	
FN1696	-0.387	15.295	3.749e-3	1.021e-2	163	161	186.4797	209.5603	AAL93811.1| UDP-N-acetylglucosamine 4,6-dehydratase
					112	249	164.1694	249.0000	
FN1697	-0.430	13.373	4.397e-3	1.269e-2	68	100	77.7952	130.1617	AAL93812.1| Hypothetical protein
					68	109	99.6743	109.0000	
FN1698	-1.310	14.499	5.128e-5	3.6e-5	78	183	89.2357	238.1958	AAL93813.1| dTDP-4-dehydrorhamnose reductase
					71	241	104.0717	241.0000	
FN1700						3		3.9048	AAL93815.1| Hypothetical protein
FN1701	-1.038	10.946	1.166e-3	2.371e-3	26	44	29.7452	57.2711	AAL93816.1| ABC transporter ATP-binding protein
					22	70	32.2476	70.0000	
FN1703	-0.907	13.890	1.637e-3	3.608e-3	74	144	84.6595	187.4328	AAL93818.1| ADP-L-glycero-D-manno-heptose-6-epimerase
					65	150	95.2769	150.0000	
FN1704	-0.700	6.703			7	10	8.0083	13.0162	AAL93819.1| Serine protease
						13		13.0000	
FN1708	0.145	18.177	1.074e-3	2.131e-3	488	406	558.2951	528.4563	AAL93823.1| Polyribonucleotide nucleotidyltransferase
					400	507	586.3194	507.0000	
FN1711					3		3.4321		AAL93826.1| Methyltransferase
					3		4.3974		
FN1713						14		18.2226	AAL93828.1| tRNA (Uracil-5-) -methyltransferase
						20		20.0000	
FN1715						9		11.7145	AAL93830.1| ATPase
						9		9.0000	
FN1716									AAL93831.1| Hypothetical protein
						5		5.0000	
FN1717	0.071	7.697	1.593e-1	8.197e-1	13	7	14.8726	9.1113	AAL93832.1| NAD-dependent DNA ligase
					10	19	14.6580	19.0000	
FN1718	0.131	17.212	1.109e-2	3.973e-2	372	284	425.5856	369.6591	AAL93833.1| Protein translocase subunit secA
					266	375	389.9024	375.0000	
FN1719	-0.707	16.963	6.007e-3	1.898e-2	261	405	298.5964	527.1547	AAL93834.1| Hypothetical protein
					178	386	260.9122	386.0000	

Log$_2$ Ratios: -6 -4 -2 0 2 4 6

Show detected proteins only
Show all proteins
Filter by category:
GO: amino acid transport

Proteins found: 1344

Enter (or paste) list of ORFs

Find ORFs

Test	Cutoff
q-Value	.005
p-Value	

	Signif	Direction	Applies To
red	yes	+	ratios, bars
yellow	no	n/a	bars
green	yes	−	ratios, bars
pink	yes	+	*p*- , *q*-Values
light green	yes	−	*p*- , *q*-Values

Dot Plots Dot Plots

Protein	*FnPgSg* vs *FnSg* Log$_2$ Ratio	Log$_2$ Sum	*q*-Value	*p*-Value	Raw *FnPgSg*	Raw *FnSg*	Normalized *FnPgSg*	Normalized *FnSg*	Description	Log$_2$ Ratios (-6 -4 -2 0 2 4 6)
**FN1722**						3		3.9048	AAL93837.1| Glucose inhibited division protein B	
						4		4.0000		
**FN1723**	0.386	12.797	1.054e-2	3.733e-2	93	65	106.3964	84.6051	AAL93838.1| Glucose inhibited division protein A	
					59	63	86.4821	63.0000		
**FN1724**	-1.586	8.096	2.555e-2	1.04e-1	9	11	10.2964	14.3178	AAL93839.1| Potassium uptake protein KtrA	
					6	43	8.7948	43.0000		
**FN1727**	-0.318	5.086			4	5	4.5762	6.5081	AAL93842.1| Chloride channel protein	
					4		5.8632			
**FN1728**	-0.573	5.985	1.109e-3	2.227e-3	5	8	5.7202	10.4129	AAL93843.1| Pyrrolidone-carboxylate peptidase	
					5	9	7.3290	9.0000		
**FN1730**	-1.565	7.838				20		26.0323	AAL93845.1| Para-aminobenzoate synthase component I	
					6	26	8.7948	26.0000		
**FN1731**	1.407	6.982			16	6	18.3048	7.8097	AAL93846.1| Anthranilate synthase component II	
						6		6.0000		
**FN1732**	-1.486	10.431	6.47e-4	1.081e-3	26	41	29.7452	53.3663	AAL93847.1| Hypothetical protein	
					10	71	14.6580	71.0000		
**FN1733**						9		11.7145	AAL93848.1| V-type sodium ATP synthase subunit D	
						22		22.0000		
**FN1734**	-0.453	14.675	6.476e-3	2.077e-2	102	151	116.6928	196.5441	AAL93849.1| V-type sodium ATP synthase subunit B	
					109	182	159.7720	182.0000		
**FN1735**	-2.255	13.388	7.896e-5	6.329e-5	38	184	43.4738	239.4974	AAL93850.1| V-type sodium ATP synthase subunit A	
					35	213	51.3030	213.0000		
**FN1736**	-2.650	9.033	1.827e-5	7.893e-6	7	42	8.0083	54.6679	AAL93851.1| V-type sodium ATP synthase subunit A	
					7	60	10.2606	60.0000		
**FN1737**						12		15.6194	AAL93852.1| V-type sodium ATP synthase subunit G	
						16		16.0000		
**FN1738**	-2.531	10.361	1.929e-4	2.079e-4	11	61	12.5845	79.3986	AAL93853.1| V-type sodium ATP synthase subunit C	
					12	95	17.5896	95.0000		
**FN1739**	1.565	6.969			17	5	19.4488	6.5081	AAL93854.1| V-type sodium ATP synthase subunit E	
					13		19.0554			
**FN1740**									AAL93855.1| V-type sodium ATP synthase subunit K	
						114		114.0000		

Show detected proteins only
Show all proteins
Filter by category:
GO: amino acid transport

Proteins found: 1344

Enter (or paste) list of ORFs

Find ORFs

Test	Cutoff
q-Value	.005
p-Value	

	Signif	Direction	Applies To
red	yes	+	ratios, bars
yellow	no	n/a	bars
green	yes	−	ratios, bars
pink	yes	+	*p*- , *q*-Values
light green	yes	−	*p*- , *q*-Values

Dot Plots | Dot Plots

Protein	FnPgSg vs FnSg Log_2 Ratio	Log_2 Sum	*q*-Value	*p*-Value	Raw *FnPgSg*	Raw *FnSg*	Normalized *FnPgSg*	Normalized *FnSg*	Description
FN1741	-2.249	8.187	3.242e-7	2.611e-8	6	28	6.8643	36.4453	AAL93856.1| V-type sodium ATP synthase subunit I
					6	38	8.7948	38.0000	
FN1745	-1.390	7.823	8.096e-4	1.464e-3	6	19	6.8643	24.7307	AAL93860.1| Cystathionine gamma-synthase
					8	24	11.7264	24.0000	
FN1746						8		10.4129	AAL93861.1| Cystathionine beta-lyase
						15		15.0000	
FN1752					3		3.4321		AAL93867.1| Regulatory protein TENI
FN1762						4		5.2065	AAL93875.1| Protein yaaA
FN1763	-2.251	9.999				55		71.5889	AAL93876.1| Hypothetical cytosolic protein
					10	68	14.6580	68.0000	
FN1764	-1.186	24.584	2.024e-7	1.149e-8	2954	5881	3379.5159	7654.8070	AAL93877.1| Enolase
					2231	7477	3270.1967	7477.0000	
FN1765	0.041	18.838	1.158e-1	5.666e-1	563	535	644.0987	696.3649	AAL93878.1| Pyruvate kinase
					508	653	744.6257	653.0000	
FN1780	1.150	8.735	1.46e-3	3.122e-3	23	9	26.3131	11.7145	AAL93879.1| Hypothetical protein
					24	16	35.1792	16.0000	
FN1781	0.095	18.183	5.114e-2	2.286e-1	496	374	567.4475	486.8046	AAL93880.1| LytB protein
					382	569	559.9351	569.0000	
FN1783									AAL93882.1| Ethanolamine utilization protein eutJ
						8		8.0000	
FN1784	-1.616	7.363				13		16.9210	AAL93883.1| unknown
					5	28	7.3290	28.0000	
FN1785	1.746	9.425			25	11	28.6012	14.3178	AAL93884.1| Hypothetical protein
					46		67.4267		
FN1786	0.549	11.463	6.926e-4	1.182e-3	56	36	64.0667	46.8582	AAL93885.1| ADP-heptose synthase
					44	41	64.4951	41.0000	
FN1787	1.323	9.137			40		45.7619		AAL93886.1| Tetratricopeptide repeat family protein
					20	15	29.3160	15.0000	
FN1788	0.638	8.890	6.02e-3	1.903e-2	27	13	30.8893	16.9210	AAL93887.1| 2C-methyl-D-erythritol 2,4-cyclodiphosphate synthase
					16	18	23.4528	18.0000	

Log_2 Ratios: -6 -4 -2 0 2 4 6

Show detected proteins only
Show all proteins
Filter by category:
GO: amino acid transport

Proteins found: 1344

Enter (or paste) list of ORFs

Find ORFs

Test	Cutoff
q-Value	.005
p-Value	

	Signif	Direction	Applies To
red	yes	+	ratios, bars
yellow	no	n/a	bars
green	yes	−	ratios, bars
pink	yes	+	*p*- , *q*-Values
light green	yes	−	

Dot Plots Dot Plots

Protein	*FnPgSg vs FnSg* Log$_2$ Ratio	Log$_2$ Sum	*q*-Value	*p*-Value	Raw *FnPgSg*	Raw *FnSg*	Normalized *FnPgSg*	Normalized *FnSg*	Description
**FN1790**	-2.203	8.995	1.027e-2	3.619e-2	12	23	13.7286	29.9372	AAL93889.1| Cob(I)alamin adenosyltransferase
					5	67	7.3290	67.0000	
**FN1791**						3		3.9048	AAL93890.1| Mutator MutT protein
						5		5.0000	
**FN1792**	2.306	24.232	4.189e-5	2.723e-5	8681	1257	9931.4752	1636.1320	AAL93891.1| Hypothetical protein
					6695	2356	9813.5217	2356.0000	
**FN1793**	-1.272	14.641	7.563e-5	5.977e-5	85	201	97.2440	261.6249	AAL93892.1| Phosphoenolpyruvate-protein phosphotransferase
					74	235	108.4691	235.0000	
**FN1794**	1.814	12.863	2.338e-3	5.629e-3	110	50	125.8452	65.0808	AAL93893.1| Phosphocarrier protein HPr
					135	27	197.8828	27.0000	
**FN1795**									AAL93894.1| Hypothetical protein
						12		12.0000	
**FN1796**									AAL93895.1| unknown
						8		8.0000	
**FN1797**	-1.268	11.275	1.183e-3	2.414e-3	33	68	37.7536	88.5099	AAL93896.1| Spermidine/putrescine transport ATP-binding protein potA
					18	66	26.3844	66.0000	
**FN1798**	-0.219	5.558	9.413e-3	3.258e-2	6	6	6.8643	7.8097	AAL93897.1| Spermidine/putrescine transport system permease protein potB
					4	7	5.8632	7.0000	
**FN1800**	-0.269	15.271	4.052e-2	1.758e-1	177	141	202.4964	183.5279	AAL93899.1| Peptidyl-prolyl cis-trans isomerase
					109	253	159.7720	253.0000	
**FN1801**	-1.701	7.704			7	20	8.0083	26.0323	AAL93900.1| Sodium/glutamate symport carrier protein
**FN1803**	1.345	7.962			22	6	25.1690	7.8097	AAL93902.1| Transcriptional regulator, TetR family
						12		12.0000	
**FN1804**						18		23.4291	AAL93903.1| Aminoacyl-histidine dipeptidase
						40		40.0000	
**FN1807**	1.109	16.659	1.354e-4	1.286e-4	407	186	465.6273	242.1007	AAL93906.1| Hypothetical protein
					327	196	479.3161	196.0000	
**FN1808**	2.154	8.084			30	6	34.3214	7.8097	AAL93907.1| Hypothetical protein
					24		35.1792		
**FN1809**	0.705	8.891	8.761e-3	2.993e-2	23	17	26.3131	22.1275	AAL93908.1| Iron/zinc/copper-binding protein
					20	12	29.3160	12.0000	

Log$_2$ Ratios: -6 -4 -2 0 2 4 6

Show detected proteins only
Show all proteins
Filter by category:
GO: amino acid transport

Proteins found: 1344

Enter (or paste) list of ORFs
Find ORFs

Test	Cutoff
q-Value	.005
p-Value	

Signif	Direction	Applies To
yes	+	ratios, bars
no	n/a	bars
yes	−	ratios, bars
yes	+	*p*- , *q*-Values
yes	−	

Dot Plots Dot Plots

Protein	FnPgSg vs FnSg Log$_2$ Ratio	Log$_2$ Sum	*q*-Value	*p*-Value	Raw *FnPgSg*	Raw *FnSg*	Normalized *FnPgSg*	Normalized *FnSg*	Description
FN1811	0.589	9.072	2.171e-2	8.683e-2	19	16	21.7369	20.8259	AAL93910.1| Manganese transport system ATP-binding protein mntA
					24	17	35.1792	17.0000	
FN1812	1.579	12.225	6.106e-4	9.948e-4	95	30	108.6845	39.0485	AAL93911.1| Manganese-binding protein
					89	41	130.4561	41.0000	
FN1813	1.152	7.295	7.008e-6	1.596e-6	16	6	18.3048	7.8097	AAL93912.1| Manganese-binding protein
					13	9	19.0554	9.0000	
FN1814	0.423	7.184			18	8	20.5929	10.4129	AAL93913.1| Hypothetical protein
					5		7.3290		
FN1816	-1.868	7.746			7		8.0083		AAL93915.1| unknown
					5	28	7.3290	28.0000	
FN1817	0.908	6.078			12		13.7286		AAL93916.1| Hemolysin
					6	6	8.7948	6.0000	
FN1825					10		11.4405		AAL93924.1| Hypothetical protein
					7		10.2606		
FN1826	0.705	10.903	1.813e-4	1.911e-4	49	25	56.0583	32.5404	AAL93925.1| Protease
					38	36	55.7003	36.0000	
FN1827	-1.457	11.802	6.894e-4	1.175e-3	31	83	35.4655	108.0342	AAL93926.1| Replicative DNA helicase
					25	90	36.6450	90.0000	
FN1828	0.327	10.700	1.534e-2	5.792e-2	35	26	40.0417	33.8420	AAL93927.1| LSU ribosomal protein L9P
					35	39	51.3030	39.0000	
FN1830	-1.796	10.007	3.083e-3	7.944e-3	16	55	18.3048	71.5889	AAL93929.1| DNA polymerase III subunits gamma and tau
					11	48	16.1238	48.0000	
FN1831	-1.561	12.336	2.961e-4	3.795e-4	45	93	51.4821	121.0503	AAL93930.1| Nitrogen assimilation regulatory protein
					22	126	32.2476	126.0000	
FN1832	-0.771	5.159			4	6	4.5762	7.8097	AAL93931.1| TonB protein
FN1833					24		27.4571		AAL93932.1| Biopolymer transport exbD protein
					15		21.9870		
FN1834	0.636	9.903	4.62e-3	1.356e-2	29	22	33.1774	28.6356	AAL93933.1| Biopolymer transport exbB protein
					30	21	43.9740	21.0000	
FN1836	0.556	12.323	9.94e-4	1.922e-3	80	40	91.5238	52.0647	AAL93935.1| Tetratricopeptide repeat family protein
					56	66	82.0847	66.0000	

Log$_2$ Ratios: -6 -4 -2 0 2 4 6

Show detected proteins only
Show all proteins
Filter by category:
GO: amino acid transport

Proteins found: 1344

Enter (or paste) list of ORFs

Find ORFs

Test	Cutoff
q-Value	.005
p-Value	

Signif	Direction	Applies To
yes	+	ratios, bars
no	n/a	bars
yes	–	ratios, bars
yes	+	*p*- , *q*-Values
yes	–	

Dot Plots Dot Plots

Protein	*FnPgSg vs FnSg* Log$_2$ Ratio	Log$_2$ Sum	*q*-Value	*p*-Value	Raw *FnPgSg*	Raw *FnSg*	Normalized *FnPgSg*	Normalized *FnSg*	Description
FN1838					11		12.5845		AAL93937.1| Glycerol uptake facilitator protein
FN1839	-1.429	18.508	3.099e-3	7.996e-3	284	914	324.9095	1189.6775	AAL93938.1| Glycerol kinase
					286	814	419.2184	814.0000	
FN1840	0.451	12.937	7.111e-7	7.961e-8	90	58	102.9643	75.4938	AAL93939.1| Dihydroxyacetone kinase
					71	76	104.0717	76.0000	
FN1841	0.611	7.356	1.443e-2	5.393e-2	11	9	12.5845	11.7145	AAL93940.1| Dihydroxyacetone kinase
					13	9	19.0554	9.0000	
FN1842	0.941	10.664	1.201e-3	2.46e-3	54	17	61.7786	22.1275	AAL93941.1| Dihydroxyacetone kinase phosphotransfer protein
					34	36	49.8372	36.0000	
FN1844						8		10.4129	AAL93943.1| Ketoacyl reductase hetN
FN1847						16		20.8259	AAL93946.1| DTDP-4-dehydrorhamnose 3,5-epimerase
						23		23.0000	
FN1848									AAL93947.1| Metal dependent hydrolase
					4		5.8632		
FN1849	-2.851	7.239			4	20	4.5762	26.0323	AAL93948.1| Coenzyme F390 synthetase
						40		40.0000	
FN1850						25		32.5404	AAL93949.1| 3-oxoacyl-[acyl-carrier-protein] synthase III
						66		66.0000	
FN1851	-1.725	12.918	1.134e-4	1.016e-4	41	129	46.9059	167.9085	AAL93950.1| Ribonuclease PH
					34	152	49.8372	152.0000	
FN1852	0.863	10.305	1.141e-2	4.112e-2	39	29	44.6178	37.7469	AAL93951.1| unknown
					35	15	51.3030	15.0000	
FN1853	-0.862	6.274	6.623e-4	1.114e-3	5	9	5.7202	11.7145	AAL93952.1| Methylaspartate mutase
					5	12	7.3290	12.0000	
FN1854	-0.229	10.175	7.205e-2	3.355e-1	28	22	32.0333	28.6356	AAL93953.1| Methylaspartate mutase
					21	45	30.7818	45.0000	
FN1855						4		5.2065	AAL93954.1| Methylaspartate mutase
						7		7.0000	
FN1856	-0.314	23.555	3.291e-2	1.384e-1	2988	2497	3418.4135	3250.1366	AAL93955.1| Butyrate-acetoacetate CoA-transferase subunit B
					1963	4579	2877.3627	4579.0000	

Log$_2$ Ratios: -6 -4 -2 0 2 4 6

Show detected proteins only
Show all proteins
Filter by category:
GO: amino acid transport

Proteins found: 1344

Enter (or paste) list of ORFs

Find ORFs

Test	Cutoff
q-Value	.005
p-Value	

	Signif	Direction	Applies To
red	yes	+	ratios, bars
yellow	no	n/a	bars
green	yes	−	ratios, bars
light red	yes	+	*p*- , *q*-Values
light green	yes	−	*p*- , *q*-Values

Dot Plots Dot Plots

Protein	FnPgSg vs FnSg Log_2 Ratio	Log_2 Sum	*q*-Value	*p*-Value	Raw *FnPgSg*	Raw *FnSg*	Normalized *FnPgSg*	Normalized *FnSg*	Description
FN1857	-0.789	20.343	1.503e-4	1.488e-4	687	1208	785.9605	1572.3528	AAL93956.1| Acetoacetate: butyrate/acetate coenzyme A transferase
					661	1459	968.8929	1459.0000	
FN1858					92		105.2524		AAL93957.1| Short-chain fatty acids transporter
					95		139.2509		
FN1859	-3.063	24.001	3.561e-3	9.549e-3	1120	11444	1281.3330	14895.6990	AAL93958.1| Major outer membrane protein
					1060	8794	1553.7465	8794.0000	
FN1860	0.744	10.028	1.785e-2	6.91e-2	27	23	30.8893	29.9372	AAL93959.1| NA+/H+ antiporter NHAC
					36	20	52.7687	20.0000	
FN1862	1.252	14.940	9.526e-6	2.674e-6	249	82	284.8678	106.7326	AAL93961.1| L-beta-lysine 5,6-aminomutase beta subunit
					179	123	262.3780	123.0000	
FN1863	1.383	17.094	5.912e-4	9.482e-4	483	183	552.5749	238.1958	AAL93962.1| L-beta-lysine 5,6-aminomutase alpha subunit
					447	225	655.2120	225.0000	
FN1864	2.141	6.927	9.178e-5	7.761e-5	20	5	22.8809	6.5081	AAL93963.1| DNA mismatch repair protein mutS
					16	4	23.4528	4.0000	
FN1865					3		3.4321		AAL93964.1| Hypothetical protein
FN1866	0.850	20.632	3.986e-4	5.542e-4	1420	722	1624.5473	939.7672	AAL93965.1| Lysine 2,3-aminomutase
					1227	959	1798.5349	959.0000	
FN1867	1.737	18.853	2.003e-4	2.19e-4	1165	281	1332.8152	365.7543	AAL93966.1| Zn-dependent alcohol dehydrogenases and related dehydrogenases
					805	388	1179.9679	388.0000	
FN1868	1.333	13.850	3.369e-7	2.837e-8	168	60	192.2000	78.0970	AAL93967.1| Hypothetical cytosolic protein
					132	75	193.4854	75.0000	
FN1869	1.065	14.174	1.298e-4	1.215e-4	167	63	191.0559	82.0018	AAL93968.1| Hypothetical protein
					138	106	202.2802	106.0000	
FN1870									AAL93969.1| unknown
					3		4.3974		
FN1872									AAL93971.1| unknown
						5		5.0000	
FN1873	2.177	9.992	6.49e-4	1.085e-3	52	10	59.4905	13.0162	AAL93972.1| Bis(5'-nucleosyl)-tetraphosphatase
					52	17	76.2215	17.0000	
FN1874	0.372	9.103	7.796e-3	2.612e-2	21	14	24.0250	18.2226	AAL93973.1| Ribose 5-phosphate isomerase
					20	23	29.3160	23.0000	

Log_2 Ratios: -6 -4 -2 0 2 4 6

Show detected proteins only
Show all proteins
Filter by category:
GO: amino acid transport

Proteins found: 1344

Enter (or paste) list of ORFs

Find ORFs

Test	Cutoff
q-Value	.005
p-Value	

Signif	Direction	Applies To
yes	+	ratios, bars
no	n/a	bars
yes	–	ratios, bars
yes	+	*p*- , *q*-Values
yes	–	

Dot Plots Dot Plots

Protein	FnPgSg vs FnSg Log$_2$ Ratio	Log$_2$ Sum	*q*-Value	*p*-Value	Raw *FnPgSg*	Raw *FnSg*	Normalized *FnPgSg*	Normalized *FnSg*	Description
FN1875	-0.642	14.821	1.012e-3	1.968e-3	133	159	152.1583	206.9570	AAL93974.1| Peptidyl-prolyl cis-trans isomerase
					82	218	120.1955	218.0000	
FN1877					7		8.0083		AAL93976.1| Guanine-hypoxanthine permease
FN1878					5		5.7202		AAL93977.1| unknown
FN1879					25		28.6012		AAL93978.1| SSU ribosomal protein S20P
					23		33.7134		
FN1880	-0.985	12.674	2.301e-3	5.514e-3	62	101	70.9309	131.4633	AAL93979.1| Oxygen-insensitive NAD(P)H nitroreductase
					30	96	43.9740	96.0000	
FN1881	-0.944	6.502			6	8	6.8643	10.4129	AAL93980.1| Esterase
						16		16.0000	
FN1884					77		88.0916		AAL93983.1| unknown
					97		142.1825		
FN1890					16		18.3048		AAL93989.1| Hypothetical protein
					17		24.9186		
FN1891	-1.166	9.074	2.615e-4	3.219e-4	13	25	14.8726	32.5404	AAL93990.1| Glycerophosphoryl diester phosphodiesterase
					11	37	16.1238	37.0000	
FN1893	-0.156	17.514	5.534e-2	2.499e-1	309	336	353.5106	437.3432	AAL93991.1| Fusobacterium outer membrane protein family
					318	476	466.1240	476.0000	
FN1895					5		5.7202		AAL93994.1| Hypothetical protein
FN1898	-0.554	11.271	1.264e-2	4.628e-2	32	38	36.6095	49.4614	AAL93997.1| Sugar transport ATP-binding protein
					31	71	45.4398	71.0000	
FN1899	-1.058	15.333	1.374e-4	1.312e-4	109	230	124.7012	299.3718	AAL93998.1| Hypothetical lipoprotein
					107	287	156.8405	287.0000	
FN1901									AAL94000.1| Transcription regulator, CRP family
						8		8.0000	
FN1902	0.329	9.555	4.047e-2	1.755e-1	23	23	26.3131	29.9372	AAL94001.1| Deoxycytidylate deaminase
					24	19	35.1792	19.0000	
FN1903					126		144.1500		AAL94002.1| Coenzyme A disulfide reductase/ disulfide bond regulator domain
					105		153.9089		

Log$_2$ Ratios: -6 -4 -2 0 2 4 6

Show detected proteins only
Show all proteins
Filter by category:
GO: amino acid transport

Proteins found: 1344

Enter (or paste) list of ORFs

Find ORFs

Test	Cutoff
q-Value	.005
p-Value	

Color	Signif	Direction	Applies To
red	yes	+	ratios, bars
yellow	no	n/a	bars
green	yes	−	ratios, bars
light red	yes	+	*p*- , *q*-Values
light green	yes	−	

Dot Plots Dot Plots

Protein	*FnPgSg vs FnSg* Log$_2$ Ratio	Log$_2$ Sum	*q*-Value	*p*-Value	Raw *FnPgSg*	Raw *FnSg*	Normalized *FnPgSg*	Normalized *FnSg*	Description
FN1905	-0.221	3.779			3		3.4321		AAL94004.1| outer membrane protein
						4		4.0000	
FN1906	-0.905	17.654	2.89e-3	7.328e-3	320	408	366.0952	531.0596	AAL94005.1| Cytosol aminopeptidase
					203	712	297.5571	712.0000	
FN1908	-0.917	16.369	6.053e-4	9.818e-4	192	330	219.6571	429.5335	AAL94007.1| Glycerophosphoryl diester phosphodiesterase
					139	370	203.7460	370.0000	
FN1909	0.954	13.425	3.667e-4	4.989e-4	127	52	145.2940	67.6841	AAL94008.1| UDP-3-O-[3-hydroxymyristoyl] glucosamine N-acyltransferase
					100	83	146.5799	83.0000	
FN1910	2.400	15.833	1.251e-5	4.166e-6	454	107	519.3975	139.2730	AAL94009.1| periplasmic protein
					403	71	590.7168	71.0000	
FN1911	0.148	21.351	4.061e-2	1.762e-1	1561	1320	1785.8579	1718.1339	AAL94010.1| Outer membrane protein
					1131	1389	1657.8182	1389.0000	
FN1912	-0.979	6.082				7		9.1113	AAL94011.1| Hypothetical protein
					4	14	5.8632	14.0000	
FN1913	-0.270	11.814	8.888e-3	3.045e-2	43	49	49.1940	63.7792	AAL94012.1| hydrolase (HD superfamily)
					41	68	60.0977	68.0000	
FN1914	-0.335	12.597	1.324e-2	4.887e-2	70	69	80.0833	89.8115	AAL94013.1| Anti-sigma F factor antagonist
					41	87	60.0977	87.0000	
FN1917						10		13.0162	AAL94016.1| tRNA delta(2)-isopentenylpyrophosphate transferase
FN1918	-0.038	13.056	1.471e-1	7.463e-1	90	73	102.9643	95.0180	AAL94017.1| SPO0B-associated GTP-binding protein
					54	92	79.1531	92.0000	
FN1919	-0.124	6.296	1.559e-1	7.988e-1	11	5	12.5845	6.5081	AAL94018.1| Methyltransferase
					3	12	4.3974	12.0000	
FN1920									AAL94019.1| tRNA (5-methylaminomethyl -2-thiouridylate) -methyltransferase
						4		4.0000	
FN1922	-0.215	6.488				8		10.4129	AAL94021.1| Hypothetical protein
					6	10	8.7948	10.0000	
FN1923									AAL94022.1| Adenine-specific methyltransferase
						3		3.0000	
FN1926	-0.135	12.450	6.65e-2	3.065e-1	71	59	81.2274	76.7954	AAL94025.1| Nitrogen regulatory IIA protein
					42	80	61.5635	80.0000	

Log$_2$ Ratios: -6 -4 -2 0 2 4 6

Show detected proteins only
Show all proteins
Filter by category:
GO: amino acid transport

Proteins found: 1344

Enter (or paste) list of ORFs

Find ORFs

Test	Cutoff
q-Value	.005
p-Value	

	Signif	Direction	Applies To
red	yes	+	ratios, bars
yellow	no	n/a	bars
green	yes	−	ratios, bars
pink	yes	+	*p*- , *q*-Values
light green	yes	−	

Dot Plots Dot Plots

Protein	*FnPgSg vs FnSg* Log$_2$ Ratio	Log$_2$ Sum	*q*-Value	*p*-Value	Raw *FnPgSg*	Raw *FnSg*	Normalized *FnPgSg*	Normalized *FnSg*	Description	Log$_2$ Ratios (-6 -4 -2 0 2 4 6)
**FN1927**	-2.197	14.935	2.101e-4	2.342e-4	74	272	84.6595	354.0397	AAL94026.1| DEGV protein	
					55	404	80.6189	404.0000		
**FN1928**	-0.245	8.229	1.09e-1	5.294e-1	15	9	17.1607	11.7145	AAL94027.1| Transcriptional regulator, MerR family	
					10	26	14.6580	26.0000		
**FN1929**	-1.513	11.200	7.144e-3	2.342e-2	22	46	25.1690	59.8744	AAL94028.1| Competence-damage protein cinA	
					22	104	32.2476	104.0000		
**FN1931**									AAL94030.1| Protease	
						13		13.0000		
**FN1933**	0.517	7.330	5.416e-2	2.44e-1	15	4	17.1607	5.2065	AAL94032.1| Hypothetical protein	
					9	16	13.1922	16.0000		
**FN1935**	-1.375	4.934			3	6	3.4321	7.8097	AAL94034.1| Adenine-specific methyltransferase	
						10		10.0000		
**FN1939**	0.061	7.221	1.685e-1	8.766e-1	9	13	10.2964	16.9210	AAL94038.1| Hypothetical protein	
					10	7	14.6580	7.0000		
**FN1941**	-2.187	15.656	1.467e-3	3.14e-3	99	314	113.2607	408.7076	AAL94040.1| ClpB protein	
					68	561	99.6743	561.0000		
**FN1942**									AAL94041.1| putative DNA-binding protein	
						4		4.0000		
**FN1943**	-2.014	21.791	6.105e-5	4.534e-5	828	2836	947.2712	3691.3846	AAL94042.1| Tryptophanase	
					647	3965	948.3717	3965.0000		
**FN1948**						10		13.0162	AAL94044.1| Hypothetical protein	
						6		6.0000		
**FN1949**	-3.915	8.189				52		67.6841	AAL94045.1| Xaa-Pro dipeptidase	
					3	65	4.3974	65.0000		
**FN1950**									AAL94046.1| Serine protease	
					4		5.8632			
**FN1951**									AAL94047.1| ATPase associated with chromosome architecture/replication	
						4		4.0000		
**FN1956**						10		13.0162	AAL94052.1| Hypothetical protein	
						13		13.0000		
**FN1964**	0.320	7.906	7.999e-2	3.761e-1	20	9	22.8809	11.7145	AAL94054.1| O-linked GLCNAC transferase	
					8	16	11.7264	16.0000		

Show detected proteins only
Show all proteins
Filter by category:
GO: amino acid transport

Proteins found: 1344

Enter (or paste) list of ORFs

Find ORFs

Test	Cutoff
q-Value	.005
p-Value	

	Signif	Direction	Applies To
red	yes	+	ratios, bars
yellow	no	n/a	bars
green	yes	−	ratios, bars
pink	yes	+	*p*- , *q*-Values
light green	yes	−	

Dot Plots Dot Plots

Protein	FnPgSg vs FnSg Log$_2$ Ratio	Log$_2$ Sum	*q*-Value	*p*-Value	Raw *FnPgSg*	Raw *FnSg*	Normalized *FnPgSg*	Normalized *FnSg*	Description
FN1965	0.108	11.026	1.362e-1	6.822e-1	47	43	53.7702	55.9695	AAL94055.1| Tetratricopeptide repeat family protein
					28	32	41.0424	32.0000	
FN1966	-0.366	12.227	8.625e-3	2.939e-2	49	67	56.0583	87.2083	AAL94056.1| Hypothetical protein
					45	70	65.9609	70.0000	
FN1970					14		16.0167		AAL94060.1| Hemin-binding periplasmic protein hmuT precursor
FN1971	1.677	7.587	5.685e-4	8.956e-4	19	5	21.7369	6.5081	AAL94061.1| Hemin receptor
					19	9	27.8502	9.0000	
FN1972	-1.209	9.164	2.551e-2	1.038e-1	16	16	18.3048	20.8259	AAL94062.1| unknown
					9	52	13.1922	52.0000	
FN1973	1.685	13.567	1.902e-5	8.481e-6	166	56	189.9119	72.8905	AAL94063.1| Translation initiation inhibitor
					140	50	205.2118	50.0000	
FN1974									AAL94064.1| DNA/RNA helicase (DEAD/DEAH BOX family)
						7		7.0000	
FN1975	0.081	13.898	1.217e-1	5.996e-1	103	107	117.8369	139.2730	AAL94065.1| ATP-dependent RNA helicase
					93	101	136.3193	101.0000	
FN1976	-1.613	10.618	9.677e-4	1.855e-3	14	62	16.0167	80.7002	AAL94066.1| 4-amino-4-deoxychorismate lyase
					20	58	29.3160	58.0000	
FN1977	-1.092	5.480			4	5	4.5762	6.5081	AAL94067.1| Cell cycle protein MesJ
						13		13.0000	
FN1978	0.759	13.903	4.993e-4	7.463e-4	129	64	147.5821	83.3035	AAL94068.1| Cell division protein ftsH
					119	107	174.4300	107.0000	
FN1979					204		233.3857		AAL94069.1| SSU ribosomal protein S15P
					210		307.8177		
FN1980	-0.244	4.517				4		5.2065	AAL94070.1| Transporter
					3		4.3974		
FN1983	1.642	17.302	4.642e-4	6.762e-4	564	186	645.2427	242.1007	AAL94073.1| Alkyl hydroperoxide reductase C22 protein
					529	213	775.4075	213.0000	
FN1984	1.105	16.684	5.058e-4	7.595e-4	376	154	430.1618	200.4490	AAL94074.1| Thioredoxin reductase
					356	242	521.8243	242.0000	
FN1985	-0.469	12.492	1.799e-3	4.055e-3	50	65	57.2024	84.6051	AAL94075.1| Inner membrane protein
					49	94	71.8241	94.0000	

Log$_2$ Ratios: -6 -4 -2 0 2 4 6

Show detected proteins only
Show all proteins
Filter by category:
GO: amino acid transport

Proteins found: 1344

Enter (or paste) list of ORFs

Find ORFs

Test	Cutoff
q-Value	.005
p-Value	

Color	Signif	Direction	Applies To
red	yes	+	ratios, bars
yellow	no	n/a	bars
green	yes	–	ratios, bars
light red	yes	+	*p*- , *q*-Values
light green	yes	–	

Dot Plots Dot Plots

Protein	*FnPgSg* vs *FnSg* Log$_2$ Ratio	Log$_2$ Sum	*q*-Value	*p*-Value	Raw *FnPgSg*	Raw *FnSg*	Normalized *FnPgSg*	Normalized *FnSg*	Description
**FN1986**	0.089	15.805	1.387e-1	6.968e-1	234	222	267.7071	288.9589	AAL94076.1| Hypothetical protein
					154	175	225.7330	175.0000	
**FN1987**	-0.246	4.924			5		5.7202		AAL94077.1| Transcriptional regulator, GntR family
					3	6	4.3974	6.0000	
**FN1988**	-3.718	17.333	2.444e-3	5.965e-3	92	1391	105.2524	1810.5486	AAL94078.1| Tyrosine phenol-lyase
					81	1137	118.7297	1137.0000	
**FN1989**						12		15.6194	AAL94079.1| Sodium-dependent tyrosine transporter
						8		8.0000	
**FN1991**	0.158	17.211	2.173e-2	8.692e-2	349	260	399.2725	338.4203	AAL94081.1| Glucosamine-1-phosphate acetyltransferase
					289	399	423.6158	399.0000	
**FN1992**	-0.918	16.269	3.882e-3	1.07e-2	173	340	197.9202	442.5496	AAL94082.1| Ribose-phosphate pyrophosphokinase
					144	330	211.0750	330.0000	
**FN1993**	0.427	5.321				3		3.9048	AAL94083.1| SUA5 protein
					5	7	7.3290	7.0000	
**FN1994**	0.911	10.173	3.862e-2	1.663e-1	43	35	49.1940	45.5566	AAL94084.1| Hypothetical protein
					30	4	43.9740	4.0000	
**FN1995**					9		10.2964		AAL94085.1| Hypothetical protein
					19		27.8502		
**FN2001**						18		23.4291	AAL94091.1| Hypothetical protein
						19		19.0000	
**FN2002**									AAL94092.1| Permease
						3		3.0000	
**FN2007**	0.028	5.454	1.798e-1	9.482e-1	4	7	4.5762	9.1113	AAL94097.1| Glutathione peroxidase
					6	4	8.7948	4.0000	
**FN2008**						4		5.2065	AAL94098.1| Glycine betaine transport ATP-binding protein
**FN2009**						6		7.8097	AAL94099.1| Glycine betaine transport system permease protein
						8		8.0000	
**FN2011**	-0.192	13.809	1.261e-1	6.243e-1	87	60	99.5321	78.0970	AAL94101.1| Valyl-tRNA synthetase
					85	178	124.5929	178.0000	
**FN2013**						5		6.5081	AAL94103.1| GTP-binding protein
						6		6.0000	

Log$_2$ Ratios: -6 -4 -2 0 2 4 6

Show detected proteins only
Show all proteins
Filter by category:
GO: amino acid transport

Proteins found: 1344

Enter (or paste) list of ORFs

Find ORFs

Test	Cutoff
q-Value	.005
p-Value	

	Signif	Direction	Applies To
red	yes	+	ratios, bars
yellow	no	n/a	bars
green	yes	−	ratios, bars
pink	yes	+	*p*- , *q*-Values
light green	yes	−	

Dot Plots Dot Plots

Protein	FnPgSg vs FnSg Log$_2$ Ratio	Log$_2$ Sum	*q*-Value	*p*-Value	Raw *FnPgSg*	Raw *FnSg*	Normalized *FnPgSg*	Normalized *FnSg*	Description
FN2014	0.253	14.801	5.745e-3	1.793e-2	152	128	173.8952	166.6069	AAL94104.1| ATP-dependent protease La
					133	143	194.9512	143.0000	
FN2015	-0.296	13.399	1.369e-2	5.077e-2	73	97	83.5155	126.2568	AAL94105.1| ATP-dependent clp protease ATP-binding subunit clpX
					71	104	104.0717	104.0000	
FN2016	1.466	9.513	2.541e-4	3.084e-4	35	15	40.0417	19.5242	AAL94106.1| ATP-dependent Clp protease proteolytic subunit
					34	13	49.8372	13.0000	
FN2017	1.230	18.442	8.06e-4	1.455e-3	711	273	813.4177	355.3413	AAL94107.1| Trigger factor, ppiase
					692	424	1014.3326	424.0000	
FN2018	1.088	8.775	2.493e-4	2.997e-4	29	9	33.1774	11.7145	AAL94108.1| Single-stranded-DNA-specific exonuclease recJ
					19	17	27.8502	17.0000	
FN2019	2.387	7.557			19		21.7369		AAL94109.1| Ribosome-binding factor A
					28	6	41.0424	6.0000	
FN2020	-0.213	16.400	6.688e-2	3.084e-1	243	196	278.0035	255.1168	AAL94110.1| Bacterial Protein Translation Initiation Factor 2 (IF-2)
					183	378	268.2411	378.0000	
FN2022	-0.172	12.823	3.686e-3	9.988e-3	71	72	81.2274	93.7164	AAL94112.1| N utilization substance protein A
					54	87	79.1531	87.0000	
FN2023	-0.570	7.739	4.309e-3	1.233e-2	12	12	13.7286	15.6194	AAL94113.1| Hypothetical cytosolic protein
					7	20	10.2606	20.0000	
FN2030	1.854	13.135	1.219e-4	1.117e-4	150	39	171.6071	50.7630	AAL94115.1| Inorganic pyrophosphatase
					129	49	189.0880	49.0000	
FN2031	1.801	5.801			9		10.2964		AAL94116.1| Thiamine biosynthesis lipoprotein apbE
					12	4	17.5896	4.0000	
FN2033									AAL94118.1| Guanylate kinase
						13		13.0000	
FN2034	1.273	9.117	4.011e-5	2.53e-5	32	11	36.6095	14.3178	AAL94119.1| Protein yicC
					25	16	36.6450	16.0000	
FN2035	-0.121	18.040	4.997e-2	2.227e-1	418	453	478.2118	589.6323	AAL94120.1| DNA-directed RNA polymerase beta' chain
					353	493	517.4269	493.0000	
FN2036	-0.151	17.382	1.088e-2	3.88e-2	327	318	374.1035	413.9141	AAL94121.1| DNA-directed RNA polymerase beta chain
					280	457	410.4236	457.0000	
FN2037	3.228	21.788	1.093e-6	1.38e-7	4959	315	5673.3309	410.0092	AAL94122.1| LSU ribosomal protein L12P (L7/L12)
					4077	833	5976.0609	833.0000	

Log$_2$ Ratios: -6 -4 -2 0 2 4 6

Show detected proteins only
Show all proteins
Filter by category:
GO: amino acid transport

Proteins found: 1344

Enter (or paste) list of ORFs

Find ORFs

Test	Cutoff
q-Value	.005
p-Value	

	Signif	Direction	Applies To
red	yes	+	ratios, bars
yellow	no	n/a	bars
green	yes	−	ratios, bars
pink	yes	+	*p*- , *q*-Values
light green	yes	−	

Dot Plots Dot Plots

Protein	*FnPgSg vs FnSg* Log$_2$ Ratio	Log$_2$ Sum	*q*-Value	*p*-Value	Raw *FnPgSg*	Raw *FnSg*	Normalized *FnPgSg*	Normalized *FnSg*	Description
**FN2038**	1.684	15.881	2.981e-9	3.763e-11	383	103	438.1701	134.0665	AAL94123.1| LSU ribosomal protein L10P
					302	140	442.6712	140.0000	
**FN2039**	0.361	16.701	1.01e-2	3.546e-2	316	252	361.5190	328.0074	AAL94124.1| LSU ribosomal protein L1P
					258	248	378.1760	248.0000	
**FN2040**	3.504	12.270	1.581e-4	1.596e-4	223	19	255.1226	24.7307	AAL94125.1| LSU ribosomal protein L11P
					149	17	218.4040	17.0000	
**FN2041**	0.534	9.920	3.5e-3	9.337e-3	36	19	41.1857	24.7307	AAL94126.1| Transcription antitermination protein nusG
					23	27	33.7134	27.0000	
**FN2045**						7		9.1113	AAL94129.1| Ferric uptake regulation protein
						17		17.0000	
**FN2046**						16		20.8259	AAL94130.1| Acetyltransferase
						14		14.0000	
**FN2047**	-0.075	13.911	1.179e-1	5.781e-1	91	92	104.1083	119.7487	AAL94131.1| Fusobacterium outer membrane protein family
					94	135	137.7851	135.0000	
**FN2048**	-0.305	16.664	8.723e-2	4.134e-1	234	180	267.7071	234.2910	AAL94132.1| Outer membrane protein
					213	482	312.2151	482.0000	
**FN2049**	-0.913	17.211	3.001e-5	1.622e-5	231	430	264.2749	559.6951	AAL94133.1| unknown
					207	509	303.4203	509.0000	
**FN2050**	2.392	13.594	2.816e-3	7.096e-3	175	40	200.2083	52.0647	AAL94134.1| Hypothetical membrane-spanning protein
					211	45	309.2835	45.0000	
**FN2051**	1.058	13.619	4.575e-3	1.338e-2	142	81	162.4547	105.4309	AAL94135.1| unknown
					110	50	161.2378	50.0000	
**FN2052**	1.004	13.451	5.799e-3	1.815e-2	121	81	138.4297	105.4309	AAL94136.1| unknown
					110	44	161.2378	44.0000	
**FN2053**	1.233	12.764	6.316e-4	1.045e-3	98	49	112.1166	63.7792	AAL94137.1| Serine/threonine sodium symporter
					98	45	143.6483	45.0000	
**FN2054**	1.112	14.803	1.403e-5	5.063e-6	227	96	259.6988	124.9552	AAL94138.1| Glucose-6-phosphate isomerase
					162	105	237.4594	105.0000	
**FN2058**	0.653	16.059	5.724e-4	9.045e-4	269	155	307.7487	201.7506	AAL94142.1| Fusobacterium outer membrane protein family
					237	215	347.3943	215.0000	
**FN2059**	-0.305	16.664	8.723e-2	4.134e-1	234	180	267.7071	234.2910	AAL94143.1| Outer membrane protein
					213	482	312.2151	482.0000	

Log$_2$ Ratios: -6 -4 -2 0 2 4 6

Show detected proteins only
Show all proteins
Filter by category:
GO: amino acid transport

Proteins found: 1344

Enter (or paste) list of ORFs

Find ORFs

Test	Cutoff
q-Value	.005
p-Value	

	Signif	Direction	Applies To
red	yes	+	ratios, bars
yellow	no	n/a	bars
green	yes	−	ratios, bars
pink	yes	+	*p*- , *q*-Values
light green	yes	−	

Dot Plots Dot Plots

Protein	*FnPgSg* vs *FnSg* Log$_2$ Ratio	Log$_2$ Sum	*q*-Value	*p*-Value	Raw *FnPgSg*	Raw *FnSg*	Normalized *FnPgSg*	Normalized *FnSg*	Description
FN2060	-0.913	17.211	3.001e-5	1.622e-5	231	430	264.2749	559.6951	AAL94144.1| unknown
					207	509	303.4203	509.0000	
FN2061	2.392	13.594	2.816e-3	7.096e-3	175	40	200.2083	52.0647	AAL94145.1| Hypothetical membrane-spanning protein
					211	45	309.2835	45.0000	
FN2062	1.058	13.619	4.575e-3	1.338e-2	142	81	162.4547	105.4309	AAL94146.1| unknown
					110	50	161.2378	50.0000	
FN2063	1.004	13.451	5.799e-3	1.815e-2	121	81	138.4297	105.4309	AAL94147.1| unknown
					110	44	161.2378	44.0000	
FN2067	1.346	6.516			10		11.4405		AAL94151.1| Thiol:disulfide interchange protein tlpA
					13	6	19.0554	6.0000	
FN2068	-2.132	6.520			4	7	4.5762	9.1113	AAL94152.1| dGTP triphosphohydrolase
						31		31.0000	
FN2070	-1.507	5.895			4	10	4.5762	13.0162	AAL94154.1| Cobyric acid synthase
						13		13.0000	
FN2073	-2.586	9.733	2.449e-3	5.982e-3	8	43	9.1524	55.9695	AAL94157.1| Adenine phosphoribosyltransferase
					10	87	14.6580	87.0000	
FN2074									AAL94158.1| BslIM
					4		5.8632		
FN2075	0.841	9.292	1.359e-2	5.034e-2	24	8	27.4571	10.4129	AAL94159.1| Hypothetical protein
					27	27	39.5766	27.0000	
FN2076									AAL94160.1| MunI regulatory protein
					3		4.3974		
FN2078									AAL94162.1| Transcriptional regulator, DeoR family
						8		8.0000	
FN2082	-0.188	21.158	8.588e-2	4.064e-1	1557	1156	1781.2817	1504.6687	AAL94166.1| Formate--tetrahydrofolate ligase
					740	1761	1084.6910	1761.0000	
FN2093	1.127	8.734	7.759e-3	2.596e-2	20	13	22.8809	16.9210	AAL94177.1| General secretion pathway protein G
					26	11	38.1108	11.0000	
FN2098	-1.688	5.246			3	7	3.4321	9.1113	AAL94182.1| MRP-family nucleotide-binding protein
						13		13.0000	
FN2100	0.775	10.407	3.135e-5	1.727e-5	42	21	48.0500	27.3339	AAL94184.1| Hypothetical protein
					33	29	48.3714	29.0000	

Log$_2$ Ratios: -6 -4 -2 0 2 4 6

Show detected proteins only
Show all proteins
Filter by category:
GO: amino acid transport

Proteins found: 1344

Enter (or paste) list of ORFs

Find ORFs

Test	Cutoff
q-Value	.005
p-Value	

	Signif	Direction	Applies To
red	yes	+	ratios, bars
yellow	no	n/a	bars
green	yes	−	ratios, bars
pink	yes	+	*p*- , *q*-Values
light green	yes	−	*p*- , *q*-Values

Dot Plots Dot Plots

Protein	*FnPgSg* vs *FnSg* Log$_2$ Ratio	Log$_2$ Sum	*q*-Value	*p*-Value	Raw *FnPgSg*	Raw *FnSg*	Normalized *FnPgSg*	Normalized *FnSg*	Description
**FN2102**	-1.324	8.022	3.408e-3	9.02e-3	5	20	5.7202	26.0323	AAL94186.1| ABC transporter ATP-binding protein
					10	25	14.6580	25.0000	
**FN2103**	0.136	19.347	3.788e-2	1.626e-1	788	651	901.5093	847.3524	AAL94187.1| tricarboxylate-binding protein
					553	711	810.5866	711.0000	
**FN2105**					12		13.7286		AAL94189.1| tricarboxylate transport membrane protein RctA
					9		13.1922		
**FN2106**	0.194	13.947	7.47e-3	2.475e-2	112	96	128.1333	124.9552	AAL94190.1| Transporter
					96	110	140.7167	110.0000	
**FN2107**	2.332	9.622	2.264e-6	3.328e-7	55	10	62.9226	13.0162	AAL94191.1| Galactokinase
					43	12	63.0293	12.0000	
**FN2108**	0.954	9.436	3.829e-4	5.266e-4	32	16	36.6095	20.8259	AAL94192.1| Galactose-1-phosphate uridylyltransferase
					25	17	36.6450	17.0000	
**FN2109**	0.359	11.889	8.152e-4	1.479e-3	63	39	72.0750	50.7630	AAL94193.1| UDP-glucose 4-epimerase
					46	58	67.4267	58.0000	
**FN2116**	-0.874	6.286	3.594e-2	1.531e-1	5	13	5.7202	16.9210	AAL94200.1| Hypothetical exported 24-amino acid repeat protein
					5	7	7.3290	7.0000	
**FN2117**									AAL94201.1| Hypothetical exported 24-amino acid repeat protein
						9		9.0000	
**FN2118**	1.759	6.929			15		17.1607		AAL94202.1| Hypothetical exported 24-amino acid repeat protein
					16	6	23.4528	6.0000	
**FN2119**	0.173	8.514	1.09e-1	5.294e-1	15	10	17.1607	13.0162	AAL94203.1| Hypothetical exported 24-amino acid repeat protein
					16	23	23.4528	23.0000	
**FN2121**	2.905	10.309			91	10	104.1083	13.0162	AAL94205.1| Hypothetical exported 24-amino acid repeat protein
					62		90.8795		
**FN2122**	-0.132	16.770	1.905e-2	7.448e-2	297	275	339.7821	357.9446	AAL94206.1| Phenylalanyl-tRNA synthetase beta chain
					204	342	299.0229	342.0000	
**FN2123**	-0.766	12.973	1.906e-2	7.453e-2	60	66	68.6428	85.9067	AAL94207.1| Phenylalanyl-tRNA synthetase alpha chain
					47	148	68.8925	148.0000	
**FN2125**	-0.204	12.238	7.585e-3	2.523e-2	53	57	60.6345	74.1921	AAL94209.1| DNA gyrase subunit A
					47	75	68.8925	75.0000	
**FN2126**	1.802	11.286	7.438e-5	5.847e-5	85	15	97.2440	19.5242	AAL94210.1| DNA gyrase subunit B
					61	34	89.4137	34.0000	

Log$_2$ Ratios: -6 -4 -2 0 2 4 6

Show detected proteins only
Show all proteins
Filter by category:
GO: amino acid transport

Proteins found: 1344

Enter (or paste) list of ORFs
Find ORFs

Test	Cutoff
q-Value	.005
p-Value	

	Signif	Direction	Applies To
(red)	yes	+	ratios, bars
(yellow)	no	n/a	bars
(green)	yes	−	ratios, bars
(pink)	yes	+	*p*- , *q*-Values
(light green)	yes	−	

Dot Plots Dot Plots
