# Supplementary material for: Proteomics of Fusobacterium nucleatum within a model developing oral microbial community
Source: Microbiologyopen. 2014 Aug 25;3(5):729–51. doi: 10.1002/mbo3.204 (PMC4234264; doi:10.1002/mbo3.204)
Supplement: Table S7 — See description for Table S3. [file mbo30003-0729-sd9.pdf]

FnSg vs Fn

Fusobacterium nucleatum

Hackett Laboratory UW

Page 1

Fn Summary Table | FnPg vs Fn | FnSg vs Fn | FnPgSg vs Fn | FnPgSg vs FnPg | FnSg vs FnPg | FnPgSg vs FnSg | Fn Coverage

Protein	*FnSg* vs *Fn* Log$_2$ Ratio	Log$_2$ Sum	*q*-Value	*p*-Value	Raw *FnSg*	Raw *Fn*	Normalized *FnSg*	Normalized *Fn*	Description
**FN0001**	-0.721	8.536	3.344e-3	1.286e-2	10	22	13.0162	28.4878	AAL94214.1| Chromosomal replication initiator protein dnaA
					17	15	17.0000	20.9761	
**FN0004**	-1.041	11.595	1.344e-4	2.314e-4	35	66	45.5566	85.4634	AAL94217.1| Inner membrane protein
					32	53	32.0000	74.1157	
**FN0005**	-1.546	10.372	4.85e-4	1.179e-3	12	41	15.6194	53.0909	AAL94218.1| Jag protein
					27	51	27.0000	71.3189	
**FN0006**	0.271	9.476	6.231e-3	2.671e-2	22	17	28.6356	22.0133	AAL94219.1| Thiophene and furan oxidation protein THDF
					30	19	30.0000	26.5698	
**FN0007**	-0.993	8.847	3.101e-3	1.17e-2	8	23	10.4129	29.7827	AAL94220.1| Glucose inhibited division protein A
					20	22	20.0000	30.7650	
**FN0008**					6		7.8097		AAL94221.1| Quinolinate synthetase A
					11		11.0000		
**FN0009**									AAL94222.1| L-aspartate oxidase
					25		25.0000		
**FN0017**	-0.525	5.744	3.621e-3	1.424e-2	4	6	5.2065	7.7694	AAL94230.1| Hypothetical protein
					7	7	7.0000	9.7889	
**FN0018**	-1.340	10.536	7.478e-5	1.095e-4	18	45	23.4291	58.2705	AAL94231.1| Hypothetical protein
					25	46	25.0000	64.3268	
**FN0019**	0.493	4.631			6		7.8097		AAL94232.1| Transcription-repair coupling factor
					4	3	4.0000	4.1952	
**FN0021**									AAL94234.1| 4-diphosphocytidyl-2-C-methyl-D-erythritol kinase
						5		6.9920	
**FN0022**	-2.619	9.954	5.088e-7	5.976e-8	8	59	10.4129	76.3991	AAL94235.1| Hypothetical protein
					15	57	15.0000	79.7094	
**FN0023**									AAL94236.1| Short-chain fatty acids transporter
					15		15.0000		
**FN0024**	-2.296	10.636				75		97.1175	AAL94237.1| Hypothetical exported 24-amino acid repeat protein
					18	57	18.0000	79.7094	
**FN0025**	-2.514	8.514				36		46.6164	AAL94238.1| Hypothetical exported 24-amino acid repeat protein
					8	32	8.0000	44.7491	
**FN0026**						9		11.6541	AAL94239.1| Hypothetical exported 24-amino acid repeat protein
						21		29.3666	

Log$_2$ Ratios: -6 -4 -2 0 2 4 6

- Show detected proteins only
- Show all proteins
- Filter by category: GO: amino acid transport

Proteins found: 1358

Enter (or paste) list of ORFs

Find ORFs

Test	Cutoff
q-Value	.005
p-Value	

	Signif	Direction	Applies To
(red)	yes	+	ratios, bars
(yellow)	no	n/a	bars
(green)	yes	–	ratios, bars
(pink)	yes	+	*p*-, *q*-Values
(light green)	yes	–	

Dot Plots | Dot Plots

Protein	*FnSg* vs *Fn* Log$_2$ Ratio	Log$_2$ Sum	*q*-Value	*p*-Value	Raw *FnSg*	Raw *Fn*	Normalized *FnSg*	Normalized *Fn*	Description
FN0029						7		9.0643	AAL94242.1| Flavodoxin
						9		12.5857	
FN0030	0.770	12.755	3.016e-4	6.467e-4	90	53	117.1455	68.6297	AAL94243.1| 5-nitroimidazole antibiotic resistance protein
					100	42	100.0000	58.7332	
FN0031	1.640	7.650	3.089e-6	9.391e-7	20	7	26.0323	9.0643	AAL94244.1| unknown
					24	5	24.0000	6.9920	
FN0033	-0.389	12.980	1.059e-7	6.028e-9	60	79	78.0970	102.2971	AAL94246.1| unknown
					79	74	79.0000	103.4823	
FN0034	-0.699	9.136	2.259e-5	1.945e-5	14	24	18.2226	31.0776	AAL94247.1| unknown
					19	21	19.0000	29.3666	
FN0039	0.521	7.561	7.269e-4	2.007e-3	13	8	16.9210	10.3592	AAL94252.1| DNA primase (bacterial type) and small primase-like proteins
					16	9	16.0000	12.5857	
FN0040	0.442	18.592	1.777e-5	1.378e-5	549	402	714.5875	520.5499	AAL94253.1| Asparaginyl-tRNA synthetase
					751	399	751.0000	557.9655	
FN0041						8		10.3592	AAL94254.1| unknown
						8		11.1873	
FN0043						15		19.4235	AAL94256.1| Hypothetical exported 24-amino acid repeat protein
						9		12.5857	
FN0045	-0.622	6.237				8		10.3592	AAL94258.1| Shikimate 5-dehydrogenase
					7	8	7.0000	11.1873	
FN0046						5		6.4745	AAL94259.1| 3-dehydroquinate dehydratase
						3		4.1952	
FN0047	-0.690	12.656	1.944e-4	3.696e-4	48	82	62.4776	106.1818	AAL94260.1| Exodeoxyribonuclease III
					64	70	64.0000	97.8887	
FN0048	-0.904	10.176	7.755e-4	2.196e-3	19	33	24.7307	42.7317	AAL94261.1| 4-nitrophenylphosphatase
					25	36	25.0000	50.3427	
FN0049	3.827	7.964			40		52.0647		AAL94262.1| Hypothetical protein
					67	3	67.0000	4.1952	
FN0050	-2.906	18.386	8.682e-5	1.311e-4	151	1157	196.5441	1498.1996	AAL94263.1| Fumarate reductase flavoprotein subunit
					231	1221	231.0000	1707.4583	
FN0052						44		56.9756	AAL94265.1| Arsenate reductase
						37		51.7412	

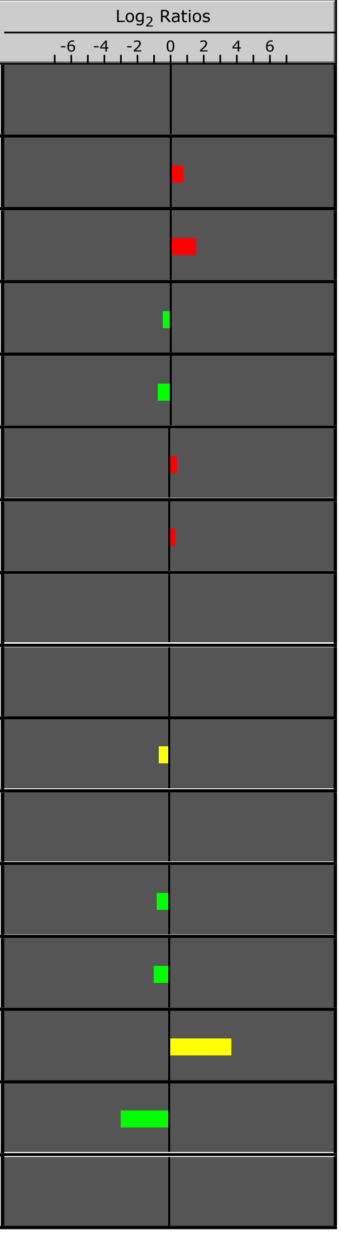

Show detected proteins only
Show all proteins
Filter by category:
GO: amino acid transport

Proteins found: 1358

Enter (or paste) list of ORFs

Find ORFs

Test	Cutoff
q-Value	.005
p-Value	

	Signif	Direction	Applies To
red	yes	+	ratios, bars
yellow	no	n/a	bars
green	yes	−	ratios, bars
light red	yes	+	*p*- , *q*-Values
light green	yes	−	

Dot Plots Dot Plots

FnSg vs Fn

Fusobacterium nucleatum

Hackett Laboratory | UW

Fn Summary Table | FnPg vs Fn | FnSg vs Fn | FnPgSg vs Fn | FnPgSg vs FnPg | FnSg vs FnPg | FnPgSg vs FnSg | Fn Coverage

Protein	FnSg vs Fn Log$_2$ Ratio	Log$_2$ Sum	*q*-Value	*p*-Value	Raw *FnSg*	Raw *Fn*	Normalized *FnSg*	Normalized *Fn*	Description
FN0054	-0.304	14.970	6.079e-6	2.647e-6	124	153	161.4005	198.1197	AAL94267.1| Tyrosyl-tRNA synthetase
					161	143	161.0000	199.9726	
FN0058	0.314	15.791	8.433e-2	4.894e-1	116	171	150.9875	221.4279	AAL94271.1| Cysteine desulfhydrase
					380	147	380.0000	205.5662	
FN0059	-0.273	11.390	6.53e-2	3.713e-1	24	48	31.2388	62.1552	AAL94272.1| NifU protein
					63	37	63.0000	51.7412	
FN0060	-2.452	8.067				30		38.8470	AAL94273.1| D-alanyl-D-alanine carboxypeptidase
					7	27	7.0000	37.7571	
FN0061	0.005	12.882	1.523e-1	9.547e-1	60	67	78.0970	86.7583	AAL94274.1| Thermostable carboxypeptidase 1
					96	62	96.0000	86.7014	
FN0062						7		9.0643	AAL94275.1| Hypothetical cytosolic protein
						11		15.3825	
FN0063									AAL94276.1| unknown
					24		24.0000		
FN0065	-0.894	14.252	2.279e-4	4.492e-4	66	157	85.9067	203.2993	AAL94278.1| Transcription accessory protein (S1 RNA binding domain)
					119	127	119.0000	177.5980	
FN0066									AAL94279.1| Two component system histidine kinase
					5		5.0000		
FN0067	-1.276	17.068	1.701e-5	1.293e-5	180	434	234.2910	561.9867	AAL94280.1| Isoleucyl-tRNA synthetase
					242	423	242.0000	591.5273	
FN0069	-0.019	15.094	1.435e-1	8.903e-1	161	159	209.5603	205.8891	AAL94282.1| Glycyl-tRNA synthetase alpha chain
					162	122	162.0000	170.6060	
FN0070	0.067	18.090	2.792e-2	1.478e-1	432	390	562.2984	505.0111	AAL94283.1| Glycyl-tRNA synthetase beta chain
					519	377	519.0000	527.2005	
FN0071	-0.296	6.671			7		9.1113		AAL94284.1| GTP cyclohydrolase I
						8		11.1873	
FN0072	0.620	13.588	2.807e-4	5.872e-4	100	67	130.1617	86.7583	AAL94285.1| 2-amino-4-hydroxy-6-hydroxymethyldihydropteridine pyrophosphokinase
					145	66	145.0000	92.2950	
FN0073	-0.528	10.367	2.285e-2	1.186e-1	15	35	19.5242	45.3215	AAL94286.1| Dihydropteroate synthase
					41	30	41.0000	41.9523	
FN0074	-0.234	8.494	9.103e-2	5.322e-1	10	21	13.0162	27.1929	AAL94287.1| Ethanolamine utilization protein eutS
					22	10	22.0000	13.9841	

Log$_2$ Ratios: -6 -4 -2 0 2 4 6

Show detected proteins only
Show all proteins
Filter by category:
GO: amino acid transport

Proteins found: 1358

Enter (or paste) list of ORFs

Find ORFs

Test	Cutoff
q-Value	.005
p-Value	

	Signif	Direction	Applies To
red	yes	+	ratios, bars
yellow	no	n/a	bars
green	yes	−	ratios, bars
light red	yes	+	*p*- , *q*-Values
light green	yes	−	

Dot Plots Dot Plots

Fusobacterium nucleatum

Protein	*FnSg* vs *Fn* Log$_2$ Ratio	Log$_2$ Sum	*q*-Value	*p*-Value	Raw *FnSg*	Raw *Fn*	Normalized *FnSg*	Normalized *Fn*	Description
FN0077	0.632	6.769							AAL94290.1| Ethanolamine two-component sensor kinase
					13	6	13.0000	8.3905	
FN0078	1.437	7.574			18		23.4291		AAL94291.1| Ethanolamine utilization protein eutA
					22	6	22.0000	8.3905	
FN0079	1.007	14.387	4.635e-5	5.506e-5	169	86	219.9732	111.3614	AAL94292.1| Ethanolamine ammonia-lyase heavy chain
					195	68	195.0000	95.0919	
FN0080	0.180	14.085	3.374e-2	1.823e-1	118	106	153.5908	137.2594	AAL94293.1| Ethanolamine ammonia-lyase light chain
					127	79	127.0000	110.4744	
FN0081	1.350	14.856	2.767e-3	1.014e-2	172	78	223.8780	101.0022	AAL94294.1| Ethanolamine utilization protein eutL
					326	82	326.0000	114.6696	
FN0082	1.343	9.229			20		26.0323		AAL94295.1| Ethanolamine utilization protein eutM
					52	11	52.0000	15.3825	
FN0083	0.714	15.208	2.881e-3	1.066e-2	164	132	213.4651	170.9268	AAL94296.1| Ethanolamine utilization protein eutM precursor
					285	95	285.0000	132.8489	
FN0084	-0.211	10.727	3.136e-2	1.681e-1	25	36	32.5404	46.6164	AAL94297.1| Acetaldehyde dehydrogenase (acetylating)
					44	30	44.0000	41.9523	
FN0087									AAL94300.1| Ethanolamine utilization protein eutN
					5		5.0000		
FN0088	0.686	5.654							AAL94301.1| Hypothetical protein
					9	4	9.0000	5.5936	
FN0090					7		9.1113		AAL94303.1| Ethanolamine utilization protein eutQ
					4		4.0000		
FN0092	0.830	11.203	1.614e-5	1.197e-5	48	26	62.4776	33.6674	AAL94305.1| NADPH-dependent butanol dehydrogenase
					67	28	67.0000	39.1555	
FN0093	-1.914	15.202	4.362e-4	1.028e-3	83	323	108.0342	418.2528	AAL94306.1| Thioredoxin
					92	240	92.0000	335.6183	
FN0100	-1.531	9.011	2.386e-3	8.457e-3	9	24	11.7145	31.0776	AAL94309.1| Flavodoxins/hemoproteins
					15	33	15.0000	46.1475	
FN0102						12		15.5388	AAL94311.1| Ribonucleoside-diphosphate reductase alpha chain
						11		15.3825	
FN0103						7		9.0643	AAL94312.1| Ribonucleoside-diphosphate reductase beta chain
						8		11.1873	

Log$_2$ Ratios: -6 -4 -2 0 2 4 6

- Show detected proteins only
- Show all proteins
- Filter by category: GO: amino acid transport

Proteins found: 1358

Enter (or paste) list of ORFs

Find ORFs

Test	Cutoff
q-Value	.005
p-Value	

Colour	Signif	Direction	Applies To
red	yes	+	ratios, bars
yellow	no	n/a	bars
green	yes	–	ratios, bars
light red	yes	+	*p*- , *q*-Values
light green	yes	–	*p*- , *q*-Values

Dot Plots | Dot Plots

FnSg vs Fn

Fusobacterium nucleatum

Hackett Laboratory UW

Fn Summary Table | FnPg vs Fn | FnSg vs Fn | FnPgSg vs Fn | FnPgSg vs FnPg | FnSg vs FnPg | FnPgSg vs FnSg | Fn Coverage

Protein	FnSg vs Fn Log_2 Ratio	Log_2 Sum	*q*-Value	*p*-Value	Raw *FnSg*	Raw *Fn*	Normalized *FnSg*	Normalized *Fn*	Description
FN0106	0.078	13.533	4.921e-2	2.742e-1	82	87	106.7326	112.6563	AAL94315.1| Hypothetical protein
					117	71	117.0000	99.2871	
FN0108	1.487	8.008	9.4e-4	2.763e-3	19	4	24.7307	5.1796	AAL94317.1| Microcin C7 self-immunity protein mccF
					29	10	29.0000	13.9841	
FN0110	-0.130	17.494	4.738e-3	1.946e-2	303	352	394.3898	455.8049	AAL94319.1| Seryl-tRNA synthetase
					427	317	427.0000	443.2959	
FN0113	1.648	12.879	2.225e-5	1.905e-5	114	39	148.3843	50.5011	AAL94322.1| Heat-inducible transcription repressor hrcA
					159	34	159.0000	47.5459	
FN0114	-0.075	14.091	1.259e-1	7.656e-1	71	109	92.4148	141.1441	AAL94323.1| GrpE protein
					165	93	165.0000	130.0521	
FN0116	0.796	20.976	2.799e-2	1.482e-1	902	797	1174.0581	1032.0355	AAL94325.1| Chaperone protein dnaK
					2611	821	2611.0000	1148.0944	
FN0117	1.687	7.298			10		13.0162		AAL94326.1| O6-methylguanine-DNA methyltransferase
					32	5	32.0000	6.9920	
FN0118	-0.444	12.870	2.452e-3	8.742e-3	51	77	66.3824	99.7073	AAL94327.1| Chaperone protein dnaJ
					82	73	82.0000	102.0839	
FN0119	2.217	9.566	7.543e-3	3.351e-2	29	10	37.7469	12.9490	AAL94328.1| Flavodoxin
					81	9	81.0000	12.5857	
FN0123	-0.005	5.782	1.538e-1	9.655e-1	6	5	7.8097	6.4745	AAL94332.1| ATPase
					7	6	7.0000	8.3905	
FN0127					5		6.5081		AAL94333.1| Fe-S oxidoreductase
					7		7.0000		
FN0128	-0.987	9.255	6.848e-3	2.989e-2	7	30	9.1113	38.8470	AAL94334.1| Spermidine/putrescine-binding protein
					26	22	26.0000	30.7650	
FN0129						3		3.8847	AAL94335.1| Urease accessory protein ureG
FN0130	-0.093	8.466			8	15	10.4129	19.4235	AAL94336.1| ABC transporter ATP-binding protein
					26		26.0000		
FN0132	-0.484	5.654							AAL93916.1| Hemolysin
					6	6	6.0000	8.3905	
FN0147	-0.653	10.659	7.029e-4	1.917e-3	27	39	35.1436	50.5011	AAL94353.1| PLSX protein
					29	36	29.0000	50.3427	

Log_2 Ratios: -6 -4 -2 0 2 4 6

Show detected proteins only
Show all proteins
Filter by category:
GO: amino acid transport

Proteins found: 1358

Enter (or paste) list of ORFs

Find ORFs

Test	Cutoff
q-Value	.005
p-Value	

	Signif	Direction	Applies To
red	yes	+	ratios, bars
yellow	no	n/a	bars
green	yes	−	ratios, bars
pink	yes	+	*p*- , *q*-Values
light green	yes	−	

Dot Plots Dot Plots

Protein	*FnSg* vs Fn: Log_2 Ratio	Log_2 Sum	*q*-Value	*p*-Value	Raw: *FnSg*	Raw: *Fn*	Normalized: *FnSg*	Normalized: *Fn*	Description
FN0148	-0.203	16.184	7.082e-2	4.05e-1	248	234	322.8009	303.0067	AAL94354.1| 3-oxoacyl-[acyl-carrier-protein] synthase III
					186	202	186.0000	282.4788	
FN0149	-1.231	15.077	7.309e-4	2.022e-3	115	252	149.6859	326.3149	AAL94355.1| Malonyl-CoA-[acyl-carrier-protein] transacylase
					93	174	93.0000	243.3233	
FN0150	-1.071	11.516	6.755e-5	9.361e-5	32	65	41.6517	84.1685	AAL94356.1| Acyl carrier protein
					33	52	33.0000	72.7173	
FN0151	-0.661	19.578	7.627e-3	3.394e-2	389	825	506.3288	1068.2927	AAL94357.1| 3-oxoacyl-[acyl-carrier-protein] synthase
					901	827	901.0000	1156.4848	
FN0152	0.571	5.429				4		5.1796	AAL94358.1| Ribonuclease III
					8	4	8.0000	5.5936	
FN0153									AAL94359.1| Oxygen-independent coproporphyrinogen III oxidase
					13		13.0000		
FN0154						7		9.0643	AAL94360.1| Ribonuclease G
FN0155	-1.221	5.865				9		11.6541	AAL94361.1| Hypothetical protein
					5		5.0000		
FN0156	-0.196	7.832	1.188e-1	7.161e-1	4	12	5.2065	15.5388	AAL94362.1| Phosphopantetheine adenylyltransferase
					23	12	23.0000	16.7809	
FN0157	-0.237	6.014	1.592e-3	5.208e-3	6	7	7.8097	9.0643	AAL94363.1| DNA repair protein RadA
					7	6	7.0000	8.3905	
FN0158	0.623	10.543	2.694e-3	9.808e-3	36	20	46.8582	25.8980	AAL94364.1| DNA-binding protein
					49	26	49.0000	36.3587	
FN0161									AAL94367.1| RNA-directed DNA polymerase
					3		3.0000		
FN0164	-0.239	16.573	5.724e-2	3.226e-1	278	243	361.8494	314.6608	AAL94370.1| Anhydro-N-acetylmuramyl-tripeptide amidase
					213	260	213.0000	363.5865	
FN0170	0.706	14.509	8.89e-3	4.064e-2	179	95	232.9894	123.0155	AAL94376.1| GTP-binding protein
					157	83	157.0000	116.0680	
FN0173	0.018	11.786	1.478e-1	9.213e-1	55	48	71.5889	62.1552	AAL94379.1| Hypothetical protein
					48	40	48.0000	55.9364	
FN0174	-0.094	12.923	4.424e-2	2.442e-1	65	65	84.6051	84.1685	AAL94380.1| Enoyl-[acyl-carrier-protein] reductase
					86	70	86.0000	97.8887	

Log₂ Ratios: -6 -4 -2 0 2 4 6

Show detected proteins only
Show all proteins
Filter by category:
GO: amino acid transport

Proteins found: 1358

Enter (or paste) list of ORFs

Find ORFs

Test	Cutoff
q-Value	.005
p-Value	

	Signif	Direction	Applies To
red	yes	+	ratios, bars
yellow	no	n/a	bars
green	yes	−	ratios, bars
pink	yes	+	*p*- , *q*-Values
light green	yes	−	

Dot Plots Dot Plots

Protein	*FnSg* vs *Fn* Log$_2$ Ratio	Log$_2$ Sum	*q*-Value	*p*-Value	Raw *FnSg*	Raw *Fn*	Normalized *FnSg*	Normalized *Fn*	Description	Log$_2$ Ratios (-6 -4 -2 0 2 4 6)
**FN0175**	0.907	9.357	1.103e-3	3.347e-3	27	17	35.1436	22.0133	AAL94381.1| Cell division inhibitor MinC	
					35	11	35.0000	15.3825		
**FN0176**	0.785	15.983	1.232e-4	2.054e-4	272	158	354.0397	204.5942	AAL94382.1| Cell division inhibitor MinD	
					314	131	314.0000	183.1917		
**FN0177**						10		12.9490	AAL94383.1| Cell division inhibitor MinE	
						12		16.7809		
**FN0178**	-1.525	11.866	9.301e-7	1.343e-7	30	78	39.0485	101.0022	AAL94384.1| UNC-44 ankyrins	
					33	76	33.0000	106.2791		
**FN0179**	-0.200	10.006	8.015e-2	4.63e-1	16	25	20.8259	32.3725	AAL94385.1| Ankyrin repeat proteins	
					39	26	39.0000	36.3587		
**FN0180**					4		5.2065		AAL94386.1| Tetratricopeptide repeat family protein	
					6		6.0000			
**FN0181**	0.505	9.734	1.944e-2	9.927e-2	25	13	32.5404	16.8337	AAL94387.1| Hypothetical protein	
					37	23	37.0000	32.1634		
**FN0182**	3.099	15.422	3.534e-7	3.74e-8	474	49	616.9663	63.4501	AAL94388.1| Sarcosine oxidase alpha subunit	
					610	57	610.0000	79.7094		
**FN0183**	1.523	15.550	3.969e-5	4.325e-5	274	97	356.6429	125.6053	AAL94389.1| Glycerol-3-phosphate dehydrogenase	
					386	95	386.0000	132.8489		
**FN0185**	0.084	8.597	1.319e-1	8.073e-1	15	9	19.5242	11.6541	AAL94391.1| Hypothetical protein	
					21	19	21.0000	26.5698		
**FN0188**						13		16.8337	AAL94394.1| Peptide methionine sulfoxide reductase	
**FN0189**					12		15.6194		AAL94395.1| Two-component response regulator yesN	
					12		12.0000			
**FN0190**	-0.535	6.047			5		6.5081		AAL94396.1| Two-component sensor kinase yesM	
					7	7	7.0000	9.7889		
**FN0191**	0.605	9.874	1.279e-2	6.207e-2	35	20	45.5566	25.8980	AAL94397.1| helix-turn-helix DNA-binding protein	
					30	17	30.0000	23.7730		
**FN0192**	-2.532	11.379	5.468e-5	6.886e-5	13	88	16.9210	113.9512	AAL94398.1| Dipeptide-binding protein	
					26	96	26.0000	134.2473		
**FN0197**	-0.162	4.806							AAL94403.1| Methyltransferase	
					5	4	5.0000	5.5936		

Show detected proteins only
Show all proteins
Filter by category:
GO: amino acid transport

Proteins found: 1358

Enter (or paste) list of ORFs

Find ORFs

Test	Cutoff
q-Value	.005
p-Value	

	Signif	Direction	Applies To
red	yes	+	ratios, bars
yellow	no	n/a	bars
green	yes	–	ratios, bars
pink	yes	+	*p*- , *q*-Values
light green	yes	–	

Dot Plots | Dot Plots

FnSg vs Fn

Fusobacterium nucleatum

Hackett Laboratory UW

Fn Summary Table | FnPg vs Fn | FnSg vs Fn | FnPgSg vs Fn | FnPgSg vs FnPg | FnSg vs FnPg | FnPgSg vs FnSg | Fn Coverage

Protein	FnSg vs Fn Log_2 Ratio	Log_2 Sum	*q*-Value	*p*-Value	Raw *FnSg*	Raw *Fn*	Normalized *FnSg*	Normalized *Fn*	Description
FN0198						6		7.7694	AAL94404.1| Transcriptional regulatory protein
						4		5.5936	
FN0199	0.776	11.484	1.755e-4	3.272e-4	50	34	65.0808	44.0266	AAL94405.1| Hypothetical protein
					75	27	75.0000	37.7571	
FN0200	-2.133	19.975	1.742e-7	1.324e-8	385	1665	501.1224	2156.0089	AAL94406.1| Biotin carboxyl carrier protein of glutaconyl-COA decarboxylase
					468	1499	468.0000	2096.2162	
FN0202	-0.967	19.622	6.741e-5	9.332e-5	454	1032	590.9339	1336.3371	AAL94408.1| Glutaconate CoA-transferase subunit A
					694	840	694.0000	1174.6642	
FN0203	-0.582	20.663	7.506e-4	2.098e-3	730	1114	950.1801	1442.5189	AAL94409.1| Glutaconate CoA-transferase subunit B
					1156	1223	1156.0000	1710.2551	
FN0204	-0.312	21.692	7.16e-3	3.155e-2	1129	1600	1469.5251	2071.8404	AAL94410.1| Glutaconyl-CoA decarboxylase A subunit
					1835	1452	1835.0000	2030.4909	
FN0206	-1.164	12.032	2.516e-4	5.09e-4	38	74	49.4614	95.8226	AAL94412.1| Activator of (R)-2-hydroxyglutaryl-CoA dehydratase
					37	70	37.0000	97.8887	
FN0207	-0.565	18.986	1.392e-5	9.757e-6	444	681	577.9178	881.8271	AAL94413.1| (R)-2-hydroxyglutaryl-CoA dehydratase alpha-subunit
					607	623	607.0000	871.2093	
FN0208	-0.732	17.742	3.443e-4	7.682e-4	254	461	330.6106	596.9490	AAL94414.1| (R)-2-hydroxyglutaryl-CoA dehydratase beta-subunit
					396	436	396.0000	609.7066	
FN0209	-0.604	17.952	5.39e-3	2.255e-2	248	486	322.8009	629.3215	AAL94415.1| Hypothetical cytosolic protein
					494	438	494.0000	612.5035	
FN0212	-0.493	8.850	1.5e-3	4.861e-3	14	21	18.2226	27.1929	AAL94418.1| Hypothetical protein
					18	17	18.0000	23.7730	
FN0218	-0.964	13.314	7.957e-5	1.179e-4	48	114	62.4776	147.6186	AAL94424.1| Anthranilate synthase component II
					82	96	82.0000	134.2473	
FN0219	1.435	6.180				4		5.1796	AAL94425.1| Autolysin response regulator
					14		14.0000		
FN0221	-0.581	10.846	2.334e-5	2.036e-5	27	40	35.1436	51.7960	AAL94427.1| Carbon starvation protein A
					35	38	35.0000	53.1396	
FN0224	-1.410	8.676	1.294e-3	4.071e-3	6	25	7.8097	32.3725	AAL94430.1| Excinuclease ABC subunit B
					17	24	17.0000	33.5618	
FN0225									AAL94431.1| Gluconate permease
					3		3.0000		

Log_2 Ratios: -6 -4 -2 0 2 4 6

Show detected proteins only
Show all proteins
Filter by category:
GO: amino acid transport

Proteins found: 1358

Enter (or paste) list of ORFs

Find ORFs

Test	Cutoff
q-Value	.005
p-Value	

	Signif	Direction	Applies To
red	yes	+	ratios, bars
yellow	no	n/a	bars
green	yes	–	ratios, bars
pink	yes	+	*p*- , *q*-Values
light green	yes	–	*p*- , *q*-Values

Dot Plots Dot Plots

Protein	FnSg vs Fn Log$_2$ Ratio	Log$_2$ Sum	*q*-Value	*p*-Value	Raw *FnSg*	Raw *Fn*	Normalized *FnSg*	Normalized *Fn*	Description
FN0226	-1.034	11.561	2.8e-4	5.852e-4	26	61	33.8420	78.9889	AAL94432.1| Pyridoxal phosphate biosynthetic protein pdxA
					43	56	43.0000	78.3109	
FN0227									AAL94433.1| Hypothetical protein
					5		5.0000		
FN0233	-0.734	12.106	2.775e-4	5.783e-4	43	61	55.9695	78.9889	AAL94439.1| Hypothetical protein
					47	66	47.0000	92.2950	
FN0234	-0.310	7.958	1.264e-2	6.125e-2	11	12	14.3178	15.5388	AAL94440.1| unknown
					14	14	14.0000	19.5777	
FN0236	-1.222	14.608	2.996e-4	6.409e-4	76	172	98.9229	222.7228	AAL94442.1| ABC transporter substrate-binding protein
					108	186	108.0000	260.1042	
FN0238	-1.033	10.270	1.535e-4	2.777e-4	17	42	22.1275	54.3858	AAL94444.1| Hypothetical protein
					27	33	27.0000	46.1475	
FN0240	-0.797	12.601	8.865e-4	2.574e-3	55	87	71.5889	112.6563	AAL94446.1| Thymidylate synthase
					48	68	48.0000	95.0919	
FN0241	0.394	9.714	2.811e-2	1.489e-1	28	25	36.4453	32.3725	AAL94447.1| Dihydrofolate reductase
					30	13	30.0000	18.1793	
FN0242	0.936	13.142	2.68e-3	9.744e-3	116	50	150.9875	64.7450	AAL94448.1| Trk system potassium uptake protein trkA
					112	52	112.0000	72.7173	
FN0243	0.487	9.651	5.927e-2	3.348e-1	17	10	22.1275	12.9490	AAL94449.1| Poly(A) polymerase
					45	25	45.0000	34.9602	
FN0244	-1.640	7.571			6	16	7.8097	20.7184	AAL94450.1| COP associated protein
						20		27.9682	
FN0245	-0.330	7.699	2.374e-3	8.403e-3	9	12	11.7145	15.5388	AAL94451.1| Copper-exporting ATPase
					14	12	14.0000	16.7809	
FN0247	-0.290	15.475	6.662e-8	3.15e-9	149	183	193.9409	236.9667	AAL94453.1| Hypothetical cytosolic protein
					192	168	192.0000	234.9328	
FN0248					28		36.4453		AAL94454.1| Hypothetical Exported Protein
					65		65.0000		
FN0249	-1.817	14.263	4.337e-4	1.021e-3	81	210	105.4309	271.9291	AAL94455.1| unknown
					44	182	44.0000	254.5106	
FN0250	-0.996	13.556	3.358e-3	1.293e-2	81	113	105.4309	146.3237	AAL94456.1| unknown
					50	117	50.0000	163.6139	

Log$_2$ Ratios: -6 -4 -2 0 2 4 6

Show detected proteins only
Show all proteins
Filter by category:
GO: amino acid transport

Proteins found: 1358

Enter (or paste) list of ORFs

Find ORFs

Test	Cutoff
q-Value	.005
p-Value	

	Signif	Direction	Applies To
red	yes	+	ratios, bars
yellow	no	n/a	bars
green	yes	−	ratios, bars
pink	yes	+	*p*- , *q*-Values
light green	yes	−	

Dot Plots Dot Plots

Protein	*FnSg* vs *Fn* Log$_2$ Ratio	Log$_2$ Sum	*q*-Value	*p*-Value	Raw *FnSg*	Raw *Fn*	Normalized *FnSg*	Normalized *Fn*	Description
FN0251	-2.496	13.698	1.488e-4	2.659e-4	40	195	52.0647	252.5055	AAL94457.1| Hypothetical membrane-spanning Protein
					45	211	45.0000	295.0644	
FN0252	0.575	17.548	2.44e-4	4.894e-4	430	270	559.6951	349.6231	AAL94458.1| unknown
					509	263	509.0000	367.7818	
FN0253	0.460	16.509	4.768e-2	2.649e-1	180	198	234.2910	256.3902	AAL94459.1| Outer membrane protein
					482	189	482.0000	264.2994	
FN0254	-1.035	19.228	3.087e-6	9.382e-7	444	851	577.9178	1101.9601	AAL94460.1| Fusobacterium outer membrane protein family
					517	816	517.0000	1141.1023	
FN0258	-1.188	6.803				16		20.7184	AAL94464.1| Zinc-transporting ATPase
					7	8	7.0000	11.1873	
FN0260									AAL94466.1| Transcriptional repressor smtB
						5		6.9920	
FN0261	-1.840	8.927	1.912e-5	1.542e-5	11	31	14.3178	40.1419	AAL94467.1| Pyruvate formate-lyase activating enzyme
					9	31	9.0000	43.3507	
FN0262	-2.335	22.689	1.237e-5	8.314e-6	878	4406	1142.8193	5705.3305	AAL94468.1| Formate acetyltransferase
					1173	4274	1173.0000	5976.8031	
FN0263	-1.850	15.700	1.178e-5	7.68e-6	106	358	137.9714	463.5743	AAL94469.1| Peptidyl-prolyl cis-trans isomerase
					105	295	105.0000	412.5309	
FN0264	-0.960	16.331	3.951e-3	1.586e-2	211	314	274.6411	406.5987	AAL94470.1| Hypothetical protein
					137	282	137.0000	394.3515	
FN0265	-1.763	7.956	4.807e-4	1.165e-3	7	20	9.1113	25.8980	AAL94471.1| Cell division protein ftsX
					8	23	8.0000	32.1634	
FN0266						14		18.1286	AAL94472.1| membrane protein related to metalloendopeptidase
						14		19.5777	
FN0267	-0.436	6.436				7		9.0643	AAL94473.1| ATP-NAD kinase
					8	9	8.0000	12.5857	
FN0268	0.250	8.535				10		12.9490	AAL94474.1| DNA repair protein recN
					21	16	21.0000	22.3746	
FN0270	-1.169	5.872	7.405e-4	2.059e-3	4	8	5.2065	10.3592	AAL94476.1| GTP-binding protein era
					5	9	5.0000	12.5857	
FN0271	0.093	6.327	1.263e-1	7.684e-1	5	8	6.5081	10.3592	AAL94477.1| Enoyl-CoA hydratase
					12	5	12.0000	6.9920	

Log$_2$ Ratios: -6 -4 -2 0 2 4 6

Show detected proteins only
Show all proteins
Filter by category:
GO: amino acid transport

Proteins found: 1358

Enter (or paste) list of ORFs

Find ORFs

Test	Cutoff
q-Value	.005
p-Value	

	Signif	Direction	Applies To
red	yes	+	ratios, bars
yellow	no	n/a	bars
green	yes	−	ratios, bars
pink	yes	+	*p*- , *q*-Values
light green	yes	−	

Dot Plots Dot Plots

Protein	*FnSg* vs *Fn* Log$_2$ Ratio	Log$_2$ Sum	*q*-Value	*p*-Value	Raw *FnSg*	Raw *Fn*	Normalized *FnSg*	Normalized *Fn*	Description
FN0272	0.588	6.199			10		13.0162		AAL94478.1| Acetoacetate: butyrate/acetate coenzyme A transferase
					8	5	8.0000	6.9920	
FN0273					4		5.2065		AAL94479.1| Butyrate-acetoacetate CoA-transferase subunit B
					11		11.0000		
FN0276	1.928	9.815			50		65.0808		AAL94482.1| Sodium-dependent phosphate transporter
					52	11	52.0000	15.3825	
FN0277	-0.494	7.388	2.194e-2	1.134e-1	6	14	7.8097	18.1286	AAL94483.1| Hypothetical protein
					14	9	14.0000	12.5857	
FN0278	-0.560	15.924	4.407e-5	5.09e-5	158	230	205.6554	297.8271	AAL94484.1| Xaa-His dipeptidase
					205	220	205.0000	307.6501	
FN0279	1.561	13.927	1.692e-4	3.134e-4	175	55	227.7829	71.2195	AAL94485.1| Lipoprotein
					201	53	201.0000	74.1157	
FN0280	-0.787	13.745	4.364e-5	5.015e-5	71	119	92.4148	154.0931	AAL94486.1| Hypothetical protein
					86	110	86.0000	153.8251	
FN0281	0.841	13.430	6.426e-3	2.77e-2	87	64	113.2406	82.8736	AAL94487.1| DNA polymerase III alpha subunit
					168	53	168.0000	74.1157	
FN0282	0.138	11.307	6.655e-2	3.789e-1	45	32	58.5727	41.4368	AAL94488.1| Hypothetical protein
					47	39	47.0000	54.5380	
FN0283	-1.636	7.976							AAL94489.1| tRNA (Guanine-N1) -methyltransferase
					9	20	9.0000	27.9682	
FN0284	1.667	9.668	1.303e-3	4.103e-3	32	15	41.6517	19.4235	AAL94490.1| 16S rRNA processing protein rimM
					60	9	60.0000	12.5857	
FN0285						4		5.1796	AAL94491.1| RNA binding protein
FN0287	-0.460	7.032	2.728e-2	1.441e-1	5	11	6.5081	14.2439	AAL94493.1| Dimethyladenosine transferase
					13	9	13.0000	12.5857	
FN0288	0.674	10.925	4.516e-5	5.287e-5	41	28	53.3663	36.2572	AAL94494.1| Hypoxanthine-guanine phosphoribosyltransferase
					58	24	58.0000	33.5618	
FN0291	0.253	4.391							AAL94497.1| Hemolysin
					5	3	5.0000	4.1952	
FN0294	-1.901	14.385	2.457e-5	2.192e-5	71	212	92.4148	274.5189	AAL94500.1| Transketolase subunit A
					59	208	59.0000	290.8692	

Log$_2$ Ratios: -6 -4 -2 0 2 4 6

Show detected proteins only
Show all proteins
Filter by category:
GO: amino acid transport

Proteins found: 1358

Enter (or paste) list of ORFs

Find ORFs

Test	Cutoff
q-Value	.005
p-Value	

	Signif	Direction	Applies To
red	yes	+	ratios, bars
yellow	no	n/a	bars
green	yes	−	ratios, bars
pink	yes	+	*p*- , *q*-Values
light green	yes	−	

Dot Plots Dot Plots

FnSg vs Fn

Fusobacterium nucleatum

Hackett Laboratory UW

Fn Summary Table | FnPg vs Fn | FnSg vs Fn | FnPgSg vs Fn | FnPgSg vs FnPg | FnSg vs FnPg | FnPgSg vs FnSg | Fn Coverage

Protein	*FnSg* vs *Fn* Log$_2$ Ratio	Log$_2$ Sum	*q*-Value	*p*-Value	Raw *FnSg*	Raw *Fn*	Normalized *FnSg*	Normalized *Fn*	Description
FN0295	-0.904	14.198	1.199e-4	1.978e-4	71	146	92.4148	189.0554	AAL94501.1| Transketolase
					108	133	108.0000	185.9885	
FN0296	0.166	11.396	6.794e-4	1.83e-3	43	39	55.9695	50.5011	AAL94502.1| Hypothetical cytosolic protein
					54	34	54.0000	47.5459	
FN0297	1.165	8.400	1.973e-3	6.701e-3	20	6	26.0323	7.7694	AAL94503.1| ATPase associated with chromosome architecture/replication
					29	12	29.0000	16.7809	
FN0298	0.466	15.903	6.287e-4	1.652e-3	225	173	292.8637	224.0177	AAL94504.1| Histidyl-tRNA synthetase
					289	141	289.0000	197.1758	
FN0299	-0.265	16.401	6.204e-4	1.623e-3	198	239	257.7201	309.4812	AAL94505.1| Aspartyl-tRNA synthetase
					279	240	279.0000	335.6183	
FN0305									AAL94511.1| Iron(III) dicitrate-binding protein
						3		4.1952	
FN0308	0.247	17.772	2.211e-3	7.692e-3	417	347	542.7741	449.3304	AAL94514.1| Iron(III)-binding protein
					488	300	488.0000	419.5229	
FN0309									AAL94515.1| Iron(III)-transport system permease protein sfuB
						8		11.1873	
FN0310	-1.748	13.044	7.401e-6	3.645e-6	44	123	57.2711	159.2727	AAL94516.1| Iron(III)-transport ATP-binding protein sfuC
					43	127	43.0000	177.5980	
FN0311	0.278	14.409	2.202e-3	7.657e-3	132	100	171.8134	129.4900	AAL94517.1| Anaerobic ribonucleoside-triphosphate reductase
					153	99	153.0000	138.4426	
FN0313	-0.127	9.558	1.263e-1	7.682e-1	25	13	32.5404	16.8337	AAL94519.1| 16S rRNA m(5)C 967 methyltransferase
					20	29	20.0000	40.5539	
FN0314					9		11.7145		AAL94520.1| Caffeoyl-CoA O-methyltransferase
					9		9.0000		
FN0315					5		6.5081		AAL94521.1| Transcriptional regulator, AraC family
					10		10.0000		
FN0316	-1.888	8.548	1.29e-3	4.053e-3	7	24	9.1113	31.0776	AAL94522.1| Hypothetical protein
					11	31	11.0000	43.3507	
FN0317	1.397	9.164	1.666e-6	3.453e-7	29	12	37.7469	15.5388	AAL94523.1| Tryptophan synthase beta chain
					40	10	40.0000	13.9841	
FN0319	-0.061	6.574	1.346e-1	8.262e-1	7	10	9.1113	12.9490	AAL94525.1| Citrate (pro-3S)-lyase ligase
					10	5	10.0000	6.9920	

Log$_2$ Ratios: -6 -4 -2 0 2 4 6

Show detected proteins only
Show all proteins
Filter by category:
GO: amino acid transport

Proteins found: 1358

Enter (or paste) list of ORFs

Find ORFs

Test	Cutoff
q-Value	.005
p-Value	

	Signif	Direction	Applies To
red	yes	+	ratios, bars
yellow	no	n/a	bars
green	yes	−	ratios, bars
pink	yes	+	*p*-, *q*-Values
light green	yes	−	

Dot Plots Dot Plots

Protein	FnSg vs Fn Log$_2$ Ratio	Log$_2$ Sum	*q*-Value	*p*-Value	Raw *FnSg*	Raw *Fn*	Normalized *FnSg*	Normalized *Fn*	Description
FN0320	-1.308	8.395	3.111e-4	6.747e-4	11	23	14.3178	29.7827	AAL94526.1| Hypothetical cytosolic protein
					9	20	9.0000	27.9682	
FN0321	0.889	14.701	4.346e-3	1.766e-2	140	101	182.2263	130.7849	AAL94527.1| Heat shock protein htpG
					262	78	262.0000	109.0760	
FN0322	0.336	21.739	1.279e-2	6.211e-2	1816	1315	2363.7357	1702.7938	AAL94528.1| Fructose-bisphosphate aldolase
					1839	1164	1839.0000	1627.7489	
FN0325	-4.235	8.235				58		75.1042	AAL94529.1| LSU ribosomal protein L20P
					4	54	4.0000	75.5141	
FN0326						7		9.0643	AAL94530.1| LSU ribosomal protein L35P
FN0327	-1.747	11.489	4.453e-4	1.056e-3	15	75	19.5242	97.1175	AAL94531.1| Bacterial Protein Translation Initiation Factor 3 (IF-3)
					39	71	39.0000	99.2871	
FN0329	-0.950	17.849	1.405e-4	2.458e-4	243	528	316.2928	683.7073	AAL94533.1| LSU ribosomal protein L13P
					383	477	383.0000	667.0414	
FN0330	-1.857	11.791	2.129e-4	4.128e-4	35	81	45.5566	104.8869	AAL94534.1| SSU ribosomal protein S9P
					17	87	17.0000	121.6616	
FN0331	-0.199	12.270	2.748e-2	1.452e-1	57	58	74.1921	75.1042	AAL94535.1| Hypothetical protein
					57	54	57.0000	75.5141	
FN0332					13		16.9210		AAL94536.1| Magnesium and cobalt transport protein corA
					8		8.0000		
FN0333					5		6.5081		AAL94537.1| Glycerol uptake operon antiterminator regulatory protein
					9		9.0000		
FN0334	0.615	15.196	1.787e-3	5.959e-3	168	123	218.6716	159.2727	AAL94538.1| Aspartate/aromatic aminotransferase
					261	110	261.0000	153.8251	
FN0335	0.528	21.589	2.478e-3	8.853e-3	1798	1093	2340.3066	1415.3260	AAL94539.1| Outer membrane porin F
					1925	1103	1925.0000	1542.4459	
FN0336	-1.423	12.122	2.481e-4	5e-4	35	76	45.5566	98.4124	AAL94540.1| Hypothetical protein
					36	86	36.0000	120.2632	
FN0341	-2.957	9.423	2.092e-4	4.04e-4	6	62	7.8097	80.2838	AAL94545.1| transport protein
					11	47	11.0000	65.7253	
FN0342	-0.833	10.292	1.718e-2	8.673e-2	30	46	39.0485	59.5654	AAL94546.1| Peptidyl-prolyl cis-trans isomerase
					14	25	14.0000	34.9602	

Log$_2$ Ratios: -6 -4 -2 0 2 4 6

Show detected proteins only
Show all proteins
Filter by category:
GO: amino acid transport

Proteins found: 1358

Enter (or paste) list of ORFs

Find ORFs

Test	Cutoff
q-Value	.005
p-Value	

	Signif	Direction	Applies To
red	yes	+	ratios, bars
yellow	no	n/a	bars
green	yes	−	ratios, bars
pink	yes	+	*p*- , *q*-Values
light green	yes	−	

Dot Plots Dot Plots

Protein	FnSg vs Fn Log2 Ratio	Log2 Sum	q-Value	p-Value	Raw FnSg	Raw Fn	Normalized FnSg	Normalized Fn	Description
FN0343						4		5.1796	AAL94547.1| Hypothetical protein
FN0344	-2.069	5.239							AAL94548.1| Methyltransferase
					3	9	3.0000	12.5857	
FN0347	-0.674	12.887	4.826e-4	1.171e-3	49	78	63.7792	101.0022	AAL94551.1| Phosphatidylserine decarboxylase
					74	85	74.0000	118.8648	
FN0348	-0.391	16.381	1.208e-2	5.806e-2	233	279	303.2767	361.2772	AAL94552.1| Nicotinate phosphoribosyltransferase
					207	220	207.0000	307.6501	
FN0349	1.812	8.670	3.665e-3	1.446e-2	22	8	28.6356	10.3592	AAL94553.1| D-Tyr-tRNATyr deacylase
					47	8	47.0000	11.1873	
FN0351	-2.482	12.351	3.184e-4	6.951e-4	27	117	35.1436	151.5033	AAL94555.1| unknown
					26	136	26.0000	190.1837	
FN0352	-0.286	11.121	3.59e-2	1.95e-1	38	47	49.4614	60.8603	AAL94556.1| NA+/H+ antiporter NHAC
					36	31	36.0000	43.3507	
FN0355	-0.041	15.483	1.287e-1	7.85e-1	189	170	246.0055	220.1330	AAL94558.1| S-adenosylmethionine synthetase
					176	153	176.0000	213.9567	
FN0356	-2.283	8.658			7	35	9.1113	45.3215	AAL94559.1| Lactoylglutathione lyase
						31		43.3507	
FN0357	-0.449	7.657	4.661e-2	2.584e-1	11	17	14.3178	22.0133	AAL94560.1| ATP synthase epsilon chain, sodium ion specific
					10	8	10.0000	11.1873	
FN0358	-0.347	18.121	1.156e-2	5.518e-2	420	470	546.6790	608.6031	AAL94561.1| ATP synthase beta chain, sodium ion specific
					400	426	400.0000	595.7225	
FN0359	-0.269	12.511	2.135e-3	7.37e-3	57	68	74.1921	88.0532	AAL94562.1| ATP synthase gamma chain, sodium ion specific
					65	57	65.0000	79.7094	
FN0360	0.487	14.273	2.811e-3	1.034e-2	140	95	182.2263	123.0155	AAL94563.1| ATP synthase alpha chain, sodium ion specific
					151	82	151.0000	114.6696	
FN0361	-0.759	10.265	9.707e-3	4.517e-2	13	37	16.9210	47.9113	AAL94564.1| ATP synthase delta chain, sodium ion specific
					37	31	37.0000	43.3507	
FN0362	-0.065	11.135	1.364e-1	8.393e-1	29	49	37.7469	63.4501	AAL94565.1| ATP synthase B chain, sodium ion specific
					55	24	55.0000	33.5618	
FN0364									AAL94567.1| ATP synthase A chain, sodium ion specific
						3		4.1952	

Log2 Ratios: -6 -4 -2 0 2 4 6

Show detected proteins only
Show all proteins
Filter by category:
GO: amino acid transport

Proteins found: 1358

Enter (or paste) list of ORFs

Find ORFs

Test	Cutoff
q-Value	.005
p-Value	

	Signif	Direction	Applies To
red	yes	+	ratios, bars
yellow	no	n/a	bars
green	yes	−	ratios, bars
pink	yes	+	p- , q-Values
light green	yes	−	

Dot Plots Dot Plots

Protein	FnSg vs Fn Log$_2$ Ratio	Log$_2$ Sum	*q*-Value	*p*-Value	Raw *FnSg*	Raw *Fn*	Normalized *FnSg*	Normalized *Fn*	Description
FN0366	-0.416	15.974	6.335e-3	2.724e-2	193	228	251.2120	295.2373	AAL94569.1| Phosphoglucosamine mutase
					188	208	188.0000	290.8692	
FN0368	-0.530	12.567	2.842e-3	1.048e-2	42	69	54.6679	89.3481	AAL94571.1| Adenylosuccinate lyase
					75	70	75.0000	97.8887	
FN0370	-0.772	10.147	5.368e-3	2.244e-2	25	28	32.5404	36.2572	AAL94573.1| Signal peptidase I
					19	37	19.0000	51.7412	
FN0371	-2.901	10.001			9	66	11.7145	85.4634	AAL94574.1| Hypothetical protein
						64		89.4982	
FN0374	2.077	6.567				3		3.8847	AAL94577.1| Single-stranded-DNA-specific exonuclease recJ
					20	4	20.0000	5.5936	
FN0375	1.101	18.363	1.719e-3	5.694e-3	740	297	963.1963	384.5854	AAL94578.1| Iron(III)-binding protein
					738	292	738.0000	408.3356	
FN0376	1.150	13.536	3.241e-4	7.114e-4	115	59	149.6859	76.3991	AAL94579.1| Iron(III)-transport ATP-binding protein sfuC
					175	50	175.0000	69.9205	
FN0377	-1.332	5.976							AAL94580.1| Iron(III)-transport system permease protein sfuB
					5	9	5.0000	12.5857	
FN0378	-0.339	6.134	7.115e-2	4.071e-1	3	7	3.9048	9.0643	AAL94581.1| UDP-glucose 4-epimerase
					11	7	11.0000	9.7889	
FN0379	-1.155	6.325				12		15.5388	AAL94582.1| Hypothetical protein
					6	8	6.0000	11.1873	
FN0380						26		33.6674	AAL94583.1| unknown
						17		23.7730	
FN0381						15		19.4235	AAL94584.1| unknown
						15		20.9761	
FN0382						8		10.3592	AAL94585.1| Hypothetical protein
						8		11.1873	
FN0383						7		9.0643	AAL94586.1| Lipopolysaccharide N-acetylglucosaminyltransferase
						10		13.9841	
FN0384	-3.317	7.723	2.241e-4	4.398e-4	4	32	5.2065	41.4368	AAL94587.1| Hypothetical protein
					4	36	4.0000	50.3427	
FN0386						12		15.5388	AAL94589.1| Hypothetical protein
						11		15.3825	

Show detected proteins only
Show all proteins
Filter by category:
GO: amino acid transport

Proteins found: 1358

Enter (or paste) list of ORFs

Find ORFs

Test	Cutoff
q-Value	.005
p-Value	

	Signif	Direction	Applies To
red	yes	+	ratios, bars
yellow	no	n/a	bars
green	yes	−	ratios, bars
pink	yes	+	*p*-, *q*-Values
light green	yes	−	*p*-, *q*-Values

Dot Plots Dot Plots

Protein	FnSg vs Fn Log2 Ratio	Log2 Sum	q-Value	p-Value	Raw FnSg	Raw Fn	Normalized FnSg	Normalized Fn	Description
FN0387	-2.598	12.135	2.352e-4	4.672e-4	5	121	6.5081	156.6829	AAL94590.1| Fusobacterium outer membrane protein family
					48	124	48.0000	173.4028	
FN0390	0.760	11.704	2.097e-3	7.21e-3	64	34	83.3035	44.0266	AAL94593.1| Hypothetical protein
					67	32	67.0000	44.7491	
FN0391	0.573	9.238	6.047e-5	7.95e-5	23	16	29.9372	20.7184	AAL94594.1| Hydrolase (HAD superfamily)
					30	14	30.0000	19.5777	
FN0392	-1.839	6.626	3.636e-5	3.78e-5	5	15	6.5081	19.4235	AAL94595.1| Oxygen-independent coproporphyrinogen III oxidase
					4	13	4.0000	18.1793	
FN0393	1.616	8.281	3.461e-3	1.344e-2	29	8	37.7469	10.3592	AAL94596.1| Polysaccharide deacetylase
					24	7	24.0000	9.7889	
FN0394	-0.481	6.856			7	11	9.1113	14.2439	AAL94597.1| Outer membrane protein
						8		11.1873	
FN0396	0.640	24.426	9.475e-3	4.386e-2	5397	2994	7024.8246	3876.9313	AAL94599.1| Dipeptide-binding protein
					4827	2667	4827.0000	3729.5587	
FN0397	-0.015	11.126	1.467e-1	9.138e-1	40	41	52.0647	53.0909	AAL94600.1| Dipeptide transport system permease protein dppB
					42	30	42.0000	41.9523	
FN0398	-0.502	12.128	1.843e-3	6.179e-3	38	56	49.4614	72.5144	AAL94601.1| Dipeptide transport system permease protein dppC
					63	62	63.0000	86.7014	
FN0399	-0.236	15.770	3.738e-2	2.036e-1	138	178	179.6231	230.4922	AAL94602.1| Dipeptide transport ATP-binding protein dppD
					256	202	256.0000	282.4788	
FN0400	-0.487	18.187	6.672e-4	1.787e-3	331	498	430.8351	644.8603	AAL94603.1| Dipeptide transport ATP-binding protein dppF
					492	464	492.0000	648.8621	
FN0405	-0.293	13.086	7.528e-3	3.343e-2	58	86	75.4938	111.3614	AAL94608.1| Tryptophanyl-tRNA synthetase
					93	68	93.0000	95.0919	
FN0406	-1.516	11.223	5.528e-4	1.399e-3	16	64	20.8259	82.8736	AAL94609.1| Alanine racemase, biosynthetic
					37	59	37.0000	82.5062	
FN0407	-1.735	11.438	4.116e-5	4.583e-5	19	75	24.7307	97.1175	AAL94610.1| Hypothetical protein
					33	68	33.0000	95.0919	
FN0408	-0.129	13.191	4.838e-2	2.692e-1	76	86	98.9229	111.3614	AAL94611.1| Acetyl-coenzyme A carboxylase carboxyl transferase subunit beta
					86	65	86.0000	90.8966	
FN0409	-0.987	17.352	2.609e-6	6.883e-7	219	453	285.0540	586.5898	AAL94612.1| Acetyl-coenzyme A carboxylase carboxyl transferase subunit alpha
					296	404	296.0000	564.9575	

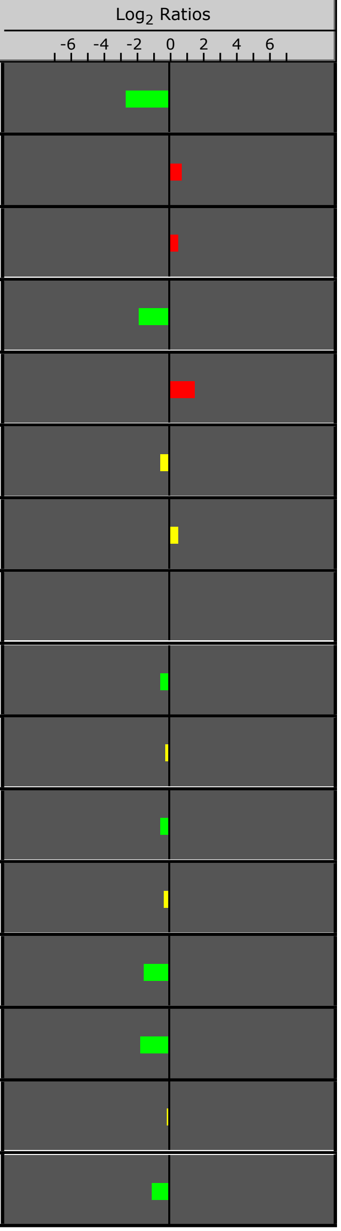

Show detected proteins only
Show all proteins
Filter by category:
GO: amino acid transport

Proteins found: 1358

Enter (or paste) list of ORFs
Find ORFs

Test	Cutoff
q-Value	.005
p-Value	

	Signif	Direction	Applies To
red	yes	+	ratios, bars
yellow	no	n/a	bars
green	yes	−	ratios, bars
pink	yes	+	p- , q-Values
light green	yes	−	

Dot Plots Dot Plots

Protein	*FnSg vs Fn* Log$_2$ Ratio	Log$_2$ Sum	*q*-Value	*p*-Value	Raw *FnSg*	Raw *Fn*	Normalized *FnSg*	Normalized *Fn*	Description	Log$_2$ Ratios -6 -4 -2 0 2 4 6
FN0410	-0.464	14.068	6.416e-4	1.696e-3	80	120	104.1293	155.3880	AAL94613.1| 6-phosphofructokinase	
					119	109	119.0000	152.4267		
FN0411	0.597	7.565			13		16.9210		AAL94614.1| putative alpha helix protein	
						8		11.1873		
FN0412									AAL94615.1| Recombination protein recR	
						10		13.9841		
FN0413						3		3.8847	AAL94616.1| unknown	
FN0414									AAL94617.1| ATP-dependent helicase HEPA	
					4		4.0000			
FN0416						18		23.3082	AAL94619.1| Type III restriction-modification system methylation subunit	
						23		32.1634		
FN0417	-2.598	9.242				46		59.5654	AAL94620.1| Type III restriction-modification system restriction subunit	
					10	44	10.0000	61.5300		
FN0418					77		100.2245		AAL94621.1| Uracil phosphoribosyltransferase	
					90		90.0000			
FN0419	-0.540	6.849			6	10	7.8097	12.9490	AAL94622.1| Aspartate carbamoyltransferase	
					10		10.0000			
FN0420	1.325	9.226	7.44e-3	3.298e-2	38	12	49.4614	15.5388	AAL94623.1| Dihydroorotase	
					28	11	28.0000	15.3825		
FN0421	0.333	9.614	2.941e-3	1.095e-2	26	18	33.8420	23.3082	AAL94624.1| Carbamoyl-phosphate synthase small chain	
					29	19	29.0000	26.5698		
FN0422	0.151	15.001	2.346e-2	1.22e-1	158	125	205.6554	161.8625	AAL94625.1| Carbamoyl-phosphate synthase large chain	
					176	130	176.0000	181.7933		
FN0423	1.262	8.485	7.005e-4	1.908e-3	22	7	28.6356	9.0643	AAL94626.1| Dihydroorotate dehydrogenase electron transfer subunit	
					30	11	30.0000	15.3825		
FN0424	1.988	6.847	1.193e-3	3.68e-3	19	4	24.7307	5.1796	AAL94627.1| Dihydroorotate dehydrogenase	
					18	4	18.0000	5.5936		
FN0426	1.669	9.837	1.307e-4	2.226e-4	46	10	59.8744	12.9490	AAL94629.1| Orotidine 5'-phosphate decarboxylase	
					48	15	48.0000	20.9761		
FN0427	-0.086	11.660	7.203e-2	4.125e-1	38	43	49.4614	55.6807	AAL94630.1| Orotate phosphoribosyltransferase	
					61	44	61.0000	61.5300		

Show detected proteins only
Show all proteins
Filter by category:
GO: amino acid transport

Proteins found: 1358

Enter (or paste) list of ORFs

Find ORFs

Test	Cutoff
q-Value	.005
p-Value	

	Signif	Direction	Applies To
	yes	+	ratios, bars
	no	n/a	bars
	yes	–	ratios, bars
	yes	+	*p*- , *q*-Values
	yes	–	

Dot Plots | Dot Plots

Protein	*FnSg vs Fn* Log$_2$ Ratio	Log$_2$ Sum	*q*-Value	*p*-Value	Raw *FnSg*	Raw *Fn*	Normalized *FnSg*	Normalized *Fn*	Description
FN0430	-0.701	15.787	1.786e-4	3.339e-4	159	223	206.9570	288.7628	AAL94633.1| LSU ribosomal protein L19P
					166	227	166.0000	317.4390	
FN0435	0.052	11.668	1.009e-1	5.961e-1	47	39	61.1760	50.5011	AAL94634.1| Purine nucleoside phosphorylase
					55	44	55.0000	61.5300	
FN0436	1.333	11.299	1.074e-2	5.071e-2	41	24	53.3663	31.0776	AAL94635.1| regulator of kinase autophosphorylation inhibitor
					106	23	106.0000	32.1634	
FN0437	0.806	7.847	7.057e-4	1.927e-3	17	8	22.1275	10.3592	AAL94636.1| kinase autophosphorylation inhibitor KipI
					18	9	18.0000	12.5857	
FN0438									AAL94637.1| transporter protein
						9		12.5857	
FN0439	-0.040	11.845	1.436e-1	8.912e-1	32	41	41.6517	53.0909	AAL94638.1| Lactam utilization protein LAMB
					78	50	78.0000	69.9205	
FN0445	-0.008	5.178				5		6.4745	AAL94641.1| Hypothetical protein
					6	4	6.0000	5.5936	
FN0446	0.027	8.501	1.438e-1	8.924e-1	18	14	23.4291	18.1286	AAL94642.1| Hypothetical protein
					15	14	15.0000	19.5777	
FN0447	1.685	6.516	9.326e-4	2.737e-3	11	5	14.3178	6.4745	AAL94643.1| NIFS protein
					20	3	20.0000	4.1952	
FN0450	-0.482	10.411	4.827e-2	2.685e-1	18	22	23.4291	28.4878	AAL94646.1| ABC transporter ATP-binding protein
					39	42	39.0000	58.7332	
FN0451	-0.319	6.512	3.795e-2	2.069e-1	7	10	9.1113	12.9490	AAL94647.1| Hypothetical protein
					8	6	8.0000	8.3905	
FN0452	-1.837	17.252	5.216e-6	2.031e-6	136	592	177.0199	766.5809	AAL94648.1| Glucosamine--fructose-6-phosphate aminotransferase (isomerizing)
					241	520	241.0000	727.1731	
FN0453	0.296	16.195	2.768e-3	1.014e-2	219	195	285.0540	252.5055	AAL94649.1| Xaa-Pro aminopeptidase
					322	173	322.0000	241.9249	
FN0454	1.417	14.629	1.243e-3	3.873e-3	173	77	225.1797	99.7073	AAL94650.1| Aldehyde dehydrogenase B
					295	68	295.0000	95.0919	
FN0455	1.718	16.964	4.709e-4	1.134e-3	547	150	711.9843	194.2350	AAL94651.1| Rubrerythrin
					585	143	585.0000	199.9726	
FN0456	-2.542	10.127	1.164e-5	7.519e-6	9	62	11.7145	80.2838	AAL94652.1| Hypothetical cytosolic protein
					16	58	16.0000	81.1078	

Log$_2$ Ratios: -6 -4 -2 0 2 4 6

Show detected proteins only
Show all proteins
Filter by category:
GO: amino acid transport

Proteins found: 1358

Enter (or paste) list of ORFs

Find ORFs

Test	Cutoff
q-Value	.005
p-Value	

	Signif	Direction	Applies To
red	yes	+	ratios, bars
yellow	no	n/a	bars
green	yes	−	ratios, bars
pink	yes	+	*p*- , *q*-Values
light green	yes	−	

Dot Plots Dot Plots

Protein	*FnSg* vs *Fn* Log$_2$ Ratio	Log$_2$ Sum	*q*-Value	*p*-Value	Raw *FnSg*	Raw *Fn*	Normalized *FnSg*	Normalized *Fn*	Description
FN0458	0.808	5.266	1.363e-2	6.682e-2	8	4	10.4129	5.1796	AAL94654.1| Hypothetical Exported Protein
					6	3	6.0000	4.1952	
FN0459						8		10.3592	AAL94655.1| Hypothetical exported 24-amino acid repeat protein
						8		11.1873	
FN0460	-2.267	9.924	2.218e-5	1.897e-5	8	57	10.4129	73.8093	AAL94656.1| Delta-aminolevulinic acid dehydratase
					18	45	18.0000	62.9284	
FN0461	-0.447	16.045	3.707e-5	3.893e-5	177	227	230.3861	293.9424	AAL94657.1| Probable sigma(54) modulation protein
					215	224	215.0000	313.2438	
FN0462	1.070	12.379	5.154e-3	2.142e-2	98	40	127.5584	51.7960	AAL94658.1| DNA mismatch repair protein mutL
					84	35	84.0000	48.9443	
FN0465	-0.612	15.596	3.061e-3	1.151e-2	166	195	216.0683	252.5055	AAL94661.1| Hypothetical protein
					144	213	144.0000	297.8613	
FN0466	0.347	17.710	9.443e-3	4.369e-2	446	323	580.5210	418.2528	AAL94662.1| Lysyl-tRNA synthetase
					464	288	464.0000	402.7420	
FN0469									AAL94665.1| Copper homeostasis protein cutC
					4		4.0000		
FN0470	-0.411	18.091	3.507e-3	1.366e-2	310	430	403.5011	556.8071	AAL94666.1| Putative efflux pump component MtrF
					513	473	513.0000	661.4478	
FN0472	1.030	23.996	1.242e-7	7.722e-9	4554	2271	5927.5618	2940.7185	AAL94668.1| Flavodoxin
					5764	1990	5764.0000	2782.8353	
FN0474	-0.688	5.449			4		5.2065		AAL94670.1| Acriflavin resistance protein B
						6		8.3905	
FN0475	-1.090	11.110	3.04e-4	6.535e-4	28	53	36.4453	68.6297	AAL94671.1| MIAB protein
					28	49	28.0000	68.5221	
FN0476	0.309	12.427	8.648e-3	3.932e-2	57	49	74.1921	63.4501	AAL94672.1| Transcription termination factor rho
					91	50	91.0000	69.9205	
FN0477	-1.264	11.449	3.036e-6	9.079e-7	24	65	31.2388	84.1685	AAL94673.1| Cell wall endopeptidase family M23/M37
					37	57	37.0000	79.7094	
FN0478	1.215	8.853	5.409e-8	1.765e-9	25	11	32.5404	14.2439	AAL94674.1| GcpE protein
					33	10	33.0000	13.9841	
FN0479					9		11.7145		AAL94675.1| RNA polymerase sigma-E factor
					25		25.0000		

Log$_2$ Ratios: -6 -4 -2 0 2 4 6

Show detected proteins only
Show all proteins
Filter by category:
GO: amino acid transport

Proteins found: 1358

Enter (or paste) list of ORFs

Find ORFs

Test	Cutoff
q-Value	.005
p-Value	

Signif	Direction	Applies To
yes	+	ratios, bars
no	n/a	bars
yes	−	ratios, bars
yes	+	*p*- , *q*-Values
yes	−	

Dot Plots Dot Plots

Protein	*FnSg* vs *Fn* Log_2 Ratio	Log_2 Sum	*q*-Value	*p*-Value	Raw *FnSg*	Raw *Fn*	Normalized *FnSg*	Normalized *Fn*	Description	Log_2 Ratios (-6 -4 -2 0 2 4 6)
FN0480									AAL94676.1| unknown	
					4		4.0000			
FN0481	-1.008	7.489	1.335e-2	6.525e-2	3	11	3.9048	14.2439	AAL94677.1| unknown	
					15	17	15.0000	23.7730		
FN0482						44		56.9756	AAL94678.1| LSU ribosomal protein L31P	
						22		30.7650		
FN0483	-1.286	13.140	8.803e-6	4.795e-6	52	120	67.6841	155.3880	AAL94679.1| Uracil phosphoribosyltransferase	
					54	101	54.0000	141.2394		
FN0484	-0.185	6.561			7	8	9.1113	10.3592	AAL94680.1| Lipase	
FN0487	-0.012	20.576	1.472e-1	9.17e-1	1078	933	1403.1427	1208.1419	AAL94683.1| 2-hydroxyglutarate dehydrogenase	
					1087	932	1087.0000	1303.3179		
FN0488	0.468	23.904	5.984e-3	2.547e-2	4005	2530	5212.9743	3276.0976	AAL94684.1| NAD-specific glutamate dehydrogenase	
					4107	2474	4107.0000	3459.6656		
FN0489	-1.567	7.907				25		32.3725	AAL94685.1| Prolipoprotein diacylglyceryl transferase	
					9	15	9.0000	20.9761		
FN0490									AAL94686.1| Integral membrane protein	
						4		5.5936		
FN0491	1.725	13.527	1.111e-3	3.377e-3	123	61	160.0988	78.9889	AAL94687.1| Alanine racemase	
					235	29	235.0000	40.5539		
FN0493	-0.771	8.440	8.499e-3	3.853e-2	15	16	19.5242	20.7184	AAL94689.1| Hypothetical protein	
					9	20	9.0000	27.9682		
FN0494	-2.331	16.329	9.05e-6	5.001e-6	69	464	89.8115	600.8337	AAL94690.1| Short chain dehydrogenase	
					166	491	166.0000	686.6192		
FN0495	0.783	25.644	2.586e-6	6.78e-7	7402	4420	9634.5658	5723.4591	AAL94691.1| Acetyl-CoA acetyltransferase	
					9364	3801	9364.0000	5315.3553		
FN0496									AAL94692.1| unknown	
						6		8.3905		
FN0501	1.084	17.363	2.303e-4	4.551e-4	420	199	546.6790	257.6851	AAL94697.1| Ornithine decarboxylase	
					649	219	649.0000	306.2517		
FN0502	1.003	8.044				8		10.3592	AAL94698.1| Phosphoheptose isomerase	
					23	9	23.0000	12.5857		

Show detected proteins only
Show all proteins
Filter by category:
GO: amino acid transport

Proteins found: 1358

Enter (or paste) list of ORFs

Find ORFs

Test	Cutoff
q-Value	.005
p-Value	

	Signif	Direction	Applies To
red	yes	+	ratios, bars
yellow	no	n/a	bars
green	yes	−	ratios, bars
pink	yes	+	*p*-, *q*-Values
light green	yes	−	

Dot Plots Dot Plots

Protein	FnSg vs Fn Log2 Ratio	Log2 Sum	q-Value	p-Value	Raw FnSg	Raw Fn	Normalized FnSg	Normalized Fn	Description
FN0503	-0.224	11.590	8.655e-3	3.936e-2	39	43	50.7630	55.6807	AAL94699.1| Transcriptional regulatory protein, LYSR family
					52	46	52.0000	64.3268	
FN0505	-0.293	10.222	3.952e-2	2.162e-1	18	31	23.4291	40.1419	AAL94701.1| Anthranilate synthase component II
					39	26	39.0000	36.3587	
FN0506	-1.284	14.532	3.941e-5	4.277e-5	67	182	87.2083	235.6718	AAL94702.1| Arginyl-tRNA synthetase
					110	175	110.0000	244.7217	
FN0511	0.593	11.550	2.722e-3	9.935e-3	58	30	75.4938	38.8470	AAL94707.1| D-lactate dehydrogenase
					59	36	59.0000	50.3427	
FN0512	0.226	15.475	1.275e-3	3.995e-3	171	145	222.5764	187.7605	AAL94708.1| Flavoprotein
					239	148	239.0000	206.9646	
FN0513	-1.158	9.508	5.895e-4	1.519e-3	17	32	22.1275	41.4368	AAL94709.1| Flavodoxin
					14	28	14.0000	39.1555	
FN0515	0.321	5.532	6.431e-2	3.652e-1	4	4	5.2065	5.1796	AAL94711.1| Acriflavin resistance protein D
					10	5	10.0000	6.9920	
FN0517					10		13.0162		AAL94713.1| Outer membrane protein tolC
					8		8.0000		
FN0519	-0.386	6.680	6.403e-2	3.635e-1	9	6	11.7145	7.7694	AAL94715.1| Hypothetical exported 24-amino acid repeat protein
					6	11	6.0000	15.3825	
FN0522	1.464	6.626	2.139e-3	7.388e-3	10	6	13.0162	7.7694	AAL94718.1| Exonuclease SBCC
					20	3	20.0000	4.1952	
FN0523	-1.612	7.522	1.603e-3	5.249e-3	5	15	6.5081	19.4235	AAL94719.1| Exonuclease SBCD
					9	20	9.0000	27.9682	
FN0524					5		6.5081		AAL94720.1| DNA helicase II
					12		12.0000		
FN0525	-0.149	15.969	6.84e-2	3.902e-1	219	196	285.0540	253.8004	AAL94721.1| Penicillin-binding protein
					196	200	196.0000	279.6819	
FN0526	0.275	11.555	8.998e-3	4.123e-2	42	36	54.6679	46.6164	AAL94722.1| Florfenicol resistance protein
					66	38	66.0000	53.1396	
FN0527	1.118	10.139	1.279e-3	4.01e-3	33	19	42.9533	24.6031	AAL94723.1| Alanyl-tRNA synthetase
					56	15	56.0000	20.9761	
FN0528	-5.943	15.825	2.971e-6	8.705e-7	28	1436	36.4453	1859.4768	AAL94724.1| Cold shock protein
					25	1373	25.0000	1920.0165	

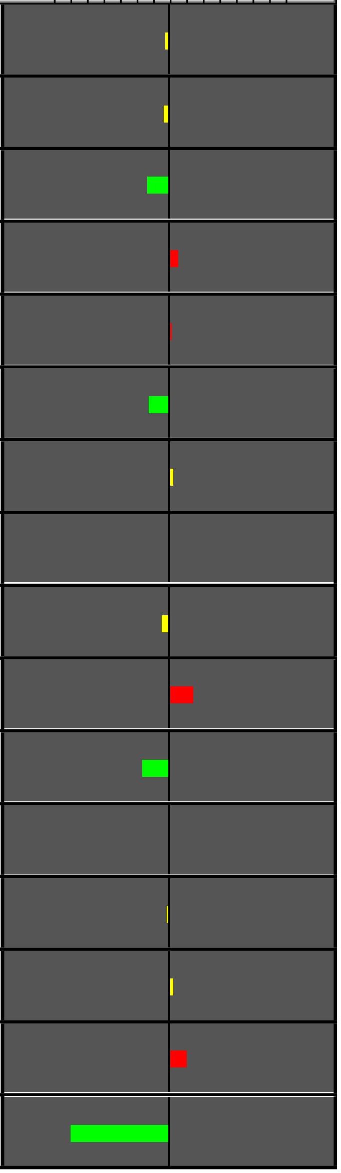

Show detected proteins only
Show all proteins
Filter by category:
GO: amino acid transport

Proteins found: 1358

Enter (or paste) list of ORFs
Find ORFs

Test	Cutoff
q-Value	.005
p-Value	

	Signif	Direction	Applies To
red	yes	+	ratios, bars
yellow	no	n/a	bars
green	yes	–	ratios, bars
pink	yes	+	p-, q-Values
light green	yes	–	p-, q-Values

Dot Plots Dot Plots

Protein	FnSg vs Fn Log$_2$ Ratio	Log$_2$ Sum	*q*-Value	*p*-Value	Raw *FnSg*	Raw *Fn*	Normalized *FnSg*	Normalized *Fn*	Description
FN0535	-0.144	10.016	6.605e-2	3.758e-1	24	22	31.2388	28.4878	AAL94731.1| Hypothetical protein
					30	28	30.0000	39.1555	
FN0536	-0.290	15.482	2.938e-3	1.093e-2	159	184	206.9570	238.2616	AAL94732.1| DNA polymerase III, beta chain
					180	168	180.0000	234.9328	
FN0540	-0.525	11.624	7.07e-4	1.932e-3	32	49	41.6517	63.4501	AAL94736.1| Glutamate-1-semialdehyde 2,1-aminomutase
					52	51	52.0000	71.3189	
FN0541	-0.612	7.079	5.117e-2	2.862e-1	6	6	7.8097	7.7694	AAL94737.1| polysaccharide deacetylase
					11	15	11.0000	20.9761	
FN0542	1.042	9.148	2.806e-3	1.032e-2	31	17	40.3501	22.0133	AAL94738.1| Beta 1,4 glucosyltransferase
					28	8	28.0000	11.1873	
FN0543	-1.605	9.994	5.674e-6	2.34e-6	12	45	15.6194	58.2705	AAL94739.1| Lipopolysaccharide heptosyltransferase-1
					21	38	21.0000	53.1396	
FN0546						6		7.7694	AAL94742.1| Lipopolysaccharide core biosynthesis protein rfaQ
FN0547	-0.434	17.816	9.961e-5	1.555e-4	304	425	395.6914	550.3326	AAL94743.1| RecA protein
					431	405	431.0000	566.3559	
FN0549	1.632	5.769							AAL94745.1| O-sialoglycoprotein endopeptidase
					13	3	13.0000	4.1952	
FN0550					12		15.6194		AAL94746.1| hypothetical Protein
					10		10.0000		
FN0552	0.596	8.994	2.955e-2	1.574e-1	15	10	19.5242	12.9490	AAL94748.1| Serine racemase
					36	17	36.0000	23.7730	
FN0553	1.235	14.471	3.442e-3	1.334e-2	144	75	187.4328	97.1175	AAL94749.1| D-serine dehydratase
					275	71	275.0000	99.2871	
FN0554	1.980	6.947			17		22.1275		AAL94750.1| D-serine permease
					22	4	22.0000	5.5936	
FN0555					6		7.8097		AAL94751.1| Transcriptional regulator, MerR family
FN0556	-0.711	11.901	3.251e-4	7.142e-4	42	65	54.6679	84.1685	AAL94752.1| unknown
					42	53	42.0000	74.1157	
FN0557	2.218	13.666	4.033e-3	1.624e-2	242	46	314.9912	59.5654	AAL94753.1| unknown
					177	33	177.0000	46.1475	

Log$_2$ Ratios

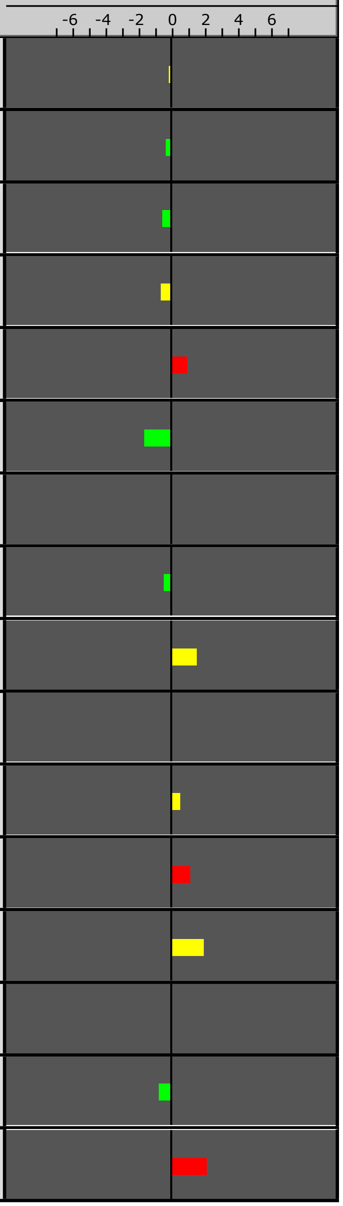

Show detected proteins only
Show all proteins
Filter by category:
GO: amino acid transport

Proteins found: 1358

Enter (or paste) list of ORFs

Find ORFs

Test	Cutoff
q-Value	.005
p-Value	

	Signif	Direction	Applies To
red	yes	+	ratios, bars
yellow	no	n/a	bars
green	yes	–	ratios, bars
pink	yes	+	*p*- , *q*-Values
light green	yes	–	

Dot Plots Dot Plots

Protein	*FnSg vs Fn* Log$_2$ Ratio	Log$_2$ Sum	*q*-Value	*p*-Value	Raw *FnSg*	Raw *Fn*	Normalized *FnSg*	Normalized *Fn*	Description
FN0558	0.685	12.886	1.435e-2	7.097e-2	105	52	136.6697	67.3348	AAL94754.1| TraT complement resistance protein precursor
					84	50	84.0000	69.9205	
FN0559	0.719	14.477	4.336e-6	1.524e-6	151	90	196.5441	116.5410	AAL94755.1| Phosphoglucomutase
					191	85	191.0000	118.8648	
FN0560									AAL94756.1| Oxygen-independent coproporphyrinogen III oxidase
					6		6.0000		
FN0561	-0.899	10.531	3.948e-5	4.288e-5	21	39	27.3339	50.5011	AAL94757.1| Proline synthetase associated protein
					29	39	29.0000	54.5380	
FN0562	-0.432	13.848	6.443e-3	2.779e-2	93	98	121.0503	126.9002	AAL94758.1| Hypothetical cytosolic protein
					88	111	88.0000	155.2235	
FN0563	-1.280	8.291	6.724e-8	3.251e-9	9	21	11.7145	27.1929	AAL94759.1| putative tRNA (5-methylaminomethyl-2-thiouridylate) -methyltransferase
					11	20	11.0000	27.9682	
FN0574						3		3.8847	AAL94770.1| Hypothetical cytosolic protein
FN0576	0.165	12.661	8.411e-2	4.88e-1	81	58	105.4309	75.1042	AAL94772.1| hypothetical protein
					65	55	65.0000	76.9125	
FN0577	1.419	6.387			13		16.9210		AAL94773.1| Hypothetical protein
					13	4	13.0000	5.5936	
FN0579	0.552	18.117	1.224e-2	5.897e-2	588	341	765.3505	441.5610	AAL94775.1| Hypothetical cytosolic protein
					526	314	526.0000	439.1006	
FN0580	-1.525	8.325	2.408e-4	4.815e-4	7	21	9.1113	27.1929	AAL94776.1| Penicillin-binding protein
					12	24	12.0000	33.5618	
FN0581	-0.356	8.287			12	19	15.6194	24.6031	AAL94777.1| Lipoprotein releasing system transmembrane protein lolE
						11		15.3825	
FN0582	-0.387	11.018	1.503e-2	7.478e-2	32	34	41.6517	44.0266	AAL94778.1| Lipoprotein releasing system ATP-binding protein lolD
					38	43	38.0000	60.1316	
FN0583	0.411	6.772			7	7	9.1113	9.0643	AAL94779.1| Hypothetical Exported Protein
					15		15.0000		
FN0585	0.034	6.524	1.367e-1	8.413e-1	8	6	10.4129	7.7694	AAL94781.1| Two-component response regulator czcR
					9	8	9.0000	11.1873	
FN0586	0.459	6.286	5.033e-2	2.811e-1	9	3	11.7145	3.8847	AAL94782.1| Two-component sensor kinase czcS
					9	8	9.0000	11.1873	

Log$_2$ Ratios: -6 -4 -2 0 2 4 6

Show detected proteins only
Show all proteins
Filter by category:
GO: amino acid transport

Proteins found: 1358

Enter (or paste) list of ORFs

Find ORFs

Test	Cutoff
q-Value	.005
p-Value	

	Signif	Direction	Applies To
red	yes	+	ratios, bars
yellow	no	n/a	bars
green	yes	−	ratios, bars
pink	yes	+	*p*- , *q*-Values
light green	yes	−	

Dot Plots Dot Plots

Protein	*FnSg vs Fn* Log$_2$ Ratio	Log$_2$ Sum	*q*-Value	*p*-Value	Raw *FnSg*	Raw *Fn*	Normalized *FnSg*	Normalized *Fn*	Description	Log$_2$ Ratios (-6 -4 -2 0 2 4 6)
**FN0590**	1.373	9.254	1.808e-3	6.042e-3	25	14	32.5404	18.1286	AAL94786.1| N-acyl-L-amino acid amidohydrolase	
					47	9	47.0000	12.5857		
**FN0592**	-0.258	13.368	5.754e-2	3.244e-1	93	84	121.0503	108.7716	AAL94788.1| ATP-dependent DNA helicase pcrA	
					67	83	67.0000	116.0680		
**FN0593**	-0.312	10.969	3.579e-3	1.403e-2	31	36	40.3501	46.6164	AAL94789.1| UDP-3-O-[3-hydroxymyristoyl] N-acetylglucosamine deacetylase	
					40	38	40.0000	53.1396		
**FN0594**	3.705	10.066			91	7	118.4471	9.0643	AAL94790.1| (3R)-hydroxymyristoyl-[acyl carrier protein] dehydratase	
					118		118.0000			
**FN0595**	-0.840	8.012	5.036e-5	6.152e-5	10	17	13.0162	22.0133	AAL94791.1| Acyl-[acyl-carrier-protein]-UDP-N-acetylglucosamine O-acyltransferase	
					11	15	11.0000	20.9761		
**FN0596**	-0.667	9.548	3.358e-3	1.293e-2	18	23	23.4291	29.7827	AAL94792.1| Hypothetical protein	
					20	28	20.0000	39.1555		
**FN0597**	-0.322	10.315	2.133e-2	1.099e-1	26	26	33.8420	33.6674	AAL94793.1| Lipid-A-disaccharide synthase	
					30	33	30.0000	46.1475		
**FN0598**	-0.762	9.422	2.596e-3	9.369e-3	14	30	18.2226	38.8470	AAL94794.1| Phospholipid-lipopolysaccharide ABC transporter	
					22	21	22.0000	29.3666		
**FN0600**	-0.004	12.761	1.483e-1	9.256e-1	61	63	79.3986	81.5787	AAL94796.1| Hypothetical protein	
					87	61	87.0000	85.3030		
**FN0601**						31		40.1419	AAL94797.1| Hypothetical exported 24-amino acid repeat protein	
						27		37.7571		
**FN0602**	-1.050	13.985	1.203e-5	7.965e-6	63	148	82.0018	191.6452	AAL94798.1| Hypothetical protein	
					95	125	95.0000	174.8012		
**FN0603**	1.234	6.202			11		14.3178		AAL94799.1| Transcriptional regulatory protein, LYSR family	
					12	4	12.0000	5.5936		
**FN0608**	1.631	12.861	3.087e-8	6.398e-10	118	39	153.5908	50.5011	AAL94804.1| Exoribonuclease II	
					150	34	150.0000	47.5459		
**FN0609**									AAL94805.1| Small protein B	
					11		11.0000			
**FN0610**	-0.706	15.862	7.534e-6	3.747e-6	153	248	199.1473	321.1353	AAL94806.1| unknown	
					183	216	183.0000	302.0565		
**FN0611**	0.328	19.160	3.215e-3	1.223e-2	612	541	796.5893	700.5410	AAL94807.1| Threonyl-tRNA synthetase	
					918	476	918.0000	665.6430		

Show detected proteins only
Show all proteins
Filter by category:
GO: amino acid transport

Proteins found: 1358

Enter (or paste) list of ORFs

Find ORFs

Test	Cutoff
q-Value	.005
p-Value	

	Signif	Direction	Applies To
red	yes	+	ratios, bars
yellow	no	n/a	bars
green	yes	−	ratios, bars
pink	yes	+	*p*- , *q*-Values
light green	yes	−	*p*- , *q*-Values

Dot Plots Dot Plots

FnSg vs Fn

Fusobacterium nucleatum

Hackett Laboratory UW

Fn Summary Table | FnPg vs Fn | FnSg vs Fn | FnPgSg vs Fn | FnPgSg vs FnPg | FnSg vs FnPg | FnPgSg vs FnSg | Fn Coverage

Protein	FnSg vs Fn Log$_2$ Ratio	Log$_2$ Sum	*q*-Value	*p*-Value	Raw *FnSg*	Raw *Fn*	Normalized *FnSg*	Normalized *Fn*	Description
FN0612	-1.557	13.116	7.648e-4	2.154e-3	36	107	46.8582	138.5543	AAL94808.1| Hypothetical protein
					63	132	63.0000	184.5901	
FN0614									AAL94810.1| Export ABC transporter
					3		3.0000		
FN0616						53		68.6297	AAL94812.1| Hypothetical protein
						41		57.3348	
FN0617	-1.376	14.679	5.643e-6	2.319e-6	73	200	95.0180	258.9800	AAL94813.1| DNA polymerase III, beta chain
					106	188	106.0000	262.9010	
FN0618	-0.791	13.948	2.997e-3	1.121e-2	90	128	117.1455	165.7472	AAL94814.1| Spermidine/putrescine-binding protein
					74	118	74.0000	165.0123	
FN0619	-1.120	8.589	4.304e-4	1.011e-3	12	22	15.6194	28.4878	AAL94815.1| Small-conductance mechanosensitive channel
					11	21	11.0000	29.3666	
FN0621	-0.505	11.363	2.115e-3	7.286e-3	37	48	48.1598	62.1552	AAL94817.1| 4-hydroxybutyrate coenzyme A transferase
					38	43	38.0000	60.1316	
FN0622	3.782	9.284	1.265e-3	3.955e-3	57	5	74.1921	6.4745	AAL94818.1| 8-oxoguanine DNA glycosylase
					111	5	111.0000	6.9920	
FN0625	-0.989	8.803				19		24.6031	AAL94821.1| Aspartate aminotransferase
					15	25	15.0000	34.9602	
FN0626									AAL94822.1| Hypothetical cytosolic protein
					12		12.0000		
FN0627	-0.867	14.665	2.127e-4	4.124e-4	95	158	123.6536	204.5942	AAL94823.1| Glucosamine--fructose-6-phosphate aminotransferase (isomerizing)
					115	165	115.0000	230.7376	
FN0628	-0.018	5.529	1.536e-1	9.644e-1	5	3	6.5081	3.8847	AAL94824.1| Glucosamine--fructose-6-phosphate aminotransferase (isomerizing)
					7	7	7.0000	9.7889	
FN0629	0.344	9.031	4.925e-2	2.745e-1	25	13	32.5404	16.8337	AAL94825.1| PTS system, IID component
					19	17	19.0000	23.7730	
FN0630									AAL94826.1| PTS system, IIC component
						11		15.3825	
FN0631	-0.404	8.623	5.102e-3	2.117e-2	15	18	19.5242	23.3082	AAL94827.1| PTS system, IIB component
					15	16	15.0000	22.3746	
FN0633	0.057	6.916	7.709e-2	4.439e-1	8	8	10.4129	10.3592	AAL94829.1| Replication protein
					12	8	12.0000	11.1873	

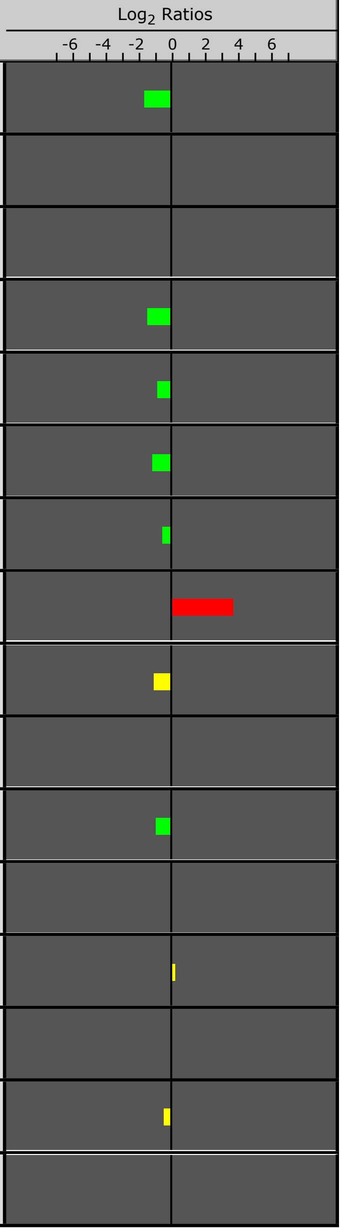

Show detected proteins only
Show all proteins
Filter by category:
GO: amino acid transport

Proteins found: 1358

Enter (or paste) list of ORFs

Find ORFs

Test	Cutoff
q-Value	.005
p-Value	

	Signif	Direction	Applies To
red	yes	+	ratios, bars
yellow	no	n/a	bars
green	yes	–	ratios, bars
pink	yes	+	*p*- , *q*-Values
light green	yes	–	

Dot Plots Dot Plots

Protein	*FnSg* vs *Fn* Log$_2$ Ratio	Log$_2$ Sum	*q*-Value	*p*-Value	Raw *FnSg*	Raw *Fn*	Normalized *FnSg*	Normalized *Fn*	Description
FN0634	-2.139	12.536	1.339e-4	2.301e-4	28	117	36.4453	151.5033	AAL94830.1| GTP-binding protein TypA/BipA
					37	123	37.0000	172.0044	
FN0636						6		7.7694	AAL94832.1| Hypothetical protein
FN0637						6		7.7694	AAL94833.1| Hypothetical exported 24-amino acid repeat protein
						7		9.7889	
FN0643	0.129	8.587	1.19e-1	7.175e-1	10	16	13.0162	20.7184	AAL94839.1| hypothetical DNA-binding protein
					28	12	28.0000	16.7809	
FN0644	-0.635	10.907	3.776e-3	1.502e-2	21	39	27.3339	50.5011	AAL94840.1| Uroporphyrin-III C-methyltransferase
					43	42	43.0000	58.7332	
FN0645	-0.851	6.021				7		9.0643	AAL94841.1| Porphobilinogen deaminase
					6	9	6.0000	12.5857	
FN0646									AAL94842.1| Glutamyl-tRNA reductase
					3		3.0000		
FN0651									AAL94847.1| Ribosomal large subunit pseudouridine synthase D
					6		6.0000		
FN0652	1.685	24.463	5.153e-4	1.277e-3	5960	2050	7757.6347	2654.5455	AAL94848.1| Glyceraldehyde 3-phosphate dehydrogenase
					9493	1937	9493.0000	2708.7196	
FN0653	0.939	12.582	4.025e-3	1.62e-2	69	42	89.8115	54.3858	AAL94849.1| unknown
					127	42	127.0000	58.7332	
FN0654	0.924	19.929	7.066e-4	1.93e-3	1156	532	1504.6687	688.8869	AAL94850.1| Phosphoglycerate kinase
					1248	545	1248.0000	762.1333	
FN0655	-2.362	10.514	8.012e-4	2.282e-3	19	54	24.7307	69.9246	AAL94851.1| unknown
					9	74	9.0000	103.4823	
FN0656						64		82.8736	AAL94852.1| Hypothetical protein
						76		106.2791	
FN0657	2.260	9.346			42	9	54.6679	11.6541	AAL94853.1| Acetyltransferase
					57		57.0000		
FN0658	-1.617	13.256	5.919e-5	7.705e-5	43	139	55.9695	179.9911	AAL94854.1| ABC transporter substrate-binding protein
					57	119	57.0000	166.4108	
FN0660	-0.733	6.770	1.002e-2	4.685e-2	4	10	5.2065	12.9490	AAL94856.1| ABC transporter ATP-binding protein
					11	10	11.0000	13.9841	

Show detected proteins only
Show all proteins
Filter by category:
GO: amino acid transport

Proteins found: 1358

Enter (or paste) list of ORFs

Find ORFs

Test	Cutoff
q-Value	.005
p-Value	

	Signif	Direction	Applies To
red	yes	+	ratios, bars
yellow	no	n/a	bars
green	yes	–	ratios, bars
pink	yes	+	*p*- , *q*-Values
light green	yes	–	*p*- , *q*-Values

Dot Plots Dot Plots

Protein	*FnSg* vs *Fn* Log$_2$ Ratio	Log$_2$ Sum	*q*-Value	*p*-Value	Raw *FnSg*	Raw *Fn*	Normalized *FnSg*	Normalized *Fn*	Description
FN0662	0.581	11.423	6.587e-4	1.756e-3	47	37	61.1760	47.9113	AAL94858.1| Formiminoglutamase
					67	27	67.0000	37.7571	
FN0664	-0.313	14.662	9.048e-2	5.286e-1	46	126	59.8744	163.1574	AAL94860.1| 2-nitropropane dioxygenase
					229	140	229.0000	195.7774	
FN0666	-1.071	6.290	1.61e-4	2.956e-4	4	9	5.2065	11.6541	AAL94862.1| Hypothetical protein
					7	10	7.0000	13.9841	
FN0668	-0.869	9.550	1.004e-6	1.511e-7	15	28	19.5242	36.2572	AAL94864.1| High-affinity zinc uptake system protein znuA precursor
					21	27	21.0000	37.7571	
FN0672					17		22.1275		AAL94868.1| ATPase
					11		11.0000		
FN0675	1.552	22.124	6.365e-3	2.739e-2	2016	936	2624.0590	1212.0266	AAL94871.1| 60 kDa chaperonin GROEL
					4698	919	4698.0000	1285.1385	
FN0676	-2.415	13.229	3.02e-4	6.479e-4	36	193	46.8582	249.9157	AAL94872.1| 10 kDa chaperonin GROES
					38	145	38.0000	202.7694	
FN0677	-0.420	12.645	9.004e-4	2.623e-3	51	76	66.3824	98.4124	AAL94873.1| Hypothetical protein
					72	62	72.0000	86.7014	
FN0678	-0.741	12.419	6.85e-5	9.561e-5	48	70	62.4776	90.6430	AAL94874.1| Ser/Thr protein kinase
					52	72	52.0000	100.6855	
FN0679	-1.004	7.344				16		20.7184	AAL94875.1| GTPase
					9	11	9.0000	15.3825	
FN0680									AAL94876.1| Ribulose-phosphate 3-epimerase
					13		13.0000		
FN0681	-0.045	16.507	1.015e-1	6.006e-1	212	227	275.9427	293.9424	AAL94877.1| Transcriptional regulator, MarR family
					325	233	325.0000	325.8295	
FN0682	0.352	9.756	1.795e-2	9.098e-2	28	24	36.4453	31.0776	AAL94878.1| Fibronectin-binding protein-like protein A
					30	15	30.0000	20.9761	
FN0684					16		20.8259		AAL94880.1| Prismane protein
					8		8.0000		
FN0685	1.229	11.493	8.405e-3	3.802e-2	81	25	105.4309	32.3725	AAL94881.1| Sodium/pantothenate symporter
					59	27	59.0000	37.7571	
FN0688	0.481	11.908	1.35e-4	2.328e-4	58	40	75.4938	51.7960	AAL94884.1| Hypothetical protein
					71	38	71.0000	53.1396	

Log$_2$ Ratios: -6 -4 -2 0 2 4 6

Show detected proteins only
Show all proteins
Filter by category:
GO: amino acid transport

Proteins found: 1358

Enter (or paste) list of ORFs

Find ORFs

Test	Cutoff
q-Value	.005
p-Value	

	Signif	Direction	Applies To
red	yes	+	ratios, bars
yellow	no	n/a	bars
green	yes	−	ratios, bars
pink	yes	+	*p*- , *q*-Values
light green	yes	−	

Dot Plots Dot Plots

Protein	*FnSg* vs *Fn* Log$_2$ Ratio	Log$_2$ Sum	*q*-Value	*p*-Value	Raw *FnSg*	Raw *Fn*	Normalized *FnSg*	Normalized *Fn*	Description
FN0689	-0.705	15.913	7.982e-3	3.579e-2	196	234	255.1168	303.0067	AAL94885.1| Hypothetical protein
					134	237	134.0000	331.4231	
FN0692	-1.624	7.070	3.245e-3	1.238e-2	4	12	5.2065	15.5388	AAL94888.1| Nitrogen regulation protein NIFR3
					8	18	8.0000	25.1714	
FN0693						21		27.1929	AAL94889.1| DNA mismatch repair protein mutS
						32		44.7491	
FN0694	-0.540	12.624	2.427e-3	8.634e-3	49	67	63.7792	86.7583	AAL94890.1| S-layer protein
					68	75	68.0000	104.8807	
FN0695	0.494	10.669	8.939e-3	4.09e-2	39	32	50.7630	41.4368	AAL94891.1| ABC transporter ATP-binding protein
					45	19	45.0000	26.5698	
FN0697	-0.654	15.441	2.525e-4	5.114e-4	117	210	152.2891	271.9291	AAL94893.1| Alanyl-tRNA synthetase
					184	184	184.0000	257.3074	
FN0699	-2.538	11.782	4.124e-5	4.597e-5	24	101	31.2388	130.7849	AAL94895.1| Protein translocase subunit secD
					18	111	18.0000	155.2235	
FN0700	-3.208	9.117	4.85e-4	1.179e-3	5	48	6.5081	62.1552	AAL94896.1| Protein translocase subunit secF
					9	58	9.0000	81.1078	
FN0701	-0.888	14.791	1.253e-5	8.483e-6	88	182	114.5423	235.6718	AAL94897.1| Methyltransferase
					133	159	133.0000	222.3471	
FN0705	-0.554	13.935	1.123e-4	1.815e-4	75	123	97.6212	159.2727	AAL94901.1| DNA polymerase I
					109	103	109.0000	144.0362	
FN0706	-1.443	5.443				6		7.7694	AAL94902.1| Hypothetical cytosolic protein
					4	10	4.0000	13.9841	
FN0707	-1.677	7.767	9.49e-4	2.796e-3	5	17	6.5081	22.0133	AAL94903.1| Riboflavin kinase
					10	22	10.0000	30.7650	
FN0710	-0.551	11.934	2.344e-3	8.271e-3	41	64	53.3663	82.8736	AAL94906.1| Hypothetical protein
					50	49	50.0000	68.5221	
FN0711	1.073	8.284	1.013e-2	4.741e-2	14	8	18.2226	10.3592	AAL94907.1| Phosphopantothenate--cysteine ligase
					33	10	33.0000	13.9841	
FN0714					8		10.4129		AAL94910.1| NADH oxidase
					16		16.0000		
FN0715	0.029	14.392	9.823e-2	5.789e-1	120	113	156.1940	146.3237	AAL94911.1| Hypothetical protein
					140	103	140.0000	144.0362	

Log$_2$ Ratios

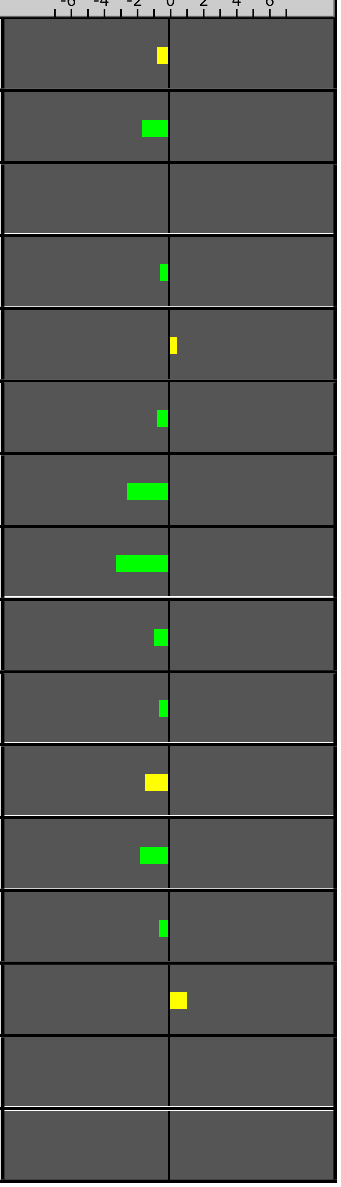

Show detected proteins only
Show all proteins
Filter by category:
GO: amino acid transport

Proteins found: 1358

Enter (or paste) list of ORFs

Find ORFs

Test	Cutoff
q-Value	.005
p-Value	

	Signif	Direction	Applies To
red	yes	+	ratios, bars
yellow	no	n/a	bars
green	yes	−	ratios, bars
pink	yes	+	*p*- , *q*-Values
light green	yes	−	

Dot Plots Dot Plots

Protein	*FnSg* vs *Fn* Log$_2$ Ratio	Log$_2$ Sum	*q*-Value	*p*-Value	Raw *FnSg*	Raw *Fn*	Normalized *FnSg*	Normalized *Fn*	Description
**FN0716**	-0.235	12.221	2.347e-2	1.221e-1	51	65	66.3824	84.1685	AAL94912.1| hypothetical protein
					61	47	61.0000	65.7253	
**FN0717**	-0.747	5.391							AAL94913.1| Ribosomal small subunit pseudouridine synthase A
					5	6	5.0000	8.3905	
**FN0719**						9		11.6541	AAL94915.1| Hypothetical cytosolic protein
						8		11.1873	
**FN0720**	-1.166	13.952	2.368e-3	8.377e-3	40	139	52.0647	179.9911	AAL94916.1| Protein Translation Elongation Factor P (EF-P)
					116	141	116.0000	197.1758	
**FN0721**	-1.317	11.714	9.684e-7	1.428e-7	28	70	36.4453	90.6430	AAL94917.1| Hypothetical protein
					37	66	37.0000	92.2950	
**FN0722**	-0.475	7.658	1.291e-2	6.278e-2	7	14	9.1113	18.1286	AAL94918.1| WD-repeat family protein
					15	11	15.0000	15.3825	
**FN0723**									AAL94919.1| Hypothetical protein
					5		5.0000		
**FN0724**	0.725	11.343	9.616e-3	4.466e-2	40	31	52.0647	40.1419	AAL94920.1| Flavodoxin
					79	28	79.0000	39.1555	
**FN0725**	1.581	8.595	3.629e-3	1.428e-2	20	10	26.0323	12.9490	AAL94921.1| Molybdopterin biosynthesis MoeB protein
					42	7	42.0000	9.7889	
**FN0728**	0.434	10.640	5.332e-4	1.337e-3	36	25	46.8582	32.3725	AAL94924.1| Hypothetical protein
					46	26	46.0000	36.3587	
**FN0729**	-1.268	14.581	6.792e-5	9.439e-5	92	197	119.7487	255.0953	AAL94925.1| Phosphoglycerate mutase
					82	165	82.0000	230.7376	
**FN0731**	-0.446	8.740	2.745e-2	1.451e-1	18	20	23.4291	25.8980	AAL94927.1| Hypothetical protein
					12	16	12.0000	22.3746	
**FN0733**	-1.280	12.632	1.505e-5	1.085e-5	34	100	44.2550	129.4900	AAL94929.1| Peptidase T
					58	85	58.0000	118.8648	
**FN0734**	-1.617	5.582	5.426e-5	6.813e-5	3	9	3.9048	11.6541	AAL94930.1| Fe-S oxidoreductase
					4	9	4.0000	12.5857	
**FN0735**	-1.818	12.063	7.361e-6	3.616e-6	32	99	41.6517	128.1951	AAL94931.1| Cell surface protein
					28	84	28.0000	117.4664	
**FN0736**					16		20.8259		AAL94932.1| Methyltransferase
					17		17.0000		

Log$_2$ Ratios: -6 -4 -2 0 2 4 6

Show detected proteins only
Show all proteins
Filter by category:
GO: amino acid transport

Proteins found: 1358

Enter (or paste) list of ORFs

Find ORFs

Test	Cutoff
q-Value	.005
p-Value	

	Signif	Direction	Applies To
red	yes	+	ratios, bars
yellow	no	n/a	bars
green	yes	−	ratios, bars
pink	yes	+	*p*- , *q*-Values
light green	yes	−	

Dot Plots Dot Plots

Protein	FnSg vs Fn Log$_2$ Ratio	FnSg vs Fn Log$_2$ Sum	FnSg vs Fn *q*-Value	FnSg vs Fn *p*-Value	Raw *FnSg*	Raw *Fn*	Normalized *FnSg*	Normalized *Fn*	Description
FN0738	-0.943	11.505	2.79e-3	1.024e-2	39	55	50.7630	71.2195	AAL94934.1| Hypothetical exported 24-amino acid repeat protein
					27	56	27.0000	78.3109	
FN0739	-0.733	15.416	5.84e-4	1.501e-3	144	196	187.4328	253.8004	AAL94935.1| Formiminotetrahydrofolate cyclodeaminase
					137	204	137.0000	285.2756	
FN0740	0.434	18.450	1.528e-2	7.616e-2	625	401	813.5104	519.2550	AAL94936.1| Imidazolonepropionase
					578	365	578.0000	510.4195	
FN0741	-0.038	18.686	1.211e-1	7.32e-1	551	525	717.1907	679.8226	AAL94937.1| Glutamate formiminotransferase
					565	455	565.0000	636.2764	
FN0742	-0.576	12.074	7.048e-3	3.095e-2	35	72	45.5566	93.2328	AAL94938.1| unknown
					62	48	62.0000	67.1237	
FN0743	-1.291	5.907	1.824e-4	3.424e-4	3	9	3.9048	11.6541	AAL94939.1| ATP-dependent helicase, DinG family
					6	9	6.0000	12.5857	
FN0745	-0.104	15.243	1.099e-1	6.559e-1	179	135	232.9894	174.8115	AAL94941.1| metal dependent phosphohydrolase
					147	167	147.0000	233.5344	
FN0746	0.239	7.325			5	9	6.5081	11.6541	AAL94942.1| Hypothetical Metal-Binding Protein
					21		21.0000		
FN0749						10		12.9490	AAL94945.1| Hypothetical protein
						6		8.3905	
FN0750	0.682	11.143	3.125e-5	3.036e-5	48	31	62.4776	40.1419	AAL94946.1| Hypothetical protein
					58	25	58.0000	34.9602	
FN0751	0.312	9.598	2.544e-2	1.334e-1	20	17	26.0323	22.0133	AAL94947.1| L-asparaginase I
					36	20	36.0000	27.9682	
FN0752	0.104	8.868	3.433e-2	1.859e-1	16	16	20.8259	20.7184	AAL94948.1| Proline iminopeptidase
					24	15	24.0000	20.9761	
FN0753	-0.116	12.044	8.057e-2	4.656e-1	46	44	59.8744	56.9756	AAL94949.1| Glutamyl-tRNA(Gln) amidotransferase subunit B
					65	56	65.0000	78.3109	
FN0754	-0.420	13.694	9.565e-3	4.437e-2	63	102	82.0018	132.0798	AAL94950.1| Glutamyl-tRNA(Gln) amidotransferase subunit A
					117	96	117.0000	134.2473	
FN0755	-1.054	11.105	7.568e-4	2.122e-3	27	58	35.1436	75.1042	AAL94951.1| Glutamyl-tRNA(Gln) amidotransferase subunit C
					30	43	30.0000	60.1316	
FN0758	-0.123	15.712	8.126e-2	4.7e-1	202	179	262.9265	231.7871	AAL94954.1| Rod shape-determining protein mreB
					181	180	181.0000	251.7137	

Log$_2$ Ratios

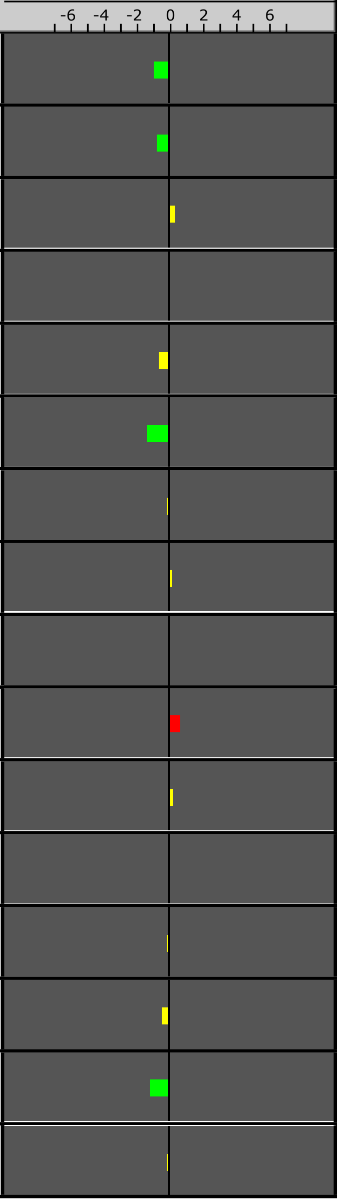

Show detected proteins only
Show all proteins
Filter by category:
GO: amino acid transport

Proteins found: 1358

Enter (or paste) list of ORFs

Find ORFs

Test	Cutoff
q-Value	.005
p-Value	

	Signif	Direction	Applies To
red	yes	+	ratios, bars
yellow	no	n/a	bars
green	yes	−	ratios, bars
light red	yes	+	*p*-, *q*-Values
light green	yes	−	

Dot Plots Dot Plots

Protein	FnSg vs Fn Log$_2$ Ratio	FnSg vs Fn Log$_2$ Sum	FnSg vs Fn *q*-Value	FnSg vs Fn *p*-Value	Raw *FnSg*	Raw *Fn*	Normalized *FnSg*	Normalized *Fn*	Description	Log$_2$ Ratios (-6 -4 -2 0 2 4 6)
FN0761	0.070	8.459	1.305e-1	7.978e-1	18	11	23.4291	14.2439	AAL94957.1| Bvg accessory factor	
					15	16	15.0000	22.3746		
FN0765	-2.020	5.190				8		10.3592	AAL94961.1| tRNA (5-methylaminomethyl -2-thiouridylate) -methyltransferase	
					3	10	3.0000	13.9841		
FN0766						15		19.4235	AAL94962.1| Large-conductance mechanosensitive channel	
						16		22.3746		
FN0768						10		12.9490	AAL94964.1| Hemin receptor	
						8		11.1873		
FN0771	-1.844	6.738			3		3.9048		AAL94967.1| Oxygen-independent coproporphyrinogen III oxidase	
					7	14	7.0000	19.5777		
FN0772						7		9.0643	AAL94968.1| Flavodoxin	
						3		4.1952		
FN0774	-3.087	12.161	1.752e-5	1.35e-5	18	148	23.4291	191.6452	AAL94970.1| Hypothetical cytosolic protein	
					23	145	23.0000	202.7694		
FN0775	-0.491	16.796	1.102e-4	1.771e-4	229	313	298.0702	405.3038	AAL94971.1| Aspartyl aminopeptidase	
					271	282	271.0000	394.3515		
FN0776	0.001	13.548	1.573e-1	9.918e-1	86	75	111.9390	97.1175	AAL94972.1| Aspartate-ammonia ligase	
					107	87	107.0000	121.6616		
FN0777	0.035	12.600	1.364e-1	8.395e-1	68	50	88.5099	64.7450	AAL94973.1| GTP-binding protein lepA	
					71	65	71.0000	90.8966		
FN0778	-0.442	9.755	5.398e-4	1.358e-3	18	27	23.4291	34.9623	AAL94974.1| Methyltransferase	
					27	24	27.0000	33.5618		
FN0779					6		7.8097		AAL94975.1| Putative GTPases (G3E family)	
					11		11.0000			
FN0783	-0.196	22.959	1.153e-2	5.503e-2	1871	2445	2435.3246	3166.0311	AAL94979.1| acyl-CoA dehydrogenase	
					2900	2107	2900.0000	2946.4493		
FN0784	-0.325	22.572	7.081e-3	3.113e-2	1910	2170	2486.0876	2809.9335	AAL94980.1| Electron transfer flavoprotein beta-subunit	
					1976	1988	1976.0000	2780.0385		
FN0785	-0.067	21.932	1.008e-1	5.956e-1	1307	1670	1701.2128	2162.4834	AAL94981.1| Electron transfer flavoprotein alpha-subunit	
					2208	1381	2208.0000	1931.2038		
FN0788	0.484	9.531	9.059e-3	4.156e-2	21	15	27.3339	19.4235	AAL94984.1| unknown	
					37	19	37.0000	26.5698		

Show detected proteins only
Show all proteins
Filter by category:
GO: amino acid transport

Proteins found: 1358

Enter (or paste) list of ORFs

Find ORFs

Test	Cutoff
q-Value	.005
p-Value	

	Signif	Direction	Applies To
red	yes	+	ratios, bars
yellow	no	n/a	bars
green	yes	−	ratios, bars
pink	yes	+	*p*- , *q*-Values
light green	yes	−	

Dot Plots Dot Plots

Protein	*FnSg vs Fn* Log$_2$ Ratio	Log$_2$ Sum	*q*-Value	*p*-Value	Raw *FnSg*	Raw *Fn*	Normalized *FnSg*	Normalized *Fn*	Description
FN0790	-0.605	7.860	1.985e-5	1.632e-5	9	15	11.7145	19.4235	AAL94986.1| Xylose repressor
					13	13	13.0000	18.1793	
FN0791	-0.247	18.614	2.21e-2	1.143e-1	513	558	667.7293	722.5543	AAL94987.1| Histidine ammonia-lyase
					495	470	495.0000	657.2526	
FN0792	0.238	20.822	5.849e-4	1.504e-3	1095	1002	1425.2701	1297.4900	AAL94988.1| Urocanate hydratase
					1532	865	1532.0000	1209.6244	
FN0793						17		22.0133	AAL94989.1| Sodium/glutamate symport carrier protein
						15		20.9761	
FN0794	1.117	7.976	1.338e-4	2.299e-4	19	8	24.7307	10.3592	AAL94990.1| Hypothetical protein
					22	8	22.0000	11.1873	
FN0796	-0.217	15.204	1.154e-3	3.536e-3	144	168	187.4328	217.5432	AAL94992.1| Pyruvate,phosphate dikinase
					173	144	173.0000	201.3710	
FN0798	1.292	8.982	2.66e-2	1.401e-1	41	6	53.3663	7.7694	AAL94994.1| Fructose-1,6-bisphosphatase
					17	15	17.0000	20.9761	
FN0799	0.442	8.575	8.966e-3	4.105e-2	15	14	19.5242	18.1286	AAL94995.1| Isoamylase
					26	11	26.0000	15.3825	
FN0800	0.133	10.934	4.685e-2	2.599e-1	32	35	41.6517	45.3215	AAL94996.1| Amino acid-binding protein
					51	28	51.0000	39.1555	
FN0801	0.906	8.105	6.488e-6	2.993e-6	18	9	23.4291	11.6541	AAL94997.1| Amino acid transport ATP-binding protein
					22	9	22.0000	12.5857	
FN0803	-1.679	12.233	1.802e-3	6.016e-3	25	114	32.5404	147.6186	AAL94999.1| Cytochrome C-TYPE biogenesis protein ccdA
					45	72	45.0000	100.6855	
FN0805	0.966	6.577			11		14.3178		AAL95001.1| Hypothetical protein
					13	5	13.0000	6.9920	
FN0806	-0.810	13.300	1.075e-3	3.245e-3	62	92	80.7002	119.1308	AAL95002.1| SpoIID homolog
					71	105	71.0000	146.8330	
FN0807	-1.467	9.175	3.587e-3	1.407e-2	13	38	16.9210	49.2062	AAL95003.1| 3-deoxy-manno-octulosonate cytidylyltransferase
					12	22	12.0000	30.7650	
FN0808	0.309	12.415	3.683e-3	1.456e-2	68	55	88.5099	71.2195	AAL95004.1| Phosphoglycerate mutase
					76	44	76.0000	61.5300	
FN0809	-0.780	8.780				23		29.7827	AAL95005.1| 23S rRNA methyltransferase
					16	18	16.0000	25.1714	

Log$_2$ Ratios

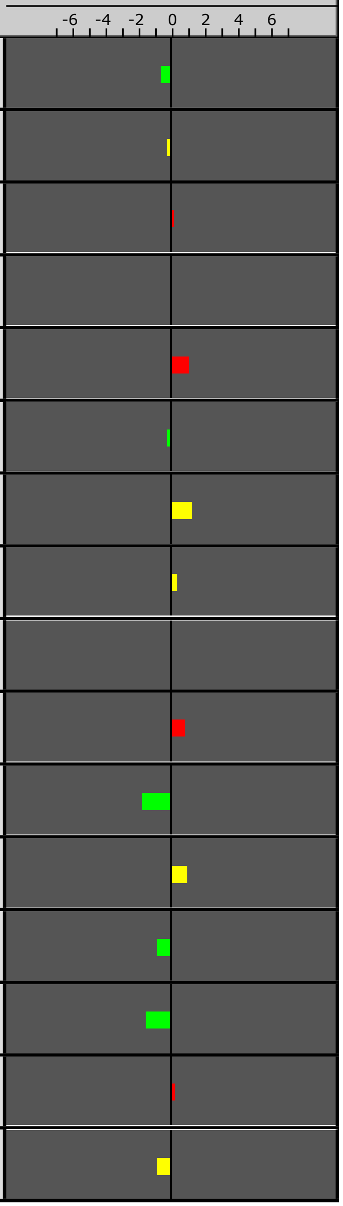

Show detected proteins only
Show all proteins
Filter by category:
GO: amino acid transport

Proteins found: 1358

Enter (or paste) list of ORFs

Find ORFs

Test	Cutoff
q-Value	.005
p-Value	

	Signif	Direction	Applies To
red	yes	+	ratios, bars
yellow	no	n/a	bars
green	yes	−	ratios, bars
pink	yes	+	*p*- , *q*-Values
light green	yes	−	

Dot Plots Dot Plots

Protein	FnSg vs Fn Log2 Ratio	Log2 Sum	q-Value	p-Value	Raw FnSg	Raw Fn	Normalized FnSg	Normalized Fn	Description
FN0810	0.027	11.872	1.541e-1	9.681e-1	12	44	15.6194	56.9756	AAL95006.1| Low-specificity threonine aldolase
					108	46	108.0000	64.3268	
FN0811					4		5.2065		AAL95007.1| Hypothetical protein
					5		5.0000		
FN0813	2.271	9.608	2.515e-6	6.469e-7	49	11	63.7792	14.2439	AAL95009.1| Transcriptional regulator, TetR family
					59	8	59.0000	11.1873	
FN0814	-4.936	12.593	2.075e-5	1.732e-5	8	322	10.4129	416.9579	AAL95010.1| Propionate CoA-transferase
					18	324	18.0000	453.0847	
FN0815						47		60.8603	AAL95011.1| Propionate permease
						39		54.5380	
FN0816						228		295.2373	AAL95012.1| dehydrogenase with MaoC-like domain
						231		323.0326	
FN0818	-2.046	18.049	3.655e-5	3.81e-5	224	761	291.5621	985.4191	AAL95014.1| DNA-binding protein HU
					221	809	221.0000	1131.3135	
FN0819	-0.439	12.040	2.865e-3	1.059e-2	38	53	49.4614	68.6297	AAL95015.1| Tetratricopeptide repeat family protein
					62	59	62.0000	82.5062	
FN0820	-0.261	14.685	3.42e-2	1.851e-1	91	134	118.4471	173.5166	AAL95016.1| Mercuric reductase
					178	130	178.0000	181.7933	
FN0821	-1.906	11.708	3.531e-5	3.619e-5	29	79	37.7469	102.2971	AAL95017.1| Hypothetical protein
					22	87	22.0000	121.6616	
FN0823	2.457	11.051	2.501e-6	6.407e-7	79	12	102.8277	15.5388	AAL95019.1| GTP-binding protein hflX
					113	17	113.0000	23.7730	
FN0824					4		5.2065		AAL95020.1| hypothetical cytosolic protein
FN0825	-1.849	11.592	1.905e-6	4.211e-7	25	83	32.5404	107.4767	AAL95021.1| Hypothetical cytosolic protein
					26	74	26.0000	103.4823	
FN0826	-1.468	9.967	3.602e-4	8.111e-4	20	37	26.0323	47.9113	AAL95022.1| periplasmic component of efflux system
					12	41	12.0000	57.3348	
FN0827	-0.594	11.207	1.769e-2	8.952e-2	27	35	35.1436	45.3215	AAL95023.1| ABC transporter ATP-binding protein
					44	53	44.0000	74.1157	
FN0828	-1.864	6.988	2.085e-4	4.025e-4	6	17	7.8097	22.0133	AAL95024.1| ABC transporter permease protein
					4	15	4.0000	20.9761	

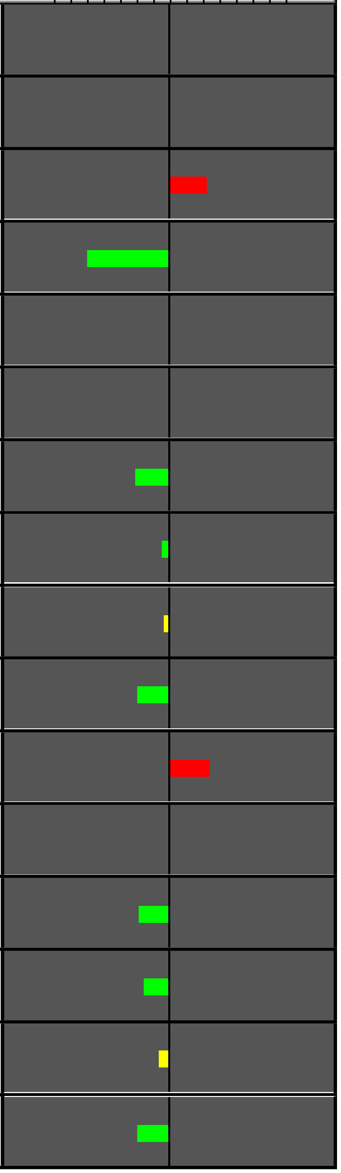

Show detected proteins only
Show all proteins
Filter by category:
GO: amino acid transport

Proteins found: 1358

Enter (or paste) list of ORFs

Find ORFs

Test	Cutoff
q-Value	.005
p-Value	

	Signif	Direction	Applies To
red	yes	+	ratios, bars
yellow	no	n/a	bars
green	yes	−	ratios, bars
pink	yes	+	p-, q-Values
light green	yes	−	p-, q-Values

Dot Plots Dot Plots

Protein	*FnSg vs Fn* Log$_2$ Ratio	Log$_2$ Sum	*q*-Value	*p*-Value	Raw *FnSg*	Raw *Fn*	Normalized *FnSg*	Normalized *Fn*	Description	Log$_2$ Ratios -6 -4 -2 0 2 4 6
FN0830	1.224	11.557	1.181e-4	1.94e-4	59	22	76.7954	28.4878	AAL95026.1| Hypothetical protein	
					91	31	91.0000	43.3507		
FN0832	-1.488	11.225	9.803e-5	1.524e-4	18	65	23.4291	84.1685	AAL95028.1| Hypothetical protein	
					35	57	35.0000	79.7094		
FN0833	-1.099	10.852	1.368e-3	4.349e-3	29	41	37.7469	53.0909	AAL95029.1| Hypothetical protein	
					21	52	21.0000	72.7173		
FN0834	-1.783	9.118	2.469e-5	2.207e-5	8	33	10.4129	42.7317	AAL95030.1| Hypothetical Exported Protein	
					15	32	15.0000	44.7491		
FN0836	-0.069	7.941	9.826e-2	5.79e-1	12	14	15.6194	18.1286	AAL95032.1| Hypothetical protein	
					15	10	15.0000	13.9841		
FN0837									AAL95033.1| Integrase/recombinase	
					8		8.0000			
FN0846	-0.255	13.195	1.815e-2	9.21e-2	74	91	96.3196	117.8359	AAL95042.1| Hypothetical Exported Protein	
					81	67	81.0000	93.6935		
FN0847	-1.184	8.319	7.413e-5	1.081e-4	9	20	11.7145	25.8980	AAL95043.1| TPR-repeat-containing proteins	
					12	20	12.0000	27.9682		
FN0849	0.565	11.018	1.443e-3	4.635e-3	39	33	50.7630	42.7317	AAL95045.1| 8-amino-7-oxononanoate synthase	
					60	23	60.0000	32.1634		
FN0850	1.012	10.133	2.691e-5	2.476e-5	37	17	48.1598	22.0133	AAL95046.1| Hypothetical cytosolic protein	
					47	18	47.0000	25.1714		
FN0853	0.915	12.750	1.01e-3	3.013e-3	96	48	124.9552	62.1552	AAL95049.1| Glycogen synthase	
					103	42	103.0000	58.7332		
FN0854	1.493	13.145	4.839e-5	5.831e-5	114	39	148.3843	50.5011	AAL95050.1| Glucose-1-phosphate adenylyltransferase	
					171	45	171.0000	62.9284		
FN0855	1.104	15.358	5.2e-5	6.425e-5	238	109	309.7847	141.1441	AAL95051.1| Glucose-1-phosphate adenylyltransferase	
					291	99	291.0000	138.4426		
FN0856	0.971	13.500	1.32e-4	2.256e-4	124	55	161.4005	71.2195	AAL95052.1| 1,4-alpha-glucan branching enzyme	
					140	59	140.0000	82.5062		
FN0857	0.743	16.433	5.978e-5	7.818e-5	304	192	395.6914	248.6208	AAL95053.1| Glycogen phosphorylase	
					374	151	374.0000	211.1599		
FN0858	1.165	10.752	1.401e-2	6.904e-2	31	18	40.3501	23.3082	AAL95054.1| 4-alpha-glucanotransferase	
					84	23	84.0000	32.1634		

Show detected proteins only
Show all proteins
Filter by category:
GO: amino acid transport

Proteins found: 1358

Enter (or paste) list of ORFs

Find ORFs

Test	Cutoff
q-Value	.005
p-Value	

Color	Signif	Direction	Applies To
red	yes	+	ratios, bars
yellow	no	n/a	bars
green	yes	–	ratios, bars
light red	yes	+	*p*- , *q*-Values
light green	yes	–	

Dot Plots Dot Plots

Protein	*FnSg* vs Fn: Log_2 Ratio	Log_2 Sum	*q*-Value	*p*-Value	Raw: *FnSg*	Raw: *Fn*	Normalized: *FnSg*	Normalized: *Fn*	Description
FN0865	2.373	13.122	4.153e-6	1.431e-6	172	36	223.8780	46.6164	AAL95061.1| unknown
					206	26	206.0000	36.3587	
FN0867	-1.510	13.119	1.533e-6	3.026e-7	39	126	50.7630	163.1574	AAL95063.1| Long-chain-fatty-acid--CoA ligase
					61	111	61.0000	155.2235	
FN0868	-0.117	6.117				8		10.3592	AAL95064.1| ATPases of the PP superfamily
					8	5	8.0000	6.9920	
FN0869	0.239	5.851			5		6.5081		AAL95065.1| Hydrolase (HAD superfamily)
					10	5	10.0000	6.9920	
FN0870									AAL95066.1| Rhodanese-related sulfurtransferases
						5		6.9920	
FN0871	-0.520	7.278	2.177e-2	1.125e-1	6	9	7.8097	11.6541	AAL95067.1| 3-dehydroquinate synthase
					13	13	13.0000	18.1793	
FN0873	1.275	10.316	2.23e-3	7.777e-3	50	16	65.0808	20.7184	AAL95069.1| Protease IV
					46	18	46.0000	25.1714	
FN0874									AAL95070.1| Phosphohydrolase (MUTT/NUDIX family protein)
					5		5.0000		
FN0875					6		7.8097		AAL95071.1| 23S rRNA methyltransferase
FN0878	0.183	9.147	5.429e-2	3.049e-1	19	14	24.7307	18.1286	AAL95074.1| Transcriptional regulator, GntR family
					26	19	26.0000	26.5698	
FN0884					3		3.9048		AAL95079.1| Hemin transport system permease protein hmuU
FN0885									AAL95081.1| Hemin-binding periplasmic protein hmuT precursor
						10		13.9841	
FN0886						15		19.4235	AAL95082.1| Hemin receptor
						9		12.5857	
FN0887	-1.043	11.455	8.556e-6	4.576e-6	26	57	33.8420	73.8093	AAL95083.1| Oligoendopeptidase F
					40	56	40.0000	78.3109	
FN0888						29		37.5521	AAL95084.1| Uracil permease
						27		37.7571	
FN0889	-0.253	6.592				9		11.6541	AAL95085.1| hypothetical protein
					9	7	9.0000	9.7889	

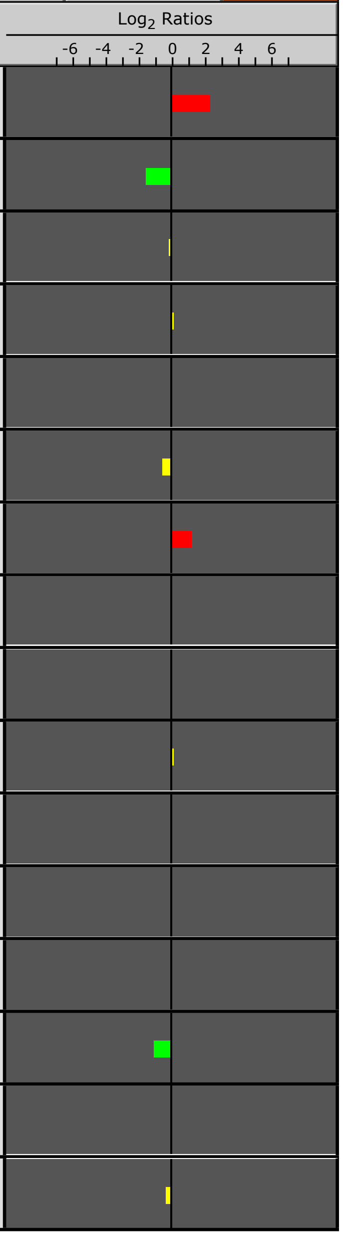

Show detected proteins only
Show all proteins
Filter by category:
GO: amino acid transport

Proteins found: 1358

Enter (or paste) list of ORFs

Find ORFs

Test	Cutoff
q-Value	.005
p-Value	

	Signif	Direction	Applies To
red	yes	+	ratios, bars
yellow	no	n/a	bars
green	yes	−	ratios, bars
pink	yes	+	*p*- , *q*-Values
light green	yes	−	

Dot Plots Dot Plots

Protein	*FnSg* vs *Fn* Log₂ Ratio	Log₂ Sum	*q*-Value	*p*-Value	Raw *FnSg*	Raw *Fn*	Normalized *FnSg*	Normalized *Fn*	Description
FN0892					10		13.0162		AAL95088.1| Phosphoserine phosphatase
					5		5.0000		
FN0893	-0.571	8.186				17		22.0133	AAL95089.1| Hypothetical protein
					14	14	14.0000	19.5777	
FN0896	-1.044	7.977	1.496e-4	2.678e-4	7	19	9.1113	24.6031	AAL95092.1| Hypothetical protein
					13	15	13.0000	20.9761	
FN0898					6		7.8097		AAL95094.1| Hypothetical protein
					6		6.0000		
FN0900	1.716	6.927				4		5.1796	AAL95096.1| Metal dependent hydrolase
					20	5	20.0000	6.9920	
FN0901	1.053	6.665			10		13.0162		AAL95097.1| DNA polymerase, bacteriophage-type
					16	5	16.0000	6.9920	
FN0903	0.982	8.331	4.677e-4	1.124e-3	18	10	23.4291	12.9490	AAL95099.1| Polysialic acid capsule expression protein kpsF
					27	9	27.0000	12.5857	
FN0905						17		22.0133	AAL95101.1| Hypothetical protein
						15		20.9761	
FN0906	0.421	9.992	3.309e-3	1.269e-2	26	21	33.8420	27.1929	AAL95102.1| Glycerol-3-phosphate dehydrogenase [NAD(P)+]
					40	20	40.0000	27.9682	
FN0908	-1.015	8.896	5.304e-4	1.328e-3	9	22	11.7145	28.4878	AAL95104.1| Tpl protein
					19	24	19.0000	33.5618	
FN0910	-0.352	5.732	9.654e-2	5.678e-1	3	3	3.9048	3.8847	AAL95106.1| Nicotinate-nucleotide--dimethylbenzimidazole phosphoribosyltransferase
					9	9	9.0000	12.5857	
FN0911	-0.764	6.276	1.296e-3	4.076e-3	5	8	6.5081	10.3592	AAL95107.1| Alpha-ribazole-5'-phosphate phosphatase
					7	9	7.0000	12.5857	
FN0912					9		11.7145		AAL95108.1| Cobalamin [5'-phosphate] synthase
					7		7.0000		
FN0913	-0.237	6.237				7		9.0643	AAL95109.1| Cobinamide kinase
					8	7	8.0000	9.7889	
FN0915	1.056	10.765	3.335e-3	1.282e-2	54	22	70.2873	28.4878	AAL95111.1| PTS system, N-acetylglucosamine-specific IIA component
					50	21	50.0000	29.3666	
FN0916	-0.058	15.153	8.072e-2	4.666e-1	153	141	199.1473	182.5809	AAL95112.1| Hypothetical Exported Protein
					175	148	175.0000	206.9646	

Log₂ Ratios: -6 -4 -2 0 2 4 6

Show detected proteins only
Show all proteins
Filter by category:
GO: amino acid transport

Proteins found: 1358

Enter (or paste) list of ORFs

Find ORFs

Test	Cutoff
q-Value	.005
p-Value	

Signif	Direction	Applies To
yes	+	ratios, bars
no	n/a	bars
yes	−	ratios, bars
yes	+	*p*-, *q*-Values
yes	−	

Dot Plots Dot Plots

Log$_2$ Ratios: -6 -4 -2 0 2 4 6

Protein	FnSg vs Fn Log$_2$ Ratio	Log$_2$ Sum	*q*-Value	*p*-Value	Raw *FnSg*	Raw *Fn*	Normalized *FnSg*	Normalized *Fn*	Description
FN0917	0.180	7.233	1.004e-1	5.928e-1	7	7	9.1113	9.0643	AAL95113.1| Hypothetical protein
					17	10	17.0000	13.9841	
FN0920									AAL95116.1| Protease HTPX
						7		9.7889	
FN0921	1.838	8.123	1.24e-4	2.07e-4	27	5	35.1436	6.4745	AAL95117.1| Hypothetical protein
					28	8	28.0000	11.1873	
FN0925	-0.349	7.023	7.453e-2	4.28e-1	4	8	5.2065	10.3592	AAL95121.1| Hypothetical protein
					15	11	15.0000	15.3825	
FN0926	2.328	8.244			30	6	39.0485	7.7694	AAL95122.1| GTP pyrophosphokinase
					39		39.0000		
FN0928	-0.189	6.172			3	7	3.9048	9.0643	AAL95124.1| O-sialoglycoprotein endopeptidase
					12		12.0000		
FN0929					22		28.6356		AAL95125.1| ATP/GTP hydrolase
					26		26.0000		
FN0930	-2.011	6.654				16		20.7184	AAL95126.1| Glycerol-3-phosphate cytidylyltransferase
					5	14	5.0000	19.5777	
FN0932	-0.123	8.440	1.146e-1	6.879e-1	9	16	11.7145	20.7184	AAL95128.1| Hypothetical protein
					24	13	24.0000	18.1793	
FN0934	1.772	8.230	2.848e-3	1.051e-2	30	8	39.0485	10.3592	AAL95130.1| Chorismate synthase
					25	6	25.0000	8.3905	
FN0938					42		54.6679		AAL95134.1| Hypothetical protein
					37		37.0000		
FN0940					14		18.2226		AAL95136.1| Hypothetical protein
					24		24.0000		
FN0941	0.983	10.104	7.167e-3	3.159e-2	44	17	57.2711	22.0133	AAL95137.1| Gamma-glutamyltranspeptidase
					36	18	36.0000	25.1714	
FN0943	1.376	7.849	8.893e-3	4.065e-2	13	7	16.9210	9.0643	AAL95139.1| Sensory Transduction Protein Kinase
					32	7	32.0000	9.7889	
FN0947	-1.150	12.309	8.938e-4	2.6e-3	32	71	41.6517	91.9379	AAL95143.1| Hypothetical protein
					54	86	54.0000	120.2632	
FN0949	-2.512	14.308	4.18e-5	4.698e-5	44	251	57.2711	325.0200	AAL95145.1| DNA helicase
					62	254	62.0000	355.1961	

Show detected proteins only
Show all proteins
Filter by category:
GO: amino acid transport

Proteins found: 1358

Enter (or paste) list of ORFs

Find ORFs

Test	Cutoff
q-Value	.005
p-Value	

	Signif	Direction	Applies To
red	yes	+	ratios, bars
yellow	no	n/a	bars
green	yes	−	ratios, bars
pink	yes	+	*p*- , *q*-Values
light green	yes	−	

Dot Plots Dot Plots

Protein	FnSg vs Fn Log₂ Ratio	Log₂ Sum	*q*-Value	*p*-Value	Raw *FnSg*	Raw *Fn*	Normalized *FnSg*	Normalized *Fn*	Description
FN0951	-1.474	7.089				16		20.7184	AAL95147.1| Precorrin-3B C17-methyltransferase
					7	13	7.0000	18.1793	
FN0952									AAL95148.1| Cobalamin biosynthesis protein G
						6		8.3905	
FN0957	-0.807	7.335	1.344e-2	6.575e-2	4	13	5.2065	16.8337	AAL95153.1| Precorrin-4 C11-methyltransferase
					14	12	14.0000	16.7809	
FN0958	-1.973	7.588				23		29.7827	AAL95154.1| unknown
					7	18	7.0000	25.1714	
FN0959	-2.898	7.542				23		29.7827	AAL95155.1| Precorrin-2 C20-methyltransferase
					5	32	5.0000	44.7491	
FN0961	1.938	7.463				4		5.1796	AAL95157.1| Hypothetical protein
					26	6	26.0000	8.3905	
FN0962	-1.888	9.545	1.759e-5	1.358e-5	8	38	10.4129	49.2062	AAL95158.1| Hypothetical cytosolic protein
					18	40	18.0000	55.9364	
FN0964					7		9.1113		AAL95160.1| Precorrin-8W decarboxylase
					8		8.0000		
FN0965	0.869	11.020	6.598e-3	2.859e-2	57	24	74.1921	31.0776	AAL95161.1| D-3-phosphoglycerate dehydrogenase
					49	26	49.0000	36.3587	
FN0966									AAL95162.1| Precorrin-6Y C5,15-methyltransferase (decarboxylating)
					5		5.0000		
FN0967									AAL95163.1| CbiD protein
					6		6.0000		
FN0970	-1.208	8.061	2.347e-3	8.283e-3	5	20	6.5081	25.8980	AAL95166.1| Precorrin-8X methylmutase
					15	17	15.0000	23.7730	
FN0971					4		5.2065		AAL95167.1| hypothetical cytosolic protein
FN0972					8		10.4129		AAL95168.1| Cobyrinic acid a,c-diamide synthase
					32		32.0000		
FN0976	0.356	11.171	3.725e-2	2.029e-1	32	31	41.6517	40.1419	AAL95172.1| Hypothetical protein
					67	32	67.0000	44.7491	
FN0977	1.904	8.650			35	8	45.5566	10.3592	AAL95173.1| Cobyric acid synthase
					32		32.0000		

Log₂ Ratios: -6 -4 -2 0 2 4 6

Show detected proteins only
Show all proteins
Filter by category:
GO: amino acid transport

Proteins found: 1358

Enter (or paste) list of ORFs

Find ORFs

Test	Cutoff
q-Value	.005
p-Value	

	Signif	Direction	Applies To
red	yes	+	ratios, bars
yellow	no	n/a	bars
green	yes	−	ratios, bars
pink	yes	+	*p*-, *q*-Values
light green	yes	−	

Dot Plots Dot Plots

Protein	*FnSg vs Fn* Log_2 Ratio	Log_2 Sum	*q*-Value	*p*-Value	Raw *FnSg*	Raw *Fn*	Normalized *FnSg*	Normalized *Fn*	Description
FN0981	-1.046	12.949	1.416e-3	4.534e-3	59	98	76.7954	126.9002	AAL95177.1| Phosphoribosylamine--glycine ligase
					47	92	47.0000	128.6537	
FN0982	-0.717	16.261	7.407e-5	1.079e-4	180	272	234.2910	352.2129	AAL95178.1| Phosphoribosylaminoimidazolecarboxamide formyltransferase
					203	262	203.0000	366.3833	
FN0983	-0.131	14.870	3.37e-2	1.821e-1	115	135	149.6859	174.8115	AAL95179.1| Hypothetical protein
					181	134	181.0000	187.3869	
FN0984	1.135	10.768	2.169e-6	5.104e-7	49	23	63.7792	29.7827	AAL95180.1| Tetracenomycin polyketide synthesis O-methyltransferase tcmP
					60	19	60.0000	26.5698	
FN0985	0.877	7.512	3.077e-3	1.159e-2	12	10	15.6194	12.9490	AAL95181.1| Phosphoribosylglycinamide formyltransferase
					21	5	21.0000	6.9920	
FN0986	0.475	15.021	3.343e-2	1.805e-1	119	119	154.8924	154.0931	AAL95182.1| Phosphoribosylformylglycinamidine cyclo-ligase
					275	111	275.0000	155.2235	
FN0987	0.022	14.528	1.348e-1	8.278e-1	112	106	145.7811	137.2594	AAL95183.1| Amidophosphoribosyltransferase
					164	120	164.0000	167.8092	
FN0988	-0.386	16.254	1.078e-2	5.092e-2	156	243	203.0522	314.6608	AAL95184.1| Phosphoribosylamidoimidazole-succinocarboxamide synthase
					286	232	286.0000	324.4311	
FN0989	0.205	15.133	7.669e-2	4.414e-1	116	141	150.9875	182.5809	AAL95185.1| Phosphoribosylaminoimidazole carboxylase catalytic subunit
					256	122	256.0000	170.6060	
FN0990	0.344	21.397	1.098e-5	6.798e-6	1412	1164	1837.8826	1507.2639	AAL95186.1| Phosphoribosylformylglycinamidine synthase
					1907	1032	1907.0000	1443.1588	
FN0991	0.565	9.062	8.382e-3	3.79e-2	24	11	31.2388	14.2439	AAL95187.1| CDP-diacylglycerol--serine O-phosphatidyltransferase
					25	17	25.0000	23.7730	
FN0992	-0.278	10.387	6.06e-3	2.585e-2	28	32	36.4453	41.4368	AAL95188.1| ADP-heptose:LPS heptosyltransferase II
					30	28	30.0000	39.1555	
FN0994	-0.481	13.827	1.253e-2	6.059e-2	60	112	78.0970	145.0288	AAL95190.1| Hypothetical protein
					126	100	126.0000	139.8410	
FN0997	0.845	11.311	3.85e-3	1.54e-2	60	30	78.0970	38.8470	AAL95193.1| Hypothetical protein
					57	26	57.0000	36.3587	
FN0998	0.938	15.794	2.681e-5	2.464e-5	248	132	322.8009	170.9268	AAL95194.1| Dipeptide-binding protein
					337	124	337.0000	173.4028	
FN0999	0.532	10.149	6.355e-3	2.734e-2	30	26	39.0485	33.6674	AAL95195.1| Deblocking aminopeptidase
					42	16	42.0000	22.3746	

Log_2 Ratios: -6 -4 -2 0 2 4 6

Show detected proteins only
Show all proteins
Filter by category:
GO: amino acid transport

Proteins found: 1358

Enter (or paste) list of ORFs

Find ORFs

Test	Cutoff
q-Value	.005
p-Value	

Signif	Direction	Applies To
yes	+	ratios, bars
no	n/a	bars
yes	−	ratios, bars
yes	+	*p*- , *q*-Values
yes	−	

Dot Plots Dot Plots

Protein	FnSg vs Fn Log$_2$ Ratio	Log$_2$ Sum	*q*-Value	*p*-Value	Raw *FnSg*	Raw *Fn*	Normalized *FnSg*	Normalized *Fn*	Description
FN1000						63		81.5787	AAL95196.1| Biotin synthase
						52		72.7173	
FN1001	0.526	10.371	7.727e-4	2.185e-3	31	22	40.3501	28.4878	AAL95197.1| Dethiobiotin synthetase
					47	23	47.0000	32.1634	
FN1002	-1.433	10.841	7.26e-5	1.046e-4	17	59	22.1275	76.3991	AAL95198.1| Adenosylmethionine-8-amino -7-oxononanoate aminotransferase
					30	46	30.0000	64.3268	
FN1003	1.665	12.176	3.113e-4	6.751e-4	84	32	109.3358	41.4368	AAL95199.1| Outer membrane protein P1 precursor
					133	25	133.0000	34.9602	
FN1004	0.192	8.589	1.141e-1	6.845e-1	23	10	29.9372	12.9490	AAL95200.1| Transcriptional regulator, TetR family
					12	17	12.0000	23.7730	
FN1005	-0.969	12.157	4.793e-4	1.161e-3	45	78	58.5727	101.0022	AAL95201.1| Hypothetical protein
					38	63	38.0000	88.0998	
FN1006									AAL95202.1| Acetyltransferase
					12		12.0000		
FN1008						4		5.1796	AAL95204.1| Hypothetical protein
FN1009						8		10.3592	AAL95205.1| Hypothetical protein
						3		4.1952	
FN1010	-0.758	14.147	2.453e-4	4.93e-4	90	128	117.1455	165.7472	AAL95206.1| Hypothetical cytosolic protein
					90	132	90.0000	184.5901	
FN1011	-0.124	7.260	1.113e-1	6.653e-1	9	7	11.7145	9.0643	AAL95207.1| MGPA protein
					12	12	12.0000	16.7809	
FN1012	0.830	9.761	3.931e-3	1.577e-2	25	19	32.5404	24.6031	AAL95208.1| HPR(Ser) kinase
					46	14	46.0000	19.5777	
FN1014	0.418	9.669	4.723e-2	2.622e-1	33	23	42.9533	29.7827	AAL95210.1| Folylpolyglutamate synthase
					23	14	23.0000	19.5777	
FN1015	-1.189	8.409	6.532e-3	2.825e-2	8	16	10.4129	20.7184	AAL95211.1| 5'-methylthioadenosine nucleosidase
					14	25	14.0000	34.9602	
FN1016	0.269	9.840	1.155e-2	5.513e-2	28	21	36.4453	27.1929	AAL95212.1| Lipid A biosynthesis lauroyl acyltransferase
					30	20	30.0000	27.9682	
FN1017	-0.888	13.443	3.443e-4	7.683e-4	70	104	91.1132	134.6696	AAL95213.1| Hypothetical Exported Protein
					64	109	64.0000	152.4267	

Log$_2$ Ratios: -6 -4 -2 0 2 4 6

Show detected proteins only
Show all proteins
Filter by category:
GO: amino acid transport

Proteins found: 1358

Enter (or paste) list of ORFs

Find ORFs

Test	Cutoff
q-Value	.005
p-Value	

	Signif	Direction	Applies To
red	yes	+	ratios, bars
yellow	no	n/a	bars
green	yes	−	ratios, bars
pink	yes	+	*p*- , *q*-Values
light green	yes	−	

Dot Plots Dot Plots

Protein	FnSg vs Fn: Log_2 Ratio	Log_2 Sum	*q*-Value	*p*-Value	Raw *FnSg*	Raw *Fn*	Normalized *FnSg*	Normalized *Fn*	Description
FN1019	1.342	22.254	4.353e-5	4.996e-5	2651	1077	3450.5855	1394.6076	AAL95215.1| 3-hydroxybutyryl-CoA dehydrogenase
					3671	1012	3671.0000	1415.1906	
FN1020	1.016	16.956	7.01e-3	3.075e-2	478	195	622.1727	252.5055	AAL95216.1| 3-hydroxybutyryl-CoA dehydratase
					392	178	392.0000	248.9169	
FN1022						26		33.6674	AAL95218.1| Calcium-transporting ATPase
						45		62.9284	
FN1023					24		31.2388		AAL95219.1| 5-Nitroimidazole antibiotic resistance protein
					15		15.0000		
FN1024	-0.340	20.600	1.236e-2	5.962e-2	726	1079	944.9736	1397.1974	AAL95220.1| DNA-binding protein HU
					1296	1029	1296.0000	1438.9636	
FN1025	-0.683	7.327				14		18.1286	AAL95221.1| Guanine-hypoxanthine permease
					10	10	10.0000	13.9841	
FN1026	-1.144	6.314				14		18.1286	AAL95222.1| tRNA pseudouridine synthase A
					6	6	6.0000	8.3905	
FN1028	-0.896	10.271	8.173e-3	3.679e-2	25	46	32.5404	59.5654	AAL95224.1| Deoxyuridine 5'-triphosphate nucleotidohydrolase
					19	26	19.0000	36.3587	
FN1029	-1.376	12.703	1.812e-5	1.419e-5	41	106	53.3663	137.2594	AAL95225.1| Zinc protease
					48	90	48.0000	125.8569	
FN1030	2.408	6.323			14	3	18.2226	3.8847	AAL95226.1| Hypothetical membrane-spanning protein
					23		23.0000		
FN1031	0.112	6.807				6		7.7694	AAL95227.1| Hypothetical membrane-spanning protein
					11	9	11.0000	12.5857	
FN1033	-0.763	12.293	2.02e-3	6.894e-3	49	81	63.7792	104.8869	AAL95229.1| Methyltransferase
					45	57	45.0000	79.7094	
FN1034					5		6.5081		AAL95230.1| Transcriptional regulator, TetR family
					13		13.0000		
FN1035									AAL95231.1| Iron-sulfur flavoprotein
					3		3.0000		
FN1036					3		3.9048		AAL95232.1| Hypothetical protein
					4		4.0000		
FN1037					11		14.3178		AAL95233.1| Hypothetical cytosolic protein
					20		20.0000		

Log_2 Ratios: -6 -4 -2 0 2 4 6

Show detected proteins only
Show all proteins
Filter by category:
GO: amino acid transport

Proteins found: 1358

Enter (or paste) list of ORFs

Find ORFs

Test	Cutoff
q-Value	.005
p-Value	

	Signif	Direction	Applies To
red	yes	+	ratios, bars
yellow	no	n/a	bars
green	yes	−	ratios, bars
pink	yes	+	*p*- , *q*-Values
light green	yes	−	

Dot Plots Dot Plots

Protein	*FnSg vs Fn* Log$_2$ Ratio	Log$_2$ Sum	*q*-Value	*p*-Value	Raw *FnSg*	Raw *Fn*	Normalized *FnSg*	Normalized *Fn*	Description
**FN1041**					11		14.3178		AAL95237.1| Acetyltransferase
					19		19.0000		
**FN1042**	-2.845	11.186	1.938e-5	1.574e-5	10	107	13.0162	138.5543	AAL95238.1| S1 RNA binding domain
					23	86	23.0000	120.2632	
**FN1045**						7		9.0643	AAL95241.1| Hypothetical protein
						4		5.5936	
**FN1048**					3		3.9048		AAL95244.1| Hypothetical membrane-spanning protein
**FN1050**						7		9.0643	AAL95246.1| Lactoylglutathione lyase
**FN1055**	0.291	12.045	8.966e-3	4.105e-2	59	40	76.7954	51.7960	AAL95251.1| Cysteine synthase
					67	47	67.0000	65.7253	
**FN1057**	0.533	6.990	7.972e-2	4.603e-1	17	8	22.1275	10.3592	AAL95253.1| Diamine acetyltransferase
					5	6	5.0000	8.3905	
**FN1060**	0.612	10.239	1.057e-3	3.183e-3	33	24	42.9533	31.0776	AAL95256.1| hypothetical cytosolic protein
					43	18	43.0000	25.1714	
**FN1062**	-1.435	11.992	5.281e-4	1.32e-3	22	80	28.6356	103.5920	AAL95258.1| Hydrolase
					49	76	49.0000	106.2791	
**FN1063**	1.285	8.472	8.544e-3	3.876e-2	16	10	20.8259	12.9490	AAL95259.1| N-acyl-L-amino acid amidohydrolase
					38	8	38.0000	11.1873	
**FN1066**	0.902	7.375	1.146e-5	7.309e-6	14	7	18.2226	9.0643	AAL95262.1| Exodeoxyribonuclease VII large subunit
					17	7	17.0000	9.7889	
**FN1067**	-0.182	12.727	3.395e-2	1.836e-1	65	75	84.6051	97.1175	AAL95263.1| Tetratricopeptide repeat family protein
					70	56	70.0000	78.3109	
**FN1068**					16		20.8259		AAL95264.1| Smf protein
					13		13.0000		
**FN1069**	-1.288	11.991	5.692e-4	1.452e-3	22	73	28.6356	94.5277	AAL95265.1| DNA topoisomerase I
					53	75	53.0000	104.8807	
**FN1070**	-1.303	7.724	9.517e-4	2.805e-3	5	18	6.5081	23.3082	AAL95266.1| Glucose inhibited division protein A
					12	16	12.0000	22.3746	
**FN1071**	-0.484	4.484							AAL95267.1| Integrase/recombinase
					4	4	4.0000	5.5936	

Log$_2$ Ratios: -6 -4 -2 0 2 4 6

Show detected proteins only
Show all proteins
Filter by category:
GO: amino acid transport

Proteins found: 1358

Enter (or paste) list of ORFs

Find ORFs

Test	Cutoff
q-Value	.005
p-Value	

	Signif	Direction	Applies To
red	yes	+	ratios, bars
yellow	no	n/a	bars
green	yes	−	ratios, bars
light red	yes	+	*p*- , *q*-Values
light green	yes	−	

Dot Plots Dot Plots

Protein	*FnSg vs Fn* Log$_2$ Ratio	Log$_2$ Sum	*q*-Value	*p*-Value	Raw *FnSg*	Raw *Fn*	Normalized *FnSg*	Normalized *Fn*	Description
FN1072	-1.506	10.713	3.42e-4	7.616e-4	12	57	15.6194	73.8093	AAL95268.1| GTP-binding protein
					33	46	33.0000	64.3268	
FN1073	-0.586	7.052	3.602e-3	1.414e-2	6	11	7.8097	14.2439	AAL95269.1| Hypothetical protein
					11	10	11.0000	13.9841	
FN1074	-0.271	10.300	1.087e-2	5.138e-2	22	30	28.6356	38.8470	AAL95270.1| Signal recognition particle receptor FtsY
					36	28	36.0000	39.1555	
FN1075									AAL95271.1| Hypothetical protein
					7		7.0000		
FN1077	0.234	6.594			11	7	14.3178	9.0643	AAL95273.1| Hypothetical protein
					7		7.0000		
FN1078	-0.089	15.193	5.043e-2	2.817e-1	144	142	187.4328	183.8758	AAL95274.1| Hypothetical exported 24-amino acid repeat protein
					188	154	188.0000	215.3551	
FN1079	0.970	18.635	7.349e-4	2.037e-3	748	364	973.6092	471.3437	AAL95275.1| Neutrophil-activating protein A
					812	315	812.0000	440.4991	
FN1080	0.710	6.498	5.753e-3	2.432e-2	11	5	14.3178	6.4745	AAL95276.1| Export ABC transporter
					10	6	10.0000	8.3905	
FN1081						34		44.0266	AAL95277.1| unknown
						25		34.9602	
FN1084	-2.644	10.928	5.242e-4	1.307e-3	11	97	14.3178	125.6053	AAL95280.1| unknown
					21	68	21.0000	95.0919	
FN1085	1.003	12.351	6.321e-4	1.663e-3	85	40	110.6374	51.7960	AAL95281.1| 4-methyl-5(B-hydroxyethyl)-thiazole monophosphate biosynthesis enzyme
					94	36	94.0000	50.3427	
FN1086	1.114	7.603	7.851e-3	3.51e-2	20	6	26.0323	7.7694	AAL95282.1| Transporter
					15	8	15.0000	11.1873	
FN1088	0.353	10.067	7.738e-4	2.19e-3	30	21	39.0485	27.1929	AAL95284.1| NADH oxidase
					35	22	35.0000	30.7650	
FN1089	2.829	13.093	6.809e-4	1.836e-3	164	25	213.4651	32.3725	AAL95285.1| ATP-binding protein (contains P-loop)
					285	27	285.0000	37.7571	
FN1091	-0.843	8.500	2.384e-3	8.445e-3	8	21	10.4129	27.1929	AAL95287.1| Sigma factor sigB regulation protein rsbU
					18	17	18.0000	23.7730	
FN1092	0.908	11.784	1.32e-3	4.169e-3	62	27	80.7002	34.9623	AAL95288.1| Hypothetical protein
					82	37	82.0000	51.7412	

Log$_2$ Ratios: -6 -4 -2 0 2 4 6

Show detected proteins only
Show all proteins
Filter by category:
GO: amino acid transport

Proteins found: 1358

Enter (or paste) list of ORFs

Find ORFs

Test	Cutoff
q-Value	.005
p-Value	

	Signif	Direction	Applies To
red	yes	+	ratios, bars
yellow	no	n/a	bars
green	yes	−	ratios, bars
light red	yes	+	*p*- , *q*-Values
light green	yes	−	

Dot Plots | Dot Plots

Protein	FnSg vs Fn Log$_2$ Ratio	Log$_2$ Sum	*q*-Value	*p*-Value	Raw *FnSg*	Raw *Fn*	Normalized *FnSg*	Normalized *Fn*	Description
FN1093	-1.931	11.305	1.177e-4	1.931e-4	15	75	19.5242	97.1175	AAL95289.1| Hypothetical protein
					32	71	32.0000	99.2871	
FN1094					13		16.9210		AAL95290.1| Dolichol-phosphate mannosyltransferase
					24		24.0000		
FN1096	-0.910	10.411	7.755e-4	2.196e-3	16	36	20.8259	46.6164	AAL95292.1| Hypothetical protein
					33	39	33.0000	54.5380	
FN1097	-3.311	9.311				47		60.8603	AAL95293.1| Hypothetical protein
					8	70	8.0000	97.8887	
FN1101	0.305	5.695				5		6.4745	AAL95297.1| ATPase
					8		8.0000		
FN1102	-2.018	8.662				28		36.2572	AAL95298.1| tRNA 2'phosphotransferase
					10	32	10.0000	44.7491	
FN1103	-0.839	12.838	1.675e-4	3.098e-4	43	85	55.9695	110.0665	AAL95299.1| Excinuclease ABC subunit A
					72	85	72.0000	118.8648	
FN1105	-1.252	12.275	5.963e-4	1.541e-3	34	76	44.2550	98.4124	AAL95301.1| Hypothetical protein
					47	85	47.0000	118.8648	
FN1106	-0.775	12.697	3.518e-4	7.885e-4	55	88	71.5889	113.9512	AAL95302.1| L-serine dehydratase
					53	71	53.0000	99.2871	
FN1111	0.485	7.453			11		14.3178		AAL95307.1| Dipeptide-binding protein
					17	8	17.0000	11.1873	
FN1117	-1.871	9.855	8.249e-4	2.362e-3	16	37	20.8259	47.9113	AAL95313.1| LSU ribosomal protein L21P
					11	49	11.0000	68.5221	
FN1119	-2.497	12.350	1.089e-5	6.706e-6	16	142	20.8259	183.8758	AAL95315.1| LSU ribosomal protein L27P
					40	114	40.0000	159.4187	
FN1120	0.750	15.433	8.342e-4	2.394e-3	207	109	269.4346	141.1441	AAL95316.1| Phosphoenolpyruvate carboxykinase (ATP)
					276	131	276.0000	183.1917	
FN1121	-0.163	12.497	2.032e-2	1.042e-1	52	67	67.6841	86.7583	AAL95317.1| hypothetical cytosolic protein
					76	53	76.0000	74.1157	
FN1122	-1.834	14.132	2.495e-4	5.036e-4	66	219	85.9067	283.5832	AAL95318.1| Long-chain-fatty-acid--CoA ligase
					56	159	56.0000	222.3471	
FN1123	-1.550	8.094	1.478e-3	4.775e-3	11	21	14.3178	27.1929	AAL95319.1| Thioredoxin-like protein
					5	21	5.0000	29.3666	

Log$_2$ Ratios: -6 -4 -2 0 2 4 6

Show detected proteins only
Show all proteins
Filter by category:
GO: amino acid transport

Proteins found: 1358

Enter (or paste) list of ORFs

Find ORFs

Test	Cutoff
q-Value	.005
p-Value	

Color	Signif	Direction	Applies To
red	yes	+	ratios, bars
yellow	no	n/a	bars
green	yes	–	ratios, bars
pink	yes	+	*p*- , *q*-Values
light green	yes	–	*p*- , *q*-Values

Dot Plots Dot Plots

Protein	*FnSg* vs *Fn*				Raw		Normalized		Description
	Log_2 Ratio	Log_2 Sum	*q*-Value	*p*-Value	*FnSg*	*Fn*	*FnSg*	*Fn*	
FN1124	-0.264	17.404	3.451e-5	3.499e-5	286	356	372.2623	460.9845	AAL95320.1| Outer membrane porin F
					388	323	388.0000	451.6863	
FN1125	1.202	11.928	1.85e-3	6.205e-3	84	29	109.3358	37.5521	AAL95321.1| LemA protein
					80	32	80.0000	44.7491	
FN1127	0.590	11.548	8.464e-4	2.436e-3	54	30	70.2873	38.8470	AAL95323.1| Hypothetical membrane-spanning protein
					64	36	64.0000	50.3427	
FN1128	-0.399	15.526	2.826e-4	5.924e-4	140	193	182.2263	249.9157	AAL95324.1| Acylamino-acid-releasing enzyme
					196	178	196.0000	248.9169	
FN1129	-1.502	7.539	2.65e-4	5.444e-4	4	16	5.2065	20.7184	AAL95325.1| Chromosome partition protein smc
					11	18	11.0000	25.1714	
FN1130	-0.550	7.469				13		16.8337	AAL95326.1| Tetraacyldisaccharide 4'-kinase
					11	11	11.0000	15.3825	
FN1131	-0.484	6.098							AAL95327.1| Hypothetical protein
					7	7	7.0000	9.7889	
FN1133	0.040	9.875	1.23e-1	7.452e-1	27	24	35.1436	31.0776	AAL95329.1| N-acetylglucosamine-6-phosphate deacetylase
					27	21	27.0000	29.3666	
FN1134	-2.614	8.614				40		51.7960	AAL95330.1| Hypothetical cytosolic protein
					8	33	8.0000	46.1475	
FN1135	-0.530	14.510	1.273e-3	3.987e-3	107	142	139.2730	183.8758	AAL95331.1| Phosphonates-binding protein
					115	131	115.0000	183.1917	
FN1136	1.407	8.072	9.993e-4	2.976e-3	18	8	23.4291	10.3592	AAL95332.1| Phosphonates transport ATP-binding protein phnC
					30	7	30.0000	9.7889	
FN1138	-0.681	18.642	6.319e-3	2.716e-2	491	644	639.0937	833.9158	AAL95334.1| Hypothetical cytosolic protein
					371	562	371.0000	785.9063	
FN1139	1.010	13.601	1.441e-3	4.628e-3	107	63	139.2730	81.5787	AAL95335.1| Activator of (R)-2-hydroxyglutaryl-CoA dehydratase
					177	54	177.0000	75.5141	
FN1140	1.120	7.800	2.05e-2	1.052e-1	10	7	13.0162	9.0643	AAL95336.1| hypothetical protein
					31	8	31.0000	11.1873	
FN1142	-1.166	6.612	3.314e-4	7.316e-4	4	11	5.2065	14.2439	AAL95338.1| Oxygen-independent coproporphyrinogen III oxidase
					8	11	8.0000	15.3825	
FN1143	1.365	14.666	4.372e-3	1.777e-2	244	72	317.5944	93.2328	AAL95339.1| Glucosamine-6-phosphate isomerase
					200	77	200.0000	107.6775	

Log_2 Ratios: -6 -4 -2 0 2 4 6

Show detected proteins only
Show all proteins
Filter by category:
GO: amino acid transport

Proteins found: 1358

Enter (or paste) list of ORFs

Find ORFs

Test	Cutoff
q-Value	.005
p-Value	

	Signif	Direction	Applies To
red	yes	+	ratios, bars
yellow	no	n/a	bars
green	yes	−	ratios, bars
pink	yes	+	*p*-, *q*-Values
light green	yes	−	

Dot Plots Dot Plots

Protein	FnSg vs Fn: Log_2 Ratio	Log_2 Sum	*q*-Value	*p*-Value	Raw *FnSg*	Raw *Fn*	Normalized *FnSg*	Normalized *Fn*	Description
FN1144	-1.023	15.026	1.15e-5	7.354e-6	107	210	139.2730	271.9291	AAL95340.1| Hypothetical Exported Protein
					117	178	117.0000	248.9169	
FN1145	-0.261	8.956	8.845e-2	5.156e-1	9	15	11.7145	19.4235	AAL95341.1| Oligoendopeptidase F
					29	21	29.0000	29.3666	
FN1146						24		31.0776	AAL95342.1| Hypothetical exported 24-amino acid repeat protein
						20		27.9682	
FN1147						20		25.8980	AAL95343.1| Hypothetical protein
						17		23.7730	
FN1148	0.232	11.418	5.646e-3	2.379e-2	41	40	53.3663	51.7960	AAL95344.1| Serine/threonine sodium symporter
					60	32	60.0000	44.7491	
FN1149	-0.203	8.048	2.973e-3	1.11e-2	11	14	14.3178	18.1286	AAL95345.1| ATP-dependent nuclease subunit A
					16	12	16.0000	16.7809	
FN1150	-0.484	6.098							AAL95346.1| unknown
					7	7	7.0000	9.7889	
FN1152	-0.662	13.990	9.151e-5	1.399e-4	79	128	102.8277	165.7472	AAL95348.1| Aspartate aminotransferase
					100	111	100.0000	155.2235	
FN1153						36		46.6164	AAL95349.1| Hypothetical protein
						26		36.3587	
FN1154	0.060	6.643			8		10.4129		AAL95350.1| Ribonuclease BN
					10	7	10.0000	9.7889	
FN1155	-1.457	5.766	5.269e-3	2.197e-2	3	7	3.9048	9.0643	AAL95351.1| Cell division protein ftsI
					5	11	5.0000	15.3825	
FN1156					10		13.0162		AAL95352.1| Primosomal protein N'
					5		5.0000		
FN1157						7		9.0643	AAL95353.1| Polypeptide deformylase
						7		9.7889	
FN1159	0.801	14.088	3.377e-4	7.494e-4	137	68	178.3215	88.0532	AAL95355.1| Fructose-1,6-bisphosphatase
					170	80	170.0000	111.8728	
FN1160	-0.124	6.144	7.389e-2	4.24e-1	7	6	9.1113	7.7694	AAL95356.1| SWF/SNF family helicase
					7	7	7.0000	9.7889	
FN1161	-0.986	6.896	3.008e-3	1.126e-2	5	14	6.5081	18.1286	AAL95357.1| Glutamate racemase
					9	9	9.0000	12.5857	

Log_2 Ratios: -6 -4 -2 0 2 4 6

Show detected proteins only
Show all proteins
Filter by category:
GO: amino acid transport

Proteins found: 1358

Enter (or paste) list of ORFs

Find ORFs

Test	Cutoff
q-Value	.005
p-Value	

	Signif	Direction	Applies To
red	yes	+	ratios, bars
yellow	no	n/a	bars
green	yes	−	ratios, bars
light red	yes	+	*p*-, *q*-Values
light green	yes	−	*p*-, *q*-Values

Dot Plots Dot Plots

Protein	*FnSg* vs *Fn* Log₂ Ratio	Log₂ Sum	*q*-Value	*p*-Value	Raw *FnSg*	Raw *Fn*	Normalized *FnSg*	Normalized *Fn*	Description	Log₂ Ratios (-6 -4 -2 0 2 4 6)
**FN1162**	0.121	5.732			4		5.2065		AAL95358.1| Hydroxyacylglutathione hydrolase	
					10	5	10.0000	6.9920		
**FN1163**	-0.499	11.635	1.334e-2	6.521e-2	46	56	59.8744	72.5144	AAL95359.1| Thioredoxin reductase	
					35	44	35.0000	61.5300		
**FN1164**	-0.764	8.088	5.827e-4	1.496e-3	11	17	14.3178	22.0133	AAL95360.1| Glucokinase	
					11	15	11.0000	20.9761		
**FN1165**	0.138	24.291	6.285e-2	3.564e-1	4182	3391	5443.3605	4391.0067	AAL95361.1| D-galactose-binding protein	
					4062	3036	4062.0000	4245.5719		
**FN1166**	-0.107	15.858	4.275e-2	2.353e-1	178	179	231.6877	231.7871	AAL95362.1| Galactoside transport ATP-binding protein mglA	
					238	196	238.0000	274.0883		
**FN1167**	-1.960	9.059	1.49e-2	7.405e-2	8	52	10.4129	67.3348	AAL95363.1| Galactoside transport system permease protein mglC	
					13	17	13.0000	23.7730		
**FN1169**	-0.078	17.277	1.066e-1	6.339e-1	245	330	318.8961	427.3171	AAL95365.1| L-lactate dehydrogenase	
					457	280	457.0000	391.5547		
**FN1170**	0.364	25.271	8.773e-6	4.768e-6	5624	4375	7320.2915	5665.1886	AAL95366.1| Pyruvate-flavodoxin oxidoreductase	
					7117	3972	7117.0000	5554.4834		
**FN1171**	-0.013	19.843	1.435e-1	8.903e-1	673	793	875.9879	1026.8559	AAL95367.1| Acetate kinase	
					1055	659	1055.0000	921.5520		
**FN1172**	-0.600	18.793	4.171e-5	4.682e-5	397	627	516.7418	811.9025	AAL95368.1| Phosphate acetyltransferase	
					578	606	578.0000	847.4363		
**FN1179**	0.101	5.069							AAL95375.1| ATP-dependent RNA helicase	
					6	4	6.0000	5.5936		
**FN1180**	-0.700	6.314				10		12.9490	AAL95376.1| Hypothetical protein	
					7	7	7.0000	9.7889		
**FN1181**	-0.991	12.938	1.55e-4	2.816e-4	42	99	54.6679	128.1951	AAL95377.1| unknown	
					71	87	71.0000	121.6616		
**FN1182**	-0.935	6.105				8		10.3592	AAL95378.1| Hypothetical protein	
					6	9	6.0000	12.5857		
**FN1183**	0.873	6.883	1.428e-2	7.06e-2	8	7	10.4129	9.0643	AAL95379.1| Hypothetical cytosolic protein	
					19	5	19.0000	6.9920		
**FN1185**	-0.111	10.095	5.219e-2	2.924e-1	22	25	28.6356	32.3725	AAL95381.1| SIR2 family protein	
					35	26	35.0000	36.3587		

Show detected proteins only
Show all proteins
Filter by category:
GO: amino acid transport

Proteins found: 1358

Enter (or paste) list of ORFs

Find ORFs

Test	Cutoff
q-Value	.005
p-Value	

Color	Signif	Direction	Applies To
red	yes	+	ratios, bars
yellow	no	n/a	bars
green	yes	−	ratios, bars
pink	yes	+	*p*- , *q*-Values
light green	yes	−	

Dot Plots Dot Plots

Protein	*FnSg* vs *Fn*				Raw		Normalized		Description
	Log_2 Ratio	Log_2 Sum	*q*-Value	*p*-Value	*FnSg*	*Fn*	*FnSg*	*Fn*	
**FN1186**	4.461	12.950	1.95e-5	1.59e-5	308	12	400.8979	15.5388	AAL95382.1| Amidohydrolase
					434	16	434.0000	22.3746	
**FN1187**					85		110.6374		AAL95383.1| Amino acid-binding protein
					61		61.0000		
**FN1188**	0.226	13.192	1.158e-2	5.527e-2	87	69	113.2406	89.3481	AAL95384.1| Hypothetical protein
					96	64	96.0000	89.4982	
**FN1189**	-0.599	12.549	5.336e-3	2.229e-2	59	77	76.7954	99.7073	AAL95385.1| Hypothetical protein
					49	65	49.0000	90.8966	
**FN1190**	0.102	14.748	2.297e-3	8.066e-3	135	121	175.7182	156.6829	AAL95386.1| Probable cadmium-transporting ATPase
					168	117	168.0000	163.6139	
**FN1191**	1.230	12.311	5.734e-3	2.423e-2	104	33	135.3681	42.7317	AAL95387.1| unknown
					83	36	83.0000	50.3427	
**FN1192**	0.877	17.961	2.91e-3	1.08e-2	451	298	587.0291	385.8803	AAL95388.1| unknown
					782	257	782.0000	359.3913	
**FN1198**	1.280	10.701	1.881e-5	1.503e-5	47	21	61.1760	27.1929	AAL95394.1| Transporter
					66	18	66.0000	25.1714	
**FN1200**					253		329.3090		AAL95396.1| Hypothetical protein
					252		252.0000		
**FN1201**									AAL95397.1| unknown
					7		7.0000		
**FN1202**	1.295	8.041			26	8	33.8420	10.3592	AAL95398.1| NH(3)-dependent NAD(+) synthetase
					17		17.0000		
**FN1203**	-0.412	7.199			10		13.0162		AAL95399.1| GTP-binding protein
					8	10	8.0000	13.9841	
**FN1204**	0.512	7.398	1.83e-2	9.29e-2	10	6	13.0162	7.7694	AAL95400.1| Methyltransferase
					18	10	18.0000	13.9841	
**FN1205**	-0.761	12.337	9.387e-3	4.337e-2	58	69	75.4938	89.3481	AAL95401.1| Protease
					35	70	35.0000	97.8887	
**FN1206**									AAL95402.1| Hemolysin
						8		11.1873	
**FN1208**	0.580	7.594				10		12.9490	AAL95404.1| 1-deoxyxylulose-5-phosphate synthase
					17	7	17.0000	9.7889	

Log_2 Ratios: -6 -4 -2 0 2 4 6

Show detected proteins only
Show all proteins
Filter by category:
GO: amino acid transport

Proteins found: 1358

Enter (or paste) list of ORFs

Find ORFs

Test	Cutoff
q-Value	.005
p-Value	

Signif	Direction	Applies To
yes	+	ratios, bars
no	n/a	bars
yes	–	ratios, bars
yes	+	*p*- , *q*-Values
yes	–	

Dot Plots Dot Plots

Protein	*FnSg* vs *Fn*				Raw		Normalized		Description
	Log_2 Ratio	Log_2 Sum	*q*-Value	*p*-Value	*FnSg*	*Fn*	*FnSg*	*Fn*	
FN1209	-1.872	10.844	2.67e-3	9.698e-3	16	77	20.8259	99.7073	AAL95405.1| Hypothetical RNA binding protein
					24	46	24.0000	64.3268	
FN1210	-1.032	12.066	5.966e-3	2.538e-2	45	55	58.5727	71.2195	AAL95406.1| Metal dependent hydrolase
					33	83	33.0000	116.0680	
FN1211	-0.699	8.252	3.479e-3	1.353e-2	8	16	10.4129	20.7184	AAL95407.1| Cell division protein ftsI
					17	17	17.0000	23.7730	
FN1213	-0.421	12.327	2.328e-3	8.199e-3	46	59	59.8744	76.3991	AAL95409.1| Hypothetical protein
					64	64	64.0000	89.4982	
FN1216	-0.881	9.049	1.107e-2	5.249e-2	13	18	16.9210	23.3082	AAL95412.1| RRF2 family protein
					17	28	17.0000	39.1555	
FN1217	-0.122	9.171	9.118e-2	5.332e-1	20	16	26.0323	20.7184	AAL95413.1| Holliday junction DNA helicase ruvB
					20	21	20.0000	29.3666	
FN1218					13		16.9210		AAL95414.1| unknown
					19		19.0000		
FN1219	0.075	7.863	9.847e-2	5.804e-1	11	10	14.3178	12.9490	AAL95415.1| Hypothetical protein
					17	12	17.0000	16.7809	
FN1220	0.134	15.776	9.04e-2	5.281e-1	150	183	195.2425	236.9667	AAL95416.1| Cysteine synthase
					301	154	301.0000	215.3551	
FN1221	0.628	9.110	1.698e-2	8.559e-2	28	13	36.4453	16.8337	AAL95417.1| Hypothetical protein
					22	15	22.0000	20.9761	
FN1222	-2.965	7.609				29		37.5521	AAL95418.1| Hypothetical protein
					5	29	5.0000	40.5539	
FN1223	-1.197	10.940	1.036e-5	6.173e-6	25	54	32.5404	69.9246	AAL95419.1| Oxygen-insensitive NAD(P)H nitroreductase
					26	46	26.0000	64.3268	
FN1224	-1.022	13.632	4.285e-4	1.006e-3	47	113	61.1760	146.3237	AAL95420.1| 2-dehydro-3-deoxyphosphooctonate aldolase
					97	125	97.0000	174.8012	
FN1225	-0.427	13.491	1.198e-2	5.752e-2	60	82	78.0970	106.1818	AAL95421.1| UDP-N-acetylmuramoyl-L-alanyl-D-glutamate--meso-lanthionine ligase
					107	102	107.0000	142.6378	
FN1226	0.365	15.117	2.48e-2	1.297e-1	195	127	253.8152	164.4523	AAL95422.1| Uracil-DNA glycosylase
					174	120	174.0000	167.8092	
FN1229						5		6.4745	AAL95425.1| Hypothetical protein
						7		9.7889	

Log_2 Ratios: -6 -4 -2 0 2 4 6

Show detected proteins only
Show all proteins
Filter by category:
GO: amino acid transport

Proteins found: 1358

Enter (or paste) list of ORFs

Find ORFs

Test	Cutoff
q-Value	.005
p-Value	

	Signif	Direction	Applies To
red	yes	+	ratios, bars
yellow	no	n/a	bars
green	yes	−	ratios, bars
pink	yes	+	*p*- , *q*-Values
light green	yes	−	*p*- , *q*-Values

Dot Plots Dot Plots

Protein	FnSg vs Fn Log2 Ratio	Log2 Sum	q-Value	p-Value	Raw FnSg	Raw Fn	Normalized FnSg	Normalized Fn	Description	Log2 Ratios (-6 -4 -2 0 2 4 6)
FN1230						50		64.7450	AAL95426.1| Hypothetical cytosolic protein	
						43		60.1316		
FN1231	0.921	19.191	1.093e-4	1.752e-4	788	431	1025.6739	558.1020	AAL95427.1| Inosine-5'-monophosphate dehydrogenase	
					1104	405	1104.0000	566.3559		
FN1233	0.340	4.830				3		3.8847	AAL95429.1| Putative NAD(P)H oxidoreductase	
					6	4	6.0000	5.5936		
FN1234	0.160	8.625				15		19.4235	AAL95430.1| Hypothetical protein	
					21	13	21.0000	18.1793		
FN1235	1.768	8.128			29	7	37.7469	9.0643	AAL95431.1| Ankyrin repeat proteins	
					24		24.0000			
FN1237	2.287	11.853	2.375e-4	4.73e-4	112	22	145.7811	28.4878	AAL95433.1| Choline kinase	
					123	19	123.0000	26.5698		
FN1238									AAL95434.1| Hypothetical protein	
					4		4.0000			
FN1240	-2.598	8.173	6.78e-5	9.412e-5	6	30	7.8097	38.8470	AAL95436.1| Lipopolysaccharide core biosynthesis protein rfaY	
					6	32	6.0000	44.7491		
FN1241	0.491	6.482	1.888e-2	9.616e-2	8	8	10.4129	10.3592	AAL95437.1| polysaccharide biosynthesis protein	
					12	4	12.0000	5.5936		
FN1242	1.104	8.315	6.253e-5	8.35e-5	21	8	27.3339	10.3592	AAL95438.1| Polysaccharide deacetylase	
					25	10	25.0000	13.9841		
FN1243	-0.234	7.523	3.163e-2	1.697e-1	10	13	13.0162	16.8337	AAL95439.1| Glycosyl transferase	
					12	9	12.0000	12.5857		
FN1244	0.771	8.692	3.719e-3	1.473e-2	17	10	22.1275	12.9490	AAL95440.1| Polysaccharide deacetylase	
					31	13	31.0000	18.1793		
FN1245	-1.567	7.344	7.032e-3	3.087e-2	6	22	7.8097	28.4878	AAL95441.1| Glycosyl transferase	
					7	11	7.0000	15.3825		
FN1246	-0.141	8.601	7.953e-2	4.591e-1	15	19	19.5242	24.6031	AAL95442.1| Lipooligosaccharide cholinephosphotransferase	
					18	12	18.0000	16.7809		
FN1247	0.349	9.857	5.002e-2	2.792e-1	19	19	24.7307	24.6031	AAL95443.1| LOS biosynthesis enzyme LBGB	
					44	21	44.0000	29.3666		
FN1248						5		6.4745	AAL95444.1| Hypothetical cytosolic protein	

Show detected proteins only
Show all proteins
Filter by category:
GO: amino acid transport

Proteins found: 1358

Enter (or paste) list of ORFs

Find ORFs

Test	Cutoff
q-Value	.005
p-Value	

	Signif	Direction	Applies To
red	yes	+	ratios, bars
yellow	no	n/a	bars
green	yes	−	ratios, bars
pink	yes	+	p- , q-Values
light green	yes	−	p- , q-Values

Dot Plots Dot Plots

Protein	*FnSg* vs *Fn* Log$_2$ Ratio	Log$_2$ Sum	*q*-Value	*p*-Value	Raw *FnSg*	Raw *Fn*	Normalized *FnSg*	Normalized *Fn*	Description
FN1250	-1.336	10.150	4.866e-4	1.184e-3	18	46	23.4291	59.5654	AAL95446.1| Guanine-hypoxanthine permease
					19	34	19.0000	47.5459	
FN1251	0.025	6.884	1.53e-1	9.601e-1	13	8	16.9210	10.3592	AAL95447.1| High-affinity iron permease
					5	8	5.0000	11.1873	
FN1252	-1.187	18.460	1.159e-4	1.892e-4	361	638	469.8836	826.1464	AAL95448.1| 34 kDa membrane antigen precursor
					326	705	326.0000	985.8788	
FN1253	-0.732	16.347	1.505e-4	2.701e-4	186	268	242.1007	347.0333	AAL95449.1| unknown
					206	284	206.0000	397.1484	
FN1254	-1.036	12.873	8.79e-5	1.331e-4	53	103	68.9857	133.3747	AAL95450.1| Oxygen-insensitive NAD(P)H nitroreductase
					52	82	52.0000	114.6696	
FN1255						15		19.4235	AAL95451.1| NagD protein
						14		19.5777	
FN1256						5		6.4745	AAL95452.1| C4-dicarboxylate transporter large subunit
FN1258	-0.557	20.198	1.495e-4	2.677e-4	741	1049	964.4979	1358.3504	AAL95454.1| C4-dicarboxylate-binding protein
					844	931	844.0000	1301.9194	
FN1259									AAL95455.1| hypothetical protein
					9		9.0000		
FN1260						4		5.1796	AAL95456.1| Sensory Transduction Protein Kinase
FN1261									AAL95457.1| Two-component response regulator
						4		5.5936	
FN1262	1.407	6.796			21	5	27.3339	6.4745	AAL95458.1| Integral membrane protein
					7		7.0000		
FN1263	-0.159	9.189	1.724e-2	8.706e-2	19	20	24.7307	25.8980	AAL95459.1| Cobalt chelatase
					21	18	21.0000	25.1714	
FN1264	-0.747	8.802	6.873e-3	3.003e-2	12	25	15.6194	32.3725	AAL95460.1| Hypothetical protein
					17	16	17.0000	22.3746	
FN1265	-0.208	13.076	3.209e-2	1.724e-1	76	83	98.9229	107.4767	AAL95461.1| Outer membrane protein
					74	66	74.0000	92.2950	
FN1266	-0.726	14.326	1.444e-3	4.639e-3	99	140	128.8600	181.2860	AAL95462.1| UTP--glucose-1-phosphate uridylyltransferase
					94	134	94.0000	187.3869	

Log$_2$ Ratios: -6 -4 -2 0 2 4 6

Show detected proteins only
Show all proteins
Filter by category:
GO: amino acid transport

Proteins found: 1358

Enter (or paste) list of ORFs

Find ORFs

Test	Cutoff
q-Value	.005
p-Value	

Signif	Direction	Applies To
yes	+	ratios, bars
no	n/a	bars
yes	–	ratios, bars
yes	+	*p*- , *q*-Values
yes	–	

Dot Plots Dot Plots

	FnSg vs *Fn*				Raw		Normalized			Log$_2$ Ratios
Protein	Log$_2$ Ratio	Log$_2$ Sum	*q*-Value	*p*-Value	*FnSg*	*Fn*	*FnSg*	*Fn*	Description	-6 -4 -2 0 2 4 6
**FN1267**	-0.323	10.440	3.336e-2	1.8e-1	32	32	41.6517	41.4368	AAL95463.1| Hypothetical protein	
					25	30	25.0000	41.9523		
**FN1268**	-1.071	15.511	1.415e-3	4.529e-3	120	272	156.1940	352.2129	AAL95464.1| Methionyl-tRNA synthetase	
					142	196	142.0000	274.0883		
**FN1269**	-0.946	7.322			7	12	9.1113	15.5388	AAL95465.1| Hypothetical lipoprotein	
						14		19.5777		
**FN1270**	-3.588	10.689			9	115	11.7145	148.9135	AAL95466.1| Hypothetical cytosolic protein	
						95		132.8489		
**FN1271**	-0.271	11.721	3.333e-2	1.799e-1	49	50	63.7792	64.7450	AAL95467.1| Protease IV	
					42	45	42.0000	62.9284		
**FN1273**	0.531	11.522	2.203e-2	1.139e-1	51	47	66.3824	60.8603	AAL95469.1| Outer membrane protein tolC	
					64	21	64.0000	29.3666		
**FN1274**	-1.250	10.223	5.095e-4	1.258e-3	16	37	20.8259	47.9113	AAL95470.1| Acriflavin resistance protein E	
					24	42	24.0000	58.7332		
**FN1275**	-1.572	10.413	2.811e-4	5.884e-4	16	53	20.8259	68.6297	AAL95471.1| Acriflavin resistance protein B	
					22	42	22.0000	58.7332		
**FN1276**					13		16.9210		AAL95472.1| Hypothetical protein	
					22		22.0000			
**FN1277**	0.267	15.369	6.773e-3	2.95e-2	160	133	208.2587	172.2217	AAL95473.1| Aminoacyl-histidine dipeptidase	
					243	145	243.0000	202.7694		
**FN1278**									AAL95474.1| Acetyltransferase	
					11		11.0000			
**FN1279**	-0.890	12.061	1.02e-4	1.603e-4	40	64	52.0647	82.8736	AAL95475.1| Zinc metallohydrolase, glyoxalase II family	
					44	68	44.0000	95.0919		
**FN1280**	3.038	10.375	3.156e-4	6.873e-4	89	11	115.8439	14.2439	AAL95476.1| Serine protease, V8 family	
					93	8	93.0000	11.1873		
**FN1281**	0.629	9.636	4.332e-3	1.759e-2	27	21	35.1436	27.1929	AAL95477.1| Cysteine protease	
					35	13	35.0000	18.1793		
**FN1282**	0.250	12.338	1.182e-2	5.664e-2	66	49	85.9067	63.4501	AAL95478.1| LSU ribosomal protein L17P	
					71	49	71.0000	68.5221		
**FN1283**	0.423	18.195	1.589e-2	7.951e-2	404	383	525.8531	495.9468	AAL95479.1| DNA-directed RNA polymerase alpha chain	
					743	322	743.0000	450.2879		

Show detected proteins only
Show all proteins
Filter by category:
GO: amino acid transport

Proteins found: 1358

Enter (or paste) list of ORFs

Find ORFs

Test	Cutoff
q-Value	.005
p-Value	

	Signif	Direction	Applies To
red	yes	+	ratios, bars
yellow	no	n/a	bars
green	yes	−	ratios, bars
light red	yes	+	*p*-, *q*-Values
light green	yes	−	*p*-, *q*-Values

Dot Plots Dot Plots

Protein	*FnSg* vs *Fn* Log$_2$ Ratio	Log$_2$ Sum	*q*-Value	*p*-Value	Raw *FnSg*	Raw *Fn*	Normalized *FnSg*	Normalized *Fn*	Description
FN1284	-1.208	18.516	3.448e-7	3.598e-8	324	705	421.7238	912.9047	AAL95480.1| SSU ribosomal protein S4P
					384	678	384.0000	948.1218	
FN1285	-2.246	11.238	2.49e-5	2.234e-5	17	80	22.1275	103.5920	AAL95481.1| SSU ribosomal protein S11P
					23	79	23.0000	110.4744	
FN1286	-2.156	15.369	1.562e-6	3.129e-7	66	323	85.9067	418.2528	AAL95482.1| SSU ribosomal protein S13P
					109	322	109.0000	450.2879	
FN1287	-2.227	8.844	5.401e-5	6.771e-5	6	36	7.8097	46.6164	AAL95483.1| Bacterial Protein Translation Initiation Factor 1 (IF-1)
					12	33	12.0000	46.1475	
FN1290	-0.935	9.035	3.475e-3	1.35e-2	17	23	22.1275	29.7827	AAL95486.1| Hypothetical protein
					11	24	11.0000	33.5618	
FN1293	-0.864	5.625			4	6	5.2065	7.7694	AAL95489.1| Hypothetical protein
						8		11.1873	
FN1296						29		37.5521	AAL95492.1| unknown
						16		22.3746	
FN1297	-0.261	10.208	5.042e-3	2.088e-2	26	29	33.8420	37.5521	AAL95493.1| Methionine aminopeptidase
					29	27	29.0000	37.7571	
FN1298	-1.411	12.551	2.195e-3	7.624e-3	53	99	68.9857	128.1951	AAL95494.1| Adenylate kinase
					26	89	26.0000	124.4585	
FN1299					4		5.2065		AAL95495.1| dTDP-glucose 4,6-dehydratase
					7		7.0000		
FN1301	-1.250	12.528	5.191e-6	2.015e-6	42	88	54.6679	113.9512	AAL95497.1| ABC transporter ATP-binding protein
					45	88	45.0000	123.0601	
FN1302	-1.858	15.374	1.188e-3	3.661e-3	81	349	105.4309	451.9202	AAL95498.1| Hypothetical protein
					111	238	111.0000	332.8215	
FN1303	-0.799	12.879	3.786e-5	4.019e-5	55	85	71.5889	110.0665	AAL95499.1| hypothetical cytosolic protein
					60	85	60.0000	118.8648	
FN1304	-0.525	14.306	1.29e-4	2.185e-4	87	133	113.2406	172.2217	AAL95500.1| Single-strand DNA binding protein
					124	121	124.0000	169.2076	
FN1305	-0.035	10.780	1.491e-1	9.309e-1	46	31	59.8744	40.1419	AAL95501.1| Hypothetical cytosolic protein
					23	32	23.0000	44.7491	
FN1306	1.030	14.306	2.074e-3	7.116e-3	135	75	175.7182	97.1175	AAL95502.1| Methyltransferase
					231	73	231.0000	102.0839	

Log$_2$ Ratios: -6 -4 -2 0 2 4 6

Show detected proteins only
Show all proteins
Filter by category:
GO: amino acid transport

Proteins found: 1358

Enter (or paste) list of ORFs

Find ORFs

Test	Cutoff
q-Value	.005
p-Value	

	Signif	Direction	Applies To
red	yes	+	ratios, bars
yellow	no	n/a	bars
green	yes	−	ratios, bars
light red	yes	+	*p*- , *q*-Values
light green	yes	−	*p*- , *q*-Values

Dot Plots Dot Plots

Protein	*FnSg* vs *Fn* Log$_2$ Ratio	Log$_2$ Sum	*q*-Value	*p*-Value	Raw *FnSg*	Raw *Fn*	Normalized *FnSg*	Normalized *Fn*	Description
FN1309	-1.337	15.556	2.779e-4	5.794e-4	87	272	113.2406	352.2129	AAL95505.1| Hypothetical protein
					163	247	163.0000	345.4072	
FN1311									AAL95507.1| Biopolymer transport exbD protein
						8		11.1873	
FN1312						44		56.9756	AAL95508.1| Biopolymer transport exbB protein
						52		72.7173	
FN1313	-0.678	12.885	1.501e-4	2.691e-4	58	90	75.4938	116.5410	AAL95509.1| Oligopeptide-binding protein oppA
					62	74	62.0000	103.4823	
FN1315	-0.215	6.145			6	7	7.8097	9.0643	AAL95511.1| Hypothetical protein
FN1317	-0.766	7.607	6.868e-4	1.857e-3	8	13	10.4129	16.8337	AAL95513.1| RNA polymerase sigma factor
					11	14	11.0000	19.5777	
FN1318	-0.955	9.979	9.638e-4	2.849e-3	22	37	28.6356	47.9113	AAL95514.1| RNA polymerase sigma factor rpoD
					17	29	17.0000	40.5539	
FN1319	0.640	8.531	4.834e-2	2.689e-1	10	13	13.0162	16.8337	AAL95515.1| DNA primase
					35	10	35.0000	13.9841	
FN1320	-1.364	16.471	5.345e-5	6.673e-5	168	385	218.6716	498.5366	AAL95516.1| Peptidyl-prolyl cis-trans isomerase
					157	335	157.0000	468.4673	
FN1321	0.313	20.055	7.656e-4	2.157e-3	849	752	1105.0725	973.7650	AAL95517.1| Acetoacetate metabolism regulatory protein atoC
					1221	643	1221.0000	899.1774	
FN1322	0.166	8.530	7.725e-2	4.449e-1	19	14	24.7307	18.1286	AAL95518.1| Membrane metalloprotease
					16	13	16.0000	18.1793	
FN1323	-1.237	7.014	7.975e-6	4.09e-6	6	14	7.8097	18.1286	AAL95519.1| Thymidylate kinase
					7	12	7.0000	16.7809	
FN1324	0.430	10.702	4.974e-3	2.057e-2	39	23	50.7630	29.7827	AAL95520.1| 1-deoxy-D-xylulose 5-phosphate reductoisomerase
					44	29	44.0000	40.5539	
FN1326	-1.212	6.826				11		14.2439	AAL95522.1| Undecaprenyl pyrophosphate synthetase
					7	13	7.0000	18.1793	
FN1327	1.946	6.083			11		14.3178		AAL95523.1| Dimethylallyltransferase
					18	3	18.0000	4.1952	
FN1328						11		14.2439	AAL95524.1| Exodeoxyribonuclease VII small subunit
						12		16.7809	

Log$_2$ Ratios: -6 -4 -2 0 2 4 6

Show detected proteins only
Show all proteins
Filter by category:
GO: amino acid transport

Proteins found: 1358

Enter (or paste) list of ORFs

Find ORFs

Test	Cutoff
q-Value	.005
p-Value	

	Signif	Direction	Applies To
red	yes	+	ratios, bars
yellow	no	n/a	bars
green	yes	−	ratios, bars
pink	yes	+	*p*- , *q*-Values
light green	yes	−	*p*- , *q*-Values

Dot Plots Dot Plots

Protein	*FnSg* vs *Fn* Log₂ Ratio	Log₂ Sum	*q*-Value	*p*-Value	Raw *FnSg*	Raw *Fn*	Normalized *FnSg*	Normalized *Fn*	Description
FN1330	0.042	3.958				3		3.8847	AAL95526.1| S-adenosylmethionine:tRNA ribosyltransferase-isomerase
					4		4.0000		
FN1331						5		6.4745	AAL95527.1| Methyltransferase
						8		11.1873	
FN1332	-1.174	11.189	2.072e-4	3.995e-4	21	57	27.3339	73.8093	AAL95528.1| Bacterial Peptide Chain Release Factor 1 (RF-1)
					37	51	37.0000	71.3189	
FN1333					7		9.1113		AAL95529.1| Hypothetical protein
					18		18.0000		
FN1334	-1.623	6.842	6.51e-6	3.013e-6	4	15	5.2065	19.4235	AAL95530.1| N-acetylmuramoyl-L-alanine amidase
					7	13	7.0000	18.1793	
FN1335	-1.236	12.265	2.61e-3	9.432e-3	18	95	23.4291	123.0155	AAL95531.1| Protein translocase subunit YajC
					68	66	68.0000	92.2950	
FN1336	-1.084	8.699				21		27.1929	AAL95532.1| Hypothetical protein
					14	23	14.0000	32.1634	
FN1337	-2.361	7.976				21		27.1929	AAL95533.1| unknown
					7	32	7.0000	44.7491	
FN1340	-0.680	16.630	3.063e-4	6.604e-4	173	288	225.1797	372.9313	AAL95536.1| Glutamyl-tRNA synthetase
					278	310	278.0000	433.5070	
FN1341	-0.459	7.432	2.206e-3	7.673e-3	8	13	10.4129	16.8337	AAL95537.1| Bacterial Peptide Chain Release Factor 2 (RF-2)
					12	10	12.0000	13.9841	
FN1343	0.735	8.881			20	13	26.0323	16.8337	AAL95539.1| seC-independent protein TATD
					30		30.0000		
FN1345									AAL95541.1| 2-hydroxy-6-oxo-6-phenylhexa-2,4-dienoate hydrolase
					4		4.0000		
FN1346					15		19.5242		AAL95542.1| Hypothetical cytosolic protein
					19		19.0000		
FN1347	-2.035	7.547	5.083e-4	1.254e-3	5	19	6.5081	24.6031	AAL95543.1| Hypothetical cytosolic protein
					7	22	7.0000	30.7650	
FN1348	0.261	9.781	3.153e-2	1.691e-1	23	17	29.9372	22.0133	AAL95544.1| ABC transporter ATP-binding protein
					35	23	35.0000	32.1634	
FN1349	-2.175	8.687	1.369e-4	2.374e-4	7	31	9.1113	40.1419	AAL95545.1| ABC transporter permease protein
					10	33	10.0000	46.1475	

Log₂ Ratios: -6 -4 -2 0 2 4 6

Show detected proteins only
Show all proteins
Filter by category:
GO: amino acid transport

Proteins found: 1358

Enter (or paste) list of ORFs

Find ORFs

Test	Cutoff
q-Value	.005
p-Value	

	Signif	Direction	Applies To
red	yes	+	ratios, bars
yellow	no	n/a	bars
green	yes	−	ratios, bars
pink	yes	+	*p*-, *q*-Values
light green	yes	−	

Dot Plots Dot Plots

Protein	FnSg vs Fn Log$_2$ Ratio	Log$_2$ Sum	*q*-Value	*p*-Value	Raw *FnSg*	Raw *Fn*	Normalized *FnSg*	Normalized *Fn*	Description
FN1351	-0.432	11.235	3.188e-2	1.711e-1	35	33	45.5566	42.7317	AAL95547.1| 15 kDa lipoprotein precursor
					39	51	39.0000	71.3189	
FN1352	-0.474	11.918	3.852e-3	1.541e-2	35	56	45.5566	72.5144	AAL95548.1| ABC transporter ATP-binding protein
					60	53	60.0000	74.1157	
FN1353	-0.205	7.892	1.059e-1	6.293e-1	9	18	11.7145	23.3082	AAL95549.1| ABC transporter permease protein
					17	7	17.0000	9.7889	
FN1354	-0.699	9.626	3.802e-3	1.516e-2	17	24	22.1275	31.0776	AAL95550.1| ABC transporter permease protein
					22	29	22.0000	40.5539	
FN1355						4		5.1796	AAL95551.1| Integral membrane protein
						7		9.7889	
FN1358					22		28.6356		AAL95554.1| Hypothetical protein
					46		46.0000		
FN1359	2.297	17.119	1.388e-3	4.424e-3	760	116	989.2286	150.2084	AAL95555.1| Dipeptide-binding protein
					683	136	683.0000	190.1837	
FN1362					76		98.9229		AAL95558.1| Dipeptide transport ATP-binding protein dppD
					82		82.0000		
FN1363	2.705	9.941	2.934e-4	6.228e-4	70	6	91.1132	7.7694	AAL95559.1| Dipeptide transport ATP-binding protein dppF
					69	12	69.0000	16.7809	
FN1364						79		102.2971	AAL95560.1| LSU ribosomal protein L32P
						86		120.2632	
FN1365	0.155	15.281	6.373e-2	3.617e-1	136	156	177.0199	202.0044	AAL95561.1| GTP-binding protein
					244	126	244.0000	176.1996	
FN1366	-0.778	14.823	8.148e-4	2.328e-3	83	190	108.0342	246.0310	AAL95562.1| Triosephosphate isomerase
					152	143	152.0000	199.9726	
FN1368									AAL95564.1| COMF operon protein 3
					4		4.0000		
FN1371									AAL95567.1| Ribonuclease HII
					5		5.0000		
FN1373									AAL95569.1| regulatory protein
					5		5.0000		
FN1374	-0.819	6.819				11		14.2439	AAL95570.1| Transcriptional regulator
					8	10	8.0000	13.9841	

Log$_2$ Ratios: -6 -4 -2 0 2 4 6

Show detected proteins only
Show all proteins
Filter by category:
GO: amino acid transport

Proteins found: 1358

Enter (or paste) list of ORFs

Find ORFs

Test	Cutoff
q-Value	.005
p-Value	

	Signif	Direction	Applies To
red	yes	+	ratios, bars
yellow	no	n/a	bars
green	yes	−	ratios, bars
pink	yes	+	*p*- , *q*-Values
light green	yes	−	

Dot Plots Dot Plots

Protein	FnSg vs Fn Log$_2$ Ratio	Log$_2$ Sum	*q*-Value	*p*-Value	Raw *FnSg*	Raw *Fn*	Normalized *FnSg*	Normalized *Fn*	Description	Log$_2$ Ratios (-6 -4 -2 0 2 4 6)
FN1375	0.467	10.557	2.935e-2	1.562e-1	44	24	57.2711	31.0776	AAL95571.1| Citrate-sodium symport	
					34	25	34.0000	34.9602		
FN1376	0.702	19.411	1.056e-2	4.97e-2	647	510	842.1459	660.3991	AAL95572.1| Oxaloacetate decarboxylase alpha chain	
					1288	464	1288.0000	648.8621		
FN1377					9		11.7145		AAL95573.1| CITG protein	
FN1378	-0.568	10.247			22	31	28.6356	40.1419	AAL95574.1| Citrate lyase acyl carrier protein	
						32		44.7491		
FN1379	0.954	16.471	1.937e-5	1.573e-5	328	179	426.9302	231.7871	AAL95575.1| Citrate lyase beta chain	
					412	144	412.0000	201.3710		
FN1380	0.865	18.410	1.552e-2	7.749e-2	431	344	560.9967	445.4457	AAL95576.1| Citrate lyase beta chain	
					1032	307	1032.0000	429.3118		
FN1381						4		5.1796	AAL95577.1| unknown	
						4		5.5936		
FN1382									AAL95578.1| ATPase	
					7		7.0000			
FN1383					11		14.3178		AAL95579.1| DNA polymerase III alpha subunit	
					16		16.0000			
FN1385	0.228	6.718	1.125e-1	6.736e-1	4	6	5.2065	7.7694	AAL95581.1| Hypothetical protein	
					17	8	17.0000	11.1873		
FN1386	1.846	7.654	2.145e-3	7.413e-3	16	4	20.8259	5.1796	AAL95582.1| SWF/SNF family helicase	
					33	7	33.0000	9.7889		
FN1391	-1.705	12.244	3.054e-4	6.578e-4	37	110	48.1598	142.4390	AAL95584.1| Acetyltransferase	
					29	78	29.0000	109.0760		
FN1392	-1.845	13.878	1.15e-8	1.589e-10	48	181	62.4776	234.3769	AAL95585.1| SSU ribosomal protein S16P	
					67	165	67.0000	230.7376		
FN1393	-0.634	11.554	1.046e-3	3.141e-3	30	58	39.0485	75.1042	AAL95586.1| Signal recognition particle, subunit FFH/SRP54	
					49	44	49.0000	61.5300		
FN1397	-1.981	13.445	3.795e-6	1.258e-6	44	161	57.2711	208.4789	AAL95590.1| Glutaminase	
					49	151	49.0000	211.1599		
FN1398	-1.505	12.465	3.555e-3	1.391e-2	34	119	44.2550	154.0931	AAL95591.1| Amino acid carrier protein alsT	
					45	71	45.0000	99.2871		

Show detected proteins only
Show all proteins
Filter by category:
GO: amino acid transport

Proteins found: 1358

Enter (or paste) list of ORFs

Find ORFs

Test	Cutoff
q-Value	.005
p-Value	

	Signif	Direction	Applies To
red	yes	+	ratios, bars
yellow	no	n/a	bars
green	yes	−	ratios, bars
pink	yes	+	*p*- , *q*-Values
light green	yes	−	*p*- , *q*-Values

Dot Plots Dot Plots

FnSg vs Fn

Fusobacterium nucleatum

Hackett Laboratory UW

Fn Summary Table | FnPg vs Fn | FnSg vs Fn | FnPgSg vs Fn | FnPgSg vs FnPg | FnSg vs FnPg | FnPgSg vs FnSg | Fn Coverage

Protein	*FnSg* vs *Fn* Log$_2$ Ratio	Log$_2$ Sum	*q*-Value	*p*-Value	Raw *FnSg*	Raw *Fn*	Normalized *FnSg*	Normalized *Fn*	Description
FN1399									AAL95592.1| Hypothetical cytosolic protein
					3		3.0000		
FN1406	0.273	12.219	1.037e-2	4.869e-2	59	43	76.7954	55.6807	AAL95599.1| Histidine ammonia-lyase
					75	50	75.0000	69.9205	
FN1407	1.445	8.655	9.711e-3	4.519e-2	34	8	44.2550	10.3592	AAL95600.1| Glutamate formiminotransferase
					22	10	22.0000	13.9841	
FN1411	2.355	16.868	1.15e-8	1.52e-10	594	112	773.1602	145.0288	AAL95604.1| Threonine dehydratase
					791	115	791.0000	160.8171	
FN1412					6		7.8097		AAL95605.1| 5-methylthioribose kinase
					10		10.0000		
FN1413									AAL95606.1| Translation initiation factor EIF-2B subunit 1
					4		4.0000		
FN1415									AAL95608.1| NADH-dependent butanol dehydrogenase A
					13		13.0000		
FN1416									AAL95609.1| Transcriptional regulator, GntR family
					4		4.0000		
FN1417	0.839	5.807			10		13.0162		AAL95610.1| L-fuculose phosphate aldolase
					7	4	7.0000	5.5936	
FN1418	-0.696	4.271			3		3.9048		AAL95611.1| Transcriptional regulator, GntR family
					3	4	3.0000	5.5936	
FN1419	2.422	21.045	1.67e-3	5.504e-3	2104	475	2738.6012	615.0776	AAL95612.1| Methionine gamma-lyase
					4072	469	4072.0000	655.8542	
FN1421	0.598	20.931	6.024e-5	7.904e-5	1356	842	1764.9921	1090.3060	AAL95614.1| Pyruvate-flavodoxin oxidoreductase
					1714	864	1714.0000	1208.2260	
FN1423	0.373	15.542	1.053e-4	1.669e-4	186	155	242.1007	200.7095	AAL95616.1| Flavoprotein
					255	131	255.0000	183.1917	
FN1424	0.371	17.677	4.366e-3	1.774e-2	362	329	471.1852	426.0222	AAL95617.1| ACYL-COA dehydrogenase, short-chain specific
					570	271	570.0000	378.9690	
FN1426	-1.045	17.652	1.195e-6	1.975e-7	232	496	301.9750	642.2705	AAL95619.1| Serine protease
					330	473	330.0000	661.4478	
FN1429						7		9.0643	AAL95622.1| Branched-chain amino acid transport ATP-binding protein livG

Log$_2$ Ratios: -6 -4 -2 0 2 4 6

Show detected proteins only
Show all proteins
Filter by category:
GO: amino acid transport

Proteins found: 1358

Enter (or paste) list of ORFs

Find ORFs

Test	Cutoff
q-Value	.005
p-Value	

	Signif	Direction	Applies To
red	yes	+	ratios, bars
yellow	no	n/a	bars
green	yes	−	ratios, bars
pink	yes	+	*p*- , *q*-Values
light green	yes	−	

Dot Plots Dot Plots

Protein	FnSg vs Fn Log$_2$ Ratio	Log$_2$ Sum	q-Value	p-Value	Raw FnSg	Raw Fn	Normalized FnSg	Normalized Fn	Description
FN1432	-3.913	7.913				52		67.3348	AAL95625.1| Leucine-, isoleucine-, valine-, threonine-, and alanine-binding protein
					4	38	4.0000	53.1396	
FN1433	-0.433	15.634	7.703e-4	2.176e-3	150	193	195.2425	249.9157	AAL95626.1| Short chain dehydrogenase
					193	196	193.0000	274.0883	
FN1434	-0.115	14.548	2.146e-2	1.107e-1	114	117	148.3843	151.5033	AAL95627.1| Tetratricopeptide repeat family protein
					149	122	149.0000	170.6060	
FN1437	-2.113	14.242	4.812e-6	1.785e-6	56	215	72.8905	278.4036	AAL95630.1| LSU ribosomal protein L28P
					61	215	61.0000	300.6581	
FN1439	-3.837	10.146	9.237e-4	2.705e-3	6	81	7.8097	104.8869	AAL95632.1| Transcriptional regulator, DeoR family
					10	107	10.0000	149.6298	
FN1440	-2.917	11.540	7.387e-5	1.075e-4	9	128	11.7145	165.7472	AAL95633.1| 1-phosphofructokinase
					28	96	28.0000	134.2473	
FN1441	-0.907	14.935	6.787e-5	9.427e-5	111	195	144.4794	252.5055	AAL95634.1| PTS system, fructose-specific IIABC component
					114	166	114.0000	232.1360	
FN1444	-0.325	17.546	2.296e-3	8.058e-3	274	362	356.6429	468.7539	AAL95637.1| GMP synthase (glutamine-hydrolyzing)
					425	365	425.0000	510.4195	
FN1445	-2.200	10.101	1.068e-3	3.221e-3	13	46	16.9210	59.5654	AAL95638.1| DNA helicase
					14	59	14.0000	82.5062	
FN1448									AAL95641.1| Hypothetical cytosolic protein
					4		4.0000		
FN1449	-4.909	14.158	5.988e-6	2.575e-6	21	560	27.3339	725.1441	AAL95642.1| Fusobacterium outer membrane protein family
					22	541	22.0000	756.5397	
FN1450	0.525	9.952			29	20	37.7469	25.8980	AAL95643.1| Integral membrane protein
						19		26.5698	
FN1451	0.586	15.628	1.133e-4	1.837e-4	204	139	265.5298	179.9911	AAL95644.1| Cell division protein ftsZ
					286	134	286.0000	187.3869	
FN1452	0.362	14.120	6.061e-3	2.585e-2	108	103	140.5746	133.3747	AAL95645.1| Cell division protein ftsA
					162	73	162.0000	102.0839	
FN1454	-0.589	9.784	4.074e-2	2.233e-1	8	26	10.4129	33.6674	AAL95647.1| D-alanine--D-alanine ligase
					38	28	38.0000	39.1555	
FN1455	-0.013	8.644	1.432e-1	8.879e-1	16	17	20.8259	22.0133	AAL95648.1| UDP-N-acetylenolpyruvoylglucosamine reductase
					19	13	19.0000	18.1793	

Log$_2$ Ratios: -6 -4 -2 0 2 4 6

Show detected proteins only
Show all proteins
Filter by category:
GO: amino acid transport

Proteins found: 1358

Enter (or paste) list of ORFs

Find ORFs

Test	Cutoff
q-Value	.005
p-Value	

	Signif	Direction	Applies To
red	yes	+	ratios, bars
yellow	no	n/a	bars
green	yes	−	ratios, bars
pink	yes	+	p- , q-Values
light green	yes	−	

Dot Plots Dot Plots

Protein	*FnSg* vs *Fn* Log$_2$ Ratio	Log$_2$ Sum	*q*-Value	*p*-Value	Raw *FnSg*	Raw *Fn*	Normalized *FnSg*	Normalized *Fn*	Description	Log$_2$ Ratios (-6 -4 -2 0 2 4 6)
FN1456	-0.316	12.360	2.725e-3	9.948e-3	53	58	68.9857	75.1042	AAL95649.1| UDP-N-acetylmuramate--alanine ligase	
					61	62	61.0000	86.7014		
FN1457	-0.428	11.775	2.24e-2	1.161e-1	50	51	65.0808	66.0399	AAL95650.1| UDP-N-acetylglucosamine-N-acetylmuramyl-Pentapeptide pyrophosphoryl-undecaprenol N- [illegible]	
					37	51	37.0000	71.3189		
FN1458	-0.350	12.361	2.451e-2	1.28e-1	38	66	49.4614	85.4634	AAL95651.1| UDP-N-acetylmuramoylalanine--D-glutamate ligase	
					79	56	79.0000	78.3109		
FN1459									AAL95652.1| Phospho-N-acetylmuramoyl-pentapeptide-transferase	
						12		16.7809		
FN1461	-1.210	10.023	5.029e-3	2.083e-2	8	38	10.4129	49.2062	AAL95654.1| Histidinol-phosphatase	
					32	35	32.0000	48.9443		
FN1462					3		3.9048		AAL95655.1| Transcriptional regulator, GntR family	
FN1463	0.404	17.015	5.54e-5	7.014e-5	332	236	432.1367	305.5965	AAL95656.1| pyridoxine biosynthesis protein	
					405	234	405.0000	327.2279		
FN1464	0.524	16.620	6.288e-5	8.419e-5	302	209	393.0882	270.6342	AAL95657.1| 1-deoxyxylulose-5-phosphate synthase	
					368	185	368.0000	258.7058		
FN1470	-4.032	8.649	2.359e-4	4.691e-4	3	69	3.9048	89.3481	AAL95663.1| Hypothetical protein	
					6	52	6.0000	72.7173		
FN1471	-1.987	5.952	7.154e-3	3.152e-2	3	8	3.9048	10.3592	AAL95664.1| LACI-family transcription regulator	
					4	15	4.0000	20.9761		
FN1472	-3.429	12.792	4.04e-4	9.34e-4	21	239	27.3339	309.4812	AAL95665.1| N-acetylneuraminate-binding protein	
					24	174	24.0000	243.3233		
FN1474						8		10.3592	AAL95667.1| N-acetylmannosamine kinase	
						7		9.7889		
FN1475	-2.397	9.041				36		46.6164	AAL95668.1| N-acetylneuraminate lyase	
					10	42	10.0000	58.7332		
FN1476						19		24.6031	AAL95669.1| N-acetylmannosamine-6-phosphate 2-epimerase	
						18		25.1714		
FN1478					16		20.8259		AAL95671.1| Hypothetical protein	
FN1479	-0.758	9.918	2.551e-4	5.183e-4	16	29	20.8259	37.5521	AAL95672.1| Hypothetical protein	
					27	31	27.0000	43.3507		

Show detected proteins only
Show all proteins
Filter by category:
GO: amino acid transport

Proteins found: 1358

Enter (or paste) list of ORFs

Find ORFs

Test	Cutoff
q-Value	.005
p-Value	

Color	Signif	Direction	Applies To
red	yes	+	ratios, bars
yellow	no	n/a	bars
green	yes	–	ratios, bars
pink	yes	+	*p*- , *q*-Values
light green	yes	–	*p*- , *q*-Values

Dot Plots Dot Plots

Protein	FnSg vs Fn Log$_2$ Ratio	Log$_2$ Sum	*q*-Value	*p*-Value	Raw FnSg	Raw Fn	Normalized FnSg	Normalized Fn	Description
FN1480	0.738	9.502	6.281e-4	1.649e-3	25	16	32.5404	20.7184	AAL95673.1| MG2+ transporter MGTE
					37	15	37.0000	20.9761	
FN1481	0.319	6.311	1.055e-1	6.27e-1	3	8	3.9048	10.3592	AAL95674.1| Queuine tRNA-ribosyltransferase
					16	4	16.0000	5.5936	
FN1482	0.667	12.619	3.33e-3	1.279e-2	66	40	85.9067	51.7960	AAL95675.1| Guanosine-3',5'-bis (Diphosphate) 3'-pyrophosphohydrolase
					114	53	114.0000	74.1157	
FN1483	-1.057	10.294	1.05e-4	1.663e-4	17	39	22.1275	50.5011	AAL95676.1| Adenine phosphoribosyltransferase
					27	37	27.0000	51.7412	
FN1484	-0.972	6.825	5.444e-3	2.281e-2	4	9	5.2065	11.6541	AAL95677.1| Tetratricopeptide repeat family protein
					10	13	10.0000	18.1793	
FN1485									AAL95678.1| Transporter
					4		4.0000		
FN1486	-0.572	5.216				5		6.4745	AAL95679.1| magnesium and cobalt efflux protein CorC
					5	6	5.0000	8.3905	
FN1487	-0.972	15.222	1.898e-6	4.186e-7	110	207	143.1778	268.0444	AAL95681.1| Chorismate mutase
					136	200	136.0000	279.6819	
FN1488									AAL95682.1| Methylenetetrahydrofolate dehydrogenase (NADP+)
					17		17.0000		
FN1489	-0.369	9.486	1.483e-2	7.364e-2	17	20	22.1275	25.8980	AAL95683.1| Methionyl-tRNA formyltransferase
					25	25	25.0000	34.9602	
FN1490	1.571	8.471	6.866e-3	2.999e-2	33	5	42.9533	6.4745	AAL95684.1| putative regulatory protein
					22	11	22.0000	15.3825	
FN1491	-0.293	8.675			15		19.5242		AAL95685.1| PTS system, IIA component
					17	16	17.0000	22.3746	
FN1492									AAL95686.1| DNA repair protein recO
					4		4.0000		
FN1493	-2.101	7.150	2.027e-3	6.924e-3	5	23	6.5081	29.7827	AAL95687.1| Hypothetical protein
					5	14	5.0000	19.5777	
FN1494	-1.536	8.430	4.557e-4	1.088e-3	6	24	7.8097	31.0776	AAL95680.1| Rod shape-determining protein mreC
					14	23	14.0000	32.1634	
FN1496	-1.536	8.430	4.557e-4	1.088e-3	6	24	7.8097	31.0776	AAL95680.1| Rod shape-determining protein mreC
					14	23	14.0000	32.1634	

Log$_2$ Ratios: -6 -4 -2 0 2 4 6

Show detected proteins only
Show all proteins
Filter by category:
GO: amino acid transport

Proteins found: 1358

Enter (or paste) list of ORFs

Find ORFs

Test	Cutoff
q-Value	.005
p-Value	

	Signif	Direction	Applies To
red	yes	+	ratios, bars
yellow	no	n/a	bars
green	yes	−	ratios, bars
pink	yes	+	*p*- , *q*-Values
light green	yes	−	*p*- , *q*-Values

Dot Plots | Dot Plots

Protein	*FnSg* vs *Fn* Log$_2$ Ratio	Log$_2$ Sum	*q*-Value	*p*-Value	Raw *FnSg*	Raw *Fn*	Normalized *FnSg*	Normalized *Fn*	Description
FN1499	-1.628	10.800	8.446e-4	2.429e-3	20	65	26.0323	84.1685	AAL93625.1| Cell surface protein
					22	46	22.0000	64.3268	
FN1504	-0.873	14.681	3.772e-3	1.5e-2	121	179	157.4956	231.7871	AAL93630.1| Nickel-binding protein
					82	148	82.0000	206.9646	
FN1505	-0.463	15.961	1.136e-3	3.469e-3	183	215	238.1958	278.4036	AAL93631.1| 6,7-dimethyl-8-ribityllumazine synthase
					192	225	192.0000	314.6422	
FN1506	-0.525	6.865				10		12.9490	AAL93632.1| Diaminohydroxyphosphoribosylaminopyrimidine deaminase
					9		9.0000		
FN1507									AAL93633.1| Riboflavin synthase alpha chain
					8		8.0000		
FN1508	-0.566	12.519	1.493e-2	7.418e-2	33	77	42.9533	99.7073	AAL93634.1| GTP cyclohydrolase II
					83	62	83.0000	86.7014	
FN1512						11		14.2439	AAL93638.1| hypothetical exported 24-amino acid repeat protein
						8		11.1873	
FN1517	-0.956	15.597	5.742e-4	1.469e-3	115	262	149.6859	339.2639	AAL93643.1| Leucyl-tRNA synthetase
					170	201	170.0000	281.0804	
FN1518	-0.740	5.910				9		11.6541	AAL93644.1| RNA polymerase sigma-H factor
					6	6	6.0000	8.3905	
FN1519	-0.504	8.993	1.798e-3	6.002e-3	13	21	16.9210	27.1929	AAL93645.1| 23S rRNA methyltransferase
					21	19	21.0000	26.5698	
FN1520	-1.051	11.266	4.193e-5	4.721e-5	23	52	29.9372	67.3348	AAL93646.1| UDP-N-acetylglucosamine 1-carboxyvinyltransferase
					39	54	39.0000	75.5141	
FN1521						15		19.4235	AAL93647.1| Dipeptide transport system permease protein dppB
						16		22.3746	
FN1523	-2.225	15.021	2.363e-7	2.072e-8	72	299	93.7164	387.1752	AAL93649.1| Dipeptide-binding protein
					75	287	75.0000	401.3436	
FN1524						5		6.4745	AAL93650.1| Dipeptide transport ATP-binding protein dppD
FN1525						5		6.4745	AAL93651.1| Dipeptide transport ATP-binding protein dppF
FN1526	-2.550	20.542	5.399e-6	2.151e-6	332	2415	432.1367	3127.1841	AAL93652.1| Fusobacterium outer membrane protein family
					589	2039	589.0000	2851.3574	

Log$_2$ Ratios: -6 -4 -2 0 2 4 6

Show detected proteins only
Show all proteins
Filter by category:
GO: amino acid transport

Proteins found: 1358

Enter (or paste) list of ORFs

Find ORFs

Test	Cutoff
q-Value	.005
p-Value	

	Signif	Direction	Applies To
red	yes	+	ratios, bars
yellow	no	n/a	bars
green	yes	−	ratios, bars
pink	yes	+	*p*- , *q*-Values
light green	yes	−	

Dot Plots Dot Plots

Protein	FnSg vs Fn Log2 Ratio	Log2 Sum	q-Value	p-Value	Raw FnSg	Raw Fn	Normalized FnSg	Normalized Fn	Description
FN1527	-3.030	12.651	5.453e-5	6.86e-5	17	164	22.1275	212.3636	AAL93653.1| Hypothetical protein
					34	176	34.0000	246.1201	
FN1528	-1.980	13.695	7.134e-5	1.018e-4	33	173	42.9533	224.0177	AAL93654.1| Hypothetical protein
					73	167	73.0000	233.5344	
FN1529	-2.743	10.835	1.903e-5	1.53e-5	20	79	26.0323	102.2971	AAL93655.1| Hypothetical protein
					7	85	7.0000	118.8648	
FN1531	0.931	10.693	2.863e-5	2.691e-5	41	25	53.3663	32.3725	AAL93657.1| murein hydrolase export regulator
					59	19	59.0000	26.5698	
FN1533	-0.564	18.290	6.836e-4	1.846e-3	329	534	428.2318	691.4767	AAL93659.1| Electron transfer flavoprotein alpha-subunit
					503	490	503.0000	685.2208	
FN1534	-0.096	19.572	3.63e-2	1.973e-1	651	657	847.3524	850.7495	AAL93660.1| Electron transfer flavoprotein beta-subunit
					860	697	860.0000	974.6916	
FN1535	0.483	20.907	6.664e-3	2.893e-2	1107	873	1440.8895	1130.4479	AAL93661.1| Acyl-CoA dehydrogenase, short-chain specific
					1875	888	1875.0000	1241.7878	
FN1536	1.421	19.115	1.569e-3	5.121e-3	806	369	1049.1029	477.8182	AAL93662.1| (S)-2-hydroxy-acid oxidase chain D
					1417	317	1417.0000	443.2959	
FN1537	-1.040	5.684				4		5.1796	AAL93663.1| Arsenical pump-driving ATPase
					5	11	5.0000	15.3825	
FN1538	0.712	8.750	4.796e-3	1.973e-2	17	11	22.1275	14.2439	AAL93664.1| Arsenical pump-driving ATPase
					31	13	31.0000	18.1793	
FN1539	-0.327	15.678	1.615e-4	2.967e-4	162	205	210.8619	265.4546	AAL93665.1| Iron-sulfur cluster-binding protein
					198	177	198.0000	247.5185	
FN1540	0.786	16.744	1.611e-4	2.958e-4	355	205	462.0739	265.4546	AAL93666.1| Iron-sulfur cluster-binding protein
					408	171	408.0000	239.1281	
FN1544	1.512	17.022	4.949e-4	1.211e-3	428	162	557.0919	209.7738	AAL93670.1| Probable electron transfer flavoprotein-quinone oxidoreductase ydiS
					675	159	675.0000	222.3471	
FN1545	0.334	10.149	2.383e-2	1.242e-1	32	28	41.6517	36.2572	AAL93671.1| Ferredoxin like protein
					34	17	34.0000	23.7730	
FN1546	0.545	20.470	5.559e-4	1.409e-3	1041	730	1354.9828	945.2772	AAL93672.1| Protein Translation Elongation Factor G (EF-G)
					1557	751	1557.0000	1050.2057	
FN1547	-0.863	14.636	2.552e-5	2.31e-5	95	165	123.6536	213.6585	AAL93673.1| PTS permease for N-acetylglucosamine and glucose
					113	155	113.0000	216.7535	

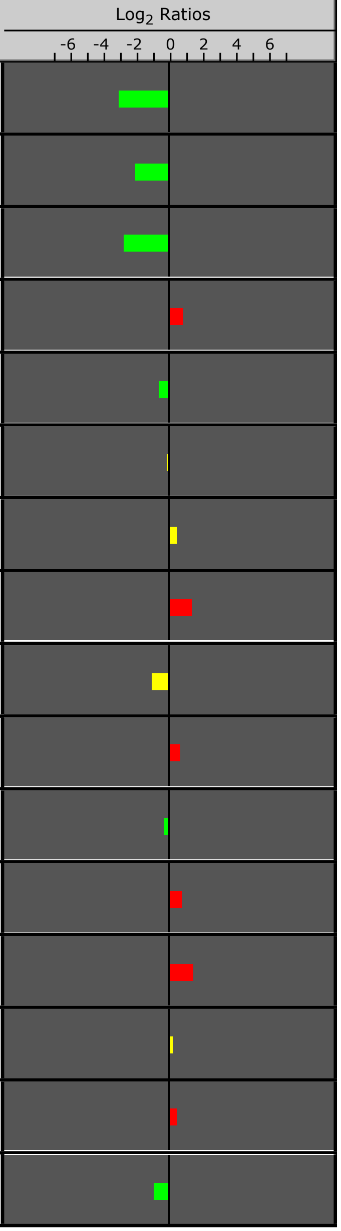

Show detected proteins only
Show all proteins
Filter by category:
GO: amino acid transport

Proteins found: 1358

Enter (or paste) list of ORFs

Find ORFs

Test	Cutoff
q-Value	.005
p-Value	

Color	Signif	Direction	Applies To
red	yes	+	ratios, bars
yellow	no	n/a	bars
green	yes	–	ratios, bars
pink	yes	+	p- , q-Values
light green	yes	–	

Dot Plots | Dot Plots

Protein	*FnSg* vs *Fn* Log$_2$ Ratio	Log$_2$ Sum	*q*-Value	*p*-Value	Raw *FnSg*	Raw *Fn*	Normalized *FnSg*	Normalized *Fn*	Description	Log$_2$ Ratios (-6 -4 -2 0 2 4 6)
FN1548	-0.370	10.345	7.522e-2	4.322e-1	38	32	49.4614	41.4368	AAL93674.1| Hypothetical protein	
					14	29	14.0000	40.5539		
FN1549	-0.254	19.544	4.367e-2	2.407e-1	750	667	976.2124	863.6985	AAL93675.1| Stomatin like protein	
					625	748	625.0000	1046.0105		
FN1552	-0.844	7.184				12		15.5388	AAL93678.1| abortive phage resistance protein	
					9	12	9.0000	16.7809		
FN1553	-0.142	9.062	1.157e-1	6.955e-1	10	17	13.0162	22.0133	AAL93679.1| abortive phage resistance protein	
					31	19	31.0000	26.5698		
FN1554	-1.338	16.473	1.007e-4	1.577e-4	114	337	148.3843	436.3814	AAL93680.1| Fusobacterium outer membrane protein family	
					231	374	231.0000	523.0052		
FN1555	0.072	24.020	1.043e-2	4.901e-2	3291	3198	4283.6201	4141.0910	AAL93681.1| Protein Translation Elongation Factor Tu	
					4174	2792	4174.0000	3904.3599		
FN1556	-1.342	19.883	4.77e-4	1.154e-3	372	1210	484.2014	1566.8293	AAL93682.1| Protein Translation Elongation Factor G (EF-G)	
					751	1119	751.0000	1564.8205		
FN1557	0.505	13.816	6.96e-5	9.795e-5	113	79	147.0827	102.2971	AAL93683.1| SSU ribosomal protein S7P	
					139	71	139.0000	99.2871		
FN1560	0.726	15.424	1.205e-5	7.989e-6	200	133	260.3233	172.2217	AAL93686.1| unknown	
					279	110	279.0000	153.8251		
FN1562	-0.144	7.989	2.775e-2	1.468e-1	11	14	14.3178	18.1286	AAL93688.1| Phospho-2-dehydro-3-deoxyheptonate aldolase	
					16	11	16.0000	15.3825		
FN1576					5		6.5081		AAL93691.1| ATPases and helicase subunits involved in DNA replication	
FN1577	1.133	16.169	4.636e-5	5.509e-5	325	134	423.0254	173.5166	AAL93692.1| Rod shape-determining protein mreB	
					381	138	381.0000	192.9805		
FN1579	0.259	13.276	1.429e-2	7.063e-2	76	78	98.9229	101.0022	AAL93694.1| Cysteinyl-tRNA synthetase	
					119	58	119.0000	81.1078		
FN1580	-1.373	4.543				6		7.7694	AAL93695.1| 2-C-methyl-D-erythritol 4-phosphate cytidylyltransferase	
					3		3.0000			
FN1581	0.409	11.994	2.001e-2	1.025e-1	67	37	87.2083	47.9113	AAL93696.1| DNA mismatch repair protein mutS	
					60	45	60.0000	62.9284		
FN1582	0.568	8.455			12		15.6194		AAL93697.1| Hypothetical protein	
					30	11	30.0000	15.3825		

Show detected proteins only
Show all proteins
Filter by category:
GO: amino acid transport

Proteins found: 1358

Enter (or paste) list of ORFs

Find ORFs

Test	Cutoff
q-Value	.005
p-Value	

	Signif	Direction	Applies To
red	yes	+	ratios, bars
yellow	no	n/a	bars
green	yes	−	ratios, bars
pink	yes	+	*p*- , *q*-Values
light green	yes	−	

Dot Plots Dot Plots

Protein	FnSg vs Fn Log_2 Ratio	Log_2 Sum	*q*-Value	*p*-Value	Raw *FnSg*	Raw *Fn*	Normalized *FnSg*	Normalized *Fn*	Description
FN1586					9		11.7145		AAL93701.1| O-succinylbenzoate-CoA synthase
					9		9.0000		
FN1589	-0.798	10.823	3.784e-3	1.506e-2	25	37	32.5404	47.9113	AAL93704.1| LexA repressor
					32	46	32.0000	64.3268	
FN1590					3		3.9048		AAL93705.1| Hypothetical lipoprotein
					4		4.0000		
FN1591	-0.828	15.915	1.573e-3	5.135e-3	173	247	225.1797	319.8404	AAL93706.1| RNFB-related protein
					148	245	148.0000	342.6104	
FN1592									AAL93707.1| Na(+)-translocating NADH-quinone reductase subunit D
					6		6.0000		
FN1594	-0.772	11.027	3.114e-4	6.754e-4	23	49	29.9372	63.4501	AAL93709.1| Nitrogen fixation protein RNFG
					40	40	40.0000	55.9364	
FN1595	-0.390	13.951	3.337e-3	1.283e-2	76	121	98.9229	156.6829	AAL93710.1| Na(+)-translocating NADH-quinone reductase subunit B
					121	94	121.0000	131.4505	
FN1596	-1.721	19.116	5.396e-5	6.761e-5	359	1055	467.2803	1366.1198	AAL93711.1| Nitrogen fixation iron-sulphur protein RNFC
					363	980	363.0000	1370.4415	
FN1597						11		14.2439	AAL93712.1| Peptidyl-tRNA hydrolase
						14		19.5777	
FN1600	-0.202	5.714			5	6	6.5081	7.7694	AAL93715.1| tRNA pseudouridine synthase A
					7		7.0000		
FN1601	0.726	5.694			5		6.5081		AAL93716.1| Hypothetical cytosolic protein
					12	4	12.0000	5.5936	
FN1603	-2.466	6.871	4.153e-4	9.665e-4	4	22	5.2065	28.4878	AAL93718.1| 2',3'-cyclic nucleotide 3'-phosphodiesterase
					4	16	4.0000	22.3746	
FN1605	-0.305	16.915	9.648e-4	2.853e-3	240	314	312.3880	406.5987	AAL93720.1| Adenylosuccinate synthetase
					320	268	320.0000	374.7738	
FN1606	-0.891	11.047	8.397e-5	1.259e-4	25	46	32.5404	59.5654	AAL93721.1| 3-deoxy-D-manno-octulosonic-acid transferase
					35	47	35.0000	65.7253	
FN1607	-0.342	8.914	5.076e-2	2.837e-1	10	22	13.0162	28.4878	AAL93722.1| Cytidylate kinase
					26	15	26.0000	20.9761	
FN1608					7		9.1113		AAL93723.1| Ribosomal protein L11 methyltransferase
					9		9.0000		

Log_2 Ratios: -6 -4 -2 0 2 4 6

Show detected proteins only
Show all proteins
Filter by category:
GO: amino acid transport

Proteins found: 1358

Enter (or paste) list of ORFs

Find ORFs

Test	Cutoff
q-Value	.005
p-Value	

	Signif	Direction	Applies To
red	yes	+	ratios, bars
yellow	no	n/a	bars
green	yes	−	ratios, bars
light red	yes	+	*p*- , *q*-Values
light green	yes	−	

Dot Plots Dot Plots

Protein	FnSg vs Fn Log2 Ratio	Log2 Sum	q-Value	p-Value	Raw FnSg	Raw Fn	Normalized FnSg	Normalized Fn	Description
FN1609	-3.883	8.192	1.548e-4	2.81e-4	3	55	3.9048	71.2195	AAL93724.1| Hypothetical protein
					5	43	5.0000	60.1316	
FN1610	0.512	9.873	4.198e-4	9.797e-4	27	18	35.1436	23.3082	AAL93725.1| 33 kDa chaperonin
					38	20	38.0000	27.9682	
FN1611									AAL93726.1| Competence protein
						3		4.1952	
FN1613	-0.305	8.802	5.135e-2	2.873e-1	10	19	13.0162	24.6031	AAL93728.1| Hypothetical protein
					25	16	25.0000	22.3746	
FN1614	-1.318	9.373	3.635e-3	1.431e-2	12	25	15.6194	32.3725	AAL93729.1| MG(2+) chelatase family protein
					17	35	17.0000	48.9443	
FN1616	-0.652	7.822				14		18.1286	AAL93731.1| N utilization substance protein B
					12	14	12.0000	19.5777	
FN1618	0.513	10.982	2.119e-3	7.304e-3	38	29	49.4614	37.5521	AAL93733.1| Hypothetical protein
					58	27	58.0000	37.7571	
FN1619	1.088	14.596	8.853e-3	4.044e-2	129	90	167.9085	116.5410	AAL93734.1| Hypothetical cytosolic protein
					291	71	291.0000	99.2871	
FN1620	-1.141	18.170	5.5e-5	6.944e-5	296	589	385.2785	762.6962	AAL93735.1| SSU ribosomal protein S2P
					346	608	346.0000	850.2331	
FN1621	-1.772	19.966	2.151e-6	5.041e-7	379	1389	493.3127	1798.6164	AAL93736.1| Protein Translation Elongation Factor Ts
					602	1388	602.0000	1940.9927	
FN1622	-1.300	14.828	2.136e-3	7.376e-3	51	207	66.3824	268.0444	AAL93737.1| Uridylate kinase
					151	191	151.0000	267.0963	
FN1623	-2.822	11.066	3.864e-4	8.838e-4	16	107	20.8259	138.5543	AAL93738.1| Ribosome Recycling Factor (RRF)
					14	77	14.0000	107.6775	
FN1624	-2.311	10.807				70		90.6430	AAL93739.1| Protein translocase subunit secY
					19	70	19.0000	97.8887	
FN1625	-1.252	13.608	3.134e-5	3.048e-5	59	140	76.7954	181.2860	AAL93740.1| LSU ribosomal protein L15P
					68	117	68.0000	163.6139	
FN1626	-4.316	8.960				75		97.1175	AAL93741.1| LSU ribosomal protein L30P
					5	73	5.0000	102.0839	
FN1627	-1.029	17.054	6.439e-6	2.95e-6	184	397	239.4974	514.0754	AAL93742.1| SSU ribosomal protein S5P
					277	386	277.0000	539.7861	

Log2 Ratios: -6 -4 -2 0 2 4 6

Show detected proteins only
Show all proteins
Filter by category:
GO: amino acid transport

Proteins found: 1358

Enter (or paste) list of ORFs

Find ORFs

Test	Cutoff
q-Value	.005
p-Value	

Signif	Direction	Applies To
yes	+	ratios, bars
no	n/a	bars
yes	−	ratios, bars
yes	+	p- , q-Values
yes	−	

Dot Plots | Dot Plots

Protein	FnSg vs Fn Log$_2$ Ratio	Log$_2$ Sum	q-Value	p-Value	Raw FnSg	Raw Fn	Normalized FnSg	Normalized Fn	Description
FN1628	-3.678	11.608			12	177	15.6194	229.1973	AAL93743.1| LSU ribosomal protein L18P
						122		170.6060	
FN1629	-1.720	16.996	1.419e-6	2.647e-7	167	524	217.3700	678.5277	AAL93744.1| LSU ribosomal protein L6P
					181	453	181.0000	633.4796	
FN1630	-0.930	13.178	2.757e-5	2.558e-5	58	108	75.4938	139.8492	AAL93745.1| SSU ribosomal protein S8P
					64	90	64.0000	125.8569	
FN1631	-1.730	5.660			3	10	3.9048	12.9490	AAL93746.1| SSU ribosomal protein S14P
FN1632	-0.907	16.744	6.301e-5	8.445e-5	208	367	270.7362	475.2284	AAL93747.1| LSU ribosomal protein L5P
					213	309	213.0000	432.1086	
FN1634	-1.227	9.388			13	32	16.9210	41.4368	AAL93749.1| LSU ribosomal protein L24P
						27		37.7571	
FN1635	-2.045	9.144	5.747e-6	2.393e-6	8	39	10.4129	50.5011	AAL93750.1| LSU ribosomal protein L14P
					13	33	13.0000	46.1475	
FN1636	-0.115	12.651	8.779e-2	5.114e-1	60	76	78.0970	98.4124	AAL93751.1| SSU ribosomal protein S17P
					76	49	76.0000	68.5221	
FN1637						18		23.3082	AAL93752.1| LSU ribosomal protein L29P
						57		79.7094	
FN1638	-3.449	13.521	2.494e-5	2.24e-5	22	264	28.6356	341.8537	AAL93753.1| LSU ribosomal protein L16P
					37	268	37.0000	374.7738	
FN1639	-1.073	18.973	3.058e-6	9.209e-7	355	778	462.0739	1007.4324	AAL93754.1| SSU ribosomal protein S3P
					527	768	527.0000	1073.9787	
FN1640	-2.077	12.650	1.394e-6	2.562e-7	30	128	39.0485	165.7472	AAL93755.1| LSU ribosomal protein L22P
					39	117	39.0000	163.6139	
FN1641	-3.818	12.292	1.272e-4	2.145e-4	9	225	11.7145	291.3526	AAL93756.1| SSU ribosomal protein S19P
					26	172	26.0000	240.5265	
FN1642	-1.562	16.848	1.176e-5	7.651e-6	175	429	227.7829	555.5122	AAL93757.1| LSU ribosomal protein L2P
					172	447	172.0000	625.0891	
FN1643	-1.515	11.817	4.153e-5	4.649e-5	30	78	39.0485	101.0022	AAL93758.1| LSU ribosomal protein L23P
					32	73	32.0000	102.0839	
FN1644	-0.267	19.903	2.718e-4	5.627e-4	721	862	938.4655	1116.2040	AAL93759.1| LSU ribosomal protein L1E
					867	755	867.0000	1055.7993	

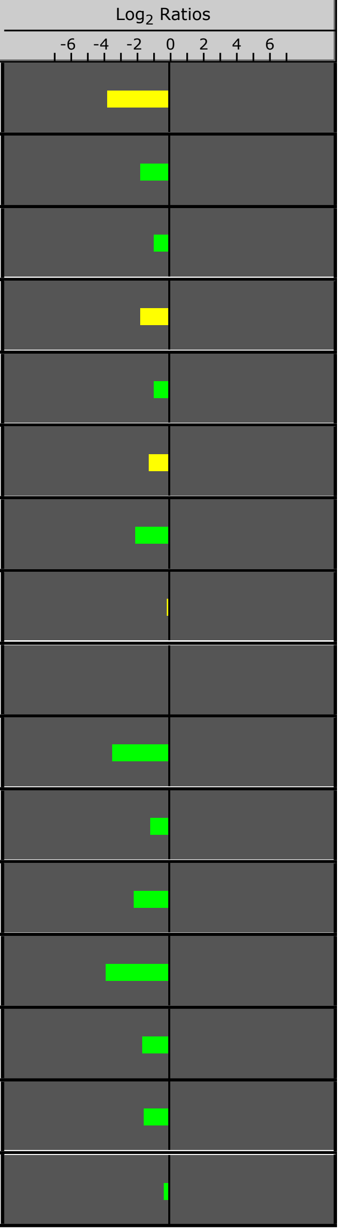

Show detected proteins only
Show all proteins
Filter by category:
GO: amino acid transport

Proteins found: 1358

Enter (or paste) list of ORFs

Find ORFs

Test	Cutoff
q-Value	.005
p-Value	

	Signif	Direction	Applies To
red	yes	+	ratios, bars
yellow	no	n/a	bars
green	yes	−	ratios, bars
pink	yes	+	p- , q-Values
light green	yes	−	

Dot Plots Dot Plots

Protein	*FnSg vs Fn* Log$_2$ Ratio	Log$_2$ Sum	*q*-Value	*p*-Value	Raw *FnSg*	Raw *Fn*	Normalized *FnSg*	Normalized *Fn*	Description
FN1645	-2.777	15.931	4.367e-5	5.02e-5	76	528	98.9229	683.7073	AAL93760.1| LSU ribosomal protein L3P
					92	447	92.0000	625.0891	
FN1646	-1.473	14.933	1.283e-6	2.219e-7	84	223	109.3358	288.7628	AAL93761.1| SSU ribosomal protein S10P
					103	215	103.0000	300.6581	
FN1647	-1.901	15.663	6.584e-4	1.755e-3	82	384	106.7326	497.2417	AAL93762.1| Hypothetical protein
					129	274	129.0000	383.1643	
FN1652	-1.609	13.990	1.009e-6	1.521e-7	60	167	78.0970	216.2483	AAL93767.1| Oligopeptide-binding protein oppA
					68	164	68.0000	229.3392	
FN1654	1.958	11.991	2.458e-7	2.208e-8	98	23	127.5584	29.7827	AAL93769.1| Hypothetical protein
					124	25	124.0000	34.9602	
FN1655	0.897	14.134	1.694e-2	8.536e-2	96	74	124.9552	95.8226	AAL93770.1| Hypothetical cytosolic protein
					241	72	241.0000	100.6855	
FN1657	-2.064	13.610	1.355e-4	2.341e-4	41	188	53.3663	243.4412	AAL93772.1| SSU ribosomal protein S6P
					56	153	56.0000	213.9567	
FN1658	-0.854	14.776	4.036e-4	9.329e-4	90	188	117.1455	243.4412	AAL93773.1| Prolyl-tRNA synthetase
					132	148	132.0000	206.9646	
FN1660	-0.162	4.806							AAL93775.1| ATP-dependent DNA helicase recG
					5	4	5.0000	5.5936	
FN1661	-1.264	10.098	4.791e-3	1.971e-2	19	49	24.7307	63.4501	AAL93776.1| Hypothetical cytosolic protein
					18	28	18.0000	39.1555	
FN1662	-0.007	10.895	1.503e-1	9.397e-1	30	33	39.0485	42.7317	AAL93777.1| Hypothetical protein
					48	32	48.0000	44.7491	
FN1663	-0.316	7.033	5.836e-2	3.294e-1	5	10	6.5081	12.9490	AAL93778.1| Hypothetical protein
					14	9	14.0000	12.5857	
FN1666									AAL93781.1| Hypothetical protein
						3		4.1952	
FN1667									AAL93782.1| dTDP-glucose 4,6-dehydratase
					3		3.0000		
FN1668									AAL93783.1| Cholinephosphate cytidylyltransferase
					3		3.0000		
FN1670	-1.179	11.194	6.02e-5	7.896e-5	21	52	27.3339	67.3348	AAL93785.1| Choline kinase
					37	56	37.0000	78.3109	

Log$_2$ Ratios: -6 -4 -2 0 2 4 6

Show detected proteins only
Show all proteins
Filter by category:
GO: amino acid transport

Proteins found: 1358

Enter (or paste) list of ORFs

Find ORFs

Test	Cutoff
q-Value	.005
p-Value	

	Signif	Direction	Applies To
red	yes	+	ratios, bars
yellow	no	n/a	bars
green	yes	−	ratios, bars
pink	yes	+	*p*- , *q*-Values
light green	yes	−	

Dot Plots Dot Plots

Protein	*FnSg* vs *Fn* Log$_2$ Ratio	Log$_2$ Sum	*q*-Value	*p*-Value	Raw *FnSg*	Raw *Fn*	Normalized *FnSg*	Normalized *Fn*	Description
FN1671						5		6.4745	AAL93786.1| hypothetical exported protein
						4		5.5936	
FN1676									AAL93791.1| Transposase
					3		3.0000		
FN1679	0.683	17.706	1.196e-4	1.972e-4	423	258	550.5838	334.0843	AAL93794.1| LPS biosynthesis protein WbpG
					621	283	621.0000	395.7499	
FN1683	-1.444	12.543	3.667e-5	3.829e-5	42	91	54.6679	117.8359	AAL93798.1| Acetyltransferase
					39	98	39.0000	137.0442	
FN1684	-0.478	16.316	1.065e-2	5.019e-2	146	257	190.0360	332.7894	AAL93799.1| N-acetylneuraminate synthase
					294	244	294.0000	341.2120	
FN1685	-0.582	12.102	5.844e-3	2.477e-2	41	54	53.3663	69.9246	AAL93800.1| dTDP-4-dehydrorhamnose reductase
					55	66	55.0000	92.2950	
FN1686	0.013	15.829	1.43e-1	8.869e-1	168	182	218.6716	235.6718	AAL93801.1| Spore coat polysaccharide biosynthesis protein spsF
					266	175	266.0000	244.7217	
FN1687	-0.434	15.507	1.237e-2	5.971e-2	117	221	152.2891	286.1730	AAL93802.1| Gluconate 5-dehydrogenase
					219	154	219.0000	215.3551	
FN1688	-0.403	11.523	5.263e-3	2.194e-2	31	51	40.3501	66.0399	AAL93803.1| Oxidoreductase
					54	42	54.0000	58.7332	
FN1689	-1.273	17.395	6.858e-6	3.251e-6	226	484	294.1653	626.7317	AAL93804.1| UDP-N-acetylglucosamine 4,6-dehydratase
					240	475	240.0000	664.2446	
FN1690	-0.929	11.719	4.889e-4	1.191e-3	27	60	35.1436	77.6940	AAL93805.1| Hypothetical protein
					49	59	49.0000	82.5062	
FN1692	-0.777	12.069	4.528e-4	1.079e-3	37	71	48.1598	91.9379	AAL93807.1| Glycosyl transferase
					52	57	52.0000	79.7094	
FN1693	-0.842	7.929	1.27e-3	3.976e-3	11	15	14.3178	19.4235	AAL93808.1| Hypothetical protein
					9	16	9.0000	22.3746	
FN1694	-1.700	12.483	1.062e-5	6.429e-6	33	108	42.9533	139.8492	AAL93809.1| UDP-N-acetyl-D-quinovosamine 4-epimerase
					41	95	41.0000	132.8489	
FN1695	-0.820	9.444	1.831e-3	6.132e-3	19	25	24.7307	32.3725	AAL93810.1| Probable quinovosaminephosphotransferae
					15	27	15.0000	37.7571	
FN1696	0.243	15.439	1.068e-2	5.038e-2	161	147	209.5603	190.3503	AAL93811.1| UDP-N-acetylglucosamine 4,6-dehydratase
					249	141	249.0000	197.1758	

Log$_2$ Ratios: -6 -4 -2 0 2 4 6

Show detected proteins only
Show all proteins
Filter by category:
GO: amino acid transport

Proteins found: 1358

Enter (or paste) list of ORFs

Find ORFs

Test	Cutoff
q-Value	.005
p-Value	

	Signif	Direction	Applies To
red	yes	+	ratios, bars
yellow	no	n/a	bars
green	yes	−	ratios, bars
pink	yes	+	*p*- , *q*-Values
light green	yes	−	

Dot Plots Dot Plots

Protein	FnSg vs Fn Log$_2$ Ratio	Log$_2$ Sum	*q*-Value	*p*-Value	Raw *FnSg*	Raw *Fn*	Normalized *FnSg*	Normalized *Fn*	Description
FN1697	-0.736	14.540	1.497e-3	4.847e-3	100	136	130.1617	176.1064	AAL93812.1| Hypothetical protein
					109	159	109.0000	222.3471	
FN1698	1.236	14.573	1.44e-4	2.542e-4	183	88	238.1958	113.9512	AAL93813.1| dTDP-4-dehydrorhamnose reductase
					241	64	241.0000	89.4982	
FN1700	-1.688	5.619			3		3.9048		AAL93815.1| Hypothetical protein
						9		12.5857	
FN1701	-0.217	12.200	1.37e-2	6.722e-2	44	57	57.2711	73.8093	AAL93816.1| ABC transporter ATP-binding protein
					70	53	70.0000	74.1157	
FN1703	-0.011	14.808	1.465e-1	9.123e-1	144	133	187.4328	172.2217	AAL93818.1| ADP-L-glycero-D-manno-heptose-6-epimerase
					150	120	150.0000	167.8092	
FN1704	-0.812	8.215	4.075e-5	4.509e-5	10	18	13.0162	23.3082	AAL93819.1| Serine protease
					13	16	13.0000	22.3746	
FN1707						7		9.0643	AAL93822.1| Aldose 1-epimerase
						4		5.5936	
FN1708	-0.649	18.681	3.79e-7	4.189e-8	406	634	528.4563	820.9668	AAL93823.1| Polyribonucleotide nucleotidyltransferase
					507	574	507.0000	802.6872	
FN1711						14		18.1286	AAL93826.1| Methyltransferase
						12		16.7809	
FN1713	1.188	7.325			14		18.2226		AAL93828.1| tRNA (Uracil-5-) - methyltransferase
					20	6	20.0000	8.3905	
FN1715					9		11.7145		AAL93830.1| ATPase
					9		9.0000		
FN1716									AAL93831.1| Hypothetical protein
					5		5.0000		
FN1717	-2.513	10.140	1.731e-5	1.326e-5	7	57	9.1113	73.8093	AAL93832.1| NAD-dependent DNA ligase
					19	62	19.0000	86.7014	
FN1718	-1.084	18.165	3.61e-4	8.134e-4	284	569	369.6591	736.7982	AAL93833.1| Protein translocase subunit secA
					375	602	375.0000	841.8426	
FN1719	-0.008	17.678	1.521e-1	9.536e-1	405	341	527.1547	441.5610	AAL93834.1| Hypothetical protein
					386	341	386.0000	476.8577	
FN1722	-0.891	4.856	9.382e-3	4.335e-2	3	7	3.9048	9.0643	AAL93837.1| Glucose inhibited division protein B
					4	4	4.0000	5.5936	

Log$_2$ Ratios: -6 -4 -2 0 2 4 6

Show detected proteins only
Show all proteins
Filter by category:
GO: amino acid transport

Proteins found: 1358

Enter (or paste) list of ORFs

Find ORFs

Test	Cutoff
q-Value	.005
p-Value	

	Signif	Direction	Applies To
red	yes	+	ratios, bars
yellow	no	n/a	bars
green	yes	−	ratios, bars
pink	yes	+	*p*-, *q*-Values
light green	yes	−	

Dot Plots Dot Plots

Protein	*FnSg* vs *Fn* Log$_2$ Ratio	Log$_2$ Sum	*q*-Value	*p*-Value	Raw *FnSg*	Raw *Fn*	Normalized *FnSg*	Normalized *Fn*	Description
FN1723	-0.775	13.186	1.12e-3	3.41e-3	65	86	84.6051	111.3614	AAL93838.1| Glucose inhibited division protein A
					63	101	63.0000	141.2394	
FN1724	1.009	8.673			11	11	14.3178	14.2439	AAL93839.1| Potassium uptake protein KtrA
					43		43.0000		
FN1727					5		6.5081		AAL93842.1| Chloride channel protein
FN1728	-1.320	7.878	3.995e-6	1.353e-6	8	18	10.4129	23.3082	AAL93843.1| Pyrrolidone-carboxylate peptidase
					9	18	9.0000	25.1714	
FN1730	0.233	9.170	2.828e-3	1.042e-2	20	18	26.0323	23.3082	AAL93845.1| Para-aminobenzoate synthase component I
					26	15	26.0000	20.9761	
FN1731					6		7.8097		AAL93846.1| Anthranilate synthase component II
					6		6.0000		
FN1732	1.181	10.736	1.07e-3	3.228e-3	41	24	53.3663	31.0776	AAL93847.1| Hypothetical protein
					71	17	71.0000	23.7730	
FN1733					9		11.7145		AAL93848.1| V-type sodium ATP synthase subunit D
					22		22.0000		
FN1734	0.735	14.394	8.88e-5	1.348e-4	151	86	196.5441	111.3614	AAL93849.1| V-type sodium ATP synthase subunit B
					182	83	182.0000	116.0680	
FN1735	1.703	13.940	3.393e-5	3.414e-5	184	49	239.4974	63.4501	AAL93850.1| V-type sodium ATP synthase subunit A
					213	54	213.0000	75.5141	
FN1736	1.958	9.725	3.525e-5	3.611e-5	42	12	54.6679	15.5388	AAL93851.1| V-type sodium ATP synthase subunit A
					60	10	60.0000	13.9841	
FN1737	0.730	7.235	8.316e-3	3.755e-2	12	5	15.6194	6.4745	AAL93852.1| V-type sodium ATP synthase subunit G
					16	9	16.0000	12.5857	
FN1738	1.818	11.075	9.609e-5	1.487e-4	61	22	79.3986	28.4878	AAL93853.1| V-type sodium ATP synthase subunit C
					95	15	95.0000	20.9761	
FN1739	-0.103	5.508			5		6.5081		AAL93854.1| V-type sodium ATP synthase subunit E
						5		6.9920	
FN1740									AAL93855.1| V-type sodium ATP synthase subunit K
					114		114.0000		
FN1741	0.833	9.603	2.926e-5	2.772e-5	28	15	36.4453	19.4235	AAL93856.1| V-type sodium ATP synthase subunit I
					38	16	38.0000	22.3746	

Log$_2$ Ratios: -6 -4 -2 0 2 4 6

Show detected proteins only
Show all proteins
Filter by category:
GO: amino acid transport

Proteins found: 1358

Enter (or paste) list of ORFs

Find ORFs

Test	Cutoff
q-Value	.005
p-Value	

Signif	Direction	Applies To
yes	+	ratios, bars
no	n/a	bars
yes	−	ratios, bars
yes	+	*p*-, *q*-Values
yes	−	

Dot Plots Dot Plots

Protein	FnSg vs Fn: Log_2 Ratio	FnSg vs Fn: Log_2 Sum	FnSg vs Fn: *q*-Value	FnSg vs Fn: *p*-Value	Raw: *FnSg*	Raw: *Fn*	Normalized: *FnSg*	Normalized: *Fn*	Description
FN1745	2.001	7.212	1.028e-5	6.096e-6	19	4	24.7307	5.1796	AAL93860.1| Cystathionine gamma-synthase
					24	5	24.0000	6.9920	
FN1746					8		10.4129		AAL93861.1| Cystathionine beta-lyase
					15		15.0000		
FN1762					4		5.2065		AAL93875.1| Protein yaaA
FN1763	2.008	10.242	9.797e-6	5.649e-6	55	16	71.5889	20.7184	AAL93876.1| Hypothetical cytosolic protein
					68	10	68.0000	13.9841	
FN1764	0.963	24.808	9.803e-6	5.654e-6	5881	3002	7654.8070	3887.2905	AAL93877.1| Enolase
					7477	2773	7477.0000	3877.7901	
FN1765	-0.922	19.718	1.549e-4	2.812e-4	535	933	696.3649	1208.1419	AAL93878.1| Pyruvate kinase
					653	964	653.0000	1348.0670	
FN1780	-1.274	8.859	6.736e-5	9.32e-5	9	28	11.7145	36.2572	AAL93879.1| Hypothetical protein
					16	22	16.0000	30.7650	
FN1781	-0.665	18.753	7.157e-5	1.022e-4	374	666	486.8046	862.4036	AAL93880.1| LytB protein
					569	580	569.0000	811.0776	
FN1783									AAL93882.1| Ethanolamine utilization protein eutJ
					8		8.0000		
FN1784	-0.466	9.445	1.392e-2	6.853e-2	13	22	16.9210	28.4878	AAL93883.1| unknown
					28	24	28.0000	33.5618	
FN1785	-1.124	8.804			11	32	14.3178	41.4368	AAL93884.1| Hypothetical protein
						15		20.9761	
FN1786	-0.867	11.781	8.295e-6	4.353e-6	36	60	46.8582	77.6940	AAL93885.1| ADP-heptose synthase
					41	59	41.0000	82.5062	
FN1787	-2.366	10.179				60		77.6940	AAL93886.1| Tetratricopeptide repeat family protein
					15	55	15.0000	76.9125	
FN1788	-0.505	8.757	3.007e-3	1.126e-2	13	21	16.9210	27.1929	AAL93887.1| 2C-methyl-D-erythritol 2,4-cyclodiphosphate synthase
					18	16	18.0000	22.3746	
FN1790	3.530	7.668			23		29.9372		AAL93889.1| Cob(I)alamin adenosyltransferase
					67	3	67.0000	4.1952	
FN1791					3		3.9048		AAL93890.1| Mutator MutT protein
					5		5.0000		

Log_2 Ratios: -6 -4 -2 0 2 4 6

Show detected proteins only
Show all proteins
Filter by category:
GO: amino acid transport

Proteins found: 1358

Enter (or paste) list of ORFs

Find ORFs

Test	Cutoff
q-Value	.005
p-Value	

	Signif	Direction	Applies To
red	yes	+	ratios, bars
yellow	no	n/a	bars
green	yes	−	ratios, bars
pink	yes	+	*p*- , *q*-Values
light green	yes	−	

Dot Plots Dot Plots

Protein	*FnSg* vs *Fn* Log$_2$ Ratio	Log$_2$ Sum	*q*-Value	*p*-Value	Raw *FnSg*	Raw *Fn*	Normalized *FnSg*	Normalized *Fn*	Description
FN1792	-0.244	22.170	3.243e-2	1.745e-1	1257	1765	1636.1320	2285.4989	AAL93891.1| Hypothetical protein
					2356	1746	2356.0000	2441.6234	
FN1793	0.619	15.293	1.327e-4	2.272e-4	201	131	261.6249	169.6319	AAL93892.1| Phosphoenolpyruvate-protein phosphotransferase
					235	110	235.0000	153.8251	
FN1794	-2.139	13.189	5.94e-5	7.745e-5	50	148	65.0808	191.6452	AAL93893.1| Phosphocarrier protein HPr
					27	153	27.0000	213.9567	
FN1795									AAL93894.1| Hypothetical protein
					12		12.0000		
FN1796	0.194	5.806							AAL93895.1| unknown
					8	5	8.0000	6.9920	
FN1797	-0.397	12.940	5.661e-3	2.387e-2	68	74	88.5099	95.8226	AAL93896.1| Spermidine/putrescine transport ATP-binding protein potA
					66	77	66.0000	107.6775	
FN1798	-1.055	6.832			6		7.8097		AAL93897.1| Spermidine/putrescine transport system permease protein potB
					7	11	7.0000	15.3825	
FN1800	-0.041	15.581	1.268e-1	7.719e-1	141	172	183.5279	222.7228	AAL93899.1| Peptidyl-prolyl cis-trans isomerase
					253	162	253.0000	226.5424	
FN1801	1.049	8.356			20		26.0323		AAL93900.1| Sodium/glutamate symport carrier protein
						9		12.5857	
FN1803	0.128	6.488			6	7	7.8097	9.0643	AAL93902.1| Transcriptional regulator, TetR family
					12		12.0000		
FN1804	1.168	8.806	7.785e-3	3.476e-2	18	11	23.4291	14.2439	AAL93903.1| Aminoacyl-histidine dipeptidase
					40	10	40.0000	13.9841	
FN1807	-1.080	16.630	3.196e-5	3.133e-5	186	348	242.1007	450.6253	AAL93906.1| Hypothetical protein
					196	340	196.0000	475.4593	
FN1808	-4.066	9.997			6	93	7.8097	120.4257	AAL93907.1| Hypothetical protein
						101		141.2394	
FN1809	-0.448	8.634	4.384e-2	2.418e-1	17	23	22.1275	29.7827	AAL93908.1| Iron/zinc/copper-binding protein
					12	12	12.0000	16.7809	
FN1811	-2.238	10.721	4.003e-5	4.383e-5	16	73	20.8259	94.5277	AAL93910.1| Manganese transport system ATP-binding protein mntA
					17	60	17.0000	83.9046	
FN1812	-2.463	13.109	6.056e-6	2.629e-6	30	167	39.0485	216.2483	AAL93911.1| Manganese-binding protein
					41	161	41.0000	225.1440	

Log$_2$ Ratios: -6 -4 -2 0 2 4 6

Show detected proteins only
Show all proteins
Filter by category: GO: amino acid transport

Proteins found: 1358

Enter (or paste) list of ORFs

Find ORFs

Test	Cutoff
q-Value	.005
p-Value	

Color	Signif	Direction	Applies To
red	yes	+	ratios, bars
yellow	no	n/a	bars
green	yes	−	ratios, bars
pink	yes	+	*p*- , *q*-Values
light green	yes	−	*p*- , *q*-Values

Dot Plots Dot Plots

Protein	FnSg vs Fn Log$_2$ Ratio	FnSg vs Fn Log$_2$ Sum	FnSg vs Fn *q*-Value	FnSg vs Fn *p*-Value	Raw *FnSg*	Raw *Fn*	Normalized *FnSg*	Normalized *Fn*	Description
FN1813	-2.467	8.610	4.626e-5	5.489e-5	6	34	7.8097	44.0266	AAL93912.1| Manganese-binding protein
					9	35	9.0000	48.9443	
FN1814	-1.854	8.615			8	29	10.4129	37.5521	AAL93913.1| Hypothetical protein
						27		37.7571	
FN1816	1.563	8.052				6		7.7694	AAL93915.1| unknown
					28	8	28.0000	11.1873	
FN1817	-0.484	5.654							AAL93916.1| Hemolysin
					6	6	6.0000	8.3905	
FN1819						8		10.3592	AAL93918.1| Export ABC transporter
						12		16.7809	
FN1820						8		10.3592	AAL93919.1| Export ABC transporter
						6		8.3905	
FN1822						16		20.7184	AAL93921.1| Flavodoxin
						10		13.9841	
FN1826	-1.115	11.313	5.451e-4	1.375e-3	25	52	32.5404	67.3348	AAL93925.1| Protease
					36	58	36.0000	81.1078	
FN1827	0.562	12.697	3.596e-3	1.412e-2	83	42	108.0342	54.3858	AAL93926.1| Replicative DNA helicase
					90	57	90.0000	79.7094	
FN1828	-1.611	11.985	1.875e-4	3.539e-4	26	79	33.8420	102.2971	AAL93927.1| LSU ribosomal protein L9P
					39	86	39.0000	120.2632	
FN1830	0.831	10.973	7.067e-3	3.105e-2	55	26	71.5889	33.6674	AAL93929.1| DNA polymerase III subunits gamma and tau
					48	24	48.0000	33.5618	
FN1831	0.765	13.132	3.158e-6	9.818e-7	93	55	121.0503	71.2195	AAL93930.1| Nitrogen assimilation regulatory protein
					126	53	126.0000	74.1157	
FN1832					6		7.8097		AAL93931.1| TonB protein
FN1834	-1.470	10.736	1.335e-5	9.22e-6	22	50	28.6356	64.7450	AAL93933.1| Biopolymer transport exbB protein
					21	52	21.0000	72.7173	
FN1836	-1.081	12.848	2.971e-5	2.831e-5	40	100	52.0647	129.4900	AAL93935.1| Tetratricopeptide repeat family protein
					66	86	66.0000	120.2632	
FN1839	2.490	17.447	1.467e-3	4.729e-3	914	135	1189.6775	174.8115	AAL93938.1| Glycerol kinase
					814	130	814.0000	181.7933	

Log$_2$ Ratios: -6 -4 -2 0 2 4 6

Show detected proteins only
Show all proteins
Filter by category:
GO: amino acid transport

Proteins found: 1358

Enter (or paste) list of ORFs

Find ORFs

Test	Cutoff
q-Value	.005
p-Value	

	Signif	Direction	Applies To
red	yes	+	ratios, bars
yellow	no	n/a	bars
green	yes	−	ratios, bars
pink	yes	+	*p*-, *q*-Values
light green	yes	−	

Dot Plots Dot Plots

Protein	FnSg vs Fn Log_2 Ratio	Log_2 Sum	*q*-Value	*p*-Value	Raw FnSg	Raw Fn	Normalized FnSg	Normalized Fn	Description
FN1840	1.229	11.257	8.826e-6	4.817e-6	58	24	75.4938	31.0776	AAL93939.1| Dihydroxyacetone kinase
					76	24	76.0000	33.5618	
FN1841	-1.358	8.103	9.292e-3	4.284e-2	9	14	11.7145	18.1286	AAL93940.1| Dihydroxyacetone kinase
					9	25	9.0000	34.9602	
FN1842	0.002	9.721	1.576e-1	9.943e-1	17	20	22.1275	25.8980	AAL93941.1| Dihydroxyacetone kinase phosphotransfer protein
					36	23	36.0000	32.1634	
FN1844	-0.425	7.186			8		10.4129		AAL93943.1| Ketoacyl reductase hetN
						10		13.9841	
FN1847	1.144	7.764	3.63e-3	1.429e-2	16	11	20.8259	14.2439	AAL93946.1| DTDP-4-dehydrorhamnose 3,5-epimerase
					23	4	23.0000	5.5936	
FN1849	0.102	9.988	1.094e-1	6.529e-1	20	27	26.0323	34.9623	AAL93948.1| Coenzyme F390 synthetase
					40	19	40.0000	26.5698	
FN1850	2.599	8.646	5.51e-3	2.313e-2	25	5	32.5404	6.4745	AAL93949.1| 3-oxoacyl-[acyl-carrier-protein] synthase III
					66	7	66.0000	9.7889	
FN1851	1.168	13.475	6.998e-5	9.877e-5	129	57	167.9085	73.8093	AAL93950.1| Ribonuclease PH
					152	49	152.0000	68.5221	
FN1852	-0.796	10.238	1.207e-2	5.8e-2	29	34	37.7469	44.0266	AAL93951.1| unknown
					15	34	15.0000	47.5459	
FN1853	-0.311	7.446	1.997e-2	1.022e-1	9	13	11.7145	16.8337	AAL93952.1| Methylaspartate mutase
					12	9	12.0000	12.5857	
FN1854	-0.685	11.090	4.229e-3	1.713e-2	22	45	28.6356	58.2705	AAL93953.1| Methylaspartate mutase
					45	43	45.0000	60.1316	
FN1855					4		5.2065		AAL93954.1| Methylaspartate mutase
					7		7.0000		
FN1856	0.079	23.790	1.052e-1	6.249e-1	2497	2855	3250.1366	3696.9402	AAL93955.1| Butyrate-acetoacetate CoA-transferase subunit B
					4579	2657	4579.0000	3715.5746	
FN1857	0.978	20.153	6.443e-6	2.954e-6	1208	633	1572.3528	819.6719	AAL93956.1| Acetoacetate: butyrate/acetate coenzyme A transferase
					1459	514	1459.0000	718.7826	
FN1858						60		77.6940	AAL93957.1| Short-chain fatty acids transporter
						59		82.5062	
FN1859	1.188	25.876	6.908e-3	3.021e-2	11444	4473	14895.699	5792.0888	AAL93958.1| Major outer membrane protein
					8794	3294	8794.0000	4606.3616	

Log_2 Ratios: -6 -4 -2 0 2 4 6

Show detected proteins only
Show all proteins
Filter by category:
GO: amino acid transport

Proteins found: 1358

Enter (or paste) list of ORFs

Find ORFs

Test	Cutoff
q-Value	.005
p-Value	

	Signif	Direction	Applies To
red	yes	+	ratios, bars
yellow	no	n/a	bars
green	yes	−	ratios, bars
pink	yes	+	*p*- , *q*-Values
light green	yes	−	

Dot Plots Dot Plots

Protein	*FnSg vs Fn* Log_2 Ratio	Log_2 Sum	*q*-Value	*p*-Value	Raw *FnSg*	Raw *Fn*	Normalized *FnSg*	Normalized *Fn*	Description
FN1860	-0.166	9.450	8.739e-2	5.089e-1	23	26	29.9372	33.6674	AAL93959.1| NA+/H+ antiporter NHAC
					20	16	20.0000	22.3746	
FN1862	-1.177	14.864	2.363e-5	2.073e-5	82	211	106.7326	273.2240	AAL93961.1| L-beta-lysine 5,6-aminomutase beta subunit
					123	176	123.0000	246.1201	
FN1863	-0.558	16.269	7.702e-4	2.175e-3	183	246	238.1958	318.5455	AAL93962.1| L-beta-lysine 5,6-aminomutase alpha subunit
					225	260	225.0000	363.5865	
FN1864	-2.269	7.056	2.158e-3	7.469e-3	5	24	6.5081	31.0776	AAL93963.1| DNA mismatch repair protein mutS
					4	14	4.0000	19.5777	
FN1865						4		5.1796	AAL93964.1| Hypothetical protein
FN1866	-0.891	20.673	3.507e-5	3.583e-5	722	1326	939.7672	1717.0377	AAL93965.1| Lysine 2,3-aminomutase
					959	1291	959.0000	1805.3469	
FN1867	-1.299	18.415	7.08e-6	3.409e-6	281	736	365.7543	953.0466	AAL93966.1| Zn-dependent alcohol dehydrogenases and related dehydrogenases
					388	645	388.0000	901.9743	
FN1868	-1.524	14.040	1.152e-4	1.877e-4	60	179	78.0970	231.7871	AAL93967.1| Hypothetical cytosolic protein
					75	149	75.0000	208.3630	
FN1869	-1.440	14.549	9.958e-5	1.555e-4	63	180	82.0018	233.0820	AAL93968.1| Hypothetical protein
					106	198	106.0000	276.8851	
FN1870									AAL93969.1| unknown
						6		8.3905	
FN1871						9		11.6541	AAL93970.1| unknown
						9		12.5857	
FN1872	-2.280	6.924				17		22.0133	AAL93971.1| unknown
					5	19	5.0000	26.5698	
FN1873	-2.301	10.116	1.864e-5	1.482e-5	10	57	13.0162	73.8093	AAL93972.1| Bis(5'-nucleosyl)-tetraphosphatase
					17	53	17.0000	74.1157	
FN1874	-1.455	10.186	3.119e-5	3.028e-5	14	43	18.2226	55.6807	AAL93973.1| Ribose 5-phosphate isomerase
					23	41	23.0000	57.3348	
FN1875	0.587	14.875	4.876e-3	2.011e-2	159	131	206.9570	169.6319	AAL93974.1| Peptidyl-prolyl cis-trans isomerase
					218	81	218.0000	113.2712	
FN1878						11		14.2439	AAL93977.1| unknown
						9		12.5857	

Log_2 Ratios: -6 -4 -2 0 2 4 6

Show detected proteins only
Show all proteins
Filter by category:
GO: amino acid transport

Proteins found: 1358

Enter (or paste) list of ORFs

Find ORFs

Test	Cutoff
q-Value	.005
p-Value	

	Signif	Direction	Applies To
red	yes	+	ratios, bars
yellow	no	n/a	bars
green	yes	–	ratios, bars
pink	yes	+	*p*- , *q*-Values
light green	yes	–	*p*- , *q*-Values

Dot Plots Dot Plots

Protein	FnSg vs Fn Log$_2$ Ratio	Log$_2$ Sum	*q*-Value	*p*-Value	Raw *FnSg*	Raw *Fn*	Normalized *FnSg*	Normalized *Fn*	Description
FN1879									AAL93978.1| SSU ribosomal protein S20P
						33		46.1475	
FN1880	1.246	12.413	1.623e-3	5.324e-3	101	33	131.4633	42.7317	AAL93979.1| Oxygen-insensitive NAD(P)H nitroreductase
					96	38	96.0000	53.1396	
FN1881					8		10.4129		AAL93980.1| Esterase
					16		16.0000		
FN1884						55		71.2195	AAL93983.1| unknown
						40		55.9364	
FN1890						8		10.3592	AAL93989.1| Hypothetical protein
						4		5.5936	
FN1891	1.317	8.923	3.924e-4	9.01e-4	25	14	32.5404	18.1286	AAL93990.1| Glycerophosphoryl diester phosphodiesterase
					37	7	37.0000	9.7889	
FN1893	-0.896	18.566	4.388e-6	1.551e-6	336	648	437.3432	839.0954	AAL93991.1| Fusobacterium outer membrane protein family
					476	615	476.0000	860.0220	
FN1898	-0.046	11.871	1.335e-1	8.185e-1	38	55	49.4614	71.2195	AAL93997.1| Sugar transport ATP-binding protein
					71	38	71.0000	53.1396	
FN1899	0.424	15.968	6.625e-4	1.77e-3	230	181	299.3718	234.3769	AAL93998.1| Hypothetical lipoprotein
					287	145	287.0000	202.7694	
FN1901									AAL94000.1| Transcription regulator, CRP family
					8		8.0000		
FN1902	-0.543	9.769	8.229e-3	3.709e-2	23	27	29.9372	34.9623	AAL94001.1| Deoxycytidylate deaminase
					19	26	19.0000	36.3587	
FN1903						55		71.2195	AAL94002.1| Coenzyme A disulfide reductase/ disulfide bond regulator domain
						49		68.5221	
FN1905	-0.069	4.069							AAL94004.1| outer membrane protein
					4	3	4.0000	4.1952	
FN1906	0.904	17.655	3.077e-3	1.158e-2	408	257	531.0596	332.7894	AAL94005.1| Cytosol aminopeptidase
					712	237	712.0000	331.4231	
FN1908	0.545	16.741	6.616e-4	1.767e-3	330	233	429.5335	301.7118	AAL94007.1| Glycerophosphoryl diester phosphodiesterase
					370	176	370.0000	246.1201	
FN1909	-1.182	13.653	2.194e-5	1.868e-5	52	129	67.6841	167.0421	AAL94008.1| UDP-3-O-[3-hydroxymyristoyl] glucosamine N-acyltransferase
					83	125	83.0000	174.8012	

Show detected proteins only
Show all proteins
Filter by category:
GO: amino acid transport

Proteins found: 1358

Enter (or paste) list of ORFs

Find ORFs

Test	Cutoff
q-Value	.005
p-Value	

	Signif	Direction	Applies To
red	yes	+	ratios, bars
yellow	no	n/a	bars
green	yes	−	ratios, bars
pink	yes	+	*p*-, *q*-Values
light green	yes	−	

Dot Plots Dot Plots

FnSg vs Fn

Fusobacterium nucleatum

Hackett Laboratory UW

Fn Summary Table | FnPg vs Fn | FnSg vs Fn | FnPgSg vs Fn | FnPgSg vs FnPg | FnSg vs FnPg | FnPgSg vs FnSg | Fn Coverage

Protein	*FnSg vs Fn* Log$_2$ Ratio	Log$_2$ Sum	*q*-Value	*p*-Value	Raw *FnSg*	Raw *Fn*	Normalized *FnSg*	Normalized *Fn*	Description
FN1910	-1.868	15.301	2.212e-4	4.327e-4	107	257	139.2730	332.7894	AAL94009.1| periplasmic protein
					71	311	71.0000	434.9054	
FN1911	-0.044	21.247	1.147e-1	6.882e-1	1320	1318	1718.1339	1706.6785	AAL94010.1| Outer membrane protein
					1389	1070	1389.0000	1496.2984	
FN1912	-1.482	8.543	3.111e-4	6.746e-4	7	25	9.1113	32.3725	AAL94011.1| Hypothetical protein
					14	23	14.0000	32.1634	
FN1913	-0.721	12.805	9.018e-4	2.628e-3	49	77	63.7792	99.7073	AAL94012.1| hydrolase (HD superfamily)
					68	84	68.0000	117.4664	
FN1914	-0.288	13.220	8.128e-3	3.655e-2	69	76	89.8115	98.4124	AAL94013.1| Anti-sigma F factor antagonist
					87	84	87.0000	117.4664	
FN1917	1.633	5.771			10		13.0162		AAL94016.1| tRNA delta(2)-isopentenylpyrophosphate transferase
						3		4.1952	
FN1918	0.327	12.767	1.635e-5	1.22e-5	73	59	95.0180	76.3991	AAL94017.1| SPO0B-associated GTP-binding protein
					92	52	92.0000	72.7173	
FN1919	-2.663	9.083	2.691e-6	7.264e-7	5	43	6.5081	55.6807	AAL94018.1| Methyltransferase
					12	44	12.0000	61.5300	
FN1920									AAL94019.1| tRNA (5-methylaminomethyl -2-thiouridylate) -methyltransferase
					4		4.0000		
FN1922	-0.162	6.865	2.667e-4	5.49e-4	8	9	10.4129	11.6541	AAL94021.1| Hypothetical protein
					10	8	10.0000	11.1873	
FN1923	-0.788	3.958				4		5.1796	AAL94022.1| Adenine-specific methyltransferase
					3		3.0000		
FN1926	0.441	12.145	1.104e-5	6.865e-6	59	46	76.7954	59.5654	AAL94025.1| Nitrogen regulatory IIA protein
					80	40	80.0000	55.9364	
FN1927	1.612	15.520	5.807e-5	7.498e-5	272	104	354.0397	134.6696	AAL94026.1| DEGV protein
					404	81	404.0000	113.2712	
FN1928	0.431	8.043			9		11.7145		AAL94027.1| Transcriptional regulator, MerR family
					26	10	26.0000	13.9841	
FN1929	0.184	12.529	1.035e-1	6.135e-1	46	38	59.8744	49.2062	AAL94028.1| Competence-damage protein cinA
					104	68	104.0000	95.0919	
FN1931	1.095	6.306				4		5.1796	AAL94030.1| Protease
					13	5	13.0000	6.9920	

Log$_2$ Ratios: -6 -4 -2 0 2 4 6

Show detected proteins only

Show all proteins

Filter by category:

GO: amino acid transport

Proteins found: 1358

Enter (or paste) list of ORFs

Find ORFs

Test	Cutoff
q-Value	.005
p-Value	

	Signif	Direction	Applies To
red	yes	+	ratios, bars
yellow	no	n/a	bars
green	yes	−	ratios, bars
pink	yes	+	*p*- , *q*-Values
light green	yes	−	

Dot Plots Dot Plots

Protein	*FnSg* vs *Fn* Log$_2$ Ratio	Log$_2$ Sum	*q*-Value	*p*-Value	Raw *FnSg*	Raw *Fn*	Normalized *FnSg*	Normalized *Fn*	Description	Log$_2$ Ratios (-6 -4 -2 0 2 4 6)
FN1933	-0.669	7.482	2.596e-2	1.364e-1	4	12	5.2065	15.5388	AAL94032.1| Hypothetical protein	
					16	13	16.0000	18.1793		
FN1935	-0.378	6.688	5.619e-2	3.163e-1	6	6	7.8097	7.7694	AAL94034.1| Adenine-specific methyltransferase	
					10	11	10.0000	15.3825		
FN1939	-0.375	7.536	5.459e-2	3.067e-1	13	11	16.9210	14.2439	AAL94038.1| Hypothetical protein	
					7	12	7.0000	16.7809		
FN1941	1.994	15.848	1.202e-3	3.715e-3	314	94	408.7076	121.7206	AAL94040.1| ClpB protein	
					561	87	561.0000	121.6616		
FN1942	-0.373	4.373				4		5.1796	AAL94041.1| putative DNA-binding protein	
					4		4.0000			
FN1943	1.724	22.081	1.794e-5	1.398e-5	2836	929	3691.3846	1202.9623	AAL94042.1| Tryptophanase	
					3965	797	3965.0000	1114.5325		
FN1948					10		13.0162		AAL94044.1| Hypothetical protein	
					6		6.0000			
FN1949	2.545	9.559	1.518e-7	1.049e-8	52	10	67.6841	12.9490	AAL94045.1| Xaa-Pro dipeptidase	
					65	7	65.0000	9.7889		
FN1950						55		71.2195	AAL94046.1| Serine protease	
						43		60.1316		
FN1951	-0.914	4.914				3		3.8847	AAL94047.1| ATPase associated with chromosome architecture/replication	
					4	8	4.0000	11.1873		
FN1956					10		13.0162		AAL94052.1| Hypothetical protein	
					13		13.0000			
FN1964	-1.125	8.710	1.536e-4	2.78e-4	9	24	11.7145	31.0776	AAL94054.1| O-linked GLCNAC transferase	
					16	21	16.0000	29.3666		
FN1965	0.148	10.770	9.843e-2	5.801e-1	43	30	55.9695	38.8470	AAL94055.1| Tetratricopeptide repeat family protein	
					32	29	32.0000	40.5539		
FN1966	0.307	12.286	1.663e-2	8.362e-2	67	42	87.2083	54.3858	AAL94056.1| Hypothetical protein	
					70	52	70.0000	72.7173		
FN1970						4		5.1796	AAL94060.1| Hemin-binding periplasmic protein hmuT precursor	
						5		6.9920		
FN1971	-1.568	7.478	9.772e-4	2.897e-3	5	15	6.5081	19.4235	AAL94061.1| Hemin receptor	
					9	19	9.0000	26.5698		

Show detected proteins only
Show all proteins
Filter by category:
GO: amino acid transport

Proteins found: 1358

Enter (or paste) list of ORFs

Find ORFs

Test	Cutoff
q-Value	.005
p-Value	

	Signif	Direction	Applies To
red	yes	+	ratios, bars
yellow	no	n/a	bars
green	yes	−	ratios, bars
pink	yes	+	*p*-, *q*-Values
light green	yes	−	

Dot Plots Dot Plots

Protein	*FnSg* vs *Fn* Log$_2$ Ratio	Log$_2$ Sum	*q*-Value	*p*-Value	Raw *FnSg*	Raw *Fn*	Normalized *FnSg*	Normalized *Fn*	Description
FN1972	1.441	8.932	1.657e-2	8.333e-2	16	11	20.8259	14.2439	AAL94062.1| unknown
					52	9	52.0000	12.5857	
FN1973	0.927	10.956	5.059e-3	2.097e-2	56	24	72.8905	31.0776	AAL94063.1| Translation initiation inhibitor
					50	24	50.0000	33.5618	
FN1974	-0.437	6.052				6		7.7694	AAL94064.1| DNA/RNA helicase (DEAD/DEAH BOX family)
					7	8	7.0000	11.1873	
FN1975	-0.734	14.551	6.127e-4	1.597e-3	107	164	139.2730	212.3636	AAL94065.1| ATP-dependent RNA helicase
					101	134	101.0000	187.3869	
FN1976	0.411	11.821	1.555e-2	7.767e-2	62	46	80.7002	59.5654	AAL94066.1| 4-amino-4-deoxychorismate lyase
					58	32	58.0000	44.7491	
FN1977	0.169	6.403	1.065e-1	6.335e-1	5	8	6.5081	10.3592	AAL94067.1| Cell cycle protein MesJ
					13	5	13.0000	6.9920	
FN1978	-1.358	14.502	1.201e-5	7.948e-6	64	178	83.3035	230.4922	AAL94068.1| Cell division protein ftsH
					107	184	107.0000	257.3074	
FN1979						75		97.1175	AAL94069.1| SSU ribosomal protein S15P
						86		120.2632	
FN1980					4		5.2065		AAL94070.1| Transporter
FN1983	-1.088	16.748	5.663e-6	2.333e-6	186	380	242.1007	492.0621	AAL94073.1| Alkyl hydroperoxide reductase C22 protein
					213	340	213.0000	475.4593	
FN1984	-0.591	16.170	4.172e-4	9.723e-4	154	276	200.4490	357.3925	AAL94074.1| Thioredoxin reductase
					242	221	242.0000	309.0485	
FN1985	-0.248	13.209	2.038e-2	1.046e-1	65	72	84.6051	93.2328	AAL94075.1| Inner membrane protein
					94	85	94.0000	118.8648	
FN1986	-0.445	16.161	1.66e-2	8.349e-2	222	248	288.9589	321.1353	AAL94076.1| Hypothetical protein
					175	222	175.0000	310.4470	
FN1987	-0.299	5.469				6		7.7694	AAL94077.1| Transcriptional regulator, GntR family
					6	5	6.0000	6.9920	
FN1988	4.705	16.346	1.624e-3	5.33e-3	1391	43	1810.5486	55.6807	AAL94078.1| Tyrosine phenol-lyase
					1137	41	1137.0000	57.3348	
FN1989					12		15.6194		AAL94079.1| Sodium-dependent tyrosine transporter
					8		8.0000		

Log$_2$ Ratios: -6 -4 -2 0 2 4 6

Show detected proteins only
Show all proteins
Filter by category:
GO: amino acid transport

Proteins found: 1358

Enter (or paste) list of ORFs

Find ORFs

Test	Cutoff
q-Value	.005
p-Value	

	Signif	Direction	Applies To
red	yes	+	ratios, bars
yellow	no	n/a	bars
green	yes	−	ratios, bars
pink	yes	+	*p*- , *q*-Values
light green	yes	−	*p*- , *q*-Values

Dot Plots Dot Plots

Protein	FnSg vs Fn Log$_2$ Ratio	FnSg vs Fn Log$_2$ Sum	FnSg vs Fn *q*-Value	FnSg vs Fn *p*-Value	Raw *FnSg*	Raw *Fn*	Normalized *FnSg*	Normalized *Fn*	Description
FN1991	-0.176	17.229	1.402e-2	6.907e-2	260	326	338.4203	422.1375	AAL94081.1| Glucosamine-1-phosphate acetyltransferase
					399	294	399.0000	411.1325	
FN1992	0.654	16.533	5.373e-3	2.247e-2	340	187	442.5496	242.1463	AAL94082.1| Ribose-phosphate pyrophosphokinase
					330	178	330.0000	248.9169	
FN1993	-0.248	5.142			3	5	3.9048	6.4745	AAL94083.1| SUA5 protein
					7		7.0000		
FN1994	-1.234	10.496	1.32e-2	6.439e-2	35	49	45.5566	63.4501	AAL94084.1| Hypothetical protein
					4	38	4.0000	53.1396	
FN1995						6		7.7694	AAL94085.1| Hypothetical protein
						8		11.1873	
FN2001	0.893	7.921	1.367e-3	4.347e-3	18	9	23.4291	11.6541	AAL94091.1| Hypothetical protein
					19	8	19.0000	11.1873	
FN2002									AAL94092.1| Permease
					3		3.0000		
FN2007	-1.896	7.321	1.205e-3	3.725e-3	7	15	9.1113	19.4235	AAL94097.1| Glutathione peroxidase
					4	21	4.0000	29.3666	
FN2008	0.312	4.449			4		5.2065		AAL94098.1| Glycine betaine transport ATP-binding protein
						3		4.1952	
FN2009					6		7.8097		AAL94099.1| Glycine betaine transport system permease protein
					8		8.0000		
FN2011	-0.766	14.767	1.108e-2	5.252e-2	60	171	78.0970	221.4279	AAL94101.1| Valyl-tRNA synthetase
					178	153	178.0000	213.9567	
FN2013	-0.050	5.340			5	5	6.5081	6.4745	AAL94103.1| GTP-binding protein
					6		6.0000		
FN2014	-1.139	15.688	8.765e-6	4.761e-6	128	275	166.6069	356.0976	AAL94104.1| ATP-dependent protease La
					143	233	143.0000	325.8295	
FN2015	-0.392	14.086	2.191e-3	7.61e-3	97	108	126.2568	139.8492	AAL94105.1| ATP-dependent clp protease ATP-binding subunit clpX
					104	116	104.0000	162.2155	
FN2016	-1.657	9.704	6.144e-5	8.136e-5	15	36	19.5242	46.6164	AAL94106.1| ATP-dependent Clp protease proteolytic subunit
					13	40	13.0000	55.9364	
FN2017	-1.253	18.465	1.917e-5	1.548e-5	273	728	355.3413	942.6874	AAL94107.1| Trigger factor, ppiase
					424	654	424.0000	914.5600	

Log$_2$ Ratios: -6 -4 -2 0 2 4 6

- Show detected proteins only
- Show all proteins
- Filter by category: GO: amino acid transport

Proteins found: 1358

Enter (or paste) list of ORFs

Find ORFs

Test	Cutoff
q-Value	.005
p-Value	

	Signif	Direction	Applies To
red	yes	+	ratios, bars
yellow	no	n/a	bars
green	yes	−	ratios, bars
pink	yes	+	*p*- , *q*-Values
light green	yes	−	

Dot Plots Dot Plots

Protein	FnSg vs Fn Log_2 Ratio	Log_2 Sum	*q*-Value	*p*-Value	Raw *FnSg*	Raw *Fn*	Normalized *FnSg*	Normalized *Fn*	Description
FN2018	-1.817	9.504	3.3e-5	3.28e-5	9	36	11.7145	46.6164	AAL94108.1| Single-stranded-DNA-specific exonuclease recJ
					17	39	17.0000	54.5380	
FN2019	-3.061	8.231				32		41.4368	AAL94109.1| Ribosome-binding factor A
					6	42	6.0000	58.7332	
FN2020	0.002	16.611	1.571e-1	9.905e-1	196	242	255.1168	313.3659	AAL94110.1| Bacterial Protein Translation Initiation Factor 2 (IF-2)
					378	228	378.0000	318.8374	
FN2022	-0.330	13.326	4.754e-4	1.149e-3	72	88	93.7164	113.9512	AAL94112.1| N utilization substance protein A
					87	81	87.0000	113.2712	
FN2023	0.634	7.675	2.435e-3	8.665e-3	12	8	15.6194	10.3592	AAL94113.1| Hypothetical cytosolic protein
					20	9	20.0000	12.5857	
FN2030	-2.696	13.977	1.006e-4	1.575e-4	39	265	50.7630	343.1486	AAL94115.1| Inorganic pyrophosphatase
					49	217	49.0000	303.4549	
FN2031	-2.199	6.199				10		12.9490	AAL94116.1| Thiamine biosynthesis lipoprotein apbE
					4	17	4.0000	23.7730	
FN2033	-0.528	7.929				16		20.7184	AAL94118.1| Guanylate kinase
					13	12	13.0000	16.7809	
FN2034	-0.761	8.605	3.446e-3	1.336e-2	11	17	14.3178	22.0133	AAL94119.1| Protein yicC
					16	21	16.0000	29.3666	
FN2035	-0.199	18.360	1.235e-2	5.959e-2	453	490	589.6323	634.5011	AAL94120.1| DNA-directed RNA polymerase beta' chain
					493	435	493.0000	608.3082	
FN2036	-0.392	17.925	8.896e-4	2.585e-3	318	469	413.9141	607.3082	AAL94121.1| DNA-directed RNA polymerase beta chain
					457	383	457.0000	535.5909	
FN2037	-0.522	19.081	2.186e-2	1.13e-1	315	682	410.0092	883.1220	AAL94122.1| LSU ribosomal protein L12P (L7/L12)
					833	645	833.0000	901.9743	
FN2038	-1.449	15.646	1.934e-3	6.543e-3	103	242	134.0665	313.3659	AAL94123.1| LSU ribosomal protein L10P
					140	311	140.0000	434.9054	
FN2039	-0.652	16.992	1.718e-3	5.69e-3	252	349	328.0074	451.9202	AAL94124.1| LSU ribosomal protein L1P
					248	324	248.0000	453.0847	
FN2040	-2.637	11.403	5.239e-4	1.306e-3	19	114	24.7307	147.6186	AAL94125.1| LSU ribosomal protein L11P
					17	80	17.0000	111.8728	
FN2041	-1.140	10.526	2.006e-4	3.838e-4	19	47	24.7307	60.8603	AAL94126.1| Transcription antitermination protein nusG
					27	38	27.0000	53.1396	

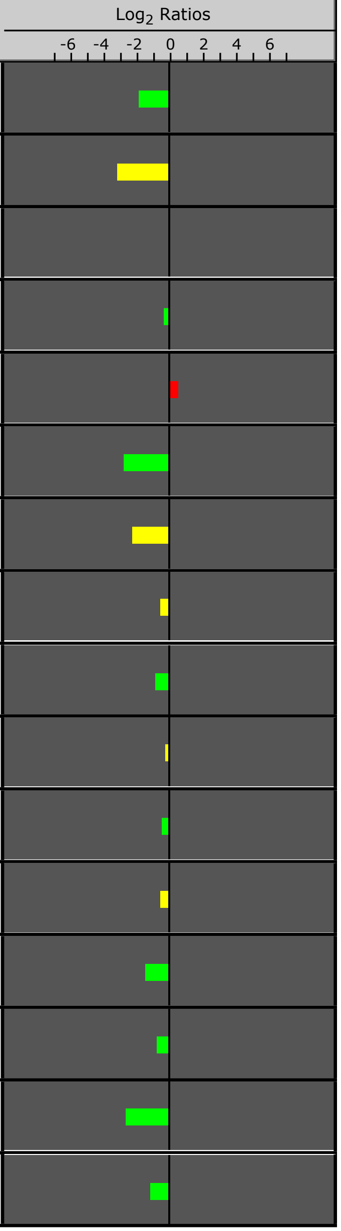

Show detected proteins only
Show all proteins
Filter by category:
GO: amino acid transport

Proteins found: 1358

Enter (or paste) list of ORFs

Find ORFs

Test	Cutoff
q-Value	.005
p-Value	

	Signif	Direction	Applies To
red	yes	+	ratios, bars
yellow	no	n/a	bars
green	yes	−	ratios, bars
pink	yes	+	*p*-, *q*-Values
light green	yes	−	

Dot Plots Dot Plots

Protein	FnSg vs Fn Log$_2$ Ratio	Log$_2$ Sum	q-Value	p-Value	Raw FnSg	Raw Fn	Normalized FnSg	Normalized Fn	Description
FN2045					7		9.1113		AAL94129.1| Ferric uptake regulation protein
					17		17.0000		
FN2046					16		20.8259		AAL94130.1| Acetyltransferase
					14		14.0000		
FN2047	-2.828	16.814	1.022e-6	1.554e-7	92	685	119.7487	887.0067	AAL94131.1| Fusobacterium outer membrane protein family
					135	659	135.0000	921.5520	
FN2048	0.460	16.509	4.768e-2	2.649e-1	180	198	234.2910	256.3902	AAL94132.1| Outer membrane protein
					482	189	482.0000	264.2994	
FN2049	0.575	17.548	2.44e-4	4.894e-4	430	270	559.6951	349.6231	AAL94133.1| unknown
					509	263	509.0000	367.7818	
FN2050	-2.496	13.698	1.488e-4	2.659e-4	40	195	52.0647	252.5055	AAL94134.1| Hypothetical membrane-spanning protein
					45	211	45.0000	295.0644	
FN2051	-0.996	13.556	3.358e-3	1.293e-2	81	113	105.4309	146.3237	AAL94135.1| unknown
					50	117	50.0000	163.6139	
FN2052	-1.817	14.263	4.337e-4	1.021e-3	81	210	105.4309	271.9291	AAL94136.1| unknown
					44	182	44.0000	254.5106	
FN2053	-0.412	11.942	8.627e-3	3.921e-2	49	61	63.7792	78.9889	AAL94137.1| Serine/threonine sodium symporter
					45	47	45.0000	65.7253	
FN2054	-1.117	14.807	4.601e-5	5.443e-5	96	195	124.9552	252.5055	AAL94138.1| Glucose-6-phosphate isomerase
					105	176	105.0000	246.1201	
FN2058	-1.249	16.655	1.385e-6	2.534e-7	155	374	201.7506	484.2927	AAL94142.1| Fusobacterium outer membrane protein family
					215	362	215.0000	506.2243	
FN2059	0.460	16.509	4.768e-2	2.649e-1	180	198	234.2910	256.3902	AAL94143.1| Outer membrane protein
					482	189	482.0000	264.2994	
FN2060	0.575	17.548	2.44e-4	4.894e-4	430	270	559.6951	349.6231	AAL94144.1| unknown
					509	263	509.0000	367.7818	
FN2061	-2.496	13.698	1.488e-4	2.659e-4	40	195	52.0647	252.5055	AAL94145.1| Hypothetical membrane-spanning protein
					45	211	45.0000	295.0644	
FN2062	-0.996	13.556	3.358e-3	1.293e-2	81	113	105.4309	146.3237	AAL94146.1| unknown
					50	117	50.0000	163.6139	
FN2063	-1.817	14.263	4.337e-4	1.021e-3	81	210	105.4309	271.9291	AAL94147.1| unknown
					44	182	44.0000	254.5106	

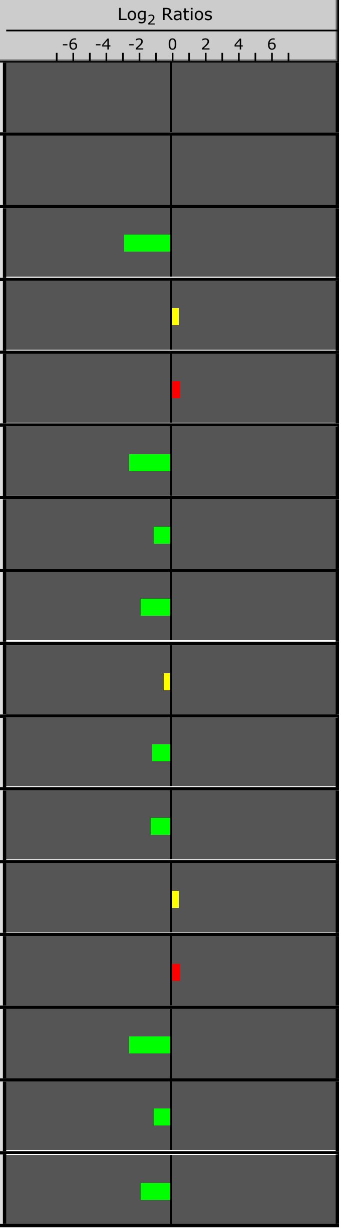

Show detected proteins only
Show all proteins
Filter by category:
GO: amino acid transport

Proteins found: 1358

Enter (or paste) list of ORFs

Find ORFs

Test	Cutoff
q-Value	.005
p-Value	

	Signif	Direction	Applies To
red	yes	+	ratios, bars
yellow	no	n/a	bars
green	yes	−	ratios, bars
pink	yes	+	p-, q-Values
light green	yes	−	

Dot Plots Dot Plots

Protein	FnSg vs Fn Log$_2$ Ratio	Log$_2$ Sum	*q*-Value	*p*-Value	Raw *FnSg*	Raw *Fn*	Normalized *FnSg*	Normalized *Fn*	Description
FN2067	-0.420	5.590				7		9.0643	AAL94151.1| Thiol:disulfide interchange protein tlpA
					6	5	6.0000	6.9920	
FN2068	1.442	7.210	2.632e-2	1.385e-1	7	6	9.1113	7.7694	AAL94152.1| dGTP triphosphohydrolase
					31	5	31.0000	6.9920	
FN2069									AAL94153.1| Amino acid carrier protein alsT
						4		5.5936	
FN2070	-0.680	8.083	4.726e-6	1.736e-6	10	16	13.0162	20.7184	AAL94154.1| Cobyric acid synthase
					13	15	13.0000	20.9761	
FN2073	1.727	10.592	2.867e-3	1.06e-2	43	15	55.9695	19.4235	AAL94157.1| Adenine phosphoribosyltransferase
					87	17	87.0000	23.7730	
FN2075	-1.773	10.224	6.886e-4	1.864e-3	8	48	10.4129	62.1552	AAL94159.1| Hypothetical protein
					27	47	27.0000	65.7253	
FN2078	0.627	5.373				4		5.1796	AAL94162.1| Transcriptional regulator, DeoR family
					8		8.0000		
FN2081						20		25.8980	AAL94165.1| ABC transporter substrate-binding protein
						17		23.7730	
FN2082	0.195	21.152	1.292e-2	6.282e-2	1156	1045	1504.6687	1353.1708	AAL94166.1| Formate--tetrahydrofolate ligase
					1761	1073	1761.0000	1500.4936	
FN2093	-1.909	9.515	7.33e-5	1.061e-4	13	41	16.9210	53.0909	AAL94177.1| General secretion pathway protein G
					11	37	11.0000	51.7412	
FN2098					7		9.1113		AAL94182.1| MRP-family nucleotide-binding protein
					13		13.0000		
FN2100	-1.352	10.984	5.926e-8	2.217e-9	21	56	27.3339	72.5144	AAL94184.1| Hypothetical protein
					29	51	29.0000	71.3189	
FN2102	0.005	9.342	1.537e-1	9.65e-1	20	22	26.0323	28.4878	AAL94186.1| ABC transporter ATP-binding protein
					25	16	25.0000	22.3746	
FN2103	-0.489	19.701	5.074e-4	1.251e-3	651	873	847.3524	1130.4479	AAL94187.1| tricarboxylate-binding protein
					711	756	711.0000	1057.1977	
FN2105						12		15.5388	AAL94189.1| tricarboxylate transport membrane protein RctA
						8		11.1873	
FN2106	-0.528	14.281	5.001e-4	1.227e-3	96	131	124.9552	169.6319	AAL94190.1| Transporter
					110	121	110.0000	169.2076	

Log$_2$ Ratios: -6 -4 -2 0 2 4 6

Show detected proteins only
Show all proteins
Filter by category: GO: amino acid transport

Proteins found: 1358

Enter (or paste) list of ORFs

Find ORFs

Test	Cutoff
q-Value	.005
p-Value	

	Signif	Direction	Applies To
red	yes	+	ratios, bars
yellow	no	n/a	bars
green	yes	−	ratios, bars
pink	yes	+	*p*- , *q*-Values
light green	yes	−	*p*- , *q*-Values

Dot Plots Dot Plots

FnSg vs Fn

Fusobacterium nucleatum

Hackett Laboratory UW

Fn Summary Table | FnPg vs Fn | FnSg vs Fn | FnPgSg vs Fn | FnPgSg vs FnPg | FnSg vs FnPg | FnPgSg vs FnSg | Fn Coverage

Protein	*FnSg* vs *Fn* Log$_2$ Ratio	Log$_2$ Sum	*q*-Value	*p*-Value	Raw *FnSg*	Raw *Fn*	Normalized *FnSg*	Normalized *Fn*	Description
FN2107	-2.959	10.249	4.61e-4	1.104e-3	10	66	13.0162	85.4634	AAL94191.1| Galactokinase
					12	78	12.0000	109.0760	
FN2108	-1.343	9.826	9.941e-4	2.957e-3	16	32	20.8259	41.4368	AAL94192.1| Galactose-1-phosphate uridylyltransferase
					17	39	17.0000	54.5380	
FN2109	-0.173	11.703	9.735e-3	4.533e-2	39	45	50.7630	58.2705	AAL94193.1| UDP-glucose 4-epimerase
					58	46	58.0000	64.3268	
FN2116	-2.952	10.112	5.602e-6	2.29e-6	13	76	16.9210	98.4124	AAL94200.1| Hypothetical exported 24-amino acid repeat protein
					7	62	7.0000	86.7014	
FN2117	-1.371	7.711				23		29.7827	AAL94201.1| Hypothetical exported 24-amino acid repeat protein
					9	12	9.0000	16.7809	
FN2118	-3.296	8.466				50		64.7450	AAL94202.1| Hypothetical exported 24-amino acid repeat protein
					6	38	6.0000	53.1396	
FN2119	-1.111	9.452	7.31e-4	2.023e-3	10	32	13.0162	41.4368	AAL94203.1| Hypothetical exported 24-amino acid repeat protein
					23	26	23.0000	36.3587	
FN2120						24		31.0776	AAL94204.1| Hypothetical exported 24-amino acid repeat protein
						30		41.9523	
FN2121	-4.149	11.554			10	171	13.0162	221.4279	AAL94205.1| Hypothetical exported 24-amino acid repeat protein
						172		240.5265	
FN2122	-0.033	16.936	3.383e-2	1.829e-1	275	280	357.9446	362.5721	AAL94206.1| Phenylalanyl-tRNA synthetase beta chain
					342	253	342.0000	353.7977	
FN2123	0.116	13.623	1.097e-1	6.545e-1	66	77	85.9067	99.7073	AAL94207.1| Phenylalanyl-tRNA synthetase alpha chain
					148	83	148.0000	116.0680	
FN2125	-1.475	13.917	6.497e-5	8.833e-5	57	154	74.1921	199.4146	AAL94209.1| DNA gyrase subunit A
					75	154	75.0000	215.3551	
FN2126	-2.209	11.693	9.972e-5	1.558e-4	15	95	19.5242	123.0155	AAL94210.1| DNA gyrase subunit B
					34	89	34.0000	124.4585	
FN2128						3		3.8847	AAL94212.1| RECF protein

Log$_2$ Ratios: -6 -4 -2 0 2 4 6

- Show detected proteins only
- Show all proteins
- Filter by category: GO: amino acid transport

Proteins found: 1358

Enter (or paste) list of ORFs

Find ORFs

Test	Cutoff
q-Value	.005
p-Value	

Color	Signif	Direction	Applies To
red	yes	+	ratios, bars
yellow	no	n/a	bars
green	yes	−	ratios, bars
light red	yes	+	*p*- , *q*-Values
light green	yes	−	

Dot Plots Dot Plots
