# Supplementary material for: Proteomics of Fusobacterium nucleatum within a model developing oral microbial community
Source: Microbiologyopen. 2014 Aug 25;3(5):729–51. doi: 10.1002/mbo3.204 (PMC4234264; doi:10.1002/mbo3.204)
Supplement: Table S8 — See description for Table S3. [file mbo30003-0729-sd10.pdf]

FnPgSg vs FnPg

Fusobacterium nucleatum

Hackett Laboratory UW

Protein	*FnPgSg vs FnPg* Log$_2$ Ratio	Log$_2$ Sum	*q*-Value	*p*-Value	Raw *FnPgSg*	Raw *FnPg*	Normalized *FnPgSg*	Normalized *FnPg*	Description
**FN0001**	-1.135	6.216	1e-1	2.195e-1	7	15	7.5124	20.5521	AAL94214.1| Chromosomal replication initiator protein dnaA
					3	5	4.1251	5.0000	
**FN0004**	1.354	12.583			145		155.6134		AAL94217.1| Inner membrane protein
					69	49	94.8764	49.0000	
**FN0005**	2.402	9.430	1.077e-4	9.734e-6	60	5	64.3917	6.8507	AAL94218.1| Jag protein
					41	16	56.3758	16.0000	
**FN0006**	-0.797	7.988			11		11.8052		AAL94219.1| Thiophene and furan oxidation protein THDF
					9	21	12.3752	21.0000	
**FN0007**	-0.200	6.476	1.591e-1	4.339e-1	10	6	10.7320	8.2208	AAL94220.1| Glucose inhibited division protein A
					5	12	6.8751	12.0000	
**FN0008**									AAL94221.1| Quinolinate synthetase A
						13		13.0000	
**FN0009**									AAL94222.1| L-aspartate oxidase
						29		29.0000	
**FN0018**	0.731	9.778			34		36.4887		AAL94231.1| Hypothetical protein
					29	23	39.8756	23.0000	
**FN0019**									AAL94232.1| Transcription-repair coupling factor
					4		5.5001		
**FN0022**	-1.261	11.838	1.31e-1	3.315e-1	28	128	30.0495	175.3779	AAL94235.1| Hypothetical protein
					35	12	48.1257	12.0000	
**FN0023**	0.322	7.241							AAL94236.1| Short-chain fatty acids transporter
					10	11	13.7502	11.0000	
**FN0024**	0.902	8.830	3.244e-2	4.269e-2	30	6	32.1959	8.2208	AAL94237.1| Hypothetical exported 24-amino acid repeat protein
					19	23	26.1254	23.0000	
**FN0025**	0.459	6.074							AAL94238.1| Hypothetical exported 24-amino acid repeat protein
					7	7	9.6251	7.0000	
**FN0026**					40		42.9278		AAL94239.1| Hypothetical exported 24-amino acid repeat protein
					20		27.5004		
**FN0029**					5		5.3660		AAL94242.1| Flavodoxin
**FN0030**	-0.969	12.713	2.885e-2	3.595e-2	63	63	67.6113	86.3188	AAL94243.1| 5-nitroimidazole antibiotic resistance protein
					36	143	49.5007	143.0000	

Log$_2$ Ratios: -6 -4 -2 0 2 4 6

Show detected proteins only
Show all proteins
Filter by category:
GO: amino acid transport

Proteins found: 1305

Enter (or paste) list of ORFs

Find ORFs

Test	Cutoff
q-Value	.005
p-Value	

	Signif	Direction	Applies To
red	yes	+	ratios, bars
yellow	no	n/a	bars
green	yes	–	ratios, bars
pink	yes	+	*p*- , *q*-Values
light green	yes	–	

Dot Plots Dot Plots

	FnPgSg vs FnPg				Raw		Normalized			Log_2 Ratios
Protein	Log_2 Ratio	Log_2 Sum	*q*-Value	*p*-Value	*FnPgSg*	*FnPg*	*FnPgSg*	*FnPg*	Description	-6 -4 -2 0 2 4 6
**FN0031**	-1.429	8.250	8.203e-3	5.576e-3	7	25	7.5124	34.2535	AAL94244.1| unknown	
					10	23	13.7502	23.0000		
**FN0033**	-0.485	11.626	1.056e-1	2.401e-1	45	65	48.2938	89.0591	AAL94246.1| unknown	
					34	44	46.7507	44.0000		
**FN0034**	0.587	10.302	3.262e-2	4.304e-2	44	27	47.2206	36.9938	AAL94247.1| unknown	
					29	21	39.8756	21.0000		
**FN0038**						77		105.5008	AAL94251.1| unknown	
**FN0039**									AAL94252.1| DNA primase (bacterial type) and small primase-like proteins	
						11		11.0000		
**FN0040**	-0.137	17.908	1.011e-1	2.234e-1	424	341	455.0350	467.2177	AAL94253.1| Asparaginyl-tRNA synthetase	
					357	573	490.8824	573.0000		
**FN0041**	0.127	5.742			4		4.2928		AAL94254.1| unknown	
					8	7	11.0002	7.0000		
**FN0043**	-0.898	5.102			4		4.2928		AAL94256.1| Hypothetical exported 24-amino acid repeat protein	
						8		8.0000		
**FN0046**	0.683	6.236				5		6.8507	AAL94259.1| 3-dehydroquinate dehydratase	
					8		11.0002			
**FN0047**	0.362	12.015	5.7e-2	9.732e-2	68	50	72.9773	68.5070	AAL94260.1| Exodeoxyribonuclease III	
					53	45	72.8761	45.0000		
**FN0048**	0.432	7.715	1.458e-2	1.347e-2	16	8	17.1711	10.9611	AAL94261.1| 4-nitrophenylphosphatase	
					12	14	16.5002	14.0000		
**FN0049**						9		12.3313	AAL94262.1| Hypothetical protein	
						31		31.0000		
**FN0050**	1.779	18.713	7.945e-3	5.254e-3	906	194	972.3154	265.8071	AAL94263.1| Fumarate reductase flavoprotein subunit	
					1059	442	1456.1470	442.0000		
**FN0052**	0.645	8.723			21	12	22.5371	16.4417	AAL94265.1| Arsenate reductase	
					21		28.8754			
**FN0054**	0.836	14.613	2.865e-2	3.557e-2	193	54	207.1268	73.9876	AAL94267.1| Tyrosyl-tRNA synthetase	
					157	163	215.8783	163.0000		
**FN0058**	-0.746	14.267	1.756e-2	1.775e-2	106	153	113.7587	209.6314	AAL94271.1| Cysteine desulfhydrase	
					75	154	103.1266	154.0000		

Show detected proteins only
Show all proteins
Filter by category:
GO: amino acid transport

Proteins found: 1305

Enter (or paste) list of ORFs

Find ORFs

Test	Cutoff
q-Value	.005
p-Value	

	Signif	Direction	Applies To
red	yes	+	ratios, bars
yellow	no	n/a	bars
green	yes	−	ratios, bars
pink	yes	+	*p*- , *q*-Values
light green	yes	−	

Dot Plots Dot Plots

Protein	*FnPgSg vs FnPg* Log$_2$ Ratio	Log$_2$ Sum	*q*-Value	*p*-Value	Raw *FnPgSg*	Raw *FnPg*	Normalized *FnPgSg*	Normalized *FnPg*	Description
**FN0059**	0.859	9.963	9.856e-4	2.352e-4	37	16	39.7082	21.9222	AAL94272.1| NifU protein
					33	25	45.3757	25.0000	
**FN0060**	1.047	7.669	1.152e-2	9.384e-3	19	5	20.3907	6.8507	AAL94273.1| D-alanyl-D-alanine carboxypeptidase
					15	13	20.6253	13.0000	
**FN0061**	-0.075	12.801	2.744e-1	9.066e-1	74	20	79.4165	27.4028	AAL94274.1| Thermostable carboxypeptidase 1
					62	146	85.2513	146.0000	
**FN0062**	1.277	6.447			13		13.9515		AAL94275.1| Hypothetical cytosolic protein
					11	6	15.1252	6.0000	
**FN0065**	0.513	12.942	1.35e-3	3.901e-4	95	50	101.9536	68.5070	AAL94278.1| Transcription accessory protein (S1 RNA binding domain)
					80	80	110.0017	80.0000	
**FN0067**	0.458	16.642	2.677e-2	3.218e-2	341	234	365.9598	320.6127	AAL94280.1| Isoleucyl-tRNA synthetase
					279	225	383.6308	225.0000	
**FN0069**	-0.457	15.217	5.162e-2	8.355e-2	163	198	174.9309	271.2877	AAL94282.1| Glycyl-tRNA synthetase alpha chain
					115	186	158.1274	186.0000	
**FN0070**	-0.161	17.077	1.322e-1	3.363e-1	326	241	349.8618	330.2037	AAL94283.1| Glycyl-tRNA synthetase beta chain
					257	456	353.3803	456.0000	
**FN0071**									AAL94284.1| GTP cyclohydrolase I
						8		8.0000	
**FN0072**	-0.643	12.460	9.599e-2	2.054e-1	53	42	56.8794	57.5459	AAL94285.1| 2-amino-4-hydroxy-6-hydroxymethyldihydropteridine pyrophosphokinase
					46	130	63.2510	130.0000	
**FN0073**	-0.323	8.984	1.884e-1	5.471e-1	17	9	18.2443	12.3313	AAL94286.1| Dihydropteroate synthase
					16	38	22.0003	38.0000	
**FN0074**	-0.439	7.561			11		11.8052		AAL94287.1| Ethanolamine utilization protein eutS
						16		16.0000	
**FN0076**									AAL94289.1| Ethanolamine two-component response regulator
						4		4.0000	
**FN0077**									AAL94290.1| Ethanolamine two-component sensor kinase
						3		3.0000	
**FN0078**	-2.092	7.626	8.903e-4	2.004e-4	5	19	5.3660	26.0327	AAL94291.1| Ethanolamine utilization protein eutA
					6	32	8.2501	32.0000	
**FN0079**	-2.464	12.415	5.957e-2	1.037e-1	33	49	35.4155	67.1369	AAL94292.1| Ethanolamine ammonia-lyase heavy chain
					20	280	27.5004	280.0000	

Log$_2$ Ratios: -6 -4 -2 0 2 4 6

Show detected proteins only
Show all proteins
Filter by category:
GO: amino acid transport

Proteins found: 1305

Enter (or paste) list of ORFs

Find ORFs

Test	Cutoff
***q*-Value**	.005
p-Value	

Signif	Direction	Applies To
yes	+	ratios, bars
no	n/a	bars
yes	−	ratios, bars
yes	+	*p*- , *q*-Values
yes	−	

Dot Plots Dot Plots

Protein	*FnPgSg vs FnPg* Log$_2$ Ratio	Log$_2$ Sum	*q*-Value	*p*-Value	Raw *FnPgSg*	Raw *FnPg*	Normalized *FnPgSg*	Normalized *FnPg*	Description	Log$_2$ Ratios (-6 -4 -2 0 2 4 6)
FN0080	-1.834	13.691	3.228e-6	6.998e-8	52	163	55.8062	223.3328	AAL94293.1| Ethanolamine ammonia-lyase light chain	
					48	211	66.0010	211.0000		
FN0081	-2.656	12.749	3.831e-2	5.419e-2	27	80	28.9763	109.6112	AAL94294.1| Ethanolamine utilization protein eutL	
					27	307	37.1256	307.0000		
FN0082	-0.522	9.292			21		22.5371		AAL94295.1| Ethanolamine utilization protein eutM	
					14	30	19.2503	30.0000		
FN0083	-2.079	14.531	4.203e-2	6.167e-2	78	126	83.7093	172.6376	AAL94296.1| Ethanolamine utilization protein eutM precursor	
					48	460	66.0010	460.0000		
FN0084	-1.048	9.943	4.175e-3	1.977e-3	24	33	25.7567	45.2146	AAL94297.1| Acetaldehyde dehydrogenase (acetylating)	
					13	45	17.8753	45.0000		
FN0086						7		9.5910	AAL94299.1| Hypothetical protein	
FN0087						13		17.8118	AAL94300.1| Ethanolamine utilization protein eutN	
FN0089					4		4.2928		AAL94302.1| Ethanolamine permease	
FN0090						9		12.3313	AAL94303.1| Ethanolamine utilization protein eutQ	
						6		6.0000		
FN0091						6		8.2208	AAL94304.1| Phosphoserine phosphatase	
FN0092	-2.272	8.900	7.374e-2	1.389e-1	7	11	7.5124	15.0715	AAL94305.1| NADPH-dependent butanol dehydrogenase	
					9	81	12.3752	81.0000		
FN0093	0.798	16.047	1.861e-4	2.318e-5	305	153	327.3247	209.6314	AAL94306.1| Thioredoxin	
					261	185	358.8804	185.0000		
FN0100	0.886	8.609	3.179e-3	1.339e-3	27	11	28.9763	15.0715	AAL94309.1| Flavodoxins/hemoproteins	
					18	14	24.7504	14.0000		
FN0102					5		5.3660		AAL94311.1| Ribonucleoside-diphosphate reductase alpha chain	
					8		11.0002			
FN0103					4		4.2928		AAL94312.1| Ribonucleoside-diphosphate reductase beta chain	
FN0106	-0.359	13.873	1.242e-2	1.048e-2	94	93	100.8804	127.4230	AAL94315.1| Hypothetical protein	
					84	150	115.5017	150.0000		

Show detected proteins only
Show all proteins
Filter by category:
GO: amino acid transport

Proteins found: 1305

Enter (or paste) list of ORFs

Find ORFs

Test	Cutoff
q-Value	.005
p-Value	

Color	Signif	Direction	Applies To
red	yes	+	ratios, bars
yellow	no	n/a	bars
green	yes	−	ratios, bars
light red	yes	+	*p*- , *q*-Values
light green	yes	−	

Dot Plots | Dot Plots

Protein	FnPgSg vs FnPg: Log_2 Ratio	Log_2 Sum	*q*-Value	*p*-Value	Raw: *FnPgSg*	Raw: *FnPg*	Normalized: *FnPgSg*	Normalized: *FnPg*	Description	Log_2 Ratios (-6 -4 -2 0 2 4 6)
**FN0108**	-1.507	8.051			9	16	9.6588	21.9222	AAL94317.1| Microcin C7 self-immunity protein mccF	
						33		33.0000		
**FN0110**	-0.302	17.590	1.079e-1	2.49e-1	342	442	367.0329	605.6018	AAL94319.1| Seryl-tRNA synthetase	
					315	381	433.1315	381.0000		
**FN0111**									AAL94320.1| unknown	
						5		5.0000		
**FN0113**	-2.117	10.223	2.542e-3	9.639e-4	13	46	13.9515	63.0264	AAL94322.1| Heat-inducible transcription repressor hrcA	
					14	81	19.2503	81.0000		
**FN0114**	0.507	14.093	1.711e-2	1.713e-2	163	83	174.9309	113.7216	AAL94323.1| GrpE protein	
					102	108	140.2521	108.0000		
**FN0116**	-0.263	20.290	1.638e-1	4.51e-1	1009	604	1082.8545	827.5645	AAL94325.1| Chaperone protein dnaK	
					716	1653	984.5148	1653.0000		
**FN0117**									AAL94326.1| O6-methylguanine-DNA methyltransferase	
						14		14.0000		
**FN0118**	0.675	11.837	1.078e-2	8.521e-3	72	29	77.2701	39.7341	AAL94327.1| Chaperone protein dnaJ	
					55	56	75.6261	56.0000		
**FN0119**									AAL94328.1| Flavodoxin	
						47		47.0000		
**FN0123**									AAL94332.1| ATPase	
						5		5.0000		
**FN0127**						4		5.4806	AAL94333.1| Fe-S oxidoreductase	
**FN0128**	-0.658	7.437	8.064e-3	5.4e-3	8	11	8.5856	15.0715	AAL94334.1| Spermidine/putrescine-binding protein	
					9	18	12.3752	18.0000		
**FN0130**	1.028	7.028			24		25.7567		AAL94336.1| ABC transporter ATP-binding protein	
					5	8	6.8751	8.0000		
**FN0132**	0.803	5.999	3.244e-2	4.268e-2	12	3	12.8783	4.1104	AAL93916.1| Hemolysin	
					6	8	8.2501	8.0000		
**FN0147**	0.129	9.325	2.133e-1	6.501e-1	25	12	26.8299	16.4417	AAL94353.1| PLSX protein	
					19	32	26.1254	32.0000		
**FN0148**	0.123	15.972	1.034e-1	2.317e-1	242	159	259.7134	217.8522	AAL94354.1| 3-oxoacyl-[acyl-carrier-protein] synthase III	
					196	268	269.5041	268.0000		

Show detected proteins only
Show all proteins
Filter by category:
GO: amino acid transport

Proteins found: 1305

Enter (or paste) list of ORFs

Find ORFs

Test	Cutoff
q-Value	.005
p-Value	

	Signif	Direction	Applies To
(red)	yes	+	ratios, bars
(yellow)	no	n/a	bars
(green)	yes	−	ratios, bars
(pink)	yes	+	*p*- , *q*-Values
(light green)	yes	−	*p*- , *q*-Values

Dot Plots | Dot Plots

Protein	FnPgSg vs FnPg Log$_2$ Ratio	Log$_2$ Sum	*q*-Value	*p*-Value	Raw *FnPgSg*	Raw *FnPg*	Normalized *FnPgSg*	Normalized *FnPg*	Description
FN0149	0.068	15.772	2.541e-1	8.167e-1	190	219	203.9072	300.0606	AAL94355.1| Malonyl-CoA-[acyl-carrier-protein] transacylase
					204	162	280.5042	162.0000	
FN0150	0.548	11.458	3.038e-3	1.249e-3	58	29	62.2454	39.7341	AAL94356.1| Acyl carrier protein
					48	48	66.0010	48.0000	
FN0151	0.776	19.246	5.85e-2	1.01e-1	893	205	958.3638	280.8787	AAL94357.1| 3-oxoacyl-[acyl-carrier-protein] synthase
					804	924	1105.5167	924.0000	
FN0152	-0.219	5.781							AAL94358.1| Ribonuclease III
					5	8	6.8751	8.0000	
FN0154									AAL94360.1| Ribonuclease G
					5		6.8751		
FN0155					13		13.9515		AAL94361.1| Hypothetical protein
					5		6.8751		
FN0156	0.804	7.654	4.903e-2	7.721e-2	17	4	18.2443	5.4806	AAL94362.1| Phosphopantetheine adenylyltransferase
					14	16	19.2503	16.0000	
FN0158	-0.200	8.476	1.591e-1	4.339e-1	20	12	21.4639	16.4417	AAL94364.1| DNA-binding protein
					10	24	13.7502	24.0000	
FN0164	-0.528	14.561	2.508e-2	2.927e-2	117	155	125.5639	212.3717	AAL94370.1| Anhydro-N-acetylmuramyl-tripeptide amidase
					97	161	133.3770	161.0000	
FN0170	0.025	13.867	2.779e-1	9.227e-1	117	63	125.5639	86.3188	AAL94376.1| GTP-binding protein
					88	156	121.0018	156.0000	
FN0173	1.062	11.089	4.654e-2	7.143e-2	68	7	72.9773	9.5910	AAL94379.1| Hypothetical protein
					45	55	61.8759	55.0000	
FN0174	0.973	13.558	1.273e-2	1.087e-2	165	56	177.0773	76.7278	AAL94380.1| Enoyl-[acyl-carrier-protein] reductase
					95	80	130.6270	80.0000	
FN0175	-0.068	7.905				10		13.7014	AAL94381.1| Cell division inhibitor MinC
					11	18	15.1252	18.0000	
FN0176	0.063	15.369	2.052e-1	6.188e-1	174	134	186.7361	183.5987	AAL94382.1| Cell division inhibitor MinD
					170	219	233.7535	219.0000	
FN0177	1.712	5.790			11	3	11.8052	4.1104	AAL94383.1| Cell division inhibitor MinE
					11		15.1252		
FN0178	0.251	10.999	7.306e-2	1.371e-1	42	35	45.0742	47.9549	AAL94384.1| UNC-44 ankyrins
					39	35	53.6258	35.0000	

Log$_2$ Ratios: -6 -4 -2 0 2 4 6

Show detected proteins only
Show all proteins
Filter by category:
GO: amino acid transport

Proteins found: 1305

Enter (or paste) list of ORFs

Find ORFs

Test: *q*-Value / *p*-Value — Cutoff: .005

Signif	Direction	Applies To
yes	+	ratios, bars
no	n/a	bars
yes	–	ratios, bars
yes	+	*p*- , *q*-Values
yes	–	

Dot Plots Dot Plots

Protein	*FnPgSg vs FnPg* Log$_2$ Ratio	Log$_2$ Sum	*q*-Value	*p*-Value	Raw *FnPgSg*	Raw *FnPg*	Normalized *FnPgSg*	Normalized *FnPg*	Description
**FN0179**	-1.721	10.821	8.15e-2	1.602e-1	27	93	28.9763	127.4230	AAL94385.1| Ankyrin repeat proteins
					13	27	17.8753	27.0000	
**FN0180**					8		8.5856		AAL94386.1| Tetratricopeptide repeat family protein
**FN0181**	-1.006	8.713	8.424e-2	1.682e-1	9	11	9.6588	15.0715	AAL94387.1| Hypothetical protein
					14	43	19.2503	43.0000	
**FN0182**	-1.761	13.860	9.97e-2	2.183e-1	76	35	81.5629	47.9549	AAL94388.1| Sarcosine oxidase alpha subunit
					37	401	50.8758	401.0000	
**FN0183**	-0.448	14.734	1.98e-1	5.877e-1	152	34	163.1258	46.5848	AAL94389.1| Glycerol-3-phosphate dehydrogenase
					87	339	119.6268	339.0000	
**FN0185**	-0.513	7.436	6.967e-3	4.177e-3	9	12	9.6588	16.4417	AAL94391.1| Hypothetical protein
					9	15	12.3752	15.0000	
**FN0189**	-1.400	7.244			9		9.6588		AAL94395.1| Two-component response regulator yesN
					4	20	5.5001	20.0000	
**FN0190**						6		8.2208	AAL94396.1| Two-component sensor kinase yesM
						3		3.0000	
**FN0191**	-1.092	8.263	2.389e-2	2.73e-2	7	14	7.5124	19.1820	AAL94397.1| helix-turn-helix DNA-binding protein
					12	32	16.5002	32.0000	
**FN0192**	1.303	9.303			39		41.8546		AAL94398.1| Dipeptide-binding protein
					27	16	37.1256	16.0000	
**FN0198**					5		5.3660		AAL94404.1| Transcriptional regulatory protein
					6		8.2501		
**FN0199**	-0.556	11.146	2.543e-2	2.985e-2	45	39	48.2938	53.4355	AAL94405.1| Hypothetical protein
					22	62	30.2505	62.0000	
**FN0200**	-0.244	19.007	2.26e-1	6.999e-1	609	942	653.5762	1290.6717	AAL94406.1| Biotin carboxyl carrier protein of glutaconyl-COA decarboxylase
					495	289	680.6353	289.0000	
**FN0202**	0.637	19.860	3.092e-2	3.975e-2	1335	585	1432.7163	801.5318	AAL94408.1| Glutaconate CoA-transferase subunit A
					727	763	999.6401	763.0000	
**FN0203**	-0.654	20.575	3.03e-2	3.86e-2	833	1361	893.9721	1864.7603	AAL94409.1| Glutaconate CoA-transferase subunit B
					799	1270	1098.6416	1270.0000	
**FN0204**	0.282	21.116	5.991e-2	1.045e-1	1462	833	1569.0122	1141.3265	AAL94410.1| Glutaconyl-CoA decarboxylase A subunit
					1277	1593	1755.9015	1593.0000	

Log$_2$ Ratios: -6 -4 -2 0 2 4 6

Show detected proteins only
Show all proteins
Filter by category:
GO: amino acid transport

Proteins found: 1305

Enter (or paste) list of ORFs

Find ORFs

Test	Cutoff
q-Value	.005
p-Value	

Signif	Direction	Applies To
yes	+	ratios, bars
no	n/a	bars
yes	–	ratios, bars
yes	+	*p*- , *q*-Values
yes	–	

Dot Plots Dot Plots

Protein	*FnPgSg vs FnPg* Log$_2$ Ratio	Log$_2$ Sum	*q*-Value	*p*-Value	Raw *FnPgSg*	Raw *FnPg*	Normalized *FnPgSg*	Normalized *FnPg*	Description
FN0206	-0.550	11.572	1.166e-1	2.787e-1	44	69	47.2206	94.5396	AAL94412.1| Activator of (R)-2-hydroxyglutaryl-CoA dehydratase
					32	39	44.0007	39.0000	
FN0207	-0.011	18.701	2.878e-1	9.689e-1	620	632	665.3814	865.9284	AAL94413.1| (R)-2-hydroxyglutaryl-CoA dehydratase alpha-subunit
					462	445	635.2596	445.0000	
FN0208	0.222	17.601	1.793e-2	1.826e-2	435	280	466.8402	383.6392	AAL94414.1| (R)-2-hydroxyglutaryl-CoA dehydratase beta-subunit
					361	442	496.3825	442.0000	
FN0209	-0.069	17.531	8.653e-2	1.75e-1	383	339	411.0340	464.4774	AAL94415.1| Hypothetical cytosolic protein
					319	427	438.6316	427.0000	
FN0212	-0.584	8.556	8.801e-2	1.795e-1	18	23	19.3175	31.5132	AAL94418.1| Hypothetical protein
					9	16	12.3752	16.0000	
FN0214						3		4.1104	AAL94420.1| Crossover junction endodeoxyribonuclease ruvC
FN0218	-1.665	13.460	1.814e-2	1.856e-2	56	173	60.0990	237.0342	AAL94424.1| Anthranilate synthase component II
					43	141	59.1259	141.0000	
FN0219	0.682	5.852							AAL94425.1| Autolysin response regulator
					7	6	9.6251	6.0000	
FN0221	0.463	12.145	4.803e-2	7.484e-2	87	45	93.3680	61.6563	AAL94427.1| Carbon starvation protein A
					47	53	64.6260	53.0000	
FN0224	0.274	8.356	1.616e-1	4.431e-1	23	16	24.6835	21.9222	AAL94430.1| Excinuclease ABC subunit B
					11	11	15.1252	11.0000	
FN0225									AAL94431.1| Gluconate permease
						4		4.0000	
FN0226	-0.183	11.711	1.556e-2	1.502e-2	50	47	53.6598	64.3966	AAL94432.1| Pyridoxal phosphate biosynthetic protein pdxA
					40	59	55.0008	59.0000	
FN0227									AAL94433.1| Hypothetical protein
						4		4.0000	
FN0233	-0.266	12.035	1.317e-1	3.341e-1	55	65	59.0258	89.0591	AAL94439.1| Hypothetical protein
					43	53	59.1259	53.0000	
FN0234									AAL94440.1| unknown
						11		11.0000	
FN0235						16		21.9222	AAL94441.1| ABC transporter ATP-binding protein

Log$_2$ Ratios: -6 -4 -2 0 2 4 6

Show detected proteins only
Show all proteins
Filter by category:
GO: amino acid transport

Proteins found: 1305

Enter (or paste) list of ORFs

Find ORFs

Test	Cutoff
q-Value	.005
p-Value	

	Signif	Direction	Applies To
red	yes	+	ratios, bars
yellow	no	n/a	bars
green	yes	−	ratios, bars
pink	yes	+	*p*- , *q*-Values
light green	yes	−	

Dot Plots Dot Plots

Protein	FnPgSg vs FnPg: Log_2 Ratio	Log_2 Sum	*q*-Value	*p*-Value	Raw: *FnPgSg*	Raw: *FnPg*	Normalized: *FnPgSg*	Normalized: *FnPg*	Description
FN0236	-1.091	16.017	1.278e-1	3.191e-1	152	486	163.1258	665.8880	AAL94442.1| ABC transporter substrate-binding protein
					138	86	189.7529	86.0000	
FN0237						3		4.1104	AAL94443.1| ABC transporter permease protein
FN0238	-0.410	10.436	1.077e-1	2.483e-1	23	40	24.6835	54.8056	AAL94444.1| Hypothetical protein
					29	31	39.8756	31.0000	
FN0240	-0.077	12.981	1.268e-1	3.154e-1	76	64	81.5629	87.6890	AAL94446.1| Thymidylate synthase
					68	97	93.5014	97.0000	
FN0241	-1.464	8.002	2.475e-2	2.871e-2	9	14	9.6588	19.1820	AAL94447.1| Dihydrofolate reductase
					7	34	9.6251	34.0000	
FN0242	-1.737	10.263			14		15.0247		AAL94448.1| Trk system potassium uptake protein trkA
					17	64	23.3754	64.0000	
FN0243	-2.241	7.160							AAL94449.1| Poly(A) polymerase
					4	26	5.5001	26.0000	
FN0244									AAL94450.1| COP associated protein
						10		10.0000	
FN0245	-0.313	5.687			6		6.4392		AAL94451.1| Copper-exporting ATPase
						8		8.0000	
FN0247	0.982	14.343	2.788e-2	3.415e-2	229	41	245.7618	56.1757	AAL94453.1| Hypothetical cytosolic protein
					116	149	159.5024	149.0000	
FN0248	-2.120	8.664			9		9.6588		AAL94454.1| Hypothetical Exported Protein
						42		42.0000	
FN0249	0.814	13.456	5.184e-2	8.412e-2	121	89	129.8567	121.9424	AAL94455.1| unknown
					110	38	151.2523	38.0000	
FN0250	0.652	13.840	7.563e-2	1.439e-1	142	106	152.3938	145.2348	AAL94456.1| unknown
					110	48	151.2523	48.0000	
FN0251	0.257	15.544	2.138e-1	6.518e-1	175	240	187.8093	328.8336	AAL94457.1| Hypothetical membrane-spanning Protein
					211	71	290.1294	71.0000	
FN0252	-1.438	17.551	5.197e-2	8.444e-2	231	753	247.9082	1031.7153	AAL94458.1| unknown
					207	411	284.6293	411.0000	
FN0253	0.488	15.687			234		251.1278		AAL94459.1| Outer membrane protein
					213	194	292.8794	194.0000	

Log_2 Ratios: -6 -4 -2 0 2 4 6

Show detected proteins only
Show all proteins
Filter by category:
GO: amino acid transport

Proteins found: 1305

Enter (or paste) list of ORFs
Find ORFs

Test	Cutoff
q-Value	.005
p-Value	

	Signif	Direction	Applies To
red	yes	+	ratios, bars
yellow	no	n/a	bars
green	yes	−	ratios, bars
pink	yes	+	*p*- , *q*-Values
light green	yes	−	

Dot Plots Dot Plots

Protein	*FnPgSg vs FnPg* Log$_2$ Ratio	Log$_2$ Sum	*q*-Value	*p*-Value	Raw *FnPgSg*	Raw *FnPg*	Normalized *FnPgSg*	Normalized *FnPg*	Description
FN0254	-0.256	18.094	1.078e-1	2.488e-1	373	352	400.3020	482.2892	AAL94460.1| Fusobacterium outer membrane protein family
					413	674	567.8836	674.0000	
FN0258	-0.370	5.289				6		8.2208	AAL94464.1| Zinc-transporting ATPase
					4	6	5.5001	6.0000	
FN0259					11		11.8052		AAL94465.1| Zinc-transporting ATPase
					12		16.5002		
FN0261	-0.304	7.774			12	12	12.8783	16.4417	AAL94467.1| Pyruvate formate-lyase activating enzyme
					10		13.7502		
FN0262	1.627	22.818	9.508e-3	6.973e-3	4443	1745	4768.2087	2390.8940	AAL94468.1| Formate acetyltransferase
					3482	704	4787.8222	704.0000	
FN0263	-0.896	15.729	1.265e-1	3.142e-1	180	386	193.1752	528.8740	AAL94469.1| Peptidyl-prolyl cis-trans isomerase
					108	107	148.5022	107.0000	
FN0264	0.647	15.932	4.078e-2	5.91e-2	273	96	292.9824	131.5334	AAL94470.1| Hypothetical protein
					242	268	332.7550	268.0000	
FN0265	-0.773	6.146			6		6.4392		AAL94471.1| Cell division protein ftsX
						11		11.0000	
FN0266	-0.956	5.044							AAL94472.1| membrane protein related to metalloendopeptidase
					3	8	4.1251	8.0000	
FN0267									AAL94473.1| ATP-NAD kinase
						6		6.0000	
FN0268	-1.869	5.958				11		15.0715	AAL94474.1| DNA repair protein recN
					3		4.1251		
FN0270					4		4.2928		AAL94476.1| GTP-binding protein era
					4		5.5001		
FN0271	-1.705	5.909			4		4.2928		AAL94477.1| Enoyl-CoA hydratase
						14		14.0000	
FN0272									AAL94478.1| Acetoacetate: butyrate/acetate coenzyme A transferase
						4		4.0000	
FN0273									AAL94479.1| Butyrate-acetoacetate CoA-transferase subunit B
						16		16.0000	
FN0276	-3.068	7.272			4		4.2928		AAL94482.1| Sodium-dependent phosphate transporter
						36		36.0000	

Log$_2$ Ratios: -6 -4 -2 0 2 4 6

Show detected proteins only
Show all proteins
Filter by category:
GO: amino acid transport

Proteins found: 1305

Enter (or paste) list of ORFs

Find ORFs

Test	Cutoff
q-Value	.005
p-Value	

	Signif	Direction	Applies To
red	yes	+	ratios, bars
yellow	no	n/a	bars
green	yes	–	ratios, bars
pink	yes	+	*p*- , *q*-Values
light green	yes	–	

Dot Plots | Dot Plots

Protein	FnPgSg vs FnPg Log$_2$ Ratio	Log$_2$ Sum	*q*-Value	*p*-Value	Raw *FnPgSg*	Raw *FnPg*	Normalized *FnPgSg*	Normalized *FnPg*	Description
FN0277	-0.275	6.814	1.447e-2	1.332e-2	9	9	9.6588	12.3313	AAL94483.1| Hypothetical protein
					7	11	9.6251	11.0000	
FN0278	0.373	16.480	3.447e-2	4.684e-2	362	209	388.4969	286.3592	AAL94484.1| Xaa-His dipeptidase
					218	245	299.7545	245.0000	
FN0279	-1.504	12.570	1.006e-1	2.215e-1	44	26	47.2206	35.6236	AAL94485.1| Lipoprotein
					33	227	45.3757	227.0000	
FN0280	0.183	12.558	2.323e-1	7.252e-1	76	21	81.5629	28.7729	AAL94486.1| Hypothetical protein
					61	117	83.8763	117.0000	
FN0281	-1.437	9.985	5.448e-2	9.099e-2	13	21	13.9515	28.7729	AAL94487.1| DNA polymerase III alpha subunit
					18	76	24.7504	76.0000	
FN0282	-0.148	10.803	1.813e-1	5.182e-1	30	27	32.1959	36.9938	AAL94488.1| Hypothetical protein
					35	52	48.1257	52.0000	
FN0283	-0.541	6.103							AAL94489.1| tRNA (Guanine-N1) - methyltransferase
					5	10	6.8751	10.0000	
FN0284	-1.455	9.946			11		11.8052		AAL94490.1| 16S rRNA processing protein rimM
					19	52	26.1254	52.0000	
FN0285	-0.531	8.978			22		23.6103		AAL94491.1| RNA binding protein
					10	27	13.7502	27.0000	
FN0287	-0.722	6.679			7		7.5124		AAL94493.1| Dimethyladenosine transferase
					6	13	8.2501	13.0000	
FN0288	-0.468	11.210	1.418e-1	3.721e-1	31	23	33.2691	31.5132	AAL94494.1| Hypoxanthine-guanine phosphoribosyltransferase
					36	83	49.5007	83.0000	
FN0290									AAL94496.1| Hemolysin
					3		4.1251		
FN0291	-1.075	5.568			5		5.3660		AAL94497.1| Hemolysin
					3	10	4.1251	10.0000	
FN0294	0.403	14.893	5.492e-2	9.217e-2	188	136	201.7608	186.3390	AAL94500.1| Transketolase subunit A
					145	117	199.3780	117.0000	
FN0295	-0.510	14.457	8.978e-2	1.851e-1	106	170	113.7587	232.9238	AAL94501.1| Transketolase
					100	125	137.5021	125.0000	
FN0296	0.363	11.980	8.364e-2	1.664e-1	60	30	64.3917	41.1042	AAL94502.1| Hypothetical cytosolic protein
					58	71	79.7512	71.0000	

Log$_2$ Ratios: -6 -4 -2 0 2 4 6

Show detected proteins only
Show all proteins
Filter by category:
GO: amino acid transport

Proteins found: 1305

Enter (or paste) list of ORFs

Find ORFs

Test	Cutoff
q-Value	.005
p-Value	

Color	Signif	Direction	Applies To
red	yes	+	ratios, bars
yellow	no	n/a	bars
green	yes	−	ratios, bars
pink	yes	+	*p*-, *q*-Values
light green	yes	−	

Dot Plots Dot Plots

Protein	*FnPgSg vs FnPg* Log$_2$ Ratio	Log$_2$ Sum	*q*-Value	*p*-Value	Raw *FnPgSg*	Raw *FnPg*	Normalized *FnPgSg*	Normalized *FnPg*	Description
**FN0297**	-0.755	6.844				5		6.8507	AAL94503.1| ATPase associated with chromosome architecture/replication
					6	21	8.2501	21.0000	
**FN0298**	-0.434	15.615	1.604e-1	4.385e-1	181	94	194.2484	128.7931	AAL94504.1| Histidyl-tRNA synthetase
					139	392	191.1279	392.0000	
**FN0299**	0.439	15.864	9.365e-2	1.976e-1	262	96	281.1773	131.5334	AAL94505.1| Aspartyl-tRNA synthetase
					209	288	287.3793	288.0000	
**FN0305**					3		3.2196		AAL94511.1| Iron(III) dicitrate-binding protein
					3		4.1251		
**FN0307**									AAL94513.1| Iron(III) dicitrate transport ATP-binding protein fecE
					3		4.1251		
**FN0308**	0.174	16.482	1.426e-1	3.748e-1	354	191	379.9113	261.6967	AAL94514.1| Iron(III)-binding protein
					191	308	262.6290	308.0000	
**FN0309**					5		5.3660		AAL94515.1| Iron(III)-transport system permease protein sfuB
					8		11.0002		
**FN0310**	0.287	13.118	1.467e-1	3.896e-1	116	83	124.4907	113.7216	AAL94516.1| Iron(III)-transport ATP-binding protein sfuC
					61	57	83.8763	57.0000	
**FN0311**	0.706	12.789	4.3e-2	6.368e-2	90	29	96.5876	39.7341	AAL94517.1| Anaerobic ribonucleoside-triphosphate reductase
					86	92	118.2518	92.0000	
**FN0313**	-1.384	6.231			5		5.3660		AAL94519.1| 16S rRNA m(5)C 967 methyltransferase
						14		14.0000	
**FN0314**									AAL94520.1| Caffeoyl-CoA O-methyltransferase
						8		8.0000	
**FN0315**									AAL94521.1| Transcriptional regulator, AraC family
						10		10.0000	
**FN0316**	0.361	9.445			25	17	26.8299	23.2924	AAL94522.1| Hypothetical protein
					24		33.0005		
**FN0317**	-1.298	7.167	6.799e-2	1.241e-1	4	7	4.2928	9.5910	AAL94523.1| Tryptophan synthase beta chain
					8	28	11.0002	28.0000	
**FN0319**	-0.348	5.267							AAL94525.1| Citrate (pro-3S)-lyase ligase
					4	7	5.5001	7.0000	
**FN0320**	0.573	9.914	9.886e-3	7.411e-3	36	16	38.6350	21.9222	AAL94526.1| Hypothetical cytosolic protein
					27	29	37.1256	29.0000	

Log$_2$ Ratios: -6 -4 -2 0 2 4 6

Show detected proteins only
Show all proteins
Filter by category: GO: amino acid transport

Proteins found: 1305

Enter (or paste) list of ORFs

Find ORFs

Test	Cutoff
q-Value	.005
p-Value	

	Signif	Direction	Applies To
red	yes	+	ratios, bars
yellow	no	n/a	bars
green	yes	−	ratios, bars
pink	yes	+	*p*- , *q*-Values
light green	yes	−	

Dot Plots | Dot Plots

Protein	*FnPgSg vs FnPg* Log$_2$ Ratio	Log$_2$ Sum	*q*-Value	*p*-Value	Raw *FnPgSg*	Raw *FnPg*	Normalized *FnPgSg*	Normalized *FnPg*	Description
FN0321	-0.193	13.971	1.807e-1	5.161e-1	103	70	110.5392	95.9098	AAL94527.1| Heat shock protein htpG
					92	175	126.5019	175.0000	
FN0322	-0.065	21.505	2.509e-1	8.028e-1	1688	934	1811.5544	1279.7106	AAL94528.1| Fructose-bisphosphate aldolase
					1136	2250	1562.0235	2250.0000	
FN0325	5.242	9.320			122	3	130.9299	4.1104	AAL94529.1| LSU ribosomal protein L20P
					131		180.1277		
FN0326					16		17.1711		AAL94530.1| LSU ribosomal protein L35P
					18		24.7504		
FN0327	0.147	9.194			18		19.3175		AAL94531.1| Bacterial Protein Translation Initiation Factor 3 (IF-3)
					23	23	31.6255	23.0000	
FN0329	-0.292	17.604	1.452e-1	3.843e-1	347	472	372.3989	646.7060	AAL94533.1| LSU ribosomal protein L13P
					316	341	434.5066	341.0000	
FN0330	1.977	13.137	1.227e-4	1.328e-5	187	45	200.6876	61.6563	AAL94534.1| SSU ribosomal protein S9P
					128	34	176.0027	34.0000	
FN0331	0.862	10.992	6.879e-2	1.261e-1	57	8	61.1722	10.9611	AAL94535.1| Hypothetical protein
					44	56	60.5009	56.0000	
FN0332	0.102	5.717			7		7.5124		AAL94536.1| Magnesium and cobalt transport protein corA
						7		7.0000	
FN0333									AAL94537.1| Glycerol uptake operon antiterminator regulatory protein
						8		8.0000	
FN0334	0.084	14.135	2.331e-1	7.288e-1	128	69	137.3691	94.5396	AAL94538.1| Aspartate/aromatic aminotransferase
					101	166	138.8771	166.0000	
FN0335	-0.207	19.484	1.993e-1	5.932e-1	764	932	819.9216	1276.9703	AAL94539.1| Outer membrane porin F
					563	563	774.1367	563.0000	
FN0336	0.969	10.878			67		71.9041		AAL94540.1| Hypothetical protein
					36	31	49.5007	31.0000	
FN0341	1.228	11.871			90		96.5876		AAL94545.1| transport protein
					66	40	90.7514	40.0000	
FN0342	0.387	10.885	1.069e-1	2.45e-1	44	38	47.2206	52.0653	AAL94546.1| Peptidyl-prolyl cis-trans isomerase
					38	24	52.2508	24.0000	
FN0347	-0.747	10.242	1.255e-1	3.104e-1	27	14	28.9763	19.1820	AAL94551.1| Phosphatidylserine decarboxylase
					18	71	24.7504	71.0000	

Log$_2$ Ratios: -6 -4 -2 0 2 4 6

Show detected proteins only
Show all proteins
Filter by category:
GO: amino acid transport

Proteins found: 1305

Enter (or paste) list of ORFs

Find ORFs

Test	Cutoff
q-Value	.005
p-Value	

	Signif	Direction	Applies To
red	yes	+	ratios, bars
yellow	no	n/a	bars
green	yes	−	ratios, bars
pink	yes	+	*p*- , *q*-Values
light green	yes	−	

Dot Plots | Dot Plots

Protein	FnPgSg vs FnPg: Log_2 Ratio	Log_2 Sum	*q*-Value	*p*-Value	Raw: *FnPgSg*	Raw: *FnPg*	Normalized: *FnPgSg*	Normalized: *FnPg*	Description
FN0348	-0.139	15.529	2.036e-1	6.118e-1	203	120	217.8587	164.4168	AAL94552.1| Nicotinate phosphoribosyltransferase
					143	292	196.6280	292.0000	
FN0349	-1.005	7.853			10	11	10.7320	15.0715	AAL94553.1| D-Tyr-tRNATyr deacylase
						28		28.0000	
FN0351	0.241	13.866	2.225e-1	6.861e-1	122	140	130.9299	191.8196	AAL94555.1| unknown
					98	33	134.7520	33.0000	
FN0352	-0.831	10.479	8.637e-2	1.745e-1	31	21	33.2691	28.7729	AAL94556.1| NA+/H+ antiporter NHAC
					17	72	23.3754	72.0000	
FN0355	-0.394	14.520	1.029e-1	2.298e-1	116	93	124.4907	127.4230	AAL94558.1| S-adenosylmethionine synthetase
					104	224	143.0022	224.0000	
FN0356	-1.084	8.239	6.211e-2	1.098e-1	12	26	12.8783	35.6236	AAL94559.1| Lactoylglutathione lyase
					8	15	11.0002	15.0000	
FN0357	0.188	7.458	2.124e-1	6.466e-1	11	5	11.8052	6.8507	AAL94560.1| ATP synthase epsilon chain, sodium ion specific
					12	18	16.5002	18.0000	
FN0358	-0.063	16.363	2.578e-2	3.046e-2	268	212	287.6165	290.4696	AAL94561.1| ATP synthase beta chain, sodium ion specific
					204	303	280.5042	303.0000	
FN0359	0.050	11.378	2.594e-1	8.397e-1	53	47	56.8794	64.3966	AAL94562.1| ATP synthase gamma chain, sodium ion specific
					35	37	48.1257	37.0000	
FN0360	-0.161	13.845	2.234e-2	2.484e-2	101	96	108.3928	131.5334	AAL94563.1| ATP synthase alpha chain, sodium ion specific
					88	125	121.0018	125.0000	
FN0361	0.258	9.481	1.512e-1	4.057e-1	25	24	26.8299	32.8834	AAL94564.1| ATP synthase delta chain, sodium ion specific
					23	16	31.6255	16.0000	
FN0362	0.017	9.926			29		31.1227		AAL94565.1| ATP synthase B chain, sodium ion specific
					23	31	31.6255	31.0000	
FN0364	-0.653	6.748			9		9.6588		AAL94567.1| ATP synthase A chain, sodium ion specific
					5	13	6.8751	13.0000	
FN0366	0.610	15.795	2.972e-3	1.209e-3	253	127	271.5185	174.0078	AAL94569.1| Phosphoglucosamine mutase
					231	212	317.6298	212.0000	
FN0368	-0.245	13.315	1.467e-1	3.897e-1	78	59	83.7093	80.8383	AAL94571.1| Adenylosuccinate lyase
					74	139	101.7515	139.0000	
FN0370	-0.329	9.055	1.841e-1	5.298e-1	14	10	15.0247	13.7014	AAL94573.1| Signal peptidase I
					19	38	26.1254	38.0000	

Log_2 Ratios: -6 -4 -2 0 2 4 6

Show detected proteins only
Show all proteins
Filter by category:
GO: amino acid transport

Proteins found: 1305

Enter (or paste) list of ORFs

Find ORFs

Test	Cutoff
q-Value	.005
p-Value	

	Signif	Direction	Applies To
red	yes	+	ratios, bars
yellow	no	n/a	bars
green	yes	−	ratios, bars
pink	yes	+	*p*- , *q*-Values
light green	yes	−	

Dot Plots Dot Plots

Protein	*FnPgSg vs FnPg* Log$_2$ Ratio	Log$_2$ Sum	*q*-Value	*p*-Value	Raw *FnPgSg*	Raw *FnPg*	Normalized *FnPgSg*	Normalized *FnPg*	Description
**FN0371**	1.802	11.250	5.343e-3	2.773e-3	100	32	107.3196	43.8445	AAL94574.1| Hypothetical protein
					56	9	77.0012	9.0000	
**FN0374**						9		12.3313	AAL94577.1| Single-stranded-DNA-specific exonuclease recJ
						7		7.0000	
**FN0375**	0.020	18.116	2.581e-1	8.339e-1	466	353	500.1092	483.6594	AAL94578.1| Iron(III)-binding protein
					417	575	573.3836	575.0000	
**FN0376**	-1.174	11.959	4.776e-3	2.368e-3	36	61	38.6350	83.5785	AAL94579.1| Iron(III)-transport ATP-binding protein sfuC
					33	106	45.3757	106.0000	
**FN0377**					39		41.8546		AAL94580.1| Iron(III)-transport system permease protein sfuB
					7		9.6251		
**FN0378**					6		6.4392		AAL94581.1| UDP-glucose 4-epimerase
					11		15.1252		
**FN0379**	0.544	6.537	9.755e-2	2.107e-1	14	8	15.0247	10.9611	AAL94582.1| Hypothetical protein
					6	5	8.2501	5.0000	
**FN0380**	0.459	5.629							AAL94583.1| unknown
					6	6	8.2501	6.0000	
**FN0381**					20		21.4639		AAL94584.1| unknown
					18		24.7504		
**FN0384**	1.679	5.758			13	3	13.9515	4.1104	AAL94587.1| Hypothetical protein
					9		12.3752		
**FN0385**									AAL94588.1| Hypothetical protein
						5		5.0000	
**FN0387**	0.954	9.949	9.124e-4	2.082e-4	38	14	40.7814	19.1820	AAL94590.1| Fusobacterium outer membrane protein family
					34	26	46.7507	26.0000	
**FN0390**	0.143	13.042	2.207e-1	6.789e-1	103	40	110.5392	54.8056	AAL94593.1| Hypothetical protein
					60	120	82.5012	120.0000	
**FN0391**	-0.910	8.767	1.328e-1	3.384e-1	13	6	13.9515	8.2208	AAL94594.1| Hydrolase (HAD superfamily)
					12	49	16.5002	49.0000	
**FN0392**	0.656	5.300			7		7.5124		AAL94595.1| Oxygen-independent coproporphyrinogen III oxidase
					6	5	8.2501	5.0000	
**FN0393**	-1.731	6.615	7.869e-3	5.162e-3	5	11	5.3660	15.0715	AAL94596.1| Polysaccharide deacetylase
					4	21	5.5001	21.0000	

Log$_2$ Ratios: -6 -4 -2 0 2 4 6

Show detected proteins only
Show all proteins
Filter by category:
GO: amino acid transport

Proteins found: 1305

Enter (or paste) list of ORFs

Find ORFs

Test	Cutoff
q-Value	.005
p-Value	

	Signif	Direction	Applies To
red	yes	+	ratios, bars
yellow	no	n/a	bars
green	yes	−	ratios, bars
pink	yes	+	*p*- , *q*-Values
light green	yes	−	

Dot Plots | Dot Plots

Protein	*FnPgSg vs FnPg* Log_2 Ratio	Log_2 Sum	*q*-Value	*p*-Value	Raw *FnPgSg*	Raw *FnPg*	Normalized *FnPgSg*	Normalized *FnPg*	Description
**FN0394**	-1.421	6.795			6	4	6.4392	5.4806	AAL94597.1| Outer membrane protein
						29		29.0000	
**FN0396**	-0.014	24.392	2.315e-1	7.221e-1	4210	3549	4518.1541	4862.6263	AAL94599.1| Dipeptide-binding protein
					3505	4566	4819.4477	4566.0000	
**FN0397**	0.344	11.196			53		56.8794		AAL94600.1| Dipeptide transport system permease protein dppB
					38	43	52.2508	43.0000	
**FN0398**	0.552	10.881	6.125e-3	3.399e-3	48	29	51.5134	39.7341	AAL94601.1| Dipeptide transport system permease protein dppC
					39	32	53.6258	32.0000	
**FN0399**	0.176	14.911	1.494e-1	3.993e-1	149	98	159.9062	134.2737	AAL94602.1| Dipeptide transport ATP-binding protein dppD
					155	196	213.1282	196.0000	
**FN0400**	-0.490	16.632	1.36e-2	1.205e-2	227	306	243.6154	419.2628	AAL94603.1| Dipeptide transport ATP-binding protein dppF
					214	336	294.2544	336.0000	
**FN0405**	1.026	13.653	1.135e-2	9.178e-3	175	38	187.8093	52.0653	AAL94608.1| Tryptophanyl-tRNA synthetase
					99	107	136.1271	107.0000	
**FN0406**	0.769	10.997	6.987e-3	4.197e-3	60	25	64.3917	34.2535	AAL94609.1| Alanine racemase, biosynthetic
					39	35	53.6258	35.0000	
**FN0407**	2.268	11.053			104		111.6124		AAL94610.1| Hypothetical protein
					66	21	90.7514	21.0000	
**FN0408**	0.375	12.016	1.543e-1	4.166e-1	61	19	65.4649	26.0327	AAL94611.1| Acetyl-coenzyme A carboxylase carboxyl transferase subunit beta
					59	87	81.1262	87.0000	
**FN0409**	1.127	17.122	9.283e-4	2.139e-4	475	219	509.7680	300.0606	AAL94612.1| Acetyl-coenzyme A carboxylase carboxyl transferase subunit alpha
					441	211	606.3841	211.0000	
**FN0410**	0.368	13.319	1.163e-3	3.104e-4	109	62	116.9783	84.9487	AAL94613.1| 6-phosphofructokinase
					82	93	112.7517	93.0000	
**FN0413**	-0.701	4.905			4	8	4.2928	10.9611	AAL94616.1| unknown
						3		3.0000	
**FN0414**					6		6.4392		AAL94617.1| ATP-dependent helicase HEPA
**FN0416**	-0.163	7.086			9	9	9.6588	12.3313	AAL94619.1| Type III restriction-modification system methylation subunit
					9		12.3752		
**FN0417**	-0.434	7.645			15	12	16.0979	16.4417	AAL94620.1| Type III restriction-modification system restriction subunit
					6		8.2501		

Show detected proteins only
Show all proteins
Filter by category:
GO: amino acid transport

Proteins found: 1305

Enter (or paste) list of ORFs
Find ORFs

Test	Cutoff
q-Value	.005
p-Value	

Color	Signif	Direction	Applies To
red	yes	+	ratios, bars
yellow	no	n/a	bars
green	yes	−	ratios, bars
pink	yes	+	*p*-, *q*-Values
light green	yes	−	*p*-, *q*-Values

Dot Plots | Dot Plots

Protein	FnPgSg vs FnPg Log$_2$ Ratio	Log$_2$ Sum	*q*-Value	*p*-Value	Raw *FnPgSg*	Raw *FnPg*	Normalized *FnPgSg*	Normalized *FnPg*	Description
**FN0418**	-3.964	8.168			4		4.2928		AAL94621.1| Uracil phosphoribosyltransferase
						67		67.0000	
**FN0419**	-0.968	6.780	1.024e-2	7.839e-3	5	9	5.3660	12.3313	AAL94622.1| Aspartate carbamoyltransferase
					7	17	9.6251	17.0000	
**FN0420**	-3.225	6.599			3	6	3.2196	8.2208	AAL94623.1| Dihydroorotase
						52		52.0000	
**FN0421**	-2.316	8.328			6		6.4392		AAL94624.1| Carbamoyl-phosphate synthase small chain
					7	40	9.6251	40.0000	
**FN0422**	-0.392	12.843	2.026e-1	6.076e-1	69	20	74.0505	27.4028	AAL94625.1| Carbamoyl-phosphate synthase large chain
					55	169	75.6261	169.0000	
**FN0423**	-1.386	6.614			5		5.3660		AAL94626.1| Dihydroorotate dehydrogenase electron transfer subunit
					5	16	6.8751	16.0000	
**FN0424**	-1.643	5.017			3	3	3.2196	4.1104	AAL94627.1| Dihydroorotate dehydrogenase
						16		16.0000	
**FN0426**	-0.596	9.561	1.705e-1	4.762e-1	25	7	26.8299	9.5910	AAL94629.1| Orotidine 5'-phosphate decarboxylase
					13	58	17.8753	58.0000	
**FN0427**	-0.244	11.133	4.001e-2	5.756e-2	44	41	47.2206	56.1757	AAL94630.1| Orotate phosphoribosyltransferase
					29	47	39.8756	47.0000	
**FN0430**	0.730	14.369	3.995e-2	5.745e-2	171	51	183.5165	69.8771	AAL94633.1| LSU ribosomal protein L19P
					139	156	191.1279	156.0000	
**FN0435**	-0.888	11.004	1.248e-1	3.081e-1	48	17	51.5134	23.2924	AAL94634.1| Purine nucleoside phosphorylase
					11	100	15.1252	100.0000	
**FN0436**	-1.698	10.539	2.224e-2	2.468e-2	13	35	13.9515	47.9549	AAL94635.1| regulator of kinase autophosphorylation inhibitor
					21	91	28.8754	91.0000	
**FN0437**	-0.877	6.536	1.355e-1	3.493e-1	3	3	3.2196	4.1104	AAL94636.1| kinase autophosphorylation inhibitor KipI
					8	22	11.0002	22.0000	
**FN0439**	-0.348	12.026	2.208e-1	6.794e-1	58	10	62.2454	13.7014	AAL94638.1| Lactam utilization protein LAMB
					38	132	52.2508	132.0000	
**FN0445**					6		6.4392		AAL94641.1| Hypothetical protein
					4		5.5001		
**FN0446**	0.441	6.714	7.506e-3	4.744e-3	12	7	12.8783	9.5910	AAL94642.1| Hypothetical protein
					8	8	11.0002	8.0000	

Log$_2$ Ratios: -6 -4 -2 0 2 4 6

Show detected proteins only
Show all proteins
Filter by category:
GO: amino acid transport

Proteins found: 1305

Enter (or paste) list of ORFs

Find ORFs

Test	Cutoff
q-Value	.005
p-Value	

	Signif	Direction	Applies To
red	yes	+	ratios, bars
yellow	no	n/a	bars
green	yes	−	ratios, bars
pink	yes	+	*p*- , *q*-Values
light green	yes	−	

Dot Plots Dot Plots

Protein	FnPgSg vs FnPg Log$_2$ Ratio	Log$_2$ Sum	*q*-Value	*p*-Value	Raw *FnPgSg*	Raw *FnPg*	Normalized *FnPgSg*	Normalized *FnPg*	Description
FN0447	-0.483	4.687			4		4.2928		AAL94643.1| NIFS protein
						6		6.0000	
FN0448									AAL94644.1| DNA-repair protein
					3		4.1251		
FN0450	0.197	8.552	9.774e-2	2.114e-1	22	14	23.6103	19.1820	AAL94646.1| ABC transporter ATP-binding protein
					13	17	17.8753	17.0000	
FN0451	-0.348	5.267							AAL94647.1| Hypothetical protein
					4	7	5.5001	7.0000	
FN0452	0.444	18.490	1.609e-1	4.405e-1	663	647	711.5288	886.4805	AAL94648.1| Glucosamine--fructose-6-phosphate aminotransferase (isomerizing)
					512	154	704.0106	154.0000	
FN0453	-1.007	16.282	7.377e-3	4.603e-3	198	255	212.4928	349.3857	AAL94649.1| Xaa-Pro aminopeptidase
					135	451	185.6278	451.0000	
FN0454	-1.363	13.375	5.399e-2	8.967e-2	57	69	61.1722	94.5396	AAL94650.1| Aldehyde dehydrogenase B
					49	236	67.3760	236.0000	
FN0455	0.106	17.315	1.844e-1	5.307e-1	331	315	355.2278	431.5941	AAL94651.1| Rubrerythrin
					351	347	482.6323	347.0000	
FN0456	1.270	8.967	1.495e-3	4.502e-4	34	13	36.4887	17.8118	AAL94652.1| Hypothetical cytosolic protein
					24	11	33.0005	11.0000	
FN0458	-0.888	6.451				12		16.4417	AAL94654.1| Hypothetical Exported Protein
					5	9	6.8751	9.0000	
FN0460	-1.301	10.313			18		19.3175		AAL94656.1| Delta-aminolevulinic acid dehydratase
					19	56	26.1254	56.0000	
FN0461	-0.798	16.288	8.224e-2	1.623e-1	187	381	200.6876	522.0233	AAL94657.1| Probable sigma(54) modulation protein
					166	224	228.2534	224.0000	
FN0462	-1.445	9.770	3.763e-2	5.285e-2	18	23	19.3175	31.5132	AAL94658.1| DNA mismatch repair protein mutL
					12	66	16.5002	66.0000	
FN0465	-1.526	15.038	8.973e-2	1.849e-1	104	377	111.6124	516.5427	AAL94661.1| Hypothetical protein
					76	106	104.5016	106.0000	
FN0466	-0.443	17.501	4.501e-2	6.802e-2	295	428	316.5927	586.4199	AAL94662.1| Lysyl-tRNA synthetase
					307	418	422.1314	418.0000	
FN0470	-0.229	18.019	9.585e-2	2.049e-1	427	343	458.2546	469.9580	AAL94666.1| Putative efflux pump component MtrF
					359	646	493.6324	646.0000	

Log$_2$ Ratios: -6 -4 -2 0 2 4 6

Show detected proteins only
Show all proteins
Filter by category:
GO: amino acid transport

Proteins found: 1305

Enter (or paste) list of ORFs

Find ORFs

Test	Cutoff
q-Value	.005
p-Value	

	Signif	Direction	Applies To
red	yes	+	ratios, bars
yellow	no	n/a	bars
green	yes	−	ratios, bars
pink	yes	+	*p*-, *q*-Values
light green	yes	−	

Dot Plots Dot Plots

Protein	FnPgSg vs FnPg Log$_2$ Ratio	Log$_2$ Sum	*q*-Value	*p*-Value	Raw *FnPgSg*	Raw *FnPg*	Normalized *FnPgSg*	Normalized *FnPg*	Description
FN0472	0.089	26.187	1.916e-1	5.605e-1	8444	7225	9062.0649	9899.2604	AAL94668.1| Flavodoxin
					6518	7052	8962.3851	7052.0000	
FN0475	-0.503	8.416			11		11.8052		AAL94671.1| MIAB protein
					14	22	19.2503	22.0000	
FN0476	0.593	12.038	4.505e-2	6.809e-2	69	26	74.0505	35.6236	AAL94672.1| Transcription termination factor rho
					62	70	85.2513	70.0000	
FN0477	-0.362	7.423	1.064e-2	8.346e-3	10	10	10.7320	13.7014	AAL94673.1| Cell wall endopeptidase family M23/M37
					9	16	12.3752	16.0000	
FN0478	-1.686	8.489			12		12.8783		AAL94674.1| GcpE protein
					6	34	8.2501	34.0000	
FN0481									AAL94677.1| unknown
						11		11.0000	
FN0482	3.233	8.403			59		63.3185		AAL94678.1| LSU ribosomal protein L31P
					36	6	49.5007	6.0000	
FN0483	0.718	12.927	2.902e-2	3.626e-2	111	34	119.1247	46.5848	AAL94679.1| Uracil phosphoribosyltransferase
					78	91	107.2516	91.0000	
FN0484	-0.220	4.424			4		4.2928		AAL94680.1| Lipase
						5		5.0000	
FN0487	0.119	20.282	2.164e-1	6.62e-1	1065	548	1142.9535	750.8366	AAL94683.1| 2-hydroxyglutarate dehydrogenase
					880	1416	1210.0182	1416.0000	
FN0488	-0.073	23.789	2.513e-1	8.049e-1	3228	1959	3464.2759	2684.1040	AAL94684.1| NAD-specific glutamate dehydrogenase
					2879	5124	3958.6847	5124.0000	
FN0489	1.675	8.594			36		38.6350		AAL94685.1| Prolipoprotein diacylglyceryl transferase
					23	11	31.6255	11.0000	
FN0491	-2.093	12.665	5.09e-2	8.176e-2	33	59	35.4155	80.8383	AAL94687.1| Alanine racemase
					31	252	42.6256	252.0000	
FN0493	-2.003	6.496	3.013e-2	3.827e-2	5	19	5.3660	26.0327	AAL94689.1| Hypothetical protein
					3	12	4.1251	12.0000	
FN0494	1.067	16.710	9.095e-4	2.072e-4	409	136	438.9371	186.3390	AAL94690.1| Short chain dehydrogenase
					370	266	508.7577	266.0000	
FN0495	-0.693	26.215	5.156e-5	2.831e-6	6337	8224	6800.8415	11268.0301	AAL94691.1| Acetyl-CoA acetyltransferase
					5153	11175	7085.4818	11175.0000	

Log$_2$ Ratios: -6 -4 -2 0 2 4 6

Show detected proteins only
Show all proteins
Filter by category:
GO: amino acid transport

Proteins found: 1305

Enter (or paste) list of ORFs

Find ORFs

Test	Cutoff
q-Value	.005
p-Value	

Signif	Direction	Applies To
yes	+	ratios, bars
no	n/a	bars
yes	–	ratios, bars
yes	+	*p*- , *q*-Values
yes	–	

Dot Plots Dot Plots

Protein	FnPgSg vs FnPg: Log_2 Ratio	Log_2 Sum	*q*-Value	*p*-Value	Raw: *FnPgSg*	Raw: *FnPg*	Normalized: *FnPgSg*	Normalized: *FnPg*	Description
FN0497									AAL94693.1| Plasmid addiction system poison protein
					5		6.8751		
FN0501	-1.208	16.157	5.254e-2	8.588e-2	156	183	167.4185	250.7356	AAL94697.1| Ornithine decarboxylase
					137	571	188.3778	571.0000	
FN0502	0.127	9.046			23		24.6835		AAL94698.1| Phosphoheptose isomerase
					17	22	23.3754	22.0000	
FN0503	0.456	10.900	7.943e-2	1.543e-1	48	18	51.5134	24.6625	AAL94699.1| Transcriptional regulatory protein, LYSR family
					37	50	50.8758	50.0000	
FN0504									AAL94700.1| Arginine permease
					3		4.1251		
FN0505	-1.445	10.779	5.441e-2	9.08e-2	23	28	24.6835	38.3639	AAL94701.1| Anthranilate synthase component II
					19	100	26.1254	100.0000	
FN0506	0.847	15.202	1.271e-2	1.085e-2	279	134	299.4216	183.5987	AAL94702.1| Arginyl-tRNA synthetase
					161	106	221.3783	106.0000	
FN0511	-1.270	10.311	1.143e-1	2.706e-1	21	10	22.5371	13.7014	AAL94707.1| D-lactate dehydrogenase
					17	97	23.3754	97.0000	
FN0512	-0.540	15.954	2.949e-7	1.311e-9	196	223	210.3464	305.5412	AAL94708.1| Flavoprotein
					151	302	207.6281	302.0000	
FN0513	2.104	9.160	5.135e-4	9.2e-5	45	11	48.2938	15.0715	AAL94709.1| Flavodoxin
					37	8	50.8758	8.0000	
FN0515									AAL94711.1| Acriflavin resistance protein D
						3		3.0000	
FN0516									AAL94712.1| Acriflavin resistance protein E
						4		4.0000	
FN0517									AAL94713.1| Outer membrane protein tolC
						5		5.0000	
FN0519	-1.477	5.566				8		10.9611	AAL94715.1| Hypothetical exported 24-amino acid repeat protein
					3	12	4.1251	12.0000	
FN0522	-1.278	5.366							AAL94718.1| Exonuclease SBCC
					3	10	4.1251	10.0000	
FN0523						3		4.1104	AAL94719.1| Exonuclease SBCD

Log_2 Ratios: -6 -4 -2 0 2 4 6

Show detected proteins only
Show all proteins
Filter by category:
GO: amino acid transport

Proteins found: 1305

Enter (or paste) list of ORFs

Find ORFs

Test	Cutoff
q-Value	.005
p-Value	

	Signif	Direction	Applies To
red	yes	+	ratios, bars
yellow	no	n/a	bars
green	yes	−	ratios, bars
pink	yes	+	*p*-, *q*-Values
light green	yes	−	

Dot Plots Dot Plots

Protein	FnPgSg vs FnPg Log_2 Ratio	Log_2 Sum	*q*-Value	*p*-Value	Raw *FnPgSg*	Raw *FnPg*	Normalized *FnPgSg*	Normalized *FnPg*	Description
**FN0524**	1.188	7.161	1.317e-3	3.751e-4	17	5	18.2443	6.8507	AAL94720.1| DNA helicase II
					13	9	17.8753	9.0000	
**FN0525**	0.038	13.901	2.818e-1	9.405e-1	108	38	115.9051	52.0653	AAL94721.1| Penicillin-binding protein
					98	192	134.7520	192.0000	
**FN0526**	-0.479	11.237			34		36.4887		AAL94722.1| Florfenicol resistance protein
					34	58	46.7507	58.0000	
**FN0527**	-1.927	8.810	5.171e-3	2.646e-3	10	26	10.7320	35.6236	AAL94723.1| Alanyl-tRNA synthetase
					8	47	11.0002	47.0000	
**FN0528**	1.857	17.912	9.068e-3	6.487e-3	951	340	1020.6092	465.8475	AAL94724.1| Cold shock protein
					633	56	870.3881	56.0000	
**FN0535**	0.476	11.748	1.946e-3	6.75e-4	61	39	65.4649	53.4355	AAL94731.1| Hypothetical protein
					53	46	72.8761	46.0000	
**FN0536**	0.579	15.463	3.396e-2	4.578e-2	242	94	259.7134	128.7931	AAL94732.1| DNA polymerase III, beta chain
					189	219	259.8789	219.0000	
**FN0540**	0.149	10.225	2.428e-1	7.688e-1	32	10	34.3423	13.7014	AAL94736.1| Glutamate-1-semialdehyde 2,1-aminomutase
					28	52	38.5006	52.0000	
**FN0542**	-0.621	9.000			17	3	18.2443	4.1104	AAL94738.1| Beta 1,4 glucosyltransferase
						52		52.0000	
**FN0543**	0.163	9.390	2.35e-1	7.363e-1	28	8	30.0495	10.9611	AAL94739.1| Lipopolysaccharide heptosyltransferase-1
					18	38	24.7504	38.0000	
**FN0544**									AAL94740.1| ADP-heptose:LPS heptosyltransferase II
						3		3.0000	
**FN0546**									AAL94742.1| Lipopolysaccharide core biosynthesis protein rfaQ
						6		6.0000	
**FN0547**	0.493	15.891	9.93e-2	2.168e-1	326	88	349.8618	120.5723	AAL94743.1| RecA protein
					171	295	235.1285	295.0000	
**FN0550**	-0.696	9.240			18	34	19.3175	46.5848	AAL94746.1| hypothetical Protein
						16		16.0000	
**FN0552**	-1.736	7.554			7	19	7.5124	26.0327	AAL94748.1| Serine racemase
						24		24.0000	
**FN0553**	-0.866	13.607	8.074e-2	1.58e-1	85	64	91.2216	87.6890	AAL94749.1| D-serine dehydratase
					54	214	74.2511	214.0000	

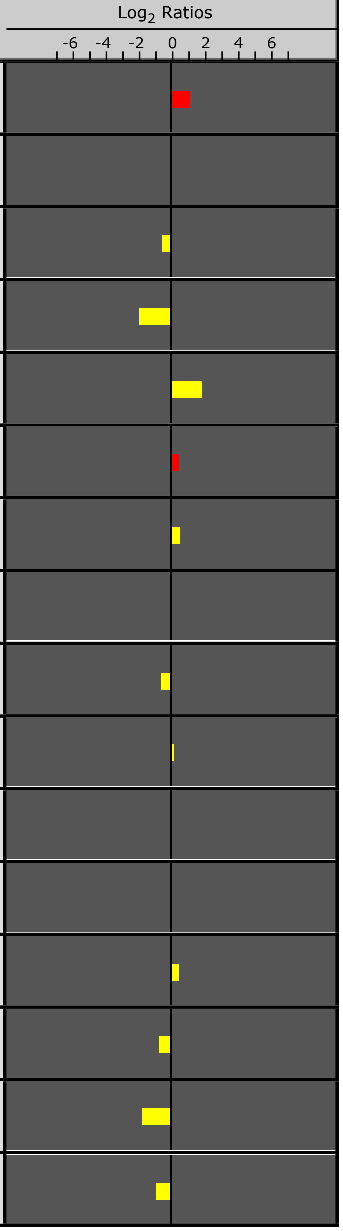

Show detected proteins only
Show all proteins
Filter by category:
GO: amino acid transport

Proteins found: 1305

Enter (or paste) list of ORFs

Find ORFs

Test	Cutoff
q-Value	.005
p-Value	

	Signif	Direction	Applies To
red	yes	+	ratios, bars
yellow	no	n/a	bars
green	yes	−	ratios, bars
pink	yes	+	*p*- , *q*-Values
light green	yes	−	

Dot Plots Dot Plots

Protein	*FnPgSg vs FnPg* Log$_2$ Ratio	Log$_2$ Sum	*q*-Value	*p*-Value	Raw *FnPgSg*	Raw *FnPg*	Normalized *FnPgSg*	Normalized *FnPg*	Description	Log$_2$ Ratios (-6 -4 -2 0 2 4 6)
**FN0554**	-2.399	5.773			3	8	3.2196	10.9611	AAL94750.1| D-serine permease	
						23		23.0000		
**FN0556**	-1.821	10.218	6.762e-2	1.232e-1	15	75	16.0979	102.7605	AAL94752.1| unknown	
					15	27	20.6253	27.0000		
**FN0557**	-2.576	11.851	3.589e-2	4.95e-2	31	59	33.2691	80.8383	AAL94753.1| unknown	
					12	216	16.5002	216.0000		
**FN0558**	-3.288	13.072			31		33.2691		AAL94754.1| TraT complement resistance protein precursor	
					19	290	26.1254	290.0000		
**FN0559**	-0.786	14.769	6.733e-2	1.224e-1	100	105	107.3196	143.8647	AAL94755.1| Phosphoglucomutase	
					107	295	147.1272	295.0000		
**FN0561**	-0.726	10.269	1.729e-2	1.737e-2	24	28	25.7567	38.3639	AAL94757.1| Proline synthetase associated protein	
					21	52	28.8754	52.0000		
**FN0562**	0.599	14.030	1.887e-2	1.958e-2	157	60	168.4917	82.2084	AAL94758.1| Hypothetical cytosolic protein	
					109	128	149.8773	128.0000		
**FN0563**	0.162	6.285	2.466e-1	7.849e-1	11	10	11.8052	13.7014	AAL94759.1| putative tRNA (5-methylaminomethyl-2-thiouridylate) -methyltransferase	
					5	3	6.8751	3.0000		
**FN0574**					4		4.2928		AAL94770.1| Hypothetical cytosolic protein	
**FN0576**	-0.250	10.474	1.173e-1	2.812e-1	26	25	27.9031	34.2535	AAL94772.1| hypothetical protein	
					30	48	41.2506	48.0000		
**FN0577**									AAL94773.1| Hypothetical protein	
						11		11.0000		
**FN0579**	-1.593	18.792	2.565e-2	3.024e-2	350	1109	375.6185	1519.4851	AAL94775.1| Hypothetical cytosolic protein	
					291	821	400.1310	821.0000		
**FN0580**									AAL94776.1| Penicillin-binding protein	
						4		4.0000		
**FN0581**	-0.219	5.781							AAL94777.1| Lipoprotein releasing system transmembrane protein lolE	
					5	8	6.8751	8.0000		
**FN0582**	0.210	8.550			26		27.9031		AAL94778.1| Lipoprotein releasing system ATP-binding protein lolD	
					10	18	13.7502	18.0000		
**FN0583**									AAL94779.1| Hypothetical Exported Protein	
						21		21.0000		

Show detected proteins only
Show all proteins
Filter by category:
GO: amino acid transport

Proteins found: 1305

Enter (or paste) list of ORFs
Find ORFs

Test	Cutoff
q-Value	.005
p-Value	

Color	Signif	Direction	Applies To
red	yes	+	ratios, bars
yellow	no	n/a	bars
green	yes	−	ratios, bars
pink	yes	+	*p*- , *q*-Values
light green	yes	−	

Dot Plots | Dot Plots

Protein	FnPgSg vs FnPg Log$_2$ Ratio	Log$_2$ Sum	*q*-Value	*p*-Value	Raw *FnPgSg*	Raw *FnPg*	Normalized *FnPgSg*	Normalized *FnPg*	Description	Log$_2$ Ratios (-6 -4 -2 0 2 4 6)
**FN0585**	-1.944	6.148			4	19	4.2928	26.0327	AAL94781.1| Two-component response regulator czcR	
						7		7.0000		
**FN0586**	-1.599	5.802			4		4.2928		AAL94782.1| Two-component sensor kinase czcS	
						13		13.0000		
**FN0590**	-2.019	7.489	6.446e-2	1.154e-1	6	8	6.4392	10.9611	AAL94786.1| N-acyl-L-amino acid amidohydrolase	
					5	43	6.8751	43.0000		
**FN0592**	-0.173	12.083	1.066e-1	2.44e-1	49	51	52.5866	69.8771	AAL94788.1| ATP-dependent DNA helicase pcrA	
					52	70	71.5011	70.0000		
**FN0593**	0.047	11.121	1.803e-1	5.145e-1	42	32	45.0742	43.8445	AAL94789.1| UDP-3-O-[3-hydroxymyristoyl] N-acetylglucosamine deacetylase	
					37	49	50.8758	49.0000		
**FN0594**	-4.406	10.610			8		8.5856		AAL94790.1| (3R)-hydroxymyristoyl-[acyl carrier protein] dehydratase	
						182		182.0000		
**FN0595**	0.403	9.913			14		15.0247		AAL94791.1| Acyl-[acyl-carrier-protein]-UDP-N-acetylglucosamine O-acyltransferase	
					41	27	56.3758	27.0000		
**FN0596**	-0.441	7.815			12	8	12.8783	10.9611	AAL94792.1| Hypothetical protein	
						24		24.0000		
**FN0597**	-1.184	9.156	2.985e-3	1.217e-3	18	27	19.3175	36.9938	AAL94793.1| Lipid-A-disaccharide synthase	
					9	35	12.3752	35.0000		
**FN0598**	0.864	8.436	1.403e-2	1.267e-2	25	7	26.8299	9.5910	AAL94794.1| Phospholipid-lipopolysaccharide ABC transporter	
					17	18	23.3754	18.0000		
**FN0600**	0.296	12.609	2.003e-1	5.977e-1	94	18	100.8804	24.6625	AAL94796.1| Hypothetical protein	
					54	118	74.2511	118.0000		
**FN0602**	-0.033	13.917	2.575e-1	8.315e-1	110	107	118.0515	146.6050	AAL94798.1| Hypothetical protein	
					93	105	127.8769	105.0000		
**FN0603**									AAL94799.1| Transcriptional regulatory protein, LYSR family	
						8		8.0000		
**FN0605**									AAL94801.1| Aspartate aminotransferase	
					5		6.8751			
**FN0608**	-1.241	12.447	3.975e-2	5.704e-2	47	56	50.4402	76.7278	AAL94804.1| Exoribonuclease II	
					34	153	46.7507	153.0000		
**FN0609**									AAL94805.1| Small protein B	
						6		6.0000		

Show detected proteins only
Show all proteins
Filter by category:
GO: amino acid transport

Proteins found: 1305

Enter (or paste) list of ORFs

Find ORFs

Test	Cutoff
q-Value	.005
p-Value	

	Signif	Direction	Applies To
(red)	yes	+	ratios, bars
(yellow)	no	n/a	bars
(green)	yes	–	ratios, bars
(pink)	yes	+	*p*-, *q*-Values
(light green)	yes	–	

Dot Plots | Dot Plots

Protein	FnPgSg vs FnPg Log$_2$ Ratio	Log$_2$ Sum	*q*-Value	*p*-Value	Raw *FnPgSg*	Raw *FnPg*	Normalized *FnPgSg*	Normalized *FnPg*	Description
FN0610	-0.157	15.454	2.281e-1	7.085e-1	201	93	215.7123	127.4230	AAL94806.1| unknown
					135	320	185.6278	320.0000	
FN0611	-0.502	18.161	8.272e-3	5.665e-3	471	461	505.4752	631.6345	AAL94807.1| Threonyl-tRNA synthetase
					294	657	404.2561	657.0000	
FN0612	1.495	12.095	3.393e-2	4.572e-2	66	13	70.8309	17.8118	AAL94808.1| Hypothetical protein
					110	61	151.2523	61.0000	
FN0615						3		4.1104	AAL94811.1| Export ABC transporter
FN0616	2.583	8.198			50		53.6598		AAL94812.1| Hypothetical protein
					22	7	30.2505	7.0000	
FN0617	-0.985	15.912	8.778e-2	1.788e-1	170	380	182.4433	520.6531	AAL94813.1| DNA polymerase III, beta chain
					124	178	170.5026	178.0000	
FN0618	-0.609	13.016	1.303e-1	3.288e-1	72	40	77.2701	54.8056	AAL94814.1| Spermidine/putrescine-binding protein
					51	170	70.1261	170.0000	
FN0619	0.192	7.646				4		5.4806	AAL94815.1| Small-conductance mechanosensitive channel
					11	21	15.1252	21.0000	
FN0621	0.106	11.642	2.437e-1	7.723e-1	67	54	71.9041	73.9876	AAL94817.1| 4-hydroxybutyrate coenzyme A transferase
					33	35	45.3757	35.0000	
FN0622	-3.105	7.252	4.485e-2	6.767e-2	4	12	4.2928	16.4417	AAL94818.1| 8-oxoguanine DNA glycosylase
					3	56	4.1251	56.0000	
FN0625	1.071	9.793			51	15	54.7330	20.5521	AAL94821.1| Aspartate aminotransferase
					23		31.6255		
FN0627	1.648	14.798	6.612e-3	3.834e-3	284	37	304.7876	50.6952	AAL94823.1| Glucosamine--fructose-6-phosphate aminotransferase (isomerizing)
					213	140	292.8794	140.0000	
FN0628					20		21.4639		AAL94824.1| Glucosamine--fructose-6-phosphate aminotransferase (isomerizing)
					14		19.2503		
FN0629	1.353	9.353			39		41.8546		AAL94825.1| PTS system, IID component
					29	16	39.8756	16.0000	
FN0630					34		36.4887		AAL94826.1| PTS system, IIC component
					11		15.1252		
FN0631	-0.378	7.797			9		9.6588		AAL94827.1| PTS system, IIB component
					12	17	16.5002	17.0000	

Log$_2$ Ratios: -6 -4 -2 0 2 4 6

Show detected proteins only
Show all proteins
Filter by category: GO: amino acid transport

Proteins found: 1305

Enter (or paste) list of ORFs

Find ORFs

Test	Cutoff
q-Value	.005
p-Value	

	Signif	Direction	Applies To
red	yes	+	ratios, bars
yellow	no	n/a	bars
green	yes	−	ratios, bars
pink	yes	+	*p*-, *q*-Values
light green	yes	−	

Dot Plots Dot Plots

Protein	FnPgSg vs FnPg Log$_2$ Ratio	Log$_2$ Sum	*q*-Value	*p*-Value	Raw *FnPgSg*	Raw *FnPg*	Normalized *FnPgSg*	Normalized *FnPg*	Description
FN0633	-1.077	6.737			3		3.2196		AAL94829.1| Replication protein
					8	15	11.0002	15.0000	
FN0634	0.016	13.546	2.897e-1	9.78e-1	105	131	112.6856	179.4883	AAL94830.1| GTP-binding protein TypA/BipA
					78	38	107.2516	38.0000	
FN0637					4		4.2928		AAL94833.1| Hypothetical exported 24-amino acid repeat protein
					5		6.8751		
FN0643	-1.016	8.785	4.744e-2	7.347e-2	16	29	17.1711	39.7341	AAL94839.1| hypothetical DNA-binding protein
					9	20	12.3752	20.0000	
FN0644	-0.395	10.952	9.26e-2	1.941e-1	48	38	51.5134	52.0653	AAL94840.1| Uroporphyrin-III C-methyltransferase
					19	50	26.1254	50.0000	
FN0645	-0.136	5.670	2.437e-1	7.726e-1	5	8	5.3660	10.9611	AAL94841.1| Porphobilinogen deaminase
					6	4	8.2501	4.0000	
FN0646					11		11.8052		AAL94842.1| Glutamyl-tRNA reductase
FN0647					5		5.3660		AAL94843.1| transcriptional regulator
FN0649						6		8.2208	AAL94845.1| Exoenzymes regulatory protein aepA precursor
FN0652	-1.204	23.474	5.014e-2	7.989e-2	2302	2352	2470.4966	3222.5689	AAL94848.1| Glyceraldehyde 3-phosphate dehydrogenase
					1474	7137	2026.7806	7137.0000	
FN0653	-1.484	10.535	2.845e-2	3.52e-2	16	32	17.1711	43.8445	AAL94849.1| unknown
					21	85	28.8754	85.0000	
FN0654	-0.528	18.139	5.338e-2	8.806e-2	366	368	392.7896	504.2115	AAL94850.1| Phosphoglycerate kinase
					365	786	501.8826	786.0000	
FN0655	2.287	9.206			36		38.6350		AAL94851.1| unknown
					50	11	68.7510	11.0000	
FN0656					33		35.4155		AAL94852.1| Hypothetical protein
					26		35.7505		
FN0657									AAL94853.1| Acetyltransferase
						22		22.0000	
FN0658	1.414	13.378	1.064e-6	1.419e-8	155	47	166.3453	64.3966	AAL94854.1| ABC transporter substrate-binding protein
					124	62	170.5026	62.0000	

Log$_2$ Ratios: -6 -4 -2 0 2 4 6

Show detected proteins only
Show all proteins
Filter by category:
GO: amino acid transport

Proteins found: 1305

Enter (or paste) list of ORFs

Find ORFs

Test	Cutoff
q-Value	.005
p-Value	

Color	Signif	Direction	Applies To
red	yes	+	ratios, bars
yellow	no	n/a	bars
green	yes	−	ratios, bars
pink	yes	+	*p*- , *q*-Values
light green	yes	−	

Dot Plots | Dot Plots

Protein	FnPgSg vs FnPg Log2 Ratio	Log2 Sum	*q*-Value	*p*-Value	Raw *FnPgSg*	Raw *FnPg*	Normalized *FnPgSg*	Normalized *FnPg*	Description
**FN0660**	-0.340	6.351	1.009e-1	2.225e-1	6	9	6.4392	12.3313	AAL94856.1| ABC transporter ATP-binding protein
					7	8	9.6251	8.0000	
**FN0662**	-0.121	11.303	2.691e-1	8.829e-1	45	5	48.2938	6.8507	AAL94858.1| Formiminoglutamase
					35	98	48.1257	98.0000	
**FN0664**	0.742	12.874	5.797e-2	9.97e-2	123	73	132.0031	100.0202	AAL94860.1| 2-nitropropane dioxygenase
					67	34	92.1264	34.0000	
**FN0668**	-0.710	7.754	1.171e-1	2.803e-1	15	7	16.0979	9.5910	AAL94864.1| High-affinity zinc uptake system protein znuA precursor
					5	28	6.8751	28.0000	
**FN0672**	0.629	6.629							AAL94868.1| ATPase
					9	8	12.3752	8.0000	
**FN0675**	-0.340	20.634	2.088e-1	6.325e-1	974	331	1045.2927	453.5163	AAL94871.1| 60 kDa chaperonin GROEL
					889	2417	1222.3934	2417.0000	
**FN0676**	0.993	11.977			76		81.5629		AAL94872.1| 10 kDa chaperonin GROES
					71	45	97.6265	45.0000	
**FN0677**	-1.397	10.579	3.454e-2	4.698e-2	18	62	19.3175	84.9487	AAL94873.1| Hypothetical protein
					21	42	28.8754	42.0000	
**FN0678**	1.217	10.191	3.294e-2	4.369e-2	51	5	54.7330	6.8507	AAL94874.1| Ser/Thr protein kinase
					36	38	49.5007	38.0000	
**FN0679**	-1.990	6.079				21		28.7729	AAL94875.1| GTPase
					3	4	4.1251	4.0000	
**FN0680**	-0.422	5.578			6		6.4392		AAL94876.1| Ribulose-phosphate 3-epimerase
					4	8	5.5001	8.0000	
**FN0681**	-0.701	15.810	2.402e-2	2.75e-2	176	260	188.8825	356.2364	AAL94877.1| Transcriptional regulator, MarR family
					136	255	187.0028	255.0000	
**FN0682**	-0.111	8.535	2.443e-1	7.748e-1	23	19	24.6835	26.0327	AAL94878.1| Fibronectin-binding protein-like protein A
					9	14	12.3752	14.0000	
**FN0684**	-2.867	6.240			3	8	3.2196	10.9611	AAL94880.1| Prismane protein
						36		36.0000	
**FN0685**	-3.531	9.978			11		11.8052		AAL94881.1| Sodium/pantothenate symporter
					5	108	6.8751	108.0000	
**FN0688**	-1.621	7.994			8		8.5856		AAL94884.1| Hypothetical protein
					7	28	9.6251	28.0000	

Log2 Ratios: -6 -4 -2 0 2 4 6

Show detected proteins only
Show all proteins
Filter by category:
GO: amino acid transport

Proteins found: 1305

Enter (or paste) list of ORFs

Find ORFs

Test	Cutoff
q-Value	.005
p-Value	

Signif	Direction	Applies To
yes	+	ratios, bars
no	n/a	bars
yes	–	ratios, bars
yes	+	*p*- , *q*-Values
yes	–	

Dot Plots Dot Plots

Protein	*FnPgSg vs FnPg* Log$_2$ Ratio	Log$_2$ Sum	*q*-Value	*p*-Value	Raw *FnPgSg*	Raw *FnPg*	Normalized *FnPgSg*	Normalized *FnPg*	Description
FN0689	0.123	13.758	2.44e-1	7.739e-1	120	42	128.7835	57.5459	AAL94885.1| Hypothetical protein
					85	168	116.8768	168.0000	
FN0692									AAL94888.1| Nitrogen regulation protein NIFR3
					3		4.1251		
FN0693	-0.800	7.923			11	15	11.8052	20.5521	AAL94889.1| DNA mismatch repair protein mutS
FN0694	0.324	10.894	2.604e-2	3.092e-2	41	27	44.0010	36.9938	AAL94890.1| S-layer protein
					39	41	53.6258	41.0000	
FN0695	-0.077	9.403	2.599e-1	8.421e-1	28	12	30.0495	16.4417	AAL94891.1| ABC transporter ATP-binding protein
					15	37	20.6253	37.0000	
FN0697	0.610	14.744	8.259e-3	5.648e-3	202	114	216.7855	156.1959	AAL94893.1| Alanyl-tRNA synthetase
					140	112	192.5029	112.0000	
FN0699	1.455	11.509	6.894e-3	4.104e-3	82	33	88.0021	45.2146	AAL94895.1| Protein translocase subunit secD
					66	20	90.7514	20.0000	
FN0700	0.847	9.046	2.532e-2	2.968e-2	28	17	30.0495	23.2924	AAL94896.1| Protein translocase subunit secF
					23	11	31.6255	11.0000	
FN0701	0.082	14.930	7.371e-2	1.388e-1	176	120	188.8825	164.4168	AAL94897.1| Methyltransferase
					127	179	174.6276	179.0000	
FN0705	0.020	12.428	2.809e-1	9.364e-1	65	39	69.7577	53.4355	AAL94901.1| DNA polymerase I
					58	94	79.7512	94.0000	
FN0706					6		6.4392		AAL94902.1| Hypothetical cytosolic protein
FN0707	-0.572	6.849	1.271e-1	3.164e-1	10	14	10.7320	19.1820	AAL94903.1| Riboflavin kinase
					5	7	6.8751	7.0000	
FN0710	-0.314	11.337	5.238e-2	8.549e-2	44	47	47.2206	64.3966	AAL94906.1| Hypothetical protein
					32	49	44.0007	49.0000	
FN0711	-0.854	7.321			6		6.4392		AAL94907.1| Phosphopantothenate--cysteine ligase
					9	17	12.3752	17.0000	
FN0714						7		9.5910	AAL94910.1| NADH oxidase
						7		7.0000	
FN0715	0.717	15.170	8.524e-2	1.711e-1	264	177	283.3237	242.5148	AAL94911.1| Hypothetical protein
					152	57	209.0032	57.0000	

Log$_2$ Ratios: -6 -4 -2 0 2 4 6

Show detected proteins only
Show all proteins
Filter by category:
GO: amino acid transport

Proteins found: 1305

Enter (or paste) list of ORFs

Find ORFs

Test	Cutoff
q-Value	.005
p-Value	

Signif	Direction	Applies To
yes	+	ratios, bars
no	n/a	bars
yes	−	ratios, bars
yes	+	*p*- , *q*-Values
yes	−	

Dot Plots Dot Plots

Protein	*FnPgSg vs FnPg* Log$_2$ Ratio	Log$_2$ Sum	*q*-Value	*p*-Value	Raw *FnPgSg*	Raw *FnPg*	Normalized *FnPgSg*	Normalized *FnPg*	Description
**FN0716**	-0.331	10.001	1.889e-1	5.491e-1	25	40	26.8299	54.8056	AAL94912.1| hypothetical protein
					22	17	30.2505	17.0000	
**FN0717**					5		5.3660		AAL94913.1| Ribosomal small subunit pseudouridine synthase A
**FN0720**	-0.610	11.783	2.682e-2	3.226e-2	46	45	49.3670	61.6563	AAL94916.1| Protein Translation Elongation Factor P (EF-P)
					34	85	46.7507	85.0000	
**FN0721**	1.392	9.191	9.474e-3	6.935e-3	32	5	34.3423	6.8507	AAL94917.1| Hypothetical protein
					32	23	44.0007	23.0000	
**FN0722**	-1.198	5.402			4	10	4.2928	13.7014	AAL94918.1| WD-repeat family protein
						6		6.0000	
**FN0723**									AAL94919.1| Hypothetical protein
						4		4.0000	
**FN0724**	-0.183	11.305	9.02e-2	1.864e-1	38	41	40.7814	56.1757	AAL94920.1| Flavodoxin
					39	51	53.6258	51.0000	
**FN0725**	-0.623	7.485	1.33e-1	3.392e-1	6	6	6.4392	8.2208	AAL94921.1| Molybdopterin biosynthesis MoeB protein
					11	25	15.1252	25.0000	
**FN0728**	-0.092	10.709	2.338e-2	2.648e-2	38	31	40.7814	42.4743	AAL94924.1| Hypothetical protein
					28	42	38.5006	42.0000	
**FN0729**	0.074	14.548	2.571e-1	8.297e-1	146	153	156.6866	209.6314	AAL94925.1| Phosphoglycerate mutase
					117	92	160.8774	92.0000	
**FN0731**	-1.028	9.912	6.179e-2	1.091e-1	20	45	21.4639	61.6563	AAL94927.1| Hypothetical protein
					16	27	22.0003	27.0000	
**FN0733**	1.123	13.570	3.011e-3	1.233e-3	147	66	157.7598	90.4292	AAL94929.1| Peptidase T
					122	59	167.7525	59.0000	
**FN0735**	-0.072	13.661	2.727e-1	8.987e-1	89	136	95.5144	186.3390	AAL94931.1| Cell surface protein
					92	47	126.5019	47.0000	
**FN0736**	0.349	5.716	1.385e-1	3.605e-1	5	5	5.3660	6.8507	AAL94932.1| Methyltransferase
					8	6	11.0002	6.0000	
**FN0737**	-0.908	8.707			15		16.0979		AAL94933.1| Hypothetical protein
					10	28	13.7502	28.0000	
**FN0738**	-0.894	9.757	9.526e-3	6.993e-3	21	33	22.5371	45.2146	AAL94934.1| Hypothetical exported 24-amino acid repeat protein
					15	35	20.6253	35.0000	

Log$_2$ Ratios: -6 -4 -2 0 2 4 6

Show detected proteins only
Show all proteins
Filter by category:
GO: amino acid transport

Proteins found: 1305

Enter (or paste) list of ORFs

Find ORFs

Test	Cutoff
q-Value	.005
p-Value	

	Signif	Direction	Applies To
red	yes	+	ratios, bars
yellow	no	n/a	bars
green	yes	−	ratios, bars
light red	yes	+	*p*- , *q*-Values
light green	yes	−	

Dot Plots Dot Plots

Protein	FnPgSg vs FnPg Log$_2$ Ratio	Log$_2$ Sum	*q*-Value	*p*-Value	Raw *FnPgSg*	Raw *FnPg*	Normalized *FnPgSg*	Normalized *FnPg*	Description
FN0739	1.104	16.041	5.335e-4	9.82e-5	338	125	362.7402	171.2675	AAL94935.1| Formiminotetrahydrofolate cyclodeaminase
					290	183	398.7560	183.0000	
FN0740	-0.393	16.992	1.717e-1	4.807e-1	308	147	330.5443	201.4106	AAL94936.1| Imidazolonepropionase
					218	626	299.7545	626.0000	
FN0741	0.542	18.574	8.821e-2	1.802e-1	659	206	707.2360	282.2488	AAL94937.1| Glutamate formiminotransferase
					582	753	800.2621	753.0000	
FN0742	-0.060	11.605			66		70.8309		AAL94938.1| unknown
					28	57	38.5006	57.0000	
FN0743					13		13.9515		AAL94939.1| ATP-dependent helicase, DinG family
					9		12.3752		
FN0745	0.039	13.594	2.765e-1	9.162e-1	114	47	122.3443	64.3966	AAL94941.1| metal dependent phosphohydrolase
					75	155	103.1266	155.0000	
FN0746						17		23.2924	AAL94942.1| Hypothetical Metal-Binding Protein
						7		7.0000	
FN0749									AAL94945.1| Hypothetical protein
					4		5.5001		
FN0750	-1.055	7.594	2.892e-2	3.608e-2	9	11	9.6588	15.0715	AAL94946.1| Hypothetical protein
					7	25	9.6251	25.0000	
FN0751	-0.070	7.927	2.538e-1	8.153e-1	13	8	13.9515	10.9611	AAL94947.1| L-asparaginase I
					12	21	16.5002	21.0000	
FN0752	-1.180	6.584	1.005e-1	2.212e-1	7	4	7.5124	5.4806	AAL94948.1| Proline iminopeptidase
					4	24	5.5001	24.0000	
FN0753	0.384	13.604	4.261e-4	6.832e-5	121	74	129.8567	101.3903	AAL94949.1| Glutamyl-tRNA(Gln) amidotransferase subunit B
					91	94	125.1269	94.0000	
FN0754	0.132	13.730	1.904e-1	5.553e-1	130	64	139.5154	87.6890	AAL94950.1| Glutamyl-tRNA(Gln) amidotransferase subunit A
					76	135	104.5016	135.0000	
FN0755	0.249	10.243	6.245e-2	1.106e-1	31	24	33.2691	32.8834	AAL94951.1| Glutamyl-tRNA(Gln) amidotransferase subunit C
					31	31	42.6256	31.0000	
FN0758	-0.315	14.064	2.591e-2	3.069e-2	111	97	119.1247	132.9036	AAL94954.1| Rod shape-determining protein mreB
					84	159	115.5017	159.0000	
FN0761	-1.488	9.008			15		16.0979		AAL94957.1| Bvg accessory factor
					8	38	11.0002	38.0000	

Log$_2$ Ratios: -6 -4 -2 0 2 4 6

Show detected proteins only
Show all proteins
Filter by category:
GO: amino acid transport

Proteins found: 1305

Enter (or paste) list of ORFs

Find ORFs

Test	Cutoff
q-Value	.005
p-Value	

Color	Signif	Direction	Applies To
red	yes	+	ratios, bars
yellow	no	n/a	bars
green	yes	−	ratios, bars
pink	yes	+	*p*-, *q*-Values
light green	yes	−	

Dot Plots Dot Plots

Protein	FnPgSg vs FnPg Log_2 Ratio	Log_2 Sum	*q*-Value	*p*-Value	Raw *FnPgSg*	Raw *FnPg*	Normalized *FnPgSg*	Normalized *FnPg*	Description
FN0765	-0.705	4.909			4		4.2928		AAL94961.1| tRNA (5-methylaminomethyl -2-thiouridylate) -methyltransferase
						7		7.0000	
FN0766									AAL94962.1| Large-conductance mechanosensitive channel
					20		27.5004		
FN0768	0.102	4.102			4		4.2928		AAL94964.1| Hemin receptor
						4		4.0000	
FN0774	1.049	13.142	9.962e-3	7.501e-3	105	60	112.6856	82.2084	AAL94970.1| Hypothetical cytosolic protein
					117	50	160.8774	50.0000	
FN0775	-0.334	16.039	1.62e-1	4.443e-1	218	126	233.9567	172.6376	AAL94971.1| Aspartyl aminopeptidase
					166	410	228.2534	410.0000	
FN0776	-0.264	12.011	1.524e-2	1.451e-2	58	48	62.2454	65.7667	AAL94972.1| Aspartate-ammonia ligase
					40	75	55.0008	75.0000	
FN0777	1.076	12.019	1.108e-4	1.037e-5	91	29	97.6608	39.7341	AAL94973.1| GTP-binding protein lepA
					65	49	89.3763	49.0000	
FN0778	-0.074	7.912				5		6.8507	AAL94974.1| Methyltransferase
					11	25	15.1252	25.0000	
FN0779									AAL94975.1| Putative GTPases (G3E family)
						5		5.0000	
FN0783	0.817	24.016	1.433e-2	1.31e-2	5918	2527	6351.1725	3462.3434	AAL94979.1| acyl-CoA dehydrogenase
					3331	2744	4580.1940	2744.0000	
FN0784	0.218	22.331	1.348e-1	3.467e-1	1997	1903	2143.1719	2607.3761	AAL94980.1| Electron transfer flavoprotein beta-subunit
					2044	1653	2810.5424	1653.0000	
FN0785	-0.366	21.485	1.978e-2	2.089e-2	1458	1553	1564.7194	2127.8272	AAL94981.1| Electron transfer flavoprotein alpha-subunit
					1057	1763	1453.3969	1763.000	
FN0788	-0.581	9.475	1.342e-2	1.181e-2	24	25	25.7567	34.2535	AAL94984.1| unknown
					13	31	17.8753	31.0000	
FN0790	-0.174	5.022			5	3	5.3660	4.1104	AAL94986.1| Xylose repressor
						8		8.0000	
FN0791	-0.262	18.129	7.291e-2	1.367e-1	459	367	492.5969	502.8413	AAL94987.1| Histidine ammonia-lyase
					353	670	485.3823	670.0000	
FN0792	-0.382	20.099	1.319e-1	3.348e-1	823	573	883.2401	785.0901	AAL94988.1| Urocanate hydratase
					708	1635	973.5147	1635.0000	

Log_2 Ratios: -6 -4 -2 0 2 4 6

Show detected proteins only
Show all proteins
Filter by category:
GO: amino acid transport

Proteins found: 1305

Enter (or paste) list of ORFs

Find ORFs

Test	Cutoff
q-Value	.005
p-Value	

	Signif	Direction	Applies To
red	yes	+	ratios, bars
yellow	no	n/a	bars
green	yes	–	ratios, bars
pink	yes	+	*p*- , *q*-Values
light green	yes	–	

Dot Plots Dot Plots

Protein	FnPgSg vs FnPg Log$_2$ Ratio	Log$_2$ Sum	*q*-Value	*p*-Value	Raw *FnPgSg*	Raw *FnPg*	Normalized *FnPgSg*	Normalized *FnPg*	Description
**FN0793**	1.334	8.504							AAL94989.1| Sodium/glutamate symport carrier protein
					22	12	30.2505	12.0000	
**FN0794**	-0.998	6.816			7		7.5124		AAL94990.1| Hypothetical protein
						15		15.0000	
**FN0796**	-0.387	14.519	6.494e-2	1.166e-1	128	104	137.3691	142.4945	AAL94992.1| Pyruvate,phosphate dikinase
					95	208	130.6270	208.0000	
**FN0798**	0.512	7.554	1.03e-1	2.302e-1	10	8	10.7320	10.9611	AAL94994.1| Fructose-1,6-bisphosphatase
					16	12	22.0003	12.0000	
**FN0799**	-0.589	8.193			13	8	13.9515	10.9611	AAL94995.1| Isoamylase
						31		31.0000	
**FN0800**	1.601	11.811	1.504e-2	1.419e-2	110	5	118.0515	6.8507	AAL94996.1| Amino acid-binding protein
					66	62	90.7514	62.0000	
**FN0801**									AAL94997.1| Amino acid transport ATP-binding protein
						18		18.0000	
**FN0802**									AAL94998.1| Amino acid transport system permease protein
						6		6.0000	
**FN0803**	-0.030	11.480	2.651e-1	8.648e-1	55	46	59.0258	63.0264	AAL94999.1| Cytochrome C-TYPE biogenesis protein ccdA
					34	45	46.7507	45.0000	
**FN0805**	-1.738	5.884	1.516e-3	4.597e-4	4	11	4.2928	15.0715	AAL95001.1| Hypothetical protein
					3	13	4.1251	13.0000	
**FN0806**	0.253	12.312	5.498e-2	9.234e-2	81	45	86.9289	61.6563	AAL95002.1| SpoIID homolog
					50	69	68.7510	69.0000	
**FN0807**	-0.207	10.006	2.849e-2	3.526e-2	30	24	32.1959	32.8834	AAL95003.1| 3-deoxy-manno-octulosonate cytidylyltransferase
					20	36	27.5004	36.0000	
**FN0808**	-0.586	11.723	1.062e-2	8.316e-3	50	58	53.6598	79.4681	AAL95004.1| Phosphoglycerate mutase
					30	63	41.2506	63.0000	
**FN0809**	0.561	6.561			11		11.8052		AAL95005.1| 23S rRNA methyltransferase
						8		8.0000	
**FN0810**	-1.603	9.977	3.696e-3	1.683e-3	16	45	17.1711	61.6563	AAL95006.1| Low-specificity threonine aldolase
					14	49	19.2503	49.0000	
**FN0813**	-2.063	8.581			14		15.0247		AAL95009.1| Transcriptional regulator, TetR family
					3	40	4.1251	40.0000	

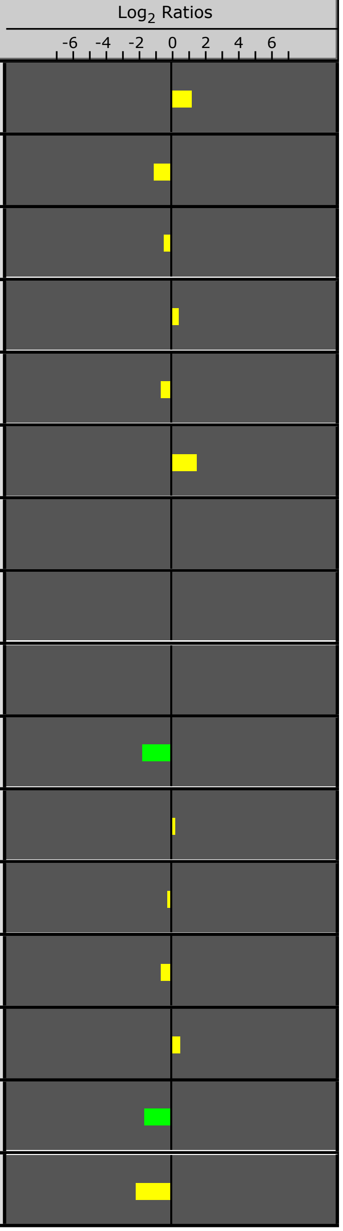

Show detected proteins only
Show all proteins
Filter by category:
GO: amino acid transport

Proteins found: 1305

Enter (or paste) list of ORFs

Find ORFs

Test	Cutoff
q-Value	.005
p-Value	

	Signif	Direction	Applies To
red	yes	+	ratios, bars
yellow	no	n/a	bars
green	yes	–	ratios, bars
pink	yes	+	*p*- , *q*-Values
light green	yes	–	

Dot Plots | Dot Plots

Protein	*FnPgSg vs FnPg* Log$_2$ Ratio	Log$_2$ Sum	*q*-Value	*p*-Value	Raw *FnPgSg*	Raw *FnPg*	Normalized *FnPgSg*	Normalized *FnPg*	Description
**FN0814**	3.210	12.677	1.028e-4	8.812e-6	241	33	258.6402	45.2146	AAL95010.1| Propionate CoA-transferase
					170	8	233.7535	8.0000	
**FN0815**					61		65.4649		AAL95011.1| Propionate permease
					41		56.3758		
**FN0816**	0.574	12.126			70	40	75.1237	54.8056	AAL95012.1| dehydrogenase with MaoC-like domain
					64		88.0013		
**FN0818**	0.777	19.073	3.084e-2	3.959e-2	852	567	914.3628	776.8693	AAL95014.1| DNA-binding protein HU
					749	358	1029.8905	358.0000	
**FN0819**	0.453	11.837	6.012e-2	1.051e-1	55	47	59.0258	64.3966	AAL95015.1| Tetratricopeptide repeat family protein
					60	39	82.5012	39.0000	
**FN0820**	-0.211	14.484	1.36e-1	3.515e-1	120	94	128.7835	128.7931	AAL95016.1| Mercuric reductase
					111	197	152.6273	197.0000	
**FN0821**	-0.841	11.986	1.092e-1	2.534e-1	40	96	42.9278	131.5334	AAL95017.1| Hypothetical protein
					38	39	52.2508	39.0000	
**FN0823**	-0.979	9.728	4.119e-2	5.994e-2	22	21	23.6103	28.7729	AAL95019.1| GTP-binding protein hflX
					13	53	17.8753	53.0000	
**FN0825**	-0.461	10.661	1.347e-1	3.462e-1	37	20	39.7082	27.4028	AAL95021.1| Hypothetical cytosolic protein
					21	67	28.8754	67.0000	
**FN0826**	1.282	8.916	4.308e-3	2.06e-3	28	14	30.0495	19.1820	AAL95022.1| periplasmic component of efflux system
					28	9	38.5006	9.0000	
**FN0827**	0.587	7.870	6.144e-3	3.415e-3	17	8	18.2443	10.9611	AAL95023.1| ABC transporter ATP-binding protein
					14	14	19.2503	14.0000	
**FN0828**					6		6.4392		AAL95024.1| ABC transporter permease protein
**FN0830**	-2.398	9.868	1.125e-3	2.942e-4	12	55	12.8783	75.3577	AAL95026.1| Hypothetical protein
					10	65	13.7502	65.0000	
**FN0831**						13		17.8118	AAL95027.1| Hemin receptor
						6		6.0000	
**FN0832**	0.117	12.865	2.631e-1	8.56e-1	92	105	98.7340	143.8647	AAL95028.1| Hypothetical protein
					59	22	81.1262	22.0000	
**FN0833**	1.516	6.160			10		10.7320		AAL95029.1| Hypothetical protein
					13	5	17.8753	5.0000	

Log$_2$ Ratios: -6 -4 -2 0 2 4 6

Show detected proteins only
Show all proteins
Filter by category:
GO: amino acid transport

Proteins found: 1305

Enter (or paste) list of ORFs

Find ORFs

Test	Cutoff
q-Value	.005
p-Value	

Signif	Direction	Applies To
yes	+	ratios, bars
no	n/a	bars
yes	–	ratios, bars
yes	+	*p*- , *q*-Values
yes	–	

Dot Plots | Dot Plots

Protein	FnPgSg vs FnPg Log$_2$ Ratio	Log$_2$ Sum	*q*-Value	*p*-Value	Raw *FnPgSg*	Raw *FnPg*	Normalized *FnPgSg*	Normalized *FnPg*	Description
FN0834	-0.455	9.246	1.962e-1	5.8e-1	20	37	21.4639	50.6952	AAL95030.1| Hypothetical Exported Protein
					15	7	20.6253	7.0000	
FN0835						27		36.9938	AAL95031.1| Hypothetical protein
						4		4.0000	
FN0836	-0.677	9.270	1.011e-1	2.233e-1	11	32	11.8052	43.8445	AAL95032.1| Hypothetical protein
					20	19	27.5004	19.0000	
FN0846	-1.126	12.326	1.663e-3	5.269e-4	43	83	46.1474	113.7216	AAL95042.1| Hypothetical Exported Protein
					37	98	50.8758	98.0000	
FN0847	-0.233	5.767			5		5.3660		AAL95043.1| TPR-repeat-containing proteins
					6	8	8.2501	8.0000	
FN0848						5		6.8507	AAL95044.1| Hypothetical protein
FN0849	-1.551	10.246	3.315e-6	7.371e-8	20	44	21.4639	60.2862	AAL95045.1| 8-amino-7-oxononanoate synthase
					14	59	19.2503	59.0000	
FN0850	-1.578	7.854	1.143e-2	9.277e-3	10	15	10.7320	20.5521	AAL95046.1| Hypothetical cytosolic protein
					5	32	6.8751	32.0000	
FN0853	-1.742	11.830	4.891e-3	2.447e-3	32	91	34.3423	124.6827	AAL95049.1| Glycogen synthase
					23	96	31.6255	96.0000	
FN0854	-1.404	13.323	6.587e-3	3.811e-3	66	139	70.8309	190.4494	AAL95050.1| Glucose-1-phosphate adenylyltransferase
					39	139	53.6258	139.0000	
FN0855	-1.327	13.719	2.082e-2	2.247e-2	70	102	75.1237	139.7543	AAL95051.1| Glucose-1-phosphate adenylyltransferase
					52	228	71.5011	228.0000	
FN0856	-1.851	12.942	1.324e-3	3.785e-4	55	124	59.0258	169.8973	AAL95052.1| 1,4-alpha-glucan branching enzyme
					25	167	34.3755	167.0000	
FN0857	-0.663	15.476	3.839e-2	5.435e-2	165	155	177.0773	212.3717	AAL95053.1| Glycogen phosphorylase
					118	325	162.2524	325.0000	
FN0858	-3.221	8.261	6.376e-4	1.248e-4	3	35	3.2196	47.9549	AAL95054.1| 4-alpha-glucanotransferase
					6	59	8.2501	59.0000	
FN0865	-3.055	12.095	8.846e-2	1.809e-1	12	17	12.8783	23.2924	AAL95061.1| unknown
					24	358	33.0005	358.0000	
FN0867	1.450	11.533	1.722e-3	5.555e-4	78	24	83.7093	32.8834	AAL95063.1| Long-chain-fatty-acid--CoA ligase
					70	33	96.2515	33.0000	

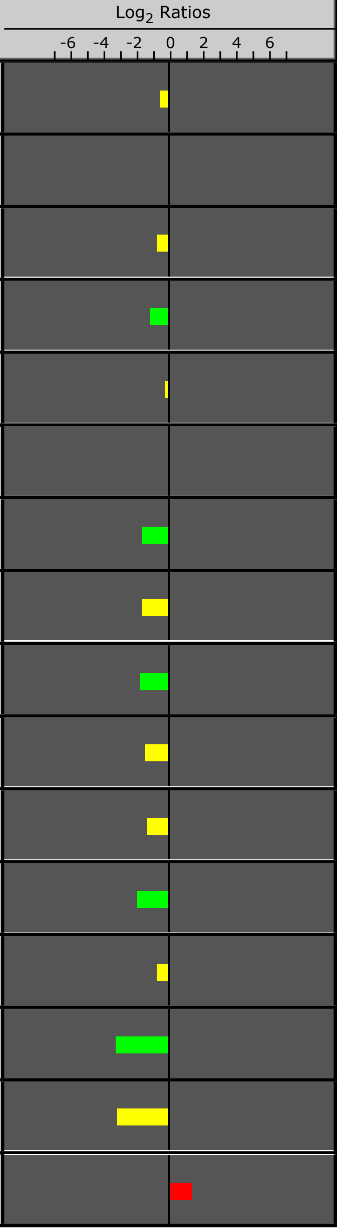

Show detected proteins only
Show all proteins
Filter by category:
GO: amino acid transport

Proteins found: 1305

Enter (or paste) list of ORFs

Find ORFs

Test	Cutoff
q-Value	.005
p-Value	

Signif	Direction	Applies To
yes	+	ratios, bars
no	n/a	bars
yes	−	ratios, bars
yes	+	*p*- , *q*-Values
yes	−	

Dot Plots | Dot Plots

Protein	FnPgSg vs FnPg: Log_2 Ratio	Log_2 Sum	*q*-Value	*p*-Value	Raw *FnPgSg*	Raw *FnPg*	Normalized *FnPgSg*	Normalized *FnPg*	Description
**FN0869**	-0.313	3.687			3		3.2196		AAL95065.1| Hydrolase (HAD superfamily)
						4		4.0000	
**FN0870**					10		10.7320		AAL95066.1| Rhodanese-related sulfurtransferases
					9		12.3752		
**FN0871**	-0.728	7.272			9		9.6588		AAL95067.1| 3-dehydroquinate synthase
						16		16.0000	
**FN0873**	-1.577	10.347	7.887e-2	1.527e-1	21	18	22.5371	24.6625	AAL95069.1| Protease IV
					14	100	19.2503	100.0000	
**FN0874**									AAL95070.1| Phosphohydrolase (MUTT/NUDIX family protein)
						11		11.0000	
**FN0875**					5		5.3660		AAL95071.1| 23S rRNA methyltransferase
**FN0878**	1.112	8.383	1.551e-2	1.494e-2	27	5	28.9763	6.8507	AAL95074.1| Transcriptional regulator, GntR family
					18	18	24.7504	18.0000	
**FN0886**					3		3.2196		AAL95082.1| Hemin receptor
**FN0887**	0.587	10.397	3.454e-2	4.699e-2	48	16	51.5134	21.9222	AAL95083.1| Oligoendopeptidase F
					28	38	38.5006	38.0000	
**FN0888**	0.754	8.976	3.335e-2	4.452e-2	21	15	22.5371	20.5521	AAL95084.1| Uracil permease
					26	14	35.7505	14.0000	
**FN0889**						37		50.6952	AAL95085.1| hypothetical protein
						9		9.0000	
**FN0893**	0.733	6.348			14		15.0247		AAL95089.1| Hypothetical protein
					6	7	8.2501	7.0000	
**FN0896**	0.187	7.824	2.098e-1	6.365e-1	12	6	12.8783	8.2208	AAL95092.1| Hypothetical protein
					14	20	19.2503	20.0000	
**FN0898**									AAL95094.1| Hypothetical protein
						5		5.0000	
**FN0900**						6		8.2208	AAL95096.1| Metal dependent hydrolase
						3		3.0000	
**FN0901**									AAL95097.1| DNA polymerase, bacteriophage-type
						9		9.0000	

Log2 Ratios: -6 -4 -2 0 2 4 6

Show detected proteins only
Show all proteins
Filter by category:
GO: amino acid transport

Proteins found: 1305

Enter (or paste) list of ORFs

Find ORFs

Test	Cutoff
q-Value	.005
p-Value	

	Signif	Direction	Applies To
red	yes	+	ratios, bars
yellow	no	n/a	bars
green	yes	–	ratios, bars
pink	yes	+	*p*-, *q*-Values
light green	yes	–	

Dot Plots | Dot Plots

Protein	*FnPgSg vs FnPg* Log_2 Ratio	Log_2 Sum	*q*-Value	*p*-Value	Raw *FnPgSg*	Raw *FnPg*	Normalized *FnPgSg*	Normalized *FnPg*	Description
FN0902					6		6.4392		AAL95098.1| 5-formyltetrahydrofolate cyclo-ligase
					8		11.0002		
FN0903	-0.527	8.607	2.865e-2	3.556e-2	14	20	15.0247	27.4028	AAL95099.1| Polysialic acid capsule expression protein kpsF
					13	20	17.8753	20.0000	
FN0904									AAL95100.1| NAD(FAD)-utilizing dehydrogenases
						4		4.0000	
FN0905					11		11.8052		AAL95101.1| Hypothetical protein
					10		13.7502		
FN0906	-0.981	8.100	3.369e-3	1.468e-3	13	15	13.9515	20.5521	AAL95102.1| Glycerol-3-phosphate dehydrogenase [NAD(P)+]
					7	26	9.6251	26.0000	
FN0908	0.159	7.973			12		12.8783		AAL95104.1| Tpl protein
					15	15	20.6253	15.0000	
FN0909						7		9.5910	AAL95105.1| DNA repair protein radC
FN0910									AAL95106.1| Nicotinate-nucleotide--dimethylbenzimidazole phosphoribosyltransferase
						8		8.0000	
FN0911	-0.261	6.079			7		7.5124		AAL95107.1| Alpha-ribazole-5'-phosphate phosphatase
						9		9.0000	
FN0912									AAL95108.1| Cobalamin [5'-phosphate] synthase
						19		19.0000	
FN0913	-1.635	5.009			3		3.2196		AAL95109.1| Cobinamide kinase
						10		10.0000	
FN0915	-0.471	10.179	1.562e-1	4.232e-1	27	14	28.9763	19.1820	AAL95111.1| PTS system, N-acetylglucosamine-specific IIA component
					21	61	28.8754	61.0000	
FN0916	1.052	15.226	7.365e-3	4.589e-3	301	70	323.0319	95.9098	AAL95112.1| Hypothetical Exported Protein
					175	176	240.6286	176.0000	
FN0917	0.044	4.044							AAL95113.1| Hypothetical protein
					3	4	4.1251	4.0000	
FN0920	-0.475	6.169			7		7.5124		AAL95116.1| Protease HTPX
					5	10	6.8751	10.0000	
FN0921	-2.019	8.964			13		13.9515		AAL95117.1| Hypothetical protein
					6	45	8.2501	45.0000	

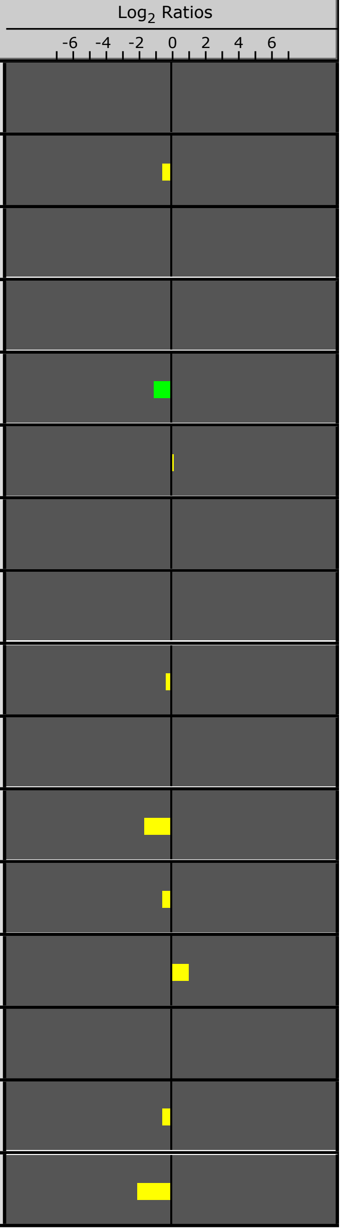

Show detected proteins only
Show all proteins
Filter by category:
GO: amino acid transport

Proteins found: 1305

Enter (or paste) list of ORFs

Find ORFs

Test	Cutoff
q-Value	.005
p-Value	

	Signif	Direction	Applies To
red	yes	+	ratios, bars
yellow	no	n/a	bars
green	yes	−	ratios, bars
pink	yes	+	*p*- , *q*-Values
light green	yes	−	

Dot Plots Dot Plots

Protein	FnPgSg vs FnPg Log$_2$ Ratio	Log$_2$ Sum	*q*-Value	*p*-Value	Raw *FnPgSg*	Raw *FnPg*	Normalized *FnPgSg*	Normalized *FnPg*	Description
FN0922					3		3.2196		AAL95118.1| Homoserine kinase
FN0923									AAL95119.1| Cardiolipin synthetase
						4		4.0000	
FN0924									AAL95120.1| Hypothetical protein
					3		4.1251		
FN0925	0.578	5.748			9		9.6588		AAL95121.1| Hypothetical protein
					6	6	8.2501	6.0000	
FN0926	-2.171	8.664	1.13e-4	1.085e-5	10	31	10.7320	42.4743	AAL95122.1| GTP pyrophosphokinase
					6	43	8.2501	43.0000	
FN0928	-0.654	5.990			8		8.5856		AAL95124.1| O-sialoglycoprotein endopeptidase
					3	10	4.1251	10.0000	
FN0929	-1.182	7.271				12		16.4417	AAL95125.1| ATP/GTP hydrolase
					6	21	8.2501	21.0000	
FN0930						7		9.5910	AAL95126.1| Glycerol-3-phosphate cytidylyltransferase
						3		3.0000	
FN0932	-1.329	6.485			6		6.4392		AAL95128.1| Hypothetical protein
					4	15	5.5001	15.0000	
FN0933					3		3.2196		AAL95129.1| 3-phosphoshikimate 1-carboxyvinyltransferase
FN0934									AAL95130.1| Chorismate synthase
						21		21.0000	
FN0938									AAL95134.1| Hypothetical protein
						47		47.0000	
FN0940	-1.827	8.888			10		10.7320		AAL95136.1| Hypothetical protein
					9	41	12.3752	41.0000	
FN0941	-1.101	11.022	6.863e-3	4.075e-3	26	42	27.9031	57.5459	AAL95137.1| Gamma-glutamyltranspeptidase
					25	76	34.3755	76.0000	
FN0943	-2.841	7.334			5		5.3660		AAL95139.1| Sensory Transduction Protein Kinase
					3	34	4.1251	34.0000	
FN0944									AAL95140.1| Na+ driven multidrug efflux pump
						4		4.0000	

Log$_2$ Ratios: -6 -4 -2 0 2 4 6

Show detected proteins only
Show all proteins
Filter by category:
GO: amino acid transport

Proteins found: 1305

Enter (or paste) list of ORFs

Find ORFs

Test	Cutoff
q-Value	.005
p-Value	

	Signif	Direction	Applies To
red	yes	+	ratios, bars
yellow	no	n/a	bars
green	yes	−	ratios, bars
pink	yes	+	*p*- , *q*-Values
light green	yes	−	*p*- , *q*-Values

Dot Plots Dot Plots

Protein	*FnPgSg vs FnPg* Log$_2$ Ratio	Log$_2$ Sum	*q*-Value	*p*-Value	Raw *FnPgSg*	Raw *FnPg*	Normalized *FnPgSg*	Normalized *FnPg*	Description
**FN0947**	0.359	13.050	1.111e-1	2.599e-1	88	80	94.4412	109.6112	AAL95143.1| Hypothetical protein
					83	53	114.1267	53.0000	
**FN0949**	0.379	14.813	1.925e-1	5.644e-1	185	196	198.5412	268.5474	AAL95145.1| DNA helicase
					137	29	188.3778	29.0000	
**FN0951**						4		5.4806	AAL95147.1| Precorrin-3B C17-methyltransferase
						17		17.0000	
**FN0957**	-0.064	9.114	2.587e-1	8.367e-1	16	22	17.1711	30.1431	AAL95153.1| Precorrin-4 C11-methyltransferase
					21	18	28.8754	18.0000	
**FN0958**						20		27.4028	AAL95154.1| unknown
**FN0959**	0.212	7.633	2.124e-1	6.467e-1	18	14	19.3175	19.1820	AAL95155.1| Precorrin-2 C20-methyltransferase
					8	7	11.0002	7.0000	
**FN0961**						6		8.2208	AAL95157.1| Hypothetical protein
						18		18.0000	
**FN0962**	-0.721	9.470	7.793e-3	5.072e-3	22	28	23.6103	38.3639	AAL95158.1| Hypothetical cytosolic protein
					13	30	17.8753	30.0000	
**FN0964**	0.098	5.465				5		6.8507	AAL95160.1| Precorrin-8W decarboxylase
					5	6	6.8751	6.0000	
**FN0965**	-0.478	11.451	4.843e-2	7.579e-2	31	51	33.2691	69.8771	AAL95161.1| D-3-phosphoglycerate dehydrogenase
					41	55	56.3758	55.0000	
**FN0966**									AAL95162.1| Precorrin-6Y C5,15-methyltransferase (decarboxylating)
						3		3.0000	
**FN0967**	-1.575	4.949			3	7	3.2196	9.5910	AAL95163.1| CbiD protein
**FN0970**	0.581	7.829			19	9	20.3907	12.3313	AAL95166.1| Precorrin-8X methylmutase
					12		16.5002		
**FN0972**						3		4.1104	AAL95168.1| Cobyrinic acid a,c-diamide synthase
						22		22.0000	
**FN0974**									AAL95170.1| Lactoylglutathione lyase
						4		4.0000	
**FN0976**	-0.113	10.941	1.466e-2	1.36e-2	41	33	44.0010	45.2146	AAL95172.1| Hypothetical protein
					30	47	41.2506	47.0000	

Show detected proteins only
Show all proteins
Filter by category:
GO: amino acid transport

Proteins found: 1305

Enter (or paste) list of ORFs

Find ORFs

Test	Cutoff
q-Value	.005
p-Value	

	Signif	Direction	Applies To
red	yes	+	ratios, bars
yellow	no	n/a	bars
green	yes	−	ratios, bars
pink	yes	+	*p*- , *q*-Values
light green	yes	−	

Dot Plots | Dot Plots

Protein	FnPgSg vs FnPg Log$_2$ Ratio	Log$_2$ Sum	*q*-Value	*p*-Value	Raw *FnPgSg*	Raw *FnPg*	Normalized *FnPgSg*	Normalized *FnPg*	Description
**FN0977**	0.440	7.934	1.22e-1	2.977e-1	16	5	17.1711	6.8507	AAL95173.1| Cobyric acid synthase
					14	20	19.2503	20.0000	
**FN0981**	0.767	13.495	5.68e-2	9.683e-2	142	29	152.3938	39.7341	AAL95177.1| Phosphoribosylamine--glycine ligase
					93	125	127.8769	125.0000	
**FN0982**	0.353	15.183	5.256e-2	8.594e-2	223	101	239.3227	138.3841	AAL95178.1| Phosphoribosylaminoimidazolecarboxamide formyltransferase
					143	203	196.6280	203.0000	
**FN0983**	-0.264	13.579	9.395e-2	1.985e-1	101	104	108.3928	142.4945	AAL95179.1| Hypothetical protein
					68	100	93.5014	100.0000	
**FN0984**	-1.717	8.256	5.642e-3	3.003e-3	9	20	9.6588	27.4028	AAL95180.1| Tetracenomycin polyketide synthesis O-methyltransferase tcmP
					7	36	9.6251	36.0000	
**FN0985**	1.047	7.047			18		19.3175		AAL95181.1| Phosphoribosylglycinamide formyltransferase
					10	8	13.7502	8.0000	
**FN0986**	0.056	14.906	2.727e-1	8.988e-1	151	64	162.0526	87.6890	AAL95182.1| Phosphoribosylformylglycinamidine cyclo-ligase
					142	256	195.2529	256.0000	
**FN0987**	-0.090	12.716	2.716e-1	8.939e-1	70	17	75.1237	23.2924	AAL95183.1| Amidophosphoribosyltransferase
					61	146	83.8763	146.0000	
**FN0988**	0.723	16.285	2.183e-2	2.406e-2	351	116	376.6917	158.9362	AAL95184.1| Phosphoribosylamidoimidazole-succinocarboxamide synthase
					254	281	349.2553	281.0000	
**FN0989**	0.212	15.265	5.29e-2	8.682e-2	184	121	197.4680	165.7869	AAL95185.1| Phosphoribosylaminoimidazole carboxylase catalytic subunit
					167	203	229.6285	203.0000	
**FN0990**	0.279	20.169	1.644e-1	4.532e-1	1063	411	1140.8071	563.1275	AAL95186.1| Phosphoribosylformylglycinamidine synthase
					910	1408	1251.2689	1408.0000	
**FN0991**	0.239	9.861	2.17e-2	2.385e-2	31	22	33.2691	30.1431	AAL95187.1| CDP-diacylglycerol--serine O-phosphatidyltransferase
					24	26	33.0005	26.0000	
**FN0992**	0.614	8.754	6.609e-2	1.194e-1	21	7	22.5371	9.5910	AAL95188.1| ADP-heptose:LPS heptosyltransferase II
					21	24	28.8754	24.0000	
**FN0994**	0.689	13.770	7.093e-2	1.315e-1	140	33	150.2474	45.2146	AAL95190.1| Hypothetical protein
					109	141	149.8773	141.0000	
**FN0997**	-0.400	10.273	1.803e-1	5.145e-1	34	13	36.4887	17.8118	AAL95193.1| Hypothetical protein
					18	63	24.7504	63.0000	
**FN0998**	-0.437	13.307	1.323e-1	3.367e-1	69	52	74.0505	71.2473	AAL95194.1| Dipeptide-binding protein
					72	163	99.0015	163.0000	

Log$_2$ Ratios: -6 -4 -2 0 2 4 6

Show detected proteins only
Show all proteins
Filter by category:
GO: amino acid transport

Proteins found: 1305

Enter (or paste) list of ORFs

Find ORFs

Test	Cutoff
q-Value	.005
p-Value	

Color	Signif	Direction	Applies To
red	yes	+	ratios, bars
yellow	no	n/a	bars
green	yes	−	ratios, bars
pink	yes	+	*p*- , *q*-Values
light green	yes	−	

Dot Plots | Dot Plots

Protein	FnPgSg vs FnPg Log$_2$ Ratio	Log$_2$ Sum	*q*-Value	*p*-Value	Raw *FnPgSg*	Raw *FnPg*	Normalized *FnPgSg*	Normalized *FnPg*	Description
**FN0999**	-2.963	8.807	6.27e-2	1.112e-1	9	14	9.6588	19.1820	AAL95195.1| Deblocking aminopeptidase
					4	99	5.5001	99.0000	
**FN1000**	1.050	9.958			41	16	44.0010	21.9222	AAL95196.1| Biotin synthase
					34		46.7507		
**FN1001**	-0.746	9.641	1.053e-1	2.39e-1	24	14	25.7567	19.1820	AAL95197.1| Dethiobiotin synthetase
					13	54	17.8753	54.0000	
**FN1002**	-0.828	11.337	7.835e-2	1.513e-1	34	69	36.4887	94.5396	AAL95198.1| Adenosylmethionine-8-amino -7-oxononanoate aminotransferase
					29	41	39.8756	41.0000	
**FN1003**	-2.119	13.112	1.106e-1	2.583e-1	47	6	50.4402	8.2208	AAL95199.1| Outer membrane protein P1 precursor
					29	384	39.8756	384.0000	
**FN1004**	-0.423	7.705	1.482e-2	1.385e-2	13	12	13.9515	16.4417	AAL95200.1| Transcriptional regulator, TetR family
					8	17	11.0002	17.0000	
**FN1005**	-0.264	9.910			22		23.6103		AAL95201.1| Hypothetical protein
					24	34	33.0005	34.0000	
**FN1006**									AAL95202.1| Acetyltransferase
						21		21.0000	
**FN1010**	0.065	14.806	2.626e-1	8.539e-1	165	73	177.0773	100.0202	AAL95206.1| Hypothetical cytosolic protein
					123	231	169.1275	231.0000	
**FN1011**	-1.022	5.940				9		12.3313	AAL95207.1| MGPA protein
					4	10	5.5001	10.0000	
**FN1012**	-1.421	8.405	4.169e-3	1.973e-3	12	25	12.8783	34.2535	AAL95208.1| HPR(Ser) kinase
					7	26	9.6251	26.0000	
**FN1014**	-1.725	7.194			6		6.4392		AAL95210.1| Folylpolyglutamate synthase
					5	22	6.8751	22.0000	
**FN1015**	-0.665	9.051			20		21.4639		AAL95211.1| 5'-methylthioadenosine nucleosidase
					11	29	15.1252	29.0000	
**FN1016**	-1.045	6.515	1.04e-1	2.341e-1	6	4	6.4392	5.4806	AAL95212.1| Lipid A biosynthesis lauroyl acyltransferase
					5	22	6.8751	22.0000	
**FN1017**	0.421	12.947	1.035e-1	2.322e-1	93	34	99.8072	46.5848	AAL95213.1| Hypothetical Exported Protein
					77	107	105.8766	107.0000	
**FN1019**	-0.359	24.345	8.252e-3	5.639e-3	3484	3572	3739.0140	4894.1395	AAL95215.1| 3-hydroxybutyryl-CoA dehydrogenase
					3209	5563	4412.4415	5563.0000	

Show detected proteins only
Show all proteins
Filter by category:
GO: amino acid transport

Proteins found: 1305

Enter (or paste) list of ORFs

Find ORFs

Test	Cutoff
q-Value	.005
p-Value	

	Signif	Direction	Applies To
red	yes	+	ratios, bars
yellow	no	n/a	bars
green	yes	−	ratios, bars
pink	yes	+	*p*- , *q*-Values
light green	yes	−	

Dot Plots Dot Plots

Protein	*FnPgSg vs FnPg* Log$_2$ Ratio	Log$_2$ Sum	*q*-Value	*p*-Value	Raw *FnPgSg*	Raw *FnPg*	Normalized *FnPgSg*	Normalized *FnPg*	Description
**FN1020**	-0.548	17.993	1.455e-2	1.343e-2	331	460	355.2278	630.2643	AAL95216.1| 3-hydroxybutyryl-CoA dehydratase
					356	605	489.5074	605.0000	
**FN1022**					9		9.6588		AAL95218.1| Calcium-transporting ATPase
					12		16.5002		
**FN1023**	-1.178	6.997			7		7.5124		AAL95219.1| 5-Nitroimidazole antibiotic resistance protein
						17		17.0000	
**FN1024**	0.096	20.332	2.291e-1	7.125e-1	1379	934	1479.9369	1279.7106	AAL95220.1| DNA-binding protein HU
					651	943	895.1385	943.0000	
**FN1025**	-0.143	8.197			15		16.0979		AAL95221.1| Guanine-hypoxanthine permease
					12	18	16.5002	18.0000	
**FN1028**	0.415	10.018	9.453e-4	2.2e-4	36	21	38.6350	28.7729	AAL95224.1| Deoxyuridine 5'-triphosphate nucleotidohydrolase
					26	27	35.7505	27.0000	
**FN1029**	-0.211	12.329	9.931e-2	2.169e-1	73	55	78.3433	75.3577	AAL95225.1| Zinc protease
					40	79	55.0008	79.0000	
**FN1030**	-1.483	7.687			8		8.5856		AAL95226.1| Hypothetical membrane-spanning protein
						24		24.0000	
**FN1031**	-1.541	5.629							AAL95227.1| Hypothetical membrane-spanning protein
					3	12	4.1251	12.0000	
**FN1033**	-1.263	10.623	3.927e-5	1.921e-6	26	46	27.9031	63.0264	AAL95229.1| Methyltransferase
					17	60	23.3754	60.0000	
**FN1034**					4		4.2928		AAL95230.1| Transcriptional regulator, TetR family
**FN1037**	-2.220	5.594			3		3.2196		AAL95233.1| Hypothetical cytosolic protein
						15		15.0000	
**FN1041**	-0.327	6.701	1.623e-1	4.455e-1	8	5	8.5856	6.8507	AAL95237.1| Acetyltransferase
					7	16	9.6251	16.0000	
**FN1042**	0.555	8.572	1.808e-2	1.847e-2	21	14	22.5371	19.1820	AAL95238.1| S1 RNA binding domain
					18	13	24.7504	13.0000	
**FN1048**					3		3.2196		AAL95244.1| Hypothetical membrane-spanning protein
**FN1050**						22		30.1431	AAL95246.1| Lactoylglutathione lyase
						3		3.0000	

Log$_2$ Ratios: -6 -4 -2 0 2 4 6

Show detected proteins only
Show all proteins
Filter by category:
GO: amino acid transport

Proteins found: 1305

Enter (or paste) list of ORFs

Find ORFs

Test	Cutoff
q-Value	.005
p-Value	

	Signif	Direction	Applies To
red	yes	+	ratios, bars
yellow	no	n/a	bars
green	yes	−	ratios, bars
pink	yes	+	*p*- , *q*-Values
light green	yes	−	

Dot Plots Dot Plots

Protein	FnPgSg vs FnPg Log₂ Ratio	Log₂ Sum	*q*-Value	*p*-Value	Raw *FnPgSg*	Raw *FnPg*	Normalized *FnPgSg*	Normalized *FnPg*	Description
**FN1053**						8		10.9611	AAL95249.1| Hypothetical protein
**FN1055**	-0.631	11.357	8.252e-2	1.631e-1	37	31	39.7082	42.4743	AAL95251.1| Cysteine synthase
					31	85	42.6256	85.0000	
**FN1060**	-0.300	9.661	1.346e-2	1.186e-2	26	22	27.9031	30.1431	AAL95256.1| hypothetical cytosolic protein
					17	33	23.3754	33.0000	
**FN1062**	0.964	13.036	6.708e-3	3.924e-3	113	60	121.2711	82.2084	AAL95258.1| Hydrolase
					98	49	134.7520	49.0000	
**FN1063**	-2.594	6.798			4	5	4.2928	6.8507	AAL95259.1| N-acyl-L-amino acid amidohydrolase
						45		45.0000	
**FN1066**	0.035	6.290	2.769e-1	9.179e-1	9	4	9.6588	5.4806	AAL95262.1| Exodeoxyribonuclease VII large subunit
					6	12	8.2501	12.0000	
**FN1067**	0.386	9.812	1.109e-1	2.592e-1	28	12	30.0495	16.4417	AAL95263.1| Tetratricopeptide repeat family protein
					28	36	38.5006	36.0000	
**FN1068**						3		4.1104	AAL95264.1| Smf protein
						16		16.0000	
**FN1069**	-0.299	11.491	1.064e-2	8.34e-3	44	46	47.2206	63.0264	AAL95265.1| DNA topoisomerase I
					36	56	49.5007	56.0000	
**FN1070**	0.653	6.993			11		11.8052		AAL95266.1| Glucose inhibited division protein A
					12	9	16.5002	9.0000	
**FN1071**					6		6.4392		AAL95267.1| Integrase/recombinase
**FN1072**	-0.490	10.065	5.198e-2	8.445e-2	22	34	23.6103	46.5848	AAL95268.1| GTP-binding protein
					23	31	31.6255	31.0000	
**FN1073**	-0.541	4.629							AAL95269.1| Hypothetical protein
					3	6	4.1251	6.0000	
**FN1074**	-0.246	8.852	1.45e-1	3.834e-1	15	13	16.0979	17.8118	AAL95270.1| Signal recognition particle receptor FtsY
					17	29	23.3754	29.0000	
**FN1077**	0.353	10.611			41		44.0010		AAL95273.1| Hypothetical protein
					33	35	45.3757	35.0000	
**FN1078**	-0.476	15.254	3.081e-2	3.954e-2	133	195	142.7350	267.1773	AAL95274.1| Hypothetical exported 24-amino acid repeat protein
					140	199	192.5029	199.0000	

Log₂ Ratios: -6 -4 -2 0 2 4 6

Show detected proteins only
Show all proteins
Filter by category:
GO: amino acid transport

Proteins found: 1305

Enter (or paste) list of ORFs

Find ORFs

Test	Cutoff
q-Value	.005
p-Value	

	Signif	Direction	Applies To
red	yes	+	ratios, bars
yellow	no	n/a	bars
green	yes	−	ratios, bars
pink	yes	+	*p*-, *q*-Values
light green	yes	−	

Dot Plots | Dot Plots

Protein	FnPgSg vs FnPg Log$_2$ Ratio	Log$_2$ Sum	*q*-Value	*p*-Value	Raw *FnPgSg*	Raw *FnPg*	Normalized *FnPgSg*	Normalized *FnPg*	Description
FN1079	0.039	18.565	2.765e-1	9.161e-1	638	267	684.6989	365.8273	AAL95275.1| Neutrophil-activating protein A
					420	863	577.5087	863.0000	
FN1080	0.748	4.408	3.152e-3	1.322e-3	6	3	6.4392	4.1104	AAL95276.1| Export ABC transporter
					4	3	5.5001	3.0000	
FN1081	-0.778	9.418			18	25	19.3175	34.2535	AAL95277.1| unknown
					15		20.6253		
FN1082					4		4.2928		AAL95278.1| unknown
FN1084	1.622	11.687	7.835e-3	5.122e-3	75	31	80.4897	42.4743	AAL95280.1| unknown
					88	23	121.0018	23.0000	
FN1085	-1.217	12.707	1.667e-3	5.287e-4	41	101	44.0010	138.3841	AAL95281.1| 4-methyl-5(B-hydroxyethyl)-thiazole monophosphate biosynthesis enzyme
					46	111	63.2510	111.0000	
FN1086	0.733	6.348			14		15.0247		AAL95282.1| Transporter
					6	7	8.2501	7.0000	
FN1088	0.703	9.585	1.4e-2	1.262e-2	30	12	32.1959	16.4417	AAL95284.1| NADH oxidase
					28	27	38.5006	27.0000	
FN1089	-2.153	12.090	2.185e-2	2.41e-2	34	69	36.4887	94.5396	AAL95285.1| ATP-binding protein (contains P-loop)
					19	184	26.1254	184.0000	
FN1091	1.040	6.623	9.028e-3	6.443e-3	15	5	16.0979	6.8507	AAL95287.1| Sigma factor sigB regulation protein rsbU
					9	7	12.3752	7.0000	
FN1092	-0.693	10.392	1.753e-1	4.947e-1	14	6	15.0247	8.2208	AAL95288.1| Hypothetical protein
					31	85	42.6256	85.0000	
FN1093	0.845	11.009	6.844e-3	4.056e-3	57	29	61.1722	39.7341	AAL95289.1| Hypothetical protein
					44	28	60.5009	28.0000	
FN1094									AAL95290.1| Dolichol-phosphate mannosyltransferase
						33		33.0000	
FN1095						8		10.9611	AAL95291.1| unknown
FN1096	-0.076	10.723	2.707e-1	8.896e-1	49	47	52.5866	64.3966	AAL95292.1| Hypothetical protein
					20	20	27.5004	20.0000	
FN1097	-1.149	9.161			12	26	12.8783	35.6236	AAL95293.1| Hypothetical protein
					14		19.2503		

Log$_2$ Ratios: -6 -4 -2 0 2 4 6

Show detected proteins only
Show all proteins
Filter by category:
GO: amino acid transport

Proteins found: 1305

Enter (or paste) list of ORFs

Find ORFs

Test	Cutoff
q-Value	.005
p-Value	

	Signif	Direction	Applies To
red	yes	+	ratios, bars
yellow	no	n/a	bars
green	yes	−	ratios, bars
pink	yes	+	*p*-, *q*-Values
light green	yes	−	*p*-, *q*-Values

Dot Plots Dot Plots

Protein	*FnPgSg vs FnPg* Log_2 Ratio	Log_2 Sum	*q*-Value	*p*-Value	Raw *FnPgSg*	Raw *FnPg*	Normalized *FnPgSg*	Normalized *FnPg*	Description
**FN1101**					3		3.2196		AAL95297.1| ATPase
**FN1103**	-0.444	9.579	6.127e-2	1.078e-1	16	23	17.1711	31.5132	AAL95299.1| Excinuclease ABC subunit A
					22	33	30.2505	33.0000	
**FN1104**					5		5.3660		AAL95300.1| Holliday junction DNA helicase ruvA
**FN1105**	-0.054	12.708	2.666e-1	8.714e-1	74	83	79.4165	113.7216	AAL95301.1| Hypothetical protein
					59	53	81.1262	53.0000	
**FN1106**	-0.563	11.411	1.249e-2	1.057e-2	39	51	41.8546	69.8771	AAL95302.1| L-serine dehydratase
					32	57	44.0007	57.0000	
**FN1111**					9		9.6588		AAL95307.1| Dipeptide-binding protein
					7		9.6251		
**FN1117**	1.286	11.603	1.516e-2	1.439e-2	79	39	84.7825	53.4355	AAL95313.1| LSU ribosomal protein L21P
					65	18	89.3763	18.0000	
**FN1119**	1.378	14.322	9.072e-3	6.491e-3	230	96	246.8350	131.5334	AAL95315.1| LSU ribosomal protein L27P
					156	46	214.5032	46.0000	
**FN1120**	-0.953	15.144	1.162e-2	9.5e-3	128	166	137.3691	227.4432	AAL95316.1| Phosphoenolpyruvate carboxykinase (ATP)
					99	302	136.1271	302.0000	
**FN1121**	0.527	12.258	2.112e-2	2.294e-2	72	34	77.2701	46.5848	AAL95317.1| hypothetical cytosolic protein
					66	70	90.7514	70.0000	
**FN1122**	1.326	13.071	2.949e-4	4.086e-5	143	41	153.4670	56.1757	AAL95318.1| Long-chain-fatty-acid--CoA ligase
					102	61	140.2521	61.0000	
**FN1123**	2.377	7.745	2.844e-2	3.518e-2	43	5	46.1474	6.8507	AAL95319.1| Thioredoxin-like protein
					15	6	20.6253	6.0000	
**FN1124**	0.779	16.209	1.569e-3	4.833e-4	312	133	334.8371	182.2286	AAL95320.1| Outer membrane porin F
					281	238	386.3808	238.0000	
**FN1125**	-0.372	11.858	1.974e-1	5.849e-1	46	18	49.3670	24.6625	AAL95321.1| LemA protein
					42	114	57.7509	114.0000	
**FN1127**	-1.102	11.845	3.37e-2	4.523e-2	40	83	42.9278	113.7216	AAL95323.1| Hypothetical membrane-spanning protein
					29	64	39.8756	64.0000	
**FN1128**	-0.752	15.657	4.541e-2	6.888e-2	156	270	167.4185	369.9378	AAL95324.1| Acylamino-acid-releasing enzyme
					133	220	182.8778	220.0000	

Log_2 Ratios: -6 -4 -2 0 2 4 6

- Show detected proteins only
- Show all proteins
- Filter by category: GO: amino acid transport

Proteins found: 1305

Enter (or paste) list of ORFs

Find ORFs

Test	Cutoff
q-Value	.005
p-Value	

	Signif	Direction	Applies To
red	yes	+	ratios, bars
yellow	no	n/a	bars
green	yes	−	ratios, bars
pink	yes	+	*p*-, *q*-Values
light green	yes	−	*p*-, *q*-Values

Dot Plots Dot Plots

Protein	FnPgSg vs FnPg Log$_2$ Ratio	Log$_2$ Sum	*q*-Value	*p*-Value	Raw *FnPgSg*	Raw *FnPg*	Normalized *FnPgSg*	Normalized *FnPg*	Description
FN1129	0.553	5.723			10		10.7320		AAL95325.1| Chromosome partition protein smc
					5	6	6.8751	6.0000	
FN1130	0.780	5.424			8		8.5856		AAL95326.1| Tetraacyldisaccharide 4'-kinase
						5		5.0000	
FN1131	-2.014	5.387			3		3.2196		AAL95327.1| Hypothetical protein
						13		13.0000	
FN1133	-0.425	10.296	1.69e-1	4.706e-1	25	14	26.8299	19.1820	AAL95329.1| N-acetylglucosamine-6-phosphate deacetylase
					25	63	34.3755	63.0000	
FN1134	-0.212	9.331	1.827e-1	5.238e-1	26	26	27.9031	35.6236	AAL95330.1| Hypothetical cytosolic protein
					14	19	19.2503	19.0000	
FN1135	-0.578	14.741	1.373e-1	3.561e-1	123	225	132.0031	308.2815	AAL95331.1| Phosphonates-binding protein
					101	96	138.8771	96.0000	
FN1136	-0.989	8.181			11		11.8052		AAL95332.1| Phosphonates transport ATP-binding protein phnC
					9	24	12.3752	24.0000	
FN1137									AAL95333.1| Phosphonates transport system permease protein phnE
					3		4.1251		
FN1138	0.684	19.288	3.659e-2	5.085e-2	1147	349	1230.9555	478.1788	AAL95334.1| Hypothetical cytosolic protein
					580	784	797.5120	784.0000	
FN1139	-3.066	9.513	5.823e-2	1.003e-1	11	20	11.8052	27.4028	AAL95335.1| Activator of (R)-2-hydroxyglutaryl-CoA dehydratase
					5	129	6.8751	129.0000	
FN1140									AAL95336.1| hypothetical protein
						25		25.0000	
FN1142	-0.635	4.009			3		3.2196		AAL95338.1| Oxygen-independent coproporphyrinogen III oxidase
						5		5.0000	
FN1143	-0.289	13.429	1.993e-1	5.93e-1	104	41	111.6124	56.1757	AAL95339.1| Glucosamine-6-phosphate isomerase
					57	176	78.3762	176.0000	
FN1144	1.772	13.589	4.621e-3	2.264e-3	199	22	213.5660	30.1431	AAL95340.1| Hypothetical Exported Protein
					143	90	196.6280	90.0000	
FN1145	0.131	8.843	2.379e-1	7.483e-1	20	8	21.4639	10.9611	AAL95341.1| Oligoendopeptidase F
					17	30	23.3754	30.0000	
FN1146	1.432	7.511			17	6	18.2443	8.2208	AAL95342.1| Hypothetical exported 24-amino acid repeat protein
					19		26.1254		

Show detected proteins only
Show all proteins
Filter by category:
GO: amino acid transport

Proteins found: 1305

Enter (or paste) list of ORFs

Find ORFs

Test	Cutoff
q-Value	.005
p-Value	

	Signif	Direction	Applies To
red	yes	+	ratios, bars
yellow	no	n/a	bars
green	yes	−	ratios, bars
pink	yes	+	*p*- , *q*-Values
light green	yes	−	

Dot Plots Dot Plots

Protein	*FnPgSg vs FnPg* Log$_2$ Ratio	Log$_2$ Sum	*q*-Value	*p*-Value	Raw *FnPgSg*	Raw *FnPg*	Normalized *FnPgSg*	Normalized *FnPg*	Description
**FN1147**					33		35.4155		AAL95343.1| Hypothetical protein
					43		59.1259		
**FN1148**	-0.208	11.035	2.527e-1	8.105e-1	41	4	44.0010	5.4806	AAL95344.1| Serine/threonine sodium symporter
					30	93	41.2506	93.0000	
**FN1149**	-0.202	6.072	2.01e-1	6.004e-1	4	7	4.2928	9.5910	AAL95345.1| ATP-dependent nuclease subunit A
					8	8	11.0002	8.0000	
**FN1150**	0.459	4.459							AAL95346.1| unknown
					4	4	5.5001	4.0000	
**FN1152**	0.567	13.821	6.229e-2	1.102e-1	146	45	156.6866	61.6563	AAL95348.1| Aspartate aminotransferase
					99	136	136.1271	136.0000	
**FN1153**					37		39.7082		AAL95349.1| Hypothetical protein
					29		39.8756		
**FN1154**									AAL95350.1| Ribonuclease BN
						20		20.0000	
**FN1157**					12		12.8783		AAL95353.1| Polypeptide deformylase
					16		22.0003		
**FN1159**	-0.542	13.574	4.806e-2	7.492e-2	77	77	82.6361	105.5008	AAL95355.1| Fructose-1,6-bisphosphatase
					73	161	100.3765	161.0000	
**FN1160**	1.684	4.854			9		9.6588		AAL95356.1| SWF/SNF family helicase
					7	3	9.6251	3.0000	
**FN1161**	0.874	4.044							AAL95357.1| Glutamate racemase
					4	3	5.5001	3.0000	
**FN1162**									AAL95358.1| Hydroxyacylglutathione hydrolase
						21		21.0000	
**FN1163**	0.413	12.393	9.506e-2	2.023e-1	77	30	82.6361	41.1042	AAL95359.1| Thioredoxin reductase
					63	86	86.6263	86.0000	
**FN1164**	-0.511	6.073				7		9.5910	AAL95360.1| Glucokinase
					5	10	6.8751	10.0000	
**FN1165**	1.181	24.201	5.659e-5	3.271e-6	6372	2224	6838.4033	3047.1910	AAL95361.1| D-galactose-binding protein
					4643	2786	6384.2212	2786.0000	
**FN1166**	-0.428	14.385	5.173e-4	9.314e-5	112	128	120.1979	175.3779	AAL95362.1| Galactoside transport ATP-binding protein mglA
					96	164	132.0020	164.0000	

Show detected proteins only
Show all proteins
Filter by category:
GO: amino acid transport

Proteins found: 1305

Enter (or paste) list of ORFs

Find ORFs

Test	Cutoff
q-Value	.005
p-Value	

	Signif	Direction	Applies To
red	yes	+	ratios, bars
yellow	no	n/a	bars
green	yes	−	ratios, bars
pink	yes	+	*p*-, *q*-Values
light green	yes	−	*p*-, *q*-Values

Dot Plots Dot Plots

Protein	FnPgSg vs FnPg Log$_2$ Ratio	Log$_2$ Sum	*q*-Value	*p*-Value	Raw *FnPgSg*	Raw *FnPg*	Normalized *FnPgSg*	Normalized *FnPg*	Description
FN1167					26		27.9031		AAL95363.1| Galactoside transport system permease protein mglC
					14		19.2503		
FN1169	-0.285	16.174	9.312e-2	1.958e-1	217	260	232.8835	356.2364	AAL95365.1| L-lactate dehydrogenase
					189	244	259.8789	244.0000	
FN1170	-0.807	24.928	4.348e-2	6.47e-2	3813	6878	4092.0954	9423.8219	AAL95366.1| Pyruvate-flavodoxin oxidoreductase
					3237	5525	4450.9421	5525.0000	
FN1171	-0.388	19.701	7.863e-2	1.521e-1	767	936	823.1411	1282.4509	AAL95367.1| Acetate kinase
					575	830	790.6369	830.0000	
FN1172	0.499	18.447	2.845e-3	1.133e-3	704	348	755.5298	476.8087	AAL95368.1| Phosphate acetyltransferase
					484	529	665.5100	529.0000	
FN1176						4		5.4806	AAL95372.1| Hypothetical cytosolic protein
FN1179						14		19.1820	AAL95375.1| ATP-dependent RNA helicase
FN1180						10		13.7014	AAL95376.1| Hypothetical protein
FN1181	-1.956	14.495	1.022e-1	2.275e-1	81	404	86.9289	553.5365	AAL95377.1| unknown
					49	45	67.3760	45.0000	
FN1182						10		13.7014	AAL95378.1| Hypothetical protein
FN1183	-1.422	6.503	1.305e-2	1.13e-2	7	14	7.5124	19.1820	AAL95379.1| Hypothetical cytosolic protein
					3	12	4.1251	12.0000	
FN1184						4		5.4806	AAL95380.1| Hypothetical protein
FN1185	-0.600	10.578	1.248e-1	3.079e-1	31	52	33.2691	71.2473	AAL95381.1| SIR2 family protein
					22	25	30.2505	25.0000	
FN1186	-3.651	13.097	5.602e-2	9.491e-2	30	82	32.1959	112.3515	AAL95382.1| Amidohydrolase
					15	551	20.6253	551.0000	
FN1187	-0.476	10.886	1.992e-1	5.927e-1	38	7	40.7814	9.5910	AAL95383.1| Amino acid-binding protein
					24	93	33.0005	93.0000	
FN1188	0.162	11.071	2.475e-1	7.886e-1	53	10	56.8794	13.7014	AAL95384.1| Hypothetical protein
					30	74	41.2506	74.0000	

Log$_2$ Ratios: -6 -4 -2 0 2 4 6

Show detected proteins only
Show all proteins
Filter by category:
GO: amino acid transport

Proteins found: 1305

Enter (or paste) list of ORFs

Find ORFs

Test	Cutoff
q-Value	.005
p-Value	

Color	Signif	Direction	Applies To
red	yes	+	ratios, bars
yellow	no	n/a	bars
green	yes	−	ratios, bars
pink	yes	+	*p*- , *q*-Values
light green	yes	−	

Dot Plots Dot Plots

Protein	FnPgSg vs FnPg Log_2 Ratio	Log_2 Sum	*q*-Value	*p*-Value	Raw *FnPgSg*	Raw *FnPg*	Normalized *FnPgSg*	Normalized *FnPg*	Description
FN1189	-0.362	11.639	4.426e-4	7.239e-5	48	46	51.5134	63.0264	AAL95385.1| Hypothetical protein
					35	65	48.1257	65.0000	
FN1190	0.164	12.753	4.181e-2	6.12e-2	87	54	93.3680	73.9876	AAL95386.1| Probable cadmium-transporting ATPase
					60	83	82.5012	83.0000	
FN1191	-1.354	9.630			20		21.4639		AAL95387.1| unknown
					10	45	13.7502	45.0000	
FN1192	0.813	17.884	2.569e-3	9.785e-4	642	227	688.9917	311.0217	AAL95388.1| unknown
					447	431	614.6343	431.0000	
FN1198	-0.414	9.587	5.934e-2	1.031e-1	23	19	24.6835	26.0327	AAL95394.1| Transporter
					17	38	23.3754	38.0000	
FN1200	-5.133	12.533			5		5.3660		AAL95396.1| Hypothetical protein
					15	456	20.6253	456.0000	
FN1202	-0.858	8.687	6.926e-3	4.136e-3	14	18	15.0247	24.6625	AAL95398.1| NH(3)-dependent NAD(+) synthetase
					11	30	15.1252	30.0000	
FN1203						8		10.9611	AAL95399.1| GTP-binding protein
						5		5.0000	
FN1204						10		13.7014	AAL95400.1| Methyltransferase
						13		13.0000	
FN1205	0.300	11.847	1.744e-1	4.912e-1	55	20	59.0258	27.4028	AAL95401.1| Protease
					55	82	75.6261	82.0000	
FN1208	-0.596	5.444			5	6	5.3660	8.2208	AAL95404.1| 1-deoxyxylulose-5-phosphate synthase
						8		8.0000	
FN1209	1.880	9.694			67		71.9041		AAL95405.1| Hypothetical RNA binding protein
					28	15	38.5006	15.0000	
FN1210	0.253	10.168	1.046e-1	2.361e-1	28	22	30.0495	30.1431	AAL95406.1| Metal dependent hydrolase
					32	32	44.0007	32.0000	
FN1211	1.275	6.642	4.827e-2	7.542e-2	20	5	21.4639	6.8507	AAL95407.1| Cell division protein ftsI
					7	6	9.6251	6.0000	
FN1213	0.479	10.515	1.034e-1	2.319e-1	47	13	50.4402	17.8118	AAL95409.1| Hypothetical protein
					29	47	39.8756	47.0000	
FN1214					6		6.4392		AAL95410.1| Fe-S oxidoreductase
					5		6.8751		

Log_2 Ratios: -6 -4 -2 0 2 4 6

Show detected proteins only
Show all proteins
Filter by category:
GO: amino acid transport

Proteins found: 1305

Enter (or paste) list of ORFs

Find ORFs

Test	Cutoff
q-Value	.005
p-Value	

	Signif	Direction	Applies To
red	yes	+	ratios, bars
yellow	no	n/a	bars
green	yes	–	ratios, bars
pink	yes	+	*p*- , *q*-Values
light green	yes	–	*p*- , *q*-Values

Dot Plots Dot Plots

Protein	*FnPgSg vs FnPg* Log$_2$ Ratio	Log$_2$ Sum	*q*-Value	*p*-Value	Raw *FnPgSg*	Raw *FnPg*	Normalized *FnPgSg*	Normalized *FnPg*	Description
**FN1216**	-0.675	6.335	8.412e-2	1.678e-1	3	10	3.2196	13.7014	AAL95412.1| RRF2 family protein
					8	9	11.0002	9.0000	
**FN1217**	-0.561	5.054			5		5.3660		AAL95413.1| Holliday junction DNA helicase ruvB
					3	7	4.1251	7.0000	
**FN1218**									AAL95414.1| unknown
						13		13.0000	
**FN1219**	0.072	8.375	2.487e-1	7.936e-1	22	15	23.6103	20.5521	AAL95415.1| Hypothetical protein
					10	15	13.7502	15.0000	
**FN1220**	0.173	15.321	5.507e-2	9.256e-2	212	127	227.5175	174.0078	AAL95416.1| Cysteine synthase
					147	207	202.1280	207.0000	
**FN1221**	-0.040	8.520	2.832e-1	9.47e-1	16	5	17.1711	6.8507	AAL95417.1| Hypothetical protein
					15	32	20.6253	32.0000	
**FN1222**	-0.009	7.575	2.889e-1	9.741e-1	9	10	9.6588	13.7014	AAL95418.1| Hypothetical protein
					13	14	17.8753	14.0000	
**FN1223**	0.124	12.932	1.753e-1	4.947e-1	90	74	96.5876	101.3903	AAL95419.1| Oxygen-insensitive NAD(P)H nitroreductase
					64	68	88.0013	68.0000	
**FN1224**	-0.022	13.201	2.847e-1	9.542e-1	100	42	107.3196	57.5459	AAL95420.1| 2-dehydro-3-deoxyphosphooctonate aldolase
					62	138	85.2513	138.0000	
**FN1225**	0.586	13.839	5.862e-2	1.013e-1	147	45	157.7598	61.6563	AAL95421.1| UDP-N-acetylmuramoyl-L-alanyl-D-glutamate--meso-lanthionine ligase
					101	136	138.8771	136.0000	
**FN1226**	0.351	12.669	1.612e-1	4.415e-1	84	24	90.1484	32.8834	AAL95422.1| Uracil-DNA glycosylase
					67	110	92.1264	110.0000	
**FN1230**					62		66.5381		AAL95426.1| Hypothetical cytosolic protein
					37		50.8758		
**FN1231**	-0.113	18.873	2.642e-1	8.607e-1	592	166	635.3319	227.4432	AAL95427.1| Inosine-5'-monophosphate dehydrogenase
					507	1214	697.1355	1214.0000	
**FN1234**					8		8.5856		AAL95430.1| Hypothetical protein
					5		6.8751		
**FN1235**	-0.850	8.132	1.028e-1	2.297e-1	13	8	13.9515	10.9611	AAL95431.1| Ankyrin repeat proteins
					8	34	11.0002	34.0000	
**FN1237**	-1.431	11.101	2.257e-3	8.196e-4	25	51	26.8299	69.8771	AAL95433.1| Choline kinase
					22	84	30.2505	84.0000	

Show detected proteins only
Show all proteins
Filter by category:
GO: amino acid transport

Proteins found: 1305

Enter (or paste) list of ORFs

Find ORFs

Test	Cutoff
***q*-Value**	.005
p-Value	

	Signif	Direction	Applies To
[Red]	yes	+	ratios, bars
[Yellow]	no	n/a	bars
[Green]	yes	–	ratios, bars
[Pink]	yes	+	*p*- , *q*-Values
[Light green]	yes	–	*p*- , *q*-Values

Dot Plots Dot Plots

Protein	FnPgSg vs FnPg Log2 Ratio	Log2 Sum	q-Value	p-Value	Raw FnPgSg	Raw FnPg	Normalized FnPgSg	Normalized FnPg	Description
FN1238									AAL95434.1| Hypothetical protein
						5		5.0000	
FN1239									AAL95435.1| Hypothetical protein
						12		12.0000	
FN1240	-0.254	6.675	1.82e-1	5.212e-1	7	11	7.5124	15.0715	AAL95436.1| Lipopolysaccharide core biosynthesis protein rfaY
					8	7	11.0002	7.0000	
FN1241						7		9.5910	AAL95437.1| polysaccharide biosynthesis protein
						4		4.0000	
FN1242	-1.592	6.748			6		6.4392		AAL95438.1| Polysaccharide deacetylase
					4	18	5.5001	18.0000	
FN1243	-0.961	6.654			7		7.5124		AAL95439.1| Glycosyl transferase
					5	14	6.8751	14.0000	
FN1244	-1.841	7.068	8.382e-2	1.669e-1	5	5	5.3660	6.8507	AAL95440.1| Polysaccharide deacetylase
					5	37	6.8751	37.0000	
FN1245						3		4.1104	AAL95441.1| Glycosyl transferase
						5		5.0000	
FN1246	-1.862	5.951							AAL95442.1| Lipooligosaccharide cholinephosphotransferase
					3	15	4.1251	15.0000	
FN1247	-1.049	7.036	4.859e-2	7.617e-2	11	8	11.8052	10.9611	AAL95443.1| LOS biosynthesis enzyme LBGB
					3	22	4.1251	22.0000	
FN1250	0.348	8.897	1.072e-1	2.464e-1	19	10	20.3907	13.7014	AAL95446.1| Guanine-hypoxanthine permease
					21	25	28.8754	25.0000	
FN1251	0.403	6.018			7		7.5124		AAL95447.1| High-affinity iron permease
					8	7	11.0002	7.0000	
FN1252	1.571	17.087	3.862e-3	1.786e-3	667	151	715.8216	206.8911	AAL95448.1| 34 kDa membrane antigen precursor
					415	226	570.6336	226.0000	
FN1253	0.554	15.782	1.26e-1	3.123e-1	240	48	257.5670	65.7667	AAL95449.1| unknown
					231	326	317.6298	326.0000	
FN1254	0.837	12.928	2.476e-2	2.872e-2	120	30	128.7835	41.1042	AAL95450.1| Oxygen-insensitive NAD(P)H nitroreductase
					78	91	107.2516	91.0000	
FN1256	0.044	4.044							AAL95452.1| C4-dicarboxylate transporter large subunit
					3	4	4.1251	4.0000	

Log2 Ratios: -6 -4 -2 0 2 4 6

Show detected proteins only
Show all proteins
Filter by category:
GO: amino acid transport

Proteins found: 1305

Enter (or paste) list of ORFs

Find ORFs

Test	Cutoff
q-Value	.005
p-Value	

	Signif	Direction	Applies To
red	yes	+	ratios, bars
yellow	no	n/a	bars
green	yes	−	ratios, bars
pink	yes	+	p- , q-Values
light green	yes	−	

Dot Plots Dot Plots

Protein	FnPgSg vs FnPg Log$_2$ Ratio	Log$_2$ Sum	q-Value	p-Value	Raw FnPgSg	Raw FnPg	Normalized FnPgSg	Normalized FnPg	Description
FN1258	0.524	19.520	1.03e-2	7.915e-3	987	595	1059.2442	815.2332	AAL95454.1| C4-dicarboxylate-binding protein
					742	631	1020.2654	631.0000	
FN1260					3		3.2196		AAL95456.1| Sensory Transduction Protein Kinase
FN1261						10		13.7014	AAL95457.1| Two-component response regulator
FN1262									AAL95458.1| Integral membrane protein
						20		20.0000	
FN1263	0.379	7.675	5.1e-2	8.2e-2	15	11	16.0979	15.0715	AAL95459.1| Cobalt chelatase
					12	10	16.5002	10.0000	
FN1264					11		11.8052		AAL95460.1| Hypothetical protein
					8		11.0002		
FN1265	-0.535	12.631	2.142e-2	2.341e-2	49	64	52.5866	87.6890	AAL95461.1| Outer membrane protein
					58	104	79.7512	104.0000	
FN1266	0.191	13.555	1.125e-1	2.645e-1	98	63	105.1732	86.3188	AAL95462.1| UTP--glucose-1-phosphate uridylyltransferase
					94	119	129.2519	119.0000	
FN1267	-0.450	10.393	5.226e-2	8.518e-2	29	37	31.1227	50.6952	AAL95463.1| Hypothetical protein
					23	35	31.6255	35.0000	
FN1268	-0.103	15.280	3.814e-2	5.386e-2	187	155	200.6876	212.3717	AAL95464.1| Methionyl-tRNA synthetase
					134	201	184.2528	201.0000	
FN1269	0.044	4.044							AAL95465.1| Hypothetical lipoprotein
					3	4	4.1251	4.0000	
FN1270	3.182	11.966			177		189.9556		AAL95466.1| Hypothetical cytosolic protein
					139	21	191.1279	21.0000	
FN1271	0.679	10.509	8.683e-2	1.759e-1	49	9	52.5866	12.3313	AAL95467.1| Protease IV
					32	48	44.0007	48.0000	
FN1273	-1.537	9.617	1.198e-1	2.898e-1	14	4	15.0247	5.4806	AAL95469.1| Outer membrane protein tolC
					13	90	17.8753	90.0000	
FN1274	0.433	7.366	1.519e-1	4.081e-1	15	3	16.0979	4.1104	AAL95470.1| Acriflavin resistance protein E
					10	18	13.7502	18.0000	
FN1275	0.666	8.536	4.553e-2	6.916e-2	26	7	27.9031	9.5910	AAL95471.1| Acriflavin resistance protein B
					15	21	20.6253	21.0000	

Log$_2$ Ratios: -6 -4 -2 0 2 4 6

Show detected proteins only
Show all proteins
Filter by category:
GO: amino acid transport

Proteins found: 1305

Enter (or paste) list of ORFs

Find ORFs

Test	Cutoff
q-Value	.005
p-Value	

Signif	Direction	Applies To
yes	+	ratios, bars
no	n/a	bars
yes	−	ratios, bars
yes	+	p- , q-Values
yes	−	

Dot Plots Dot Plots

Protein	FnPgSg vs FnPg Log$_2$ Ratio	Log$_2$ Sum	*q*-Value	*p*-Value	Raw *FnPgSg*	Raw *FnPg*	Normalized *FnPgSg*	Normalized *FnPg*	Description
**FN1276**	-1.157	7.168	3.697e-3	1.684e-3	6	13	6.4392	17.8118	AAL95472.1| Hypothetical protein
					7	18	9.6251	18.0000	
**FN1277**	-0.298	15.359	1.58e-1	4.298e-1	169	107	181.3701	146.6050	AAL95473.1| Aminoacyl-histidine dipeptidase
					137	308	188.3778	308.0000	
**FN1278**	2.044	5.214							AAL95474.1| Acetyltransferase
					9	3	12.3752	3.0000	
**FN1279**	-0.194	11.489	8.004e-2	1.56e-1	46	37	49.3670	50.6952	AAL95475.1| Zinc metallohydrolase, glyoxalase II family
					37	64	50.8758	64.0000	
**FN1280**	-1.994	9.717	5.39e-4	9.995e-5	13	40	13.9515	54.8056	AAL95476.1| Serine protease, V8 family
					11	61	15.1252	61.0000	
**FN1281**						12		16.4417	AAL95477.1| Cysteine protease
						18		18.0000	
**FN1282**	0.233	13.079	1.369e-1	3.545e-1	97	80	104.1000	109.6112	AAL95478.1| LSU ribosomal protein L17P
					71	62	97.6265	62.0000	
**FN1283**	1.265	18.687	8.716e-5	6.423e-6	947	287	1016.3164	393.2301	AAL95479.1| DNA-directed RNA polymerase alpha chain
					726	445	998.2650	445.0000	
**FN1284**	0.448	18.380	1.071e-1	2.46e-1	628	525	673.9669	719.3234	AAL95480.1| SSU ribosomal protein S4P
					502	281	690.2604	281.0000	
**FN1285**	-1.064	15.052	1.489e-1	3.973e-1	112	373	120.1979	511.0622	AAL95481.1| SSU ribosomal protein S11P
					98	22	134.7520	22.0000	
**FN1286**	1.043	12.504	1.179e-2	9.704e-3	118	41	126.6371	56.1757	AAL95482.1| SSU ribosomal protein S13P
					67	50	92.1264	50.0000	
**FN1287**	-1.375	7.470	7.396e-2	1.394e-1	9	24	9.6588	32.8834	AAL95483.1| Bacterial Protein Translation Initiation Factor 1 (IF-1)
					5	10	6.8751	10.0000	
**FN1290**	-2.128	8.949	9.586e-2	2.05e-1	7	62	7.5124	84.9487	AAL95486.1| Hypothetical protein
					10	8	13.7502	8.0000	
**FN1293**									AAL95489.1| Hypothetical protein
					4		5.5001		
**FN1296**					10		10.7320		AAL95492.1| unknown
					6		8.2501		
**FN1297**	1.292	9.895	1.69e-3	5.399e-4	40	12	42.9278	16.4417	AAL95493.1| Methionine aminopeptidase
					39	23	53.6258	23.0000	

Log$_2$ Ratios: -6 -4 -2 0 2 4 6

Show detected proteins only
Show all proteins
Filter by category:
GO: amino acid transport

Proteins found: 1305

Enter (or paste) list of ORFs
Find ORFs

Test	Cutoff
q-Value	.005
p-Value	

	Signif	Direction	Applies To
red	yes	+	ratios, bars
yellow	no	n/a	bars
green	yes	−	ratios, bars
light red	yes	+	*p*-, *q*-Values
light green	yes	−	

Dot Plots Dot Plots

Protein	FnPgSg vs FnPg Log_2 Ratio	Log_2 Sum	*q*-Value	*p*-Value	Raw *FnPgSg*	Raw *FnPg*	Normalized *FnPgSg*	Normalized *FnPg*	Description
FN1298	0.289	12.628	1.183e-1	2.843e-1	64	62	68.6845	84.9487	AAL95494.1| Adenylate kinase
					78	59	107.2516	59.0000	
FN1299									AAL95495.1| dTDP-glucose 4,6-dehydratase
						6		6.0000	
FN1301	0.371	12.414	1.407e-1	3.68e-1	72	70	77.2701	95.9098	AAL95497.1| ABC transporter ATP-binding protein
					66	34	90.7514	34.0000	
FN1302	0.452	17.786	1.5e-1	4.013e-1	566	489	607.4288	669.9984	AAL95498.1| Hypothetical protein
					367	143	504.6326	143.0000	
FN1303	-0.130	12.095	1.106e-1	2.58e-1	64	55	68.6845	75.3577	AAL95499.1| hypothetical cytosolic protein
					42	63	57.7509	63.0000	
FN1304	0.162	14.215	1.013e-1	2.242e-1	145	83	155.6134	113.7216	AAL95500.1| Single-strand DNA binding protein
					99	147	136.1271	147.0000	
FN1305	-0.851	10.558	1.935e-2	2.027e-2	18	36	19.3175	49.3250	AAL95501.1| Hypothetical cytosolic protein
					28	55	38.5006	55.0000	
FN1306	-2.626	12.326			23		24.6835		AAL95502.1| Methyltransferase
					24	178	33.0005	178.0000	
FN1309	1.405	15.589	4.227e-4	6.75e-5	312	121	334.8371	165.7869	AAL95505.1| Hypothetical protein
					282	107	387.7558	107.0000	
FN1313	0.203	12.132	1.645e-1	4.536e-1	84	51	90.1484	69.8771	AAL95509.1| Oligopeptide-binding protein oppA
					39	55	53.6258	55.0000	
FN1317						12		16.4417	AAL95513.1| RNA polymerase sigma factor
						9		9.0000	
FN1318	0.262	9.943	8.204e-2	1.617e-1	32	17	34.3423	23.2924	AAL95514.1| RNA polymerase sigma factor rpoD
					25	34	34.3755	34.0000	
FN1319	-0.286	6.298	7.803e-2	1.504e-1	6	7	6.4392	9.5910	AAL95515.1| DNA primase
					7	10	9.6251	10.0000	
FN1320	0.643	15.331	1.754e-2	1.772e-2	205	116	220.0051	158.9362	AAL95516.1| Peptidyl-prolyl cis-trans isomerase
					209	166	287.3793	166.0000	
FN1321	0.032	19.594	2.554e-1	8.225e-1	969	641	1039.9267	878.2596	AAL95517.1| Acetoacetate metabolism regulatory protein atoC
					552	881	759.0114	881.0000	
FN1322	-2.664	7.511			5		5.3660		AAL95518.1| Membrane metalloprotease
						34		34.0000	

Log_2 Ratios: -6 -4 -2 0 2 4 6

Show detected proteins only
Show all proteins
Filter by category:
GO: amino acid transport

Proteins found: 1305

Enter (or paste) list of ORFs

Find ORFs

Test	Cutoff
q-Value	.005
p-Value	

	Signif	Direction	Applies To
red	yes	+	ratios, bars
yellow	no	n/a	bars
green	yes	−	ratios, bars
pink	yes	+	*p*- , *q*-Values
light green	yes	−	

Dot Plots Dot Plots

Protein	FnPgSg vs FnPg Log$_2$ Ratio	Log$_2$ Sum	*q*-Value	*p*-Value	Raw *FnPgSg*	Raw *FnPg*	Normalized *FnPgSg*	Normalized *FnPg*	Description
FN1323	-0.092	6.341	2.242e-1	6.929e-1	6	7	6.4392	9.5910	AAL95519.1| Thymidylate kinase
					8	9	11.0002	9.0000	
FN1324	-0.778	9.503	8.943e-2	1.84e-1	14	15	15.0247	20.5521	AAL95520.1| 1-deoxy-D-xylulose 5-phosphate reductoisomerase
					19	50	26.1254	50.0000	
FN1326	0.267	6.267							AAL95522.1| Undecaprenyl pyrophosphate synthetase
					7	8	9.6251	8.0000	
FN1327					8		8.5856		AAL95523.1| Dimethylallyltransferase
FN1328					4		4.2928		AAL95524.1| Exodeoxyribonuclease VII small subunit
					3		4.1251		
FN1330									AAL95526.1| S-adenosylmethionine:tRNA ribosyltransferase-isomerase
						3		3.0000	
FN1331					14		15.0247		AAL95527.1| Methyltransferase
					4		5.5001		
FN1332	0.677	10.946	9.577e-3	7.052e-3	47	25	50.4402	34.2535	AAL95528.1| Bacterial Peptide Chain Release Factor 1 (RF-1)
					45	36	61.8759	36.0000	
FN1334	-1.119	8.493			12	35	12.8783	47.9549	AAL95530.1| N-acetylmuramoyl-L-alanine amidase
						8		8.0000	
FN1335	1.764	14.060	1.02e-2	7.791e-3	176	40	188.8825	54.8056	AAL95531.1| Protein translocase subunit YajC
					213	87	292.8794	87.0000	
FN1336						16		21.9222	AAL95532.1| Hypothetical protein
FN1337	-0.216	5.590			6	8	6.4392	10.9611	AAL95533.1| unknown
						4		4.0000	
FN1340	0.565	16.702	4.847e-2	7.589e-2	403	259	432.4979	354.8662	AAL95536.1| Glutamyl-tRNA synthetase
					263	182	361.6305	182.0000	
FN1341	0.678	7.848			14		15.0247		AAL95537.1| Bacterial Peptide Chain Release Factor 2 (RF-2)
					17	12	23.3754	12.0000	
FN1343									AAL95539.1| seC-independent protein TATD
						45		45.0000	
FN1346									AAL95542.1| Hypothetical cytosolic protein
						6		6.0000	

Log$_2$ Ratios: -6 -4 -2 0 2 4 6

Show detected proteins only
Show all proteins
Filter by category:
GO: amino acid transport

Proteins found: 1305

Enter (or paste) list of ORFs

Find ORFs

Test	Cutoff
q-Value	.005
p-Value	

Signif	Direction	Applies To
yes	+	ratios, bars
no	n/a	bars
yes	−	ratios, bars
yes	+	*p*-, *q*-Values
yes	−	

Dot Plots Dot Plots

Protein	FnPgSg vs FnPg Log$_2$ Ratio	Log$_2$ Sum	*q*-Value	*p*-Value	Raw *FnPgSg*	Raw *FnPg*	Normalized *FnPgSg*	Normalized *FnPg*	Description
**FN1347**	0.638	6.423	1.853e-3	6.23e-4	10	5	10.7320	6.8507	AAL95543.1| Hypothetical cytosolic protein
					9	8	12.3752	8.0000	
**FN1348**	0.864	7.845	1.433e-1	3.773e-1	33	4	35.4155	5.4806	AAL95544.1| ABC transporter ATP-binding protein
					4	17	5.5001	17.0000	
**FN1349**	0.295	5.909			8		8.5856		AAL95545.1| ABC transporter permease protein
						7		7.0000	
**FN1351**	-0.686	9.966	2.08e-2	2.244e-2	17	33	18.2443	45.2146	AAL95547.1| 15 kDa lipoprotein precursor
					23	35	31.6255	35.0000	
**FN1352**	0.330	11.495	1.903e-2	1.981e-2	61	32	65.4649	43.8445	AAL95548.1| ABC transporter ATP-binding protein
					40	52	55.0008	52.0000	
**FN1353**	-0.427	7.188			13		13.9515		AAL95549.1| ABC transporter permease protein
					5	14	6.8751	14.0000	
**FN1354**	-0.395	9.513			26		27.9031		AAL95550.1| ABC transporter permease protein
					14	31	19.2503	31.0000	
**FN1355**					3		3.2196		AAL95551.1| Integral membrane protein
**FN1358**	-0.327	10.843			38		40.7814		AAL95554.1| Hypothetical protein
					26	48	35.7505	48.0000	
**FN1359**	-2.440	15.548	5.137e-2	8.292e-2	97	167	104.1000	228.8134	AAL95555.1| Dipeptide-binding protein
					61	791	83.8763	791.0000	
**FN1362**						25		34.2535	AAL95558.1| Dipeptide transport ATP-binding protein dppD
						70		70.0000	
**FN1363**	-3.037	7.531	3.106e-2	4.001e-2	5	16	5.3660	21.9222	AAL95559.1| Dipeptide transport ATP-binding protein dppF
					3	56	4.1251	56.0000	
**FN1364**	0.510	11.233			54	30	57.9526	41.1042	AAL95560.1| LSU ribosomal protein L32P
					43		59.1259		
**FN1365**	0.808	14.560	4.973e-2	7.89e-2	186	43	199.6144	58.9160	AAL95561.1| GTP-binding protein
					154	176	211.7532	176.0000	
**FN1366**	1.333	15.947	1.247e-4	1.386e-5	350	99	375.6185	135.6438	AAL95562.1| Triosephosphate isomerase
					307	181	422.1314	181.0000	
**FN1371**									AAL95567.1| Ribonuclease HII
						4		4.0000	

Log$_2$ Ratios: -6 -4 -2 0 2 4 6

○ Show detected proteins only
○ Show all proteins
☐ Filter by category: GO: amino acid transport

Proteins found: 1305

Enter (or paste) list of ORFs

Find ORFs

Test	Cutoff
q-Value	.005
p-Value	

Color	Signif	Direction	Applies To
red	yes	+	ratios, bars
yellow	no	n/a	bars
green	yes	−	ratios, bars
pink	yes	+	*p*-, *q*-Values
light green	yes	−	*p*-, *q*-Values

Dot Plots | Dot Plots

Protein	*FnPgSg vs FnPg* Log$_2$ Ratio	Log$_2$ Sum	*q*-Value	*p*-Value	Raw *FnPgSg*	Raw *FnPg*	Normalized *FnPgSg*	Normalized *FnPg*	Description
**FN1374**	0.464	6.079			9		9.6588		AAL95570.1| Transcriptional regulator
						7		7.0000	
**FN1375**	-1.984	11.274			21		22.5371		AAL95571.1| Citrate-sodium symport
					20	99	27.5004	99.0000	
**FN1376**	-0.980	17.500	5.701e-2	9.735e-2	319	282	342.3494	386.3794	AAL95572.1| Oxaloacetate decarboxylase alpha chain
					197	823	270.8791	823.0000	
**FN1378**	0.296	9.334	2.048e-1	6.171e-1	23	5	24.6835	6.8507	AAL95574.1| Citrate lyase acyl carrier protein
					23	39	31.6255	39.0000	
**FN1379**	-0.539	15.584	1.748e-1	4.929e-1	185	60	198.5412	82.2084	AAL95575.1| Citrate lyase beta chain
					123	452	169.1275	452.0000	
**FN1380**	-0.467	17.815	1.994e-1	5.936e-1	359	81	385.2773	110.9813	AAL95576.1| Citrate lyase beta chain
					314	1018	431.7565	1018.0000	
**FN1381**									AAL95577.1| unknown
					3		4.1251		
**FN1382**									AAL95578.1| ATPase
						13		13.0000	
**FN1383**	-1.428	4.802			3	9	3.2196	12.3313	AAL95579.1| DNA polymerase III alpha subunit
						5		5.0000	
**FN1385**									AAL95581.1| Hypothetical protein
						3		3.0000	
**FN1386**	0.411	7.330			17		18.2443		AAL95582.1| SWF/SNF family helicase
					8	11	11.0002	11.0000	
**FN1391**	-0.221	11.457	1.331e-1	3.396e-1	39	50	41.8546	68.5070	AAL95584.1| Acetyltransferase
					41	46	56.3758	46.0000	
**FN1392**	0.468	14.662	1.487e-1	3.967e-1	185	167	198.5412	228.8134	AAL95585.1| SSU ribosomal protein S16P
					131	45	180.1277	45.0000	
**FN1393**	0.974	8.961	3.775e-2	5.309e-2	25	5	26.8299	6.8507	AAL95586.1| Signal recognition particle, subunit FFH/SRP54
					26	25	35.7505	25.0000	
**FN1397**	1.005	13.309	1.533e-2	1.465e-2	157	33	168.4917	45.2146	AAL95590.1| Glutaminase
					85	97	116.8768	97.0000	
**FN1398**	1.722	13.334	9.724e-3	7.22e-3	171	16	183.5165	21.9222	AAL95591.1| Amino acid carrier protein alsT
					135	90	185.6278	90.0000	

Log$_2$ Ratios: -6 -4 -2 0 2 4 6

- Show detected proteins only
- Show all proteins
- Filter by category: GO: amino acid transport

Proteins found: 1305

Enter (or paste) list of ORFs

Find ORFs

Test	Cutoff
q-Value	.005
p-Value	

	Signif	Direction	Applies To
red	yes	+	ratios, bars
yellow	no	n/a	bars
green	yes	−	ratios, bars
pink	yes	+	*p*-, *q*-Values
light green	yes	−	

Dot Plots | Dot Plots

Protein	*FnPgSg vs FnPg* Log$_2$ Ratio	Log$_2$ Sum	*q*-Value	*p*-Value	Raw *FnPgSg*	Raw *FnPg*	Normalized *FnPgSg*	Normalized *FnPg*	Description
**FN1400**	1.437	5.097				3		4.1104	AAL95593.1| serine/threonine kinase
					7	3	9.6251	3.0000	
**FN1406**	-0.229	11.949	2.214e-1	6.816e-1	48	22	51.5134	30.1431	AAL95599.1| Histidine ammonia-lyase
					47	106	64.6260	106.0000	
**FN1407**	-0.870	9.130			7		7.5124		AAL95600.1| Glutamate formiminotransferase
					20	32	27.5004	32.0000	
**FN1411**	-1.075	16.082	3.508e-2	4.799e-2	210	196	225.3711	268.5474	AAL95604.1| Threonine dehydratase
					100	496	137.5021	496.0000	
**FN1412**						20		27.4028	AAL95605.1| 5-methylthioribose kinase
**FN1413**	-2.089	5.463			3	10	3.2196	13.7014	AAL95606.1| Translation initiation factor EIF-2B subunit 1
**FN1415**						11		15.0715	AAL95608.1| NADH-dependent butanol dehydrogenase A
						7		7.0000	
**FN1416**	-2.338	6.832			5		5.3660		AAL95609.1| Transcriptional regulator, GntR family
					3	24	4.1251	24.0000	
**FN1417**	-1.009	5.856			5	7	5.3660	9.5910	AAL95610.1| L-fuculose phosphate aldolase
						12		12.0000	
**FN1418**						3		4.1104	AAL95611.1| Transcriptional regulator, GntR family
**FN1419**	-1.223	23.727	6.688e-2	1.213e-1	2312	6075	2481.2286	8323.5996	AAL95612.1| Methionine gamma-lyase
					1743	3065	2396.6611	3065.0000	
**FN1420**	-1.344	9.163			14	44	15.0247	60.2862	AAL95613.1| NA+/H+ antiporter NHAC
						16		16.0000	
**FN1421**	-0.809	21.378	7.487e-2	1.419e-1	1134	2193	1217.0040	3004.7167	AAL95614.1| Pyruvate-flavodoxin oxidoreductase
					929	1365	1277.3943	1365.0000	
**FN1423**	1.183	14.461	1.71e-2	1.712e-2	227	36	243.6154	49.3250	AAL95616.1| Flavoprotein
					152	150	209.0032	150.0000	
**FN1424**	1.480	16.056	1.038e-2	8.016e-3	478	45	512.9876	61.6563	AAL95617.1| ACYL-COA dehydrogenase, short-chain specific
					261	251	358.8804	251.0000	
**FN1426**	0.252	14.534	2.048e-1	6.173e-1	130	44	139.5154	60.2862	AAL95619.1| Serine protease
					143	222	196.6280	222.0000	

Log$_2$ Ratios: -6 -4 -2 0 2 4 6

Show detected proteins only
Show all proteins
Filter by category: GO: amino acid transport

Proteins found: 1305

Enter (or paste) list of ORFs

Find ORFs

Test	Cutoff
q-Value	.005
p-Value	

Color	Signif	Direction	Applies To
red	yes	+	ratios, bars
yellow	no	n/a	bars
green	yes	–	ratios, bars
pink	yes	+	*p*-, *q*-Values
light green	yes	–	

Dot Plots | Dot Plots

Protein	FnPgSg vs FnPg Log$_2$ Ratio	Log$_2$ Sum	*q*-Value	*p*-Value	Raw *FnPgSg*	Raw *FnPg*	Normalized *FnPgSg*	Normalized *FnPg*	Description	Log$_2$ Ratios (-6 -4 -2 0 2 4 6)
**FN1427**	-0.744	5.900			6		6.4392		AAL95620.1| Phenazine biosynthesis protein phzF	
					4	10	5.5001	10.0000		
**FN1432**					25		26.8299		AAL95625.1| Leucine-, isoleucine-, valine-, threonine-, and alanine-binding protein	
					19		26.1254			
**FN1433**	0.278	16.457	3.338e-2	4.457e-2	336	202	360.5938	276.7682	AAL95626.1| Short chain dehydrogenase	
					218	268	299.7545	268.0000		
**FN1434**	-0.143	13.211	2.049e-1	6.177e-1	92	53	98.7340	72.6174	AAL95627.1| Tetratricopeptide repeat family protein	
					63	132	86.6263	132.0000		
**FN1437**	0.330	13.980	1.178e-1	2.828e-1	117	110	125.5639	150.7154	AAL95630.1| LSU ribosomal protein L28P	
					116	76	159.5024	76.0000		
**FN1439**	0.413	9.460			34		36.4887		AAL95632.1| Transcriptional regulator, DeoR family	
					18	23	24.7504	23.0000		
**FN1440**	0.397	10.717	1.84e-2	1.892e-2	48	23	51.5134	31.5132	AAL95633.1| 1-phosphofructokinase	
					31	40	42.6256	40.0000		
**FN1441**	1.419	12.966	9.978e-3	7.52e-3	129	20	138.4423	27.4028	AAL95634.1| PTS system, fructose-specific IIABC component	
					112	82	154.0023	82.0000		
**FN1444**	-0.302	17.446	2.514e-2	2.937e-2	375	374	402.4484	512.4323	AAL95637.1| GMP synthase (glutamine-hydrolyzing)	
					261	426	358.8804	426.0000		
**FN1445**	0.798	9.316	9.897e-3	7.423e-3	30	17	32.1959	23.2924	AAL95638.1| DNA helicase	
					25	15	34.3755	15.0000		
**FN1449**	1.945	13.670	8.015e-3	5.339e-3	165	55	177.0773	75.3577	AAL95642.1| Fusobacterium outer membrane protein family	
					197	41	270.8791	41.0000		
**FN1450**	-0.659	7.681			11		11.8052		AAL95643.1| Integral membrane protein	
					8	18	11.0002	18.0000		
**FN1451**	-0.324	14.456	2.679e-2	3.221e-2	110	120	118.0515	164.4168	AAL95644.1| Cell division protein ftsZ	
					109	171	149.8773	171.0000		
**FN1452**	-0.652	12.996	1.991e-2	2.109e-2	78	96	83.7093	131.5334	AAL95645.1| Cell division protein ftsA	
					44	95	60.5009	95.0000		
**FN1454**	0.400	9.184			26		27.9031		AAL95647.1| D-alanine--D-alanine ligase	
					20	21	27.5004	21.0000		
**FN1455**	-0.668	8.119	5.658e-2	9.629e-2	8	19	8.5856	26.0327	AAL95648.1| UDP-N-acetylenolpyruvoylglucosamine reductase	
					13	16	17.8753	16.0000		

Show detected proteins only
Show all proteins
Filter by category:
GO: amino acid transport

Proteins found: 1305

Enter (or paste) list of ORFs

Find ORFs

Test	Cutoff
q-Value	.005
p-Value	

	Signif	Direction	Applies To
(red)	yes	+	ratios, bars
(yellow)	no	n/a	bars
(green)	yes	−	ratios, bars
(pink)	yes	+	*p*- , *q*-Values
(light green)	yes	−	

Dot Plots Dot Plots

Protein	FnPgSg vs FnPg: Log_2 Ratio	Log_2 Sum	*q*-Value	*p*-Value	Raw *FnPgSg*	Raw *FnPg*	Normalized *FnPgSg*	Normalized *FnPg*	Description
**FN1456**	-0.141	11.337	1.179e-1	2.831e-1	39	40	41.8546	54.8056	AAL95649.1| UDP-N-acetylmuramate--alanine ligase
					40	52	55.0008	52.0000	
**FN1457**	-0.882	9.875	1.051e-1	2.38e-1	19	14	20.3907	19.1820	AAL95650.1| UDP-N-acetylglucosamine-N-acetylmuramyl-Pentapeptide pyrophosphoryl-undecaprenol N-
					18	64	24.7504	64.0000	
**FN1458**	-0.882	11.745	8.629e-2	1.743e-1	42	84	45.0742	115.0917	AAL95651.1| UDP-N-acetylmuramoylalanine--D-glutamate ligase
					30	44	41.2506	44.0000	
**FN1461**	-0.519	9.012	1.223e-2	1.024e-2	20	20	21.4639	27.4028	AAL95654.1| Histidinol-phosphatase
					12	27	16.5002	27.0000	
**FN1463**	0.460	17.251	2.016e-2	2.146e-2	448	285	480.7917	390.4899	AAL95656.1| pyridoxine biosynthesis protein
					324	283	445.5067	283.0000	
**FN1464**	-0.614	15.050	1.163e-1	2.776e-1	125	91	134.1495	124.6827	AAL95657.1| 1-deoxyxylulose-5-phosphate synthase
					119	331	163.6275	331.0000	
**FN1470**	2.237	10.196	2.787e-4	3.797e-5	63	15	67.6113	20.5521	AAL95663.1| Hypothetical protein
					59	11	81.1262	11.0000	
**FN1471**					5		5.3660		AAL95664.1| LACI-family transcription regulator
**FN1472**	2.046	11.534	5.896e-3	3.207e-3	105	7	112.6856	9.5910	AAL95665.1| N-acetylneuraminate-binding protein
					79	44	108.6266	44.0000	
**FN1475**	2.477	10.769	6.392e-4	1.253e-4	103	20	110.5392	27.4028	AAL95668.1| N-acetylneuraminate lyase
					63	8	86.6263	8.0000	
**FN1476**					11		11.8052		AAL95669.1| N-acetylmannosamine-6-phosphate 2-epimerase
					9		12.3752		
**FN1478**	-0.894	8.153							AAL95671.1| Hypothetical protein
					9	23	12.3752	23.0000	
**FN1479**	0.090	8.784	2.494e-1	7.965e-1	25	10	26.8299	13.7014	AAL95672.1| Hypothetical protein
					12	27	16.5002	27.0000	
**FN1480**	-1.480	7.050	3.565e-3	1.595e-3	9	12	9.6588	16.4417	AAL95673.1| MG2+ transporter MGTE
					3	22	4.1251	22.0000	
**FN1481**	-1.722	5.926			4	9	4.2928	12.3313	AAL95674.1| Queuine tRNA-ribosyltransferase
						16		16.0000	
**FN1482**	-1.516	9.828	1.034e-4	8.913e-6	14	35	15.0247	47.9549	AAL95675.1| Guanosine-3',5'-bis (Diphosphate) 3'-pyrophosphohydrolase
					15	54	20.6253	54.0000	

Log_2 Ratios: -6 -4 -2 0 2 4 6

Show detected proteins only
Show all proteins
Filter by category:
GO: amino acid transport

Proteins found: 1305

Enter (or paste) list of ORFs

Find ORFs

Test	Cutoff
q-Value	.005
p-Value	

	Signif	Direction	Applies To
red	yes	+	ratios, bars
yellow	no	n/a	bars
green	yes	−	ratios, bars
pink	yes	+	*p*- , *q*-Values
light green	yes	−	

Dot Plots | Dot Plots

	FnPgSg vs FnPg				Raw		Normalized			Log_2 Ratios
Protein	Log_2 Ratio	Log_2 Sum	*q*-Value	*p*-Value	*FnPgSg*	*FnPg*	*FnPgSg*	*FnPg*	Description	-6 -4 -2 0 2 4 6
**FN1483**	0.976	10.717	9.76e-5	7.935e-6	56	23	60.0990	31.5132	AAL95676.1| Adenine phosphoribosyltransferase	
					40	27	55.0008	27.0000		
**FN1484**					8		8.5856		AAL95677.1| Tetratricopeptide repeat family protein	
					5		6.8751			
**FN1486**					5		5.3660		AAL95679.1| magnesium and cobalt efflux protein CorC	
					3		4.1251			
**FN1487**	0.896	14.076	2.117e-2	2.301e-2	197	45	211.4196	61.6563	AAL95681.1| Chorismate mutase	
					107	131	147.1272	131.0000		
**FN1488**	-0.722	8.073	9.775e-2	2.114e-1	11	22	11.8052	30.1431	AAL95682.1| Methylenetetrahydrofolate dehydrogenase (NADP+)	
					10	12	13.7502	12.0000		
**FN1489**	-1.192	7.310	1.698e-2	1.695e-2	4	11	4.2928	15.0715	AAL95683.1| Methionyl-tRNA formyltransferase	
					9	23	12.3752	23.0000		
**FN1490**	-1.192	7.011			7	9	7.5124	12.3313	AAL95684.1| putative regulatory protein	
						22		22.0000		
**FN1491**									AAL95685.1| PTS system, IIA component	
						10		10.0000		
**FN1493**						7		9.5910	AAL95687.1| Hypothetical protein	
						12		12.0000		
**FN1494**	0.525	7.169			14		15.0247		AAL95680.1| Rod shape-determining protein mreC	
					10	10	13.7502	10.0000		
**FN1496**	0.525	7.169			14		15.0247		AAL95680.1| Rod shape-determining protein mreC	
					10	10	13.7502	10.0000		
**FN1499**	1.149	12.680	3.316e-2	4.413e-2	98	13	105.1732	17.8118	AAL93625.1| Cell surface protein	
					99	91	136.1271	91.0000		
**FN1501**									AAL93627.1| Nickel transport ATP-binding protein nikD	
					3		4.1251			
**FN1504**	0.693	12.929	7.972e-3	5.287e-3	117	43	125.5639	58.9160	AAL93630.1| Nickel-binding protein	
					72	80	99.0015	80.0000		
**FN1505**	-0.756	15.444	1.474e-2	1.372e-2	158	172	169.5649	235.6641	AAL93631.1| 6,7-dimethyl-8-ribityllumazine synthase	
					113	313	155.3773	313.0000		
**FN1506**	0.033	8.606	2.755e-1	9.117e-1	18	19	19.3175	26.0327	AAL93632.1| Diaminohydroxyphosphoribosylaminopyrimidine deaminase	
					15	13	20.6253	13.0000		

Show detected proteins only
Show all proteins
Filter by category:
GO: amino acid transport

Proteins found: 1305

Enter (or paste) list of ORFs

Find ORFs

Test	Cutoff
q-Value	.005
p-Value	

	Signif	Direction	Applies To
(red)	yes	+	ratios, bars
(yellow)	no	n/a	bars
(green)	yes	–	ratios, bars
(pink)	yes	+	*p*-, *q*-Values
(light green)	yes	–	

Dot Plots | Dot Plots

Protein	FnPgSg vs FnPg Log2 Ratio	Log2 Sum	q-Value	p-Value	Raw FnPgSg	Raw FnPg	Normalized FnPgSg	Normalized FnPg	Description
FN1508	0.397	13.751	7.947e-2	1.544e-1	123	53	132.0031	72.6174	AAL93634.1| GTP cyclohydrolase II
					100	132	137.5021	132.0000	
FN1512					3		3.2196		AAL93638.1| hypothetical exported 24-amino acid repeat protein
					3		4.1251		
FN1517	0.306	15.584	1.128e-1	2.654e-1	235	102	252.2010	139.7543	AAL93643.1| Leucyl-tRNA synthetase
					175	259	240.6286	259.0000	
FN1519	0.904	8.305			21		22.5371		AAL93645.1| 23S rRNA methyltransferase
					19	13	26.1254	13.0000	
FN1520	1.973	11.802	1.938e-3	6.702e-4	122	9	130.9299	12.3313	AAL93646.1| UDP-N-acetylglucosamine 1-carboxyvinyltransferase
					77	48	105.8766	48.0000	
FN1523	1.503	14.521	7.781e-4	1.643e-4	253	68	271.5185	93.1695	AAL93649.1| Dipeptide-binding protein
					178	89	244.7537	89.0000	
FN1525									AAL93651.1| Dipeptide transport ATP-binding protein dppF
					6		8.2501		
FN1526	-0.286	21.917	2.052e-1	6.188e-1	1491	2501	1600.1349	3426.7198	AAL93652.1| Fusobacterium outer membrane protein family
					1457	968	2003.4052	968.0000	
FN1527	1.938	11.812	2.33e-3	8.551e-4	97	25	104.1000	34.2535	AAL93653.1| Hypothetical protein
					95	27	130.6270	27.0000	
FN1528	1.037	12.554	1.318e-2	1.148e-2	84	52	90.1484	71.2473	AAL93654.1| Hypothetical protein
					96	37	132.0020	37.0000	
FN1529	0.058	12.459	2.761e-1	9.146e-1	53	84	56.8794	115.0917	AAL93655.1| Hypothetical protein
					70	32	96.2515	32.0000	
FN1531	0.554	10.667	9.789e-2	2.119e-1	59	15	63.3185	20.5521	AAL93657.1| murein hydrolase export regulator
					25	46	34.3755	46.0000	
FN1533	-0.141	19.382	2.545e-1	8.186e-1	744	1043	798.4576	1429.0559	AAL93659.1| Electron transfer flavoprotein alpha-subunit
					564	307	775.5117	307.0000	
FN1534	-0.628	20.216	1.251e-1	3.091e-1	868	1502	931.5339	2057.9500	AAL93660.1| Electron transfer flavoprotein beta-subunit
					614	686	844.2627	686.0000	
FN1535	-0.790	21.547	1.079e-2	8.541e-3	1208	1883	1296.4205	2579.9733	AAL93661.1| Acyl-CoA dehydrogenase, short-chain specific
					993	2023	1365.3956	2023.0000	
FN1536	-1.310	19.626	6.391e-3	3.632e-3	539	905	578.4525	1239.9766	AAL93662.1| (S)-2-hydroxy-acid oxidase chain D
					410	1593	563.7585	1593.0000	

Log2 Ratios

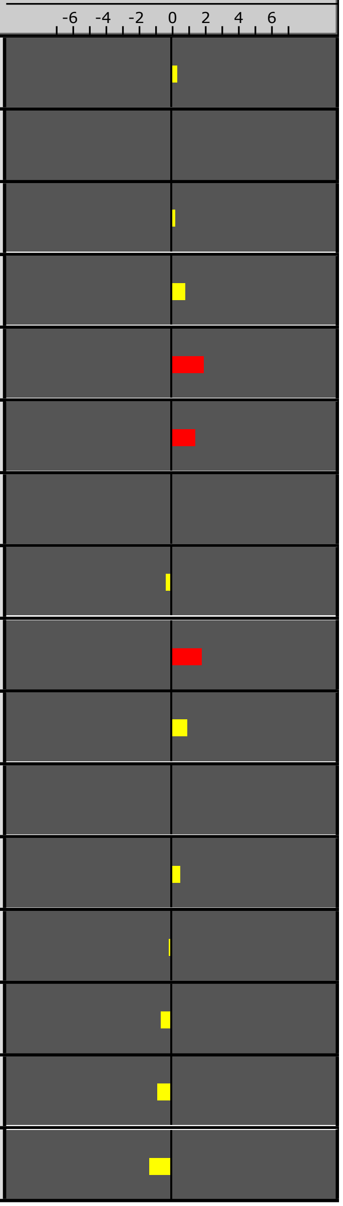

- Show detected proteins only
- Show all proteins
- Filter by category: GO: amino acid transport

Proteins found: 1305

Enter (or paste) list of ORFs

Find ORFs

Test	Cutoff
q-Value	.005
p-Value	

	Signif	Direction	Applies To
red	yes	+	ratios, bars
yellow	no	n/a	bars
green	yes	−	ratios, bars
pink	yes	+	p-, q-Values
light green	yes	−	

Dot Plots | Dot Plots

Protein	*FnPgSg vs FnPg* Log$_2$ Ratio	Log$_2$ Sum	*q*-Value	*p*-Value	Raw *FnPgSg*	Raw *FnPg*	Normalized *FnPgSg*	Normalized *FnPg*	Description
**FN1537**						4		5.4806	AAL93663.1| Arsenical pump-driving ATPase
**FN1538**	-0.584	7.128			9	8	9.6588	10.9611	AAL93664.1| Arsenical pump-driving ATPase
						18		18.0000	
**FN1539**	-0.510	15.676	6.401e-2	1.143e-1	187	246	200.6876	337.0544	AAL93665.1| Iron-sulfur cluster-binding protein
					133	209	182.8778	209.0000	
**FN1540**	-1.799	16.108	1.516e-2	1.439e-2	122	450	130.9299	616.5629	AAL93666.1| Iron-sulfur cluster-binding protein
					112	375	154.0023	375.0000	
**FN1544**	-1.944	17.036	4.389e-2	6.559e-2	156	762	167.4185	1044.0466	AAL93670.1| Probable electron transfer flavoprotein-quinone oxidoreductase ydiS
					150	394	206.2531	394.0000	
**FN1545**	-2.497	11.678	7.813e-2	1.507e-1	18	173	19.3175	237.0342	AAL93671.1| Ferredoxin like protein
					21	35	28.8754	35.0000	
**FN1546**	-0.465	21.417	1.501e-1	4.019e-1	1330	2138	1427.3504	2929.3590	AAL93672.1| Protein Translation Elongation Factor G (EF-G)
					1034	1003	1421.7714	1003.0000	
**FN1547**	0.404	14.279	2.769e-2	3.382e-2	151	76	162.0526	104.1306	AAL93673.1| PTS permease for N-acetylglucosamine and glucose
					118	141	162.2524	141.0000	
**FN1548**	0.967	10.518	3.4e-2	4.586e-2	37	21	39.7082	28.7729	AAL93674.1| Hypothetical protein
					49	26	67.3760	26.0000	
**FN1549**	0.945	19.354	1.889e-3	6.427e-4	1019	362	1093.5865	495.9906	AAL93675.1| Stomatin like protein
					857	684	1178.3928	684.0000	
**FN1552**									AAL93678.1| abortive phage resistance protein
						4		4.0000	
**FN1553**	1.083	7.862	2.959e-2	3.729e-2	26	8	27.9031	10.9611	AAL93679.1| abortive phage resistance protein
					12	10	16.5002	10.0000	
**FN1554**	-0.065	15.787	2.053e-1	6.193e-1	195	158	209.2732	216.4821	AAL93680.1| Fusobacterium outer membrane protein family
					186	270	255.7539	270.0000	
**FN1555**	0.590	24.162	3.299e-5	1.409e-6	5050	2528	5419.6386	3463.7135	AAL93681.1| Protein Translation Elongation Factor Tu
					3790	3600	5211.3286	3600.0000	
**FN1556**	1.026	20.209	1.398e-4	1.616e-5	1392	621	1493.8885	850.8568	AAL93682.1| Protein Translation Elongation Factor G (EF-G)
					1199	692	1648.6499	692.0000	
**FN1557**	1.182	16.033	9.898e-4	2.369e-4	381	124	408.8876	169.8973	AAL93683.1| SSU ribosomal protein S7P
					270	174	371.2556	174.0000	

Log$_2$ Ratios: -6 -4 -2 0 2 4 6

Show detected proteins only
Show all proteins
Filter by category:
GO: amino acid transport

Proteins found: 1305

Enter (or paste) list of ORFs

Find ORFs

Test	Cutoff
q-Value	.005
p-Value	

	Signif	Direction	Applies To
red	yes	+	ratios, bars
yellow	no	n/a	bars
green	yes	−	ratios, bars
pink	yes	+	*p*- , *q*-Values
light green	yes	−	

Dot Plots Dot Plots

Protein	FnPgSg vs FnPg: Log_2 Ratio	Log_2 Sum	*q*-Value	*p*-Value	Raw: *FnPgSg*	Raw: *FnPg*	Normalized: *FnPgSg*	Normalized: *FnPg*	Description
**FN1558**	0.138	13.531				128		175.3779	AAL93684.1| SSU ribosomal protein S12P
					83	32	114.1267	32.0000	
**FN1560**	-0.096	13.297	2.736e-1	9.028e-1	86	12	92.2948	16.4417	AAL93686.1| unknown
					74	191	101.7515	191.0000	
**FN1562**	-0.395	5.243			5	3	5.3660	4.1104	AAL93688.1| Phospho-2-dehydro-3-deoxyheptonate aldolase
						10		10.0000	
**FN1577**	-0.517	15.208	1.011e-1	2.234e-1	152	112	163.1258	153.4557	AAL93692.1| Rod shape-determining protein mreB
					118	312	162.2524	312.0000	
**FN1579**	-0.425	12.341	1.029e-1	2.3e-1	62	43	66.5381	58.9160	AAL93694.1| Cysteinyl-tRNA synthetase
					42	108	57.7509	108.0000	
**FN1581**	-0.035	10.214	2.59e-1	8.38e-1	34	21	36.4887	28.7729	AAL93696.1| DNA mismatch repair protein mutS
					23	41	31.6255	41.0000	
**FN1582**									AAL93697.1| Hypothetical protein
						22		22.0000	
**FN1586**									AAL93701.1| O-succinylbenzoate-CoA synthase
						18		18.0000	
**FN1589**	0.495	8.389	1.205e-1	2.925e-1	20	5	21.4639	6.8507	AAL93704.1| LexA repressor
					16	24	22.0003	24.0000	
**FN1590**									AAL93705.1| Hypothetical lipoprotein
						5		5.0000	
**FN1591**	0.431	15.252	1.435e-2	1.313e-2	202	141	216.7855	193.1897	AAL93706.1| RNFB-related protein
					176	147	242.0036	147.0000	
**FN1592**	0.344	7.745							AAL93707.1| Na(+)-translocating NADH-quinone reductase subunit D
					12	13	16.5002	13.0000	
**FN1594**	1.334	11.910	2.603e-3	9.967e-4	90	22	96.5876	30.1431	AAL93709.1| Nitrogen fixation protein RNFG
					73	48	100.3765	48.0000	
**FN1595**	0.361	14.681	1.798e-1	5.125e-1	177	35	189.9556	47.9549	AAL93710.1| Na(+)-translocating NADH-quinone reductase subunit B
					129	238	177.3777	238.0000	
**FN1596**	0.350	19.226	1.36e-1	3.515e-1	748	722	802.7504	989.2410	AAL93711.1| Nitrogen fixation iron-sulphur protein RNFC
					702	398	965.2646	398.0000	
**FN1597**	-2.674	6.878			4	20	4.2928	27.4028	AAL93712.1| Peptidyl-tRNA hydrolase

Log_2 Ratios: -6 -4 -2 0 2 4 6

Show detected proteins only
Show all proteins
Filter by category:
GO: amino acid transport

Proteins found: 1305

Enter (or paste) list of ORFs

Find ORFs

Test	Cutoff
***q*-Value**	.005
p-Value	

Color	Signif	Direction	Applies To
red	yes	+	ratios, bars
yellow	no	n/a	bars
green	yes	−	ratios, bars
pink	yes	+	*p*- , *q*-Values
light green	yes	−	

Dot Plots | Dot Plots

Protein	*FnPgSg vs FnPg* Log$_2$ Ratio	Log$_2$ Sum	*q*-Value	*p*-Value	Raw *FnPgSg*	Raw *FnPg*	Normalized *FnPgSg*	Normalized *FnPg*	Description
**FN1600**									AAL93715.1| tRNA pseudouridine synthase A
						11		11.0000	
**FN1602**									AAL93717.1| Hypothetical cytosolic protein
					4		5.5001		
**FN1603**	0.345	5.253			4	4	4.2928	5.4806	AAL93718.1| 2',3'-cyclic nucleotide 3'-phosphodiesterase
					7		9.6251		
**FN1605**	-0.233	16.203	1.305e-1	3.296e-1	239	169	256.4938	231.5536	AAL93720.1| Adenylosuccinate synthetase
					182	364	250.2538	364.0000	
**FN1606**	-0.294	8.179	1.352e-1	3.481e-1	12	10	12.8783	13.7014	AAL93721.1| 3-deoxy-D-manno-octulosonic-acid transferase
					13	24	17.8753	24.0000	
**FN1607**	-0.673	7.592				11		15.0715	AAL93722.1| Cytidylate kinase
					8	20	11.0002	20.0000	
**FN1608**									AAL93723.1| Ribosomal protein L11 methyltransferase
						8		8.0000	
**FN1609**	-1.483	5.687			4		4.2928		AAL93724.1| Hypothetical protein
						12		12.0000	
**FN1610**	-1.511	9.013	3.332e-5	1.433e-6	11	29	11.8052	39.7341	AAL93725.1| 33 kDa chaperonin
					11	37	15.1252	37.0000	
**FN1613**	0.062	8.599	1.88e-1	5.455e-1	17	15	18.2443	20.5521	AAL93728.1| Hypothetical protein
					16	18	22.0003	18.0000	
**FN1614**	-0.524	9.186	2.064e-2	2.22e-2	17	24	18.2443	32.8834	AAL93729.1| MG(2+) chelatase family protein
					16	25	22.0003	25.0000	
**FN1616**	1.848	9.848			74		79.4165		AAL93731.1| N utilization substance protein B
					26	16	35.7505	16.0000	
**FN1618**	0.500	10.812	6.944e-2	1.277e-1	44	17	47.2206	23.2924	AAL93733.1| Hypothetical protein
					39	48	53.6258	48.0000	
**FN1619**	0.725	16.205	1.195e-2	9.897e-3	282	134	302.6412	183.5987	AAL93734.1| Hypothetical cytosolic protein
					294	244	404.2561	244.0000	
**FN1620**	0.500	18.254	7.539e-2	1.433e-1	624	468	669.6741	641.2254	AAL93735.1| SSU ribosomal protein S2P
					480	299	660.0099	299.0000	
**FN1621**	0.551	20.474	3.868e-2	5.494e-2	1181	923	1267.4442	1264.6391	AAL93736.1| Protein Translation Elongation Factor Ts
					1203	730	1654.1499	730.0000	

Log$_2$ Ratios: -6 -4 -2 0 2 4 6

Show detected proteins only
Show all proteins
Filter by category:
GO: amino acid transport

Proteins found: 1305

Enter (or paste) list of ORFs

Find ORFs

Test	Cutoff
q-Value	.005
p-Value	

	Signif	Direction	Applies To
red	yes	+	ratios, bars
yellow	no	n/a	bars
green	yes	−	ratios, bars
pink	yes	+	*p*- , *q*-Values
light green	yes	−	

Dot Plots Dot Plots

Protein	*FnPgSg vs FnPg* Log$_2$ Ratio	Log$_2$ Sum	*q*-Value	*p*-Value	Raw *FnPgSg*	Raw *FnPg*	Normalized *FnPgSg*	Normalized *FnPg*	Description
**FN1622**	1.232	14.365	1.129e-3	2.962e-4	224	53	240.3958	72.6174	AAL93737.1| Uridylate kinase
					149	117	204.8781	117.0000	
**FN1623**	0.982	11.407	3.319e-3	1.433e-3	66	22	70.8309	30.1431	AAL93738.1| Ribosome Recycling Factor (RRF)
					55	44	75.6261	44.0000	
**FN1624**	1.666	10.584			66		70.8309		AAL93739.1| Protein translocase subunit secY
					50	22	68.7510	22.0000	
**FN1625**	0.625	13.277	6.993e-2	1.29e-1	114	85	122.3443	116.4619	AAL93740.1| LSU ribosomal protein L15P
					91	44	125.1269	44.0000	
**FN1626**	2.475	8.815			51		54.7330		AAL93741.1| LSU ribosomal protein L30P
					33	9	45.3757	9.0000	
**FN1627**	1.420	17.495	1.469e-2	1.365e-2	791	193	848.8978	264.4370	AAL93742.1| SSU ribosomal protein S5P
					405	261	556.8834	261.0000	
**FN1628**	-0.145	15.795			242	183	259.7134	250.7356	AAL93743.1| LSU ribosomal protein L18P
					141		193.8779		
**FN1629**	-0.359	16.420	1.793e-1	5.103e-1	217	369	232.8835	505.5816	AAL93744.1| LSU ribosomal protein L6P
					211	165	290.1294	165.0000	
**FN1630**	1.095	13.592	4.901e-2	7.716e-2	204	57	218.9319	78.0980	AAL93745.1| SSU ribosomal protein S8P
					77	74	105.8766	74.0000	
**FN1631**	2.663	7.307			27		28.9763		AAL93746.1| SSU ribosomal protein S14P
					25	5	34.3755	5.0000	
**FN1632**	0.770	16.207	3.245e-4	4.639e-5	326	152	349.8618	208.2613	AAL93747.1| LSU ribosomal protein L5P
					268	213	368.5056	213.0000	
**FN1634**	-0.445	11.573			51	47	54.7330	64.3966	AAL93749.1| LSU ribosomal protein L24P
					29		39.8756		
**FN1635**	2.328	10.593	6.093e-3	3.372e-3	68	11	72.9773	15.0715	AAL93750.1| LSU ribosomal protein L14P
					75	20	103.1266	20.0000	
**FN1636**	0.556	9.799	1.266e-1	3.146e-1	29	6	31.1227	8.2208	AAL93751.1| SSU ribosomal protein S17P
					30	41	41.2506	41.0000	
**FN1637**					34		36.4887		AAL93752.1| LSU ribosomal protein L29P
					31		42.6256		
**FN1638**	2.135	14.475	1.758e-3	5.738e-4	292	73	313.3732	100.0202	AAL93753.1| LSU ribosomal protein L16P
					232	44	319.0048	44.0000	

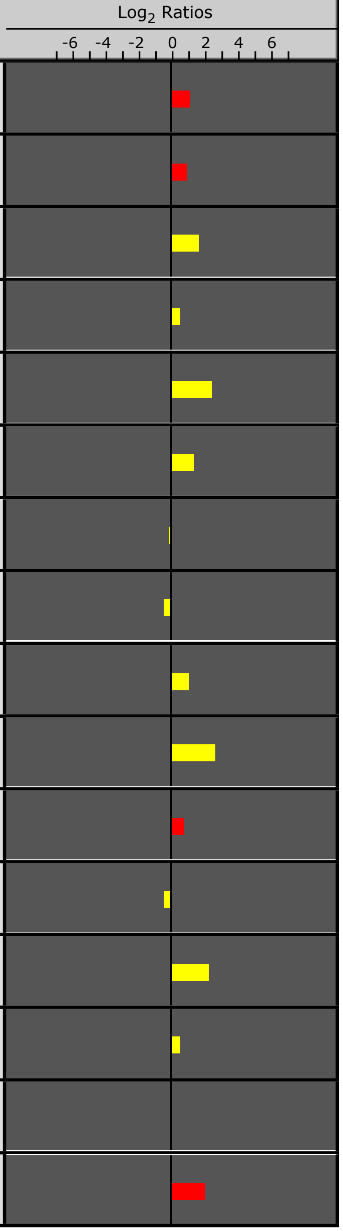

Show detected proteins only
Show all proteins
Filter by category:
GO: amino acid transport

Proteins found: 1305

Enter (or paste) list of ORFs

Find ORFs

Test	Cutoff
q-Value	.005
p-Value	

	Signif	Direction	Applies To
red	yes	+	ratios, bars
yellow	no	n/a	bars
green	yes	−	ratios, bars
pink	yes	+	*p*- , *q*-Values
light green	yes	−	

Dot Plots | Dot Plots

Protein	*FnPgSg vs FnPg* Log_2 Ratio	Log_2 Sum	*q*-Value	*p*-Value	Raw *FnPgSg*	Raw *FnPg*	Normalized *FnPgSg*	Normalized *FnPg*	Description
FN1639	0.889	19.546	1.293e-2	1.113e-2	1241	619	1331.8359	848.1166	AAL93754.1| SSU ribosomal protein S3P
					763	438	1049.1408	438.0000	
FN1640	2.300	15.503	1.902e-3	6.501e-4	380	98	407.8144	134.2737	AAL93755.1| LSU ribosomal protein L22P
					399	60	548.6333	60.0000	
FN1641	0.916	14.954	4.351e-2	6.477e-2	246	147	264.0062	201.4106	AAL93756.1| SSU ribosomal protein S19P
					164	58	225.5034	58.0000	
FN1642	0.080	15.932	2.689e-1	8.816e-1	233	285	250.0546	390.4899	AAL93757.1| LSU ribosomal protein L2P
					192	96	264.0040	96.0000	
FN1643	-0.241	13.367	2.237e-1	6.907e-1	84	131	90.1484	179.4883	AAL93758.1| LSU ribosomal protein L23P
					72	44	99.0015	44.0000	
FN1644	0.978	19.726	7.507e-3	4.746e-3	1202	594	1289.9813	813.8631	AAL93759.1| LSU ribosomal protein L1E
					963	513	1324.1450	513.0000	
FN1645	1.949	17.537	3.325e-3	1.438e-3	764	248	819.9216	339.7947	AAL93760.1| LSU ribosomal protein L3P
					650	104	893.7635	104.0000	
FN1646	0.215	15.106	1.93e-1	5.663e-1	163	180	174.9309	246.6252	AAL93761.1| SSU ribosomal protein S10P
					167	102	229.6285	102.0000	
FN1647	0.589	17.336	1.227e-1	3.002e-1	530	409	568.7937	560.3872	AAL93762.1| Hypothetical protein
					312	103	429.0065	103.0000	
FN1652	0.828	12.259	2.003e-5	6.731e-7	88	38	94.4412	52.0653	AAL93767.1| Oligopeptide-binding protein oppA
					67	53	92.1264	53.0000	
FN1654	-0.612	11.064	1.319e-1	3.35e-1	39	20	41.8546	27.4028	AAL93769.1| Hypothetical protein
					24	87	33.0005	87.0000	
FN1655	-1.076	11.732	4.52e-2	6.843e-2	39	82	41.8546	112.3515	AAL93770.1| Hypothetical cytosolic protein
					28	57	38.5006	57.0000	
FN1656					21		22.5371		AAL93771.1| SSU ribosomal protein S18P
					27		37.1256		
FN1657	-0.687	12.974	1.618e-1	4.438e-1	60	145	64.3917	198.6703	AAL93772.1| SSU ribosomal protein S6P
					56	29	77.0012	29.0000	
FN1658	1.436	14.077	1.326e-3	3.795e-4	189	59	202.8340	80.8383	AAL93773.1| Prolyl-tRNA synthetase
					167	79	229.6285	79.0000	
FN1661	0.507	10.602	6.373e-3	3.615e-3	44	22	47.2206	30.1431	AAL93776.1| Hypothetical cytosolic protein
					34	36	46.7507	36.0000	

Log_2 Ratios: -6 -4 -2 0 2 4 6

Show detected proteins only
Show all proteins
Filter by category:
GO: amino acid transport

Proteins found: 1305

Enter (or paste) list of ORFs

Find ORFs

Test	Cutoff
q-Value	.005
p-Value	

Color	Signif	Direction	Applies To
red	yes	+	ratios, bars
yellow	no	n/a	bars
green	yes	−	ratios, bars
pink	yes	+	*p*- , *q*-Values
light green	yes	−	

Dot Plots Dot Plots

Protein	FnPgSg vs FnPg Log2 Ratio	Log2 Sum	q-Value	p-Value	Raw FnPgSg	Raw FnPg	Normalized FnPgSg	Normalized FnPg	Description
FN1662	0.220	9.356	1.743e-1	4.909e-1	31	12	33.2691	16.4417	AAL93777.1| Hypothetical protein
					16	31	22.0003	31.0000	
FN1663	1.099	7.439			18		19.3175		AAL93778.1| Hypothetical protein
					14	9	19.2503	9.0000	
FN1667						3		4.1104	AAL93782.1| dTDP-glucose 4,6-dehydratase
FN1668									AAL93783.1| Cholinephosphate cytidylyltransferase
						5		5.0000	
FN1670	-0.256	9.582	8.4e-2	1.675e-1	28	23	30.0495	31.5132	AAL93785.1| Choline kinase
					15	29	20.6253	29.0000	
FN1679	-0.323	17.283	1.052e-1	2.385e-1	354	249	379.9113	341.1648	AAL93794.1| LPS biosynthesis protein WbpG
					243	552	334.1300	552.0000	
FN1683	0.896	10.706	4.808e-2	7.497e-2	68	16	72.9773	21.9222	AAL93798.1| Acetyltransferase
					28	38	38.5006	38.0000	
FN1684	-0.166	17.072	1.777e-1	5.042e-1	311	358	333.7639	490.5101	AAL93799.1| N-acetylneuraminate synthase
					267	296	367.1305	296.0000	
FN1685	-0.271	11.975	1.921e-1	5.627e-1	50	74	53.6598	101.3903	AAL93800.1| dTDP-4-dehydrorhamnose reductase
					45	38	61.8759	38.0000	
FN1686	-0.161	15.922	2.246e-1	6.942e-1	256	270	274.7381	369.9378	AAL93801.1| Spore coat polysaccharide biosynthesis protein spsF
					143	157	196.6280	157.0000	
FN1687	0.119	15.373	1.23e-1	3.015e-1	222	147	238.2495	201.4106	AAL93802.1| Gluconate 5-dehydrogenase
					139	194	191.1279	194.0000	
FN1688	-0.486	13.781	1.738e-1	4.888e-1	92	167	98.7340	228.8134	AAL93803.1| Oxidoreductase
					74	52	101.7515	52.0000	
FN1689	0.804	17.324	1.119e-3	2.917e-4	494	241	530.1587	330.2037	AAL93804.1| UDP-N-acetylglucosamine 4,6-dehydratase
					393	283	540.3831	283.0000	
FN1690	-0.975	10.093	4.943e-2	7.816e-2	26	45	27.9031	61.6563	AAL93805.1| Hypothetical protein
					14	31	19.2503	31.0000	
FN1692	0.499	10.397	1.218e-1	2.969e-1	57	21	61.1722	28.7729	AAL93807.1| Glycosyl transferase
					19	33	26.1254	33.0000	
FN1693	0.183	8.180	1.997e-1	5.95e-1	21	8	22.5371	10.9611	AAL93808.1| Hypothetical protein
					10	21	13.7502	21.0000	

Log2 Ratios: -6 -4 -2 0 2 4 6

Show detected proteins only
Show all proteins
Filter by category: GO: amino acid transport

Proteins found: 1305

Enter (or paste) list of ORFs

Find ORFs

Test	Cutoff
q-Value	.005
p-Value	

	Signif	Direction	Applies To
red	yes	+	ratios, bars
yellow	no	n/a	bars
green	yes	−	ratios, bars
pink	yes	+	p- , q-Values
light green	yes	−	

Dot Plots | Dot Plots

Protein	*FnPgSg vs FnPg* Log$_2$ Ratio	Log$_2$ Sum	*q*-Value	*p*-Value	Raw *FnPgSg*	Raw *FnPg*	Normalized *FnPgSg*	Normalized *FnPg*	Description
**FN1694**	0.870	12.342	2.391e-2	2.733e-2	88	53	94.4412	72.6174	AAL93809.1| UDP-N-acetyl-D-quinovosamine 4-epimerase
					73	34	100.3765	34.0000	
**FN1695**	1.619	9.044	2.539e-3	9.623e-4	43	6	46.1474	8.2208	AAL93810.1| Probable quinovosaminephosphotransferae
					25	18	34.3755	18.0000	
**FN1696**	0.020	14.703	2.774e-1	9.202e-1	163	93	174.9309	127.4230	AAL93811.1| UDP-N-acetylglucosamine 4,6-dehydratase
					112	197	154.0023	197.0000	
**FN1697**	-0.155	12.913	2.349e-1	7.36e-1	68	36	72.9773	49.3250	AAL93812.1| Hypothetical protein
					68	136	93.5014	136.0000	
**FN1698**	-0.733	13.739	5.955e-2	1.036e-1	78	77	83.7093	105.5008	AAL93813.1| dTDP-4-dehydrorhamnose reductase
					71	196	97.6265	196.0000	
**FN1701**	-0.358	10.081	5.329e-2	8.782e-2	26	23	27.9031	31.5132	AAL93816.1| ABC transporter ATP-binding protein
					22	43	30.2505	43.0000	
**FN1703**	-0.779	13.578	1.007e-1	2.219e-1	74	56	79.4165	76.7278	AAL93818.1| ADP-L-glycero-D-manno-heptose-6-epimerase
					65	213	89.3763	213.0000	
**FN1704**	-0.299	6.117			7	4	7.5124	5.4806	AAL93819.1| Serine protease
						13		13.0000	
**FN1708**	0.356	17.781	5.334e-4	9.817e-5	488	294	523.7195	402.8211	AAL93823.1| Polyribonucleotide nucleotidyltransferase
					400	436	550.0083	436.0000	
**FN1711**					3		3.2196		AAL93826.1| Methyltransferase
					3		4.1251		
**FN1713**									AAL93828.1| tRNA (Uracil-5-) -methyltransferase
						11		11.0000	
**FN1715**									AAL93830.1| ATPase
						3		3.0000	
**FN1717**	-0.404	7.988	1.247e-1	3.078e-1	13	18	13.9515	24.6625	AAL93832.1| NAD-dependent DNA ligase
					10	12	13.7502	12.0000	
**FN1718**	0.130	17.029	1.095e-1	2.543e-1	372	284	399.2288	389.1197	AAL93833.1| Protein translocase subunit secA
					266	310	365.7555	310.0000	
**FN1719**	-0.176	16.248	7.711e-2	1.479e-1	261	238	280.1041	326.0933	AAL93834.1| Hypothetical protein
					178	267	244.7537	267.0000	
**FN1723**	0.619	12.380	8.064e-3	5.4e-3	93	40	99.8072	54.8056	AAL93838.1| Glucose inhibited division protein A
					59	63	81.1262	63.0000	

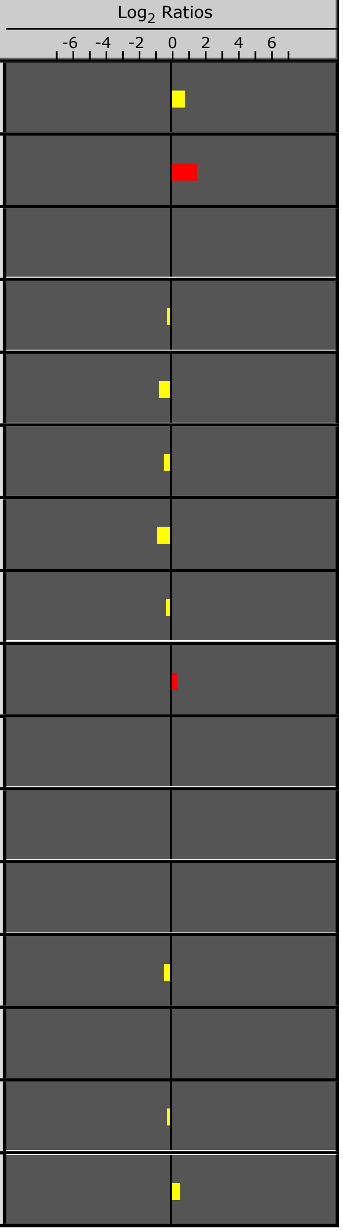

Show detected proteins only
Show all proteins
Filter by category:
GO: amino acid transport

Proteins found: 1305

Enter (or paste) list of ORFs

Find ORFs

Test	Cutoff
q-Value	.005
p-Value	

	Signif	Direction	Applies To
red	yes	+	ratios, bars
yellow	no	n/a	bars
green	yes	–	ratios, bars
pink	yes	+	*p*- , *q*-Values
light green	yes	–	*p*- , *q*-Values

Dot Plots Dot Plots

Protein	FnPgSg vs FnPg: Log_2 Ratio	Log_2 Sum	*q*-Value	*p*-Value	Raw *FnPgSg*	Raw *FnPg*	Normalized *FnPgSg*	Normalized *FnPg*	Description
**FN1724**	-1.154	7.479	1.061e-1	2.421e-1	9	5	9.6588	6.8507	AAL93839.1| Potassium uptake protein KtrA
					6	33	8.2501	33.0000	
**FN1727**					4		4.2928		AAL93842.1| Chloride channel protein
					4		5.5001		
**FN1728**	-1.508	6.735	8.793e-5	6.524e-6	5	13	5.3660	17.8118	AAL93843.1| Pyrrolidone-carboxylate peptidase
					5	17	6.8751	17.0000	
**FN1730**	-0.688	6.777				7		9.5910	AAL93845.1| Para-aminobenzoate synthase component I
					6	17	8.2501	17.0000	
**FN1731**	0.965	7.239			16	7	17.1711	9.5910	AAL93846.1| Anthranilate synthase component II
						8		8.0000	
**FN1732**	-1.246	10.006	5.457e-2	9.121e-2	26	21	27.9031	28.7729	AAL93847.1| Hypothetical protein
					10	70	13.7502	70.0000	
**FN1733**									AAL93848.1| V-type sodium ATP synthase subunit D
						8		8.0000	
**FN1734**	-0.129	14.167	1.446e-1	3.821e-1	102	99	109.4660	135.6438	AAL93849.1| V-type sodium ATP synthase subunit B
					109	148	149.8773	148.0000	
**FN1735**	-1.290	12.238	5.271e-2	8.632e-2	38	47	40.7814	64.3966	AAL93850.1| V-type sodium ATP synthase subunit A
					35	153	48.1257	153.0000	
**FN1736**	-1.821	8.019	2.54e-2	2.981e-2	7	15	7.5124	20.5521	AAL93851.1| V-type sodium ATP synthase subunit A
					7	40	9.6251	40.0000	
**FN1737**						3		4.1104	AAL93852.1| V-type sodium ATP synthase subunit G
						6		6.0000	
**FN1738**	-2.659	10.305	3.368e-2	4.519e-2	11	94	11.8052	128.7931	AAL93853.1| V-type sodium ATP synthase subunit C
					12	50	16.5002	50.0000	
**FN1739**	1.155	7.194	8.725e-7	7.771e-9	17	6	18.2443	8.2208	AAL93854.1| V-type sodium ATP synthase subunit E
					13	8	17.8753	8.0000	
**FN1740**									AAL93855.1| V-type sodium ATP synthase subunit K
						185		185.0000	
**FN1741**	-2.087	7.840	2.797e-3	1.105e-3	6	20	6.4392	27.4028	AAL93856.1| V-type sodium ATP synthase subunit I
					6	35	8.2501	35.0000	
**FN1742**						9		12.3313	AAL93857.1| V-type sodium ATP synthase subunit G
						8		8.0000	

Log_2 Ratios: -6 -4 -2 0 2 4 6

Show detected proteins only
Show all proteins
Filter by category:
GO: amino acid transport

Proteins found: 1305

Enter (or paste) list of ORFs

Find ORFs

Test	Cutoff
q-Value	.005
p-Value	

	Signif	Direction	Applies To
red	yes	+	ratios, bars
yellow	no	n/a	bars
green	yes	−	ratios, bars
pink	yes	+	*p*- , *q*-Values
light green	yes	−	*p*- , *q*-Values

Dot Plots | Dot Plots

Protein	*FnPgSg vs FnPg* Log$_2$ Ratio	Log$_2$ Sum	*q*-Value	*p*-Value	Raw *FnPgSg*	Raw *FnPg*	Normalized *FnPgSg*	Normalized *FnPg*	Description
FN1743									AAL93858.1| Multidrug-efflux transporter 2 regulator
						8		8.0000	
FN1745	-1.461	7.709			6		6.4392		AAL93860.1| Cystathionine gamma-synthase
					8	24	11.0002	24.0000	
FN1746									AAL93861.1| Cystathionine beta-lyase
						7		7.0000	
FN1752					3		3.2196		AAL93867.1| Regulatory protein TENI
FN1754						11		15.0715	AAL93869.1| Thiazole biosynthesis protein thiG
FN1763	-2.203	9.765				34		46.5848	AAL93876.1| Hypothetical cytosolic protein
					10	80	13.7502	80.0000	
FN1764	-0.410	23.624	1.963e-1	5.803e-1	2954	926	3170.2203	1268.7495	AAL93877.1| Enolase
					2231	7022	3067.6712	7022.0000	
FN1765	0.411	18.283	1.14e-1	2.698e-1	563	207	604.2092	283.6189	AAL93878.1| Pyruvate kinase
					508	696	698.5105	696.0000	
FN1780	1.215	8.485	1.004e-2	7.598e-3	23	5	24.6835	6.8507	AAL93879.1| Hypothetical protein
					24	18	33.0005	18.0000	
FN1781	0.121	17.972	1.867e-1	5.402e-1	496	433	532.3051	593.2706	AAL93880.1| LytB protein
					382	379	525.2579	379.0000	
FN1783									AAL93882.1| Ethanolamine utilization protein eutJ
						11		11.0000	
FN1784	-0.541	6.103							AAL93883.1| unknown
					5	10	6.8751	10.0000	
FN1785	1.793	9.194			25		26.8299		AAL93884.1| Hypothetical protein
					46	13	63.2510	13.0000	
FN1786	1.290	10.539	1.576e-2	1.535e-2	56	9	60.0990	12.3313	AAL93885.1| ADP-heptose synthase
					44	37	60.5009	37.0000	
FN1787	1.097	9.179	1.653e-2	1.635e-2	40	16	42.9278	21.9222	AAL93886.1| Tetratricopeptide repeat family protein
					20	11	27.5004	11.0000	
FN1788	0.044	9.299	2.706e-1	8.895e-1	27	12	28.9763	16.4417	AAL93887.1| 2C-methyl-D-erythritol 2,4-cyclodiphosphate synthase
					16	33	22.0003	33.0000	

Log$_2$ Ratios: -6 -4 -2 0 2 4 6

Show detected proteins only
Show all proteins
Filter by category:
GO: amino acid transport

Proteins found: 1305

Enter (or paste) list of ORFs

Find ORFs

Test	Cutoff
q-Value	.005
p-Value	

	Signif	Direction	Applies To
red	yes	+	ratios, bars
yellow	no	n/a	bars
green	yes	−	ratios, bars
pink	yes	+	*p*- , *q*-Values
light green	yes	−	

Dot Plots | Dot Plots

Protein	FnPgSg vs FnPg: Log_2 Ratio	Log_2 Sum	*q*-Value	*p*-Value	Raw *FnPgSg*	Raw *FnPg*	Normalized *FnPgSg*	Normalized *FnPg*	Description
FN1790	-1.822	8.430	1.002e-1	2.2e-1	12	5	12.8783	6.8507	AAL93889.1| Cob(I)alamin adenosyltransferase
					5	63	6.8751	63.0000	
FN1792	1.318	25.036	7.919e-3	5.222e-3	8681	3669	9316.4123	5027.0431	AAL93891.1| Hypothetical protein
					6695	2401	9205.7638	2401.0000	
FN1793	-0.993	14.177	1.286e-1	3.223e-1	85	38	91.2216	52.0653	AAL93892.1| Phosphoenolpyruvate-protein phosphotransferase
					74	332	101.7515	332.0000	
FN1794	1.911	12.582	1.253e-2	1.062e-2	110	29	118.0515	39.7341	AAL93893.1| Phosphocarrier protein HPr
					135	41	185.6278	41.0000	
FN1797	-0.700	10.522	9.57e-2	2.044e-1	33	21	35.4155	28.7729	AAL93896.1| Spermidine/putrescine transport ATP-binding protein potA
					18	69	24.7504	69.0000	
FN1798	-0.744	5.900			6		6.4392		AAL93897.1| Spermidine/putrescine transport system permease protein potB
					4	10	5.5001	10.0000	
FN1800	0.853	13.964	4.957e-3	2.494e-3	177	57	189.9556	78.0980	AAL93899.1| Peptidyl-prolyl cis-trans isomerase
					109	110	149.8773	110.0000	
FN1801	-1.949	7.767			7		7.5124		AAL93900.1| Sodium/glutamate symport carrier protein
						29		29.0000	
FN1803					22		23.6103		AAL93902.1| Transcriptional regulator, TetR family
FN1804									AAL93903.1| Aminoacyl-histidine dipeptidase
						22		22.0000	
FN1807	0.435	17.149	1.233e-3	3.388e-4	407	251	436.7907	343.9051	AAL93906.1| Hypothetical protein
					327	312	449.6318	312.0000	
FN1808	1.442	8.612			30		32.1959		AAL93907.1| Hypothetical protein
					24	12	33.0005	12.0000	
FN1809	0.210	9.201	2.412e-1	7.621e-1	23	3	24.6835	4.1104	AAL93908.1| Iron/zinc/copper-binding protein
					20	41	27.5004	41.0000	
FN1811	0.301	9.176	1.695e-1	4.725e-1	19	9	20.3907	12.3313	AAL93910.1| Manganese transport system ATP-binding protein mntA
					24	31	33.0005	31.0000	
FN1812	-0.120	13.739	2.616e-1	8.492e-1	95	148	101.9536	202.7807	AAL93911.1| Manganese-binding protein
					89	41	122.3768	41.0000	
FN1813	0.809	7.453			16		17.1711		AAL93912.1| Manganese-binding protein
					13	10	17.8753	10.0000	

Log_2 Ratios: -6 -4 -2 0 2 4 6

Show detected proteins only
Show all proteins
Filter by category:
GO: amino acid transport

Proteins found: 1305

Enter (or paste) list of ORFs

Find ORFs

Test	Cutoff
q-Value	.005
p-Value	

Color	Signif	Direction	Applies To
red	yes	+	ratios, bars
yellow	no	n/a	bars
green	yes	−	ratios, bars
pink	yes	+	*p*- , *q*-Values
light green	yes	−	

Dot Plots Dot Plots

Protein	FnPgSg vs FnPg Log$_2$ Ratio	Log$_2$ Sum	*q*-Value	*p*-Value	Raw *FnPgSg*	Raw *FnPg*	Normalized *FnPgSg*	Normalized *FnPg*	Description
FN1814					18		19.3175		AAL93913.1| Hypothetical protein
					5		6.8751		
FN1816	-0.398	6.092	4.697e-2	7.241e-2	7	8	7.5124	10.9611	AAL93915.1| unknown
					5	8	6.8751	8.0000	
FN1817	0.803	5.999	3.244e-2	4.268e-2	12	3	12.8783	4.1104	AAL93916.1| Hemolysin
					6	8	8.2501	8.0000	
FN1825					10		10.7320		AAL93924.1| Hypothetical protein
					7		9.6251		
FN1826	0.832	10.592	3.215e-3	1.363e-3	49	24	52.5866	32.8834	AAL93925.1| Protease
					38	26	52.2508	26.0000	
FN1827	-0.438	10.598	7.193e-2	1.341e-1	31	26	33.2691	35.6236	AAL93926.1| Replicative DNA helicase
					25	56	34.3755	56.0000	
FN1828	-0.942	11.784	1.199e-1	2.902e-1	35	99	37.5619	135.6438	AAL93927.1| LSU ribosomal protein L9P
					35	29	48.1257	29.0000	
FN1830	-1.284	9.311	4.641e-2	7.113e-2	16	18	17.1711	24.6625	AAL93929.1| DNA polymerase III subunits gamma and tau
					11	54	15.1252	54.0000	
FN1831	-1.301	11.892	9.341e-4	2.159e-4	45	77	48.2938	105.5008	AAL93930.1| Nitrogen assimilation regulatory protein
					22	88	30.2505	88.0000	
FN1832					4		4.2928		AAL93931.1| TonB protein
FN1833	0.448	8.623			24		25.7567		AAL93932.1| Biopolymer transport exbD protein
					15	17	20.6253	17.0000	
FN1834	0.395	9.960	3.835e-2	5.428e-2	29	19	31.1227	26.0327	AAL93933.1| Biopolymer transport exbB protein
					30	29	41.2506	29.0000	
FN1836	0.846	11.849	6.353e-3	3.598e-3	80	26	85.8557	35.6236	AAL93935.1| Tetratricopeptide repeat family protein
					56	55	77.0012	55.0000	
FN1838	-1.439	8.561			11		11.8052		AAL93937.1| Glycerol uptake facilitator protein
						32		32.0000	
FN1839	-0.907	17.801	7.273e-2	1.362e-1	284	286	304.7876	391.8600	AAL93938.1| Glycerol kinase
					286	917	393.2559	917.0000	
FN1840	0.195	13.008	8.779e-2	1.789e-1	90	53	96.5876	72.6174	AAL93939.1| Dihydroxyacetone kinase
					71	97	97.6265	97.0000	

Log$_2$ Ratios: -6 -4 -2 0 2 4 6

Show detected proteins only
Show all proteins
Filter by category:
GO: amino acid transport

Proteins found: 1305

Enter (or paste) list of ORFs

Find ORFs

Test	Cutoff
q-Value	.005
p-Value	

	Signif	Direction	Applies To
red	yes	+	ratios, bars
yellow	no	n/a	bars
green	yes	−	ratios, bars
pink	yes	+	*p*-, *q*-Values
light green	yes	−	

Dot Plots Dot Plots

Protein	FnPgSg vs FnPg Log$_2$ Ratio	Log$_2$ Sum	*q*-Value	*p*-Value	Raw *FnPgSg*	Raw *FnPg*	Normalized *FnPgSg*	Normalized *FnPg*	Description
**FN1841**	-0.334	8.117	1.795e-1	5.11e-1	11	20	11.8052	27.4028	AAL93940.1| Dihydroxyacetone kinase
					13	10	17.8753	10.0000	
**FN1842**	1.112	10.308	5.622e-3	2.987e-3	54	12	57.9526	16.4417	AAL93941.1| Dihydroxyacetone kinase phosphotransfer protein
					34	32	46.7507	32.0000	
**FN1843**						3		4.1104	AAL93942.1| Surface antigen
**FN1844**									AAL93943.1| Ketoacyl reductase hetN
						5		5.0000	
**FN1847**						5		6.8507	AAL93946.1| DTDP-4-dehydrorhamnose 3,5-epimerase
						24		24.0000	
**FN1848**	0.138	4.781							AAL93947.1| Metal dependent hydrolase
					4	5	5.5001	5.0000	
**FN1849**	-2.483	6.687			4		4.2928		AAL93948.1| Coenzyme F390 synthetase
						24		24.0000	
**FN1850**									AAL93949.1| 3-oxoacyl-[acyl-carrier-protein] synthase III
						48		48.0000	
**FN1851**	-1.047	12.055	1.144e-1	2.709e-1	41	23	44.0010	31.5132	AAL93950.1| Ribonuclease PH
					34	156	46.7507	156.0000	
**FN1852**	0.471	10.512	6.999e-2	1.291e-1	39	16	41.8546	21.9222	AAL93951.1| unknown
					35	43	48.1257	43.0000	
**FN1853**	-1.649	6.877	4.563e-2	6.937e-2	5	20	5.3660	27.4028	AAL93952.1| Methylaspartate mutase
					5	11	6.8751	11.0000	
**FN1854**	-0.352	10.114	8.161e-2	1.605e-1	28	33	30.0495	45.2146	AAL93953.1| Methylaspartate mutase
					21	30	28.8754	30.0000	
**FN1855**									AAL93954.1| Methylaspartate mutase
						4		4.0000	
**FN1856**	-0.081	23.136	1.603e-1	4.384e-1	2988	2099	3206.7089	2875.9235	AAL93955.1| Butyrate-acetoacetate CoA-transferase subunit B
					1963	3369	2699.1657	3369.0000	
**FN1857**	-0.611	19.981	3.737e-3	1.711e-3	687	897	737.2855	1229.0154	AAL93956.1| Acetoacetate: butyrate/acetate coenzyme A transferase
					661	1285	908.8887	1285.0000	
**FN1858**	1.480	12.203			92	30	98.7340	41.1042	AAL93957.1| Short-chain fatty acids transporter
					95		130.6270		

Log$_2$ Ratios: -6 -4 -2 0 2 4 6

Show detected proteins only
Show all proteins
Filter by category:
GO: amino acid transport

Proteins found: 1305

Enter (or paste) list of ORFs

Find ORFs

Test	Cutoff
q-Value	.005
p-Value	

	Signif	Direction	Applies To
red	yes	+	ratios, bars
yellow	no	n/a	bars
green	yes	−	ratios, bars
pink	yes	+	*p*-, *q*-Values
light green	yes	−	

Dot Plots Dot Plots

Protein	*FnPgSg vs FnPg* Log_2 Ratio	Log_2 Sum	*q*-Value	*p*-Value	Raw *FnPgSg*	Raw *FnPg*	Normalized *FnPgSg*	Normalized *FnPg*	Description
**FN1859**	-1.872	22.626	1.071e-1	2.46e-1	1120	443	1201.9792	606.9720	AAL93958.1| Major outer membrane protein
					1060	9128	1457.5220	9128.0000	
**FN1860**	-0.814	11.403			27		28.9763		AAL93959.1| NA+/H+ antiporter NHAC
					36	69	49.5007	69.0000	
**FN1862**	0.128	15.880	2.215e-1	6.821e-1	249	232	267.2257	317.8724	AAL93961.1| L-beta-lysine 5,6-aminomutase beta subunit
					179	152	246.1287	152.0000	
**FN1863**	-0.463	18.754	1.856e-1	5.357e-1	483	957	518.3535	1311.2238	AAL93962.1| L-beta-lysine 5,6-aminomutase alpha subunit
					447	250	614.6343	250.0000	
**FN1864**	0.671	8.212	1.062e-1	2.425e-1	20	17	21.4639	23.2924	AAL93963.1| DNA mismatch repair protein mutS
					16	4	22.0003	4.0000	
**FN1865**					3		3.2196		AAL93964.1| Hypothetical protein
**FN1866**	0.438	20.860	6.768e-2	1.233e-1	1420	1117	1523.9380	1530.4462	AAL93965.1| Lysine 2,3-aminomutase
					1227	840	1687.1504	840.0000	
**FN1867**	0.197	20.209	1.97e-1	5.833e-1	1165	1058	1250.2731	1449.6080	AAL93966.1| Zn-dependent alcohol dehydrogenases and related dehydrogenases
					805	607	1106.8917	607.0000	
**FN1868**	0.761	14.237	1.486e-2	1.39e-2	168	96	180.2969	131.5334	AAL93967.1| Hypothetical cytosolic protein
					132	82	181.5027	82.0000	
**FN1869**	0.057	14.998	2.608e-1	8.458e-1	167	172	179.2237	235.6641	AAL93968.1| Hypothetical protein
					138	119	189.7529	119.0000	
**FN1870**									AAL93969.1| unknown
					3		4.1251		
**FN1873**	0.956	11.028	6.534e-3	3.762e-3	52	26	55.8062	35.6236	AAL93972.1| Bis(5'-nucleosyl)-tetraphosphatase
					52	30	71.5011	30.0000	
**FN1874**	0.311	8.979	1.413e-1	3.702e-1	21	9	22.5371	12.3313	AAL93973.1| Ribose 5-phosphate isomerase
					20	28	27.5004	28.0000	
**FN1875**	-0.098	14.092	1.804e-1	5.147e-1	133	112	142.7350	153.4557	AAL93974.1| Peptidyl-prolyl cis-trans isomerase
					82	120	112.7517	120.0000	
**FN1877**	0.324	5.494			7		7.5124		AAL93976.1| Guanine-hypoxanthine permease
						6		6.0000	
**FN1878**					5		5.3660		AAL93977.1| unknown

Log₂ Ratios: -6 -4 -2 0 2 4 6

- Show detected proteins only
- Show all proteins
- Filter by category: GO: amino acid transport

Proteins found: 1305

Enter (or paste) list of ORFs

Find ORFs

Test	Cutoff
q-Value	.005
p-Value	

	Signif	Direction	Applies To
red	yes	+	ratios, bars
yellow	no	n/a	bars
green	yes	−	ratios, bars
pink	yes	+	*p*-, *q*-Values
light green	yes	−	

Dot Plots | Dot Plots

Protein	FnPgSg vs FnPg: Log_2 Ratio	Log_2 Sum	*q*-Value	*p*-Value	Raw: *FnPgSg*	Raw: *FnPg*	Normalized: *FnPgSg*	Normalized: *FnPg*	Description
FN1879					25		26.8299		AAL93978.1| SSU ribosomal protein S20P
					23		31.6255		
FN1880	-1.546	13.050	1.109e-3	2.871e-4	62	118	66.5381	161.6765	AAL93979.1| Oxygen-insensitive NAD(P)H nitroreductase
					30	153	41.2506	153.0000	
FN1881	-0.635	6.009			6		6.4392		AAL93980.1| Esterase
						10		10.0000	
FN1884	1.301	12.209			77	32	82.6361	43.8445	AAL93983.1| unknown
					97		133.3770		
FN1890	-0.572	9.255			16	22	17.1711	30.1431	AAL93989.1| Hypothetical protein
					17		23.3754		
FN1891	-0.788	8.512	1.377e-1	3.577e-1	13	6	13.9515	8.2208	AAL93990.1| Glycerophosphoryl diester phosphodiesterase
					11	42	15.1252	42.0000	
FN1893	-0.469	17.642	1.688e-2	1.682e-2	309	389	331.6175	532.9844	AAL93991.1| Fusobacterium outer membrane protein family
					318	531	437.2566	531.0000	
FN1895					5		5.3660		AAL93994.1| Hypothetical protein
FN1898	0.557	9.976	1.009e-1	2.226e-1	32	9	34.3423	12.3313	AAL93997.1| Sugar transport ATP-binding protein
					31	40	42.6256	40.0000	
FN1899	-0.695	14.785	3.412e-2	4.611e-2	109	123	116.9783	168.5272	AAL93998.1| Hypothetical lipoprotein
					107	259	147.1272	259.0000	
FN1902	-0.186	9.886	1.019e-1	2.263e-1	23	26	24.6835	35.6236	AAL94001.1| Deoxycytidylate deaminase
					24	30	33.0005	30.0000	
FN1903	3.357	10.898	1.618e-4	1.943e-5	126	17	135.2227	23.2924	AAL94002.1| Coenzyme A disulfide reductase/ disulfide bond regulator domain
					105	4	144.3772	4.0000	
FN1905					3		3.2196		AAL94004.1| outer membrane protein
FN1906	-0.593	17.157	9.311e-2	1.958e-1	320	222	343.4226	304.1710	AAL94005.1| Cytosol aminopeptidase
					203	635	279.1292	635.0000	
FN1908	-0.756	16.023	1.28e-2	1.097e-2	192	213	206.0536	291.8398	AAL94007.1| Glycerophosphoryl diester phosphodiesterase
					139	379	191.1279	379.0000	
FN1909	0.723	13.471	8.19e-3	5.559e-3	127	51	136.2959	69.8771	AAL94008.1| UDP-3-O-[3-hydroxymyristoyl] glucosamine N-acyltransferase
					100	96	137.5021	96.0000	

Log_2 Ratios: -6 -4 -2 0 2 4 6

Show detected proteins only
Show all proteins
Filter by category:
GO: amino acid transport

Proteins found: 1305

Enter (or paste) list of ORFs

Find ORFs

Test	Cutoff
q-Value	.005
p-Value	

	Signif	Direction	Applies To
red	yes	+	ratios, bars
yellow	no	n/a	bars
green	yes	−	ratios, bars
pink	yes	+	*p*-, *q*-Values
light green	yes	−	

Dot Plots Dot Plots

Protein	*FnPgSg vs FnPg* Log$_2$ Ratio	Log$_2$ Sum	*q*-Value	*p*-Value	Raw *FnPgSg*	Raw *FnPg*	Normalized *FnPgSg*	Normalized *FnPg*	Description
**FN1910**	1.607	16.441	1.193e-4	1.237e-5	454	148	487.2309	202.7807	AAL94009.1| periplasmic protein
					403	139	554.1334	139.0000	
**FN1911**	-0.434	21.749	3.584e-2	4.941e-2	1561	1822	1675.2586	2496.3948	AAL94010.1| Outer membrane protein
					1131	1869	1555.1484	1869.0000	
**FN1912**	-0.955	5.874				9		12.3313	AAL94011.1| Hypothetical protein
					4	9	5.5001	9.0000	
**FN1913**	-0.294	11.654	1.722e-1	4.826e-1	43	64	46.1474	87.6890	AAL94012.1| hydrolase (HD superfamily)
					41	38	56.3758	38.0000	
**FN1914**	-1.278	13.356	1.248e-1	3.081e-1	70	213	75.1237	291.8398	AAL94013.1| Anti-sigma F factor antagonist
					41	27	56.3758	27.0000	
**FN1917**									AAL94016.1| tRNA delta(2)-isopentenylpyrophosphate transferase
						6		6.0000	
**FN1918**	0.846	11.987	3.456e-2	4.702e-2	90	19	96.5876	26.0327	AAL94017.1| SPO0B-associated GTP-binding protein
					54	69	74.2511	69.0000	
**FN1919**	0.091	5.897	2.678e-1	8.767e-1	11	8	11.8052	10.9611	AAL94018.1| Methyltransferase
					3	4	4.1251	4.0000	
**FN1920**						7		9.5910	AAL94019.1| tRNA (5-methylaminomethyl -2-thiouridylate) -methyltransferase
**FN1922**	-0.975	7.064				12		16.4417	AAL94021.1| Hypothetical protein
					6	16	8.2501	16.0000	
**FN1925**									AAL94024.1| Arsenical pump membrane protein
						6		6.0000	
**FN1926**	-0.291	12.422	1.894e-1	5.513e-1	71	32	76.1969	43.8445	AAL94025.1| Nitrogen regulatory IIA protein
					42	120	57.7509	120.0000	
**FN1927**	-1.243	13.796	5.654e-2	9.618e-2	74	78	79.4165	106.8709	AAL94026.1| DEGV protein
					55	260	75.6261	260.0000	
**FN1928**	-0.908	8.707			15		16.0979		AAL94027.1| Transcriptional regulator, MerR family
					10	28	13.7502	28.0000	
**FN1929**	-0.666	10.168	1.428e-1	3.756e-1	22	12	23.6103	16.4417	AAL94028.1| Competence-damage protein cinA
					22	69	30.2505	69.0000	
**FN1931**									AAL94030.1| Protease
						7		7.0000	

Show detected proteins only
Show all proteins
Filter by category:
GO: amino acid transport

Proteins found: 1305

Enter (or paste) list of ORFs

Find ORFs

Test	Cutoff
q-Value	.005
p-Value	

Signif	Direction	Applies To
yes	+	ratios, bars
no	n/a	bars
yes	−	ratios, bars
yes	+	*p*- , *q*-Values
yes	−	

Dot Plots | Dot Plots

Protein	*FnPgSg vs FnPg* Log_2 Ratio	Log_2 Sum	*q*-Value	*p*-Value	Raw *FnPgSg*	Raw *FnPg*	Normalized *FnPgSg*	Normalized *FnPg*	Description
FN1933	1.040	6.623	9.028e-3	6.443e-3	15	5	16.0979	6.8507	AAL94032.1| Hypothetical protein
					9	7	12.3752	7.0000	
FN1935	-0.898	4.272			3		3.2196		AAL94034.1| Adenine-specific methyltransferase
						6		6.0000	
FN1939	-0.699	7.797			9		9.6588		AAL94038.1| Hypothetical protein
					10	19	13.7502	19.0000	
FN1941	-1.674	14.958	4.273e-6	1.14e-7	99	239	106.2464	327.4634	AAL94040.1| ClpB protein
					68	310	93.5014	310.0000	
FN1942						8		10.9611	AAL94041.1| putative DNA-binding protein
FN1943	-2.422	22.014	5.062e-3	2.568e-3	828	4000	888.6061	5480.5594	AAL94042.1| Tryptophanase
					647	4049	889.6384	4049.0000	
FN1948									AAL94044.1| Hypothetical protein
						7		7.0000	
FN1949	-3.735	7.824				24		32.8834	AAL94045.1| Xaa-Pro dipeptidase
					3	77	4.1251	77.0000	
FN1950									AAL94046.1| Serine protease
					4		5.5001		
FN1956									AAL94052.1| Hypothetical protein
						15		15.0000	
FN1964	-0.196	8.237	1.749e-1	4.93e-1	20	14	21.4639	19.1820	AAL94054.1| O-linked GLCNAC transferase
					8	18	11.0002	18.0000	
FN1965	-0.230	11.179	1.057e-1	2.403e-1	47	44	50.4402	60.2862	AAL94055.1| Tetratricopeptide repeat family protein
					28	44	38.5006	44.0000	
FN1966	0.792	10.885	9.561e-3	7.033e-3	49	30	52.5866	41.1042	AAL94056.1| Hypothetical protein
					45	25	61.8759	25.0000	
FN1970					14		15.0247		AAL94060.1| Hemin-binding periplasmic protein hmuT precursor
FN1971	-0.670	9.749			19		20.3907		AAL94061.1| Hemin receptor
					19	37	26.1254	37.0000	
FN1972	-2.046	9.816			16		17.1711		AAL94062.1| unknown
					9	61	12.3752	61.0000	

Log_2 Ratios: -6 -4 -2 0 2 4 6

Show detected proteins only
Show all proteins
Filter by category:
GO: amino acid transport

Proteins found: 1305

Enter (or paste) list of ORFs

Find ORFs

Test	Cutoff
q-Value	.005
p-Value	

	Signif	Direction	Applies To
red	yes	+	ratios, bars
yellow	no	n/a	bars
green	yes	−	ratios, bars
pink	yes	+	*p*- , *q*-Values
light green	yes	−	

Dot Plots Dot Plots

Protein	FnPgSg vs FnPg Log$_2$ Ratio	Log$_2$ Sum	q-Value	p-Value	Raw FnPgSg	Raw FnPg	Normalized FnPgSg	Normalized FnPg	Description
FN1973	0.430	14.638	8.101e-3	5.447e-3	166	111	178.1505	152.0855	AAL94063.1| Translation initiation inhibitor
					140	123	192.5029	123.0000	
FN1975	0.746	13.048	2.137e-3	7.62e-4	103	49	110.5392	67.1369	AAL94065.1| ATP-dependent RNA helicase
					93	75	127.8769	75.0000	
FN1976	-0.403	9.223	2.016e-1	6.031e-1	14	6	15.0247	8.2208	AAL94066.1| 4-amino-4-deoxychorismate lyase
					20	48	27.5004	48.0000	
FN1977	-1.160	5.364			4	7	4.2928	9.5910	AAL94067.1| Cell cycle protein MesJ
FN1978	1.336	13.142	1.571e-2	1.528e-2	129	18	138.4423	24.6625	AAL94068.1| Cell division protein ftsH
					119	95	163.6275	95.0000	
FN1979	4.204	11.772	2.512e-3	9.481e-4	204	15	218.9319	20.5521	AAL94069.1| SSU ribosomal protein S15P
					210	7	288.7544	7.0000	
FN1980									AAL94070.1| Transporter
					3		4.1251		
FN1983	1.624	17.136	1.102e-3	2.838e-4	564	139	605.2824	190.4494	AAL94073.1| Alkyl hydroperoxide reductase C22 protein
					529	242	727.3860	242.0000	
FN1984	0.309	17.296	1.223e-1	2.988e-1	376	348	403.5216	476.8087	AAL94074.1| Thioredoxin reductase
					356	244	489.5074	244.0000	
FN1985	-0.134	11.973	2.613e-1	8.482e-1	50	13	53.6598	17.8118	AAL94075.1| Inner membrane protein
					49	115	67.3760	115.0000	
FN1986	-0.356	16.065	6.746e-2	1.227e-1	234	255	251.1278	349.3857	AAL94076.1| Hypothetical protein
					154	243	211.7532	243.0000	
FN1987	-2.667	7.160			5	22	5.3660	30.1431	AAL94077.1| Transcriptional regulator, GntR family
					3		4.1251		
FN1988	-2.609	16.039	4.636e-2	7.101e-2	92	221	98.7340	302.8009	AAL94078.1| Tyrosine phenol-lyase
					81	979	111.3767	979.0000	
FN1989									AAL94079.1| Sodium-dependent tyrosine transporter
						16		16.0000	
FN1991	-0.159	17.343	3.886e-2	5.53e-2	349	334	374.5453	457.6267	AAL94081.1| Glucosamine-1-phosphate acetyltransferase
					289	404	397.3810	404.0000	
FN1992	-0.845	16.012	1.1e-2	8.775e-3	173	219	185.6629	300.0606	AAL94082.1| Ribose-phosphate pyrophosphokinase
					144	389	198.0030	389.0000	

Log$_2$ Ratios: -6 -4 -2 0 2 4 6

Show detected proteins only
Show all proteins
Filter by category:
GO: amino acid transport

Proteins found: 1305

Enter (or paste) list of ORFs

Find ORFs

Test	Cutoff
q-Value	.005
p-Value	

Signif	Direction	Applies To
yes	+	ratios, bars
no	n/a	bars
yes	−	ratios, bars
yes	+	p- , q-Values
yes	−	

Dot Plots Dot Plots

Protein	*FnPgSg vs FnPg* Log$_2$ Ratio	Log$_2$ Sum	*q*-Value	*p*-Value	Raw *FnPgSg*	Raw *FnPg*	Normalized *FnPgSg*	Normalized *FnPg*	Description
**FN1993**	0.196	5.366							AAL94083.1| SUA5 protein
					5	6	6.8751	6.0000	
**FN1994**	-0.235	11.134	2.212e-1	6.809e-1	43	59	46.1474	80.8383	AAL94084.1| Hypothetical protein
					30	22	41.2506	22.0000	
**FN1995**	2.161	6.161			9		9.6588		AAL94085.1| Hypothetical protein
					19	4	26.1254	4.0000	
**FN2001**									AAL94091.1| Hypothetical protein
						20		20.0000	
**FN2007**	-1.027	6.324	1.069e-1	2.45e-1	4	15	4.2928	20.5521	AAL94097.1| Glutathione peroxidase
					6	5	8.2501	5.0000	
**FN2008**									AAL94098.1| Glycine betaine transport ATP-binding protein
						19		19.0000	
**FN2009**									AAL94099.1| Glycine betaine transport system permease protein
						8		8.0000	
**FN2011**	0.379	13.053	1.128e-1	2.655e-1	87	37	93.3680	50.6952	AAL94101.1| Valyl-tRNA synthetase
					85	111	116.8768	111.0000	
**FN2013**									AAL94103.1| GTP-binding protein
						5		5.0000	
**FN2014**	0.531	14.339	5.067e-3	2.571e-3	152	77	163.1258	105.5008	AAL94104.1| ATP-dependent protease La
					133	134	182.8778	134.0000	
**FN2015**	0.061	12.857	1.945e-1	5.725e-1	73	64	78.3433	87.6890	AAL94105.1| ATP-dependent clp protease ATP-binding subunit clpX
					71	81	97.6265	81.0000	
**FN2016**	0.540	10.256			35		37.5619		AAL94106.1| ATP-dependent Clp protease proteolytic subunit
					34	29	46.7507	29.0000	
**FN2017**	0.549	18.938	3.72e-2	5.202e-2	711	543	763.0422	743.9859	AAL94107.1| Trigger factor, ppiase
					692	428	951.5143	428.0000	
**FN2018**	1.108	8.570	2.515e-2	2.937e-2	29	15	31.1227	20.5521	AAL94108.1| Single-stranded-DNA-specific exonuclease recJ
					19	6	26.1254	6.0000	
**FN2019**	-0.432	10.192			19	29	20.3907	39.7341	AAL94109.1| Ribosome-binding factor A
					28		38.5006		
**FN2020**	0.058	15.945	1.263e-1	3.136e-1	243	190	260.7866	260.3266	AAL94110.1| Bacterial Protein Translation Initiation Factor 2 (IF-2)
					183	232	251.6288	232.0000	

Log$_2$ Ratios: -6 -4 -2 0 2 4 6

Show detected proteins only
Show all proteins
Filter by category:
GO: amino acid transport

Proteins found: 1305

Enter (or paste) list of ORFs

Find ORFs

Test	Cutoff
q-Value	.005
p-Value	

Signif	Direction	Applies To
yes	+	ratios, bars
no	n/a	bars
yes	−	ratios, bars
yes	+	*p*- , *q*-Values
yes	−	

Dot Plots Dot Plots

Protein	FnPgSg vs FnPg				Raw		Normalized		Description
	Log_2 Ratio	Log_2 Sum	*q*-Value	*p*-Value	*FnPgSg*	*FnPg*	*FnPgSg*	*FnPg*	
FN2022	0.518	11.948	3.487e-5	1.551e-6	71	38	76.1969	52.0653	AAL94112.1| N utilization substance protein A
					54	53	74.2511	53.0000	
FN2023	-0.526	7.510	1.771e-1	5.018e-1	12	20	12.8783	27.4028	AAL94113.1| Hypothetical cytosolic protein
					7	5	9.6251	5.0000	
FN2030	2.142	12.663	2.631e-4	3.529e-5	150	18	160.9794	24.6625	AAL94115.1| Inorganic pyrophosphatase
					129	52	177.3777	52.0000	
FN2031	1.255	6.164			9	4	9.6588	5.4806	AAL94116.1| Thiamine biosynthesis lipoprotein apbE
					12		16.5002		
FN2033						15		20.5521	AAL94118.1| Guanylate kinase
FN2034	0.987	9.218	2.408e-2	2.759e-2	32	18	34.3423	24.6625	AAL94119.1| Protein yicC
					25	10	34.3755	10.0000	
FN2035	0.036	17.698	2.41e-1	7.613e-1	418	375	448.5958	513.8024	AAL94120.1| DNA-directed RNA polymerase beta' chain
					353	397	485.3823	397.0000	
FN2036	0.358	16.689	6.347e-2	1.131e-1	327	163	350.9350	223.3328	AAL94121.1| DNA-directed RNA polymerase beta chain
					280	351	385.0058	351.0000	
FN2037	2.416	22.416	1.069e-3	2.693e-4	4959	1107	5321.9777	1516.7448	AAL94122.1| LSU ribosomal protein L12P (L7/L12)
					4077	531	5605.9595	531.0000	
FN2038	1.072	16.309	2.7e-2	3.258e-2	383	216	411.0340	295.9502	AAL94123.1| LSU ribosomal protein L10P
					302	97	415.2563	97.0000	
FN2039	0.322	16.555	3.595e-3	1.615e-3	316	214	339.1299	293.2099	AAL94124.1| LSU ribosomal protein L1P
					258	262	354.7553	262.0000	
FN2040	2.026	13.565	5.44e-4	1.016e-4	223	57	239.3227	78.0980	AAL94125.1| LSU ribosomal protein L11P
					149	31	204.8781	31.0000	
FN2041	-1.166	11.436	9.352e-2	1.971e-1	36	91	38.6350	124.6827	AAL94126.1| Transcription antitermination protein nusG
					23	33	31.6255	33.0000	
FN2045									AAL94129.1| Ferric uptake regulation protein
						18		18.0000	
FN2046									AAL94130.1| Acetyltransferase
						24		24.0000	
FN2047	0.226	13.426	1.36e-1	3.511e-1	91	54	97.6608	73.9876	AAL94131.1| Fusobacterium outer membrane protein family
					94	120	129.2519	120.0000	

Log_2 Ratios: -6 -4 -2 0 2 4 6

Show detected proteins only
Show all proteins
Filter by category:
GO: amino acid transport

Proteins found: 1305

Enter (or paste) list of ORFs

Find ORFs

Test	Cutoff
q-Value	.005
p-Value	

	Signif	Direction	Applies To
red	yes	+	ratios, bars
yellow	no	n/a	bars
green	yes	−	ratios, bars
pink	yes	+	*p*-, *q*-Values
light green	yes	−	

Dot Plots Dot Plots

Protein	FnPgSg vs FnPg Log$_2$ Ratio	Log$_2$ Sum	*q*-Value	*p*-Value	Raw *FnPgSg*	Raw *FnPg*	Normalized *FnPgSg*	Normalized *FnPg*	Description
**FN2048**	0.488	15.687			234		251.1278		AAL94132.1| Outer membrane protein
					213	194	292.8794	194.0000	
**FN2049**	-1.438	17.551	5.197e-2	8.444e-2	231	753	247.9082	1031.7153	AAL94133.1| unknown
					207	411	284.6293	411.0000	
**FN2050**	0.257	15.544	2.138e-1	6.518e-1	175	240	187.8093	328.8336	AAL94134.1| Hypothetical membrane-spanning protein
					211	71	290.1294	71.0000	
**FN2051**	0.652	13.840	7.563e-2	1.439e-1	142	106	152.3938	145.2348	AAL94135.1| unknown
					110	48	151.2523	48.0000	
**FN2052**	0.814	13.456	5.184e-2	8.412e-2	121	89	129.8567	121.9424	AAL94136.1| unknown
					110	38	151.2523	38.0000	
**FN2053**	0.872	12.941	2.047e-2	2.193e-2	98	30	105.1732	41.1042	AAL94137.1| Serine/threonine sodium symporter
					98	90	134.7520	90.0000	
**FN2054**	0.661	15.070	3.764e-4	5.696e-5	227	100	243.6154	137.0140	AAL94138.1| Glucose-6-phosphate isomerase
					162	158	222.7534	158.0000	
**FN2058**	-1.075	17.602	7.17e-2	1.335e-1	269	683	288.6897	935.8055	AAL94142.1| Fusobacterium outer membrane protein family
					237	359	325.8799	359.0000	
**FN2059**	0.488	15.687			234		251.1278		AAL94143.1| Outer membrane protein
					213	194	292.8794	194.0000	
**FN2060**	-1.438	17.551	5.197e-2	8.444e-2	231	753	247.9082	1031.7153	AAL94144.1| unknown
					207	411	284.6293	411.0000	
**FN2061**	0.257	15.544	2.138e-1	6.518e-1	175	240	187.8093	328.8336	AAL94145.1| Hypothetical membrane-spanning protein
					211	71	290.1294	71.0000	
**FN2062**	0.652	13.840	7.563e-2	1.439e-1	142	106	152.3938	145.2348	AAL94146.1| unknown
					110	48	151.2523	48.0000	
**FN2063**	0.814	13.456	5.184e-2	8.412e-2	121	89	129.8567	121.9424	AAL94147.1| unknown
					110	38	151.2523	38.0000	
**FN2067**					10		10.7320		AAL94151.1| Thiol:disulfide interchange protein tlpA
					13		17.8753		
**FN2068**	-2.271	6.475			4	12	4.2928	16.4417	AAL94152.1| dGTP triphosphohydrolase
						25		25.0000	
**FN2070**	-1.358	5.561			4		4.2928		AAL94154.1| Cobyric acid synthase
						11		11.0000	

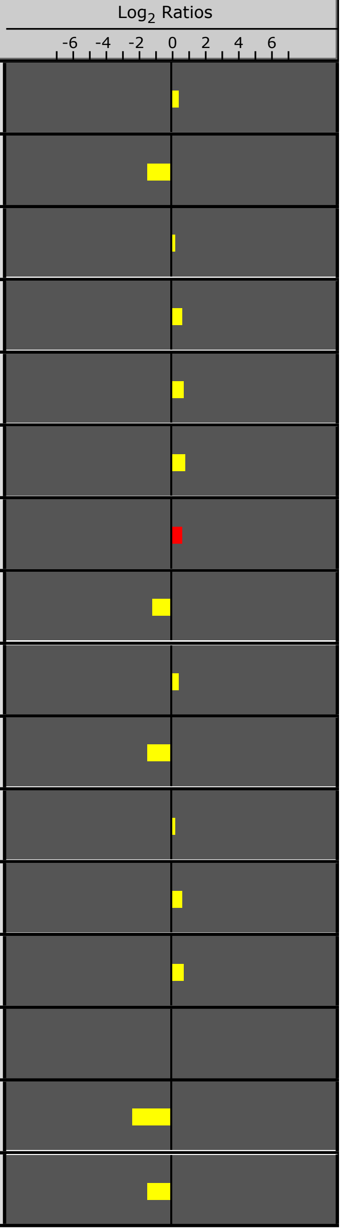

Show detected proteins only
Show all proteins
Filter by category:
GO: amino acid transport

Proteins found: 1305

Enter (or paste) list of ORFs

Find ORFs

Test	Cutoff
q-Value	.005
p-Value	

	Signif	Direction	Applies To
(red)	yes	+	ratios, bars
(yellow)	no	n/a	bars
(green)	yes	−	ratios, bars
(pink)	yes	+	*p*-, *q*-Values
(light green)	yes	−	

Dot Plots Dot Plots

Protein	FnPgSg vs FnPg Log$_2$ Ratio	Log$_2$ Sum	*q*-Value	*p*-Value	Raw *FnPgSg*	Raw *FnPg*	Normalized *FnPgSg*	Normalized *FnPg*	Description
**FN2073**	-1.325	8.288	8.984e-2	1.853e-1	8	8	8.5856	10.9611	AAL94157.1| Adenine phosphoribosyltransferase
					10	45	13.7502	45.0000	
**FN2074**	0.874	4.044							AAL94158.1| BslIM
					4	3	5.5001	3.0000	
**FN2075**	0.620	9.329	3.111e-2	4.011e-2	24	16	25.7567	21.9222	AAL94159.1| Hypothetical protein
					27	19	37.1256	19.0000	
**FN2076**									AAL94160.1| MunI regulatory protein
					3		4.1251		
**FN2078**									AAL94162.1| Transcriptional regulator, DeoR family
						3		3.0000	
**FN2082**	-0.060	20.845	2.705e-1	8.89e-1	1557	599	1670.9658	820.7138	AAL94166.1| Formate--tetrahydrofolate ligase
					740	1981	1017.5153	1981.0000	
**FN2093**	0.035	9.642	2.836e-1	9.489e-1	20	32	21.4639	43.8445	AAL94177.1| General secretion pathway protein G
					26	12	35.7505	12.0000	
**FN2098**	-1.026	4.400			3	3	3.2196	4.1104	AAL94182.1| MRP-family nucleotide-binding protein
						9		9.0000	
**FN2100**	0.521	10.477	2.095e-2	2.267e-2	42	19	45.0742	26.0327	AAL94184.1| Hypothetical protein
					33	37	45.3757	37.0000	
**FN2102**	-0.784	7.298	7.281e-2	1.364e-1	5	16	5.3660	21.9222	AAL94186.1| ABC transporter ATP-binding protein
					10	11	13.7502	11.0000	
**FN2103**	0.230	19.069	1.024e-1	2.28e-1	788	598	845.6783	819.3436	AAL94187.1| tricarboxylate-binding protein
					553	550	760.3865	550.0000	
**FN2105**	0.851	6.466			12		12.8783		AAL94189.1| tricarboxylate transport membrane protein RctA
					9	7	12.3752	7.0000	
**FN2106**	-0.814	14.771	1.085e-1	2.51e-1	112	247	120.1979	338.4245	AAL94190.1| Transporter
					96	105	132.0020	105.0000	
**FN2107**	0.891	10.878	5.318e-2	8.754e-2	55	37	59.0258	50.6952	AAL94191.1| Galactokinase
					43	13	59.1259	13.0000	
**FN2108**	0.547	9.658	7.714e-3	4.98e-3	32	19	34.3423	26.0327	AAL94192.1| Galactose-1-phosphate uridylyltransferase
					25	21	34.3755	21.0000	
**FN2109**	-0.171	12.235	1.342e-1	3.441e-1	63	63	67.6113	86.3188	AAL94193.1| UDP-glucose 4-epimerase
					46	61	63.2510	61.0000	

Log$_2$ Ratios: -6 -4 -2 0 2 4 6

Show detected proteins only
Show all proteins
Filter by category:
GO: amino acid transport

Proteins found: 1305

Enter (or paste) list of ORFs

Find ORFs

Test	Cutoff
q-Value	.005
p-Value	

Signif	Direction	Applies To
yes	+	ratios, bars
no	n/a	bars
yes	−	ratios, bars
yes	+	*p*- , *q*-Values
yes	−	

Dot Plots Dot Plots

Protein	*FnPgSg vs FnPg* Log$_2$ Ratio	Log$_2$ Sum	*q*-Value	*p*-Value	Raw *FnPgSg*	Raw *FnPg*	Normalized *FnPgSg*	Normalized *FnPg*	Description
FN2116	-2.030	7.258			5		5.3660		AAL94200.1| Hypothetical exported 24-amino acid repeat protein
					5	25	6.8751	25.0000	
FN2117									AAL94201.1| Hypothetical exported 24-amino acid repeat protein
						6		6.0000	
FN2118	1.444	7.059			15		16.0979		AAL94202.1| Hypothetical exported 24-amino acid repeat protein
					16	7	22.0003	7.0000	
FN2119	-0.996	9.500			15		16.0979		AAL94203.1| Hypothetical exported 24-amino acid repeat protein
					16	38	22.0003	38.0000	
FN2121	0.317	12.713	2.038e-1	6.13e-1	91	94	97.6608	128.7931	AAL94205.1| Hypothetical exported 24-amino acid repeat protein
					62	18	85.2513	18.0000	
FN2122	-0.487	16.941	3.608e-2	4.988e-2	297	356	318.7391	487.7698	AAL94206.1| Phenylalanyl-tRNA synthetase beta chain
					204	352	280.5042	352.0000	
FN2123	-0.606	12.629	1.18e-1	2.833e-1	60	39	64.3917	53.4355	AAL94207.1| Phenylalanyl-tRNA synthetase alpha chain
					47	143	64.6260	143.0000	
FN2125	-0.458	12.308	1.447e-1	3.824e-1	53	89	56.8794	121.9424	AAL94209.1| DNA gyrase subunit A
					47	45	64.6260	45.0000	
FN2126	1.530	11.374	1.21e-4	1.281e-5	85	26	91.2216	35.6236	AAL94210.1| DNA gyrase subunit B
					61	25	83.8763	25.0000	

Log$_2$ Ratios: -6 -4 -2 0 2 4 6

Show detected proteins only
Show all proteins
Filter by category:
GO: amino acid transport

Proteins found: 1305

Enter (or paste) list of ORFs

Find ORFs

Test	Cutoff
q-Value	.005
p-Value	

	Signif	Direction	Applies To
red	yes	+	ratios, bars
yellow	no	n/a	bars
green	yes	−	ratios, bars
pink	yes	+	*p*- , *q*-Values
light green	yes	−	

Dot Plots Dot Plots
