# Supplementary material for: Proteomics of Fusobacterium nucleatum within a model developing oral microbial community
Source: Microbiologyopen. 2014 Aug 25;3(5):729–51. doi: 10.1002/mbo3.204 (PMC4234264; doi:10.1002/mbo3.204)
Supplement: Table S10 — FileMaker results for adhesion and outer membrane proteins. Results and color coding as listed in Table S9 above. [file mbo30003-0729-sd12.pdf]

ORF		FnPg vs Fn				FnSg vs Fn				FnPgSg vs Fn				FnPgSg vs FnPg				FnSg vs FnPg				FnPgSg vs FnSg				Log_2 Ratios
		Ratio	Sum	q-Val	p-Val	Ratio	Sum	q-Val	p-Val	Ratio	Sum	q-Val	p-Val	Ratio	Sum	q-Val	p-Val	Ratio	Sum	q-Val	p-Val	Ratio	Sum	q-Val	p-Val	-6 -4 -2 0 2 4 6
**FN0264**	AAL94470.1| Hypothetical protein	-0.911	16.196	1.619e-2	2.05e-2	-0.960	16.331	3.951e-3	1.586e-2	-0.264	16.639	2.791e-3	9.033e-3	0.647	15.932	4.078e-2	5.91e-2	-0.049	15.420	4.096e-1	9.058e-1	0.696	16.067	1.196e-2	4.339e-2	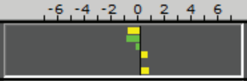
**FN0682**	AAL94878.1| Fibronectin-binding protein-like protein A	-0.287	8.933	1.441e-1	3.602e-1	0.352	9.756	1.795e-2	9.098e-2	-0.398	8.618	4.682e-2	2.403e-1	-0.111	8.535	2.443e-1	7.748e-1	0.639	9.469	6.915e-2	4.046e-2	-0.750	9.358	8.597e-3	2.928e-2	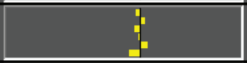
**FN1526**	AAL93652.1| Fusobacterium outer membrane protein family	-0.352	22.555	1.721e-1	4.564e-1	-2.550	20.542	5.399e-6	2.151e-6	-0.638	22.065	3.842e-4	7.109e-4	-0.286	21.917	2.052e-1	6.188e-1	-2.198	20.190	1.055e-1	9.383e-2	1.911	19.903	4.134e-4	5.808e-4	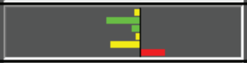
**FN1529**	AAL93655.1| Hypothetical protein	-0.496	12.897	1.244e-1	2.989e-1	-2.743	10.835	1.903e-5	1.53e-5	-0.438	12.752	2.029e-2	9.178e-2	0.058	12.459	2.761e-1	9.146e-1	-2.247	10.339	1.041e-1	9.151e-2	2.305	10.397	2.849e-3	7.201e-3	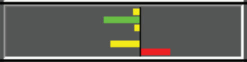
**FN1859**	AAL93958.1| Major outer membrane protein	-0.003	24.501	3.09e-1	9.971e-1	1.188	25.876	6.908e-3	3.021e-2	-1.875	22.425	5.322e-4	1.08e-3	-1.872	22.626	1.071e-1	2.46e-1	1.191	25.873	1.008e-1	8.631e-2	-3.063	24.001	3.561e-3	9.549e-3	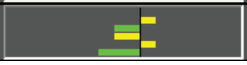
