# Supplementary material for: Proteomics of Fusobacterium nucleatum within a model developing oral microbial community
Source: Microbiologyopen. 2014 Aug 25;3(5):729–51. doi: 10.1002/mbo3.204 (PMC4234264; doi:10.1002/mbo3.204)
Supplement: Table S11 — FileMaker results for protein secretion proteins. Results and color coding as listed in Table S9 above. [file mbo30003-0729-sd13.pdf]

ORF	FnPg vs Fn Ratio	FnPg vs Fn Sum	FnPg vs Fn q-Val	FnPg vs Fn p-Val	FnSg vs Fn Ratio	FnSg vs Fn Sum	FnSg vs Fn q-Val	FnSg vs Fn p-Val	FnPgSg vs Fn Ratio	FnPgSg vs Fn Sum	FnPgSg vs Fn q-Val	FnPgSg vs Fn p-Val	FnPgSg vs FnPg Ratio	FnPgSg vs FnPg Sum	FnPgSg vs FnPg q-Val	FnPgSg vs FnPg p-Val	FnSg vs FnPg Ratio	FnSg vs FnPg Sum	FnSg vs FnPg q-Val	FnSg vs FnPg p-Val	FnPgSg vs FnSg Ratio	FnPgSg vs FnSg Sum	FnPgSg vs FnSg q-Val	FnPgSg vs FnSg p-Val	Log_2 Ratios (-6 -4 -2 0 2 4 6)
FN0699	-2.041	12.095	2.411e-4	5.118e-5	-2.538	11.782	4.124e-5	4.597e-5	-0.586	13.346	2.026e-3	6.156e-3	1.455	11.509	6.894e-3	4.104e-3	-0.498	9.741	1.948e-1	3.009e-1	1.952	11.196	1.933e-4	2.085e-4	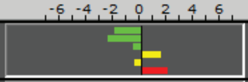
	AAL94895.1| Protein translocase subunit secD																								
FN0700	-1.970	10.170	8.457e-4	3.581e-4	-3.208	9.117	4.85e-4	1.179e-3	-1.124	10.813	1.864e-3	5.557e-3	0.847	9.046	2.532e-2	2.968e-2	-1.237	7.147	8.815e-2	6.643e-2	2.084	7.994	3.144e-6	5.274e-7	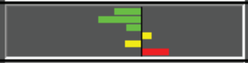
	AAL94896.1| Protein translocase subunit secF																								
FN0826	-1.809	9.442	4.137e-4	1.131e-4	-1.468	9.967	3.602e-4	8.111e-4	-0.526	10.521	1.786e-3	5.264e-3	1.282	8.916	4.308e-3	2.06e-3	0.340	8.158	2.634e-1	4.668e-1	0.942	9.440	4.821e-3	1.433e-2	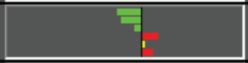
	AAL95022.1| periplasmic component of efflux system																								
FN1274	-2.178	9.111	8.321e-4	3.478e-4	-1.250	10.223	5.095e-4	1.258e-3	-1.745	9.340	4.319e-4	8.273e-4	0.433	7.366	1.519e-1	4.081e-1	0.927	8.045	1.006e-1	8.603e-2	-0.494	8.478	8.989e-4	1.686e-3	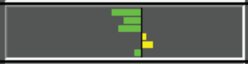
	AAL95470.1| Acriflavin resistance protein E																								
FN2093	-0.816	10.423	5.559e-2	1.038e-1	-1.909	9.515	7.33e-5	1.061e-4	-0.781	10.254	4.272e-3	1.511e-2	0.035	9.642	2.836e-1	9.489e-1	-1.092	8.699	1.561e-1	2.051e-1	1.127	8.734	7.759e-3	2.596e-2	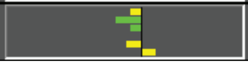
	AAL94177.1| General secretion pathway protein G																								
