# Supplementary material for: Proteomics of Fusobacterium nucleatum within a model developing oral microbial community
Source: Microbiologyopen. 2014 Aug 25;3(5):729–51. doi: 10.1002/mbo3.204 (PMC4234264; doi:10.1002/mbo3.204)

ORF	Description	FnPg vs Fn				FnSg vs Fn				FnPgSg vs Fn				FnPgSg vs FnPg				FnSg vs FnPg				FnPgSg vs FnSg			
		Ratio	Sum	q-Val	p-Val	Ratio	Sum	q-Val	p-Val	Ratio	Sum	q-Val	p-Val	Ratio	Sum	q-Val	p-Val	Ratio	Sum	q-Val	p-Val	Ratio	Sum	q-Val	p-Val
FN0113	AAL94322.1| Heat-inducible transcription repressor hrcA	0.647	11.693	1.201e-2	1.411e-2	1.648	12.879	2.225e-5	1.905e-5	-1.470	9.373	3.872e-5	2.807e-5	-2.117	10.223	2.542e-3	9.639e-4	1.001	13.526	1.855e-3	1.012e-4	-3.118	11.409	3.382e-6	5.898e-7
FN0189	AAL94395.1| Two-component response regulator yesN													-1.400	7.244			-0.627	8.202			-0.773	6.802	4.787e-3	1.42e-2
FN0503	AAL94699.1| Transcriptional regulatory protein, LYSR family	-0.592	11.037	4.431e-2	7.591e-2	-0.224	11.590	8.655e-3	3.936e-2	-0.137	11.289	2.524e-2	1.175e-1	0.456	10.900	7.943e-2	1.543e-1	0.369	10.998	1.683e-1	2.34e-1	0.087	11.453	4.988e-4	7.451e-4
FN0528	AAL94724.1| Cold shock protein	-2.764	18.819	1.531e-3	8.813e-4	-5.943	15.825	2.971e-6	8.705e-7	-0.907	20.473	7.938e-5	7.754e-5	1.857	17.912	9.068e-3	6.487e-3	-3.178	13.061	1.321e-1	1.446e-1	5.036	14.918	2e-4	2.185e-4
FN0681	AAL94877.1| Transcriptional regulator, MarR family	0.072	16.439	2.266e-1	6.533e-1	-0.045	16.507	1.015e-1	6.006e-1	-0.629	15.534	5.92e-4	1.237e-3	-0.701	15.810	2.402e-2	2.75e-2	-0.117	16.579	2.764e-1	4.986e-1	-0.585	15.877	2.382e-3	5.769e-3
FN0813	AAL95009.1| Transcriptional regulator, TetR family	1.746	8.898			2.271	9.608	2.515e-6	6.469e-7	-0.317	6.632	9.265e-2	5.159e-1	-2.063	8.581			0.526	11.354			-2.588	9.291	1.507e-4	1.494e-4
FN0943	AAL95139.1| Sensory Transduction Protein Kinase	1.943	8.232			1.376	7.849	8.893e-3	4.065e-2	-0.898	5.187	1.816e-4	2.518e-4	-2.841	7.334			-0.567	9.792			-2.274	6.951	6.423e-3	2.057e-2
FN1091	AAL95287.1| Sigma factor sigB regulation protein rsbU	-1.787	7.371	8.297e-4	3.461e-4	-0.843	8.500	2.384e-3	8.445e-3	-0.748	8.207	3.136e-4	5.364e-4	1.040	6.623	9.028e-3	6.443e-3	0.944	6.713	7.9e-2	5.261e-2	0.095	7.752	1.414e-1	7.125e-1
FN1317	AAL95513.1| RNA polymerase sigma factor	-0.425	7.763	6.64e-2	1.333e-1	-0.766	7.607	6.868e-4	1.857e-3									-0.341	7.182	1.95e-1	3.013e-1				
FN1318	AAL95514.1| RNA polymerase sigma factor rpoD	-0.535	10.215	1.379e-2	1.671e-2	-0.955	9.979	9.638e-4	2.849e-3	-0.272	10.274	9.167e-3	3.726e-2	0.262	9.943	8.204e-2	1.617e-1	-0.420	9.445	1.354e-1	1.52e-1	0.683	9.707	7.783e-3	2.606e-2
FN1321	AAL95517.1| Acetoacetate metabolism regulatory protein atoC	0.002	19.560	3.006e-1	9.58e-1	0.313	20.055	7.656e-4	2.157e-3	0.034	19.388	1.389e-1	8.162e-1	0.032	19.594	2.554e-1	8.225e-1	0.311	20.057	2.495e-2	6.668e-3	-0.279	20.089	2.344e-2	9.448e-2
FN1439	AAL95632.1| Transcriptional regulator, DeoR family	-2.376	11.423			-3.837	10.146	9.237e-4	2.705e-3	-1.963	11.632	1.338e-3	3.582e-3	0.413	9.460			-1.461	7.770			1.874	8.183	2.529e-3	6.227e-3
FN1831	AAL93930.1| Nitrogen assimilation regulatory protein	0.505	12.687	8.971e-3	9.957e-3	0.765	13.132	3.158e-6	9.818e-7	-0.796	11.183	3.185e-3	1.06e-2	-1.301	11.892	9.341e-4	2.159e-4	0.260	13.637	5.632e-2	2.828e-2	-1.561	12.336	2.961e-4	3.795e-4
FN1914	AAL94013.1| Anti-sigma F factor antagonist	0.655	13.979	1.845e-1	5.025e-1	-0.288	13.220	8.128e-3	3.655e-2	-0.623	12.497	1.233e-3	3.184e-3	-1.278	13.356	1.248e-1	3.081e-1	-0.943	13.875	2.323e-1	3.908e-1	-0.335	12.597	1.324e-2	4.887e-2
FN1987	AAL94077.1| Transcriptional regulator, GntR family	2.122	7.705			-0.299	5.469			-0.545	4.834	1.357e-3	3.658e-3	-2.667	7.160			-2.421	7.591			-0.246	4.924		

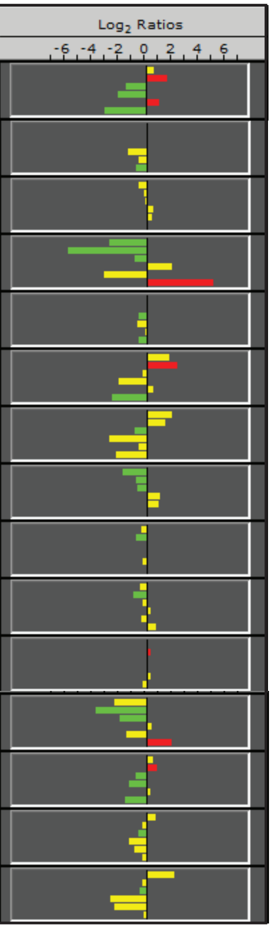

Supplement: Table S12 — FileMaker results for significantly altered transcriptional regulators. Results and color coding as listed in Table S9 above. [file mbo30003-0729-sd14.pdf]
