# Supplementary material for: Proteomics of Fusobacterium nucleatum within a model developing oral microbial community
Source: Microbiologyopen. 2014 Aug 25;3(5):729–51. doi: 10.1002/mbo3.204 (PMC4234264; doi:10.1002/mbo3.204)

		FnPg vs Fn				FnSg vs Fn				FnPgSg vs Fn				FnPgSg vs FnPg				FnSg vs FnPg				FnPgSg vs FnSg			
ORF		Ratio	Sum	q-Val	p-Val	Ratio	Sum	q-Val	p-Val	Ratio	Sum	q-Val	p-Val	Ratio	Sum	q-Val	p-Val	Ratio	Sum	q-Val	p-Val	Ratio	Sum	q-Val	p-Val
FN0074	AAL94287.1| Ethanolamine utilization protein eutS	-0.272	8.272			-0.234	8.494	9.103e-2	5.322e-1	-0.710	7.629			-0.439	7.561			0.038	8.222			-0.476	7.784		
FN0076	AAL94289.1| Ethanolamine two-component response regulator																								
FN0077	AAL94290.1| Ethanolamine two-component sensor kinase	-1.392	4.561			0.632	6.769											2.023	5.378						
FN0078	AAL94291.1| Ethanolamine utilization protein eutA	1.882	7.835			1.437	7.574			-0.209	5.540			-2.092	7.626	8.903e-4	2.004e-4	-0.445	9.457	4.438e-2	1.819e-2	-1.646	7.365	7.87e-5	6.302e-5
FN0079	AAL94292.1| Ethanolamine ammonia-lyase heavy chain	0.842	14.037	1.25e-1	3.005e-1	1.007	14.387	4.635e-5	5.506e-5	-1.622	11.369	8.866e-5	9.085e-5	-2.464	12.415	5.957e-2	1.037e-1	0.165	15.228	3.665e-1	7.552e-1	-2.629	12.764	5.808e-5	4.252e-5
FN0080	AAL94293.1| Ethanolamine ammonia-lyase light chain	0.902	14.623	4.69e-4	1.392e-4	0.180	14.085	3.374e-2	1.823e-1	-0.932	12.585	9.368e-4	2.197e-3	-1.834	13.691	3.228e-6	6.998e-8	-0.723	14.987	3.075e-3	2.684e-4	-1.112	13.153	5.42e-4	8.365e-4
FN0081	AAL94294.1| Ethanolamine utilization protein eutL	1.042	14.363	7.517e-2	1.565e-1	1.350	14.856	2.767e-3	1.014e-2	-1.614	11.503	3.069e-5	1.767e-5	-2.656	12.749	3.831e-2	5.419e-2	0.308	15.898	2.664e-1	4.741e-1	-2.964	13.242	1.679e-3	3.728e-3
FN0082	AAL94295.1| Ethanolamine utilization protein eutM	1.056	8.758			1.343	9.229			0.534	8.032			-0.522	9.292			0.287	10.285			-0.809	9.763	2.699e-2	1.106e-1
FN0083	AAL94296.1| Ethanolamine utilization protein eutM precursor	1.151	15.460	6.376e-2	1.263e-1	0.714	15.208	2.881e-3	1.066e-2	-0.929	13.177	1.214e-3	3.115e-3	-2.079	14.531	4.203e-2	6.167e-2	-0.436	16.359	2.349e-1	3.977e-1	-1.643	14.280	1.247e-3	2.574e-3
FN0084	AAL94297.1| Acetaldehyde dehydrogenase (acetylating)	0.119	10.872	4.019e-2	6.639e-2	-0.211	10.727	3.136e-2	1.681e-1	-0.929	9.620	4.369e-4	8.395e-4	-1.048	9.943	4.175e-3	1.977e-3	-0.329	10.846	8.358e-2	5.918e-2	-0.719	9.798	4.306e-3	1.232e-2
FN0086	AAL94299.1| Hypothetical protein																								
FN0087	AAL94300.1| Ethanolamine utilization protein eutN																	-1.925	6.569						
FN0088	AAL94301.1| Hypothetical protein					0.686	5.654																		
FN0090	AAL94303.1| Ethanolamine utilization protein eutQ																	-0.576	6.001	1.706e-1	2.397e-1				
FN1891	AAL93990.1| Glycerophosphoryl diester phosphodiesterase	0.939	8.361	1.275e-1	3.085e-1	1.317	8.923	3.924e-4	9.01e-4	0.151	7.369	1.013e-1	5.703e-1	-0.788	8.512	1.377e-1	3.577e-1	0.377	9.862	2.765e-1	4.99e-1	-1.166	9.074	2.615e-4	3.219e-4
FN1908	AAL94007.1| Glycerophosphoryl diester phosphodiesterase	0.384	16.395	2.985e-2	4.49e-2	0.545	16.741	6.616e-4	1.767e-3	-0.372	15.435	6.722e-3	2.592e-2	-0.756	16.023	1.28e-2	1.097e-2	0.161	17.125	1.713e-1	2.414e-1	-0.917	16.369	6.053e-4	9.818e-4
FN0089	AAL94302.1| Ethanolamine permease																								
FN0075	AAL94288.1| Ethanolamine utilization protein eutP																								
FN0085	AAL94298.1| Ethanolamine utilization cobalamin adenosyltransferase																								
FN1954	AAL94050.1| Glycerophosphoryl diester phosphodiesterase																								

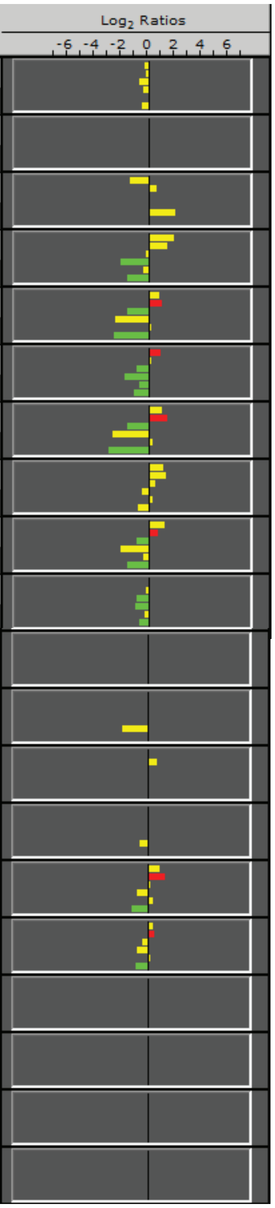

Supplement: Table S9 — FileMaker results for ethanolamine metabolism proteins. Proteins are listed by FN number. For each of the six comparisons, the log2 ratio of the normalized spectral counts, log2 sum of normalized spectral counts, P-value, and q-value are shown. Statistically significant increases are shown in red with the corresponding q-value in light red while decreases are shown in green with the corresponding q-value in light green. Color-coded bar graphs are presented for all six ratio comparisons. [file mbo30003-0729-sd11.pdf]
